# Do Multicomponent Workplace Health and Wellbeing Programs Predict Changes in Health and Wellbeing?

**DOI:** 10.3390/ijerph18178964

**Published:** 2021-08-25

**Authors:** Kevin Daniels, Roberta Fida, Martin Stepanek, Cloé Gendronneau

**Affiliations:** 1 Employment Systems and Institutions Group, Norwich Business School, University of East Anglia, Norwich NR4 7TJ, UK; r.fida@uea.ac.uk; 2 Institute of Economic Studies, Faculty of Social Sciences, Charles University, 110 00 Staré Město, Czech Republic; stepanek.martin@hotmail.com; 3 RAND Europe, Cambridge CB4 1YG, UK; cloe.gend@live.fr

**Keywords:** workplace health and wellbeing programs, wellbeing, wellbeing practices, psychosocial hazards

## Abstract

Organizations typically deploy multiple health and wellbeing practices in an overall program. We explore whether practices in workplace health and wellbeing programs cohere around a small number of archetypal categories or whether differences between organizations are better explained by a continuum. We also examine whether adopting multiple practices predicts subsequent changes in health and wellbeing. Using survey data from 146 organizations, we found differences between organizations were best characterized by a continuum ranging from less to more extensive adoption of practices. Using two-wave multilevel survey data at both individual and organizational levels (N = 6968 individuals, N = 58 organizations), we found that, in organizations that adopt a wider range of health and wellbeing practices, workers with poor baseline psychological wellbeing were more likely to report subsequent improvements in wellbeing and workers who reported good physical health at baseline were less likely to report experiencing poor health at follow-up. We found no evidence that adopting multiple health and wellbeing practices buffered the impact of individuals’ workplace psychosocial hazards on physical health or psychological wellbeing.

## 1. Introduction

Workplace health and wellbeing programs encompass a range of different practices, including workplace health promotion, provision of self-regulatory skills to manage exposure to risk (e.g., resilience training), rehabilitation, return to work and management of chronic conditions [1,2,3]. Systematic reviews of controlled trials attest to the benefits of the specific practices that make up such programs [4,5,6]. However, it is recommended that multiple practices should be used in the management of workplace health and wellbeing. For example, internationally accepted standards recommend the use of multiple strategies in a ‘hierarchy of controls’, in which successive practices build on each other to eliminate risk [7] and the practice-based literature suggests multiple practices should be actively managed in a coherent program [8]. Studies of exemplary organizational practice [2,9] and surveys of organizations also indicate that organizations typically have multiple practices in their workplace health and wellbeing programs [10]. 

As far as we are aware, only two cross-sectional studies to date have examined the effects of using multiple practices together [11,12]. Both found associations between the adoption of multiple practices on the one hand and perceptions of program effectiveness, reports of practices use [11] and perceptions of others’ wellbeing in the organization [12] on the other. The purpose of this study is to address four salient issues that have not been addressed to date.

First, none of the studies of adopting multiple workplace health and wellbeing practices has directly assessed markers of individuals’ physical health and psychological wellbeing. Second, existing cross-sectional studies cannot supply evidence on whether adopting multiple workplace health and wellbeing practices are related to subsequent changes in physical health and psychological wellbeing. This limits inferences regarding whether programs of health and wellbeing practices are causes of changes in health and psychological wellbeing or whether organizations with healthy workers are more likely to adopt a fuller range of health and wellbeing practices. Third, existing research has not examined directly whether workplace health and wellbeing programs are differentially effective for those exposed to risk factors. Fourth, existing research has examined differences in the extent to which organizations adopt a range of practices on a single continuum. However, it is possible that differences between organizations in their adoption of health and wellbeing practices are not based on a continuum, but really reflect distinct and internally consistent categories, some of which may be equally effective as categories characterized by a wide range of practices yet they are characterized by a smaller number of practices. 

### 1.1. Configuring Workplace Health and Wellbeing Programs

The two existing studies on the correlates of the adoption of multiple workplace health and wellbeing practices have treated organizational adoption as a continuum ranging from low adoption to more extensive adoption of practices [11] or as a continuum on the extent to which organizations focus on a narrow to a wider range of psychological health goals [12] (e.g., practices for dealing with existing conditions, practices for preventing conditions developing). It is parsimonious to treat the organizational adoption of health and wellbeing practices as a single continuum, but by doing so, research may miss important relationships. An alternative approach is to consider whether differences in adoption of practices could be represented by discrete categories [10]. 

There are arguments for and against modelling adoption as a continuum/continuums or as a set of discrete categories. On the one hand, the adoption of best practices along a single continuum reflects that the extensive use of practices can lead to practices reinforcing each other and an organizational strategy towards workplace health and wellbeing (cf. [13]), especially if indicators of program support (e.g., service promotion) and co-ordination (e.g., steering groups) are included alongside indicators of practices.

On the other hand, choices made by managers may not simply reflect the choice to use best practices in a coherent strategy, but more nuanced questions in respect of how much to invest in workplace health and wellbeing and in what services, the focus of specific health and wellbeing practices, whether to invest in practices that are tailored to meet the specific concerns or needs of their workers or to adopt universal best practice guidelines, the overall goal to be achieved by a health and wellbeing program [14] and industry norms in respect of health and wellbeing [15]. Such factors may lead to a differentiation of organizations into distinct categories, within which each category represents a coherent bundle of practices that are mutually reinforcing and serve different organizational goals. For example, organizations concerned with employer attractiveness and brand may differentially invest in highly visible and symbolic practices (e.g., gym membership, workplace health promotion), those concerned with social responsibility goals or meeting industry best practice may invest across a wider range of practices, those concerned with reducing health insurance costs may differentially invest in vocational rehabilitation and those concerned with compliance with legal regulatory standards may make minimal investments. Only one previous study has investigated whether organizations can be categorized according to the number and type of practices they adopt, and that study used exploratory cluster analytic methods [10]. Therefore, we had no expectations regarding the number or composition of categories. 

In light of the forgoing arguments, we asked whether a small number of categories characterize workplace health and wellbeing programs according to the types of specific health and wellbeing practices, or whether differences between organizations are better represented by a single continuum, resulting in two competing hypotheses:

**Hypothesis** **1a** **(H1a).** *Differences between organizations in their health and wellbeing programs are better represented by a small number of categories than a single continuum.*

**Hypothesis** **1b** **(H1b).** *Differences between organizations in their health and wellbeing programs are better represented by a single continuum than a small number of categories.*

### 1.2. The Effects of Multiple Workplace Health and Wellbeing Practices

Given that specific practices can have effects on health and wellbeing outcomes [3,4,5,6], then a program of practices may have effects on health outcomes too. Extensive workplace health and wellbeing programs may have effects through six potential routes. The first and second are generic to physical health and psychological wellbeing. The four remaining routes relate specifically to psychological wellbeing. 

First, an extensive program offers a suite of practices that can allow individuals to engage with those practices best suited to their own preferences, circumstances and/or health needs [16], thus enabling a wider range of individuals to engage with tailored activities than would be possible in less extensive programs. Second, an extensive program of activities, especially if accompanied by management actions that communicate, reinforce and legitimize the adoption of those practices [17], provides clear social cues that may change workplace norms around health behaviors [18]. Third, health and wellbeing practices that involve group activities (fitness classes, group-based mindfulness or resilience training) may improve psychological wellbeing because of the benefits of social activities [19]. Fourth, health and wellbeing practices that enhance physical health may also have effects on psychological wellbeing [20,21]. Fifth, some practices in health and wellbeing programs are targeted at psychological wellbeing (e.g., counselling services, resilience training, flexible working to reduce work-family conflict). Sixth, extensive adoption of workplace health and wellbeing practices, especially if accompanied by management actions that communicate, reinforce, and legitimize the adoption of those practices, may lead employees to perceive organizational care and resources for supporting employee health and wellbeing [12], in turn leading to higher levels of psychological wellbeing [22,23]. Given these six routes, which apply to a range of physical health and psychological outcomes, we expect that effects of a program to become manifest more readily on general physical health and psychological wellbeing rather than markers of specific conditions, especially those conditions that are chronic or slow to develop. 

Organizations that adopt a wider range of practices typically include components that are both preventive (e.g., smoking cessation) and rehabilitative (e.g., counselling services) [2,9], (see also [7]). It is therefore important to differentiate between the potential preventive effects of workplace health and wellbeing programs for healthy workers (Hypothesis 2a) and the rehabilitative effects for workers suffering with poor physical health and psychological wellbeing (Hypothesis 2b). In the latter case, individuals with initially poor health/wellbeing may have access to a wider range of practices to aid recovery (e.g., physical activity promotion) on top of rehabilitation practices, and perceive more organizational support, in turn promoting positive attitudes to work and recovery [24].

**Hypothesis** **2a** **(H2a).** *Individuals with initially good physical health/psychological wellbeing subsequently report better physical health/psychological wellbeing if they work for an organization with extensive workplace health and wellbeing programs.*

**Hypothesis** **2b** **(H2b).** *Individuals with initially poor physical health/psychological wellbeing subsequently report better physical health/psychological wellbeing if they work for an organization with extensive workplace health and wellbeing programs.*

In contrast to the expectation in Hypothesis 2, Batorsky et al. [11] found smaller differences in perceptions of program effectiveness and use of practices between organizations that adopted an extensive range of practices and those that adopted a more moderate range of practices, compared to those adopting a moderate range of practices and those with minimum coverage. Although Batorsky et al. did not assess health outcomes directly, Batorsky et al.’s findings might indicate there is a ceiling on the number of practices needed, after which employee choice of practices to engage with becomes saturated. Batorsky et al.’s findings therefore suggest a non-linear relationship between the number of practices in workplace health and wellbeing programs and the extent to which those with initially good/poor health or wellbeing report deterioration/improvement in health or wellbeing.

**Hypothesis** **3a** **(H3a).** *There is a non-linear relationship between the extent to which organizations adopt workplace health and wellbeing practices and subsequent physical health/psychological wellbeing for individuals with initially good physical health/psychological wellbeing, such that there is a threshold after which more practices are associated with diminishing benefits.*

**Hypothesis** **3b** **(H3b).** *There is a non-linear relationship between the extent to which organizations adopt workplace health and wellbeing practices and subsequent physical health/psychological wellbeing for individuals with initially poor physical health/psychological wellbeing, such that there is a threshold after which more practices are associated with diminishing benefits.*

There is evidence that specific workplace health and wellbeing practices can be particularly beneficial for employees experiencing poor psychological wellbeing who are at risk because of individuals’ workplace psychosocial hazards (e.g., time pressure, low job autonomy) [6]. A program of health and wellbeing activities may enhance individuals’ attitudes (e.g., self-efficacy) to coping with their workplace psychosocial hazards [12]. Therefore, it is possible that extensive adoption of workplace health and wellbeing programs buffers the negative impact of individuals’ workplace psychosocial hazards over time, protecting individuals with initially good physical health or psychological wellbeing (Hypothesis 4a) and/or allowing those with poor physical health or psychological wellbeing a better chance of improvement (Hypothesis 4b).

**Hypothesis** **4a** **(H4a).** *The relationship between individuals’ workplace psychosocial hazards and subsequent poor physical health/psychological wellbeing in workers with initially good physical health/psychological wellbeing is moderated by the extent to which organizations adopt extensive workplace health and wellbeing programs.*

**Hypothesis** **4b** **(H4b).** *The relationship between individuals’ workplace psychosocial hazards and subsequent poor physical health/psychological wellbeing in workers with initially poor physical health/psychological wellbeing is moderated by the extent to which organizations adopt extensive workplace health and wellbeing programs.*

## 2. Materials and Methods

### 2.1. Study Design

We used a two-wave multiple informant design, in which data were collected from workers twice with a year-long interval and from senior managers who provided linked data on organizational level variables at baseline. We used organization and employee data collected in 2015 (T1) and 2016 (T2) through the Britain’s Healthiest Workplace survey. The annual survey is open to all UK-based organizations with at least 20 employees from any sector. The survey is voluntary and is designed and conducted by Vitality Health from 2013. The survey linked responses from individual employees with data provided by the most senior manager with responsibility for occupational health (e.g., occupational health or human resources professionals). Individual employee and organizational responses are matched over time. The present study is therefore based on secondary data analyses of an existing data source, wherein which the development of hypotheses and choice of key variables occurred after data had been collected.

### 2.2. Sample

We used organizational data collected in 2015 (T1) which included 146 large (38%, ≥500 employees), medium (34%, 50–499 employees) and small (29%, ≤49 employees) organizations from a range of sectors (number of employee responses = 39,255). We also used the sample of employees that participated at both T1 and T2 (N = 6968, 53% males, average age 39.5, SD = 10.7, average job tenure 9.5 years, SD = 9.6) nested in 58 organizations that provided data at T1 and T2. Major job categories in the sample were professional (48.7%), clerical and administrative support (20.9%), executive or senior manager (15.7%), technical support (5.9%) or sales (5.6%). Analysis of dropouts considering the organizational data showed that data were missing completely at random and none of the organizational study variables at T1 affected the likelihood of dropping out (Little’s MCAR Test χ2 = 13.01, *p* = 0.95). Similarly, for those participants in organizations that chose to participate at T1 and T2, individual level data at T1 and T2 were also missing completely at random (Little’s MCAR test χ2 = 53.31, *p* = 0.14). There were no missing data on organizational level data for those participants that responded at T1 and T2. For individual level data, data at T1 and T2 were missing completely at random (Little’s MCAR test χ2 = 53.58, *p* = 0.13).

### 2.3. Organizational Level Measures 

We defined workplace health and wellbeing practices as practices introduced into an organization that are targeted at physical health and/psychological wellbeing, including practices aimed at primary prevention (e.g., flexible working hours), secondary prevention (e.g., stress management training) or tertiary rehabilitation (e.g., disease management (see [3,7]). We used a comprehensive range of health and wellbeing practices from the T1 survey. We also included indicators or how those services were promoted, coordinated and management support for health and wellbeing because these activities are important for developing a coherent program of activities [1,4]. Appendix A gives the full list of items.

Active wellbeing governance was measured by three indicators assessing if the organization had a committee responsible for health promotion and wellness, if the organization organized wellness days, and if employee feedback on health promotion activities was collected (0 = no, 1 = yes). The categorical omega reliability coefficient showed good reliability (0.80). 

Benefits were measured by four items assessing the percentage of employees either full time or part time with private medical insurance or other health benefits. The omega reliability coefficient demonstrated good reliability (0.78).

Flexible time was measured by one item assessing the percentage of employees working flexible time.

Accumulated overtime was measured by one item assessing if the organization allowed accumulated hours working overtime to be used for vacations.

Health and wellbeing activities were measured by asking the employer to indicate the availability or not (0 = no, 1 = yes) of 27 specific services targeted at different aspects of health and wellbeing. Three services referred to medical services (omega = 0.77), seven targeting smoking cessation (omega = 0.84), three targeting drinking support (omega = 0.68), five physical health (omega = 0.68), five healthy eating (omega = 0.75) and four targeting psychological problems (omega = 0.71). 

Service promotion was measured by asking the employer five questions on how the organization actively promoted wellbeing facilities, services or programs (0 = no, 1 = yes). The categorical omega reliability coefficient demonstrated good reliability (0.89). 

Support for health promotion was measured by asking the employer 10 questions on how the organization supported employees’ health (0 = no, 1 = yes). The categorical omega reliability coefficient demonstrated good reliability (0.86). 

Management support was measured by 10 items assessing how strongly the management representative agreed (from 1= strongly disagree to 5 = strongly agree) with a list of statements related support for employee health and wellbeing. The omega reliability coefficient demonstrated good reliability (0.85).

### 2.4. Employee Level Measures

The following employee level dimensions were used (Appendix A provides the full list of items).

Individuals’ workplace psychosocial hazards at T1 were assessed with four single item indicators. Participants rated their level of agreement with items assessing their experiences of job autonomy, bullying, time pressure and role clarity (1 = strongly disagree to 5 = strongly agree).

Psychological distress was used as a marker of psychological wellbeing at T1 and T2, given the centrality of affective states in models of psychological wellbeing [25]. Psychological distress was measured by six items from the Kessler scale [26,27,28] assessing employees’ feelings (e.g., feeling nervous, hopeless and restless) in the previous 30 days (from 0 = none of the time to 4 = all the time). The omega reliability coefficients demonstrated good reliability at both T1 and T2 (T1 = 0.85 and T2 = 0.87). To differentiate between those with high/low psychological distress in analyses on the increase/decrease in distress, we used cut-offs of an average score on each item of one or less to denote those with low psychological distress (N = 5885 at T1, N = 5521 at T2, N = 720 experienced more distress between T1 and T2) and an average score of 1 or more to denote those with high psychological distress (N = 1113 at T1, N = 1447 at T2, N = 386 less distress between T1 and T2). The Kessler scale has flexible cut-off points [26], and our choice of cut-off was intended to allow us to differentiate those with good psychological wellbeing from those with milder and commoner forms of distress, as well as from those with severe mental health problems [26,28]. A robustness check was conducted to ensure the dichotomization of psychological distress in this way did not affect the results (Appendix B).

Poor physical health: We used a single item assessing employee physical health (from 0 = very good, 1 = good, 2 = fair, 3 = bad, 4 = very bad). Single item indicators of self-reported health have been shown to have good reliability [29] and ability to predict subsequent health outcomes (e.g., sickness absence) [30]. Similar to psychological distress, we needed to differentiate between those who might improve/deteriorate over time. Scores of zero or one were denoted as good physical health (N = 5546 at T1, N = 5401 at T2, N = 662 deteriorated between T1 and T2) and two or more as poor physical health (N = 1422 at T1, N = 1567 at T2, N = 517 improved between T1 and T2). The choice of cut-off again was intended to allow us to differentiate those with good health from those with milder and more frequent health complaints (e.g., muscular-skeletal problems) as well as from those with more severe health complaints. A robustness check was also conducted on the dichotomization of physical health (Appendix B).

## 3. Results

Table 1 and Table 2 show the descriptive statistics and correlations for the variables.

### 3.1. Hypothesis 1: Representing Differences between Organizations in Their Health and Wellbeing Practices

We have two competing hypotheses, namely that differences between organizations in their health and wellbeing programs are better represented by a small number of categories (H1a) or by a single continuum (H1b). We used latent profile analysis (LPA, implemented using Mplus 8.4) [31] and principal components analysis (PCA) using the organizational level data at T1 (146 organizations). LPA was used to identify categories of organizations according to the adoption of health and wellbeing practices (H1a). We analyzed and compared models from a one to a five-categories solution. Decisions on model retention were guided by the following criteria: Akaike’s information criterion (AIC), Bayesian information criterion (BIC), Vuong–Lo–Mendell–Rubin adjusted likelihood ratio (VLMR), bootstrapped likelihood ratio (BLRT), entropy values and finally category size. Solutions with lower AIC and BIC indicate better quality models [32]; BLRT and VLRM of the selected model should be significant (*p* < 0.05) [31]; entropy should be greater than 0.80 [33] and no category should have less than 10% of the sample. We used PCA to investigate whether a single continuum represents differences between organizations (H1b). The emergence of a relatively large first component would provide evidence for a single continuum. However, as an exploratory method, PCA also allows us to investigate whether there are multiple continuums, which has not been investigated in the literature to date. 

Table 3 shows the results of four comparable solutions. The solution with three distinct categories satisfied all the criteria. ANOVA indicated significant differences on all organizational level variables between the three categories identified as the best solution for categorizing organizations (*p* < 0.001) with the exception of accumulated overtime (*p* = 0.079). All three categories were rank ordered according to comparatively high, medium or low rates of adoption across all practices. The pattern of results may indicate that the categories can be distinguished by a single continuum ranging from low to high adoption of practices.

PCA revealed two components with eigenvalues greater than 1 (6.03, accounting for 46% of the variation and 1.29 accounting for 10% of the variation). Prior to rotation, nine variables had component loadings over 0.70 on the first component, drinking support services loaded at 0.60, loadings for benefits and flexible working were over 0.30, and the loading for accumulated overtime was 0.20. Extracting two components with oblique rotation revealed five and six variables with cross-loadings >|0.20| in the pattern and structure matrices respectively. PCA therefore suggests a single dominant component. We used confirmatory factor analysis (CFA) to check the appropriateness of a single factor solution, using Bayesian estimation given the relatively low number of organizations (<150). CFA indicated a single factor model had reasonable fit to the data (confirmatory fit index = 0.90; root mean square error of approximation = 0.09; all factor loadings positive and significant, *p* < 0.05). A Spearman’s rank correlation between category membership from the best LPA solution and scores on the first principal component identified by PCA was 0.93 (*p* < 0.001). Discriminant function analysis using category membership as a criterion and the adoption of specific practices as predictors revealed a single large function accounted for over 96% of the variance between categories. A histogram representing the frequencies of values on the first principal component did not provide any evidence of a multimodal distribution. Accordingly, we concluded a single continuum of practices is a better representation of major differences between organizations than a categorical structure. Therefore, H1b is supported and H1a is not.

Using data from the T2 organization survey, we attempted to replicate our the findings for Hypotheses 1a and 1b. Full details of these supplementary analyses are available from the authors on request and at the University of East Anglia institutional repository. In respect of the LPA, we found that a two-category solution was superior. Solutions with three, four and five categories all had categories with less than 10% of the sample. Therefore, we did not replicate the same category structure with T2 data. All practices were consistently higher in one category compared to the other (*p* < 0.05, excepting flexible time *p* = 0.05). PCA again revealed one large component accounting for over 40% of the variation, with two smaller components accounting for less than 12% of the variation each. Prior to rotation, nine variables had component loadings over 0.60 on the first component with the remainder loading over 0.20. A Bayesian CFA indicated slightly worse fit for a single factor solution than with the T1 data (Confirmatory Fit Index = 0.85; root mean square error of approximation = 0.11), although all factor loadings positive and significant, *p* < 0.05). Again, a histogram on the frequencies of values on the first principal component did not provide any evidence of a multimodal distribution. Therefore, the T2 data also indicated that a single continuum may better represent differences between organizations (H1b) than a small number of discrete categories (H1a).

### 3.2. Hypotheses 2–4: Effects of Workplace Health and Wellbeing Programs

We had three expectations with respect to the effects of workplace health and wellbeing programs, namely that more extensive workplace health and wellbeing programs are associated with better physical health and less psychological distress (H2), that there is a non-linear relationship between the number of practices in workplace health and wellbeing programs and physical health and lower psychological distress (H3) and that more extensive health and wellbeing programs buffer the effects of individuals’ workplace psychosocial hazards on physical health and psychological distress (H4). Each hypothesis was further differentiated according to whether workplace health and wellbeing programs have effects on maintaining good physical health or low psychological distress amongst workers with good physical health or low psychological distress (H2a, H3a, H4a) or facilitating recovery for workers with poor physical health or high psychological distress (H2b, H3b, H4b). 

Hypotheses 2–4 were examined using multilevel probit regressions with a Bayesian estimator on T1 and T2 data with categorical outcomes differentiating those with poor physical health or high psychological distress from those with good health and low psychological distress at T2. Bayesian analysis has less restrictive assumptions concerning the distributions of data and allows more flexible model fitting, especially with a relatively small number of organizations supplying data over time, missing data and fitting of complex models for robustness checks that might otherwise have convergence problems (see Appendix B). 

In the analyses, we used three Markov chains, thinning was applied every 20 iterations, the number of iterations was set to a minimum of 1000 and there was good convergence for the models with the data (as indicated by potential scale reduction values of < 1.02 at the last iteration and values <1.05 for the second half of the iterations). We used MPlus default values of uninformative priors [31], given there are no existing studies directly examining relationships between workplace health and wellbeing programs and changes in physical health or psychological distress. Analyses based on uninformative priors converge with estimates derived from other methods with large sample sizes [34]. Given the relatively small sample size of organizations in this study, we checked our Bayesian estimates led to the same inferences using other estimation methods (see Appendix B). Except for terms involved in cross-level interactions (H4a, H4b), all individual level regression slopes were fixed to be invariant across organizations. 

For hypotheses concerning whether workplace health and wellbeing programs prevent deterioration of physical health or increases in psychological distress (H2a, H3a, H4a), we selected those individuals at T1 for analyses that were categorized as having good physical health or low psychological distress. For hypotheses concerning whether workplace health and wellbeing programs facilitate recovery from poor physical health/psychological distress (H2b, H3b, H4b), we selected those individuals at T1 for analyses that were categorized as having poor physical health or high psychological distress. To represent the adoption of health and wellbeing practices (predictor variable), we used the component scores for the first and largest component derived from PCA of the organizational level data. Component scores were derived using the regression method in SPSS [35]. Doing so reflects that some practices may provide a greater contribution to an overall health and wellbeing program than others. Therefore, using the component score avoids the restrictive assumption that all practices have equal weighting in a program. 

Other predictor variables in each analysis were individual reports of physical health, psychological distress and individuals’ workplace psychosocial hazards at T1. Hypotheses 3a and 3b were examined using the linear term for the first principal component and its squared value, which is a common approach to investigating non-linear regression slopes with a single inflection point [36]. Hypotheses 4a and 4b were examined by cross-level interactions represented by the regression of the scores for the first principal component on the regression slope of each of the four indicators of individuals’ workplace psychosocial hazard on physical health or psychological distress. Separate analyses were run for each of the four indicators of individuals’ workplace psychosocial hazards: Given the number of organizations, fitting all moderator effects in one model would have been intractable. For tests of Hypotheses 2a, 2b, 3a and 3b, all individual level predictors were centered at the grand mean value for the sample. For Hypothesis 4a and 4b, individuals’ workplace psychosocial hazards involved in the cross-level interactions were centered at each organization’s mean [37] and the mean for each organization was entered as an organizational level variable. 

Table 4 shows the main results of the regression analyses for Hypotheses 2a–3b and Table 5 shows the 95% credibility intervals for each regression coefficient. The results show that organizations with the most extensive range of health and wellbeing practices are more likely to have workers that maintain good physical health from T1 to T2 and workers who are more likely to recover from psychological distress from T1 and T2. That is, extensive adoption of workplace health and wellbeing practices appears to prevent deterioration of physical health (H2a) and facilitate recovery from high levels of psychological distress (H2b). The results provide no evidence that workplace health and wellbeing programs facilitate recovery from poor physical health or help to prevent future occurrence of psychological distress. Overall, Hypothesis 2a is supported for self-reported physical health only and Hypothesis 2b for psychological distress only. Table 4 shows there is no support for any curvilinear effects of health and wellbeing programs such that effects level off above a certain point (H3a, H3b). In three models, the curvilinear terms are not significant. For those with high psychological distress at T1, both the linear and curvilinear terms are negative and significant, indicating rates of recovery from high levels of psychological distress are highest in organizations that adopt the widest range of practices. These findings are more in line with Hypothesis 2b than Hypothesis 3b. Therefore, there is no support for H3a or H3b in respect of physical health or psychological distress. None of the interactions between adoption of practices and individuals’ workplace psychosocial hazards were significant, and so there is no support for Hypotheses 4a and 4b in respect of physical health or psychological distress.

We conducted a series of robustness checks (Appendix B) to validate the findings in respect of: (i) using alternative estimation methods; (ii) the dichotomization of T1 psychological wellbeing and physical health to create sub-samples for analyses; (iii) comparing findings from the analyses with the first principal component with analogous analyses conducted using categories derived from the LPA; (iv) controlling for organizational size and sector and (v) checking that the findings could not be explained by the adoption of a single practice or smaller set of practices adopted as part of a larger set of practices, rather than the combined effects of an entire program. The results replicate those reported here in each instance.

## 4. Discussion

We found that organizations can be differentiated on a continuum representing more or less extensive adoption of workplace health and wellbeing practices and supporting structures (e.g., governance, service promotion). We also found more extensive adoption is associated with maintenance of good self-reported physical health and recovery from poor psychological wellbeing (as indicated by psychological distress) over time. We found no evidence that adopting a wide range of health and wellbeing practices is associated with improvements in physical health or that health and wellbeing practices prevent deterioration in psychological wellbeing or protect those individuals at risk due to exposure to workplace psychosocial hazards.

### 4.1. Implications for Research

The present study has extended previous research on extensive approaches to employee health and wellbeing [11,12] by linking adoption of practices to individual physical health and psychological wellbeing rather than perceptions of program effectiveness, reports of practices use [11] or perceptions of others’ wellbeing in the organization [12]. The results indicated that more extensive approaches to employee health and wellbeing and supporting infrastructure (governance, management support, communications strategy) tend to co-occur in organizations, suggesting organizations that adopt an extensive range of health and wellbeing practices generally do so adopting a coherent and strategic approach. The results indicated that a single continuum is the major differentiator between organizations. However, PCA did indicate some evidence of a minor, second dimension and a CFA indicated reasonable but not good fit for a single dimension. Although the second component was categorized by cross-loadings, it may still be feasible that a second dimension can account for additional, albeit relatively small, variation between organizations. Further evidence suggesting the presence of one or more smaller dimensions was found in Supplementary Material (analyses of T2 data). However, at this point, it is not clear what other dimensions may represent and further conceptual and/or case study research may be needed to explore this issue.

Our results indicated differential directionality in effects on reports of physical health and psychological wellbeing, by preventing the deterioration of physical health status for those with good physical health at baseline, and rehabilitative effects on psychological distress. In respect of physical heath, extensive adoption may create norms around healthy behaviors, as well as providing a range of resources to engage in healthy behaviors. Future research may explore whether the effects on maintaining physical health can be accounted for by changing norms or through access to resources.

In respect of recovery from poor psychological wellbeing, Huettermann and Bruch [12] proposed that extensive approaches to psychological health signal organizational care to employees, in turn promoting positive attitudes to stress, which in turn promotes adaptive responses to stress and wellbeing. Integrating Huettermann and Bruch’s findings with the findings from the present study, we suggest any positive attitudes to stress associated with health and wellbeing practices develop after harm to psychological wellbeing has occurred, rather than as a protective factor for those with good wellbeing. This is because the effect on psychological wellbeing was specific to recovery from poor wellbeing and there was no evidence that health and wellbeing practices buffer the effects of individuals’ workplace psychosocial hazards on wellbeing. It may be that extensive health and wellbeing programs provide signals that encourage help seeking through both formal and informal routes once problems have developed. Additional factors, either organizational or individual, may also encourage proactive help seeking before problems develop. However, an important implication from the current findings is that we found no evidence that extensive workplace health and wellbeing programs can be used to offset risk from workplace psychosocial hazards, although specific practices may do so notwithstanding integration into a wider program [6] (e.g., resilience training, mindfulness).

Our results indicated that the relationship between the organizational adoption of practices and subsequent health and wellbeing does not level off after a threshold has been reached (cf. [11]), but that organizations with the most extensive adoption of practices are more likely to have better subsequent employee health and wellbeing outcomes. Batorsky et al. [11] assessed perceptions of program effectiveness and patterns of use, rather than effects on markers of health and wellbeing. It may be the case that there is a threshold of activity required to make workers aware of a program and to engage with it, but the more extensive approaches are more likely to provide resources that can be tailored to individual needs.

In the present study, we have focused on self-reported physical health (measured by a single item) and psychological wellbeing (indexed through a measure of psychological distress). As noted earlier, two previous cross-sectional studies did not use direct markers of health or wellbeing. Therefore, future research may usefully examine a wider range of health, wellbeing and productivity outcomes, such as absence, and use more extensive measurements. Research on productivity outcomes could examine whether any effects of extensive adoption of health and wellbeing practices are mediated by wellbeing, as might be predicted by social exchange theory [38].

Another area for future research is to examine the role of psychosocial safety climate [39], which refers to shared perceptions of policies and practices for employee health and wellbeing. Dollard and Karasek [39] argued that health and wellbeing activities are easier to implement where a psychosocial safety climate is well developed and there is also evidence that implementing specific health and wellbeing interventions can lead to changes in workplace attitudes to health and wellbeing [40]. Thus, we might expect reciprocal relationships between psychosocial safety climate and the implementation and evolution of workplace health and wellbeing programs, such that one reinforces the other. This line of reasoning could also be extended to individuals’ experience of workplace psychosocial hazards, in so far that developing a more extensive program may lead to the implementation of more practices focused on primary prevention through minimization of individuals’ exposure to workplace psychosocial hazards.

Finally, it may be worth investigating the extent to which employee physical health and psychological wellbeing are reciprocally related to organizational approaches to health and wellbeing. In a review of reciprocal relationships between workplace psychosocial hazards and psychological wellbeing, Tang [41] described potential mechanisms through which employee health and wellbeing may be antecedent to workplace health and wellbeing programs. The health selection hypothesis would apply when employees with poor health or wellbeing ‘drift’ into workplaces with low provision for health and wellbeing. In contrast, the refuge hypothesis would predict that workers with poor physical health or psychological wellbeing (or concerns around these issues) would seek out workplaces with good health and wellbeing provision. A variant of this Refuge Hypothesis is that employees and/or managers seek out opportunities to address employee health and wellbeing concerns, such that management action is taken to address those concerns. Finally, the perception hypothesis would apply when employees with poor psychological well-being perceive the provision of health and wellbeing services as inadequate.

### 4.2. Strengths and Limitations

A strength of the current study is the use of a longitudinal, multisource data that enabled us to assess whether organizational approaches to health and wellbeing could predict changes in employee reports of physical health and psychological wellbeing. As well as enhancing causal inference by assessing changes in outcomes, the multisource and longitudinal data removes concerns over common method bias as data on the independent variables and dependent variables were collected from different sources [42]. Moreover, as data were collected on organizational possession of different activities, strategies, and supporting infra-structure, rather than employee awareness or use data, the hypothesis tests are conservative.

As with all data, the data were collected in a specific context (UK) and historical period (e.g., before UK’s exit from the European Union). There are questions therefore our conclusions apply to different contexts and periods, especially a post-COVID-19 context. Our results are broadly in line (although with important differences) with the results of two previous studies, indicating health and wellbeing benefits for workplace health and programs with multiple components. However, each of these studies was conducted in developed economies and the UK has universal healthcare funded through general taxation. It may be the case that workplace health and wellbeing programs become even more influential on employee health and wellbeing outcomes where there are salient risks to health (e.g., COVID-19 as a risk to mental as well as physical health) or where state provision of healthcare is less extensive than in the UK.

Although the sample size at the employee level is relatively large at T1 and T2 (N > 1100 in all analyses), the number of organizations at both waves of data collection (N = 58) may have limited statistical power. Two previous cross-sectional studies in this area also had relatively small sample sizes of organizations (N = 81, N = 88) [11,12] and our cross-sectional analyses were based on a sample of N = 146. The use of smaller samples of organizations underscores the resource intensive nature of data collection for the previous studies and the present study. In the present study, the advantages of using longitudinal data to build on findings from the two previous studies and study changes in outcomes necessitated a smaller sample size. The use of small samples of organizations in this and previous studies on health and wellbeing practices does indicate that research linking organizational data with employee data is most likely to detect stronger effects. Other methodologies may be required to investigate smaller effect sizes. Moreover, our analyses were based on Bayesian estimation with uninformative priors. This reflects that there are only two previous studies [11,12], both of which are cross-sectional and neither of which assessed markers of physical or psychological health. Although Bayesian estimation can be useful for model estimation with small sample sizes, uninformative priors can be problematic [43]. In the present study, we used multiple robustness checks, including other estimation methods (Appendix B), mitigating against problems with uninformative priors. However, future research could use the results of the present study to specify more informative priors.

As noted in the previous section, our measure of physical health was based on a single-item self-report scale and therefore future research should consider using more extensive, multi-item measures of physical health. Using a single-item measure might have limitations compared to multi-item measures. However, as noted earlier, there is evidence for the reliability and validity of single item measures of health [29,30] (see also [44,45,46]).

Although we used multisource data, the data on health and wellbeing activities came from a single source and from organizations that volunteered to opt into data collection rather than through contact with a research team. These issues raise concerns about the representativeness of data and reliability of the reports. Similar concerns over representativeness could be raised with the two previous cross-sectional studies, given samples of organizations of less than 100. However, if the sample were biased towards organizations with more extensive adoption of health and wellbeing practices, this would lead to more conservative hypothesis tests due to range restriction. In relation to the reliability of reports of health and wellbeing activities, similar to the present study, Huettermann and Bruch [12] used single HR manager reports in their study. The use of single informant reports of organizational practices is not uncommon in organizational research (e.g., analyses of the UK’s Workplace Employment Relations Survey have single informant reports of organizational practices) [47] and single informant reports are not considered problematic where informants can be considered knowledgeable of organizational practices [48], as in the present study and that of Huettermann and Bruch [12].

## 5. Conclusions

We have found evidence that the extensive adoption of workplace health and wellbeing practices can have beneficial effects on employees’ experiences of physical health and psychological wellbeing. However, although workplace health and wellbeing practices may help to prevent deterioration of experienced physical health, we found no evidence of a protective effect on psychological wellbeing, thus indicating that health and wellbeing practices cannot be used to offset risks to psychological health from how working practices are managed.

## Figures and Tables

**Table 1:** Descriptive statistics.

	M	SD
T1 Values		
1. Governance	0.50	0.38
2. Benefits	51.71	36.21
3. Flexible time	49.00	41.72
4. Accumulated overtime	% (yes) = 47	
5. Medical services	0.38	0.36
6. Smoking cessation	0.18	0.21
7. Drinking support	0.31	0.32
8. Physical health	0.49	0.28
9. Healthy eating	0.46	0.32
10. Psych. problems	0.42	0.35
11. Service promotion	0.48	0.32
12. Health promotion	0.35	0.24
13. Management support	3.99	0.55
14. Job autonomy	3.15	1.07
15. Bullying	1.20	0.54
16. Time pressure	2.52	1.03
17. Role clarity	4.07	0.95
18. Poor physical health	1.96	0.78
19. Psychological distress	0.61	0.63
T2 Values		
20. Poor physical health	1.97	0.78
21. Psychological distress	0.67	0.66

**Table 2:** Correlations.

	1	2	3	4	5	6	7	8	9	10	11	12	13	14	15	16	17	18	19	20	21
T1 values																					
1. Governance	−																				
2. Benefits	21 *	−																			
3. Flexible time	19 *	28 **	−																		
4. Accumulated overtime	11	−06	−02	−																	
5. Medical services	44 **	36 **	18 *	01	−																
6. Smoking cessation	54 **	32 **	12	11	61 **	−															
7. Drinking support	38 **	21 *	09	18 *	39 **	43 **	−														
8. Physical health	55 **	30 **	21 *	25 **	48 **	47 **	37 **	−													
9. Healthy eating	65 **	31 **	25 **	06	61 **	64 **	38 **	63 **	−												
10. Psych. problems	52 **	25 **	22 **	14	55 **	53 **	51 **	58 **	59 **	−											
11. Service promotion	61 **	22 *	15	23 **	47 **	54 **	50 **	64 **	67 **	53 **	−										
12. Health promotion	58 **	27 **	15	13	50 **	53 **	48 **	67 **	64 **	59 **	76 **	−									
13. Management support	49 **	21 *	24 **	18 *	42 **	42 **	42 **	56 **	51 **	64 **	53 **	67 **	−								
14. Job autonomy	20	00	10	−10	−10	10	10	10	20	20	20	20	32*	−	−14 **	−11 **	18 **	−15 **	−19 **	−13 **	−16 **
15. Bullying	20	20	−10	27*	00	00	00	00	−10	−10	10	−10	−30	−51 **	−	23 **	−19 **	11 **	24 **	10 **	21 **
16. Time pressure	00	00	00	10	00	−10	10	−20	−10	00	00	−10	−10	−34 **	37 **	−	−22 **	14 **	22 **	10 **	16 **
17. Role clarity	00	10	10	−10	10	27 *	10	20	20	27 *	20	28 *	34 **	43 **	−40 **	−53 **	−	−13 **	−25 **	−11 **	−21 **
18. Poor physical health	−20	−10	−26 *	10	−10	−37 **	−10	−46 **	−36 **	−29 *	−20	−30	−29 *	−40 **	32 *	49 **	−50 **	−	35 **	63 **	30 **
19. Psychological distress	00	−10	−20	10	−20	−29*	−20	−20	−30*	−35 **	−20	−10	−20	−40 **	31 *	30 *	−40 **	62 **	−	31 **	68 **
T2 values																					
20. Poor physical health	−30 *	−30	−34 **	10	−20	−48 **	−20	−50 **	−38 **	−42 **	−33 *	−34 **	−38 **	−42 **	20	30	−39 **	83 **	0.59 **	−	35 **
21. Psychological distress	−20	−20	−10	00	−30	−40 **	−20	−30	−30 *	−45 **	−29 *	−20	−27 *	−29 *	00	10	−40 **	52 **	0.73 **	0.59 **	−

* *p* < 0.05, ** *p* < 0.01. Significant correlations ranged from |0.10| (1% of shared variance) to |0.83| (69% of shared variance). Values below primary diagonal are correlations aggregated at the organizational level, those above based on individual level data.

**Table 3:** Results of the latent profile analyses.

No. Categories	L H0 Value	AIC	BIC	c1	c2	c3	c4	c5	Entropy	BLRT	VLMR
1	−3112.17	6274.35	6348.94								
2	−2822.58	5723.15	5839.51	0.49	0.51				0.89	0.40	0.39
3	−2706.90	5519.81	5677.94	0.36	0.21	0.43			0.92	0.01	0.01
4	−2655.64	5445.28	5645.18	0.36	0.36	0.10	0.18		0.95	0.19	0.20
5 *	−2618.73	5399.46	5641.13	0.35	0.38	0.05	0.16	0.06	0.96	0.46	0.47

L H0: Loglikelihood Value; AIC: Akaike’s information criterion; BIC: Bayesian Information Criterion; cx: Proportion of sample in category X; BLRT: bootstrapped likelihood ratio; VLMR: Vuong-Lo-Mendell-Rubin adjusted likelihood ratio. * Note that this solution was not identified.

**Table 4:** Regression coefficients of multilevel probit linear and curvilinear regression analyses with Bayesian estimation.

Health/Wellbeing Status at T1	Good Physical Health		Poor Physical Health		Low Psych. Distress		High Psych. Distress	
	N = 5546		N = 1422		N = 5563		N = 1113	
Criterion Categorical Variable T2	Poor Physical Health		Poor Physical Health		High Psych. Distress		High Psych. Distress	
	*B*	*B*	*B*	*B*	*B*	*B*	*B*	*B*
	Linear	Curvilinear	Linear	Curvilinear	Linear	Curvilinear	Linear	Curvilinear
	Model	Model	Model	Model	Model	Model	Model	Model
Individual predictors T1								
Poor physical health	0.97 **	0.97 **	0.82 **	0.82 **	0.14 **	0.14 **	0.16 **	0.16 **
Psychological distress	0.23 **	0.22 **	0.17 **	0.17 **	1.61 **	1.61 **	0.79 **	0.80 **
Job autonomy	−0.07 **	−0.07 **	−0.05	−0.05	−0.06 **	−0.06 **	0.03	0.03
Bullying	0.03	0.03	0.05	0.05	0.11 *	0.11 *	0.11	0.10
Time pressure	−0.03	−0.03	0.00	0.00	−0.03	−0.03	−0.04	−0.05
Role clarity	−0.03	−0.03	0.04	0.03	−0.08 **	−0.08 **	−0.08	−0.08
Organizational predictors T1								
Health and wellbeing practices ^†^	−0.08 **	−0.09 **	−0.05	−0.06	0.01	0.02	−0.12 *	−0.13 **
Health and wellbeing practices squared		−0.03		0.03		−0.01		−0.10 **
R^2^								
Within organizations	0.22	0.22	0.14	0.13	0.25	0.25	0.22	0.22
Between organizations	0.43	0.54	0.32	0.52	0.06	0.19	0.49	0.71

* *p* < 0.05, ** *p* < 0.01. ^†^ Represented by the component scores for the first and largest component derived from Principal Components Analysis. Further details of these analyses can be obtained by contacting the first author and through public access at the University of East Anglia institutional repository.

**Table 5:** 95% credibility intervals of regression coefficients and variance accounted for multilevel probit linear and curvilinear regression analyses with Bayesian estimation.

Health/Wellbeing Status at T1	Good Physical Health		Poor Physical Health		Low Psych. Distress		High Psych. Distress	
	N = 5546		N = 1422		N = 5563		N = 1113	
Criterion Categorical Variable T2	Poor Physical Health		Poor Physical Health		High Psych. Distress		High Psych. Distress	
	95% CI	95% CI	95% CI	95% CI	95% CI	95% CI	95% CI	95% CI
	Linear	Curvilinear	Linear	Curvilinear	Linear	Curvilinear	Linear	Curvilinear
	Model	Model	Model	Model	Model	Model	Model	Model
Individual predictors T1								
Poor physical health	0.84/1.10	0.84/1.10	0.59/1.07	0.60/1.06	0.07/0.20	0.07/0.20	0.06/0.26	0.06/0.26
Psychological distress	0.14/0.31	0.14/0.31	0.06/0.27	0.06/0.27	1.44/1.78	1.45/1.78	0.61/0.99	0.62/1.00
Job autonomy	−0.12/−0.02	−0.12/−0.02	−0.11/0.02	−0.11/0.02	−0.11/−0.01	−0.11/−0.01	−0.05/0.10	−0.05/0.10
Bullying	−0.07/0.12	−0.07/0.12	−0.06/0.16	−0.06/0.16	0.02/0.19	0.01/0.20	−0.01/0.23	−0.01/0.22
Time pressure	−0.08/0.02	−0.08/0.02	−0.07/0.07	−0.07/0.07	−0.09/0.01	−0.09/0.02	−0.13/0.04	−0.12/0.03
Role clarity	−0.08/0.02	−0.09/0.02	−0.04/0.11	−0.04/0.11	−0.14/−0.03	−0.14/−0.03	−0.17/0.01	−0.17/0.00
Organizational predictors T1								
Health and wellbeing practices ^†^	−0.15/−0.02	−0.16/−0.02	−0.13/0.03	−0.14/0.02	−0.04/0.07	−0.04/0.07	−0.22/−0.02	−0.24/−0.03
Health and wellbeing practices squared		−0.08/0.02		−0.10/0.04		−0.06/0.03		−0.20/−0.02
R^2^								
Within organizations	0.18/0.26	0.18/0.26	0.09/0.20	0.09/0.19	0.21/0.28	0.21/0.28	0.15/0.20	0.16/0.29
Between organizations	0.04/0.92	0.24/0.92	0.01/0.92	0.03/0.95	0.02/0.97	0.26/0.98	0.00/0.49	0.01/0.73

95% CI is 95% credibility interval on each regression coefficient or R^2^ value. Bayesian credibility intervals can be interpreted in a similar manner to confidence intervals derived from other estimators. One key difference between credibility and confidence intervals is that credibility intervals are not symmetrical around the point estimate [34].

## Data Availability

The data used in this study were provided by Vitality. Please contact Martin Stepanek (martin.stepanek@vitality.co.uk) for questions concerning data availability.

## Supplementary Materials

The following are available online at https://www.mdpi.com/article/10.3390/ijerph18178964/s1, Analyses of T2 data.

## Appendix A

**Table A1:** List of the items used for measuring the organizational practice adoption.

Indicator	Item	Scale
Benefit	What percentage of full-time employees receives Private Medical Insurance?	Percentage ranging from 0 to 100
	2. What percentage of full-time employees receives other health benefits (e.g., cash plan or dental cover)	
	3. What percentage of part-time employees receives Private Medical Insurance?	
	4. What percentage of part-time employees receives other health benefits (e.g., cash plan or dental cover)	
Flexible time	Approximately what percentage of employees have the possibility to adapt—within certain limits—the time when they begin or finish their daily work according to their personal needs or wishes? (This question refers to what is often called ‘flexitime’, where employees choose their starting time, for instance, between 7:00 and 10:00 and finishing time, for instance, between 15:00 and 18:00.)	Percentage ranging from 0 to 100
Accumulated overtime	Is it possible for employees to use accumulated overtime for days off? This can be full or half days.	0 = No; 1 = Yes
Health and Wellbeing programs	*Are any of the following wellness facilities, services or programs available to employees through your company?*	0 = No; 1 = Yes
Medical services	1. Clinical screening (e.g., blood glucose, blood pressure)	
	2. Disease management (management of long-term conditions such as diabetes, asthma, chronic obstructive pulmonary disease)	
	3. On-site health clinics / medical services	
Smoking cessation	4. Smoking cessation information	
	5. Smoking cessation program	
	6. Discounted / free nicotine patches or medication provided by your company	
	7. Discounted / free nicotine patches or medications provided by health insurer	
	8. Discounted / free smoking cessation programs provided by your company	
	9. Discounted / free smoking cessation programs provided by health insurer	
	10. Other assistance to quit smoking	
Drinking support	11. Information on problem drinking and options on seeking help	
	12. Counselling	
	13. Support groups	
Physical health	14. Onsite gym or fitness facility	
	15. Offsite gym / health club membership discount	
	16. Unpaid fitness breaks	
	17. Paid fitness breaks (fitness classes)	
	18. Other exercise opportunities (walking trails, inviting staircases, etc.)	
Healthy eating	19. Healthy eating information	
	20. Healthy food alternatives at canteens	
	21. Healthy food alternatives in vending machines	
	22. Fresh fruit and vegetables in the workplace	
	23. Dietician/nutritionist services	
Psychological problems	24. Stress management programs	
	25. Stress management information	
	26. Work-life balance programs	
	27. Training on identifying and reducing workplace stress	
Service promotion	*Are you currently actively promoting the wellness facilities, services or programs to your employees?* 1. Outbound communications from senior leadership	0 = No; 1 = Yes
	2. Newsletters or emails to promote program or campaigns	
	3. Mailed letters, post cards, or materials	
	4. Visual reminders at worksite (posters, flyers, video displays, etc.)	
	5. Development of peer-based champions to promote to promote programs and initiatives	
Health promotion	*Our working environment supports employees in their health promotion efforts by* 1. Providing incentives for participation	0 = No; 1 = Yes
	2. Recognizing or rewarding employees for healthy behavior and health improvement	
	3. Allowing participation in activities/events during work time	
	4. Distributing regular messages from senior management regarding policies and practices that support healthy behaviors and health promotion	
	5. Providing managers with performance objectives and/or goals related to worksite health improvement	
	6. Providing managers with training on the importance of employee health promotion	
	7. Providing employee referrals to community resources (e.g., hotlines, screening clinics)	
	8. Developing and publicizing a program theme or logo for health improvement	
	9. Having company leaders who champion the program by acting as role models	
	10. Providing program access to spouses and/or family members	
Active governance	1. Does your organization have a committee responsible for employee health promotion and wellness?	0 = No; 1 = Yes
	2. Do you provide wellness days where employees have health checks and get advice on improving their wellbeing?	
	3. In the past year, has your organization asked for employee feedback on the types of health promotion programs and services that employees felt would be beneficial to them?	
Management support	*Below are statements that illustrate different aspects of work environment and culture at your workplace. We ask that your ratings of agreement with each statement describe your work in the last six months.* 1. Our employees receive the respect at work they deserve	1 = Strongly disagree; 2 = Disagree; 3 = Neutral; 4 = Agree; 5 = Strongly agree
	2. Our employees are always consulted about change at work.	
	3. Our line manager / senior leadership encourage employees at work.	
	4. Our line manager / senior leadership care about health and wellbeing.	
	5. All levels of employees are informed about the importance of staff health and wellbeing.	
	6. Our organization provides line managers with training on staff health and wellbeing	
	7. Our organization supports staff who return to work after an illness.	
	8. Our organization refers staff with poor health to an occupational health provider (internal or external).	
	9. Our health benefits and insurance programs support health promotion.	
	10. Our leaders view the level of employee health and wellbeing as one important indicator of the organization’s success.	

**Table A2:** List of the items used for measuring employee variables.

Indicator	Item	Scale
Psychosocial hazards	1. I am subject to bullying at work	1 Never 2 Seldom 3 Sometimes 4 Often 5 Always
	2. I have a choice in deciding what I do at work	
	3. I have unrealistic time pressures.	
	4. I am clear what my duties and responsibilities are	
Wellbeing Kessler scale	In this step, we ask questions about how you have been feeling during the past 30 days. For each question, please select the option that best describes how often you had this feeling. During the last 30 days, about how often did you feel: 1. nervous 2. hopeless 3. restless or fidgety 4. that everything was an effort 5. so depressed that nothing could cheer you up 6. worthless	0 None of the time 1 A little of the time 2 Some of the time 3 Most of the time 4 All of the time
Physical health	In this step, we ask you about your| overall health and wellbeing. This includes your height and weight, existing medical conditions, and other measures you may have had checked, such as blood pressure and cholesterol. 1. How is your physical health in general?	0 Very good 1 Good 2 Fair 3 Bad 4 Very bad

## Appendix B

### Appendix B.1. Robustness Checks

To examine whether the choice of Bayesian estimation led to divergence with other estimation methods, we examined hypotheses H2a–H4b using multilevel logistic regression implemented using both the HLM-7 and SPSS software. The results yielded the same inferences with respect to the hypotheses, i.e., support for H2a for self-reported physical health only, H2b for psychological distress only, no support for H3a–H4b and a curvilinear effect of practices such that rates of recovery from high levels of psychological distress were highest in organizations that adopted the widest range of practices.

Using Bayesian estimation, we examined various alternative models. Unless otherwise stated, we used the same modelling procedures (e.g., Mplus defaults for uninformative priors) as models reported in the main text. Further details of all analyses reported in this Appendix (full results, syntax for Mplus models) can be obtained by contacting the first author and through public access at the University of East Anglia institutional repository.

### Appendix B.2. Maximum Likelihood Estimation

Using maximum likelihood estimation, we examined the linear effects of organizational adoption of practices on physical health as a dichotomous variable at T2 for those with good health at T1 (replication of support for H2a) and the linear and curvilinear effects of adoption of practices on psychological distress as a dichotomous variable at T2 for those with high levels of psychological distress at T1. These models contained no control variables, given convergence issues with maximum likelihood estimation with some of the more extensive models. Nevertheless, both models captured the transition from good physical health/high psychological distress to poor physical health/low psychological distress from T1 to T2. We found support for the relationship with physical health (H2a, B = −0.16, *p* < 0.01) and psychological distress (H2b, linear effects B = −0.22, *p* < 0.01, curvilinear effect B = −0.15, *p* < 0.01).

### Appendix B.3. Not Dichotomizing Psychological Distress and Physical Health at T1

Instead of dichotomizing psychological distress and physical health at T1 to create sub-samples for the analysis of improvement or deterioration in psychological distress and physical health, we conducted cross-level moderated regression analyses, in which organizational adoption of practices moderated the relationship of psychological distress and physical health at T1 on their respective outcomes at T2. In these analyses, psychological distress and physical health at T1 were centered at the organizational means and their regression slopes at the individual level allowed to vary across organizations. Average values of psychological distress and physical health for each organization at T1 were also included as predictors. Organizational adoption of practices was then regressed onto the individual level regression slopes of T1 physical health or psychological distress on the T2 values of the relevant outcome. These procedures are the same for testing cross-level interactions as those to examine H4a and H4b. Although there was a significant main effect of physical health at T1 on T2 values (B = 0.60, *p* < 0.01), there was no evidence of moderation (B = −0.01, ns). In this case, although adoption of practices was associated with less poor physical health at T2 (B = −0.02, *p* < 0.05), the was no evidence of differential effectiveness for either those with good or poor physical health. Given the majority of the sample reported good physical health, the results can be interpreted as not contradicting support for Hypothesis 2a for physical health (Table 4). For psychological distress, there was a significant main effect of the T1 values on T2 values (B = 0.65, *p* < 0.01) moderated by the adoption of practices (B = −0.04, *p* < 0.05), which indicates those with high distress are more likely to benefit from extensive adoption of practices than those with low distress. These results do not contradict support for Hypothesis 2b (Table 4).

### Appendix B.4. Comparing Findings from the Analyses with the First Principal Component with Analogous Analyses Conducted Using Categories Derived from the Latent Profile Analysis (LPA)

In all but one of the analyses, replicating those reported in the main text but using the categories identified by LPA, none of the categories were related to any outcomes either directly or in moderating the effects of individuals’ workplace psychosocial hazards. The exception in the analyses on deterioration of reports of physical health from T1 to T2. In this analysis, workers reporting good health at T1 were more likely to report good health at T2 if they worked in organizations in the category with the most extensive adoption of practices (*p* < 0.05), which replicates findings reported in Table 4, in that the extensive adoption of practices was associated with the maintenance of good health over time. Therefore, compared to analyses in which the adoption of health and wellbeing practices is treated as a continuum, we find little evidence that categories of the adoption of practices are related to physical health or psychological distress.

### Appendix B.5. Controls for Organizational Size and Sector

We ran analyses analogous to those reported in Table 4 that reported significant effects for the adoption of practices, but including controls for organizational size (dummy variable comparing organizations with 500 of fewer employees versus more than 500 employees) and sector (coded as dummy variables: financial and professional services (38% of sample); other knowledge intensive sectors (19%), e.g., life sciences; manufacturing (7%); reference category—other (36%)). The results replicated those in Table 4 in respect of sustained levels of physical health at T2 for those with good physical health at T1 (Hypothesis 2a, B = −0.11, *p* < 0.01,) and improvements in psychological wellbeing at T2 for those with poor wellbeing at T1 (Hypothesis 2b, linear model, B = −0.13, *p* < 0.05; curvilinear model: linear effect B = −0.14, *p* < 0.01, quadratic effect B = −0.11, *p* < 0.01).

### Appendix B.6. Exploring Whether the Findings Can Be Explained by Adoption of a Single Practice or Smaller Set of Practices Adopted as Part of a Larger Set of Practices

First, we conducted analyses analogous to those reported in Table 4 but using single practices instead of the composite indicator from the first principal component for the PCA. Separate analyses were conducted for each practice in isolation, thus representing a liberal approach to examining an alternative explanation for the pattern of results reported in Table 4, namely whether adoption of a single practice or smaller set of practices could explain the pattern of results. For recovery from poor psychological wellbeing, we used the linear and curvilinear terms of each practice. We found that maintenance of good physical health over time (Hypothesis 2a) was associated with active wellbeing governance, practices targeted at physical health, practices targeted at psychological problems, support for health promotion, management cultures and flexible working (*p* < 0.05). We found recovery from poor psychological wellbeing (Hypothesis 2b) was associated with curvilinear terms representing support for health promotion and management cultures (*p* < 0.05). At the next step of the analyses, we performed regressions with two latent variables. The first was a composite of all practices, which replicates using the first principal component in the main analyses. The second was a latent variable indicated by only one of the single practices found to be associated with health or wellbeing categories at T2. This second latent variable therefore represented the unique effects of a practice that is unrelated to a wider and managed program of activities. Separating the variables in this way overcomes problems of multicollinearity given large correlations between each practice and the first principal component derived from PCA. Latent curvilinear terms for both factors were used the analyses for poor psychological wellbeing. In each case, the composite latent variable representing all practices was related to the health and wellbeing outcomes, replicating the results reported in Table 4. We then repeated the analyses with two latent variables, one latent variable representing the composite of all practices and the second latent variable a composite of those single practices found to have a significant relationship with outcomes. Latent curvilinear terms for both factors were again used in the analyses for poor psychological wellbeing. In the analyses, the composite representing all the practices (for physical health) or the curvilinear term for the composite representing all the practices (for psychological wellbeing) was significantly related to outcomes in the direction consistent with coefficients reported in Table 4 (although in one of three analyses for recovery from poor psychological wellbeing *p* < 0.06), but the composite representing the smaller number of practices was not.
