# Supplementary material for: Do Multicomponent Workplace Health and Wellbeing Programs Predict Changes in Health and Wellbeing?
Source: Int J Environ Res Public Health. 2021 Aug 25;18(17):8964. doi: 10.3390/ijerph18178964 (PMC8430978; doi:10.3390/ijerph18178964)
Supplement: Supplementary file 1 [file ijerph-1222591-supplementary.pdf › ijerph-1222591-supplementary.pdf]

Contents:

Latent profile, principal components and confirmatory factor analyses results from 2016	2
Mplus syntax for probit regressions	3
SPSS logistic regression results	9
HLM-7 regression results	351
Mplus results	400

Latent profile analyses

No. categories	L H0 Value	AIC	BIC	c1	c2	c3	c4	c5	Entropy	BLRT	VLMR
1	-2284.24	4618.48	4695.83	163							
2	-2009.32	4096.64	4217.30	88	75				.92	.01	.01
3*	-1899.06	3904.11	4068.08	82	68	13			.95	.02	.02
4*	-1857.00	3847.99	4055.27	57	12	34	60		.89	.15	.15
5*	-1824.65	3811.31	4061.90	57	34	39	21	12	.88	.59	.61

L H0: Loglikelihood Value; AIC: Akaike's information criterion; BIC: Bayesian Information Criterion; cx: Proportion of sample in category X; BLRT: bootstrapped likelihood ratio; VLMR: Vuong-Lo-Mendell-Rubin adjusted likelihood ratio.
* Note solution had estimation problems

Principal components and confirmatory factor analysis

	Unrotated PCA loadings 2015			CFA loadings 2015
	1st component	2nd component	3rd component	
Management support	.66	.21	.29	1.00 (fixed)
Governance	.22	.75	.13	0.45*
Benefits	.29	-.55	.53	0.67**
Flexible time	.29	.14	.51	0.79**
Accumulated overtime	.32	.42	-.55	0.90**
Medical services	.67	-.47	-.23	2.37**
Smoking cessation	.74	-.22	-.30	1.38**
Drinking support	.73	-.21	-.35	1.54**
Physical health	.77	.07	.07	1.52**
Healthy eating	.83	-.01	-.02	1.81**
Psych. problems	.78	.03	.04	1.85**
Service promotion	.72	.22	.10	1.63**
Health promotion	.76	.10	.21	1.31**
	Three components with eigenvalues > 1<br>1st component = 40.37% variance<br>2nd component = 11.32% variance<br>3rd component = 9.76% variance			Confirmatory Fit Index = 0.85; Root Mean Square Error of Approximation = 0.11

Mplus syntax

Basic linear model:

```
categorical = phycat16;          !!DEFINES OUTCOME AS CATEGORICAL
missing ARE ALL (-9999);

WITHIN = phy15 kes15 choice15 bully15 press15 duties15;
BETWEEN = prafac;
cluster = cId15;

DEFINE:
prafacsq = prafac * prafac;  !! Calculates curvilinear terms for wellbeing practices if included in the model
CENTER phy15 kes15 choice15 bully15 press15 duties15 (GRANDMEAN);
!! These are level 1 control variables
ANALYSIS:
TYPE = TWOLEVEL RANDOM;
ESTIMATOR = BAYES;
THIN = 20;
CHAINS = 3;
BCONVERGENCE = .01;
BITERATIONS = (1000);

MODEL:

%WITHIN%
phycat16 ON phy15 choice15 bully15 duties15 press15;
phycat16 ON kes15;

kes15 WITH phy15 choice15 bully15 press15 duties15;
```

```
phy15 WITH choice15 bully15 press15 duties15;
choice15 WITH bully15 press15 duties15;
bully15 WITH press15 duties15;
press15 WITH duties15;

  %BETWEEN%
  phycat16 ON prafac;
```

Example of moderation test

```
categorical = phycat16;
missing ARE ALL (-9999);

categorical = phycat16;          !!DEFINES OUTCOME AS CATEGORICAL
missing ARE ALL (-9999);

WITHIN = phy15 kes15 choice15 bully15 press15 duties15;
BETWEEN = prafac;
cluster = cId15;

DEFINE:
prafacsq = prafac * prafac;  !! Calculates curvilinear terms for wellbeing practices if included in the model
CENTER phy15 kes15 bully15 press15 duties15 (GRANDMEAN);
!! These are level 1 control variables, note variable in the moderation test is not included in this command so that the default of group mean
centering is used for that variable

ANALYSIS:
TYPE = TWOLEVEL RANDOM;
ESTIMATOR = BAYES;
THIN = 20;
CHAINS = 3;
BCONVERGENCE = .01;
BITERATIONS = (1000);

MODEL:

%WITHIN%
phycat16 ON phy15 bully15 duties15 press15;
phycat16 ON kes15;
s1 | phycat16 ON choice15;
```

```
kes15 WITH phy15 choice15 bully15 press15 duties15;
phy15 WITH choice15 bully15 press15 duties15;
choice15 WITH bully15 press15 duties15;
bully15 WITH press15 duties15;
press15 WITH duties15;

%BETWEEN%
phycat16 ON prafac choice15;
prafac WITH choice15;
s1 ON prafac;
```

Example of robustness test, separating out effects of single practices from the combined effects of multiple practices

```
categorical = phycat16;
missing ARE ALL (-9999);

WITHIN = phy15 kes15 choice15 bully15 press15 duties15;
BETWEEN = ACC ACT EAT ALC PHY PSY SMO TER BEN HPR SPR CUL FEL;
cluster = cId15;

DEFINE:
single = ACC;
CENTER phy15 kes15 choice15 bully15 press15 duties15 (GRANDMEAN);

ANALYSIS:
TYPE = TWOLEVEL RANDOM;
ESTIMATOR = BAYES;
THIN = 20;
CHAINS = 3;
BCONVERGENCE = .01;
BITERATIONS = (1000);

MODEL:

%WITHIN%
phycat16 ON phy15 choice15 bully15 duties15 press15;
phycat16 ON kes15;

kes15 WITH phy15 choice15 bully15 press15 duties15;
phy15 WITH choice15 bully15 press15 duties15;
choice15 WITH bully15 press15 duties15;
bully15 WITH press15 duties15;
press15 WITH duties15;
```

```
%BETWEEN%
PROG BY ACC ACT EAT ALC PHY PSY SMO TER BEN HPR SPR CUL FEL;
!!This is a latent variable comprising all health and wellbeing practices
SING BY HPR; !!This is a latent variable representing a single practice
phycat16 ON PROG SING;
PROG WITH SING;
```

SPSS logistic regression results

Variable labels

Kescat15 – Kessler dichotomised for selecting cases for analysis (1=high distress)

Kescat16 – Kessler dichotomised as outcome (1=high distress)

Phycat15 – Physical health dichotomised for selecting cases for analysis (1=poor physical health)

Phycat16 – Physical health dichotomised as outcome (1=poor physical health)

Physical_health - 2105 physical health, continuous variable

Kess_t15 – 2015 Kessler score, continuous variable

choice_freq – job control

bullying_freq - bullying

time_pressure_freq – time pressure

duties_freq – role clarity

FAC1_1 – first principal component of health and wellbeing practices

Facsq – FAC1_1 * FAC1_1

```
* Encoding: UTF-8.

COMPUTE facsq = FAC1_1 * FAC1_1.

USE ALL.
COMPUTE filter_$=(kescat15 = 0).
VARIABLE LABELS filter_$ 'kescat15 = 0 (FILTER)'.
VALUE LABELS filter_$ 0 'Not Selected' 1 'Selected'.
FORMATS filter_$ (f1.0).
FILTER BY filter_$.
EXECUTE.

correlations /variables = kescat16 kescat15 fac1_1.
```

Correlations

Notes

Output Created		16-AUG-2021 11:07:25
Comments		
Input	Data	C:\Users\jwb12ppu\Documents\Kevin\MPlus backup\Multicomponent health and wellbeing v2\Multicomponent for Mplus cat.sav
	Active Dataset	DataSet2
	Filter	kescat15 = 0 (FILTER)
	Weight	<none>

	Split File	<none>
	N of Rows in Working Data File	5855
Missing Value Handling	Definition of Missing	User-defined missing values are treated as missing.
	Cases Used	Statistics for each pair of variables are based on all the cases with valid data for that pair.
Syntax		correlations /variables = kescat16 kescat15 fac1_1.
Resources	Processor Time	00:00:00.02
	Elapsed Time	00:00:00.04

Correlations

		Kessler16 > 1 = 1	Kessler15 > 1 = 1	REGR factor score 1 for analysis 1
Kessler16 > 1 = 1	Pearson Correlation	1	.[a]	-.012
	Sig. (2-tailed)		.	.344
	N	5855	5855	5855
Kessler15 > 1 = 1	Pearson Correlation	.[a]	.[a]	.[a]
	Sig. (2-tailed)	.		.
	N	5855	5855	5855
REGR factor score 1 for analysis 1	Pearson Correlation	-.012	.[a]	1
	Sig. (2-tailed)	.344	.	
	N	5855	5855	5855

a. Cannot be computed because at least one of the variables is constant.

```
*Generalized Linear Mixed Models.
GENLINMIXED
  /DATA_STRUCTURE SUBJECTS=companyId15
  /FIELDS TARGET=kescat16 TRIALS=NONE OFFSET=NONE
  /TARGET_OPTIONS DISTRIBUTION=BINOMIAL LINK=LOGIT
  /FIXED  EFFECTS=physical_health kess_t15 choice_freq bullying_freq time_pressure_freq duties_freq
    FAC1_1 USE_INTERCEPT=TRUE
  /RANDOM USE_INTERCEPT=TRUE SUBJECTS=companyId15 COVARIANCE_TYPE=VARIANCE_COMPONENTS SOLUTION=TRUE
  /BUILD_OPTIONS TARGET_CATEGORY_ORDER=DESCENDING INPUTS_CATEGORY_ORDER=DESCENDING
    MAX_ITERATIONS=100 CONFIDENCE_LEVEL=95 DF_METHOD=RESIDUAL COVB=ROBUST PCONVERGE=0.000001(ABSOLUTE)
    SCORING=0 SINGULAR=0.000000000001
  /EMMEANS_OPTIONS SCALE=ORIGINAL PADJUST=LSD.
```

Generalized Linear Mixed Models

Notes

Output Created		16-AUG-2021 11:07:25
Comments		
Input	Data	C:\Users\jwb12ppu\Documents\Kevin\MPlus backup\Multicomponent health and wellbeing v2\Multicomponent for Mplus cat.sav
	Active Dataset	DataSet2
	Filter	kescat15 = 0 (FILTER)

	Weight	<none>
	Split File	<none>
	N of Rows in Working Data File	5855

Syntax	GENLINMIXED<br>/DATA_STRUCTURE<br>SUBJECTS=companyId15<br>/FIELDS TARGET=kescat16<br>TRIALS=NONE OFFSET=NONE<br>/TARGET_OPTIONS<br>DISTRIBUTION=BINOMIAL<br>LINK=LOGIT<br>/FIXED<br>EFFECTS=physical_health<br>kess_t15 choice_freq bullying_freq<br>time_pressure_freq duties_freq<br>FAC1_1<br>USE_INTERCEPT=TRUE<br>/RANDOM<br>USE_INTERCEPT=TRUE<br>SUBJECTS=companyId15<br>COVARIANCE_TYPE=VARIANCE<br>_COMPONENTS<br>SOLUTION=TRUE<br>/BUILD_OPTIONS<br>TARGET_CATEGORY_ORDER=<br>DESCENDING<br>INPUTS_CATEGORY_ORDER=D<br>ESCENDING<br>MAX_ITERATIONS=100<br>CONFIDENCE_LEVEL=95<br>DF_METHOD=RESIDUAL

		COVB=ROBUST PCONVERGE=0.000001(ABSOLUTE) SCORING=0 SINGULAR=0.000000000001 /EMMEANS_OPTIONS SCALE=ORIGINAL PADJUST=LSD.
Resources	Processor Time	00:00:01.80
	Elapsed Time	00:00:01.85

Warnings

glmm: One or more records are not used in the analysis because they have one or more fields with invalid or missing values.
glmm: Valid values for events (target) and trials variables are non-negative and positive integers respectively, and the number of trials cannot be less than the number of events.

Case Processing Summary

	N	Percent
Included	5339	91.2%
Excluded	516	8.8%
Total	5855	100.0%

Model Summary

Target		Kessler16 > 1 = 1
Probability Distribution		Binomial
Link Function		Logit
Information Criterion	Akaike Corrected	29406.079
	Bayesian	29412.659

Information criteria are based on the -2 log likelihood (29404.078) and are used to compare models. Models with smaller information criterion values fit better.

Data Structure[a]

	Subjects company_code	Target Kessler16 > 1 = 1
Data for First Subject	2	.00
	2	.00
	2	.00
	2	.00
	2	1.00
	2	1.00
	2	.00
	2	.00
	2	.00
	2	1.00
Total Number of Levels	58	

Only the first 10 records are displayed.

a. Target: Kessler16 > 1 = 1

Classification
Overall Percent Correct = 88.8%[a]

Observed		Predicted 1.00	.00
1.00	Count	18	583
	% within Observed	3.0%	97.0%
.00	Count	15	4723
	% within Observed	0.3%	99.7%

a. Target: Kessler16 > 1 = 1

Fixed Effects[a]

Source	F	df1	df2	Sig.
Corrected Model	167.653	7	5331	.000
physical_health	33.715	1	5331	.000
kess_t15	575.934	1	5331	.000
choice_freq	4.516	1	5331	.034
bullying_freq	6.654	1	5331	.010
time_pressure_freq	2.064	1	5331	.151
duties_freq	4.472	1	5331	.034
FAC1_1	.672	1	5331	.412

Probability distribution: Binomial

Link function: Logit

a. Target: Kessler16 > 1 = 1

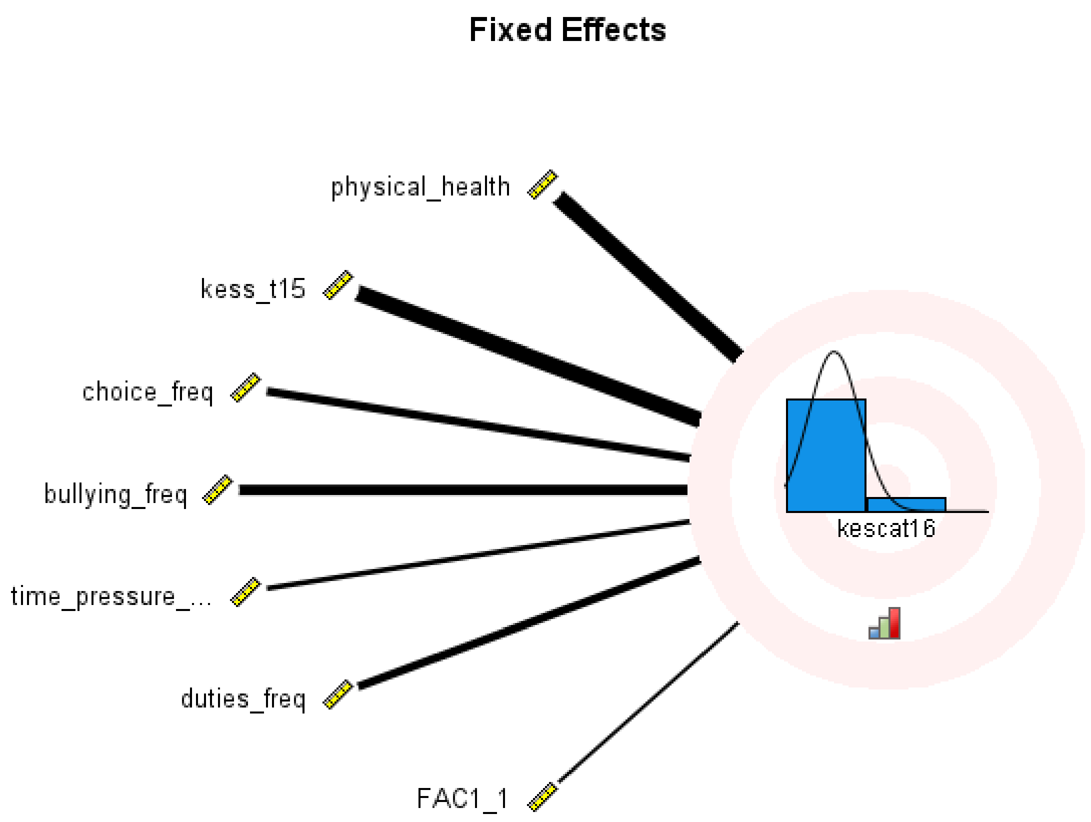

Fixed Coefficients[a]

Model Term	Coefficient	Std. Error	t	Sig.	95% Confidence Interval		Exp(Coefficient)	95% Confidence Interval for Exp(Coefficient)	
					Lower	Upper		Lower	Upper
Intercept	-3.182	.4691	-6.783	.000	-4.101	-2.262	.042	.017	.104
physical_health	.241	.0415	5.806	.000	.159	.322	1.272	1.173	1.380
kess_t15	3.019	.1258	23.999	.000	2.773	3.266	20.479	16.002	26.207
choice_freq	-.097	.0458	-2.125	.034	-.187	-.008	.907	.829	.992
bullying_freq	.199	.0771	2.579	.010	.048	.350	1.220	1.049	1.419
time_pressure_freq	-.065	.0450	-1.437	.151	-.153	.024	.937	.858	1.024
duties_freq	-.151	.0713	-2.115	.034	-.291	-.011	.860	.748	.989
FAC1_1	.035	.0426	.820	.412	-.049	.119	1.036	.953	1.126

Probability distribution: Binomial

Link function: Logit

a. Target: Kessler16 > 1 = 1

Fixed Coefficients

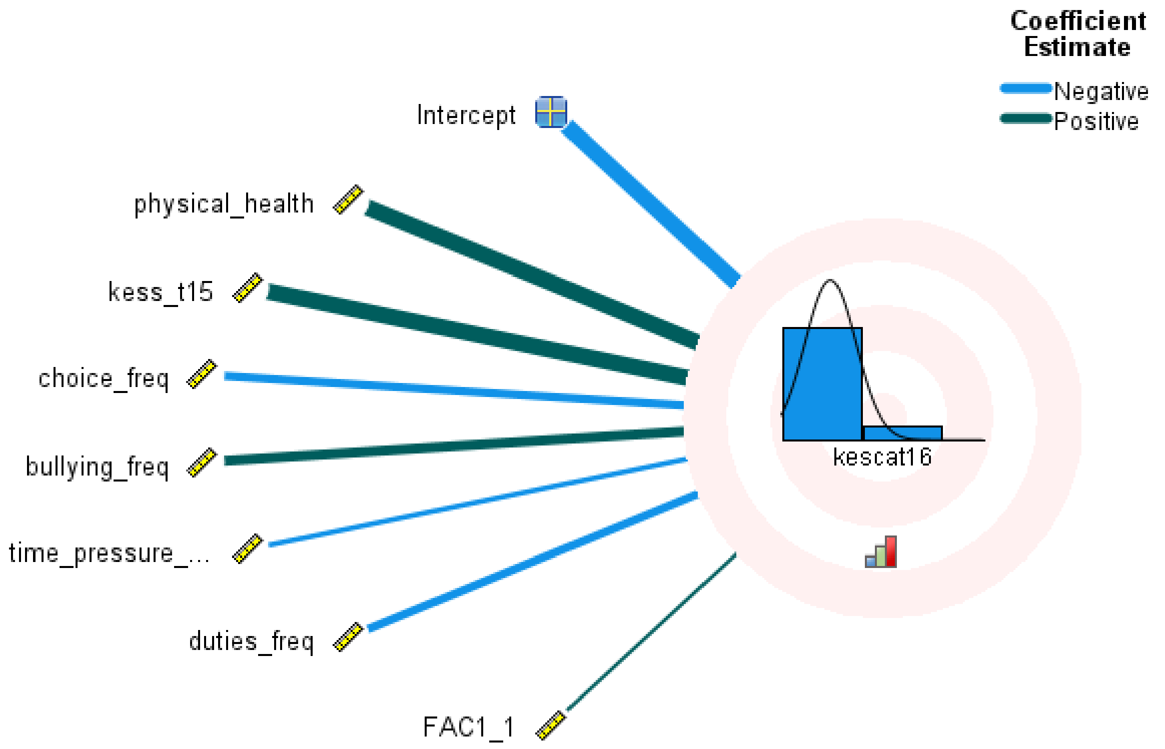

Random Effect Covariances

Random Effect Block 1

Random Effect Block	Intercept
Intercept	.021

Covariance Structure: Variance components

Subject Specification: companyId15

Covariance Parameters

Covariance Parameters Summary

Covariance Parameters	Residual Effect	0
	Random Effects	1
Design Matrix Columns	Fixed Effects	8
	Random Effects	1[a]
Common Subjects		58

Common subjects are based on the subject specifications for the residual and random effects and are used to chunk the data for better performance.

a. This is the number of columns per common subject.

Residual Effect

Residual Effect	Estimate	Std. Error	Z	Sig.	95% Confidence Interval	
					Lower	Upper
Variance	1.000	.	.	.	.	.

Covariance Structure: Scaled Identity

Subject Specification: (None)

Random Effect

Random Effect Covariance	Estimate	Std. Error	Z	Sig.	95% Confidence Interval	
					Lower	Upper
Var(Intercept)	.021	.019	1.063	.288	.003	.130

Covariance Structure: Variance components

Subject Specification: companyId15

Random Effects Parameters

Empirical Best Linear Unbiased Predictions[a]

company_code	Parameter	Prediction	Std. Error	df	Sig.	95% Confidence Interval	
						Lower Bound	Upper Bound
2	Intercept	.007	.134	5331	.961	-.256	.269

4	Intercept	.011	.138	5331	.938	-.259	.280
6	Intercept	.003	.136	5331	.980	-.264	.271
22	Intercept	-.009	.143	5331	.949	-.289	.271
24	Intercept	-.029	.126	5331	.817	-.276	.218
30	Intercept	-.005	.139	5331	.973	-.277	.268
36	Intercept	.037	.136	5331	.787	-.230	.304
40	Intercept	.084	.137	5331	.541	-.185	.353
50	Intercept	.007	.139	5331	.962	-.267	.280
61	Intercept	-.028	.137	5331	.836	-.297	.240
65	Intercept	.021	.141	5331	.884	-.256	.297
68	Intercept	-.037	.136	5331	.786	-.304	.230
69	Intercept	.030	.136	5331	.823	-.237	.298
71	Intercept	.033	.140	5331	.814	-.242	.308
72	Intercept	-.007	.140	5331	.959	-.281	.267
74	Intercept	-.037	.140	5331	.792	-.312	.238
75	Intercept	-.041	.138	5331	.769	-.312	.231
80	Intercept	.018	.142	5331	.902	-.261	.296
81	Intercept	-.014	.138	5331	.921	-.284	.257
83	Intercept	.023	.138	5331	.870	-.248	.293
84	Intercept	-.020	.139	5331	.888	-.292	.253
92	Intercept	-.003	.137	5331	.980	-.271	.264
93	Intercept	-.003	.137	5331	.980	-.272	.265
98	Intercept	.066	.133	5331	.621	-.195	.327
100	Intercept	.062	.096	5331	.519	-.126	.250
106	Intercept	.021	.139	5331	.881	-.252	.293
107	Intercept	-.015	.138	5331	.912	-.286	.255

108	Intercept	-.026	.140	5331	.854	-.300	.248
110	Intercept	-.172	.104	5331	.096	-.375	.031
114	Intercept	.022	.138	5331	.872	-.248	.293
116	Intercept	.039	.135	5331	.776	-.227	.304
122	Intercept	.063	.130	5331	.627	-.192	.319
125	Intercept	-.039	.122	5331	.748	-.279	.200
142	Intercept	-.064	.132	5331	.625	-.322	.194
144	Intercept	.010	.135	5331	.938	-.254	.275
145	Intercept	.004	.136	5331	.978	-.263	.271
152	Intercept	.005	.136	5331	.971	-.261	.271
154	Intercept	-.033	.136	5331	.808	-.299	.233
156	Intercept	.036	.134	5331	.787	-.227	.299
162	Intercept	.030	.137	5331	.825	-.238	.298
165	Intercept	.022	.138	5331	.876	-.249	.293
166	Intercept	-.028	.142	5331	.846	-.306	.250
169	Intercept	-.047	.138	5331	.730	-.317	.223
170	Intercept	.054	.139	5331	.697	-.218	.326
173	Intercept	-.047	.138	5331	.735	-.317	.224
174	Intercept	-.018	.138	5331	.897	-.287	.252
178	Intercept	.019	.134	5331	.889	-.244	.282
181	Intercept	.040	.140	5331	.774	-.234	.315
182	Intercept	.018	.140	5331	.895	-.256	.293
192	Intercept	-.037	.139	5331	.791	-.310	.236
202	Intercept	.058	.132	5331	.660	-.201	.318
204	Intercept	-.147	.124	5331	.237	-.390	.097
205	Intercept	-.020	.141	5331	.885	-.297	.256

206	Intercept	-.011	.138	5331	.936	-.281	.259
209	Intercept	.038	.124	5331	.756	-.204	.281
217	Intercept	.132	.113	5331	.245	-.090	.355
226	Intercept	-.021	.138	5331	.877	-.292	.250
228	Intercept	-.053	.140	5331	.704	-.327	.220

a. Target: Kessler16 > 1 = 1

```
GENLINMIXED
  /DATA_STRUCTURE SUBJECTS=companyId15
  /FIELDS TARGET=kescat16 TRIALS=NONE OFFSET=NONE
  /TARGET_OPTIONS DISTRIBUTION=BINOMIAL LINK=LOGIT
  /FIXED  EFFECTS=physical_health kess_t15 choice_freq bullying_freq time_pressure_freq duties_freq
    FAC1_1 facsq USE_INTERCEPT=TRUE
  /RANDOM USE_INTERCEPT=TRUE SUBJECTS=companyId15 COVARIANCE_TYPE=VARIANCE_COMPONENTS SOLUTION=TRUE
  /BUILD_OPTIONS TARGET_CATEGORY_ORDER=DESCENDING INPUTS_CATEGORY_ORDER=DESCENDING
    MAX_ITERATIONS=100 CONFIDENCE_LEVEL=95 DF_METHOD=RESIDUAL COVB=ROBUST PCONVERGE=0.000001(ABSOLUTE)
    SCORING=0 SINGULAR=0.000000000001
  /EMMEANS_OPTIONS SCALE=ORIGINAL PADJUST=LSD.
```

Generalized Linear Mixed Models

Notes

Output Created	16-AUG-2021 11:07:27
Comments	

Input	Data	C:\Users\jwb12ppu\Documents\Kevin\MPlus backup\Multicomponent health and wellbeing v2\Multicomponent for Mplus cat.sav
	Active Dataset	DataSet2
	Filter	kescat15 = 0 (FILTER)
	Weight	<none>
	Split File	<none>
	N of Rows in Working Data File	5855

Syntax	GENLINMIXED<br>/DATA_STRUCTURE SUBJECTS=companyId15<br>/FIELDS TARGET=kescat16 TRIALS=NONE OFFSET=NONE<br>/TARGET_OPTIONS DISTRIBUTION=BINOMIAL LINK=LOGIT<br>/FIXED EFFECTS=physical_health kess_t15 choice_freq bullying_freq time_pressure_freq duties_freq<br>FAC1_1 facsq USE_INTERCEPT=TRUE<br>/RANDOM USE_INTERCEPT=TRUE SUBJECTS=companyId15 COVARIANCE_TYPE=VARIANCE_COMPONENTS SOLUTION=TRUE<br>/BUILD_OPTIONS TARGET_CATEGORY_ORDER=DESCENDING INPUTS_CATEGORY_ORDER=DESCENDING<br>MAX_ITERATIONS=100 CONFIDENCE_LEVEL=95 DF_METHOD=RESIDUAL

		COVB=ROBUST PCONVERGE=0.000001(ABSOLUTE) SCORING=0 SINGULAR=0.000000000001 /EMMEANS_OPTIONS SCALE=ORIGINAL PADJUST=LSD.
Resources	Processor Time	00:00:02.09
	Elapsed Time	00:00:02.02

Warnings

glmm: One or more records are not used in the analysis because they have one or more fields with invalid or missing values.
glmm: Valid values for events (target) and trials variables are non-negative and positive integers respectively, and the number of trials cannot be less than the number of events.

Case Processing Summary

	N	Percent
Included	5339	91.2%
Excluded	516	8.8%
Total	5855	100.0%

Model Summary

Target		Kessler16 > 1 = 1
Probability Distribution		Binomial
Link Function		Logit
Information Criterion	Akaike Corrected	29409.337
	Bayesian	29415.917

Information criteria are based on the -2 log likelihood (29407.336) and are used to compare models. Models with smaller information criterion values fit better.

Data Structure[a]

	Subjects<br>company_code	Target<br>Kessler16 > 1 = 1
Data for First Subject	2	.00
	2	.00
	2	.00
	2	.00
	2	1.00
	2	1.00
	2	.00
	2	.00
	2	.00
	2	1.00
Total Number of Levels	58	

Only the first 10 records are displayed.

a. Target: Kessler16 > 1 = 1

Classification

Overall Percent Correct = 88.8%[a]

Observed		Predicted 1.00	.00
1.00	Count	18	583
	% within Observed	3.0%	97.0%
.00	Count	14	4724
	% within Observed	0.3%	99.7%

a. Target: Kessler16 > 1 = 1

Fixed Effects[a]

Source	F	df1	df2	Sig.
Corrected Model	149.705	8	5330	.000
physical_health	33.511	1	5330	.000
kess_t15	576.846	1	5330	.000
choice_freq	4.387	1	5330	.036
bullying_freq	6.637	1	5330	.010
time_pressure_freq	2.120	1	5330	.145
duties_freq	4.469	1	5330	.035
FAC1_1	.570	1	5330	.450
facsq	.306	1	5330	.580

Probability distribution: Binomial

Link function: Logit

a. Target: Kessler16 > 1 = 1

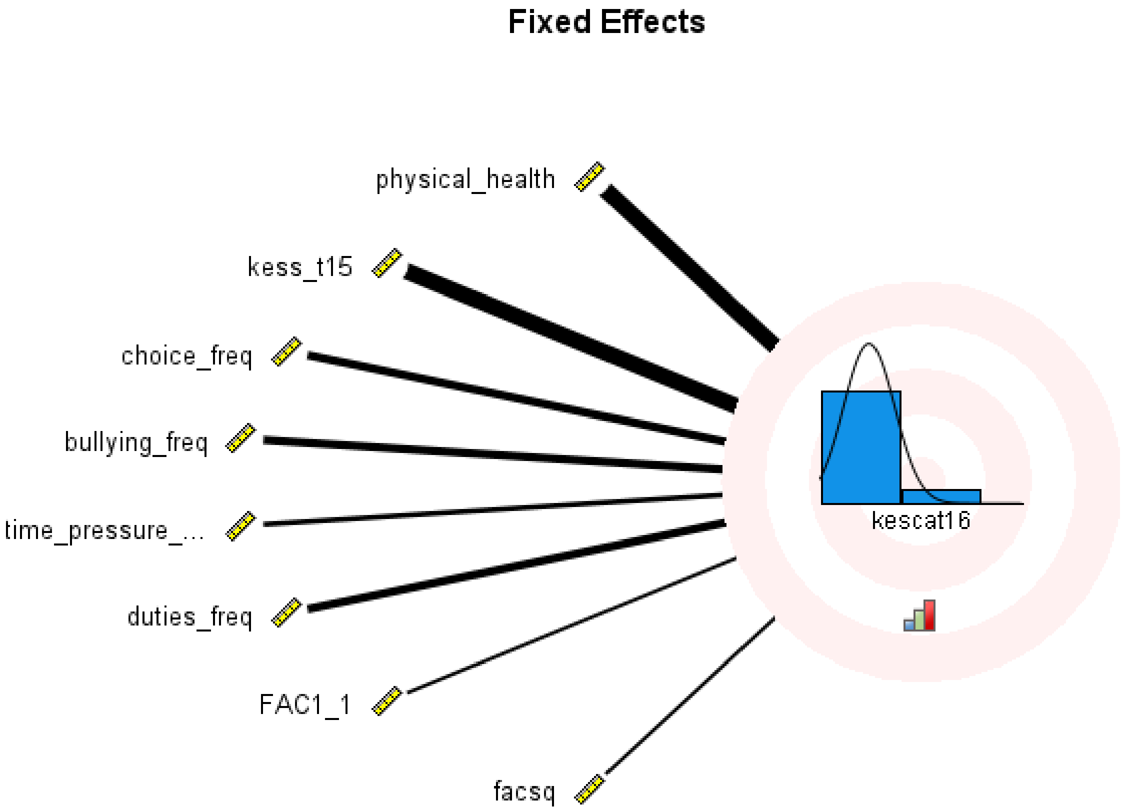

Fixed Coefficients[a]

Model Term	Coefficient	Std. Error	t	Sig.	95% Confidence Interval		Exp(Coefficient)	95% Confidence Interval for Exp(Coefficient)	
					Lower	Upper		Lower	Upper
Intercept	-3.162	.4693	-6.737	.000	-4.082	-2.242	.042	.017	.106
physical_health	.240	.0415	5.789	.000	.159	.321	1.271	1.172	1.379
kess_t15	3.019	.1257	24.018	.000	2.773	3.266	20.475	16.003	26.197
choice_freq	-.096	.0460	-2.095	.036	-.187	-.006	.908	.830	.994
bullying_freq	.199	.0771	2.576	.010	.047	.350	1.220	1.049	1.419
time_pressure_freq	-.065	.0448	-1.456	.145	-.153	.023	.937	.858	1.023
duties_freq	-.151	.0713	-2.114	.035	-.291	-.011	.860	.748	.989
FAC1_1	.032	.0426	.755	.450	-.051	.116	1.033	.950	1.123
facsq	-.018	.0325	-.553	.580	-.082	.046	.982	.922	1.047

Probability distribution: Binomial

Link function: Logit

a. Target: Kessler16 > 1 = 1

Fixed Coefficients

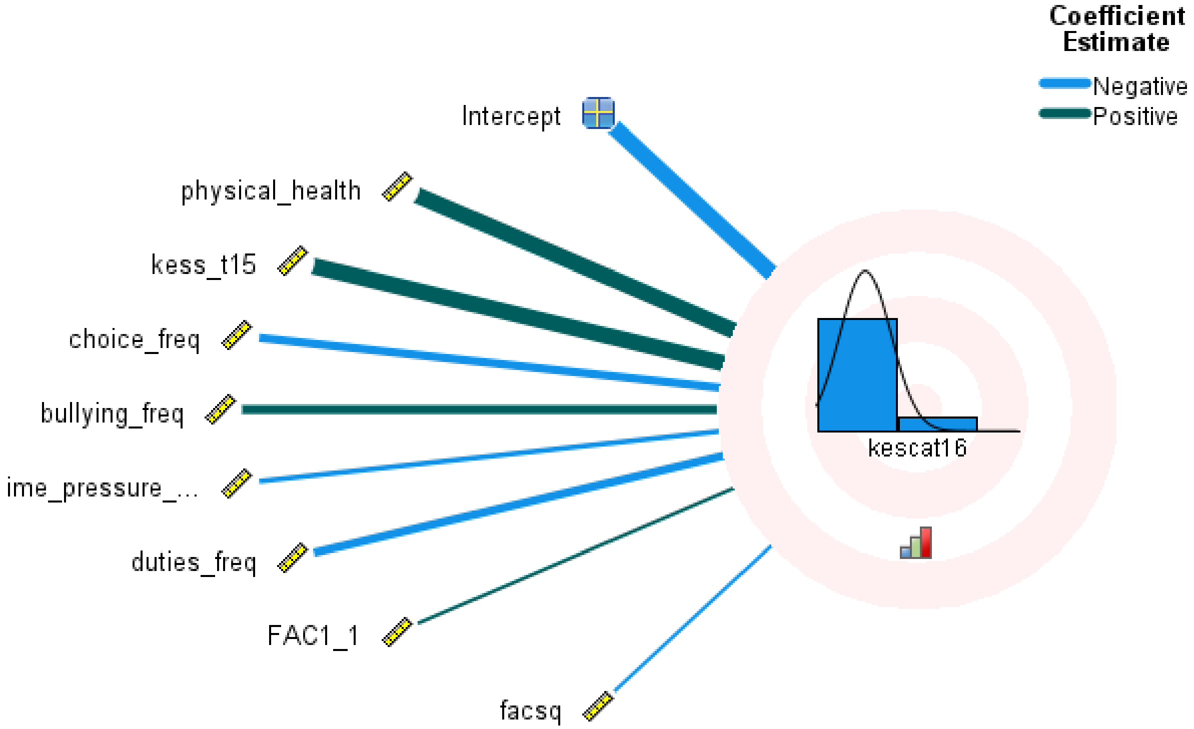

Random Effect Covariances

Random Effect Block 1

Random Effect Block	Intercept
Intercept	.021

Covariance Structure: Variance components
Subject Specification: companyId15

Covariance Parameters

Covariance Parameters Summary

Covariance Parameters	Residual Effect	0
	Random Effects	1
Design Matrix Columns	Fixed Effects	9
	Random Effects	1[a]
Common Subjects		58

Common subjects are based on the subject specifications for the residual and random effects and are used to chunk the data for better performance.
a. This is the number of columns per common subject.

Residual Effect

Residual Effect	Estimate	Std. Error	Z	Sig.	95% Confidence Interval	
					Lower	Upper
Variance	1.000	.	.	.	.	.

Covariance Structure: Scaled Identity

Subject Specification: (None)

Random Effect

Random Effect Covariance	Estimate	Std. Error	Z	Sig.	95% Confidence Interval	
					Lower	Upper
Var(Intercept)	.021	.020	1.058	.290	.003	.135

Covariance Structure: Variance components

Subject Specification: companyId15

Random Effects Parameters

Empirical Best Linear Unbiased Predictions[a]

company_code	Parameter	Prediction	Std. Error	df	Sig.	95% Confidence Interval	
						Lower Bound	Upper Bound
2	Intercept	.008	.136	5330	.951	-.258	.274

4	Intercept	.018	.140	5330	.897	-.256	.293
6	Intercept	.009	.139	5330	.947	-.262	.281
22	Intercept	-.009	.145	5330	.948	-.293	.275
24	Intercept	-.031	.127	5330	.810	-.280	.219
30	Intercept	-.003	.141	5330	.982	-.279	.273
36	Intercept	.036	.138	5330	.795	-.234	.306
40	Intercept	.085	.139	5330	.542	-.188	.357
50	Intercept	.006	.141	5330	.966	-.271	.283
61	Intercept	-.028	.139	5330	.838	-.300	.243
65	Intercept	.024	.143	5330	.868	-.257	.304
68	Intercept	-.039	.138	5330	.776	-.309	.231
69	Intercept	.029	.138	5330	.832	-.242	.300
71	Intercept	.035	.142	5330	.804	-.243	.313
72	Intercept	-.005	.142	5330	.972	-.283	.273
74	Intercept	-.037	.142	5330	.792	-.316	.241
75	Intercept	-.040	.140	5330	.773	-.315	.234
80	Intercept	.018	.144	5330	.902	-.264	.300
81	Intercept	-.015	.140	5330	.912	-.289	.258
83	Intercept	.024	.140	5330	.861	-.249	.298
84	Intercept	-.021	.141	5330	.880	-.297	.255
92	Intercept	-.002	.138	5330	.991	-.273	.269
93	Intercept	2.226E-5	.139	5330	1.000	-.273	.273
98	Intercept	.070	.135	5330	.606	-.195	.334
100	Intercept	.053	.099	5330	.594	-.142	.248
106	Intercept	.020	.141	5330	.886	-.256	.296
107	Intercept	-.017	.140	5330	.903	-.291	.257

108	Intercept	-.022	.142	5330	.874	-.301	.256
110	Intercept	-.174	.104	5330	.095	-.379	.030
114	Intercept	.022	.140	5330	.877	-.252	.296
116	Intercept	.038	.137	5330	.782	-.231	.307
122	Intercept	.061	.132	5330	.642	-.197	.320
125	Intercept	-.039	.123	5330	.754	-.280	.203
142	Intercept	-.068	.133	5330	.607	-.329	.192
144	Intercept	.014	.137	5330	.916	-.253	.282
145	Intercept	.002	.138	5330	.989	-.268	.272
152	Intercept	.009	.137	5330	.951	-.261	.278
154	Intercept	-.034	.137	5330	.803	-.303	.235
156	Intercept	.039	.136	5330	.773	-.227	.306
162	Intercept	.033	.138	5330	.814	-.239	.304
165	Intercept	.024	.140	5330	.862	-.250	.299
166	Intercept	-.029	.144	5330	.841	-.311	.253
169	Intercept	-.043	.140	5330	.761	-.317	.232
170	Intercept	.054	.140	5330	.698	-.221	.329
173	Intercept	-.049	.140	5330	.728	-.323	.225
174	Intercept	-.020	.139	5330	.887	-.293	.253
178	Intercept	.017	.136	5330	.903	-.250	.283
181	Intercept	.041	.142	5330	.775	-.237	.318
182	Intercept	.018	.142	5330	.897	-.259	.296
192	Intercept	-.036	.141	5330	.799	-.313	.241
202	Intercept	.058	.134	5330	.663	-.204	.321
204	Intercept	-.155	.126	5330	.219	-.402	.092
205	Intercept	-.021	.143	5330	.881	-.302	.259

206	Intercept	-.013	.139	5330	.927	-.286	.261
209	Intercept	.034	.125	5330	.786	-.212	.280
217	Intercept	.130	.115	5330	.256	-.095	.356
226	Intercept	-.022	.140	5330	.873	-.297	.252
228	Intercept	-.056	.141	5330	.694	-.332	.221

a. Target: Kessler16 > 1 = 1

```
AGGREGATE
  /OUTFILE=* MODE=ADDVARIABLES OVERWRITE = YES
  /BREAK=companyId15
  /choicem1=MEAN(choice_freq)
  /bullym1=MEAN(bullying_freq)
  /timem1=MEAN(time_pressure_freq)
  /dutiesm1=MEAN(duties_freq).

COMPUTE choicecwc1 = choice_freq - choicem1.
COMPUTE bullycwc1 = bullying_freq - bullym1.
COMPUTE timecwc1 = time_pressure_freq - timem1.
COMPUTE dutiescwc1 = duties_freq - dutiesm1.

COMPUTE fac1choi = fac1_1 * choicecwc1.
COMPUTE fac1bull = fac1_1 * bullycwc1.
COMPUTE fac1time = fac1_1 *  timecwc1.
COMPUTE fac1duti = fac1_1 * dutiescwc1.

GENLINMIXED
  /DATA_STRUCTURE SUBJECTS=companyId15
  /FIELDS TARGET=kescat16 TRIALS=NONE OFFSET=NONE
  /TARGET_OPTIONS DISTRIBUTION=BINOMIAL LINK=LOGIT
  /FIXED  EFFECTS=physical_health kess_t15 choicecwc1 choicem1 fac1choi
  bullying_freq time_pressure_freq duties_freq
    FAC1_1 USE_INTERCEPT=TRUE
  /RANDOM USE_INTERCEPT=TRUE SUBJECTS=companyId15 COVARIANCE_TYPE=VARIANCE_COMPONENTS SOLUTION=TRUE
  /BUILD_OPTIONS TARGET_CATEGORY_ORDER=DESCENDING INPUTS_CATEGORY_ORDER=DESCENDING
    MAX_ITERATIONS=100 CONFIDENCE_LEVEL=95 DF_METHOD=RESIDUAL COVB=ROBUST PCONVERGE=0.000001(ABSOLUTE)
    SCORING=0 SINGULAR=0.000000000001
  /EMMEANS_OPTIONS SCALE=ORIGINAL PADJUST=LSD.
```

Generalized Linear Mixed Models

Notes

Output Created		16-AUG-2021 11:07:29
Comments		
Input	Data	C:\Users\jwb12ppu\Documents\Kevin\MPlus backup\Multicomponent health and wellbeing v2\Multicomponent for Mplus cat.sav
	Active Dataset	DataSet2
	Filter	kescat15 = 0 (FILTER)
	Weight	<none>
	Split File	<none>
	N of Rows in Working Data File	5855

Syntax	GENLINMIXED<br>/DATA_STRUCTURE SUBJECTS=companyId15<br>/FIELDS TARGET=kescat16 TRIALS=NONE OFFSET=NONE<br>/TARGET_OPTIONS DISTRIBUTION=BINOMIAL LINK=LOGIT<br>/FIXED EFFECTS=physical_health kess_t15 choicecwc1 choicem1 fac1choi<br>bullying_freq time_pressure_freq duties_freq<br>FAC1_1 USE_INTERCEPT=TRUE<br>/RANDOM USE_INTERCEPT=TRUE SUBJECTS=companyId15 COVARIANCE_TYPE=VARIANCE_COMPONENTS SOLUTION=TRUE<br>/BUILD_OPTIONS TARGET_CATEGORY_ORDER=DESCENDING INPUTS_CATEGORY_ORDER=DESCENDING

		MAX_ITERATIONS=100<br>CONFIDENCE_LEVEL=95<br>DF_METHOD=RESIDUAL<br>COVB=ROBUST<br>PCONVERGE=0.000001(ABSOLUTE)<br>SCORING=0<br>SINGULAR=0.000000000001<br>/EMMEANS_OPTIONS<br>SCALE=ORIGINAL<br>PADJUST=LSD.
Resources	Processor Time	00:00:01.97
	Elapsed Time	00:00:01.97

Warnings

glmm: One or more records are not used in the analysis because they have one or more fields with invalid or missing values.
glmm: Valid values for events (target) and trials variables are non-negative and positive integers respectively, and the number of trials cannot be less than the number of events.

Case Processing Summary

	N	Percent
Included	5339	91.2%

Excluded	516	8.8%
Total	5855	100.0%

Model Summary

Target		Kessler16 > 1 = 1
Probability Distribution		Binomial
Link Function		Logit
Information Criterion	Akaike Corrected	29410.821
	Bayesian	29417.402

Information criteria are based on the -2 log likelihood (29408.821) and are used to compare models. Models with smaller information criterion values fit better.

Data Structure[a]

	Subjects<br>company_code	Target<br>Kessler16 > 1 = 1
Data for First Subject	2	.00
	2	.00
	2	.00
	2	.00
	2	1.00
	2	1.00
	2	.00
	2	.00
	2	.00

	2	1.00
Total Number of Levels	58	

Only the first 10 records are displayed.

a. Target: Kessler16 > 1 = 1

Classification
Overall Percent Correct = 88.8%[a]

Observed		Predicted 1.00	.00
1.00	Count	18	583
	% within Observed	3.0%	97.0%
.00	Count	14	4724
	% within Observed	0.3%	99.7%

a. Target: Kessler16 > 1 = 1

Fixed Effects[a]

Source	F	df1	df2	Sig.
Corrected Model	130.547	9	5329	.000
physical_health	33.912	1	5329	.000
kess_t15	570.175	1	5329	.000
choicecwc1	4.133	1	5329	.042
choicem1	.110	1	5329	.740
fac1choi	.005	1	5329	.942
bullying_freq	6.619	1	5329	.010

time_pressure_freq	2.120	1	5329	.145
duties_freq	4.452	1	5329	.035
FAC1_1	.524	1	5329	.469

Probability distribution: Binomial

Link function: Logit

a. Target: Kessler16 > 1 = 1

Fixed Effects

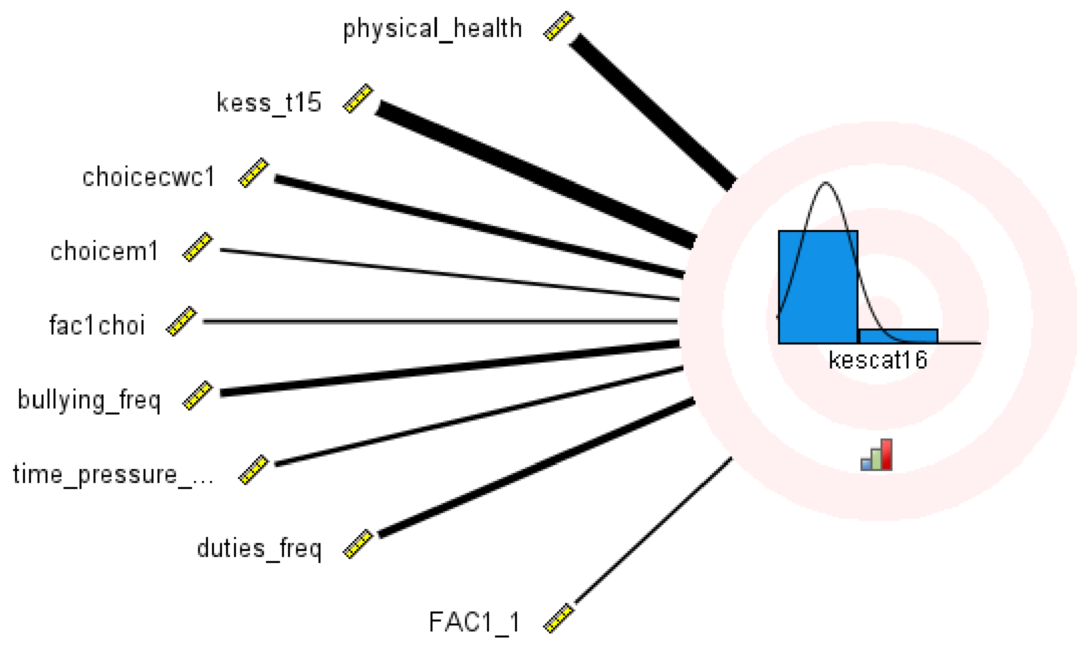

Fixed Coefficients[a]

Model Term	Coefficient	Std. Error	t	Sig.	95% Confidence Interval		Exp(Coefficient)	95% Confidence Interval for Exp(Coefficient)	
					Lower	Upper		Lower	Upper
Intercept	-3.219	.8637	-3.727	.000	-4.912	-1.526	.040	.007	.217
physical_health	.241	.0413	5.823	.000	.160	.322	1.272	1.173	1.379
kess_t15	3.020	.1265	23.878	.000	2.772	3.268	20.491	15.991	26.257
choicecwc1	-.098	.0480	-2.033	.042	-.192	-.003	.907	.825	.997
choicem1	-.086	.2593	-.332	.740	-.594	.422	.918	.552	1.525
fac1choi	-.004	.0523	-.072	.942	-.106	.099	.996	.899	1.104
bullying_freq	.200	.0776	2.573	.010	.048	.352	1.221	1.049	1.422
time_pressure_freq	-.064	.0442	-1.456	.145	-.151	.022	.938	.860	1.023
duties_freq	-.151	.0716	-2.110	.035	-.291	-.011	.860	.747	.989
FAC1_1	.035	.0482	.724	.469	-.060	.129	1.036	.942	1.138

Probability distribution: Binomial

Link function: Logit

a. Target: Kessler16 > 1 = 1

Fixed Coefficients

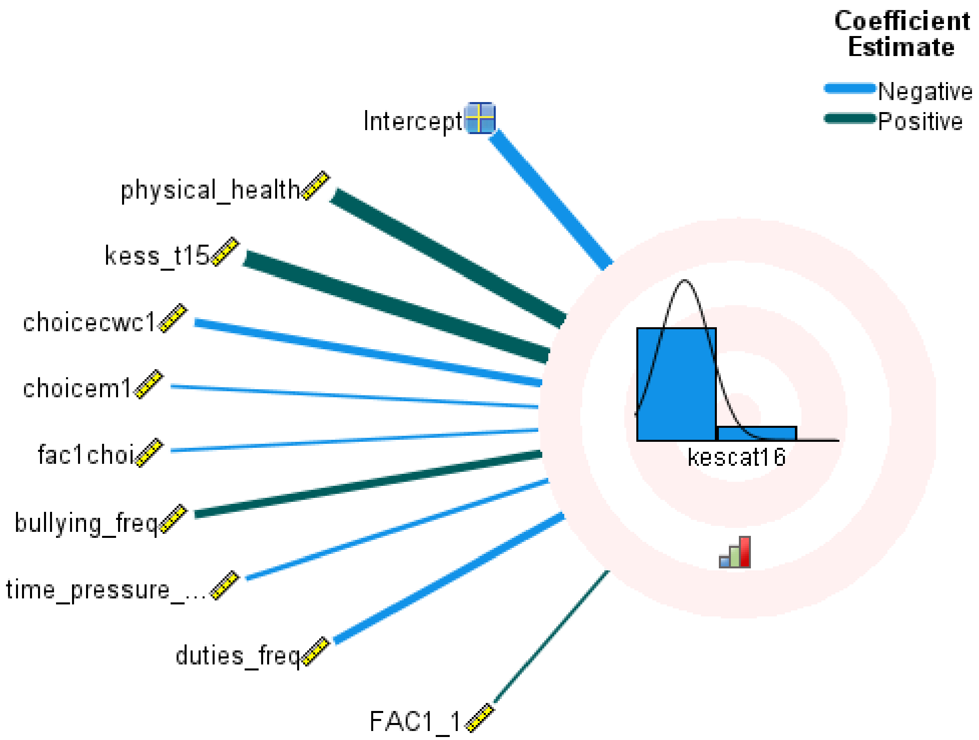

Random Effect Covariances

Random Effect Block 1

Random Effect Block	Intercept
Intercept	.023

Covariance Structure: Variance components

Subject Specification: companyId15

Covariance Parameters

Covariance Parameters Summary

Covariance Parameters	Residual Effect	0
	Random Effects	1
Design Matrix Columns	Fixed Effects	10
	Random Effects	1[a]
Common Subjects		58

Common subjects are based on the subject specifications for the residual and random effects and are used to chunk the data for better performance.

a. This is the number of columns per common subject.

Residual Effect

Residual Effect	Estimate	Std. Error	Z	Sig.	95% Confidence Interval	
					Lower	Upper
Variance	1.000	.	.	.	.	.

Covariance Structure: Scaled Identity

Subject Specification: (None)

Random Effect

Random Effect Covariance	Estimate	Std. Error	Z	Sig.	95% Confidence Interval	
					Lower	Upper
Var(Intercept)	.023	.020	1.105	.269	.004	.133

Covariance Structure: Variance components

Subject Specification: companyId15

Random Effects Parameters

Empirical Best Linear Unbiased Predictions[a]

company_code	Parameter	Prediction	Std. Error	df	Sig.	95% Confidence Interval	
						Lower Bound	Upper Bound
2	Intercept	.007	.140	5329	.962	-.267	.280

4	Intercept	.011	.144	5329	.939	-.270	.293
6	Intercept	.004	.142	5329	.979	-.275	.283
22	Intercept	-.010	.150	5329	.947	-.304	.284
24	Intercept	-.031	.131	5329	.810	-.288	.225
30	Intercept	-.005	.145	5329	.971	-.290	.279
36	Intercept	.040	.142	5329	.779	-.238	.318
40	Intercept	.091	.143	5329	.524	-.189	.372
50	Intercept	.007	.146	5329	.962	-.279	.293
61	Intercept	-.031	.143	5329	.827	-.311	.249
65	Intercept	.023	.148	5329	.879	-.267	.312
68	Intercept	-.041	.142	5329	.774	-.320	.238
69	Intercept	.033	.142	5329	.816	-.246	.312
71	Intercept	.036	.146	5329	.805	-.251	.323
72	Intercept	-.008	.146	5329	.956	-.294	.278
74	Intercept	-.040	.147	5329	.784	-.328	.247
75	Intercept	-.044	.145	5329	.760	-.328	.239
80	Intercept	.019	.149	5329	.897	-.272	.311
81	Intercept	-.015	.144	5329	.918	-.297	.268
83	Intercept	.025	.144	5329	.863	-.258	.307
84	Intercept	-.022	.145	5329	.881	-.306	.263
92	Intercept	-.004	.142	5329	.979	-.283	.275
93	Intercept	-.004	.143	5329	.979	-.285	.277
98	Intercept	.072	.139	5329	.606	-.200	.343
100	Intercept	.066	.112	5329	.554	-.153	.285
106	Intercept	.022	.145	5329	.878	-.263	.308
107	Intercept	-.017	.144	5329	.907	-.299	.266

108	Intercept	-.029	.146	5329	.845	-.315	.258
110	Intercept	-.180	.107	5329	.093	-.390	.030
114	Intercept	.024	.144	5329	.867	-.259	.307
116	Intercept	.042	.141	5329	.766	-.235	.319
122	Intercept	.069	.136	5329	.614	-.198	.336
125	Intercept	-.042	.127	5329	.739	-.290	.206
142	Intercept	-.069	.137	5329	.613	-.338	.199
144	Intercept	.011	.140	5329	.938	-.264	.286
145	Intercept	.004	.142	5329	.976	-.274	.283
152	Intercept	.005	.142	5329	.971	-.272	.283
154	Intercept	-.036	.141	5329	.798	-.313	.241
156	Intercept	.040	.140	5329	.777	-.235	.314
162	Intercept	.032	.143	5329	.820	-.247	.312
165	Intercept	.023	.144	5329	.873	-.260	.306
166	Intercept	-.030	.148	5329	.838	-.321	.261
169	Intercept	-.052	.144	5329	.719	-.334	.230
170	Intercept	.059	.145	5329	.685	-.225	.343
173	Intercept	-.051	.144	5329	.724	-.334	.232
174	Intercept	-.020	.144	5329	.890	-.301	.262
178	Intercept	.020	.140	5329	.887	-.254	.294
181	Intercept	.044	.146	5329	.764	-.243	.331
182	Intercept	.020	.146	5329	.892	-.267	.307
192	Intercept	-.040	.146	5329	.782	-.326	.245
202	Intercept	.063	.138	5329	.649	-.207	.333
204	Intercept	-.156	.135	5329	.249	-.421	.109
205	Intercept	-.022	.148	5329	.880	-.312	.267

206	Intercept	-.012	.144	5329	.933	-.294	.270
209	Intercept	.042	.131	5329	.749	-.215	.299
217	Intercept	.140	.124	5329	.256	-.102	.383
226	Intercept	-.023	.144	5329	.873	-.306	.260
228	Intercept	-.058	.146	5329	.690	-.344	.228

a. Target: Kessler16 > 1 = 1

```
GENLINMIXED
  /DATA_STRUCTURE SUBJECTS=companyId15
  /FIELDS TARGET=kescat16 TRIALS=NONE OFFSET=NONE
  /TARGET_OPTIONS DISTRIBUTION=BINOMIAL LINK=LOGIT
  /FIXED  EFFECTS=physical_health kess_t15 choice_freq bullycwc1 bullym1 fac1bull
   time_pressure_freq duties_freq
    FAC1_1 USE_INTERCEPT=TRUE
  /RANDOM USE_INTERCEPT=TRUE SUBJECTS=companyId15 COVARIANCE_TYPE=VARIANCE_COMPONENTS SOLUTION=TRUE
  /BUILD_OPTIONS TARGET_CATEGORY_ORDER=DESCENDING INPUTS_CATEGORY_ORDER=DESCENDING
    MAX_ITERATIONS=100 CONFIDENCE_LEVEL=95 DF_METHOD=RESIDUAL COVB=ROBUST PCONVERGE=0.000001(ABSOLUTE)
    SCORING=0 SINGULAR=0.000000000001
  /EMMEANS_OPTIONS SCALE=ORIGINAL PADJUST=LSD.
```

Generalized Linear Mixed Models

Notes

Output Created	16-AUG-2021 11:07:31
Comments	

Input	Data	C:\Users\jwb12ppu\Documents\Kevin\MPlus backup\Multicomponent health and wellbeing v2\Multicomponent for Mplus cat.sav
	Active Dataset	DataSet2
	Filter	kescat15 = 0 (FILTER)
	Weight	<none>
	Split File	<none>
	N of Rows in Working Data File	5855

Syntax	GENLINMIXED<br>/DATA_STRUCTURE<br>SUBJECTS=companyId15<br>/FIELDS TARGET=kescat16<br>TRIALS=NONE OFFSET=NONE<br>/TARGET_OPTIONS<br>DISTRIBUTION=BINOMIAL<br>LINK=LOGIT<br>/FIXED<br>EFFECTS=physical_health<br>kess_t15 choice_freq bullycwc1<br>bullym1 fac1bull<br>time_pressure_freq duties_freq<br>FAC1_1<br>USE_INTERCEPT=TRUE<br>/RANDOM<br>USE_INTERCEPT=TRUE<br>SUBJECTS=companyId15<br>COVARIANCE_TYPE=VARIANCE<br>_COMPONENTS<br>SOLUTION=TRUE<br>/BUILD_OPTIONS<br>TARGET_CATEGORY_ORDER=<br>DESCENDING<br>INPUTS_CATEGORY_ORDER=D<br>ESCENDING<br>MAX_ITERATIONS=100<br>CONFIDENCE_LEVEL=95

		DF_METHOD=RESIDUAL COVB=ROBUST PCONVERGE=0.000001(ABSOLUTE) SCORING=0 SINGULAR=0.000000000001 /EMMEANS_OPTIONS SCALE=ORIGINAL PADJUST=LSD.
Resources	Processor Time	00:00:01.39
	Elapsed Time	00:00:01.42

Warnings

glmm: One or more records are not used in the analysis because they have one or more fields with invalid or missing values.
glmm: Valid values for events (target) and trials variables are non-negative and positive integers respectively, and the number of trials cannot be less than the number of events.
glmm: The final Hessian matrix is not positive definite although all convergence criteria are satisfied. The procedure continues despite this warning. Subsequent results produced are based on the last iteration. Validity of the model fit is uncertain.

Case Processing Summary

	N	Percent
Included	5339	91.2%
Excluded	516	8.8%
Total	5855	100.0%

Model Summary

Target		Kessler16 > 1 = 1
Probability Distribution		Binomial
Link Function		Logit
Information Criterion	Akaike Corrected	27761.298
	Bayesian	27767.878

Information criteria are based on the -2 log likelihood (27759.297) and are used to compare models. Models with smaller information criterion values fit better.

Data Structure[a]

	Subjects company_code	Target Kessler16 > 1 = 1
Data for First Subject	2	.00
	2	.00
	2	.00
	2	.00
	2	1.00
	2	1.00
	2	.00

	2	.00
	2	.00
	2	1.00
Total Number of Levels	58	

Only the first 10 records are displayed.

a. Target: Kessler16 > 1 = 1

Classification

Overall Percent Correct = 88.8%[a]

Observed		Predicted 1.00	.00
1.00	Count	10	591
	% within Observed	1.7%	98.3%
.00	Count	6	4732
	% within Observed	0.1%	99.9%

a. Target: Kessler16 > 1 = 1

Fixed Effects[a]

Source	F	df1	df2	Sig.
Corrected Model	137.435	9	5329	.000
physical_health	28.295	1	5329	.000
kess_t15	482.619	1	5329	.000
choice_freq	4.615	1	5329	.032
bullycwc1	7.909	1	5329	.005

bullym1	5.174	1	5329	.023
fac1bull	.015	1	5329	.902
time_pressure_freq	1.394	1	5329	.238
duties_freq	3.570	1	5329	.059
FAC1_1	.432	1	5329	.511

Probability distribution: Binomial

Link function: Logit

a. Target: Kessler16 > 1 = 1

Fixed Effects

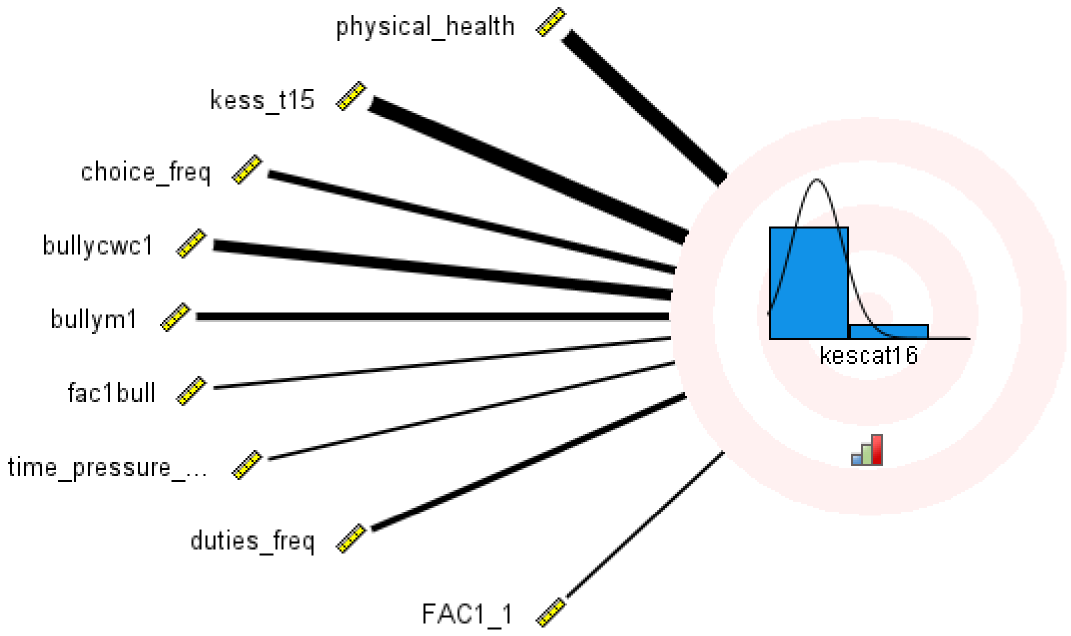

Fixed Coefficients[a]

Model Term	Coefficient	Std. Error	t	Sig.	95% Confidence Interval		Exp(Coefficient)	95% Confidence Interval for Exp(Coefficient)	
					Lower	Upper		Lower	Upper
Intercept	-.740	1.1056	-.670	.503	-2.908	1.427	.477	.055	4.166
physical_health	.206	.0387	5.319	.000	.130	.282	1.229	1.139	1.325
kess_t15	2.595	.1181	21.969	.000	2.363	2.826	13.392	10.624	16.881
choice_freq	-.087	.0407	-2.148	.032	-.167	-.008	.916	.846	.992
bullycwc1	.212	.0753	2.812	.005	.064	.360	1.236	1.066	1.433
bullym1	-1.716	.7542	-2.275	.023	-3.194	-.237	.180	.041	.789
fac1bull	-.010	.0804	-.124	.902	-.168	.148	.990	.846	1.159
time_pressure_freq	-.048	.0404	-1.181	.238	-.127	.031	.953	.881	1.032
duties_freq	-.125	.0661	-1.889	.059	-.255	.005	.883	.775	1.005
FAC1_1	.025	.0385	.657	.511	-.050	.101	1.026	.951	1.106

Probability distribution: Binomial

Link function: Logit

a. Target: Kessler16 > 1 = 1

Fixed Coefficients

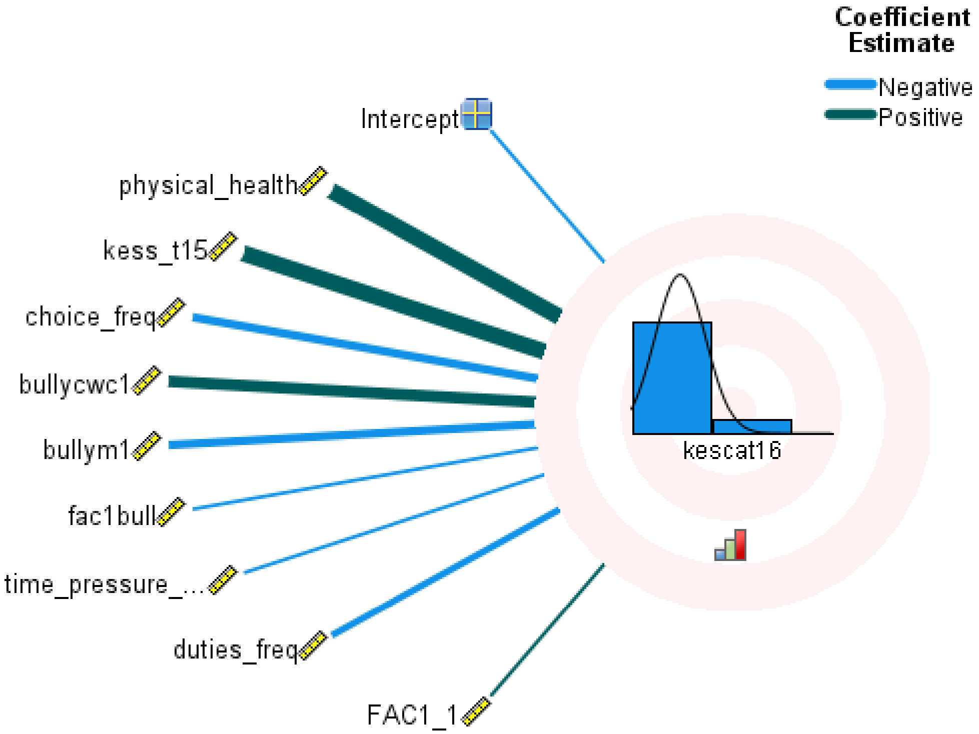

Random Effect Covariances

Random Effect Block 1

Random Effect Block	Intercept
Intercept	.000

Covariance Structure: Variance components

Subject Specification: companyId15

Covariance Parameters

Covariance Parameters Summary

Covariance Parameters	Residual Effect	0
	Random Effects	1
Design Matrix Columns	Fixed Effects	10
	Random Effects	1[a]
Common Subjects		58

Common subjects are based on the subject specifications for the residual and random effects and are used to chunk the data for better performance.

a. This is the number of columns per common subject.

Residual Effect

Residual Effect	Estimate	Std. Error	Z	Sig.	95% Confidence Interval	
					Lower	Upper
Variance	1.000	.	.	.	.	.

Covariance Structure: Scaled Identity

Subject Specification: (None)

Random Effect

Random Effect Covariance	Estimate	Std. Error	Z	Sig.	95% Confidence Interval	
					Lower	Upper
Var(Intercept)	1.030E-11[a]	.	.	.	.	.

Covariance Structure: Variance components

Subject Specification: companyId15

a. This parameter is redundant.

Random Effects Parameters

Empirical Best Linear Unbiased Predictions[a]

company_code	Parameter	Prediction	Std. Error	df	Sig.	95% Confidence Interval	
						Lower Bound	Upper Bound

2	Intercept	-2.236E-12	3.209E-6	5329	1.000	-6.291E-6	6.291E-6
4	Intercept	2.520E-12	3.209E-6	5329	1.000	-6.291E-6	6.291E-6
6	Intercept	-5.782E-12	3.209E-6	5329	1.000	-6.291E-6	6.291E-6
22	Intercept	-1.089E-13	3.209E-6	5329	1.000	-6.291E-6	6.291E-6
24	Intercept	-7.614E-12	3.209E-6	5329	1.000	-6.291E-6	6.291E-6
30	Intercept	1.745E-12	3.209E-6	5329	1.000	-6.291E-6	6.291E-6
36	Intercept	1.812E-11	3.209E-6	5329	1.000	-6.291E-6	6.291E-6
40	Intercept	3.689E-11	3.209E-6	5329	1.000	-6.291E-6	6.291E-6
50	Intercept	3.675E-12	3.209E-6	5329	1.000	-6.291E-6	6.291E-6
61	Intercept	-1.603E-11	3.209E-6	5329	1.000	-6.291E-6	6.291E-6
65	Intercept	1.293E-11	3.209E-6	5329	1.000	-6.291E-6	6.291E-6
68	Intercept	-2.652E-11	3.209E-6	5329	1.000	-6.291E-6	6.291E-6
69	Intercept	1.339E-11	3.209E-6	5329	1.000	-6.291E-6	6.291E-6
71	Intercept	1.788E-11	3.209E-6	5329	1.000	-6.291E-6	6.291E-6
72	Intercept	-5.321E-12	3.209E-6	5329	1.000	-6.291E-6	6.291E-6
74	Intercept	-1.594E-11	3.209E-6	5329	1.000	-6.291E-6	6.291E-6
75	Intercept	-2.445E-11	3.209E-6	5329	1.000	-6.291E-6	6.291E-6
80	Intercept	4.513E-12	3.209E-6	5329	1.000	-6.291E-6	6.291E-6
81	Intercept	-7.347E-12	3.209E-6	5329	1.000	-6.291E-6	6.291E-6
83	Intercept	1.360E-12	3.209E-6	5329	1.000	-6.291E-6	6.291E-6
84	Intercept	-1.824E-11	3.209E-6	5329	1.000	-6.291E-6	6.291E-6
92	Intercept	-7.583E-12	3.209E-6	5329	1.000	-6.291E-6	6.291E-6
93	Intercept	1.535E-12	3.209E-6	5329	1.000	-6.291E-6	6.291E-6
98	Intercept	3.321E-11	3.209E-6	5329	1.000	-6.291E-6	6.291E-6
100	Intercept	1.153E-10	3.209E-6	5329	1.000	-6.291E-6	6.291E-6
106	Intercept	6.036E-12	3.209E-6	5329	1.000	-6.291E-6	6.291E-6

107	Intercept	-1.562E-11	3.209E-6	5329	1.000	-6.291E-6	6.291E-6
108	Intercept	-2.093E-11	3.209E-6	5329	1.000	-6.291E-6	6.291E-6
110	Intercept	-1.061E-10	3.209E-6	5329	1.000	-6.291E-6	6.291E-6
114	Intercept	3.930E-12	3.209E-6	5329	1.000	-6.291E-6	6.291E-6
116	Intercept	1.201E-11	3.209E-6	5329	1.000	-6.291E-6	6.291E-6
122	Intercept	4.152E-11	3.209E-6	5329	1.000	-6.291E-6	6.291E-6
125	Intercept	-4.056E-11	3.209E-6	5329	1.000	-6.291E-6	6.291E-6
142	Intercept	-2.845E-11	3.209E-6	5329	1.000	-6.291E-6	6.291E-6
144	Intercept	-3.032E-12	3.209E-6	5329	1.000	-6.291E-6	6.291E-6
145	Intercept	-1.302E-11	3.209E-6	5329	1.000	-6.291E-6	6.291E-6
152	Intercept	8.773E-12	3.209E-6	5329	1.000	-6.291E-6	6.291E-6
154	Intercept	-1.298E-12	3.209E-6	5329	1.000	-6.291E-6	6.291E-6
156	Intercept	4.353E-12	3.209E-6	5329	1.000	-6.291E-6	6.291E-6
162	Intercept	2.094E-11	3.209E-6	5329	1.000	-6.291E-6	6.291E-6
165	Intercept	7.077E-12	3.209E-6	5329	1.000	-6.291E-6	6.291E-6
166	Intercept	-1.538E-11	3.209E-6	5329	1.000	-6.291E-6	6.291E-6
169	Intercept	-3.135E-11	3.209E-6	5329	1.000	-6.291E-6	6.291E-6
170	Intercept	2.198E-11	3.209E-6	5329	1.000	-6.291E-6	6.291E-6
173	Intercept	-3.346E-11	3.209E-6	5329	1.000	-6.291E-6	6.291E-6
174	Intercept	-1.689E-12	3.209E-6	5329	1.000	-6.291E-6	6.291E-6
178	Intercept	6.052E-12	3.209E-6	5329	1.000	-6.291E-6	6.291E-6
181	Intercept	3.149E-11	3.209E-6	5329	1.000	-6.291E-6	6.291E-6
182	Intercept	1.021E-12	3.209E-6	5329	1.000	-6.291E-6	6.291E-6
192	Intercept	-1.675E-11	3.209E-6	5329	1.000	-6.291E-6	6.291E-6
202	Intercept	3.522E-11	3.209E-6	5329	1.000	-6.291E-6	6.291E-6
204	Intercept	-5.082E-11	3.209E-6	5329	1.000	-6.291E-6	6.291E-6

205	Intercept	-1.012E-11	3.209E-6	5329	1.000	-6.291E-6	6.291E-6
206	Intercept	-9.643E-12	3.209E-6	5329	1.000	-6.291E-6	6.291E-6
209	Intercept	2.572E-11	3.209E-6	5329	1.000	-6.291E-6	6.291E-6
217	Intercept	9.219E-11	3.209E-6	5329	1.000	-6.291E-6	6.291E-6
226	Intercept	-1.646E-11	3.209E-6	5329	1.000	-6.291E-6	6.291E-6
228	Intercept	-2.957E-11	3.209E-6	5329	1.000	-6.291E-6	6.291E-6

a. Target: Kessler16 > 1 = 1

```
GENLINMIXED
  /DATA_STRUCTURE SUBJECTS=companyId15
  /FIELDS TARGET=kescat16 TRIALS=NONE OFFSET=NONE
  /TARGET_OPTIONS DISTRIBUTION=BINOMIAL LINK=LOGIT
  /FIXED  EFFECTS=physical_health kess_t15 choice_freq bullying_freq timecwc1 timem1 fac1time
  duties_freq
    FAC1_1 USE_INTERCEPT=TRUE
  /RANDOM USE_INTERCEPT=TRUE SUBJECTS=companyId15 COVARIANCE_TYPE=VARIANCE_COMPONENTS SOLUTION=TRUE
  /BUILD_OPTIONS TARGET_CATEGORY_ORDER=DESCENDING INPUTS_CATEGORY_ORDER=DESCENDING
    MAX_ITERATIONS=100 CONFIDENCE_LEVEL=95 DF_METHOD=RESIDUAL COVB=ROBUST PCONVERGE=0.000001(ABSOLUTE)
    SCORING=0 SINGULAR=0.000000000001
  /EMMEANS_OPTIONS SCALE=ORIGINAL PADJUST=LSD.
```

Generalized Linear Mixed Models

Notes

Output Created	16-AUG-2021 11:07:33

Comments		
Input	Data	C:\Users\jwb12ppu\Documents\Kevin\MPlus backup\Multicomponent health and wellbeing v2\Multicomponent for Mplus cat.sav
	Active Dataset	DataSet2
	Filter	kescat15 = 0 (FILTER)
	Weight	<none>
	Split File	<none>
	N of Rows in Working Data File	5855

Syntax	GENLINMIXED<br>/DATA_STRUCTURE<br>SUBJECTS=companyId15<br>/FIELDS TARGET=kescat16<br>TRIALS=NONE OFFSET=NONE<br>/TARGET_OPTIONS<br>DISTRIBUTION=BINOMIAL<br>LINK=LOGIT<br>/FIXED<br>EFFECTS=physical_health<br>kess_t15 choice_freq bullying_freq<br>timecwc1 timem1 fac1time<br>duties_freq<br>FAC1_1<br>USE_INTERCEPT=TRUE<br>/RANDOM<br>USE_INTERCEPT=TRUE<br>SUBJECTS=companyId15<br>COVARIANCE_TYPE=VARIANCE<br>_COMPONENTS<br>SOLUTION=TRUE<br>/BUILD_OPTIONS<br>TARGET_CATEGORY_ORDER=<br>DESCENDING<br>INPUTS_CATEGORY_ORDER=D<br>ESCENDING<br>MAX_ITERATIONS=100<br>CONFIDENCE_LEVEL=95

		DF_METHOD=RESIDUAL<br>COVB=ROBUST<br>PCONVERGE=0.000001(ABSOLUTE)<br>SCORING=0<br>SINGULAR=0.000000000001<br>/EMMEANS_OPTIONS<br>SCALE=ORIGINAL<br>PADJUST=LSD.
Resources	Processor Time	00:00:02.00
	Elapsed Time	00:00:01.89

Warnings

glmm: One or more records are not used in the analysis because they have one or more fields with invalid or missing values.
glmm: Valid values for events (target) and trials variables are non-negative and positive integers respectively, and the number of trials cannot be less than the number of events.

Case Processing Summary

	N	Percent
Included	5339	91.2%
Excluded	516	8.8%
Total	5855	100.0%

Model Summary

Target		Kessler16 > 1 = 1
Probability Distribution		Binomial
Link Function		Logit
Information Criterion	Akaike Corrected	29422.407
	Bayesian	29428.987

Information criteria are based on the -2 log likelihood (29420.406) and are used to compare models. Models with smaller information criterion values fit better.

Data Structure[a]

	Subjects<br>company_code	Target<br>Kessler16 > 1 = 1
Data for First Subject	2	.00
	2	.00
	2	.00
	2	.00
	2	1.00
	2	1.00
	2	.00
	2	.00
	2	.00
	2	1.00
Total Number of Levels	58	

Only the first 10 records are displayed.

a. Target: Kessler16 > 1 = 1

Classification

Overall Percent Correct = 88.9%[a]

Observed		Predicted 1.00	Predicted .00
1.00	Count	20	581
	% within Observed	3.3%	96.7%
.00	Count	14	4724
	% within Observed	0.3%	99.7%

a. Target: Kessler16 > 1 = 1

Fixed Effects[a]

Source	F	df1	df2	Sig.
Corrected Model	150.562	9	5329	.000
physical_health	34.361	1	5329	.000
kess_t15	573.911	1	5329	.000
choice_freq	4.732	1	5329	.030
bullying_freq	6.754	1	5329	.009
timecwc1	1.622	1	5329	.203
timem1	3.651	1	5329	.056
fac1time	1.340	1	5329	.247
duties_freq	4.344	1	5329	.037

FAC1_1	.804	1	5329	.370

Probability distribution: Binomial

Link function: Logit

a. Target: Kessler16 > 1 = 1

Fixed Effects

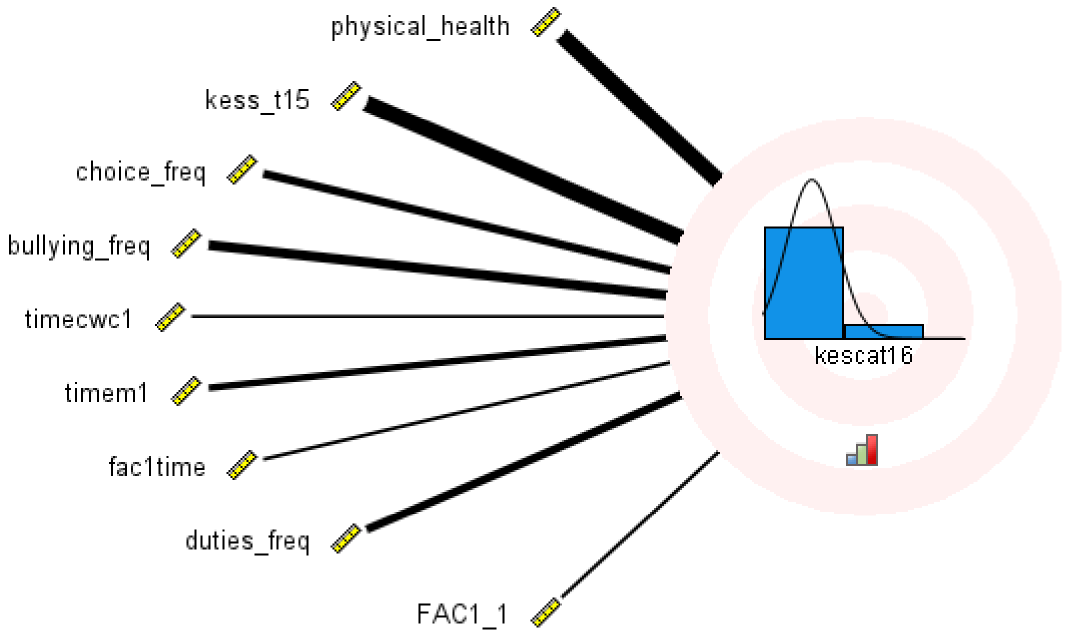

Fixed Coefficients[a]

Model Term	Coefficient	Std. Error	t	Sig.	95% Confidence Interval		Exp(Coefficient)	95% Confidence Interval for Exp(Coefficient)	
					Lower	Upper		Lower	Upper
Intercept	-2.469	.6828	-3.616	.000	-3.807	-1.130	.085	.022	.323
physical_health	.243	.0414	5.862	.000	.162	.324	1.275	1.175	1.383
kess_t15	3.016	.1259	23.956	.000	2.769	3.262	20.402	15.940	26.112
choice_freq	-.099	.0457	-2.175	.030	-.189	-.010	.905	.828	.990
bullying_freq	.201	.0773	2.599	.009	.049	.353	1.223	1.051	1.423
timecwc1	-.050	.0390	-1.274	.203	-.126	.027	.952	.881	1.027
timem1	-.362	.1896	-1.911	.056	-.734	.009	.696	.480	1.009
fac1time	-.039	.0333	-1.157	.247	-.104	.027	.962	.901	1.027
duties_freq	-.149	.0716	-2.084	.037	-.290	-.009	.861	.749	.991
FAC1_1	.037	.0415	.897	.370	-.044	.119	1.038	.957	1.126

Probability distribution: Binomial

Link function: Logit

a. Target: Kessler16 > 1 = 1

Fixed Coefficients

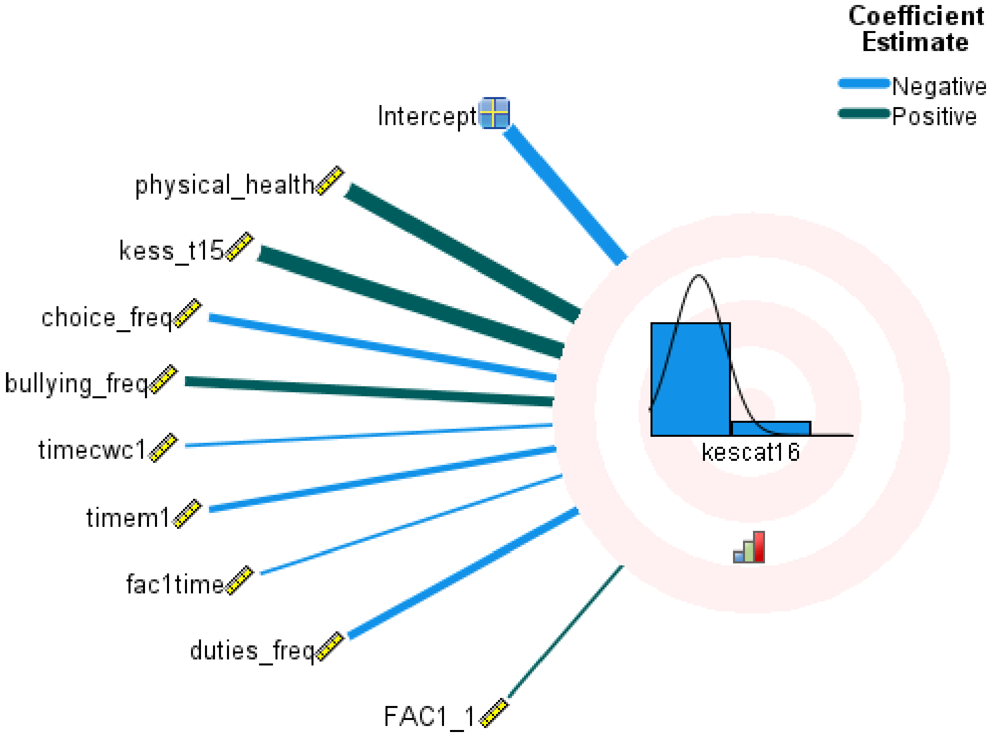

Random Effect Covariances

Random Effect Block 1

Random Effect Block	Intercept
Intercept	.017

Covariance Structure: Variance components

Subject Specification: companyId15

Covariance Parameters

Covariance Parameters Summary

Covariance Parameters	Residual Effect	0
	Random Effects	1
Design Matrix Columns	Fixed Effects	10
	Random Effects	1[a]
Common Subjects		58

Common subjects are based on the subject specifications for the residual and random effects and are used to chunk the data for better performance.

a. This is the number of columns per common subject.

Residual Effect

Residual Effect	Estimate	Std. Error	Z	Sig.	95% Confidence Interval	
					Lower	Upper
Variance	1.000	.	.	.	.	.

Covariance Structure: Scaled Identity

Subject Specification: (None)

Random Effect

Random Effect Covariance	Estimate	Std. Error	Z	Sig.	95% Confidence Interval	
					Lower	Upper
Var(Intercept)	.017	.018	.935	.350	.002	.136

Covariance Structure: Variance components

Subject Specification: companyId15

Random Effects Parameters

Empirical Best Linear Unbiased Predictions[a]

company_code	Parameter	Prediction	Std. Error	df	Sig.	95% Confidence Interval	
						Lower Bound	Upper Bound
2	Intercept	.007	.122	5329	.956	-.233	.247

4	Intercept	.001	.125	5329	.994	-.244	.246
6	Intercept	.000	.124	5329	.998	-.243	.243
22	Intercept	-.008	.129	5329	.953	-.261	.245
24	Intercept	-.009	.117	5329	.936	-.239	.220
30	Intercept	-.008	.126	5329	.948	-.255	.239
36	Intercept	.030	.124	5329	.807	-.213	.273
40	Intercept	.070	.125	5329	.576	-.175	.315
50	Intercept	.000	.126	5329	.999	-.247	.247
61	Intercept	-.030	.124	5329	.812	-.273	.214
65	Intercept	.012	.128	5329	.928	-.238	.262
68	Intercept	-.034	.124	5329	.786	-.277	.209
69	Intercept	.032	.125	5329	.795	-.212	.277
71	Intercept	.031	.127	5329	.809	-.218	.280
72	Intercept	-.011	.126	5329	.928	-.259	.236
74	Intercept	-.030	.127	5329	.814	-.279	.219
75	Intercept	-.034	.126	5329	.787	-.280	.212
80	Intercept	.011	.128	5329	.932	-.240	.262
81	Intercept	-.021	.125	5329	.869	-.266	.225
83	Intercept	.018	.125	5329	.883	-.227	.264
84	Intercept	-.015	.126	5329	.906	-.262	.232
92	Intercept	-.003	.124	5329	.981	-.246	.241
93	Intercept	-.004	.125	5329	.973	-.249	.240
98	Intercept	.061	.122	5329	.619	-.178	.300
100	Intercept	.075	.093	5329	.417	-.107	.257
106	Intercept	.017	.126	5329	.893	-.230	.264
107	Intercept	-.017	.125	5329	.895	-.262	.229

108	Intercept	-.016	.127	5329	.899	-.265	.233
110	Intercept	-.140	.098	5329	.152	-.333	.052
114	Intercept	.016	.125	5329	.898	-.230	.262
116	Intercept	.024	.123	5329	.843	-.217	.266
122	Intercept	.066	.120	5329	.585	-.170	.301
125	Intercept	-.005	.115	5329	.964	-.231	.221
142	Intercept	-.034	.122	5329	.778	-.273	.204
144	Intercept	.001	.123	5329	.993	-.239	.242
145	Intercept	.004	.124	5329	.972	-.239	.247
152	Intercept	.004	.124	5329	.971	-.238	.247
154	Intercept	-.026	.124	5329	.834	-.268	.216
156	Intercept	.018	.122	5329	.885	-.222	.257
162	Intercept	.022	.124	5329	.859	-.222	.266
165	Intercept	.018	.126	5329	.887	-.228	.264
166	Intercept	-.021	.128	5329	.870	-.272	.231
169	Intercept	-.040	.125	5329	.752	-.285	.206
170	Intercept	.037	.126	5329	.766	-.209	.284
173	Intercept	-.053	.125	5329	.673	-.298	.193
174	Intercept	-.013	.125	5329	.919	-.258	.232
178	Intercept	.018	.123	5329	.884	-.222	.258
181	Intercept	.031	.127	5329	.807	-.217	.280
182	Intercept	.004	.126	5329	.977	-.244	.252
192	Intercept	-.033	.126	5329	.795	-.280	.215
202	Intercept	.042	.121	5329	.730	-.195	.279
204	Intercept	-.107	.116	5329	.356	-.334	.120
205	Intercept	-.021	.127	5329	.867	-.271	.229

206	Intercept	-.011	.125	5329	.928	-.256	.234
209	Intercept	.022	.114	5329	.849	-.202	.246
217	Intercept	.116	.106	5329	.273	-.092	.324
226	Intercept	-.024	.125	5329	.849	-.270	.222
228	Intercept	-.041	.127	5329	.744	-.289	.207

a. Target: Kessler16 > 1 = 1

```
GENLINMIXED
  /DATA_STRUCTURE SUBJECTS=companyId15
  /FIELDS TARGET=kescat16 TRIALS=NONE OFFSET=NONE
  /TARGET_OPTIONS DISTRIBUTION=BINOMIAL LINK=LOGIT
  /FIXED  EFFECTS=physical_health kess_t15 choice_freq bullying_freq time_pressure_freq dutiescwc1 dutiesm1 fac1duti
    FAC1_1 USE_INTERCEPT=TRUE
  /RANDOM USE_INTERCEPT=TRUE SUBJECTS=companyId15 COVARIANCE_TYPE=VARIANCE_COMPONENTS SOLUTION=TRUE
  /BUILD_OPTIONS TARGET_CATEGORY_ORDER=DESCENDING INPUTS_CATEGORY_ORDER=DESCENDING
    MAX_ITERATIONS=100 CONFIDENCE_LEVEL=95 DF_METHOD=RESIDUAL COVB=ROBUST PCONVERGE=0.000001(ABSOLUTE)
    SCORING=0 SINGULAR=0.000000000001
  /EMMEANS_OPTIONS SCALE=ORIGINAL PADJUST=LSD.
```

Generalized Linear Mixed Models

Notes

Output Created	16-AUG-2021 11:07:35
Comments	

Input	Data	C:\Users\jwb12ppu\Documents\Kevin\MPlus backup\Multicomponent health and wellbeing v2\Multicomponent for Mplus cat.sav
	Active Dataset	DataSet2
	Filter	kescat15 = 0 (FILTER)
	Weight	<none>
	Split File	<none>
	N of Rows in Working Data File	5855

Syntax	GENLINMIXED<br>/DATA_STRUCTURE<br>SUBJECTS=companyId15<br>/FIELDS TARGET=kescat16<br>TRIALS=NONE OFFSET=NONE<br>/TARGET_OPTIONS<br>DISTRIBUTION=BINOMIAL<br>LINK=LOGIT<br>/FIXED<br>EFFECTS=physical_health<br>kess_t15 choice_freq bullying_freq<br>time_pressure_freq dutiescwc1<br>dutiesm1 fac1duti<br>FAC1_1<br>USE_INTERCEPT=TRUE<br>/RANDOM<br>USE_INTERCEPT=TRUE<br>SUBJECTS=companyId15<br>COVARIANCE_TYPE=VARIANCE<br>_COMPONENTS<br>SOLUTION=TRUE<br>/BUILD_OPTIONS<br>TARGET_CATEGORY_ORDER=<br>DESCENDING<br>INPUTS_CATEGORY_ORDER=D<br>ESCENDING<br>MAX_ITERATIONS=100<br>CONFIDENCE_LEVEL=95

		DF_METHOD=RESIDUAL COVB=ROBUST PCONVERGE=0.000001(ABSOLUTE) SCORING=0 SINGULAR=0.000000000001 /EMMEANS_OPTIONS SCALE=ORIGINAL PADJUST=LSD.
Resources	Processor Time	00:00:02.03
	Elapsed Time	00:00:02.10

Warnings

glmm: One or more records are not used in the analysis because they have one or more fields with invalid or missing values.
glmm: Valid values for events (target) and trials variables are non-negative and positive integers respectively, and the number of trials cannot be less than the number of events.

Case Processing Summary

	N	Percent
Included	5339	91.2%
Excluded	516	8.8%
Total	5855	100.0%

Model Summary

Target		Kessler16 > 1 = 1
Probability Distribution		Binomial
Link Function		Logit
Information Criterion	Akaike Corrected	29431.948
	Bayesian	29438.528

Information criteria are based on the -2 log likelihood (29429.948) and are used to compare models. Models with smaller information criterion values fit better.

Data Structure[a]

	Subjects company_code	Target Kessler16 > 1 = 1
Data for First Subject	2	.00
	2	.00
	2	.00
	2	.00
	2	1.00
	2	1.00
	2	.00
	2	.00
	2	.00
	2	1.00
Total Number of Levels	58	

Only the first 10 records are displayed.

a. Target: Kessler16 > 1 = 1

Classification
Overall Percent Correct = 88.8%[a]

Observed		Predicted 1.00	Predicted .00
1.00	Count	18	583
	% within Observed	3.0%	97.0%
.00	Count	16	4722
	% within Observed	0.3%	99.7%

a. Target: Kessler16 > 1 = 1

Fixed Effects[a]

Source	F	df1	df2	Sig.
Corrected Model	138.195	9	5329	.000
physical_health	33.860	1	5329	.000
kess_t15	595.085	1	5329	.000
choice_freq	4.511	1	5329	.034
bullying_freq	6.711	1	5329	.010
time_pressure_freq	2.027	1	5329	.155
dutiescwc1	5.149	1	5329	.023
dutiesm1	.041	1	5329	.839
fac1duti	.950	1	5329	.330

FAC1_1	.162	1	5329	.687

Probability distribution: Binomial

Link function: Logit

a. Target: Kessler16 > 1 = 1

Fixed Effects

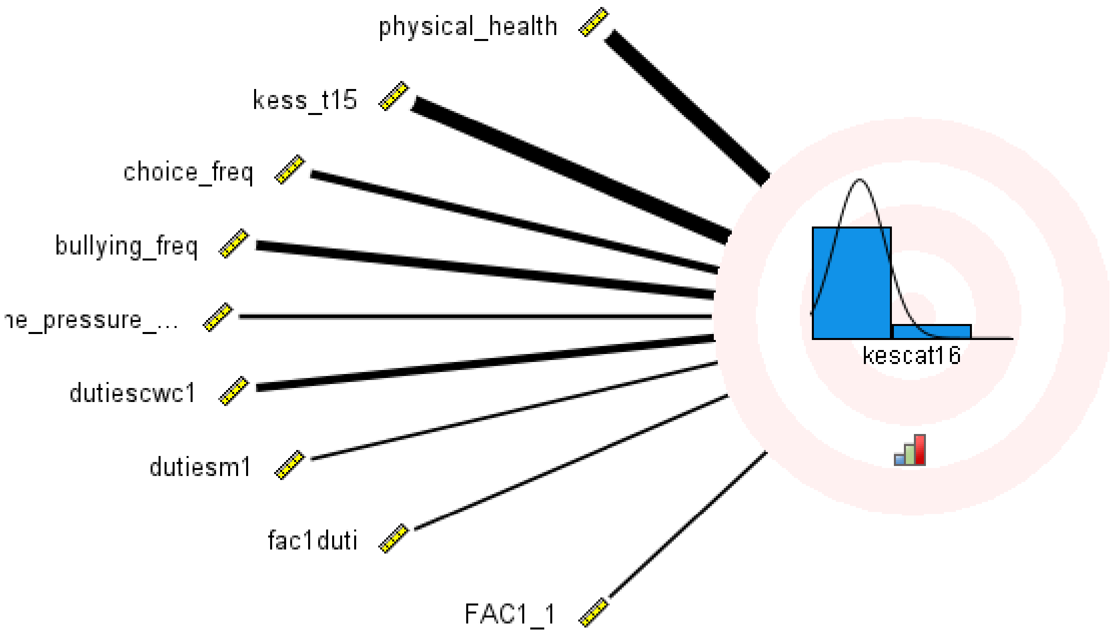

Fixed Coefficients[a]

Model Term	Coefficient	Std. Error	t	Sig.	95% Confidence Interval		Exp(Coefficient)	95% Confidence Interval for Exp(Coefficient)	
					Lower	Upper		Lower	Upper
Intercept	-3.488	1.5376	-2.268	.023	-6.502	-.473	.031	.002	.623
physical_health	.239	.0411	5.819	.000	.159	.320	1.270	1.172	1.377
kess_t15	3.021	.1238	24.394	.000	2.778	3.263	20.506	16.086	26.140
choice_freq	-.098	.0459	-2.124	.034	-.188	-.008	.907	.829	.993
bullying_freq	.199	.0769	2.591	.010	.048	.350	1.221	1.050	1.419
time_pressure_freq	-.063	.0443	-1.424	.155	-.150	.024	.939	.861	1.024
dutiescwc1	-.154	.0679	-2.269	.023	-.287	-.021	.857	.750	.979
dutiesm1	-.078	.3842	-.203	.839	-.831	.675	.925	.435	1.964
fac1duti	-.061	.0626	-.975	.330	-.184	.062	.941	.832	1.064
FAC1_1	.022	.0534	.403	.687	-.083	.126	1.022	.920	1.135

Probability distribution: Binomial

Link function: Logit

a. Target: Kessler16 > 1 = 1

Fixed Coefficients

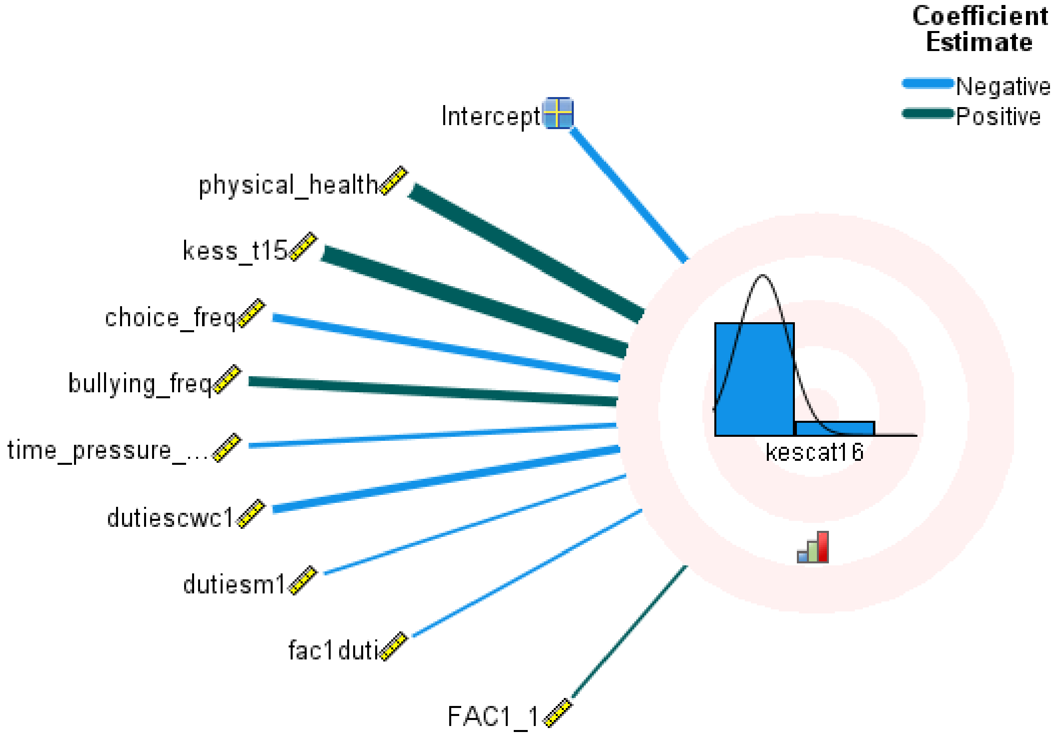

Random Effect Covariances

Random Effect Block 1

Random Effect Block	Intercept
Intercept	.021

Covariance Structure: Variance components
Subject Specification: companyId15

Covariance Parameters

Covariance Parameters Summary

Covariance Parameters	Residual Effect	0
	Random Effects	1
Design Matrix Columns	Fixed Effects	10
	Random Effects	1[a]
Common Subjects		58

Common subjects are based on the subject specifications for the residual and random effects and are used to chunk the data for better performance.

a. This is the number of columns per common subject.

Residual Effect

Residual Effect	Estimate	Std. Error	Z	Sig.	95% Confidence Interval	
					Lower	Upper
Variance	1.000	.	.	.	.	.

Covariance Structure: Scaled Identity

Subject Specification: (None)

Random Effect

Random Effect Covariance	Estimate	Std. Error	Z	Sig.	95% Confidence Interval	
					Lower	Upper
Var(Intercept)	.021	.022	.963	.336	.003	.159

Covariance Structure: Variance components

Subject Specification: companyId15

Random Effects Parameters

Empirical Best Linear Unbiased Predictions[a]

company_code	Parameter	Prediction	Std. Error	df	Sig.	95% Confidence Interval	
						Lower Bound	Upper Bound
2	Intercept	.005	.134	5329	.971	-.259	.269

4	Intercept	.008	.138	5329	.953	-.263	.279
6	Intercept	.002	.137	5329	.986	-.266	.271
22	Intercept	-.009	.144	5329	.948	-.291	.272
24	Intercept	-.028	.127	5329	.824	-.277	.220
30	Intercept	-.004	.140	5329	.977	-.278	.270
36	Intercept	.037	.137	5329	.784	-.231	.306
40	Intercept	.086	.138	5329	.535	-.185	.356
50	Intercept	.006	.140	5329	.966	-.268	.280
61	Intercept	-.031	.138	5329	.821	-.301	.239
65	Intercept	.020	.142	5329	.886	-.258	.298
68	Intercept	-.037	.137	5329	.785	-.306	.231
69	Intercept	.031	.137	5329	.820	-.238	.300
71	Intercept	.036	.141	5329	.798	-.240	.313
72	Intercept	-.007	.140	5329	.960	-.282	.268
74	Intercept	-.037	.141	5329	.795	-.313	.240
75	Intercept	-.043	.139	5329	.756	-.316	.230
80	Intercept	.017	.143	5329	.906	-.263	.297
81	Intercept	-.015	.139	5329	.915	-.287	.257
83	Intercept	.021	.139	5329	.882	-.251	.292
84	Intercept	-.021	.140	5329	.880	-.295	.253
92	Intercept	-.001	.137	5329	.992	-.271	.268
93	Intercept	-.004	.138	5329	.974	-.275	.266
98	Intercept	.065	.134	5329	.625	-.197	.328
100	Intercept	.066	.100	5329	.511	-.130	.261
106	Intercept	.022	.140	5329	.878	-.253	.296
107	Intercept	-.016	.139	5329	.908	-.288	.256

108	Intercept	-.026	.140	5329	.855	-.301	.250
110	Intercept	-.162	.129	5329	.210	-.415	.091
114	Intercept	.022	.139	5329	.875	-.250	.294
116	Intercept	.039	.136	5329	.773	-.227	.306
122	Intercept	.063	.131	5329	.631	-.193	.319
125	Intercept	-.040	.122	5329	.741	-.280	.199
142	Intercept	-.066	.132	5329	.619	-.324	.193
144	Intercept	.011	.135	5329	.933	-.254	.277
145	Intercept	.005	.137	5329	.969	-.263	.274
152	Intercept	.004	.136	5329	.979	-.263	.271
154	Intercept	-.033	.136	5329	.809	-.301	.235
156	Intercept	.037	.135	5329	.782	-.227	.301
162	Intercept	.029	.137	5329	.833	-.240	.298
165	Intercept	.021	.139	5329	.879	-.251	.293
166	Intercept	-.028	.143	5329	.842	-.308	.251
169	Intercept	-.048	.138	5329	.727	-.320	.223
170	Intercept	.055	.139	5329	.692	-.218	.328
173	Intercept	-.048	.139	5329	.731	-.320	.224
174	Intercept	-.019	.138	5329	.891	-.290	.252
178	Intercept	.019	.135	5329	.886	-.245	.284
181	Intercept	.041	.141	5329	.769	-.235	.317
182	Intercept	.018	.140	5329	.900	-.258	.293
192	Intercept	-.038	.140	5329	.788	-.312	.237
202	Intercept	.058	.133	5329	.662	-.203	.319
204	Intercept	-.147	.125	5329	.242	-.392	.099
205	Intercept	-.021	.142	5329	.881	-.299	.257

206	Intercept	-.012	.138	5329	.930	-.283	.259
209	Intercept	.038	.124	5329	.756	-.205	.282
217	Intercept	.131	.113	5329	.248	-.091	.353
226	Intercept	-.021	.139	5329	.879	-.293	.251
228	Intercept	-.052	.140	5329	.710	-.328	.223

a. Target: Kessler16 > 1 = 1

```
FILTER OFF.
USE ALL.
EXECUTE.
```

```
USE ALL.
COMPUTE filter_$=(kescat15 = 1).
VARIABLE LABELS filter_$ 'kescat15 = 0 (FILTER)'.
VALUE LABELS filter_$ 0 'Not Selected' 1 'Selected'.
FORMATS filter_$ (f1.0).
FILTER BY filter_$.
EXECUTE.

correlations /variables = kescat16 kescat15 fac1_1.
```

Correlations

Notes

Output Created		16-AUG-2021 11:07:37
Comments		
Input	Data	C:\Users\jwb12ppu\Documents\Kevin\MPlus backup\Multicomponent health and wellbeing v2\Multicomponent for Mplus cat.sav
	Active Dataset	DataSet2
	Filter	kescat15 = 0 (FILTER)
	Weight	<none>
	Split File	<none>
	N of Rows in Working Data File	1113
Missing Value Handling	Definition of Missing	User-defined missing values are treated as missing.
	Cases Used	Statistics for each pair of variables are based on all the cases with valid data for that pair.
Syntax		correlations /variables = kescat16 kescat15 fac1_1.
Resources	Processor Time	00:00:00.02
	Elapsed Time	00:00:00.03

Correlations

		Kessler16 > 1 = 1	Kessler15 > 1 = 1	REGR factor score 1 for analysis 1
Kessler16 > 1 = 1	Pearson Correlation	1	.[a]	-.092
	Sig. (2-tailed)		.	.002
	N	1113	1113	1113
Kessler15 > 1 = 1	Pearson Correlation	.[a]	.[a]	.[a]
	Sig. (2-tailed)	.		.
	N	1113	1113	1113
REGR factor score 1 for analysis 1	Pearson Correlation	-.092	.[a]	1
	Sig. (2-tailed)	.002	.	
	N	1113	1113	1113

a. Cannot be computed because at least one of the variables is constant.

```
*Generalized Linear Mixed Models.
GENLINMIXED
  /DATA_STRUCTURE SUBJECTS=companyId15
  /FIELDS TARGET=kescat16 TRIALS=NONE OFFSET=NONE
  /TARGET_OPTIONS DISTRIBUTION=BINOMIAL LINK=LOGIT
  /FIXED  EFFECTS=physical_health kess_t15 choice_freq bullying_freq time_pressure_freq duties_freq
    FAC1_1 USE_INTERCEPT=TRUE
  /RANDOM USE_INTERCEPT=TRUE SUBJECTS=companyId15 COVARIANCE_TYPE=VARIANCE_COMPONENTS SOLUTION=TRUE
  /BUILD_OPTIONS TARGET_CATEGORY_ORDER=DESCENDING INPUTS_CATEGORY_ORDER=DESCENDING
    MAX_ITERATIONS=100 CONFIDENCE_LEVEL=95 DF_METHOD=RESIDUAL COVB=ROBUST PCONVERGE=0.000001(ABSOLUTE)
    SCORING=0 SINGULAR=0.000000000001
  /EMMEANS_OPTIONS SCALE=ORIGINAL PADJUST=LSD.
```

Generalized Linear Mixed Models

Notes

Output Created		16-AUG-2021 11:07:37
Comments		
Input	Data	C:\Users\jwb12ppu\Documents\Kevin\MPlus backup\Multicomponent health and wellbeing v2\Multicomponent for Mplus cat.sav
	Active Dataset	DataSet2
	Filter	kescat15 = 0 (FILTER)
	Weight	<none>
	Split File	<none>
	N of Rows in Working Data File	1113

Syntax	GENLINMIXED<br>/DATA_STRUCTURE<br>SUBJECTS=companyId15<br>/FIELDS TARGET=kescat16<br>TRIALS=NONE OFFSET=NONE<br>/TARGET_OPTIONS<br>DISTRIBUTION=BINOMIAL<br>LINK=LOGIT<br>/FIXED<br>EFFECTS=physical_health<br>kess_t15 choice_freq bullying_freq<br>time_pressure_freq duties_freq<br>FAC1_1<br>USE_INTERCEPT=TRUE<br>/RANDOM<br>USE_INTERCEPT=TRUE<br>SUBJECTS=companyId15<br>COVARIANCE_TYPE=VARIANCE<br>_COMPONENTS<br>SOLUTION=TRUE<br>/BUILD_OPTIONS<br>TARGET_CATEGORY_ORDER=<br>DESCENDING<br>INPUTS_CATEGORY_ORDER=D<br>ESCENDING<br>MAX_ITERATIONS=100<br>CONFIDENCE_LEVEL=95<br>DF_METHOD=RESIDUAL

		COVB=ROBUST PCONVERGE=0.000001(ABSOLUTE) SCORING=0 SINGULAR=0.000000000001 /EMMEANS_OPTIONS SCALE=ORIGINAL PADJUST=LSD.
Resources	Processor Time	00:00:01.09
	Elapsed Time	00:00:01.27

Warnings

glmm: One or more records are not used in the analysis because they have one or more fields with invalid or missing values.
glmm: Valid values for events (target) and trials variables are non-negative and positive integers respectively, and the number of trials cannot be less than the number of events.
glmm: The estimated covariance matrix of the random effects (the G matrix) is not positive definite. The procedure continues despite this warning. Subsequent results produced are based on the last iteration. Validity of the model fit is uncertain.
glmm: The final Hessian matrix is not positive definite although all convergence criteria are satisfied. The procedure continues despite this warning. Subsequent results produced are based on the last iteration. Validity of the model fit is uncertain.

Case Processing Summary

	N	Percent
Included	1100	98.8%
Excluded	13	1.2%
Total	1113	100.0%

Model Summary

Target		Kessler16 > 1 = 1
Probability Distribution		Binomial
Link Function		Logit
Information Criterion	Akaike Corrected	4993.082
	Bayesian	4998.074

Information criteria are based on the -2 log likelihood (4991.079) and are used to compare models. Models with smaller information criterion values fit better.

Data Structure[a]

	Subjects	Target
	company_code	Kessler16 > 1 = 1
Data for First Subject	2	1.00
	2	1.00
	2	.00

	2	.00
	2	1.00
	2	1.00
	2	.00
	2	.00
	2	.00
	2	.00
Total Number of Levels	58	

Only the first 10 records are displayed.

a. Target: Kessler16 > 1 = 1

Classification

Overall Percent Correct = 67.8%[a]

Observed		Predicted 1.00	Predicted .00
1.00	Count	638	81
	% within Observed	88.7%	11.3%
.00	Count	273	108
	% within Observed	71.7%	28.3%

a. Target: Kessler16 > 1 = 1

Fixed Effects[a]

Source	F	df1	df2	Sig.
Corrected Model	21.022	7	1092	.000

physical_health	9.258	1	1092	.002
kess_t15	71.516	1	1092	.000
choice_freq	.263	1	1092	.608
bullying_freq	2.888	1	1092	.090
time_pressure_freq	1.706	1	1092	.192
duties_freq	6.551	1	1092	.011
FAC1_1	5.071	1	1092	.025

Probability distribution: Binomial

Link function: Logit

a. Target: Kessler16 > 1 = 1

Fixed Effects

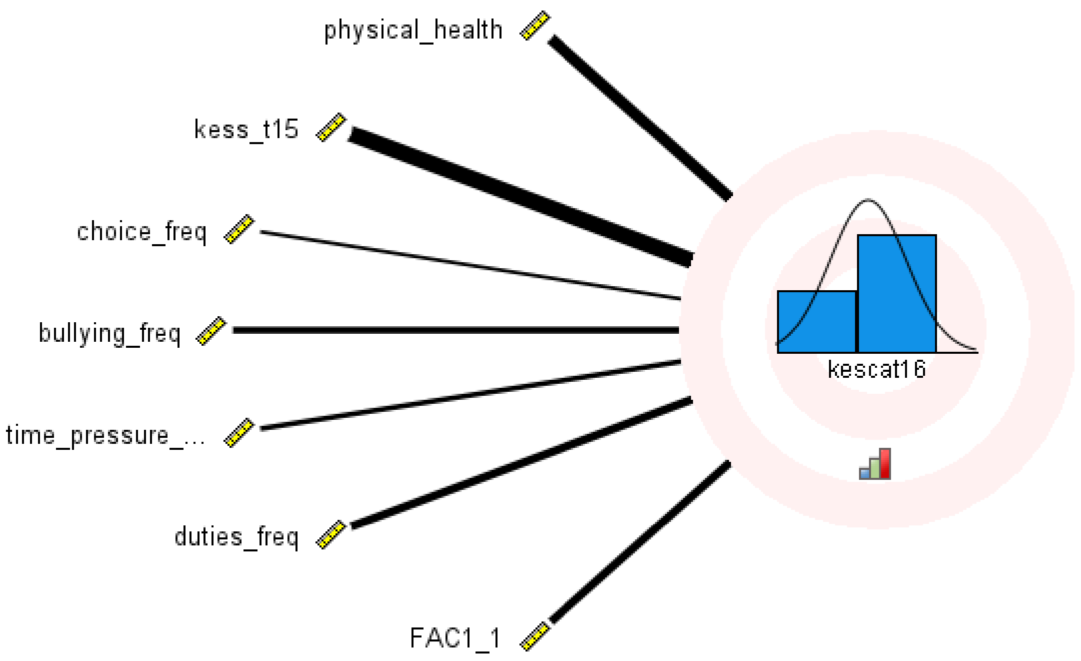

Fixed Coefficients[a]

Model Term	Coefficient	Std. Error	t	Sig.	95% Confidence Interval		Exp(Coefficient)	95% Confidence Interval for Exp(Coefficient)	
					Lower	Upper		Lower	Upper
Intercept	-1.752	.3791	-4.622	.000	-2.496	-1.009	.173	.082	.365
physical_health	.263	.0866	3.043	.002	.094	.433	1.301	1.098	1.542
kess_t15	1.310	.1549	8.457	.000	1.006	1.614	3.705	2.734	5.021
choice_freq	.027	.0536	.513	.608	-.078	.133	1.028	.925	1.142
bullying_freq	.181	.1063	1.699	.090	-.028	.389	1.198	.972	1.476
time_pressure_freq	-.074	.0565	-1.306	.192	-.185	.037	.929	.831	1.038
duties_freq	-.136	.0533	-2.560	.011	-.241	-.032	.872	.786	.969
FAC1_1	-.167	.0743	-2.252	.025	-.313	-.022	.846	.731	.979

Probability distribution: Binomial

Link function: Logit

a. Target: Kessler16 > 1 = 1

Fixed Coefficients

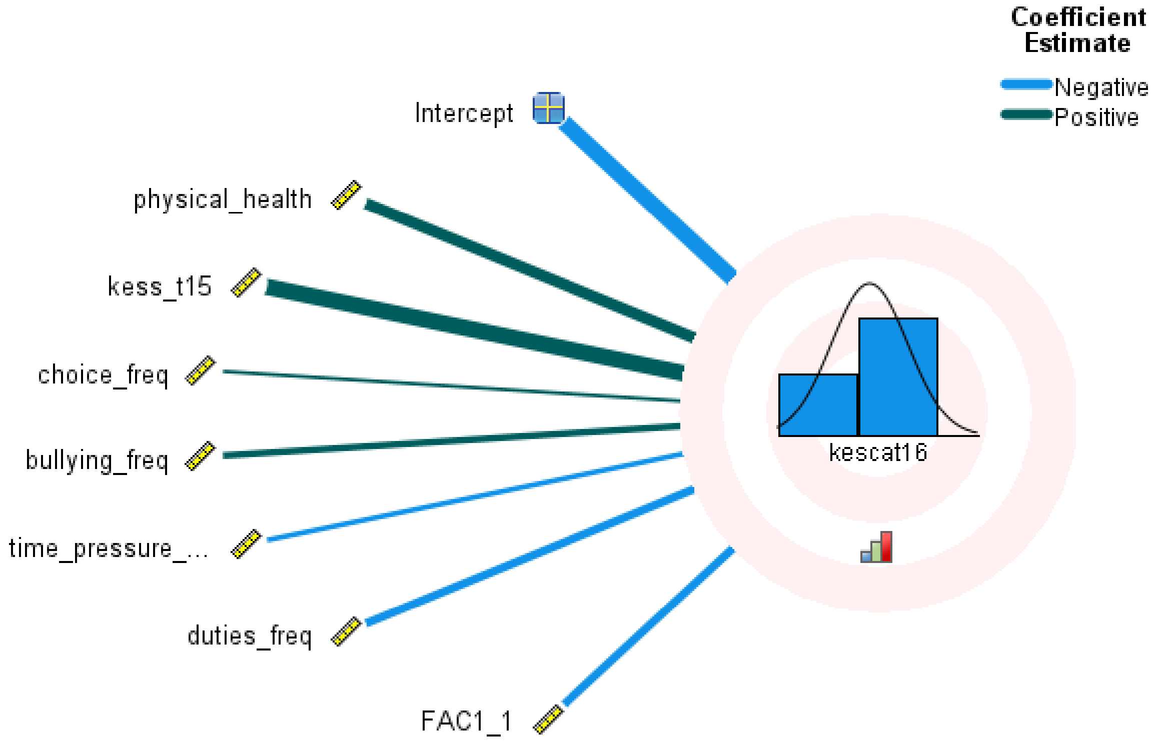

Random Effect Covariances

Random Effect Block 1

Random Effect Block	Intercept
Intercept	.000

Covariance Structure: Variance components

Subject Specification: companyId15

Covariance Parameters

Covariance Parameters Summary

Covariance Parameters	Residual Effect	0
	Random Effects	1
Design Matrix Columns	Fixed Effects	8
	Random Effects	1[a]
Common Subjects		58

Common subjects are based on the subject specifications for the residual and random effects and are used to chunk the data for better performance.

a. This is the number of columns per common subject.

Residual Effect

Residual Effect	Estimate	Std. Error	Z	Sig.	95% Confidence Interval	
					Lower	Upper
Variance	1.000	.	.	.	.	.

Covariance Structure: Scaled Identity

Subject Specification: (None)

Random Effect

Random Effect Covariance	Estimate	Std. Error	Z	Sig.	95% Confidence Interval	
					Lower	Upper
Var(Intercept)	.000[a]	.	.	.	.	.

Covariance Structure: Variance components

Subject Specification: companyId15

a. This parameter is redundant.

Random Effects Parameters

Empirical Best Linear Unbiased Predictions[a]

company_code	Parameter	Prediction	Std. Error	df	Sig.	95% Confidence Interval	
						Lower Bound	Upper Bound

2	Intercept	-4.130E-14	5.900E-15	1092	3.212E-12	-5.280E-14	-2.980E-14
4	Intercept	-9.100E-15	2.900E-15	1092	.002	-1.480E-14	-3.500E-15
6	Intercept	-1.000E-14	7.400E-15	1092	.179	-2.460E-14	4.600E-15
22	Intercept	-3.300E-14	1.300E-15	1092	.000	-3.550E-14	-3.050E-14
24	Intercept	-2.700E-14	1.020E-14	1092	.008	-4.710E-14	-6.900E-15
30	Intercept	1.530E-14	3.900E-15	1092	8.728E-5	7.700E-15	2.290E-14
36	Intercept	-2.450E-14	2.700E-15	1092	.000	-2.970E-14	-1.920E-14
40	Intercept	3.040E-14	1.700E-15	1092	.000	2.700E-14	3.380E-14
50	Intercept	2.300E-14	1.600E-15	1092	.000	1.980E-14	2.620E-14
61	Intercept	-1.920E-14	3.600E-15	1092	1.301E-7	-2.630E-14	-1.210E-14
65	Intercept	-3.140E-14	2.900E-15	1092	.000	-3.700E-14	-2.570E-14
68	Intercept	3.680E-14	2.900E-15	1092	.000	3.100E-14	4.260E-14
69	Intercept	-1.000E-16	2.800E-15	1092	.961	-5.700E-15	5.500E-15
71	Intercept	6.800E-15	1.900E-15	1092	.000	3.100E-15	1.050E-14
72	Intercept	-2.920E-14	3.600E-15	1092	7.000E-16	-3.620E-14	-2.220E-14
74	Intercept	-8.700E-15	8.000E-16	1092	.000	-1.020E-14	-7.200E-15
75	Intercept	-3.000E-14	2.800E-15	1092	.000	-3.540E-14	-2.450E-14
80	Intercept	-5.700E-15	2.000E-15	1092	.004	-9.500E-15	-1.800E-15
81	Intercept	1.600E-15	3.100E-15	1092	.603	-4.500E-15	7.700E-15
83	Intercept	3.480E-14	4.000E-15	1092	.000	2.690E-14	4.260E-14
84	Intercept	1.090E-14	1.800E-15	1092	4.534E-9	7.300E-15	1.450E-14
92	Intercept	-5.200E-15	8.700E-15	1092	.555	-2.230E-14	1.200E-14
93	Intercept	3.800E-15	1.900E-15	1092	.051	.000	7.500E-15
98	Intercept	-3.540E-14	5.300E-15	1092	3.641E-11	-4.580E-14	-2.500E-14
100	Intercept	8.900E-15	3.880E-14	1092	.818	-6.720E-14	8.510E-14
106	Intercept	-2.200E-15	9.000E-16	1092	.022	-4.000E-15	-3.000E-16

107	Intercept	2.930E-14	2.000E-15	1092	.000	2.550E-14	3.320E-14
108	Intercept	-6.170E-14	8.000E-15	1092	2.530E-14	-7.740E-14	-4.610E-14
110	Intercept	1.990E-14	5.240E-14	1092	.704	-8.290E-14	1.228E-13
114	Intercept	-4.750E-14	2.900E-15	1092	.000	-5.320E-14	-4.180E-14
116	Intercept	-7.800E-15	2.300E-15	1092	.001	-1.240E-14	-3.200E-15
122	Intercept	2.650E-14	6.200E-15	1092	1.928E-5	1.440E-14	3.860E-14
125	Intercept	4.030E-14	1.780E-14	1092	.024	5.300E-15	7.520E-14
142	Intercept	9.100E-15	6.100E-15	1092	.134	-2.800E-15	2.100E-14
144	Intercept	3.660E-14	4.400E-15	1092	4.000E-16	2.790E-14	4.520E-14
145	Intercept	1.410E-14	2.200E-15	1092	3.472E-10	9.700E-15	1.840E-14
152	Intercept	2.060E-14	4.600E-15	1092	9.817E-6	1.150E-14	2.980E-14
154	Intercept	-8.500E-15	3.900E-15	1092	.029	-1.610E-14	-9.000E-16
156	Intercept	-3.900E-15	5.800E-15	1092	.508	-1.530E-14	7.600E-15
162	Intercept	-1.470E-14	4.600E-15	1092	.001	-2.370E-14	-5.700E-15
165	Intercept	2.900E-15	1.500E-15	1092	.055	-1.000E-16	5.800E-15
166	Intercept	6.000E-15	4.000E-16	1092	.000	5.200E-15	6.800E-15
169	Intercept	-1.850E-14	3.700E-15	1092	5.011E-7	-2.570E-14	-1.130E-14
170	Intercept	-1.150E-14	1.200E-15	1092	.000	-1.380E-14	-9.200E-15
173	Intercept	1.980E-14	2.900E-15	1092	2.049E-11	1.400E-14	2.550E-14
174	Intercept	-2.190E-14	2.600E-15	1092	2.000E-16	-2.700E-14	-1.680E-14
178	Intercept	-4.330E-14	4.900E-15	1092	.000	-5.290E-14	-3.380E-14
181	Intercept	-1.570E-14	2.700E-15	1092	6.899E-9	-2.100E-14	-1.050E-14
182	Intercept	7.200E-15	1.700E-15	1092	3.759E-5	3.800E-15	1.060E-14
192	Intercept	4.100E-15	2.100E-15	1092	.047	1.000E-16	8.200E-15
202	Intercept	1.200E-14	6.000E-15	1092	.047	2.000E-16	2.390E-14
204	Intercept	5.210E-14	1.070E-14	1092	1.421E-6	3.100E-14	7.310E-14

205	Intercept	1.200E-14	7.000E-16	1092	.000	1.060E-14	1.340E-14
206	Intercept	2.410E-14	2.600E-15	1092	.000	1.890E-14	2.920E-14
209	Intercept	-2.620E-14	8.600E-15	1092	.002	-4.300E-14	-9.400E-15
217	Intercept	4.200E-14	1.800E-14	1092	.020	6.600E-15	7.730E-14
226	Intercept	2.070E-14	3.600E-15	1092	1.510E-8	1.360E-14	2.780E-14
228	Intercept	2.180E-14	1.700E-15	1092	.000	1.850E-14	2.510E-14

a. Target: Kessler16 > 1 = 1

```
GENLINMIXED
  /DATA_STRUCTURE SUBJECTS=companyId15
  /FIELDS TARGET=kescat16 TRIALS=NONE OFFSET=NONE
  /TARGET_OPTIONS DISTRIBUTION=BINOMIAL LINK=LOGIT
  /FIXED  EFFECTS=physical_health kess_t15 choice_freq bullying_freq time_pressure_freq duties_freq
    FAC1_1 facsq USE_INTERCEPT=TRUE
  /RANDOM USE_INTERCEPT=TRUE SUBJECTS=companyId15 COVARIANCE_TYPE=VARIANCE_COMPONENTS SOLUTION=TRUE
  /BUILD_OPTIONS TARGET_CATEGORY_ORDER=DESCENDING INPUTS_CATEGORY_ORDER=DESCENDING
    MAX_ITERATIONS=100 CONFIDENCE_LEVEL=95 DF_METHOD=RESIDUAL COVB=ROBUST PCONVERGE=0.000001(ABSOLUTE)
    SCORING=0 SINGULAR=0.000000000001
  /EMMEANS_OPTIONS SCALE=ORIGINAL PADJUST=LSD.
```

Generalized Linear Mixed Models

Notes

Output Created	16-AUG-2021 11:07:38

Comments		
Input	Data	C:\Users\jwb12ppu\Documents\Kevin\MPlus backup\Multicomponent health and wellbeing v2\Multicomponent for Mplus cat.sav
	Active Dataset	DataSet2
	Filter	kescat15 = 0 (FILTER)
	Weight	<none>
	Split File	<none>
	N of Rows in Working Data File	1113

Syntax	GENLINMIXED<br>/DATA_STRUCTURE<br>SUBJECTS=companyId15<br>/FIELDS TARGET=kescat16<br>TRIALS=NONE OFFSET=NONE<br>/TARGET_OPTIONS<br>DISTRIBUTION=BINOMIAL<br>LINK=LOGIT<br>/FIXED<br>EFFECTS=physical_health<br>kess_t15 choice_freq bullying_freq<br>time_pressure_freq duties_freq<br>FAC1_1 facsq<br>USE_INTERCEPT=TRUE<br>/RANDOM<br>USE_INTERCEPT=TRUE<br>SUBJECTS=companyId15<br>COVARIANCE_TYPE=VARIANCE<br>_COMPONENTS<br>SOLUTION=TRUE<br>/BUILD_OPTIONS<br>TARGET_CATEGORY_ORDER=<br>DESCENDING<br>INPUTS_CATEGORY_ORDER=D<br>ESCENDING<br>MAX_ITERATIONS=100<br>CONFIDENCE_LEVEL=95<br>DF_METHOD=RESIDUAL

		COVB=ROBUST PCONVERGE=0.000001(ABSOLUTE) SCORING=0 SINGULAR=0.000000000001 /EMMEANS_OPTIONS SCALE=ORIGINAL PADJUST=LSD.
Resources	Processor Time	00:00:01.30
	Elapsed Time	00:00:01.22

Warnings

glmm: One or more records are not used in the analysis because they have one or more fields with invalid or missing values.
glmm: Valid values for events (target) and trials variables are non-negative and positive integers respectively, and the number of trials cannot be less than the number of events.
glmm: The final Hessian matrix is not positive definite although all convergence criteria are satisfied. The procedure continues despite this warning. Subsequent results produced are based on the last iteration. Validity of the model fit is uncertain.

Case Processing Summary

	N	Percent

Included	1100	98.8%
Excluded	13	1.2%
Total	1113	100.0%

Model Summary

Target		Kessler16 > 1 = 1
Probability Distribution		Binomial
Link Function		Logit
Information Criterion	Akaike Corrected	5011.551
	Bayesian	5016.543

Information criteria are based on the -2 log likelihood (5009.548) and are used to compare models. Models with smaller information criterion values fit better.

Data Structure[a]

	Subjects	Target
	company_code	Kessler16 > 1 = 1
Data for First Subject	2	1.00
	2	1.00
	2	.00
	2	.00
	2	1.00
	2	1.00
	2	.00
	2	.00

	2	.00
	2	.00
Total Number of Levels	58	

Only the first 10 records are displayed.

a. Target: Kessler16 > 1 = 1

Classification
Overall Percent Correct = 67.5%[a]

Observed		Predicted 1.00	.00
1.00	Count	639	80
	% within Observed	88.9%	11.1%
.00	Count	277	104
	% within Observed	72.7%	27.3%

a. Target: Kessler16 > 1 = 1

Fixed Effects[a]

Source	F	df1	df2	Sig.
Corrected Model	17.058	8	1091	.000
physical_health	8.977	1	1091	.003
kess_t15	70.521	1	1091	.000
choice_freq	.472	1	1091	.492
bullying_freq	2.621	1	1091	.106
time_pressure_freq	1.963	1	1091	.162

duties_freq	6.842	1	1091	.009
FAC1_1	7.959	1	1091	.005
facsq	6.503	1	1091	.011

Probability distribution: Binomial

Link function: Logit

a. Target: Kessler16 > 1 = 1

Fixed Effects

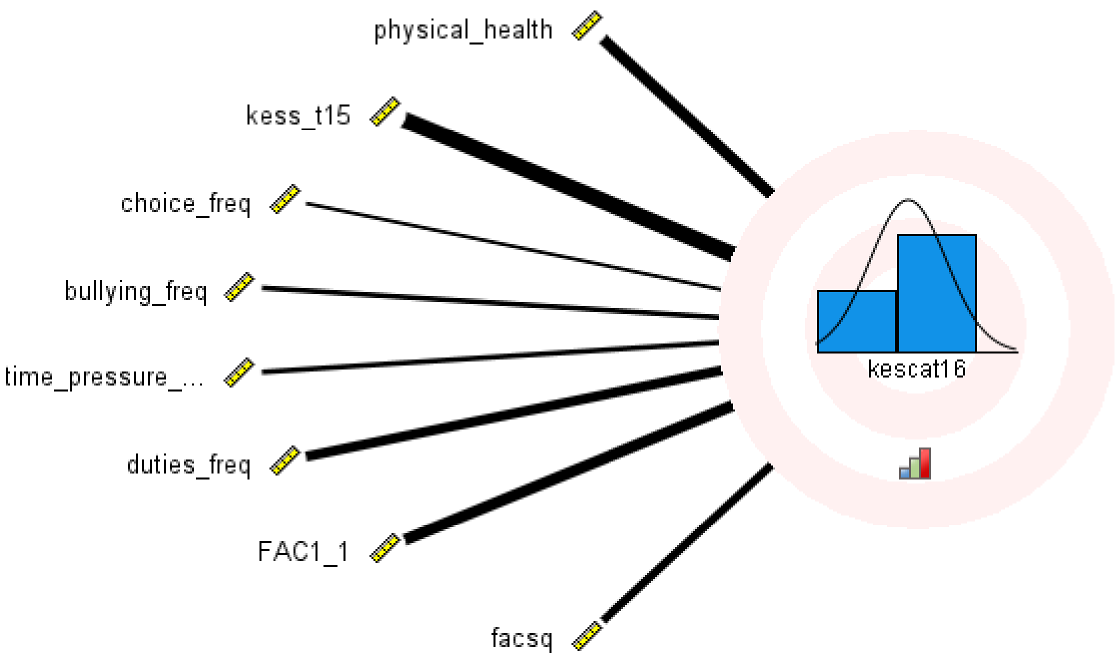

Fixed Coefficients[a]

Model Term	Coefficient	Std. Error	t	Sig.	95% Confidence Interval		Exp(Coefficient)	95% Confidence Interval for Exp(Coefficient)	
					Lower	Upper		Lower	Upper
Intercept	-1.619	.3812	-4.246	.000	-2.367	-.871	.198	.094	.419
physical_health	.261	.0871	2.996	.003	.090	.432	1.298	1.094	1.540
kess_t15	1.329	.1583	8.398	.000	1.019	1.640	3.778	2.769	5.154
choice_freq	.036	.0531	.687	.492	-.068	.141	1.037	.934	1.151
bullying_freq	.166	.1025	1.619	.106	-.035	.367	1.180	.965	1.443
time_pressure_freq	-.079	.0566	-1.401	.162	-.190	.032	.924	.827	1.032
duties_freq	-.137	.0525	-2.616	.009	-.240	-.034	.872	.786	.966
FAC1_1	-.184	.0651	-2.821	.005	-.311	-.056	.832	.732	.946
facsq	-.153	.0601	-2.550	.011	-.271	-.035	.858	.762	.965

Probability distribution: Binomial

Link function: Logit

a. Target: Kessler16 > 1 = 1

Fixed Coefficients

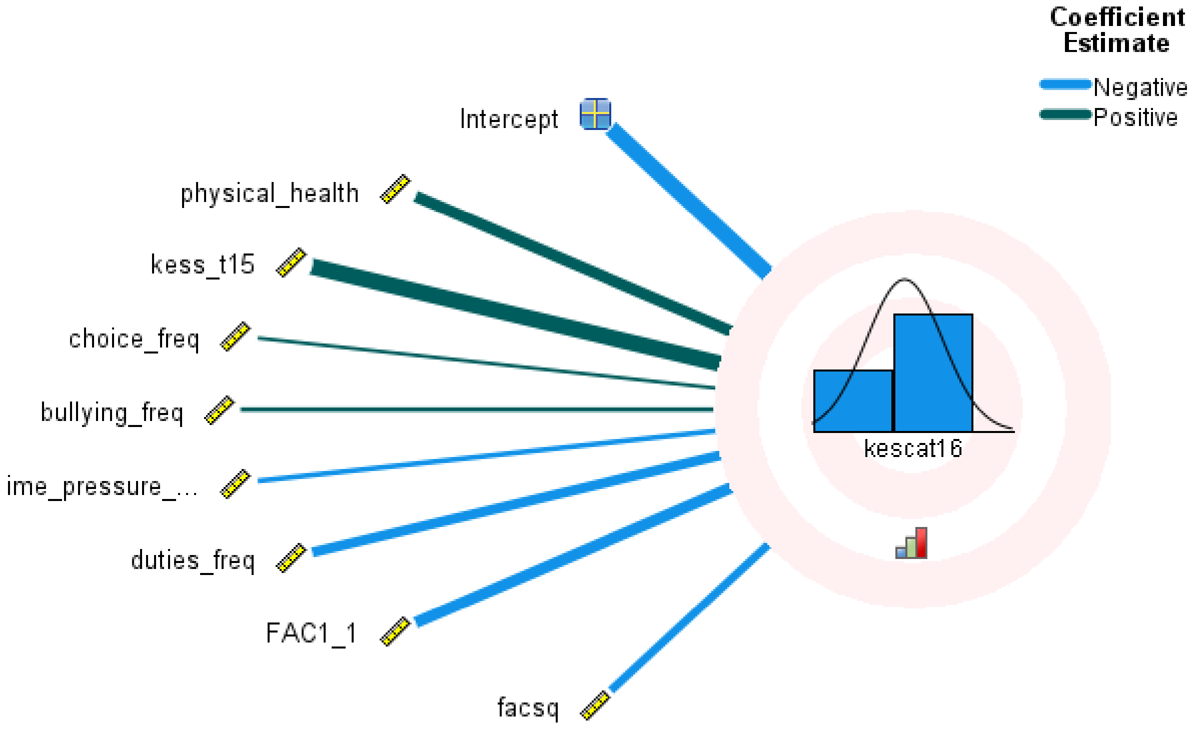

Random Effect Covariances

Random Effect Block 1

Random Effect Block	Intercept
Intercept	.000

Covariance Structure: Variance components

Subject Specification: companyId15

Covariance Parameters

Covariance Parameters Summary

Covariance Parameters	Residual Effect	0
	Random Effects	1
Design Matrix Columns	Fixed Effects	9
	Random Effects	1[a]
Common Subjects		58

Common subjects are based on the subject specifications for the residual and random effects and are used to chunk the data for better performance.

a. This is the number of columns per common subject.

Residual Effect

Residual Effect	Estimate	Std. Error	Z	Sig.	95% Confidence Interval	
					Lower	Upper
Variance	1.000	.	.	.	.	.

Covariance Structure: Scaled Identity

Subject Specification: (None)

Random Effect

Random Effect Covariance	Estimate	Std. Error	Z	Sig.	95% Confidence Interval	
					Lower	Upper
Var(Intercept)	6.553E-10[a]	.	.	.	.	.

Covariance Structure: Variance components

Subject Specification: companyId15

a. This parameter is redundant.

Random Effects Parameters

Empirical Best Linear Unbiased Predictions[a]

company_code	Parameter	Prediction	Std. Error	df	Sig.	95% Confidence Interval	
						Lower Bound	Upper Bound

2	Intercept	-1.482E-9	2.560E-5	1091	1.000	-5.023E-5	5.023E-5
4	Intercept	8.679E-12	2.560E-5	1091	1.000	-5.023E-5	5.023E-5
6	Intercept	7.065E-10	2.560E-5	1091	1.000	-5.023E-5	5.023E-5
22	Intercept	-1.368E-9	2.560E-5	1091	1.000	-5.023E-5	5.023E-5
24	Intercept	-1.159E-9	2.560E-5	1091	1.000	-5.023E-5	5.023E-5
30	Intercept	9.041E-10	2.560E-5	1091	1.000	-5.023E-5	5.023E-5
36	Intercept	-1.215E-9	2.560E-5	1091	1.000	-5.023E-5	5.023E-5
40	Intercept	1.207E-9	2.560E-5	1091	1.000	-5.023E-5	5.023E-5
50	Intercept	9.149E-10	2.560E-5	1091	1.000	-5.023E-5	5.023E-5
61	Intercept	-7.401E-10	2.560E-5	1091	1.000	-5.023E-5	5.023E-5
65	Intercept	-8.590E-10	2.560E-5	1091	1.000	-5.023E-5	5.023E-5
68	Intercept	1.417E-9	2.560E-5	1091	1.000	-5.023E-5	5.023E-5
69	Intercept	-2.348E-10	2.560E-5	1091	1.000	-5.023E-5	5.023E-5
71	Intercept	4.341E-10	2.560E-5	1091	1.000	-5.023E-5	5.023E-5
72	Intercept	-8.376E-10	2.560E-5	1091	1.000	-5.023E-5	5.023E-5
74	Intercept	-3.429E-10	2.560E-5	1091	1.000	-5.023E-5	5.023E-5
75	Intercept	-1.110E-9	2.560E-5	1091	1.000	-5.023E-5	5.023E-5
80	Intercept	-3.078E-10	2.560E-5	1091	1.000	-5.023E-5	5.023E-5
81	Intercept	-9.483E-11	2.560E-5	1091	1.000	-5.023E-5	5.023E-5
83	Intercept	1.672E-9	2.560E-5	1091	1.000	-5.023E-5	5.023E-5
84	Intercept	3.651E-10	2.560E-5	1091	1.000	-5.023E-5	5.023E-5
92	Intercept	3.160E-10	2.560E-5	1091	1.000	-5.023E-5	5.023E-5
93	Intercept	3.736E-10	2.560E-5	1091	1.000	-5.023E-5	5.023E-5
98	Intercept	-1.196E-9	2.560E-5	1091	1.000	-5.023E-5	5.023E-5
100	Intercept	-2.417E-9	2.560E-5	1091	1.000	-5.023E-5	5.023E-5
106	Intercept	-1.427E-10	2.560E-5	1091	1.000	-5.023E-5	5.023E-5

107	Intercept	1.131E-9	2.560E-5	1091	1.000	-5.023E-5	5.023E-5
108	Intercept	-1.534E-9	2.560E-5	1091	1.000	-5.023E-5	5.023E-5
110	Intercept	1.161E-9	2.560E-5	1091	1.000	-5.023E-5	5.023E-5
114	Intercept	-2.182E-9	2.560E-5	1091	1.000	-5.023E-5	5.023E-5
116	Intercept	-4.617E-10	2.560E-5	1091	1.000	-5.023E-5	5.023E-5
122	Intercept	7.133E-10	2.560E-5	1091	1.000	-5.023E-5	5.023E-5
125	Intercept	2.104E-9	2.560E-5	1091	1.000	-5.023E-5	5.023E-5
142	Intercept	3.641E-12	2.560E-5	1091	1.000	-5.023E-5	5.023E-5
144	Intercept	1.900E-9	2.560E-5	1091	1.000	-5.023E-5	5.023E-5
145	Intercept	4.146E-10	2.560E-5	1091	1.000	-5.023E-5	5.023E-5
152	Intercept	1.327E-9	2.560E-5	1091	1.000	-5.023E-5	5.023E-5
154	Intercept	-4.037E-10	2.560E-5	1091	1.000	-5.023E-5	5.023E-5
156	Intercept	1.914E-10	2.560E-5	1091	1.000	-5.023E-5	5.023E-5
162	Intercept	-3.989E-10	2.560E-5	1091	1.000	-5.023E-5	5.023E-5
165	Intercept	2.353E-10	2.560E-5	1091	1.000	-5.023E-5	5.023E-5
166	Intercept	2.281E-10	2.560E-5	1091	1.000	-5.023E-5	5.023E-5
169	Intercept	-1.299E-10	2.560E-5	1091	1.000	-5.023E-5	5.023E-5
170	Intercept	-5.306E-10	2.560E-5	1091	1.000	-5.023E-5	5.023E-5
173	Intercept	7.604E-10	2.560E-5	1091	1.000	-5.023E-5	5.023E-5
174	Intercept	-1.147E-9	2.560E-5	1091	1.000	-5.023E-5	5.023E-5
178	Intercept	-2.267E-9	2.560E-5	1091	1.000	-5.023E-5	5.023E-5
181	Intercept	-7.634E-10	2.560E-5	1091	1.000	-5.023E-5	5.023E-5
182	Intercept	2.683E-10	2.560E-5	1091	1.000	-5.023E-5	5.023E-5
192	Intercept	3.495E-10	2.560E-5	1091	1.000	-5.023E-5	5.023E-5
202	Intercept	3.995E-10	2.560E-5	1091	1.000	-5.023E-5	5.023E-5
204	Intercept	1.543E-9	2.560E-5	1091	1.000	-5.023E-5	5.023E-5

205	Intercept	4.711E-10	2.560E-5	1091	1.000	-5.023E-5	5.023E-5
206	Intercept	8.376E-10	2.560E-5	1091	1.000	-5.023E-5	5.023E-5
209	Intercept	-1.866E-9	2.560E-5	1091	1.000	-5.023E-5	5.023E-5
217	Intercept	1.227E-9	2.560E-5	1091	1.000	-5.023E-5	5.023E-5
226	Intercept	8.248E-10	2.560E-5	1091	1.000	-5.023E-5	5.023E-5
228	Intercept	7.797E-10	2.560E-5	1091	1.000	-5.023E-5	5.023E-5

a. Target: Kessler16 > 1 = 1

```
AGGREGATE
  /OUTFILE=* MODE=ADDVARIABLES OVERWRITE = YES
  /BREAK=companyId15
  /choicem2=MEAN(choice_freq)
  /bullym2=MEAN(bullying_freq)
  /timem2=MEAN(time_pressure_freq)
  /dutiesm2=MEAN(duties_freq).

COMPUTE choicecwc2 = choice_freq - choicem2.
COMPUTE bullycwc2 = bullying_freq - bullym2.
COMPUTE timecwc2 = time_pressure_freq - timem2.
COMPUTE dutiescwc2 = duties_freq - dutiesm2.

COMPUTE fac2choi = fac1_1 * choicecwc2.
COMPUTE fac2bull = fac1_1 * bullycwc2.
COMPUTE fac2time = fac1_1 *  timecwc2.
COMPUTE fac2duti = fac1_1 * dutiescwc2.

GENLINMIXED
  /DATA_STRUCTURE SUBJECTS=companyId15
  /FIELDS TARGET=kescat16 TRIALS=NONE OFFSET=NONE
  /TARGET_OPTIONS DISTRIBUTION=BINOMIAL LINK=LOGIT
  /FIXED  EFFECTS=physical_health kess_t15 choicecwc2 choicem2 fac2choi
  bullying_freq time_pressure_freq duties_freq
    FAC1_1 USE_INTERCEPT=TRUE
  /RANDOM USE_INTERCEPT=TRUE SUBJECTS=companyId15 COVARIANCE_TYPE=VARIANCE_COMPONENTS SOLUTION=TRUE
  /BUILD_OPTIONS TARGET_CATEGORY_ORDER=DESCENDING INPUTS_CATEGORY_ORDER=DESCENDING
    MAX_ITERATIONS=100 CONFIDENCE_LEVEL=95 DF_METHOD=RESIDUAL COVB=ROBUST PCONVERGE=0.000001(ABSOLUTE)
```

```
  SCORING=0 SINGULAR=0.000000000001
/EMMEANS_OPTIONS SCALE=ORIGINAL PADJUST=LSD.
```

Generalized Linear Mixed Models

Notes

Output Created		16-AUG-2021 11:07:40
Comments		
Input	Data	C:\Users\jwb12ppu\Documents\Kevin\MPlus backup\Multicomponent health and wellbeing v2\Multicomponent for Mplus cat.sav
	Active Dataset	DataSet2
	Filter	kescat15 = 0 (FILTER)
	Weight	<none>
	Split File	<none>
	N of Rows in Working Data File	1113

Syntax	GENLINMIXED<br>/DATA_STRUCTURE SUBJECTS=companyId15<br>/FIELDS TARGET=kescat16 TRIALS=NONE OFFSET=NONE<br>/TARGET_OPTIONS DISTRIBUTION=BINOMIAL LINK=LOGIT<br>/FIXED EFFECTS=physical_health kess_t15 choicecwc2 choicem2 fac2choi<br>bullying_freq time_pressure_freq duties_freq<br>FAC1_1 USE_INTERCEPT=TRUE<br>/RANDOM USE_INTERCEPT=TRUE SUBJECTS=companyId15 COVARIANCE_TYPE=VARIANCE_COMPONENTS SOLUTION=TRUE<br>/BUILD_OPTIONS TARGET_CATEGORY_ORDER=DESCENDING INPUTS_CATEGORY_ORDER=DESCENDING

		MAX_ITERATIONS=100 CONFIDENCE_LEVEL=95 DF_METHOD=RESIDUAL COVB=ROBUST PCONVERGE=0.000001(ABSOLUTE) SCORING=0 SINGULAR=0.000000000001 /EMMEANS_OPTIONS SCALE=ORIGINAL PADJUST=LSD.
Resources	Processor Time	00:00:01.30
	Elapsed Time	00:00:01.15

Warnings

glmm: One or more records are not used in the analysis because they have one or more fields with invalid or missing values.
glmm: Valid values for events (target) and trials variables are non-negative and positive integers respectively, and the number of trials cannot be less than the number of events.
glmm: The estimated covariance matrix of the random effects (the G matrix) is not positive definite. The procedure continues despite this warning. Subsequent results produced are based on the last iteration. Validity of the model fit is uncertain.

glmm: The final Hessian matrix is not positive definite although all convergence criteria are satisfied. The procedure continues despite this warning. Subsequent results produced are based on the last iteration. Validity of the model fit is uncertain.

Case Processing Summary

	N	Percent
Included	1100	98.8%
Excluded	13	1.2%
Total	1113	100.0%

Model Summary

Target		Kessler16 > 1 = 1
Probability Distribution		Binomial
Link Function		Logit
Information Criterion	Akaike Corrected	5003.905
	Bayesian	5008.895

Information criteria are based on the -2 log likelihood (5001.901) and are used to compare models. Models with smaller information criterion values fit better.

Data Structure[a]

Subjects	Target

	company_code	Kessler16 > 1 = 1
Data for First Subject	2	1.00
	2	1.00
	2	.00
	2	.00
	2	1.00
	2	1.00
	2	.00
	2	.00
	2	.00
	2	.00
Total Number of Levels	58	

Only the first 10 records are displayed.

a. Target: Kessler16 > 1 = 1

Classification

Overall Percent Correct = 67.6%[a]

Observed		Predicted 1.00	Predicted .00
1.00	Count	631	88
	% within Observed	87.8%	12.2%
.00	Count	268	113
	% within Observed	70.3%	29.7%

a. Target: Kessler16 > 1 = 1

Fixed Effects[a]

Source	F	df1	df2	Sig.
Corrected Model	16.362	9	1090	.000
physical_health	8.782	1	1090	.003
kess_t15	70.779	1	1090	.000
choicecwc2	.807	1	1090	.369
choicem2	1.534	1	1090	.216
fac2choi	.384	1	1090	.536
bullying_freq	2.836	1	1090	.092
time_pressure_freq	2.067	1	1090	.151
duties_freq	6.981	1	1090	.008
FAC1_1	3.905	1	1090	.048

Probability distribution: Binomial

Link function: Logit

a. Target: Kessler16 > 1 = 1

Fixed Effects

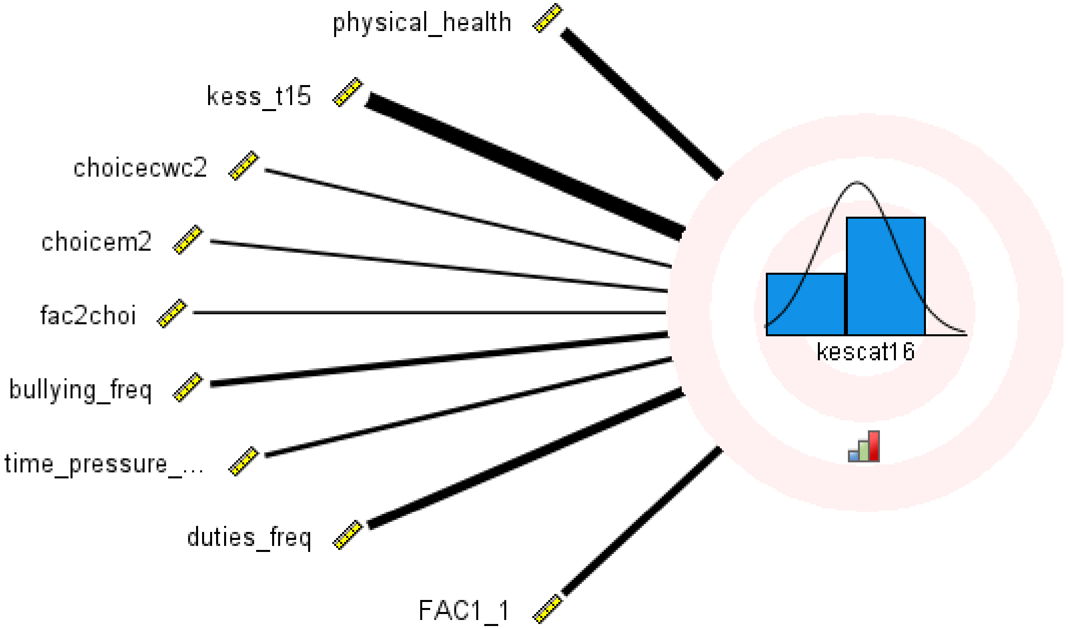

Fixed Coefficients[a]

Model Term	Coefficient	Std. Error	t	Sig.	95% Confidence Interval		Exp(Coefficient)	95% Confidence Interval for Exp(Coefficient)	
					Lower	Upper		Lower	Upper
Intercept	-.903	.6530	-1.383	.167	-2.184	.378	.405	.113	1.460
physical_health	.256	.0864	2.963	.003	.086	.425	1.292	1.090	1.530
kess_t15	1.307	.1554	8.413	.000	1.002	1.612	3.696	2.725	5.014
choicecwc2	.051	.0563	.898	.369	-.060	.161	1.052	.942	1.175
choicem2	-.252	.2036	-1.238	.216	-.652	.147	.777	.521	1.159
fac2choi	.036	.0586	.619	.536	-.079	.151	1.037	.924	1.163
bullying_freq	.175	.1039	1.684	.092	-.029	.379	1.191	.972	1.460
time_pressure_freq	-.083	.0580	-1.438	.151	-.197	.030	.920	.821	1.031
duties_freq	-.137	.0519	-2.642	.008	-.239	-.035	.872	.787	.965
FAC1_1	-.150	.0757	-1.976	.048	-.298	-.001	.861	.742	.999

Probability distribution: Binomial

Link function: Logit

a. Target: Kessler16 > 1 = 1

Fixed Coefficients

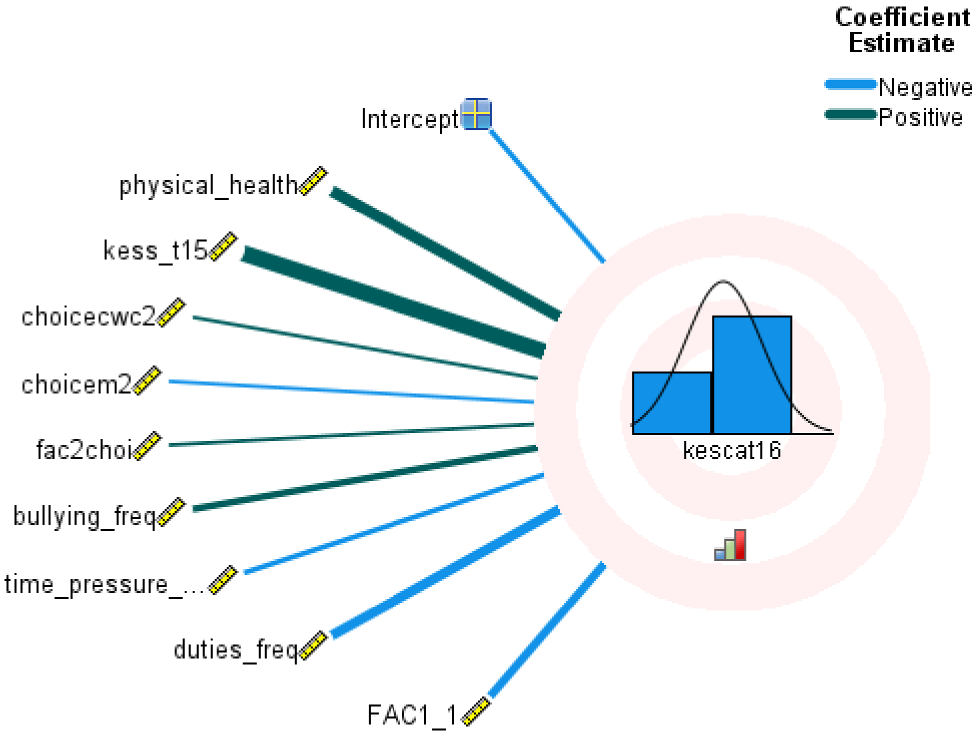

Random Effect Covariances

Random Effect Block 1

Random Effect Block	Intercept
Intercept	.000

Covariance Structure: Variance components

Subject Specification: companyId15

Covariance Parameters

Covariance Parameters Summary

Covariance Parameters	Residual Effect	0
	Random Effects	1
Design Matrix Columns	Fixed Effects	10
	Random Effects	1[a]
Common Subjects		58

Common subjects are based on the subject specifications for the residual and random effects and are used to chunk the data for better performance.

a. This is the number of columns per common subject.

Residual Effect

Residual Effect	Estimate	Std. Error	Z	Sig.	95% Confidence Interval	
					Lower	Upper
Variance	1.000	.	.	.	.	.

Covariance Structure: Scaled Identity

Subject Specification: (None)

Random Effect

Random Effect Covariance	Estimate	Std. Error	Z	Sig.	95% Confidence Interval	
					Lower	Upper
Var(Intercept)	.000[a]	.	.	.	.	.

Covariance Structure: Variance components

Subject Specification: companyId15

a. This parameter is redundant.

Random Effects Parameters

Empirical Best Linear Unbiased Predictions[a]

company_code	Parameter	Prediction	Std. Error	df	Sig.	95% Confidence Interval	
						Lower Bound	Upper Bound

2	Intercept	-1.642E-13	2.390E-14	1090	1.107E-11	-2.111E-13	-1.173E-13
4	Intercept	-2.620E-14	1.360E-14	1090	.054	-5.280E-14	4.000E-16
6	Intercept	-4.190E-14	2.980E-14	1090	.160	-1.004E-13	1.660E-14
22	Intercept	-1.400E-13	5.600E-15	1090	.000	-1.510E-13	-1.290E-13
24	Intercept	-8.140E-14	5.240E-14	1090	.121	-1.843E-13	2.140E-14
30	Intercept	5.540E-14	1.650E-14	1090	.001	2.300E-14	8.780E-14
36	Intercept	-6.730E-14	2.780E-14	1090	.016	-1.219E-13	-1.270E-14
40	Intercept	1.255E-13	6.900E-15	1090	.000	1.121E-13	1.390E-13
50	Intercept	8.970E-14	7.300E-15	1090	.000	7.540E-14	1.041E-13
61	Intercept	-7.190E-14	1.560E-14	1090	4.376E-6	-1.025E-13	-4.140E-14
65	Intercept	-1.274E-13	1.140E-14	1090	.000	-1.497E-13	-1.051E-13
68	Intercept	1.809E-13	2.570E-14	1090	3.300E-12	1.305E-13	2.313E-13
69	Intercept	2.370E-14	2.330E-14	1090	.310	-2.200E-14	6.940E-14
71	Intercept	2.570E-14	7.300E-15	1090	.000	1.140E-14	4.000E-14
72	Intercept	-1.088E-13	1.800E-14	1090	1.937E-9	-1.441E-13	-7.360E-14
74	Intercept	-3.460E-14	3.300E-15	1090	.000	-4.100E-14	-2.820E-14
75	Intercept	-1.141E-13	1.460E-14	1090	1.110E-14	-1.426E-13	-8.550E-14
80	Intercept	-1.450E-14	1.080E-14	1090	.179	-3.570E-14	6.700E-15
81	Intercept	3.180E-14	2.370E-14	1090	.180	-1.470E-14	7.820E-14
83	Intercept	1.592E-13	2.270E-14	1090	3.885E-12	1.147E-13	2.037E-13
84	Intercept	5.160E-14	9.500E-15	1090	6.516E-8	3.300E-14	7.030E-14
92	Intercept	-1.350E-14	3.640E-14	1090	.711	-8.480E-14	5.790E-14
93	Intercept	1.230E-14	7.300E-15	1090	.094	-2.100E-15	2.660E-14
98	Intercept	-1.274E-13	2.750E-14	1090	4.099E-6	-1.814E-13	-7.340E-14
100	Intercept	-9.120E-14	1.474E-13	1090	.536	-3.804E-13	1.979E-13
106	Intercept	-2.700E-15	6.700E-15	1090	.684	-1.580E-14	1.040E-14

107	Intercept	1.440E-13	2.050E-14	1090	4.139E-12	1.037E-13	1.842E-13
108	Intercept	-2.554E-13	3.270E-14	1090	1.240E-14	-3.195E-13	-1.913E-13
110	Intercept	9.590E-14	2.142E-13	1090	.655	-3.244E-13	5.162E-13
114	Intercept	-1.599E-13	3.170E-14	1090	5.256E-7	-2.221E-13	-9.780E-14
116	Intercept	-2.700E-14	1.080E-14	1090	.013	-4.820E-14	-5.800E-15
122	Intercept	5.620E-14	3.640E-14	1090	.122	-1.520E-14	1.276E-13
125	Intercept	1.643E-13	7.230E-14	1090	.023	2.250E-14	3.061E-13
142	Intercept	3.570E-14	2.380E-14	1090	.134	-1.100E-14	8.240E-14
144	Intercept	1.565E-13	1.850E-14	1090	.000	1.202E-13	1.929E-13
145	Intercept	6.470E-14	1.130E-14	1090	1.486E-8	4.240E-14	8.690E-14
152	Intercept	8.380E-14	1.850E-14	1090	6.297E-6	4.760E-14	1.201E-13
154	Intercept	-1.990E-14	2.000E-14	1090	.320	-5.920E-14	1.940E-14
156	Intercept	-4.090E-14	2.520E-14	1090	.105	-9.040E-14	8.500E-15
162	Intercept	-3.450E-14	2.690E-14	1090	.200	-8.740E-14	1.830E-14
165	Intercept	1.680E-14	7.100E-15	1090	.018	2.900E-15	3.070E-14
166	Intercept	2.980E-14	4.200E-15	1090	3.007E-12	2.150E-14	3.800E-14
169	Intercept	-7.080E-14	1.610E-14	1090	1.243E-5	-1.024E-13	-3.910E-14
170	Intercept	-3.720E-14	9.800E-15	1090	.000	-5.650E-14	-1.790E-14
173	Intercept	8.370E-14	1.200E-14	1090	4.832E-12	6.020E-14	1.073E-13
174	Intercept	-7.820E-14	1.470E-14	1090	1.134E-7	-1.070E-13	-4.950E-14
178	Intercept	-1.590E-13	2.630E-14	1090	1.964E-9	-2.105E-13	-1.074E-13
181	Intercept	-8.030E-14	1.430E-14	1090	2.794E-8	-1.084E-13	-5.210E-14
182	Intercept	4.040E-14	1.130E-14	1090	.000	1.830E-14	6.260E-14
192	Intercept	2.090E-14	9.300E-15	1090	.025	2.600E-15	3.920E-14
202	Intercept	2.750E-14	2.790E-14	1090	.325	-2.730E-14	8.230E-14
204	Intercept	1.380E-13	5.770E-14	1090	.017	2.480E-14	2.513E-13

205	Intercept	4.240E-14	5.100E-15	1090	2.000E-16	3.240E-14	5.240E-14
206	Intercept	8.930E-14	1.110E-14	1090	2.200E-15	6.750E-14	1.111E-13
209	Intercept	-1.587E-13	4.350E-14	1090	.000	-2.439E-13	-7.340E-14
217	Intercept	1.190E-13	7.550E-14	1090	.115	-2.910E-14	2.671E-13
226	Intercept	9.230E-14	1.660E-14	1090	3.680E-8	5.960E-14	1.249E-13
228	Intercept	9.180E-14	7.300E-15	1090	.000	7.740E-14	1.061E-13

a. Target: Kessler16 > 1 = 1

```
GENLINMIXED
  /DATA_STRUCTURE SUBJECTS=companyId15
  /FIELDS TARGET=kescat16 TRIALS=NONE OFFSET=NONE
  /TARGET_OPTIONS DISTRIBUTION=BINOMIAL LINK=LOGIT
  /FIXED  EFFECTS=physical_health kess_t15 choice_freq bullycwc2 bullym2 fac2bull
   time_pressure_freq duties_freq
    FAC1_1 USE_INTERCEPT=TRUE
  /RANDOM USE_INTERCEPT=TRUE SUBJECTS=companyId15 COVARIANCE_TYPE=VARIANCE_COMPONENTS SOLUTION=TRUE
  /BUILD_OPTIONS TARGET_CATEGORY_ORDER=DESCENDING INPUTS_CATEGORY_ORDER=DESCENDING
    MAX_ITERATIONS=100 CONFIDENCE_LEVEL=95 DF_METHOD=RESIDUAL COVB=ROBUST PCONVERGE=0.000001(ABSOLUTE)
    SCORING=0 SINGULAR=0.000000000001
  /EMMEANS_OPTIONS SCALE=ORIGINAL PADJUST=LSD.
```

Generalized Linear Mixed Models

Notes

Output Created	16-AUG-2021 11:07:41

Comments		
Input	Data	C:\Users\jwb12ppu\Documents\Kevin\MPlus backup\Multicomponent health and wellbeing v2\Multicomponent for Mplus cat.sav
	Active Dataset	DataSet2
	Filter	kescat15 = 0 (FILTER)
	Weight	<none>
	Split File	<none>
	N of Rows in Working Data File	1113

Syntax	GENLINMIXED<br>/DATA_STRUCTURE<br>SUBJECTS=companyId15<br>/FIELDS TARGET=kescat16<br>TRIALS=NONE OFFSET=NONE<br>/TARGET_OPTIONS<br>DISTRIBUTION=BINOMIAL<br>LINK=LOGIT<br>/FIXED<br>EFFECTS=physical_health<br>kess_t15 choice_freq bullycwc2<br>bullym2 fac2bull<br>time_pressure_freq duties_freq<br>FAC1_1<br>USE_INTERCEPT=TRUE<br>/RANDOM<br>USE_INTERCEPT=TRUE<br>SUBJECTS=companyId15<br>COVARIANCE_TYPE=VARIANCE<br>_COMPONENTS<br>SOLUTION=TRUE<br>/BUILD_OPTIONS<br>TARGET_CATEGORY_ORDER=<br>DESCENDING<br>INPUTS_CATEGORY_ORDER=D<br>ESCENDING<br>MAX_ITERATIONS=100<br>CONFIDENCE_LEVEL=95

		DF_METHOD=RESIDUAL COVB=ROBUST PCONVERGE=0.000001(ABSOLUTE) SCORING=0 SINGULAR=0.000000000001 /EMMEANS_OPTIONS SCALE=ORIGINAL PADJUST=LSD.
Resources	Processor Time	00:00:01.58
	Elapsed Time	00:00:01.42

Warnings

glmm: One or more records are not used in the analysis because they have one or more fields with invalid or missing values.
glmm: Valid values for events (target) and trials variables are non-negative and positive integers respectively, and the number of trials cannot be less than the number of events.
glmm: The estimated covariance matrix of the random effects (the G matrix) is not positive definite. The procedure continues despite this warning. Subsequent results produced are based on the last iteration. Validity of the model fit is uncertain.
glmm: The final Hessian matrix is not positive definite although all convergence criteria are satisfied. The procedure continues despite this warning. Subsequent results produced are based on the last iteration. Validity of the model fit is uncertain.

Case Processing Summary

	N	Percent
Included	1100	98.8%
Excluded	13	1.2%
Total	1113	100.0%

Model Summary

Target		Kessler16 > 1 = 1
Probability Distribution		Binomial
Link Function		Logit
Information Criterion	Akaike Corrected	5013.818
	Bayesian	5018.808

Information criteria are based on the -2 log likelihood (5011.814) and are used to compare models. Models with smaller information criterion values fit better.

Data Structure[a]

	Subjects	Target
	company_code	Kessler16 > 1 = 1
Data for First Subject	2	1.00
	2	1.00
	2	.00

	2	.00
	2	1.00
	2	1.00
	2	.00
	2	.00
	2	.00
	2	.00
Total Number of Levels	58	

Only the first 10 records are displayed.

a. Target: Kessler16 > 1 = 1

Classification
Overall Percent Correct = 67.6%[a]

Observed		Predicted 1.00	Predicted .00
1.00	Count	635	84
	% within Observed	88.3%	11.7%
.00	Count	272	109
	% within Observed	71.4%	28.6%

a. Target: Kessler16 > 1 = 1

Fixed Effects[a]

Source	F	df1	df2	Sig.
Corrected Model	15.015	9	1090	.000

physical_health	9.094	1	1090	.003
kess_t15	73.191	1	1090	.000
choice_freq	.336	1	1090	.562
bullycwc2	5.416	1	1090	.020
bullym2	.539	1	1090	.463
fac2bull	3.612	1	1090	.058
time_pressure_freq	1.643	1	1090	.200
duties_freq	6.163	1	1090	.013
FAC1_1	5.494	1	1090	.019

Probability distribution: Binomial

Link function: Logit

a. Target: Kessler16 > 1 = 1

Fixed Effects

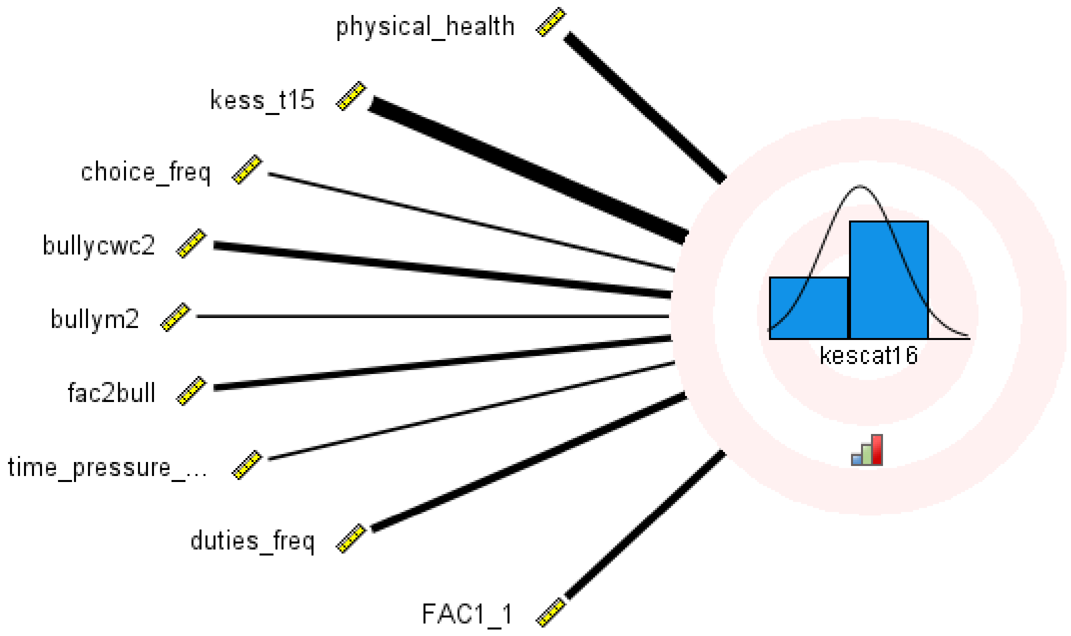

Fixed Coefficients[a]

Model Term	Coefficient	Std. Error	t	Sig.	95% Confidence Interval		Exp(Coefficient)	95% Confidence Interval for Exp(Coefficient)	
					Lower	Upper		Lower	Upper
Intercept	-2.012	.7817	-2.573	.010	-3.546	-.478	.134	.029	.620
physical_health	.257	.0852	3.016	.003	.090	.424	1.293	1.094	1.528
kess_t15	1.307	.1528	8.555	.000	1.007	1.607	3.694	2.738	4.986
choice_freq	.031	.0533	.580	.562	-.074	.135	1.031	.929	1.145
bullycwc2	.203	.0874	2.327	.020	.032	.375	1.225	1.032	1.455
bullym2	.360	.4896	.734	.463	-.601	1.320	1.433	.548	3.745
fac2bull	-.192	.1009	-1.900	.058	-.390	.006	.825	.677	1.006
time_pressure_freq	-.073	.0570	-1.282	.200	-.185	.039	.930	.831	1.040
duties_freq	-.132	.0531	-2.483	.013	-.236	-.028	.876	.790	.973
FAC1_1	-.184	.0785	-2.344	.019	-.338	-.030	.832	.713	.970

Probability distribution: Binomial

Link function: Logit

a. Target: Kessler16 > 1 = 1

Fixed Coefficients

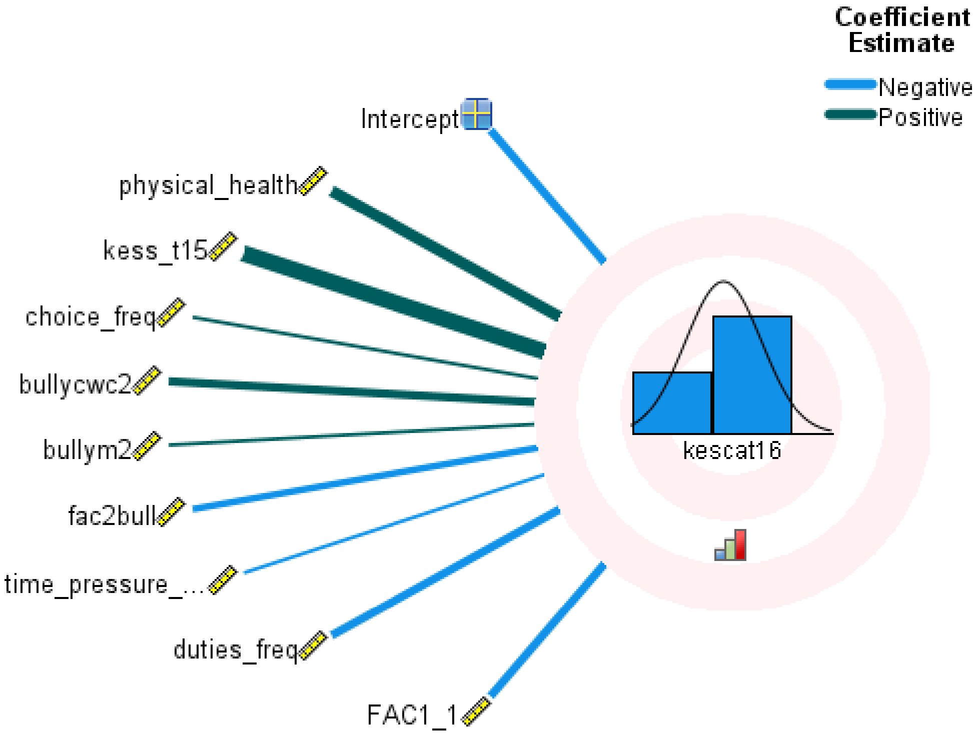

Random Effect Covariances

Random Effect Block 1

Random Effect Block	Intercept
Intercept	.000

Covariance Structure: Variance components

Subject Specification: companyId15

Covariance Parameters

Covariance Parameters Summary

Covariance Parameters	Residual Effect	0
	Random Effects	1
Design Matrix Columns	Fixed Effects	10
	Random Effects	1[a]
Common Subjects		58

Common subjects are based on the subject specifications for the residual and random effects and are used to chunk the data for better performance.

a. This is the number of columns per common subject.

Residual Effect

Residual Effect	Estimate	Std. Error	Z	Sig.	95% Confidence Interval	
					Lower	Upper
Variance	1.000	.	.	.	.	.

Covariance Structure: Scaled Identity

Subject Specification: (None)

Random Effect

Random Effect Covariance	Estimate	Std. Error	Z	Sig.	95% Confidence Interval	
					Lower	Upper
Var(Intercept)	.000[a]	.	.	.	.	.

Covariance Structure: Variance components

Subject Specification: companyId15

a. This parameter is redundant.

Random Effects Parameters

Empirical Best Linear Unbiased Predictions[a]

company_code	Parameter	Prediction	Std. Error	df	Sig.	95% Confidence Interval	
						Lower Bound	Upper Bound

2	Intercept	-1.737E-12	3.440E-13	1090	5.195E-7	-2.412E-12	-1.062E-12
4	Intercept	-3.412E-13	2.087E-13	1090	.102	-7.508E-13	6.830E-14
6	Intercept	-3.770E-13	3.175E-13	1090	.235	-1.000E-12	2.460E-13
22	Intercept	-1.494E-12	2.844E-13	1090	1.813E-7	-2.052E-12	-9.355E-13
24	Intercept	-1.024E-12	6.798E-13	1090	.132	-2.358E-12	3.095E-13
30	Intercept	7.716E-13	3.016E-13	1090	.011	1.799E-13	1.363E-12
36	Intercept	-1.041E-12	1.825E-13	1090	1.487E-8	-1.400E-12	-6.833E-13
40	Intercept	1.299E-12	1.366E-13	1090	.000	1.031E-12	1.567E-12
50	Intercept	1.043E-12	1.004E-13	1090	.000	8.461E-13	1.240E-12
61	Intercept	-8.415E-13	1.892E-13	1090	9.608E-6	-1.213E-12	-4.702E-13
65	Intercept	-1.372E-12	1.222E-13	1090	.000	-1.612E-12	-1.132E-12
68	Intercept	1.676E-12	1.707E-13	1090	.000	1.340E-12	2.010E-12
69	Intercept	1.490E-14	1.432E-13	1090	.917	-2.661E-13	2.959E-13
71	Intercept	3.284E-13	1.252E-13	1090	.009	8.270E-14	5.742E-13
72	Intercept	-1.263E-12	1.953E-13	1090	1.491E-10	-1.646E-12	-8.800E-13
74	Intercept	-3.781E-13	7.980E-14	1090	2.415E-6	-5.347E-13	-2.216E-13
75	Intercept	-1.324E-12	1.353E-13	1090	.000	-1.589E-12	-1.058E-12
80	Intercept	-3.010E-13	8.840E-14	1090	.001	-4.745E-13	-1.276E-13
81	Intercept	1.396E-13	2.535E-13	1090	.582	-3.579E-13	6.370E-13
83	Intercept	1.586E-12	2.046E-13	1090	2.040E-14	1.185E-12	1.988E-12
84	Intercept	5.109E-13	1.214E-13	1090	2.792E-5	2.727E-13	7.492E-13
92	Intercept	-2.102E-13	3.937E-13	1090	.594	-9.826E-13	5.623E-13
93	Intercept	1.928E-13	1.170E-13	1090	.099	-3.660E-14	4.223E-13
98	Intercept	-1.508E-12	2.265E-13	1090	4.421E-11	-1.952E-12	-1.064E-12
100	Intercept	1.402E-13	2.137E-12	1090	.948	-4.054E-12	4.334E-12
106	Intercept	-9.160E-14	4.460E-14	1090	.040	-1.791E-13	-4.100E-15

107	Intercept	1.285E-12	1.005E-13	1090	.000	1.088E-12	1.483E-12
108	Intercept	-2.581E-12	5.349E-13	1090	1.596E-6	-3.631E-12	-1.532E-12
110	Intercept	4.707E-13	2.230E-12	1090	.833	-3.905E-12	4.847E-12
114	Intercept	-2.104E-12	1.405E-13	1090	.000	-2.379E-12	-1.828E-12
116	Intercept	-3.619E-13	1.032E-13	1090	.000	-5.644E-13	-1.594E-13
122	Intercept	1.112E-12	3.064E-13	1090	.000	5.110E-13	1.714E-12
125	Intercept	1.693E-12	7.514E-13	1090	.024	2.191E-13	3.168E-12
142	Intercept	5.094E-13	4.414E-13	1090	.249	-3.567E-13	1.375E-12
144	Intercept	1.674E-12	2.374E-13	1090	3.199E-12	1.208E-12	2.139E-12
145	Intercept	6.337E-13	9.670E-14	1090	8.773E-11	4.439E-13	8.236E-13
152	Intercept	1.020E-12	2.199E-13	1090	3.949E-6	5.885E-13	1.452E-12
154	Intercept	-3.154E-13	2.835E-13	1090	.266	-8.718E-13	2.409E-13
156	Intercept	-1.247E-13	2.615E-13	1090	.634	-6.379E-13	3.885E-13
162	Intercept	-7.142E-13	2.167E-13	1090	.001	-1.139E-12	-2.891E-13
165	Intercept	1.275E-13	7.140E-14	1090	.074	-1.260E-14	2.676E-13
166	Intercept	2.779E-13	3.760E-14	1090	2.887E-13	2.042E-13	3.517E-13
169	Intercept	-8.336E-13	1.851E-13	1090	7.359E-6	-1.197E-12	-4.705E-13
170	Intercept	-5.362E-13	6.300E-14	1090	.000	-6.598E-13	-4.127E-13
173	Intercept	8.630E-13	1.226E-13	1090	3.404E-12	6.225E-13	1.104E-12
174	Intercept	-1.021E-12	1.494E-13	1090	1.366E-11	-1.314E-12	-7.279E-13
178	Intercept	-1.947E-12	2.111E-13	1090	.000	-2.361E-12	-1.533E-12
181	Intercept	-7.843E-13	2.763E-13	1090	.005	-1.327E-12	-2.421E-13
182	Intercept	3.413E-13	1.088E-13	1090	.002	1.278E-13	5.548E-13
192	Intercept	1.660E-13	1.003E-13	1090	.098	-3.070E-14	3.627E-13
202	Intercept	6.482E-13	4.137E-13	1090	.117	-1.636E-13	1.460E-12
204	Intercept	1.868E-12	1.235E-12	1090	.131	-5.553E-13	4.292E-12

205	Intercept	5.002E-13	8.850E-14	1090	2.018E-8	3.265E-13	6.738E-13
206	Intercept	1.028E-12	1.868E-13	1090	4.669E-8	6.612E-13	1.394E-12
209	Intercept	-1.180E-12	3.725E-13	1090	.002	-1.911E-12	-4.496E-13
217	Intercept	2.097E-12	1.081E-12	1090	.053	-2.510E-14	4.219E-12
226	Intercept	8.882E-13	1.804E-13	1090	9.872E-7	5.341E-13	1.242E-12
228	Intercept	9.015E-13	1.774E-13	1090	4.374E-7	5.535E-13	1.249E-12

a. Target: Kessler16 > 1 = 1

```
GENLINMIXED
  /DATA_STRUCTURE SUBJECTS=companyId15
  /FIELDS TARGET=kescat16 TRIALS=NONE OFFSET=NONE
  /TARGET_OPTIONS DISTRIBUTION=BINOMIAL LINK=LOGIT
  /FIXED  EFFECTS=physical_health kess_t15 choice_freq bullying_freq timecwc2 timem2 fac2time
  duties_freq
    FAC1_1 USE_INTERCEPT=TRUE
  /RANDOM USE_INTERCEPT=TRUE SUBJECTS=companyId15 COVARIANCE_TYPE=VARIANCE_COMPONENTS SOLUTION=TRUE
  /BUILD_OPTIONS TARGET_CATEGORY_ORDER=DESCENDING INPUTS_CATEGORY_ORDER=DESCENDING
    MAX_ITERATIONS=100 CONFIDENCE_LEVEL=95 DF_METHOD=RESIDUAL COVB=ROBUST PCONVERGE=0.000001(ABSOLUTE)
    SCORING=0 SINGULAR=0.000000000001
  /EMMEANS_OPTIONS SCALE=ORIGINAL PADJUST=LSD.
```

Generalized Linear Mixed Models

Notes

Output Created	16-AUG-2021 11:07:42

Comments		
Input	Data	C:\Users\jwb12ppu\Documents\Kevin\MPlus backup\Multicomponent health and wellbeing v2\Multicomponent for Mplus cat.sav
	Active Dataset	DataSet2
	Filter	kescat15 = 0 (FILTER)
	Weight	<none>
	Split File	<none>
	N of Rows in Working Data File	1113

Syntax	GENLINMIXED<br>/DATA_STRUCTURE<br>SUBJECTS=companyId15<br>/FIELDS TARGET=kescat16<br>TRIALS=NONE OFFSET=NONE<br>/TARGET_OPTIONS<br>DISTRIBUTION=BINOMIAL<br>LINK=LOGIT<br>/FIXED<br>EFFECTS=physical_health<br>kess_t15 choice_freq bullying_freq<br>timecwc2 timem2 fac2time<br>duties_freq<br>FAC1_1<br>USE_INTERCEPT=TRUE<br>/RANDOM<br>USE_INTERCEPT=TRUE<br>SUBJECTS=companyId15<br>COVARIANCE_TYPE=VARIANCE<br>_COMPONENTS<br>SOLUTION=TRUE<br>/BUILD_OPTIONS<br>TARGET_CATEGORY_ORDER=<br>DESCENDING<br>INPUTS_CATEGORY_ORDER=D<br>ESCENDING<br>MAX_ITERATIONS=100<br>CONFIDENCE_LEVEL=95

		DF_METHOD=RESIDUAL COVB=ROBUST PCONVERGE=0.000001(ABSOLUTE) SCORING=0 SINGULAR=0.000000000001 /EMMEANS_OPTIONS SCALE=ORIGINAL PADJUST=LSD.
Resources	Processor Time	00:00:01.33
	Elapsed Time	00:00:01.38

Warnings

glmm: One or more records are not used in the analysis because they have one or more fields with invalid or missing values.
glmm: Valid values for events (target) and trials variables are non-negative and positive integers respectively, and the number of trials cannot be less than the number of events.
glmm: The estimated covariance matrix of the random effects (the G matrix) is not positive definite. The procedure continues despite this warning. Subsequent results produced are based on the last iteration. Validity of the model fit is uncertain.
glmm: The final Hessian matrix is not positive definite although all convergence criteria are satisfied. The procedure continues despite this warning. Subsequent results produced are based on the last iteration. Validity of the model fit is uncertain.

Case Processing Summary

	N	Percent
Included	1100	98.8%
Excluded	13	1.2%
Total	1113	100.0%

Model Summary

Target		Kessler16 > 1 = 1
Probability Distribution		Binomial
Link Function		Logit
Information Criterion	Akaike Corrected	5003.543
	Bayesian	5008.533

Information criteria are based on the -2 log likelihood (5001.539) and are used to compare models. Models with smaller information criterion values fit better.

Data Structure[a]

	Subjects	Target
	company_code	Kessler16 > 1 = 1
Data for First Subject	2	1.00
	2	1.00
	2	.00

	2	.00
	2	1.00
	2	1.00
	2	.00
	2	.00
	2	.00
	2	.00
Total Number of Levels	58	

Only the first 10 records are displayed.

a. Target: Kessler16 > 1 = 1

Classification

Overall Percent Correct = 68.5%[a]

Observed		Predicted 1.00	Predicted .00
1.00	Count	641	78
	% within Observed	89.2%	10.8%
.00	Count	268	113
	% within Observed	70.3%	29.7%

a. Target: Kessler16 > 1 = 1

Fixed Effects[a]

Source	F	df1	df2	Sig.
Corrected Model	16.549	9	1090	.000

physical_health	9.784	1	1090	.002
kess_t15	71.460	1	1090	.000
choice_freq	.146	1	1090	.702
bullying_freq	2.891	1	1090	.089
timecwc2	1.080	1	1090	.299
timem2	1.247	1	1090	.264
fac2time	.013	1	1090	.910
duties_freq	6.774	1	1090	.009
FAC1_1	4.319	1	1090	.038

Probability distribution: Binomial

Link function: Logit

a. Target: Kessler16 > 1 = 1

Fixed Effects

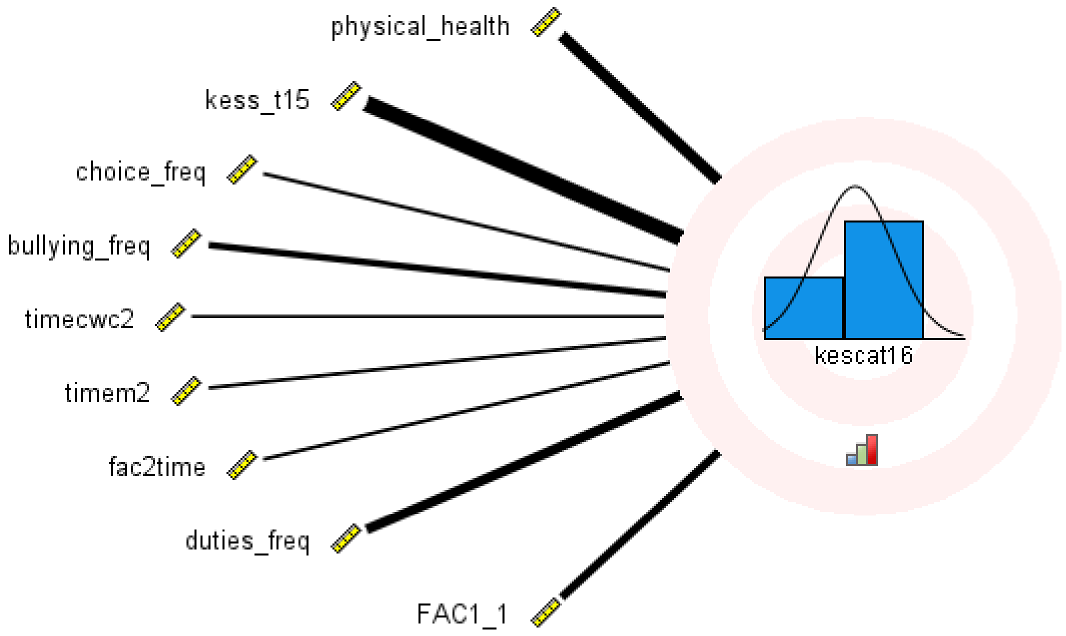

Fixed Coefficients[a]

Model Term	Coefficient	Std. Error	t	Sig.	95% Confidence Interval		Exp(Coefficient)	95% Confidence Interval for Exp(Coefficient)	
					Lower	Upper		Lower	Upper
Intercept	-1.188	.7612	-1.561	.119	-2.682	.306	.305	.068	1.357
physical_health	.272	.0870	3.128	.002	.101	.443	1.313	1.107	1.557
kess_t15	1.303	.1541	8.453	.000	1.000	1.605	3.679	2.719	4.978
choice_freq	.021	.0547	.382	.702	-.086	.128	1.021	.917	1.137
bullying_freq	.183	.1075	1.700	.089	-.028	.394	1.200	.972	1.482
timecwc2	-.058	.0557	-1.039	.299	-.167	.051	.944	.846	1.053
timem2	-.261	.2335	-1.117	.264	-.719	.197	.770	.487	1.218
fac2time	.007	.0632	.113	.910	-.117	.131	1.007	.890	1.140
duties_freq	-.139	.0536	-2.603	.009	-.245	-.034	.870	.783	.966
FAC1_1	-.153	.0738	-2.078	.038	-.298	-.009	.858	.742	.991

Probability distribution: Binomial

Link function: Logit

a. Target: Kessler16 > 1 = 1

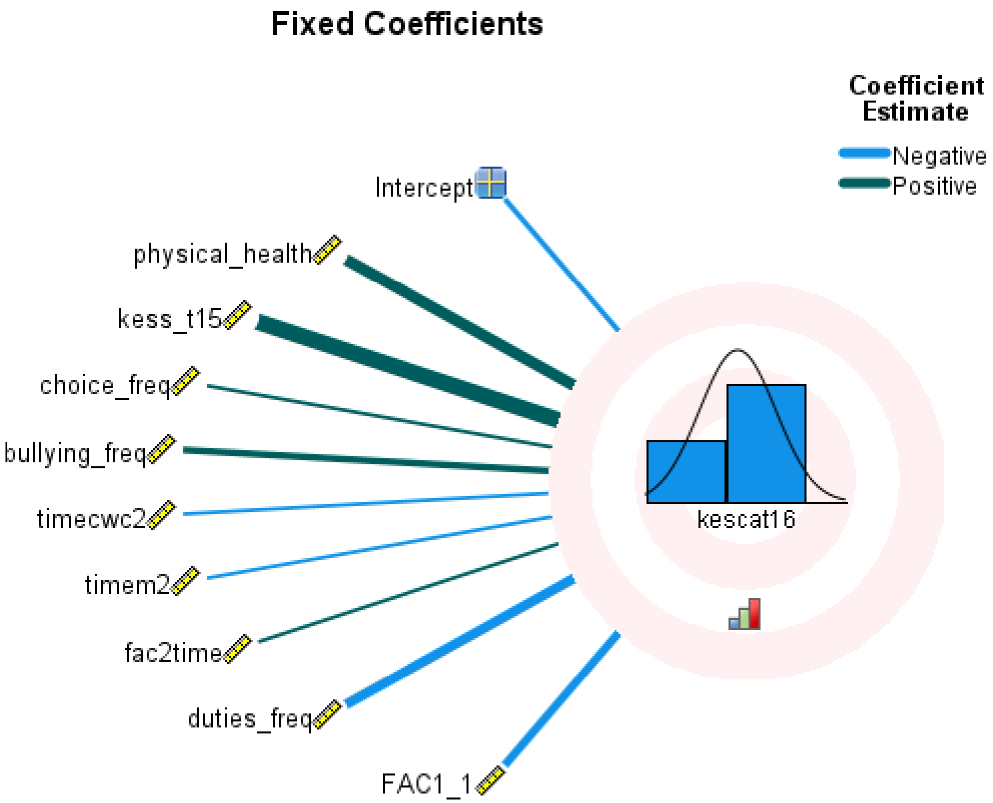

Random Effect Covariances

Random Effect Block 1

Random Effect Block	Intercept
Intercept	.000

Covariance Structure: Variance components

Subject Specification: companyId15

Covariance Parameters

Covariance Parameters Summary

Covariance Parameters	Residual Effect	0
	Random Effects	1
Design Matrix Columns	Fixed Effects	10
	Random Effects	1[a]
Common Subjects		58

Common subjects are based on the subject specifications for the residual and random effects and are used to chunk the data for better performance.

a. This is the number of columns per common subject.

Residual Effect

Residual Effect	Estimate	Std. Error	Z	Sig.	95% Confidence Interval	
					Lower	Upper
Variance	1.000	.	.	.	.	.

Covariance Structure: Scaled Identity

Subject Specification: (None)

Random Effect

Random Effect Covariance	Estimate	Std. Error	Z	Sig.	95% Confidence Interval	
					Lower	Upper
Var(Intercept)	.000[a]	.	.	.	.	.

Covariance Structure: Variance components

Subject Specification: companyId15

a. This parameter is redundant.

Random Effects Parameters

Empirical Best Linear Unbiased Predictions[a]

company_code	Parameter	Prediction	Std. Error	df	Sig.	95% Confidence Interval	
						Lower Bound	Upper Bound

2	Intercept	-1.899E-13	3.440E-14	1090	4.343E-8	-2.575E-13	-1.224E-13
4	Intercept	-6.230E-14	2.480E-14	1090	.012	-1.110E-13	-1.370E-14
6	Intercept	-4.180E-14	3.690E-14	1090	.257	-1.141E-13	3.060E-14
22	Intercept	-1.516E-13	1.560E-14	1090	.000	-1.821E-13	-1.210E-13
24	Intercept	-1.210E-13	5.210E-14	1090	.020	-2.231E-13	-1.880E-14
30	Intercept	5.820E-14	2.640E-14	1090	.028	6.400E-15	1.099E-13
36	Intercept	-1.340E-13	2.080E-14	1090	1.638E-10	-1.747E-13	-9.330E-14
40	Intercept	1.433E-13	1.090E-14	1090	.000	1.219E-13	1.648E-13
50	Intercept	9.950E-14	1.690E-14	1090	5.283E-9	6.630E-14	1.327E-13
61	Intercept	-9.770E-14	1.760E-14	1090	3.443E-8	-1.322E-13	-6.320E-14
65	Intercept	-1.575E-13	1.360E-14	1090	.000	-1.841E-13	-1.308E-13
68	Intercept	1.702E-13	1.800E-14	1090	.000	1.349E-13	2.055E-13
69	Intercept	-2.040E-14	2.690E-14	1090	.448	-7.310E-14	3.230E-14
71	Intercept	4.070E-14	1.250E-14	1090	.001	1.610E-14	6.530E-14
72	Intercept	-1.384E-13	1.810E-14	1090	4.840E-14	-1.739E-13	-1.028E-13
74	Intercept	-4.380E-14	4.000E-15	1090	.000	-5.170E-14	-3.600E-14
75	Intercept	-1.618E-13	2.320E-14	1090	5.604E-12	-2.073E-13	-1.162E-13
80	Intercept	-2.890E-14	9.800E-15	1090	.003	-4.810E-14	-9.700E-15
81	Intercept	-9.700E-15	2.720E-14	1090	.721	-6.310E-14	4.370E-14
83	Intercept	1.553E-13	2.850E-14	1090	5.952E-8	9.950E-14	2.112E-13
84	Intercept	4.810E-14	1.110E-14	1090	1.466E-5	2.640E-14	6.980E-14
92	Intercept	-5.490E-14	5.850E-14	1090	.349	-1.696E-13	5.990E-14
93	Intercept	1.790E-14	9.500E-15	1090	.061	-8.000E-16	3.660E-14
98	Intercept	-1.765E-13	2.730E-14	1090	1.405E-10	-2.300E-13	-1.231E-13
100	Intercept	6.990E-14	2.011E-13	1090	.728	-3.246E-13	4.644E-13
106	Intercept	-1.000E-14	4.800E-15	1090	.038	-1.940E-14	-5.000E-16

107	Intercept	1.353E-13	1.420E-14	1090	.000	1.074E-13	1.632E-13
108	Intercept	-2.867E-13	4.590E-14	1090	6.181E-10	-3.769E-13	-1.966E-13
110	Intercept	6.820E-14	2.529E-13	1090	.788	-4.281E-13	5.645E-13
114	Intercept	-2.378E-13	1.550E-14	1090	.000	-2.683E-13	-2.074E-13
116	Intercept	-2.250E-14	2.290E-14	1090	.327	-6.750E-14	2.250E-14
122	Intercept	1.742E-13	6.490E-14	1090	.007	4.680E-14	3.015E-13
125	Intercept	2.330E-13	1.039E-13	1090	.025	2.920E-14	4.368E-13
142	Intercept	8.110E-14	5.390E-14	1090	.133	-2.470E-14	1.868E-13
144	Intercept	1.729E-13	2.210E-14	1090	1.220E-14	1.295E-13	2.163E-13
145	Intercept	7.780E-14	1.570E-14	1090	8.318E-7	4.700E-14	1.085E-13
152	Intercept	1.014E-13	2.340E-14	1090	1.620E-5	5.550E-14	1.474E-13
154	Intercept	-5.270E-14	2.230E-14	1090	.018	-9.650E-14	-9.000E-15
156	Intercept	-3.500E-14	3.520E-14	1090	.319	-1.040E-13	3.390E-14
162	Intercept	-7.730E-14	2.200E-14	1090	.000	-1.205E-13	-3.410E-14
165	Intercept	1.380E-14	7.500E-15	1090	.065	-9.000E-16	2.840E-14
166	Intercept	3.000E-14	2.100E-15	1090	.000	2.600E-14	3.410E-14
169	Intercept	-9.120E-14	1.770E-14	1090	2.849E-7	-1.259E-13	-5.660E-14
170	Intercept	-6.330E-14	9.700E-15	1090	1.092E-10	-8.240E-14	-4.430E-14
173	Intercept	8.770E-14	1.830E-14	1090	1.925E-6	5.180E-14	1.237E-13
174	Intercept	-1.043E-13	1.410E-14	1090	2.678E-13	-1.320E-13	-7.670E-14
178	Intercept	-2.250E-13	2.810E-14	1090	2.900E-15	-2.802E-13	-1.699E-13
181	Intercept	-6.870E-14	1.980E-14	1090	.001	-1.075E-13	-2.980E-14
182	Intercept	2.710E-14	1.360E-14	1090	.047	4.000E-16	5.390E-14
192	Intercept	1.700E-14	1.080E-14	1090	.117	-4.200E-15	3.830E-14
202	Intercept	4.820E-14	3.100E-14	1090	.120	-1.250E-14	1.090E-13
204	Intercept	3.130E-13	9.620E-14	1090	.001	1.243E-13	5.017E-13

205	Intercept	5.600E-14	4.600E-15	1090	.000	4.700E-14	6.510E-14
206	Intercept	9.570E-14	2.900E-14	1090	.001	3.880E-14	1.526E-13
209	Intercept	-1.311E-13	4.260E-14	1090	.002	-2.148E-13	-4.750E-14
217	Intercept	2.354E-13	1.004E-13	1090	.019	3.840E-14	4.324E-13
226	Intercept	1.017E-13	1.800E-14	1090	1.879E-8	6.650E-14	1.369E-13
228	Intercept	1.231E-13	2.310E-14	1090	1.167E-7	7.780E-14	1.684E-13

a. Target: Kessler16 > 1 = 1

```
GENLINMIXED
  /DATA_STRUCTURE SUBJECTS=companyId15
  /FIELDS TARGET=kescat16 TRIALS=NONE OFFSET=NONE
  /TARGET_OPTIONS DISTRIBUTION=BINOMIAL LINK=LOGIT
  /FIXED  EFFECTS=physical_health kess_t15 choice_freq bullying_freq time_pressure_freq dutiescwc2 dutiesm2 fac2duti
    FAC1_1 USE_INTERCEPT=TRUE
  /RANDOM USE_INTERCEPT=TRUE SUBJECTS=companyId15 COVARIANCE_TYPE=VARIANCE_COMPONENTS SOLUTION=TRUE
  /BUILD_OPTIONS TARGET_CATEGORY_ORDER=DESCENDING INPUTS_CATEGORY_ORDER=DESCENDING
    MAX_ITERATIONS=100 CONFIDENCE_LEVEL=95 DF_METHOD=RESIDUAL COVB=ROBUST PCONVERGE=0.000001(ABSOLUTE)
    SCORING=0 SINGULAR=0.000000000001
  /EMMEANS_OPTIONS SCALE=ORIGINAL PADJUST=LSD.
```

Generalized Linear Mixed Models

Notes

Output Created	16-AUG-2021 11:07:44
Comments	

Input	Data	C:\Users\jwb12ppu\Documents\Kevin\MPlus backup\Multicomponent health and wellbeing v2\Multicomponent for Mplus cat.sav
	Active Dataset	DataSet2
	Filter	kescat15 = 0 (FILTER)
	Weight	<none>
	Split File	<none>
	N of Rows in Working Data File	1113

Syntax	GENLINMIXED<br>/DATA_STRUCTURE<br>SUBJECTS=companyId15<br>/FIELDS TARGET=kescat16<br>TRIALS=NONE OFFSET=NONE<br>/TARGET_OPTIONS<br>DISTRIBUTION=BINOMIAL<br>LINK=LOGIT<br>/FIXED<br>EFFECTS=physical_health<br>kess_t15 choice_freq bullying_freq<br>time_pressure_freq dutiescwc2<br>dutiesm2 fac2duti<br>FAC1_1<br>USE_INTERCEPT=TRUE<br>/RANDOM<br>USE_INTERCEPT=TRUE<br>SUBJECTS=companyId15<br>COVARIANCE_TYPE=VARIANCE<br>_COMPONENTS<br>SOLUTION=TRUE<br>/BUILD_OPTIONS<br>TARGET_CATEGORY_ORDER=<br>DESCENDING<br>INPUTS_CATEGORY_ORDER=D<br>ESCENDING<br>MAX_ITERATIONS=100<br>CONFIDENCE_LEVEL=95

		DF_METHOD=RESIDUAL COVB=ROBUST PCONVERGE=0.000001(ABSOLUTE) SCORING=0 SINGULAR=0.000000000001 /EMMEANS_OPTIONS SCALE=ORIGINAL PADJUST=LSD.
Resources	Processor Time	00:00:01.06
	Elapsed Time	00:00:01.10

Warnings

glmm: One or more records are not used in the analysis because they have one or more fields with invalid or missing values.
glmm: Valid values for events (target) and trials variables are non-negative and positive integers respectively, and the number of trials cannot be less than the number of events.
glmm: The final Hessian matrix is not positive definite although all convergence criteria are satisfied. The procedure continues despite this warning. Subsequent results produced are based on the last iteration. Validity of the model fit is uncertain.

Case Processing Summary

	N	Percent
Included	1100	98.8%
Excluded	13	1.2%
Total	1113	100.0%

Model Summary

Target		Kessler16 > 1 = 1
Probability Distribution		Binomial
Link Function		Logit
Information Criterion	Akaike Corrected	4992.058
	Bayesian	4997.048

Information criteria are based on the -2 log likelihood (4990.054) and are used to compare models. Models with smaller information criterion values fit better.

Data Structure[a]

	Subjects company_code	Target Kessler16 > 1 = 1
Data for First Subject	2	1.00
	2	1.00
	2	.00
	2	.00
	2	1.00
	2	1.00
	2	.00

	2	.00
	2	.00
	2	.00
Total Number of Levels	58	

Only the first 10 records are displayed.

a. Target: Kessler16 > 1 = 1

Classification
Overall Percent Correct = 67.8%[a]

Observed		Predicted 1.00	.00
1.00	Count	636	83
	% within Observed	88.5%	11.5%
.00	Count	271	110
	% within Observed	71.1%	28.9%

a. Target: Kessler16 > 1 = 1

Fixed Effects[a]

Source	F	df1	df2	Sig.
Corrected Model	16.818	9	1090	.000
physical_health	8.797	1	1090	.003
kess_t15	72.114	1	1090	.000
choice_freq	.298	1	1090	.585
bullying_freq	2.797	1	1090	.095

time_pressure_freq	1.693	1	1090	.193
dutiescwc2	4.705	1	1090	.030
dutiesm2	1.752	1	1090	.186
fac2duti	.150	1	1090	.699
FAC1_1	5.143	1	1090	.024

Probability distribution: Binomial

Link function: Logit

a. Target: Kessler16 > 1 = 1

Fixed Effects

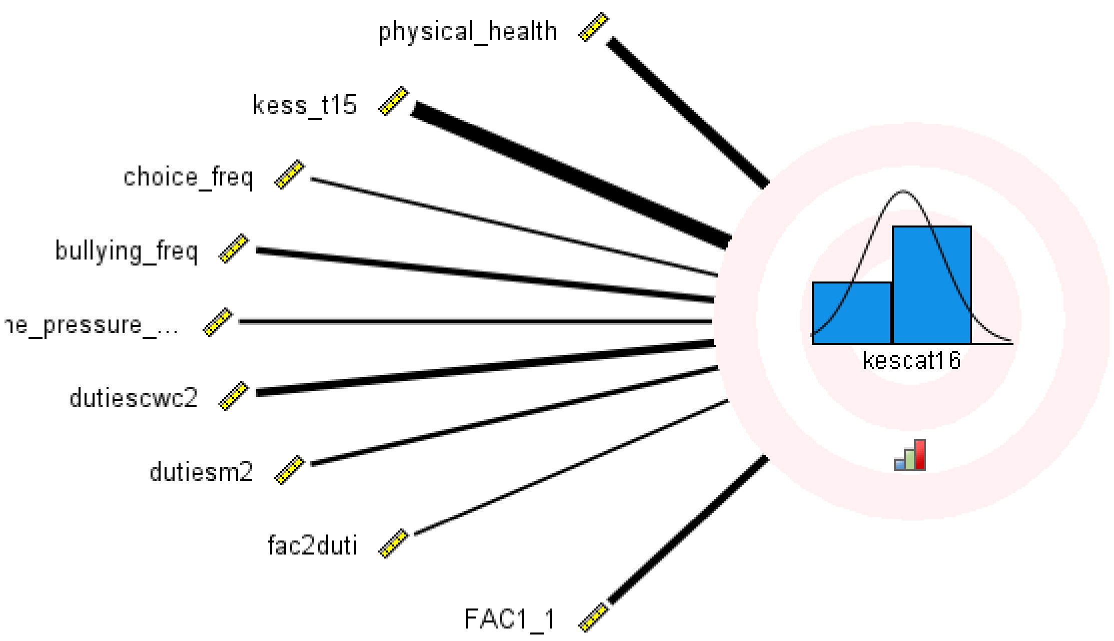

Fixed Coefficients[a]

Model Term	Coefficient	Std. Error	t	Sig.	95% Confidence Interval		Exp(Coefficient)	95% Confidence Interval for Exp(Coefficient)	
					Lower	Upper		Lower	Upper
Intercept	-1.100	.9709	-1.133	.258	-3.005	.805	.333	.050	2.238
physical_health	.257	.0868	2.966	.003	.087	.428	1.294	1.091	1.534
kess_t15	1.309	.1541	8.492	.000	1.006	1.611	3.702	2.736	5.009
choice_freq	.029	.0537	.546	.585	-.076	.135	1.030	.927	1.144
bullying_freq	.178	.1063	1.672	.095	-.031	.386	1.195	.970	1.472
time_pressure_freq	-.074	.0572	-1.301	.193	-.187	.038	.928	.830	1.039
dutiescwc2	-.117	.0540	-2.169	.030	-.223	-.011	.890	.800	.989
dutiesm2	-.310	.2341	-1.323	.186	-.769	.150	.734	.463	1.161
fac2duti	-.020	.0522	-.387	.699	-.123	.082	.980	.885	1.086
FAC1_1	-.176	.0775	-2.268	.024	-.328	-.024	.839	.720	.977

Probability distribution: Binomial

Link function: Logit

a. Target: Kessler16 > 1 = 1

Fixed Coefficients

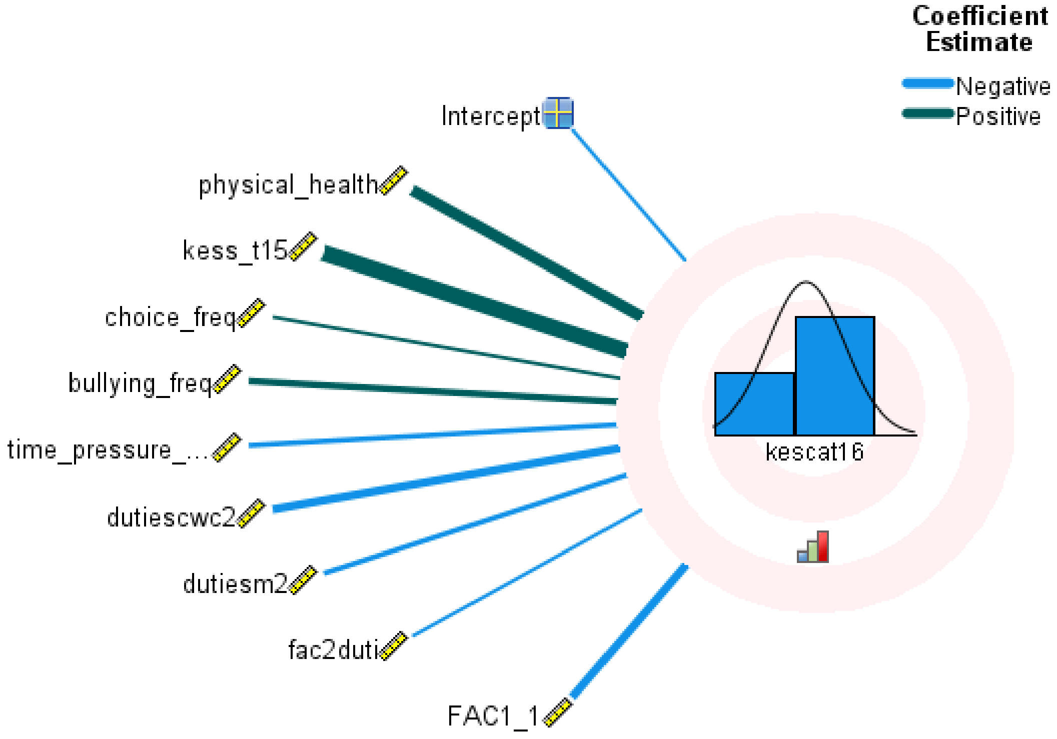

Random Effect Covariances

Random Effect Block 1

Random Effect Block	Intercept
Intercept	.000

Covariance Structure: Variance components

Subject Specification: companyId15

Covariance Parameters

Covariance Parameters Summary

Covariance Parameters	Residual Effect	0
	Random Effects	1
Design Matrix Columns	Fixed Effects	10
	Random Effects	1[a]
Common Subjects		58

Common subjects are based on the subject specifications for the residual and random effects and are used to chunk the data for better performance.

a. This is the number of columns per common subject.

Residual Effect

Residual Effect	Estimate	Std. Error	Z	Sig.	95% Confidence Interval	
					Lower	Upper
Variance	1.000	.	.	.	.	.

Covariance Structure: Scaled Identity

Subject Specification: (None)

Random Effect

Random Effect Covariance	Estimate	Std. Error	Z	Sig.	95% Confidence Interval	
					Lower	Upper
Var(Intercept)	1.184E-12[a]	.	.	.	.	.

Covariance Structure: Variance components

Subject Specification: companyId15

a. This parameter is redundant.

Random Effects Parameters

Empirical Best Linear Unbiased Predictions[a]

company_code	Parameter	Prediction	Std. Error	df	Sig.	95% Confidence Interval	
						Lower Bound	Upper Bound

2	Intercept	-2.880E-12	1.088E-6	1090	1.000	-2.135E-6	2.135E-6
4	Intercept	-5.703E-13	1.088E-6	1090	1.000	-2.135E-6	2.135E-6
6	Intercept	-8.793E-13	1.088E-6	1090	1.000	-2.135E-6	2.135E-6
22	Intercept	-2.648E-12	1.088E-6	1090	1.000	-2.135E-6	2.135E-6
24	Intercept	-2.381E-12	1.088E-6	1090	1.000	-2.135E-6	2.135E-6
30	Intercept	1.356E-12	1.088E-6	1090	1.000	-2.135E-6	2.135E-6
36	Intercept	-1.812E-12	1.088E-6	1090	1.000	-2.135E-6	2.135E-6
40	Intercept	2.340E-12	1.088E-6	1090	1.000	-2.135E-6	2.135E-6
50	Intercept	1.636E-12	1.088E-6	1090	1.000	-2.135E-6	2.135E-6
61	Intercept	-1.153E-12	1.088E-6	1090	1.000	-2.135E-6	2.135E-6
65	Intercept	-2.292E-12	1.088E-6	1090	1.000	-2.135E-6	2.135E-6
68	Intercept	2.762E-12	1.088E-6	1090	1.000	-2.135E-6	2.135E-6
69	Intercept	-7.500E-15	1.088E-6	1090	1.000	-2.135E-6	2.135E-6
71	Intercept	4.097E-13	1.088E-6	1090	1.000	-2.135E-6	2.135E-6
72	Intercept	-2.244E-12	1.088E-6	1090	1.000	-2.135E-6	2.135E-6
74	Intercept	-7.154E-13	1.088E-6	1090	1.000	-2.135E-6	2.135E-6
75	Intercept	-2.001E-12	1.088E-6	1090	1.000	-2.135E-6	2.135E-6
80	Intercept	-2.701E-13	1.088E-6	1090	1.000	-2.135E-6	2.135E-6
81	Intercept	3.576E-13	1.088E-6	1090	1.000	-2.135E-6	2.135E-6
83	Intercept	2.918E-12	1.088E-6	1090	1.000	-2.135E-6	2.135E-6
84	Intercept	1.052E-12	1.088E-6	1090	1.000	-2.135E-6	2.135E-6
92	Intercept	1.412E-13	1.088E-6	1090	1.000	-2.135E-6	2.135E-6
93	Intercept	2.451E-13	1.088E-6	1090	1.000	-2.135E-6	2.135E-6
98	Intercept	-2.413E-12	1.088E-6	1090	1.000	-2.135E-6	2.135E-6
100	Intercept	3.398E-13	1.088E-6	1090	1.000	-2.135E-6	2.135E-6
106	Intercept	-2.471E-13	1.088E-6	1090	1.000	-2.135E-6	2.135E-6

107	Intercept	2.167E-12	1.088E-6	1090	1.000	-2.135E-6	2.135E-6
108	Intercept	-4.548E-12	1.088E-6	1090	1.000	-2.135E-6	2.135E-6
110	Intercept	-3.678E-13	1.088E-6	1090	1.000	-2.135E-6	2.135E-6
114	Intercept	-3.530E-12	1.088E-6	1090	1.000	-2.135E-6	2.135E-6
116	Intercept	-6.376E-13	1.088E-6	1090	1.000	-2.135E-6	2.135E-6
122	Intercept	1.810E-12	1.088E-6	1090	1.000	-2.135E-6	2.135E-6
125	Intercept	2.800E-12	1.088E-6	1090	1.000	-2.135E-6	2.135E-6
142	Intercept	6.808E-13	1.088E-6	1090	1.000	-2.135E-6	2.135E-6
144	Intercept	2.892E-12	1.088E-6	1090	1.000	-2.135E-6	2.135E-6
145	Intercept	8.365E-13	1.088E-6	1090	1.000	-2.135E-6	2.135E-6
152	Intercept	1.432E-12	1.088E-6	1090	1.000	-2.135E-6	2.135E-6
154	Intercept	-4.282E-13	1.088E-6	1090	1.000	-2.135E-6	2.135E-6
156	Intercept	-2.423E-13	1.088E-6	1090	1.000	-2.135E-6	2.135E-6
162	Intercept	-1.057E-12	1.088E-6	1090	1.000	-2.135E-6	2.135E-6
165	Intercept	-1.800E-15	1.088E-6	1090	1.000	-2.135E-6	2.135E-6
166	Intercept	4.711E-13	1.088E-6	1090	1.000	-2.135E-6	2.135E-6
169	Intercept	-1.567E-12	1.088E-6	1090	1.000	-2.135E-6	2.135E-6
170	Intercept	-8.303E-13	1.088E-6	1090	1.000	-2.135E-6	2.135E-6
173	Intercept	1.557E-12	1.088E-6	1090	1.000	-2.135E-6	2.135E-6
174	Intercept	-1.538E-12	1.088E-6	1090	1.000	-2.135E-6	2.135E-6
178	Intercept	-3.282E-12	1.088E-6	1090	1.000	-2.135E-6	2.135E-6
181	Intercept	-1.300E-12	1.088E-6	1090	1.000	-2.135E-6	2.135E-6
182	Intercept	6.810E-13	1.088E-6	1090	1.000	-2.135E-6	2.135E-6
192	Intercept	2.637E-13	1.088E-6	1090	1.000	-2.135E-6	2.135E-6
202	Intercept	9.151E-13	1.088E-6	1090	1.000	-2.135E-6	2.135E-6
204	Intercept	4.054E-12	1.088E-6	1090	1.000	-2.135E-6	2.135E-6

205	Intercept	9.451E-13	1.088E-6	1090	1.000	-2.135E-6	2.135E-6
206	Intercept	1.771E-12	1.088E-6	1090	1.000	-2.135E-6	2.135E-6
209	Intercept	-1.912E-12	1.088E-6	1090	1.000	-2.135E-6	2.135E-6
217	Intercept	3.734E-12	1.088E-6	1090	1.000	-2.135E-6	2.135E-6
226	Intercept	1.581E-12	1.088E-6	1090	1.000	-2.135E-6	2.135E-6
228	Intercept	1.607E-12	1.088E-6	1090	1.000	-2.135E-6	2.135E-6

a. Target: Kessler16 > 1 = 1

```
FILTER OFF.
USE ALL.
EXECUTE.
```

```
USE ALL.
COMPUTE filter_$=(phycat15 = 0).
VARIABLE LABELS filter_$ 'kescat15 = 0 (FILTER)'.
VALUE LABELS filter_$ 0 'Not Selected' 1 'Selected'.
FORMATS filter_$ (f1.0).
FILTER BY filter_$.
EXECUTE.

correlations /variables = phycat16 phycat15 fac1_1.
```

Correlations

Notes

Output Created		16-AUG-2021 11:07:45
Comments		
Input	Data	C:\Users\jwb12ppu\Documents\Kevin\MPlus backup\Multicomponent health and wellbeing v2\Multicomponent for Mplus cat.sav
	Active Dataset	DataSet2
	Filter	kescat15 = 0 (FILTER)
	Weight	<none>
	Split File	<none>
	N of Rows in Working Data File	5546
Missing Value Handling	Definition of Missing	User-defined missing values are treated as missing.
	Cases Used	Statistics for each pair of variables are based on all the cases with valid data for that pair.
Syntax		correlations /variables = phycat16 phycat15 fac1_1.
Resources	Processor Time	00:00:00.06
	Elapsed Time	00:00:00.02

Correlations

		Phy16 > 2 = 1	phy15 > 2 = 1	REGR factor score 1 for analysis 1
Phy16 > 2 = 1	Pearson Correlation	1	.[a]	-.049
	Sig. (2-tailed)		.	.000
	N	5546	5546	5546
phy15 > 2 = 1	Pearson Correlation	.[a]	.[a]	.[a]
	Sig. (2-tailed)	.		.
	N	5546	5546	5546
REGR factor score 1 for analysis 1	Pearson Correlation	-.049	.[a]	1
	Sig. (2-tailed)	.000	.	
	N	5546	5546	5546

a. Cannot be computed because at least one of the variables is constant.

```
*Generalized Linear Mixed Models.
GENLINMIXED
  /DATA_STRUCTURE SUBJECTS=companyId15
  /FIELDS TARGET=phycat16 TRIALS=NONE OFFSET=NONE
  /TARGET_OPTIONS DISTRIBUTION=BINOMIAL LINK=LOGIT
  /FIXED  EFFECTS=physical_health kess_t15 choice_freq bullying_freq time_pressure_freq duties_freq
    FAC1_1 USE_INTERCEPT=TRUE
  /RANDOM USE_INTERCEPT=TRUE SUBJECTS=companyId15 COVARIANCE_TYPE=VARIANCE_COMPONENTS SOLUTION=TRUE
  /BUILD_OPTIONS TARGET_CATEGORY_ORDER=DESCENDING INPUTS_CATEGORY_ORDER=DESCENDING
    MAX_ITERATIONS=100 CONFIDENCE_LEVEL=95 DF_METHOD=RESIDUAL COVB=ROBUST PCONVERGE=0.000001(ABSOLUTE)
    SCORING=0 SINGULAR=0.000000000001
  /EMMEANS_OPTIONS SCALE=ORIGINAL PADJUST=LSD.
```

Generalized Linear Mixed Models

Notes

Output Created		16-AUG-2021 11:07:45
Comments		
Input	Data	C:\Users\jwb12ppu\Documents\Kevin\MPlus backup\Multicomponent health and wellbeing v2\Multicomponent for Mplus cat.sav
	Active Dataset	DataSet2
	Filter	kescat15 = 0 (FILTER)
	Weight	<none>
	Split File	<none>
	N of Rows in Working Data File	5546

Syntax	GENLINMIXED<br>/DATA_STRUCTURE SUBJECTS=companyId15<br>/FIELDS TARGET=phycat16 TRIALS=NONE OFFSET=NONE<br>/TARGET_OPTIONS DISTRIBUTION=BINOMIAL LINK=LOGIT<br>/FIXED EFFECTS=physical_health kess_t15 choice_freq bullying_freq time_pressure_freq duties_freq<br>FAC1_1 USE_INTERCEPT=TRUE<br>/RANDOM USE_INTERCEPT=TRUE SUBJECTS=companyId15 COVARIANCE_TYPE=VARIANCE_COMPONENTS SOLUTION=TRUE<br>/BUILD_OPTIONS TARGET_CATEGORY_ORDER=DESCENDING INPUTS_CATEGORY_ORDER=DESCENDING<br>MAX_ITERATIONS=100 CONFIDENCE_LEVEL=95 DF_METHOD=RESIDUAL

		COVB=ROBUST PCONVERGE=0.000001(ABSOLUTE) SCORING=0 SINGULAR=0.000000000001 /EMMEANS_OPTIONS SCALE=ORIGINAL PADJUST=LSD.
Resources	Processor Time	00:00:02.13
	Elapsed Time	00:00:02.12

Warnings

glmm: One or more records are not used in the analysis because they have one or more fields with invalid or missing values.
glmm: Valid values for events (target) and trials variables are non-negative and positive integers respectively, and the number of trials cannot be less than the number of events.

Case Processing Summary

	N	Percent
Included	5069	91.4%
Excluded	477	8.6%
Total	5546	100.0%

Model Summary

Target		Phy16 > 2 = 1
Probability Distribution		Binomial
Link Function		Logit
Information Criterion	Akaike Corrected	28024.204
	Bayesian	28030.732

Information criteria are based on the -2 log likelihood (28022.203) and are used to compare models. Models with smaller information criterion values fit better.

Data Structure[a]

	Subjects company_code	Target Phy16 > 2 = 1
Data for First Subject	2	.00
	2	.00
	2	.00
	2	.00
	2	.00
	2	.00
	2	.00
	2	.00
	2	.00
	2	.00
Total Number of Levels	58	

Only the first 10 records are displayed.

a. Target: Phy16 > 2 = 1

Classification

Overall Percent Correct = 88.8%[a]

Observed		Predicted 1.00	.00
1.00	Count	1	570
	% within Observed	0.2%	99.8%
.00	Count	0	4498
	% within Observed	0.0%	100.0%

a. Target: Phy16 > 2 = 1

Fixed Effects[a]

Source	F	df1	df2	Sig.
Corrected Model	56.271	7	5061	.000
physical_health	169.866	1	5061	.000
kess_t15	45.318	1	5061	.000
choice_freq	8.049	1	5061	.005
bullying_freq	.269	1	5061	.604
time_pressure_freq	3.046	1	5061	.081
duties_freq	1.578	1	5061	.209
FAC1_1	22.255	1	5061	.000

Probability distribution: Binomial

Link function: Logit

a. Target: Phy16 > 2 = 1

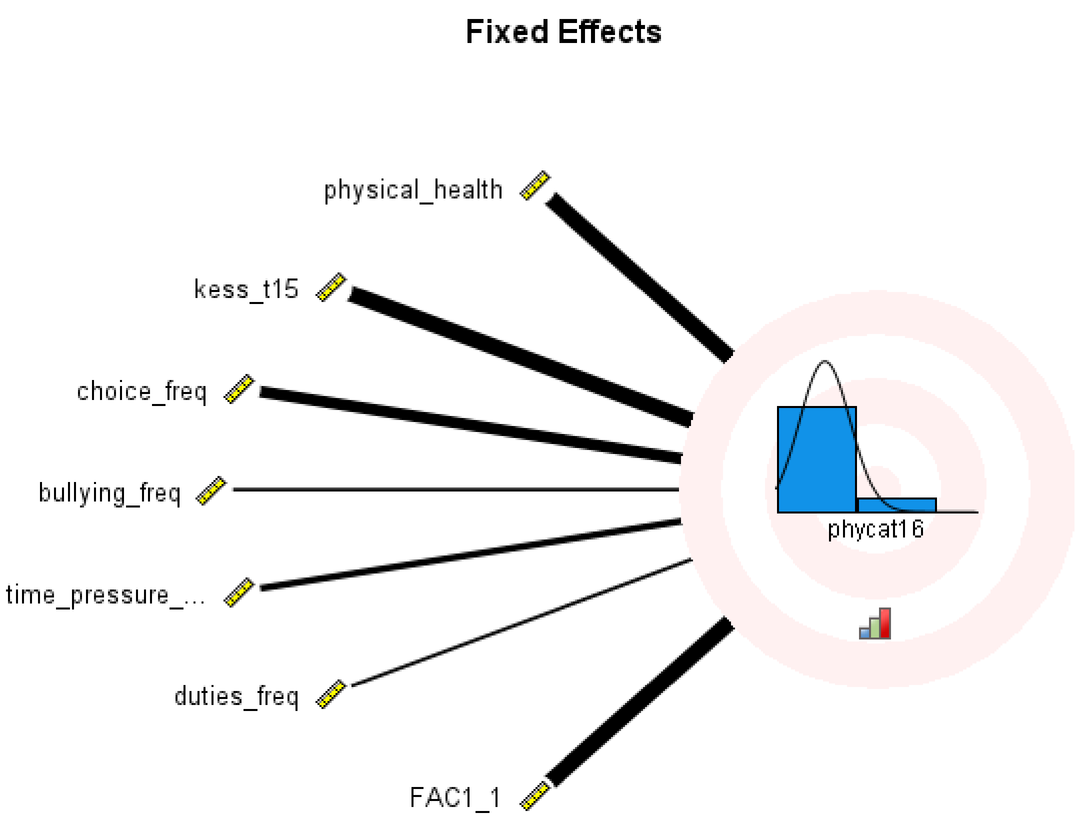

Fixed Coefficients[a]

Model Term	Coefficient	Std. Error	t	Sig.	95% Confidence Interval		Exp(Coefficient)	95% Confidence Interval for Exp(Coefficient)	
					Lower	Upper		Lower	Upper
Intercept	-5.224	.4147	-12.598	.000	-6.037	-4.411	.005	.002	.012
physical_health	2.000	.1534	13.033	.000	1.699	2.300	7.387	5.468	9.979
kess_t15	.421	.0626	6.732	.000	.299	.544	1.524	1.348	1.723
choice_freq	-.121	.0427	-2.837	.005	-.205	-.037	.886	.815	.963
bullying_freq	.064	.1231	.519	.604	-.177	.305	1.066	.837	1.357
time_pressure_freq	-.063	.0360	-1.745	.081	-.133	.008	.939	.875	1.008
duties_freq	-.056	.0444	-1.256	.209	-.143	.031	.946	.867	1.032
FAC1_1	-.185	.0391	-4.717	.000	-.261	-.108	.831	.770	.898

Probability distribution: Binomial

Link function: Logit

a. Target: Phy16 > 2 = 1

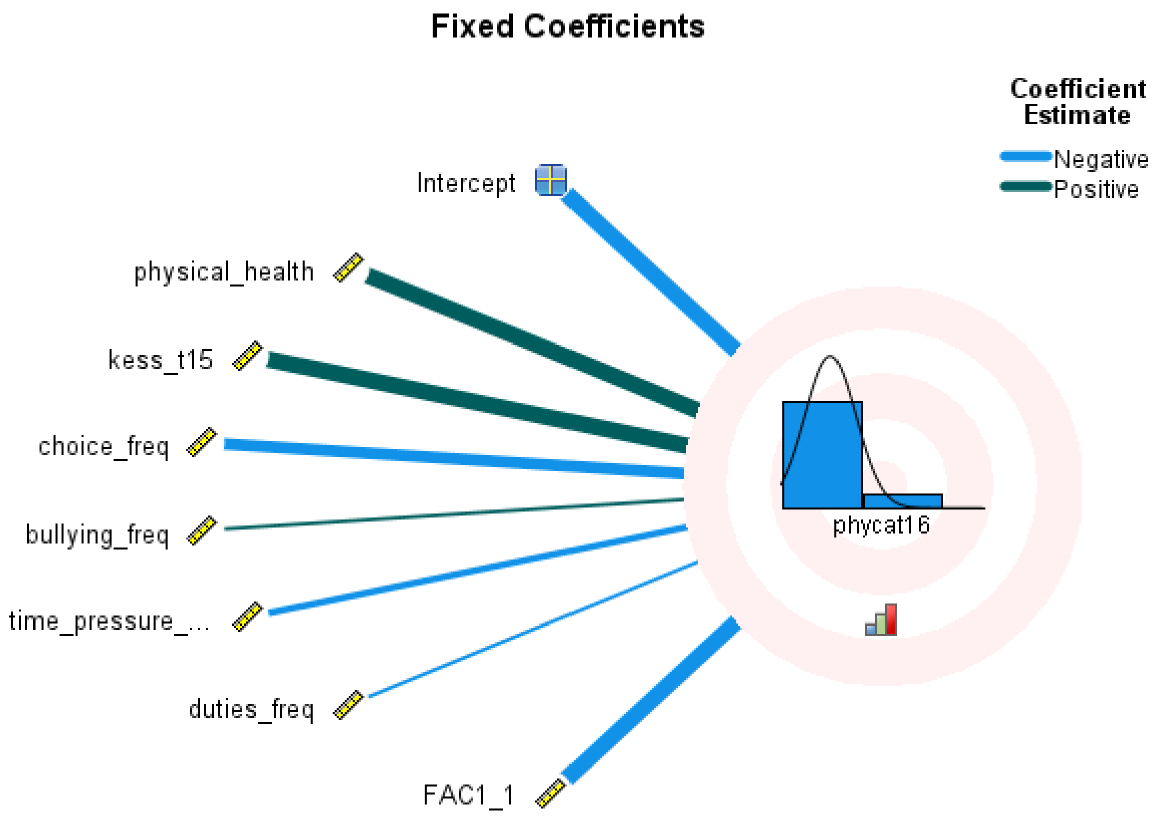

Random Effect Covariances

Random Effect Block 1

Random Effect Block	Intercept
Intercept	.035

Covariance Structure: Variance components

Subject Specification: companyId15

Covariance Parameters

Covariance Parameters Summary

Covariance Parameters	Residual Effect	0
	Random Effects	1
Design Matrix Columns	Fixed Effects	8
	Random Effects	1[a]
Common Subjects		58

Common subjects are based on the subject specifications for the residual and random effects and are used to chunk the data for better performance.

a. This is the number of columns per common subject.

Residual Effect

Residual Effect	Estimate	Std. Error	Z	Sig.	95% Confidence Interval	
					Lower	Upper
Variance	1.000	.	.	.	.	.

Covariance Structure: Scaled Identity

Subject Specification: (None)

Random Effect

Random Effect Covariance	Estimate	Std. Error	Z	Sig.	95% Confidence Interval	
					Lower	Upper
Var(Intercept)	.035	.034	1.028	.304	.005	.239

Covariance Structure: Variance components

Subject Specification: companyId15

Random Effects Parameters

Empirical Best Linear Unbiased Predictions[a]

company_code	Parameter	Prediction	Std. Error	df	Sig.	95% Confidence Interval	
						Lower Bound	Upper Bound
2	Intercept	.021	.170	5061	.904	-.313	.355

4	Intercept	-.051	.179	5061	.774	-.402	.299
6	Intercept	-.035	.173	5061	.838	-.375	.304
22	Intercept	-.025	.186	5061	.894	-.390	.341
24	Intercept	-.013	.152	5061	.930	-.310	.284
30	Intercept	.005	.178	5061	.976	-.344	.355
36	Intercept	-.033	.176	5061	.849	-.378	.311
40	Intercept	.088	.175	5061	.616	-.255	.431
50	Intercept	-.010	.180	5061	.956	-.362	.342
61	Intercept	.034	.175	5061	.847	-.310	.378
65	Intercept	.013	.184	5061	.945	-.347	.373
68	Intercept	-.025	.174	5061	.887	-.366	.317
69	Intercept	-.112	.173	5061	.516	-.452	.227
71	Intercept	.049	.180	5061	.785	-.303	.401
72	Intercept	.049	.176	5061	.779	-.295	.393
74	Intercept	.049	.180	5061	.784	-.303	.402
75	Intercept	.000	.176	5061	.999	-.346	.346
80	Intercept	-.021	.184	5061	.911	-.381	.340
81	Intercept	.068	.174	5061	.697	-.274	.409
83	Intercept	-.054	.175	5061	.760	-.397	.290
84	Intercept	-.068	.178	5061	.701	-.417	.280
92	Intercept	.011	.170	5061	.948	-.322	.344
93	Intercept	-.001	.171	5061	.997	-.336	.334
98	Intercept	-.119	.168	5061	.477	-.448	.210
100	Intercept	-.120	.107	5061	.263	-.331	.090
106	Intercept	-.026	.181	5061	.884	-.381	.328
107	Intercept	-.016	.177	5061	.927	-.363	.331

108	Intercept	-.096	.181	5061	.594	-.451	.258
110	Intercept	.070	.118	5061	.554	-.162	.302
114	Intercept	.107	.176	5061	.544	-.239	.452
116	Intercept	.009	.173	5061	.957	-.329	.348
122	Intercept	.091	.156	5061	.559	-.215	.398
125	Intercept	.047	.147	5061	.747	-.240	.335
142	Intercept	-.161	.163	5061	.324	-.481	.159
144	Intercept	-.014	.172	5061	.933	-.352	.323
145	Intercept	-.019	.173	5061	.912	-.357	.319
152	Intercept	-.101	.171	5061	.555	-.436	.234
154	Intercept	-.050	.173	5061	.774	-.388	.289
156	Intercept	.005	.162	5061	.975	-.312	.322
162	Intercept	-.026	.178	5061	.882	-.376	.323
165	Intercept	-.008	.180	5061	.966	-.360	.344
166	Intercept	-.056	.184	5061	.760	-.417	.304
169	Intercept	-.009	.176	5061	.959	-.354	.336
170	Intercept	.027	.178	5061	.878	-.321	.376
173	Intercept	-.068	.176	5061	.699	-.414	.277
174	Intercept	.026	.175	5061	.882	-.317	.369
178	Intercept	.349	.163	5061	.032	.030	.668
181	Intercept	-.025	.179	5061	.889	-.375	.326
182	Intercept	-.064	.181	5061	.722	-.419	.290
192	Intercept	-.034	.179	5061	.851	-.385	.317
202	Intercept	-.001	.168	5061	.994	-.330	.327
204	Intercept	.111	.146	5061	.447	-.175	.397
205	Intercept	.034	.183	5061	.854	-.325	.392

206	Intercept	.009	.174	5061	.958	-.333	.351
209	Intercept	.037	.151	5061	.809	-.260	.333
217	Intercept	.118	.134	5061	.378	-.144	.380
226	Intercept	.143	.173	5061	.407	-.195	.482
228	Intercept	-.106	.178	5061	.551	-.456	.243

a. Target: Phy16 > 2 = 1

```
GENLINMIXED
  /DATA_STRUCTURE SUBJECTS=companyId15
  /FIELDS TARGET=phycat16 TRIALS=NONE OFFSET=NONE
  /TARGET_OPTIONS DISTRIBUTION=BINOMIAL LINK=LOGIT
  /FIXED  EFFECTS=physical_health kess_t15 choice_freq bullying_freq time_pressure_freq duties_freq
    FAC1_1 facsq USE_INTERCEPT=TRUE
  /RANDOM USE_INTERCEPT=TRUE SUBJECTS=companyId15 COVARIANCE_TYPE=VARIANCE_COMPONENTS SOLUTION=TRUE
  /BUILD_OPTIONS TARGET_CATEGORY_ORDER=DESCENDING INPUTS_CATEGORY_ORDER=DESCENDING
    MAX_ITERATIONS=100 CONFIDENCE_LEVEL=95 DF_METHOD=RESIDUAL COVB=ROBUST PCONVERGE=0.000001(ABSOLUTE)
    SCORING=0 SINGULAR=0.000000000001
  /EMMEANS_OPTIONS SCALE=ORIGINAL PADJUST=LSD.
```

Generalized Linear Mixed Models

Notes

Output Created	16-AUG-2021 11:07:47

Comments		
Input	Data	C:\Users\jwb12ppu\Documents\Kevin\MPlus backup\Multicomponent health and wellbeing v2\Multicomponent for Mplus cat.sav
	Active Dataset	DataSet2
	Filter	kescat15 = 0 (FILTER)
	Weight	<none>
	Split File	<none>
	N of Rows in Working Data File	5546

Syntax	GENLINMIXED<br>/DATA_STRUCTURE SUBJECTS=companyId15<br>/FIELDS TARGET=phycat16 TRIALS=NONE OFFSET=NONE<br>/TARGET_OPTIONS DISTRIBUTION=BINOMIAL LINK=LOGIT<br>/FIXED EFFECTS=physical_health kess_t15 choice_freq bullying_freq time_pressure_freq duties_freq<br>FAC1_1 facsq USE_INTERCEPT=TRUE<br>/RANDOM USE_INTERCEPT=TRUE SUBJECTS=companyId15 COVARIANCE_TYPE=VARIANCE_COMPONENTS SOLUTION=TRUE<br>/BUILD_OPTIONS TARGET_CATEGORY_ORDER=DESCENDING INPUTS_CATEGORY_ORDER=DESCENDING<br>MAX_ITERATIONS=100 CONFIDENCE_LEVEL=95 DF_METHOD=RESIDUAL

		COVB=ROBUST PCONVERGE=0.000001(ABSOLUTE) SCORING=0 SINGULAR=0.000000000001 /EMMEANS_OPTIONS SCALE=ORIGINAL PADJUST=LSD.
Resources	Processor Time	00:00:01.89
	Elapsed Time	00:00:01.95

Warnings

glmm: One or more records are not used in the analysis because they have one or more fields with invalid or missing values.
glmm: Valid values for events (target) and trials variables are non-negative and positive integers respectively, and the number of trials cannot be less than the number of events.

Case Processing Summary

	N	Percent
Included	5069	91.4%
Excluded	477	8.6%
Total	5546	100.0%

Model Summary

Target		Phy16 > 2 = 1
Probability Distribution		Binomial
Link Function		Logit
Information Criterion	Akaike Corrected	28065.210
	Bayesian	28071.739

Information criteria are based on the -2 log likelihood (28063.210) and are used to compare models. Models with smaller information criterion values fit better.

Data Structure[a]

	Subjects company_code	Target Phy16 > 2 = 1
Data for First Subject	2	.00
	2	.00
	2	.00
	2	.00
	2	.00
	2	.00
	2	.00
	2	.00
	2	.00
	2	.00
Total Number of Levels	58	

Only the first 10 records are displayed.

a. Target: Phy16 > 2 = 1

Classification

Overall Percent Correct = 88.8%[a]

Observed		Predicted	
		1.00	.00
1.00	Count	1	570
	% within Observed	0.2%	99.8%
.00	Count	0	4498
	% within Observed	0.0%	100.0%

a. Target: Phy16 > 2 = 1

Fixed Effects[a]

Source	F	df1	df2	Sig.
Corrected Model	47.886	8	5060	.000
physical_health	169.366	1	5060	.000
kess_t15	44.783	1	5060	.000
choice_freq	7.424	1	5060	.006
bullying_freq	.250	1	5060	.617
time_pressure_freq	3.236	1	5060	.072
duties_freq	1.565	1	5060	.211
FAC1_1	22.475	1	5060	.000
facsq	2.410	1	5060	.121

Probability distribution: Binomial

Link function: Logit

a. Target: Phy16 > 2 = 1

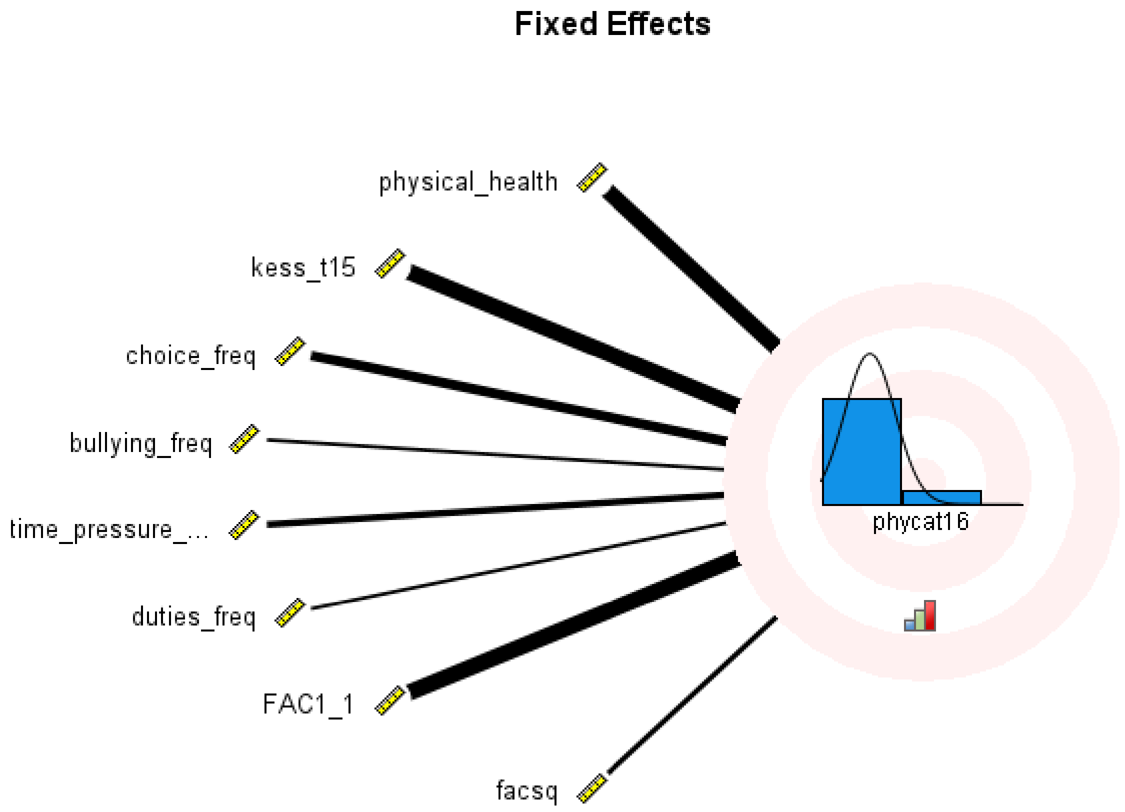

Fixed Coefficients[a]

Model Term	Coefficient	Std. Error	t	Sig.	95% Confidence Interval		Exp(Coefficient)	95% Confidence Interval for Exp(Coefficient)	
					Lower	Upper		Lower	Upper
Intercept	-5.149	.4071	-12.647	.000	-5.947	-4.351	.006	.003	.013
physical_health	1.996	.1533	13.014	.000	1.695	2.296	7.356	5.446	9.936
kess_t15	.421	.0628	6.692	.000	.297	.544	1.523	1.346	1.722
choice_freq	-.118	.0433	-2.725	.006	-.203	-.033	.889	.817	.967
bullying_freq	.061	.1227	.500	.617	-.179	.302	1.063	.836	1.352
time_pressure_freq	-.064	.0357	-1.799	.072	-.134	.006	.938	.874	1.006
duties_freq	-.056	.0444	-1.251	.211	-.143	.032	.946	.867	1.032
FAC1_1	-.204	.0431	-4.741	.000	-.289	-.120	.815	.749	.887
facsq	-.067	.0430	-1.552	.121	-.151	.018	.935	.860	1.018

Probability distribution: Binomial

Link function: Logit

a. Target: Phy16 > 2 = 1

Fixed Coefficients

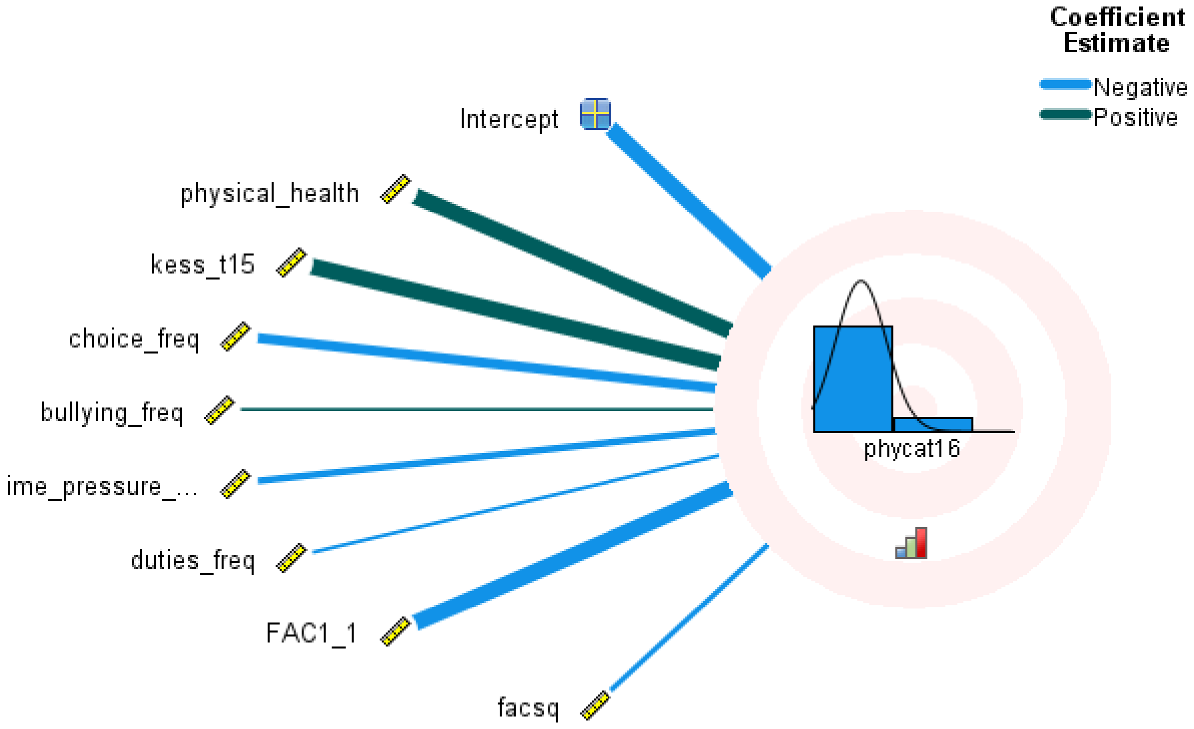

Random Effect Covariances

Random Effect Block 1

Random Effect Block	Intercept
Intercept	.035

Covariance Structure: Variance components

Subject Specification: companyId15

Covariance Parameters

Covariance Parameters Summary

Covariance Parameters	Residual Effect	0
	Random Effects	1
Design Matrix Columns	Fixed Effects	9
	Random Effects	1[a]
Common Subjects		58

Common subjects are based on the subject specifications for the residual and random effects and are used to chunk the data for better performance.

a. This is the number of columns per common subject.

Residual Effect

Residual Effect	Estimate	Std. Error	Z	Sig.	95% Confidence Interval	
					Lower	Upper
Variance	1.000	.	.	.	.	.

Covariance Structure: Scaled Identity

Subject Specification: (None)

Random Effect

Random Effect Covariance	Estimate	Std. Error	Z	Sig.	95% Confidence Interval	
					Lower	Upper
Var(Intercept)	.035	.032	1.088	.277	.006	.209

Covariance Structure: Variance components

Subject Specification: companyId15

Random Effects Parameters

Empirical Best Linear Unbiased Predictions[a]

company_code	Parameter	Prediction	Std. Error	df	Sig.	95% Confidence Interval	
						Lower Bound	Upper Bound
2	Intercept	.031	.169	5060	.855	-.301	.363

4	Intercept	-.021	.180	5060	.908	-.373	.331
6	Intercept	-.006	.173	5060	.972	-.345	.333
22	Intercept	-.025	.184	5060	.894	-.385	.336
24	Intercept	-.021	.151	5060	.888	-.316	.274
30	Intercept	.016	.177	5060	.929	-.331	.363
36	Intercept	-.041	.174	5060	.812	-.382	.299
40	Intercept	.078	.173	5060	.652	-.261	.417
50	Intercept	-.013	.177	5060	.941	-.361	.334
61	Intercept	.038	.174	5060	.827	-.303	.378
65	Intercept	.026	.182	5060	.888	-.332	.384
68	Intercept	-.030	.172	5060	.863	-.367	.308
69	Intercept	-.121	.171	5060	.478	-.456	.214
71	Intercept	.055	.178	5060	.757	-.294	.404
72	Intercept	.068	.174	5060	.698	-.274	.410
74	Intercept	.051	.178	5060	.775	-.298	.399
75	Intercept	.006	.175	5060	.972	-.336	.349
80	Intercept	-.022	.182	5060	.905	-.378	.334
81	Intercept	.057	.172	5060	.742	-.281	.394
83	Intercept	-.046	.173	5060	.791	-.386	.294
84	Intercept	-.073	.176	5060	.679	-.417	.272
92	Intercept	.021	.168	5060	.898	-.308	.351
93	Intercept	.022	.170	5060	.897	-.311	.355
98	Intercept	-.108	.166	5060	.514	-.434	.218
100	Intercept	-.164	.115	5060	.156	-.390	.062
106	Intercept	-.031	.179	5060	.861	-.381	.319
107	Intercept	-.024	.175	5060	.892	-.366	.319

108	Intercept	-.073	.180	5060	.687	-.426	.281
110	Intercept	.077	.116	5060	.510	-.151	.305
114	Intercept	.096	.174	5060	.580	-.245	.438
116	Intercept	.000	.171	5060	.998	-.335	.334
122	Intercept	.069	.156	5060	.659	-.237	.374
125	Intercept	.059	.145	5060	.687	-.226	.343
142	Intercept	-.174	.162	5060	.283	-.491	.144
144	Intercept	.006	.172	5060	.972	-.331	.343
145	Intercept	-.030	.170	5060	.859	-.364	.304
152	Intercept	-.082	.170	5060	.631	-.414	.251
154	Intercept	-.049	.171	5060	.772	-.384	.285
156	Intercept	.018	.160	5060	.909	-.296	.333
162	Intercept	-.019	.177	5060	.917	-.365	.328
165	Intercept	.003	.178	5060	.988	-.347	.352
166	Intercept	-.058	.181	5060	.749	-.414	.298
169	Intercept	.021	.176	5060	.905	-.324	.366
170	Intercept	.020	.176	5060	.907	-.324	.365
173	Intercept	-.072	.174	5060	.679	-.413	.269
174	Intercept	.016	.173	5060	.925	-.323	.355
178	Intercept	.324	.162	5060	.045	.007	.641
181	Intercept	-.029	.177	5060	.870	-.375	.317
182	Intercept	-.066	.178	5060	.710	-.416	.284
192	Intercept	-.023	.177	5060	.899	-.370	.325
202	Intercept	-.005	.166	5060	.976	-.330	.320
204	Intercept	.080	.146	5060	.584	-.207	.367
205	Intercept	.030	.180	5060	.870	-.324	.383

206	Intercept	-.001	.172	5060	.997	-.338	.337
209	Intercept	.011	.151	5060	.939	-.284	.307
217	Intercept	.103	.133	5060	.437	-.157	.364
226	Intercept	.135	.171	5060	.429	-.199	.470
228	Intercept	-.111	.176	5060	.527	-.456	.234

a. Target: Phy16 > 2 = 1

```
AGGREGATE
  /OUTFILE=* MODE=ADDVARIABLES OVERWRITE = YES
  /BREAK=companyId15
  /choicem3=MEAN(choice_freq)
  /bullym3=MEAN(bullying_freq)
  /timem3=MEAN(time_pressure_freq)
  /dutiesm3=MEAN(duties_freq).

COMPUTE choicecwc3 = choice_freq - choicem3.
COMPUTE bullycwc3 = bullying_freq - bullym3.
COMPUTE timecwc3 = time_pressure_freq - timem3.
COMPUTE dutiescwc3 = duties_freq - dutiesm3.

COMPUTE fac3choi = fac1_1 * choicecwc3.
COMPUTE fac3bull = fac1_1 * bullycwc3.
COMPUTE fac3time = fac1_1 *  timecwc3.
COMPUTE fac3duti = fac1_1 * dutiescwc3.

GENLINMIXED
  /DATA_STRUCTURE SUBJECTS=companyId15
  /FIELDS TARGET=phycat16 TRIALS=NONE OFFSET=NONE
  /TARGET_OPTIONS DISTRIBUTION=BINOMIAL LINK=LOGIT
  /FIXED  EFFECTS=physical_health kess_t15 choicecwc3 choicem3 fac3choi
  bullying_freq time_pressure_freq duties_freq
    FAC1_1 USE_INTERCEPT=TRUE
  /RANDOM USE_INTERCEPT=TRUE SUBJECTS=companyId15 COVARIANCE_TYPE=VARIANCE_COMPONENTS SOLUTION=TRUE
  /BUILD_OPTIONS TARGET_CATEGORY_ORDER=DESCENDING INPUTS_CATEGORY_ORDER=DESCENDING
    MAX_ITERATIONS=100 CONFIDENCE_LEVEL=95 DF_METHOD=RESIDUAL COVB=ROBUST PCONVERGE=0.000001(ABSOLUTE)
    SCORING=0 SINGULAR=0.000000000001
  /EMMEANS_OPTIONS SCALE=ORIGINAL PADJUST=LSD.
```

Generalized Linear Mixed Models

Notes

Output Created		16-AUG-2021 11:07:49
Comments		
Input	Data	C:\Users\jwb12ppu\Documents\Kevin\MPlus backup\Multicomponent health and wellbeing v2\Multicomponent for Mplus cat.sav
	Active Dataset	DataSet2
	Filter	kescat15 = 0 (FILTER)
	Weight	<none>
	Split File	<none>
	N of Rows in Working Data File	5546

Syntax	GENLINMIXED<br>/DATA_STRUCTURE<br>SUBJECTS=companyId15<br>/FIELDS TARGET=phycat16<br>TRIALS=NONE OFFSET=NONE<br>/TARGET_OPTIONS<br>DISTRIBUTION=BINOMIAL<br>LINK=LOGIT<br>/FIXED<br>EFFECTS=physical_health<br>kess_t15 choicecwc3 choicem3<br>fac3choi<br>bullying_freq time_pressure_freq<br>duties_freq<br>FAC1_1<br>USE_INTERCEPT=TRUE<br>/RANDOM<br>USE_INTERCEPT=TRUE<br>SUBJECTS=companyId15<br>COVARIANCE_TYPE=VARIANCE<br>_COMPONENTS<br>SOLUTION=TRUE<br>/BUILD_OPTIONS<br>TARGET_CATEGORY_ORDER=<br>DESCENDING<br>INPUTS_CATEGORY_ORDER=D<br>ESCENDING

		MAX_ITERATIONS=100 CONFIDENCE_LEVEL=95 DF_METHOD=RESIDUAL COVB=ROBUST PCONVERGE=0.000001(ABSOLUTE) SCORING=0 SINGULAR=0.000000000001 /EMMEANS_OPTIONS SCALE=ORIGINAL PADJUST=LSD.
Resources	Processor Time	00:00:03.09
	Elapsed Time	00:00:02.13

Warnings

glmm: One or more records are not used in the analysis because they have one or more fields with invalid or missing values.
glmm: Valid values for events (target) and trials variables are non-negative and positive integers respectively, and the number of trials cannot be less than the number of events.

Case Processing Summary

	N	Percent
Included	5069	91.4%

Excluded	477	8.6%
Total	5546	100.0%

Model Summary

Target		Phy16 > 2 = 1
Probability Distribution		Binomial
Link Function		Logit
Information Criterion	Akaike Corrected	28032.218
	Bayesian	28038.746

Information criteria are based on the -2 log likelihood (28030.217) and are used to compare models. Models with smaller information criterion values fit better.

Data Structure[a]

	Subjects company_code	Target Phy16 > 2 = 1
Data for First Subject	2	.00
	2	.00
	2	.00
	2	.00
	2	.00
	2	.00
	2	.00
	2	.00
	2	.00

	2	.00
Total Number of Levels	58	

Only the first 10 records are displayed.

a. Target: Phy16 > 2 = 1

Classification

Overall Percent Correct = 88.7%[a]

Observed		Predicted 1.00	.00
1.00	Count	1	570
	% within Observed	0.2%	99.8%
.00	Count	1	4497
	% within Observed	0.0%	100.0%

a. Target: Phy16 > 2 = 1

Fixed Effects[a]

Source	F	df1	df2	Sig.
Corrected Model	44.675	9	5059	.000
physical_health	170.792	1	5059	.000
kess_t15	43.498	1	5059	.000
choicecwc3	4.911	1	5059	.027
choicem3	2.603	1	5059	.107
fac3choi	2.793	1	5059	.095
bullying_freq	.232	1	5059	.630

time_pressure_freq	3.308	1	5059	.069
duties_freq	1.495	1	5059	.221
FAC1_1	18.093	1	5059	.000

Probability distribution: Binomial

Link function: Logit

a. Target: Phy16 > 2 = 1

Fixed Effects

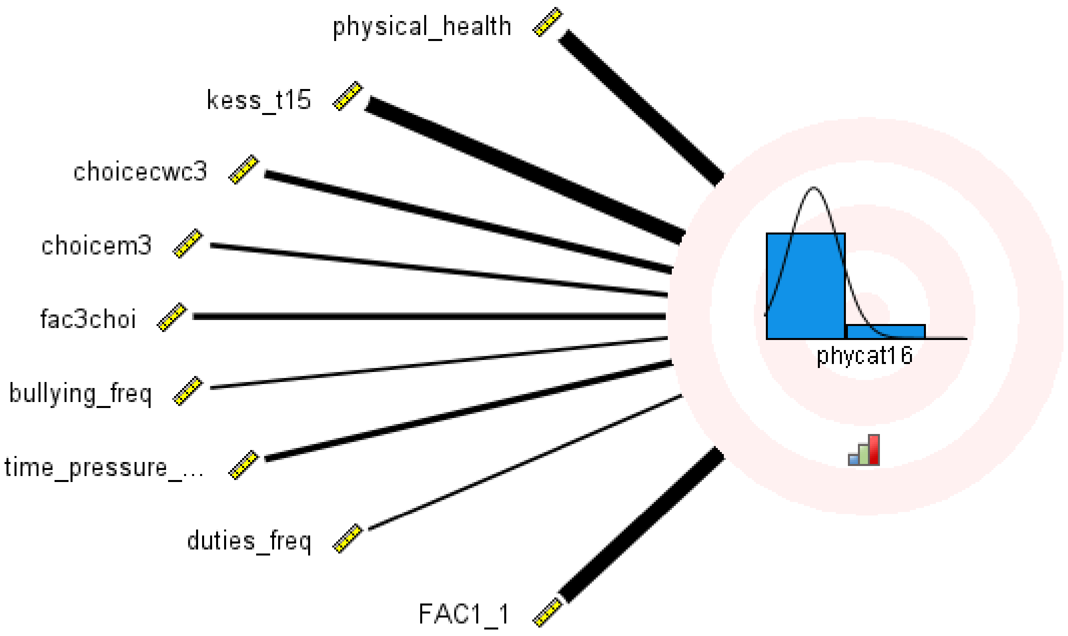

Fixed Coefficients[a]

Model Term	Coefficient	Std. Error	t	Sig.	95% Confidence Interval		Exp(Coefficient)	95% Confidence Interval for Exp(Coefficient)	
					Lower	Upper		Lower	Upper
Intercept	-4.306	.8335	-5.166	.000	-5.940	-2.672	.013	.003	.069
physical_health	1.999	.1530	13.069	.000	1.699	2.299	7.381	5.469	9.962
kess_t15	.423	.0641	6.595	.000	.297	.549	1.526	1.346	1.731
choicecwc3	-.102	.0462	-2.216	.027	-.193	-.012	.903	.825	.988
choicem3	-.401	.2485	-1.613	.107	-.888	.086	.670	.412	1.090
fac3choi	.078	.0466	1.671	.095	-.013	.169	1.081	.987	1.185
bullying_freq	.059	.1234	.481	.630	-.183	.301	1.061	.833	1.352
time_pressure_freq	-.065	.0358	-1.819	.069	-.135	.005	.937	.874	1.005
duties_freq	-.055	.0448	-1.223	.221	-.143	.033	.947	.867	1.034
FAC1_1	-.170	.0400	-4.254	.000	-.249	-.092	.844	.780	.912

Probability distribution: Binomial

Link function: Logit

a. Target: Phy16 > 2 = 1

Fixed Coefficients

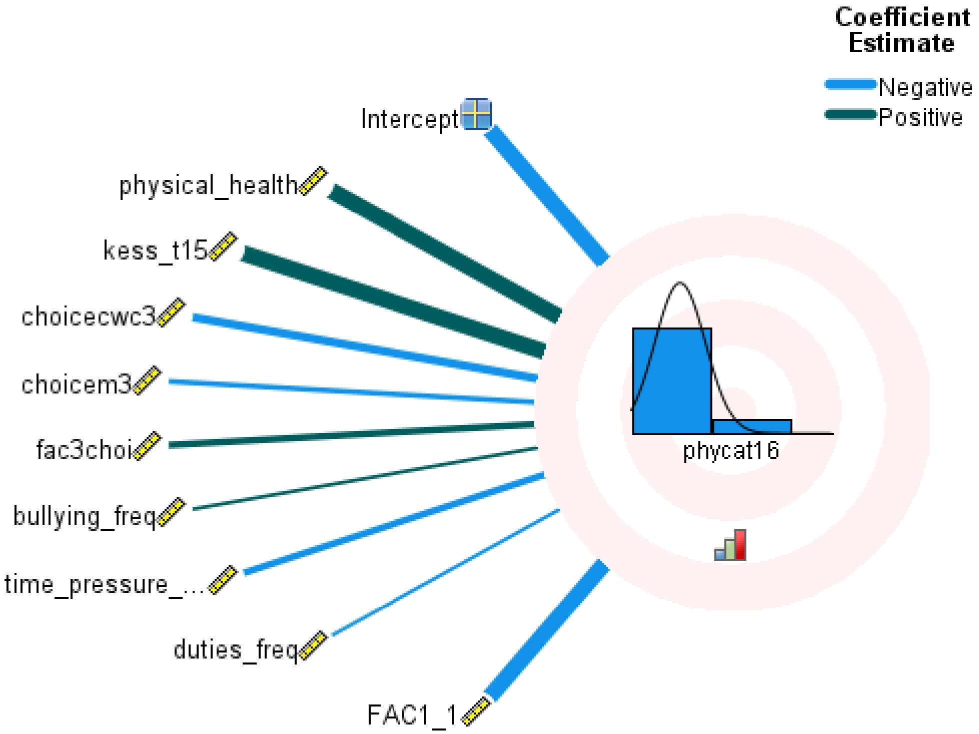

Random Effect Covariances

Random Effect Block 1

Random Effect Block	Intercept
Intercept	.043

Covariance Structure: Variance components

Subject Specification: companyId15

Covariance Parameters

Covariance Parameters Summary

Covariance Parameters	Residual Effect	0
	Random Effects	1
Design Matrix Columns	Fixed Effects	10
	Random Effects	1[a]
Common Subjects		58

Common subjects are based on the subject specifications for the residual and random effects and are used to chunk the data for better performance.

a. This is the number of columns per common subject.

Residual Effect

Residual Effect	Estimate	Std. Error	Z	Sig.	95% Confidence Interval	
					Lower	Upper
Variance	1.000	.	.	.	.	.

Covariance Structure: Scaled Identity

Subject Specification: (None)

Random Effect

Random Effect Covariance	Estimate	Std. Error	Z	Sig.	95% Confidence Interval	
					Lower	Upper
Var(Intercept)	.043	.036	1.198	.231	.008	.222

Covariance Structure: Variance components

Subject Specification: companyId15

Random Effects Parameters

Empirical Best Linear Unbiased Predictions[a]

company_code	Parameter	Prediction	Std. Error	df	Sig.	95% Confidence Interval	
						Lower Bound	Upper Bound
2	Intercept	.032	.185	5059	.864	-.331	.394

4	Intercept	-.053	.196	5059	.785	-.438	.331
6	Intercept	-.040	.188	5059	.832	-.409	.329
22	Intercept	-.032	.205	5059	.876	-.434	.370
24	Intercept	-.006	.162	5059	.968	-.324	.312
30	Intercept	.002	.194	5059	.991	-.378	.383
36	Intercept	-.033	.192	5059	.864	-.409	.343
40	Intercept	.107	.190	5059	.575	-.266	.480
50	Intercept	-.001	.197	5059	.996	-.387	.386
61	Intercept	.048	.191	5059	.803	-.327	.423
65	Intercept	.014	.201	5059	.943	-.380	.409
68	Intercept	-.008	.191	5059	.965	-.384	.367
69	Intercept	-.130	.188	5059	.489	-.499	.238
71	Intercept	.054	.196	5059	.784	-.331	.438
72	Intercept	.072	.192	5059	.707	-.304	.449
74	Intercept	.051	.196	5059	.793	-.333	.436
75	Intercept	.000	.192	5059	.999	-.377	.377
80	Intercept	-.020	.202	5059	.921	-.416	.376
81	Intercept	.076	.189	5059	.689	-.295	.446
83	Intercept	-.075	.190	5059	.693	-.447	.297
84	Intercept	-.070	.195	5059	.721	-.452	.313
92	Intercept	.018	.184	5059	.922	-.343	.379
93	Intercept	.007	.186	5059	.971	-.358	.372
98	Intercept	-.131	.182	5059	.474	-.488	.227
100	Intercept	-.183	.116	5059	.115	-.412	.045
106	Intercept	-.019	.199	5059	.926	-.409	.372
107	Intercept	-.011	.193	5059	.955	-.390	.368

108	Intercept	-.109	.198	5059	.582	-.498	.280
110	Intercept	.070	.121	5059	.561	-.167	.308
114	Intercept	.145	.193	5059	.455	-.235	.524
116	Intercept	.015	.187	5059	.937	-.353	.382
122	Intercept	.069	.167	5059	.679	-.259	.397
125	Intercept	.052	.155	5059	.737	-.251	.355
142	Intercept	-.199	.175	5059	.258	-.543	.145
144	Intercept	-.009	.188	5059	.963	-.376	.359
145	Intercept	-.032	.187	5059	.866	-.397	.334
152	Intercept	-.104	.187	5059	.578	-.471	.263
154	Intercept	-.051	.188	5059	.787	-.419	.318
156	Intercept	-.016	.173	5059	.926	-.355	.323
162	Intercept	-.026	.195	5059	.894	-.408	.356
165	Intercept	.005	.198	5059	.979	-.382	.393
166	Intercept	-.060	.203	5059	.766	-.457	.337
169	Intercept	.004	.193	5059	.983	-.374	.383
170	Intercept	.040	.194	5059	.836	-.341	.421
173	Intercept	-.077	.192	5059	.690	-.454	.300
174	Intercept	.050	.192	5059	.796	-.327	.427
178	Intercept	.424	.176	5059	.016	.078	.769
181	Intercept	-.035	.195	5059	.858	-.417	.347
182	Intercept	-.064	.199	5059	.747	-.454	.326
192	Intercept	-.032	.196	5059	.869	-.417	.352
202	Intercept	.002	.181	5059	.990	-.352	.357
204	Intercept	.064	.158	5059	.686	-.246	.374
205	Intercept	.038	.200	5059	.849	-.354	.431

206	Intercept	.013	.189	5059	.947	-.359	.384
209	Intercept	.013	.160	5059	.935	-.301	.327
217	Intercept	.094	.139	5059	.498	-.179	.368
226	Intercept	.167	.187	5059	.371	-.199	.533
228	Intercept	-.120	.195	5059	.538	-.502	.262

a. Target: Phy16 > 2 = 1

```
GENLINMIXED
  /DATA_STRUCTURE SUBJECTS=companyId15
  /FIELDS TARGET=phycat16 TRIALS=NONE OFFSET=NONE
  /TARGET_OPTIONS DISTRIBUTION=BINOMIAL LINK=LOGIT
  /FIXED  EFFECTS=physical_health kess_t15 choice_freq bullycwc3 bullym3 fac3bull
   time_pressure_freq duties_freq
    FAC1_1 USE_INTERCEPT=TRUE
  /RANDOM USE_INTERCEPT=TRUE SUBJECTS=companyId15 COVARIANCE_TYPE=VARIANCE_COMPONENTS SOLUTION=TRUE
  /BUILD_OPTIONS TARGET_CATEGORY_ORDER=DESCENDING INPUTS_CATEGORY_ORDER=DESCENDING
    MAX_ITERATIONS=100 CONFIDENCE_LEVEL=95 DF_METHOD=RESIDUAL COVB=ROBUST PCONVERGE=0.000001(ABSOLUTE)
    SCORING=0 SINGULAR=0.000000000001
  /EMMEANS_OPTIONS SCALE=ORIGINAL PADJUST=LSD.
```

Generalized Linear Mixed Models

Notes

Output Created	16-AUG-2021 11:07:51
Comments	

Input	Data	C:\Users\jwb12ppu\Documents\Kevin\MPlus backup\Multicomponent health and wellbeing v2\Multicomponent for Mplus cat.sav
	Active Dataset	DataSet2
	Filter	kescat15 = 0 (FILTER)
	Weight	<none>
	Split File	<none>
	N of Rows in Working Data File	5546

Syntax	GENLINMIXED<br>/DATA_STRUCTURE<br>SUBJECTS=companyId15<br>/FIELDS TARGET=phycat16<br>TRIALS=NONE OFFSET=NONE<br>/TARGET_OPTIONS<br>DISTRIBUTION=BINOMIAL<br>LINK=LOGIT<br>/FIXED<br>EFFECTS=physical_health<br>kess_t15 choice_freq bullycwc3<br>bullym3 fac3bull<br>time_pressure_freq duties_freq<br>FAC1_1<br>USE_INTERCEPT=TRUE<br>/RANDOM<br>USE_INTERCEPT=TRUE<br>SUBJECTS=companyId15<br>COVARIANCE_TYPE=VARIANCE<br>_COMPONENTS<br>SOLUTION=TRUE<br>/BUILD_OPTIONS<br>TARGET_CATEGORY_ORDER=<br>DESCENDING<br>INPUTS_CATEGORY_ORDER=D<br>ESCENDING<br>MAX_ITERATIONS=100<br>CONFIDENCE_LEVEL=95

		DF_METHOD=RESIDUAL COVB=ROBUST PCONVERGE=0.000001(ABSOLUTE) SCORING=0 SINGULAR=0.000000000001 /EMMEANS_OPTIONS SCALE=ORIGINAL PADJUST=LSD.
Resources	Processor Time	00:00:01.80
	Elapsed Time	00:00:01.92

Warnings

glmm: One or more records are not used in the analysis because they have one or more fields with invalid or missing values.
glmm: Valid values for events (target) and trials variables are non-negative and positive integers respectively, and the number of trials cannot be less than the number of events.

Case Processing Summary

	N	Percent
Included	5069	91.4%
Excluded	477	8.6%
Total	5546	100.0%

Model Summary

Target		Phy16 > 2 = 1
Probability Distribution		Binomial
Link Function		Logit
Information Criterion	Akaike Corrected	28026.548
	Bayesian	28033.076

Information criteria are based on the -2 log likelihood (28024.547) and are used to compare models. Models with smaller information criterion values fit better.

Data Structure[a]

	Subjects company_code	Target Phy16 > 2 = 1
Data for First Subject	2	.00
	2	.00
	2	.00
	2	.00
	2	.00
	2	.00
	2	.00
	2	.00
	2	.00
	2	.00
Total Number of Levels	58	

Only the first 10 records are displayed.

a. Target: Phy16 > 2 = 1

Classification

Overall Percent Correct = 88.8%[a]

Observed		Predicted 1.00	Predicted .00
1.00	Count	1	570
	% within Observed	0.2%	99.8%
.00	Count	0	4498
	% within Observed	0.0%	100.0%

a. Target: Phy16 > 2 = 1

Fixed Effects[a]

Source	F	df1	df2	Sig.
Corrected Model	45.069	9	5059	.000
physical_health	171.005	1	5059	.000
kess_t15	44.528	1	5059	.000
choice_freq	7.702	1	5059	.006
bullycwc3	.244	1	5059	.621
bullym3	.102	1	5059	.750
fac3bull	.007	1	5059	.935
time_pressure_freq	3.043	1	5059	.081
duties_freq	1.555	1	5059	.212

FAC1_1	19.710	1	5059	.000

Probability distribution: Binomial

Link function: Logit

a. Target: Phy16 > 2 = 1

Fixed Effects

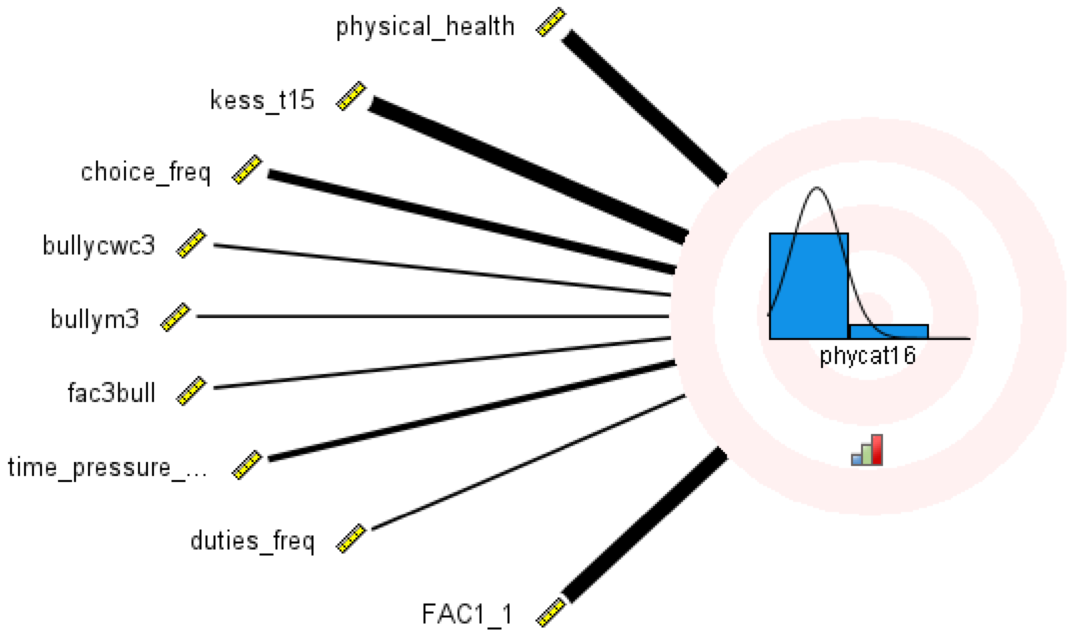

Fixed Coefficients[a]

Model Term	Coefficient	Std. Error	t	Sig.	95% Confidence Interval		Exp(Coefficient)	95% Confidence Interval for Exp(Coefficient)	
					Lower	Upper		Lower	Upper
Intercept	-5.425	.9406	-5.768	.000	-7.269	-3.581	.004	.001	.028
physical_health	1.999	.1529	13.077	.000	1.700	2.299	7.385	5.472	9.966
kess_t15	.422	.0632	6.673	.000	.298	.546	1.525	1.347	1.726
choice_freq	-.120	.0434	-2.775	.006	-.205	-.035	.887	.814	.965
bullycwc3	.062	.1246	.494	.621	-.183	.306	1.063	.833	1.358
bullym3	.233	.7291	.319	.750	-1.197	1.662	1.262	.302	5.269
fac3bull	-.010	.1247	-.082	.935	-.255	.234	.990	.775	1.264
time_pressure_freq	-.063	.0362	-1.744	.081	-.134	.008	.939	.875	1.008
duties_freq	-.055	.0443	-1.247	.212	-.142	.032	.946	.868	1.032
FAC1_1	-.188	.0423	-4.440	.000	-.271	-.105	.829	.763	.900

Probability distribution: Binomial

Link function: Logit

a. Target: Phy16 > 2 = 1

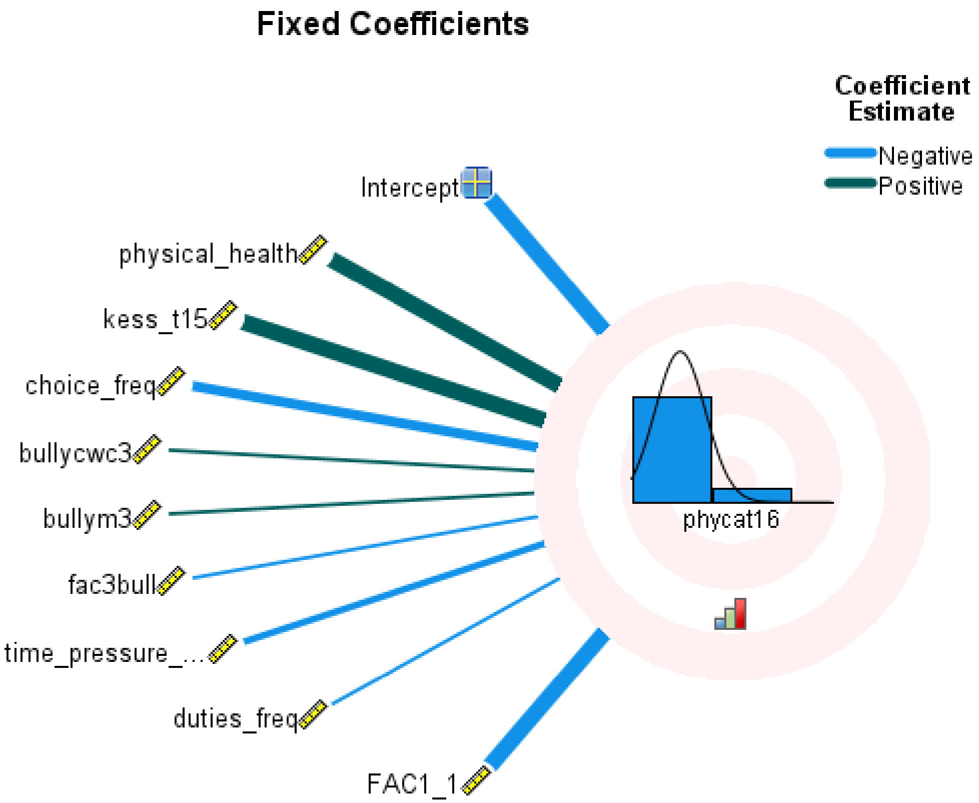

Random Effect Covariances

Random Effect Block 1

Random Effect Block	Intercept
Intercept	.040

Covariance Structure: Variance components

Subject Specification: companyId15

Covariance Parameters

Covariance Parameters Summary

Covariance Parameters	Residual Effect	0
	Random Effects	1
Design Matrix Columns	Fixed Effects	10
	Random Effects	1[a]
Common Subjects		58

Common subjects are based on the subject specifications for the residual and random effects and are used to chunk the data for better performance.

a. This is the number of columns per common subject.

Residual Effect

Residual Effect	Estimate	Std. Error	Z	Sig.	95% Confidence Interval	
					Lower	Upper
Variance	1.000	.	.	.	.	.

Covariance Structure: Scaled Identity

Subject Specification: (None)

Random Effect

Random Effect Covariance	Estimate	Std. Error	Z	Sig.	95% Confidence Interval	
					Lower	Upper
Var(Intercept)	.040	.036	1.120	.263	.007	.230

Covariance Structure: Variance components

Subject Specification: companyId15

Random Effects Parameters

Empirical Best Linear Unbiased Predictions[a]

company_code	Parameter	Prediction	Std. Error	df	Sig.	95% Confidence Interval	
						Lower Bound	Upper Bound
2	Intercept	.025	.179	5059	.891	-.326	.376

4	Intercept	-.055	.189	5059	.771	-.426	.316
6	Intercept	-.038	.182	5059	.835	-.395	.319
22	Intercept	-.028	.198	5059	.888	-.415	.360
24	Intercept	-.016	.158	5059	.922	-.325	.294
30	Intercept	.006	.188	5059	.976	-.363	.374
36	Intercept	-.035	.185	5059	.849	-.399	.328
40	Intercept	.098	.184	5059	.593	-.263	.459
50	Intercept	-.010	.190	5059	.956	-.382	.361
61	Intercept	.039	.185	5059	.831	-.323	.402
65	Intercept	.013	.194	5059	.946	-.368	.394
68	Intercept	-.027	.183	5059	.884	-.386	.332
69	Intercept	-.124	.182	5059	.495	-.481	.232
71	Intercept	.054	.190	5059	.776	-.318	.426
72	Intercept	.055	.185	5059	.766	-.307	.417
74	Intercept	.052	.190	5059	.786	-.321	.424
75	Intercept	.000	.186	5059	.999	-.365	.364
80	Intercept	-.021	.195	5059	.912	-.403	.360
81	Intercept	.078	.183	5059	.672	-.282	.437
83	Intercept	-.057	.185	5059	.759	-.419	.305
84	Intercept	-.074	.188	5059	.694	-.442	.294
92	Intercept	.012	.178	5059	.946	-.337	.361
93	Intercept	-2.747E-6	.179	5059	1.000	-.352	.352
98	Intercept	-.130	.176	5059	.460	-.475	.215
100	Intercept	-.131	.116	5059	.260	-.359	.097
106	Intercept	-.028	.191	5059	.882	-.403	.346
107	Intercept	-.017	.186	5059	.927	-.383	.348

108	Intercept	-.105	.191	5059	.582	-.481	.270
110	Intercept	.067	.124	5059	.589	-.177	.311
114	Intercept	.121	.186	5059	.516	-.244	.485
116	Intercept	.012	.182	5059	.946	-.344	.368
122	Intercept	.095	.164	5059	.564	-.227	.417
125	Intercept	.054	.152	5059	.722	-.244	.353
142	Intercept	-.177	.171	5059	.300	-.513	.158
144	Intercept	-.013	.181	5059	.941	-.369	.342
145	Intercept	-.018	.182	5059	.923	-.374	.339
152	Intercept	-.115	.181	5059	.523	-.470	.239
154	Intercept	-.057	.182	5059	.755	-.413	.300
156	Intercept	.010	.169	5059	.954	-.322	.342
162	Intercept	-.029	.188	5059	.876	-.398	.339
165	Intercept	-.006	.190	5059	.973	-.379	.366
166	Intercept	-.063	.195	5059	.748	-.444	.319
169	Intercept	-.009	.185	5059	.962	-.372	.355
170	Intercept	.031	.187	5059	.870	-.337	.398
173	Intercept	-.074	.186	5059	.689	-.439	.290
174	Intercept	.028	.184	5059	.881	-.334	.389
178	Intercept	.379	.170	5059	.025	.047	.712
181	Intercept	-.032	.189	5059	.866	-.403	.339
182	Intercept	-.070	.191	5059	.715	-.445	.305
192	Intercept	-.040	.189	5059	.833	-.411	.331
202	Intercept	-.002	.176	5059	.993	-.346	.343
204	Intercept	.114	.153	5059	.455	-.185	.413
205	Intercept	.038	.193	5059	.845	-.341	.417

206	Intercept	.012	.184	5059	.946	-.348	.372
209	Intercept	.040	.157	5059	.801	-.268	.347
217	Intercept	.132	.140	5059	.347	-.143	.406
226	Intercept	.159	.181	5059	.380	-.196	.515
228	Intercept	-.120	.188	5059	.523	-.489	.249

a. Target: Phy16 > 2 = 1

```
GENLINMIXED
  /DATA_STRUCTURE SUBJECTS=companyId15
  /FIELDS TARGET=phycat16 TRIALS=NONE OFFSET=NONE
  /TARGET_OPTIONS DISTRIBUTION=BINOMIAL LINK=LOGIT
  /FIXED  EFFECTS=physical_health kess_t15 choice_freq bullying_freq timecwc3 timem3 fac3time
  duties_freq
    FAC1_1 USE_INTERCEPT=TRUE
  /RANDOM USE_INTERCEPT=TRUE SUBJECTS=companyId15 COVARIANCE_TYPE=VARIANCE_COMPONENTS SOLUTION=TRUE
  /BUILD_OPTIONS TARGET_CATEGORY_ORDER=DESCENDING INPUTS_CATEGORY_ORDER=DESCENDING
    MAX_ITERATIONS=100 CONFIDENCE_LEVEL=95 DF_METHOD=RESIDUAL COVB=ROBUST PCONVERGE=0.000001(ABSOLUTE)
    SCORING=0 SINGULAR=0.000000000001
  /EMMEANS_OPTIONS SCALE=ORIGINAL PADJUST=LSD.
```

Generalized Linear Mixed Models

Notes

Output Created	16-AUG-2021 11:07:53
Comments	

Input	Data	C:\Users\jwb12ppu\Documents\Kevin\MPlus backup\Multicomponent health and wellbeing v2\Multicomponent for Mplus cat.sav
	Active Dataset	DataSet2
	Filter	kescat15 = 0 (FILTER)
	Weight	<none>
	Split File	<none>
	N of Rows in Working Data File	5546

Syntax	GENLINMIXED<br>/DATA_STRUCTURE<br>SUBJECTS=companyId15<br>/FIELDS TARGET=phycat16<br>TRIALS=NONE OFFSET=NONE<br>/TARGET_OPTIONS<br>DISTRIBUTION=BINOMIAL<br>LINK=LOGIT<br>/FIXED<br>EFFECTS=physical_health<br>kess_t15 choice_freq bullying_freq<br>timecwc3 timem3 fac3time<br>duties_freq<br>FAC1_1<br>USE_INTERCEPT=TRUE<br>/RANDOM<br>USE_INTERCEPT=TRUE<br>SUBJECTS=companyId15<br>COVARIANCE_TYPE=VARIANCE<br>_COMPONENTS<br>SOLUTION=TRUE<br>/BUILD_OPTIONS<br>TARGET_CATEGORY_ORDER=<br>DESCENDING<br>INPUTS_CATEGORY_ORDER=D<br>ESCENDING<br>MAX_ITERATIONS=100<br>CONFIDENCE_LEVEL=95

		DF_METHOD=RESIDUAL COVB=ROBUST PCONVERGE=0.000001(ABSOLUTE) SCORING=0 SINGULAR=0.000000000001 /EMMEANS_OPTIONS SCALE=ORIGINAL PADJUST=LSD.
Resources	Processor Time	00:00:02.47
	Elapsed Time	00:00:02.47

Warnings

glmm: One or more records are not used in the analysis because they have one or more fields with invalid or missing values.
glmm: Valid values for events (target) and trials variables are non-negative and positive integers respectively, and the number of trials cannot be less than the number of events.

Case Processing Summary

	N	Percent
Included	5069	91.4%
Excluded	477	8.6%
Total	5546	100.0%

Model Summary

Target		Phy16 > 2 = 1
Probability Distribution		Binomial
Link Function		Logit
Information Criterion	Akaike Corrected	28030.813
	Bayesian	28037.342

Information criteria are based on the -2 log likelihood (28028.813) and are used to compare models. Models with smaller information criterion values fit better.

Data Structure[a]

	Subjects company_code	Target Phy16 > 2 = 1
Data for First Subject	2	.00
	2	.00
	2	.00
	2	.00
	2	.00
	2	.00
	2	.00
	2	.00
	2	.00
	2	.00
Total Number of Levels	58	

Only the first 10 records are displayed.

a. Target: Phy16 > 2 = 1

Classification

Overall Percent Correct = 88.8%[a]

Observed		Predicted 1.00	Predicted .00
1.00	Count	1	570
	% within Observed	0.2%	99.8%
.00	Count	0	4498
	% within Observed	0.0%	100.0%

a. Target: Phy16 > 2 = 1

Fixed Effects[a]

Source	F	df1	df2	Sig.
Corrected Model	45.995	9	5059	.000
physical_health	169.836	1	5059	.000
kess_t15	45.965	1	5059	.000
choice_freq	8.160	1	5059	.004
bullying_freq	.265	1	5059	.607
timecwc3	2.622	1	5059	.105
timem3	.762	1	5059	.383
fac3time	.001	1	5059	.975
duties_freq	1.621	1	5059	.203

FAC1_1	21.699	1	5059	.000

Probability distribution: Binomial

Link function: Logit

a. Target: Phy16 > 2 = 1

Fixed Effects

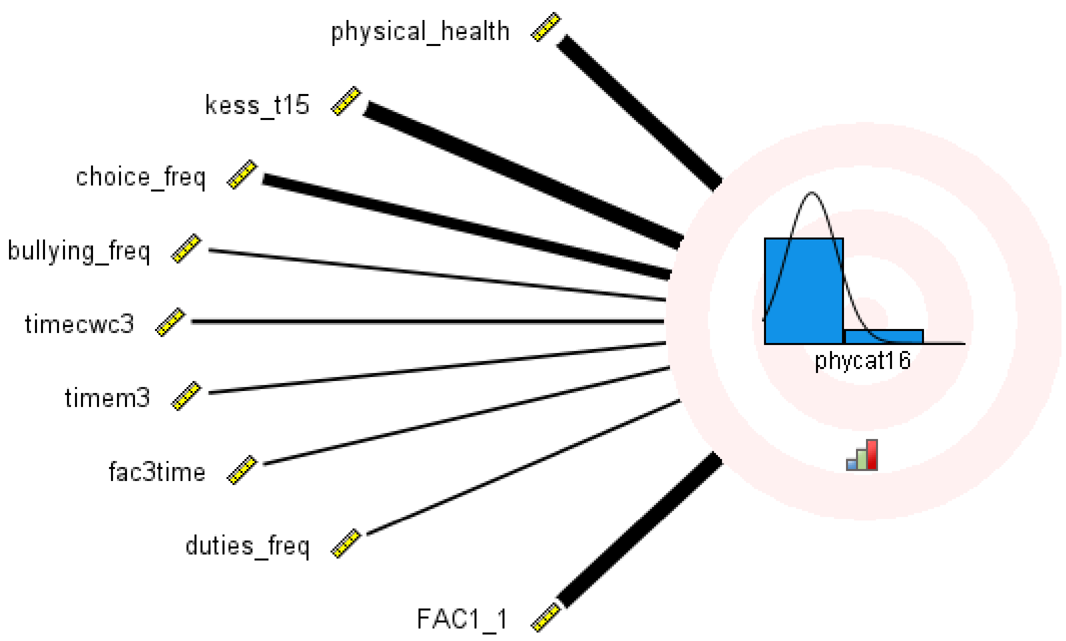

Fixed Coefficients[a]

Model Term	Coefficient	Std. Error	t	Sig.	95% Confidence Interval Lower	95% Confidence Interval Upper	Exp(Coefficient)	95% Confidence Interval for Exp(Coefficient) Lower	95% Confidence Interval for Exp(Coefficient) Upper
Intercept	-4.889	.7196	-6.794	.000	-6.300	-3.478	.008	.002	.031
physical_health	2.001	.1535	13.032	.000	1.700	2.302	7.397	5.474	9.995
kess_t15	.420	.0620	6.780	.000	.299	.542	1.522	1.348	1.719
choice_freq	-.122	.0427	-2.857	.004	-.206	-.038	.885	.814	.962
bullying_freq	.064	.1235	.515	.607	-.178	.306	1.066	.837	1.358
timecwc3	-.056	.0348	-1.619	.105	-.124	.012	.945	.883	1.012
timem3	-.200	.2289	-.873	.383	-.648	.249	.819	.523	1.283
fac3time	.001	.0373	.031	.975	-.072	.074	1.001	.931	1.077
duties_freq	-.057	.0444	-1.273	.203	-.144	.031	.945	.866	1.031
FAC1_1	-.184	.0394	-4.658	.000	-.261	-.106	.832	.770	.899

Probability distribution: Binomial

Link function: Logit

a. Target: Phy16 > 2 = 1

Fixed Coefficients

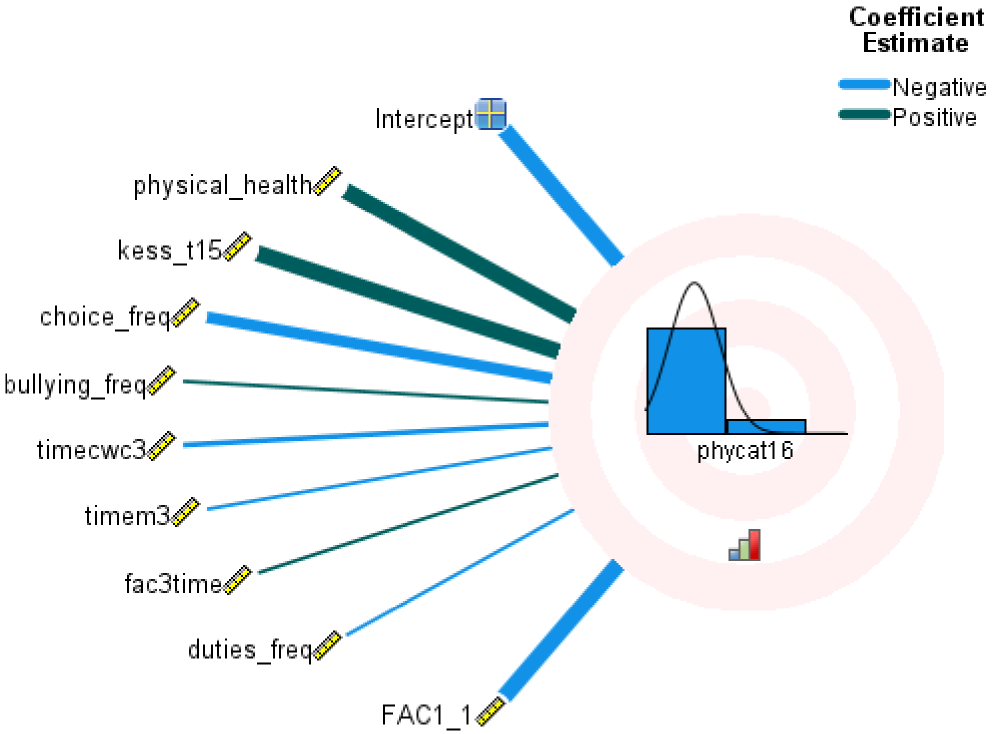

Random Effect Covariances

Random Effect Block 1

Random Effect Block	Intercept
Intercept	.039

Covariance Structure: Variance components

Subject Specification: companyId15

Covariance Parameters

Covariance Parameters Summary

Covariance Parameters	Residual Effect	0
	Random Effects	1
Design Matrix Columns	Fixed Effects	10
	Random Effects	1[a]
Common Subjects		58

Common subjects are based on the subject specifications for the residual and random effects and are used to chunk the data for better performance.

a. This is the number of columns per common subject.

Residual Effect

Residual Effect	Estimate	Std. Error	Z	Sig.	95% Confidence Interval	
					Lower	Upper
Variance	1.000	.	.	.	.	.

Covariance Structure: Scaled Identity

Subject Specification: (None)

Random Effect

Random Effect Covariance	Estimate	Std. Error	Z	Sig.	95% Confidence Interval	
					Lower	Upper
Var(Intercept)	.039	.036	1.077	.282	.006	.240

Covariance Structure: Variance components

Subject Specification: companyId15

Random Effects Parameters

Empirical Best Linear Unbiased Predictions[a]

company_code	Parameter	Prediction	Std. Error	df	Sig.	95% Confidence Interval	
						Lower Bound	Upper Bound
2	Intercept	.024	.177	5059	.894	-.323	.370

4	Intercept	-.063	.186	5059	.735	-.428	.302
6	Intercept	-.039	.180	5059	.828	-.392	.313
22	Intercept	-.027	.195	5059	.891	-.409	.355
24	Intercept	.000	.159	5059	.998	-.312	.313
30	Intercept	.002	.185	5059	.992	-.362	.365
36	Intercept	-.039	.183	5059	.831	-.397	.319
40	Intercept	.095	.182	5059	.602	-.262	.451
50	Intercept	-.016	.187	5059	.933	-.382	.351
61	Intercept	.031	.182	5059	.864	-.326	.388
65	Intercept	.009	.191	5059	.961	-.366	.385
68	Intercept	-.030	.181	5059	.867	-.385	.324
69	Intercept	-.115	.181	5059	.526	-.469	.240
71	Intercept	.058	.188	5059	.759	-.310	.425
72	Intercept	.048	.182	5059	.793	-.310	.405
74	Intercept	.054	.187	5059	.774	-.313	.421
75	Intercept	.001	.184	5059	.996	-.359	.361
80	Intercept	-.026	.192	5059	.894	-.402	.350
81	Intercept	.061	.181	5059	.738	-.295	.416
83	Intercept	-.059	.182	5059	.745	-.416	.298
84	Intercept	-.073	.185	5059	.695	-.436	.291
92	Intercept	.012	.176	5059	.946	-.333	.357
93	Intercept	-.001	.177	5059	.993	-.349	.346
98	Intercept	-.121	.175	5059	.488	-.464	.222
100	Intercept	-.109	.114	5059	.340	-.332	.115
106	Intercept	-.030	.189	5059	.873	-.400	.340
107	Intercept	-.021	.184	5059	.908	-.382	.339

108	Intercept	-.100	.189	5059	.598	-.470	.271
110	Intercept	.083	.124	5059	.502	-.159	.325
114	Intercept	.112	.183	5059	.539	-.246	.471
116	Intercept	.004	.179	5059	.984	-.347	.355
122	Intercept	.112	.164	5059	.496	-.210	.434
125	Intercept	.074	.158	5059	.642	-.236	.383
142	Intercept	-.153	.174	5059	.380	-.493	.188
144	Intercept	-.021	.179	5059	.908	-.371	.330
145	Intercept	-.021	.179	5059	.909	-.372	.331
152	Intercept	-.108	.178	5059	.542	-.456	.240
154	Intercept	-.049	.180	5059	.783	-.402	.303
156	Intercept	-.008	.168	5059	.963	-.336	.321
162	Intercept	-.031	.185	5059	.869	-.394	.333
165	Intercept	-.007	.187	5059	.971	-.374	.360
166	Intercept	-.059	.192	5059	.759	-.436	.318
169	Intercept	-.009	.183	5059	.961	-.368	.350
170	Intercept	.021	.185	5059	.908	-.341	.384
173	Intercept	-.087	.184	5059	.637	-.447	.274
174	Intercept	.031	.182	5059	.867	-.327	.388
178	Intercept	.380	.169	5059	.025	.049	.710
181	Intercept	-.028	.186	5059	.881	-.393	.337
182	Intercept	-.080	.189	5059	.672	-.449	.290
192	Intercept	-.040	.186	5059	.830	-.405	.325
202	Intercept	-.006	.173	5059	.974	-.346	.334
204	Intercept	.135	.155	5059	.382	-.168	.439
205	Intercept	.032	.191	5059	.868	-.342	.405

206	Intercept	.007	.181	5059	.969	-.348	.362
209	Intercept	.029	.155	5059	.853	-.275	.333
217	Intercept	.124	.137	5059	.366	-.144	.392
226	Intercept	.147	.179	5059	.411	-.204	.498
228	Intercept	-.111	.186	5059	.551	-.476	.254

a. Target: Phy16 > 2 = 1

```
GENLINMIXED
  /DATA_STRUCTURE SUBJECTS=companyId15
  /FIELDS TARGET=phycat16 TRIALS=NONE OFFSET=NONE
  /TARGET_OPTIONS DISTRIBUTION=BINOMIAL LINK=LOGIT
  /FIXED  EFFECTS=physical_health kess_t15 choice_freq bullying_freq time_pressure_freq dutiescwc3 dutiesm3 fac3duti
    FAC1_1 USE_INTERCEPT=TRUE
  /RANDOM USE_INTERCEPT=TRUE SUBJECTS=companyId15 COVARIANCE_TYPE=VARIANCE_COMPONENTS SOLUTION=TRUE
  /BUILD_OPTIONS TARGET_CATEGORY_ORDER=DESCENDING INPUTS_CATEGORY_ORDER=DESCENDING
    MAX_ITERATIONS=100 CONFIDENCE_LEVEL=95 DF_METHOD=RESIDUAL COVB=ROBUST PCONVERGE=0.000001(ABSOLUTE)
    SCORING=0 SINGULAR=0.000000000001
  /EMMEANS_OPTIONS SCALE=ORIGINAL PADJUST=LSD.
```

Generalized Linear Mixed Models

Notes

Output Created	16-AUG-2021 11:07:56
Comments	

Input	Data	C:\Users\jwb12ppu\Documents\Kevin\MPlus backup\Multicomponent health and wellbeing v2\Multicomponent for Mplus cat.sav
	Active Dataset	DataSet2
	Filter	kescat15 = 0 (FILTER)
	Weight	<none>
	Split File	<none>
	N of Rows in Working Data File	5546

Syntax	GENLINMIXED<br>/DATA_STRUCTURE<br>SUBJECTS=companyId15<br>/FIELDS TARGET=phycat16<br>TRIALS=NONE OFFSET=NONE<br>/TARGET_OPTIONS<br>DISTRIBUTION=BINOMIAL<br>LINK=LOGIT<br>/FIXED<br>EFFECTS=physical_health<br>kess_t15 choice_freq bullying_freq<br>time_pressure_freq dutiescwc3<br>dutiesm3 fac3duti<br>FAC1_1<br>USE_INTERCEPT=TRUE<br>/RANDOM<br>USE_INTERCEPT=TRUE<br>SUBJECTS=companyId15<br>COVARIANCE_TYPE=VARIANCE<br>_COMPONENTS<br>SOLUTION=TRUE<br>/BUILD_OPTIONS<br>TARGET_CATEGORY_ORDER=<br>DESCENDING<br>INPUTS_CATEGORY_ORDER=D<br>ESCENDING<br>MAX_ITERATIONS=100<br>CONFIDENCE_LEVEL=95

		DF_METHOD=RESIDUAL COVB=ROBUST PCONVERGE=0.000001(ABSOLUTE) SCORING=0 SINGULAR=0.000000000001 /EMMEANS_OPTIONS SCALE=ORIGINAL PADJUST=LSD.
Resources	Processor Time	00:00:01.83
	Elapsed Time	00:00:01.86

Warnings

glmm: One or more records are not used in the analysis because they have one or more fields with invalid or missing values.
glmm: Valid values for events (target) and trials variables are non-negative and positive integers respectively, and the number of trials cannot be less than the number of events.

Case Processing Summary

	N	Percent
Included	5069	91.4%
Excluded	477	8.6%
Total	5546	100.0%

Model Summary

Target		Phy16 > 2 = 1
Probability Distribution		Binomial
Link Function		Logit
Information Criterion	Akaike Corrected	28029.443
	Bayesian	28035.971

Information criteria are based on the -2 log likelihood (28027.442) and are used to compare models. Models with smaller information criterion values fit better.

Data Structure[a]

	Subjects company_code	Target Phy16 > 2 = 1
Data for First Subject	2	.00
	2	.00
	2	.00
	2	.00
	2	.00
	2	.00
	2	.00
	2	.00
	2	.00
	2	.00
Total Number of Levels	58	

Only the first 10 records are displayed.

a. Target: Phy16 > 2 = 1

Classification

Overall Percent Correct = 88.8%[a]

Observed		Predicted 1.00	Predicted .00
1.00	Count	1	570
	% within Observed	0.2%	99.8%
.00	Count	0	4498
	% within Observed	0.0%	100.0%

a. Target: Phy16 > 2 = 1

Fixed Effects[a]

Source	F	df1	df2	Sig.
Corrected Model	45.588	9	5059	.000
physical_health	169.855	1	5059	.000
kess_t15	44.107	1	5059	.000
choice_freq	7.904	1	5059	.005
bullying_freq	.270	1	5059	.603
time_pressure_freq	3.180	1	5059	.075
dutiescwc3	1.460	1	5059	.227
dutiesm3	.298	1	5059	.585
fac3duti	.257	1	5059	.612

FAC1_1	23.042	1	5059	.000

Probability distribution: Binomial

Link function: Logit

a. Target: Phy16 > 2 = 1

Fixed Effects

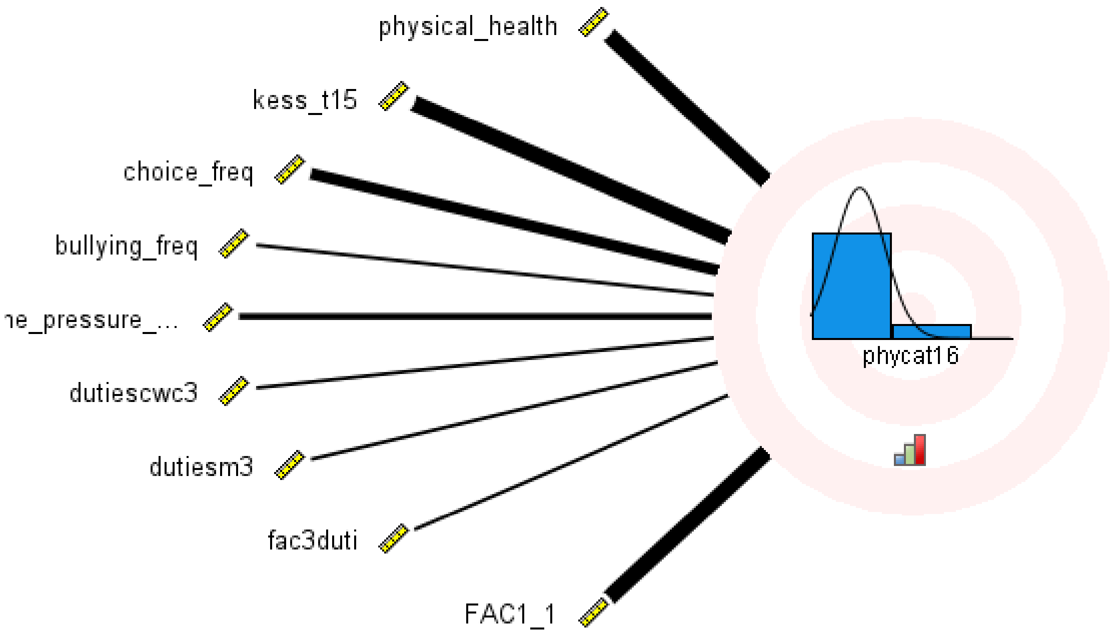

Fixed Coefficients[a]

Model Term	Coefficient	Std. Error	t	Sig.	95% Confidence Interval		Exp(Coefficient)	95% Confidence Interval for Exp(Coefficient)	
					Lower	Upper		Lower	Upper
Intercept	-4.759	1.2911	-3.686	.000	-7.290	-2.228	.009	.001	.108
physical_health	1.999	.1534	13.033	.000	1.699	2.300	7.384	5.466	9.975
kess_t15	.421	.0633	6.641	.000	.296	.545	1.523	1.345	1.724
choice_freq	-.121	.0430	-2.811	.005	-.205	-.037	.886	.814	.964
bullying_freq	.064	.1224	.520	.603	-.176	.304	1.066	.838	1.355
time_pressure_freq	-.064	.0359	-1.783	.075	-.135	.006	.938	.874	1.006
dutiescwc3	-.056	.0463	-1.208	.227	-.147	.035	.946	.864	1.035
dutiesm3	-.167	.3062	-.546	.585	-.767	.433	.846	.464	1.542
fac3duti	-.020	.0388	-.507	.612	-.096	.056	.981	.909	1.058
FAC1_1	-.187	.0390	-4.800	.000	-.264	-.111	.829	.768	.895

Probability distribution: Binomial

Link function: Logit

a. Target: Phy16 > 2 = 1

Fixed Coefficients

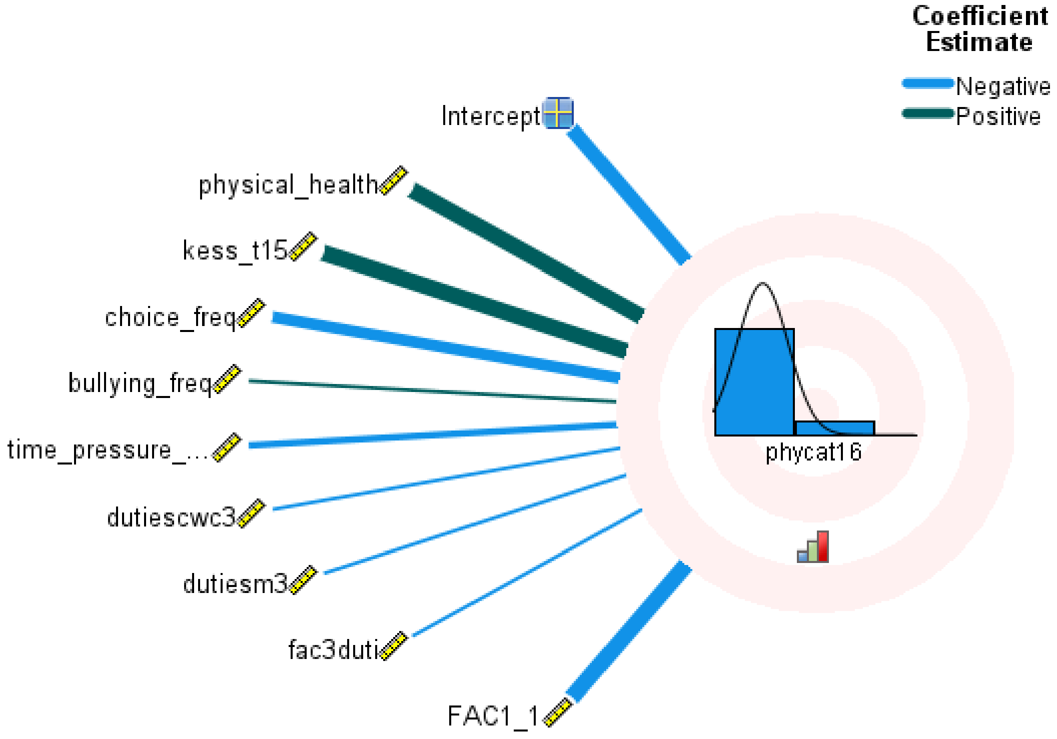

Random Effect Covariances

Random Effect Block 1

Random Effect Block	Intercept
Intercept	.040

Covariance Structure: Variance components

Subject Specification: companyId15

Covariance Parameters

Covariance Parameters Summary

Covariance Parameters	Residual Effect	0
	Random Effects	1
Design Matrix Columns	Fixed Effects	10
	Random Effects	1[a]
Common Subjects		58

Common subjects are based on the subject specifications for the residual and random effects and are used to chunk the data for better performance.

a. This is the number of columns per common subject.

Residual Effect

Residual Effect	Estimate	Std. Error	Z	Sig.	95% Confidence Interval	
					Lower	Upper
Variance	1.000	.	.	.	.	.

Covariance Structure: Scaled Identity

Subject Specification: (None)

Random Effect

Random Effect Covariance	Estimate	Std. Error	Z	Sig.	95% Confidence Interval	
					Lower	Upper
Var(Intercept)	.040	.035	1.136	.256	.007	.226

Covariance Structure: Variance components

Subject Specification: companyId15

Random Effects Parameters

Empirical Best Linear Unbiased Predictions[a]

company_code	Parameter	Prediction	Std. Error	df	Sig.	95% Confidence Interval	
						Lower Bound	Upper Bound
2	Intercept	.027	.180	5059	.880	-.325	.379

4	Intercept	-.055	.190	5059	.774	-.427	.318
6	Intercept	-.041	.183	5059	.821	-.399	.317
22	Intercept	-.028	.199	5059	.888	-.417	.361
24	Intercept	-.019	.159	5059	.906	-.330	.293
30	Intercept	.007	.189	5059	.971	-.363	.377
36	Intercept	-.037	.186	5059	.842	-.401	.327
40	Intercept	.096	.185	5059	.602	-.266	.458
50	Intercept	-.010	.190	5059	.960	-.383	.364
61	Intercept	.044	.186	5059	.813	-.321	.409
65	Intercept	.015	.195	5059	.938	-.367	.398
68	Intercept	-.029	.184	5059	.876	-.389	.332
69	Intercept	-.127	.183	5059	.487	-.485	.231
71	Intercept	.051	.191	5059	.788	-.322	.425
72	Intercept	.057	.185	5059	.760	-.307	.420
74	Intercept	.052	.191	5059	.786	-.322	.425
75	Intercept	.005	.187	5059	.978	-.362	.372
80	Intercept	-.020	.196	5059	.919	-.404	.364
81	Intercept	.083	.185	5059	.653	-.280	.446
83	Intercept	-.059	.185	5059	.752	-.421	.304
84	Intercept	-.072	.189	5059	.704	-.442	.298
92	Intercept	.012	.179	5059	.948	-.339	.362
93	Intercept	.000	.180	5059	.998	-.353	.353
98	Intercept	-.131	.177	5059	.458	-.477	.215
100	Intercept	-.134	.116	5059	.250	-.361	.094
106	Intercept	-.030	.192	5059	.877	-.406	.347
107	Intercept	-.016	.187	5059	.931	-.383	.351

108	Intercept	-.108	.192	5059	.573	-.484	.268
110	Intercept	.051	.142	5059	.720	-.228	.330
114	Intercept	.120	.186	5059	.518	-.245	.485
116	Intercept	.011	.182	5059	.950	-.345	.368
122	Intercept	.103	.163	5059	.529	-.217	.423
125	Intercept	.053	.152	5059	.726	-.245	.352
142	Intercept	-.180	.172	5059	.294	-.518	.157
144	Intercept	-.015	.182	5059	.933	-.372	.341
145	Intercept	-.026	.182	5059	.888	-.383	.332
152	Intercept	-.111	.180	5059	.539	-.464	.243
154	Intercept	-.055	.182	5059	.763	-.412	.302
156	Intercept	.007	.169	5059	.969	-.325	.338
162	Intercept	-.025	.189	5059	.893	-.396	.345
165	Intercept	-.008	.190	5059	.967	-.381	.365
166	Intercept	-.061	.196	5059	.754	-.445	.322
169	Intercept	-.008	.186	5059	.964	-.373	.357
170	Intercept	.030	.188	5059	.875	-.339	.398
173	Intercept	-.075	.187	5059	.688	-.441	.291
174	Intercept	.032	.185	5059	.863	-.331	.395
178	Intercept	.380	.171	5059	.026	.046	.714
181	Intercept	-.032	.190	5059	.867	-.403	.340
182	Intercept	-.070	.192	5059	.717	-.446	.307
192	Intercept	-.037	.190	5059	.847	-.408	.335
202	Intercept	.002	.176	5059	.990	-.344	.348
204	Intercept	.116	.152	5059	.446	-.182	.414
205	Intercept	.040	.194	5059	.836	-.341	.421

206	Intercept	.014	.184	5059	.941	-.348	.375
209	Intercept	.046	.157	5059	.771	-.263	.355
217	Intercept	.130	.138	5059	.346	-.140	.400
226	Intercept	.159	.182	5059	.383	-.198	.515
228	Intercept	-.125	.189	5059	.510	-.496	.246

a. Target: Phy16 > 2 = 1

```
FILTER OFF.
USE ALL.
EXECUTE.
```

```
USE ALL.
COMPUTE filter_$=(phycat15 = 1).
VARIABLE LABELS filter_$ 'kescat15 = 0 (FILTER)'.
VALUE LABELS filter_$ 0 'Not Selected' 1 'Selected'.
FORMATS filter_$ (f1.0).
FILTER BY filter_$.
EXECUTE.
```

```
correlations /variables = phycat16 phycat15 fac1_1.
```

Correlations

Notes

Output Created		16-AUG-2021 11:07:58
Comments		
Input	Data	C:\Users\jwb12ppu\Documents\Kevin\MPlus backup\Multicomponent health and wellbeing v2\Multicomponent for Mplus cat.sav
	Active Dataset	DataSet2
	Filter	kescat15 = 0 (FILTER)
	Weight	<none>
	Split File	<none>
	N of Rows in Working Data File	1422
Missing Value Handling	Definition of Missing	User-defined missing values are treated as missing.
	Cases Used	Statistics for each pair of variables are based on all the cases with valid data for that pair.
Syntax		correlations /variables = phycat16 phycat15 fac1_1.
Resources	Processor Time	00:00:00.02
	Elapsed Time	00:00:00.02

Correlations

		Phy16 > 2 = 1	phy15 > 2 = 1	REGR factor score 1 for analysis 1
Phy16 > 2 = 1	Pearson Correlation	1	.[a]	-.029
	Sig. (2-tailed)		.	.269
	N	1422	1422	1422
phy15 > 2 = 1	Pearson Correlation	.[a]	.[a]	.[a]
	Sig. (2-tailed)	.		.
	N	1422	1422	1422
REGR factor score 1 for analysis 1	Pearson Correlation	-.029	.[a]	1
	Sig. (2-tailed)	.269	.	
	N	1422	1422	1422

a. Cannot be computed because at least one of the variables is constant.

```
*Generalized Linear Mixed Models.
GENLINMIXED
  /DATA_STRUCTURE SUBJECTS=companyId15
  /FIELDS TARGET=phycat16 TRIALS=NONE OFFSET=NONE
  /TARGET_OPTIONS DISTRIBUTION=BINOMIAL LINK=LOGIT
  /FIXED  EFFECTS=physical_health kess_t15 choice_freq bullying_freq time_pressure_freq duties_freq
    FAC1_1 USE_INTERCEPT=TRUE
  /RANDOM USE_INTERCEPT=TRUE SUBJECTS=companyId15 COVARIANCE_TYPE=VARIANCE_COMPONENTS SOLUTION=TRUE
  /BUILD_OPTIONS TARGET_CATEGORY_ORDER=DESCENDING INPUTS_CATEGORY_ORDER=DESCENDING
    MAX_ITERATIONS=100 CONFIDENCE_LEVEL=95 DF_METHOD=RESIDUAL COVB=ROBUST PCONVERGE=0.000001(ABSOLUTE)
    SCORING=0 SINGULAR=0.000000000001
  /EMMEANS_OPTIONS SCALE=ORIGINAL PADJUST=LSD.
```

Generalized Linear Mixed Models

Notes

Output Created		16-AUG-2021 11:07:58
Comments		
Input	Data	C:\Users\jwb12ppu\Documents\Kevin\MPlus backup\Multicomponent health and wellbeing v2\Multicomponent for Mplus cat.sav
	Active Dataset	DataSet2
	Filter	kescat15 = 0 (FILTER)
	Weight	<none>
	Split File	<none>
	N of Rows in Working Data File	1422

Syntax	GENLINMIXED<br>/DATA_STRUCTURE<br>SUBJECTS=companyId15<br>/FIELDS TARGET=phycat16<br>TRIALS=NONE OFFSET=NONE<br>/TARGET_OPTIONS<br>DISTRIBUTION=BINOMIAL<br>LINK=LOGIT<br>/FIXED<br>EFFECTS=physical_health<br>kess_t15 choice_freq bullying_freq<br>time_pressure_freq duties_freq<br>FAC1_1<br>USE_INTERCEPT=TRUE<br>/RANDOM<br>USE_INTERCEPT=TRUE<br>SUBJECTS=companyId15<br>COVARIANCE_TYPE=VARIANCE<br>_COMPONENTS<br>SOLUTION=TRUE<br>/BUILD_OPTIONS<br>TARGET_CATEGORY_ORDER=<br>DESCENDING<br>INPUTS_CATEGORY_ORDER=D<br>ESCENDING<br>MAX_ITERATIONS=100<br>CONFIDENCE_LEVEL=95<br>DF_METHOD=RESIDUAL

		COVB=ROBUST PCONVERGE=0.000001(ABSOLUTE) SCORING=0 SINGULAR=0.000000000001 /EMMEANS_OPTIONS SCALE=ORIGINAL PADJUST=LSD.
Resources	Processor Time	00:00:01.58
	Elapsed Time	00:00:01.36

Warnings

glmm: One or more records are not used in the analysis because they have one or more fields with invalid or missing values.
glmm: Valid values for events (target) and trials variables are non-negative and positive integers respectively, and the number of trials cannot be less than the number of events.
glmm: The final Hessian matrix is not positive definite although all convergence criteria are satisfied. The procedure continues despite this warning. Subsequent results produced are based on the last iteration. Validity of the model fit is uncertain.

Case Processing Summary

	N	Percent

Included	1370	96.3%
Excluded	52	3.7%
Total	1422	100.0%

Model Summary

Target		Phy16 > 2 = 1
Probability Distribution		Binomial
Link Function		Logit
Information Criterion	Akaike Corrected	6099.700
	Bayesian	6104.914

Information criteria are based on the -2 log likelihood (6097.697) and are used to compare models. Models with smaller information criterion values fit better.

Data Structure[a]

	Subjects<br>company_code	Target<br>Phy16 > 2 = 1
Data for First Subject	2	1.00
	2	.00
	2	1.00
	2	.00
	2	1.00
	2	1.00
	2	.00
	2	1.00

	2	.00
	2	1.00
Total Number of Levels	57	

Only the first 10 records are displayed.

a. Target: Phy16 > 2 = 1

Classification
Overall Percent Correct = 63.9%[a]

Observed		Predicted 1.00	.00
1.00	Count	858	12
	% within Observed	98.6%	1.4%
.00	Count	483	17
	% within Observed	96.6%	3.4%

a. Target: Phy16 > 2 = 1

Fixed Effects[a]

Source	F	df1	df2	Sig.
Corrected Model	18.437	7	1362	.000
physical_health	32.185	1	1362	.000
kess_t15	11.684	1	1362	.001
choice_freq	4.970	1	1362	.026
bullying_freq	1.151	1	1362	.283
time_pressure_freq	.000	1	1362	.983

duties_freq	.807	1	1362	.369
FAC1_1	2.999	1	1362	.084

Probability distribution: Binomial

Link function: Logit

a. Target: Phy16 > 2 = 1

Fixed Effects

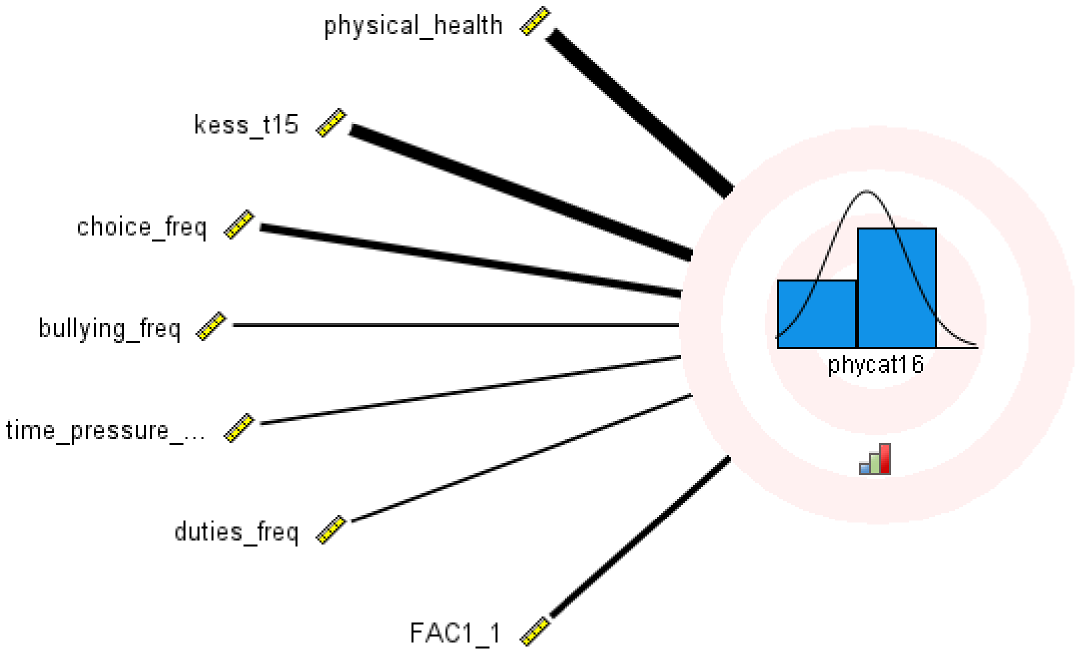

Fixed Coefficients[a]

Model Term	Coefficient	Std. Error	t	Sig.	95% Confidence Interval		Exp(Coefficient)	95% Confidence Interval for Exp(Coefficient)	
					Lower	Upper		Lower	Upper
Intercept	-4.193	.7711	-5.438	.000	-5.706	-2.680	.015	.003	.069
physical_health	1.427	.2516	5.673	.000	.934	1.921	4.168	2.544	6.828
kess_t15	.272	.0796	3.418	.001	.116	.428	1.313	1.123	1.535
choice_freq	-.080	.0360	-2.229	.026	-.151	-.010	.923	.860	.990
bullying_freq	.097	.0902	1.073	.283	-.080	.274	1.102	.923	1.315
time_pressure_freq	-.001	.0587	-.021	.983	-.116	.114	.999	.890	1.121
duties_freq	.041	.0457	.898	.369	-.049	.131	1.042	.953	1.140
FAC1_1	-.080	.0460	-1.732	.084	-.170	.011	.923	.844	1.011

Probability distribution: Binomial

Link function: Logit

a. Target: Phy16 > 2 = 1

Fixed Coefficients

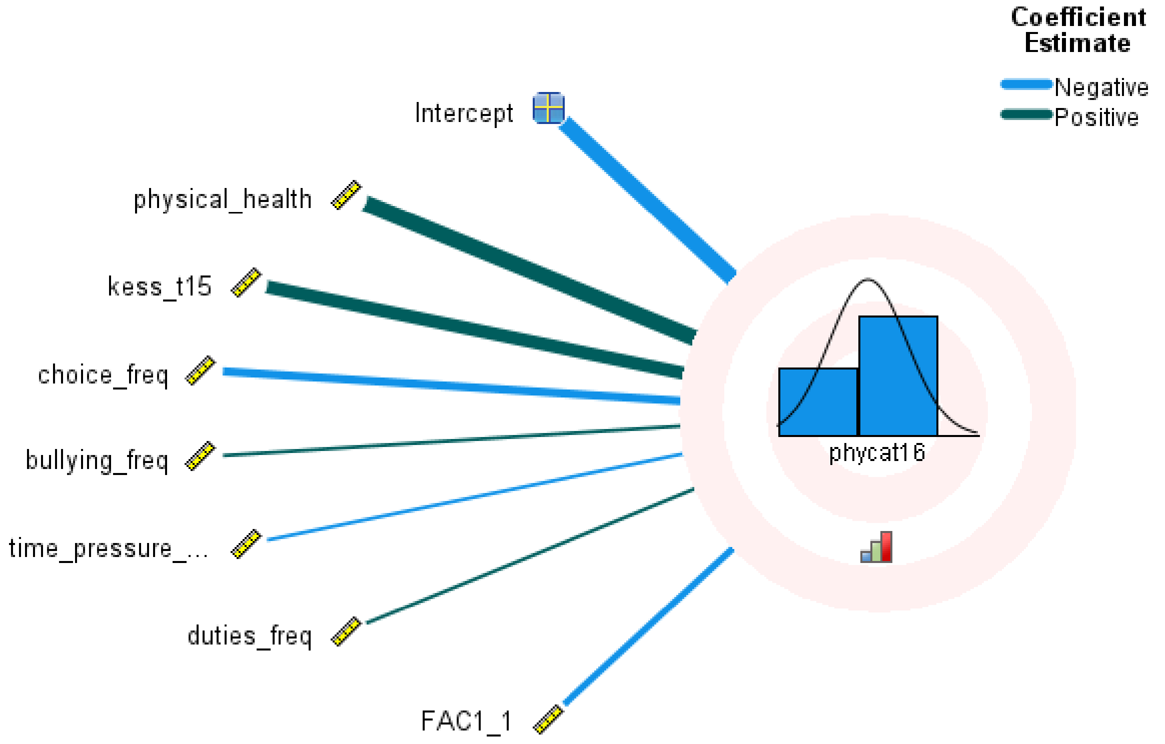

Random Effect Covariances

Random Effect Block 1

Random Effect Block	Intercept
Intercept	.000

Covariance Structure: Variance components

Subject Specification: companyId15

Covariance Parameters

Covariance Parameters Summary

Covariance Parameters	Residual Effect	0
	Random Effects	1
Design Matrix Columns	Fixed Effects	8
	Random Effects	1[a]
Common Subjects		57

Common subjects are based on the subject specifications for the residual and random effects and are used to chunk the data for better performance.

a. This is the number of columns per common subject.

Residual Effect

Residual Effect	Estimate	Std. Error	Z	Sig.	95% Confidence Interval	
					Lower	Upper
Variance	1.000	.	.	.	.	.

Covariance Structure: Scaled Identity

Subject Specification: (None)

Random Effect

Random Effect Covariance	Estimate	Std. Error	Z	Sig.	95% Confidence Interval	
					Lower	Upper
Var(Intercept)	9.733E-12[a]	.	.	.	.	.

Covariance Structure: Variance components

Subject Specification: companyId15

a. This parameter is redundant.

Random Effects Parameters

Empirical Best Linear Unbiased Predictions[a]

company_code	Parameter	Prediction	Std. Error	df	Sig.	95% Confidence Interval	
						Lower Bound	Upper Bound

2	Intercept	-1.258E-11	3.120E-6	1362	1.000	-6.120E-6	6.120E-6
4	Intercept	-1.054E-11	3.120E-6	1362	1.000	-6.120E-6	6.120E-6
6	Intercept	-8.709E-12	3.120E-6	1362	1.000	-6.120E-6	6.120E-6
22	Intercept	-1.927E-12	3.120E-6	1362	1.000	-6.120E-6	6.120E-6
24	Intercept	-1.562E-11	3.120E-6	1362	1.000	-6.120E-6	6.120E-6
30	Intercept	3.789E-12	3.120E-6	1362	1.000	-6.120E-6	6.120E-6
36	Intercept	1.458E-12	3.120E-6	1362	1.000	-6.120E-6	6.120E-6
40	Intercept	-9.591E-12	3.120E-6	1362	1.000	-6.120E-6	6.120E-6
50	Intercept	-2.975E-12	3.120E-6	1362	1.000	-6.120E-6	6.120E-6
61	Intercept	1.424E-12	3.120E-6	1362	1.000	-6.120E-6	6.120E-6
65	Intercept	9.606E-12	3.120E-6	1362	1.000	-6.120E-6	6.120E-6
68	Intercept	3.279E-12	3.120E-6	1362	1.000	-6.120E-6	6.120E-6
69	Intercept	-6.365E-12	3.120E-6	1362	1.000	-6.120E-6	6.120E-6
71	Intercept	1.806E-11	3.120E-6	1362	1.000	-6.120E-6	6.120E-6
72	Intercept	-8.108E-12	3.120E-6	1362	1.000	-6.120E-6	6.120E-6
74	Intercept	9.402E-12	3.120E-6	1362	1.000	-6.120E-6	6.120E-6
75	Intercept	-1.084E-11	3.120E-6	1362	1.000	-6.120E-6	6.120E-6
80	Intercept	-1.089E-11	3.120E-6	1362	1.000	-6.120E-6	6.120E-6
81	Intercept	9.830E-12	3.120E-6	1362	1.000	-6.120E-6	6.120E-6
83	Intercept	-9.819E-12	3.120E-6	1362	1.000	-6.120E-6	6.120E-6
84	Intercept	-2.716E-12	3.120E-6	1362	1.000	-6.120E-6	6.120E-6
92	Intercept	-1.304E-11	3.120E-6	1362	1.000	-6.120E-6	6.120E-6
93	Intercept	4.467E-12	3.120E-6	1362	1.000	-6.120E-6	6.120E-6
98	Intercept	-2.287E-11	3.120E-6	1362	1.000	-6.120E-6	6.120E-6
100	Intercept	-7.786E-11	3.120E-6	1362	1.000	-6.120E-6	6.120E-6
106	Intercept	-9.667E-12	3.120E-6	1362	1.000	-6.120E-6	6.120E-6

107	Intercept	-4.792E-12	3.120E-6	1362	1.000	-6.120E-6	6.120E-6
108	Intercept	-2.017E-11	3.120E-6	1362	1.000	-6.120E-6	6.120E-6
110	Intercept	-7.294E-12	3.120E-6	1362	1.000	-6.120E-6	6.120E-6
114	Intercept	-9.833E-13	3.120E-6	1362	1.000	-6.120E-6	6.120E-6
116	Intercept	8.577E-12	3.120E-6	1362	1.000	-6.120E-6	6.120E-6
122	Intercept	3.404E-11	3.120E-6	1362	1.000	-6.120E-6	6.120E-6
125	Intercept	2.209E-11	3.120E-6	1362	1.000	-6.120E-6	6.120E-6
142	Intercept	-3.431E-11	3.120E-6	1362	1.000	-6.120E-6	6.120E-6
144	Intercept	-2.004E-11	3.120E-6	1362	1.000	-6.120E-6	6.120E-6
145	Intercept	-6.376E-12	3.120E-6	1362	1.000	-6.120E-6	6.120E-6
152	Intercept	3.107E-11	3.120E-6	1362	1.000	-6.120E-6	6.120E-6
154	Intercept	1.068E-11	3.120E-6	1362	1.000	-6.120E-6	6.120E-6
156	Intercept	2.125E-11	3.120E-6	1362	1.000	-6.120E-6	6.120E-6
162	Intercept	7.155E-12	3.120E-6	1362	1.000	-6.120E-6	6.120E-6
165	Intercept	3.543E-12	3.120E-6	1362	1.000	-6.120E-6	6.120E-6
166	Intercept	-1.112E-12	3.120E-6	1362	1.000	-6.120E-6	6.120E-6
169	Intercept	7.615E-12	3.120E-6	1362	1.000	-6.120E-6	6.120E-6
170	Intercept	-8.652E-12	3.120E-6	1362	1.000	-6.120E-6	6.120E-6
173	Intercept	1.108E-11	3.120E-6	1362	1.000	-6.120E-6	6.120E-6
174	Intercept	2.035E-11	3.120E-6	1362	1.000	-6.120E-6	6.120E-6
178	Intercept	6.604E-12	3.120E-6	1362	1.000	-6.120E-6	6.120E-6
181	Intercept	2.059E-11	3.120E-6	1362	1.000	-6.120E-6	6.120E-6
192	Intercept	-1.355E-11	3.120E-6	1362	1.000	-6.120E-6	6.120E-6
202	Intercept	2.595E-11	3.120E-6	1362	1.000	-6.120E-6	6.120E-6
204	Intercept	4.697E-11	3.120E-6	1362	1.000	-6.120E-6	6.120E-6
205	Intercept	-1.249E-11	3.120E-6	1362	1.000	-6.120E-6	6.120E-6

206	Intercept	2.536E-12	3.120E-6	1362	1.000	-6.120E-6	6.120E-6
209	Intercept	2.989E-11	3.120E-6	1362	1.000	-6.120E-6	6.120E-6
217	Intercept	-1.465E-11	3.120E-6	1362	1.000	-6.120E-6	6.120E-6
226	Intercept	8.853E-12	3.120E-6	1362	1.000	-6.120E-6	6.120E-6
228	Intercept	-1.639E-12	3.120E-6	1362	1.000	-6.120E-6	6.120E-6

a. Target: Phy16 > 2 = 1

```
GENLINMIXED
  /DATA_STRUCTURE SUBJECTS=companyId15
  /FIELDS TARGET=phycat16 TRIALS=NONE OFFSET=NONE
  /TARGET_OPTIONS DISTRIBUTION=BINOMIAL LINK=LOGIT
  /FIXED  EFFECTS=physical_health kess_t15 choice_freq bullying_freq time_pressure_freq duties_freq
    FAC1_1 facsq USE_INTERCEPT=TRUE
  /RANDOM USE_INTERCEPT=TRUE SUBJECTS=companyId15 COVARIANCE_TYPE=VARIANCE_COMPONENTS SOLUTION=TRUE
  /BUILD_OPTIONS TARGET_CATEGORY_ORDER=DESCENDING INPUTS_CATEGORY_ORDER=DESCENDING
    MAX_ITERATIONS=100 CONFIDENCE_LEVEL=95 DF_METHOD=RESIDUAL COVB=ROBUST PCONVERGE=0.000001(ABSOLUTE)
    SCORING=0 SINGULAR=0.000000000001
  /EMMEANS_OPTIONS SCALE=ORIGINAL PADJUST=LSD.
```

Generalized Linear Mixed Models

Notes

Output Created	16-AUG-2021 11:07:59
Comments	

Input	Data	C:\Users\jwb12ppu\Documents\Kevin\MPlus backup\Multicomponent health and wellbeing v2\Multicomponent for Mplus cat.sav
	Active Dataset	DataSet2
	Filter	kescat15 = 0 (FILTER)
	Weight	<none>
	Split File	<none>
	N of Rows in Working Data File	1422

Syntax	GENLINMIXED<br>/DATA_STRUCTURE SUBJECTS=companyId15<br>/FIELDS TARGET=phycat16 TRIALS=NONE OFFSET=NONE<br>/TARGET_OPTIONS DISTRIBUTION=BINOMIAL LINK=LOGIT<br>/FIXED EFFECTS=physical_health kess_t15 choice_freq bullying_freq time_pressure_freq duties_freq<br>FAC1_1 facsq USE_INTERCEPT=TRUE<br>/RANDOM USE_INTERCEPT=TRUE SUBJECTS=companyId15 COVARIANCE_TYPE=VARIANCE_COMPONENTS SOLUTION=TRUE<br>/BUILD_OPTIONS TARGET_CATEGORY_ORDER=DESCENDING INPUTS_CATEGORY_ORDER=DESCENDING<br>MAX_ITERATIONS=100 CONFIDENCE_LEVEL=95 DF_METHOD=RESIDUAL

		COVB=ROBUST PCONVERGE=0.000001(ABSOLUTE) SCORING=0 SINGULAR=0.000000000001 /EMMEANS_OPTIONS SCALE=ORIGINAL PADJUST=LSD.
Resources	Processor Time	00:00:01.09
	Elapsed Time	00:00:01.17

Warnings

glmm: One or more records are not used in the analysis because they have one or more fields with invalid or missing values.
glmm: Valid values for events (target) and trials variables are non-negative and positive integers respectively, and the number of trials cannot be less than the number of events.
glmm: The final Hessian matrix is not positive definite although all convergence criteria are satisfied. The procedure continues despite this warning. Subsequent results produced are based on the last iteration. Validity of the model fit is uncertain.

Case Processing Summary

	N	Percent

Included	1370	96.3%
Excluded	52	3.7%
Total	1422	100.0%

Model Summary

Target		Phy16 > 2 = 1
Probability Distribution		Binomial
Link Function		Logit
Information Criterion	Akaike Corrected	6101.657
	Bayesian	6106.870

Information criteria are based on the -2 log likelihood (6099.654) and are used to compare models. Models with smaller information criterion values fit better.

Data Structure[a]

	Subjects company_code	Target Phy16 > 2 = 1
Data for First Subject	2	1.00
	2	.00
	2	1.00
	2	.00
	2	1.00
	2	1.00
	2	.00
	2	1.00

	2	.00
	2	1.00
Total Number of Levels	57	

Only the first 10 records are displayed.

a. Target: Phy16 > 2 = 1

Classification
Overall Percent Correct = 63.8%[a]

Observed		Predicted 1.00	Predicted .00
1.00	Count	854	16
	% within Observed	98.2%	1.8%
.00	Count	480	20
	% within Observed	96.0%	4.0%

a. Target: Phy16 > 2 = 1

Fixed Effects[a]

Source	F	df1	df2	Sig.
Corrected Model	15.850	8	1361	.000
physical_health	32.228	1	1361	.000
kess_t15	11.619	1	1361	.001
choice_freq	4.393	1	1361	.036
bullying_freq	1.119	1	1361	.290
time_pressure_freq	.002	1	1361	.965

duties_freq	.726	1	1361	.394
FAC1_1	2.807	1	1361	.094
facsq	.397	1	1361	.529

Probability distribution: Binomial

Link function: Logit

a. Target: Phy16 > 2 = 1

Fixed Effects

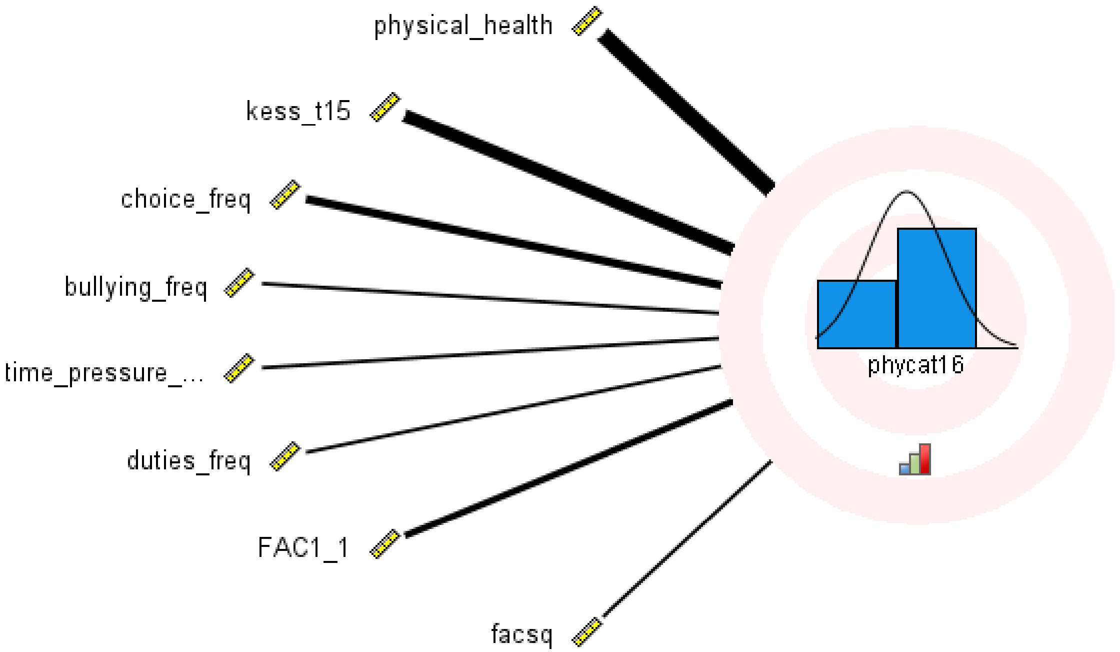

Fixed Coefficients[a]

Model Term	Coefficient	Std. Error	t	Sig.	95% Confidence Interval		Exp(Coefficient)	95% Confidence Interval for Exp(Coefficient)	
					Lower	Upper		Lower	Upper
Intercept	-4.159	.7805	-5.329	.000	-5.690	-2.628	.016	.003	.072
physical_health	1.428	.2515	5.677	.000	.934	1.921	4.169	2.545	6.827
kess_t15	.273	.0800	3.409	.001	.116	.430	1.313	1.123	1.537
choice_freq	-.077	.0370	-2.096	.036	-.150	-.005	.925	.861	.995
bullying_freq	.095	.0900	1.058	.290	-.081	.272	1.100	.922	1.312
time_pressure_freq	-.003	.0594	-.043	.965	-.119	.114	.997	.888	1.121
duties_freq	.040	.0465	.852	.394	-.052	.131	1.040	.950	1.140
FAC1_1	-.094	.0559	-1.675	.094	-.203	.016	.911	.816	1.016
facsq	-.033	.0524	-.630	.529	-.136	.070	.968	.873	1.072

Probability distribution: Binomial

Link function: Logit

a. Target: Phy16 > 2 = 1

Fixed Coefficients

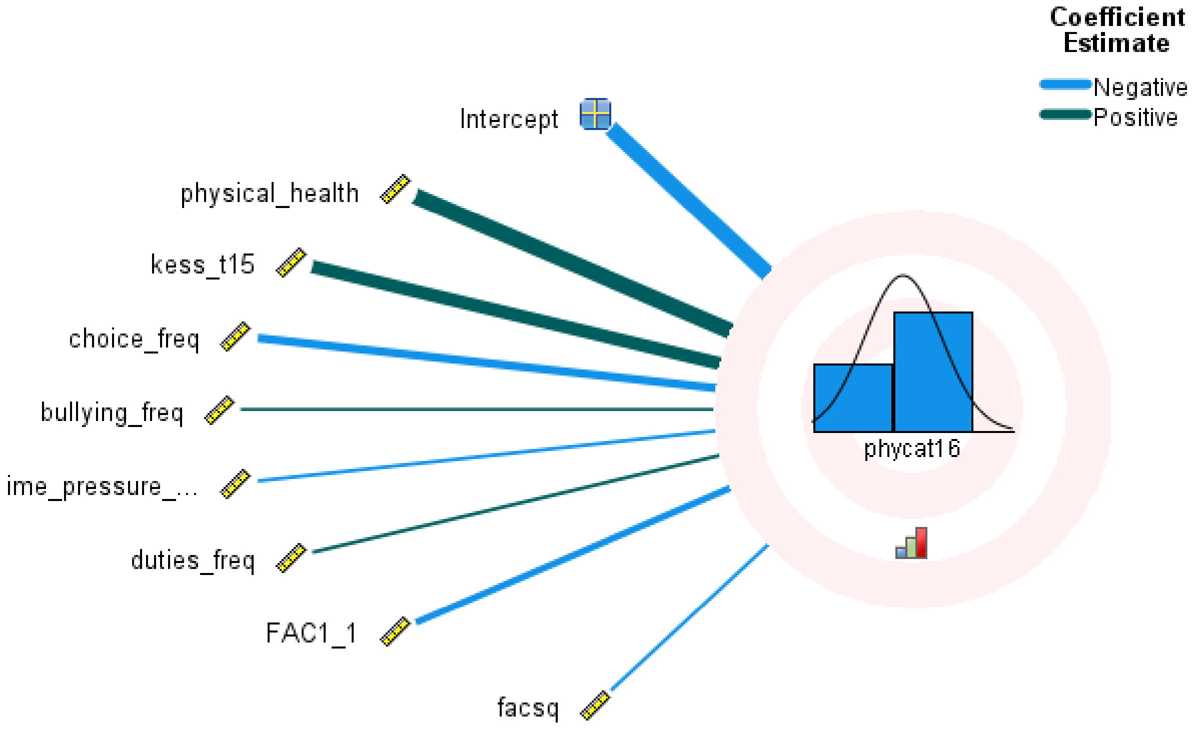

Random Effect Covariances

Random Effect Block 1

Random Effect Block	Intercept
Intercept	.000

Covariance Structure: Variance components

Subject Specification: companyId15

Covariance Parameters

Covariance Parameters Summary

Covariance Parameters	Residual Effect	0
	Random Effects	1
Design Matrix Columns	Fixed Effects	9
	Random Effects	1[a]
Common Subjects		57

Common subjects are based on the subject specifications for the residual and random effects and are used to chunk the data for better performance.

a. This is the number of columns per common subject.

Residual Effect

Residual Effect	Estimate	Std. Error	Z	Sig.	95% Confidence Interval	
					Lower	Upper
Variance	1.000	.	.	.	.	.

Covariance Structure: Scaled Identity

Subject Specification: (None)

Random Effect

Random Effect Covariance	Estimate	Std. Error	Z	Sig.	95% Confidence Interval	
					Lower	Upper
Var(Intercept)	4.352E-12[a]	.	.	.	.	.

Covariance Structure: Variance components

Subject Specification: companyId15

a. This parameter is redundant.

Random Effects Parameters

Empirical Best Linear Unbiased Predictions[a]

company_code	Parameter	Prediction	Std. Error	df	Sig.	95% Confidence Interval	
						Lower Bound	Upper Bound

2	Intercept	-4.984E-12	2.086E-6	1361	1.000	-4.092E-6	4.092E-6
4	Intercept	-4.309E-12	2.086E-6	1361	1.000	-4.092E-6	4.092E-6
6	Intercept	-1.445E-12	2.086E-6	1361	1.000	-4.092E-6	4.092E-6
22	Intercept	-8.646E-13	2.086E-6	1361	1.000	-4.092E-6	4.092E-6
24	Intercept	-7.522E-12	2.086E-6	1361	1.000	-4.092E-6	4.092E-6
30	Intercept	1.900E-12	2.086E-6	1361	1.000	-4.092E-6	4.092E-6
36	Intercept	5.660E-14	2.086E-6	1361	1.000	-4.092E-6	4.092E-6
40	Intercept	-4.501E-12	2.086E-6	1361	1.000	-4.092E-6	4.092E-6
50	Intercept	-1.426E-12	2.086E-6	1361	1.000	-4.092E-6	4.092E-6
61	Intercept	8.932E-13	2.086E-6	1361	1.000	-4.092E-6	4.092E-6
65	Intercept	4.732E-12	2.086E-6	1361	1.000	-4.092E-6	4.092E-6
68	Intercept	1.340E-12	2.086E-6	1361	1.000	-4.092E-6	4.092E-6
69	Intercept	-3.229E-12	2.086E-6	1361	1.000	-4.092E-6	4.092E-6
71	Intercept	8.348E-12	2.086E-6	1361	1.000	-4.092E-6	4.092E-6
72	Intercept	-3.040E-12	2.086E-6	1361	1.000	-4.092E-6	4.092E-6
74	Intercept	4.240E-12	2.086E-6	1361	1.000	-4.092E-6	4.092E-6
75	Intercept	-4.661E-12	2.086E-6	1361	1.000	-4.092E-6	4.092E-6
80	Intercept	-4.890E-12	2.086E-6	1361	1.000	-4.092E-6	4.092E-6
81	Intercept	4.066E-12	2.086E-6	1361	1.000	-4.092E-6	4.092E-6
83	Intercept	-4.177E-12	2.086E-6	1361	1.000	-4.092E-6	4.092E-6
84	Intercept	-1.305E-12	2.086E-6	1361	1.000	-4.092E-6	4.092E-6
92	Intercept	-5.459E-12	2.086E-6	1361	1.000	-4.092E-6	4.092E-6
93	Intercept	2.388E-12	2.086E-6	1361	1.000	-4.092E-6	4.092E-6
98	Intercept	-9.799E-12	2.086E-6	1361	1.000	-4.092E-6	4.092E-6
100	Intercept	-4.041E-11	2.086E-6	1361	1.000	-4.092E-6	4.092E-6
106	Intercept	-4.584E-12	2.086E-6	1361	1.000	-4.092E-6	4.092E-6

107	Intercept	-2.501E-12	2.086E-6	1361	1.000	-4.092E-6	4.092E-6
108	Intercept	-7.807E-12	2.086E-6	1361	1.000	-4.092E-6	4.092E-6
110	Intercept	-7.086E-13	2.086E-6	1361	1.000	-4.092E-6	4.092E-6
114	Intercept	-9.717E-13	2.086E-6	1361	1.000	-4.092E-6	4.092E-6
116	Intercept	3.267E-12	2.086E-6	1361	1.000	-4.092E-6	4.092E-6
122	Intercept	1.446E-11	2.086E-6	1361	1.000	-4.092E-6	4.092E-6
125	Intercept	1.133E-11	2.086E-6	1361	1.000	-4.092E-6	4.092E-6
142	Intercept	-1.592E-11	2.086E-6	1361	1.000	-4.092E-6	4.092E-6
144	Intercept	-8.437E-12	2.086E-6	1361	1.000	-4.092E-6	4.092E-6
145	Intercept	-3.544E-12	2.086E-6	1361	1.000	-4.092E-6	4.092E-6
152	Intercept	1.489E-11	2.086E-6	1361	1.000	-4.092E-6	4.092E-6
154	Intercept	4.817E-12	2.086E-6	1361	1.000	-4.092E-6	4.092E-6
156	Intercept	9.921E-12	2.086E-6	1361	1.000	-4.092E-6	4.092E-6
162	Intercept	3.741E-12	2.086E-6	1361	1.000	-4.092E-6	4.092E-6
165	Intercept	2.158E-12	2.086E-6	1361	1.000	-4.092E-6	4.092E-6
166	Intercept	-5.576E-13	2.086E-6	1361	1.000	-4.092E-6	4.092E-6
169	Intercept	5.876E-12	2.086E-6	1361	1.000	-4.092E-6	4.092E-6
170	Intercept	-4.096E-12	2.086E-6	1361	1.000	-4.092E-6	4.092E-6
173	Intercept	4.782E-12	2.086E-6	1361	1.000	-4.092E-6	4.092E-6
174	Intercept	8.814E-12	2.086E-6	1361	1.000	-4.092E-6	4.092E-6
178	Intercept	2.123E-12	2.086E-6	1361	1.000	-4.092E-6	4.092E-6
181	Intercept	9.020E-12	2.086E-6	1361	1.000	-4.092E-6	4.092E-6
192	Intercept	-5.686E-12	2.086E-6	1361	1.000	-4.092E-6	4.092E-6
202	Intercept	1.152E-11	2.086E-6	1361	1.000	-4.092E-6	4.092E-6
204	Intercept	1.935E-11	2.086E-6	1361	1.000	-4.092E-6	4.092E-6
205	Intercept	-5.678E-12	2.086E-6	1361	1.000	-4.092E-6	4.092E-6

206	Intercept	8.503E-13	2.086E-6	1361	1.000	-4.092E-6	4.092E-6
209	Intercept	1.177E-11	2.086E-6	1361	1.000	-4.092E-6	4.092E-6
217	Intercept	-7.062E-12	2.086E-6	1361	1.000	-4.092E-6	4.092E-6
226	Intercept	3.813E-12	2.086E-6	1361	1.000	-4.092E-6	4.092E-6
228	Intercept	-8.903E-13	2.086E-6	1361	1.000	-4.092E-6	4.092E-6

a. Target: Phy16 > 2 = 1

```
AGGREGATE
  /OUTFILE=* MODE=ADDVARIABLES OVERWRITE = YES
  /BREAK=companyId15
  /choicem4=MEAN(choice_freq)
  /bullym4=MEAN(bullying_freq)
  /timem4=MEAN(time_pressure_freq)
  /dutiesm4=MEAN(duties_freq).

COMPUTE choicecwc4 = choice_freq - choicem4.
COMPUTE bullycwc4 = bullying_freq - bullym4.
COMPUTE timecwc4 = time_pressure_freq - timem4.
COMPUTE dutiescwc4 = duties_freq - dutiesm4.

COMPUTE fac4choi = fac1_1 * choicecwc4.
COMPUTE fac4bull = fac1_1 * bullycwc4.
COMPUTE fac4time = fac1_1 *  timecwc4.
COMPUTE fac4duti = fac1_1 * dutiescwc4.

GENLINMIXED
  /DATA_STRUCTURE SUBJECTS=companyId15
  /FIELDS TARGET=phycat16 TRIALS=NONE OFFSET=NONE
  /TARGET_OPTIONS DISTRIBUTION=BINOMIAL LINK=LOGIT
  /FIXED  EFFECTS=physical_health kess_t15 choicecwc4 choicem4 fac4choi
  bullying_freq time_pressure_freq duties_freq
    FAC1_1 USE_INTERCEPT=TRUE
  /RANDOM USE_INTERCEPT=TRUE SUBJECTS=companyId15 COVARIANCE_TYPE=VARIANCE_COMPONENTS SOLUTION=TRUE
  /BUILD_OPTIONS TARGET_CATEGORY_ORDER=DESCENDING INPUTS_CATEGORY_ORDER=DESCENDING
    MAX_ITERATIONS=100 CONFIDENCE_LEVEL=95 DF_METHOD=RESIDUAL COVB=ROBUST PCONVERGE=0.000001(ABSOLUTE)
    SCORING=0 SINGULAR=0.000000000001
  /EMMEANS_OPTIONS SCALE=ORIGINAL PADJUST=LSD.
```

Generalized Linear Mixed Models

Notes

Output Created		16-AUG-2021 11:08:00
Comments		
Input	Data	C:\Users\jwb12ppu\Documents\Kevin\MPlus backup\Multicomponent health and wellbeing v2\Multicomponent for Mplus cat.sav
	Active Dataset	DataSet2
	Filter	kescat15 = 0 (FILTER)
	Weight	<none>
	Split File	<none>
	N of Rows in Working Data File	1422

Syntax	GENLINMIXED<br>/DATA_STRUCTURE<br>SUBJECTS=companyId15<br>/FIELDS TARGET=phycat16<br>TRIALS=NONE OFFSET=NONE<br>/TARGET_OPTIONS<br>DISTRIBUTION=BINOMIAL<br>LINK=LOGIT<br>/FIXED<br>EFFECTS=physical_health<br>kess_t15 choicecwc4 choicem4<br>fac4choi<br>bullying_freq time_pressure_freq<br>duties_freq<br>FAC1_1<br>USE_INTERCEPT=TRUE<br>/RANDOM<br>USE_INTERCEPT=TRUE<br>SUBJECTS=companyId15<br>COVARIANCE_TYPE=VARIANCE<br>_COMPONENTS<br>SOLUTION=TRUE<br>/BUILD_OPTIONS<br>TARGET_CATEGORY_ORDER=<br>DESCENDING<br>INPUTS_CATEGORY_ORDER=D<br>ESCENDING

		MAX_ITERATIONS=100 CONFIDENCE_LEVEL=95 DF_METHOD=RESIDUAL COVB=ROBUST PCONVERGE=0.000001(ABSOLUTE) SCORING=0 SINGULAR=0.000000000001 /EMMEANS_OPTIONS SCALE=ORIGINAL PADJUST=LSD.
Resources	Processor Time	00:00:01.86
	Elapsed Time	00:00:01.67

Warnings

glmm: One or more records are not used in the analysis because they have one or more fields with invalid or missing values.
glmm: Valid values for events (target) and trials variables are non-negative and positive integers respectively, and the number of trials cannot be less than the number of events.
glmm: The final Hessian matrix is not positive definite although all convergence criteria are satisfied. The procedure continues despite this warning. Subsequent results produced are based on the last iteration. Validity of the model fit is uncertain.

Case Processing Summary

	N	Percent
Included	1370	96.3%
Excluded	52	3.7%
Total	1422	100.0%

Model Summary

Target		Phy16 > 2 = 1
Probability Distribution		Binomial
Link Function		Logit
Information Criterion	Akaike Corrected	6102.163
	Bayesian	6107.375

Information criteria are based on the -2 log likelihood (6100.160) and are used to compare models. Models with smaller information criterion values fit better.

Data Structure[a]

	Subjects company_code	Target Phy16 > 2 = 1
Data for First Subject	2	1.00
	2	.00
	2	1.00
	2	.00
	2	1.00

	2	1.00
	2	.00
	2	1.00
	2	.00
	2	1.00
Total Number of Levels	57	

Only the first 10 records are displayed.

a. Target: Phy16 > 2 = 1

Classification
Overall Percent Correct = 63.6%[a]

Observed		Predicted 1.00	.00
1.00	Count	857	13
	% within Observed	98.5%	1.5%
.00	Count	486	14
	% within Observed	97.2%	2.8%

a. Target: Phy16 > 2 = 1

Fixed Effects[a]

Source	F	df1	df2	Sig.
Corrected Model	15.733	9	1360	.000
physical_health	31.945	1	1360	.000
kess_t15	11.434	1	1360	.001

choicecwc4	3.080	1	1360	.079
choicem4	2.040	1	1360	.153
fac4choi	1.087	1	1360	.297
bullying_freq	1.031	1	1360	.310
time_pressure_freq	.004	1	1360	.950
duties_freq	.654	1	1360	.419
FAC1_1	3.000	1	1360	.084

Probability distribution: Binomial

Link function: Logit

a. Target: Phy16 > 2 = 1

Fixed Effects

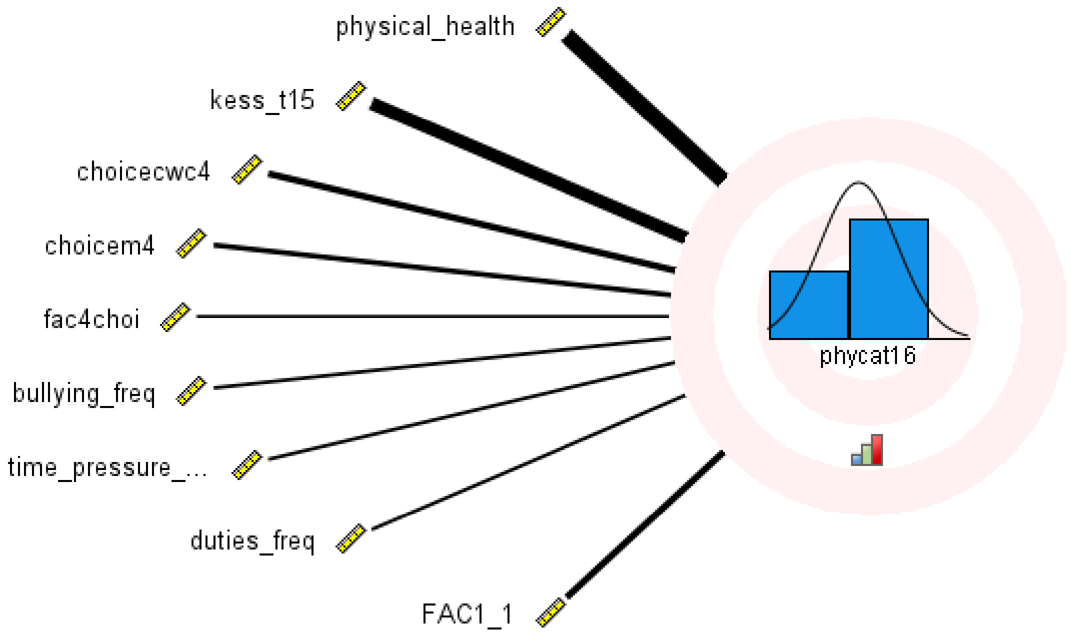

Fixed Coefficients[a]

Model Term	Coefficient	Std. Error	t	Sig.	95% Confidence Interval		Exp(Coefficient)	95% Confidence Interval for Exp(Coefficient)	
					Lower	Upper		Lower	Upper
Intercept	-3.717	.9522	-3.904	.000	-5.585	-1.849	.024	.004	.157
physical_health	1.421	.2514	5.652	.000	.928	1.914	4.141	2.529	6.780
kess_t15	.272	.0803	3.381	.001	.114	.429	1.312	1.121	1.536
choicecwc4	-.066	.0376	-1.755	.079	-.140	.008	.936	.870	1.008
choicem4	-.229	.1600	-1.428	.153	-.542	.085	.796	.581	1.089
fac4choi	.042	.0407	1.043	.297	-.037	.122	1.043	.963	1.130
bullying_freq	.093	.0912	1.015	.310	-.086	.272	1.097	.917	1.312
time_pressure_freq	-.004	.0607	-.062	.950	-.123	.115	.996	.884	1.122
duties_freq	.037	.0460	.809	.419	-.053	.127	1.038	.948	1.136
FAC1_1	-.082	.0471	-1.732	.084	-.174	.011	.922	.840	1.011

Probability distribution: Binomial

Link function: Logit

a. Target: Phy16 > 2 = 1

Fixed Coefficients

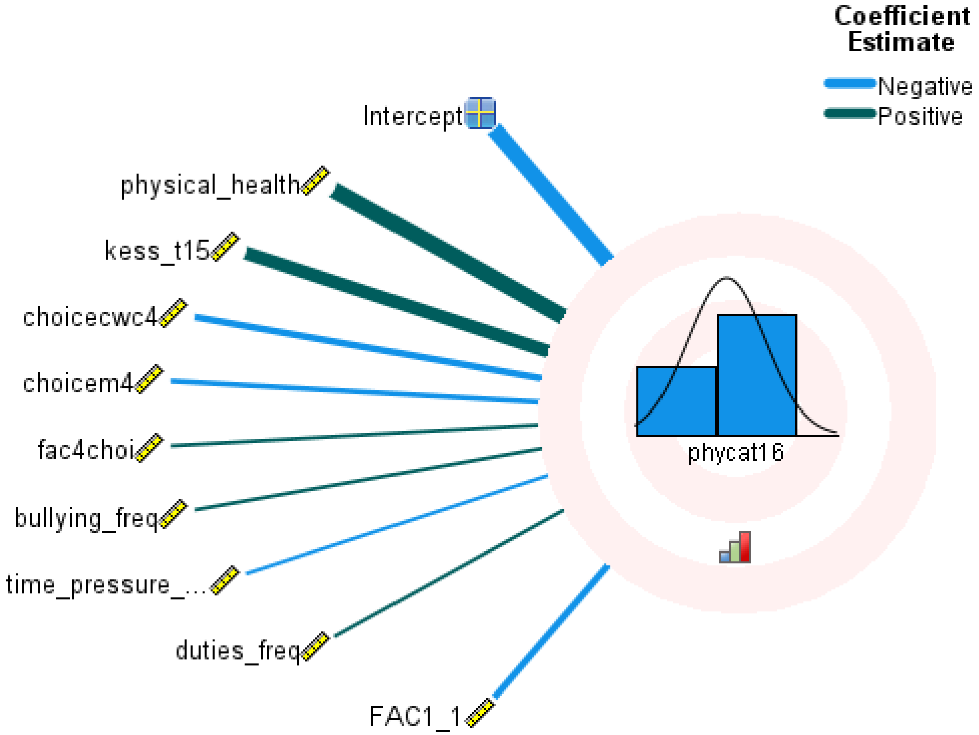

Random Effect Covariances

Random Effect Block 1

Random Effect Block	Intercept
Intercept	.000

Covariance Structure: Variance components

Subject Specification: companyId15

Covariance Parameters

Covariance Parameters Summary

Covariance Parameters	Residual Effect	0
	Random Effects	1
Design Matrix Columns	Fixed Effects	10
	Random Effects	1[a]
Common Subjects		57

Common subjects are based on the subject specifications for the residual and random effects and are used to chunk the data for better performance.

a. This is the number of columns per common subject.

Residual Effect

Residual Effect	Estimate	Std. Error	Z	Sig.	95% Confidence Interval	
					Lower	Upper
Variance	1.000	.	.	.	.	.

Covariance Structure: Scaled Identity

Subject Specification: (None)

Random Effect

Random Effect Covariance	Estimate	Std. Error	Z	Sig.	95% Confidence Interval	
					Lower	Upper
Var(Intercept)	2.856E-10[a]	.	.	.	.	.

Covariance Structure: Variance components

Subject Specification: companyId15

a. This parameter is redundant.

Random Effects Parameters

Empirical Best Linear Unbiased Predictions[a]

company_code	Parameter	Prediction	Std. Error	df	Sig.	95% Confidence Interval	
						Lower Bound	Upper Bound

2	Intercept	-2.878E-10	1.690E-5	1360	1.000	-3.315E-5	3.315E-5
4	Intercept	-3.161E-10	1.690E-5	1360	1.000	-3.315E-5	3.315E-5
6	Intercept	-2.180E-10	1.690E-5	1360	1.000	-3.315E-5	3.315E-5
22	Intercept	-7.584E-11	1.690E-5	1360	1.000	-3.315E-5	3.315E-5
24	Intercept	-3.422E-10	1.690E-5	1360	1.000	-3.315E-5	3.315E-5
30	Intercept	1.047E-10	1.690E-5	1360	1.000	-3.315E-5	3.315E-5
36	Intercept	1.419E-10	1.690E-5	1360	1.000	-3.315E-5	3.315E-5
40	Intercept	-3.031E-10	1.690E-5	1360	1.000	-3.315E-5	3.315E-5
50	Intercept	-7.151E-11	1.690E-5	1360	1.000	-3.315E-5	3.315E-5
61	Intercept	1.025E-10	1.690E-5	1360	1.000	-3.315E-5	3.315E-5
65	Intercept	2.843E-10	1.690E-5	1360	1.000	-3.315E-5	3.315E-5
68	Intercept	1.577E-10	1.690E-5	1360	1.000	-3.315E-5	3.315E-5
69	Intercept	-1.289E-10	1.690E-5	1360	1.000	-3.315E-5	3.315E-5
71	Intercept	5.630E-10	1.690E-5	1360	1.000	-3.315E-5	3.315E-5
72	Intercept	-2.113E-10	1.690E-5	1360	1.000	-3.315E-5	3.315E-5
74	Intercept	2.639E-10	1.690E-5	1360	1.000	-3.315E-5	3.315E-5
75	Intercept	-3.233E-10	1.690E-5	1360	1.000	-3.315E-5	3.315E-5
80	Intercept	-3.326E-10	1.690E-5	1360	1.000	-3.315E-5	3.315E-5
81	Intercept	3.477E-10	1.690E-5	1360	1.000	-3.315E-5	3.315E-5
83	Intercept	-2.202E-10	1.690E-5	1360	1.000	-3.315E-5	3.315E-5
84	Intercept	-5.389E-11	1.690E-5	1360	1.000	-3.315E-5	3.315E-5
92	Intercept	-3.497E-10	1.690E-5	1360	1.000	-3.315E-5	3.315E-5
93	Intercept	1.765E-10	1.690E-5	1360	1.000	-3.315E-5	3.315E-5
98	Intercept	-6.771E-10	1.690E-5	1360	1.000	-3.315E-5	3.315E-5
100	Intercept	-2.745E-9	1.690E-5	1360	1.000	-3.316E-5	3.315E-5
106	Intercept	-2.569E-10	1.690E-5	1360	1.000	-3.315E-5	3.315E-5

107	Intercept	-1.204E-10	1.690E-5	1360	1.000	-3.315E-5	3.315E-5
108	Intercept	-5.826E-10	1.690E-5	1360	1.000	-3.315E-5	3.315E-5
110	Intercept	-5.152E-11	1.690E-5	1360	1.000	-3.315E-5	3.315E-5
114	Intercept	4.740E-13	1.690E-5	1360	1.000	-3.315E-5	3.315E-5
116	Intercept	2.615E-10	1.690E-5	1360	1.000	-3.315E-5	3.315E-5
122	Intercept	9.792E-10	1.690E-5	1360	1.000	-3.315E-5	3.315E-5
125	Intercept	6.325E-10	1.690E-5	1360	1.000	-3.315E-5	3.315E-5
142	Intercept	-9.742E-10	1.690E-5	1360	1.000	-3.315E-5	3.315E-5
144	Intercept	-5.793E-10	1.690E-5	1360	1.000	-3.315E-5	3.315E-5
145	Intercept	-1.352E-10	1.690E-5	1360	1.000	-3.315E-5	3.315E-5
152	Intercept	9.479E-10	1.690E-5	1360	1.000	-3.315E-5	3.315E-5
154	Intercept	3.943E-10	1.690E-5	1360	1.000	-3.315E-5	3.315E-5
156	Intercept	5.850E-10	1.690E-5	1360	1.000	-3.315E-5	3.315E-5
162	Intercept	2.566E-10	1.690E-5	1360	1.000	-3.315E-5	3.315E-5
165	Intercept	1.260E-10	1.690E-5	1360	1.000	-3.315E-5	3.315E-5
166	Intercept	-4.072E-11	1.690E-5	1360	1.000	-3.315E-5	3.315E-5
169	Intercept	2.708E-10	1.690E-5	1360	1.000	-3.315E-5	3.315E-5
170	Intercept	-1.890E-10	1.690E-5	1360	1.000	-3.315E-5	3.315E-5
173	Intercept	3.227E-10	1.690E-5	1360	1.000	-3.315E-5	3.315E-5
174	Intercept	5.733E-10	1.690E-5	1360	1.000	-3.315E-5	3.315E-5
178	Intercept	2.380E-10	1.690E-5	1360	1.000	-3.315E-5	3.315E-5
181	Intercept	6.052E-10	1.690E-5	1360	1.000	-3.315E-5	3.315E-5
192	Intercept	-3.938E-10	1.690E-5	1360	1.000	-3.315E-5	3.315E-5
202	Intercept	8.085E-10	1.690E-5	1360	1.000	-3.315E-5	3.315E-5
204	Intercept	1.072E-9	1.690E-5	1360	1.000	-3.315E-5	3.315E-5
205	Intercept	-3.718E-10	1.690E-5	1360	1.000	-3.315E-5	3.315E-5

206	Intercept	6.845E-11	1.690E-5	1360	1.000	-3.315E-5	3.315E-5
209	Intercept	6.221E-10	1.690E-5	1360	1.000	-3.315E-5	3.315E-5
217	Intercept	-6.929E-10	1.690E-5	1360	1.000	-3.315E-5	3.315E-5
226	Intercept	1.905E-10	1.690E-5	1360	1.000	-3.315E-5	3.315E-5
228	Intercept	-5.186E-11	1.690E-5	1360	1.000	-3.315E-5	3.315E-5

a. Target: Phy16 > 2 = 1

```
GENLINMIXED
  /DATA_STRUCTURE SUBJECTS=companyId15
  /FIELDS TARGET=phycat16 TRIALS=NONE OFFSET=NONE
  /TARGET_OPTIONS DISTRIBUTION=BINOMIAL LINK=LOGIT
  /FIXED  EFFECTS=physical_health kess_t15 choice_freq bullycwc4 bullym4 fac4bull
   time_pressure_freq duties_freq
    FAC1_1 USE_INTERCEPT=TRUE
  /RANDOM USE_INTERCEPT=TRUE SUBJECTS=companyId15 COVARIANCE_TYPE=VARIANCE_COMPONENTS SOLUTION=TRUE
  /BUILD_OPTIONS TARGET_CATEGORY_ORDER=DESCENDING INPUTS_CATEGORY_ORDER=DESCENDING
    MAX_ITERATIONS=100 CONFIDENCE_LEVEL=95 DF_METHOD=RESIDUAL COVB=ROBUST PCONVERGE=0.000001(ABSOLUTE)
    SCORING=0 SINGULAR=0.000000000001
  /EMMEANS_OPTIONS SCALE=ORIGINAL PADJUST=LSD.
```

Generalized Linear Mixed Models

Notes

Output Created	16-AUG-2021 11:08:02
Comments	

Input	Data	C:\Users\jwb12ppu\Documents\Kevin\MPlus backup\Multicomponent health and wellbeing v2\Multicomponent for Mplus cat.sav
	Active Dataset	DataSet2
	Filter	kescat15 = 0 (FILTER)
	Weight	<none>
	Split File	<none>
	N of Rows in Working Data File	1422

Syntax	GENLINMIXED<br>/DATA_STRUCTURE<br>SUBJECTS=companyId15<br>/FIELDS TARGET=phycat16<br>TRIALS=NONE OFFSET=NONE<br>/TARGET_OPTIONS<br>DISTRIBUTION=BINOMIAL<br>LINK=LOGIT<br>/FIXED<br>EFFECTS=physical_health<br>kess_t15 choice_freq bullycwc4<br>bullym4 fac4bull<br>time_pressure_freq duties_freq<br>FAC1_1<br>USE_INTERCEPT=TRUE<br>/RANDOM<br>USE_INTERCEPT=TRUE<br>SUBJECTS=companyId15<br>COVARIANCE_TYPE=VARIANCE<br>_COMPONENTS<br>SOLUTION=TRUE<br>/BUILD_OPTIONS<br>TARGET_CATEGORY_ORDER=<br>DESCENDING<br>INPUTS_CATEGORY_ORDER=D<br>ESCENDING<br>MAX_ITERATIONS=100<br>CONFIDENCE_LEVEL=95

		DF_METHOD=RESIDUAL COVB=ROBUST PCONVERGE=0.000001(ABSOLUTE) SCORING=0 SINGULAR=0.000000000001 /EMMEANS_OPTIONS SCALE=ORIGINAL PADJUST=LSD.
Resources	Processor Time	00:00:01.44
	Elapsed Time	00:00:01.44

Warnings

glmm: One or more records are not used in the analysis because they have one or more fields with invalid or missing values.
glmm: Valid values for events (target) and trials variables are non-negative and positive integers respectively, and the number of trials cannot be less than the number of events.
glmm: The final Hessian matrix is not positive definite although all convergence criteria are satisfied. The procedure continues despite this warning. Subsequent results produced are based on the last iteration. Validity of the model fit is uncertain.

Case Processing Summary

	N	Percent
Included	1370	96.3%
Excluded	52	3.7%
Total	1422	100.0%

Model Summary

Target		Phy16 > 2 = 1
Probability Distribution		Binomial
Link Function		Logit
Information Criterion	Akaike Corrected	6104.947
	Bayesian	6110.160

Information criteria are based on the -2 log likelihood (6102.944) and are used to compare models. Models with smaller information criterion values fit better.

Data Structure[a]

	Subjects company_code	Target Phy16 > 2 = 1
Data for First Subject	2	1.00
	2	.00
	2	1.00
	2	.00
	2	1.00
	2	1.00
	2	.00

	2	1.00
	2	.00
	2	1.00
Total Number of Levels	57	

Only the first 10 records are displayed.

a. Target: Phy16 > 2 = 1

Classification

Overall Percent Correct = 63.4%[a]

Observed		Predicted 1.00	.00
1.00	Count	855	15
	% within Observed	98.3%	1.7%
.00	Count	486	14
	% within Observed	97.2%	2.8%

a. Target: Phy16 > 2 = 1

Fixed Effects[a]

Source	F	df1	df2	Sig.
Corrected Model	14.588	9	1360	.000
physical_health	32.081	1	1360	.000
kess_t15	11.175	1	1360	.001
choice_freq	4.727	1	1360	.030
bullycwc4	.992	1	1360	.319

bullym4	1.044	1	1360	.307
fac4bull	1.415	1	1360	.234
time_pressure_freq	.001	1	1360	.976
duties_freq	.717	1	1360	.397
FAC1_1	3.525	1	1360	.061

Probability distribution: Binomial

Link function: Logit

a. Target: Phy16 > 2 = 1

Fixed Effects

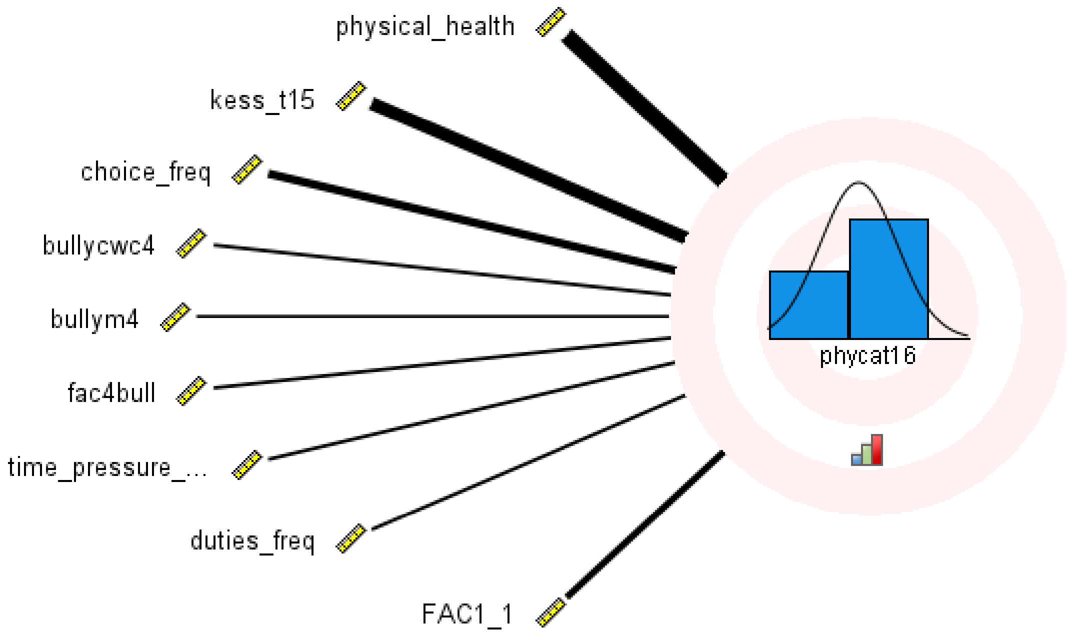

Fixed Coefficients[a]

Model Term	Coefficient	Std. Error	t	Sig.	95% Confidence Interval		Exp(Coefficient)	95% Confidence Interval for Exp(Coefficient)	
					Lower	Upper		Lower	Upper
Intercept	-4.429	.7843	-5.647	.000	-5.967	-2.890	.012	.003	.056
physical_health	1.420	.2507	5.664	.000	.928	1.912	4.138	2.530	6.767
kess_t15	.271	.0812	3.343	.001	.112	.431	1.312	1.119	1.538
choice_freq	-.077	.0356	-2.174	.030	-.147	-.008	.926	.863	.992
bullycwc4	.094	.0946	.996	.319	-.091	.280	1.099	.913	1.323
bullym4	.298	.2918	1.022	.307	-.274	.871	1.347	.760	2.388
fac4bull	-.120	.1006	-1.190	.234	-.317	.078	.887	.728	1.081
time_pressure_freq	-.002	.0589	-.030	.976	-.117	.114	.998	.889	1.120
duties_freq	.038	.0454	.847	.397	-.051	.127	1.039	.951	1.136
FAC1_1	-.088	.0468	-1.878	.061	-.179	.004	.916	.836	1.004

Probability distribution: Binomial

Link function: Logit

a. Target: Phy16 > 2 = 1

Fixed Coefficients

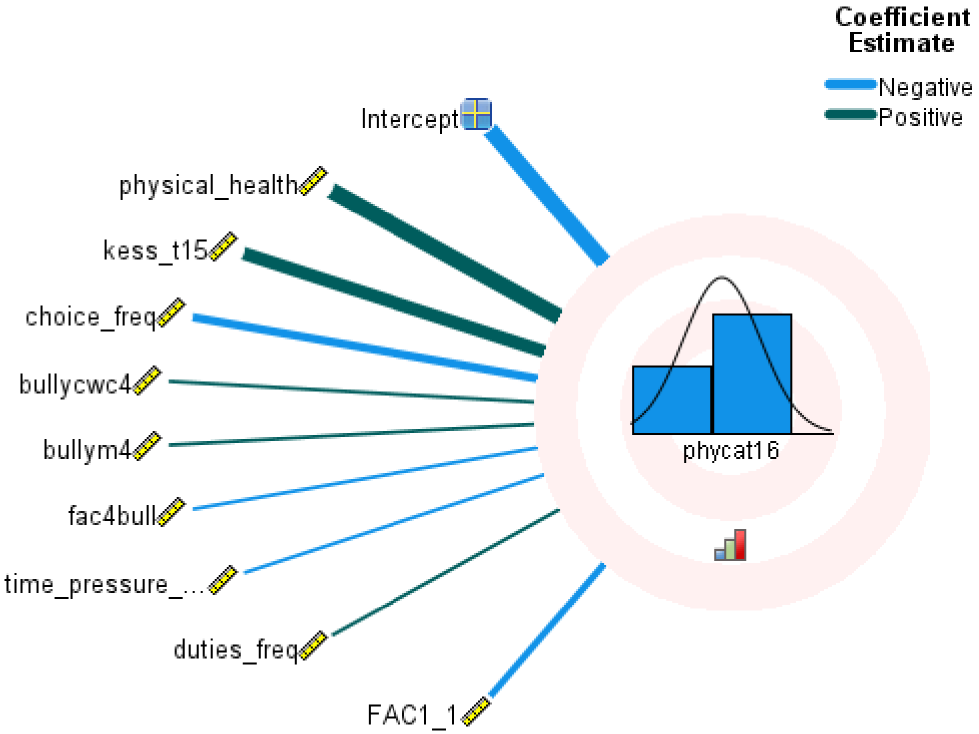

Random Effect Covariances

Random Effect Block 1

Random Effect Block	Intercept
Intercept	.000

Covariance Structure: Variance components

Subject Specification: companyId15

Covariance Parameters

Covariance Parameters Summary

Covariance Parameters	Residual Effect	0
	Random Effects	1
Design Matrix Columns	Fixed Effects	10
	Random Effects	1[a]
Common Subjects		57

Common subjects are based on the subject specifications for the residual and random effects and are used to chunk the data for better performance.

a. This is the number of columns per common subject.

Residual Effect

Residual Effect	Estimate	Std. Error	Z	Sig.	95% Confidence Interval	
					Lower	Upper
Variance	1.000	.	.	.	.	.

Covariance Structure: Scaled Identity

Subject Specification: (None)

Random Effect

Random Effect Covariance	Estimate	Std. Error	Z	Sig.	95% Confidence Interval	
					Lower	Upper
Var(Intercept)	2.950E-11[a]	.	.	.	.	.

Covariance Structure: Variance components

Subject Specification: companyId15

a. This parameter is redundant.

Random Effects Parameters

Empirical Best Linear Unbiased Predictions[a]

company_code	Parameter	Prediction	Std. Error	df	Sig.	95% Confidence Interval	
						Lower Bound	Upper Bound

2	Intercept	-3.296E-11	5.431E-6	1360	1.000	-1.065E-5	1.065E-5
4	Intercept	-3.219E-11	5.431E-6	1360	1.000	-1.065E-5	1.065E-5
6	Intercept	-2.539E-11	5.431E-6	1360	1.000	-1.065E-5	1.065E-5
22	Intercept	-1.341E-11	5.431E-6	1360	1.000	-1.065E-5	1.065E-5
24	Intercept	-4.419E-11	5.431E-6	1360	1.000	-1.065E-5	1.065E-5
30	Intercept	1.313E-11	5.431E-6	1360	1.000	-1.065E-5	1.065E-5
36	Intercept	6.235E-12	5.431E-6	1360	1.000	-1.065E-5	1.065E-5
40	Intercept	-2.868E-11	5.431E-6	1360	1.000	-1.065E-5	1.065E-5
50	Intercept	-8.218E-12	5.431E-6	1360	1.000	-1.065E-5	1.065E-5
61	Intercept	4.300E-12	5.431E-6	1360	1.000	-1.065E-5	1.065E-5
65	Intercept	3.059E-11	5.431E-6	1360	1.000	-1.065E-5	1.065E-5
68	Intercept	1.481E-11	5.431E-6	1360	1.000	-1.065E-5	1.065E-5
69	Intercept	-1.606E-11	5.431E-6	1360	1.000	-1.065E-5	1.065E-5
71	Intercept	5.705E-11	5.431E-6	1360	1.000	-1.065E-5	1.065E-5
72	Intercept	-2.354E-11	5.431E-6	1360	1.000	-1.065E-5	1.065E-5
74	Intercept	2.944E-11	5.431E-6	1360	1.000	-1.065E-5	1.065E-5
75	Intercept	-2.989E-11	5.431E-6	1360	1.000	-1.065E-5	1.065E-5
80	Intercept	-3.588E-11	5.431E-6	1360	1.000	-1.065E-5	1.065E-5
81	Intercept	2.961E-11	5.431E-6	1360	1.000	-1.065E-5	1.065E-5
83	Intercept	-2.881E-11	5.431E-6	1360	1.000	-1.065E-5	1.065E-5
84	Intercept	-6.387E-12	5.431E-6	1360	1.000	-1.065E-5	1.065E-5
92	Intercept	-3.517E-11	5.431E-6	1360	1.000	-1.065E-5	1.065E-5
93	Intercept	1.068E-11	5.431E-6	1360	1.000	-1.065E-5	1.065E-5
98	Intercept	-6.545E-11	5.431E-6	1360	1.000	-1.065E-5	1.065E-5
100	Intercept	-2.492E-10	5.431E-6	1360	1.000	-1.065E-5	1.065E-5
106	Intercept	-2.763E-11	5.431E-6	1360	1.000	-1.065E-5	1.065E-5

107	Intercept	-1.302E-11	5.431E-6	1360	1.000	-1.065E-5	1.065E-5
108	Intercept	-5.710E-11	5.431E-6	1360	1.000	-1.065E-5	1.065E-5
110	Intercept	-4.057E-11	5.431E-6	1360	1.000	-1.065E-5	1.065E-5
114	Intercept	-1.221E-12	5.431E-6	1360	1.000	-1.065E-5	1.065E-5
116	Intercept	2.816E-11	5.431E-6	1360	1.000	-1.065E-5	1.065E-5
122	Intercept	1.090E-10	5.431E-6	1360	1.000	-1.065E-5	1.065E-5
125	Intercept	6.609E-11	5.431E-6	1360	1.000	-1.065E-5	1.065E-5
142	Intercept	-1.030E-10	5.431E-6	1360	1.000	-1.065E-5	1.065E-5
144	Intercept	-5.712E-11	5.431E-6	1360	1.000	-1.065E-5	1.065E-5
145	Intercept	-1.676E-11	5.431E-6	1360	1.000	-1.065E-5	1.065E-5
152	Intercept	9.701E-11	5.431E-6	1360	1.000	-1.065E-5	1.065E-5
154	Intercept	3.257E-11	5.431E-6	1360	1.000	-1.065E-5	1.065E-5
156	Intercept	6.727E-11	5.431E-6	1360	1.000	-1.065E-5	1.065E-5
162	Intercept	1.895E-11	5.431E-6	1360	1.000	-1.065E-5	1.065E-5
165	Intercept	1.081E-11	5.431E-6	1360	1.000	-1.065E-5	1.065E-5
166	Intercept	-2.442E-12	5.431E-6	1360	1.000	-1.065E-5	1.065E-5
169	Intercept	2.088E-11	5.431E-6	1360	1.000	-1.065E-5	1.065E-5
170	Intercept	-2.337E-11	5.431E-6	1360	1.000	-1.065E-5	1.065E-5
173	Intercept	3.526E-11	5.431E-6	1360	1.000	-1.065E-5	1.065E-5
174	Intercept	5.831E-11	5.431E-6	1360	1.000	-1.065E-5	1.065E-5
178	Intercept	2.321E-11	5.431E-6	1360	1.000	-1.065E-5	1.065E-5
181	Intercept	5.925E-11	5.431E-6	1360	1.000	-1.065E-5	1.065E-5
192	Intercept	-4.006E-11	5.431E-6	1360	1.000	-1.065E-5	1.065E-5
202	Intercept	8.750E-11	5.431E-6	1360	1.000	-1.065E-5	1.065E-5
204	Intercept	1.095E-10	5.431E-6	1360	1.000	-1.065E-5	1.065E-5
205	Intercept	-3.890E-11	5.431E-6	1360	1.000	-1.065E-5	1.065E-5

206	Intercept	1.072E-12	5.431E-6	1360	1.000	-1.065E-5	1.065E-5
209	Intercept	9.074E-11	5.431E-6	1360	1.000	-1.065E-5	1.065E-5
217	Intercept	-3.623E-11	5.431E-6	1360	1.000	-1.065E-5	1.065E-5
226	Intercept	2.546E-11	5.431E-6	1360	1.000	-1.065E-5	1.065E-5
228	Intercept	-4.014E-12	5.431E-6	1360	1.000	-1.065E-5	1.065E-5

a. Target: Phy16 > 2 = 1

```
GENLINMIXED
  /DATA_STRUCTURE SUBJECTS=companyId15
  /FIELDS TARGET=phycat16 TRIALS=NONE OFFSET=NONE
  /TARGET_OPTIONS DISTRIBUTION=BINOMIAL LINK=LOGIT
  /FIXED  EFFECTS=physical_health kess_t15 choice_freq bullying_freq timecwc4 timem4 fac4time
  duties_freq
    FAC1_1 USE_INTERCEPT=TRUE
  /RANDOM USE_INTERCEPT=TRUE SUBJECTS=companyId15 COVARIANCE_TYPE=VARIANCE_COMPONENTS SOLUTION=TRUE
  /BUILD_OPTIONS TARGET_CATEGORY_ORDER=DESCENDING INPUTS_CATEGORY_ORDER=DESCENDING
    MAX_ITERATIONS=100 CONFIDENCE_LEVEL=95 DF_METHOD=RESIDUAL COVB=ROBUST PCONVERGE=0.000001(ABSOLUTE)
    SCORING=0 SINGULAR=0.000000000001
  /EMMEANS_OPTIONS SCALE=ORIGINAL PADJUST=LSD.
```

Generalized Linear Mixed Models

Notes

Output Created	16-AUG-2021 11:08:04
Comments	

Input	Data	C:\Users\jwb12ppu\Documents\Kevin\MPlus backup\Multicomponent health and wellbeing v2\Multicomponent for Mplus cat.sav
	Active Dataset	DataSet2
	Filter	kescat15 = 0 (FILTER)
	Weight	<none>
	Split File	<none>
	N of Rows in Working Data File	1422

Syntax	GENLINMIXED<br>/DATA_STRUCTURE SUBJECTS=companyId15<br>/FIELDS TARGET=phycat16 TRIALS=NONE OFFSET=NONE<br>/TARGET_OPTIONS DISTRIBUTION=BINOMIAL LINK=LOGIT<br>/FIXED EFFECTS=physical_health kess_t15 choice_freq bullying_freq timecwc4 timem4 fac4time<br>duties_freq<br>FAC1_1 USE_INTERCEPT=TRUE<br>/RANDOM USE_INTERCEPT=TRUE SUBJECTS=companyId15 COVARIANCE_TYPE=VARIANCE_COMPONENTS SOLUTION=TRUE<br>/BUILD_OPTIONS TARGET_CATEGORY_ORDER=DESCENDING INPUTS_CATEGORY_ORDER=DESCENDING<br>MAX_ITERATIONS=100 CONFIDENCE_LEVEL=95

		DF_METHOD=RESIDUAL COVB=ROBUST PCONVERGE=0.000001(ABSOLUTE) SCORING=0 SINGULAR=0.000000000001 /EMMEANS_OPTIONS SCALE=ORIGINAL PADJUST=LSD.
Resources	Processor Time	00:00:01.20
	Elapsed Time	00:00:01.21

Warnings

glmm: One or more records are not used in the analysis because they have one or more fields with invalid or missing values.
glmm: Valid values for events (target) and trials variables are non-negative and positive integers respectively, and the number of trials cannot be less than the number of events.
glmm: The final Hessian matrix is not positive definite although all convergence criteria are satisfied. The procedure continues despite this warning. Subsequent results produced are based on the last iteration. Validity of the model fit is uncertain.

Case Processing Summary

	N	Percent
Included	1370	96.3%
Excluded	52	3.7%
Total	1422	100.0%

Model Summary

Target		Phy16 > 2 = 1
Probability Distribution		Binomial
Link Function		Logit
Information Criterion	Akaike Corrected	6107.889
	Bayesian	6113.101

Information criteria are based on the -2 log likelihood (6105.886) and are used to compare models. Models with smaller information criterion values fit better.

Data Structure[a]

	Subjects company_code	Target Phy16 > 2 = 1
Data for First Subject	2	1.00
	2	.00
	2	1.00
	2	.00
	2	1.00
	2	1.00
	2	.00

	2	1.00
	2	.00
	2	1.00
Total Number of Levels	57	

Only the first 10 records are displayed.

a. Target: Phy16 > 2 = 1

Classification
Overall Percent Correct = 63.8%[a]

Observed		Predicted 1.00	.00
1.00	Count	856	14
	% within Observed	98.4%	1.6%
.00	Count	482	18
	% within Observed	96.4%	3.6%

a. Target: Phy16 > 2 = 1

Fixed Effects[a]

Source	F	df1	df2	Sig.
Corrected Model	16.080	9	1360	.000
physical_health	33.070	1	1360	.000
kess_t15	11.596	1	1360	.001
choice_freq	4.810	1	1360	.028
bullying_freq	1.115	1	1360	.291

timecwc4	.006	1	1360	.939
timem4	.111	1	1360	.739
fac4time	.114	1	1360	.736
duties_freq	.764	1	1360	.382
FAC1_1	2.963	1	1360	.085

Probability distribution: Binomial

Link function: Logit

a. Target: Phy16 > 2 = 1

Fixed Effects

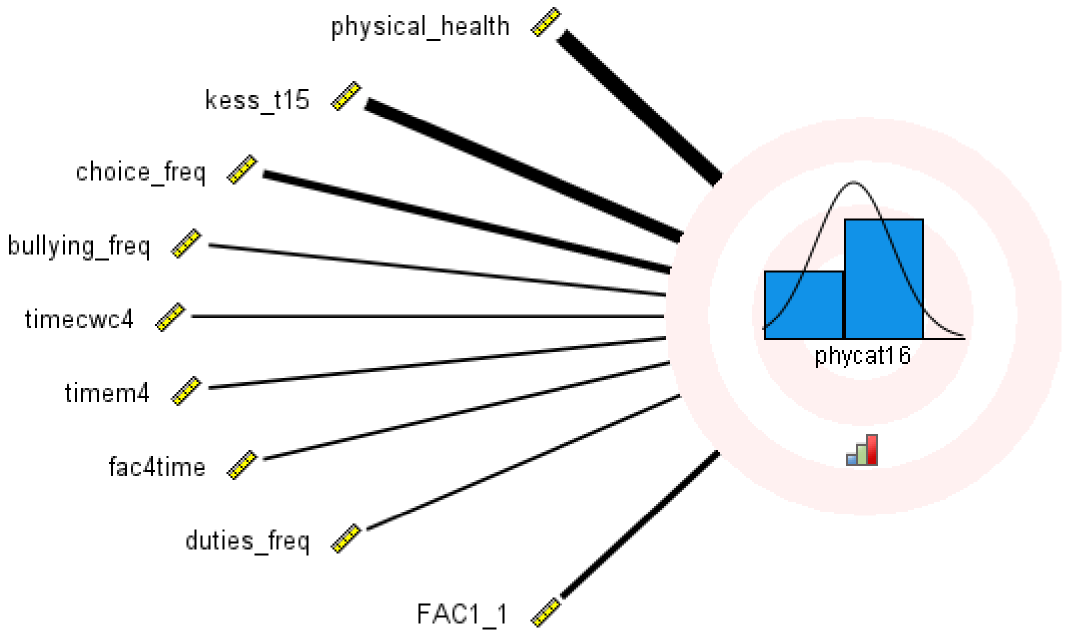

Fixed Coefficients[a]

Model Term	Coefficient	Std. Error	t	Sig.	95% Confidence Interval		Exp(Coefficient)	95% Confidence Interval for Exp(Coefficient)	
					Lower	Upper		Lower	Upper
Intercept	-4.382	.9653	-4.539	.000	-6.275	-2.488	.013	.002	.083
physical_health	1.429	.2485	5.751	.000	.941	1.916	4.174	2.564	6.795
kess_t15	.272	.0800	3.405	.001	.115	.429	1.313	1.122	1.536
choice_freq	-.079	.0362	-2.193	.028	-.150	-.008	.924	.860	.992
bullying_freq	.095	.0904	1.056	.291	-.082	.273	1.100	.921	1.314
timecwc4	-.005	.0630	-.077	.939	-.129	.119	.995	.879	1.126
timem4	.066	.1994	.333	.739	-.325	.458	1.069	.723	1.580
fac4time	.021	.0619	.338	.736	-.101	.142	1.021	.904	1.153
duties_freq	.040	.0461	.874	.382	-.050	.131	1.041	.951	1.140
FAC1_1	-.081	.0473	-1.721	.085	-.174	.011	.922	.840	1.011

Probability distribution: Binomial

Link function: Logit

a. Target: Phy16 > 2 = 1

Fixed Coefficients

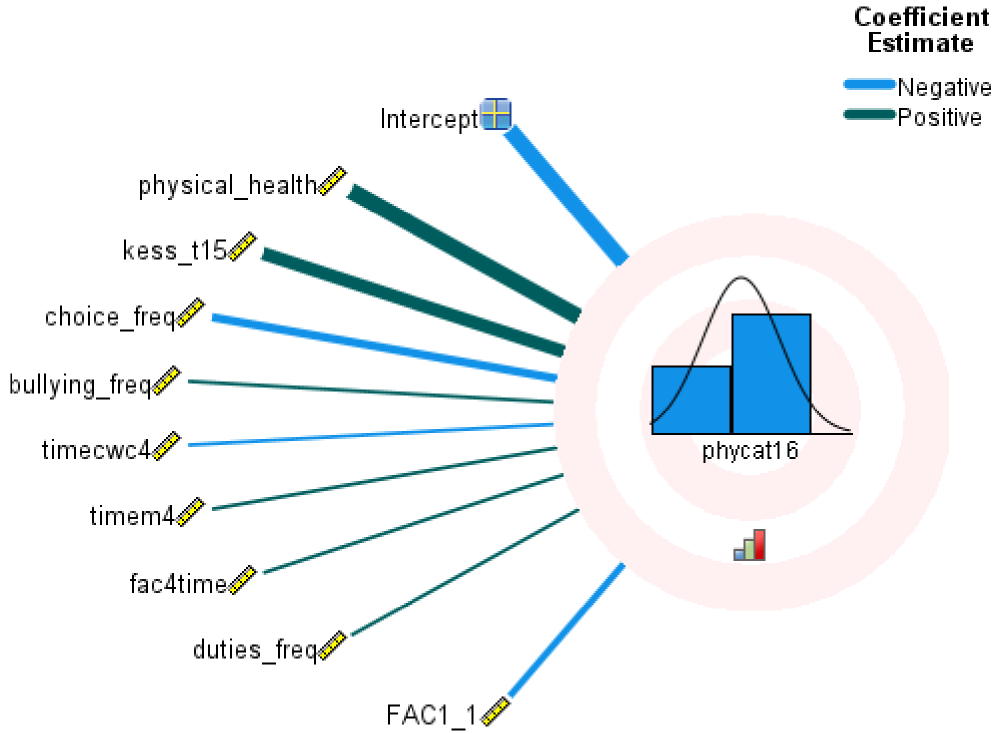

Random Effect Covariances

Random Effect Block 1

Random Effect Block	Intercept
Intercept	.000

Covariance Structure: Variance components

Subject Specification: companyId15

Covariance Parameters

Covariance Parameters Summary

Covariance Parameters	Residual Effect	0
	Random Effects	1
Design Matrix Columns	Fixed Effects	10
	Random Effects	1[a]
Common Subjects		57

Common subjects are based on the subject specifications for the residual and random effects and are used to chunk the data for better performance.

a. This is the number of columns per common subject.

Residual Effect

Residual Effect	Estimate	Std. Error	Z	Sig.	95% Confidence Interval	
					Lower	Upper
Variance	1.000	.	.	.	.	.

Covariance Structure: Scaled Identity

Subject Specification: (None)

Random Effect

Random Effect Covariance	Estimate	Std. Error	Z	Sig.	95% Confidence Interval	
					Lower	Upper
Var(Intercept)	3.206E-11[a]	.	.	.	.	.

Covariance Structure: Variance components

Subject Specification: companyId15

a. This parameter is redundant.

Random Effects Parameters

Empirical Best Linear Unbiased Predictions[a]

company_code	Parameter	Prediction	Std. Error	df	Sig.	95% Confidence Interval	
						Lower Bound	Upper Bound

2	Intercept	-4.362E-11	5.662E-6	1360	1.000	-1.111E-5	1.111E-5
4	Intercept	-3.487E-11	5.662E-6	1360	1.000	-1.111E-5	1.111E-5
6	Intercept	-2.992E-11	5.662E-6	1360	1.000	-1.111E-5	1.111E-5
22	Intercept	-7.670E-12	5.662E-6	1360	1.000	-1.111E-5	1.111E-5
24	Intercept	-5.678E-11	5.662E-6	1360	1.000	-1.111E-5	1.111E-5
30	Intercept	1.324E-11	5.662E-6	1360	1.000	-1.111E-5	1.111E-5
36	Intercept	5.723E-12	5.662E-6	1360	1.000	-1.111E-5	1.111E-5
40	Intercept	-3.115E-11	5.662E-6	1360	1.000	-1.111E-5	1.111E-5
50	Intercept	-6.560E-12	5.662E-6	1360	1.000	-1.111E-5	1.111E-5
61	Intercept	6.802E-12	5.662E-6	1360	1.000	-1.111E-5	1.111E-5
65	Intercept	3.186E-11	5.662E-6	1360	1.000	-1.111E-5	1.111E-5
68	Intercept	1.098E-11	5.662E-6	1360	1.000	-1.111E-5	1.111E-5
69	Intercept	-1.961E-11	5.662E-6	1360	1.000	-1.111E-5	1.111E-5
71	Intercept	5.778E-11	5.662E-6	1360	1.000	-1.111E-5	1.111E-5
72	Intercept	-2.432E-11	5.662E-6	1360	1.000	-1.111E-5	1.111E-5
74	Intercept	3.087E-11	5.662E-6	1360	1.000	-1.111E-5	1.111E-5
75	Intercept	-3.189E-11	5.662E-6	1360	1.000	-1.111E-5	1.111E-5
80	Intercept	-3.567E-11	5.662E-6	1360	1.000	-1.111E-5	1.111E-5
81	Intercept	3.304E-11	5.662E-6	1360	1.000	-1.111E-5	1.111E-5
83	Intercept	-3.065E-11	5.662E-6	1360	1.000	-1.111E-5	1.111E-5
84	Intercept	-8.384E-12	5.662E-6	1360	1.000	-1.111E-5	1.111E-5
92	Intercept	-3.971E-11	5.662E-6	1360	1.000	-1.111E-5	1.111E-5
93	Intercept	1.551E-11	5.662E-6	1360	1.000	-1.111E-5	1.111E-5
98	Intercept	-7.326E-11	5.662E-6	1360	1.000	-1.111E-5	1.111E-5
100	Intercept	-2.688E-10	5.662E-6	1360	1.000	-1.111E-5	1.111E-5
106	Intercept	-3.289E-11	5.662E-6	1360	1.000	-1.111E-5	1.111E-5

107	Intercept	-1.312E-11	5.662E-6	1360	1.000	-1.111E-5	1.111E-5
108	Intercept	-6.821E-11	5.662E-6	1360	1.000	-1.111E-5	1.111E-5
110	Intercept	-2.380E-11	5.662E-6	1360	1.000	-1.111E-5	1.111E-5
114	Intercept	-2.498E-12	5.662E-6	1360	1.000	-1.111E-5	1.111E-5
116	Intercept	2.751E-11	5.662E-6	1360	1.000	-1.111E-5	1.111E-5
122	Intercept	1.057E-10	5.662E-6	1360	1.000	-1.111E-5	1.111E-5
125	Intercept	6.651E-11	5.662E-6	1360	1.000	-1.111E-5	1.111E-5
142	Intercept	-1.174E-10	5.662E-6	1360	1.000	-1.111E-5	1.111E-5
144	Intercept	-6.403E-11	5.662E-6	1360	1.000	-1.111E-5	1.111E-5
145	Intercept	-2.314E-11	5.662E-6	1360	1.000	-1.111E-5	1.111E-5
152	Intercept	1.010E-10	5.662E-6	1360	1.000	-1.111E-5	1.111E-5
154	Intercept	4.072E-11	5.662E-6	1360	1.000	-1.111E-5	1.111E-5
156	Intercept	7.463E-11	5.662E-6	1360	1.000	-1.111E-5	1.111E-5
162	Intercept	2.442E-11	5.662E-6	1360	1.000	-1.111E-5	1.111E-5
165	Intercept	1.437E-11	5.662E-6	1360	1.000	-1.111E-5	1.111E-5
166	Intercept	-3.372E-12	5.662E-6	1360	1.000	-1.111E-5	1.111E-5
169	Intercept	2.731E-11	5.662E-6	1360	1.000	-1.111E-5	1.111E-5
170	Intercept	-2.764E-11	5.662E-6	1360	1.000	-1.111E-5	1.111E-5
173	Intercept	3.852E-11	5.662E-6	1360	1.000	-1.111E-5	1.111E-5
174	Intercept	6.630E-11	5.662E-6	1360	1.000	-1.111E-5	1.111E-5
178	Intercept	2.657E-11	5.662E-6	1360	1.000	-1.111E-5	1.111E-5
181	Intercept	6.807E-11	5.662E-6	1360	1.000	-1.111E-5	1.111E-5
192	Intercept	-4.582E-11	5.662E-6	1360	1.000	-1.111E-5	1.111E-5
202	Intercept	8.958E-11	5.662E-6	1360	1.000	-1.111E-5	1.111E-5
204	Intercept	1.454E-10	5.662E-6	1360	1.000	-1.111E-5	1.111E-5
205	Intercept	-4.000E-11	5.662E-6	1360	1.000	-1.111E-5	1.111E-5

206	Intercept	1.054E-11	5.662E-6	1360	1.000	-1.111E-5	1.111E-5
209	Intercept	9.760E-11	5.662E-6	1360	1.000	-1.111E-5	1.111E-5
217	Intercept	-4.775E-11	5.662E-6	1360	1.000	-1.111E-5	1.111E-5
226	Intercept	2.887E-11	5.662E-6	1360	1.000	-1.111E-5	1.111E-5
228	Intercept	-6.867E-12	5.662E-6	1360	1.000	-1.111E-5	1.111E-5

a. Target: Phy16 > 2 = 1

```
GENLINMIXED
  /DATA_STRUCTURE SUBJECTS=companyId15
  /FIELDS TARGET=phycat16 TRIALS=NONE OFFSET=NONE
  /TARGET_OPTIONS DISTRIBUTION=BINOMIAL LINK=LOGIT
  /FIXED  EFFECTS=physical_health kess_t15 choice_freq bullying_freq time_pressure_freq dutiescwc4 dutiesm4 fac4duti
    FAC1_1 USE_INTERCEPT=TRUE
  /RANDOM USE_INTERCEPT=TRUE SUBJECTS=companyId15 COVARIANCE_TYPE=VARIANCE_COMPONENTS SOLUTION=TRUE
  /BUILD_OPTIONS TARGET_CATEGORY_ORDER=DESCENDING INPUTS_CATEGORY_ORDER=DESCENDING
    MAX_ITERATIONS=100 CONFIDENCE_LEVEL=95 DF_METHOD=RESIDUAL COVB=ROBUST PCONVERGE=0.000001(ABSOLUTE)
    SCORING=0 SINGULAR=0.000000000001
  /EMMEANS_OPTIONS SCALE=ORIGINAL PADJUST=LSD.
```

Generalized Linear Mixed Models

Notes

Output Created	16-AUG-2021 11:08:05
Comments	

Input	Data	C:\Users\jwb12ppu\Documents\Kevin\MPlus backup\Multicomponent health and wellbeing v2\Multicomponent for Mplus cat.sav
	Active Dataset	DataSet2
	Filter	kescat15 = 0 (FILTER)
	Weight	<none>
	Split File	<none>
	N of Rows in Working Data File	1422

Syntax	GENLINMIXED<br>/DATA_STRUCTURE<br>SUBJECTS=companyId15<br>/FIELDS TARGET=phycat16<br>TRIALS=NONE OFFSET=NONE<br>/TARGET_OPTIONS<br>DISTRIBUTION=BINOMIAL<br>LINK=LOGIT<br>/FIXED<br>EFFECTS=physical_health<br>kess_t15 choice_freq bullying_freq<br>time_pressure_freq dutiescwc4<br>dutiesm4 fac4duti<br>FAC1_1<br>USE_INTERCEPT=TRUE<br>/RANDOM<br>USE_INTERCEPT=TRUE<br>SUBJECTS=companyId15<br>COVARIANCE_TYPE=VARIANCE<br>_COMPONENTS<br>SOLUTION=TRUE<br>/BUILD_OPTIONS<br>TARGET_CATEGORY_ORDER=<br>DESCENDING<br>INPUTS_CATEGORY_ORDER=D<br>ESCENDING<br>MAX_ITERATIONS=100<br>CONFIDENCE_LEVEL=95

		DF_METHOD=RESIDUAL COVB=ROBUST PCONVERGE=0.000001(ABSOLUTE) SCORING=0 SINGULAR=0.000000000001 /EMMEANS_OPTIONS SCALE=ORIGINAL PADJUST=LSD.
Resources	Processor Time	00:00:01.11
	Elapsed Time	00:00:01.13

Warnings

glmm: One or more records are not used in the analysis because they have one or more fields with invalid or missing values.
glmm: Valid values for events (target) and trials variables are non-negative and positive integers respectively, and the number of trials cannot be less than the number of events.
glmm: The final Hessian matrix is not positive definite although all convergence criteria are satisfied. The procedure continues despite this warning. Subsequent results produced are based on the last iteration. Validity of the model fit is uncertain.

Case Processing Summary

	N	Percent
Included	1370	96.3%
Excluded	52	3.7%
Total	1422	100.0%

Model Summary

Target		Phy16 > 2 = 1
Probability Distribution		Binomial
Link Function		Logit
Information Criterion	Akaike Corrected	6120.947
	Bayesian	6126.159

Information criteria are based on the -2 log likelihood (6118.944) and are used to compare models. Models with smaller information criterion values fit better.

Data Structure[a]

	Subjects company_code	Target Phy16 > 2 = 1
Data for First Subject	2	1.00
	2	.00
	2	1.00
	2	.00
	2	1.00
	2	1.00
	2	.00

	2	1.00
	2	.00
	2	1.00
Total Number of Levels	57	

Only the first 10 records are displayed.

a. Target: Phy16 > 2 = 1

Classification

Overall Percent Correct = 63.8%[a]

Observed		Predicted 1.00	.00
1.00	Count	842	28
	% within Observed	96.8%	3.2%
.00	Count	468	32
	% within Observed	93.6%	6.4%

a. Target: Phy16 > 2 = 1

Fixed Effects[a]

Source	F	df1	df2	Sig.
Corrected Model	15.750	9	1360	.000
physical_health	31.158	1	1360	.000
kess_t15	11.974	1	1360	.001
choice_freq	5.162	1	1360	.023
bullying_freq	.917	1	1360	.338

time_pressure_freq	.000	1	1360	.993
dutiescwc4	1.938	1	1360	.164
dutiesm4	4.151	1	1360	.042
fac4duti	.565	1	1360	.452
FAC1_1	3.519	1	1360	.061

Probability distribution: Binomial

Link function: Logit

a. Target: Phy16 > 2 = 1

Fixed Effects

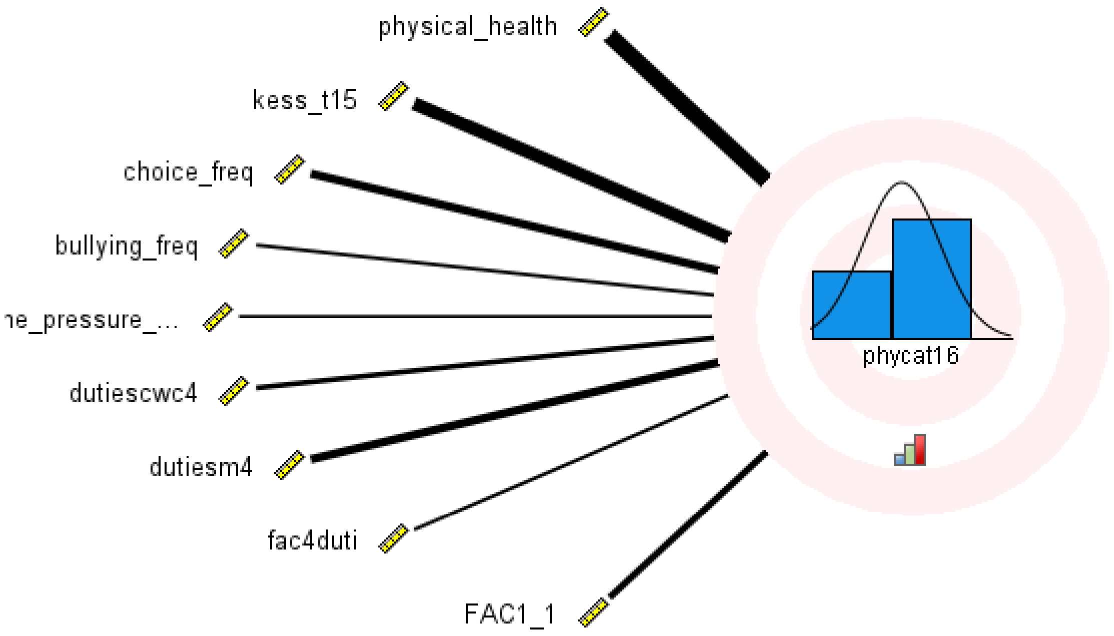

Fixed Coefficients[a]

Model Term	Coefficient	Std. Error	t	Sig.	95% Confidence Interval		Exp(Coefficient)	95% Confidence Interval for Exp(Coefficient)	
					Lower	Upper		Lower	Upper
Intercept	-2.578	.7872	-3.275	.001	-4.123	-1.034	.076	.016	.356
physical_health	1.434	.2569	5.582	.000	.930	1.938	4.195	2.534	6.944
kess_t15	.272	.0785	3.460	.001	.118	.426	1.312	1.125	1.531
choice_freq	-.081	.0355	-2.272	.023	-.150	-.011	.922	.860	.989
bullying_freq	.088	.0916	.958	.338	-.092	.267	1.092	.912	1.306
time_pressure_freq	.001	.0588	.009	.993	-.115	.116	1.001	.892	1.123
dutiescwc4	.065	.0467	1.392	.164	-.027	.157	1.067	.974	1.169
dutiesm4	-.379	.1858	-2.037	.042	-.743	-.014	.685	.476	.986
fac4duti	-.032	.0421	-.752	.452	-.114	.051	.969	.892	1.052
FAC1_1	-.087	.0465	-1.876	.061	-.178	.004	.917	.837	1.004

Probability distribution: Binomial

Link function: Logit

a. Target: Phy16 > 2 = 1

Fixed Coefficients

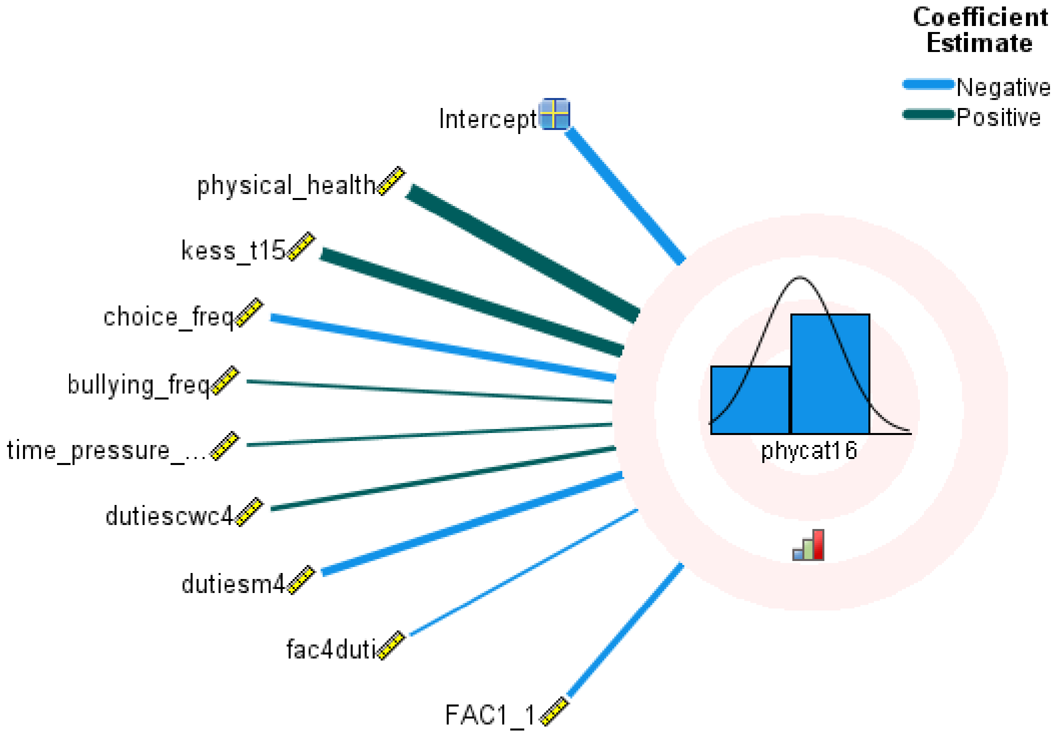

Random Effect Covariances

Random Effect Block 1

Random Effect Block	Intercept
Intercept	.000

Covariance Structure: Variance components

Subject Specification: companyId15

Covariance Parameters

Covariance Parameters Summary

Covariance Parameters	Residual Effect	0
	Random Effects	1
Design Matrix Columns	Fixed Effects	10
	Random Effects	1[a]
Common Subjects		57

Common subjects are based on the subject specifications for the residual and random effects and are used to chunk the data for better performance.

a. This is the number of columns per common subject.

Residual Effect

Residual Effect	Estimate	Std. Error	Z	Sig.	95% Confidence Interval	
					Lower	Upper
Variance	1.000	.	.	.	.	.

Covariance Structure: Scaled Identity

Subject Specification: (None)

Random Effect

Random Effect Covariance	Estimate	Std. Error	Z	Sig.	95% Confidence Interval	
					Lower	Upper
Var(Intercept)	2.927E-11[a]	.	.	.	.	.

Covariance Structure: Variance components

Subject Specification: companyId15

a. This parameter is redundant.

Random Effects Parameters

Empirical Best Linear Unbiased Predictions[a]

company_code	Parameter	Prediction	Std. Error	df	Sig.	95% Confidence Interval	
						Lower Bound	Upper Bound

2	Intercept	-1.924E-11	5.410E-6	1360	1.000	-1.061E-5	1.061E-5
4	Intercept	-2.756E-11	5.410E-6	1360	1.000	-1.061E-5	1.061E-5
6	Intercept	-3.730E-11	5.410E-6	1360	1.000	-1.061E-5	1.061E-5
22	Intercept	-1.161E-11	5.410E-6	1360	1.000	-1.061E-5	1.061E-5
24	Intercept	-4.456E-11	5.410E-6	1360	1.000	-1.061E-5	1.061E-5
30	Intercept	1.885E-11	5.410E-6	1360	1.000	-1.061E-5	1.061E-5
36	Intercept	1.481E-11	5.410E-6	1360	1.000	-1.061E-5	1.061E-5
40	Intercept	-3.181E-11	5.410E-6	1360	1.000	-1.061E-5	1.061E-5
50	Intercept	-6.173E-12	5.410E-6	1360	1.000	-1.061E-5	1.061E-5
61	Intercept	2.189E-11	5.410E-6	1360	1.000	-1.061E-5	1.061E-5
65	Intercept	3.496E-11	5.410E-6	1360	1.000	-1.061E-5	1.061E-5
68	Intercept	3.070E-11	5.410E-6	1360	1.000	-1.061E-5	1.061E-5
69	Intercept	-1.819E-11	5.410E-6	1360	1.000	-1.061E-5	1.061E-5
71	Intercept	3.841E-11	5.410E-6	1360	1.000	-1.061E-5	1.061E-5
72	Intercept	-2.520E-11	5.410E-6	1360	1.000	-1.061E-5	1.061E-5
74	Intercept	3.416E-11	5.410E-6	1360	1.000	-1.061E-5	1.061E-5
75	Intercept	-1.676E-11	5.410E-6	1360	1.000	-1.061E-5	1.061E-5
80	Intercept	-3.185E-11	5.410E-6	1360	1.000	-1.061E-5	1.061E-5
81	Intercept	2.423E-11	5.410E-6	1360	1.000	-1.061E-5	1.061E-5
83	Intercept	-9.262E-12	5.410E-6	1360	1.000	-1.061E-5	1.061E-5
84	Intercept	-5.014E-13	5.410E-6	1360	1.000	-1.061E-5	1.061E-5
92	Intercept	-3.776E-11	5.410E-6	1360	1.000	-1.061E-5	1.061E-5
93	Intercept	1.798E-11	5.410E-6	1360	1.000	-1.061E-5	1.061E-5
98	Intercept	-4.912E-11	5.410E-6	1360	1.000	-1.061E-5	1.061E-5
100	Intercept	-2.482E-10	5.410E-6	1360	1.000	-1.061E-5	1.061E-5
106	Intercept	-4.012E-11	5.410E-6	1360	1.000	-1.061E-5	1.061E-5

107	Intercept	-1.937E-11	5.410E-6	1360	1.000	-1.061E-5	1.061E-5
108	Intercept	-6.019E-11	5.410E-6	1360	1.000	-1.061E-5	1.061E-5
110	Intercept	-1.247E-10	5.410E-6	1360	1.000	-1.061E-5	1.061E-5
114	Intercept	1.157E-11	5.410E-6	1360	1.000	-1.061E-5	1.061E-5
116	Intercept	1.506E-11	5.410E-6	1360	1.000	-1.061E-5	1.061E-5
122	Intercept	9.436E-11	5.410E-6	1360	1.000	-1.061E-5	1.061E-5
125	Intercept	4.962E-11	5.410E-6	1360	1.000	-1.061E-5	1.061E-5
142	Intercept	-8.107E-11	5.410E-6	1360	1.000	-1.061E-5	1.061E-5
144	Intercept	-5.137E-11	5.410E-6	1360	1.000	-1.061E-5	1.061E-5
145	Intercept	-2.694E-11	5.410E-6	1360	1.000	-1.061E-5	1.061E-5
152	Intercept	9.892E-11	5.410E-6	1360	1.000	-1.061E-5	1.061E-5
154	Intercept	2.710E-11	5.410E-6	1360	1.000	-1.061E-5	1.061E-5
156	Intercept	6.369E-11	5.410E-6	1360	1.000	-1.061E-5	1.061E-5
162	Intercept	1.375E-11	5.410E-6	1360	1.000	-1.061E-5	1.061E-5
165	Intercept	1.136E-11	5.410E-6	1360	1.000	-1.061E-5	1.061E-5
166	Intercept	4.702E-13	5.410E-6	1360	1.000	-1.061E-5	1.061E-5
169	Intercept	-1.538E-12	5.410E-6	1360	1.000	-1.061E-5	1.061E-5
170	Intercept	-2.651E-11	5.410E-6	1360	1.000	-1.061E-5	1.061E-5
173	Intercept	3.890E-11	5.410E-6	1360	1.000	-1.061E-5	1.061E-5
174	Intercept	6.811E-11	5.410E-6	1360	1.000	-1.061E-5	1.061E-5
178	Intercept	3.406E-11	5.410E-6	1360	1.000	-1.061E-5	1.061E-5
181	Intercept	5.790E-11	5.410E-6	1360	1.000	-1.061E-5	1.061E-5
192	Intercept	-3.325E-11	5.410E-6	1360	1.000	-1.061E-5	1.061E-5
202	Intercept	6.197E-11	5.410E-6	1360	1.000	-1.061E-5	1.061E-5
204	Intercept	1.508E-10	5.410E-6	1360	1.000	-1.061E-5	1.061E-5
205	Intercept	-3.282E-11	5.410E-6	1360	1.000	-1.061E-5	1.061E-5

206	Intercept	1.870E-12	5.410E-6	1360	1.000	-1.061E-5	1.061E-5
209	Intercept	5.152E-11	5.410E-6	1360	1.000	-1.061E-5	1.061E-5
217	Intercept	7.164E-12	5.410E-6	1360	1.000	-1.061E-5	1.061E-5
226	Intercept	1.962E-11	5.410E-6	1360	1.000	-1.061E-5	1.061E-5
228	Intercept	-8.730E-13	5.410E-6	1360	1.000	-1.061E-5	1.061E-5

a. Target: Phy16 > 2 = 1

```
FILTER OFF.
USE ALL.
EXECUTE.
```

HLM-7 results

Variable labels

Physical_health - 2105 physical health, continuous variable

Kess_t15 – 2015 Kessler score, continuous variable

Choice – job control

Bully - bullying

Time – time pressure

Duties – role clarity

Wellpca – first principal component of health and wellbeing practices

Wellpcas – Wellpca * Wellpca

These are results for developing low distress in 2016 (i.e. recovery from high distress)

Main effects

Final estimation of fixed effects: (Unit-specific model)

Fixed Effect	Coefficient	Standard Error	t	Approx. d.f.	p
For INTRCPT1, ß0					
INTRCPT2, ?00	0.793725	0.073256	10.835	56	<0.001
WELLPCA, ?01	-0.167877	0.070168	-2.392	56	0.020
For CHOICE slope, ß1					
INTRCPT2, ?10	0.027361	0.063667	0.430	1036	0.667
For BULLY slope, ß2					
INTRCPT2, ?20	0.183660	0.100089	1.835	1036	0.067
For TIME slope, ß3					
INTRCPT2, ?30	-0.074334	0.067184	-1.106	1036	0.269
For DUTIES slope, ß4					
INTRCPT2, ?40	-0.136498	0.072338	-1.887	1036	0.059
For PHYCON15 slope, ß5					
INTRCPT2, ?50	0.265914	0.085556	3.108	1036	0.002
For KESCON15 slope, ß6					
INTRCPT2, ?60	1.331429	0.164615	8.088	1036	<0.001

Fixed Effect	Coefficient	Odds Ratio	Confidence Interval
For INTRCPT1, ß0			
INTRCPT2, ?00	0.793725	2.211619	(1.910,2.561)
WELLPCA, ?01	-0.167877	0.845458	(0.735,0.973)
For CHOICE slope, ß1			
INTRCPT2, ?10	0.027361	1.027739	(0.907,1.165)
For BULLY slope, ß2			
INTRCPT2, ?20	0.183660	1.201607	(0.987,1.462)
For TIME slope, ß3			
INTRCPT2, ?30	-0.074334	0.928362	(0.814,1.059)
For DUTIES slope, ß4			
INTRCPT2, ?40	-0.136498	0.872408	(0.757,1.006)
For PHYCON15 slope, ß5			
INTRCPT2, ?50	0.265914	1.304623	(1.103,1.543)
For KESCON15 slope, ß6			
INTRCPT2, ?60	1.331429	3.786451	(2.741,5.231)

Final estimation of fixed effects
(Unit-specific model with robust standard errors)

Fixed Effect	Coefficient	Standard	t	Approx.p

		Error		d.f.	
For INTRCPT1, ß0					
INTRCPT2, ?00	0.793725	0.060283	13.167	56	<0.001
WELLPCA, ?01	-0.167877	0.074709	-2.247	56	0.029
For CHOICE slope, ß1					
INTRCPT2, ?10	0.027361	0.053786	0.509	1036	0.611
For BULLY slope, ß2					
INTRCPT2, ?20	0.183660	0.106842	1.719	1036	0.086
For TIME slope, ß3					
INTRCPT2, ?30	-0.074334	0.056663	-1.312	1036	0.190
For DUTIES slope, ß4					
INTRCPT2, ?40	-0.136498	0.053497	-2.552	1036	0.011
For PHYCON15 slope, ß5					
INTRCPT2, ?50	0.265914	0.086951	3.058	1036	0.002
For KESCON15 slope, ß6					
INTRCPT2, ?60	1.331429	0.157037	8.478	1036	<0.001

Fixed Effect	Coefficient	Odds Ratio	Confidence Interval
For INTRCPT1, ß0			
INTRCPT2, ?00	0.793725	2.211619	(1.960,2.496)
WELLPCA, ?01	-0.167877	0.845458	(0.728,0.982)
For CHOICE slope, ß1			
INTRCPT2, ?10	0.027361	1.027739	(0.925,1.142)
For BULLY slope, ß2			
INTRCPT2, ?20	0.183660	1.201607	(0.974,1.482)
For TIME slope, ß3			
INTRCPT2, ?30	-0.074334	0.928362	(0.831,1.038)
For DUTIES slope, ß4			
INTRCPT2, ?40	-0.136498	0.872408	(0.785,0.969)
For PHYCON15 slope, ß5			
INTRCPT2, ?50	0.265914	1.304623	(1.100,1.547)
For KESCON15 slope, ß6			
INTRCPT2, ?60	1.331429	3.786451	(2.782,5.154)

Final estimation of variance components

Random Effect	Standard Deviation	Variance Component	df	Chi-square	p
INTRCPT1, u0	0.01631	0.00027	56	68.62298	0.120

Curvilinear effects

Final estimation of fixed effects: (Unit-specific model)

Fixed Effect	Coefficient	Standard Error	t	Approx. d.f.	p
For INTRCPT1, ß0					
INTRCPT2, ?00	0.731526	0.076504	9.562	55	<0.001
WELLPCA, ?01	-0.184162	0.069709	-2.642	55	0.011
WELLPCAS, ?02	-0.154170	0.059785	-2.579	55	0.013
For CHOICE slope, ß1					
INTRCPT2, ?10	0.036442	0.063973	0.570	1036	0.569
For BULLY slope, ß2					
INTRCPT2, ?20	0.168854	0.100331	1.683	1036	0.093
For TIME slope, ß3					
INTRCPT2, ?30	-0.080011	0.067336	-1.188	1036	0.235
For DUTIES slope, ß4					
INTRCPT2, ?40	-0.137367	0.072544	-1.894	1036	0.059
For PHYCON15 slope, ß5					
INTRCPT2, ?50	0.263463	0.085702	3.074	1036	0.002
For KESCON15 slope, ß6					
INTRCPT2, ?60	1.352508	0.166048	8.145	1036	<0.001

Fixed Effect	Coefficient	Odds Ratio	Confidence Interval
For INTRCPT1, ß0			
INTRCPT2, ?00	0.731526	2.078249	(1.783,2.423)
WELLPCA, ?01	-0.184162	0.831801	(0.723,0.957)
WELLPCAS, ?02	-0.154170	0.857126	(0.760,0.966)
For CHOICE slope, ß1			
INTRCPT2, ?10	0.036442	1.037114	(0.915,1.176)
For BULLY slope, ß2			
INTRCPT2, ?20	0.168854	1.183947	(0.972,1.442)
For TIME slope, ß3			
INTRCPT2, ?30	-0.080011	0.923106	(0.809,1.054)
For DUTIES slope, ß4			
INTRCPT2, ?40	-0.137367	0.871651	(0.756,1.005)
For PHYCON15 slope, ß5			
INTRCPT2, ?50	0.263463	1.301430	(1.100,1.540)
For KESCON15 slope, ß6			
INTRCPT2, ?60	1.352508	3.867112	(2.791,5.357)

Final estimation of fixed effects
(Unit-specific model with robust standard errors)

Fixed Effect	Coefficient	Standard Error	t	Approx. d.f.	p

For INTRCPT1, ß0					
INTRCPT2, ?00	0.731526	0.070520	10.373	55	<0.001
WELLPCA, ?01	-0.184162	0.065438	-2.814	55	0.007
WELLPCAS, ?02	-0.154170	0.060380	-2.553	55	0.013
For CHOICE slope, ß1					
INTRCPT2, ?10	0.036442	0.053301	0.684	1036	0.494
For BULLY slope, ß2					
INTRCPT2, ?20	0.168854	0.102984	1.640	1036	0.101
For TIME slope, ß3					
INTRCPT2, ?30	-0.080011	0.056859	-1.407	1036	0.160
For DUTIES slope, ß4					
INTRCPT2, ?40	-0.137367	0.052693	-2.607	1036	0.009
For PHYCON15 slope, ß5					
INTRCPT2, ?50	0.263463	0.087501	3.011	1036	0.003
For KESCON15 slope, ß6					
INTRCPT2, ?60	1.352508	0.160791	8.412	1036	<0.001

Fixed Effect	Coefficient	Odds Ratio	Confidence Interval
For INTRCPT1, ß0			
INTRCPT2, ?00	0.731526	2.078249	(1.804,2.394)
WELLPCA, ?01	-0.184162	0.831801	(0.730,0.948)
WELLPCAS, ?02	-0.154170	0.857126	(0.759,0.967)
For CHOICE slope, ß1			
INTRCPT2, ?10	0.036442	1.037114	(0.934,1.152)
For BULLY slope, ß2			
INTRCPT2, ?20	0.168854	1.183947	(0.967,1.449)
For TIME slope, ß3			
INTRCPT2, ?30	-0.080011	0.923106	(0.826,1.032)
For DUTIES slope, ß4			
INTRCPT2, ?40	-0.137367	0.871651	(0.786,0.967)
For PHYCON15 slope, ß5			
INTRCPT2, ?50	0.263463	1.301430	(1.096,1.545)
For KESCON15 slope, ß6			
INTRCPT2, ?60	1.352508	3.867112	(2.820,5.302)

Final estimation of variance components

Random Effect	Standard Deviation	Variance Component	df	Chi-square	p
INTRCPT1, u0	0.01789	0.00032	55	62.26207	0.233

Choice interaction

Final estimation of fixed effects: (Unit-specific model)

Fixed Effect	Coefficient	Standard Error	t	Approx. d.f.	p
For INTRCPT1, ß0					
INTRCPT2, ?00	0.794484	0.073213	10.852	56	<0.001
WELLPCA, ?01	-0.167655	0.070132	-2.391	56	0.020
For CHOICE slope, ß1					
INTRCPT2, ?10	0.044565	0.068810	0.648	56	0.520
WELLPCA, ?11	0.036630	0.071208	0.514	56	0.609
For BULLY slope, ß2					
INTRCPT2, ?20	0.185083	0.099931	1.852	979	0.064
For TIME slope, ß3					
INTRCPT2, ?30	-0.074120	0.067026	-1.106	979	0.269
For DUTIES slope, ß4					
INTRCPT2, ?40	-0.139986	0.072313	-1.936	979	0.053
For PHYCON15 slope, ß5					
INTRCPT2, ?50	0.265807	0.085552	3.107	979	0.002
For KESCON15 slope, ß6					
INTRCPT2, ?60	1.332734	0.164734	8.090	979	<0.001

Fixed Effect	Coefficient	Odds Ratio	Confidence Interval
For INTRCPT1, ß0			
INTRCPT2, ?00	0.794484	2.213298	(1.911,2.563)
WELLPCA, ?01	-0.167655	0.845646	(0.735,0.973)
For CHOICE slope, ß1			
INTRCPT2, ?10	0.044565	1.045573	(0.911,1.200)
WELLPCA, ?11	0.036630	1.037309	(0.899,1.196)
For BULLY slope, ß2			
INTRCPT2, ?20	0.185083	1.203319	(0.989,1.464)
For TIME slope, ß3			
INTRCPT2, ?30	-0.074120	0.928560	(0.814,1.059)
For DUTIES slope, ß4			
INTRCPT2, ?40	-0.139986	0.869371	(0.754,1.002)
For PHYCON15 slope, ß5			
INTRCPT2, ?50	0.265807	1.304483	(1.103,1.543)
For KESCON15 slope, ß6			
INTRCPT2, ?60	1.332734	3.791397	(2.744,5.239)

Final estimation of fixed effects

(Unit-specific model with robust standard errors)

Fixed Effect	Coefficient	Standard Error	t	Approx. d.f.	p
For INTRCPT1, ß0					
INTRCPT2, ?00	0.794484	0.060252	13.186	56	<0.001
WELLPCA, ?01	-0.167655	0.074051	-2.264	56	0.027
For CHOICE slope, ß1					
INTRCPT2, ?10	0.044565	0.059598	0.748	56	0.458
WELLPCA, ?11	0.036630	0.058980	0.621	56	0.537
For BULLY slope, ß2					
INTRCPT2, ?20	0.185083	0.106898	1.731	979	0.084
For TIME slope, ß3					
INTRCPT2, ?30	-0.074120	0.057522	-1.289	979	0.198
For DUTIES slope, ß4					
INTRCPT2, ?40	-0.139986	0.053081	-2.637	979	0.008
For PHYCON15 slope, ß5					
INTRCPT2, ?50	0.265807	0.086939	3.057	979	0.002
For KESCON15 slope, ß6					
INTRCPT2, ?60	1.332734	0.157061	8.485	979	<0.001

Fixed Effect	Coefficient	Odds Ratio	Confidence Interval
For INTRCPT1, ß0			
INTRCPT2, ?00	0.794484	2.213298	(1.962,2.497)
WELLPCA, ?01	-0.167655	0.845646	(0.729,0.981)
For CHOICE slope, ß1			
INTRCPT2, ?10	0.044565	1.045573	(0.928,1.178)
WELLPCA, ?11	0.036630	1.037309	(0.922,1.167)
For BULLY slope, ß2			
INTRCPT2, ?20	0.185083	1.203319	(0.976,1.484)
For TIME slope, ß3			
INTRCPT2, ?30	-0.074120	0.928560	(0.829,1.040)
For DUTIES slope, ß4			
INTRCPT2, ?40	-0.139986	0.869371	(0.783,0.965)
For PHYCON15 slope, ß5			
INTRCPT2, ?50	0.265807	1.304483	(1.100,1.547)
For KESCON15 slope, ß6			
INTRCPT2, ?60	1.332734	3.791397	(2.785,5.161)

Final estimation of variance components

Random Effect	Standard Deviation	Variance Component	df	Chi-square	p
INTRCPT1, u0	0.00776	0.00006	53	65.27129	0.120
CHOICE slope, u1	0.01058	0.00011	53	50.95174	>0.500

Bully interaction

Final estimation of fixed effects: (Unit-specific model)

Fixed Effect	Coefficient	Standard Error	t	Approx. d.f.	p
For INTRCPT1, ß0					
INTRCPT2, ?00	0.797673	0.073983	10.782	56	<0.001
WELLPCA, ?01	-0.175129	0.071081	-2.464	56	0.017
For CHOICE slope, ß1					
INTRCPT2, ?10	0.024326	0.063621	0.382	979	0.702
For BULLY slope, ß2					
INTRCPT2, ?20	0.237053	0.112841	2.101	56	0.040
WELLPCA, ?21	-0.189065	0.118850	-1.591	56	0.117
For TIME slope, ß3					
INTRCPT2, ?30	-0.069261	0.067160	-1.031	979	0.303
For DUTIES slope, ß4					
INTRCPT2, ?40	-0.140375	0.072276	-1.942	979	0.052
For PHYCON15 slope, ß5					
INTRCPT2, ?50	0.264618	0.085754	3.086	979	0.002
For KESCON15 slope, ß6					
INTRCPT2, ?60	1.328184	0.164314	8.083	979	<0.001

Fixed Effect	Coefficient	Odds Ratio	Confidence Interval
For INTRCPT1, ß0			
INTRCPT2, ?00	0.797673	2.220367	(1.914,2.575)
WELLPCA, ?01	-0.175129	0.839349	(0.728,0.968)
For CHOICE slope, ß1			
INTRCPT2, ?10	0.024326	1.024624	(0.904,1.161)
For BULLY slope, ß2			
INTRCPT2, ?20	0.237053	1.267509	(1.011,1.589)
WELLPCA, ?21	-0.189065	0.827733	(0.652,1.050)
For TIME slope, ß3			
INTRCPT2, ?30	-0.069261	0.933083	(0.818,1.065)
For DUTIES slope, ß4			
INTRCPT2, ?40	-0.140375	0.869032	(0.754,1.002)
For PHYCON15 slope, ß5			
INTRCPT2, ?50	0.264618	1.302934	(1.101,1.542)
For KESCON15 slope, ß6			
INTRCPT2, ?60	1.328184	3.774183	(2.734,5.211)

Final estimation of fixed effects
(Unit-specific model with robust standard errors)

Fixed Effect	Coefficient	Standard	t	Approx.p

		Error		d.f.	
For INTRCPT1, ß0					
INTRCPT2, ?00	0.797673	0.063023	12.657	56	<0.001
WELLPCA, ?01	-0.175129	0.076077	-2.302	56	0.025
For CHOICE slope, ß1					
INTRCPT2, ?10	0.024326	0.054896	0.443	979	0.658
For BULLY slope, ß2					
INTRCPT2, ?20	0.237053	0.093896	2.525	56	0.014
WELLPCA, ?21	-0.189065	0.101311	-1.866	56	0.067
For TIME slope, ß3					
INTRCPT2, ?30	-0.069261	0.055981	-1.237	979	0.216
For DUTIES slope, ß4					
INTRCPT2, ?40	-0.140375	0.053343	-2.632	979	0.009
For PHYCON15 slope, ß5					
INTRCPT2, ?50	0.264618	0.085615	3.091	979	0.002
For KESCON15 slope, ß6					
INTRCPT2, ?60	1.328184	0.155914	8.519	979	<0.001

Fixed Effect	Coefficient	Odds Ratio	Confidence Interval
For INTRCPT1, ß0			
INTRCPT2, ?00	0.797673	2.220367	(1.957,2.519)
WELLPCA, ?01	-0.175129	0.839349	(0.721,0.978)
For CHOICE slope, ß1			
INTRCPT2, ?10	0.024326	1.024624	(0.920,1.141)
For BULLY slope, ß2			
INTRCPT2, ?20	0.237053	1.267509	(1.050,1.530)
WELLPCA, ?21	-0.189065	0.827733	(0.676,1.014)
For TIME slope, ß3			
INTRCPT2, ?30	-0.069261	0.933083	(0.836,1.041)
For DUTIES slope, ß4			
INTRCPT2, ?40	-0.140375	0.869032	(0.783,0.965)
For PHYCON15 slope, ß5			
INTRCPT2, ?50	0.264618	1.302934	(1.101,1.541)
For KESCON15 slope, ß6			
INTRCPT2, ?60	1.328184	3.774183	(2.779,5.126)

Random Effect	Standard Deviation	Variance Component	df	Chi-square	p
INTRCPT1, u0	0.02874	0.00083	49	57.80993	0.182
BULLY slope, u2	0.04223	0.00178	49	44.76029	>0.500

Time interaction

Final estimation of fixed effects: (Unit-specific model)

Fixed Effect	Coefficient	Standard Error	t	Approx. d.f.	p
For INTRCPT1, ß0					
INTRCPT2, ?00	0.793428	0.073281	10.827	56	<0.001
WELLPCA, ?01	-0.172700	0.070159	-2.462	56	0.017
For CHOICE slope, ß1					
INTRCPT2, ?10	0.031288	0.063476	0.493	979	0.622
For BULLY slope, ß2					
INTRCPT2, ?20	0.176280	0.099743	1.767	979	0.077
For TIME slope, ß3					
INTRCPT2, ?30	-0.053212	0.072094	-0.738	56	0.464
WELLPCA, ?31	0.005522	0.069417	0.080	56	0.937
For DUTIES slope, ß4					
INTRCPT2, ?40	-0.130057	0.071933	-1.808	979	0.071
For PHYCON15 slope, ß5					
INTRCPT2, ?50	0.260138	0.085183	3.054	979	0.002
For KESCON15 slope, ß6					
INTRCPT2, ?60	1.330609	0.164630	8.082	979	<0.001

Fixed Effect	Coefficient	Odds Ratio	Confidence Interval
For INTRCPT1, ß0			
INTRCPT2, ?00	0.793428	2.210963	(1.909,2.561)
WELLPCA, ?01	-0.172700	0.841390	(0.731,0.968)
For CHOICE slope, ß1			
INTRCPT2, ?10	0.031288	1.031783	(0.911,1.169)
For BULLY slope, ß2			
INTRCPT2, ?20	0.176280	1.192772	(0.981,1.451)
For TIME slope, ß3			
INTRCPT2, ?30	-0.053212	0.948179	(0.821,1.096)
WELLPCA, ?31	0.005522	1.005538	(0.875,1.156)
For DUTIES slope, ß4			
INTRCPT2, ?40	-0.130057	0.878045	(0.762,1.011)
For PHYCON15 slope, ß5			
INTRCPT2, ?50	0.260138	1.297110	(1.097,1.533)
For KESCON15 slope, ß6			
INTRCPT2, ?60	1.330609	3.783348	(2.739,5.227)

Final estimation of fixed effects
(Unit-specific model with robust standard errors)

Fixed Effect	Coefficient	Standard	t	Approx.p

		Error		d.f.	
For INTRCPT1, ß0					
INTRCPT2, ?00	0.793428	0.059746	13.280	56	<0.001
WELLPCA, ?01	-0.172700	0.076543	-2.256	56	0.028
For CHOICE slope, ß1					
INTRCPT2, ?10	0.031288	0.054046	0.579	979	0.563
For BULLY slope, ß2					
INTRCPT2, ?20	0.176280	0.106386	1.657	979	0.098
For TIME slope, ß3					
INTRCPT2, ?30	-0.053212	0.057101	-0.932	56	0.355
WELLPCA, ?31	0.005522	0.063317	0.087	56	0.931
For DUTIES slope, ß4					
INTRCPT2, ?40	-0.130057	0.053848	-2.415	979	0.016
For PHYCON15 slope, ß5					
INTRCPT2, ?50	0.260138	0.087156	2.985	979	0.003
For KESCON15 slope, ß6					
INTRCPT2, ?60	1.330609	0.156262	8.515	979	<0.001

Fixed Effect	Coefficient	Odds Ratio	Confidence Interval
For INTRCPT1, ß0			
INTRCPT2, ?00	0.793428	2.210963	(1.961,2.492)
WELLPCA, ?01	-0.172700	0.841390	(0.722,0.981)
For CHOICE slope, ß1			
INTRCPT2, ?10	0.031288	1.031783	(0.928,1.147)
For BULLY slope, ß2			
INTRCPT2, ?20	0.176280	1.192772	(0.968,1.470)
For TIME slope, ß3			
INTRCPT2, ?30	-0.053212	0.948179	(0.846,1.063)
WELLPCA, ?31	0.005522	1.005538	(0.886,1.142)
For DUTIES slope, ß4			
INTRCPT2, ?40	-0.130057	0.878045	(0.790,0.976)
For PHYCON15 slope, ß5			
INTRCPT2, ?50	0.260138	1.297110	(1.093,1.539)
For KESCON15 slope, ß6			
INTRCPT2, ?60	1.330609	3.783348	(2.784,5.142)

Random Effect	Standard Deviation	Variance Component	df	Chi-square	p
INTRCPT1, u0	0.01966	0.00039	55	64.01487	0.190
TIME slope, u3	0.02198	0.00048	55	56.02161	0.436

Final estimation of fixed effects: (Unit-specific model)

Fixed Effect	Coefficient	Standard Error	t	Approx. d.f.	p
For INTRCPT1, ß0					
INTRCPT2, ?00	0.791673	0.073305	10.800	56	<0.001
WELLPCA, ?01	-0.159618	0.070182	-2.274	56	0.027
For CHOICE slope, ß1					
INTRCPT2, ?10	0.024326	0.063585	0.383	979	0.702
For BULLY slope, ß2					
INTRCPT2, ?20	0.192117	0.099865	1.924	979	0.055
For TIME slope, ß3					
INTRCPT2, ?30	-0.067038	0.066959	-1.001	979	0.317
For DUTIES slope, ß4					
INTRCPT2, ?40	-0.106721	0.078100	-1.366	56	0.177
WELLPCA, ?41	-0.022672	0.078276	-0.290	56	0.773
For PHYCON15 slope, ß5					
INTRCPT2, ?50	0.269595	0.085522	3.152	979	0.002
For KESCON15 slope, ß6					
INTRCPT2, ?60	1.336126	0.164668	8.114	979	<0.001

Fixed Effect	Coefficient	Odds Ratio	Confidence Interval
For INTRCPT1, ß0			
INTRCPT2, ?00	0.791673	2.207085	(1.906,2.556)
WELLPCA, ?01	-0.159618	0.852469	(0.741,0.981)
For CHOICE slope, ß1			
INTRCPT2, ?10	0.024326	1.024624	(0.904,1.161)
For BULLY slope, ß2			
INTRCPT2, ?20	0.192117	1.211812	(0.996,1.474)
For TIME slope, ß3			
INTRCPT2, ?30	-0.067038	0.935160	(0.820,1.067)
For DUTIES slope, ß4			
INTRCPT2, ?40	-0.106721	0.898776	(0.769,1.051)
WELLPCA, ?41	-0.022672	0.977583	(0.836,1.144)
For PHYCON15 slope, ß5			
INTRCPT2, ?50	0.269595	1.309434	(1.107,1.549)
For KESCON15 slope, ß6			
INTRCPT2, ?60	1.336126	3.804277	(2.754,5.256)

Final estimation of fixed effects
(Unit-specific model with robust standard errors)

Fixed Effect	Coefficient	Standard Error	t	Approx. d.f.	p
For INTRCPT1, ß0					
INTRCPT2, ?00	0.791673	0.061897	12.790	56	<0.001
WELLPCA, ?01	-0.159618	0.076236	-2.094	56	0.041

For CHOICE slope, ß1					
INTRCPT2, ?10	0.024326	0.053472	0.455	979	0.649
For BULLY slope, ß2					
INTRCPT2, ?20	0.192117	0.107119	1.793	979	0.073
For TIME slope, ß3					
INTRCPT2, ?30	-0.067038	0.055938	-1.198	979	0.231
For DUTIES slope, ß4					
INTRCPT2, ?40	-0.106721	0.058328	-1.830	56	0.073
WELLPCA, ?41	-0.022672	0.051938	-0.437	56	0.664
For PHYCON15 slope, ß5					
INTRCPT2, ?50	0.269595	0.086795	3.106	979	0.002
For KESCON15 slope, ß6					
INTRCPT2, ?60	1.336126	0.157420	8.488	979	<0.001

Fixed Effect	Coefficient	Odds Ratio	Confidence Interval
For INTRCPT1, ß0			
INTRCPT2, ?00	0.791673	2.207085	(1.950,2.499)
WELLPCA, ?01	-0.159618	0.852469	(0.732,0.993)
For CHOICE slope, ß1			
INTRCPT2, ?10	0.024326	1.024624	(0.923,1.138)
For BULLY slope, ß2			
INTRCPT2, ?20	0.192117	1.211812	(0.982,1.495)
For TIME slope, ß3			
INTRCPT2, ?30	-0.067038	0.935160	(0.838,1.044)
For DUTIES slope, ß4			
INTRCPT2, ?40	-0.106721	0.898776	(0.800,1.010)
WELLPCA, ?41	-0.022672	0.977583	(0.881,1.085)
For PHYCON15 slope, ß5			
INTRCPT2, ?50	0.269595	1.309434	(1.104,1.553)
For KESCON15 slope, ß6			
INTRCPT2, ?60	1.336126	3.804277	(2.793,5.182)

Random Effect	Standard Deviation	Variance Component	df	Chi-square	p
INTRCPT1, u0	0.02144	0.00046	55	67.79429	0.115
DUTIES slope, u4	0.01594	0.00025	55	48.14812	>0.500

These are results for developing high distress in 2016

Main effects

Final estimation of fixed effects: (Unit-specific model)

Fixed Effect	Coefficient	Standard Error	t	Approx. d.f.	p
For INTRCPT1, ß0					
INTRCPT2, ?00	-0.439296	0.038324	-11.463	56	<0.001
WELLPCA, ?01	-0.054957	0.038755	-1.418	56	0.162
For CHOICE slope, ß1					
INTRCPT2, ?10	0.011675	0.035615	0.328	1036	0.743
For BULLY slope, ß2					
INTRCPT2, ?20	0.037461	0.048138	0.778	1036	0.437
For TIME slope, ß3					
INTRCPT2, ?30	-0.020118	0.037026	-0.543	1036	0.587
For DUTIES slope, ß4					
INTRCPT2, ?40	-0.043021	0.038129	-1.128	1036	0.259
For PHYCON15 slope, ß5					
INTRCPT2, ?50	0.068391	0.045290	1.510	1036	0.131
For KESCON15 slope, ß6					
INTRCPT2, ?60	0.283659	0.063150	4.492	1036	<0.001

Fixed Effect	Coefficient	Odds Ratio	Confidence Interval
For INTRCPT1, ß0			
INTRCPT2, ?00	-0.439296	0.644490	(0.597,0.696)
WELLPCA, ?01	-0.054957	0.946525	(0.876,1.023)
For CHOICE slope, ß1			
INTRCPT2, ?10	0.011675	1.011743	(0.943,1.085)
For BULLY slope, ß2			
INTRCPT2, ?20	0.037461	1.038171	(0.945,1.141)
For TIME slope, ß3			
INTRCPT2, ?30	-0.020118	0.980083	(0.911,1.054)
For DUTIES slope, ß4			
INTRCPT2, ?40	-0.043021	0.957891	(0.889,1.032)
For PHYCON15 slope, ß5			
INTRCPT2, ?50	0.068391	1.070784	(0.980,1.170)
For KESCON15 slope, ß6			
INTRCPT2, ?60	0.283659	1.327980	(1.173,1.503)

Final estimation of fixed effects
(Unit-specific model with robust standard errors)

Fixed Effect	Coefficient	Standard Error	t	Approx. d.f.	p

For INTRCPT1, ß0					
INTRCPT2, ?00	-0.439296	0.018566	-23.661	56	<0.001
WELLPCA, ?01	-0.054957	0.023621	-2.327	56	0.024
For CHOICE slope, ß1					
INTRCPT2, ?10	0.011675	0.016440	0.710	1036	0.478
For BULLY slope, ß2					
INTRCPT2, ?20	0.037461	0.028570	1.311	1036	0.190
For TIME slope, ß3					
INTRCPT2, ?30	-0.020118	0.016788	-1.198	1036	0.231
For DUTIES slope, ß4					
INTRCPT2, ?40	-0.043021	0.015883	-2.709	1036	0.007
For PHYCON15 slope, ß5					
INTRCPT2, ?50	0.068391	0.023996	2.850	1036	0.004
For KESCON15 slope, ß6					
INTRCPT2, ?60	0.283659	0.027900	10.167	1036	<0.001

Fixed Effect	Coefficient	Odds Ratio	Confidence Interval
For INTRCPT1, ß0			
INTRCPT2, ?00	-0.439296	0.644490	(0.621,0.669)
WELLPCA, ?01	-0.054957	0.946525	(0.903,0.992)
For CHOICE slope, ß1			
INTRCPT2, ?10	0.011675	1.011743	(0.980,1.045)
For BULLY slope, ß2			
INTRCPT2, ?20	0.037461	1.038171	(0.982,1.098)
For TIME slope, ß3			
INTRCPT2, ?30	-0.020118	0.980083	(0.948,1.013)
For DUTIES slope, ß4			
INTRCPT2, ?40	-0.043021	0.957891	(0.928,0.988)
For PHYCON15 slope, ß5			
INTRCPT2, ?50	0.068391	1.070784	(1.022,1.122)
For KESCON15 slope, ß6			
INTRCPT2, ?60	0.283659	1.327980	(1.257,1.403)

Final estimation of variance components

Random Effect	Standard Deviation	Variance Component	df	Chi-square	p
INTRCPT1, u0	0.01172	0.00014	56	22.28645	>0.500

Curvilinear model

Final estimation of fixed effects: (Unit-specific model)

Fixed Effect	Coefficient	Standard Error	t	Approx. d.f.	p
For INTRCPT1, ß0					
INTRCPT2, ?00	-0.463209	0.041566	-11.144	55	<0.001
WELLPCA, ?01	-0.070521	0.041287	-1.708	55	0.093
WELLPCAS, ?02	-0.055835	0.034992	-1.596	55	0.116
For CHOICE slope, ß1					
INTRCPT2, ?10	0.013836	0.035561	0.389	1036	0.697
For BULLY slope, ß2					
INTRCPT2, ?20	0.032602	0.048281	0.675	1036	0.500
For TIME slope, ß3					
INTRCPT2, ?30	-0.020881	0.037063	-0.563	1036	0.573
For DUTIES slope, ß4					
INTRCPT2, ?40	-0.043618	0.038146	-1.143	1036	0.253
For PHYCON15 slope, ß5					
INTRCPT2, ?50	0.069239	0.045415	1.525	1036	0.128
For KESCON15 slope, ß6					
INTRCPT2, ?60	0.285543	0.063063	4.528	1036	<0.001

Fixed Effect	Coefficient	Odds Ratio	Confidence Interval
For INTRCPT1, ß0			
INTRCPT2, ?00	-0.463209	0.629261	(0.579,0.684)
WELLPCA, ?01	-0.070521	0.931908	(0.858,1.012)
WELLPCAS, ?02	-0.055835	0.945695	(0.882,1.014)
For CHOICE slope, ß1			
INTRCPT2, ?10	0.013836	1.013933	(0.946,1.087)
For BULLY slope, ß2			
INTRCPT2, ?20	0.032602	1.033139	(0.940,1.136)
For TIME slope, ß3			
INTRCPT2, ?30	-0.020881	0.979336	(0.911,1.053)
For DUTIES slope, ß4			
INTRCPT2, ?40	-0.043618	0.957320	(0.888,1.032)
For PHYCON15 slope, ß5			
INTRCPT2, ?50	0.069239	1.071692	(0.980,1.172)
For KESCON15 slope, ß6			
INTRCPT2, ?60	0.285543	1.330484	(1.176,1.506)

Final estimation of fixed effects
(Unit-specific model with robust standard errors)

Fixed Effect	Coefficient	Standard Error	t	Approx. d.f.	p

For INTRCPT1, ß0					
INTRCPT2, ?00	-0.463209	0.022723	-20.385	55	<0.001
WELLPCA, ?01	-0.070521	0.025992	-2.713	55	0.009
WELLPCAS, ?02	-0.055835	0.022919	-2.436	55	0.018
For CHOICE slope, ß1					
INTRCPT2, ?10	0.013836	0.016187	0.855	1036	0.393
For BULLY slope, ß2					
INTRCPT2, ?20	0.032602	0.027298	1.194	1036	0.233
For TIME slope, ß3					
INTRCPT2, ?30	-0.020881	0.016421	-1.272	1036	0.204
For DUTIES slope, ß4					
INTRCPT2, ?40	-0.043618	0.015602	-2.796	1036	0.005
For PHYCON15 slope, ß5					
INTRCPT2, ?50	0.069239	0.023933	2.893	1036	0.004
For KESCON15 slope, ß6					
INTRCPT2, ?60	0.285543	0.028639	9.971	1036	<0.001

Fixed Effect	Coefficient	Odds Ratio	Confidence Interval
For INTRCPT1, ß0			
INTRCPT2, ?00	-0.463209	0.629261	(0.601,0.659)
WELLPCA, ?01	-0.070521	0.931908	(0.885,0.982)
WELLPCAS, ?02	-0.055835	0.945695	(0.903,0.990)
For CHOICE slope, ß1			
INTRCPT2, ?10	0.013836	1.013933	(0.982,1.047)
For BULLY slope, ß2			
INTRCPT2, ?20	0.032602	1.033139	(0.979,1.090)
For TIME slope, ß3			
INTRCPT2, ?30	-0.020881	0.979336	(0.948,1.011)
For DUTIES slope, ß4			
INTRCPT2, ?40	-0.043618	0.957320	(0.928,0.987)
For PHYCON15 slope, ß5			
INTRCPT2, ?50	0.069239	1.071692	(1.023,1.123)
For KESCON15 slope, ß6			
INTRCPT2, ?60	0.285543	1.330484	(1.258,1.407)

Final estimation of variance components

Random Effect	Standard Deviation	Variance Component	df	Chi-square	p
INTRCPT1, u0	0.00941	0.00009	55	20.70177	>0.500

Choice interaction

Final estimation of fixed effects: (Unit-specific model)

Fixed Effect	Coefficient	Standard	t	Approx.p	
		Error		d.f.	
For INTRCPT1, ß0					
INTRCPT2, ?00	-0.439508	0.038222	-11.499	56	<0.001
WELLPCA, ?01	-0.054099	0.038587	-1.402	56	0.166
For CHOICE slope, ß1					
INTRCPT2, ?10	0.019485	0.037256	0.523	56	0.603
WELLPCA, ?11	0.006134	0.037623	0.163	56	0.871
For BULLY slope, ß2					
INTRCPT2, ?20	0.038525	0.048088	0.801	979	0.423
For TIME slope, ß3					
INTRCPT2, ?30	-0.019828	0.036920	-0.537	979	0.591
For DUTIES slope, ß4					
INTRCPT2, ?40	-0.043650	0.038162	-1.144	979	0.253
For PHYCON15 slope, ß5					
INTRCPT2, ?50	0.068422	0.045261	1.512	979	0.131
For KESCON15 slope, ß6					
INTRCPT2, ?60	0.284340	0.063207	4.499	979	<0.001

Fixed Effect	Coefficient	Odds Ratio	Confidence Interval
For INTRCPT1, ß0			
INTRCPT2, ?00	-0.439508	0.644354	(0.597,0.696)
WELLPCA, ?01	-0.054099	0.947338	(0.877,1.024)
For CHOICE slope, ß1			
INTRCPT2, ?10	0.019485	1.019676	(0.946,1.099)
WELLPCA, ?11	0.006134	1.006153	(0.933,1.085)
For BULLY slope, ß2			
INTRCPT2, ?20	0.038525	1.039277	(0.946,1.142)
For TIME slope, ß3			
INTRCPT2, ?30	-0.019828	0.980367	(0.912,1.054)
For DUTIES slope, ß4			
INTRCPT2, ?40	-0.043650	0.957289	(0.888,1.032)
For PHYCON15 slope, ß5			
INTRCPT2, ?50	0.068422	1.070817	(0.980,1.170)
For KESCON15 slope, ß6			
INTRCPT2, ?60	0.284340	1.328884	(1.174,1.504)

Final estimation of fixed effects
(Unit-specific model with robust standard errors)

Fixed Effect	Coefficient	Standard	t	Approx.p

		Error		d.f.		
For INTRCPT1, ß0						
INTRCPT2, ?00	-0.439508	0.018247	-24.087		56	<0.001
WELLPCA, ?01	-0.054099	0.023483	-2.304	56	0.025	
For CHOICE slope, ß1						
INTRCPT2, ?10	0.019485	0.016768	1.162	56	0.250	
WELLPCA, ?11	0.006134	0.016914	0.363	56	0.718	
For BULLY slope, ß2						
INTRCPT2, ?20	0.038525	0.028783	1.338	979	0.181	
For TIME slope, ß3						
INTRCPT2, ?30	-0.019828	0.017051	-1.163	979	0.245	
For DUTIES slope, ß4						
INTRCPT2, ?40	-0.043650	0.015910	-2.744	979	0.006	
For PHYCON15 slope, ß5						
INTRCPT2, ?50	0.068422	0.023913	2.861	979	0.004	
For KESCON15 slope, ß6						
INTRCPT2, ?60	0.284340	0.027657	10.281	979	<0.001	

Fixed Effect	Coefficient	Odds Ratio	Confidence Interval
For INTRCPT1, ß0			
INTRCPT2, ?00	-0.439508	0.644354	(0.621,0.668)
WELLPCA, ?01	-0.054099	0.947338	(0.904,0.993)
For CHOICE slope, ß1			
INTRCPT2, ?10	0.019485	1.019676	(0.986,1.055)
WELLPCA, ?11	0.006134	1.006153	(0.973,1.041)
For BULLY slope, ß2			
INTRCPT2, ?20	0.038525	1.039277	(0.982,1.100)
For TIME slope, ß3			
INTRCPT2, ?30	-0.019828	0.980367	(0.948,1.014)
For DUTIES slope, ß4			
INTRCPT2, ?40	-0.043650	0.957289	(0.928,0.988)
For PHYCON15 slope, ß5			
INTRCPT2, ?50	0.068422	1.070817	(1.022,1.122)
For KESCON15 slope, ß6			
INTRCPT2, ?60	0.284340	1.328884	(1.259,1.403)

Final estimation of variance components

Random Effect	Standard Deviation	Variance Component	df	Chi-square	p
INTRCPT1, u0	0.00448	0.00002	53	21.26146	>0.500
CHOICE slope, u1	0.00441	0.00002	53	17.58960	>0.500

Bully interaction

Fixed Effect	Coefficient	Standard Error	t	Approx. d.f.	p
For INTRCPT1, ß0					
INTRCPT2, ?00	-0.439785	0.038269	-11.492	56	<0.001
WELLPCA, ?01	-0.052319	0.038680	-1.353	56	0.182
For CHOICE slope, ß1					
INTRCPT2, ?10	0.010840	0.035536	0.305	979	0.760
For BULLY slope, ß2					
INTRCPT2, ?20	0.038833	0.050308	0.772	56	0.443
WELLPCA, ?21	-0.034001	0.051380	-0.662	56	0.511
For TIME slope, ß3					
INTRCPT2, ?30	-0.018914	0.036905	-0.513	979	0.608
For DUTIES slope, ß4					
INTRCPT2, ?40	-0.043369	0.038000	-1.141	979	0.254
For PHYCON15 slope, ß5					
INTRCPT2, ?50	0.068627	0.045274	1.516	979	0.130
For KESCON15 slope, ß6					
INTRCPT2, ?60	0.284987	0.063313	4.501	979	<0.001

Fixed Effect	Coefficient	Odds Ratio	Confidence Interval
For INTRCPT1, ß0			
INTRCPT2, ?00	-0.439785	0.644175	(0.597,0.696)
WELLPCA, ?01	-0.052319	0.949026	(0.878,1.026)
For CHOICE slope, ß1			
INTRCPT2, ?10	0.010840	1.010899	(0.943,1.084)
For BULLY slope, ß2			
INTRCPT2, ?20	0.038833	1.039597	(0.940,1.150)
WELLPCA, ?21	-0.034001	0.966571	(0.872,1.071)
For TIME slope, ß3			
INTRCPT2, ?30	-0.018914	0.981263	(0.913,1.055)
For DUTIES slope, ß4			
INTRCPT2, ?40	-0.043369	0.957558	(0.889,1.032)
For PHYCON15 slope, ß5			
INTRCPT2, ?50	0.068627	1.071037	(0.980,1.171)
For KESCON15 slope, ß6			
INTRCPT2, ?60	0.284987	1.329745	(1.174,1.506)

Final estimation of fixed effects
(Unit-specific model with robust standard errors)

Fixed Effect	Coefficient	Standard Error	t	Approx. d.f.	p

For INTRCPT1, ß0					
INTRCPT2, ?00	-0.439785	0.018942	-23.217	56	<0.001
WELLPCA, ?01	-0.052319	0.023903	-2.189	56	0.033
For CHOICE slope, ß1					
INTRCPT2, ?10	0.010840	0.016757	0.647	979	0.518
For BULLY slope, ß2					
INTRCPT2, ?20	0.038833	0.022556	1.722	56	0.091
WELLPCA, ?21	-0.034001	0.023855	-1.425	56	0.160
For TIME slope, ß3					
INTRCPT2, ?30	-0.018914	0.016671	-1.135	979	0.257
For DUTIES slope, ß4					
INTRCPT2, ?40	-0.043369	0.015968	-2.716	979	0.007
For PHYCON15 slope, ß5					
INTRCPT2, ?50	0.068627	0.023847	2.878	979	0.004
For KESCON15 slope, ß6					
INTRCPT2, ?60	0.284987	0.028429	10.024	979	<0.001

Fixed Effect	Coefficient	Odds Ratio	Confidence Interval
For INTRCPT1, ß0			
INTRCPT2, ?00	-0.439785	0.644175	(0.620,0.669)
WELLPCA, ?01	-0.052319	0.949026	(0.905,0.996)
For CHOICE slope, ß1			
INTRCPT2, ?10	0.010840	1.010899	(0.978,1.045)
For BULLY slope, ß2			
INTRCPT2, ?20	0.038833	1.039597	(0.994,1.088)
WELLPCA, ?21	-0.034001	0.966571	(0.921,1.014)
For TIME slope, ß3			
INTRCPT2, ?30	-0.018914	0.981263	(0.950,1.014)
For DUTIES slope, ß4			
INTRCPT2, ?40	-0.043369	0.957558	(0.928,0.988)
For PHYCON15 slope, ß5			
INTRCPT2, ?50	0.068627	1.071037	(1.022,1.122)
For KESCON15 slope, ß6			
INTRCPT2, ?60	0.284987	1.329745	(1.258,1.406)

Random Effect	Standard Deviation	Variance Component	df	Chi-square	p
INTRCPT1, u0	0.00748	0.00006	49	22.17320	>0.500
BULLY slope, u2	0.00988	0.00010	49	16.30252	>0.500

Time pressure interaction

Final estimation of fixed effects: (Unit-specific model)

Fixed Effect	Coefficient	Standard Error	t	Approx. d.f.	p
For INTRCPT1, ß0					
INTRCPT2, ?00	-0.438945	0.038229	-11.482	56	<0.001
WELLPCA, ?01	-0.056517	0.038652	-1.462	56	0.149
For CHOICE slope, ß1					
INTRCPT2, ?10	0.012688	0.035496	0.357	979	0.721
For BULLY slope, ß2					
INTRCPT2, ?20	0.035857	0.048020	0.747	979	0.455
For TIME slope, ß3					
INTRCPT2, ?30	-0.015935	0.038809	-0.411	56	0.683
WELLPCA, ?31	0.006279	0.036297	0.173	56	0.863
For DUTIES slope, ß4					
INTRCPT2, ?40	-0.041694	0.038006	-1.097	979	0.273
For PHYCON15 slope, ß5					
INTRCPT2, ?50	0.067282	0.045184	1.489	979	0.137
For KESCON15 slope, ß6					
INTRCPT2, ?60	0.284073	0.063199	4.495	979	<0.001

Fixed Effect	Coefficient	Odds Ratio	Confidence Interval
For INTRCPT1, ß0			
INTRCPT2, ?00	-0.438945	0.644716	(0.597,0.696)
WELLPCA, ?01	-0.056517	0.945051	(0.875,1.021)
For CHOICE slope, ß1			
INTRCPT2, ?10	0.012688	1.012768	(0.945,1.086)
For BULLY slope, ß2			
INTRCPT2, ?20	0.035857	1.036508	(0.943,1.139)
For TIME slope, ß3			
INTRCPT2, ?30	-0.015935	0.984191	(0.911,1.064)
WELLPCA, ?31	0.006279	1.006299	(0.936,1.082)
For DUTIES slope, ß4			
INTRCPT2, ?40	-0.041694	0.959163	(0.890,1.033)
For PHYCON15 slope, ß5			
INTRCPT2, ?50	0.067282	1.069597	(0.979,1.169)
For KESCON15 slope, ß6			
INTRCPT2, ?60	0.284073	1.328530	(1.174,1.504)

Final estimation of fixed effects
(Unit-specific model with robust standard errors)

Fixed Effect	Coefficient	Standard	t	Approx.p

		Error		d.f.		
For INTRCPT1, ß0						
INTRCPT2, ?00	-0.438945	0.018378	-23.885		56	<0.001
WELLPCA, ?01	-0.056517	0.023812	-2.373	56	0.021	
For CHOICE slope, ß1						
INTRCPT2, ?10	0.012688	0.016541	0.767	979	0.443	
For BULLY slope, ß2						
INTRCPT2, ?20	0.035857	0.028626	1.253	979	0.211	
For TIME slope, ß3						
INTRCPT2, ?30	-0.015935	0.016620	-0.959	56	0.342	
WELLPCA, ?31	0.006279	0.018193	0.345	56	0.731	
For DUTIES slope, ß4						
INTRCPT2, ?40	-0.041694	0.016009	-2.604	979	0.009	
For PHYCON15 slope, ß5						
INTRCPT2, ?50	0.067282	0.024095	2.792	979	0.005	
For KESCON15 slope, ß6						
INTRCPT2, ?60	0.284073	0.027914	10.177	979	<0.001	

Fixed Effect	Coefficient	Odds Ratio	Confidence Interval
For INTRCPT1, ß0			
INTRCPT2, ?00	-0.438945	0.644716	(0.621,0.669)
WELLPCA, ?01	-0.056517	0.945051	(0.901,0.991)
For CHOICE slope, ß1			
INTRCPT2, ?10	0.012688	1.012768	(0.980,1.046)
For BULLY slope, ß2			
INTRCPT2, ?20	0.035857	1.036508	(0.980,1.096)
For TIME slope, ß3			
INTRCPT2, ?30	-0.015935	0.984191	(0.952,1.018)
WELLPCA, ?31	0.006279	1.006299	(0.970,1.044)
For DUTIES slope, ß4			
INTRCPT2, ?40	-0.041694	0.959163	(0.929,0.990)
For PHYCON15 slope, ß5			
INTRCPT2, ?50	0.067282	1.069597	(1.020,1.121)
For KESCON15 slope, ß6			
INTRCPT2, ?60	0.284073	1.328530	(1.258,1.403)

Final estimation of variance components

Random Effect	Standard Deviation	Variance Component	df	Chi-square	p
INTRCPT1, u0	0.00705	0.00005	55	23.58446	>0.500
TIME slope, u3	0.00687	0.00005	55	21.17111	>0.500

Role clarity interaction

Final estimation of fixed effects: (Unit-specific model)

Fixed Effect	Coefficient	Standard Error	t	Approx. d.f.	p
For INTRCPT1, ß0					
INTRCPT2, ?00	-0.439201	0.038214	-11.493	56	<0.001
WELLPCA, ?01	-0.053449	0.038707	-1.381	56	0.173
For CHOICE slope, ß1					
INTRCPT2, ?10	0.011212	0.035596	0.315	979	0.753
For BULLY slope, ß2					
INTRCPT2, ?20	0.040498	0.047993	0.844	979	0.399
For TIME slope, ß3					
INTRCPT2, ?30	-0.017309	0.036868	-0.469	979	0.639
For DUTIES slope, ß4					
INTRCPT2, ?40	-0.033206	0.040132	-0.827	56	0.412
WELLPCA, ?41	-0.012441	0.039523	-0.315	56	0.754
For PHYCON15 slope, ß5					
INTRCPT2, ?50	0.068829	0.045296	1.520	979	0.129
For KESCON15 slope, ß6					
INTRCPT2, ?60	0.284636	0.063124	4.509	979	<0.001

Fixed Effect	Coefficient	Odds Ratio	Confidence Interval
For INTRCPT1, ß0			
INTRCPT2, ?00	-0.439201	0.644551	(0.597,0.696)
WELLPCA, ?01	-0.053449	0.947955	(0.877,1.024)
For CHOICE slope, ß1			
INTRCPT2, ?10	0.011212	1.011275	(0.943,1.084)
For BULLY slope, ß2			
INTRCPT2, ?20	0.040498	1.041329	(0.948,1.144)
For TIME slope, ß3			
INTRCPT2, ?30	-0.017309	0.982840	(0.914,1.057)
For DUTIES slope, ß4			
INTRCPT2, ?40	-0.033206	0.967339	(0.893,1.048)
WELLPCA, ?41	-0.012441	0.987636	(0.912,1.069)
For PHYCON15 slope, ß5			
INTRCPT2, ?50	0.068829	1.071253	(0.980,1.171)
For KESCON15 slope, ß6			
INTRCPT2, ?60	0.284636	1.329278	(1.174,1.505)

Final estimation of fixed effects
(Unit-specific model with robust standard errors)

Fixed Effect	Coefficient	Standard	t	Approx.p

		Error		d.f.	
For INTRCPT1, ß0					
INTRCPT2, ?00	-0.439201	0.018733	-23.445	56	<0.001
WELLPCA, ?01	-0.053449	0.024371	-2.193	56	0.032
For CHOICE slope, ß1					
INTRCPT2, ?10	0.011212	0.016426	0.683	979	0.495
For BULLY slope, ß2					
INTRCPT2, ?20	0.040498	0.028715	1.410	979	0.159
For TIME slope, ß3					
INTRCPT2, ?30	-0.017309	0.016606	-1.042	979	0.298
For DUTIES slope, ß4					
INTRCPT2, ?40	-0.033206	0.015696	-2.116	56	0.039
WELLPCA, ?41	-0.012441	0.014394	-0.864	56	0.391
For PHYCON15 slope, ß5					
INTRCPT2, ?50	0.068829	0.024044	2.863	979	0.004
For KESCON15 slope, ß6					
INTRCPT2, ?60	0.284636	0.027885	10.207	979	<0.001

Fixed Effect	Coefficient	Odds Ratio	Confidence Interval
For INTRCPT1, ß0			
INTRCPT2, ?00	-0.439201	0.644551	(0.621,0.669)
WELLPCA, ?01	-0.053449	0.947955	(0.903,0.995)
For CHOICE slope, ß1			
INTRCPT2, ?10	0.011212	1.011275	(0.979,1.044)
For BULLY slope, ß2			
INTRCPT2, ?20	0.040498	1.041329	(0.984,1.102)
For TIME slope, ß3			
INTRCPT2, ?30	-0.017309	0.982840	(0.951,1.015)
For DUTIES slope, ß4			
INTRCPT2, ?40	-0.033206	0.967339	(0.937,0.998)
WELLPCA, ?41	-0.012441	0.987636	(0.960,1.017)
For PHYCON15 slope, ß5			
INTRCPT2, ?50	0.068829	1.071253	(1.022,1.123)
For KESCON15 slope, ß6			
INTRCPT2, ?60	0.284636	1.329278	(1.258,1.404)

Final estimation of variance components

Random Effect	Standard Deviation	Variance Component	df	Chi-square	p
INTRCPT1, u0	0.00500	0.00002	55	23.53751	>0.500
DUTIES slope, u4	0.00483	0.00002	55	16.18731	>0.500

These are results for developing good physical health in 2016 (i.e., recovery from poor health)

Main effects

Final estimation of fixed effects: (Unit-specific model)

Fixed Effect	Coefficient	Standard Error	t	Approx. d.f.	p
For INTRCPT1, ß0					
INTRCPT2, ?00	0.639086	0.061739	10.351	55	<0.001
WELLPCA, ?01	-0.080109	0.059338	-1.350	55	0.183
For CHOICE slope, ß1					
INTRCPT2, ?10	-0.080533	0.053473	-1.506	1307	0.132
For BULLY slope, ß2					
INTRCPT2, ?20	0.096909	0.093134	1.041	1307	0.298
For TIME slope, ß3					
INTRCPT2, ?30	-0.001167	0.056950	-0.020	1307	0.984
For DUTIES slope, 4					
INTRCPT2, ?40	0.041389	0.063012	0.657	1307	0.511
For PHYCON15 slope, ß5					
INTRCPT2, ?50	1.455259	0.224263	6.489	1307	<0.001
For KESCON15 slope, ß6					
INTRCPT2, ?60	0.273112	0.086984	3.140	1307	0.002

Fixed Effect	Coefficient	Odds Ratio	Confidence Interval
For INTRCPT1, ß0			
INTRCPT2, ?00	0.639086	1.894747	(1.674,2.144)
WELLPCA, ?01	-0.080109	0.923016	(0.819,1.040)
For CHOICE slope, ß1			
INTRCPT2, ?10	-0.080533	0.922625	(0.831,1.025)
For BULLY slope, ß2			
INTRCPT2, ?20	0.096909	1.101760	(0.918,1.323)
For TIME slope, ß3			
INTRCPT2, ?30	-0.001167	0.998834	(0.893,1.117)
For DUTIES slope, ß4			
INTRCPT2, ?40	0.041389	1.042258	(0.921,1.179)
For PHYCON15 slope, ß5			
INTRCPT2, ?50	1.455259	4.285594	(2.760,6.654)
For KESCON15 slope, ß6			
INTRCPT2, ?60	0.273112	1.314048	(1.108,1.559)

Final estimation of fixed effects
(Unit-specific model with robust standard errors)

Fixed Effect	Coefficient	Standard	t	Approx.p

		Error		d.f.	
For INTRCPT1, ß0					
INTRCPT2, ?00	0.639086	0.054259	11.778	55	<0.001
WELLPCA, ?01	-0.080109	0.046224	-1.733	55	0.089
For CHOICE slope, ß1					
INTRCPT2, ?10	-0.080533	0.036071	-2.233	1307	0.026
For BULLY slope, ß2					
INTRCPT2, ?20	0.096909	0.090387	1.072	1307	0.284
For TIME slope, ß3					
INTRCPT2, ?30	-0.001167	0.058883	-0.020	1307	0.984
For DUTIES slope, ß4					
INTRCPT2, ?40	0.041389	0.045816	0.903	1307	0.366
For PHYCON15 slope, ß5					
INTRCPT2, ?50	1.455259	0.257053	5.661	1307	<0.001
For KESCON15 slope, ß6					
INTRCPT2, ?60	0.273112	0.079758	3.424	1307	<0.001

Fixed Effect	Coefficient	Odds Ratio	Confidence Interval
For INTRCPT1, ß0			
INTRCPT2, ?00	0.639086	1.894747	(1.699,2.113)
WELLPCA, ?01	-0.080109	0.923016	(0.841,1.013)
For CHOICE slope, ß1			
INTRCPT2, ?10	-0.080533	0.922625	(0.860,0.990)
For BULLY slope, ß2			
INTRCPT2, ?20	0.096909	1.101760	(0.923,1.316)
For TIME slope, ß3			
INTRCPT2, ?30	-0.001167	0.998834	(0.890,1.121)
For DUTIES slope, ß4			
INTRCPT2, ?40	0.041389	1.042258	(0.953,1.140)
For PHYCON15 slope, ß5			
INTRCPT2, ?50	1.455259	4.285594	(2.588,7.096)
For KESCON15 slope, ß6			
INTRCPT2, ?60	0.273112	1.314048	(1.124,1.537)

Final estimation of variance components

Random Effect	Standard Deviation	Variance Component	df	Chi-square	p
INTRCPT1, u0	0.02612	0.00068	55	43.80595	>0.500

Curvilinear model

Final estimation of fixed effects: (Unit-specific model)

Fixed Effect	Coefficient	Standard Error	t	Approx. d.f.	p
For INTRCPT1, ß0					
INTRCPT2, ?00	0.625949	0.064528	9.700	54	<0.001
WELLPCA, ?01	-0.093895	0.062925	-1.492	54	0.141
WELLPCAS, ?02	-0.033137	0.052082	-0.636	54	0.527
For CHOICE slope, ß1					
INTRCPT2, ?10	-0.077760	0.053652	-1.449	1307	0.147
For BULLY slope, ß2					
INTRCPT2, ?20	0.095324	0.093129	1.024	1307	0.306
For TIME slope, ß3					
INTRCPT2, ?30	-0.002537	0.056977	-0.045	1307	0.964
For DUTIES slope, ß4					
INTRCPT2, ?40	0.039852	0.063046	0.632	1307	0.527
For PHYCON15 slope, ß5					
INTRCPT2, ?50	1.455084	0.224254	6.489	1307	<0.001
For KESCON15 slope, ß6					
INTRCPT2, ?60	0.273619	0.087010	3.145	1307	0.002

Fixed Effect	Coefficient	Odds Ratio	Confidence Interval
For INTRCPT1, ß0			
INTRCPT2, ?00	0.625949	1.870020	(1.643,2.128)
WELLPCA, ?01	-0.093895	0.910379	(0.802,1.033)
WELLPCAS, ?02	-0.033137	0.967406	(0.871,1.074)
For CHOICE slope, ß1			
INTRCPT2, ?10	-0.077760	0.925186	(0.833,1.028)
For BULLY slope, ß2			
INTRCPT2, ?20	0.095324	1.100016	(0.916,1.321)
For TIME slope, ß3			
INTRCPT2, ?30	-0.002537	0.997466	(0.892,1.115)
For DUTIES slope, ß4			
INTRCPT2, ?40	0.039852	1.040657	(0.920,1.178)
For PHYCON15 slope, ß5			
INTRCPT2, ?50	1.455084	4.284842	(2.760,6.653)
For KESCON15 slope, ß6			
INTRCPT2, ?60	0.273619	1.314713	(1.108,1.559)

Final estimation of fixed effects
(Unit-specific model with robust standard errors)

Fixed Effect	Coefficient	Standard	t	Approx.p

		Error		d.f.	
For INTRCPT1, ß0					
INTRCPT2, ?00	0.625949	0.055226	11.334	54	<0.001
WELLPCA, ?01	-0.093895	0.056001	-1.677	54	0.099
WELLPCAS, ?02	-0.033137	0.052434	-0.632	54	0.530
For CHOICE slope, ß1					
INTRCPT2, ?10	-0.077760	0.037029	-2.100	1307	0.036
For BULLY slope, ß2					
INTRCPT2, ?20	0.095324	0.090130	1.058	1307	0.290
For TIME slope, ß3					
INTRCPT2, ?30	-0.002537	0.059481	-0.043	1307	0.966
For DUTIES slope, ß4					
INTRCPT2, ?40	0.039852	0.046559	0.856	1307	0.392
For PHYCON15 slope, ß5					
INTRCPT2, ?50	1.455084	0.256864	5.665	1307	<0.001
For KESCON15 slope, ß6					
INTRCPT2, ?60	0.273619	0.080187	3.412	1307	<0.001

Fixed Effect	Coefficient	Odds Ratio	Confidence Interval
For INTRCPT1, ß0			
INTRCPT2, ?00	0.625949	1.870020	(1.674,2.089)
WELLPCA, ?01	-0.093895	0.910379	(0.814,1.019)
WELLPCAS, ?02	-0.033137	0.967406	(0.871,1.075)
For CHOICE slope, ß1			
INTRCPT2, ?10	-0.077760	0.925186	(0.860,0.995)
For BULLY slope, ß2			
INTRCPT2, ?20	0.095324	1.100016	(0.922,1.313)
For TIME slope, ß3			
INTRCPT2, ?30	-0.002537	0.997466	(0.888,1.121)
For DUTIES slope, ß4			
INTRCPT2, ?40	0.039852	1.040657	(0.950,1.140)
For PHYCON15 slope, ß5			
INTRCPT2, ?50	1.455084	4.284842	(2.589,7.093)
For KESCON15 slope, ß6			
INTRCPT2, ?60	0.273619	1.314713	(1.123,1.539)

Final estimation of variance components

Random Effect	Standard Deviation	Variance Component	df	Chi-square	p
INTRCPT1, u0	0.01407	0.00020	54	43.26008	>0.500

Control interaction

Final estimation of fixed effects: (Unit-specific model)

Fixed Effect	Coefficient	Standard Error	t	Approx. d.f.	p
For INTRCPT1, ß0					
INTRCPT2, ?00	0.639142	0.061627	10.371	55	<0.001
WELLPCA, ?01	-0.081898	0.059409	-1.379	55	0.174
For CHOICE slope, ß1					
INTRCPT2, ?10	-0.071572	0.055788	-1.283	55	0.205
WELLPCA, ?11	0.042324	0.056987	0.743	55	0.461
For BULLY slope, ß2					
INTRCPT2, ?20	0.104282	0.092847	1.123	1251	0.262
For TIME slope, ß3					
INTRCPT2, ?30	0.000149	0.056895	0.003	1251	0.998
For DUTIES slope, ß4					
INTRCPT2, ?40	0.038500	0.063066	0.610	1251	0.542
For PHYCON15 slope, ß5					
INTRCPT2, ?50	1.456789	0.224199	6.498	1251	<0.001
For KESCON15 slope, ß6					
INTRCPT2, ?60	0.272105	0.086992	3.128	1251	0.002

Fixed Effect	Coefficient	Odds Ratio	Confidence Interval
For INTRCPT1, ß0			
INTRCPT2, ?00	0.639142	1.894854	(1.675,2.144)
WELLPCA, ?01	-0.081898	0.921366	(0.818,1.038)
For CHOICE slope, ß1			
INTRCPT2, ?10	-0.071572	0.930929	(0.832,1.041)
WELLPCA, ?11	0.042324	1.043232	(0.931,1.170)
For BULLY slope, ß2			
INTRCPT2, ?20	0.104282	1.109914	(0.925,1.332)
For TIME slope, ß3			
INTRCPT2, ?30	0.000149	1.000149	(0.895,1.118)
For DUTIES slope, ß4			
INTRCPT2, ?40	0.038500	1.039251	(0.918,1.176)
For PHYCON15 slope, ß5			
INTRCPT2, ?50	1.456789	4.292156	(2.765,6.664)
For KESCON15 slope, ß6			
INTRCPT2, ?60	0.272105	1.312725	(1.107,1.557)

Final estimation of fixed effects
(Unit-specific model with robust standard errors)

Fixed Effect	Coefficient	Standard Error	t	Approx. d.f.	p

For INTRCPT1, ß0					
INTRCPT2, ?00	0.639142	0.053871	11.864	55	<0.001
WELLPCA, ?01	-0.081898	0.046850	-1.748	55	0.086
For CHOICE slope, ß1					
INTRCPT2, ?10	-0.071572	0.039189	-1.826	55	0.073
WELLPCA, ?11	0.042324	0.041014	1.032	55	0.307
For BULLY slope, ß2					
INTRCPT2, ?20	0.104282	0.090467	1.153	1251	0.249
For TIME slope, ß3					
INTRCPT2, ?30	0.000149	0.059335	0.003	1251	0.998
For DUTIES slope, ß4					
INTRCPT2, ?40	0.038500	0.045808	0.840	1251	0.401
For PHYCON15 slope, ß5					
INTRCPT2, ?50	1.456789	0.258775	5.630	1251	<0.001
For KESCON15 slope, ß6					
INTRCPT2, ?60	0.272105	0.079817	3.409	1251	<0.001

Fixed Effect	Coefficient	Odds Ratio	Confidence Interval
For INTRCPT1, ß0			
INTRCPT2, ?00	0.639142	1.894854	(1.701,2.111)
WELLPCA, ?01	-0.081898	0.921366	(0.839,1.012)
For CHOICE slope, ß1			
INTRCPT2, ?10	-0.071572	0.930929	(0.861,1.007)
WELLPCA, ?11	0.042324	1.043232	(0.961,1.133)
For BULLY slope, ß2			
INTRCPT2, ?20	0.104282	1.109914	(0.929,1.325)
For TIME slope, ß3			
INTRCPT2, ?30	0.000149	1.000149	(0.890,1.124)
For DUTIES slope, ß4			
INTRCPT2, ?40	0.038500	1.039251	(0.950,1.137)
For PHYCON15 slope, ß5			
INTRCPT2, ?50	1.456789	4.292156	(2.583,7.131)
For KESCON15 slope, ß6			
INTRCPT2, ?60	0.272105	1.312725	(1.122,1.535)

Final estimation of variance components

Random Effect	Standard Deviation	Variance Component	df	Chi-square	p
INTRCPT1, u0	0.02070	0.00043	55	44.19242	>0.500
CHOICE slope, u1	0.01544	0.00024	55	55.99530	0.437

Bullying interaction

Final estimation of fixed effects: (Unit-specific model)

Fixed Effect	Coefficient	Standard Error	t	Approx. d.f.	p
For INTRCPT1, ß0					
INTRCPT2, ?00	0.641307	0.061820	10.374	55	<0.001
WELLPCA, ?01	-0.081199	0.059514	-1.364	55	0.178
For CHOICE slope, ß1					
INTRCPT2, ?10	-0.083734	0.053386	-1.568	1251	0.117
For BULLY slope, ß2					
INTRCPT2, ?20	0.104842	0.103532	1.013	55	0.316
WELLPCA, ?21	-0.117709	0.105856	-1.112	55	0.271
For TIME slope, ß3					
INTRCPT2, ?30	0.000212	0.056950	0.004	1251	0.997
For DUTIES slope, ß4					
INTRCPT2, ?40	0.036860	0.063051	0.585	1251	0.559
For PHYCON15 slope, ß5					
INTRCPT2, ?50	1.452381	0.224390	6.473	1251	<0.001
For KESCON15 slope, ß6					
INTRCPT2, ?60	0.273836	0.087081	3.145	1251	0.002

Fixed Effect	Coefficient	Odds Ratio	Confidence Interval
For INTRCPT1, ß0			
INTRCPT2, ?00	0.641307	1.898961	(1.678,2.150)
WELLPCA, ?01	-0.081199	0.922010	(0.818,1.039)
For CHOICE slope, ß1			
INTRCPT2, ?10	-0.083734	0.919676	(0.828,1.021)
For BULLY slope, ß2			
INTRCPT2, ?20	0.104842	1.110535	(0.902,1.367)
WELLPCA, ?21	-0.117709	0.888955	(0.719,1.099)
For TIME slope, ß3			
INTRCPT2, ?30	0.000212	1.000212	(0.894,1.118)
For DUTIES slope, ß4			
INTRCPT2, ?40	0.036860	1.037547	(0.917,1.174)
For PHYCON15 slope, ß5			
INTRCPT2, ?50	1.452381	4.273277	(2.751,6.637)
For KESCON15 slope, ß6			
INTRCPT2, ?60	0.273836	1.315000	(1.108,1.560)

Final estimation of fixed effects
(Unit-specific model with robust standard errors)

Fixed Effect	Coefficient	Standard	t	Approx.p

		Error		d.f.	
For INTRCPT1, ß0					
INTRCPT2, ?00	0.641307	0.053553	11.975	55	<0.001
WELLPCA, ?01	-0.081199	0.046410	-1.750	55	0.086
For CHOICE slope, ß1					
INTRCPT2, ?10	-0.083734	0.036948	-2.266	1251	0.024
For BULLY slope, ß2					
INTRCPT2, ?20	0.104842	0.104828	1.000	55	0.322
WELLPCA, ?21	-0.117709	0.099507	-1.183	55	0.242
For TIME slope, ß3					
INTRCPT2, ?30	0.000212	0.058107	0.004	1251	0.997
For DUTIES slope, ß4					
INTRCPT2, ?40	0.036860	0.045416	0.812	1251	0.417
For PHYCON15 slope, ß5					
INTRCPT2, ?50	1.452381	0.255456	5.685	1251	<0.001
For KESCON15 slope, ß6					
INTRCPT2, ?60	0.273836	0.081875	3.345	1251	<0.001

Fixed Effect	Coefficient	Odds Ratio	Confidence Interval
For INTRCPT1, ß0			
INTRCPT2, ?00	0.641307	1.898961	(1.706,2.114)
WELLPCA, ?01	-0.081199	0.922010	(0.840,1.012)
For CHOICE slope, ß1			
INTRCPT2, ?10	-0.083734	0.919676	(0.855,0.989)
For BULLY slope, ß2			
INTRCPT2, ?20	0.104842	1.110535	(0.900,1.370)
WELLPCA, ?21	-0.117709	0.888955	(0.728,1.085)
For TIME slope, ß3			
INTRCPT2, ?30	0.000212	1.000212	(0.892,1.121)
For DUTIES slope, ß4			
INTRCPT2, ?40	0.036860	1.037547	(0.949,1.134)
For PHYCON15 slope, ß5			
INTRCPT2, ?50	1.452381	4.273277	(2.589,7.054)
For KESCON15 slope, ß6			
INTRCPT2, ?60	0.273836	1.315000	(1.120,1.544)

Final estimation of variance components

Random Effect	Standard Deviation	Variance Component	df	Chi-square	p
INTRCPT1, u0	0.02629	0.00069	44	30.16969	>0.500
BULLY slope, u2	0.07368	0.00543	44	51.50461	0.204

This is time pressure interaction

Final estimation of fixed effects: (Unit-specific model)

Fixed Effect	Coefficient	Standard Error	t	Approx. d.f.	p
For INTRCPT1, ß0					
INTRCPT2, ?00	0.645305	0.062168	10.380	55	<0.001
WELLPCA, ?01	-0.077801	0.059623	-1.305	55	0.197
For CHOICE slope, ß1					
INTRCPT2, ?10	-0.079381	0.053543	-1.483	1251	0.138
For BULLY slope, ß2					
INTRCPT2, ?20	0.097628	0.093232	1.047	1251	0.295
For TIME slope, ß3					
INTRCPT2, ?30	-0.026786	0.064062	-0.418	55	0.677
WELLPCA, ?31	0.014480	0.058533	0.247	55	0.806
For DUTIES slope, ß4					
INTRCPT2, ?40	0.039622	0.063161	0.627	1251	0.531
For PHYCON15 slope, ß5					
INTRCPT2, ?50	1.460550	0.224621	6.502	1251	<0.001
For KESCON15 slope, ß6					
INTRCPT2, ?60	0.276827	0.087210	3.174	1251	0.002

Fixed Effect	Coefficient	Odds Ratio	Confidence Interval
For INTRCPT1, ß0			
INTRCPT2, ?00	0.645305	1.906568	(1.683,2.160)
WELLPCA, ?01	-0.077801	0.925148	(0.821,1.043)
For CHOICE slope, ß1			
INTRCPT2, ?10	-0.079381	0.923688	(0.832,1.026)
For BULLY slope, ß2			
INTRCPT2, ?20	0.097628	1.102552	(0.918,1.324)
For TIME slope, ß3			
INTRCPT2, ?30	-0.026786	0.973569	(0.856,1.107)
WELLPCA, ?31	0.014480	1.014585	(0.902,1.141)
For DUTIES slope, ß4			
INTRCPT2, ?40	0.039622	1.040418	(0.919,1.178)
For PHYCON15 slope, ß5			
INTRCPT2, ?50	1.460550	4.308327	(2.773,6.694)
For KESCON15 slope, ß6			
INTRCPT2, ?60	0.276827	1.318938	(1.112,1.565)

Final estimation of fixed effects
(Unit-specific model with robust standard errors)

Fixed Effect	Coefficient	Standard Error	t	Approx. d.f.	p

For INTRCPT1, ß0					
INTRCPT2, ?00	0.645305	0.053360	12.093	55	<0.001
WELLPCA, ?01	-0.077801	0.046016	-1.691	55	0.097
For CHOICE slope, ß1					
INTRCPT2, ?10	-0.079381	0.036761	-2.159	1251	0.031
For BULLY slope, ß2					
INTRCPT2, ?20	0.097628	0.091058	1.072	1251	0.284
For TIME slope, ß3					
INTRCPT2, ?30	-0.026786	0.060419	-0.443	55	0.659
WELLPCA, ?31	0.014480	0.060829	0.238	55	0.813
For DUTIES slope, ß4					
INTRCPT2, ?40	0.039622	0.046157	0.858	1251	0.391
For PHYCON15 slope, ß5					
INTRCPT2, ?50	1.460550	0.252619	5.782	1251	<0.001
For KESCON15 slope, ß6					
INTRCPT2, ?60	0.276827	0.081181	3.410	1251	<0.001

Fixed Effect	Coefficient	Odds Ratio	Confidence Interval
For INTRCPT1, ß0			
INTRCPT2, ?00	0.645305	1.906568	(1.713,2.122)
WELLPCA, ?01	-0.077801	0.925148	(0.844,1.015)
For CHOICE slope, ß1			
INTRCPT2, ?10	-0.079381	0.923688	(0.859,0.993)
For BULLY slope, ß2			
INTRCPT2, ?20	0.097628	1.102552	(0.922,1.318)
For TIME slope, ß3			
INTRCPT2, ?30	-0.026786	0.973569	(0.862,1.099)
WELLPCA, ?31	0.014480	1.014585	(0.898,1.146)
For DUTIES slope, ß4			
INTRCPT2, ?40	0.039622	1.040418	(0.950,1.139)
For PHYCON15 slope, ß5			
INTRCPT2, ?50	1.460550	4.308327	(2.625,7.072)
For KESCON15 slope, ß6			
INTRCPT2, ?60	0.276827	1.318938	(1.125,1.547)

Random Effect	Standard Deviation	Variance Component	df	Chi-square	p
INTRCPT1, u0	0.03956	0.00156	54	42.30932	>0.500
TIME slope, u3	0.10000	0.01000	54	51.57999	>0.500

This is role clarity interaction

Final estimation of fixed effects: (Unit-specific model)

Fixed Effect	Coefficient	Standard Error	t	Approx. d.f.	p
For INTRCPT1, ß0					
INTRCPT2, ?00	0.639551	0.061604	10.382	55	<0.001
WELLPCA, ?01	-0.081219	0.059247	-1.371	55	0.176
For CHOICE slope, ß1					
INTRCPT2, ?10	-0.082029	0.053531	-1.532	1251	0.126
For BULLY slope, ß2					
INTRCPT2, ?20	0.097192	0.093217	1.043	1251	0.297
For TIME slope, ß3					
INTRCPT2, ?30	0.003058	0.056988	0.054	1251	0.957
For DUTIES slope, ß4					
INTRCPT2, ?40	0.075186	0.065101	1.155	55	0.253
WELLPCA, ?41	-0.031686	0.062252	-0.509	55	0.613
For PHYCON15 slope, ß5					
INTRCPT2, ?50	1.454278	0.224383	6.481	1251	<0.001
For KESCON15 slope, ß6					
INTRCPT2, ?60	0.280522	0.086939	3.227	1251	0.001

Fixed Effect	Coefficient	Odds Ratio	Confidence Interval
For INTRCPT1, ß0			
INTRCPT2, ?00	0.639551	1.895629	(1.675,2.145)
WELLPCA, ?01	-0.081219	0.921992	(0.819,1.038)
For CHOICE slope, ß1			
INTRCPT2, ?10	-0.082029	0.921245	(0.829,1.023)
For BULLY slope, ß2			
INTRCPT2, ?20	0.097192	1.102072	(0.918,1.323)
For TIME slope, ß3			
INTRCPT2, ?30	0.003058	1.003063	(0.897,1.122)
For DUTIES slope, ß4			
INTRCPT2, ?40	0.075186	1.078085	(0.946,1.228)
WELLPCA, ?41	-0.031686	0.968810	(0.855,1.098)
For PHYCON15 slope, ß5			
INTRCPT2, ?50	1.454278	4.281392	(2.757,6.649)
For KESCON15 slope, ß6			
INTRCPT2, ?60	0.280522	1.323821	(1.116,1.570)

Final estimation of fixed effects
(Unit-specific model with robust standard errors)

Fixed Effect	Coefficient	Standard	t	Approx.p

		Error		d.f.	
For INTRCPT1, ß0					
INTRCPT2, ?00	0.639551	0.054533	11.728	55	<0.001
WELLPCA, ?01	-0.081219	0.045707	-1.777	55	0.081
For CHOICE slope, ß1					
INTRCPT2, ?10	-0.082029	0.035933	-2.283	1251	0.023
For BULLY slope, ß2					
INTRCPT2, ?20	0.097192	0.090384	1.075	1251	0.282
For TIME slope, ß3					
INTRCPT2, ?30	0.003058	0.058896	0.052	1251	0.959
For DUTIES slope, ß4					
INTRCPT2, ?40	0.075186	0.049374	1.523	55	0.134
WELLPCA, ?41	-0.031686	0.041886	-0.756	55	0.453
For PHYCON15 slope, ß5					
INTRCPT2, ?50	1.454278	0.256964	5.659	1251	<0.001
For KESCON15 slope, ß6					
INTRCPT2, ?60	0.280522	0.079718	3.519	1251	<0.001

Fixed Effect	Coefficient	Odds Ratio	Confidence Interval
For INTRCPT1, ß0			
INTRCPT2, ?00	0.639551	1.895629	(1.699,2.115)
WELLPCA, ?01	-0.081219	0.921992	(0.841,1.010)
For CHOICE slope, ß1			
INTRCPT2, ?10	-0.082029	0.921245	(0.859,0.989)
For BULLY slope, ß2			
INTRCPT2, ?20	0.097192	1.102072	(0.923,1.316)
For TIME slope, ß3			
INTRCPT2, ?30	0.003058	1.003063	(0.894,1.126)
For DUTIES slope, ß4			
INTRCPT2, ?40	0.075186	1.078085	(0.976,1.190)
WELLPCA, ?41	-0.031686	0.968810	(0.891,1.054)
For PHYCON15 slope, ß5			
INTRCPT2, ?50	1.454278	4.281392	(2.586,7.088)
For KESCON15 slope, ß6			
INTRCPT2, ?60	0.280522	1.323821	(1.132,1.548)

Final estimation of variance components

Random Effect	Standard Deviation	Variance Component	df	Chi-square	p
INTRCPT1, u0	0.01864	0.00035	55	45.06786	>0.500
DUTIES slope, u4	0.01489	0.00022	55	59.34633	0.320

These are results for developing poor physical health in 2016 (i.e. recovery from high distress)

Linear model

Final estimation of fixed effects: (Unit-specific model)

Fixed Effect	Coefficient	Standard Error	t	Approx. d.f.	p
For INTRCPT1, ß0					
INTRCPT2, ?00	-2.415673	0.073632	-32.807	56	<0.001
WELLPCA, ?01	-0.184605	0.055389	-3.333	56	0.002
For CHOICE slope, ß1					
INTRCPT2, ?10	-0.121033	0.045653	-2.651	5005	0.008
For BULLY slope, ß2					
INTRCPT2, ?20	0.063853	0.087589	0.729	5005	0.466
For TIME slope, ß3					
INTRCPT2, ?30	-0.062788	0.048097	-1.305	5005	0.192
For DUTIES slope, ß4					
INTRCPT2, ?40	-0.055760	0.050899	-1.096	5005	0.273
For PHYCON15 slope, ß5					
INTRCPT2, ?50	1.999662	0.161122	12.411	5005	<0.001
For KESCON15 slope, ß6					
INTRCPT2, ?60	0.421297	0.077476	5.438	5005	<0.001

Fixed Effect	Coefficient	Odds Ratio	Confidence Interval
For INTRCPT1, ß0			
INTRCPT2, ?00	-2.415673	0.089307	(0.077,0.104)
WELLPCA, ?01	-0.184605	0.831432	(0.744,0.929)
For CHOICE slope, ß1			
INTRCPT2, ?10	-0.121033	0.886004	(0.810,0.969)
For BULLY slope, ß2			
INTRCPT2, ?20	0.063853	1.065936	(0.898,1.266)
For TIME slope, ß3			
INTRCPT2, ?30	-0.062788	0.939142	(0.855,1.032)
For DUTIES slope, ß4			
INTRCPT2, ?40	-0.055760	0.945766	(0.856,1.045)
For PHYCON15 slope, ß5			
INTRCPT2, ?50	1.999662	7.386557	(5.386,10.130)
For KESCON15 slope, ß6			
INTRCPT2, ?60	0.421297	1.523938	(1.309,1.774)

Final estimation of fixed effects
(Unit-specific model with robust standard errors)

Fixed Effect   Coefficient   Standard   t   Approx.p

		Error		d.f.	
For INTRCPT1, ß0					
INTRCPT2, ?00	-2.415673	0.070058	-34.481	56	<0.001
WELLPCA, ?01	-0.184605	0.039134	-4.717	56	<0.001
For CHOICE slope, ß1					
INTRCPT2, ?10	-0.121033	0.042661	-2.837	5005	0.005
For BULLY slope, ß2					
INTRCPT2, ?20	0.063853	0.123104	0.519	5005	0.604
For TIME slope, ß3					
INTRCPT2, ?30	-0.062788	0.035977	-1.745	5005	0.081
For DUTIES slope, ß4					
INTRCPT2, ?40	-0.055760	0.044387	-1.256	5005	0.209
For PHYCON15 slope, ß5					
INTRCPT2, ?50	1.999662	0.153428	13.033	5005	<0.001
For KESCON15 slope, ß6					
INTRCPT2, ?60	0.421297	0.062582	6.732	5005	<0.001

Fixed Effect	Coefficient	Odds Ratio	Confidence Interval
For INTRCPT1, ß0			
INTRCPT2, ?00	-2.415673	0.089307	(0.078,0.103)
WELLPCA, ?01	-0.184605	0.831432	(0.769,0.899)
For CHOICE slope, ß1			
INTRCPT2, ?10	-0.121033	0.886004	(0.815,0.963)
For BULLY slope, ß2			
INTRCPT2, ?20	0.063853	1.065936	(0.837,1.357)
For TIME slope, ß3			
INTRCPT2, ?30	-0.062788	0.939142	(0.875,1.008)
For DUTIES slope, ß4			
INTRCPT2, ?40	-0.055760	0.945766	(0.867,1.032)
For PHYCON15 slope, ß5			
INTRCPT2, ?50	1.999662	7.386557	(5.468,9.978)
For KESCON15 slope, ß6			
INTRCPT2, ?60	0.421297	1.523938	(1.348,1.723)

Final estimation of variance components

Random Effect	Standard Deviation	Variance Component	df	Chi-square	p
INTRCPT1, u0	0.18818	0.03541	56	67.75345	0.135

```
This is curvilinear model

Final estimation of fixed effects: (Unit-specific model)
Fixed Effect        Coefficient  Standard     t      Approx.p
                                 Error               d.f.

For INTRCPT1, ß0
    INTRCPT2, ?00   -2.431661    0.074717     -32.545       55     <0.001
     WELLPCA, ?01   -0.204323    0.058709     -3.480 55     <0.001
    WELLPCAS, ?02   -0.066750    0.047356     -1.410 55     0.164
For CHOICE slope, ß1
    INTRCPT2, ?10   -0.117841    0.045671     -2.580 5005   0.010
For BULLY slope, ß2
    INTRCPT2, ?20   0.061323     0.087600     0.700  5005   0.484
For TIME slope, ß3
    INTRCPT2, ?30   -0.064256    0.048123     -1.335 5005   0.182
For DUTIES slope, ß4
    INTRCPT2, ?40   -0.055596    0.050889     -1.092 5005   0.275
For PHYCON15 slope, ß5
    INTRCPT2, ?50   1.995520     0.161148     12.383 5005   <0.001
For KESCON15 slope, ß6
    INTRCPT2, ?60   0.420519     0.077461     5.429  5005   <0.001

Fixed Effect        Coefficient   Odds Ratio   Confidence Interval
For INTRCPT1, ß0
    INTRCPT2, ?00   -2.431661    0.087891     (0.076,0.102)
     WELLPCA, ?01   -0.204323    0.815199     (0.725,0.917)
    WELLPCAS, ?02   -0.066750    0.935429     (0.851,1.029)
For CHOICE slope, ß1
    INTRCPT2, ?10   -0.117841    0.888837     (0.813,0.972)
For BULLY slope, ß2
    INTRCPT2, ?20   0.061323     1.063242     (0.896,1.262)
For TIME slope, ß3
    INTRCPT2, ?30   -0.064256    0.937765     (0.853,1.031)
For DUTIES slope, ß4
    INTRCPT2, ?40   -0.055596    0.945921     (0.856,1.045)
For PHYCON15 slope, ß5
    INTRCPT2, ?50   1.995520     7.356029     (5.364,10.088)
For KESCON15 slope, ß6
    INTRCPT2, ?60   0.420519     1.522751     (1.308,1.772)

Final estimation of fixed effects
(Unit-specific model with robust standard errors)
Fixed Effect        Coefficient  Standard     t      Approx.p
```

		Error		d.f.	
For INTRCPT1, ß0					
INTRCPT2, ?00	-2.431661	0.066103	-36.786	55	<0.001
WELLPCA, ?01	-0.204323	0.043101	-4.741	55	<0.001
WELLPCAS, ?02	-0.066750	0.043001	-1.552	55	0.126
For CHOICE slope, ß1					
INTRCPT2, ?10	-0.117841	0.043250	-2.725	5005	0.006
For BULLY slope, ß2					
INTRCPT2, ?20	0.061323	0.122698	0.500	5005	0.617
For TIME slope, ß3					
INTRCPT2, ?30	-0.064256	0.035719	-1.799	5005	0.072
For DUTIES slope, ß4					
INTRCPT2, ?40	-0.055596	0.044435	-1.251	5005	0.211
For PHYCON15 slope, ß5					
INTRCPT2, ?50	1.995520	0.153336	13.014	5005	<0.001
For KESCON15 slope, ß6					
INTRCPT2, ?60	0.420519	0.062839	6.692	5005	<0.001

Fixed Effect	Coefficient	Odds Ratio	Confidence Interval
For INTRCPT1, ß0			
INTRCPT2, ?00	-2.431661	0.087891	(0.077,0.100)
WELLPCA, ?01	-0.204323	0.815199	(0.748,0.889)
WELLPCAS, ?02	-0.066750	0.935429	(0.858,1.020)
For CHOICE slope, ß1			
INTRCPT2, ?10	-0.117841	0.888837	(0.817,0.967)
For BULLY slope, ß2			
INTRCPT2, ?20	0.061323	1.063242	(0.836,1.352)
For TIME slope, ß3			
INTRCPT2, ?30	-0.064256	0.937765	(0.874,1.006)
For DUTIES slope, ß4			
INTRCPT2, ?40	-0.055596	0.945921	(0.867,1.032)
For PHYCON15 slope, ß5			
INTRCPT2, ?50	1.995520	7.356029	(5.447,9.935)
For KESCON15 slope, ß6			
INTRCPT2, ?60	0.420519	1.522751	(1.346,1.722)

Final estimation of variance components

Random Effect	Standard Deviation	Variance Component	df	Chi-square	p
INTRCPT1, u0	0.18578	0.03452	55	66.20465	0.143

Control interaction

Final estimation of fixed effects: (Unit-specific model)

Fixed Effect	Coefficient	Standard Error	t	Approx. d.f.	p
For INTRCPT1, ß0					
INTRCPT2, ?00	-2.419984	0.075763	-31.941	56	<0.001
WELLPCA, ?01	-0.175356	0.057703	-3.039	56	0.004
For CHOICE slope, ß1					
INTRCPT2, ?10	-0.118805	0.048688	-2.440	56	0.018
WELLPCA, ?11	0.068537	0.049009	1.398	56	0.167
For BULLY slope, ß2					
INTRCPT2, ?20	0.063806	0.087652	0.728	4948	0.467
For TIME slope, ß3					
INTRCPT2, ?30	-0.062339	0.048148	-1.295	4948	0.195
For DUTIES slope, ß4					
INTRCPT2, ?40	-0.056625	0.051041	-1.109	4948	0.267
For PHYCON15 slope, ß5					
INTRCPT2, ?50	2.002902	0.161181	12.426	4948	<0.001
For KESCON15 slope, ß6					
INTRCPT2, ?60	0.423342	0.077612	5.455	4948	<0.001

Fixed Effect	Coefficient	Odds Ratio	Confidence Interval
For INTRCPT1, ß0			
INTRCPT2, ?00	-2.419984	0.088923	(0.076,0.104)
WELLPCA, ?01	-0.175356	0.839158	(0.748,0.942)
For CHOICE slope, ß1			
INTRCPT2, ?10	-0.118805	0.887981	(0.805,0.979)
WELLPCA, ?11	0.068537	1.070940	(0.971,1.181)
For BULLY slope, ß2			
INTRCPT2, ?20	0.063806	1.065885	(0.898,1.266)
For TIME slope, ß3			
INTRCPT2, ?30	-0.062339	0.939564	(0.855,1.033)
For DUTIES slope, ß4			
INTRCPT2, ?40	-0.056625	0.944948	(0.855,1.044)
For PHYCON15 slope, ß5			
INTRCPT2, ?50	2.002902	7.410534	(5.403,10.164)
For KESCON15 slope, ß6			
INTRCPT2, ?60	0.423342	1.527056	(1.312,1.778)

Final estimation of fixed effects
(Unit-specific model with robust standard errors)

Fixed Effect Coefficient Standard t Approx.p

		Error		d.f.	
For INTRCPT1, ß0					
INTRCPT2, ?00	-2.419984	0.071385	-33.900	56	<0.001
WELLPCA, ?01	-0.175356	0.042197	-4.156	56	<0.001
For CHOICE slope, ß1					
INTRCPT2, ?10	-0.118805	0.045119	-2.633	56	0.011
WELLPCA, ?11	0.068537	0.047075	1.456	56	0.151
For BULLY slope, ß2					
INTRCPT2, ?20	0.063806	0.124245	0.514	4948	0.608
For TIME slope, ß3					
INTRCPT2, ?30	-0.062339	0.035956	-1.734	4948	0.083
For DUTIES slope, ß4					
INTRCPT2, ?40	-0.056625	0.044417	-1.275	4948	0.202
For PHYCON15 slope, ß5					
INTRCPT2, ?50	2.002902	0.152885	13.101	4948	<0.001
For KESCON15 slope, ß6					
INTRCPT2, ?60	0.423342	0.063287	6.689	4948	<0.001

Fixed Effect	Coefficient	Odds Ratio	Confidence Interval
For INTRCPT1, ß0			
INTRCPT2, ?00	-2.419984	0.088923	(0.077,0.103)
WELLPCA, ?01	-0.175356	0.839158	(0.771,0.913)
For CHOICE slope, ß1			
INTRCPT2, ?10	-0.118805	0.887981	(0.811,0.972)
WELLPCA, ?11	0.068537	1.070940	(0.975,1.177)
For BULLY slope, ß2			
INTRCPT2, ?20	0.063806	1.065885	(0.836,1.360)
For TIME slope, ß3			
INTRCPT2, ?30	-0.062339	0.939564	(0.876,1.008)
For DUTIES slope, ß4			
INTRCPT2, ?40	-0.056625	0.944948	(0.866,1.031)
For PHYCON15 slope, ß5			
INTRCPT2, ?50	2.002902	7.410534	(5.492,10.000)
For KESCON15 slope, ß6			
INTRCPT2, ?60	0.423342	1.527056	(1.349,1.729)

Final estimation of variance components

Random Effect	Standard Deviation	Variance Component	df	Chi-square	p
INTRCPT1, u0	0.21785	0.04746	56	71.61313	0.078
CHOICE slope, u1	0.06746	0.00455	56	46.12938	>0.500

This is bullying interaction

Final estimation of fixed effects: (Unit-specific model)

Fixed Effect	Coefficient	Standard Error	t	Approx. d.f.	p
For INTRCPT1, ß0					
INTRCPT2, ?00	-2.416783	0.070363	-34.347	56	<0.001
WELLPCA, ?01	-0.186004	0.052287	-3.557	56	<0.001
For CHOICE slope, ß1					
INTRCPT2, ?10	-0.122648	0.045617	-2.689	4948	0.007
For BULLY slope, ß2					
INTRCPT2, ?20	0.052693	0.109281	0.482	56	0.632
WELLPCA, ?21	-0.117058	0.113968	-1.027	56	0.309
For TIME slope, ß3					
INTRCPT2, ?30	-0.064449	0.048064	-1.341	4948	0.180
For DUTIES slope, ß4					
INTRCPT2, ?40	-0.053944	0.050797	-1.062	4948	0.288
For PHYCON15 slope, ß5					
INTRCPT2, ?50	2.003516	0.161230	12.426	4948	<0.001
For KESCON15 slope, ß6					
INTRCPT2, ?60	0.423353	0.077580	5.457	4948	<0.001

Fixed Effect	Coefficient	Odds Ratio	Confidence Interval
For INTRCPT1, ß0			
INTRCPT2, ?00	-2.416783	0.089208	(0.077,0.103)
WELLPCA, ?01	-0.186004	0.830270	(0.748,0.922)
For CHOICE slope, ß1			
INTRCPT2, ?10	-0.122648	0.884575	(0.809,0.967)
For BULLY slope, ß2			
INTRCPT2, ?20	0.052693	1.054106	(0.847,1.312)
WELLPCA, ?21	-0.117058	0.889534	(0.708,1.118)
For TIME slope, ß3			
INTRCPT2, ?30	-0.064449	0.937584	(0.853,1.030)
For DUTIES slope, ß4			
INTRCPT2, ?40	-0.053944	0.947485	(0.858,1.047)
For PHYCON15 slope, ß5			
INTRCPT2, ?50	2.003516	7.415079	(5.406,10.171)
For KESCON15 slope, ß6			
INTRCPT2, ?60	0.423353	1.527073	(1.312,1.778)

Final estimation of fixed effects
(Unit-specific model with robust standard errors)

Fixed Effect	Coefficient	Standard Error	t	Approx. d.f.	p

For INTRCPT1, ß0					
INTRCPT2, ?00	-2.416783	0.068280	-35.395	56	<0.001
WELLPCA, ?01	-0.186004	0.036921	-5.038	56	<0.001
For CHOICE slope, ß1					
INTRCPT2, ?10	-0.122648	0.043165	-2.841	4948	0.005
For BULLY slope, ß2					
INTRCPT2, ?20	0.052693	0.103558	0.509	56	0.613
WELLPCA, ?21	-0.117058	0.101262	-1.156	56	0.253
For TIME slope, ß3					
INTRCPT2, ?30	-0.064449	0.036609	-1.760	4948	0.078
For DUTIES slope, ß4					
INTRCPT2, ?40	-0.053944	0.043889	-1.229	4948	0.219
For PHYCON15 slope, ß5					
INTRCPT2, ?50	2.003516	0.154297	12.985	4948	<0.001
For KESCON15 slope, ß6					
INTRCPT2, ?60	0.423353	0.062824	6.739	4948	<0.001

Fixed Effect	Coefficient	Odds Ratio	Confidence Interval
For INTRCPT1, ß0			
INTRCPT2, ?00	-2.416783	0.089208	(0.078,0.102)
WELLPCA, ?01	-0.186004	0.830270	(0.771,0.894)
For CHOICE slope, ß1			
INTRCPT2, ?10	-0.122648	0.884575	(0.813,0.963)
For BULLY slope, ß2			
INTRCPT2, ?20	0.052693	1.054106	(0.857,1.297)
WELLPCA, ?21	-0.117058	0.889534	(0.726,1.090)
For TIME slope, ß3			
INTRCPT2, ?30	-0.064449	0.937584	(0.873,1.007)
For DUTIES slope, ß4			
INTRCPT2, ?40	-0.053944	0.947485	(0.869,1.033)
For PHYCON15 slope, ß5			
INTRCPT2, ?50	2.003516	7.415079	(5.480,10.034)
For KESCON15 slope, ß6			
INTRCPT2, ?60	0.423353	1.527073	(1.350,1.727)

Final estimation of variance components

Random Effect	Standard Deviation	Variance Component	df	Chi-square	p
INTRCPT1, u0	0.14542	0.02115	55	68.03134	0.112
BULLY slope, u2	0.28337	0.08030	55	63.07120	0.212

This is time pressure interaction

Final estimation of fixed effects: (Unit-specific model)

Fixed Effect	Coefficient	Standard Error	t	Approx. d.f.	p
For INTRCPT1, ß0					
INTRCPT2, ?00	-2.412825	0.073743	-32.720	56	<0.001
WELLPCA, ?01	-0.186473	0.055622	-3.352	56	0.001
For CHOICE slope, ß1					
INTRCPT2, ?10	-0.120713	0.045668	-2.643	4948	0.008
For BULLY slope, ß2					
INTRCPT2, ?20	0.060255	0.087615	0.688	4948	0.492
For TIME slope, ß3					
INTRCPT2, ?30	-0.055536	0.049636	-1.119	56	0.268
WELLPCA, ?31	-0.001038	0.049302	-0.021	56	0.983
For DUTIES slope, ß4					
INTRCPT2, ?40	-0.054049	0.050890	-1.062	4948	0.288
For PHYCON15 slope, ß5					
INTRCPT2, ?50	1.998397	0.161108	12.404	4948	<0.001
For KESCON15 slope, ß6					
INTRCPT2, ?60	0.420129	0.077503	5.421	4948	<0.001

Fixed Effect	Coefficient	Odds Ratio	Confidence Interval
For INTRCPT1, ß0			
INTRCPT2, ?00	-2.412825	0.089562	(0.077,0.104)
WELLPCA, ?01	-0.186473	0.829881	(0.742,0.928)
For CHOICE slope, ß1			
INTRCPT2, ?10	-0.120713	0.886288	(0.810,0.969)
For BULLY slope, ß2			
INTRCPT2, ?20	0.060255	1.062108	(0.895,1.261)
For TIME slope, ß3			
INTRCPT2, ?30	-0.055536	0.945978	(0.856,1.045)
WELLPCA, ?31	-0.001038	0.998963	(0.905,1.103)
For DUTIES slope, ß4			
INTRCPT2, ?40	-0.054049	0.947386	(0.857,1.047)
For PHYCON15 slope, ß5			
INTRCPT2, ?50	1.998397	7.377220	(5.380,10.117)
For KESCON15 slope, ß6			
INTRCPT2, ?60	0.420129	1.522158	(1.308,1.772)

Final estimation of fixed effects
(Unit-specific model with robust standard errors)

Fixed Effect	Coefficient	Standard	t	Approx.p

		Error		d.f.	
For INTRCPT1, ß0					
INTRCPT2, ?00	-2.412825	0.069505	-34.714	56	<0.001
WELLPCA, ?01	-0.186473	0.039647	-4.703	56	<0.001
For CHOICE slope, ß1					
INTRCPT2, ?10	-0.120713	0.042736	-2.825	4948	0.005
For BULLY slope, ß2					
INTRCPT2, ?20	0.060255	0.123767	0.487	4948	0.626
For TIME slope, ß3					
INTRCPT2, ?30	-0.055536	0.035607	-1.560	56	0.124
WELLPCA, ?31	-0.001038	0.037323	-0.028	56	0.978
For DUTIES slope, ß4					
INTRCPT2, ?40	-0.054049	0.044336	-1.219	4948	0.223
For PHYCON15 slope, ß5					
INTRCPT2, ?50	1.998397	0.153207	13.044	4948	<0.001
For KESCON15 slope, ß6					
INTRCPT2, ?60	0.420129	0.062326	6.741	4948	<0.001

Fixed Effect	Coefficient	Odds Ratio	Confidence Interval
For INTRCPT1, ß0			
INTRCPT2, ?00	-2.412825	0.089562	(0.078,0.103)
WELLPCA, ?01	-0.186473	0.829881	(0.766,0.899)
For CHOICE slope, ß1			
INTRCPT2, ?10	-0.120713	0.886288	(0.815,0.964)
For BULLY slope, ß2			
INTRCPT2, ?20	0.060255	1.062108	(0.833,1.354)
For TIME slope, ß3			
INTRCPT2, ?30	-0.055536	0.945978	(0.881,1.016)
WELLPCA, ?31	-0.001038	0.998963	(0.927,1.077)
For DUTIES slope, ß4			
INTRCPT2, ?40	-0.054049	0.947386	(0.869,1.033)
For PHYCON15 slope, ß5			
INTRCPT2, ?50	1.998397	7.377220	(5.464,9.961)
For KESCON15 slope, ß6			
INTRCPT2, ?60	0.420129	1.522158	(1.347,1.720)

Final estimation of variance components

Random Effect	Standard Deviation	Variance Component	df	Chi-square	p
INTRCPT1, u0	0.19075	0.03639	56	66.28329	0.164
TIME slope, u3	0.01953	0.00038	56	41.44008	>0.500

This is role clarity interaction

Final estimation of fixed effects: (Unit-specific model)

Fixed Effect	Coefficient	Standard Error	t	Approx. d.f.	p
or INTRCPT1, ß0					
INTRCPT2, ?00	-2.416440	0.073361	-32.939	56	<0.001
WELLPCA, ?01	-0.186411	0.055328	-3.369	56	0.001
For CHOICE slope, ß1					
INTRCPT2, ?10	-0.121816	0.045624	-2.670	4948	0.008
For BULLY slope, ß2					
INTRCPT2, ?20	0.065026	0.087570	0.743	4948	0.458
For TIME slope, ß3					
INTRCPT2, ?30	-0.062211	0.048005	-1.296	4948	0.195
For DUTIES slope, ß4					
INTRCPT2, ?40	-0.052388	0.051786	-1.012	56	0.316
WELLPCA, ?41	-0.017049	0.051093	-0.334	56	0.740
For PHYCON15 slope, ß5					
INTRCPT2, ?50	1.999649	0.161125	12.411	4948	<0.001
For KESCON15 slope, ß6					
INTRCPT2, ?60	0.420544	0.077538	5.424	4948	<0.001

Fixed Effect	Coefficient	Odds Ratio	Confidence Interval
For INTRCPT1, ß0			
INTRCPT2, ?00	-2.416440	0.089239	(0.077,0.103)
WELLPCA, ?01	-0.186411	0.829933	(0.743,0.927)
For CHOICE slope, ß1			
INTRCPT2, ?10	-0.121816	0.885312	(0.810,0.968)
For BULLY slope, ß2			
INTRCPT2, ?20	0.065026	1.067186	(0.899,1.267)
For TIME slope, ß3			
INTRCPT2, ?30	-0.062211	0.939685	(0.855,1.032)
For DUTIES slope, ß4			
INTRCPT2, ?40	-0.052388	0.948961	(0.855,1.053)
WELLPCA, ?41	-0.017049	0.983096	(0.887,1.089)
For PHYCON15 slope, ß5			
INTRCPT2, ?50	1.999649	7.386462	(5.386,10.130)
For KESCON15 slope, ß6			
INTRCPT2, ?60	0.420544	1.522790	(1.308,1.773)

Final estimation of fixed effects
(Unit-specific model with robust standard errors)

Fixed Effect	Coefficient	Standard Error	t	Approx. d.f.	p
For INTRCPT1, ß0					
INTRCPT2, ?00	-2.416440	0.069623	-34.707	56	<0.001
WELLPCA, ?01	-0.186411	0.039488	-4.721	56	<0.001
For CHOICE slope, ß1					
INTRCPT2, ?10	-0.121816	0.042617	-2.858	4948	0.004
For BULLY slope, ß2					
INTRCPT2, ?20	0.065026	0.122941	0.529	4948	0.597
For TIME slope, ß3					
INTRCPT2, ?30	-0.062211	0.036045	-1.726	4948	0.084
For DUTIES slope, ß4					
INTRCPT2, ?40	-0.052388	0.046663	-1.123	56	0.266
WELLPCA, ?41	-0.017049	0.038911	-0.438	56	0.663
For PHYCON15 slope, ß5					
INTRCPT2, ?50	1.999649	0.153180	13.054	4948	<0.001
For KESCON15 slope, ß6					
INTRCPT2, ?60	0.420544	0.062928	6.683	4948	<0.001

Fixed Effect	Coefficient	Odds Ratio	Confidence Interval
For INTRCPT1, ß0			
INTRCPT2, ?00	-2.416440	0.089239	(0.078,0.103)
WELLPCA, ?01	-0.186411	0.829933	(0.767,0.898)
For CHOICE slope, ß1			
INTRCPT2, ?10	-0.121816	0.885312	(0.814,0.962)
For BULLY slope, ß2			
INTRCPT2, ?20	0.065026	1.067186	(0.839,1.358)
For TIME slope, ß3			
INTRCPT2, ?30	-0.062211	0.939685	(0.876,1.008)
For DUTIES slope, ß4			
INTRCPT2, ?40	-0.052388	0.948961	(0.864,1.042)
WELLPCA, ?41	-0.017049	0.983096	(0.909,1.063)
For PHYCON15 slope, ß5			
INTRCPT2, ?50	1.999649	7.386462	(5.471,9.973)
For KESCON15 slope, ß6			
INTRCPT2, ?60	0.420544	1.522790	(1.346,1.723)

Final estimation of variance components

Random Effect	Standard Deviation	Variance Component	df	Chi-square	p
INTRCPT1, u0	0.18465	0.03410	56	67.43242	0.141
DUTIES slope, u4	0.02557	0.00065	56	53.97416	>0.500

Mplus results

Variable labels

Phy15 - 2105 physical health, continuous variable

Kes15 – 2015 Kessler score, continuous variable

Choice15 – job control

Bully15 - bullying

Press15 – time pressure

Duties15 – role clarity

Prafac1 – first principal component of health and wellbeing practices

Prafacsq – FAC1_1 * FAC1_1

sizcat – organisational size

finpro – sector - financial and professional services

know – sector - knowledge intensive industries

manu – sector manufacrturing

clu1 clu2 clu3 – clusters from latent profile analysis, clu1 is least extensive category, clu2 is most extensive category, clu3 is intermediate category

ACC – single practice, accumulated overtime

ACT – single practice, active governance

EAT – single practice, healthy eating

ALC – single practice, drinking support

PHY – single practice, physical health

PSY – single practice, psychological problems

SMO – single practice, smoking cessation

TER – single practice, medical services

BEN – single practice, provision of health benefits

HPR – single practice, health promotion

SPR – single practice, service promotion

CUL – single practice, management support

FEL – single practice, flexible time

Results are presented in the following order: a) Table 4 from the main text; b) Replications of significant results from the main text using simple models and a maximum likelihood estimator; c) Moderators; c) outcomes as continuous variables; d) using latent profile analysis clusters instead of the first principal component; e) controls for organisational size and sector; f) assessing impact of single practices.

```
Linear effects only deterioration of physical health in 2016.

SUMMARY OF DATA

     Number of clusters                        58

       Size (s)    Cluster ID with Size s

          7        22
         16        166
         23        80 71 205
         25        74
         27        192
         31        65
         32        106
```

35	228
37	170 72
39	50 75 181
40	182
42	165
44	114
45	173
46	107
47	169 30
48	226 81
49	36
50	116 108 40
56	162 84
57	61
59	206 93 83
60	92
61	69
63	6
64	152
65	145
66	4
68	68
69	154
72	174
78	98
84	178 2
95	156 202
96	144
108	142
131	122
172	24
175	209
224	204
269	125
364	217
738	110
860	100

COVARIANCE COVERAGE OF DATA

Minimum covariance coverage value   0.100

Number of missing data patterns 8

PROPORTION OF DATA PRESENT

Covariance Coverage

	PHYCAT16	PHY15	KES15	CHOICE15	BULLY15
PHYCAT16	1.000				
PHY15	0.947	0.947			
KES15	0.922	0.922	0.922		
CHOICE15	0.915	0.915	0.915	0.915	
BULLY15	0.915	0.915	0.915	0.915	0.915
PRESS15	0.915	0.915	0.915	0.915	0.914
DUTIES15	0.915	0.915	0.915	0.915	0.915
PRAFAC	1.000	0.947	0.922	0.915	0.915

Covariance Coverage

	PRESS15	DUTIES15	PRAFAC
PRESS15	0.915		
DUTIES15	0.915	0.915	
PRAFAC	0.915	0.915	1.000

UNIVARIATE PROPORTIONS AND COUNTS FOR CATEGORICAL VARIABLES

PHYCAT16

Category 1	0.881	4884.000
Category 2	0.119	662.000

UNIVARIATE SAMPLE STATISTICS

UNIVARIATE HIGHER-ORDER MOMENT DESCRIPTIVE STATISTICS

Variable/ Sample Size	Mean/ Variance	Skewness/ Kurtosis	Minimum/ Maximum	% with Min/Max	Percentiles 20%/60%	40%/80%	Median
PHY15	0.000	-0.546	-0.632	36.83%	-0.632	0.368	0.368

```
       5254.000       0.233      -1.702       0.368   63.17%       0.368      0.368
     KES15            0.000       1.717      -0.511   21.65%      -0.511     -0.178     -0.178
       5114.000       0.289       3.835       3.489    0.04%      -0.011      0.322
     CHOICE15         0.000      -0.402      -2.225    7.41%      -1.225     -0.225     -0.225
       5077.000       1.081      -0.336       1.775    8.49%       0.775      0.775
     BULLY15          0.000       3.272      -0.170   87.25%      -0.170     -0.170     -0.170
       5073.000       0.239      11.772       3.830    0.08%      -0.170     -0.170
     PRESS15          0.000       0.252      -1.462   19.10%      -0.462     -0.462     -0.462
       5074.000       1.001      -0.371       2.538    2.76%       0.538      0.538
     DUTIES15         0.000      -1.246      -3.120    2.40%      -0.120     -0.120     -0.120
       5077.000       0.861       1.673       0.880   38.74%      -0.120      0.880
     PRAFAC          -0.185       0.245      -2.338    1.72%      -1.448     -0.527     -0.318
         58.000       1.345      -0.636       2.338    1.72%       0.080      0.752
```

THE MODEL ESTIMATION TERMINATED NORMALLY

USE THE FBITERATIONS OPTION TO INCREASE THE NUMBER OF ITERATIONS BY A FACTOR
OF AT LEAST TWO TO CHECK CONVERGENCE AND THAT THE PSR VALUE DOES NOT INCREASE.

MODEL FIT INFORMATION

Number of Free Parameters   36

Bayesian Posterior Predictive Checking using Chi-Square

```
          95% Confidence Interval for the Difference Between
          the Observed and the Replicated Chi-Square Values

                              -17.297           22.027

          Posterior Predictive P-Value              0.464
```

MODEL RESULTS

```
                                Posterior  One-Tailed         95% C.I.
                    Estimate       S.D.      P-Value   Lower 2.5%  Upper 2.5%  Significance
```

Within Level

PHYCAT16 ON						
PHY15	0.969	0.066	0.000	0.843	1.105	*
CHOICE15	-0.071	0.024	0.002	-0.117	-0.024	*
BULLY15	0.033	0.050	0.259	-0.068	0.129	
DUTIES15	-0.032	0.028	0.134	-0.082	0.024	
PRESS15	-0.033	0.026	0.110	-0.084	0.018	
KES15	0.225	0.044	0.000	0.142	0.313	*
KES15 WITH						
PHY15	0.051	0.004	0.000	0.044	0.058	*
CHOICE15	-0.089	0.008	0.000	-0.105	-0.074	*
BULLY15	0.057	0.004	0.000	0.050	0.065	*
PRESS15	0.100	0.008	0.000	0.084	0.115	*
DUTIES15	-0.106	0.007	0.000	-0.121	-0.091	*
PHY15 WITH						
CHOICE15	-0.036	0.007	0.000	-0.050	-0.023	*
BULLY15	0.012	0.003	0.000	0.005	0.018	*
PRESS15	0.039	0.007	0.000	0.025	0.052	*
DUTIES15	-0.036	0.006	0.000	-0.048	-0.024	*
CHOICE15 WITH						
BULLY15	-0.058	0.007	0.000	-0.071	-0.044	*
PRESS15	-0.087	0.014	0.000	-0.115	-0.059	*
DUTIES15	0.173	0.014	0.000	0.146	0.200	*
BULLY15 WITH						
PRESS15	0.105	0.007	0.000	0.091	0.118	*
DUTIES15	-0.078	0.007	0.000	-0.091	-0.066	*
PRESS15 WITH						
DUTIES15	-0.186	0.013	0.000	-0.211	-0.161	*
Means						
PHY15	0.002	0.007	0.358	-0.010	0.016	
KES15	0.001	0.008	0.435	-0.014	0.017	
CHOICE15	-0.001	0.015	0.456	-0.030	0.027	
BULLY15	0.001	0.007	0.464	-0.013	0.014	
PRESS15	0.000	0.014	0.490	-0.027	0.029	
DUTIES15	-0.002	0.013	0.443	-0.026	0.025	
Variances						
PHY15	0.233	0.005	0.000	0.225	0.243	*
KES15	0.290	0.006	0.000	0.279	0.301	*

```
    CHOICE15           1.081       0.021      0.000       1.041       1.123      *
    BULLY15            0.239       0.005      0.000       0.230       0.249      *
    PRESS15            1.002       0.020      0.000       0.964       1.041      *
    DUTIES15           0.862       0.017      0.000       0.830       0.896      *

Between Level

 PHYCAT16   ON
    PRAFAC            -0.082       0.031      0.002      -0.147      -0.023      *

 Thresholds
    PHYCAT16$1         1.347       0.035      0.000       1.282       1.420      *

 Residual Variances
    PHYCAT16           0.012       0.012      0.000       0.001       0.046      *


STANDARDIZED MODEL RESULTS


STDYX Standardization

                                Posterior  One-Tailed         95% C.I.
                    Estimate       S.D.      P-Value   Lower 2.5%  Upper 2.5%  Significance
Within Level

 PHYCAT16   ON
    PHY15              0.414       0.023      0.000       0.370       0.460      *
    CHOICE15          -0.065       0.022      0.002      -0.107      -0.022      *
    BULLY15            0.014       0.021      0.259      -0.030       0.055
    DUTIES15          -0.026       0.023      0.134      -0.068       0.020
    PRESS15           -0.029       0.023      0.110      -0.074       0.016
    KES15              0.107       0.021      0.000       0.068       0.148      *

 KES15    WITH
    PHY15              0.198       0.013      0.000       0.171       0.223      *
    CHOICE15          -0.159       0.014      0.000      -0.185      -0.131      *
    BULLY15            0.219       0.013      0.000       0.194       0.243      *
    PRESS15            0.185       0.014      0.000       0.158       0.211      *
    DUTIES15          -0.212       0.013      0.000      -0.239      -0.184      *

 PHY15    WITH
    CHOICE15          -0.072       0.014      0.000      -0.099      -0.046      *
    BULLY15            0.049       0.014      0.000       0.022       0.077      *
```

```
   PRESS15            0.080       0.014       0.000       0.052       0.107      *
   DUTIES15          -0.080       0.014       0.000      -0.108      -0.055      *

 CHOICE15 WITH
   BULLY15           -0.114       0.013       0.000      -0.138      -0.087      *
   PRESS15           -0.083       0.014       0.000      -0.111      -0.057      *
   DUTIES15           0.179       0.014       0.000       0.152       0.206      *

 BULLY15  WITH
   PRESS15            0.214       0.013       0.000       0.189       0.239      *
   DUTIES15          -0.172       0.014       0.000      -0.198      -0.145      *

 PRESS15  WITH
   DUTIES15          -0.200       0.013       0.000      -0.226      -0.174      *

 Means
   PHY15              0.005       0.014       0.358      -0.022       0.033
   KES15              0.002       0.014       0.435      -0.025       0.031
   CHOICE15          -0.001       0.014       0.456      -0.029       0.026
   BULLY15            0.001       0.014       0.464      -0.027       0.030
   PRESS15            0.000       0.014       0.490      -0.027       0.029
   DUTIES15          -0.002       0.014       0.443      -0.027       0.027

 Variances
   PHY15              1.000       0.000       0.000       1.000       1.000
   KES15              1.000       0.000       0.000       1.000       1.000
   CHOICE15           1.000       0.000       0.000       1.000       1.000
   BULLY15            1.000       0.000       0.000       1.000       1.000
   PRESS15            1.000       0.000       0.000       1.000       1.000
   DUTIES15           1.000       0.000       0.000       1.000       1.000

Between Level

 PHYCAT16   ON
   PRAFAC            -0.657       0.198       0.002      -0.956      -0.208      *

 Thresholds
   PHYCAT16$1         8.871       3.256       0.000       5.493      17.444      *

 Residual Variances
   PHYCAT16           0.568       0.236       0.000       0.085       0.956      *


R-SQUARE
```

```
Within Level

                              Posterior  One-Tailed         95% C.I.
    Variable        Estimate       S.D.      P-Value   Lower 2.5%  Upper 2.5%

    PHYCAT16           0.216       0.020      0.000       0.177       0.257

Between Level

                              Posterior  One-Tailed         95% C.I.
    Variable        Estimate       S.D.      P-Value   Lower 2.5%  Upper 2.5%

    PHYCAT16           0.432       0.236      0.000       0.044       0.915


TECHNICAL 1 OUTPUT


     PARAMETER SPECIFICATION FOR WITHIN


           TAU
              PHYCAT16
              ________
                    0


           NU
              PHYCAT16      PHY15         KES15         CHOICE15      BULLY15
              ________      ________      ________      ________      ________
                    0             0             0             0             0


           NU
              PRESS15       DUTIES15
              ________      ________
                    0             0


           LAMBDA
              PHYCAT16      PHY15         KES15         CHOICE15      BULLY15
              ________      ________      ________      ________      ________
 PHYCAT16           0             0             0             0             0
```

PHY15	0	0	0	0	0
KES15	0	0	0	0	0
CHOICE15	0	0	0	0	0
BULLY15	0	0	0	0	0
PRESS15	0	0	0	0	0
DUTIES15	0	0	0	0	0

LAMBDA

	PRESS15	DUTIES15
PHYCAT16	0	0
PHY15	0	0
KES15	0	0
CHOICE15	0	0
BULLY15	0	0
PRESS15	0	0
DUTIES15	0	0

THETA

	PHYCAT16	PHY15	KES15	CHOICE15	BULLY15
PHYCAT16	0				
PHY15	0	0			
KES15	0	0	0		
CHOICE15	0	0	0	0	
BULLY15	0	0	0	0	0
PRESS15	0	0	0	0	0
DUTIES15	0	0	0	0	0

THETA

	PRESS15	DUTIES15
PRESS15	0	
DUTIES15	0	0

ALPHA

PHYCAT16	PHY15	KES15	CHOICE15	BULLY15
0	1	2	3	4

ALPHA

PRESS15	DUTIES15
5	6

BETA

	PHYCAT16	PHY15	KES15	CHOICE15	BULLY15
PHYCAT16	0	7	8	9	10
PHY15	0	0	0	0	0
KES15	0	0	0	0	0
CHOICE15	0	0	0	0	0
BULLY15	0	0	0	0	0
PRESS15	0	0	0	0	0
DUTIES15	0	0	0	0	0

BETA

	PRESS15	DUTIES15
PHYCAT16	11	12
PHY15	0	0
KES15	0	0
CHOICE15	0	0
BULLY15	0	0
PRESS15	0	0
DUTIES15	0	0

PSI

	PHYCAT16	PHY15	KES15	CHOICE15	BULLY15
PHYCAT16	0				
PHY15	0	13			
KES15	0	14	15		
CHOICE15	0	16	17	18	
BULLY15	0	19	20	21	22
PRESS15	0	23	24	25	26
DUTIES15	0	28	29	30	31

PSI

```
              PRESS15       DUTIES15
              ________      ________
PRESS15            27
DUTIES15           32            33


    PARAMETER SPECIFICATION FOR BETWEEN


          TAU
              PHYCAT16
              ________
                   36


          NU
              PHYCAT16      PRAFAC
              ________      ________
                    0             0


          LAMBDA
              PHYCAT16      PRAFAC
              ________      ________
PHYCAT16            0             0
PRAFAC              0             0


          THETA
              PHYCAT16      PRAFAC
              ________      ________
PHYCAT16            0
PRAFAC              0             0


          ALPHA
              PHYCAT16      PRAFAC
              ________      ________
                    0             0


          BETA
              PHYCAT16      PRAFAC
              ________      ________
```

PHYCAT16	0	34
PRAFAC	0	0

PSI

	PHYCAT16	PRAFAC
PHYCAT16	35	
PRAFAC	0	0

STARTING VALUES FOR WITHIN

TAU

PHYCAT16
0.000

NU

PHYCAT16	PHY15	KES15	CHOICE15	BULLY15
0.000	0.000	0.000	0.000	0.000

NU

PRESS15	DUTIES15
0.000	0.000

LAMBDA

	PHYCAT16	PHY15	KES15	CHOICE15	BULLY15
PHYCAT16	1.000	0.000	0.000	0.000	0.000
PHY15	0.000	1.000	0.000	0.000	0.000
KES15	0.000	0.000	1.000	0.000	0.000
CHOICE15	0.000	0.000	0.000	1.000	0.000
BULLY15	0.000	0.000	0.000	0.000	1.000
PRESS15	0.000	0.000	0.000	0.000	0.000
DUTIES15	0.000	0.000	0.000	0.000	0.000

LAMBDA

	PRESS15	DUTIES15
PHYCAT16	0.000	0.000
PHY15	0.000	0.000
KES15	0.000	0.000
CHOICE15	0.000	0.000
BULLY15	0.000	0.000
PRESS15	1.000	0.000
DUTIES15	0.000	1.000

THETA

	PHYCAT16	PHY15	KES15	CHOICE15	BULLY15
PHYCAT16	0.000				
PHY15	0.000	0.000			
KES15	0.000	0.000	0.000		
CHOICE15	0.000	0.000	0.000	0.000	
BULLY15	0.000	0.000	0.000	0.000	0.000
PRESS15	0.000	0.000	0.000	0.000	0.000
DUTIES15	0.000	0.000	0.000	0.000	0.000

THETA

	PRESS15	DUTIES15
PRESS15	0.000	
DUTIES15	0.000	0.000

ALPHA

PHYCAT16	PHY15	KES15	CHOICE15	BULLY15
0.000	0.000	0.000	0.000	0.000

ALPHA

PRESS15	DUTIES15
0.000	0.000

BETA

	PHYCAT16	PHY15	KES15	CHOICE15	BULLY15
PHYCAT16	0.000	0.000	0.000	0.000	0.000
PHY15	0.000	0.000	0.000	0.000	0.000
KES15	0.000	0.000	0.000	0.000	0.000
CHOICE15	0.000	0.000	0.000	0.000	0.000
BULLY15	0.000	0.000	0.000	0.000	0.000
PRESS15	0.000	0.000	0.000	0.000	0.000
DUTIES15	0.000	0.000	0.000	0.000	0.000

BETA

	PRESS15	DUTIES15
PHYCAT16	0.000	0.000
PHY15	0.000	0.000
KES15	0.000	0.000
CHOICE15	0.000	0.000
BULLY15	0.000	0.000
PRESS15	0.000	0.000
DUTIES15	0.000	0.000

PSI

	PHYCAT16	PHY15	KES15	CHOICE15	BULLY15
PHYCAT16	1.000				
PHY15	0.000	0.116			
KES15	0.000	0.000	0.145		
CHOICE15	0.000	0.000	0.000	0.541	
BULLY15	0.000	0.000	0.000	0.000	0.119
PRESS15	0.000	0.000	0.000	0.000	0.000
DUTIES15	0.000	0.000	0.000	0.000	0.000

PSI

	PRESS15	DUTIES15
PRESS15	0.501	
DUTIES15	0.000	0.430

STARTING VALUES FOR BETWEEN

TAU

PHYCAT16
1.110

NU

PHYCAT16	PRAFAC
0.000	0.000

LAMBDA

	PHYCAT16	PRAFAC
PHYCAT16	1.000	0.000
PRAFAC	0.000	1.000

THETA

	PHYCAT16	PRAFAC
PHYCAT16	0.000	
PRAFAC	0.000	0.000

ALPHA

PHYCAT16	PRAFAC
0.000	0.000

BETA

	PHYCAT16	PRAFAC
PHYCAT16	0.000	0.000
PRAFAC	0.000	0.000

PSI

	PHYCAT16	PRAFAC
PHYCAT16	1.000	

PRAFAC	0.000	0.504

PRIORS FOR ALL PARAMETERS	PRIOR MEAN	PRIOR VARIANCE	PRIOR STD. DEV.
Parameter 1~N(0.000,infinity)	0.0000	infinity	infinity
Parameter 2~N(0.000,infinity)	0.0000	infinity	infinity
Parameter 3~N(0.000,infinity)	0.0000	infinity	infinity
Parameter 4~N(0.000,infinity)	0.0000	infinity	infinity
Parameter 5~N(0.000,infinity)	0.0000	infinity	infinity
Parameter 6~N(0.000,infinity)	0.0000	infinity	infinity
Parameter 7~N(0.000,5.000)	0.0000	5.0000	2.2361
Parameter 8~N(0.000,5.000)	0.0000	5.0000	2.2361
Parameter 9~N(0.000,5.000)	0.0000	5.0000	2.2361
Parameter 10~N(0.000,5.000)	0.0000	5.0000	2.2361
Parameter 11~N(0.000,5.000)	0.0000	5.0000	2.2361
Parameter 12~N(0.000,5.000)	0.0000	5.0000	2.2361
Parameter 13~IW(1.000,7)	infinity	infinity	infinity
Parameter 14~IW(0.000,7)	infinity	infinity	infinity
Parameter 15~IW(1.000,7)	infinity	infinity	infinity
Parameter 16~IW(0.000,7)	infinity	infinity	infinity
Parameter 17~IW(0.000,7)	infinity	infinity	infinity
Parameter 18~IW(1.000,7)	infinity	infinity	infinity
Parameter 19~IW(0.000,7)	infinity	infinity	infinity
Parameter 20~IW(0.000,7)	infinity	infinity	infinity
Parameter 21~IW(0.000,7)	infinity	infinity	infinity
Parameter 22~IW(1.000,7)	infinity	infinity	infinity
Parameter 23~IW(0.000,7)	infinity	infinity	infinity
Parameter 24~IW(0.000,7)	infinity	infinity	infinity
Parameter 25~IW(0.000,7)	infinity	infinity	infinity
Parameter 26~IW(0.000,7)	infinity	infinity	infinity
Parameter 27~IW(1.000,7)	infinity	infinity	infinity
Parameter 28~IW(0.000,7)	infinity	infinity	infinity
Parameter 29~IW(0.000,7)	infinity	infinity	infinity
Parameter 30~IW(0.000,7)	infinity	infinity	infinity
Parameter 31~IW(0.000,7)	infinity	infinity	infinity
Parameter 32~IW(0.000,7)	infinity	infinity	infinity
Parameter 33~IW(1.000,7)	infinity	infinity	infinity
Parameter 34~N(0.000,5.000)	0.0000	5.0000	2.2361
Parameter 35~IG(-1.000,0.000)	infinity	infinity	infinity
Parameter 36~N(0.000,5.000)	0.0000	5.0000	2.2361

TECHNICAL 3 OUTPUT

ESTIMATED COVARIANCE MATRIX FOR PARAMETER ESTIMATES

	1	2	3	4	5
1	0.450486D-04				
2	0.997951D-05	0.604930D-04			
3	-0.753058D-05	-0.221281D-04	0.213901D-03		
4	0.337152D-05	0.123644D-04	-0.913498D-05	0.473260D-04	
5	0.114664D-04	0.198158D-04	-0.196348D-04	0.221015D-04	0.197382D-03
6	-0.553474D-05	-0.214118D-04	0.418984D-04	-0.152100D-04	-0.365529D-04
7	-0.319584D-05	0.105079D-05	-0.299755D-04	-0.161188D-04	0.160576D-04
8	0.693293D-05	0.505720D-05	-0.207466D-05	0.155962D-04	0.255509D-04
9	-0.560437D-05	-0.223649D-05	0.563694D-05	-0.736016D-06	-0.161244D-04
10	0.781428D-05	0.953005D-06	-0.328032D-04	-0.532710D-05	-0.189586D-04
11	-0.442864D-05	0.595988D-05	-0.216141D-04	0.292113D-05	0.118026D-04
12	0.157469D-05	-0.244559D-05	-0.108942D-04	0.110956D-05	0.169878D-04
13	0.229983D-07	0.964489D-06	-0.186243D-05	-0.687844D-06	-0.191049D-05
14	0.990854D-06	0.720612D-06	-0.305602D-05	-0.125151D-05	0.377066D-06
15	0.352231D-06	0.154923D-06	-0.302392D-05	0.349100D-06	-0.580093D-06
16	-0.662154D-06	-0.107686D-05	-0.119918D-05	0.612909D-06	-0.267963D-05
17	-0.472853D-06	-0.207583D-05	0.448533D-05	0.339416D-05	0.677026D-05
18	-0.433000D-05	-0.469523D-05	0.462824D-05	-0.748047D-05	-0.278765D-05
19	0.101072D-05	0.724319D-06	-0.135894D-05	0.186588D-06	-0.827767D-06
20	0.284277D-06	-0.266761D-06	-0.427127D-06	-0.379787D-06	-0.160241D-05
21	-0.930959D-06	-0.417119D-07	0.422241D-06	0.300191D-05	0.478762D-05
22	0.153869D-06	0.193394D-05	0.427959D-06	-0.813580D-06	0.189138D-05
23	0.994719D-06	0.787881D-06	0.126813D-05	-0.123986D-06	0.212741D-05
24	0.147235D-05	0.165014D-05	-0.283183D-05	-0.238502D-06	-0.554577D-05
25	0.119110D-06	0.221452D-05	0.874296D-05	0.133012D-05	0.640579D-05
26	0.496519D-06	0.701370D-06	0.451698D-05	-0.203180D-06	0.492987D-05
27	-0.228662D-05	0.498399D-05	0.948168D-05	0.652988D-06	0.395037D-05
28	-0.985772D-06	-0.208619D-05	-0.119963D-05	-0.425884D-06	0.211492D-05
29	-0.125402D-05	-0.873887D-06	0.434149D-05	-0.203609D-06	0.908985D-05
30	-0.133885D-06	0.887616D-06	0.124387D-05	-0.170187D-05	-0.139304D-04
31	-0.996769D-06	0.853473D-06	0.156064D-05	0.635077D-06	0.237212D-05
32	-0.131193D-05	0.503662D-06	0.633704D-05	0.168727D-05	-0.602739D-05
33	-0.126726D-05	-0.373716D-05	-0.332961D-05	-0.111506D-05	-0.590608D-05
34	-0.665825D-05	0.501018D-05	0.437792D-05	-0.419604D-05	0.632722D-05
35	-0.685340D-06	0.210923D-05	0.798844D-05	0.399107D-06	-0.312268D-05
36	0.287091D-05	0.307618D-05	-0.822961D-05	-0.645408D-05	0.232032D-04

ESTIMATED COVARIANCE MATRIX FOR PARAMETER ESTIMATES

	6	7	8	9	10
6	0.172642D-03				
7	-0.238805D-04	0.435490D-02			
8	-0.113961D-05	-0.361015D-03	0.190480D-02		
9	0.550626D-06	0.445875D-05	0.118398D-03	0.569941D-03	
10	-0.225483D-05	-0.249055D-04	-0.365399D-03	0.527453D-04	0.245396D-02
11	-0.517870D-05	-0.212849D-04	-0.858730D-04	0.243848D-05	-0.250975D-03
12	0.339858D-05	-0.534132D-04	0.160527D-03	-0.107518D-03	0.125267D-03
13	-0.127660D-05	-0.796734D-05	0.181192D-05	-0.150826D-05	0.106507D-05
14	-0.484553D-06	0.300042D-05	0.107046D-05	-0.131602D-05	0.445056D-05
15	0.550491D-06	-0.168221D-04	-0.173837D-05	0.234898D-05	0.742437D-05
16	0.185446D-05	-0.411929D-04	0.644356D-05	-0.378475D-05	0.947681D-05
17	0.339472D-05	-0.265273D-05	0.735901D-06	0.692258D-06	-0.230056D-05
18	0.391285D-05	-0.151205D-04	-0.180561D-05	0.246239D-05	-0.445171D-04
19	0.815346D-06	0.694357D-06	-0.335743D-05	0.181418D-05	0.784737D-05
20	0.233470D-05	-0.330423D-05	0.228017D-05	0.305633D-05	-0.321983D-05
21	0.417851D-06	-0.148788D-04	-0.565412D-05	0.153558D-05	0.386454D-05
22	-0.486790D-06	0.450338D-05	-0.363646D-06	0.246365D-05	-0.150287D-04
23	-0.233612D-05	-0.776745D-05	0.437760D-05	-0.565065D-05	0.219308D-04
24	-0.168042D-05	-0.804871D-05	0.540673D-05	0.315652D-05	0.985324D-05
25	-0.221413D-05	-0.222746D-04	0.623829D-05	0.256251D-05	0.141653D-04
26	-0.193548D-06	0.579873D-05	-0.227702D-05	0.501682D-05	-0.298470D-05
27	-0.276712D-05	0.203007D-04	0.140966D-05	-0.486312D-06	-0.166610D-04
28	0.296243D-05	0.639215D-05	-0.936121D-05	-0.611865D-05	-0.912236D-05
29	0.228119D-05	0.182861D-04	-0.131575D-04	-0.480139D-05	-0.336273D-06
30	0.105539D-04	-0.127298D-04	0.423603D-06	-0.111372D-05	-0.214792D-04
31	-0.325562D-05	-0.646384D-05	-0.159889D-05	-0.611622D-05	-0.483465D-05
32	-0.288118D-05	-0.320715D-05	-0.121134D-05	-0.400534D-05	0.274989D-04
33	0.798554D-05	-0.808013D-04	0.217555D-04	0.465222D-05	-0.256183D-04
34	-0.524641D-05	-0.547998D-04	0.515616D-04	-0.154455D-04	-0.283642D-04
35	-0.398890D-05	0.275407D-04	-0.736146D-07	-0.153481D-04	-0.105657D-04
36	-0.172655D-04	0.839427D-03	0.102792D-03	-0.120257D-04	-0.326886D-04

ESTIMATED COVARIANCE MATRIX FOR PARAMETER ESTIMATES

	11	12	13	14	15
11	0.656817D-03				
12	0.129812D-03	0.780344D-03			
13	0.966950D-06	0.425733D-05	0.209783D-04		
14	0.187392D-05	0.192566D-05	0.446997D-05	0.131004D-04	
15	0.423394D-05	0.168305D-05	0.352632D-06	0.529044D-05	0.319548D-04

16	-0.988619D-05	0.410006D-05	-0.391636D-05	-0.438091D-05	0.578874D-06
17	0.284562D-05	0.735244D-05	-0.347782D-05	-0.414944D-05	-0.100673D-04
18	0.111017D-04	-0.302129D-05	0.256755D-05	0.541189D-06	0.119704D-05
19	0.535643D-05	0.406712D-06	0.953914D-06	0.225394D-05	0.445484D-06
20	0.195029D-05	-0.659794D-06	0.236555D-06	0.745259D-06	0.514697D-05
21	0.406663D-06	0.221858D-05	-0.855325D-06	-0.882885D-06	0.706719D-07
22	0.602556D-06	-0.385300D-05	0.786427D-06	0.381393D-06	0.514061D-06
23	-0.334951D-05	0.706832D-05	0.247574D-05	0.392774D-05	0.330047D-06
24	-0.103024D-05	0.634670D-06	0.165330D-05	0.334543D-05	0.101639D-04
25	-0.899325D-05	0.128392D-04	-0.215698D-05	-0.187204D-05	0.247343D-05
26	0.205624D-05	0.300817D-05	0.811777D-06	-0.414187D-06	0.742101D-06
27	0.858586D-05	0.333103D-05	-0.290143D-06	0.135948D-05	0.210843D-05
28	0.253149D-05	0.310890D-05	-0.316031D-05	-0.404041D-05	-0.562771D-06
29	-0.106698D-04	-0.422066D-05	-0.482947D-07	-0.267212D-05	-0.121476D-04
30	-0.140241D-05	-0.245313D-04	0.272470D-05	0.143178D-05	0.293468D-06
31	-0.424245D-05	-0.939511D-06	-0.657329D-06	-0.233409D-06	-0.132616D-05
32	-0.365864D-05	0.725958D-06	-0.443453D-06	-0.127940D-05	-0.190462D-05
33	0.340261D-05	0.208069D-04	0.171036D-05	0.106587D-05	0.186443D-05
34	-0.189173D-04	-0.155977D-05	-0.129704D-05	-0.200996D-05	0.220588D-05
35	0.119164D-05	0.157723D-04	0.226602D-05	-0.109181D-05	0.187990D-05
36	-0.188559D-04	-0.115147D-04	-0.118677D-05	-0.190782D-05	0.196413D-05

ESTIMATED COVARIANCE MATRIX FOR PARAMETER ESTIMATES

	16	17	18	19	20
16	0.487858D-04				
17	0.116877D-04	0.630629D-04			
18	-0.166528D-04	-0.375780D-04	0.455391D-03		
19	-0.191148D-05	-0.182408D-05	0.264162D-05	0.104692D-04	
20	-0.817960D-06	-0.456138D-05	0.522654D-06	0.301726D-05	0.133090D-04
21	0.304932D-05	0.110293D-04	-0.288232D-04	-0.233199D-05	-0.434891D-05
22	0.844568D-06	-0.337745D-05	-0.193490D-06	0.200322D-05	0.495586D-05
23	-0.285239D-05	0.845181D-07	0.229113D-05	0.498441D-05	0.124014D-05
24	-0.377697D-05	-0.690495D-05	-0.111959D-05	0.977771D-06	0.684403D-05
25	0.869617D-05	0.159935D-04	-0.477182D-04	-0.335447D-05	-0.110477D-05
26	-0.298472D-05	-0.138446D-05	0.431385D-05	0.225351D-05	0.579125D-05
27	-0.611142D-05	-0.439886D-05	0.195017D-04	0.496763D-05	0.711942D-05
28	0.619253D-05	0.382806D-05	-0.403695D-05	-0.351002D-05	-0.125948D-05
29	0.139184D-06	0.123662D-04	-0.326956D-05	-0.154951D-05	-0.501923D-05
30	-0.670381D-05	-0.249434D-04	0.646095D-04	0.186031D-05	0.143520D-05
31	0.949561D-06	0.612900D-05	-0.172490D-05	-0.252561D-05	-0.610807D-05
32	0.551498D-05	0.666014D-05	-0.160300D-04	-0.137337D-05	-0.428755D-05
33	0.443063D-05	-0.113337D-04	0.776350D-05	0.956668D-06	0.261603D-05

34	0.769016D-05	0.183435D-05	-0.523834D-05	0.530898D-06	0.295657D-05
35	-0.125085D-05	-0.161359D-05	0.107276D-05	0.810559D-06	0.638230D-06
36	-0.109049D-04	0.601799D-05	-0.204332D-04	-0.102930D-05	-0.488980D-05

ESTIMATED COVARIANCE MATRIX FOR PARAMETER ESTIMATES

	21	22	23	24	25
21	0.478232D-04				
22	-0.602844D-05	0.233604D-04			
23	-0.981669D-06	0.153768D-05	0.461699D-04		
24	-0.347927D-05	0.420562D-05	0.970748D-05	0.591044D-04	
25	0.235137D-04	-0.166117D-05	-0.574578D-05	-0.162043D-04	0.208199D-03
26	-0.462280D-05	0.106536D-04	0.268469D-05	0.129440D-04	-0.794647D-05
27	-0.507961D-05	0.100908D-04	0.146347D-04	0.375361D-04	-0.198702D-04
28	0.675329D-06	-0.178007D-05	-0.911662D-05	-0.159735D-05	-0.863974D-07
29	0.282485D-05	-0.238641D-05	-0.316800D-05	-0.137647D-04	0.414504D-05
30	-0.175196D-04	0.318993D-05	0.284894D-07	0.618198D-05	-0.443955D-04
31	0.825090D-05	-0.792194D-05	-0.264990D-05	-0.586423D-05	0.506375D-05
32	0.435981D-05	-0.447409D-05	-0.583381D-05	-0.203138D-04	0.221364D-04
33	-0.701298D-05	0.537333D-05	0.213768D-05	0.614392D-05	-0.109177D-04
34	-0.353400D-05	-0.285298D-05	-0.432063D-05	0.455555D-05	0.542977D-05
35	0.225393D-05	0.535972D-06	-0.119388D-05	0.247314D-05	0.711097D-05
36	0.631548D-05	0.252767D-05	-0.808958D-06	0.643417D-05	0.250881D-04

ESTIMATED COVARIANCE MATRIX FOR PARAMETER ESTIMATES

	26	27	28	29	30
26	0.475443D-04				
27	0.414791D-04	0.387706D-03			
28	-0.857955D-06	-0.484867D-05	0.375047D-04		
29	-0.194238D-05	-0.878439D-05	0.796891D-05	0.516376D-04	
30	0.462451D-05	0.262766D-04	-0.700537D-05	-0.186122D-04	0.191145D-03
31	-0.957124D-05	-0.967686D-05	0.280193D-05	0.130462D-04	-0.110255D-04
32	-0.152178D-04	-0.649130D-04	0.420802D-05	0.198491D-04	-0.194369D-04
33	0.632364D-05	0.118342D-04	-0.747099D-05	-0.373247D-04	0.567816D-04
34	0.313177D-05	-0.207314D-04	0.523153D-05	0.366868D-05	-0.787170D-05
35	0.200157D-05	0.279372D-05	0.335815D-07	-0.158846D-05	0.195772D-05
36	0.661774D-05	0.211659D-04	-0.710165D-05	0.165110D-05	-0.577874D-05

ESTIMATED COVARIANCE MATRIX FOR PARAMETER ESTIMATES

	31	32	33	34	35

31	0.422960D-04				
32	0.239142D-04	0.160896D-03			
33	-0.287895D-04	-0.530740D-04	0.285440D-03		
34	0.395424D-05	-0.339180D-05	-0.332282D-04	0.949379D-03	
35	-0.433636D-05	0.323164D-05	-0.277083D-05	-0.349982D-04	0.138089D-03
36	-0.499402D-05	0.170032D-05	-0.861927D-05	-0.157200D-03	0.820578D-04

ESTIMATED COVARIANCE MATRIX FOR PARAMETER ESTIMATES

	36
36	0.119297D-02

ESTIMATED CORRELATION MATRIX FOR PARAMETER ESTIMATES

	1	2	3	4	5
1	1.000				
2	0.191	1.000			
3	-0.077	-0.195	1.000		
4	0.073	0.231	-0.091	1.000	
5	0.122	0.181	-0.096	0.229	1.000
6	-0.063	-0.210	0.218	-0.168	-0.198
7	-0.007	0.002	-0.031	-0.036	0.017
8	0.024	0.015	-0.003	0.052	0.042
9	-0.035	-0.012	0.016	-0.004	-0.048
10	0.024	0.002	-0.045	-0.016	-0.027
11	-0.026	0.030	-0.058	0.017	0.033
12	0.008	-0.011	-0.027	0.006	0.043
13	0.001	0.027	-0.028	-0.022	-0.030
14	0.041	0.026	-0.058	-0.050	0.007
15	0.009	0.004	-0.037	0.009	-0.007
16	-0.014	-0.020	-0.012	0.013	-0.027
17	-0.009	-0.034	0.039	0.062	0.061
18	-0.030	-0.028	0.015	-0.051	-0.009
19	0.047	0.029	-0.029	0.008	-0.018
20	0.012	-0.009	-0.008	-0.015	-0.031
21	-0.020	-0.001	0.004	0.063	0.049
22	0.005	0.051	0.006	-0.024	0.028
23	0.022	0.015	0.013	-0.003	0.022
24	0.029	0.028	-0.025	-0.005	-0.051
25	0.001	0.020	0.041	0.013	0.032
26	0.011	0.013	0.045	-0.004	0.051

27	-0.017	0.033	0.033	0.005	0.014
28	-0.024	-0.044	-0.013	-0.010	0.025
29	-0.026	-0.016	0.041	-0.004	0.090
30	-0.001	0.008	0.006	-0.018	-0.072
31	-0.023	0.017	0.016	0.014	0.026
32	-0.015	0.005	0.034	0.019	-0.034
33	-0.011	-0.028	-0.013	-0.010	-0.025
34	-0.032	0.021	0.010	-0.020	0.015
35	-0.009	0.023	0.046	0.005	-0.019
36	0.012	0.011	-0.016	-0.027	0.048

ESTIMATED CORRELATION MATRIX FOR PARAMETER ESTIMATES

	6	7	8	9	10
6	1.000				
7	-0.028	1.000			
8	-0.002	-0.125	1.000		
9	0.002	0.003	0.114	1.000	
10	-0.003	-0.008	-0.169	0.045	1.000
11	-0.015	-0.013	-0.077	0.004	-0.198
12	0.009	-0.029	0.132	-0.161	0.091
13	-0.021	-0.026	0.009	-0.014	0.005
14	-0.010	0.013	0.007	-0.015	0.025
15	0.007	-0.045	-0.007	0.017	0.027
16	0.020	-0.089	0.021	-0.023	0.027
17	0.033	-0.005	0.002	0.004	-0.006
18	0.014	-0.011	-0.002	0.005	-0.042
19	0.019	0.003	-0.024	0.023	0.049
20	0.049	-0.014	0.014	0.035	-0.018
21	0.005	-0.033	-0.019	0.009	0.011
22	-0.008	0.014	-0.002	0.021	-0.063
23	-0.026	-0.017	0.015	-0.035	0.065
24	-0.017	-0.016	0.016	0.017	0.026
25	-0.012	-0.023	0.010	0.007	0.020
26	-0.002	0.013	-0.008	0.030	-0.009
27	-0.011	0.016	0.002	-0.001	-0.017
28	0.037	0.016	-0.035	-0.042	-0.030
29	0.024	0.039	-0.042	-0.028	-0.001
30	0.058	-0.014	0.001	-0.003	-0.031
31	-0.038	-0.015	-0.006	-0.039	-0.015
32	-0.017	-0.004	-0.002	-0.013	0.044
33	0.036	-0.072	0.030	0.012	-0.031
34	-0.013	-0.027	0.038	-0.021	-0.019

35	-0.026	0.036	0.000	-0.055	-0.018
36	-0.038	0.368	0.068	-0.015	-0.019

ESTIMATED CORRELATION MATRIX FOR PARAMETER ESTIMATES

	11	12	13	14	15
11	1.000				
12	0.181	1.000			
13	0.008	0.033	1.000		
14	0.020	0.019	0.270	1.000	
15	0.029	0.011	0.014	0.259	1.000
16	-0.055	0.021	-0.122	-0.173	0.015
17	0.014	0.033	-0.096	-0.144	-0.224
18	0.020	-0.005	0.026	0.007	0.010
19	0.065	0.004	0.064	0.192	0.024
20	0.021	-0.006	0.014	0.056	0.250
21	0.002	0.011	-0.027	-0.035	0.002
22	0.005	-0.029	0.036	0.022	0.019
23	-0.019	0.037	0.080	0.160	0.009
24	-0.005	0.003	0.047	0.120	0.234
25	-0.024	0.032	-0.033	-0.036	0.030
26	0.012	0.016	0.026	-0.017	0.019
27	0.017	0.006	-0.003	0.019	0.019
28	0.016	0.018	-0.113	-0.182	-0.016
29	-0.058	-0.021	-0.001	-0.103	-0.299
30	-0.004	-0.064	0.043	0.029	0.004
31	-0.025	-0.005	-0.022	-0.010	-0.036
32	-0.011	0.002	-0.008	-0.028	-0.027
33	0.008	0.044	0.022	0.017	0.020
34	-0.024	-0.002	-0.009	-0.018	0.013
35	0.004	0.048	0.042	-0.026	0.028
36	-0.021	-0.012	-0.008	-0.015	0.010

ESTIMATED CORRELATION MATRIX FOR PARAMETER ESTIMATES

	16	17	18	19	20
16	1.000				
17	0.211	1.000			
18	-0.112	-0.222	1.000		
19	-0.085	-0.071	0.038	1.000	
20	-0.032	-0.157	0.007	0.256	1.000
21	0.063	0.201	-0.195	-0.104	-0.172

22	0.025	-0.088	-0.002	0.128	0.281
23	-0.060	0.002	0.016	0.227	0.050
24	-0.070	-0.113	-0.007	0.039	0.244
25	0.086	0.140	-0.155	-0.072	-0.021
26	-0.062	-0.025	0.029	0.101	0.230
27	-0.044	-0.028	0.046	0.078	0.099
28	0.145	0.079	-0.031	-0.177	-0.056
29	0.003	0.217	-0.021	-0.067	-0.191
30	-0.069	-0.227	0.219	0.042	0.028
31	0.021	0.119	-0.012	-0.120	-0.257
32	0.062	0.066	-0.059	-0.033	-0.093
33	0.038	-0.084	0.022	0.018	0.042
34	0.036	0.007	-0.008	0.005	0.026
35	-0.015	-0.017	0.004	0.021	0.015
36	-0.045	0.022	-0.028	-0.009	-0.039

ESTIMATED CORRELATION MATRIX FOR PARAMETER ESTIMATES

	21	22	23	24	25
21	1.000				
22	-0.180	1.000			
23	-0.021	0.047	1.000		
24	-0.065	0.113	0.186	1.000	
25	0.236	-0.024	-0.059	-0.146	1.000
26	-0.097	0.320	0.057	0.244	-0.080
27	-0.037	0.106	0.109	0.248	-0.070
28	0.016	-0.060	-0.219	-0.034	-0.001
29	0.057	-0.069	-0.065	-0.249	0.040
30	-0.183	0.048	0.000	0.058	-0.223
31	0.183	-0.252	-0.060	-0.117	0.054
32	0.050	-0.073	-0.068	-0.208	0.121
33	-0.060	0.066	0.019	0.047	-0.045
34	-0.017	-0.019	-0.021	0.019	0.012
35	0.028	0.009	-0.015	0.027	0.042
36	0.026	0.015	-0.003	0.024	0.050

ESTIMATED CORRELATION MATRIX FOR PARAMETER ESTIMATES

	26	27	28	29	30
26	1.000				
27	0.306	1.000			
28	-0.020	-0.040	1.000		

29	-0.039	-0.062	0.181	1.000	
30	0.049	0.097	-0.083	-0.187	1.000
31	-0.213	-0.076	0.070	0.279	-0.123
32	-0.174	-0.260	0.054	0.218	-0.111
33	0.054	0.036	-0.072	-0.307	0.243
34	0.015	-0.034	0.028	0.017	-0.018
35	0.025	0.012	0.000	-0.019	0.012
36	0.028	0.031	-0.034	0.007	-0.012

ESTIMATED CORRELATION MATRIX FOR PARAMETER ESTIMATES

	31	32	33	34	35
31	1.000				
32	0.290	1.000			
33	-0.262	-0.248	1.000		
34	0.020	-0.009	-0.064	1.000	
35	-0.057	0.022	-0.014	-0.097	1.000
36	-0.022	0.004	-0.015	-0.148	0.202

ESTIMATED CORRELATION MATRIX FOR PARAMETER ESTIMATES

	36
36	1.000

TECHNICAL 8 OUTPUT

TECHNICAL 8 OUTPUT FOR BAYES ESTIMATION

CHAIN	BSEED
1	0
2	285380
3	253358

ITERATION	POTENTIAL SCALE REDUCTION	PARAMETER WITH HIGHEST PSR
100	1.013	35
200	1.021	36
300	1.009	8
400	1.009	33
500	1.005	3

600	1.006	35
700	1.007	35
800	1.004	35
900	1.002	35
1000	1.001	36

Curvilinear effects deterioration of physical health 2016

SUMMARY OF DATA

Number of clusters 58

Size (s)	Cluster ID with Size s
7	22
16	166
23	80 71 205
25	74
27	192
31	65
32	106
35	228
37	170 72
39	50 75 181
40	182
42	165
44	114
45	173
46	107
47	169 30
48	226 81
49	36
50	116 108 40
56	162 84
57	61
59	206 93 83
60	92
61	69
63	6
64	152
65	145
66	4
68	68
69	154
72	174
78	98
84	178 2
95	156 202
96	144

108	142
131	122
172	24
175	209
224	204
269	125
364	217
738	110
860	100

COVARIANCE COVERAGE OF DATA

Minimum covariance coverage value 0.100

Number of missing data patterns 8

PROPORTION OF DATA PRESENT

Covariance Coverage

	PHYCAT16	PRAFAC	PRAFACSQ	PHY15	KES15
PHYCAT16	1.000				
PRAFAC	1.000	1.000			
PRAFACSQ	1.000	1.000	1.000		
PHY15	0.947	0.947	0.947	0.947	
KES15	0.922	0.922	0.922	0.922	0.922
CHOICE15	0.915	0.915	0.915	0.915	0.915
BULLY15	0.915	0.915	0.915	0.915	0.915
PRESS15	0.915	0.915	0.915	0.915	0.915
DUTIES15	0.915	0.915	0.915	0.915	0.915

Covariance Coverage

	CHOICE15	BULLY15	PRESS15	DUTIES15
CHOICE15	0.915			
BULLY15	0.915	0.915		
PRESS15	0.915	0.914	0.915	
DUTIES15	0.915	0.915	0.915	0.915

UNIVARIATE PROPORTIONS AND COUNTS FOR CATEGORICAL VARIABLES

PHYCAT16

Category 1	0.881	4884.000
Category 2	0.119	662.000

UNIVARIATE SAMPLE STATISTICS

UNIVARIATE HIGHER-ORDER MOMENT DESCRIPTIVE STATISTICS

Variable/ Sample Size	Mean/ Variance	Skewness/ Kurtosis	Minimum/ Maximum	% with Min/Max	Percentiles 20%/60%	Percentiles 40%/80%	Median
PRAFAC	-0.185	0.245	-2.338	1.72%	-1.448	-0.527	-0.318
58.000	1.345	-0.636	2.338	1.72%	0.080	0.752	
PRAFACSQ	1.379	1.240	0.002	1.72%	0.151	0.381	0.681
58.000	2.369	0.557	5.467	1.72%	1.168	2.366	
PHY15	0.000	-0.546	-0.632	36.83%	-0.632	0.368	0.368
5254.000	0.233	-1.702	0.368	63.17%	0.368	0.368	
KES15	0.000	1.717	-0.511	21.65%	-0.511	-0.178	-0.178
5114.000	0.289	3.835	3.489	0.04%	-0.011	0.322	
CHOICE15	0.000	-0.402	-2.225	7.41%	-1.225	-0.225	-0.225
5077.000	1.081	-0.336	1.775	8.49%	0.775	0.775	
BULLY15	0.000	3.272	-0.170	87.25%	-0.170	-0.170	-0.170
5073.000	0.239	11.772	3.830	0.08%	-0.170	-0.170	
PRESS15	0.000	0.252	-1.462	19.10%	-0.462	-0.462	-0.462
5074.000	1.001	-0.371	2.538	2.76%	0.538	0.538	
DUTIES15	0.000	-1.246	-3.120	2.40%	-0.120	-0.120	-0.120
5077.000	0.861	1.673	0.880	38.74%	-0.120	0.880	

THE MODEL ESTIMATION TERMINATED NORMALLY

USE THE FBITERATIONS OPTION TO INCREASE THE NUMBER OF ITERATIONS BY A FACTOR
OF AT LEAST TWO TO CHECK CONVERGENCE AND THAT THE PSR VALUE DOES NOT INCREASE.

MODEL FIT INFORMATION

Number of Free Parameters 42

Bayesian Posterior Predictive Checking using Chi-Square

95% Confidence Interval for the Difference Between the Observed and the Replicated Chi-Square Values

-28.399 21.278

Posterior Predictive P-Value 0.556

MODEL RESULTS

	Estimate	Posterior S.D.	One-Tailed P-Value	95% C.I. Lower 2.5%	95% C.I. Upper 2.5%	Significance
**Within Level**						
PHYCAT16 ON						
PHY15	0.970	0.068	0.000	0.838	1.103	*
CHOICE15	-0.067	0.025	0.003	-0.116	-0.018	*
BULLY15	0.029	0.049	0.275	-0.069	0.120	
DUTIES15	-0.029	0.027	0.138	-0.085	0.023	
PRESS15	-0.032	0.027	0.103	-0.083	0.021	
KES15	0.224	0.044	0.000	0.137	0.311	*
KES15 WITH						
PHY15	0.051	0.004	0.000	0.044	0.059	*
CHOICE15	-0.089	0.008	0.000	-0.105	-0.074	*
BULLY15	0.057	0.004	0.000	0.050	0.065	*
PRESS15	0.100	0.008	0.000	0.084	0.115	*
DUTIES15	-0.106	0.007	0.000	-0.121	-0.093	*
PHY15 WITH						
CHOICE15	-0.036	0.007	0.000	-0.050	-0.023	*
BULLY15	0.012	0.003	0.001	0.005	0.018	*
PRESS15	0.038	0.007	0.000	0.025	0.052	*
DUTIES15	-0.036	0.006	0.000	-0.048	-0.024	*
CHOICE15 WITH						
BULLY15	-0.058	0.007	0.000	-0.072	-0.044	*
PRESS15	-0.086	0.015	0.000	-0.118	-0.060	*

DUTIES15	0.173	0.013	0.000	0.148	0.201	*
BULLY15 WITH						
PRESS15	0.104	0.007	0.000	0.092	0.118	*
DUTIES15	-0.077	0.006	0.000	-0.090	-0.065	*
PRESS15 WITH						
DUTIES15	-0.185	0.013	0.000	-0.212	-0.160	*
Means						
PHY15	0.002	0.007	0.368	-0.011	0.015	
KES15	0.001	0.008	0.444	-0.014	0.015	
CHOICE15	-0.002	0.015	0.449	-0.029	0.028	
BULLY15	0.001	0.007	0.474	-0.013	0.015	
PRESS15	0.001	0.014	0.481	-0.026	0.029	
DUTIES15	-0.001	0.013	0.471	-0.026	0.025	
Variances						
PHY15	0.233	0.005	0.000	0.225	0.243	*
KES15	0.289	0.006	0.000	0.279	0.301	*
CHOICE15	1.081	0.021	0.000	1.041	1.122	*
BULLY15	0.239	0.005	0.000	0.230	0.248	*
PRESS15	1.000	0.020	0.000	0.963	1.040	*
DUTIES15	0.861	0.017	0.000	0.829	0.895	*
Between Level						
PHYCAT16 ON						
PRAFAC	-0.091	0.031	0.001	-0.155	-0.034	*
PRAFACSQ	-0.028	0.024	0.114	-0.077	0.017	
PRAFAC WITH						
PRAFACSQ	-0.108	0.237	0.316	-0.597	0.356	
Means						
PRAFAC	-0.188	0.152	0.104	-0.495	0.121	
PRAFACSQ	1.379	0.211	0.000	0.958	1.794	*
Thresholds						
PHYCAT16$1	1.312	0.045	0.000	1.227	1.409	*
Variances						
PRAFAC	1.356	0.260	0.000	0.979	1.964	*
PRAFACSQ	2.360	0.447	0.000	1.674	3.419	*

Residual Variances

	Estimate	S.D.	P-Value	Lower 2.5%	Upper 2.5%	Significance
PHYCAT16	0.012	0.011	0.000	0.001	0.043	*

STANDARDIZED MODEL RESULTS

STDYX Standardization

	Estimate	Posterior S.D.	One-Tailed P-Value	95% C.I. Lower 2.5%	Upper 2.5%	Significance
**Within Level**						
PHYCAT16 ON						
PHY15	0.415	0.024	0.000	0.366	0.461	*
CHOICE15	-0.062	0.023	0.003	-0.106	-0.017	*
BULLY15	0.013	0.021	0.275	-0.029	0.052	
DUTIES15	-0.024	0.022	0.138	-0.069	0.019	
PRESS15	-0.028	0.024	0.103	-0.074	0.019	
KES15	0.106	0.021	0.000	0.066	0.148	*
KES15 WITH						
PHY15	0.198	0.013	0.000	0.170	0.223	*
CHOICE15	-0.159	0.014	0.000	-0.187	-0.132	*
BULLY15	0.218	0.013	0.000	0.193	0.244	*
PRESS15	0.186	0.014	0.000	0.157	0.212	*
DUTIES15	-0.213	0.013	0.000	-0.240	-0.187	*
PHY15 WITH						
CHOICE15	-0.072	0.014	0.000	-0.099	-0.045	*
BULLY15	0.049	0.014	0.001	0.021	0.076	*
PRESS15	0.079	0.014	0.000	0.053	0.107	*
DUTIES15	-0.081	0.014	0.000	-0.107	-0.054	*
CHOICE15 WITH						
BULLY15	-0.114	0.014	0.000	-0.141	-0.088	*
PRESS15	-0.083	0.014	0.000	-0.113	-0.058	*
DUTIES15	0.179	0.013	0.000	0.154	0.206	*
BULLY15 WITH						
PRESS15	0.214	0.013	0.000	0.189	0.239	*
DUTIES15	-0.171	0.013	0.000	-0.197	-0.145	*

```
 PRESS15  WITH
    DUTIES15          -0.200       0.013      0.000      -0.227      -0.174      *

 Means
    PHY15              0.004       0.014      0.368      -0.024       0.031
    KES15              0.002       0.014      0.444      -0.025       0.028
    CHOICE15          -0.002       0.014      0.449      -0.028       0.026
    BULLY15            0.001       0.014      0.474      -0.026       0.030
    PRESS15            0.001       0.014      0.481      -0.026       0.029
    DUTIES15          -0.001       0.014      0.471      -0.028       0.026

 Variances
    PHY15              1.000       0.000      0.000       1.000       1.000
    KES15              1.000       0.000      0.000       1.000       1.000
    CHOICE15           1.000       0.000      0.000       1.000       1.000
    BULLY15            1.000       0.000      0.000       1.000       1.000
    PRESS15            1.000       0.000      0.000       1.000       1.000
    DUTIES15           1.000       0.000      0.000       1.000       1.000

Between Level

 PHYCAT16   ON
    PRAFAC            -0.653       0.168      0.001      -0.936      -0.283      *
    PRAFACSQ          -0.275       0.216      0.114      -0.672       0.169

 PRAFAC   WITH
    PRAFACSQ          -0.061       0.125      0.316      -0.314       0.186

 Means
    PRAFAC            -0.162       0.130      0.104      -0.421       0.104
    PRAFACSQ           0.900       0.157      0.000       0.592       1.199      *

 Thresholds
    PHYCAT16$1         7.917       2.669      0.000       5.027      14.530      *

 Variances
    PRAFAC             1.000       0.000      0.000       1.000       1.000
    PRAFACSQ           1.000       0.000      0.000       1.000       1.000

 Residual Variances
    PHYCAT16           0.465       0.211      0.000       0.080       0.864      *


R-SQUARE
```

```
Within Level

                                Posterior  One-Tailed         95% C.I.
    Variable        Estimate       S.D.      P-Value   Lower 2.5%  Upper 2.5%

    PHYCAT16           0.215       0.021      0.000       0.177       0.258

Between Level

                                Posterior  One-Tailed         95% C.I.
    Variable        Estimate       S.D.      P-Value   Lower 2.5%  Upper 2.5%

    PHYCAT16           0.535       0.211      0.000       0.136       0.920


TECHNICAL 1 OUTPUT


     PARAMETER SPECIFICATION FOR WITHIN


           TAU
              PHYCAT16
              ________
                    0


           NU
              PHYCAT16      PHY15         KES15         CHOICE15      BULLY15
              ________      ________      ________      ________      ________
                    0             0             0             0             0


           NU
              PRESS15       DUTIES15
              ________      ________
                    0             0


           LAMBDA
              PHYCAT16      PHY15         KES15         CHOICE15      BULLY15
              ________      ________      ________      ________      ________
 PHYCAT16           0             0             0             0             0
```

PHY15	0	0	0	0	0
KES15	0	0	0	0	0
CHOICE15	0	0	0	0	0
BULLY15	0	0	0	0	0
PRESS15	0	0	0	0	0
DUTIES15	0	0	0	0	0

LAMBDA

	PRESS15	DUTIES15
PHYCAT16	0	0
PHY15	0	0
KES15	0	0
CHOICE15	0	0
BULLY15	0	0
PRESS15	0	0
DUTIES15	0	0

THETA

	PHYCAT16	PHY15	KES15	CHOICE15	BULLY15
PHYCAT16	0				
PHY15	0	0			
KES15	0	0	0		
CHOICE15	0	0	0	0	
BULLY15	0	0	0	0	0
PRESS15	0	0	0	0	0
DUTIES15	0	0	0	0	0

THETA

	PRESS15	DUTIES15
PRESS15	0	
DUTIES15	0	0

ALPHA

PHYCAT16	PHY15	KES15	CHOICE15	BULLY15
0	1	2	3	4

ALPHA

PRESS15	DUTIES15
5	6

BETA

	PHYCAT16	PHY15	KES15	CHOICE15	BULLY15
PHYCAT16	0	7	8	9	10
PHY15	0	0	0	0	0
KES15	0	0	0	0	0
CHOICE15	0	0	0	0	0
BULLY15	0	0	0	0	0
PRESS15	0	0	0	0	0
DUTIES15	0	0	0	0	0

BETA

	PRESS15	DUTIES15
PHYCAT16	11	12
PHY15	0	0
KES15	0	0
CHOICE15	0	0
BULLY15	0	0
PRESS15	0	0
DUTIES15	0	0

PSI

	PHYCAT16	PHY15	KES15	CHOICE15	BULLY15
PHYCAT16	0				
PHY15	0	13			
KES15	0	14	15		
CHOICE15	0	16	17	18	
BULLY15	0	19	20	21	22
PRESS15	0	23	24	25	26
DUTIES15	0	28	29	30	31

PSI

	PRESS15	DUTIES15
PRESS15	27	
DUTIES15	32	33

PARAMETER SPECIFICATION FOR BETWEEN

TAU

PHYCAT16
42

NU

PHYCAT16	PRAFAC	PRAFACSQ
0	0	0

LAMBDA

	PHYCAT16	PRAFAC	PRAFACSQ
PHYCAT16	0	0	0
PRAFAC	0	0	0
PRAFACSQ	0	0	0

THETA

	PHYCAT16	PRAFAC	PRAFACSQ
PHYCAT16	0		
PRAFAC	0	0	
PRAFACSQ	0	0	0

ALPHA

PHYCAT16	PRAFAC	PRAFACSQ
0	34	35

BETA

	PHYCAT16	PRAFAC	PRAFACSQ
PHYCAT16	0	36	37
PRAFAC	0	0	0
PRAFACSQ	0	0	0

PSI

	PHYCAT16	PRAFAC	PRAFACSQ
PHYCAT16	38		
PRAFAC	0	39	
PRAFACSQ	0	40	41

STARTING VALUES FOR WITHIN

TAU

PHYCAT16
0.000

NU

PHYCAT16	PHY15	KES15	CHOICE15	BULLY15
0.000	0.000	0.000	0.000	0.000

NU

PRESS15	DUTIES15
0.000	0.000

LAMBDA

	PHYCAT16	PHY15	KES15	CHOICE15	BULLY15
PHYCAT16	1.000	0.000	0.000	0.000	0.000
PHY15	0.000	1.000	0.000	0.000	0.000
KES15	0.000	0.000	1.000	0.000	0.000
CHOICE15	0.000	0.000	0.000	1.000	0.000
BULLY15	0.000	0.000	0.000	0.000	1.000

PRESS15	0.000	0.000	0.000	0.000	0.000
DUTIES15	0.000	0.000	0.000	0.000	0.000

LAMBDA

	PRESS15	DUTIES15
PHYCAT16	0.000	0.000
PHY15	0.000	0.000
KES15	0.000	0.000
CHOICE15	0.000	0.000
BULLY15	0.000	0.000
PRESS15	1.000	0.000
DUTIES15	0.000	1.000

THETA

	PHYCAT16	PHY15	KES15	CHOICE15	BULLY15
PHYCAT16	0.000				
PHY15	0.000	0.000			
KES15	0.000	0.000	0.000		
CHOICE15	0.000	0.000	0.000	0.000	
BULLY15	0.000	0.000	0.000	0.000	0.000
PRESS15	0.000	0.000	0.000	0.000	0.000
DUTIES15	0.000	0.000	0.000	0.000	0.000

THETA

	PRESS15	DUTIES15
PRESS15	0.000	
DUTIES15	0.000	0.000

ALPHA

PHYCAT16	PHY15	KES15	CHOICE15	BULLY15
0.000	0.000	0.000	0.000	0.000

ALPHA

PRESS15	DUTIES15

	0.000	0.000

BETA

	PHYCAT16	PHY15	KES15	CHOICE15	BULLY15
PHYCAT16	0.000	0.000	0.000	0.000	0.000
PHY15	0.000	0.000	0.000	0.000	0.000
KES15	0.000	0.000	0.000	0.000	0.000
CHOICE15	0.000	0.000	0.000	0.000	0.000
BULLY15	0.000	0.000	0.000	0.000	0.000
PRESS15	0.000	0.000	0.000	0.000	0.000
DUTIES15	0.000	0.000	0.000	0.000	0.000

BETA

	PRESS15	DUTIES15
PHYCAT16	0.000	0.000
PHY15	0.000	0.000
KES15	0.000	0.000
CHOICE15	0.000	0.000
BULLY15	0.000	0.000
PRESS15	0.000	0.000
DUTIES15	0.000	0.000

PSI

	PHYCAT16	PHY15	KES15	CHOICE15	BULLY15
PHYCAT16	1.000				
PHY15	0.000	0.116			
KES15	0.000	0.000	0.145		
CHOICE15	0.000	0.000	0.000	0.541	
BULLY15	0.000	0.000	0.000	0.000	0.119
PRESS15	0.000	0.000	0.000	0.000	0.000
DUTIES15	0.000	0.000	0.000	0.000	0.000

PSI

	PRESS15	DUTIES15
PRESS15	0.501	
DUTIES15	0.000	0.430

```
     STARTING VALUES FOR BETWEEN


           TAU
              PHYCAT16
              ________
                1.110


           NU
              PHYCAT16      PRAFAC        PRAFACSQ
              ________      ________      ________
                0.000         0.000         0.000


           LAMBDA
              PHYCAT16      PRAFAC        PRAFACSQ
              ________      ________      ________
 PHYCAT16       1.000         0.000         0.000
 PRAFAC         0.000         1.000         0.000
 PRAFACSQ       0.000         0.000         1.000


           THETA
              PHYCAT16      PRAFAC        PRAFACSQ
              ________      ________      ________
 PHYCAT16       0.000
 PRAFAC         0.000         0.000
 PRAFACSQ       0.000         0.000         0.000


           ALPHA
              PHYCAT16      PRAFAC        PRAFACSQ
              ________      ________      ________
                0.000         0.020         1.009


           BETA
              PHYCAT16      PRAFAC        PRAFACSQ
              ________      ________      ________
 PHYCAT16       0.000         0.000         0.000
 PRAFAC         0.000         0.000         0.000
```

PRAFACSQ	0.000	0.000	0.000

PSI

	PHYCAT16	PRAFAC	PRAFACSQ
PHYCAT16	1.000		
PRAFAC	0.000	0.504	
PRAFACSQ	0.000	0.000	0.745

PRIORS FOR ALL PARAMETERS	PRIOR MEAN	PRIOR VARIANCE	PRIOR STD. DEV.
Parameter 1~N(0.000,infinity)	0.0000	infinity	infinity
Parameter 2~N(0.000,infinity)	0.0000	infinity	infinity
Parameter 3~N(0.000,infinity)	0.0000	infinity	infinity
Parameter 4~N(0.000,infinity)	0.0000	infinity	infinity
Parameter 5~N(0.000,infinity)	0.0000	infinity	infinity
Parameter 6~N(0.000,infinity)	0.0000	infinity	infinity
Parameter 7~N(0.000,5.000)	0.0000	5.0000	2.2361
Parameter 8~N(0.000,5.000)	0.0000	5.0000	2.2361
Parameter 9~N(0.000,5.000)	0.0000	5.0000	2.2361
Parameter 10~N(0.000,5.000)	0.0000	5.0000	2.2361
Parameter 11~N(0.000,5.000)	0.0000	5.0000	2.2361
Parameter 12~N(0.000,5.000)	0.0000	5.0000	2.2361
Parameter 13~IW(1.000,7)	infinity	infinity	infinity
Parameter 14~IW(0.000,7)	infinity	infinity	infinity
Parameter 15~IW(1.000,7)	infinity	infinity	infinity
Parameter 16~IW(0.000,7)	infinity	infinity	infinity
Parameter 17~IW(0.000,7)	infinity	infinity	infinity
Parameter 18~IW(1.000,7)	infinity	infinity	infinity
Parameter 19~IW(0.000,7)	infinity	infinity	infinity
Parameter 20~IW(0.000,7)	infinity	infinity	infinity
Parameter 21~IW(0.000,7)	infinity	infinity	infinity
Parameter 22~IW(1.000,7)	infinity	infinity	infinity
Parameter 23~IW(0.000,7)	infinity	infinity	infinity
Parameter 24~IW(0.000,7)	infinity	infinity	infinity
Parameter 25~IW(0.000,7)	infinity	infinity	infinity
Parameter 26~IW(0.000,7)	infinity	infinity	infinity
Parameter 27~IW(1.000,7)	infinity	infinity	infinity
Parameter 28~IW(0.000,7)	infinity	infinity	infinity
Parameter 29~IW(0.000,7)	infinity	infinity	infinity
Parameter 30~IW(0.000,7)	infinity	infinity	infinity

Parameter 31~IW(0.000,7)	infinity	infinity	infinity
Parameter 32~IW(0.000,7)	infinity	infinity	infinity
Parameter 33~IW(1.000,7)	infinity	infinity	infinity
Parameter 34~N(0.000,infinity)	0.0000	infinity	infinity
Parameter 35~N(0.000,infinity)	0.0000	infinity	infinity
Parameter 36~N(0.000,5.000)	0.0000	5.0000	2.2361
Parameter 37~N(0.000,5.000)	0.0000	5.0000	2.2361
Parameter 38~IG(-1.000,0.000)	infinity	infinity	infinity
Parameter 39~IW(1.000,3)	infinity	infinity	infinity
Parameter 40~IW(0.000,3)	infinity	infinity	infinity
Parameter 41~IW(1.000,3)	infinity	infinity	infinity
Parameter 42~N(0.000,5.000)	0.0000	5.0000	2.2361

TECHNICAL 3 OUTPUT

ESTIMATED COVARIANCE MATRIX FOR PARAMETER ESTIMATES

	1	2	3	4	5
1	0.448715D-04				
2	0.119511D-04	0.581457D-04			
3	-0.838923D-05	-0.194678D-04	0.221297D-03		
4	0.414711D-05	0.135844D-04	-0.120752D-04	0.493773D-04	
5	0.582680D-05	0.187762D-04	-0.187262D-04	0.223977D-04	0.195613D-03
6	-0.947227D-05	-0.167695D-04	0.353880D-04	-0.142983D-04	-0.310541D-04
7	-0.128025D-04	-0.378183D-05	-0.168271D-04	-0.867851D-05	-0.444532D-04
8	-0.360072D-05	-0.619411D-05	0.222913D-04	0.268619D-05	-0.927373D-05
9	-0.933509D-05	-0.283443D-05	0.177603D-04	-0.130963D-05	0.225270D-05
10	0.101222D-04	-0.531193D-05	-0.108935D-04	0.103832D-04	-0.230369D-04
11	0.881251D-05	0.609790D-05	0.104511D-04	-0.248018D-05	0.477918D-05
12	0.437128D-05	0.399760D-05	-0.157799D-04	0.521688D-05	0.192786D-05
13	-0.408427D-06	0.663662D-06	0.208215D-05	-0.109374D-05	-0.883130D-06
14	-0.577930D-06	0.106010D-05	0.128622D-05	-0.678533D-06	-0.471782D-06
15	-0.190359D-05	0.128874D-05	0.171363D-06	0.195570D-06	0.839888D-06
16	-0.360446D-06	-0.143635D-07	0.112423D-05	0.487557D-06	0.176933D-05
17	0.211579D-05	-0.105687D-06	-0.209660D-05	0.270987D-06	0.212994D-05
18	0.503414D-06	0.455433D-05	0.316168D-05	0.252356D-05	-0.254510D-05
19	0.372728D-06	-0.217108D-06	-0.112674D-05	-0.261644D-06	0.137462D-06
20	-0.317349D-06	-0.142839D-06	-0.581387D-06	0.628898D-07	0.111536D-06
21	0.197741D-06	0.956673D-06	0.432648D-07	-0.185555D-07	-0.347394D-06
22	-0.825867D-07	-0.690233D-06	0.129857D-05	-0.547962D-06	0.195286D-05
23	-0.107255D-05	-0.343294D-06	-0.377751D-05	-0.160502D-05	-0.648686D-08
24	-0.329911D-06	0.214159D-05	-0.136563D-05	0.127819D-05	-0.682583D-06

25	0.169511D-05	-0.344564D-06	-0.459392D-05	-0.115919D-05	-0.479902D-05
26	-0.117068D-05	-0.181536D-05	0.137000D-05	0.457445D-06	0.365049D-06
27	-0.368185D-05	-0.546637D-05	-0.408590D-05	0.630887D-06	-0.412978D-05
28	-0.108346D-05	0.308756D-06	0.788870D-06	0.475976D-06	-0.435219D-06
29	0.199157D-05	0.498992D-06	0.205791D-05	-0.515343D-06	0.846182D-06
30	-0.127617D-05	0.651519D-06	-0.280189D-05	0.587067D-06	-0.405055D-05
31	0.152709D-05	0.181278D-05	0.798685D-06	-0.344909D-06	0.170923D-05
32	0.677112D-06	0.161329D-06	0.404421D-05	-0.375470D-05	-0.316974D-05
33	-0.730152D-06	-0.271511D-06	0.119694D-05	0.722513D-06	0.381007D-05
34	-0.173638D-04	-0.229438D-04	-0.933167D-04	-0.728735D-05	-0.132678D-04
35	0.242931D-05	0.901130D-05	0.909060D-04	-0.599016D-05	0.522223D-04
36	-0.278495D-05	-0.766690D-06	-0.662237D-06	0.787105D-06	0.802687D-05
37	-0.782533D-05	0.475866D-05	-0.676829D-06	-0.765558D-06	0.194023D-05
38	-0.100539D-05	-0.399197D-05	0.193496D-05	0.309609D-07	-0.199000D-05
39	-0.210424D-04	0.155109D-04	-0.122835D-04	0.341611D-04	0.569962D-05
40	0.626010D-05	-0.137769D-04	-0.160621D-03	-0.378795D-04	0.194049D-03
41	0.487238D-04	0.234210D-03	0.381320D-03	0.861424D-04	-0.113015D-03
42	-0.211427D-04	0.831872D-05	-0.106860D-04	-0.104757D-04	-0.283671D-04

ESTIMATED COVARIANCE MATRIX FOR PARAMETER ESTIMATES

	6	7	8	9	10
6	0.172160D-03				
7	0.254407D-05	0.458376D-02			
8	0.119631D-04	-0.336205D-03	0.191079D-02		
9	-0.449742D-05	-0.179550D-07	0.165146D-03	0.604485D-03	
10	0.619814D-05	0.639327D-04	-0.337367D-03	0.538598D-04	0.236878D-02
11	0.100223D-04	-0.259278D-04	-0.110861D-03	-0.135423D-04	-0.223648D-03
12	0.114204D-04	0.399529D-04	0.158190D-03	-0.631052D-04	0.149327D-03
13	0.205145D-06	0.109952D-05	0.216127D-05	-0.652526D-06	-0.675369D-05
14	-0.148828D-05	0.232169D-05	-0.621089D-06	-0.215643D-05	-0.845294D-05
15	0.104590D-05	-0.315144D-05	0.618296D-05	-0.147652D-05	-0.139116D-05
16	0.142866D-05	-0.123167D-05	0.105516D-04	-0.137188D-05	0.366192D-06
17	0.294246D-05	-0.144539D-05	-0.152237D-04	-0.324608D-05	-0.106990D-04
18	0.411362D-05	0.185577D-04	0.562947D-04	0.245866D-04	-0.359630D-05
19	0.272549D-05	0.110215D-04	0.919268D-07	0.112936D-07	-0.589867D-05
20	0.309596D-06	-0.231155D-05	-0.410420D-05	0.122160D-05	-0.360884D-05
21	-0.107358D-05	-0.753728D-05	-0.675014D-05	-0.761381D-06	0.144367D-04
22	0.901995D-06	-0.784131D-05	-0.134203D-05	0.462079D-08	-0.629608D-05
23	0.280344D-05	-0.177796D-05	0.959377D-05	-0.241812D-05	-0.106609D-04
24	0.256955D-05	-0.130198D-05	-0.188066D-05	-0.163448D-05	-0.872772D-05
25	0.446418D-05	0.265163D-04	-0.190748D-04	-0.248432D-04	0.217956D-04
26	0.317532D-05	-0.409610D-05	0.172429D-05	-0.212619D-05	-0.175042D-04

27	0.349045D-05	0.216740D-04	-0.183433D-04	0.211599D-05	0.372717D-05
28	-0.388586D-06	-0.590678D-05	0.160714D-05	0.103480D-04	0.172322D-04
29	-0.774903D-06	0.170046D-04	-0.498581D-05	-0.314473D-05	0.650473D-06
30	0.137111D-05	0.287821D-04	0.122082D-04	-0.286655D-05	-0.823766D-06
31	-0.379056D-05	-0.507973D-05	0.271596D-05	0.774491D-06	0.284232D-05
32	-0.334834D-06	-0.109167D-05	-0.867762D-05	-0.766107D-06	0.156264D-04
33	0.841326D-05	0.302764D-04	0.120910D-06	0.860839D-05	0.865900D-05
34	-0.194987D-04	-0.316248D-03	0.107049D-03	-0.177571D-04	0.347856D-03
35	-0.596001D-04	-0.376344D-04	-0.534416D-03	0.206719D-05	-0.145497D-03
36	0.810337D-05	-0.447645D-04	-0.466244D-04	-0.441156D-05	-0.875070D-05
37	0.121414D-05	0.239373D-04	-0.130626D-04	-0.252711D-04	0.355553D-04
38	-0.311520D-05	0.151960D-04	0.827131D-05	-0.909396D-05	-0.135436D-04
39	0.935982D-04	-0.318675D-03	-0.485475D-03	-0.928736D-04	0.407301D-03
40	0.755312D-04	0.490598D-03	-0.351157D-03	0.544608D-04	0.357346D-03
41	-0.110286D-03	-0.937862D-03	0.627004D-03	0.377115D-03	-0.230195D-03
42	-0.138571D-04	0.102738D-02	0.133831D-03	-0.565884D-04	0.905548D-05

ESTIMATED COVARIANCE MATRIX FOR PARAMETER ESTIMATES

	11	12	13	14	15
11	0.703965D-03				
12	0.711494D-04	0.735283D-03			
13	0.436902D-05	0.976114D-06	0.214992D-04		
14	0.177110D-05	0.136229D-05	0.484968D-05	0.138775D-04	
15	-0.292617D-05	0.539694D-05	0.853185D-06	0.596873D-05	0.329753D-04
16	-0.413003D-05	0.768496D-05	-0.438661D-05	-0.441589D-05	-0.104894D-05
17	0.261885D-05	0.256999D-05	-0.146061D-05	-0.322482D-05	-0.827880D-05
18	0.392712D-06	-0.127491D-04	0.140357D-05	0.354723D-05	0.277582D-05
19	0.628979D-05	0.396568D-05	0.888971D-06	0.307396D-05	0.118555D-05
20	0.440382D-05	0.700328D-06	0.582111D-06	0.130410D-05	0.640540D-05
21	-0.424067D-05	0.537027D-05	0.795887D-07	-0.117508D-05	-0.154085D-05
22	0.418170D-07	-0.336534D-05	-0.137753D-07	0.870843D-06	0.144530D-05
23	0.755787D-05	0.441619D-05	0.443390D-05	0.518212D-05	0.181553D-05
24	0.113572D-04	-0.385968D-05	0.176428D-05	0.357035D-05	0.120599D-04
25	-0.480589D-05	0.227066D-04	-0.875222D-06	-0.256590D-05	-0.182736D-05
26	0.545840D-05	-0.999637D-05	-0.792552D-06	0.472796D-06	0.213389D-05
27	-0.450705D-05	-0.856587D-05	-0.269052D-06	0.101951D-05	0.141889D-05
28	-0.723592D-05	-0.158900D-05	-0.511964D-05	-0.562966D-05	-0.259126D-05
29	-0.219629D-05	-0.697875D-06	-0.138467D-05	-0.339925D-05	-0.106580D-04
30	-0.519198D-06	0.138029D-04	-0.358625D-06	0.166218D-05	0.241604D-05
31	-0.154614D-05	0.215188D-05	-0.572359D-06	-0.612326D-06	-0.107902D-06
32	-0.108440D-04	0.754947D-05	-0.108953D-05	-0.192423D-05	-0.137048D-05
33	-0.550409D-06	0.371333D-05	-0.195302D-07	0.407158D-06	0.157376D-05

34	-0.148318D-03	0.106511D-03	-0.195878D-04	0.183233D-05	0.251758D-04
35	-0.144461D-03	0.149641D-03	0.108369D-04	0.235123D-04	-0.427889D-04
36	-0.243837D-04	-0.293606D-04	0.412319D-06	0.138647D-05	-0.706259D-05
37	0.170757D-05	0.264823D-05	-0.414350D-05	-0.194330D-05	0.633448D-05
38	0.152270D-04	0.221765D-04	0.744149D-06	0.124132D-05	0.291172D-05
39	-0.202082D-03	0.211635D-03	-0.140561D-04	-0.299950D-04	0.341524D-04
40	-0.269109D-04	0.774106D-04	-0.231803D-04	-0.248652D-04	0.316588D-04
41	-0.386084D-04	-0.836329D-04	0.919413D-04	-0.765062D-05	-0.833172D-04
42	-0.315916D-04	0.253672D-04	-0.768676D-05	0.213639D-05	0.180433D-04

ESTIMATED COVARIANCE MATRIX FOR PARAMETER ESTIMATES

	16	17	18	19	20
16	0.505694D-04				
17	0.100018D-04	0.615491D-04			
18	-0.149584D-04	-0.320717D-04	0.447081D-03		
19	-0.295794D-05	-0.110857D-05	0.160730D-05	0.111912D-04	
20	-0.131998D-05	-0.249469D-05	-0.108987D-05	0.277560D-05	0.141286D-04
21	0.220171D-05	0.116354D-04	-0.167751D-04	-0.198661D-05	-0.522956D-05
22	-0.799431D-06	-0.172473D-05	0.162329D-05	0.126004D-05	0.574579D-05
23	-0.229123D-05	-0.107457D-05	-0.156636D-05	0.436217D-05	0.207625D-05
24	-0.149852D-05	-0.747806D-05	0.449594D-05	0.150450D-05	0.788855D-05
25	0.123178D-04	0.250391D-04	-0.446181D-04	-0.239957D-06	-0.246284D-05
26	-0.579401D-06	-0.163028D-05	0.251125D-05	0.202299D-05	0.627749D-05
27	-0.156458D-05	-0.273935D-05	-0.122341D-04	0.160842D-05	0.444295D-05
28	0.782091D-05	0.160566D-05	-0.939828D-06	-0.406816D-05	-0.104095D-05
29	0.229336D-05	0.106681D-04	-0.696264D-05	-0.149189D-05	-0.450217D-05
30	-0.447315D-05	-0.206477D-04	0.725757D-04	0.192693D-06	-0.130063D-05
31	0.812787D-06	0.175810D-05	-0.746857D-05	-0.106445D-05	-0.499832D-05
32	-0.107666D-05	0.299204D-05	0.184632D-05	-0.157660D-05	-0.124675D-05
33	-0.634957D-06	-0.168021D-05	-0.319965D-06	0.289692D-05	0.271256D-05
34	-0.344419D-04	0.311390D-04	-0.580311D-04	-0.746341D-05	0.943632D-06
35	-0.666967D-06	0.419604D-04	-0.113435D-03	-0.178639D-05	-0.836306D-05
36	0.309873D-05	0.139744D-04	-0.145311D-04	-0.996322D-06	0.221081D-05
37	-0.261708D-05	0.139118D-05	-0.489885D-05	-0.270009D-05	-0.583606D-06
38	0.116413D-05	-0.264219D-05	0.289443D-05	0.806676D-06	0.189792D-05
39	0.315008D-04	-0.574156D-04	-0.178053D-04	-0.309009D-04	0.337933D-04
40	-0.119572D-04	-0.356050D-04	0.491929D-04	0.116318D-04	-0.189970D-04
41	-0.932767D-04	0.483265D-04	0.228177D-03	0.173414D-04	0.320602D-05
42	0.163482D-05	-0.505709D-05	0.114882D-04	-0.367593D-06	0.124719D-06

ESTIMATED COVARIANCE MATRIX FOR PARAMETER ESTIMATES

	21	22	23	24	25
21	0.513655D-04				
22	-0.542527D-05	0.215550D-04			
23	-0.397692D-06	0.131689D-05	0.437006D-04		
24	-0.394488D-05	0.284612D-05	0.108538D-04	0.616712D-04	
25	0.179157D-04	-0.120957D-05	-0.675410D-05	-0.274084D-04	0.217487D-03
26	-0.760617D-05	0.788741D-05	0.189944D-05	0.116611D-04	-0.151837D-04
27	-0.872549D-05	0.400287D-05	0.144441D-04	0.426069D-04	-0.512569D-04
28	0.165419D-05	-0.106074D-05	-0.764385D-05	-0.277396D-05	0.281119D-05
29	0.327587D-05	-0.225885D-05	-0.362150D-05	-0.121359D-04	0.632539D-05
30	-0.146318D-04	0.133795D-05	-0.650832D-08	0.326731D-05	-0.433266D-04
31	0.929448D-05	-0.688889D-05	-0.542994D-06	-0.363064D-05	0.705929D-05
32	0.638248D-05	-0.430570D-05	-0.108958D-04	-0.243254D-04	0.435572D-04
33	-0.322610D-05	0.311878D-05	0.284023D-05	-0.527268D-06	-0.103636D-04
34	0.200660D-04	-0.168564D-04	-0.628095D-04	-0.240375D-04	0.132782D-04
35	0.661755D-05	-0.127349D-04	0.200808D-04	-0.323584D-06	0.126334D-03
36	-0.715861D-05	0.665136D-05	0.687364D-05	-0.882028D-06	-0.668063D-05
37	-0.242972D-05	-0.265699D-05	-0.132517D-05	-0.353423D-05	0.407207D-05
38	0.329713D-06	0.204602D-05	-0.127391D-05	0.692162D-06	0.186316D-06
39	-0.367037D-04	-0.966344D-05	0.647853D-04	0.147834D-03	0.427186D-04
40	0.466466D-05	-0.454861D-05	-0.303111D-04	-0.820338D-04	0.110887D-03
41	0.186844D-03	-0.637287D-04	-0.583276D-04	0.630551D-04	0.265341D-03
42	-0.510218D-05	-0.106792D-04	0.424911D-05	0.116085D-04	-0.990058D-05

ESTIMATED COVARIANCE MATRIX FOR PARAMETER ESTIMATES

	26	27	28	29	30
26	0.454234D-04				
27	0.374000D-04	0.389067D-03			
28	0.256097D-07	-0.181763D-06	0.388890D-04		
29	-0.137901D-05	-0.101977D-04	0.845471D-05	0.494197D-04	
30	0.303467D-06	0.105602D-04	-0.576751D-05	-0.173732D-04	0.180866D-03
31	-0.788901D-05	-0.783455D-05	0.367360D-06	0.100099D-04	-0.125915D-04
32	-0.153488D-04	-0.672076D-04	0.665054D-05	0.182929D-04	-0.280042D-04
33	-0.137011D-05	0.309374D-05	-0.101898D-04	-0.372509D-04	0.620887D-04
34	-0.107630D-04	-0.849490D-04	0.459695D-04	-0.379418D-05	0.253384D-04
35	-0.501782D-04	0.235128D-04	0.302027D-04	0.182766D-04	-0.738440D-04
36	-0.391558D-06	0.169961D-04	0.219895D-05	0.104549D-05	-0.240581D-04
37	0.298855D-05	0.109603D-04	0.111450D-05	0.107993D-05	0.805011D-05
38	0.191899D-05	0.729888D-05	-0.221233D-05	-0.171288D-06	0.496644D-05
39	0.248846D-04	-0.552365D-04	0.277180D-04	-0.143446D-04	-0.759574D-04
40	-0.291984D-04	-0.157782D-05	0.152324D-04	0.167485D-04	-0.941728D-04

41	-0.291558D-04	0.467609D-03	-0.749131D-04	-0.166884D-04	0.162709D-03
42	-0.445882D-05	0.571836D-05	-0.867320D-05	-0.677483D-05	0.392282D-04

ESTIMATED COVARIANCE MATRIX FOR PARAMETER ESTIMATES

	31	32	33	34	35
31	0.403397D-04				
32	0.190736D-04	0.174397D-03			
33	-0.287466D-04	-0.649361D-04	0.289358D-03		
34	0.222146D-04	0.375233D-04	0.853571D-04	0.232323D-01	
35	0.277129D-04	-0.352653D-04	0.177744D-04	-0.922880D-03	0.446847D-01
36	-0.537275D-07	-0.141315D-04	0.381095D-06	0.552936D-04	0.800991D-04
37	0.504144D-05	-0.406094D-05	0.787863D-05	0.659595D-04	0.141763D-04
38	0.137077D-05	0.319631D-05	0.130124D-05	0.208671D-04	-0.414949D-04
39	-0.145183D-06	-0.580306D-05	-0.803952D-04	-0.438391D-03	0.189440D-02
40	0.642330D-04	0.146701D-03	0.112957D-03	0.113045D-03	0.184541D-02
41	-0.724780D-04	-0.461837D-03	0.508131D-03	0.232467D-02	0.747724D-04
42	0.167644D-04	0.491521D-05	0.116136D-04	-0.111937D-03	-0.116220D-03

ESTIMATED COVARIANCE MATRIX FOR PARAMETER ESTIMATES

	36	37	38	39	40
36	0.935505D-03				
37	0.103383D-03	0.567524D-03			
38	-0.514533D-04	0.115407D-05	0.127095D-03		
39	0.324703D-03	-0.827809D-04	-0.286592D-04	0.675862D-01	
40	-0.194599D-03	0.475643D-04	-0.179035D-04	-0.518809D-02	0.560216D-01
41	0.424983D-03	-0.297951D-03	0.476990D-04	-0.235646D-02	-0.153124D-01
42	-0.556204D-04	0.645494D-03	0.850141D-04	-0.104284D-03	-0.199113D-03

ESTIMATED COVARIANCE MATRIX FOR PARAMETER ESTIMATES

	41	42
41	0.199466D+00	
42	-0.886609D-03	0.205399D-02

ESTIMATED CORRELATION MATRIX FOR PARAMETER ESTIMATES

	1	2	3	4	5
1	1.000				

2	0.234	1.000			
3	-0.084	-0.172	1.000		
4	0.088	0.254	-0.116	1.000	
5	0.062	0.176	-0.090	0.228	1.000
6	-0.108	-0.168	0.181	-0.155	-0.169
7	-0.028	-0.007	-0.017	-0.018	-0.047
8	-0.012	-0.019	0.034	0.009	-0.015
9	-0.057	-0.015	0.049	-0.008	0.007
10	0.031	-0.014	-0.015	0.030	-0.034
11	0.050	0.030	0.026	-0.013	0.013
12	0.024	0.019	-0.039	0.027	0.005
13	-0.013	0.019	0.030	-0.034	-0.014
14	-0.023	0.037	0.023	-0.026	-0.009
15	-0.049	0.029	0.002	0.005	0.010
16	-0.008	0.000	0.011	0.010	0.018
17	0.040	-0.002	-0.018	0.005	0.019
18	0.004	0.028	0.010	0.017	-0.009
19	0.017	-0.009	-0.023	-0.011	0.003
20	-0.013	-0.005	-0.010	0.002	0.002
21	0.004	0.018	0.000	0.000	-0.003
22	-0.003	-0.019	0.019	-0.017	0.030
23	-0.024	-0.007	-0.038	-0.035	0.000
24	-0.006	0.036	-0.012	0.023	-0.006
25	0.017	-0.003	-0.021	-0.011	-0.023
26	-0.026	-0.035	0.014	0.010	0.004
27	-0.028	-0.036	-0.014	0.005	-0.015
28	-0.026	0.006	0.009	0.011	-0.005
29	0.042	0.009	0.020	-0.010	0.009
30	-0.014	0.006	-0.014	0.006	-0.022
31	0.036	0.037	0.008	-0.008	0.019
32	0.008	0.002	0.021	-0.040	-0.017
33	-0.006	-0.002	0.005	0.006	0.016
34	-0.017	-0.020	-0.041	-0.007	-0.006
35	0.002	0.006	0.029	-0.004	0.018
36	-0.014	-0.003	-0.001	0.004	0.019
37	-0.049	0.026	-0.002	-0.005	0.006
38	-0.013	-0.046	0.012	0.000	-0.013
39	-0.012	0.008	-0.003	0.019	0.002
40	0.004	-0.008	-0.046	-0.023	0.059
41	0.016	0.069	0.057	0.027	-0.018
42	-0.070	0.024	-0.016	-0.033	-0.045

ESTIMATED CORRELATION MATRIX FOR PARAMETER ESTIMATES

	6	7	8	9	10
6	1.000				
7	0.003	1.000			
8	0.021	-0.114	1.000		
9	-0.014	0.000	0.154	1.000	
10	0.010	0.019	-0.159	0.045	1.000
11	0.029	-0.014	-0.096	-0.021	-0.173
12	0.032	0.022	0.133	-0.095	0.113
13	0.003	0.004	0.011	-0.006	-0.030
14	-0.030	0.009	-0.004	-0.024	-0.047
15	0.014	-0.008	0.025	-0.010	-0.005
16	0.015	-0.003	0.034	-0.008	0.001
17	0.029	-0.003	-0.044	-0.017	-0.028
18	0.015	0.013	0.061	0.047	-0.003
19	0.062	0.049	0.001	0.000	-0.036
20	0.006	-0.009	-0.025	0.013	-0.020
21	-0.011	-0.016	-0.022	-0.004	0.041
22	0.015	-0.025	-0.007	0.000	-0.028
23	0.032	-0.004	0.033	-0.015	-0.033
24	0.025	-0.002	-0.005	-0.008	-0.023
25	0.023	0.027	-0.030	-0.069	0.030
26	0.036	-0.009	0.006	-0.013	-0.053
27	0.013	0.016	-0.021	0.004	0.004
28	-0.005	-0.014	0.006	0.067	0.057
29	-0.008	0.036	-0.016	-0.018	0.002
30	0.008	0.032	0.021	-0.009	-0.001
31	-0.045	-0.012	0.010	0.005	0.009
32	-0.002	-0.001	-0.015	-0.002	0.024
33	0.038	0.026	0.000	0.021	0.010
34	-0.010	-0.031	0.016	-0.005	0.047
35	-0.021	-0.003	-0.058	0.000	-0.014
36	0.020	-0.022	-0.035	-0.006	-0.006
37	0.004	0.015	-0.013	-0.043	0.031
38	-0.021	0.020	0.017	-0.033	-0.025
39	0.027	-0.018	-0.043	-0.015	0.032
40	0.024	0.031	-0.034	0.009	0.031
41	-0.019	-0.031	0.032	0.034	-0.011
42	-0.023	0.335	0.068	-0.051	0.004

ESTIMATED CORRELATION MATRIX FOR PARAMETER ESTIMATES

	11	12	13	14	15

11	1.000				
12	0.099	1.000			
13	0.036	0.008	1.000		
14	0.018	0.013	0.281	1.000	
15	-0.019	0.035	0.032	0.279	1.000
16	-0.022	0.040	-0.133	-0.167	-0.026
17	0.013	0.012	-0.040	-0.110	-0.184
18	0.001	-0.022	0.014	0.045	0.023
19	0.071	0.044	0.057	0.247	0.062
20	0.044	0.007	0.033	0.093	0.297
21	-0.022	0.028	0.002	-0.044	-0.037
22	0.000	-0.027	-0.001	0.050	0.054
23	0.043	0.025	0.145	0.210	0.048
24	0.055	-0.018	0.048	0.122	0.267
25	-0.012	0.057	-0.013	-0.047	-0.022
26	0.031	-0.055	-0.025	0.019	0.055
27	-0.009	-0.016	-0.003	0.014	0.013
28	-0.044	-0.009	-0.177	-0.242	-0.072
29	-0.012	-0.004	-0.042	-0.130	-0.264
30	-0.001	0.038	-0.006	0.033	0.031
31	-0.009	0.012	-0.019	-0.026	-0.003
32	-0.031	0.021	-0.018	-0.039	-0.018
33	-0.001	0.008	0.000	0.006	0.016
34	-0.037	0.026	-0.028	0.003	0.029
35	-0.026	0.026	0.011	0.030	-0.035
36	-0.030	-0.035	0.003	0.012	-0.040
37	0.003	0.004	-0.038	-0.022	0.046
38	0.051	0.073	0.014	0.030	0.045
39	-0.029	0.030	-0.012	-0.031	0.023
40	-0.004	0.012	-0.021	-0.028	0.023
41	-0.003	-0.007	0.044	-0.005	-0.032
42	-0.026	0.021	-0.037	0.013	0.069

ESTIMATED CORRELATION MATRIX FOR PARAMETER ESTIMATES

	16	17	18	19	20
16	1.000				
17	0.179	1.000			
18	-0.099	-0.193	1.000		
19	-0.124	-0.042	0.023	1.000	
20	-0.049	-0.085	-0.014	0.221	1.000
21	0.043	0.207	-0.111	-0.083	-0.194
22	-0.024	-0.047	0.017	0.081	0.329

23	-0.049	-0.021	-0.011	0.197	0.084
24	-0.027	-0.121	0.027	0.057	0.267
25	0.117	0.216	-0.143	-0.005	-0.044
26	-0.012	-0.031	0.018	0.090	0.248
27	-0.011	-0.018	-0.029	0.024	0.060
28	0.176	0.033	-0.007	-0.195	-0.044
29	0.046	0.193	-0.047	-0.063	-0.170
30	-0.047	-0.196	0.255	0.004	-0.026
31	0.018	0.035	-0.056	-0.050	-0.209
32	-0.011	0.029	0.007	-0.036	-0.025
33	-0.005	-0.013	-0.001	0.051	0.042
34	-0.032	0.026	-0.018	-0.015	0.002
35	0.000	0.025	-0.025	-0.003	-0.011
36	0.014	0.058	-0.022	-0.010	0.019
37	-0.015	0.007	-0.010	-0.034	-0.007
38	0.015	-0.030	0.012	0.021	0.045
39	0.017	-0.028	-0.003	-0.036	0.035
40	-0.007	-0.019	0.010	0.015	-0.021
41	-0.029	0.014	0.024	0.012	0.002
42	0.005	-0.014	0.012	-0.002	0.001

ESTIMATED CORRELATION MATRIX FOR PARAMETER ESTIMATES

	21	22	23	24	25
21	1.000				
22	-0.163	1.000			
23	-0.008	0.043	1.000		
24	-0.070	0.078	0.209	1.000	
25	0.170	-0.018	-0.069	-0.237	1.000
26	-0.157	0.252	0.043	0.220	-0.153
27	-0.062	0.044	0.111	0.275	-0.176
28	0.037	-0.037	-0.185	-0.057	0.031
29	0.065	-0.069	-0.078	-0.220	0.061
30	-0.152	0.021	0.000	0.031	-0.218
31	0.204	-0.234	-0.013	-0.073	0.075
32	0.067	-0.070	-0.125	-0.235	0.224
33	-0.026	0.039	0.025	-0.004	-0.041
34	0.018	-0.024	-0.062	-0.020	0.006
35	0.004	-0.013	0.014	0.000	0.041
36	-0.033	0.047	0.034	-0.004	-0.015
37	-0.014	-0.024	-0.008	-0.019	0.012
38	0.004	0.039	-0.017	0.008	0.001
39	-0.020	-0.008	0.038	0.072	0.011

40	0.003	-0.004	-0.019	-0.044	0.032
41	0.058	-0.031	-0.020	0.018	0.040
42	-0.016	-0.051	0.014	0.033	-0.015

ESTIMATED CORRELATION MATRIX FOR PARAMETER ESTIMATES

	26	27	28	29	30
26	1.000				
27	0.281	1.000			
28	0.001	-0.001	1.000		
29	-0.029	-0.074	0.193	1.000	
30	0.003	0.040	-0.069	-0.184	1.000
31	-0.184	-0.063	0.009	0.224	-0.147
32	-0.172	-0.258	0.081	0.197	-0.158
33	-0.012	0.009	-0.096	-0.312	0.271
34	-0.010	-0.028	0.048	-0.004	0.012
35	-0.035	0.006	0.023	0.012	-0.026
36	-0.002	0.028	0.012	0.005	-0.058
37	0.019	0.023	0.008	0.006	0.025
38	0.025	0.033	-0.031	-0.002	0.033
39	0.014	-0.011	0.017	-0.008	-0.022
40	-0.018	0.000	0.010	0.010	-0.030
41	-0.010	0.053	-0.027	-0.005	0.027
42	-0.015	0.006	-0.031	-0.021	0.064

ESTIMATED CORRELATION MATRIX FOR PARAMETER ESTIMATES

	31	32	33	34	35
31	1.000				
32	0.227	1.000			
33	-0.266	-0.289	1.000		
34	0.023	0.019	0.033	1.000	
35	0.021	-0.013	0.005	-0.029	1.000
36	0.000	-0.035	0.001	0.012	0.012
37	0.033	-0.013	0.019	0.018	0.003
38	0.019	0.021	0.007	0.012	-0.017
39	0.000	-0.002	-0.018	-0.011	0.034
40	0.043	0.047	0.028	0.003	0.037
41	-0.026	-0.078	0.067	0.034	0.001
42	0.058	0.008	0.015	-0.016	-0.012

ESTIMATED CORRELATION MATRIX FOR PARAMETER ESTIMATES

	36	37	38	39	40
36	1.000				
37	0.142	1.000			
38	-0.149	0.004	1.000		
39	0.041	-0.013	-0.010	1.000	
40	-0.027	0.008	-0.007	-0.084	1.000
41	0.031	-0.028	0.009	-0.020	-0.145
42	-0.040	0.598	0.166	-0.009	-0.019

ESTIMATED CORRELATION MATRIX FOR PARAMETER ESTIMATES

	41	42
41	1.000	
42	-0.044	1.000

TECHNICAL 8 OUTPUT

TECHNICAL 8 OUTPUT FOR BAYES ESTIMATION

CHAIN	BSEED
1	0
2	285380
3	253358

ITERATION	POTENTIAL SCALE REDUCTION	PARAMETER WITH HIGHEST PSR
100	1.014	40
200	1.004	37
300	1.001	30
400	1.005	36
500	1.005	37
600	1.004	37
700	1.001	37
800	1.002	8
900	1.004	37
1000	1.003	42

Linear effects, improvement in physical health

2	4 166
3	65 74 80 30
4	84 22
5	205
6	228
7	93
8	106 144 165 71 170 173 192 72 50
9	40 108
11	107 83 181 61 75 68
12	69 174 206 226 81
13	162
16	92 154
19	114
20	145 2
22	116
23	36 156 169 142
24	152
26	98
28	122 202
30	178
32	6
46	24
59	204
62	125
64	209
108	217
216	110
223	100

COVARIANCE COVERAGE OF DATA

Minimum covariance coverage value 0.100

Number of missing data patterns 4

PROPORTION OF DATA PRESENT

Covariance Coverage

	PHYCAT16	PHY15	KES15	CHOICE15	BULLY15
PHYCAT16	1.000				
PHY15	1.000	1.000			
KES15	0.971	0.971	0.971		
CHOICE15	0.963	0.963	0.963	0.963	
BULLY15	0.966	0.966	0.966	0.963	0.966
PRESS15	0.966	0.966	0.966	0.963	0.966
DUTIES15	0.966	0.966	0.966	0.963	0.966
PRAFAC	1.000	1.000	0.971	0.963	0.966

Covariance Coverage

	PRESS15	DUTIES15	PRAFAC
PRESS15	0.966		
DUTIES15	0.966	0.966	
PRAFAC	0.966	0.966	1.000

UNIVARIATE PROPORTIONS AND COUNTS FOR CATEGORICAL VARIABLES

PHYCAT16		
Category 1	0.364	517.000
Category 2	0.636	905.000

UNIVARIATE SAMPLE STATISTICS

UNIVARIATE HIGHER-ORDER MOMENT DESCRIPTIVE STATISTICS

Variable/ Sample Size	Mean/ Variance	Skewness/ Kurtosis	Minimum/ Maximum	% with Min/Max	Percentiles 20%/60%	40%/80%	Median
PHY15	0.000	2.446	-0.155	85.58%	-0.155	-0.155	-0.155
1422.000	0.154	5.391	1.845	1.13%	-0.155	-0.155	
KES15	0.000	1.141	-0.953	8.62%	-0.620	-0.287	-0.120
1381.000	0.613	1.071	3.047	0.36%	0.047	0.547	
CHOICE15	0.000	-0.138	-1.878	14.60%	-0.878	0.122	0.122
1370.000	1.250	-0.758	2.122	5.77%	0.122	1.122	
BULLY15	0.000	2.479	-0.312	79.02%	-0.312	-0.312	-0.312

```
        1373.000       0.474       6.315       3.688    0.44%      -0.312      0.688
     PRESS15           0.000       0.183      -1.756   14.06%      -0.756     -0.756      0.244
        1373.000       1.207      -0.514       2.244    7.21%       0.244      1.244
     DUTIES15          0.000      -0.896      -2.873    3.06%      -0.873      0.127      0.127
        1373.000       0.975       0.587       1.127   27.75%       0.127      1.127
     PRAFAC           -0.173       0.219      -2.338    1.75%      -1.489     -0.520     -0.258
          57.000       1.361      -0.662       2.338    1.75%       0.080      0.848


THE MODEL ESTIMATION TERMINATED NORMALLY

     USE THE FBITERATIONS OPTION TO INCREASE THE NUMBER OF ITERATIONS BY A FACTOR
     OF AT LEAST TWO TO CHECK CONVERGENCE AND THAT THE PSR VALUE DOES NOT INCREASE.



MODEL FIT INFORMATION

Number of Free Parameters                              36

Bayesian Posterior Predictive Checking using Chi-Square

          95% Confidence Interval for the Difference Between
          the Observed and the Replicated Chi-Square Values

                              -27.990            21.351

          Posterior Predictive P-Value              0.490



MODEL RESULTS

                                Posterior  One-Tailed         95% C.I.
                    Estimate       S.D.      P-Value   Lower 2.5%  Upper 2.5%  Significance

Within Level

 PHYCAT16   ON
    PHY15              0.824       0.119      0.000       0.593       1.071      *
    CHOICE15          -0.048       0.033      0.071      -0.112       0.015
    BULLY15            0.049       0.057      0.193      -0.061       0.158
    DUTIES15           0.038       0.038      0.167      -0.037       0.113
    PRESS15           -0.001       0.036      0.491      -0.072       0.069
```

KES15	0.166	0.053	0.003	0.055	0.265	*
**KES15 WITH**						
PHY15	0.070	0.009	0.000	0.054	0.086	*
CHOICE15	-0.135	0.025	0.000	-0.187	-0.089	*
BULLY15	0.127	0.015	0.000	0.098	0.156	*
PRESS15	0.206	0.024	0.000	0.163	0.254	*
DUTIES15	-0.205	0.022	0.000	-0.248	-0.164	*
**PHY15 WITH**						
CHOICE15	-0.033	0.012	0.003	-0.057	-0.010	*
BULLY15	0.013	0.007	0.049	-0.002	0.027	
PRESS15	0.015	0.012	0.102	-0.008	0.039	
DUTIES15	-0.008	0.010	0.221	-0.030	0.012	
**CHOICE15 WITH**						
BULLY15	-0.132	0.021	0.000	-0.176	-0.095	*
PRESS15	-0.183	0.034	0.000	-0.253	-0.121	*
DUTIES15	0.160	0.031	0.000	0.102	0.224	*
**BULLY15 WITH**						
PRESS15	0.178	0.021	0.000	0.138	0.220	*
DUTIES15	-0.139	0.019	0.000	-0.179	-0.102	*
**PRESS15 WITH**						
DUTIES15	-0.242	0.030	0.000	-0.300	-0.182	*
**Means**						
PHY15	0.000	0.010	0.497	-0.020	0.020	
KES15	0.001	0.021	0.477	-0.041	0.044	
CHOICE15	-0.001	0.030	0.480	-0.059	0.057	
BULLY15	0.002	0.019	0.465	-0.037	0.038	
PRESS15	0.003	0.031	0.472	-0.059	0.064	
DUTIES15	0.001	0.027	0.492	-0.052	0.052	
**Variances**						
PHY15	0.154	0.006	0.000	0.144	0.166	*
KES15	0.614	0.023	0.000	0.570	0.662	*
CHOICE15	1.251	0.050	0.000	1.162	1.350	*
BULLY15	0.475	0.018	0.000	0.441	0.510	*
PRESS15	1.207	0.047	0.000	1.120	1.297	*
DUTIES15	0.974	0.038	0.000	0.906	1.055	*

Between Level

```
 PHYCAT16   ON
    PRAFAC            -0.050       0.040      0.109      -0.126       0.027

 Thresholds
    PHYCAT16$1        -0.390       0.042      0.000      -0.477      -0.308      *

 Residual Variances
    PHYCAT16           0.007       0.009      0.000       0.001       0.035      *


STANDARDIZED MODEL RESULTS


STDYX Standardization

                                Posterior  One-Tailed         95% C.I.
                    Estimate       S.D.      P-Value   Lower 2.5%  Upper 2.5%  Significance
Within Level

 PHYCAT16   ON
    PHY15              0.301       0.040      0.000       0.221       0.381      *
    CHOICE15          -0.050       0.034      0.071      -0.117       0.016
    BULLY15            0.031       0.036      0.193      -0.038       0.102
    DUTIES15           0.034       0.035      0.167      -0.033       0.102
    PRESS15           -0.001       0.037      0.491      -0.073       0.070
    KES15              0.121       0.038      0.003       0.040       0.192      *

 KES15    WITH
    PHY15              0.228       0.026      0.000       0.179       0.277      *
    CHOICE15          -0.155       0.027      0.000      -0.210      -0.101      *
    BULLY15            0.235       0.025      0.000       0.183       0.284      *
    PRESS15            0.240       0.025      0.000       0.193       0.293      *
    DUTIES15          -0.265       0.025      0.000      -0.313      -0.215      *

 PHY15    WITH
    CHOICE15          -0.074       0.027      0.003      -0.128      -0.024      *
    BULLY15            0.046       0.027      0.049      -0.008       0.099
    PRESS15            0.034       0.027      0.102      -0.018       0.088
    DUTIES15          -0.022       0.027      0.221      -0.076       0.031

 CHOICE15 WITH
    BULLY15           -0.172       0.025      0.000      -0.223      -0.125      *
    PRESS15           -0.149       0.027      0.000      -0.202      -0.099      *
```

```
   DUTIES15           0.145       0.027       0.000       0.093       0.200      *

 BULLY15  WITH
   PRESS15            0.236       0.025       0.000       0.186       0.283      *
   DUTIES15          -0.205       0.026       0.000      -0.257      -0.152      *

 PRESS15  WITH
   DUTIES15          -0.224       0.026       0.000      -0.272      -0.170      *

 Means
   PHY15             -0.001       0.026       0.497      -0.051       0.052
   KES15              0.001       0.027       0.477      -0.052       0.056
   CHOICE15          -0.001       0.027       0.480      -0.054       0.050
   BULLY15            0.003       0.027       0.465      -0.053       0.055
   PRESS15            0.003       0.028       0.472      -0.054       0.058
   DUTIES15           0.001       0.027       0.492      -0.052       0.052

 Variances
   PHY15              1.000       0.000       0.000       1.000       1.000
   KES15              1.000       0.000       0.000       1.000       1.000
   CHOICE15           1.000       0.000       0.000       1.000       1.000
   BULLY15            1.000       0.000       0.000       1.000       1.000
   PRESS15            1.000       0.000       0.000       1.000       1.000
   DUTIES15           1.000       0.000       0.000       1.000       1.000

Between Level

 PHYCAT16   ON
   PRAFAC            -0.558       0.355       0.109      -0.958       0.397

 Thresholds
   PHYCAT16$1        -3.514       1.795       0.000      -8.769      -1.724      *

 Residual Variances
   PHYCAT16           0.680       0.276       0.000       0.082       0.999      *


R-SQUARE

Within Level

                                Posterior  One-Tailed         95% C.I.
   Variable        Estimate       S.D.      P-Value   Lower 2.5%  Upper 2.5%
```

PHYCAT16	0.136	0.027	0.000	0.088	0.195

Between Level

Variable	Estimate	Posterior S.D.	One-Tailed P-Value	95% C.I. Lower 2.5%	Upper 2.5%
PHYCAT16	0.320	0.276	0.000	0.001	0.918

TECHNICAL 1 OUTPUT

PARAMETER SPECIFICATION FOR WITHIN

TAU

PHYCAT16
0

NU

PHYCAT16	PHY15	KES15	CHOICE15	BULLY15
0	0	0	0	0

NU

PRESS15	DUTIES15
0	0

LAMBDA

	PHYCAT16	PHY15	KES15	CHOICE15	BULLY15
PHYCAT16	0	0	0	0	0
PHY15	0	0	0	0	0
KES15	0	0	0	0	0
CHOICE15	0	0	0	0	0
BULLY15	0	0	0	0	0
PRESS15	0	0	0	0	0
DUTIES15	0	0	0	0	0

LAMBDA

	PRESS15	DUTIES15
PHYCAT16	0	0
PHY15	0	0
KES15	0	0
CHOICE15	0	0
BULLY15	0	0
PRESS15	0	0
DUTIES15	0	0

THETA

	PHYCAT16	PHY15	KES15	CHOICE15	BULLY15
PHYCAT16	0				
PHY15	0	0			
KES15	0	0	0		
CHOICE15	0	0	0	0	
BULLY15	0	0	0	0	0
PRESS15	0	0	0	0	0
DUTIES15	0	0	0	0	0

THETA

	PRESS15	DUTIES15
PRESS15	0	
DUTIES15	0	0

ALPHA

PHYCAT16	PHY15	KES15	CHOICE15	BULLY15
0	1	2	3	4

ALPHA

PRESS15	DUTIES15
5	6

BETA

	PHYCAT16	PHY15	KES15	CHOICE15	BULLY15
PHYCAT16	0	7	8	9	10
PHY15	0	0	0	0	0
KES15	0	0	0	0	0
CHOICE15	0	0	0	0	0
BULLY15	0	0	0	0	0
PRESS15	0	0	0	0	0
DUTIES15	0	0	0	0	0

BETA

	PRESS15	DUTIES15
PHYCAT16	11	12
PHY15	0	0
KES15	0	0
CHOICE15	0	0
BULLY15	0	0
PRESS15	0	0
DUTIES15	0	0

PSI

	PHYCAT16	PHY15	KES15	CHOICE15	BULLY15
PHYCAT16	0				
PHY15	0	13			
KES15	0	14	15		
CHOICE15	0	16	17	18	
BULLY15	0	19	20	21	22
PRESS15	0	23	24	25	26
DUTIES15	0	28	29	30	31

PSI

	PRESS15	DUTIES15
PRESS15	27	
DUTIES15	32	33

PARAMETER SPECIFICATION FOR BETWEEN

TAU

	PHYCAT16
	36

NU

	PHYCAT16	PRAFAC
	0	0

LAMBDA

	PHYCAT16	PRAFAC
PHYCAT16	0	0
PRAFAC	0	0

THETA

	PHYCAT16	PRAFAC
PHYCAT16	0	
PRAFAC	0	0

ALPHA

	PHYCAT16	PRAFAC
	0	0

BETA

	PHYCAT16	PRAFAC
PHYCAT16	0	34
PRAFAC	0	0

PSI

PHYCAT16 PRAFAC

PHYCAT16	35	
PRAFAC	0	0

STARTING VALUES FOR WITHIN

TAU

PHYCAT16
0.000

NU

PHYCAT16	PHY15	KES15	CHOICE15	BULLY15
0.000	0.000	0.000	0.000	0.000

NU

PRESS15	DUTIES15
0.000	0.000

LAMBDA

	PHYCAT16	PHY15	KES15	CHOICE15	BULLY15
PHYCAT16	1.000	0.000	0.000	0.000	0.000
PHY15	0.000	1.000	0.000	0.000	0.000
KES15	0.000	0.000	1.000	0.000	0.000
CHOICE15	0.000	0.000	0.000	1.000	0.000
BULLY15	0.000	0.000	0.000	0.000	1.000
PRESS15	0.000	0.000	0.000	0.000	0.000
DUTIES15	0.000	0.000	0.000	0.000	0.000

LAMBDA

	PRESS15	DUTIES15
PHYCAT16	0.000	0.000
PHY15	0.000	0.000
KES15	0.000	0.000

CHOICE15	0.000	0.000
BULLY15	0.000	0.000
PRESS15	1.000	0.000
DUTIES15	0.000	1.000

THETA

	PHYCAT16	PHY15	KES15	CHOICE15	BULLY15
PHYCAT16	0.000				
PHY15	0.000	0.000			
KES15	0.000	0.000	0.000		
CHOICE15	0.000	0.000	0.000	0.000	
BULLY15	0.000	0.000	0.000	0.000	0.000
PRESS15	0.000	0.000	0.000	0.000	0.000
DUTIES15	0.000	0.000	0.000	0.000	0.000

THETA

	PRESS15	DUTIES15
PRESS15	0.000	
DUTIES15	0.000	0.000

ALPHA

PHYCAT16	PHY15	KES15	CHOICE15	BULLY15
0.000	0.000	0.000	0.000	0.000

ALPHA

PRESS15	DUTIES15
0.000	0.000

BETA

	PHYCAT16	PHY15	KES15	CHOICE15	BULLY15
PHYCAT16	0.000	0.000	0.000	0.000	0.000
PHY15	0.000	0.000	0.000	0.000	0.000
KES15	0.000	0.000	0.000	0.000	0.000
CHOICE15	0.000	0.000	0.000	0.000	0.000

BULLY15	0.000	0.000	0.000	0.000	0.000
PRESS15	0.000	0.000	0.000	0.000	0.000
DUTIES15	0.000	0.000	0.000	0.000	0.000

BETA

	PRESS15	DUTIES15
PHYCAT16	0.000	0.000
PHY15	0.000	0.000
KES15	0.000	0.000
CHOICE15	0.000	0.000
BULLY15	0.000	0.000
PRESS15	0.000	0.000
DUTIES15	0.000	0.000

PSI

	PHYCAT16	PHY15	KES15	CHOICE15	BULLY15
PHYCAT16	1.000				
PHY15	0.000	0.077			
KES15	0.000	0.000	0.306		
CHOICE15	0.000	0.000	0.000	0.625	
BULLY15	0.000	0.000	0.000	0.000	0.237
PRESS15	0.000	0.000	0.000	0.000	0.000
DUTIES15	0.000	0.000	0.000	0.000	0.000

PSI

	PRESS15	DUTIES15
PRESS15	0.604	
DUTIES15	0.000	0.488

STARTING VALUES FOR BETWEEN

TAU

PHYCAT16
-0.311

NU

PHYCAT16	PRAFAC
0.000	0.000

LAMBDA

	PHYCAT16	PRAFAC
PHYCAT16	1.000	0.000
PRAFAC	0.000	1.000

THETA

	PHYCAT16	PRAFAC
PHYCAT16	0.000	
PRAFAC	0.000	0.000

ALPHA

PHYCAT16	PRAFAC
0.000	0.000

BETA

	PHYCAT16	PRAFAC
PHYCAT16	0.000	0.000
PRAFAC	0.000	0.000

PSI

	PHYCAT16	PRAFAC
PHYCAT16	1.000	
PRAFAC	0.000	0.480

PRIORS FOR ALL PARAMETERS PRIOR MEAN PRIOR VARIANCE PRIOR STD. DEV.

```
     Parameter 1~N(0.000,infinity)          0.0000            infinity            infinity
     Parameter 2~N(0.000,infinity)          0.0000            infinity            infinity
     Parameter 3~N(0.000,infinity)          0.0000            infinity            infinity
     Parameter 4~N(0.000,infinity)          0.0000            infinity            infinity
     Parameter 5~N(0.000,infinity)          0.0000            infinity            infinity
     Parameter 6~N(0.000,infinity)          0.0000            infinity            infinity
     Parameter 7~N(0.000,5.000)             0.0000              5.0000              2.2361
     Parameter 8~N(0.000,5.000)             0.0000              5.0000              2.2361
     Parameter 9~N(0.000,5.000)             0.0000              5.0000              2.2361
     Parameter 10~N(0.000,5.000)            0.0000              5.0000              2.2361
     Parameter 11~N(0.000,5.000)            0.0000              5.0000              2.2361
     Parameter 12~N(0.000,5.000)            0.0000              5.0000              2.2361
     Parameter 13~IW(1.000,7)             infinity            infinity            infinity
     Parameter 14~IW(0.000,7)             infinity            infinity            infinity
     Parameter 15~IW(1.000,7)             infinity            infinity            infinity
     Parameter 16~IW(0.000,7)             infinity            infinity            infinity
     Parameter 17~IW(0.000,7)             infinity            infinity            infinity
     Parameter 18~IW(1.000,7)             infinity            infinity            infinity
     Parameter 19~IW(0.000,7)             infinity            infinity            infinity
     Parameter 20~IW(0.000,7)             infinity            infinity            infinity
     Parameter 21~IW(0.000,7)             infinity            infinity            infinity
     Parameter 22~IW(1.000,7)             infinity            infinity            infinity
     Parameter 23~IW(0.000,7)             infinity            infinity            infinity
     Parameter 24~IW(0.000,7)             infinity            infinity            infinity
     Parameter 25~IW(0.000,7)             infinity            infinity            infinity
     Parameter 26~IW(0.000,7)             infinity            infinity            infinity
     Parameter 27~IW(1.000,7)             infinity            infinity            infinity
     Parameter 28~IW(0.000,7)             infinity            infinity            infinity
     Parameter 29~IW(0.000,7)             infinity            infinity            infinity
     Parameter 30~IW(0.000,7)             infinity            infinity            infinity
     Parameter 31~IW(0.000,7)             infinity            infinity            infinity
     Parameter 32~IW(0.000,7)             infinity            infinity            infinity
     Parameter 33~IW(1.000,7)             infinity            infinity            infinity
     Parameter 34~N(0.000,5.000)            0.0000              5.0000              2.2361
     Parameter 35~IG(-1.000,0.000)        infinity            infinity            infinity
     Parameter 36~N(0.000,5.000)            0.0000              5.0000              2.2361


TECHNICAL 3 OUTPUT


           ESTIMATED COVARIANCE MATRIX FOR PARAMETER ESTIMATES
                  1             2             3             4             5
              ________      ________      ________      ________      ________
```

1	0.108202D-03				
2	0.520260D-04	0.459835D-03			
3	-0.164374D-04	-0.859966D-04	0.879573D-03		
4	0.739645D-05	0.100545D-03	-0.941884D-04	0.355344D-03	
5	0.174757D-04	0.152264D-03	-0.137242D-03	0.138161D-03	0.952070D-03
6	-0.221231D-04	-0.152978D-03	0.142969D-03	-0.882361D-04	-0.183816D-03
7	-0.829533D-05	-0.426381D-04	0.512133D-04	0.727148D-06	-0.644874D-04
8	0.412277D-05	0.517346D-04	0.163539D-04	0.129475D-04	0.142186D-04
9	-0.949745D-05	0.184300D-06	0.237654D-04	-0.277036D-04	0.429866D-05
10	0.686255D-05	-0.309444D-04	0.326599D-04	0.853406D-05	-0.175653D-04
11	-0.924988D-05	-0.328688D-04	-0.211058D-04	-0.286981D-04	0.424169D-04
12	-0.714377D-05	-0.402845D-05	0.616120D-05	0.930526D-05	-0.339732D-04
13	-0.189435D-05	-0.392722D-05	0.303006D-05	-0.540336D-06	-0.824684D-05
14	0.252894D-05	0.227999D-05	-0.670076D-05	0.214988D-05	-0.805231D-05
15	-0.170226D-06	0.663639D-05	-0.460839D-04	0.265448D-05	-0.660765D-05
16	0.127439D-05	0.608506D-05	0.986902D-05	-0.570990D-05	-0.819924D-05
17	0.681203D-07	-0.157933D-04	0.832492D-05	-0.181672D-04	-0.403234D-05
18	-0.505410D-05	-0.349826D-04	0.579785D-04	0.185109D-04	-0.412602D-04
19	0.729180D-07	-0.191661D-05	-0.448039D-05	0.748823D-05	0.590161D-05
20	-0.397484D-05	0.439244D-05	-0.164643D-04	0.554238D-05	-0.353603D-06
21	-0.553779D-05	0.754269D-05	0.945741D-05	0.234896D-05	0.152251D-04
22	0.602548D-05	0.144195D-05	-0.180234D-04	0.730613D-06	0.191109D-04
23	0.103765D-05	0.500084D-05	-0.194907D-04	0.105747D-04	0.236292D-04
24	0.491609D-05	0.208596D-04	-0.438305D-04	-0.853681D-05	0.126713D-05
25	-0.644681D-05	-0.464800D-05	-0.371094D-05	-0.118136D-04	-0.351045D-04
26	-0.221103D-05	0.195536D-04	-0.127713D-04	0.803394D-05	0.855512D-05
27	-0.783897D-05	0.172975D-04	-0.312933D-04	-0.107114D-04	0.329789D-04
28	0.378642D-05	-0.332963D-05	-0.326881D-05	-0.193737D-05	-0.229934D-05
29	0.425994D-05	0.180073D-04	0.269734D-04	0.285738D-06	0.166129D-04
30	-0.204954D-05	0.343948D-04	-0.310416D-05	0.193238D-04	-0.740422D-05
31	0.154229D-06	0.586513D-05	0.339854D-04	-0.758992D-06	-0.364144D-05
32	-0.133407D-04	-0.184422D-04	0.224418D-04	0.189116D-04	-0.160011D-04
33	0.160199D-04	0.886064D-05	-0.797929D-04	0.108899D-04	0.610569D-05
34	0.914665D-05	-0.738534D-05	0.496513D-05	0.210628D-05	0.159689D-04
35	-0.333211D-05	-0.468215D-05	0.168178D-06	-0.177627D-05	-0.145070D-04
36	-0.425320D-05	0.517778D-05	-0.401964D-05	-0.823062D-05	0.159931D-05

ESTIMATED COVARIANCE MATRIX FOR PARAMETER ESTIMATES

	6	7	8	9	10
6	0.716046D-03				
7	0.416027D-04	0.142486D-01			
8	-0.350258D-04	-0.100937D-02	0.285341D-02		

9	-0.285449D-04	-0.222820D-04	0.290635D-04	0.106023D-02	
10	0.314117D-04	-0.118182D-03	-0.404294D-03	0.235717D-03	0.320870D-02
11	0.102761D-04	0.120670D-03	-0.365621D-03	0.582287D-04	-0.362047D-03
12	-0.719906D-05	0.642151D-04	0.482293D-03	-0.147818D-03	0.256274D-03
13	0.501519D-05	0.258455D-04	0.161854D-04	0.318252D-05	0.621272D-05
14	-0.354449D-06	0.426626D-04	-0.236067D-05	-0.891627D-05	0.859058D-07
15	-0.150299D-04	0.116791D-03	0.325799D-04	-0.245369D-06	-0.307718D-04
16	-0.332502D-05	0.268904D-04	-0.189995D-04	-0.192231D-05	0.307759D-04
17	-0.211593D-05	-0.872856D-04	0.546436D-05	0.126587D-04	-0.534607D-05
18	0.367830D-04	0.155985D-03	-0.171833D-03	-0.153756D-04	0.465891D-04
19	0.317418D-05	0.368533D-04	-0.156468D-04	-0.717224D-05	0.765467D-05
20	0.179438D-05	0.743518D-04	-0.590271D-05	-0.786631D-05	-0.931581D-05
21	-0.285377D-04	-0.113030D-03	0.357008D-04	0.544848D-05	0.168238D-04
22	-0.889224D-06	0.934807D-05	0.309792D-05	0.122621D-04	0.249072D-04
23	-0.966797D-05	0.309583D-04	0.464715D-05	0.152137D-05	-0.300977D-04
24	-0.767649D-05	-0.152894D-04	-0.616826D-04	0.218577D-05	-0.242222D-04
25	0.446485D-04	0.215490D-04	-0.679861D-05	0.295894D-04	0.769762D-05
26	-0.652616D-06	0.419732D-05	-0.142526D-04	-0.157211D-04	0.507025D-04
27	0.341823D-04	-0.148960D-03	-0.257409D-04	-0.117483D-04	-0.667189D-05
28	0.194608D-06	-0.283183D-04	0.416958D-05	-0.404706D-05	-0.887429D-05
29	-0.262468D-05	0.351010D-04	-0.461968D-05	-0.472388D-05	-0.780417D-05
30	-0.160329D-04	-0.354966D-04	0.577406D-04	0.287121D-05	0.233260D-04
31	-0.122785D-05	-0.967299D-04	0.103238D-04	0.229215D-05	0.309724D-05
32	0.192540D-04	-0.766248D-07	0.114536D-04	-0.676662D-05	0.248486D-04
33	0.809003D-05	0.259294D-04	0.334998D-04	-0.647986D-05	0.222997D-04
34	-0.455757D-05	-0.113954D-03	-0.409401D-06	0.345921D-04	0.338957D-04
35	0.552355D-05	0.359554D-04	0.109086D-04	-0.254502D-05	0.223726D-05
36	0.183717D-04	-0.908908D-03	-0.779187D-04	0.483583D-04	0.147827D-04

ESTIMATED COVARIANCE MATRIX FOR PARAMETER ESTIMATES

	11	12	13	14	15
11	0.131626D-02				
12	0.195249D-03	0.145632D-02			
13	0.514093D-05	-0.311089D-05	0.317444D-04		
14	-0.265432D-05	-0.963989D-05	0.138068D-04	0.724647D-04	
15	0.107627D-04	0.341945D-04	0.344249D-05	0.686086D-04	0.541896D-03
16	0.716084D-05	0.112515D-04	-0.430461D-05	-0.138122D-04	-0.168869D-04
17	0.785695D-06	-0.270876D-04	0.405182D-05	-0.195595D-04	-0.129142D-03
18	-0.306152D-05	-0.678571D-04	0.713231D-05	0.722691D-05	0.305749D-04
19	-0.300486D-05	-0.594009D-05	0.296880D-05	0.159397D-04	0.162992D-04
20	0.123605D-04	0.214845D-04	0.420505D-05	0.118970D-04	0.935761D-04
21	-0.170482D-04	-0.625104D-05	0.961233D-06	-0.256751D-05	-0.272941D-04

22	0.120453D-04	-0.211072D-05	0.266330D-05	0.375781D-05	0.161663D-04
23	-0.117507D-04	-0.138622D-04	0.389805D-05	0.243360D-04	0.215858D-04
24	0.322549D-04	-0.789510D-05	-0.281780D-05	0.183093D-04	0.184825D-03
25	-0.301795D-04	-0.201678D-04	-0.495690D-05	-0.932066D-05	-0.440732D-04
26	0.298229D-04	0.330401D-04	0.175623D-05	-0.115872D-07	0.239867D-04
27	-0.743050D-05	0.868618D-05	-0.316880D-05	0.427892D-05	0.183474D-04
28	0.564952D-05	-0.940057D-05	-0.217535D-05	-0.229202D-04	-0.241810D-04
29	0.150489D-04	-0.302229D-06	-0.271769D-05	-0.165250D-04	-0.178839D-03
30	-0.156907D-04	-0.105412D-05	0.141094D-06	0.578981D-05	0.430782D-04
31	-0.107379D-05	-0.628210D-05	-0.408817D-06	-0.384564D-05	-0.318111D-04
32	-0.303755D-04	0.263437D-04	-0.209417D-05	0.406247D-05	-0.377225D-04
33	-0.329939D-04	0.108911D-04	0.128020D-05	0.590520D-05	0.552412D-04
34	-0.587434D-04	-0.661673D-05	-0.492009D-05	-0.478912D-05	-0.319065D-04
35	-0.144357D-04	0.932860D-05	0.519200D-06	-0.234495D-05	0.132409D-05
36	-0.286551D-04	-0.950060D-04	0.607279D-05	-0.387986D-05	-0.243007D-04

ESTIMATED COVARIANCE MATRIX FOR PARAMETER ESTIMATES

	16	17	18	19	20
16	0.140170D-03				
17	0.650777D-04	0.625609D-03			
18	-0.614881D-04	-0.270631D-03	0.245177D-02		
19	-0.132653D-04	-0.105990D-04	0.916271D-05	0.523435D-04	
20	-0.139176D-04	-0.817485D-04	0.470982D-04	0.290944D-04	0.215676D-03
21	0.135319D-04	0.118720D-03	-0.286104D-03	-0.102867D-04	-0.553182D-04
22	0.197402D-05	-0.179269D-04	0.452998D-04	0.847349D-05	0.865268D-04
23	-0.229751D-04	-0.136507D-04	0.218938D-04	0.229207D-04	0.116924D-04
24	-0.960784D-05	-0.118256D-03	0.604622D-04	0.124600D-04	0.914506D-04
25	0.358221D-04	0.247344D-03	-0.395221D-03	-0.847087D-05	-0.436850D-04
26	0.602462D-05	-0.478750D-04	0.332843D-04	0.633382D-05	0.925654D-04
27	-0.981694D-05	-0.916012D-04	0.149680D-03	0.166743D-04	0.441873D-04
28	0.203006D-04	0.157389D-04	-0.332080D-04	-0.160109D-04	-0.109978D-04
29	0.161186D-04	0.101309D-03	-0.431841D-04	-0.680667D-05	-0.841988D-04
30	-0.227529D-04	-0.252448D-03	0.345835D-03	0.473627D-05	0.441851D-04
31	0.218677D-05	0.315657D-04	-0.668537D-04	-0.741879D-05	-0.853115D-04
32	0.137258D-04	0.824763D-04	-0.116365D-03	-0.252037D-05	-0.382707D-04
33	-0.142820D-04	-0.411883D-04	0.365035D-04	0.830301D-05	0.762331D-04
34	-0.134367D-04	0.139311D-04	-0.355132D-04	0.476939D-05	-0.160333D-04
35	-0.988851D-06	0.566563D-05	-0.473332D-05	0.195406D-05	0.137508D-05
36	0.162411D-04	0.147348D-04	-0.676085D-04	-0.744284D-05	-0.884337D-05

ESTIMATED COVARIANCE MATRIX FOR PARAMETER ESTIMATES

	21	22	23	24	25
21	0.425445D-03				
22	-0.100074D-03	0.319823D-03			
23	-0.507426D-05	0.372290D-05	0.140543D-03		
24	-0.431857D-04	0.304613D-04	0.604401D-04	0.575115D-03	
25	0.166387D-03	-0.214336D-04	-0.335548D-04	-0.163535D-03	0.116607D-02
26	-0.735109D-04	0.115580D-03	0.938267D-05	0.138023D-03	-0.160480D-03
27	-0.557643D-04	0.374276D-04	0.321761D-04	0.390627D-03	-0.325344D-03
28	-0.621895D-05	-0.836587D-05	-0.332766D-04	-0.194517D-04	-0.497040D-05
29	0.108309D-04	-0.285291D-04	-0.166237D-04	-0.138216D-03	0.443836D-04
30	-0.139142D-03	0.256037D-04	0.412745D-05	0.534694D-04	-0.255939D-03
31	0.637201D-04	-0.997464D-04	0.648613D-05	-0.664365D-04	0.440876D-04
32	0.481581D-04	-0.292744D-04	-0.102169D-04	-0.203897D-03	0.217312D-03
33	-0.456479D-05	0.398244D-04	-0.561285D-05	0.823996D-04	-0.102818D-03
34	0.807850D-05	-0.176960D-04	-0.452826D-06	-0.301011D-05	0.615918D-04
35	0.251272D-05	0.126830D-06	-0.140592D-05	-0.139430D-05	-0.540305D-05
36	-0.707124D-05	0.223838D-04	-0.129299D-04	0.228890D-04	0.452415D-05

ESTIMATED COVARIANCE MATRIX FOR PARAMETER ESTIMATES

	26	27	28	29	30
26	0.428185D-03				
27	0.337196D-03	0.216271D-02			
28	0.196447D-05	-0.129664D-04	0.108523D-03		
29	-0.583761D-04	-0.856570D-04	0.521453D-04	0.472069D-03	
30	0.342187D-04	0.575469D-04	-0.288190D-04	-0.134479D-03	0.979189D-03
31	-0.102690D-03	-0.765404D-04	0.163943D-04	0.114165D-03	-0.105928D-03
32	-0.167034D-03	-0.440713D-03	0.115537D-04	0.179261D-03	-0.210380D-03
33	0.695002D-04	0.677726D-04	-0.130264D-04	-0.287403D-03	0.244542D-03
34	0.141974D-04	-0.242196D-04	-0.741925D-06	0.719928D-05	0.822537D-05
35	-0.657093D-05	-0.409331D-05	0.286135D-05	-0.214650D-05	-0.918504D-05
36	-0.119793D-04	-0.113257D-04	-0.825144D-05	-0.675121D-05	0.248723D-04

ESTIMATED COVARIANCE MATRIX FOR PARAMETER ESTIMATES

	31	32	33	34	35
31	0.368039D-03				
32	0.165789D-03	0.925398D-03			
33	-0.242401D-03	-0.367388D-03	0.142327D-02		
34	0.239661D-04	0.237615D-06	-0.438657D-04	0.156014D-02	
35	-0.197356D-05	0.539932D-05	-0.242321D-05	-0.236137D-04	0.871315D-04

36	-0.408571D-05	-0.328592D-04	-0.238602D-04	-0.189513D-03	-0.163444D-04

ESTIMATED COVARIANCE MATRIX FOR PARAMETER ESTIMATES

	36
36	0.173347D-02

ESTIMATED CORRELATION MATRIX FOR PARAMETER ESTIMATES

	1	2	3	4	5
1	1.000				
2	0.233	1.000			
3	-0.053	-0.135	1.000		
4	0.038	0.249	-0.168	1.000	
5	0.054	0.230	-0.150	0.238	1.000
6	-0.079	-0.267	0.180	-0.175	-0.223
7	-0.007	-0.017	0.014	0.000	-0.018
8	0.007	0.045	0.010	0.013	0.009
9	-0.028	0.000	0.025	-0.045	0.004
10	0.012	-0.025	0.019	0.008	-0.010
11	-0.025	-0.042	-0.020	-0.042	0.038
12	-0.018	-0.005	0.005	0.013	-0.029
13	-0.032	-0.033	0.018	-0.005	-0.047
14	0.029	0.012	-0.027	0.013	-0.031
15	-0.001	0.013	-0.067	0.006	-0.009
16	0.010	0.024	0.028	-0.026	-0.022
17	0.000	-0.029	0.011	-0.039	-0.005
18	-0.010	-0.033	0.039	0.020	-0.027
19	0.001	-0.012	-0.021	0.055	0.026
20	-0.026	0.014	-0.038	0.020	-0.001
21	-0.026	0.017	0.015	0.006	0.024
22	0.032	0.004	-0.034	0.002	0.035
23	0.008	0.020	-0.055	0.047	0.065
24	0.020	0.041	-0.062	-0.019	0.002
25	-0.018	-0.006	-0.004	-0.018	-0.033
26	-0.010	0.044	-0.021	0.021	0.013
27	-0.016	0.017	-0.023	-0.012	0.023
28	0.035	-0.015	-0.011	-0.010	-0.007
29	0.019	0.039	0.042	0.001	0.025
30	-0.006	0.051	-0.003	0.033	-0.008
31	0.001	0.014	0.060	-0.002	-0.006
32	-0.042	-0.028	0.025	0.033	-0.017

33	0.041	0.011	-0.071	0.015	0.005
34	0.022	-0.009	0.004	0.003	0.013
35	-0.034	-0.023	0.001	-0.010	-0.050
36	-0.010	0.006	-0.003	-0.010	0.001

ESTIMATED CORRELATION MATRIX FOR PARAMETER ESTIMATES

	6	7	8	9	10
6	1.000				
7	0.013	1.000			
8	-0.025	-0.158	1.000		
9	-0.033	-0.006	0.017	1.000	
10	0.021	-0.017	-0.134	0.128	1.000
11	0.011	0.028	-0.189	0.049	-0.176
12	-0.007	0.014	0.237	-0.119	0.119
13	0.033	0.038	0.054	0.017	0.019
14	-0.002	0.042	-0.005	-0.032	0.000
15	-0.024	0.042	0.026	0.000	-0.023
16	-0.010	0.019	-0.030	-0.005	0.046
17	-0.003	-0.029	0.004	0.016	-0.004
18	0.028	0.026	-0.065	-0.010	0.017
19	0.016	0.043	-0.040	-0.030	0.019
20	0.005	0.042	-0.008	-0.016	-0.011
21	-0.052	-0.046	0.032	0.008	0.014
22	-0.002	0.004	0.003	0.021	0.025
23	-0.030	0.022	0.007	0.004	-0.045
24	-0.012	-0.005	-0.048	0.003	-0.018
25	0.049	0.005	-0.004	0.027	0.004
26	-0.001	0.002	-0.013	-0.023	0.043
27	0.027	-0.027	-0.010	-0.008	-0.003
28	0.001	-0.023	0.007	-0.012	-0.015
29	-0.005	0.014	-0.004	-0.007	-0.006
30	-0.019	-0.010	0.035	0.003	0.013
31	-0.002	-0.042	0.010	0.004	0.003
32	0.024	0.000	0.007	-0.007	0.014
33	0.008	0.006	0.017	-0.005	0.010
34	-0.004	-0.024	0.000	0.027	0.015
35	0.022	0.032	0.022	-0.008	0.004
36	0.016	-0.183	-0.035	0.036	0.006

ESTIMATED CORRELATION MATRIX FOR PARAMETER ESTIMATES

	11	12	13	14	15

11	1.000				
12	0.141	1.000			
13	0.025	-0.014	1.000		
14	-0.009	-0.030	0.288	1.000	
15	0.013	0.038	0.026	0.346	1.000
16	0.017	0.025	-0.065	-0.137	-0.061
17	0.001	-0.028	0.029	-0.092	-0.222
18	-0.002	-0.036	0.026	0.017	0.027
19	-0.011	-0.022	0.073	0.259	0.097
20	0.023	0.038	0.051	0.095	0.274
21	-0.023	-0.008	0.008	-0.015	-0.057
22	0.019	-0.003	0.026	0.025	0.039
23	-0.027	-0.031	0.058	0.241	0.078
24	0.037	-0.009	-0.021	0.090	0.331
25	-0.024	-0.015	-0.026	-0.032	-0.055
26	0.040	0.042	0.015	0.000	0.050
27	-0.004	0.005	-0.012	0.011	0.017
28	0.015	-0.024	-0.037	-0.258	-0.100
29	0.019	0.000	-0.022	-0.089	-0.354
30	-0.014	-0.001	0.001	0.022	0.059
31	-0.002	-0.009	-0.004	-0.024	-0.071
32	-0.028	0.023	-0.012	0.016	-0.053
33	-0.024	0.008	0.006	0.018	0.063
34	-0.041	-0.004	-0.022	-0.014	-0.035
35	-0.043	0.026	0.010	-0.030	0.006
36	-0.019	-0.060	0.026	-0.011	-0.025

ESTIMATED CORRELATION MATRIX FOR PARAMETER ESTIMATES

	16	17	18	19	20
16	1.000				
17	0.220	1.000			
18	-0.105	-0.219	1.000		
19	-0.155	-0.059	0.026	1.000	
20	-0.080	-0.223	0.065	0.274	1.000
21	0.055	0.230	-0.280	-0.069	-0.183
22	0.009	-0.040	0.051	0.065	0.329
23	-0.164	-0.046	0.037	0.267	0.067
24	-0.034	-0.197	0.051	0.072	0.260
25	0.089	0.290	-0.234	-0.034	-0.087
26	0.025	-0.092	0.032	0.042	0.305
27	-0.018	-0.079	0.065	0.050	0.065

28	0.165	0.060	-0.064	-0.212	-0.072
29	0.063	0.186	-0.040	-0.043	-0.264
30	-0.061	-0.323	0.223	0.021	0.096
31	0.010	0.066	-0.070	-0.053	-0.303
32	0.038	0.108	-0.077	-0.011	-0.086
33	-0.032	-0.044	0.020	0.030	0.138
34	-0.029	0.014	-0.018	0.017	-0.028
35	-0.009	0.024	-0.010	0.029	0.010
36	0.033	0.014	-0.033	-0.025	-0.014

ESTIMATED CORRELATION MATRIX FOR PARAMETER ESTIMATES

	21	22	23	24	25
21	1.000				
22	-0.271	1.000			
23	-0.021	0.018	1.000		
24	-0.087	0.071	0.213	1.000	
25	0.236	-0.035	-0.083	-0.200	1.000
26	-0.172	0.312	0.038	0.278	-0.227
27	-0.058	0.045	0.058	0.350	-0.205
28	-0.029	-0.045	-0.269	-0.078	-0.014
29	0.024	-0.073	-0.065	-0.265	0.060
30	-0.216	0.046	0.011	0.071	-0.240
31	0.161	-0.291	0.029	-0.144	0.067
32	0.077	-0.054	-0.028	-0.279	0.209
33	-0.006	0.059	-0.013	0.091	-0.080
34	0.010	-0.025	-0.001	-0.003	0.046
35	0.013	0.001	-0.013	-0.006	-0.017
36	-0.008	0.030	-0.026	0.023	0.003

ESTIMATED CORRELATION MATRIX FOR PARAMETER ESTIMATES

	26	27	28	29	30
26	1.000				
27	0.350	1.000			
28	0.009	-0.027	1.000		
29	-0.130	-0.085	0.230	1.000	
30	0.053	0.040	-0.088	-0.198	1.000
31	-0.259	-0.086	0.082	0.274	-0.176
32	-0.265	-0.312	0.036	0.271	-0.221
33	0.089	0.039	-0.033	-0.351	0.207
34	0.017	-0.013	-0.002	0.008	0.007

35	-0.034	-0.009	0.029	-0.011	-0.031
36	-0.014	-0.006	-0.019	-0.007	0.019

ESTIMATED CORRELATION MATRIX FOR PARAMETER ESTIMATES

	31	32	33	34	35
31	1.000				
32	0.284	1.000			
33	-0.335	-0.320	1.000		
34	0.032	0.000	-0.029	1.000	
35	-0.011	0.019	-0.007	-0.064	1.000
36	-0.005	-0.026	-0.015	-0.115	-0.042

ESTIMATED CORRELATION MATRIX FOR PARAMETER ESTIMATES

	36
36	1.000

TECHNICAL 8 OUTPUT

TECHNICAL 8 OUTPUT FOR BAYES ESTIMATION

CHAIN	BSEED
1	0
2	285380
3	253358

ITERATION	POTENTIAL SCALE REDUCTION	PARAMETER WITH HIGHEST PSR
100	1.032	34
200	1.005	18
300	1.010	32
400	1.004	32
500	1.003	32
600	1.004	9
700	1.004	9
800	1.004	9
900	1.003	34
1000	1.003	34

Improvement in physical health, curvilinear effects

SUMMARY OF DATA

Number of clusters 57

Size (s)	Cluster ID with Size s
2	4 166
3	65 74 80 30
4	84 22
5	205
6	228
7	93
8	106 144 165 71 170 173 192 72 50
9	40 108
11	107 83 181 61 75 68
12	69 174 206 226 81
13	162
16	92 154
19	114
20	145 2
22	116
23	36 156 169 142
24	152
26	98
28	122 202
30	178
32	6
46	24
59	204
62	125
64	209
108	217
216	110
223	100

COVARIANCE COVERAGE OF DATA

Minimum covariance coverage value 0.100

Number of missing data patterns 4

PROPORTION OF DATA PRESENT

Covariance Coverage

	PHYCAT16	PRAFAC	PRAFACSQ	PHY15	KES15
PHYCAT16	1.000				
PRAFAC	1.000	1.000			
PRAFACSQ	1.000	1.000	1.000		
PHY15	1.000	1.000	1.000	1.000	
KES15	0.971	0.971	0.971	0.971	0.971
CHOICE15	0.963	0.963	0.963	0.963	0.963
BULLY15	0.966	0.966	0.966	0.966	0.966
PRESS15	0.966	0.966	0.966	0.966	0.966
DUTIES15	0.966	0.966	0.966	0.966	0.966

Covariance Coverage

	CHOICE15	BULLY15	PRESS15	DUTIES15
CHOICE15	0.963			
BULLY15	0.963	0.966		
PRESS15	0.963	0.966	0.966	
DUTIES15	0.963	0.966	0.966	0.966

UNIVARIATE PROPORTIONS AND COUNTS FOR CATEGORICAL VARIABLES

PHYCAT16		
Category 1	0.364	517.000
Category 2	0.636	905.000

UNIVARIATE SAMPLE STATISTICS

UNIVARIATE HIGHER-ORDER MOMENT DESCRIPTIVE STATISTICS

Variable/ Sample Size	Mean/ Variance	Skewness/ Kurtosis	Minimum/ Maximum	% with Min/Max	Percentiles 20%/60%	40%/80%	Median

```
     PRAFAC                -0.173       0.219      -2.338    1.75%      -1.489     -0.520     -0.258
              57.000        1.361      -0.662       2.338    1.75%       0.080      0.848
     PRAFACSQ               1.391       1.214       0.002    1.75%       0.143      0.381      0.669
              57.000        2.402       0.482       5.467    1.75%       1.168      2.415
     PHY15                  0.000       2.446      -0.155   85.58%      -0.155     -0.155     -0.155
            1422.000        0.154       5.391       1.845    1.13%      -0.155     -0.155
     KES15                  0.000       1.141      -0.953    8.62%      -0.620     -0.287     -0.120
            1381.000        0.613       1.071       3.047    0.36%       0.047      0.547
     CHOICE15               0.000      -0.138      -1.878   14.60%      -0.878      0.122      0.122
            1370.000        1.250      -0.758       2.122    5.77%       0.122      1.122
     BULLY15                0.000       2.479      -0.312   79.02%      -0.312     -0.312     -0.312
            1373.000        0.474       6.315       3.688    0.44%      -0.312      0.688
     PRESS15                0.000       0.183      -1.756   14.06%      -0.756     -0.756      0.244
            1373.000        1.207      -0.514       2.244    7.21%       0.244      1.244
     DUTIES15               0.000      -0.896      -2.873    3.06%      -0.873      0.127      0.127
            1373.000        0.975       0.587       1.127   27.75%       0.127      1.127


THE MODEL ESTIMATION TERMINATED NORMALLY

     USE THE FBITERATIONS OPTION TO INCREASE THE NUMBER OF ITERATIONS BY A FACTOR
     OF AT LEAST TWO TO CHECK CONVERGENCE AND THAT THE PSR VALUE DOES NOT INCREASE.



MODEL FIT INFORMATION

Number of Free Parameters                              42

Bayesian Posterior Predictive Checking using Chi-Square

          95% Confidence Interval for the Difference Between
          the Observed and the Replicated Chi-Square Values

                              -23.725           26.610

          Posterior Predictive P-Value             0.477



MODEL RESULTS

                                Posterior  One-Tailed         95% C.I.
                    Estimate       S.D.      P-Value   Lower 2.5%  Upper 2.5%  Significance
```

Within Level

PHYCAT16 ON						
PHY15	0.816	0.115	0.000	0.596	1.055	*
CHOICE15	-0.047	0.033	0.079	-0.112	0.015	
BULLY15	0.048	0.056	0.209	-0.060	0.160	
DUTIES15	0.032	0.038	0.178	-0.037	0.112	
PRESS15	-0.004	0.035	0.459	-0.073	0.065	
KES15	0.167	0.051	0.002	0.062	0.265	*
KES15 WITH						
PHY15	0.070	0.008	0.000	0.055	0.087	*
CHOICE15	-0.134	0.024	0.000	-0.182	-0.088	*
BULLY15	0.128	0.015	0.000	0.098	0.158	*
PRESS15	0.206	0.024	0.000	0.158	0.253	*
DUTIES15	-0.206	0.021	0.000	-0.250	-0.166	*
PHY15 WITH						
CHOICE15	-0.032	0.012	0.003	-0.056	-0.009	*
BULLY15	0.012	0.007	0.045	-0.002	0.026	
PRESS15	0.016	0.012	0.107	-0.008	0.037	
DUTIES15	-0.009	0.010	0.181	-0.029	0.011	
CHOICE15 WITH						
BULLY15	-0.133	0.021	0.000	-0.174	-0.092	*
PRESS15	-0.181	0.033	0.000	-0.253	-0.119	*
DUTIES15	0.161	0.029	0.000	0.107	0.219	*
BULLY15 WITH						
PRESS15	0.177	0.020	0.000	0.139	0.219	*
DUTIES15	-0.140	0.019	0.000	-0.178	-0.103	*
PRESS15 WITH						
DUTIES15	-0.242	0.030	0.000	-0.306	-0.182	*
Means						
PHY15	0.000	0.010	0.484	-0.020	0.020	
KES15	0.001	0.021	0.487	-0.040	0.041	
CHOICE15	-0.001	0.030	0.487	-0.060	0.059	
BULLY15	0.000	0.019	0.499	-0.035	0.036	
PRESS15	0.002	0.031	0.473	-0.061	0.063	
DUTIES15	-0.002	0.027	0.466	-0.053	0.053	

```
 Variances
    PHY15              0.154       0.006      0.000       0.144       0.167      *
    KES15              0.613       0.023      0.000       0.571       0.660      *
    CHOICE15           1.251       0.048      0.000       1.162       1.356      *
    BULLY15            0.475       0.018      0.000       0.441       0.513      *
    PRESS15            1.209       0.045      0.000       1.124       1.299      *
    DUTIES15           0.974       0.038      0.000       0.908       1.053      *

Between Level

 PHYCAT16   ON
    PRAFAC            -0.057       0.041      0.079      -0.139       0.018
    PRAFACSQ          -0.026       0.034      0.212      -0.097       0.040

 PRAFAC   WITH
    PRAFACSQ          -0.117       0.246      0.312      -0.603       0.343

 Means
    PRAFAC            -0.171       0.154      0.135      -0.484       0.127
    PRAFACSQ           1.381       0.215      0.000       0.959       1.796      *

 Thresholds
    PHYCAT16$1        -0.414       0.055      0.000      -0.522      -0.310      *

 Variances
    PRAFAC             1.371       0.265      0.000       0.970       2.010      *
    PRAFACSQ           2.422       0.459      0.000       1.704       3.465      *

 Residual Variances
    PHYCAT16           0.008       0.010      0.000       0.000       0.037      *

New/Additional Parameters
    MIN               -0.662      70.998      0.215      -7.948      10.444


STANDARDIZED MODEL RESULTS


STDYX Standardization

                                Posterior  One-Tailed         95% C.I.
                    Estimate       S.D.      P-Value   Lower 2.5%  Upper 2.5%  Significance
Within Level
```

PHYCAT16 ON						
PHY15	0.298	0.039	0.000	0.223	0.376	*
CHOICE15	-0.049	0.034	0.079	-0.116	0.016	
BULLY15	0.031	0.036	0.209	-0.038	0.102	
DUTIES15	0.030	0.035	0.178	-0.034	0.103	
PRESS15	-0.004	0.035	0.459	-0.074	0.067	
KES15	0.123	0.037	0.002	0.045	0.192	*
KES15 WITH						
PHY15	0.230	0.025	0.000	0.181	0.280	*
CHOICE15	-0.153	0.027	0.000	-0.205	-0.102	*
BULLY15	0.237	0.026	0.000	0.185	0.286	*
PRESS15	0.240	0.026	0.000	0.187	0.288	*
DUTIES15	-0.266	0.024	0.000	-0.315	-0.217	*
PHY15 WITH						
CHOICE15	-0.073	0.027	0.003	-0.125	-0.021	*
BULLY15	0.045	0.027	0.045	-0.007	0.096	
PRESS15	0.036	0.027	0.107	-0.017	0.087	
DUTIES15	-0.023	0.027	0.181	-0.076	0.029	
CHOICE15 WITH						
BULLY15	-0.172	0.027	0.000	-0.224	-0.119	*
PRESS15	-0.148	0.026	0.000	-0.204	-0.097	*
DUTIES15	0.146	0.025	0.000	0.097	0.196	*
BULLY15 WITH						
PRESS15	0.234	0.025	0.000	0.187	0.282	*
DUTIES15	-0.205	0.026	0.000	-0.257	-0.152	*
PRESS15 WITH						
DUTIES15	-0.223	0.026	0.000	-0.276	-0.172	*
Means						
PHY15	0.001	0.026	0.484	-0.052	0.052	
KES15	0.001	0.027	0.487	-0.051	0.053	
CHOICE15	-0.001	0.027	0.487	-0.054	0.053	
BULLY15	0.000	0.027	0.499	-0.051	0.053	
PRESS15	0.002	0.028	0.473	-0.055	0.057	
DUTIES15	-0.002	0.027	0.466	-0.053	0.053	
Variances						
PHY15	1.000	0.000	0.000	1.000	1.000	
KES15	1.000	0.000	0.000	1.000	1.000	

```
    CHOICE15           1.000       0.000       0.000       1.000       1.000
    BULLY15            1.000       0.000       0.000       1.000       1.000
    PRESS15            1.000       0.000       0.000       1.000       1.000
    DUTIES15           1.000       0.000       0.000       1.000       1.000

Between Level

 PHYCAT16   ON
    PRAFAC            -0.530       0.307       0.079      -0.930       0.249
    PRAFACSQ          -0.332       0.355       0.212      -0.820       0.525

 PRAFAC   WITH
    PRAFACSQ          -0.065       0.128       0.312      -0.309       0.176

 Means
    PRAFAC            -0.145       0.131       0.135      -0.406       0.109
    PRAFACSQ           0.896       0.158       0.000       0.583       1.193      *

 Thresholds
    PHYCAT16$1        -3.069       1.553       0.000      -7.820      -1.596      *

 Variances
    PRAFAC             1.000       0.000       0.000       1.000       1.000
    PRAFACSQ           1.000       0.000       0.000       1.000       1.000

 Residual Variances
    PHYCAT16           0.485       0.265       0.000       0.053       0.973      *


R-SQUARE

Within Level

                                Posterior  One-Tailed         95% C.I.
    Variable        Estimate       S.D.      P-Value   Lower 2.5%  Upper 2.5%

    PHYCAT16           0.134       0.027       0.000       0.085       0.188

Between Level

                                Posterior  One-Tailed         95% C.I.
    Variable        Estimate       S.D.      P-Value   Lower 2.5%  Upper 2.5%

    PHYCAT16           0.515       0.265       0.000       0.027       0.947
```

TECHNICAL 1 OUTPUT

PARAMETER SPECIFICATION FOR WITHIN

```
           TAU
              PHYCAT16
              ________
                    0


           NU
              PHYCAT16      PHY15         KES15         CHOICE15      BULLY15
              ________      ________      ________      ________      ________
                    0             0             0             0             0


           NU
              PRESS15       DUTIES15
              ________      ________
                    0             0


           LAMBDA
              PHYCAT16      PHY15         KES15         CHOICE15      BULLY15
              ________      ________      ________      ________      ________
 PHYCAT16           0             0             0             0             0
 PHY15              0             0             0             0             0
 KES15              0             0             0             0             0
 CHOICE15           0             0             0             0             0
 BULLY15            0             0             0             0             0
 PRESS15            0             0             0             0             0
 DUTIES15           0             0             0             0             0


           LAMBDA
              PRESS15       DUTIES15
              ________      ________
 PHYCAT16           0             0
 PHY15              0             0
 KES15              0             0
```

CHOICE15	0	0
BULLY15	0	0
PRESS15	0	0
DUTIES15	0	0

THETA

	PHYCAT16	PHY15	KES15	CHOICE15	BULLY15
PHYCAT16	0				
PHY15	0	0			
KES15	0	0	0		
CHOICE15	0	0	0	0	
BULLY15	0	0	0	0	0
PRESS15	0	0	0	0	0
DUTIES15	0	0	0	0	0

THETA

	PRESS15	DUTIES15
PRESS15	0	
DUTIES15	0	0

ALPHA

PHYCAT16	PHY15	KES15	CHOICE15	BULLY15
0	1	2	3	4

ALPHA

PRESS15	DUTIES15
5	6

BETA

	PHYCAT16	PHY15	KES15	CHOICE15	BULLY15
PHYCAT16	0	7	8	9	10
PHY15	0	0	0	0	0
KES15	0	0	0	0	0
CHOICE15	0	0	0	0	0

BULLY15	0	0	0	0	0
PRESS15	0	0	0	0	0
DUTIES15	0	0	0	0	0

BETA

	PRESS15	DUTIES15
PHYCAT16	11	12
PHY15	0	0
KES15	0	0
CHOICE15	0	0
BULLY15	0	0
PRESS15	0	0
DUTIES15	0	0

PSI

	PHYCAT16	PHY15	KES15	CHOICE15	BULLY15
PHYCAT16	0				
PHY15	0	13			
KES15	0	14	15		
CHOICE15	0	16	17	18	
BULLY15	0	19	20	21	22
PRESS15	0	23	24	25	26
DUTIES15	0	28	29	30	31

PSI

	PRESS15	DUTIES15
PRESS15	27	
DUTIES15	32	33

PARAMETER SPECIFICATION FOR BETWEEN

TAU

PHYCAT16
42

NU

PHYCAT16	PRAFAC	PRAFACSQ
0	0	0

LAMBDA

	PHYCAT16	PRAFAC	PRAFACSQ
PHYCAT16	0	0	0
PRAFAC	0	0	0
PRAFACSQ	0	0	0

THETA

	PHYCAT16	PRAFAC	PRAFACSQ
PHYCAT16	0		
PRAFAC	0	0	
PRAFACSQ	0	0	0

ALPHA

PHYCAT16	PRAFAC	PRAFACSQ
0	34	35

BETA

	PHYCAT16	PRAFAC	PRAFACSQ
PHYCAT16	0	36	37
PRAFAC	0	0	0
PRAFACSQ	0	0	0

PSI

	PHYCAT16	PRAFAC	PRAFACSQ
PHYCAT16	38		
PRAFAC	0	39	
PRAFACSQ	0	40	41

PARAMETER SPECIFICATION FOR THE ADDITIONAL PARAMETERS

NEW/ADDITIONAL PARAMETERS

MIN
43

STARTING VALUES FOR WITHIN

TAU

PHYCAT16
0.000

NU

PHYCAT16	PHY15	KES15	CHOICE15	BULLY15
0.000	0.000	0.000	0.000	0.000

NU

PRESS15	DUTIES15
0.000	0.000

LAMBDA

	PHYCAT16	PHY15	KES15	CHOICE15	BULLY15
PHYCAT16	1.000	0.000	0.000	0.000	0.000
PHY15	0.000	1.000	0.000	0.000	0.000
KES15	0.000	0.000	1.000	0.000	0.000
CHOICE15	0.000	0.000	0.000	1.000	0.000
BULLY15	0.000	0.000	0.000	0.000	1.000
PRESS15	0.000	0.000	0.000	0.000	0.000
DUTIES15	0.000	0.000	0.000	0.000	0.000

LAMBDA

	PRESS15	DUTIES15
PHYCAT16	0.000	0.000
PHY15	0.000	0.000
KES15	0.000	0.000
CHOICE15	0.000	0.000
BULLY15	0.000	0.000
PRESS15	1.000	0.000
DUTIES15	0.000	1.000

THETA

	PHYCAT16	PHY15	KES15	CHOICE15	BULLY15
PHYCAT16	0.000				
PHY15	0.000	0.000			
KES15	0.000	0.000	0.000		
CHOICE15	0.000	0.000	0.000	0.000	
BULLY15	0.000	0.000	0.000	0.000	0.000
PRESS15	0.000	0.000	0.000	0.000	0.000
DUTIES15	0.000	0.000	0.000	0.000	0.000

THETA

	PRESS15	DUTIES15
PRESS15	0.000	
DUTIES15	0.000	0.000

ALPHA

PHYCAT16	PHY15	KES15	CHOICE15	BULLY15
0.000	0.000	0.000	0.000	0.000

ALPHA

PRESS15	DUTIES15
0.000	0.000

BETA

PHYCAT16	PHY15	KES15	CHOICE15	BULLY15

PHYCAT16	0.000	0.000	0.000	0.000	0.000
PHY15	0.000	0.000	0.000	0.000	0.000
KES15	0.000	0.000	0.000	0.000	0.000
CHOICE15	0.000	0.000	0.000	0.000	0.000
BULLY15	0.000	0.000	0.000	0.000	0.000
PRESS15	0.000	0.000	0.000	0.000	0.000
DUTIES15	0.000	0.000	0.000	0.000	0.000

BETA

	PRESS15	DUTIES15
PHYCAT16	0.000	0.000
PHY15	0.000	0.000
KES15	0.000	0.000
CHOICE15	0.000	0.000
BULLY15	0.000	0.000
PRESS15	0.000	0.000
DUTIES15	0.000	0.000

PSI

	PHYCAT16	PHY15	KES15	CHOICE15	BULLY15
PHYCAT16	1.000				
PHY15	0.000	0.077			
KES15	0.000	0.000	0.306		
CHOICE15	0.000	0.000	0.000	0.625	
BULLY15	0.000	0.000	0.000	0.000	0.237
PRESS15	0.000	0.000	0.000	0.000	0.000
DUTIES15	0.000	0.000	0.000	0.000	0.000

PSI

	PRESS15	DUTIES15
PRESS15	0.604	
DUTIES15	0.000	0.488

STARTING VALUES FOR BETWEEN

```
           TAU
              PHYCAT16
              ________
               -0.311


           NU
              PHYCAT16      PRAFAC        PRAFACSQ
              ________      ________      ________
                0.000         0.000         0.000


           LAMBDA
              PHYCAT16      PRAFAC        PRAFACSQ
              ________      ________      ________
 PHYCAT16       1.000         0.000         0.000
 PRAFAC         0.000         1.000         0.000
 PRAFACSQ       0.000         0.000         1.000


           THETA
              PHYCAT16      PRAFAC        PRAFACSQ
              ________      ________      ________
 PHYCAT16       0.000
 PRAFAC         0.000         0.000
 PRAFACSQ       0.000         0.000         0.000


           ALPHA
              PHYCAT16      PRAFAC        PRAFACSQ
              ________      ________      ________
                0.000        -0.078         0.966


           BETA
              PHYCAT16      PRAFAC        PRAFACSQ
              ________      ________      ________
 PHYCAT16       0.000         0.000         0.000
 PRAFAC         0.000         0.000         0.000
 PRAFACSQ       0.000         0.000         0.000


           PSI
              PHYCAT16      PRAFAC        PRAFACSQ
```

PHYCAT16	1.000		
PRAFAC	0.000	0.480	
PRAFACSQ	0.000	0.000	0.712

STARTING VALUES FOR THE ADDITIONAL PARAMETERS

NEW/ADDITIONAL PARAMETERS

MIN
0.500

PRIORS FOR ALL PARAMETERS	PRIOR MEAN	PRIOR VARIANCE	PRIOR STD. DEV.
Parameter 1~N(0.000,infinity)	0.0000	infinity	infinity
Parameter 2~N(0.000,infinity)	0.0000	infinity	infinity
Parameter 3~N(0.000,infinity)	0.0000	infinity	infinity
Parameter 4~N(0.000,infinity)	0.0000	infinity	infinity
Parameter 5~N(0.000,infinity)	0.0000	infinity	infinity
Parameter 6~N(0.000,infinity)	0.0000	infinity	infinity
Parameter 7~N(0.000,5.000)	0.0000	5.0000	2.2361
Parameter 8~N(0.000,5.000)	0.0000	5.0000	2.2361
Parameter 9~N(0.000,5.000)	0.0000	5.0000	2.2361
Parameter 10~N(0.000,5.000)	0.0000	5.0000	2.2361
Parameter 11~N(0.000,5.000)	0.0000	5.0000	2.2361
Parameter 12~N(0.000,5.000)	0.0000	5.0000	2.2361
Parameter 13~IW(1.000,7)	infinity	infinity	infinity
Parameter 14~IW(0.000,7)	infinity	infinity	infinity
Parameter 15~IW(1.000,7)	infinity	infinity	infinity
Parameter 16~IW(0.000,7)	infinity	infinity	infinity
Parameter 17~IW(0.000,7)	infinity	infinity	infinity
Parameter 18~IW(1.000,7)	infinity	infinity	infinity
Parameter 19~IW(0.000,7)	infinity	infinity	infinity
Parameter 20~IW(0.000,7)	infinity	infinity	infinity
Parameter 21~IW(0.000,7)	infinity	infinity	infinity
Parameter 22~IW(1.000,7)	infinity	infinity	infinity
Parameter 23~IW(0.000,7)	infinity	infinity	infinity
Parameter 24~IW(0.000,7)	infinity	infinity	infinity
Parameter 25~IW(0.000,7)	infinity	infinity	infinity
Parameter 26~IW(0.000,7)	infinity	infinity	infinity

Parameter 27~IW(1.000,7)	infinity	infinity	infinity
Parameter 28~IW(0.000,7)	infinity	infinity	infinity
Parameter 29~IW(0.000,7)	infinity	infinity	infinity
Parameter 30~IW(0.000,7)	infinity	infinity	infinity
Parameter 31~IW(0.000,7)	infinity	infinity	infinity
Parameter 32~IW(0.000,7)	infinity	infinity	infinity
Parameter 33~IW(1.000,7)	infinity	infinity	infinity
Parameter 34~N(0.000,infinity)	0.0000	infinity	infinity
Parameter 35~N(0.000,infinity)	0.0000	infinity	infinity
Parameter 36~N(0.000,5.000)	0.0000	5.0000	2.2361
Parameter 37~N(0.000,5.000)	0.0000	5.0000	2.2361
Parameter 38~IG(-1.000,0.000)	infinity	infinity	infinity
Parameter 39~IW(1.000,3)	infinity	infinity	infinity
Parameter 40~IW(0.000,3)	infinity	infinity	infinity
Parameter 41~IW(1.000,3)	infinity	infinity	infinity
Parameter 42~N(0.000,5.000)	0.0000	5.0000	2.2361

TECHNICAL 3 OUTPUT

ESTIMATED COVARIANCE MATRIX FOR PARAMETER ESTIMATES

	1	2	3	4	5
1	0.105262D-03				
2	0.522127D-04	0.442652D-03			
3	-0.262475D-04	-0.110228D-03	0.929534D-03		
4	0.322823D-05	0.104687D-03	-0.755418D-04	0.348422D-03	
5	0.640124D-06	0.156524D-03	-0.148971D-03	0.121329D-03	0.967279D-03
6	-0.227530D-04	-0.163709D-03	0.101532D-03	-0.989934D-04	-0.194195D-03
7	-0.490918D-04	-0.973527D-05	0.884995D-04	-0.104626D-03	0.439479D-04
8	0.859421D-05	-0.138274D-04	-0.382372D-04	-0.245383D-04	0.296637D-04
9	0.135194D-06	-0.854486D-05	-0.887813D-05	0.204269D-06	0.128290D-04
10	0.138540D-04	-0.296649D-04	0.141500D-04	-0.112860D-04	-0.531161D-04
11	-0.779519D-05	-0.243219D-04	0.120039D-04	-0.815190D-05	-0.701049D-05
12	0.462752D-05	-0.142145D-04	0.456973D-04	-0.715663D-05	-0.115888D-04
13	-0.667504D-06	-0.223412D-05	0.697095D-06	-0.102970D-05	-0.391293D-05
14	-0.117507D-06	0.675636D-05	-0.115327D-04	0.535175D-05	-0.837094D-05
15	0.384881D-06	0.753893D-05	-0.385093D-05	-0.169319D-04	-0.620221D-04
16	-0.633354D-06	-0.519816D-05	0.146997D-05	-0.363880D-05	-0.864681D-05
17	-0.166955D-05	0.956467D-05	0.335747D-04	-0.676937D-05	0.119277D-04
18	0.125506D-04	0.306154D-05	0.132752D-04	0.371142D-04	0.489681D-04
19	-0.133714D-05	0.742430D-05	-0.120022D-05	0.889759D-05	0.978154D-05
20	-0.413106D-05	0.951202D-06	-0.215312D-04	0.535561D-06	-0.216484D-04

21	0.223564D-05	0.139060D-07	0.826218D-05	-0.405151D-05	-0.206685D-04
22	-0.118007D-05	-0.236656D-05	0.111854D-05	-0.141526D-05	-0.652837D-05
23	0.383133D-07	0.766699D-05	0.186037D-05	0.372044D-05	0.900441D-05
24	-0.192837D-06	-0.123358D-05	0.154070D-04	-0.646991D-05	-0.439432D-04
25	0.402872D-05	0.221330D-04	0.255196D-04	0.103763D-04	0.319510D-04
26	0.226067D-05	0.196994D-05	-0.217992D-04	0.140030D-04	-0.336326D-04
27	0.239637D-05	-0.139072D-04	0.261992D-04	0.105031D-04	-0.672421D-04
28	-0.829001D-06	0.302567D-05	-0.550873D-05	-0.187012D-05	0.163324D-05
29	-0.251610D-05	-0.626315D-05	-0.111689D-04	0.133238D-04	0.275830D-04
30	-0.407433D-05	-0.139534D-05	-0.108130D-04	0.649515D-05	0.102930D-04
31	0.385205D-05	0.485795D-05	0.637060D-05	0.267546D-05	0.150311D-04
32	0.352540D-05	-0.220135D-05	-0.170481D-04	-0.129303D-04	0.251340D-04
33	0.863966D-05	-0.117417D-04	0.912647D-05	-0.170925D-04	-0.238293D-04
34	-0.344374D-04	-0.194994D-04	0.517737D-04	0.629082D-04	0.810527D-04
35	0.210400D-04	0.203951D-04	-0.961574D-05	-0.638932D-04	0.652032D-04
36	-0.525639D-05	-0.191630D-04	0.672878D-05	-0.231895D-05	-0.830903D-05
37	0.740010D-06	0.413424D-05	-0.281631D-04	-0.262664D-04	-0.323699D-05
38	-0.289931D-05	-0.363186D-05	0.717027D-05	0.291958D-05	0.531742D-05
39	-0.862511D-04	-0.171056D-03	0.278182D-03	-0.305620D-04	-0.378235D-03
40	-0.628448D-04	-0.713248D-04	-0.214779D-03	-0.179899D-03	0.913793D-04
41	-0.214084D-03	-0.938042D-05	-0.245121D-05	0.138226D-04	0.834849D-04
42	0.551824D-05	0.190153D-04	-0.703044D-05	-0.272267D-04	-0.410028D-04
43	0.294023D-02	0.717957D-01	-0.583261D-01	0.601407D-01	0.968142D-01

ESTIMATED COVARIANCE MATRIX FOR PARAMETER ESTIMATES

	6	7	8	9	10
6	0.732628D-03				
7	-0.444287D-04	0.132572D-01			
8	0.320302D-04	-0.605809D-03	0.264297D-02		
9	0.214264D-05	0.122059D-03	0.109196D-03	0.105853D-02	
10	0.294751D-04	-0.422755D-04	-0.302436D-03	0.184758D-03	0.312250D-02
11	-0.867660D-05	0.368489D-04	-0.222818D-03	0.917346D-04	-0.288167D-03
12	0.430072D-04	0.116006D-05	0.383510D-03	-0.121411D-03	0.315902D-03
13	0.130934D-05	0.285612D-04	-0.371362D-05	0.458175D-05	-0.499317D-05
14	0.854642D-05	-0.727850D-05	0.953078D-05	-0.275740D-05	-0.265266D-04
15	0.226859D-04	0.956932D-04	0.522997D-04	-0.357280D-04	-0.320284D-04
16	-0.397254D-05	-0.444972D-04	0.552362D-05	-0.839495D-06	0.190176D-04
17	0.166233D-04	0.702265D-04	-0.465627D-04	0.807128D-05	0.625771D-04
18	0.854729D-05	-0.118284D-03	0.311994D-04	0.539614D-04	-0.505820D-04
19	-0.104775D-05	0.208878D-04	0.459754D-05	-0.453460D-06	0.603275D-05
20	0.108382D-04	-0.203746D-04	-0.271651D-05	0.598415D-06	0.344102D-04
21	0.135333D-04	0.169126D-04	-0.593271D-04	0.275889D-04	-0.264729D-04

22	0.645105D-05	0.221477D-04	0.302292D-04	-0.110891D-04	-0.327374D-04
23	0.238405D-05	0.225683D-04	0.230722D-04	0.626436D-05	0.981164D-05
24	0.177662D-04	0.317820D-04	0.153990D-04	-0.203844D-04	-0.679677D-05
25	-0.555246D-05	0.101753D-03	0.897401D-05	0.641177D-04	-0.594692D-04
26	-0.423474D-05	-0.614720D-04	0.339108D-04	-0.158758D-04	-0.246722D-04
27	0.275040D-04	-0.405723D-04	0.398076D-04	-0.290928D-04	-0.343388D-04
28	-0.150331D-04	0.254297D-04	-0.162120D-04	-0.499201D-05	-0.135913D-05
29	-0.960245D-05	0.675810D-04	-0.282552D-04	0.172237D-04	-0.303213D-04
30	0.157947D-04	-0.114424D-03	0.104385D-03	0.362213D-06	0.442515D-04
31	-0.144650D-04	0.740736D-04	-0.219142D-04	0.439585D-05	-0.192138D-04
32	-0.112096D-04	0.179922D-03	-0.249562D-04	0.336589D-04	-0.308274D-04
33	0.112192D-04	0.334361D-04	0.526504D-04	-0.834881D-05	0.153794D-04
34	0.805738D-04	-0.269871D-03	-0.634584D-04	-0.125473D-03	-0.722040D-05
35	0.119972D-03	-0.175519D-03	-0.156107D-03	-0.160722D-03	0.484683D-03
36	0.198776D-05	-0.403206D-03	0.409862D-04	0.307068D-04	0.313669D-04
37	0.320744D-04	-0.227082D-03	-0.981401D-04	-0.743354D-04	0.916287D-04
38	0.472781D-05	0.277613D-04	0.276641D-05	-0.166466D-04	-0.217868D-05
39	0.177932D-03	0.123094D-02	-0.138852D-03	0.133212D-03	-0.294335D-03
40	-0.291721D-04	0.271861D-03	-0.259484D-03	0.315696D-04	-0.452747D-04
41	0.229056D-03	0.822146D-03	-0.300306D-03	-0.229962D-03	0.842276D-03
42	0.254930D-04	-0.119591D-02	-0.149271D-03	0.126470D-04	-0.127766D-04
43	-0.570390D-01	0.283139D+00	-0.194303D+00	0.621413D-01	-0.298863D+00

ESTIMATED COVARIANCE MATRIX FOR PARAMETER ESTIMATES

	11	12	13	14	15
11	0.119590D-02				
12	0.202560D-03	0.144292D-02			
13	0.964893D-05	0.455840D-05	0.334100D-04		
14	0.210486D-05	-0.494794D-05	0.127734D-04	0.699862D-04	
15	0.119098D-04	0.120437D-04	0.233498D-05	0.637445D-04	0.513316D-03
16	-0.621677D-05	0.156437D-04	-0.737967D-05	-0.171872D-04	-0.242555D-04
17	-0.194228D-04	0.221925D-04	0.162239D-05	-0.161072D-04	-0.104909D-03
18	0.310179D-04	0.162651D-04	-0.134233D-05	-0.132380D-05	0.184283D-04
19	-0.624667D-05	-0.742109D-05	0.291000D-05	0.157607D-04	0.220947D-04
20	0.254306D-04	-0.237772D-05	0.507317D-06	0.751665D-05	0.103436D-03
21	0.196570D-04	-0.462991D-04	0.337742D-05	-0.257812D-05	-0.256511D-04
22	0.131500D-04	0.212791D-04	-0.360537D-08	0.735575D-06	0.267525D-04
23	-0.202963D-04	-0.671917D-05	0.267124D-05	0.214798D-04	0.283837D-04
24	0.658968D-05	-0.216388D-04	0.136583D-05	0.141323D-04	0.175229D-03
25	-0.107314D-04	0.155575D-04	0.266847D-05	0.181100D-05	-0.392655D-04
26	0.853077D-05	-0.477497D-05	0.404063D-05	-0.402832D-06	0.341653D-04
27	0.362424D-04	0.274588D-04	0.110910D-04	-0.180478D-05	0.255629D-05

28	0.143608D-04	0.876419D-05	0.121569D-05	-0.251596D-04	-0.206330D-04
29	-0.175268D-04	0.406607D-05	0.331040D-05	-0.138406D-04	-0.167869D-03
30	-0.511947D-04	0.151026D-04	-0.277942D-05	0.616093D-05	0.425359D-04
31	-0.514778D-05	-0.149977D-04	-0.485293D-06	0.783979D-05	-0.219704D-04
32	0.197408D-04	-0.139540D-04	-0.629161D-05	-0.412067D-05	-0.508528D-04
33	-0.256560D-04	0.245230D-04	0.254776D-05	-0.181110D-05	0.172081D-04
34	-0.859385D-04	-0.603979D-06	-0.233184D-04	-0.309996D-04	-0.233123D-04
35	-0.126200D-03	0.292763D-03	-0.295916D-05	-0.610060D-04	-0.763857D-04
36	-0.262776D-04	-0.127989D-04	-0.112636D-05	-0.721001D-05	-0.562851D-04
37	0.298667D-04	0.396565D-04	-0.206175D-05	-0.119121D-04	-0.462379D-04
38	0.161376D-04	0.154470D-04	-0.145077D-05	-0.936887D-06	-0.654141D-05
39	0.407249D-04	0.124749D-03	0.302646D-04	-0.323428D-04	0.150750D-03
40	0.138857D-03	0.106657D-03	-0.830219D-05	0.308857D-04	-0.206480D-03
41	0.303748D-03	-0.599030D-03	-0.252882D-05	-0.527293D-04	0.106154D-03
42	0.480571D-04	0.184012D-04	-0.754720D-05	-0.623741D-05	-0.430715D-04
43	0.872602D-01	-0.738920D-01	0.522767D-03	-0.131680D-01	-0.122957D-01

ESTIMATED COVARIANCE MATRIX FOR PARAMETER ESTIMATES

	16	17	18	19	20
16	0.141549D-03				
17	0.782496D-04	0.576815D-03			
18	-0.662721D-04	-0.227251D-03	0.233909D-02		
19	-0.161756D-04	-0.115775D-04	0.905487D-05	0.542283D-04	
20	-0.163508D-04	-0.733088D-04	0.142807D-04	0.276617D-04	0.226603D-03
21	0.155142D-04	0.125387D-03	-0.193874D-03	-0.123313D-04	-0.619523D-04
22	-0.327744D-05	-0.295867D-04	-0.377189D-05	0.782368D-05	0.902458D-04
23	-0.166881D-04	-0.844059D-05	0.543467D-05	0.199120D-04	0.844297D-05
24	-0.584914D-06	-0.104487D-03	0.295978D-05	0.556294D-05	0.989489D-04
25	0.261268D-04	0.206569D-03	-0.346667D-03	0.627316D-05	-0.645918D-04
26	0.534663D-05	-0.312996D-04	-0.144659D-04	0.106694D-05	0.931830D-04
27	-0.161706D-04	-0.519231D-04	0.397574D-04	0.193080D-05	0.669272D-04
28	0.145871D-04	0.139828D-04	0.138883D-04	-0.172220D-04	-0.123162D-04
29	0.154150D-04	0.922963D-04	0.475967D-05	-0.111137D-04	-0.840766D-04
30	-0.135160D-04	-0.170942D-03	0.262980D-03	0.867079D-05	0.392337D-04
31	0.101184D-04	0.398722D-04	-0.887903D-05	-0.431627D-05	-0.925012D-04
32	0.250309D-05	0.653593D-04	-0.563514D-04	0.789913D-05	-0.407000D-04
33	-0.130704D-04	-0.872650D-04	0.751154D-04	0.234807D-05	0.311122D-04
34	-0.614343D-04	0.482228D-04	0.183742D-03	0.266831D-04	-0.250395D-04
35	0.124426D-03	-0.122550D-04	-0.289739D-03	0.412236D-04	0.360398D-04
36	0.120807D-04	-0.805915D-05	0.880187D-04	-0.127169D-04	-0.987124D-05
37	0.235561D-04	0.165753D-04	0.671447D-05	-0.114941D-04	-0.299839D-05
38	0.953737D-07	0.266334D-05	0.111288D-04	-0.486563D-07	-0.568348D-06

39	-0.218901D-04	0.122064D-03	-0.438808D-03	0.835395D-06	-0.156156D-04
40	-0.269525D-05	0.796657D-04	0.117830D-03	0.147995D-03	0.119986D-04
41	-0.169398D-04	0.352616D-03	-0.119539D-02	-0.402995D-04	-0.690511D-04
42	0.302949D-04	0.998795D-05	-0.934969D-05	-0.225437D-04	-0.258167D-05
43	0.525196D-02	0.469838D-01	-0.981364D-01	0.237565D-02	0.574489D-01

ESTIMATED COVARIANCE MATRIX FOR PARAMETER ESTIMATES

	21	22	23	24	25
21	0.456509D-03				
22	-0.105285D-03	0.326145D-03			
23	0.109025D-05	0.167406D-05	0.132918D-03		
24	-0.288719D-04	0.333323D-04	0.555605D-04	0.586184D-03	
25	0.175257D-03	-0.354348D-04	-0.292597D-04	-0.124293D-03	0.108533D-02
26	-0.728166D-04	0.124649D-03	0.558246D-05	0.126722D-03	-0.126809D-03
27	-0.584912D-04	0.272598D-04	0.241416D-04	0.351945D-03	-0.280861D-03
28	-0.266728D-05	-0.862472D-05	-0.255696D-04	-0.115766D-04	-0.215823D-05
29	0.288300D-04	-0.318892D-04	-0.112182D-04	-0.142975D-03	0.502619D-04
30	-0.132969D-03	0.425911D-04	0.828489D-05	0.553707D-04	-0.245977D-03
31	0.914187D-04	-0.100627D-03	-0.130305D-05	-0.558411D-04	0.754699D-04
32	0.201264D-04	-0.387981D-04	-0.127953D-04	-0.196012D-03	0.196389D-03
33	-0.427972D-04	0.485595D-04	-0.495334D-06	0.463622D-04	-0.521577D-04
34	0.654608D-04	-0.174013D-04	-0.346378D-05	-0.312113D-04	-0.746277D-04
35	-0.170317D-04	0.107788D-03	-0.145727D-04	-0.254602D-04	0.176974D-03
36	-0.457332D-05	-0.172246D-07	-0.207837D-04	-0.576305D-04	0.880985D-05
37	0.382638D-04	-0.292919D-04	-0.175611D-04	-0.169314D-04	0.548358D-04
38	0.159689D-05	-0.116908D-06	0.278307D-05	-0.892205D-05	0.898558D-05
39	0.287434D-03	0.111902D-03	0.596541D-04	0.948969D-04	0.357626D-03
40	0.372454D-04	-0.276310D-04	-0.114571D-03	-0.109705D-03	-0.921578D-04
41	0.106445D-03	0.133153D-04	0.123643D-03	-0.256984D-04	0.267432D-03
42	0.161404D-04	-0.261856D-04	-0.257426D-04	0.178881D-04	0.890018D-04
43	0.315877D-01	-0.809478D-02	0.398826D-02	0.198428D-01	0.963824D-01

ESTIMATED COVARIANCE MATRIX FOR PARAMETER ESTIMATES

	26	27	28	29	30
26	0.418026D-03				
27	0.288599D-03	0.203282D-02			
28	-0.112685D-04	0.400700D-05	0.110113D-03		
29	-0.381384D-04	-0.786282D-04	0.554571D-04	0.448340D-03	
30	0.411041D-04	0.131323D-04	-0.328550D-04	-0.117506D-03	0.848400D-03
31	-0.952201D-04	-0.566536D-04	0.582782D-05	0.107267D-03	-0.122337D-03

32	-0.137273D-03	-0.367301D-03	0.112990D-04	0.179866D-03	-0.192975D-03
33	0.464710D-04	0.835436D-04	-0.199004D-04	-0.305548D-03	0.253361D-03
34	0.409801D-04	0.179295D-03	0.384789D-04	0.140857D-03	-0.211883D-03
35	0.159914D-04	-0.179557D-03	-0.714246D-04	-0.402349D-04	0.553381D-04
36	0.329173D-05	-0.814527D-04	0.147559D-04	0.312793D-04	-0.838326D-05
37	-0.166732D-04	-0.674979D-04	0.264941D-05	-0.114114D-04	-0.619843D-05
38	-0.134136D-04	-0.133645D-04	-0.328814D-05	0.446850D-05	-0.107813D-04
39	0.979456D-04	-0.689331D-04	-0.154282D-03	-0.677443D-04	-0.183008D-03
40	-0.123681D-03	0.498416D-03	0.275386D-04	-0.543904D-04	0.214570D-03
41	0.518791D-04	0.509172D-04	-0.323012D-04	0.432084D-04	-0.477557D-03
42	-0.220281D-04	-0.410557D-04	0.749644D-05	-0.547616D-04	0.148953D-04
43	0.254519D-01	0.398709D-01	0.216306D-01	0.190109D-01	-0.581966D-01

ESTIMATED COVARIANCE MATRIX FOR PARAMETER ESTIMATES

	31	32	33	34	35
31	0.365375D-03				
32	0.162426D-03	0.917692D-03			
33	-0.191997D-03	-0.354678D-03	0.145035D-02		
34	0.931856D-05	0.118548D-03	-0.375968D-03	0.237388D-01	
35	-0.667909D-04	-0.171839D-03	-0.160225D-03	-0.134010D-02	0.463975D-01
36	0.488045D-05	0.315098D-04	-0.542229D-04	0.263934D-03	-0.129495D-03
37	0.442778D-04	0.237208D-04	-0.369658D-04	0.872708D-04	0.413520D-04
38	0.600680D-05	0.128660D-04	-0.591866D-05	-0.427860D-04	0.753253D-04
39	-0.138923D-03	0.199131D-03	0.240752D-03	-0.671356D-03	0.149561D-02
40	-0.165498D-06	0.269220D-03	0.703555D-04	-0.100383D-02	0.975629D-03
41	-0.187115D-03	0.156798D-03	0.603225D-03	-0.140705D-04	0.387693D-03
42	0.289189D-04	0.237141D-04	-0.133251D-04	0.287153D-04	0.177680D-03
43	-0.354496D-01	-0.206450D-01	0.110146D+00	-0.131041D+00	0.274277D+00

ESTIMATED COVARIANCE MATRIX FOR PARAMETER ESTIMATES

	36	37	38	39	40
36	0.168363D-02				
37	0.466776D-03	0.118002D-02			
38	-0.621309D-04	-0.521653D-04	0.109657D-03		
39	0.262800D-03	-0.268898D-03	-0.569549D-04	0.703490D-01	
40	-0.107573D-03	0.369621D-04	-0.678478D-06	-0.633633D-02	0.604981D-01
41	0.111149D-02	0.643150D-03	0.539085D-04	-0.188615D-02	-0.755848D-02
42	0.309649D-03	0.122762D-02	-0.802302D-04	-0.537555D-03	0.662021D-04
43	0.664030D-01	0.598974D-01	0.887498D-02	-0.423959D+00	-0.305162D-02

ESTIMATED COVARIANCE MATRIX FOR PARAMETER ESTIMATES

	41	42	43
41	0.210596D+00		
42	-0.247612D-03	0.299197D-02	
43	0.976638D+00	0.126691D+00	0.503737D+04

ESTIMATED CORRELATION MATRIX FOR PARAMETER ESTIMATES

	1	2	3	4	5
1	1.000				
2	0.242	1.000			
3	-0.084	-0.172	1.000		
4	0.017	0.267	-0.133	1.000	
5	0.002	0.239	-0.157	0.209	1.000
6	-0.082	-0.287	0.123	-0.196	-0.231
7	-0.042	-0.004	0.025	-0.049	0.012
8	0.016	-0.013	-0.024	-0.026	0.019
9	0.000	-0.012	-0.009	0.000	0.013
10	0.024	-0.025	0.008	-0.011	-0.031
11	-0.022	-0.033	0.011	-0.013	-0.007
12	0.012	-0.018	0.039	-0.010	-0.010
13	-0.011	-0.018	0.004	-0.010	-0.022
14	-0.001	0.038	-0.045	0.034	-0.032
15	0.002	0.016	-0.006	-0.040	-0.088
16	-0.005	-0.021	0.004	-0.016	-0.023
17	-0.007	0.019	0.046	-0.015	0.016
18	0.025	0.003	0.009	0.041	0.033
19	-0.018	0.048	-0.005	0.065	0.043
20	-0.027	0.003	-0.047	0.002	-0.046
21	0.010	0.000	0.013	-0.010	-0.031
22	-0.006	-0.006	0.002	-0.004	-0.012
23	0.000	0.032	0.005	0.017	0.025
24	-0.001	-0.002	0.021	-0.014	-0.058
25	0.012	0.032	0.025	0.017	0.031
26	0.011	0.005	-0.035	0.037	-0.053
27	0.005	-0.015	0.019	0.012	-0.048
28	-0.008	0.014	-0.017	-0.010	0.005
29	-0.012	-0.014	-0.017	0.034	0.042
30	-0.014	-0.002	-0.012	0.012	0.011
31	0.020	0.012	0.011	0.007	0.025
32	0.011	-0.003	-0.018	-0.023	0.027

33	0.022	-0.015	0.008	-0.024	-0.020
34	-0.022	-0.006	0.011	0.022	0.017
35	0.010	0.005	-0.001	-0.016	0.010
36	-0.012	-0.022	0.005	-0.003	-0.007
37	0.002	0.006	-0.027	-0.041	-0.003
38	-0.027	-0.016	0.022	0.015	0.016
39	-0.032	-0.031	0.034	-0.006	-0.046
40	-0.025	-0.014	-0.029	-0.039	0.012
41	-0.045	-0.001	0.000	0.002	0.006
42	0.010	0.017	-0.004	-0.027	-0.024
43	0.004	0.048	-0.027	0.045	0.044

ESTIMATED CORRELATION MATRIX FOR PARAMETER ESTIMATES

	6	7	8	9	10
6	1.000				
7	-0.014	1.000			
8	0.023	-0.102	1.000		
9	0.002	0.033	0.065	1.000	
10	0.019	-0.007	-0.105	0.102	1.000
11	-0.009	0.009	-0.125	0.082	-0.149
12	0.042	0.000	0.196	-0.098	0.149
13	0.008	0.043	-0.012	0.024	-0.015
14	0.038	-0.008	0.022	-0.010	-0.057
15	0.037	0.037	0.045	-0.048	-0.025
16	-0.012	-0.032	0.009	-0.002	0.029
17	0.026	0.025	-0.038	0.010	0.047
18	0.007	-0.021	0.013	0.034	-0.019
19	-0.005	0.025	0.012	-0.002	0.015
20	0.027	-0.012	-0.004	0.001	0.041
21	0.023	0.007	-0.054	0.040	-0.022
22	0.013	0.011	0.033	-0.019	-0.032
23	0.008	0.017	0.039	0.017	0.015
24	0.027	0.011	0.012	-0.026	-0.005
25	-0.006	0.027	0.005	0.060	-0.032
26	-0.008	-0.026	0.032	-0.024	-0.022
27	0.023	-0.008	0.017	-0.020	-0.014
28	-0.053	0.021	-0.030	-0.015	-0.002
29	-0.017	0.028	-0.026	0.025	-0.026
30	0.020	-0.034	0.070	0.000	0.027
31	-0.028	0.034	-0.022	0.007	-0.018
32	-0.014	0.052	-0.016	0.034	-0.018
33	0.011	0.008	0.027	-0.007	0.007

34	0.019	-0.015	-0.008	-0.025	-0.001
35	0.021	-0.007	-0.014	-0.023	0.040
36	0.002	-0.085	0.019	0.023	0.014
37	0.034	-0.057	-0.056	-0.067	0.048
38	0.017	0.023	0.005	-0.049	-0.004
39	0.025	0.040	-0.010	0.015	-0.020
40	-0.004	0.010	-0.021	0.004	-0.003
41	0.018	0.016	-0.013	-0.015	0.033
42	0.017	-0.190	-0.053	0.007	-0.004
43	-0.030	0.035	-0.053	0.027	-0.075

ESTIMATED CORRELATION MATRIX FOR PARAMETER ESTIMATES

	11	12	13	14	15
11	1.000				
12	0.154	1.000			
13	0.048	0.021	1.000		
14	0.007	-0.016	0.264	1.000	
15	0.015	0.014	0.018	0.336	1.000
16	-0.015	0.035	-0.107	-0.173	-0.090
17	-0.023	0.024	0.012	-0.080	-0.193
18	0.019	0.009	-0.005	-0.003	0.017
19	-0.025	-0.027	0.068	0.256	0.132
20	0.049	-0.004	0.006	0.060	0.303
21	0.027	-0.057	0.027	-0.014	-0.053
22	0.021	0.031	0.000	0.005	0.065
23	-0.051	-0.015	0.040	0.223	0.109
24	0.008	-0.024	0.010	0.070	0.319
25	-0.009	0.012	0.014	0.007	-0.053
26	0.012	-0.006	0.034	-0.002	0.074
27	0.023	0.016	0.043	-0.005	0.003
28	0.040	0.022	0.020	-0.287	-0.087
29	-0.024	0.005	0.027	-0.078	-0.350
30	-0.051	0.014	-0.017	0.025	0.064
31	-0.008	-0.021	-0.004	0.049	-0.051
32	0.019	-0.012	-0.036	-0.016	-0.074
33	-0.019	0.017	0.012	-0.006	0.020
34	-0.016	0.000	-0.026	-0.024	-0.007
35	-0.017	0.036	-0.002	-0.034	-0.016
36	-0.019	-0.008	-0.005	-0.021	-0.061
37	0.025	0.030	-0.010	-0.041	-0.059
38	0.045	0.039	-0.024	-0.011	-0.028
39	0.004	0.012	0.020	-0.015	0.025

40	0.016	0.011	-0.006	0.015	-0.037
41	0.019	-0.034	-0.001	-0.014	0.010
42	0.025	0.009	-0.024	-0.014	-0.035
43	0.036	-0.027	0.001	-0.022	-0.008

ESTIMATED CORRELATION MATRIX FOR PARAMETER ESTIMATES

	16	17	18	19	20
16	1.000				
17	0.274	1.000			
18	-0.115	-0.196	1.000		
19	-0.185	-0.065	0.025	1.000	
20	-0.091	-0.203	0.020	0.250	1.000
21	0.061	0.244	-0.188	-0.078	-0.193
22	-0.015	-0.068	-0.004	0.059	0.332
23	-0.122	-0.030	0.010	0.235	0.049
24	-0.002	-0.180	0.003	0.031	0.271
25	0.067	0.261	-0.218	0.026	-0.130
26	0.022	-0.064	-0.015	0.007	0.303
27	-0.030	-0.048	0.018	0.006	0.099
28	0.117	0.055	0.027	-0.223	-0.078
29	0.061	0.181	0.005	-0.071	-0.264
30	-0.039	-0.244	0.187	0.040	0.089
31	0.044	0.087	-0.010	-0.031	-0.321
32	0.007	0.090	-0.038	0.035	-0.089
33	-0.029	-0.095	0.041	0.008	0.054
34	-0.034	0.013	0.025	0.024	-0.011
35	0.049	-0.002	-0.028	0.026	0.011
36	0.025	-0.008	0.044	-0.042	-0.016
37	0.058	0.020	0.004	-0.045	-0.006
38	0.001	0.011	0.022	-0.001	-0.004
39	-0.007	0.019	-0.034	0.000	-0.004
40	-0.001	0.013	0.010	0.082	0.003
41	-0.003	0.032	-0.054	-0.012	-0.010
42	0.047	0.008	-0.004	-0.056	-0.003
43	0.006	0.028	-0.029	0.005	0.054

ESTIMATED CORRELATION MATRIX FOR PARAMETER ESTIMATES

	21	22	23	24	25
21	1.000				
22	-0.273	1.000			

23	0.004	0.008	1.000		
24	-0.056	0.076	0.199	1.000	
25	0.249	-0.060	-0.077	-0.156	1.000
26	-0.167	0.338	0.024	0.256	-0.188
27	-0.061	0.033	0.046	0.322	-0.189
28	-0.012	-0.046	-0.211	-0.046	-0.006
29	0.064	-0.083	-0.046	-0.279	0.072
30	-0.214	0.081	0.025	0.079	-0.256
31	0.224	-0.292	-0.006	-0.121	0.120
32	0.031	-0.071	-0.037	-0.267	0.197
33	-0.053	0.071	-0.001	0.050	-0.042
34	0.020	-0.006	-0.002	-0.008	-0.015
35	-0.004	0.028	-0.006	-0.005	0.025
36	-0.005	0.000	-0.044	-0.058	0.007
37	0.052	-0.047	-0.044	-0.020	0.048
38	0.007	-0.001	0.023	-0.035	0.026
39	0.051	0.023	0.020	0.015	0.041
40	0.007	-0.006	-0.040	-0.018	-0.011
41	0.011	0.002	0.023	-0.002	0.018
42	0.014	-0.027	-0.041	0.014	0.049
43	0.021	-0.006	0.005	0.012	0.041

ESTIMATED CORRELATION MATRIX FOR PARAMETER ESTIMATES

	26	27	28	29	30
26	1.000				
27	0.313	1.000			
28	-0.053	0.008	1.000		
29	-0.088	-0.082	0.250	1.000	
30	0.069	0.010	-0.107	-0.191	1.000
31	-0.244	-0.066	0.029	0.265	-0.220
32	-0.222	-0.269	0.036	0.280	-0.219
33	0.060	0.049	-0.050	-0.379	0.228
34	0.013	0.026	0.024	0.043	-0.047
35	0.004	-0.018	-0.032	-0.009	0.009
36	0.004	-0.044	0.034	0.036	-0.007
37	-0.024	-0.044	0.007	-0.016	-0.006
38	-0.063	-0.028	-0.030	0.020	-0.035
39	0.018	-0.006	-0.055	-0.012	-0.024
40	-0.025	0.045	0.011	-0.010	0.030
41	0.006	0.002	-0.007	0.004	-0.036
42	-0.020	-0.017	0.013	-0.047	0.009
43	0.018	0.012	0.029	0.013	-0.028

ESTIMATED CORRELATION MATRIX FOR PARAMETER ESTIMATES

	31	32	33	34	35
31	1.000				
32	0.281	1.000			
33	-0.264	-0.307	1.000		
34	0.003	0.025	-0.064	1.000	
35	-0.016	-0.026	-0.020	-0.040	1.000
36	0.006	0.025	-0.035	0.042	-0.015
37	0.067	0.023	-0.028	0.016	0.006
38	0.030	0.041	-0.015	-0.027	0.033
39	-0.027	0.025	0.024	-0.016	0.026
40	0.000	0.036	0.008	-0.026	0.018
41	-0.021	0.011	0.035	0.000	0.004
42	0.028	0.014	-0.006	0.003	0.015
43	-0.026	-0.010	0.041	-0.012	0.018

ESTIMATED CORRELATION MATRIX FOR PARAMETER ESTIMATES

	36	37	38	39	40
36	1.000				
37	0.331	1.000			
38	-0.145	-0.145	1.000		
39	0.024	-0.030	-0.021	1.000	
40	-0.011	0.004	0.000	-0.097	1.000
41	0.059	0.041	0.011	-0.015	-0.067
42	0.138	0.653	-0.140	-0.037	0.005
43	0.023	0.025	0.012	-0.023	0.000

ESTIMATED CORRELATION MATRIX FOR PARAMETER ESTIMATES

	41	42	43
41	1.000		
42	-0.010	1.000	
43	0.030	0.033	1.000

TECHNICAL 8 OUTPUT

TECHNICAL 8 OUTPUT FOR BAYES ESTIMATION

CHAIN	BSEED
1	0
2	285380
3	253358

ITERATION	POTENTIAL SCALE REDUCTION	PARAMETER WITH HIGHEST PSR
100	1.022	32
200	1.009	42
300	1.005	40
400	1.006	28
500	1.004	28
600	1.007	36
700	1.002	38
800	1.001	27
900	1.002	2
1000	1.003	38

Worsening of distress, main effects

SUMMARY OF DATA

Number of clusters 58

Size (s)	Cluster ID with Size s
7	22
17	166
20	80
26	71 74 205
29	65
31	192
33	228
37	182 106
39	72
41	170 181
42	50 30
43	75
45	173
47	226 165
48	108
50	107 114
51	81
52	40
55	84 92
57	162
59	206
61	61 83 116
62	36 93 169
63	69
64	4
69	68
73	174 152
74	6 154
75	145
89	98
90	178 2
95	144
100	156
104	202
106	142
134	122

186	24
192	209
246	204
278	125
405	217
780	110
873	100

COVARIANCE COVERAGE OF DATA

Minimum covariance coverage value 0.100

Number of missing data patterns 9

PROPORTION OF DATA PRESENT

Covariance Coverage

	KESCAT16	PHY15	KES15	CHOICE15	BULLY15
KESCAT16	1.000				
PHY15	0.950	0.950			
KES15	0.919	0.919	0.919		
CHOICE15	0.913	0.913	0.913	0.913	
BULLY15	0.913	0.913	0.913	0.912	0.913
PRESS15	0.913	0.913	0.913	0.913	0.912
DUTIES15	0.913	0.913	0.913	0.913	0.913
PRAFAC	1.000	0.950	0.919	0.913	0.913

Covariance Coverage

	PRESS15	DUTIES15	PRAFAC
PRESS15	0.913		
DUTIES15	0.913	0.913	
PRAFAC	0.913	0.913	1.000

UNIVARIATE PROPORTIONS AND COUNTS FOR CATEGORICAL VARIABLES

KESCAT16

Category 1	0.877	5135.000
Category 2	0.123	720.000

UNIVARIATE SAMPLE STATISTICS

UNIVARIATE HIGHER-ORDER MOMENT DESCRIPTIVE STATISTICS

Variable/ Sample Size	Mean/ Variance	Skewness/ Kurtosis	Minimum/ Maximum	% with Min/Max	Percentiles 20%/60%	40%/80%	Median
PHY15	0.000	0.526	-0.865	32.48%	-0.865	0.135	0.135
5563.000	0.536	0.021	3.135	0.07%	0.135	0.135	
KES15	0.000	0.475	-0.374	22.78%	-0.374	-0.207	-0.041
5382.000	0.095	-0.842	0.626	6.60%	-0.041	0.293	
CHOICE15	0.000	-0.400	-2.221	7.67%	-1.221	-0.221	-0.221
5345.000	1.097	-0.356	1.779	8.62%	0.779	0.779	
BULLY15	0.000	3.522	-0.153	88.29%	-0.153	-0.153	-0.153
5344.000	0.216	14.164	3.847	0.09%	-0.153	-0.153	
PRESS15	0.000	0.267	-1.445	19.70%	-0.445	-0.445	-0.445
5345.000	1.001	-0.357	2.555	2.75%	0.555	0.555	
DUTIES15	0.000	-1.296	-3.149	2.34%	-0.149	-0.149	-0.149
5347.000	0.828	1.939	0.851	39.74%	-0.149	0.851	
PRAFAC	-0.185	0.245	-2.338	1.72%	-1.448	-0.527	-0.318
58.000	1.345	-0.636	2.338	1.72%	0.080	0.752	

THE MODEL ESTIMATION TERMINATED NORMALLY

USE THE FBITERATIONS OPTION TO INCREASE THE NUMBER OF ITERATIONS BY A FACTOR
OF AT LEAST TWO TO CHECK CONVERGENCE AND THAT THE PSR VALUE DOES NOT INCREASE.

MODEL FIT INFORMATION

Number of Free Parameters 36

Bayesian Posterior Predictive Checking using Chi-Square

95% Confidence Interval for the Difference Between
the Observed and the Replicated Chi-Square Values

```
                        -22.143           28.089

          Posterior Predictive P-Value              0.490



MODEL RESULTS

                                Posterior  One-Tailed         95% C.I.
                    Estimate       S.D.      P-Value   Lower 2.5%  Upper 2.5%  Significance

Within Level

 KESCAT16   ON
    PHY15              0.135       0.033      0.000       0.073       0.200      *
    CHOICE15          -0.058       0.025      0.009      -0.108      -0.009      *
    BULLY15            0.111       0.048      0.013       0.016       0.199      *
    DUTIES15          -0.082       0.027      0.001      -0.135      -0.027      *
    PRESS15           -0.033       0.026      0.110      -0.085       0.017
    KES15              1.613       0.085      0.000       1.444       1.780      *

 KES15    WITH
    PHY15              0.055       0.003      0.000       0.048       0.061      *
    CHOICE15          -0.037       0.004      0.000      -0.045      -0.028      *
    BULLY15            0.022       0.002      0.000       0.019       0.026      *
    PRESS15            0.053       0.004      0.000       0.045       0.062      *
    DUTIES15          -0.051       0.004      0.000      -0.058      -0.043      *

 PHY15    WITH
    CHOICE15          -0.092       0.011      0.000      -0.113      -0.071      *
    BULLY15            0.025       0.005      0.000       0.015       0.034      *
    PRESS15            0.062       0.010      0.000       0.044       0.083      *
    DUTIES15          -0.057       0.009      0.000      -0.074      -0.039      *

 CHOICE15 WITH
    BULLY15           -0.052       0.007      0.000      -0.064      -0.039      *
    PRESS15           -0.078       0.014      0.000      -0.105      -0.049      *
    DUTIES15           0.159       0.013      0.000       0.136       0.184      *

 BULLY15  WITH
    PRESS15            0.091       0.006      0.000       0.079       0.104      *
    DUTIES15          -0.059       0.006      0.000      -0.070      -0.047      *
```

```
 PRESS15  WITH
    DUTIES15          -0.156       0.013      0.000      -0.183      -0.131      *

 Means
    PHY15              0.001       0.010      0.453      -0.016       0.021
    KES15              0.003       0.004      0.264      -0.005       0.011
    CHOICE15          -0.003       0.014      0.429      -0.030       0.026
    BULLY15            0.001       0.006      0.424      -0.011       0.013
    PRESS15            0.002       0.013      0.451      -0.023       0.028
    DUTIES15          -0.003       0.012      0.400      -0.026       0.022

 Variances
    PHY15              0.536       0.010      0.000       0.517       0.557      *
    KES15              0.096       0.002      0.000       0.092       0.100      *
    CHOICE15           1.098       0.021      0.000       1.059       1.141      *
    BULLY15            0.216       0.004      0.000       0.208       0.224      *
    PRESS15            1.001       0.020      0.000       0.964       1.043      *
    DUTIES15           0.829       0.016      0.000       0.799       0.861      *

Between Level

 KESCAT16   ON
    PRAFAC             0.014       0.028      0.303      -0.038       0.071

 Thresholds
    KESCAT16$1         1.354       0.035      0.000       1.290       1.427      *

 Residual Variances
    KESCAT16           0.009       0.008      0.000       0.002       0.032      *


STANDARDIZED MODEL RESULTS


STDYX Standardization

                                Posterior  One-Tailed         95% C.I.
                    Estimate       S.D.      P-Value   Lower 2.5%  Upper 2.5%  Significance
Within Level

 KESCAT16   ON
    PHY15              0.086       0.021      0.000       0.046       0.126      *
    CHOICE15          -0.053       0.023      0.009      -0.098      -0.008      *
    BULLY15            0.045       0.019      0.013       0.006       0.081      *
```

```
   DUTIES15          -0.065       0.021      0.001      -0.106      -0.022      *
   PRESS15           -0.029       0.023      0.110      -0.074       0.015
   KES15              0.433       0.019      0.000       0.394       0.471      *

KES15    WITH
   PHY15              0.241       0.013      0.000       0.215       0.266      *
   CHOICE15          -0.113       0.013      0.000      -0.139      -0.087      *
   BULLY15            0.154       0.013      0.000       0.129       0.179      *
   PRESS15            0.172       0.013      0.000       0.146       0.198      *
   DUTIES15          -0.180       0.013      0.000      -0.205      -0.153      *

PHY15    WITH
   CHOICE15          -0.120       0.014      0.000      -0.146      -0.093      *
   BULLY15            0.072       0.014      0.000       0.044       0.099      *
   PRESS15            0.085       0.014      0.000       0.060       0.113      *
   DUTIES15          -0.085       0.014      0.000      -0.111      -0.059      *

CHOICE15 WITH
   BULLY15           -0.106       0.013      0.000      -0.132      -0.081      *
   PRESS15           -0.074       0.013      0.000      -0.099      -0.047      *
   DUTIES15           0.167       0.013      0.000       0.143       0.192      *

BULLY15  WITH
   PRESS15            0.195       0.013      0.000       0.171       0.221      *
   DUTIES15          -0.138       0.013      0.000      -0.165      -0.113      *

PRESS15  WITH
   DUTIES15          -0.171       0.013      0.000      -0.199      -0.144      *

Means
   PHY15              0.002       0.013      0.453      -0.022       0.029
   KES15              0.010       0.014      0.264      -0.017       0.036
   CHOICE15          -0.003       0.014      0.429      -0.029       0.025
   BULLY15            0.002       0.013      0.424      -0.024       0.028
   PRESS15            0.001       0.013      0.451      -0.023       0.028
   DUTIES15          -0.004       0.013      0.400      -0.029       0.024

Variances
   PHY15              1.000       0.000      0.000       1.000       1.000
   KES15              1.000       0.000      0.000       1.000       1.000
   CHOICE15           1.000       0.000      0.000       1.000       1.000
   BULLY15            1.000       0.000      0.000       1.000       1.000
   PRESS15            1.000       0.000      0.000       1.000       1.000
   DUTIES15           1.000       0.000      0.000       1.000       1.000
```

```
Between Level

 KESCAT16   ON
    PRAFAC             0.171       0.301      0.303      -0.457       0.688

 Thresholds
    KESCAT16$1        13.068       5.980      0.000       7.260      29.913      *

 Residual Variances
    KESCAT16           0.940       0.135      0.000       0.511       1.000      *


R-SQUARE

Within Level

                                Posterior  One-Tailed         95% C.I.
    Variable        Estimate       S.D.      P-Value   Lower 2.5%  Upper 2.5%

    KESCAT16           0.245       0.017      0.000       0.213       0.279

Between Level

                                Posterior  One-Tailed         95% C.I.
    Variable        Estimate       S.D.      P-Value   Lower 2.5%  Upper 2.5%

    KESCAT16           0.060       0.135      0.000       0.000       0.489


TECHNICAL 1 OUTPUT


     PARAMETER SPECIFICATION FOR WITHIN


           TAU
              KESCAT16
              ________
                    0


           NU
              KESCAT16      PHY15         KES15         CHOICE15      BULLY15
```

0	0	0	0	0

NU

PRESS15	DUTIES15
0	0

LAMBDA

	KESCAT16	PHY15	KES15	CHOICE15	BULLY15
KESCAT16	0	0	0	0	0
PHY15	0	0	0	0	0
KES15	0	0	0	0	0
CHOICE15	0	0	0	0	0
BULLY15	0	0	0	0	0
PRESS15	0	0	0	0	0
DUTIES15	0	0	0	0	0

LAMBDA

	PRESS15	DUTIES15
KESCAT16	0	0
PHY15	0	0
KES15	0	0
CHOICE15	0	0
BULLY15	0	0
PRESS15	0	0
DUTIES15	0	0

THETA

	KESCAT16	PHY15	KES15	CHOICE15	BULLY15
KESCAT16	0				
PHY15	0	0			
KES15	0	0	0		
CHOICE15	0	0	0	0	
BULLY15	0	0	0	0	0
PRESS15	0	0	0	0	0
DUTIES15	0	0	0	0	0

THETA

	PRESS15	DUTIES15
PRESS15	0	
DUTIES15	0	0

ALPHA

KESCAT16	PHY15	KES15	CHOICE15	BULLY15
0	1	2	3	4

ALPHA

PRESS15	DUTIES15
5	6

BETA

	KESCAT16	PHY15	KES15	CHOICE15	BULLY15
KESCAT16	0	7	8	9	10
PHY15	0	0	0	0	0
KES15	0	0	0	0	0
CHOICE15	0	0	0	0	0
BULLY15	0	0	0	0	0
PRESS15	0	0	0	0	0
DUTIES15	0	0	0	0	0

BETA

	PRESS15	DUTIES15
KESCAT16	11	12
PHY15	0	0
KES15	0	0
CHOICE15	0	0
BULLY15	0	0
PRESS15	0	0
DUTIES15	0	0

PSI

	KESCAT16	PHY15	KES15	CHOICE15	BULLY15
KESCAT16	0				
PHY15	0	13			
KES15	0	14	15		
CHOICE15	0	16	17	18	
BULLY15	0	19	20	21	22
PRESS15	0	23	24	25	26
DUTIES15	0	28	29	30	31

PSI

	PRESS15	DUTIES15
PRESS15	27	
DUTIES15	32	33

PARAMETER SPECIFICATION FOR BETWEEN

TAU

KESCAT16
36

NU

KESCAT16	PRAFAC
0	0

LAMBDA

	KESCAT16	PRAFAC
KESCAT16	0	0
PRAFAC	0	0

THETA

KESCAT16 PRAFAC

	KESCAT16	PRAFAC
KESCAT16	0	
PRAFAC	0	0

ALPHA

KESCAT16	PRAFAC
0	0

BETA

	KESCAT16	PRAFAC
KESCAT16	0	34
PRAFAC	0	0

PSI

	KESCAT16	PRAFAC
KESCAT16	35	
PRAFAC	0	0

STARTING VALUES FOR WITHIN

TAU

KESCAT16
0.000

NU

KESCAT16	PHY15	KES15	CHOICE15	BULLY15
0.000	0.000	0.000	0.000	0.000

NU

PRESS15	DUTIES15
0.000	0.000

LAMBDA

	KESCAT16	PHY15	KES15	CHOICE15	BULLY15
KESCAT16	1.000	0.000	0.000	0.000	0.000
PHY15	0.000	1.000	0.000	0.000	0.000
KES15	0.000	0.000	1.000	0.000	0.000
CHOICE15	0.000	0.000	0.000	1.000	0.000
BULLY15	0.000	0.000	0.000	0.000	1.000
PRESS15	0.000	0.000	0.000	0.000	0.000
DUTIES15	0.000	0.000	0.000	0.000	0.000

LAMBDA

	PRESS15	DUTIES15
KESCAT16	0.000	0.000
PHY15	0.000	0.000
KES15	0.000	0.000
CHOICE15	0.000	0.000
BULLY15	0.000	0.000
PRESS15	1.000	0.000
DUTIES15	0.000	1.000

THETA

	KESCAT16	PHY15	KES15	CHOICE15	BULLY15
KESCAT16	0.000				
PHY15	0.000	0.000			
KES15	0.000	0.000	0.000		
CHOICE15	0.000	0.000	0.000	0.000	
BULLY15	0.000	0.000	0.000	0.000	0.000
PRESS15	0.000	0.000	0.000	0.000	0.000
DUTIES15	0.000	0.000	0.000	0.000	0.000

THETA

	PRESS15	DUTIES15
PRESS15	0.000	
DUTIES15	0.000	0.000

ALPHA

KESCAT16	PHY15	KES15	CHOICE15	BULLY15
0.000	0.000	0.000	0.000	0.000

ALPHA

PRESS15	DUTIES15
0.000	0.000

BETA

	KESCAT16	PHY15	KES15	CHOICE15	BULLY15
KESCAT16	0.000	0.000	0.000	0.000	0.000
PHY15	0.000	0.000	0.000	0.000	0.000
KES15	0.000	0.000	0.000	0.000	0.000
CHOICE15	0.000	0.000	0.000	0.000	0.000
BULLY15	0.000	0.000	0.000	0.000	0.000
PRESS15	0.000	0.000	0.000	0.000	0.000
DUTIES15	0.000	0.000	0.000	0.000	0.000

BETA

	PRESS15	DUTIES15
KESCAT16	0.000	0.000
PHY15	0.000	0.000
KES15	0.000	0.000
CHOICE15	0.000	0.000
BULLY15	0.000	0.000
PRESS15	0.000	0.000
DUTIES15	0.000	0.000

PSI

	KESCAT16	PHY15	KES15	CHOICE15	BULLY15
KESCAT16	1.000				
PHY15	0.000	0.268			
KES15	0.000	0.000	0.048		
CHOICE15	0.000	0.000	0.000	0.549	

```
BULLY15        0.000          0.000          0.000          0.000          0.108
PRESS15        0.000          0.000          0.000          0.000          0.000
DUTIES15       0.000          0.000          0.000          0.000          0.000


           PSI
              PRESS15       DUTIES15
              ________      ________
PRESS15        0.501
DUTIES15       0.000          0.414


    STARTING VALUES FOR BETWEEN


           TAU
              KESCAT16
              ________
               1.091


           NU
              KESCAT16      PRAFAC
              ________      ________
               0.000          0.000


           LAMBDA
              KESCAT16      PRAFAC
              ________      ________
KESCAT16       1.000          0.000
PRAFAC         0.000          1.000


           THETA
              KESCAT16      PRAFAC
              ________      ________
KESCAT16       0.000
PRAFAC         0.000          0.000


           ALPHA
              KESCAT16      PRAFAC
              ________      ________
```

```
              0.000         0.000


           BETA
              KESCAT16      PRAFAC
              ________      ________
 KESCAT16       0.000         0.000
 PRAFAC         0.000         0.000


           PSI
              KESCAT16      PRAFAC
              ________      ________
 KESCAT16       1.000
 PRAFAC         0.000         0.506


     PRIORS FOR ALL PARAMETERS            PRIOR MEAN      PRIOR VARIANCE     PRIOR STD. DEV.

     Parameter 1~N(0.000,infinity)           0.0000            infinity            infinity
     Parameter 2~N(0.000,infinity)           0.0000            infinity            infinity
     Parameter 3~N(0.000,infinity)           0.0000            infinity            infinity
     Parameter 4~N(0.000,infinity)           0.0000            infinity            infinity
     Parameter 5~N(0.000,infinity)           0.0000            infinity            infinity
     Parameter 6~N(0.000,infinity)           0.0000            infinity            infinity
     Parameter 7~N(0.000,5.000)              0.0000              5.0000              2.2361
     Parameter 8~N(0.000,5.000)              0.0000              5.0000              2.2361
     Parameter 9~N(0.000,5.000)              0.0000              5.0000              2.2361
     Parameter 10~N(0.000,5.000)             0.0000              5.0000              2.2361
     Parameter 11~N(0.000,5.000)             0.0000              5.0000              2.2361
     Parameter 12~N(0.000,5.000)             0.0000              5.0000              2.2361
     Parameter 13~IW(1.000,7)              infinity            infinity            infinity
     Parameter 14~IW(0.000,7)              infinity            infinity            infinity
     Parameter 15~IW(1.000,7)              infinity            infinity            infinity
     Parameter 16~IW(0.000,7)              infinity            infinity            infinity
     Parameter 17~IW(0.000,7)              infinity            infinity            infinity
     Parameter 18~IW(1.000,7)              infinity            infinity            infinity
     Parameter 19~IW(0.000,7)              infinity            infinity            infinity
     Parameter 20~IW(0.000,7)              infinity            infinity            infinity
     Parameter 21~IW(0.000,7)              infinity            infinity            infinity
     Parameter 22~IW(1.000,7)              infinity            infinity            infinity
     Parameter 23~IW(0.000,7)              infinity            infinity            infinity
     Parameter 24~IW(0.000,7)              infinity            infinity            infinity
```

Parameter 25~IW(0.000,7)	infinity	infinity	infinity
Parameter 26~IW(0.000,7)	infinity	infinity	infinity
Parameter 27~IW(1.000,7)	infinity	infinity	infinity
Parameter 28~IW(0.000,7)	infinity	infinity	infinity
Parameter 29~IW(0.000,7)	infinity	infinity	infinity
Parameter 30~IW(0.000,7)	infinity	infinity	infinity
Parameter 31~IW(0.000,7)	infinity	infinity	infinity
Parameter 32~IW(0.000,7)	infinity	infinity	infinity
Parameter 33~IW(1.000,7)	infinity	infinity	infinity
Parameter 34~N(0.000,5.000)	0.0000	5.0000	2.2361
Parameter 35~IG(-1.000,0.000)	infinity	infinity	infinity
Parameter 36~N(0.000,5.000)	0.0000	5.0000	2.2361

TECHNICAL 3 OUTPUT

ESTIMATED COVARIANCE MATRIX FOR PARAMETER ESTIMATES

	1	2	3	4	5
1	0.938683D-04				
2	0.110560D-04	0.175189D-04			
3	-0.175439D-04	-0.719224D-05	0.201542D-03		
4	0.303738D-05	0.402189D-05	-0.967992D-05	0.385371D-04	
5	0.395283D-05	0.655954D-05	-0.148262D-04	0.141004D-04	0.174206D-03
6	-0.874466D-05	-0.906851D-05	0.283325D-04	-0.115244D-04	-0.228653D-04
7	-0.501655D-05	0.145965D-05	0.449380D-05	0.357671D-05	0.103765D-04
8	0.128742D-05	0.112194D-04	-0.295509D-04	-0.641052D-05	0.259075D-04
9	0.502076D-05	-0.271759D-06	0.132947D-04	-0.495224D-05	0.518231D-05
10	-0.194210D-04	-0.698845D-06	0.243800D-04	0.137872D-04	0.332484D-05
11	0.227071D-05	0.643833D-06	-0.248445D-05	0.222070D-05	0.533832D-06
12	0.358548D-05	-0.160668D-05	0.108539D-04	0.361168D-05	-0.843662D-05
13	0.760738D-07	0.139427D-05	-0.666209D-05	0.230798D-05	-0.180164D-05
14	-0.218241D-06	0.273818D-06	-0.368652D-05	0.480914D-06	-0.594153D-06
15	-0.346169D-06	0.107129D-06	-0.141328D-05	0.118306D-06	0.645162D-06
16	0.389693D-06	0.363356D-06	0.133985D-05	-0.755485D-06	-0.388040D-05
17	0.171962D-05	0.106374D-05	0.112763D-05	0.608644D-06	0.631110D-08
18	0.497777D-05	0.370695D-05	0.320315D-05	-0.495728D-06	0.890489D-05
19	0.154202D-05	0.127913D-05	-0.177005D-05	0.378289D-06	0.531297D-06
20	0.447701D-06	0.442980D-06	-0.303454D-06	-0.163859D-06	-0.134135D-06
21	-0.541013D-05	-0.707051D-06	0.564073D-06	0.637963D-06	-0.195137D-05
22	0.571903D-06	0.188785D-06	-0.140301D-05	0.121286D-05	0.207884D-05
23	0.344091D-05	-0.208222D-06	-0.101362D-06	-0.971900D-06	0.325537D-05
24	0.292079D-06	0.655795D-06	-0.115318D-05	-0.142941D-06	0.559810D-07

25	-0.751599D-05	-0.176742D-05	0.696858D-06	0.185636D-05	0.437051D-05
26	-0.234921D-05	-0.597078D-06	-0.199788D-05	0.776143D-06	-0.934538D-06
27	0.518308D-06	-0.119253D-05	-0.790713D-05	0.149710D-05	-0.515588D-05
28	-0.178660D-05	-0.152240D-05	0.339655D-05	0.117502D-05	-0.508425D-06
29	0.118925D-05	-0.240002D-06	0.173173D-05	0.338439D-06	0.141179D-05
30	-0.959182D-06	-0.103686D-06	-0.969555D-05	-0.358203D-05	-0.590045D-05
31	-0.119349D-05	-0.744946D-06	0.195138D-05	-0.372809D-07	0.566338D-06
32	0.929545D-06	-0.290811D-06	0.608105D-05	0.227268D-05	0.680666D-05
33	0.126513D-07	0.291698D-05	-0.110119D-04	-0.155689D-05	-0.276545D-05
34	-0.769887D-05	-0.199016D-05	0.376229D-05	-0.342253D-05	-0.263985D-05
35	-0.715080D-06	0.708126D-06	0.249330D-05	0.135981D-05	-0.506401D-05
36	-0.638628D-05	0.717910D-05	0.813637D-05	-0.430307D-05	-0.598660D-05

ESTIMATED COVARIANCE MATRIX FOR PARAMETER ESTIMATES

	6	7	8	9	10
6	0.149526D-03				
7	-0.121886D-04	0.111537D-02			
8	-0.407597D-04	-0.365892D-03	0.715260D-02		
9	0.968312D-05	0.508696D-04	0.136112D-03	0.626289D-03	
10	-0.230776D-04	-0.182277D-05	-0.492093D-03	0.106916D-03	0.228506D-02
11	0.750348D-05	-0.297866D-04	-0.365304D-03	0.151371D-04	-0.231487D-03
12	0.443108D-05	0.549675D-04	0.896658D-04	-0.746696D-04	0.938434D-04
13	-0.160397D-05	0.513919D-05	0.372591D-04	-0.734816D-05	-0.270960D-05
14	-0.647154D-06	0.212634D-05	0.971638D-05	-0.103114D-05	0.291294D-05
15	-0.377660D-06	0.220949D-06	0.617709D-05	-0.152712D-06	0.890130D-08
16	-0.190045D-05	0.707440D-05	0.297309D-05	0.156805D-04	-0.249466D-05
17	-0.152485D-05	0.280939D-05	0.193696D-04	0.298197D-05	0.215771D-05
18	-0.100445D-05	0.323577D-04	0.292084D-04	0.162699D-04	0.223677D-04
19	0.197108D-06	-0.334503D-05	-0.126510D-05	0.437008D-05	0.243250D-05
20	0.223375D-06	-0.272312D-05	-0.104434D-05	0.541627D-07	0.296241D-05
21	0.175171D-05	-0.540865D-05	-0.362253D-05	-0.220181D-05	0.443349D-05
22	-0.231602D-07	0.594999D-06	0.158962D-05	0.505414D-05	0.146935D-05
23	0.386772D-05	0.768317D-05	0.158538D-04	-0.646532D-05	-0.840729D-06
24	0.974281D-06	0.308706D-05	0.924296D-05	-0.443964D-06	-0.719762D-05
25	0.114082D-04	-0.540084D-05	0.877195D-05	0.353477D-05	-0.113146D-04
26	-0.184065D-05	0.581307D-05	-0.991863D-05	0.602970D-05	-0.642471D-05
27	-0.296154D-05	0.215090D-04	-0.584681D-04	0.240335D-04	-0.234965D-04
28	-0.153850D-05	0.856864D-05	-0.136491D-04	0.931854D-05	-0.193706D-05
29	-0.941514D-06	0.232404D-05	-0.151546D-05	0.201098D-05	-0.308816D-05
30	0.135701D-05	-0.853634D-05	-0.439108D-04	-0.711223D-05	-0.552014D-05
31	0.418365D-06	-0.859474D-05	0.146161D-04	-0.875768D-05	-0.352979D-05
32	-0.109718D-05	-0.148427D-04	0.189460D-04	-0.115400D-04	-0.211363D-04

33	0.104574D-05	0.710942D-05	-0.285038D-04	0.519177D-05	0.200923D-04
34	0.320623D-05	0.545972D-04	0.660970D-05	-0.228133D-05	-0.338781D-04
35	0.518878D-06	-0.392564D-05	0.484682D-04	-0.171547D-05	0.765881D-05
36	-0.154495D-04	0.146715D-03	0.110397D-02	-0.211845D-04	0.376178D-04

ESTIMATED COVARIANCE MATRIX FOR PARAMETER ESTIMATES

	11	12	13	14	15
11	0.690560D-03				
12	0.102879D-03	0.727443D-03			
13	0.434716D-05	0.116318D-04	0.103504D-03		
14	-0.209671D-05	0.179684D-06	0.943782D-05	0.102167D-04	
15	-0.129213D-05	-0.410169D-06	0.541963D-06	0.194496D-05	0.347990D-05
16	0.188985D-05	0.163385D-04	-0.157056D-04	-0.412515D-05	-0.127742D-05
17	-0.334487D-06	0.543486D-05	-0.179977D-05	-0.152712D-05	-0.962981D-06
18	-0.146902D-04	0.178964D-05	0.346284D-05	0.353473D-05	0.353519D-06
19	0.242341D-05	-0.142756D-05	0.508166D-05	0.261670D-05	0.434366D-06
20	-0.671840D-06	0.778682D-06	0.100367D-06	0.765520D-06	0.698237D-06
21	-0.784124D-06	-0.725809D-05	-0.129355D-05	-0.123302D-05	0.478936D-07
22	0.226759D-05	-0.200108D-06	-0.139371D-05	0.346158D-07	0.582289D-07
23	-0.445971D-05	0.110306D-04	0.710503D-05	0.560302D-05	0.174713D-05
24	0.121284D-05	0.395532D-05	0.144041D-05	0.170020D-05	0.168369D-05
25	0.775484D-05	-0.152753D-04	-0.232983D-05	-0.102751D-05	0.136714D-06
26	0.219809D-05	-0.365049D-05	-0.872226D-06	0.911576D-06	0.386114D-06
27	0.168868D-04	0.112714D-04	0.390811D-05	0.219192D-05	0.232512D-06
28	-0.250915D-05	-0.874563D-05	-0.113000D-04	-0.574661D-05	-0.747382D-06
29	0.117345D-05	0.536871D-05	-0.248665D-05	-0.177203D-05	-0.191853D-05
30	0.273263D-05	-0.151930D-04	0.218312D-05	0.936971D-06	0.788571D-06
31	0.399617D-05	-0.209335D-05	-0.304934D-05	-0.198028D-05	-0.286296D-06
32	0.306495D-05	0.256051D-05	-0.652254D-05	-0.231329D-05	-0.725195D-06
33	-0.436861D-06	0.602459D-07	0.695026D-05	0.257663D-05	0.190927D-05
34	-0.892261D-05	0.245979D-05	0.443630D-05	0.301041D-05	0.245534D-06
35	0.773498D-05	0.168581D-06	0.115071D-05	-0.724643D-06	-0.250658D-06
36	-0.913495D-04	-0.688398D-04	-0.739374D-06	0.538948D-05	0.290576D-05

ESTIMATED COVARIANCE MATRIX FOR PARAMETER ESTIMATES

	16	17	18	19	20
16	0.112761D-03				
17	0.121434D-04	0.193258D-04			
18	-0.387349D-04	-0.181040D-04	0.457917D-03		
19	-0.512634D-05	-0.599434D-06	0.313046D-05	0.226790D-04	

20	-0.943896D-06	-0.678003D-06	-0.119744D-05	0.272035D-05	0.379299D-05
21	0.714998D-05	0.453636D-05	-0.215711D-04	-0.360729D-05	-0.841716D-06
22	0.125495D-05	-0.694479D-06	-0.541660D-05	0.213853D-05	0.164519D-05
23	-0.818243D-05	-0.110168D-05	0.894927D-05	0.945273D-05	0.190683D-05
24	-0.199325D-05	-0.711109D-06	-0.972864D-06	0.124245D-05	0.174560D-05
25	0.154574D-04	0.116897D-04	-0.344989D-04	-0.690885D-06	-0.187670D-06
26	0.601330D-06	-0.113765D-06	0.609394D-05	0.341320D-05	0.212458D-05
27	0.833258D-05	-0.112015D-06	0.527576D-05	0.434973D-05	0.218114D-05
28	0.188888D-04	0.255626D-05	-0.184238D-04	-0.661558D-05	-0.138018D-05
29	0.306449D-05	0.358872D-05	-0.419084D-05	-0.873505D-06	-0.119987D-05
30	-0.153990D-04	-0.101145D-04	0.637044D-04	0.100989D-05	0.613226D-06
31	0.266778D-05	0.165680D-06	-0.791096D-05	-0.424554D-05	-0.210470D-05
32	0.464602D-05	0.163364D-05	-0.919299D-05	-0.230616D-05	-0.151692D-05
33	-0.663158D-05	-0.379216D-05	0.671385D-05	0.376330D-05	0.174480D-05
34	-0.791960D-05	-0.432094D-06	0.181533D-04	0.488594D-05	-0.103149D-05
35	0.407226D-06	0.475491D-06	0.814479D-05	-0.293239D-06	-0.578092D-06
36	-0.274330D-05	0.896725D-05	0.208500D-06	0.256467D-05	-0.289209D-06

ESTIMATED COVARIANCE MATRIX FOR PARAMETER ESTIMATES

	21	22	23	24	25
21	0.446292D-04				
22	-0.374338D-05	0.172506D-04			
23	-0.281317D-05	0.936519D-06	0.100371D-03		
24	-0.121489D-05	0.138372D-05	0.115222D-04	0.183584D-04	
25	0.244860D-04	-0.245242D-05	-0.151663D-04	-0.486518D-05	0.201203D-03
26	-0.331763D-05	0.668186D-05	0.449717D-05	0.514925D-05	-0.731149D-05
27	-0.858289D-05	0.118166D-05	0.212478D-04	0.209812D-04	-0.313189D-04
28	0.236666D-05	-0.152554D-05	-0.157800D-04	-0.170628D-05	0.710927D-05
29	0.336659D-06	-0.475120D-06	-0.250872D-05	-0.411813D-05	0.271098D-05
30	-0.139444D-04	0.154404D-05	0.506777D-05	0.111769D-05	-0.387684D-04
31	0.631082D-05	-0.433331D-05	-0.125494D-06	-0.218769D-05	0.473512D-05
32	0.387603D-05	-0.125736D-05	-0.127881D-04	-0.124840D-04	0.306541D-04
33	-0.424090D-05	0.134335D-05	0.539471D-05	0.193722D-05	-0.121056D-04
34	0.534862D-05	-0.191671D-05	-0.314749D-07	-0.203089D-05	-0.708185D-05
35	-0.624669D-06	0.593304D-07	-0.640215D-06	-0.913656D-06	-0.622934D-06
36	0.228053D-05	-0.276039D-05	0.125691D-04	-0.898519D-06	0.371348D-05

ESTIMATED COVARIANCE MATRIX FOR PARAMETER ESTIMATES

	26	27	28	29	30
26	0.402637D-04				

27	0.346864D-04	0.398754D-03			
28	-0.282463D-05	-0.451797D-05	0.828440D-04		
29	-0.832834D-06	-0.575658D-05	0.100633D-04	0.159380D-04	
30	0.412074D-05	-0.290243D-06	-0.172786D-04	-0.725513D-05	0.161413D-03
31	-0.675175D-05	-0.103625D-04	0.830636D-05	0.462720D-05	-0.909053D-05
32	-0.138352D-04	-0.643166D-04	0.146374D-04	0.113582D-04	-0.222901D-04
33	0.398170D-05	0.105888D-04	-0.169486D-04	-0.157794D-04	0.485957D-04
34	-0.309117D-05	-0.199606D-04	-0.342192D-05	-0.121153D-05	0.143446D-04
35	-0.842967D-07	0.691166D-06	0.184640D-05	0.801103D-06	0.444820D-06
36	-0.418577D-05	-0.575297D-05	0.122131D-06	-0.463374D-05	-0.459853D-05

ESTIMATED COVARIANCE MATRIX FOR PARAMETER ESTIMATES

	31	32	33	34	35
31	0.344438D-04				
32	0.183498D-04	0.166722D-03			
33	-0.184664D-04	-0.547187D-04	0.255545D-03		
34	-0.537674D-05	-0.130775D-05	0.785758D-05	0.786983D-03	
35	0.131005D-06	-0.925937D-06	0.333482D-05	0.334548D-04	0.653232D-04
36	0.107845D-05	0.564238D-05	0.243334D-05	-0.686343D-04	0.465635D-04

ESTIMATED COVARIANCE MATRIX FOR PARAMETER ESTIMATES

	36
36	0.121347D-02

ESTIMATED CORRELATION MATRIX FOR PARAMETER ESTIMATES

	1	2	3	4	5
1	1.000				
2	0.273	1.000			
3	-0.128	-0.121	1.000		
4	0.051	0.155	-0.110	1.000	
5	0.031	0.119	-0.079	0.172	1.000
6	-0.074	-0.177	0.163	-0.152	-0.142
7	-0.016	0.010	0.009	0.017	0.024
8	0.002	0.032	-0.025	-0.012	0.023
9	0.021	-0.003	0.037	-0.032	0.016
10	-0.042	-0.003	0.036	0.046	0.005
11	0.009	0.006	-0.007	0.014	0.002
12	0.014	-0.014	0.028	0.022	-0.024

13	0.001	0.033	-0.046	0.037	-0.013
14	-0.007	0.020	-0.081	0.024	-0.014
15	-0.019	0.014	-0.053	0.010	0.026
16	0.004	0.008	0.009	-0.011	-0.028
17	0.040	0.058	0.018	0.022	0.000
18	0.024	0.041	0.011	-0.004	0.032
19	0.033	0.064	-0.026	0.013	0.008
20	0.024	0.054	-0.011	-0.014	-0.005
21	-0.084	-0.025	0.006	0.015	-0.022
22	0.014	0.011	-0.024	0.047	0.038
23	0.035	-0.005	-0.001	-0.016	0.025
24	0.007	0.037	-0.019	-0.005	0.001
25	-0.055	-0.030	0.003	0.021	0.023
26	-0.038	-0.022	-0.022	0.020	-0.011
27	0.003	-0.014	-0.028	0.012	-0.020
28	-0.020	-0.040	0.026	0.021	-0.004
29	0.031	-0.014	0.031	0.014	0.027
30	-0.008	-0.002	-0.054	-0.045	-0.035
31	-0.021	-0.030	0.023	-0.001	0.007
32	0.007	-0.005	0.033	0.028	0.040
33	0.000	0.044	-0.049	-0.016	-0.013
34	-0.028	-0.017	0.009	-0.020	-0.007
35	-0.009	0.021	0.022	0.027	-0.047
36	-0.019	0.049	0.016	-0.020	-0.013

ESTIMATED CORRELATION MATRIX FOR PARAMETER ESTIMATES

	6	7	8	9	10
6	1.000				
7	-0.030	1.000			
8	-0.039	-0.130	1.000		
9	0.032	0.061	0.064	1.000	
10	-0.039	-0.001	-0.122	0.089	1.000
11	0.023	-0.034	-0.164	0.023	-0.184
12	0.013	0.061	0.039	-0.111	0.073
13	-0.013	0.015	0.043	-0.029	-0.006
14	-0.017	0.020	0.036	-0.013	0.019
15	-0.017	0.004	0.039	-0.003	0.000
16	-0.015	0.020	0.003	0.059	-0.005
17	-0.028	0.019	0.052	0.027	0.010
18	-0.004	0.045	0.016	0.030	0.022
19	0.003	-0.021	-0.003	0.037	0.011
20	0.009	-0.042	-0.006	0.001	0.032

21	0.021	-0.024	-0.006	-0.013	0.014
22	0.000	0.004	0.005	0.049	0.007
23	0.032	0.023	0.019	-0.026	-0.002
24	0.019	0.022	0.026	-0.004	-0.035
25	0.066	-0.011	0.007	0.010	-0.017
26	-0.024	0.027	-0.018	0.038	-0.021
27	-0.012	0.032	-0.035	0.048	-0.025
28	-0.014	0.028	-0.018	0.041	-0.004
29	-0.019	0.017	-0.004	0.020	-0.016
30	0.009	-0.020	-0.041	-0.022	-0.009
31	0.006	-0.044	0.029	-0.060	-0.013
32	-0.007	-0.034	0.017	-0.036	-0.034
33	0.005	0.013	-0.021	0.013	0.026
34	0.009	0.058	0.003	-0.003	-0.025
35	0.005	-0.015	0.071	-0.008	0.020
36	-0.036	0.126	0.375	-0.024	0.023

ESTIMATED CORRELATION MATRIX FOR PARAMETER ESTIMATES

	11	12	13	14	15
11	1.000				
12	0.145	1.000			
13	0.016	0.042	1.000		
14	-0.025	0.002	0.290	1.000	
15	-0.026	-0.008	0.029	0.326	1.000
16	0.007	0.057	-0.145	-0.122	-0.064
17	-0.003	0.046	-0.040	-0.109	-0.117
18	-0.026	0.003	0.016	0.052	0.009
19	0.019	-0.011	0.105	0.172	0.049
20	-0.013	0.015	0.005	0.123	0.192
21	-0.004	-0.040	-0.019	-0.058	0.004
22	0.021	-0.002	-0.033	0.003	0.008
23	-0.017	0.041	0.070	0.175	0.093
24	0.011	0.034	0.033	0.124	0.211
25	0.021	-0.040	-0.016	-0.023	0.005
26	0.013	-0.021	-0.014	0.045	0.033
27	0.032	0.021	0.019	0.034	0.006
28	-0.010	-0.036	-0.122	-0.198	-0.044
29	0.011	0.050	-0.061	-0.139	-0.258
30	0.008	-0.044	0.017	0.023	0.033
31	0.026	-0.013	-0.051	-0.106	-0.026
32	0.009	0.007	-0.050	-0.056	-0.030
33	-0.001	0.000	0.043	0.050	0.064

34	-0.012	0.003	0.016	0.034	0.005
35	0.036	0.001	0.014	-0.028	-0.017
36	-0.100	-0.073	-0.002	0.048	0.045

ESTIMATED CORRELATION MATRIX FOR PARAMETER ESTIMATES

	16	17	18	19	20
16	1.000				
17	0.260	1.000			
18	-0.170	-0.192	1.000		
19	-0.101	-0.029	0.031	1.000	
20	-0.046	-0.079	-0.029	0.293	1.000
21	0.101	0.154	-0.151	-0.113	-0.065
22	0.028	-0.038	-0.061	0.108	0.203
23	-0.077	-0.025	0.042	0.198	0.098
24	-0.044	-0.038	-0.011	0.061	0.209
25	0.103	0.187	-0.114	-0.010	-0.007
26	0.009	-0.004	0.045	0.113	0.172
27	0.039	-0.001	0.012	0.046	0.056
28	0.195	0.064	-0.095	-0.153	-0.078
29	0.072	0.204	-0.049	-0.046	-0.154
30	-0.114	-0.181	0.234	0.017	0.025
31	0.043	0.006	-0.063	-0.152	-0.184
32	0.034	0.029	-0.033	-0.038	-0.060
33	-0.039	-0.054	0.020	0.049	0.056
34	-0.027	-0.004	0.030	0.037	-0.019
35	0.005	0.013	0.047	-0.008	-0.037
36	-0.007	0.059	0.000	0.015	-0.004

ESTIMATED CORRELATION MATRIX FOR PARAMETER ESTIMATES

	21	22	23	24	25
21	1.000				
22	-0.135	1.000			
23	-0.042	0.023	1.000		
24	-0.042	0.078	0.268	1.000	
25	0.258	-0.042	-0.107	-0.080	1.000
26	-0.078	0.254	0.071	0.189	-0.081
27	-0.064	0.014	0.106	0.245	-0.111
28	0.039	-0.040	-0.173	-0.044	0.055
29	0.013	-0.029	-0.063	-0.241	0.048
30	-0.164	0.029	0.040	0.021	-0.215

31	0.161	-0.178	-0.002	-0.087	0.057
32	0.045	-0.023	-0.099	-0.226	0.167
33	-0.040	0.020	0.034	0.028	-0.053
34	0.029	-0.016	0.000	-0.017	-0.018
35	-0.012	0.002	-0.008	-0.026	-0.005
36	0.010	-0.019	0.036	-0.006	0.008

ESTIMATED CORRELATION MATRIX FOR PARAMETER ESTIMATES

	26	27	28	29	30
26	1.000				
27	0.274	1.000			
28	-0.049	-0.025	1.000		
29	-0.033	-0.072	0.277	1.000	
30	0.051	-0.001	-0.149	-0.143	1.000
31	-0.181	-0.088	0.155	0.197	-0.122
32	-0.169	-0.249	0.125	0.220	-0.136
33	0.039	0.033	-0.116	-0.247	0.239
34	-0.017	-0.036	-0.013	-0.011	0.040
35	-0.002	0.004	0.025	0.025	0.004
36	-0.019	-0.008	0.000	-0.033	-0.010

ESTIMATED CORRELATION MATRIX FOR PARAMETER ESTIMATES

	31	32	33	34	35
31	1.000				
32	0.242	1.000			
33	-0.197	-0.265	1.000		
34	-0.033	-0.004	0.018	1.000	
35	0.003	-0.009	0.026	0.148	1.000
36	0.005	0.013	0.004	-0.070	0.165

ESTIMATED CORRELATION MATRIX FOR PARAMETER ESTIMATES

	36
36	1.000

TECHNICAL 8 OUTPUT

```
TECHNICAL 8 OUTPUT FOR BAYES ESTIMATION

  CHAIN    BSEED
  1        0
  2        285380
  3        253358

                 POTENTIAL       PARAMETER WITH
  ITERATION    SCALE REDUCTION      HIGHEST PSR
  100              1.010               13
  200              1.007               18
  300              1.006               6
  400              1.003               30
  500              1.005               32
  600              1.003               32
  700              1.002               18
  800              1.002               25
  900              1.002               27
  1000             1.001               27
```

Worsening of distress, curvilinear effects

SUMMARY OF DATA

Number of clusters 58

Size (s)	Cluster ID with Size s
7	22
17	166
20	80
26	71 74 205
29	65
31	192
33	228
37	182 106
39	72
41	170 181
42	50 30
43	75
45	173
47	226 165
48	108
50	107 114
51	81
52	40
55	84 92
57	162
59	206
61	61 83 116
62	36 93 169
63	69
64	4
69	68
73	174 152
74	6 154
75	145
89	98
90	178 2
95	144
100	156
104	202
106	142
134	122

186	24
192	209
246	204
278	125
405	217
780	110
873	100

COVARIANCE COVERAGE OF DATA

Minimum covariance coverage value   0.100

Number of missing data patterns   9

PROPORTION OF DATA PRESENT

Covariance Coverage

	KESCAT16	PRAFAC	PRAFACSQ	PHY15	KES15
KESCAT16	1.000				
PRAFAC	1.000	1.000			
PRAFACSQ	1.000	1.000	1.000		
PHY15	0.950	0.950	0.950	0.950	
KES15	0.919	0.919	0.919	0.919	0.919
CHOICE15	0.913	0.913	0.913	0.913	0.913
BULLY15	0.913	0.913	0.913	0.913	0.913
PRESS15	0.913	0.913	0.913	0.913	0.913
DUTIES15	0.913	0.913	0.913	0.913	0.913

Covariance Coverage

	CHOICE15	BULLY15	PRESS15	DUTIES15
CHOICE15	0.913			
BULLY15	0.912	0.913		
PRESS15	0.913	0.912	0.913	
DUTIES15	0.913	0.913	0.913	0.913

UNIVARIATE PROPORTIONS AND COUNTS FOR CATEGORICAL VARIABLES

KESCAT16

Category 1	0.877	5135.000
Category 2	0.123	720.000

UNIVARIATE SAMPLE STATISTICS

UNIVARIATE HIGHER-ORDER MOMENT DESCRIPTIVE STATISTICS

Variable/ Sample Size	Mean/ Variance	Skewness/ Kurtosis	Minimum/ Maximum	% with Min/Max	Percentiles 20%/60%	Percentiles 40%/80%	Median
PRAFAC	-0.185	0.245	-2.338	1.72%	-1.448	-0.527	-0.318
58.000	1.345	-0.636	2.338	1.72%	0.080	0.752	
PRAFACSQ	1.379	1.240	0.002	1.72%	0.151	0.381	0.681
58.000	2.369	0.557	5.467	1.72%	1.168	2.366	
PHY15	0.000	0.526	-0.865	32.48%	-0.865	0.135	0.135
5563.000	0.536	0.021	3.135	0.07%	0.135	0.135	
KES15	0.000	0.475	-0.374	22.78%	-0.374	-0.207	-0.041
5382.000	0.095	-0.842	0.626	6.60%	-0.041	0.293	
CHOICE15	0.000	-0.400	-2.221	7.67%	-1.221	-0.221	-0.221
5345.000	1.097	-0.356	1.779	8.62%	0.779	0.779	
BULLY15	0.000	3.522	-0.153	88.29%	-0.153	-0.153	-0.153
5344.000	0.216	14.164	3.847	0.09%	-0.153	-0.153	
PRESS15	0.000	0.267	-1.445	19.70%	-0.445	-0.445	-0.445
5345.000	1.001	-0.357	2.555	2.75%	0.555	0.555	
DUTIES15	0.000	-1.296	-3.149	2.34%	-0.149	-0.149	-0.149
5347.000	0.828	1.939	0.851	39.74%	-0.149	0.851	

THE MODEL ESTIMATION TERMINATED NORMALLY

USE THE FBITERATIONS OPTION TO INCREASE THE NUMBER OF ITERATIONS BY A FACTOR
OF AT LEAST TWO TO CHECK CONVERGENCE AND THAT THE PSR VALUE DOES NOT INCREASE.

MODEL FIT INFORMATION

Number of Free Parameters 42

Bayesian Posterior Predictive Checking using Chi-Square

95% Confidence Interval for the Difference Between
the Observed and the Replicated Chi-Square Values

-21.646 30.855

Posterior Predictive P-Value 0.458

MODEL RESULTS

	Estimate	Posterior S.D.	One-Tailed P-Value	95% C.I. Lower 2.5%	Upper 2.5%	Significance
**Within Level**						
KESCAT16 ON						
PHY15	0.137	0.034	0.000	0.070	0.200	*
CHOICE15	-0.058	0.025	0.012	-0.106	-0.006	*
BULLY15	0.111	0.048	0.013	0.010	0.204	*
DUTIES15	-0.081	0.028	0.004	-0.135	-0.027	*
PRESS15	-0.034	0.025	0.095	-0.085	0.018	
KES15	1.611	0.087	0.000	1.445	1.787	*
KES15 WITH						
PHY15	0.055	0.003	0.000	0.048	0.061	*
CHOICE15	-0.036	0.004	0.000	-0.045	-0.028	*
BULLY15	0.022	0.002	0.000	0.018	0.026	*
PRESS15	0.053	0.004	0.000	0.045	0.062	*
DUTIES15	-0.051	0.004	0.000	-0.059	-0.043	*
PHY15 WITH						
CHOICE15	-0.093	0.010	0.000	-0.112	-0.073	*
BULLY15	0.024	0.005	0.000	0.016	0.033	*
PRESS15	0.063	0.010	0.000	0.042	0.084	*
DUTIES15	-0.057	0.009	0.000	-0.075	-0.040	*
CHOICE15 WITH						
BULLY15	-0.052	0.007	0.000	-0.065	-0.038	*
PRESS15	-0.078	0.014	0.000	-0.106	-0.050	*
DUTIES15	0.160	0.013	0.000	0.134	0.185	*

```
 BULLY15  WITH
    PRESS15            0.090       0.007      0.000       0.078       0.103      *
    DUTIES15          -0.059       0.006      0.000      -0.070      -0.047      *

 PRESS15  WITH
    DUTIES15          -0.156       0.013      0.000      -0.182      -0.132      *

 Means
    PHY15              0.002       0.010      0.424      -0.017       0.021
    KES15              0.003       0.004      0.259      -0.005       0.011
    CHOICE15          -0.004       0.014      0.407      -0.031       0.025
    BULLY15            0.001       0.006      0.402      -0.011       0.014
    PRESS15            0.003       0.014      0.432      -0.025       0.028
    DUTIES15          -0.004       0.013      0.395      -0.027       0.021

 Variances
    PHY15              0.536       0.010      0.000       0.516       0.556      *
    KES15              0.096       0.002      0.000       0.093       0.100      *
    CHOICE15           1.098       0.022      0.000       1.058       1.143      *
    BULLY15            0.216       0.004      0.000       0.208       0.224      *
    PRESS15            1.001       0.020      0.000       0.964       1.040      *
    DUTIES15           0.829       0.016      0.000       0.798       0.861      *

Between Level

 KESCAT16   ON
    PRAFAC             0.015       0.030      0.318      -0.044       0.072
    PRAFACSQ          -0.014       0.022      0.274      -0.059       0.030

 PRAFAC   WITH
    PRAFACSQ          -0.098       0.242      0.328      -0.604       0.364

 Means
    PRAFAC            -0.193       0.155      0.111      -0.517       0.115
    PRAFACSQ           1.375       0.205      0.000       0.980       1.780      *

 Thresholds
    KESCAT16$1         1.338       0.041      0.000       1.260       1.422      *

 Variances
    PRAFAC             1.363       0.265      0.000       0.970       1.980      *
    PRAFACSQ           2.382       0.468      0.000       1.702       3.513      *

 Residual Variances
```

KESCAT16	0.010	0.008	0.000	0.001	0.030	*

STANDARDIZED MODEL RESULTS

STDYX Standardization

	Estimate	Posterior S.D.	One-Tailed P-Value	95% C.I. Lower 2.5%	95% C.I. Upper 2.5%	Significance
**Within Level**						
KESCAT16 ON						
PHY15	0.087	0.021	0.000	0.045	0.127	*
CHOICE15	-0.053	0.023	0.012	-0.096	-0.005	*
BULLY15	0.045	0.019	0.013	0.004	0.082	*
DUTIES15	-0.064	0.022	0.004	-0.106	-0.021	*
PRESS15	-0.029	0.022	0.095	-0.073	0.015	
KES15	0.433	0.020	0.000	0.395	0.472	*
KES15 WITH						
PHY15	0.241	0.013	0.000	0.215	0.266	*
CHOICE15	-0.113	0.013	0.000	-0.137	-0.088	*
BULLY15	0.154	0.013	0.000	0.128	0.179	*
PRESS15	0.172	0.013	0.000	0.146	0.197	*
DUTIES15	-0.181	0.014	0.000	-0.206	-0.153	*
PHY15 WITH						
CHOICE15	-0.121	0.013	0.000	-0.146	-0.095	*
BULLY15	0.072	0.013	0.000	0.046	0.098	*
PRESS15	0.086	0.014	0.000	0.058	0.114	*
DUTIES15	-0.085	0.014	0.000	-0.113	-0.060	*
CHOICE15 WITH						
BULLY15	-0.107	0.014	0.000	-0.133	-0.078	*
PRESS15	-0.074	0.013	0.000	-0.101	-0.048	*
DUTIES15	0.168	0.013	0.000	0.141	0.193	*
BULLY15 WITH						
PRESS15	0.195	0.013	0.000	0.168	0.220	*
DUTIES15	-0.139	0.014	0.000	-0.164	-0.111	*
PRESS15 WITH						
DUTIES15	-0.171	0.013	0.000	-0.199	-0.146	*

```
 Means
    PHY15              0.002       0.013      0.424      -0.024       0.029
    KES15              0.010       0.014      0.259      -0.017       0.037
    CHOICE15          -0.004       0.013      0.407      -0.030       0.024
    BULLY15            0.003       0.013      0.402      -0.023       0.030
    PRESS15            0.003       0.014      0.432      -0.025       0.028
    DUTIES15          -0.004       0.014      0.395      -0.030       0.023

 Variances
    PHY15              1.000       0.000      0.000       1.000       1.000
    KES15              1.000       0.000      0.000       1.000       1.000
    CHOICE15           1.000       0.000      0.000       1.000       1.000
    BULLY15            1.000       0.000      0.000       1.000       1.000
    PRESS15            1.000       0.000      0.000       1.000       1.000
    DUTIES15           1.000       0.000      0.000       1.000       1.000

Between Level

 KESCAT16   ON
    PRAFAC             0.162       0.304      0.318      -0.487       0.683
    PRAFACSQ          -0.196       0.307      0.274      -0.778       0.410

 PRAFAC   WITH
    PRAFACSQ          -0.057       0.126      0.328      -0.301       0.189

 Means
    PRAFAC            -0.163       0.133      0.111      -0.432       0.101
    PRAFACSQ           0.895       0.158      0.000       0.592       1.214      *

 Thresholds
    KESCAT16$1        11.816       4.848      0.000       6.917      25.915      *

 Variances
    PRAFAC             1.000       0.000      0.000       1.000       1.000
    PRAFACSQ           1.000       0.000      0.000       1.000       1.000

 Residual Variances
    KESCAT16           0.810       0.197      0.000       0.271       0.993      *


R-SQUARE

Within Level
```

```
                              Posterior  One-Tailed         95% C.I.
    Variable        Estimate       S.D.      P-Value   Lower 2.5%  Upper 2.5%

    KESCAT16           0.245       0.017      0.000       0.212       0.279

Between Level

                              Posterior  One-Tailed         95% C.I.
    Variable        Estimate       S.D.      P-Value   Lower 2.5%  Upper 2.5%

    KESCAT16           0.190       0.197      0.000       0.007       0.729


TECHNICAL 1 OUTPUT


     PARAMETER SPECIFICATION FOR WITHIN


           TAU
              KESCAT16
              ________
                    0


           NU
              KESCAT16      PHY15         KES15         CHOICE15      BULLY15
              ________      ________      ________      ________      ________
                    0             0             0             0             0


           NU
              PRESS15       DUTIES15
              ________      ________
                    0             0


           LAMBDA
              KESCAT16      PHY15         KES15         CHOICE15      BULLY15
              ________      ________      ________      ________      ________
 KESCAT16           0             0             0             0             0
 PHY15              0             0             0             0             0
 KES15              0             0             0             0             0
```

CHOICE15	0	0	0	0	0
BULLY15	0	0	0	0	0
PRESS15	0	0	0	0	0
DUTIES15	0	0	0	0	0

LAMBDA

	PRESS15	DUTIES15
KESCAT16	0	0
PHY15	0	0
KES15	0	0
CHOICE15	0	0
BULLY15	0	0
PRESS15	0	0
DUTIES15	0	0

THETA

	KESCAT16	PHY15	KES15	CHOICE15	BULLY15
KESCAT16	0				
PHY15	0	0			
KES15	0	0	0		
CHOICE15	0	0	0	0	
BULLY15	0	0	0	0	0
PRESS15	0	0	0	0	0
DUTIES15	0	0	0	0	0

THETA

	PRESS15	DUTIES15
PRESS15	0	
DUTIES15	0	0

ALPHA

KESCAT16	PHY15	KES15	CHOICE15	BULLY15
0	1	2	3	4

ALPHA

	PRESS15	DUTIES15
	5	6

BETA

	KESCAT16	PHY15	KES15	CHOICE15	BULLY15
KESCAT16	0	7	8	9	10
PHY15	0	0	0	0	0
KES15	0	0	0	0	0
CHOICE15	0	0	0	0	0
BULLY15	0	0	0	0	0
PRESS15	0	0	0	0	0
DUTIES15	0	0	0	0	0

BETA

	PRESS15	DUTIES15
KESCAT16	11	12
PHY15	0	0
KES15	0	0
CHOICE15	0	0
BULLY15	0	0
PRESS15	0	0
DUTIES15	0	0

PSI

	KESCAT16	PHY15	KES15	CHOICE15	BULLY15
KESCAT16	0				
PHY15	0	13			
KES15	0	14	15		
CHOICE15	0	16	17	18	
BULLY15	0	19	20	21	22
PRESS15	0	23	24	25	26
DUTIES15	0	28	29	30	31

PSI

	PRESS15	DUTIES15

```
 PRESS15           27
 DUTIES15          32            33


     PARAMETER SPECIFICATION FOR BETWEEN


           TAU
              KESCAT16
              ________
                   42


           NU
              KESCAT16      PRAFAC        PRAFACSQ
              ________      ________      ________
                    0             0             0


           LAMBDA
              KESCAT16      PRAFAC        PRAFACSQ
              ________      ________      ________
 KESCAT16           0             0             0
 PRAFAC             0             0             0
 PRAFACSQ           0             0             0


           THETA
              KESCAT16      PRAFAC        PRAFACSQ
              ________      ________      ________
 KESCAT16           0
 PRAFAC             0             0
 PRAFACSQ           0             0             0


           ALPHA
              KESCAT16      PRAFAC        PRAFACSQ
              ________      ________      ________
                    0            34            35


           BETA
              KESCAT16      PRAFAC        PRAFACSQ
              ________      ________      ________
```

KESCAT16	0	36	37
PRAFAC	0	0	0
PRAFACSQ	0	0	0

PSI

	KESCAT16	PRAFAC	PRAFACSQ
KESCAT16	38		
PRAFAC	0	39	
PRAFACSQ	0	40	41

STARTING VALUES FOR WITHIN

TAU

KESCAT16
0.000

NU

KESCAT16	PHY15	KES15	CHOICE15	BULLY15
0.000	0.000	0.000	0.000	0.000

NU

PRESS15	DUTIES15
0.000	0.000

LAMBDA

	KESCAT16	PHY15	KES15	CHOICE15	BULLY15
KESCAT16	1.000	0.000	0.000	0.000	0.000
PHY15	0.000	1.000	0.000	0.000	0.000
KES15	0.000	0.000	1.000	0.000	0.000
CHOICE15	0.000	0.000	0.000	1.000	0.000
BULLY15	0.000	0.000	0.000	0.000	1.000
PRESS15	0.000	0.000	0.000	0.000	0.000
DUTIES15	0.000	0.000	0.000	0.000	0.000

LAMBDA

	PRESS15	DUTIES15
KESCAT16	0.000	0.000
PHY15	0.000	0.000
KES15	0.000	0.000
CHOICE15	0.000	0.000
BULLY15	0.000	0.000
PRESS15	1.000	0.000
DUTIES15	0.000	1.000

THETA

	KESCAT16	PHY15	KES15	CHOICE15	BULLY15
KESCAT16	0.000				
PHY15	0.000	0.000			
KES15	0.000	0.000	0.000		
CHOICE15	0.000	0.000	0.000	0.000	
BULLY15	0.000	0.000	0.000	0.000	0.000
PRESS15	0.000	0.000	0.000	0.000	0.000
DUTIES15	0.000	0.000	0.000	0.000	0.000

THETA

	PRESS15	DUTIES15
PRESS15	0.000	
DUTIES15	0.000	0.000

ALPHA

KESCAT16	PHY15	KES15	CHOICE15	BULLY15
0.000	0.000	0.000	0.000	0.000

ALPHA

PRESS15	DUTIES15
0.000	0.000

BETA

	KESCAT16	PHY15	KES15	CHOICE15	BULLY15
KESCAT16	0.000	0.000	0.000	0.000	0.000
PHY15	0.000	0.000	0.000	0.000	0.000
KES15	0.000	0.000	0.000	0.000	0.000
CHOICE15	0.000	0.000	0.000	0.000	0.000
BULLY15	0.000	0.000	0.000	0.000	0.000
PRESS15	0.000	0.000	0.000	0.000	0.000
DUTIES15	0.000	0.000	0.000	0.000	0.000

BETA

	PRESS15	DUTIES15
KESCAT16	0.000	0.000
PHY15	0.000	0.000
KES15	0.000	0.000
CHOICE15	0.000	0.000
BULLY15	0.000	0.000
PRESS15	0.000	0.000
DUTIES15	0.000	0.000

PSI

	KESCAT16	PHY15	KES15	CHOICE15	BULLY15
KESCAT16	1.000				
PHY15	0.000	0.268			
KES15	0.000	0.000	0.048		
CHOICE15	0.000	0.000	0.000	0.549	
BULLY15	0.000	0.000	0.000	0.000	0.108
PRESS15	0.000	0.000	0.000	0.000	0.000
DUTIES15	0.000	0.000	0.000	0.000	0.000

PSI

	PRESS15	DUTIES15
PRESS15	0.501	
DUTIES15	0.000	0.414

STARTING VALUES FOR BETWEEN

TAU

KESCAT16
1.091

NU

KESCAT16	PRAFAC	PRAFACSQ
0.000	0.000	0.000

LAMBDA

	KESCAT16	PRAFAC	PRAFACSQ
KESCAT16	1.000	0.000	0.000
PRAFAC	0.000	1.000	0.000
PRAFACSQ	0.000	0.000	1.000

THETA

	KESCAT16	PRAFAC	PRAFACSQ
KESCAT16	0.000		
PRAFAC	0.000	0.000	
PRAFACSQ	0.000	0.000	0.000

ALPHA

KESCAT16	PRAFAC	PRAFACSQ
0.000	0.006	1.011

BETA

	KESCAT16	PRAFAC	PRAFACSQ
KESCAT16	0.000	0.000	0.000
PRAFAC	0.000	0.000	0.000
PRAFACSQ	0.000	0.000	0.000

PSI

	KESCAT16	PRAFAC	PRAFACSQ
KESCAT16	1.000		
PRAFAC	0.000	0.506	
PRAFACSQ	0.000	0.000	0.754

PRIORS FOR ALL PARAMETERS	PRIOR MEAN	PRIOR VARIANCE	PRIOR STD. DEV.
Parameter 1~N(0.000,infinity)	0.0000	infinity	infinity
Parameter 2~N(0.000,infinity)	0.0000	infinity	infinity
Parameter 3~N(0.000,infinity)	0.0000	infinity	infinity
Parameter 4~N(0.000,infinity)	0.0000	infinity	infinity
Parameter 5~N(0.000,infinity)	0.0000	infinity	infinity
Parameter 6~N(0.000,infinity)	0.0000	infinity	infinity
Parameter 7~N(0.000,5.000)	0.0000	5.0000	2.2361
Parameter 8~N(0.000,5.000)	0.0000	5.0000	2.2361
Parameter 9~N(0.000,5.000)	0.0000	5.0000	2.2361
Parameter 10~N(0.000,5.000)	0.0000	5.0000	2.2361
Parameter 11~N(0.000,5.000)	0.0000	5.0000	2.2361
Parameter 12~N(0.000,5.000)	0.0000	5.0000	2.2361
Parameter 13~IW(1.000,7)	infinity	infinity	infinity
Parameter 14~IW(0.000,7)	infinity	infinity	infinity
Parameter 15~IW(1.000,7)	infinity	infinity	infinity
Parameter 16~IW(0.000,7)	infinity	infinity	infinity
Parameter 17~IW(0.000,7)	infinity	infinity	infinity
Parameter 18~IW(1.000,7)	infinity	infinity	infinity
Parameter 19~IW(0.000,7)	infinity	infinity	infinity
Parameter 20~IW(0.000,7)	infinity	infinity	infinity
Parameter 21~IW(0.000,7)	infinity	infinity	infinity
Parameter 22~IW(1.000,7)	infinity	infinity	infinity
Parameter 23~IW(0.000,7)	infinity	infinity	infinity
Parameter 24~IW(0.000,7)	infinity	infinity	infinity
Parameter 25~IW(0.000,7)	infinity	infinity	infinity
Parameter 26~IW(0.000,7)	infinity	infinity	infinity
Parameter 27~IW(1.000,7)	infinity	infinity	infinity
Parameter 28~IW(0.000,7)	infinity	infinity	infinity
Parameter 29~IW(0.000,7)	infinity	infinity	infinity
Parameter 30~IW(0.000,7)	infinity	infinity	infinity
Parameter 31~IW(0.000,7)	infinity	infinity	infinity
Parameter 32~IW(0.000,7)	infinity	infinity	infinity

Parameter 33~IW(1.000,7)	infinity	infinity	infinity
Parameter 34~N(0.000,infinity)	0.0000	infinity	infinity
Parameter 35~N(0.000,infinity)	0.0000	infinity	infinity
Parameter 36~N(0.000,5.000)	0.0000	5.0000	2.2361
Parameter 37~N(0.000,5.000)	0.0000	5.0000	2.2361
Parameter 38~IG(-1.000,0.000)	infinity	infinity	infinity
Parameter 39~IW(1.000,3)	infinity	infinity	infinity
Parameter 40~IW(0.000,3)	infinity	infinity	infinity
Parameter 41~IW(1.000,3)	infinity	infinity	infinity
Parameter 42~N(0.000,5.000)	0.0000	5.0000	2.2361

TECHNICAL 3 OUTPUT

ESTIMATED COVARIANCE MATRIX FOR PARAMETER ESTIMATES

	1	2	3	4	5
1	0.936376D-04				
2	0.798366D-05	0.180550D-04			
3	-0.139165D-04	-0.513896D-05	0.197589D-03		
4	0.428187D-05	0.342311D-05	-0.777480D-05	0.390046D-04	
5	0.825446D-05	0.991003D-05	-0.150790D-04	0.148541D-04	0.189934D-03
6	-0.776397D-05	-0.106769D-04	0.290380D-04	-0.114346D-04	-0.247479D-04
7	0.172071D-04	0.277540D-05	0.143274D-04	-0.503293D-05	0.450042D-05
8	0.157626D-04	0.143151D-04	0.138590D-05	-0.122530D-04	-0.698018D-04
9	0.444078D-05	-0.287563D-05	0.888645D-05	-0.312326D-05	-0.726351D-05
10	0.105927D-04	-0.604540D-05	-0.916626D-05	0.354423D-05	0.113812D-04
11	-0.750425D-05	0.109195D-05	0.668481D-05	0.439885D-05	0.212506D-04
12	-0.264777D-05	-0.749300D-06	0.137097D-04	0.118408D-05	-0.208235D-05
13	-0.542251D-06	-0.935162D-07	-0.382717D-05	0.823720D-06	-0.365313D-05
14	0.997927D-06	0.313457D-06	-0.167799D-05	0.362463D-06	-0.252420D-05
15	-0.603042D-06	0.178470D-06	-0.813909D-06	0.161104D-06	-0.848163D-06
16	0.325538D-05	0.190566D-05	0.426367D-06	-0.265576D-05	-0.336404D-05
17	0.237843D-05	-0.314499D-06	0.235277D-05	0.110500D-07	0.222123D-05
18	0.126620D-05	-0.251506D-05	-0.719580D-05	0.318858D-05	0.337478D-05
19	-0.252857D-06	0.300966D-07	-0.362052D-05	0.722086D-06	-0.133027D-05
20	-0.200829D-06	-0.144424D-06	-0.118382D-05	0.736533D-06	0.296962D-06
21	0.611410D-07	0.126804D-05	0.434402D-05	-0.164904D-05	0.293353D-05
22	-0.610743D-06	-0.186243D-06	-0.405319D-06	0.352253D-07	-0.129221D-05
23	-0.636655D-06	0.109159D-05	0.425256D-06	0.415755D-06	-0.169736D-06
24	0.139711D-05	-0.316878D-06	-0.255060D-05	-0.183652D-06	-0.842914D-06
25	-0.272720D-05	-0.115012D-05	-0.396317D-06	0.134804D-06	0.355169D-05
26	-0.159663D-05	-0.437511D-06	-0.162127D-05	0.205379D-05	-0.239065D-05

27	-0.544231D-05	0.180993D-06	-0.288022D-05	0.771292D-05	0.947329D-05
28	0.281154D-05	-0.685744D-06	0.584488D-06	-0.661520D-06	-0.315334D-06
29	0.114462D-05	-0.343219D-06	0.662875D-06	-0.336570D-06	0.360754D-06
30	0.131046D-06	-0.115108D-06	-0.641392D-05	0.181413D-05	0.162024D-05
31	-0.325257D-06	0.701267D-06	0.647244D-06	0.111801D-06	-0.339825D-05
32	-0.262871D-06	0.253804D-06	0.124549D-05	0.152323D-05	-0.594614D-05
33	0.281106D-05	0.295460D-05	0.899521D-06	0.340270D-06	0.461483D-05
34	-0.501569D-04	-0.361975D-04	0.138492D-04	-0.289004D-04	-0.951293D-04
35	-0.184473D-04	-0.357845D-05	-0.628552D-04	-0.576733D-05	0.780104D-04
36	0.105755D-04	0.687630D-06	0.795754D-05	0.174293D-05	0.559290D-05
37	0.755044D-05	-0.357276D-05	0.787148D-06	0.221980D-05	0.221149D-04
38	0.304715D-05	-0.802135D-06	0.252475D-05	-0.119748D-05	-0.453141D-05
39	0.631123D-04	0.731793D-05	0.285957D-04	0.255995D-04	0.326797D-04
40	0.714708D-04	-0.452067D-04	0.755049D-04	0.299641D-04	0.262083D-04
41	-0.656249D-04	0.867958D-05	-0.306502D-04	-0.794914D-04	0.895938D-04
42	0.684324D-05	-0.171187D-05	0.891961D-05	-0.522134D-05	0.856064D-05

ESTIMATED COVARIANCE MATRIX FOR PARAMETER ESTIMATES

	6	7	8	9	10
6	0.159097D-03				
7	0.147111D-04	0.113637D-02			
8	-0.169300D-04	-0.519239D-03	0.751088D-02		
9	0.661862D-05	0.731145D-04	0.466895D-04	0.621778D-03	
10	0.447360D-05	-0.103191D-03	-0.303258D-03	0.133235D-03	0.231090D-02
11	-0.287684D-06	0.266631D-04	-0.241751D-03	0.363344D-04	-0.248389D-03
12	0.598323D-05	-0.549501D-04	0.226588D-03	-0.864860D-04	0.132760D-03
13	-0.186349D-05	0.302217D-05	0.354013D-04	0.565575D-05	-0.446150D-05
14	-0.110071D-05	-0.217111D-05	-0.134730D-04	0.217762D-05	-0.166146D-05
15	0.533760D-06	0.118923D-05	-0.393903D-05	-0.191578D-05	-0.376659D-05
16	0.874712D-06	0.100741D-04	-0.184030D-04	0.238484D-05	0.228652D-04
17	0.119028D-05	0.564686D-05	-0.135748D-04	0.286480D-05	0.764003D-05
18	-0.844904D-05	0.774765D-05	0.786659D-04	0.232232D-04	-0.327712D-04
19	-0.953784D-06	-0.444667D-05	-0.650617D-05	0.189996D-05	-0.286594D-05
20	0.549754D-06	-0.859039D-06	-0.454772D-07	-0.149481D-05	-0.224479D-06
21	-0.167357D-06	0.424401D-05	-0.204377D-04	-0.480394D-06	0.126743D-04
22	0.699982D-06	-0.280941D-06	-0.206755D-05	-0.498132D-05	-0.432695D-05
23	0.591731D-05	-0.122057D-04	-0.374653D-04	0.462366D-05	-0.219357D-04
24	0.119562D-06	0.455118D-06	-0.274521D-04	-0.481839D-05	-0.888635D-07
25	-0.120865D-05	0.107105D-04	0.168672D-04	-0.191219D-05	0.260835D-04
26	-0.282489D-05	-0.343151D-05	-0.116012D-04	-0.272208D-05	-0.896016D-05
27	-0.763339D-05	0.756672D-05	-0.484187D-04	0.511294D-05	-0.401999D-04
28	0.631431D-05	0.994513D-06	-0.141634D-04	-0.100015D-04	-0.161750D-04

29	-0.442053D-06	-0.100214D-05	0.399358D-05	0.357225D-06	-0.165566D-05
30	-0.847478D-06	0.123977D-04	-0.345000D-05	0.405364D-05	-0.125133D-04
31	-0.258278D-05	-0.978871D-06	0.177815D-04	0.443962D-05	-0.272319D-05
32	0.867448D-06	0.351479D-05	0.140579D-04	0.118035D-04	0.160325D-04
33	0.227722D-05	0.222644D-04	0.389210D-04	-0.472781D-05	0.554492D-05
34	0.551248D-04	-0.845585D-04	0.378094D-03	0.233877D-04	-0.106517D-03
35	0.752659D-04	-0.745201D-04	-0.786902D-03	0.403442D-04	-0.282621D-03
36	0.139616D-04	0.262190D-04	0.842349D-04	0.957143D-05	0.754860D-05
37	0.144420D-05	0.591610D-04	0.409939D-06	0.114770D-04	0.675244D-06
38	-0.139282D-05	-0.120001D-04	0.860331D-04	0.791273D-07	0.545885D-05
39	-0.162206D-03	-0.212642D-04	0.234836D-03	-0.356067D-04	0.860694D-04
40	0.928749D-04	0.160448D-03	-0.297286D-04	0.225675D-03	0.362081D-03
41	-0.592667D-05	0.694818D-03	0.553473D-03	-0.530326D-04	-0.675805D-03
42	0.925171D-06	0.136104D-03	0.106923D-02	0.119305D-05	-0.123636D-04

ESTIMATED COVARIANCE MATRIX FOR PARAMETER ESTIMATES

	11	12	13	14	15
11	0.649403D-03				
12	0.438172D-04	0.756573D-03			
13	0.113471D-04	-0.390401D-05	0.100574D-03		
14	0.387696D-05	-0.243870D-05	0.104525D-04	0.103680D-04	
15	0.619117D-06	-0.127442D-05	0.440537D-06	0.197778D-05	0.319128D-05
16	-0.582476D-05	-0.169281D-05	-0.158118D-04	-0.344695D-05	-0.486830D-06
17	-0.199409D-05	-0.461963D-06	-0.143519D-05	-0.197098D-05	-0.144204D-05
18	-0.214895D-05	-0.141434D-04	-0.152721D-05	-0.438176D-06	0.300765D-06
19	0.384942D-05	-0.320620D-05	0.326620D-05	0.257574D-05	0.438089D-06
20	-0.111119D-05	0.227005D-05	-0.558227D-06	0.518323D-06	0.824246D-06
21	0.150019D-05	0.898004D-07	0.136539D-05	-0.465619D-06	-0.323423D-06
22	-0.274846D-05	0.847172D-05	-0.315592D-06	-0.377755D-06	0.271254D-08
23	0.185634D-04	-0.560123D-06	0.132194D-04	0.629707D-05	0.131111D-05
24	0.314546D-05	-0.107129D-05	0.168452D-05	0.226957D-05	0.192678D-05
25	-0.116203D-04	-0.472042D-05	-0.301977D-05	-0.265591D-05	-0.113179D-05
26	0.805256D-05	0.301799D-05	-0.270752D-06	0.394769D-06	0.303543D-06
27	0.346999D-04	-0.824826D-05	0.214210D-05	0.260825D-05	0.422167D-06
28	-0.542253D-05	-0.603467D-05	-0.870745D-05	-0.529695D-05	-0.154981D-05
29	-0.234475D-05	0.254876D-06	-0.511309D-06	-0.142145D-05	-0.195565D-05
30	-0.252784D-05	-0.393925D-05	0.117195D-05	0.318582D-06	0.328542D-07
31	-0.156314D-05	-0.611671D-06	-0.161751D-05	-0.235350D-06	-0.372045D-06
32	0.379159D-05	-0.427406D-05	0.167067D-06	-0.197475D-05	-0.127000D-05
33	-0.638025D-05	-0.723987D-07	0.336361D-05	0.248436D-05	0.537111D-06
34	-0.895078D-04	0.399648D-04	0.301747D-04	-0.867784D-05	0.974836D-05
35	-0.761229D-04	0.147959D-03	0.144337D-03	0.116368D-04	-0.381594D-05

36	0.168125D-04	-0.127808D-04	0.577202D-05	0.234184D-05	0.100888D-05
37	0.260907D-04	0.293157D-05	0.638698D-05	0.115854D-05	0.261200D-05
38	0.558848D-05	-0.441818D-05	-0.250894D-05	-0.750235D-06	-0.440692D-06
39	0.277805D-04	-0.982712D-04	-0.928312D-04	0.152161D-04	-0.923182D-05
40	-0.532866D-04	-0.402027D-04	0.962812D-07	0.151931D-04	0.377091D-05
41	-0.101980D-03	0.991491D-04	-0.306529D-04	-0.111714D-04	-0.109583D-04
42	0.236772D-04	-0.930998D-05	0.195497D-04	-0.769977D-06	0.532461D-05

ESTIMATED COVARIANCE MATRIX FOR PARAMETER ESTIMATES

	16	17	18	19	20
16	0.105695D-03				
17	0.110289D-04	0.187708D-04			
18	-0.369038D-04	-0.131481D-04	0.475333D-03		
19	-0.574518D-05	-0.728626D-06	-0.236230D-06	0.211002D-04	
20	-0.758194D-06	-0.678403D-06	0.147940D-07	0.204817D-05	0.373963D-05
21	0.600202D-05	0.600208D-05	-0.202867D-04	-0.367669D-05	-0.175588D-05
22	-0.187824D-05	0.296044D-07	0.297412D-05	0.823431D-06	0.183088D-05
23	-0.837103D-05	-0.134098D-05	-0.687443D-05	0.908284D-05	0.110489D-05
24	-0.127740D-05	-0.192354D-05	0.315858D-05	0.215537D-05	0.181381D-05
25	0.139951D-04	0.987417D-05	-0.177998D-04	-0.415229D-05	-0.109456D-05
26	-0.415642D-06	0.353088D-07	0.108477D-05	0.228915D-05	0.230117D-05
27	-0.119059D-05	-0.278889D-05	-0.310386D-06	0.132760D-05	0.229358D-05
28	0.200709D-04	0.165650D-05	-0.141874D-05	-0.677312D-05	-0.168160D-05
29	0.317159D-05	0.374112D-05	-0.770272D-05	-0.157172D-05	-0.142962D-05
30	-0.119144D-04	-0.100380D-04	0.624701D-04	0.175638D-05	0.490262D-06
31	0.659495D-06	0.101655D-05	-0.387504D-05	-0.230501D-05	-0.203898D-05
32	0.306063D-05	0.230527D-05	-0.208348D-05	-0.278087D-05	-0.173627D-05
33	-0.272658D-05	-0.231570D-05	0.222853D-04	0.333777D-05	0.186872D-05
34	-0.422025D-04	-0.156983D-04	-0.786013D-05	-0.143857D-04	0.833947D-05
35	-0.499590D-04	0.141645D-04	-0.861108D-04	-0.129444D-04	-0.110995D-05
36	0.328145D-05	-0.106693D-05	0.112064D-04	0.577235D-05	-0.175772D-05
37	0.372073D-05	0.147909D-05	-0.131083D-04	-0.288753D-05	0.129310D-05
38	0.129700D-05	-0.761496D-06	0.707680D-05	-0.597038D-07	-0.236081D-06
39	-0.419593D-04	0.498939D-04	-0.113525D-03	0.736222D-05	0.172463D-05
40	-0.233754D-04	-0.252458D-04	0.139287D-03	-0.176018D-05	0.405224D-05
41	0.177922D-03	0.690945D-04	-0.101292D-03	0.934580D-05	-0.204697D-04
42	-0.429792D-05	-0.590624D-05	0.190138D-04	-0.257616D-05	0.557262D-06

ESTIMATED COVARIANCE MATRIX FOR PARAMETER ESTIMATES

	21	22	23	24	25

21	0.458436D-04				
22	-0.330044D-05	0.170144D-04			
23	0.110388D-05	-0.210349D-05	0.106924D-03		
24	-0.466840D-06	0.365379D-06	0.110371D-04	0.180382D-04	
25	0.242270D-04	-0.234490D-05	-0.193859D-04	-0.790458D-05	0.196916D-03
26	-0.283642D-05	0.781312D-05	0.355459D-05	0.457684D-05	-0.131372D-04
27	-0.201778D-05	0.186976D-05	0.204426D-04	0.187654D-04	-0.225937D-04
28	0.232128D-07	-0.689167D-06	-0.146955D-04	-0.346030D-05	0.659038D-05
29	0.196373D-05	-0.359116D-06	-0.164920D-05	-0.363940D-05	0.209071D-05
30	-0.145602D-04	-0.179364D-06	0.265358D-05	0.183067D-05	-0.350349D-04
31	0.719298D-05	-0.501977D-05	-0.129441D-05	-0.159020D-05	0.525034D-05
32	-0.384654D-06	-0.236441D-05	-0.181576D-04	-0.118768D-04	0.213787D-04
33	-0.287212D-05	0.183331D-05	0.465109D-05	0.457505D-05	-0.127064D-04
34	-0.206906D-04	0.565016D-05	-0.118435D-04	0.161091D-04	-0.644786D-05
35	0.135624D-04	0.483789D-04	0.165749D-04	-0.885117D-05	0.329565D-04
36	-0.285392D-05	-0.717848D-06	0.218229D-06	-0.302246D-05	0.116619D-04
37	0.440634D-05	-0.133222D-05	0.582675D-05	0.229604D-05	0.362106D-05
38	-0.225222D-05	0.191423D-06	-0.427673D-06	-0.131804D-05	0.433801D-05
39	-0.836329D-05	-0.160629D-04	-0.227216D-04	0.439022D-06	-0.321689D-04
40	-0.178854D-04	-0.547235D-05	-0.971060D-04	0.418885D-04	0.114366D-03
41	0.178099D-03	0.124087D-04	-0.107239D-03	0.148765D-04	0.646812D-04
42	-0.735854D-05	0.962635D-06	0.478357D-06	-0.290690D-06	0.154865D-05

ESTIMATED COVARIANCE MATRIX FOR PARAMETER ESTIMATES

	26	27	28	29	30
26	0.429073D-04				
27	0.309082D-04	0.381411D-03			
28	-0.244499D-05	-0.361976D-05	0.836208D-04		
29	0.364815D-06	-0.261184D-05	0.108049D-04	0.172511D-04	
30	0.171814D-05	0.645759D-05	-0.101494D-04	-0.925366D-05	0.176384D-03
31	-0.759520D-05	-0.694752D-05	0.587960D-05	0.451893D-05	-0.107452D-04
32	-0.137044D-04	-0.519626D-04	0.170987D-04	0.107471D-04	-0.111699D-04
33	0.358190D-06	-0.160437D-05	-0.280994D-04	-0.165662D-04	0.516838D-04
34	0.440951D-05	0.430705D-04	-0.671801D-04	-0.323214D-04	0.690480D-04
35	-0.563659D-05	0.614844D-04	0.205998D-04	0.957378D-05	0.341783D-04
36	-0.345625D-05	-0.195904D-04	-0.136696D-04	0.183726D-05	0.119387D-05
37	0.387662D-06	0.137986D-04	0.127373D-05	0.265179D-05	-0.202521D-05
38	-0.789682D-06	0.189550D-05	0.750429D-06	0.117449D-05	-0.512112D-05
39	-0.126075D-04	-0.187320D-03	-0.635806D-04	-0.189193D-04	0.157089D-04
40	-0.558518D-04	0.507137D-04	-0.418224D-04	0.129709D-04	0.271241D-04
41	0.446933D-04	-0.117524D-04	0.945241D-04	0.750360D-04	-0.305802D-04
42	0.236552D-05	-0.135803D-04	0.700610D-05	0.268076D-05	0.524864D-06

ESTIMATED COVARIANCE MATRIX FOR PARAMETER ESTIMATES

	31	32	33	34	35
31	0.349419D-04				
32	0.136876D-04	0.158417D-03			
33	-0.203419D-04	-0.524502D-04	0.259598D-03		
34	-0.263082D-04	-0.682052D-04	0.225060D-04	0.240404D-01	
35	-0.251741D-04	-0.125081D-04	-0.100896D-03	-0.154445D-02	0.418731D-01
36	-0.478218D-05	0.120179D-04	0.572839D-05	0.971567D-04	0.216609D-03
37	0.235646D-05	0.185329D-05	0.526547D-05	-0.136980D-03	0.767372D-04
38	0.178709D-05	0.229105D-05	-0.404966D-05	0.314829D-04	0.304299D-04
39	0.298318D-04	0.108147D-06	0.802925D-04	0.707416D-03	-0.329513D-03
40	0.637716D-05	-0.167134D-04	-0.346625D-04	0.149440D-02	-0.329287D-04
41	-0.132518D-03	0.138840D-03	0.220156D-03	-0.166102D-02	-0.139544D-02
42	0.730318D-05	0.636106D-05	-0.888519D-05	-0.153816D-03	0.511760D-04

ESTIMATED COVARIANCE MATRIX FOR PARAMETER ESTIMATES

	36	37	38	39	40
36	0.869677D-03				
37	0.756770D-04	0.502045D-03			
38	0.198257D-04	-0.147942D-05	0.599294D-04		
39	-0.142626D-04	-0.120295D-03	-0.605942D-04	0.700192D-01	
40	-0.561082D-04	-0.796275D-04	0.546837D-04	-0.591139D-02	0.585098D-01
41	-0.150318D-03	-0.217174D-04	-0.115225D-03	0.782589D-02	-0.102352D-01
42	0.117906D-03	0.581286D-03	0.170840D-04	-0.516372D-03	-0.109062D-03

ESTIMATED COVARIANCE MATRIX FOR PARAMETER ESTIMATES

	41	42
41	0.218445D+00	
42	-0.326025D-03	0.172045D-02

ESTIMATED CORRELATION MATRIX FOR PARAMETER ESTIMATES

	1	2	3	4	5
1	1.000				
2	0.194	1.000			
3	-0.102	-0.086	1.000		

4	0.071	0.129	-0.089	1.000	
5	0.062	0.169	-0.078	0.173	1.000
6	-0.064	-0.199	0.164	-0.145	-0.142
7	0.053	0.019	0.030	-0.024	0.010
8	0.019	0.039	0.001	-0.023	-0.058
9	0.018	-0.027	0.025	-0.020	-0.021
10	0.023	-0.030	-0.014	0.012	0.017
11	-0.030	0.010	0.019	0.028	0.061
12	-0.010	-0.006	0.035	0.007	-0.005
13	-0.006	-0.002	-0.027	0.013	-0.026
14	0.032	0.023	-0.037	0.018	-0.057
15	-0.035	0.024	-0.032	0.014	-0.034
16	0.033	0.044	0.003	-0.041	-0.024
17	0.057	-0.017	0.039	0.000	0.037
18	0.006	-0.027	-0.023	0.023	0.011
19	-0.006	0.002	-0.056	0.025	-0.021
20	-0.011	-0.018	-0.044	0.061	0.011
21	0.001	0.044	0.046	-0.039	0.031
22	-0.015	-0.011	-0.007	0.001	-0.023
23	-0.006	0.025	0.003	0.006	-0.001
24	0.034	-0.018	-0.043	-0.007	-0.014
25	-0.020	-0.019	-0.002	0.002	0.018
26	-0.025	-0.016	-0.018	0.050	-0.026
27	-0.029	0.002	-0.010	0.063	0.035
28	0.032	-0.018	0.005	-0.012	-0.003
29	0.028	-0.019	0.011	-0.013	0.006
30	0.001	-0.002	-0.034	0.022	0.009
31	-0.006	0.028	0.008	0.003	-0.042
32	-0.002	0.005	0.007	0.019	-0.034
33	0.018	0.043	0.004	0.003	0.021
34	-0.033	-0.055	0.006	-0.030	-0.045
35	-0.009	-0.004	-0.022	-0.005	0.028
36	0.037	0.005	0.019	0.009	0.014
37	0.035	-0.038	0.002	0.016	0.072
38	0.041	-0.024	0.023	-0.025	-0.042
39	0.025	0.007	0.008	0.015	0.009
40	0.031	-0.044	0.022	0.020	0.008
41	-0.015	0.004	-0.005	-0.027	0.014
42	0.017	-0.010	0.015	-0.020	0.015

ESTIMATED CORRELATION MATRIX FOR PARAMETER ESTIMATES

	6	7	8	9	10
	________	________	________	________	________

6	1.000				
7	0.035	1.000			
8	-0.015	-0.178	1.000		
9	0.021	0.087	0.022	1.000	
10	0.007	-0.064	-0.073	0.111	1.000
11	-0.001	0.031	-0.109	0.057	-0.203
12	0.017	-0.059	0.095	-0.126	0.100
13	-0.015	0.009	0.041	0.023	-0.009
14	-0.027	-0.020	-0.048	0.027	-0.011
15	0.024	0.020	-0.025	-0.043	-0.044
16	0.007	0.029	-0.021	0.009	0.046
17	0.022	0.039	-0.036	0.027	0.037
18	-0.031	0.011	0.042	0.043	-0.031
19	-0.016	-0.029	-0.016	0.017	-0.013
20	0.023	-0.013	0.000	-0.031	-0.002
21	-0.002	0.019	-0.035	-0.003	0.039
22	0.013	-0.002	-0.006	-0.048	-0.022
23	0.045	-0.035	-0.042	0.018	-0.044
24	0.002	0.003	-0.075	-0.045	0.000
25	-0.007	0.023	0.014	-0.005	0.039
26	-0.034	-0.016	-0.020	-0.017	-0.028
27	-0.031	0.011	-0.029	0.010	-0.043
28	0.055	0.003	-0.018	-0.044	-0.037
29	-0.008	-0.007	0.011	0.003	-0.008
30	-0.005	0.028	-0.003	0.012	-0.020
31	-0.035	-0.005	0.035	0.030	-0.010
32	0.005	0.008	0.013	0.038	0.026
33	0.011	0.041	0.028	-0.012	0.007
34	0.028	-0.016	0.028	0.006	-0.014
35	0.029	-0.011	-0.044	0.008	-0.029
36	0.038	0.026	0.033	0.013	0.005
37	0.005	0.078	0.000	0.021	0.001
38	-0.014	-0.046	0.128	0.000	0.015
39	-0.049	-0.002	0.010	-0.005	0.007
40	0.030	0.020	-0.001	0.037	0.031
41	-0.001	0.044	0.014	-0.005	-0.030
42	0.002	0.097	0.297	0.001	-0.006

ESTIMATED CORRELATION MATRIX FOR PARAMETER ESTIMATES

	11	12	13	14	15
11	1.000				
12	0.063	1.000			

13	0.044	-0.014	1.000		
14	0.047	-0.028	0.324	1.000	
15	0.014	-0.026	0.025	0.344	1.000
16	-0.022	-0.006	-0.153	-0.104	-0.027
17	-0.018	-0.004	-0.033	-0.141	-0.186
18	-0.004	-0.024	-0.007	-0.006	0.008
19	0.033	-0.025	0.071	0.174	0.053
20	-0.023	0.043	-0.029	0.083	0.239
21	0.009	0.000	0.020	-0.021	-0.027
22	-0.026	0.075	-0.008	-0.028	0.000
23	0.070	-0.002	0.127	0.189	0.071
24	0.029	-0.009	0.040	0.166	0.254
25	-0.032	-0.012	-0.021	-0.059	-0.045
26	0.048	0.017	-0.004	0.019	0.026
27	0.070	-0.015	0.011	0.041	0.012
28	-0.023	-0.024	-0.095	-0.180	-0.095
29	-0.022	0.002	-0.012	-0.106	-0.264
30	-0.007	-0.011	0.009	0.007	0.001
31	-0.010	-0.004	-0.027	-0.012	-0.035
32	0.012	-0.012	0.001	-0.049	-0.056
33	-0.016	0.000	0.021	0.048	0.019
34	-0.023	0.009	0.019	-0.017	0.035
35	-0.015	0.026	0.070	0.018	-0.010
36	0.022	-0.016	0.020	0.025	0.019
37	0.046	0.005	0.028	0.016	0.065
38	0.028	-0.021	-0.032	-0.030	-0.032
39	0.004	-0.014	-0.035	0.018	-0.020
40	-0.009	-0.006	0.000	0.020	0.009
41	-0.009	0.008	-0.007	-0.007	-0.013
42	0.022	-0.008	0.047	-0.006	0.072

ESTIMATED CORRELATION MATRIX FOR PARAMETER ESTIMATES

	16	17	18	19	20
16	1.000				
17	0.248	1.000			
18	-0.165	-0.139	1.000		
19	-0.122	-0.037	-0.002	1.000	
20	-0.038	-0.081	0.000	0.231	1.000
21	0.086	0.205	-0.137	-0.118	-0.134
22	-0.044	0.002	0.033	0.043	0.230
23	-0.079	-0.030	-0.030	0.191	0.055
24	-0.029	-0.105	0.034	0.110	0.221

25	0.097	0.162	-0.058	-0.064	-0.040
26	-0.006	0.001	0.008	0.076	0.182
27	-0.006	-0.033	-0.001	0.015	0.061
28	0.213	0.042	-0.007	-0.161	-0.095
29	0.074	0.208	-0.085	-0.082	-0.178
30	-0.087	-0.174	0.216	0.029	0.019
31	0.011	0.040	-0.030	-0.085	-0.178
32	0.024	0.042	-0.008	-0.048	-0.071
33	-0.016	-0.033	0.063	0.045	0.060
34	-0.026	-0.023	-0.002	-0.020	0.028
35	-0.024	0.016	-0.019	-0.014	-0.003
36	0.011	-0.008	0.017	0.043	-0.031
37	0.016	0.015	-0.027	-0.028	0.030
38	0.016	-0.023	0.042	-0.002	-0.016
39	-0.015	0.044	-0.020	0.006	0.003
40	-0.009	-0.024	0.026	-0.002	0.009
41	0.037	0.034	-0.010	0.004	-0.023
42	-0.010	-0.033	0.021	-0.014	0.007

ESTIMATED CORRELATION MATRIX FOR PARAMETER ESTIMATES

	21	22	23	24	25
21	1.000				
22	-0.118	1.000			
23	0.016	-0.049	1.000		
24	-0.016	0.021	0.251	1.000	
25	0.255	-0.041	-0.134	-0.133	1.000
26	-0.064	0.289	0.052	0.165	-0.143
27	-0.015	0.023	0.101	0.226	-0.082
28	0.000	-0.018	-0.155	-0.089	0.051
29	0.070	-0.021	-0.038	-0.206	0.036
30	-0.162	-0.003	0.019	0.032	-0.188
31	0.180	-0.206	-0.021	-0.063	0.063
32	-0.005	-0.046	-0.140	-0.222	0.121
33	-0.026	0.028	0.028	0.067	-0.056
34	-0.020	0.009	-0.007	0.024	-0.003
35	0.010	0.057	0.008	-0.010	0.011
36	-0.014	-0.006	0.001	-0.024	0.028
37	0.029	-0.014	0.025	0.024	0.012
38	-0.043	0.006	-0.005	-0.040	0.040
39	-0.005	-0.015	-0.008	0.000	-0.009
40	-0.011	-0.005	-0.039	0.041	0.034
41	0.056	0.006	-0.022	0.007	0.010

42	-0.026	0.006	0.001	-0.002	0.003

ESTIMATED CORRELATION MATRIX FOR PARAMETER ESTIMATES

	26	27	28	29	30
26	1.000				
27	0.242	1.000			
28	-0.041	-0.020	1.000		
29	0.013	-0.032	0.284	1.000	
30	0.020	0.025	-0.084	-0.168	1.000
31	-0.196	-0.060	0.109	0.184	-0.137
32	-0.166	-0.211	0.149	0.206	-0.067
33	0.003	-0.005	-0.191	-0.248	0.242
34	0.004	0.014	-0.047	-0.050	0.034
35	-0.004	0.015	0.011	0.011	0.013
36	-0.018	-0.034	-0.051	0.015	0.003
37	0.003	0.032	0.006	0.028	-0.007
38	-0.016	0.013	0.011	0.037	-0.050
39	-0.007	-0.036	-0.026	-0.017	0.004
40	-0.035	0.011	-0.019	0.013	0.008
41	0.015	-0.001	0.022	0.039	-0.005
42	0.009	-0.017	0.018	0.016	0.001

ESTIMATED CORRELATION MATRIX FOR PARAMETER ESTIMATES

	31	32	33	34	35
31	1.000				
32	0.184	1.000			
33	-0.214	-0.259	1.000		
34	-0.029	-0.035	0.009	1.000	
35	-0.021	-0.005	-0.031	-0.049	1.000
36	-0.027	0.032	0.012	0.021	0.036
37	0.018	0.007	0.015	-0.039	0.017
38	0.039	0.024	-0.032	0.026	0.019
39	0.019	0.000	0.019	0.017	-0.006
40	0.004	-0.005	-0.009	0.040	-0.001
41	-0.048	0.024	0.029	-0.023	-0.015
42	0.030	0.012	-0.013	-0.024	0.006

ESTIMATED CORRELATION MATRIX FOR PARAMETER ESTIMATES

	36	37	38	39	40

36	1.000				
37	0.115	1.000			
38	0.087	-0.009	1.000		
39	-0.002	-0.020	-0.030	1.000	
40	-0.008	-0.015	0.029	-0.092	1.000
41	-0.011	-0.002	-0.032	0.063	-0.091
42	0.096	0.625	0.053	-0.047	-0.011

ESTIMATED CORRELATION MATRIX FOR PARAMETER ESTIMATES

	41	42
41	1.000	
42	-0.017	1.000

TECHNICAL 8 OUTPUT

TECHNICAL 8 OUTPUT FOR BAYES ESTIMATION

CHAIN	BSEED
1	0
2	285380
3	253358

ITERATION	POTENTIAL SCALE REDUCTION	PARAMETER WITH HIGHEST PSR
100	1.013	9
200	1.006	34
300	1.003	30
400	1.004	36
500	1.004	42
600	1.004	23
700	1.002	9
800	1.003	8
900	1.003	8
1000	1.002	8

Reduced distress, main effects

SUMMARY OF DATA

Number of clusters 58

Size (s)	Cluster ID with Size s
1	166
2	74 205
3	106 182 165
4	93 170 22 192 4
5	65 71 50 84
6	80 72
7	40 107 61 75
8	169 30 173 228
9	144 81 83 181
10	69 145 36 68
11	108 116 174 154
12	206 162
13	226 114
14	2
15	152 98
18	156
19	202
21	92 6
24	178
25	142 122
32	24
37	204
47	209
53	125
67	217
174	110
210	100

COVARIANCE COVERAGE OF DATA

Minimum covariance coverage value 0.100

Number of missing data patterns 5

PROPORTION OF DATA PRESENT

Covariance Coverage

	KESCAT16	PHY15	KES15	CHOICE15	BULLY15
KESCAT16	1.000				
PHY15	1.000	1.000			
KES15	1.000	1.000	1.000		
CHOICE15	0.990	0.990	0.990	0.990	
BULLY15	0.990	0.990	0.990	0.989	0.990
PRESS15	0.990	0.990	0.990	0.989	0.989
DUTIES15	0.991	0.991	0.991	0.990	0.990
PRAFAC	1.000	1.000	1.000	0.990	0.990

Covariance Coverage

	PRESS15	DUTIES15	PRAFAC
PRESS15	0.990		
DUTIES15	0.990	0.991	
PRAFAC	0.990	0.991	1.000

UNIVARIATE PROPORTIONS AND COUNTS FOR CATEGORICAL VARIABLES

KESCAT16

Category 1	0.347	386.000
Category 2	0.653	727.000

UNIVARIATE SAMPLE STATISTICS

UNIVARIATE HIGHER-ORDER MOMENT DESCRIPTIVE STATISTICS

Variable/ Sample Size	Mean/ Variance	Skewness/ Kurtosis	Minimum/ Maximum	% with Min/Max	Percentiles 20%/60%	40%/80%	Median
PHY15	0.000	0.390	-1.412	11.50%	-0.412	-0.412	-0.412
1113.000	0.702	0.082	2.588	1.08%	0.588	0.588	
KES15	0.000	1.366	-0.556	20.75%	-0.556	-0.222	-0.222

```
        1113.000       0.313       1.871       2.278    0.63%      -0.056      0.444
     CHOICE15          0.000      -0.116      -1.814   15.06%      -0.814      0.186      0.186
        1102.000       1.193      -0.747       2.186    4.45%       0.186      1.186
     BULLY15           0.000       1.887      -0.426   71.96%      -0.426     -0.426     -0.426
        1102.000       0.602       3.132       3.574    0.45%      -0.426      0.574
     PRESS15           0.000       0.096      -1.915    9.89%      -0.915      0.085      0.085
        1102.000       1.160      -0.512       2.085    8.35%       0.085      1.085
     DUTIES15          0.000      -0.677      -2.672    3.54%      -0.672      0.328      0.328
        1103.000       1.031       0.049       1.328   20.22%       0.328      1.328
     PRAFAC           -0.185       0.245      -2.338    1.72%      -1.448     -0.527     -0.318
          58.000       1.345      -0.636       2.338    1.72%       0.080      0.752


THE MODEL ESTIMATION TERMINATED NORMALLY

     USE THE FBITERATIONS OPTION TO INCREASE THE NUMBER OF ITERATIONS BY A FACTOR
     OF AT LEAST TWO TO CHECK CONVERGENCE AND THAT THE PSR VALUE DOES NOT INCREASE.



MODEL FIT INFORMATION

Number of Free Parameters                              36

Bayesian Posterior Predictive Checking using Chi-Square

          95% Confidence Interval for the Difference Between
          the Observed and the Replicated Chi-Square Values

                            -27.722            29.442

          Posterior Predictive P-Value              0.425



MODEL RESULTS

                                Posterior  One-Tailed         95% C.I.
                    Estimate       S.D.      P-Value   Lower 2.5%  Upper 2.5%  Significance

Within Level

 KESCAT16   ON
    PHY15              0.161       0.052      0.001       0.058       0.261      *
```

CHOICE15	0.028	0.038	0.239	-0.047	0.100	
BULLY15	0.112	0.061	0.036	-0.011	0.225	
DUTIES15	-0.082	0.045	0.035	-0.169	0.006	
PRESS15	-0.044	0.041	0.133	-0.126	0.036	
KES15	0.793	0.095	0.000	0.612	0.992	*
KES15 WITH						
PHY15	0.107	0.014	0.000	0.078	0.132	*
CHOICE15	-0.097	0.018	0.000	-0.131	-0.059	*
BULLY15	0.072	0.014	0.000	0.047	0.099	*
PRESS15	0.061	0.019	0.001	0.024	0.100	*
DUTIES15	-0.073	0.018	0.000	-0.109	-0.038	*
PHY15 WITH						
CHOICE15	-0.097	0.027	0.001	-0.150	-0.044	*
BULLY15	0.040	0.020	0.027	-0.001	0.078	
PRESS15	0.118	0.028	0.000	0.067	0.177	*
DUTIES15	-0.059	0.026	0.011	-0.108	-0.009	*
CHOICE15 WITH						
BULLY15	-0.139	0.025	0.000	-0.189	-0.091	*
PRESS15	-0.195	0.035	0.000	-0.267	-0.129	*
DUTIES15	0.151	0.033	0.000	0.085	0.214	*
BULLY15 WITH						
PRESS15	0.200	0.026	0.000	0.150	0.253	*
DUTIES15	-0.176	0.025	0.000	-0.225	-0.129	*
PRESS15 WITH						
DUTIES15	-0.287	0.036	0.000	-0.359	-0.216	*
Means						
PHY15	-0.001	0.025	0.485	-0.046	0.047	
KES15	0.000	0.017	0.494	-0.034	0.034	
CHOICE15	0.000	0.032	0.500	-0.062	0.063	
BULLY15	0.000	0.023	0.496	-0.047	0.045	
PRESS15	-0.003	0.032	0.464	-0.063	0.061	
DUTIES15	0.000	0.032	0.497	-0.062	0.065	
Variances						
PHY15	0.702	0.029	0.000	0.648	0.763	*
KES15	0.314	0.013	0.000	0.288	0.341	*
CHOICE15	1.192	0.051	0.000	1.098	1.298	*
BULLY15	0.604	0.026	0.000	0.553	0.657	*

PRESS15	1.161	0.051	0.000	1.064	1.261	*
DUTIES15	1.033	0.045	0.000	0.943	1.126	*

Between Level

KESCAT16 ON						
PRAFAC	-0.117	0.050	0.011	-0.222	-0.021	*
Thresholds						
KESCAT16$1	-0.452	0.054	0.000	-0.556	-0.339	*
Residual Variances						
KESCAT16	0.018	0.030	0.000	0.001	0.118	*

STANDARDIZED MODEL RESULTS

STDYX Standardization

	Estimate	Posterior S.D.	One-Tailed P-Value	95% C.I. Lower 2.5%	Upper 2.5%	Significance
Within Level						
KESCAT16 ON						
PHY15	0.119	0.038	0.001	0.044	0.192	*
CHOICE15	0.026	0.037	0.239	-0.047	0.096	
BULLY15	0.077	0.041	0.036	-0.008	0.153	
DUTIES15	-0.073	0.040	0.035	-0.152	0.005	
PRESS15	-0.042	0.039	0.133	-0.118	0.034	
KES15	0.392	0.041	0.000	0.313	0.473	*
KES15 WITH						
PHY15	0.227	0.027	0.000	0.172	0.276	*
CHOICE15	-0.158	0.029	0.000	-0.211	-0.099	*
BULLY15	0.167	0.030	0.000	0.108	0.224	*
PRESS15	0.101	0.031	0.001	0.040	0.162	*
DUTIES15	-0.128	0.031	0.000	-0.187	-0.068	*
PHY15 WITH						
CHOICE15	-0.106	0.029	0.001	-0.163	-0.048	*
BULLY15	0.061	0.030	0.027	-0.001	0.118	
PRESS15	0.132	0.030	0.000	0.075	0.194	*
DUTIES15	-0.069	0.030	0.011	-0.126	-0.010	*

```
 CHOICE15 WITH
    BULLY15          -0.164       0.029      0.000      -0.218      -0.108      *
    PRESS15          -0.166       0.029      0.000      -0.224      -0.109      *
    DUTIES15          0.136       0.029      0.000       0.078       0.191      *

 BULLY15  WITH
    PRESS15           0.240       0.028      0.000       0.183       0.294      *
    DUTIES15         -0.223       0.029      0.000      -0.279      -0.165      *

 PRESS15  WITH
    DUTIES15         -0.262       0.029      0.000      -0.318      -0.202      *

 Means
    PHY15            -0.001       0.029      0.485      -0.055       0.056
    KES15             0.000       0.031      0.494      -0.061       0.062
    CHOICE15          0.000       0.029      0.500      -0.057       0.057
    BULLY15           0.001       0.030      0.496      -0.061       0.058
    PRESS15          -0.002       0.030      0.464      -0.059       0.056
    DUTIES15          0.000       0.031      0.497      -0.062       0.063

 Variances
    PHY15             1.000       0.000      0.000       1.000       1.000
    KES15             1.000       0.000      0.000       1.000       1.000
    CHOICE15          1.000       0.000      0.000       1.000       1.000
    BULLY15           1.000       0.000      0.000       1.000       1.000
    PRESS15           1.000       0.000      0.000       1.000       1.000
    DUTIES15          1.000       0.000      0.000       1.000       1.000

Between Level

 KESCAT16   ON
    PRAFAC           -0.697       0.234      0.011      -0.984      -0.127      *

 Thresholds
    KESCAT16$1       -2.249       1.117      0.000      -5.438      -1.079      *

 Residual Variances
    KESCAT16          0.515       0.277      0.000       0.032       0.979      *


R-SQUARE

Within Level
```

```
                               Posterior  One-Tailed         95% C.I.
    Variable        Estimate       S.D.      P-Value   Lower 2.5%  Upper 2.5%

    KESCAT16           0.219       0.035      0.000       0.152       0.289

Between Level

                               Posterior  One-Tailed         95% C.I.
    Variable        Estimate       S.D.      P-Value   Lower 2.5%  Upper 2.5%

    KESCAT16           0.485       0.277      0.000       0.021       0.968


TECHNICAL 1 OUTPUT


     PARAMETER SPECIFICATION FOR WITHIN


           TAU
              KESCAT16
              ________
                    0


           NU
              KESCAT16      PHY15         KES15         CHOICE15      BULLY15
              ________      ________      ________      ________      ________
                    0             0             0             0             0


           NU
              PRESS15       DUTIES15
              ________      ________
                    0             0


           LAMBDA
              KESCAT16      PHY15         KES15         CHOICE15      BULLY15
              ________      ________      ________      ________      ________
 KESCAT16           0             0             0             0             0
 PHY15              0             0             0             0             0
 KES15              0             0             0             0             0
```

CHOICE15	0	0	0	0	0
BULLY15	0	0	0	0	0
PRESS15	0	0	0	0	0
DUTIES15	0	0	0	0	0

LAMBDA

	PRESS15	DUTIES15
KESCAT16	0	0
PHY15	0	0
KES15	0	0
CHOICE15	0	0
BULLY15	0	0
PRESS15	0	0
DUTIES15	0	0

THETA

	KESCAT16	PHY15	KES15	CHOICE15	BULLY15
KESCAT16	0				
PHY15	0	0			
KES15	0	0	0		
CHOICE15	0	0	0	0	
BULLY15	0	0	0	0	0
PRESS15	0	0	0	0	0
DUTIES15	0	0	0	0	0

THETA

	PRESS15	DUTIES15
PRESS15	0	
DUTIES15	0	0

ALPHA

KESCAT16	PHY15	KES15	CHOICE15	BULLY15
0	1	2	3	4

ALPHA

	PRESS15	DUTIES15
	5	6

BETA

	KESCAT16	PHY15	KES15	CHOICE15	BULLY15
KESCAT16	0	7	8	9	10
PHY15	0	0	0	0	0
KES15	0	0	0	0	0
CHOICE15	0	0	0	0	0
BULLY15	0	0	0	0	0
PRESS15	0	0	0	0	0
DUTIES15	0	0	0	0	0

BETA

	PRESS15	DUTIES15
KESCAT16	11	12
PHY15	0	0
KES15	0	0
CHOICE15	0	0
BULLY15	0	0
PRESS15	0	0
DUTIES15	0	0

PSI

	KESCAT16	PHY15	KES15	CHOICE15	BULLY15
KESCAT16	0				
PHY15	0	13			
KES15	0	14	15		
CHOICE15	0	16	17	18	
BULLY15	0	19	20	21	22
PRESS15	0	23	24	25	26
DUTIES15	0	28	29	30	31

PSI

	PRESS15	DUTIES15

```
PRESS15            27
DUTIES15           32            33


    PARAMETER SPECIFICATION FOR BETWEEN


          TAU
             KESCAT16
             ________
                  36


          NU
             KESCAT16      PRAFAC
             ________      ________
                   0             0


          LAMBDA
             KESCAT16      PRAFAC
             ________      ________
KESCAT16           0             0
PRAFAC             0             0


          THETA
             KESCAT16      PRAFAC
             ________      ________
KESCAT16           0
PRAFAC             0             0


          ALPHA
             KESCAT16      PRAFAC
             ________      ________
                   0             0


          BETA
             KESCAT16      PRAFAC
             ________      ________
KESCAT16           0            34
PRAFAC             0             0
```

```
           PSI
              KESCAT16      PRAFAC
              ________      ________
 KESCAT16            35
 PRAFAC               0             0


     STARTING VALUES FOR WITHIN


           TAU
              KESCAT16
              ________
                0.000


           NU
              KESCAT16      PHY15         KES15         CHOICE15      BULLY15
              ________      ________      ________      ________      ________
                0.000         0.000         0.000         0.000         0.000


           NU
              PRESS15       DUTIES15
              ________      ________
                0.000         0.000


           LAMBDA
              KESCAT16      PHY15         KES15         CHOICE15      BULLY15
              ________      ________      ________      ________      ________
 KESCAT16       1.000         0.000         0.000         0.000         0.000
 PHY15          0.000         1.000         0.000         0.000         0.000
 KES15          0.000         0.000         1.000         0.000         0.000
 CHOICE15       0.000         0.000         0.000         1.000         0.000
 BULLY15        0.000         0.000         0.000         0.000         1.000
 PRESS15        0.000         0.000         0.000         0.000         0.000
 DUTIES15       0.000         0.000         0.000         0.000         0.000


           LAMBDA
              PRESS15       DUTIES15
```

KESCAT16	0.000	0.000
PHY15	0.000	0.000
KES15	0.000	0.000
CHOICE15	0.000	0.000
BULLY15	0.000	0.000
PRESS15	1.000	0.000
DUTIES15	0.000	1.000

THETA

	KESCAT16	PHY15	KES15	CHOICE15	BULLY15
KESCAT16	0.000				
PHY15	0.000	0.000			
KES15	0.000	0.000	0.000		
CHOICE15	0.000	0.000	0.000	0.000	
BULLY15	0.000	0.000	0.000	0.000	0.000
PRESS15	0.000	0.000	0.000	0.000	0.000
DUTIES15	0.000	0.000	0.000	0.000	0.000

THETA

	PRESS15	DUTIES15
PRESS15	0.000	
DUTIES15	0.000	0.000

ALPHA

KESCAT16	PHY15	KES15	CHOICE15	BULLY15
0.000	0.000	0.000	0.000	0.000

ALPHA

PRESS15	DUTIES15
0.000	0.000

BETA

KESCAT16	PHY15	KES15	CHOICE15	BULLY15

KESCAT16	0.000	0.000	0.000	0.000	0.000
PHY15	0.000	0.000	0.000	0.000	0.000
KES15	0.000	0.000	0.000	0.000	0.000
CHOICE15	0.000	0.000	0.000	0.000	0.000
BULLY15	0.000	0.000	0.000	0.000	0.000
PRESS15	0.000	0.000	0.000	0.000	0.000
DUTIES15	0.000	0.000	0.000	0.000	0.000

BETA

	PRESS15	DUTIES15
KESCAT16	0.000	0.000
PHY15	0.000	0.000
KES15	0.000	0.000
CHOICE15	0.000	0.000
BULLY15	0.000	0.000
PRESS15	0.000	0.000
DUTIES15	0.000	0.000

PSI

	KESCAT16	PHY15	KES15	CHOICE15	BULLY15
KESCAT16	1.000				
PHY15	0.000	0.351			
KES15	0.000	0.000	0.157		
CHOICE15	0.000	0.000	0.000	0.597	
BULLY15	0.000	0.000	0.000	0.000	0.301
PRESS15	0.000	0.000	0.000	0.000	0.000
DUTIES15	0.000	0.000	0.000	0.000	0.000

PSI

	PRESS15	DUTIES15
PRESS15	0.580	
DUTIES15	0.000	0.516

STARTING VALUES FOR BETWEEN

TAU

	KESCAT16
	-0.352

NU

	KESCAT16	PRAFAC
	0.000	0.000

LAMBDA

	KESCAT16	PRAFAC
KESCAT16	1.000	0.000
PRAFAC	0.000	1.000

THETA

	KESCAT16	PRAFAC
KESCAT16	0.000	
PRAFAC	0.000	0.000

ALPHA

	KESCAT16	PRAFAC
	0.000	0.000

BETA

	KESCAT16	PRAFAC
KESCAT16	0.000	0.000
PRAFAC	0.000	0.000

PSI

	KESCAT16	PRAFAC
KESCAT16	1.000	
PRAFAC	0.000	0.469

PRIORS FOR ALL PARAMETERS	PRIOR MEAN	PRIOR VARIANCE	PRIOR STD. DEV.
Parameter 1~N(0.000,infinity)	0.0000	infinity	infinity
Parameter 2~N(0.000,infinity)	0.0000	infinity	infinity
Parameter 3~N(0.000,infinity)	0.0000	infinity	infinity
Parameter 4~N(0.000,infinity)	0.0000	infinity	infinity
Parameter 5~N(0.000,infinity)	0.0000	infinity	infinity
Parameter 6~N(0.000,infinity)	0.0000	infinity	infinity
Parameter 7~N(0.000,5.000)	0.0000	5.0000	2.2361
Parameter 8~N(0.000,5.000)	0.0000	5.0000	2.2361
Parameter 9~N(0.000,5.000)	0.0000	5.0000	2.2361
Parameter 10~N(0.000,5.000)	0.0000	5.0000	2.2361
Parameter 11~N(0.000,5.000)	0.0000	5.0000	2.2361
Parameter 12~N(0.000,5.000)	0.0000	5.0000	2.2361
Parameter 13~IW(1.000,7)	infinity	infinity	infinity
Parameter 14~IW(0.000,7)	infinity	infinity	infinity
Parameter 15~IW(1.000,7)	infinity	infinity	infinity
Parameter 16~IW(0.000,7)	infinity	infinity	infinity
Parameter 17~IW(0.000,7)	infinity	infinity	infinity
Parameter 18~IW(1.000,7)	infinity	infinity	infinity
Parameter 19~IW(0.000,7)	infinity	infinity	infinity
Parameter 20~IW(0.000,7)	infinity	infinity	infinity
Parameter 21~IW(0.000,7)	infinity	infinity	infinity
Parameter 22~IW(1.000,7)	infinity	infinity	infinity
Parameter 23~IW(0.000,7)	infinity	infinity	infinity
Parameter 24~IW(0.000,7)	infinity	infinity	infinity
Parameter 25~IW(0.000,7)	infinity	infinity	infinity
Parameter 26~IW(0.000,7)	infinity	infinity	infinity
Parameter 27~IW(1.000,7)	infinity	infinity	infinity
Parameter 28~IW(0.000,7)	infinity	infinity	infinity
Parameter 29~IW(0.000,7)	infinity	infinity	infinity
Parameter 30~IW(0.000,7)	infinity	infinity	infinity
Parameter 31~IW(0.000,7)	infinity	infinity	infinity
Parameter 32~IW(0.000,7)	infinity	infinity	infinity
Parameter 33~IW(1.000,7)	infinity	infinity	infinity
Parameter 34~N(0.000,5.000)	0.0000	5.0000	2.2361
Parameter 35~IG(-1.000,0.000)	infinity	infinity	infinity
Parameter 36~N(0.000,5.000)	0.0000	5.0000	2.2361

TECHNICAL 3 OUTPUT

ESTIMATED COVARIANCE MATRIX FOR PARAMETER ESTIMATES

	1	2	3	4	5
1	0.603642D-03				
2	0.899850D-04	0.296593D-03			
3	-0.591057D-04	-0.721338D-04	0.103105D-02		
4	0.409773D-04	0.770698D-04	-0.127031D-03	0.540699D-03	
5	0.945170D-04	0.469306D-04	-0.140565D-03	0.181351D-03	0.104656D-02
6	-0.366895D-04	-0.865774D-04	0.154964D-03	-0.164964D-03	-0.288483D-03
7	-0.104483D-04	0.136509D-04	0.557229D-04	0.183806D-04	0.427812D-04
8	-0.830143D-04	-0.579282D-05	-0.780820D-04	-0.630649D-04	0.876926D-05
9	-0.307338D-04	-0.200110D-04	-0.359075D-04	0.664874D-05	0.151930D-04
10	-0.186296D-04	-0.225216D-04	0.407522D-04	-0.370885D-05	0.306596D-04
11	-0.764701D-05	-0.513629D-05	0.130043D-04	-0.538085D-05	0.501944D-04
12	0.117472D-04	0.143923D-06	0.978588D-04	-0.242555D-04	0.201258D-04
13	-0.188693D-05	-0.214909D-05	0.166460D-05	-0.114366D-04	-0.103539D-04
14	0.113538D-04	0.319508D-06	0.368492D-05	-0.155659D-04	-0.278568D-04
15	-0.155407D-05	0.148945D-04	0.629825D-05	-0.166503D-04	-0.216209D-04
16	0.850917D-05	-0.327069D-05	0.128830D-05	0.276349D-04	0.181681D-05
17	-0.160712D-04	-0.101675D-04	-0.159528D-05	0.411915D-05	-0.922466D-05
18	0.190860D-05	-0.194782D-04	0.499083D-04	-0.332218D-04	-0.321350D-04
19	0.550795D-05	-0.279251D-06	0.301774D-05	-0.102907D-04	0.388176D-05
20	0.495898D-05	0.112368D-05	-0.661410D-05	0.683555D-05	0.558955D-05
21	0.522359D-05	0.117129D-04	0.153543D-04	-0.160166D-04	0.237419D-04
22	-0.209459D-04	-0.151734D-04	0.120195D-04	0.184402D-05	0.165140D-04
23	0.216425D-04	0.725365D-05	-0.374961D-04	0.169287D-04	-0.474637D-05
24	-0.674831D-06	0.161528D-04	0.923860D-05	0.154804D-04	0.384635D-05
25	0.315691D-04	-0.180917D-05	-0.106865D-05	0.117712D-04	0.236097D-04
26	0.709739D-06	-0.250965D-04	-0.173345D-04	0.860231D-05	-0.204631D-04
27	0.381944D-04	0.211190D-05	0.873891D-05	0.108227D-05	0.383745D-04
28	-0.188152D-04	-0.127038D-04	0.619676D-05	-0.178006D-04	0.287981D-04
29	0.605802D-06	-0.595428D-05	-0.257063D-04	-0.756121D-05	0.119797D-04
30	0.330134D-05	0.231166D-04	-0.375877D-05	-0.174213D-04	-0.544030D-04
31	-0.252104D-04	-0.659203D-07	-0.100655D-04	-0.819540D-05	-0.105302D-04
32	0.308076D-05	0.120146D-04	-0.173317D-05	-0.149680D-04	0.742347D-05
33	-0.881457D-05	0.222487D-04	-0.775412D-05	-0.786232D-05	-0.438844D-05
34	0.129699D-06	-0.281143D-04	0.339951D-04	-0.386502D-04	-0.450203D-04
35	0.288863D-04	0.134743D-04	-0.348152D-05	0.136402D-04	0.431525D-04
36	0.401664D-04	-0.171314D-04	0.142282D-04	-0.193660D-04	-0.260990D-04

ESTIMATED COVARIANCE MATRIX FOR PARAMETER ESTIMATES

	6	7	8	9	10

6	0.100188D-02				
7	-0.635948D-04	0.274147D-02			
8	-0.688835D-05	-0.338760D-03	0.904127D-02		
9	0.456409D-04	0.149431D-03	0.530399D-03	0.147556D-02	
10	-0.656212D-04	-0.404659D-05	-0.503044D-04	0.165359D-03	0.372619D-02
11	0.476630D-04	-0.241931D-03	-0.129857D-03	0.151498D-03	-0.352553D-03
12	-0.337601D-05	0.114458D-04	0.530402D-03	-0.242833D-03	0.281159D-03
13	0.120102D-04	-0.329386D-04	-0.719407D-04	-0.223064D-04	0.678052D-05
14	0.192113D-04	-0.426608D-05	0.881143D-05	0.101582D-05	-0.970948D-05
15	0.104841D-04	0.182805D-04	0.433482D-04	0.299441D-05	0.134288D-04
16	0.883863D-05	-0.161861D-04	0.127844D-03	-0.852590D-05	0.741200D-04
17	0.315377D-05	0.159433D-04	-0.369298D-04	-0.181065D-04	0.218699D-04
18	0.363661D-04	-0.219954D-04	0.756275D-04	-0.326607D-04	0.850512D-04
19	0.104319D-04	0.426810D-04	0.418078D-04	0.103129D-04	0.495031D-04
20	0.109351D-05	0.269716D-04	-0.238239D-04	0.262746D-05	0.158668D-04
21	-0.200031D-04	-0.534068D-04	0.661875D-04	-0.416103D-04	-0.226809D-04
22	-0.204770D-04	-0.927317D-05	0.391381D-04	0.111236D-04	-0.509618D-04
23	-0.252784D-04	-0.346795D-04	0.830796D-04	0.122936D-04	0.949404D-05
24	-0.840508D-06	0.321060D-04	0.674790D-06	-0.114961D-04	-0.833706D-05
25	-0.690596D-05	-0.117459D-05	0.562550D-05	-0.554570D-04	0.474043D-05
26	0.873662D-05	0.114371D-04	-0.858115D-05	-0.125671D-04	0.190196D-05
27	0.428164D-04	0.654583D-05	-0.147183D-03	0.186688D-04	0.223603D-03
28	0.534328D-05	0.616559D-05	-0.163249D-04	-0.375767D-05	-0.348925D-04
29	-0.174733D-04	0.292211D-04	0.142250D-04	0.245400D-04	0.723965D-05
30	0.386126D-04	0.409202D-05	-0.318801D-04	-0.242125D-04	-0.983746D-04
31	0.219239D-04	-0.583605D-05	-0.166920D-04	0.455844D-04	0.228715D-04
32	0.690088D-05	-0.694281D-04	0.610596D-04	0.284335D-04	-0.333197D-05
33	0.360664D-04	0.112440D-03	-0.371679D-04	-0.423554D-04	0.666091D-04
34	0.595785D-05	-0.808818D-04	0.267003D-05	-0.199771D-03	-0.543166D-04
35	0.110377D-04	0.410259D-04	0.280489D-03	0.954422D-04	-0.452970D-04
36	0.180914D-04	-0.117052D-03	-0.809301D-03	0.123278D-03	-0.242812D-03

ESTIMATED COVARIANCE MATRIX FOR PARAMETER ESTIMATES

	11	12	13	14	15
11	0.168491D-02				
12	0.426798D-03	0.204465D-02			
13	-0.166354D-04	-0.333208D-04	0.854931D-03		
14	-0.553582D-05	0.329070D-06	0.123511D-03	0.193660D-03	
15	0.797378D-05	-0.154173D-04	-0.110167D-05	0.584729D-04	0.178349D-03
16	0.248640D-05	-0.661854D-06	-0.990576D-04	-0.537278D-04	-0.177566D-04
17	-0.172806D-04	-0.233974D-04	0.528376D-06	-0.338253D-04	-0.602292D-04

18	-0.200120D-04	0.691259D-04	0.751958D-05	0.439797D-05	0.249971D-05
19	-0.103584D-04	0.541135D-05	0.374215D-04	0.414552D-04	0.149638D-04
20	-0.135034D-04	-0.905044D-05	0.842113D-05	0.167601D-04	0.412058D-04
21	0.151667D-05	-0.379420D-04	0.104184D-04	-0.157043D-04	-0.832805D-05
22	0.161288D-04	0.250493D-05	-0.886056D-05	0.152960D-04	0.193329D-04
23	0.106668D-04	0.213942D-04	0.123178D-03	0.428757D-04	0.170259D-04
24	-0.591523D-05	0.179708D-06	0.190305D-04	0.330991D-04	0.475063D-04
25	0.217519D-04	0.838788D-04	0.407597D-05	0.158088D-05	0.831338D-05
26	0.224098D-04	0.302329D-04	-0.946479D-05	0.724602D-05	0.395605D-05
27	0.733672D-04	0.570100D-04	0.832239D-05	-0.438513D-04	-0.345785D-04
28	0.161719D-04	0.242989D-04	-0.527570D-04	-0.310976D-04	-0.101345D-04
29	-0.138531D-04	0.969732D-05	0.901694D-05	-0.118102D-04	-0.378481D-04
30	0.339226D-04	-0.482617D-04	-0.919418D-05	0.272223D-05	-0.264320D-05
31	-0.255225D-04	-0.545571D-04	0.367026D-04	-0.202186D-05	-0.786557D-05
32	-0.222994D-04	-0.207193D-04	0.350137D-04	0.498865D-05	0.170693D-04
33	0.612889D-04	0.628332D-04	-0.184189D-04	0.119618D-04	-0.117046D-04
34	-0.117040D-03	-0.160981D-04	0.612284D-04	0.372905D-05	0.426657D-04
35	0.373792D-04	0.779753D-04	-0.275360D-04	-0.634826D-06	-0.296270D-05
36	0.761828D-04	0.799668D-04	-0.102266D-04	0.103730D-05	-0.126000D-04

ESTIMATED COVARIANCE MATRIX FOR PARAMETER ESTIMATES

	16	17	18	19	20
16	0.745447D-03				
17	0.115667D-03	0.334077D-03			
18	-0.195618D-03	-0.178127D-03	0.260385D-02		
19	-0.842161D-04	-0.205677D-05	0.544322D-04	0.389266D-03	
20	-0.136865D-05	-0.369549D-04	0.123876D-04	0.566933D-04	0.183492D-03
21	0.655907D-04	0.736181D-04	-0.274306D-03	-0.670977D-04	-0.579388D-04
22	-0.147044D-04	-0.226706D-04	0.601174D-04	0.430586D-04	0.921889D-04
23	-0.138166D-03	-0.166496D-04	-0.145956D-04	0.117096D-03	0.348530D-04
24	-0.239086D-04	-0.625248D-04	0.189076D-04	0.223003D-04	0.788531D-04
25	0.133878D-03	0.604972D-04	-0.316421D-03	-0.496504D-04	-0.464423D-04
26	-0.791059D-06	-0.214898D-04	0.474439D-04	0.704277D-04	0.524027D-04
27	-0.161948D-04	-0.122198D-04	0.111304D-03	0.440783D-04	0.386497D-04
28	0.102222D-03	0.208038D-04	0.225009D-04	-0.113672D-03	-0.149985D-04
29	-0.778885D-05	0.446986D-04	-0.452411D-04	-0.100615D-04	-0.584143D-04
30	-0.723226D-04	-0.744839D-04	0.247875D-03	0.247571D-04	0.336320D-04
31	0.116282D-05	0.161263D-04	-0.505005D-04	-0.271187D-04	-0.745749D-04
32	-0.183377D-05	-0.214370D-04	-0.425618D-04	-0.568036D-04	-0.286224D-04
33	-0.345556D-04	-0.113975D-04	0.528358D-04	0.213010D-04	0.193448D-04
34	0.231766D-04	-0.351166D-04	-0.324389D-04	-0.257573D-04	0.412446D-05
35	-0.120232D-04	-0.118724D-04	0.110946D-03	0.310040D-04	0.351622D-05

36	0.269095D-04	-0.137801D-04	-0.128558D-04	0.283064D-06	0.404016D-04

ESTIMATED COVARIANCE MATRIX FOR PARAMETER ESTIMATES

	21	22	23	24	25
21	0.643431D-03				
22	-0.141710D-03	0.665211D-03			
23	-0.236789D-04	0.271757D-04	0.768819D-03		
24	-0.191268D-04	0.492242D-04	0.152494D-03	0.355170D-03	
25	0.249563D-03	-0.908369D-04	-0.137315D-03	-0.131472D-03	0.123569D-02
26	-0.123281D-03	0.220274D-03	0.837291D-04	0.113694D-03	-0.153223D-03
27	-0.833651D-04	0.542388D-04	0.233971D-03	0.122790D-03	-0.418637D-03
28	0.345926D-05	-0.927443D-05	-0.198823D-03	-0.672505D-04	0.365807D-04
29	0.196557D-04	-0.473525D-04	-0.463827D-04	-0.118196D-03	0.637318D-04
30	-0.209042D-03	0.586880D-04	0.677098D-04	0.508358D-04	-0.313986D-03
31	0.928235D-04	-0.191741D-03	-0.486486D-05	-0.503379D-04	0.905924D-04
32	0.355242D-04	-0.106115D-03	-0.821297D-04	-0.966608D-04	0.190956D-03
33	-0.632201D-04	0.915263D-04	0.257542D-04	0.282301D-04	-0.127693D-03
34	-0.309429D-04	0.437564D-04	-0.752563D-06	0.385550D-05	-0.225495D-04
35	0.191287D-04	-0.303661D-04	-0.466066D-05	-0.552627D-06	0.208246D-04
36	0.731992D-05	0.439850D-04	-0.128184D-04	0.459179D-04	0.263695D-04

ESTIMATED COVARIANCE MATRIX FOR PARAMETER ESTIMATES

	26	27	28	29	30
26	0.666913D-03				
27	0.435012D-03	0.256673D-02			
28	-0.312055D-04	-0.824775D-04	0.655080D-03		
29	-0.526859D-04	-0.330863D-04	0.120338D-03	0.329933D-03	
30	0.478349D-04	0.155117D-03	-0.101186D-03	-0.100891D-03	0.110764D-02
31	-0.196720D-03	-0.113053D-03	0.344362D-04	0.103375D-03	-0.155104D-03
32	-0.264369D-03	-0.646022D-03	0.112800D-03	0.866075D-04	-0.253907D-03
33	0.114041D-03	0.224307D-03	-0.134951D-03	-0.160239D-03	0.292145D-03
34	0.150186D-04	-0.137639D-04	-0.423209D-04	-0.111500D-05	0.387724D-04
35	-0.659776D-05	0.722927D-05	-0.228553D-04	-0.125721D-04	-0.162980D-04
36	0.686901D-04	0.858373D-04	0.531199D-04	-0.639276D-05	0.893114D-05

ESTIMATED COVARIANCE MATRIX FOR PARAMETER ESTIMATES

	31	32	33	34	35
31	0.626340D-03				

32	0.286287D-03	0.128265D-02			
33	-0.358523D-03	-0.619079D-03	0.205964D-02		
34	-0.250640D-04	-0.118132D-04	0.346400D-04	0.253908D-02	
35	0.101913D-04	0.913928D-05	-0.221912D-04	-0.181824D-03	0.901483D-03
36	-0.525036D-04	-0.668148D-04	0.284714D-04	-0.229433D-03	0.482287D-04

ESTIMATED COVARIANCE MATRIX FOR PARAMETER ESTIMATES

	36
36	0.296792D-02

ESTIMATED CORRELATION MATRIX FOR PARAMETER ESTIMATES

	1	2	3	4	5
1	1.000				
2	0.213	1.000			
3	-0.075	-0.130	1.000		
4	0.072	0.192	-0.170	1.000	
5	0.119	0.084	-0.135	0.241	1.000
6	-0.047	-0.159	0.152	-0.224	-0.282
7	-0.008	0.015	0.033	0.015	0.025
8	-0.036	-0.004	-0.026	-0.029	0.003
9	-0.033	-0.030	-0.029	0.007	0.012
10	-0.012	-0.021	0.021	-0.003	0.016
11	-0.008	-0.007	0.010	-0.006	0.038
12	0.011	0.000	0.067	-0.023	0.014
13	-0.003	-0.004	0.002	-0.017	-0.011
14	0.033	0.001	0.008	-0.048	-0.062
15	-0.005	0.065	0.015	-0.054	-0.050
16	0.013	-0.007	0.001	0.044	0.002
17	-0.036	-0.032	-0.003	0.010	-0.016
18	0.002	-0.022	0.030	-0.028	-0.019
19	0.011	-0.001	0.005	-0.022	0.006
20	0.015	0.005	-0.015	0.022	0.013
21	0.008	0.027	0.019	-0.027	0.029
22	-0.033	-0.034	0.015	0.003	0.020
23	0.032	0.015	-0.042	0.026	-0.005
24	-0.001	0.050	0.015	0.035	0.006
25	0.037	-0.003	-0.001	0.014	0.021
26	0.001	-0.056	-0.021	0.014	-0.024
27	0.031	0.002	0.005	0.001	0.023
28	-0.030	-0.029	0.008	-0.030	0.035

29	0.001	-0.019	-0.044	-0.018	0.020
30	0.004	0.040	-0.004	-0.023	-0.051
31	-0.041	0.000	-0.013	-0.014	-0.013
32	0.004	0.019	-0.002	-0.018	0.006
33	-0.008	0.028	-0.005	-0.007	-0.003
34	0.000	-0.032	0.021	-0.033	-0.028
35	0.039	0.026	-0.004	0.020	0.044
36	0.030	-0.018	0.008	-0.015	-0.015

ESTIMATED CORRELATION MATRIX FOR PARAMETER ESTIMATES

	6	7	8	9	10
6	1.000				
7	-0.038	1.000			
8	-0.002	-0.068	1.000		
9	0.038	0.074	0.145	1.000	
10	-0.034	-0.001	-0.009	0.071	1.000
11	0.037	-0.113	-0.033	0.096	-0.141
12	-0.002	0.005	0.123	-0.140	0.102
13	0.013	-0.022	-0.026	-0.020	0.004
14	0.044	-0.006	0.007	0.002	-0.011
15	0.025	0.026	0.034	0.006	0.016
16	0.010	-0.011	0.049	-0.008	0.044
17	0.005	0.017	-0.021	-0.026	0.020
18	0.023	-0.008	0.016	-0.017	0.027
19	0.017	0.041	0.022	0.014	0.041
20	0.003	0.038	-0.018	0.005	0.019
21	-0.025	-0.040	0.027	-0.043	-0.015
22	-0.025	-0.007	0.016	0.011	-0.032
23	-0.029	-0.024	0.032	0.012	0.006
24	-0.001	0.033	0.000	-0.016	-0.007
25	-0.006	-0.001	0.002	-0.041	0.002
26	0.011	0.008	-0.003	-0.013	0.001
27	0.027	0.002	-0.031	0.010	0.072
28	0.007	0.005	-0.007	-0.004	-0.022
29	-0.030	0.031	0.008	0.035	0.007
30	0.037	0.002	-0.010	-0.019	-0.048
31	0.028	-0.004	-0.007	0.047	0.015
32	0.006	-0.037	0.018	0.021	-0.002
33	0.025	0.047	-0.009	-0.024	0.024
34	0.004	-0.031	0.001	-0.103	-0.018
35	0.012	0.026	0.098	0.083	-0.025
36	0.010	-0.041	-0.156	0.059	-0.073

ESTIMATED CORRELATION MATRIX FOR PARAMETER ESTIMATES

	11	12	13	14	15
11	1.000				
12	0.230	1.000			
13	-0.014	-0.025	1.000		
14	-0.010	0.001	0.304	1.000	
15	0.015	-0.026	-0.003	0.315	1.000
16	0.002	-0.001	-0.124	-0.141	-0.049
17	-0.023	-0.028	0.001	-0.133	-0.247
18	-0.010	0.030	0.005	0.006	0.004
19	-0.013	0.006	0.065	0.151	0.057
20	-0.024	-0.015	0.021	0.089	0.228
21	0.001	-0.033	0.014	-0.044	-0.025
22	0.015	0.002	-0.012	0.043	0.056
23	0.009	0.017	0.152	0.111	0.046
24	-0.008	0.000	0.035	0.126	0.189
25	0.015	0.053	0.004	0.003	0.018
26	0.021	0.026	-0.013	0.020	0.011
27	0.035	0.025	0.006	-0.062	-0.051
28	0.015	0.021	-0.070	-0.087	-0.030
29	-0.019	0.012	0.017	-0.047	-0.156
30	0.025	-0.032	-0.009	0.006	-0.006
31	-0.025	-0.048	0.050	-0.006	-0.024
32	-0.015	-0.013	0.033	0.010	0.036
33	0.033	0.031	-0.014	0.019	-0.019
34	-0.057	-0.007	0.042	0.005	0.063
35	0.030	0.057	-0.031	-0.002	-0.007
36	0.034	0.032	-0.006	0.001	-0.017

ESTIMATED CORRELATION MATRIX FOR PARAMETER ESTIMATES

	16	17	18	19	20
16	1.000				
17	0.232	1.000			
18	-0.140	-0.191	1.000		
19	-0.156	-0.006	0.054	1.000	
20	-0.004	-0.149	0.018	0.212	1.000
21	0.095	0.159	-0.212	-0.134	-0.169
22	-0.021	-0.048	0.046	0.085	0.264
23	-0.183	-0.033	-0.010	0.214	0.093

24	-0.046	-0.182	0.020	0.060	0.309
25	0.139	0.094	-0.176	-0.072	-0.098
26	-0.001	-0.046	0.036	0.138	0.150
27	-0.012	-0.013	0.043	0.044	0.056
28	0.146	0.044	0.017	-0.225	-0.043
29	-0.016	0.135	-0.049	-0.028	-0.237
30	-0.080	-0.122	0.146	0.038	0.075
31	0.002	0.035	-0.040	-0.055	-0.220
32	-0.002	-0.033	-0.023	-0.080	-0.059
33	-0.028	-0.014	0.023	0.024	0.031
34	0.017	-0.038	-0.013	-0.026	0.006
35	-0.015	-0.022	0.072	0.052	0.009
36	0.018	-0.014	-0.005	0.000	0.055

ESTIMATED CORRELATION MATRIX FOR PARAMETER ESTIMATES

	21	22	23	24	25
21	1.000				
22	-0.217	1.000			
23	-0.034	0.038	1.000		
24	-0.040	0.101	0.292	1.000	
25	0.280	-0.100	-0.141	-0.198	1.000
26	-0.188	0.331	0.117	0.234	-0.169
27	-0.065	0.042	0.167	0.129	-0.235
28	0.005	-0.014	-0.280	-0.139	0.041
29	0.043	-0.101	-0.092	-0.345	0.100
30	-0.248	0.068	0.073	0.081	-0.268
31	0.146	-0.297	-0.007	-0.107	0.103
32	0.039	-0.115	-0.083	-0.143	0.152
33	-0.055	0.078	0.020	0.033	-0.080
34	-0.024	0.034	-0.001	0.004	-0.013
35	0.025	-0.039	-0.006	-0.001	0.020
36	0.005	0.031	-0.008	0.045	0.014

ESTIMATED CORRELATION MATRIX FOR PARAMETER ESTIMATES

	26	27	28	29	30
26	1.000				
27	0.332	1.000			
28	-0.047	-0.064	1.000		
29	-0.112	-0.036	0.259	1.000	
30	0.056	0.092	-0.119	-0.167	1.000

31	-0.304	-0.089	0.054	0.227	-0.186
32	-0.286	-0.356	0.123	0.133	-0.213
33	0.097	0.098	-0.116	-0.194	0.193
34	0.012	-0.005	-0.033	-0.001	0.023
35	-0.009	0.005	-0.030	-0.023	-0.016
36	0.049	0.031	0.038	-0.006	0.005

ESTIMATED CORRELATION MATRIX FOR PARAMETER ESTIMATES

	31	32	33	34	35
31	1.000				
32	0.319	1.000			
33	-0.316	-0.381	1.000		
34	-0.020	-0.007	0.015	1.000	
35	0.014	0.008	-0.016	-0.120	1.000
36	-0.039	-0.034	0.012	-0.084	0.029

ESTIMATED CORRELATION MATRIX FOR PARAMETER ESTIMATES

	36
36	1.000

TECHNICAL 8 OUTPUT

TECHNICAL 8 OUTPUT FOR BAYES ESTIMATION

CHAIN	BSEED
1	0
2	285380
3	253358

ITERATION	POTENTIAL SCALE REDUCTION	PARAMETER WITH HIGHEST PSR
100	1.018	29
200	1.010	13
300	1.005	35
400	1.005	12
500	1.002	35
600	1.007	35
700	1.003	35

800	1.011	35
900	1.003	35
1000	1.003	28

Reduced distress, curvilinear effects

SUMMARY OF DATA

Number of clusters 58

Size (s)	Cluster ID with Size s
1	166
2	74 205
3	106 182 165
4	93 170 22 192 4
5	65 71 50 84
6	80 72
7	40 107 61 75
8	169 30 173 228
9	144 81 83 181
10	69 145 36 68
11	108 116 174 154
12	206 162
13	226 114
14	2
15	152 98
18	156
19	202
21	92 6
24	178
25	142 122
32	24
37	204
47	209
53	125
67	217
174	110
210	100

COVARIANCE COVERAGE OF DATA

Minimum covariance coverage value 0.100

Number of missing data patterns 5

PROPORTION OF DATA PRESENT

Covariance Coverage

	KESCAT16	PRAFAC	PRAFACSQ	PHY15	KES15
KESCAT16	1.000				
PRAFAC	1.000	1.000			
PRAFACSQ	1.000	1.000	1.000		
PHY15	1.000	1.000	1.000	1.000	
KES15	1.000	1.000	1.000	1.000	1.000
CHOICE15	0.990	0.990	0.990	0.990	0.990
BULLY15	0.990	0.990	0.990	0.990	0.990
PRESS15	0.990	0.990	0.990	0.990	0.990
DUTIES15	0.991	0.991	0.991	0.991	0.991

Covariance Coverage

	CHOICE15	BULLY15	PRESS15	DUTIES15
CHOICE15	0.990			
BULLY15	0.989	0.990		
PRESS15	0.989	0.989	0.990	
DUTIES15	0.990	0.990	0.990	0.991

UNIVARIATE PROPORTIONS AND COUNTS FOR CATEGORICAL VARIABLES

KESCAT16		
Category 1	0.347	386.000
Category 2	0.653	727.000

UNIVARIATE SAMPLE STATISTICS

UNIVARIATE HIGHER-ORDER MOMENT DESCRIPTIVE STATISTICS

Variable/ Sample Size	Mean/ Variance	Skewness/ Kurtosis	Minimum/ Maximum	% with Min/Max	Percentiles 20%/60%	40%/80%	Median
PRAFAC	-0.185	0.245	-2.338	1.72%	-1.448	-0.527	-0.318

```
           58.000       1.345      -0.636       2.338    1.72%       0.080      0.752
     PRAFACSQ           1.379       1.240       0.002    1.72%       0.151      0.381      0.681
           58.000       2.369       0.557       5.467    1.72%       1.168      2.366
     PHY15              0.000       0.390      -1.412   11.50%      -0.412     -0.412     -0.412
         1113.000       0.702       0.082       2.588    1.08%       0.588      0.588
     KES15              0.000       1.366      -0.556   20.75%      -0.556     -0.222     -0.222
         1113.000       0.313       1.871       2.278    0.63%      -0.056      0.444
     CHOICE15           0.000      -0.116      -1.814   15.06%      -0.814      0.186      0.186
         1102.000       1.193      -0.747       2.186    4.45%       0.186      1.186
     BULLY15            0.000       1.887      -0.426   71.96%      -0.426     -0.426     -0.426
         1102.000       0.602       3.132       3.574    0.45%      -0.426      0.574
     PRESS15            0.000       0.096      -1.915    9.89%      -0.915      0.085      0.085
         1102.000       1.160      -0.512       2.085    8.35%       0.085      1.085
     DUTIES15           0.000      -0.677      -2.672    3.54%      -0.672      0.328      0.328
         1103.000       1.031       0.049       1.328   20.22%       0.328      1.328


THE MODEL ESTIMATION TERMINATED NORMALLY

     USE THE FBITERATIONS OPTION TO INCREASE THE NUMBER OF ITERATIONS BY A FACTOR
     OF AT LEAST TWO TO CHECK CONVERGENCE AND THAT THE PSR VALUE DOES NOT INCREASE.



MODEL FIT INFORMATION

Number of Free Parameters                              42

Bayesian Posterior Predictive Checking using Chi-Square

          95% Confidence Interval for the Difference Between
          the Observed and the Replicated Chi-Square Values

                              -23.392           29.508

          Posterior Predictive P-Value              0.516



MODEL RESULTS

                                Posterior  One-Tailed         95% C.I.
                    Estimate       S.D.      P-Value   Lower 2.5%  Upper 2.5%  Significance
```

Within Level

KESCAT16 ON						
PHY15	0.161	0.051	0.001	0.060	0.258	*
CHOICE15	0.031	0.039	0.217	-0.047	0.108	
BULLY15	0.102	0.059	0.038	-0.007	0.221	
DUTIES15	-0.080	0.043	0.027	-0.168	0.003	
PRESS15	-0.046	0.040	0.126	-0.123	0.033	
KES15	0.803	0.095	0.000	0.621	0.995	*
KES15 WITH						
PHY15	0.107	0.014	0.000	0.080	0.136	*
CHOICE15	-0.096	0.019	0.000	-0.134	-0.061	*
BULLY15	0.073	0.013	0.000	0.045	0.100	*
PRESS15	0.063	0.018	0.001	0.029	0.101	*
DUTIES15	-0.073	0.017	0.000	-0.106	-0.041	*
PHY15 WITH						
CHOICE15	-0.096	0.028	0.000	-0.152	-0.043	*
BULLY15	0.040	0.020	0.021	0.003	0.077	*
PRESS15	0.120	0.027	0.000	0.068	0.174	*
DUTIES15	-0.059	0.026	0.010	-0.112	-0.007	*
CHOICE15 WITH						
BULLY15	-0.139	0.026	0.000	-0.191	-0.089	*
PRESS15	-0.197	0.036	0.000	-0.266	-0.123	*
DUTIES15	0.149	0.034	0.000	0.086	0.218	*
BULLY15 WITH						
PRESS15	0.201	0.026	0.000	0.152	0.253	*
DUTIES15	-0.176	0.024	0.000	-0.222	-0.130	*
PRESS15 WITH						
DUTIES15	-0.285	0.034	0.000	-0.357	-0.223	*
Means						
PHY15	0.000	0.025	0.496	-0.049	0.047	
KES15	0.001	0.017	0.491	-0.033	0.033	
CHOICE15	0.000	0.033	0.497	-0.064	0.065	
BULLY15	-0.001	0.023	0.494	-0.046	0.044	
PRESS15	0.000	0.033	0.497	-0.065	0.066	
DUTIES15	0.001	0.030	0.479	-0.058	0.060	
Variances						

```
    PHY15              0.704       0.030       0.000       0.650       0.766      *
    KES15              0.314       0.013       0.000       0.289       0.342      *
    CHOICE15           1.194       0.049       0.000       1.107       1.298      *
    BULLY15            0.603       0.026       0.000       0.559       0.657      *
    PRESS15            1.160       0.049       0.000       1.074       1.259      *
    DUTIES15           1.031       0.044       0.000       0.947       1.123      *

Between Level

 KESCAT16   ON
    PRAFAC            -0.132       0.051       0.004      -0.238      -0.034      *
    PRAFACSQ          -0.102       0.041       0.006      -0.186      -0.018      *

 PRAFAC   WITH
    PRAFACSQ          -0.119       0.241       0.311      -0.595       0.350

 Means
    PRAFAC            -0.188       0.152       0.107      -0.483       0.114
    PRAFACSQ           1.397       0.211       0.000       0.957       1.806      *

 Thresholds
    KESCAT16$1        -0.557       0.068       0.000      -0.699      -0.428      *

 Variances
    PRAFAC             1.348       0.257       0.000       0.966       2.005      *
    PRAFACSQ           2.380       0.447       0.000       1.677       3.409      *

 Residual Variances
    KESCAT16           0.019       0.027       0.000       0.001       0.100      *

New/Additional Parameters
    MIN               -0.643      23.085       0.010      -2.620      -0.150      *


STANDARDIZED MODEL RESULTS


STDYX Standardization

                                Posterior  One-Tailed         95% C.I.
                    Estimate       S.D.      P-Value   Lower 2.5%  Upper 2.5%  Significance
Within Level

 KESCAT16   ON
```

PHY15	0.119	0.037	0.001	0.045	0.189	*
CHOICE15	0.030	0.037	0.217	-0.045	0.104	
BULLY15	0.070	0.040	0.038	-0.005	0.152	
DUTIES15	-0.072	0.038	0.027	-0.147	0.002	
PRESS15	-0.044	0.038	0.126	-0.116	0.032	
KES15	0.397	0.040	0.000	0.313	0.474	*
KES15 WITH						
PHY15	0.227	0.028	0.000	0.173	0.282	*
CHOICE15	-0.157	0.030	0.000	-0.215	-0.101	*
BULLY15	0.168	0.030	0.000	0.106	0.224	*
PRESS15	0.104	0.030	0.001	0.047	0.165	*
DUTIES15	-0.128	0.029	0.000	-0.185	-0.072	*
PHY15 WITH						
CHOICE15	-0.104	0.030	0.000	-0.164	-0.048	*
BULLY15	0.062	0.030	0.021	0.004	0.119	*
PRESS15	0.132	0.029	0.000	0.075	0.190	*
DUTIES15	-0.069	0.030	0.010	-0.129	-0.008	*
CHOICE15 WITH						
BULLY15	-0.164	0.030	0.000	-0.220	-0.105	*
PRESS15	-0.168	0.029	0.000	-0.224	-0.105	*
DUTIES15	0.135	0.029	0.000	0.077	0.192	*
BULLY15 WITH						
PRESS15	0.241	0.028	0.000	0.186	0.295	*
DUTIES15	-0.223	0.029	0.000	-0.277	-0.168	*
PRESS15 WITH						
DUTIES15	-0.261	0.028	0.000	-0.319	-0.206	*
Means						
PHY15	0.000	0.029	0.496	-0.058	0.057	
KES15	0.001	0.030	0.491	-0.059	0.060	
CHOICE15	0.000	0.031	0.497	-0.060	0.060	
BULLY15	-0.001	0.030	0.494	-0.061	0.057	
PRESS15	0.000	0.031	0.497	-0.060	0.062	
DUTIES15	0.001	0.029	0.479	-0.057	0.060	
Variances						
PHY15	1.000	0.000	0.000	1.000	1.000	
KES15	1.000	0.000	0.000	1.000	1.000	
CHOICE15	1.000	0.000	0.000	1.000	1.000	

```
    BULLY15            1.000       0.000       0.000       1.000       1.000
    PRESS15            1.000       0.000       0.000       1.000       1.000
    DUTIES15           1.000       0.000       0.000       1.000       1.000

Between Level

 KESCAT16   ON
    PRAFAC            -0.588       0.183       0.004      -0.889      -0.193      *
    PRAFACSQ          -0.596       0.187       0.006      -0.902      -0.164      *

 PRAFAC   WITH
    PRAFACSQ          -0.069       0.127       0.311      -0.313       0.194

 Means
    PRAFAC            -0.163       0.130       0.107      -0.405       0.095
    PRAFACSQ           0.902       0.158       0.000       0.598       1.208      *

 Thresholds
    KESCAT16$1        -2.072       0.648       0.000      -3.758      -1.260      *

 Variances
    PRAFAC             1.000       0.000       0.000       1.000       1.000
    PRAFACSQ           1.000       0.000       0.000       1.000       1.000

 Residual Variances
    KESCAT16           0.290       0.200       0.000       0.017       0.744      *


R-SQUARE

Within Level

                                Posterior  One-Tailed         95% C.I.
    Variable        Estimate       S.D.      P-Value   Lower 2.5%  Upper 2.5%

    KESCAT16           0.221       0.034       0.000       0.157       0.287

Between Level

                                Posterior  One-Tailed         95% C.I.
    Variable        Estimate       S.D.      P-Value   Lower 2.5%  Upper 2.5%

    KESCAT16           0.710       0.200       0.000       0.256       0.983
```

```
TECHNICAL 1 OUTPUT


     PARAMETER SPECIFICATION FOR WITHIN


           TAU
              KESCAT16
              ________
                    0


           NU
              KESCAT16      PHY15         KES15         CHOICE15      BULLY15
              ________      ________      ________      ________      ________
                    0             0             0             0             0


           NU
              PRESS15       DUTIES15
              ________      ________
                    0             0


           LAMBDA
              KESCAT16      PHY15         KES15         CHOICE15      BULLY15
              ________      ________      ________      ________      ________
 KESCAT16           0             0             0             0             0
 PHY15              0             0             0             0             0
 KES15              0             0             0             0             0
 CHOICE15           0             0             0             0             0
 BULLY15            0             0             0             0             0
 PRESS15            0             0             0             0             0
 DUTIES15           0             0             0             0             0


           LAMBDA
              PRESS15       DUTIES15
              ________      ________
 KESCAT16           0             0
 PHY15              0             0
 KES15              0             0
 CHOICE15           0             0
```

BULLY15	0	0
PRESS15	0	0
DUTIES15	0	0

THETA

	KESCAT16	PHY15	KES15	CHOICE15	BULLY15
KESCAT16	0				
PHY15	0	0			
KES15	0	0	0		
CHOICE15	0	0	0	0	
BULLY15	0	0	0	0	0
PRESS15	0	0	0	0	0
DUTIES15	0	0	0	0	0

THETA

	PRESS15	DUTIES15
PRESS15	0	
DUTIES15	0	0

ALPHA

KESCAT16	PHY15	KES15	CHOICE15	BULLY15
0	1	2	3	4

ALPHA

PRESS15	DUTIES15
5	6

BETA

	KESCAT16	PHY15	KES15	CHOICE15	BULLY15
KESCAT16	0	7	8	9	10
PHY15	0	0	0	0	0
KES15	0	0	0	0	0
CHOICE15	0	0	0	0	0
BULLY15	0	0	0	0	0

PRESS15	0	0	0	0	0
DUTIES15	0	0	0	0	0

BETA

	PRESS15	DUTIES15
KESCAT16	11	12
PHY15	0	0
KES15	0	0
CHOICE15	0	0
BULLY15	0	0
PRESS15	0	0
DUTIES15	0	0

PSI

	KESCAT16	PHY15	KES15	CHOICE15	BULLY15
KESCAT16	0				
PHY15	0	13			
KES15	0	14	15		
CHOICE15	0	16	17	18	
BULLY15	0	19	20	21	22
PRESS15	0	23	24	25	26
DUTIES15	0	28	29	30	31

PSI

	PRESS15	DUTIES15
PRESS15	27	
DUTIES15	32	33

PARAMETER SPECIFICATION FOR BETWEEN

TAU

KESCAT16
42

NU

KESCAT16	PRAFAC	PRAFACSQ
0	0	0

LAMBDA

	KESCAT16	PRAFAC	PRAFACSQ
KESCAT16	0	0	0
PRAFAC	0	0	0
PRAFACSQ	0	0	0

THETA

	KESCAT16	PRAFAC	PRAFACSQ
KESCAT16	0		
PRAFAC	0	0	
PRAFACSQ	0	0	0

ALPHA

KESCAT16	PRAFAC	PRAFACSQ
0	34	35

BETA

	KESCAT16	PRAFAC	PRAFACSQ
KESCAT16	0	36	37
PRAFAC	0	0	0
PRAFACSQ	0	0	0

PSI

	KESCAT16	PRAFAC	PRAFACSQ
KESCAT16	38		
PRAFAC	0	39	
PRAFACSQ	0	40	41

PARAMETER SPECIFICATION FOR THE ADDITIONAL PARAMETERS

NEW/ADDITIONAL PARAMETERS

MIN
43

STARTING VALUES FOR WITHIN

TAU

KESCAT16
0.000

NU

KESCAT16	PHY15	KES15	CHOICE15	BULLY15
0.000	0.000	0.000	0.000	0.000

NU

PRESS15	DUTIES15
0.000	0.000

LAMBDA

	KESCAT16	PHY15	KES15	CHOICE15	BULLY15
KESCAT16	1.000	0.000	0.000	0.000	0.000
PHY15	0.000	1.000	0.000	0.000	0.000
KES15	0.000	0.000	1.000	0.000	0.000
CHOICE15	0.000	0.000	0.000	1.000	0.000
BULLY15	0.000	0.000	0.000	0.000	1.000
PRESS15	0.000	0.000	0.000	0.000	0.000
DUTIES15	0.000	0.000	0.000	0.000	0.000

LAMBDA

PRESS15 DUTIES15

KESCAT16	0.000	0.000
PHY15	0.000	0.000
KES15	0.000	0.000
CHOICE15	0.000	0.000
BULLY15	0.000	0.000
PRESS15	1.000	0.000
DUTIES15	0.000	1.000

THETA

	KESCAT16	PHY15	KES15	CHOICE15	BULLY15
KESCAT16	0.000				
PHY15	0.000	0.000			
KES15	0.000	0.000	0.000		
CHOICE15	0.000	0.000	0.000	0.000	
BULLY15	0.000	0.000	0.000	0.000	0.000
PRESS15	0.000	0.000	0.000	0.000	0.000
DUTIES15	0.000	0.000	0.000	0.000	0.000

THETA

	PRESS15	DUTIES15
PRESS15	0.000	
DUTIES15	0.000	0.000

ALPHA

KESCAT16	PHY15	KES15	CHOICE15	BULLY15
0.000	0.000	0.000	0.000	0.000

ALPHA

PRESS15	DUTIES15
0.000	0.000

BETA

KESCAT16	PHY15	KES15	CHOICE15	BULLY15

KESCAT16	0.000	0.000	0.000	0.000	0.000
PHY15	0.000	0.000	0.000	0.000	0.000
KES15	0.000	0.000	0.000	0.000	0.000
CHOICE15	0.000	0.000	0.000	0.000	0.000
BULLY15	0.000	0.000	0.000	0.000	0.000
PRESS15	0.000	0.000	0.000	0.000	0.000
DUTIES15	0.000	0.000	0.000	0.000	0.000

BETA

	PRESS15	DUTIES15
KESCAT16	0.000	0.000
PHY15	0.000	0.000
KES15	0.000	0.000
CHOICE15	0.000	0.000
BULLY15	0.000	0.000
PRESS15	0.000	0.000
DUTIES15	0.000	0.000

PSI

	KESCAT16	PHY15	KES15	CHOICE15	BULLY15
KESCAT16	1.000				
PHY15	0.000	0.351			
KES15	0.000	0.000	0.157		
CHOICE15	0.000	0.000	0.000	0.597	
BULLY15	0.000	0.000	0.000	0.000	0.301
PRESS15	0.000	0.000	0.000	0.000	0.000
DUTIES15	0.000	0.000	0.000	0.000	0.000

PSI

	PRESS15	DUTIES15
PRESS15	0.580	
DUTIES15	0.000	0.516

STARTING VALUES FOR BETWEEN

TAU

KESCAT16
-0.352

NU

KESCAT16	PRAFAC	PRAFACSQ
0.000	0.000	0.000

LAMBDA

	KESCAT16	PRAFAC	PRAFACSQ
KESCAT16	1.000	0.000	0.000
PRAFAC	0.000	1.000	0.000
PRAFACSQ	0.000	0.000	1.000

THETA

	KESCAT16	PRAFAC	PRAFACSQ
KESCAT16	0.000		
PRAFAC	0.000	0.000	
PRAFACSQ	0.000	0.000	0.000

ALPHA

KESCAT16	PRAFAC	PRAFACSQ
0.000	-0.033	0.940

BETA

	KESCAT16	PRAFAC	PRAFACSQ
KESCAT16	0.000	0.000	0.000
PRAFAC	0.000	0.000	0.000
PRAFACSQ	0.000	0.000	0.000

PSI

KESCAT16	PRAFAC	PRAFACSQ

KESCAT16	1.000		
PRAFAC	0.000	0.469	
PRAFACSQ	0.000	0.000	0.654

STARTING VALUES FOR THE ADDITIONAL PARAMETERS

NEW/ADDITIONAL PARAMETERS

MIN
0.500

PRIORS FOR ALL PARAMETERS	PRIOR MEAN	PRIOR VARIANCE	PRIOR STD. DEV.
Parameter 1~N(0.000,infinity)	0.0000	infinity	infinity
Parameter 2~N(0.000,infinity)	0.0000	infinity	infinity
Parameter 3~N(0.000,infinity)	0.0000	infinity	infinity
Parameter 4~N(0.000,infinity)	0.0000	infinity	infinity
Parameter 5~N(0.000,infinity)	0.0000	infinity	infinity
Parameter 6~N(0.000,infinity)	0.0000	infinity	infinity
Parameter 7~N(0.000,5.000)	0.0000	5.0000	2.2361
Parameter 8~N(0.000,5.000)	0.0000	5.0000	2.2361
Parameter 9~N(0.000,5.000)	0.0000	5.0000	2.2361
Parameter 10~N(0.000,5.000)	0.0000	5.0000	2.2361
Parameter 11~N(0.000,5.000)	0.0000	5.0000	2.2361
Parameter 12~N(0.000,5.000)	0.0000	5.0000	2.2361
Parameter 13~IW(1.000,7)	infinity	infinity	infinity
Parameter 14~IW(0.000,7)	infinity	infinity	infinity
Parameter 15~IW(1.000,7)	infinity	infinity	infinity
Parameter 16~IW(0.000,7)	infinity	infinity	infinity
Parameter 17~IW(0.000,7)	infinity	infinity	infinity
Parameter 18~IW(1.000,7)	infinity	infinity	infinity
Parameter 19~IW(0.000,7)	infinity	infinity	infinity
Parameter 20~IW(0.000,7)	infinity	infinity	infinity
Parameter 21~IW(0.000,7)	infinity	infinity	infinity
Parameter 22~IW(1.000,7)	infinity	infinity	infinity
Parameter 23~IW(0.000,7)	infinity	infinity	infinity
Parameter 24~IW(0.000,7)	infinity	infinity	infinity
Parameter 25~IW(0.000,7)	infinity	infinity	infinity
Parameter 26~IW(0.000,7)	infinity	infinity	infinity
Parameter 27~IW(1.000,7)	infinity	infinity	infinity

Parameter 28~IW(0.000,7)	infinity	infinity	infinity
Parameter 29~IW(0.000,7)	infinity	infinity	infinity
Parameter 30~IW(0.000,7)	infinity	infinity	infinity
Parameter 31~IW(0.000,7)	infinity	infinity	infinity
Parameter 32~IW(0.000,7)	infinity	infinity	infinity
Parameter 33~IW(1.000,7)	infinity	infinity	infinity
Parameter 34~N(0.000,infinity)	0.0000	infinity	infinity
Parameter 35~N(0.000,infinity)	0.0000	infinity	infinity
Parameter 36~N(0.000,5.000)	0.0000	5.0000	2.2361
Parameter 37~N(0.000,5.000)	0.0000	5.0000	2.2361
Parameter 38~IG(-1.000,0.000)	infinity	infinity	infinity
Parameter 39~IW(1.000,3)	infinity	infinity	infinity
Parameter 40~IW(0.000,3)	infinity	infinity	infinity
Parameter 41~IW(1.000,3)	infinity	infinity	infinity
Parameter 42~N(0.000,5.000)	0.0000	5.0000	2.2361

TECHNICAL 3 OUTPUT

ESTIMATED COVARIANCE MATRIX FOR PARAMETER ESTIMATES

	1	2	3	4	5
1	0.606069D-03				
2	0.104890D-03	0.277347D-03			
3	-0.925403D-04	-0.649467D-04	0.111147D-02		
4	0.452016D-04	0.736602D-04	-0.136547D-03	0.544179D-03	
5	0.101048D-03	0.445892D-04	-0.215373D-03	0.177224D-03	0.108753D-02
6	-0.408600D-04	-0.473529D-04	0.976563D-04	-0.161993D-03	-0.253045D-03
7	-0.273297D-04	-0.151099D-04	-0.675900D-04	-0.118439D-04	0.291519D-04
8	0.652205D-04	-0.145242D-04	0.831992D-04	-0.197003D-04	-0.619981D-04
9	0.145694D-04	0.111113D-04	0.399582D-04	0.158692D-04	0.642973D-04
10	-0.736303D-05	0.319810D-04	0.314773D-04	0.111839D-04	0.413392D-04
11	0.352222D-04	0.115564D-04	-0.182794D-04	-0.128168D-04	0.119608D-04
12	0.353477D-06	0.145086D-04	-0.176458D-04	0.135613D-04	-0.930975D-04
13	0.611674D-04	0.212389D-04	-0.183340D-04	0.535825D-06	0.322988D-04
14	-0.108350D-05	0.118213D-04	-0.293529D-05	-0.151616D-04	-0.196775D-04
15	0.586004D-05	0.141469D-05	0.195638D-04	-0.166343D-04	-0.973881D-05
16	-0.436266D-04	-0.212690D-04	0.227537D-05	0.727469D-05	-0.173843D-04
17	0.588439D-06	0.109626D-04	-0.790011D-05	0.169812D-06	0.138792D-04
18	-0.383215D-04	-0.124767D-05	-0.341716D-04	0.775125D-05	0.209440D-04
19	0.997801D-05	0.174767D-04	0.153506D-04	-0.703240D-05	-0.129206D-04
20	0.517473D-05	0.215723D-05	0.665184D-06	0.934579D-05	0.687300D-05
21	0.829795D-05	-0.828140D-05	0.110280D-04	0.130390D-05	-0.288691D-04

22	0.169984D-04	0.430439D-05	-0.559299D-05	0.192113D-04	0.568025D-04
23	0.254840D-04	0.125192D-04	0.830951D-05	-0.153165D-04	0.219585D-04
24	0.452008D-05	0.211680D-05	0.163332D-04	0.963052D-05	0.159474D-04
25	-0.285335D-05	-0.388003D-05	-0.173839D-04	0.186647D-04	-0.515778D-05
26	-0.327055D-05	-0.116906D-04	0.200027D-05	0.236699D-04	0.304961D-04
27	0.343405D-04	-0.447851D-05	-0.247241D-04	0.183719D-04	0.476718D-04
28	-0.949475D-05	-0.209425D-04	-0.881299D-05	-0.345137D-04	-0.194154D-04
29	-0.212992D-05	0.178703D-05	-0.109494D-04	-0.407937D-05	0.198081D-04
30	-0.608737D-05	-0.126544D-04	-0.169003D-04	0.102245D-04	0.467582D-04
31	-0.881385D-05	0.161787D-05	0.306030D-04	-0.184734D-04	-0.473581D-05
32	-0.428796D-04	0.405135D-05	0.729187D-04	-0.344735D-04	-0.114599D-04
33	-0.109119D-05	0.623437D-05	-0.420158D-04	0.265206D-04	-0.688057D-05
34	-0.201220D-04	-0.334604D-04	0.243957D-04	0.177522D-03	0.437102D-04
35	0.711450D-04	0.171883D-04	-0.192169D-03	-0.103537D-03	0.239088D-04
36	-0.244725D-04	-0.118109D-04	-0.695409D-05	0.194430D-04	0.365180D-04
37	0.125864D-04	0.225382D-04	-0.696985D-05	-0.480394D-05	0.105853D-04
38	0.669076D-05	0.543898D-05	0.546176D-05	0.407664D-04	0.342415D-05
39	0.379783D-04	0.104002D-03	-0.179919D-03	0.119617D-04	0.100496D-05
40	0.304632D-04	0.386017D-05	-0.195559D-03	-0.921123D-06	0.291981D-04
41	-0.268371D-03	-0.145667D-03	-0.161081D-03	-0.110472D-03	0.741815D-03
42	0.563132D-04	0.328600D-04	0.169994D-04	0.308336D-04	0.800645D-04
43	0.158942D-01	0.993566D-02	-0.253594D-02	-0.282695D-02	0.221421D-01

ESTIMATED COVARIANCE MATRIX FOR PARAMETER ESTIMATES

	6	7	8	9	10
6	0.881811D-03				
7	-0.547627D-05	0.259711D-02			
8	-0.892956D-05	-0.566277D-03	0.895226D-02		
9	0.691654D-05	0.814270D-04	0.461983D-03	0.149826D-02	
10	-0.505466D-05	0.141775D-05	-0.233715D-03	0.147746D-03	0.351865D-02
11	-0.173442D-04	-0.276012D-03	-0.116134D-03	0.415091D-04	-0.290443D-03
12	0.180603D-04	-0.799307D-05	0.418092D-03	-0.186593D-03	0.329978D-03
13	-0.161096D-04	0.745511D-04	0.959625D-05	0.651763D-05	-0.227844D-04
14	0.167006D-04	0.259959D-04	-0.394908D-04	0.317758D-05	0.222988D-04
15	0.692744D-05	-0.689304D-05	-0.154392D-04	0.133227D-04	0.361786D-04
16	-0.201680D-04	0.363207D-05	0.112261D-04	0.126420D-04	0.556620D-06
17	-0.197610D-04	0.269839D-05	-0.239176D-04	-0.840953D-05	0.487650D-05
18	-0.428539D-04	0.547701D-04	-0.662488D-04	-0.222629D-04	0.389224D-04
19	0.116505D-04	-0.151475D-04	-0.709900D-05	-0.425310D-06	-0.283490D-04
20	0.329695D-05	-0.982185D-05	0.610696D-04	0.604762D-05	-0.120410D-04
21	0.473448D-05	0.418233D-05	-0.142705D-03	-0.277422D-04	0.104770D-04
22	-0.325295D-04	0.170328D-04	0.592500D-04	-0.395269D-05	-0.288801D-04

23	-0.408335D-05	-0.123538D-04	0.632676D-05	-0.179430D-04	-0.304842D-04
24	-0.331907D-05	0.112336D-04	-0.270266D-04	0.179121D-04	0.145449D-04
25	-0.215206D-04	0.288408D-04	-0.139350D-03	-0.144861D-04	-0.536079D-04
26	-0.220728D-05	0.297358D-04	0.926064D-04	0.426198D-04	0.327975D-06
27	-0.132799D-04	0.116348D-03	0.512918D-04	0.582606D-04	0.744422D-04
28	0.255275D-04	-0.145589D-04	-0.787947D-04	0.320386D-04	0.516944D-04
29	-0.446041D-05	0.199643D-04	-0.501617D-04	0.404423D-05	-0.172363D-04
30	-0.524755D-05	0.289316D-05	0.117868D-03	-0.357455D-06	0.849786D-04
31	0.259528D-05	-0.142079D-04	-0.115800D-03	-0.218783D-04	0.897654D-05
32	-0.582012D-05	0.238407D-04	-0.141204D-03	-0.368022D-04	0.514000D-05
33	0.387351D-04	0.512777D-04	0.119138D-03	0.182041D-04	0.249655D-04
34	-0.121657D-03	0.715890D-04	-0.393937D-03	-0.359125D-03	-0.608335D-04
35	-0.123629D-03	0.219772D-03	-0.470168D-04	-0.102417D-03	-0.474574D-04
36	-0.309295D-04	0.649939D-04	0.545989D-04	-0.710855D-04	-0.921621D-04
37	-0.106092D-04	-0.734076D-04	-0.217956D-03	-0.183766D-03	0.827326D-04
38	0.247016D-04	0.495661D-04	0.133250D-03	0.512590D-04	0.380526D-04
39	-0.711664D-04	-0.652219D-04	0.289423D-03	0.776086D-04	-0.220762D-03
40	-0.242136D-03	0.219476D-03	0.714344D-03	-0.494808D-04	-0.340545D-03
41	-0.359507D-03	-0.123070D-03	-0.115224D-03	-0.241144D-03	-0.259243D-03
42	-0.127185D-04	-0.294366D-03	-0.113856D-02	-0.135411D-03	-0.108940D-03
43	-0.356525D-01	-0.469754D-01	0.842784D-01	-0.521760D-02	-0.227717D-01

ESTIMATED COVARIANCE MATRIX FOR PARAMETER ESTIMATES

	11	12	13	14	15
11	0.160814D-02				
12	0.309063D-03	0.184699D-02			
13	-0.438460D-05	-0.181178D-04	0.887204D-03		
14	0.106958D-04	0.282416D-04	0.144168D-03	0.209518D-03	
15	-0.405845D-05	0.301900D-05	0.245872D-04	0.632900D-04	0.178541D-03
16	0.833025D-05	-0.388715D-04	-0.152810D-03	-0.702332D-04	-0.312448D-04
17	0.398052D-04	-0.180896D-04	-0.515534D-04	-0.385943D-04	-0.503950D-04
18	-0.448481D-04	0.962768D-04	0.342630D-04	0.115795D-04	-0.743134D-05
19	-0.199403D-04	0.938445D-05	0.672088D-04	0.296086D-04	0.604212D-05
20	-0.304971D-04	-0.668374D-05	0.115501D-04	0.103519D-04	0.370384D-04
21	0.301944D-04	0.103319D-04	-0.192693D-04	-0.531391D-05	-0.922817D-05
22	0.186610D-06	0.779087D-05	0.109825D-04	-0.118870D-04	0.892011D-05
23	0.119164D-04	0.416134D-04	0.167285D-03	0.404953D-04	0.600917D-05
24	-0.239400D-04	0.125339D-04	0.306004D-04	0.384751D-04	0.344834D-04
25	0.329231D-04	-0.334657D-04	-0.228347D-04	-0.347908D-04	-0.408033D-05
26	-0.213905D-05	0.184050D-04	0.649637D-05	0.160400D-05	0.119012D-04
27	0.444729D-04	0.831464D-04	0.386250D-04	-0.102826D-04	-0.152221D-04
28	-0.103619D-04	-0.191425D-04	-0.849164D-04	-0.497636D-04	-0.142762D-04

29	0.350939D-04	0.218991D-04	-0.126011D-04	-0.204242D-04	-0.363761D-04
30	0.792322D-06	-0.104751D-05	0.759268D-04	0.268102D-04	0.103322D-04
31	0.192231D-05	0.128960D-05	-0.109871D-04	-0.694713D-05	-0.112139D-04
32	-0.857438D-05	0.208090D-04	0.795404D-05	-0.177063D-04	-0.663095D-06
33	0.219099D-05	0.587042D-06	0.344600D-04	0.562811D-05	0.131856D-05
34	-0.101468D-04	-0.526057D-05	0.128757D-06	0.130054D-04	0.539957D-04
35	-0.650016D-04	0.331414D-03	-0.107134D-03	-0.111041D-03	-0.131637D-03
36	-0.598813D-04	0.443397D-05	-0.374984D-04	-0.204878D-04	0.435721D-05
37	0.738143D-04	0.639100D-04	-0.746698D-04	-0.203760D-04	0.827701D-05
38	0.636064D-04	0.594471D-04	0.567363D-04	0.297647D-04	0.101516D-04
39	0.721709D-04	-0.251257D-04	0.117447D-03	0.184507D-03	0.163420D-03
40	-0.369044D-03	-0.224595D-03	0.278407D-03	0.108785D-03	-0.489982D-05
41	-0.426667D-03	0.109114D-03	-0.252568D-03	-0.110338D-03	0.550372D-04
42	0.414029D-04	0.104520D-03	-0.328487D-04	-0.338372D-04	-0.156176D-04
43	0.315429D-01	0.112067D-02	0.303610D-03	0.102241D-01	0.252866D-02

ESTIMATED COVARIANCE MATRIX FOR PARAMETER ESTIMATES

	16	17	18	19	20
16	0.769016D-03				
17	0.156207D-03	0.354720D-03			
18	-0.227012D-03	-0.189492D-03	0.243856D-02		
19	-0.865660D-04	-0.203766D-04	0.315804D-04	0.382766D-03	
20	-0.222572D-04	-0.469570D-04	-0.743455D-05	0.626871D-04	0.180146D-03
21	0.291528D-04	0.785591D-04	-0.291574D-03	-0.493652D-04	-0.695878D-04
22	-0.111645D-04	-0.191158D-04	0.505655D-04	0.403960D-04	0.785318D-04
23	-0.105870D-03	-0.310492D-04	0.595192D-04	0.140411D-03	0.267869D-04
24	-0.243656D-04	-0.662477D-04	0.113748D-04	0.237662D-04	0.630438D-04
25	0.126704D-03	0.658587D-04	-0.445705D-03	-0.673502D-06	-0.137370D-04
26	-0.289881D-05	-0.195014D-04	0.622148D-04	0.585900D-04	0.497112D-04
27	-0.681585D-05	-0.268233D-04	0.178524D-03	0.227351D-04	0.208899D-04
28	0.981634D-04	0.245293D-04	-0.701009D-04	-0.123485D-03	-0.325806D-04
29	0.251541D-04	0.505788D-04	0.577364D-05	-0.167958D-04	-0.546281D-04
30	-0.612796D-04	-0.710996D-04	0.278619D-03	0.387074D-04	0.125572D-04
31	0.718654D-05	0.232372D-04	-0.158045D-05	-0.352299D-04	-0.495729D-04
32	0.706974D-05	0.328403D-05	-0.325227D-04	-0.122240D-04	-0.577707D-05
33	-0.293294D-05	-0.142171D-04	-0.379616D-04	-0.591372D-07	0.177210D-04
34	0.107814D-03	-0.140331D-03	0.306002D-03	0.967462D-04	0.255529D-05
35	0.851150D-04	0.169492D-03	0.477318D-04	0.159284D-04	-0.116436D-03
36	0.534592D-04	0.621172D-04	-0.865688D-04	-0.138426D-05	0.965404D-05
37	-0.114260D-04	-0.664626D-05	0.202200D-05	-0.190801D-04	-0.358777D-05
38	-0.209158D-04	-0.812055D-05	0.766970D-04	0.108711D-05	0.331463D-05
39	-0.168424D-03	-0.243308D-03	0.147288D-03	-0.780160D-04	-0.133393D-03

40	-0.807029D-04	-0.812896D-05	-0.160491D-03	0.940761D-04	0.477274D-04
41	0.157159D-03	-0.185853D-04	-0.935243D-03	-0.303311D-03	-0.137481D-03
42	-0.492619D-04	-0.718461D-05	-0.648364D-04	0.247782D-04	0.156228D-05
43	0.891353D-02	0.963491D-02	-0.634568D-01	0.100952D-01	-0.843894D-02

ESTIMATED COVARIANCE MATRIX FOR PARAMETER ESTIMATES

	21	22	23	24	25
21	0.679791D-03				
22	-0.137505D-03	0.652660D-03			
23	-0.299776D-04	0.352291D-04	0.732792D-03		
24	-0.239266D-04	0.149461D-04	0.119156D-03	0.338061D-03	
25	0.221866D-03	-0.480972D-04	-0.103079D-03	-0.980232D-04	0.129234D-02
26	-0.139739D-03	0.196822D-03	0.696386D-04	0.830265D-04	-0.137398D-03
27	-0.645590D-04	0.875603D-04	0.222561D-03	0.134551D-03	-0.394986D-03
28	0.286508D-04	-0.259977D-04	-0.206210D-03	-0.374909D-04	0.515165D-04
29	0.956591D-05	-0.238952D-04	-0.251796D-04	-0.784307D-04	0.282681D-04
30	-0.168720D-03	0.559311D-04	0.104468D-03	0.317103D-04	-0.300935D-03
31	0.801613D-04	-0.186266D-03	-0.155541D-04	-0.182815D-04	0.402104D-04
32	0.216885D-04	-0.449599D-04	-0.836030D-04	-0.752769D-04	0.166227D-03
33	-0.190080D-04	0.252090D-04	0.296324D-04	0.414216D-04	-0.419334D-04
34	-0.220885D-03	0.879558D-04	-0.616986D-04	0.613595D-04	-0.160852D-03
35	0.726461D-04	0.141928D-03	0.440886D-04	-0.218313D-04	-0.235778D-03
36	-0.139475D-04	0.268521D-04	-0.914595D-04	0.444965D-05	-0.558480D-05
37	-0.277335D-04	-0.144748D-04	-0.555155D-04	-0.667879D-05	-0.225169D-04
38	0.126637D-04	0.973406D-05	0.202760D-04	0.122054D-04	0.354172D-05
39	0.195563D-03	-0.339089D-04	0.327593D-04	0.335036D-04	0.229925D-03
40	0.435897D-04	-0.273973D-03	-0.126757D-03	-0.108702D-03	0.323066D-03
41	0.543816D-04	0.169057D-04	-0.100136D-03	0.212473D-03	-0.223107D-03
42	0.678429D-05	-0.423372D-04	0.957579D-06	0.422890D-05	0.261690D-04
43	0.170287D-01	0.963447D-02	0.606873D-02	0.210379D-01	0.587736D-02

ESTIMATED COVARIANCE MATRIX FOR PARAMETER ESTIMATES

	26	27	28	29	30
26	0.650058D-03				
27	0.437865D-03	0.241818D-02			
28	-0.322529D-04	-0.687110D-04	0.686011D-03		
29	-0.368431D-04	-0.517487D-04	0.112370D-03	0.287862D-03	
30	0.842508D-04	0.150279D-03	-0.120944D-03	-0.785299D-04	0.112434D-02
31	-0.185721D-03	-0.123262D-03	0.743574D-04	0.729797D-04	-0.167898D-03
32	-0.234899D-03	-0.575813D-03	0.152286D-03	0.920662D-04	-0.231380D-03

33	0.832355D-04	0.153448D-03	-0.160468D-03	-0.131426D-03	0.271629D-03
34	0.673208D-04	-0.670576D-04	0.253560D-04	0.444467D-06	0.577610D-04
35	0.140268D-03	-0.970137D-04	-0.620743D-04	0.368088D-04	0.990886D-04
36	0.495342D-04	0.600329D-05	0.385570D-04	-0.436591D-05	-0.686404D-04
37	0.157002D-05	-0.234441D-04	0.298399D-04	0.300692D-05	-0.381689D-04
38	-0.210701D-04	-0.139717D-04	-0.264818D-04	-0.601512D-05	0.453890D-04
39	0.781943D-04	-0.292351D-03	0.263874D-05	-0.112284D-03	-0.140140D-04
40	-0.676336D-04	-0.209171D-03	0.189398D-03	0.639469D-04	-0.247936D-03
41	0.469645D-03	0.755315D-03	0.185481D-03	0.187722D-03	-0.316191D-03
42	-0.395881D-04	-0.295964D-04	0.964902D-06	0.352980D-05	-0.172744D-04
43	-0.232780D-01	-0.324004D-01	-0.481723D-02	0.456028D-02	-0.292732D-01

ESTIMATED COVARIANCE MATRIX FOR PARAMETER ESTIMATES

	31	32	33	34	35
31	0.597729D-03				
32	0.241922D-03	0.115788D-02			
33	-0.349920D-03	-0.537298D-03	0.195424D-02		
34	0.623475D-04	-0.195941D-03	0.582793D-04	0.231672D-01	
35	-0.796920D-04	-0.646826D-04	0.540747D-04	-0.315707D-02	0.446903D-01
36	-0.202233D-04	-0.130833D-05	-0.108797D-03	-0.129327D-04	0.446550D-04
37	-0.498101D-05	0.285147D-04	0.298244D-04	0.878372D-05	0.218059D-03
38	-0.462827D-06	-0.113448D-04	0.332437D-04	-0.345522D-04	0.525404D-04
39	0.412223D-04	-0.444040D-04	0.268717D-03	-0.201006D-03	-0.758775D-03
40	-0.692459D-04	0.263943D-03	0.123790D-03	0.159806D-02	-0.202303D-03
41	0.532094D-03	-0.195481D-03	-0.524885D-03	-0.213367D-02	0.133078D-02
42	0.708742D-05	0.671802D-04	0.102299D-04	0.111512D-03	-0.844166D-04
43	-0.145781D-01	-0.228040D-01	0.666282D-02	-0.271262D-01	0.113707D+00

ESTIMATED COVARIANCE MATRIX FOR PARAMETER ESTIMATES

	36	37	38	39	40
36	0.258271D-02				
37	0.389545D-03	0.168300D-02			
38	-0.274512D-03	-0.192755D-03	0.715394D-03		
39	0.237366D-03	0.274980D-04	-0.997583D-04	0.660430D-01	
40	0.432998D-03	0.161306D-03	-0.170522D-03	-0.897464D-02	0.580166D-01
41	-0.254195D-05	0.676289D-03	-0.395870D-03	0.645498D-02	-0.985188D-02
42	0.141125D-03	0.169368D-02	-0.285727D-03	0.521515D-03	0.219736D-04
43	0.369492D-01	0.400301D-01	-0.784983D-02	-0.121247D-01	-0.623898D-02

ESTIMATED COVARIANCE MATRIX FOR PARAMETER ESTIMATES

	41	42	43
41	0.199492D+00		
42	0.767440D-03	0.467649D-02	
43	-0.120918D+00	0.399233D-01	0.532563D+03

ESTIMATED CORRELATION MATRIX FOR PARAMETER ESTIMATES

	1	2	3	4	5
1	1.000				
2	0.256	1.000			
3	-0.113	-0.117	1.000		
4	0.079	0.190	-0.176	1.000	
5	0.124	0.081	-0.196	0.230	1.000
6	-0.056	-0.096	0.099	-0.234	-0.258
7	-0.022	-0.018	-0.040	-0.010	0.017
8	0.028	-0.009	0.026	-0.009	-0.020
9	0.015	0.017	0.031	0.018	0.050
10	-0.005	0.032	0.016	0.008	0.021
11	0.036	0.017	-0.014	-0.014	0.009
12	0.000	0.020	-0.012	0.014	-0.066
13	0.083	0.043	-0.018	0.001	0.033
14	-0.003	0.049	-0.006	-0.045	-0.041
15	0.018	0.006	0.044	-0.053	-0.022
16	-0.064	-0.046	0.002	0.011	-0.019
17	0.001	0.035	-0.013	0.000	0.022
18	-0.032	-0.002	-0.021	0.007	0.013
19	0.021	0.054	0.024	-0.015	-0.020
20	0.016	0.010	0.001	0.030	0.016
21	0.013	-0.019	0.013	0.002	-0.034
22	0.027	0.010	-0.007	0.032	0.067
23	0.038	0.028	0.009	-0.024	0.025
24	0.010	0.007	0.027	0.022	0.026
25	-0.003	-0.006	-0.015	0.022	-0.004
26	-0.005	-0.028	0.002	0.040	0.036
27	0.028	-0.005	-0.015	0.016	0.029
28	-0.015	-0.048	-0.010	-0.056	-0.022
29	-0.005	0.006	-0.019	-0.010	0.035
30	-0.007	-0.023	-0.015	0.013	0.042
31	-0.015	0.004	0.038	-0.032	-0.006
32	-0.051	0.007	0.064	-0.043	-0.010
33	-0.001	0.008	-0.029	0.026	-0.005

34	-0.005	-0.013	0.005	0.050	0.009
35	0.014	0.005	-0.027	-0.021	0.003
36	-0.020	-0.014	-0.004	0.016	0.022
37	0.012	0.033	-0.005	-0.005	0.008
38	0.010	0.012	0.006	0.065	0.004
39	0.006	0.024	-0.021	0.002	0.000
40	0.005	0.001	-0.024	0.000	0.004
41	-0.024	-0.020	-0.011	-0.011	0.050
42	0.033	0.029	0.007	0.019	0.036
43	0.028	0.026	-0.003	-0.005	0.029

ESTIMATED CORRELATION MATRIX FOR PARAMETER ESTIMATES

	6	7	8	9	10
6	1.000				
7	-0.004	1.000			
8	-0.003	-0.117	1.000		
9	0.006	0.041	0.126	1.000	
10	-0.003	0.000	-0.042	0.064	1.000
11	-0.015	-0.135	-0.031	0.027	-0.122
12	0.014	-0.004	0.103	-0.112	0.129
13	-0.018	0.049	0.003	0.006	-0.013
14	0.039	0.035	-0.029	0.006	0.026
15	0.017	-0.010	-0.012	0.026	0.046
16	-0.024	0.003	0.004	0.012	0.000
17	-0.035	0.003	-0.013	-0.012	0.004
18	-0.029	0.022	-0.014	-0.012	0.013
19	0.020	-0.015	-0.004	-0.001	-0.024
20	0.008	-0.014	0.048	0.012	-0.015
21	0.006	0.003	-0.058	-0.027	0.007
22	-0.043	0.013	0.025	-0.004	-0.019
23	-0.005	-0.009	0.002	-0.017	-0.019
24	-0.006	0.012	-0.016	0.025	0.013
25	-0.020	0.016	-0.041	-0.010	-0.025
26	-0.003	0.023	0.038	0.043	0.000
27	-0.009	0.046	0.011	0.031	0.026
28	0.033	-0.011	-0.032	0.032	0.033
29	-0.009	0.023	-0.031	0.006	-0.017
30	-0.005	0.002	0.037	0.000	0.043
31	0.004	-0.011	-0.050	-0.023	0.006
32	-0.006	0.014	-0.044	-0.028	0.003
33	0.030	0.023	0.028	0.011	0.010
34	-0.027	0.009	-0.027	-0.061	-0.007

35	-0.020	0.020	-0.002	-0.013	-0.004
36	-0.020	0.025	0.011	-0.036	-0.031
37	-0.009	-0.035	-0.056	-0.116	0.034
38	0.031	0.036	0.053	0.050	0.024
39	-0.009	-0.005	0.012	0.008	-0.014
40	-0.034	0.018	0.031	-0.005	-0.024
41	-0.027	-0.005	-0.003	-0.014	-0.010
42	-0.006	-0.084	-0.176	-0.051	-0.027
43	-0.052	-0.040	0.039	-0.006	-0.017

ESTIMATED CORRELATION MATRIX FOR PARAMETER ESTIMATES

	11	12	13	14	15
11	1.000				
12	0.179	1.000			
13	-0.004	-0.014	1.000		
14	0.018	0.045	0.334	1.000	
15	-0.008	0.005	0.062	0.327	1.000
16	0.007	-0.033	-0.185	-0.175	-0.084
17	0.053	-0.022	-0.092	-0.142	-0.200
18	-0.023	0.045	0.023	0.016	-0.011
19	-0.025	0.011	0.115	0.105	0.023
20	-0.057	-0.012	0.029	0.053	0.207
21	0.029	0.009	-0.025	-0.014	-0.026
22	0.000	0.007	0.014	-0.032	0.026
23	0.011	0.036	0.207	0.103	0.017
24	-0.032	0.016	0.056	0.145	0.140
25	0.023	-0.022	-0.021	-0.067	-0.008
26	-0.002	0.017	0.009	0.004	0.035
27	0.023	0.039	0.026	-0.014	-0.023
28	-0.010	-0.017	-0.109	-0.131	-0.041
29	0.052	0.030	-0.025	-0.083	-0.160
30	0.001	-0.001	0.076	0.055	0.023
31	0.002	0.001	-0.015	-0.020	-0.034
32	-0.006	0.014	0.008	-0.036	-0.001
33	0.001	0.000	0.026	0.009	0.002
34	-0.002	-0.001	0.000	0.006	0.027
35	-0.008	0.036	-0.017	-0.036	-0.047
36	-0.029	0.002	-0.025	-0.028	0.006
37	0.045	0.036	-0.061	-0.034	0.015
38	0.059	0.052	0.071	0.077	0.028
39	0.007	-0.002	0.015	0.050	0.048
40	-0.038	-0.022	0.039	0.031	-0.002

41	-0.024	0.006	-0.019	-0.017	0.009
42	0.015	0.036	-0.016	-0.034	-0.017
43	0.034	0.001	0.000	0.031	0.008

ESTIMATED CORRELATION MATRIX FOR PARAMETER ESTIMATES

	16	17	18	19	20
16	1.000				
17	0.299	1.000			
18	-0.166	-0.204	1.000		
19	-0.160	-0.055	0.033	1.000	
20	-0.060	-0.186	-0.011	0.239	1.000
21	0.040	0.160	-0.226	-0.097	-0.199
22	-0.016	-0.040	0.040	0.081	0.229
23	-0.141	-0.061	0.045	0.265	0.074
24	-0.048	-0.191	0.013	0.066	0.255
25	0.127	0.097	-0.251	-0.001	-0.028
26	-0.004	-0.041	0.049	0.117	0.145
27	-0.005	-0.029	0.074	0.024	0.032
28	0.135	0.050	-0.054	-0.241	-0.093
29	0.053	0.158	0.007	-0.051	-0.240
30	-0.066	-0.113	0.168	0.059	0.028
31	0.011	0.050	-0.001	-0.074	-0.151
32	0.007	0.005	-0.019	-0.018	-0.013
33	-0.002	-0.017	-0.017	0.000	0.030
34	0.026	-0.049	0.041	0.032	0.001
35	0.015	0.043	0.005	0.004	-0.041
36	0.038	0.065	-0.034	-0.001	0.014
37	-0.010	-0.009	0.001	-0.024	-0.007
38	-0.028	-0.016	0.058	0.002	0.009
39	-0.024	-0.050	0.012	-0.016	-0.039
40	-0.012	-0.002	-0.013	0.020	0.015
41	0.013	-0.002	-0.042	-0.035	-0.023
42	-0.026	-0.006	-0.019	0.019	0.002
43	0.014	0.022	-0.056	0.022	-0.027

ESTIMATED CORRELATION MATRIX FOR PARAMETER ESTIMATES

	21	22	23	24	25
21	1.000				
22	-0.206	1.000			
23	-0.042	0.051	1.000		

24	-0.050	0.032	0.239	1.000	
25	0.237	-0.052	-0.106	-0.148	1.000
26	-0.210	0.302	0.101	0.177	-0.150
27	-0.050	0.070	0.167	0.149	-0.223
28	0.042	-0.039	-0.291	-0.078	0.055
29	0.022	-0.055	-0.055	-0.251	0.046
30	-0.193	0.065	0.115	0.051	-0.250
31	0.126	-0.298	-0.024	-0.041	0.046
32	0.024	-0.052	-0.091	-0.120	0.136
33	-0.016	0.022	0.025	0.051	-0.026
34	-0.056	0.023	-0.015	0.022	-0.029
35	0.013	0.026	0.008	-0.006	-0.031
36	-0.011	0.021	-0.066	0.005	-0.003
37	-0.026	-0.014	-0.050	-0.009	-0.015
38	0.018	0.014	0.028	0.025	0.004
39	0.029	-0.005	0.005	0.007	0.025
40	0.007	-0.045	-0.019	-0.025	0.037
41	0.005	0.001	-0.008	0.026	-0.014
42	0.004	-0.024	0.001	0.003	0.011
43	0.028	0.016	0.010	0.050	0.007

ESTIMATED CORRELATION MATRIX FOR PARAMETER ESTIMATES

	26	27	28	29	30
26	1.000				
27	0.349	1.000			
28	-0.048	-0.053	1.000		
29	-0.085	-0.062	0.253	1.000	
30	0.099	0.091	-0.138	-0.138	1.000
31	-0.298	-0.103	0.116	0.176	-0.205
32	-0.271	-0.344	0.171	0.159	-0.203
33	0.074	0.071	-0.139	-0.175	0.183
34	0.017	-0.009	0.006	0.000	0.011
35	0.026	-0.009	-0.011	0.010	0.014
36	0.038	0.002	0.029	-0.005	-0.040
37	0.002	-0.012	0.028	0.004	-0.028
38	-0.031	-0.011	-0.038	-0.013	0.051
39	0.012	-0.023	0.000	-0.026	-0.002
40	-0.011	-0.018	0.030	0.016	-0.031
41	0.041	0.034	0.016	0.025	-0.021
42	-0.023	-0.009	0.001	0.003	-0.008
43	-0.040	-0.029	-0.008	0.012	-0.038

ESTIMATED CORRELATION MATRIX FOR PARAMETER ESTIMATES

	31	32	33	34	35
31	1.000				
32	0.291	1.000			
33	-0.324	-0.357	1.000		
34	0.017	-0.038	0.009	1.000	
35	-0.015	-0.009	0.006	-0.098	1.000
36	-0.016	-0.001	-0.048	-0.002	0.004
37	-0.005	0.020	0.016	0.001	0.025
38	-0.001	-0.012	0.028	-0.008	0.009
39	0.007	-0.005	0.024	-0.005	-0.014
40	-0.012	0.032	0.012	0.044	-0.004
41	0.049	-0.013	-0.027	-0.031	0.014
42	0.004	0.029	0.003	0.011	-0.006
43	-0.026	-0.029	0.007	-0.008	0.023

ESTIMATED CORRELATION MATRIX FOR PARAMETER ESTIMATES

	36	37	38	39	40
36	1.000				
37	0.187	1.000			
38	-0.202	-0.176	1.000		
39	0.018	0.003	-0.015	1.000	
40	0.035	0.016	-0.026	-0.145	1.000
41	0.000	0.037	-0.033	0.056	-0.092
42	0.041	0.604	-0.156	0.030	0.001
43	0.032	0.042	-0.013	-0.002	-0.001

ESTIMATED CORRELATION MATRIX FOR PARAMETER ESTIMATES

	41	42	43
41	1.000		
42	0.025	1.000	
43	-0.012	0.025	1.000

TECHNICAL 8 OUTPUT

TECHNICAL 8 OUTPUT FOR BAYES ESTIMATION

CHAIN	BSEED
1	0
2	285380
3	253358

ITERATION	POTENTIAL SCALE REDUCTION	PARAMETER WITH HIGHEST PSR
100	1.017	13
200	1.016	38
300	1.005	13
400	1.004	37
500	1.007	38
600	1.004	38
700	1.002	6
800	1.003	42
900	1.005	42
1000	1.001	42

Simple model, maximum likelihood estimation, deterioration in physical health

COVARIANCE COVERAGE OF DATA

Minimum covariance coverage value   0.100

UNIVARIATE PROPORTIONS AND COUNTS FOR CATEGORICAL VARIABLES

```
    PHYCAT16
      Category 1    0.881         4884.000
      Category 2    0.119          662.000
```

UNIVARIATE SAMPLE STATISTICS

UNIVARIATE HIGHER-ORDER MOMENT DESCRIPTIVE STATISTICS

```
         Variable/         Mean/     Skewness/   Minimum/ % with                Percentiles
        Sample Size      Variance    Kurtosis    Maximum  Min/Max      20%/60%    40%/80%    Median

     PRAFAC              -0.185       0.245      -2.338    1.72%      -1.448     -0.527     -0.318
              58.000       1.345      -0.636       2.338    1.72%       0.080      0.752
```

THE MODEL ESTIMATION TERMINATED NORMALLY

MODEL FIT INFORMATION

```
Number of Free Parameters                        3

Loglikelihood

          H0 Value                        -2021.083
          H0 Scaling Correction Factor      2.0801
            for MLR
```

Information Criteria

```
          Akaike (AIC)                    4048.165
          Bayesian (BIC)                  4068.028
          Sample-Size Adjusted BIC        4058.495
            (n* = (n + 2) / 24)



MODEL RESULTS

                                                    Two-Tailed
                    Estimate       S.E.  Est./S.E.    P-Value

Within Level

Between Level

 PHYCAT16   ON
    PRAFAC            -0.164      0.049     -3.348      0.001

 Thresholds
    PHYCAT16$1         2.038      0.078     26.164      0.000

 Residual Variances
    PHYCAT16           0.029      0.082      0.357      0.721


RESULTS IN PROBABILITY SCALE

                    Estimate

Within Level


Between Level

 PHYCAT16
    Category 1         0.883
    Category 2         0.117


QUALITY OF NUMERICAL RESULTS

     Condition Number for the Information Matrix              0.157E+00
       (ratio of smallest to largest eigenvalue)
```

```
TECHNICAL 1 OUTPUT


     PARAMETER SPECIFICATION FOR WITHIN


           TAU
              PHYCAT16
              ________
                     0


           NU
              PHYCAT16
              ________
                     0


           LAMBDA
              PHYCAT16
              ________
 PHYCAT16            0


           THETA
              PHYCAT16
              ________
 PHYCAT16            0


           ALPHA
              PHYCAT16
              ________
                     0


           BETA
              PHYCAT16
              ________
 PHYCAT16            0
```

```
           PSI
              PHYCAT16
              ________
 PHYCAT16            0


     PARAMETER SPECIFICATION FOR BETWEEN


           TAU
              PHYCAT16
              ________
                     3


           NU
              PHYCAT16      PRAFAC
              ________      ________
                     0             0


           LAMBDA
              PHYCAT16      PRAFAC
              ________      ________
 PHYCAT16            0             0
 PRAFAC              0             0


           THETA
              PHYCAT16      PRAFAC
              ________      ________
 PHYCAT16            0
 PRAFAC              0             0


           ALPHA
              PHYCAT16      PRAFAC
              ________      ________
                     0             0


           BETA
              PHYCAT16      PRAFAC
              ________      ________
```

```
PHYCAT16           0             1
PRAFAC             0             0


          PSI
             PHYCAT16      PRAFAC
             ________      ________
PHYCAT16           2
PRAFAC             0             0


    STARTING VALUES FOR WITHIN


          TAU
             PHYCAT16
             ________
               0.000


          NU
             PHYCAT16
             ________
               0.000


          LAMBDA
             PHYCAT16
             ________
PHYCAT16       1.000


          THETA
             PHYCAT16
             ________
PHYCAT16       0.000


          ALPHA
             PHYCAT16
             ________
               0.000
```

```
           BETA
              PHYCAT16
              ________
 PHYCAT16         0.000


           PSI
              PHYCAT16
              ________
 PHYCAT16         1.000


     STARTING VALUES FOR BETWEEN


           TAU
              PHYCAT16
              ________
                  1.998


           NU
              PHYCAT16      PRAFAC
              ________      ________
                  0.000         0.000


           LAMBDA
              PHYCAT16      PRAFAC
              ________      ________
 PHYCAT16         1.000         0.000
 PRAFAC           0.000         1.000


           THETA
              PHYCAT16      PRAFAC
              ________      ________
 PHYCAT16         0.000
 PRAFAC           0.000         0.000


           ALPHA
              PHYCAT16      PRAFAC
              ________      ________
```

```
                0.000         0.000


           BETA
              PHYCAT16      PRAFAC
              ________      ________
 PHYCAT16       0.000         0.000
 PRAFAC         0.000         0.000


           PSI
              PHYCAT16      PRAFAC
              ________      ________
 PHYCAT16       1.000
 PRAFAC         0.000         0.504
```

TECHNICAL 3 OUTPUT

```
           ESTIMATED COVARIANCE MATRIX FOR PARAMETER ESTIMATES
                      1             2             3
              ________      ________      ________
        1   0.238946D-02
        2  -0.235560D-02  0.664368D-02
        3  -0.156323D-02  0.478324D-02  0.606795D-02


           ESTIMATED CORRELATION MATRIX FOR PARAMETER ESTIMATES
                      1             2             3
              ________      ________      ________
        1       1.000
        2      -0.591         1.000
        3      -0.411         0.753         1.000
```

TECHNICAL 8 OUTPUT

```
   E STEP  ITER  LOGLIKELIHOOD    ABS CHANGE   REL CHANGE  ALGORITHM
              1 -0.20458672D+04    0.0000000    0.0000000  EM
              2 -0.20252002D+04   20.6669185    0.0101018  FS
              3 -0.20213261D+04    3.8740890    0.0019129  FS
              4 -0.20212521D+04    0.0740163    0.0000366  FS
```

5	-0.20212181D+04	0.0339911	0.0000168	FS
6	-0.20211873D+04	0.0308504	0.0000153	FS
7	-0.20211605D+04	0.0267960	0.0000133	FS
8	-0.20211385D+04	0.0219717	0.0000109	FS
9	-0.20211214D+04	0.0171106	0.0000085	FS
10	-0.20211087D+04	0.0127337	0.0000063	FS
11	-0.20210996D+04	0.0091095	0.0000045	FS
12	-0.20210933D+04	0.0062983	0.0000031	FS
13	-0.20210890D+04	0.0042299	0.0000021	FS
14	-0.20210863D+04	0.0027719	0.0000014	FS
15	-0.20210845D+04	0.0017799	0.0000009	FS
16	-0.20210834D+04	0.0011239	0.0000006	FS
17	-0.20210827D+04	0.0007001	0.0000003	FS

Simple model, maximum likelihood estimation, reduced psychological distress (linear and curvilinear effects)

COVARIANCE COVERAGE OF DATA

Minimum covariance coverage value   0.100

UNIVARIATE PROPORTIONS AND COUNTS FOR CATEGORICAL VARIABLES

KESCAT16		
Category 1	0.347	386.000
Category 2	0.653	727.000

UNIVARIATE SAMPLE STATISTICS

UNIVARIATE HIGHER-ORDER MOMENT DESCRIPTIVE STATISTICS

Variable/ Sample Size	Mean/ Variance	Skewness/ Kurtosis	Minimum/ Maximum	% with Min/Max	Percentiles 20%/60%	40%/80%	Median
PRAFAC	-0.185	0.245	-2.338	1.72%	-1.448	-0.527	-0.318
58.000	1.345	-0.636	2.338	1.72%	0.080	0.752	
PRAFACSQ	1.379	1.240	0.002	1.72%	0.151	0.381	0.681
58.000	2.369	0.557	5.467	1.72%	1.168	2.366	

THE MODEL ESTIMATION TERMINATED NORMALLY

MODEL FIT INFORMATION

Number of Free Parameters   4

Loglikelihood

H0 Value	-710.220
H0 Scaling Correction Factor for MLR	0.6696

```
Information Criteria

          Akaike (AIC)                    1428.441
          Bayesian (BIC)                  1448.500
          Sample-Size Adjusted BIC        1435.795
            (n* = (n + 2) / 24)



MODEL RESULTS

                                                    Two-Tailed
                    Estimate       S.E.  Est./S.E.    P-Value

Within Level

Between Level

 KESCAT16   ON
    PRAFAC            -0.219      0.064     -3.414      0.001
    PRAFACSQ          -0.146      0.056     -2.624      0.009

 Thresholds
    KESCAT16$1        -0.770      0.065    -11.927      0.000

 Residual Variances
    KESCAT16           0.000      0.000      0.526      0.599

RESULTS IN PROBABILITY SCALE

                    Estimate

Within Level


Between Level

 KESCAT16
    Category 1         0.347
    Category 2         0.653


STANDARDIZED MODEL RESULTS
```

```
STDYX Standardization

                                                    Two-Tailed
                    Estimate       S.E.  Est./S.E.    P-Value

Within Level

Between Level

 KESCAT16   ON
    PRAFAC            -0.774      0.167     -4.619      0.000
    PRAFACSQ          -0.685      0.188     -3.636      0.000

 Thresholds
    KESCAT16$1        -2.350      0.566     -4.154      0.000

 Residual Variances
    KESCAT16           0.000      0.000      0.576      0.565


R-SQUARE

Within Level

Between Level

    Observed                                        Two-Tailed
    Variable        Estimate       S.E.  Est./S.E.    P-Value

    KESCAT16           1.000      0.000   3933.540      0.000


QUALITY OF NUMERICAL RESULTS

     Condition Number for the Information Matrix              0.575E-04
       (ratio of smallest to largest eigenvalue)


TECHNICAL 1 OUTPUT


     PARAMETER SPECIFICATION FOR WITHIN
```

```
          TAU
             KESCAT16
             ________
                    0


          NU
             KESCAT16
             ________
                    0


          LAMBDA
             KESCAT16
             ________
KESCAT16            0


          THETA
             KESCAT16
             ________
KESCAT16            0


          ALPHA
             KESCAT16
             ________
                    0


          BETA
             KESCAT16
             ________
KESCAT16            0


          PSI
             KESCAT16
             ________
KESCAT16            0
```

PARAMETER SPECIFICATION FOR BETWEEN

TAU

	KESCAT16
	4

NU

KESCAT16	PRAFAC	PRAFACSQ
0	0	0

LAMBDA

	KESCAT16	PRAFAC	PRAFACSQ
KESCAT16	0	0	0
PRAFAC	0	0	0
PRAFACSQ	0	0	0

THETA

	KESCAT16	PRAFAC	PRAFACSQ
KESCAT16	0		
PRAFAC	0	0	
PRAFACSQ	0	0	0

ALPHA

KESCAT16	PRAFAC	PRAFACSQ
0	0	0

BETA

	KESCAT16	PRAFAC	PRAFACSQ
KESCAT16	0	1	2
PRAFAC	0	0	0
PRAFACSQ	0	0	0

```
           PSI
              KESCAT16      PRAFAC        PRAFACSQ
              ________      ________      ________
 KESCAT16           3
 PRAFAC             0             0
 PRAFACSQ           0             0             0


     STARTING VALUES FOR WITHIN


           TAU
              KESCAT16
              ________
                0.000


           NU
              KESCAT16
              ________
                0.000


           LAMBDA
              KESCAT16
              ________
 KESCAT16       1.000


           THETA
              KESCAT16
              ________
 KESCAT16       0.000


           ALPHA
              KESCAT16
              ________
                0.000


           BETA
              KESCAT16
```

```
              ________
 KESCAT16       0.000


           PSI
              KESCAT16
              ________
 KESCAT16       1.000


     STARTING VALUES FOR BETWEEN


           TAU
              KESCAT16
              ________
               -0.633


           NU
              KESCAT16      PRAFAC        PRAFACSQ
              ________      ________      ________
                0.000         0.000         0.000


           LAMBDA
              KESCAT16      PRAFAC        PRAFACSQ
              ________      ________      ________
 KESCAT16       1.000         0.000         0.000
 PRAFAC         0.000         1.000         0.000
 PRAFACSQ       0.000         0.000         1.000


           THETA
              KESCAT16      PRAFAC        PRAFACSQ
              ________      ________      ________
 KESCAT16       0.000
 PRAFAC         0.000         0.000
 PRAFACSQ       0.000         0.000         0.000


           ALPHA
              KESCAT16      PRAFAC        PRAFACSQ
              ________      ________      ________
```

	0.000	0.000	0.000

BETA

	KESCAT16	PRAFAC	PRAFACSQ
KESCAT16	0.000	0.000	0.000
PRAFAC	0.000	0.000	0.000
PRAFACSQ	0.000	0.000	0.000

PSI

	KESCAT16	PRAFAC	PRAFACSQ
KESCAT16	1.000		
PRAFAC	0.000	0.469	
PRAFACSQ	0.000	0.000	0.654

TECHNICAL 3 OUTPUT

ESTIMATED COVARIANCE MATRIX FOR PARAMETER ESTIMATES

	1	2	3	4
1	0.410390D-02			
2	0.416890D-03	0.309108D-02		
3	-0.973636D-06	-0.307842D-06	0.893592D-09	
4	0.305265D-03	0.218999D-02	0.450982D-06	0.417206D-02

ESTIMATED CORRELATION MATRIX FOR PARAMETER ESTIMATES

	1	2	3	4
1	1.000			
2	0.117	1.000		
3	-0.508	-0.185	1.000	
4	0.074	0.610	0.234	1.000

TECHNICAL 8 OUTPUT

E STEP ITER LOGLIKELIHOOD ABS CHANGE REL CHANGE ALGORITHM

1	-0.72971915D+03	0.0000000	0.0000000	EM
2	-0.71559020D+03	14.1289541	0.0193622	FS
3	-0.71238856D+03	3.2016327	0.0044741	FS
4	-0.71230692D+03	0.0816451	0.0001146	FS
5	-0.71112637D+03	1.1805481	0.0016574	FS
6	-0.71072634D+03	0.4000244	0.0005625	EM
7	-0.71050218D+03	0.2241602	0.0003154	EM
8	-0.71037151D+03	0.1306764	0.0001839	EM
9	-0.71029865D+03	0.0728608	0.0001026	EM
10	-0.71025984D+03	0.0388080	0.0000546	EM
11	-0.71023986D+03	0.0199763	0.0000281	EM
12	-0.71022980D+03	0.0100662	0.0000142	EM
13	-0.71022478D+03	0.0050125	0.0000071	EM
14	-0.71022230D+03	0.0024804	0.0000035	EM
15	-0.71022108D+03	0.0012235	0.0000017	EM
16	-0.71022048D+03	0.0006026	0.0000008	EM

Deterioration in physical health, job control interaction

SUMMARY OF DATA

Number of clusters 58

Size (s)	Cluster ID with Size s
7	22
16	166
23	80 71 205
25	74
27	192
31	65
32	106
35	228
37	170 72
39	50 75 181
40	182
42	165
44	114
45	173
46	107
47	169 30
48	226 81
49	36
50	116 108 40
56	162 84
57	61
59	206 93 83
60	92
61	69
63	6
64	152
65	145
66	4
68	68
69	154
72	174
78	98
84	178 2
95	156 202
96	144
108	142

131	122
172	24
175	209
224	204
269	125
364	217
738	110
860	100

COVARIANCE COVERAGE OF DATA

Minimum covariance coverage value 0.100

Number of missing data patterns 8

PROPORTION OF DATA PRESENT

Covariance Coverage

	PHYCAT16	PRAFAC	PHY15	KES15	CHOICE15
PHYCAT16	1.000				
PRAFAC	1.000	1.000			
PHY15	0.947	0.947	0.947		
KES15	0.922	0.922	0.922	0.922	
CHOICE15	0.915	0.915	0.915	0.915	0.915
BULLY15	0.915	0.915	0.915	0.915	0.915
PRESS15	0.915	0.915	0.915	0.915	0.915
DUTIES15	0.915	0.915	0.915	0.915	0.915

Covariance Coverage

	BULLY15	PRESS15	DUTIES15
BULLY15	0.915		
PRESS15	0.914	0.915	
DUTIES15	0.915	0.915	0.915

UNIVARIATE PROPORTIONS AND COUNTS FOR CATEGORICAL VARIABLES

PHYCAT16

Category 1	0.881	4884.000
Category 2	0.119	662.000

UNIVARIATE SAMPLE STATISTICS

UNIVARIATE HIGHER-ORDER MOMENT DESCRIPTIVE STATISTICS

Variable/ Sample Size	Mean/ Variance	Skewness/ Kurtosis	Minimum/ Maximum	% with Min/Max	Percentiles 20%/60%	Percentiles 40%/80%	Median
PRAFAC	-0.185	0.245	-2.338	1.72%	-1.448	-0.527	-0.318
58.000	1.345	-0.636	2.338	1.72%	0.080	0.752	
PHY15	0.000	-0.546	-0.632	36.83%	-0.632	0.368	0.368
5254.000	0.233	-1.702	0.368	63.17%	0.368	0.368	
KES15	0.000	1.717	-0.511	21.65%	-0.511	-0.178	-0.178
5114.000	0.289	3.835	3.489	0.04%	-0.011	0.322	
CHOICE15	3.225	-0.402	1.000	7.41%	2.000	3.000	3.000
5077.000	1.081	-0.336	5.000	8.49%	4.000	4.000	
BULLY15	0.000	3.272	-0.170	87.25%	-0.170	-0.170	-0.170
5073.000	0.239	11.772	3.830	0.08%	-0.170	-0.170	
PRESS15	0.000	0.252	-1.462	19.10%	-0.462	-0.462	-0.462
5074.000	1.001	-0.371	2.538	2.76%	0.538	0.538	
DUTIES15	0.000	-1.246	-3.120	2.40%	-0.120	-0.120	-0.120
5077.000	0.861	1.673	0.880	38.74%	-0.120	0.880	

THE MODEL ESTIMATION TERMINATED NORMALLY

USE THE FBITERATIONS OPTION TO INCREASE THE NUMBER OF ITERATIONS BY A FACTOR
OF AT LEAST TWO TO CHECK CONVERGENCE AND THAT THE PSR VALUE DOES NOT INCREASE.

MODEL FIT INFORMATION

Number of Free Parameters 43

MODEL RESULTS

	Estimate	Posterior S.D.	One-Tailed P-Value	95% C.I. Lower 2.5%	Upper 2.5%	Significance
**Within Level**						
PHYCAT16 ON						
PHY15	0.976	0.070	0.000	0.834	1.118	*
BULLY15	0.029	0.050	0.276	-0.071	0.126	
DUTIES15	-0.032	0.026	0.126	-0.084	0.020	
PRESS15	-0.033	0.027	0.121	-0.086	0.019	
KES15	0.227	0.045	0.000	0.140	0.314	*
KES15 WITH						
PHY15	0.051	0.004	0.000	0.044	0.059	*
CHOICE15	-0.088	0.008	0.000	-0.103	-0.072	*
BULLY15	0.057	0.004	0.000	0.050	0.066	*
PRESS15	0.099	0.007	0.000	0.085	0.115	*
DUTIES15	-0.106	0.007	0.000	-0.120	-0.091	*
PHY15 WITH						
CHOICE15	-0.033	0.007	0.000	-0.047	-0.020	*
BULLY15	0.012	0.003	0.000	0.005	0.018	*
PRESS15	0.038	0.007	0.000	0.025	0.052	*
DUTIES15	-0.036	0.006	0.000	-0.049	-0.024	*
CHOICE15 WITH						
BULLY15	-0.056	0.007	0.000	-0.070	-0.042	*
PRESS15	-0.075	0.015	0.000	-0.107	-0.047	*
DUTIES15	0.169	0.013	0.000	0.142	0.196	*
BULLY15 WITH						
PRESS15	0.105	0.007	0.000	0.091	0.118	*
DUTIES15	-0.078	0.006	0.000	-0.090	-0.065	*
PRESS15 WITH						
DUTIES15	-0.185	0.014	0.000	-0.213	-0.159	*
Means						
PHY15	0.002	0.007	0.380	-0.011	0.016	
KES15	0.001	0.008	0.430	-0.014	0.017	
BULLY15	0.001	0.007	0.467	-0.013	0.016	
PRESS15	0.001	0.014	0.483	-0.025	0.030	
DUTIES15	0.000	0.013	0.496	-0.026	0.024	

Variances						
PHY15	0.233	0.005	0.000	0.225	0.242	*
KES15	0.290	0.006	0.000	0.278	0.302	*
CHOICE15	1.041	0.020	0.000	1.003	1.080	*
BULLY15	0.239	0.005	0.000	0.230	0.249	*
PRESS15	1.001	0.020	0.000	0.963	1.044	*
DUTIES15	0.861	0.018	0.000	0.829	0.897	*

Between Level

S1 ON						
PRAFAC	0.051	0.028	0.031	-0.003	0.107	
PHYCAT16 ON						
PRAFAC	-0.079	0.031	0.006	-0.140	-0.017	*
CHOICE15	-0.165	0.173	0.179	-0.500	0.162	
PRAFAC WITH						
CHOICE15	0.039	0.045	0.183	-0.050	0.128	
Means						
PRAFAC	-0.182	0.155	0.122	-0.484	0.116	
CHOICE15	3.311	0.038	0.000	3.237	3.384	*
Intercepts						
S1	-0.052	0.031	0.047	-0.110	0.009	
Thresholds						
PHYCAT16$1	0.816	0.567	0.075	-0.285	1.881	
Variances						
PRAFAC	1.348	0.260	0.000	0.954	1.984	*
CHOICE15	0.061	0.014	0.000	0.040	0.094	*
Residual Variances						
PHYCAT16	0.015	0.012	0.000	0.001	0.048	*
S1	0.004	0.005	0.000	0.000	0.020	*

STANDARDIZED MODEL RESULTS

STDYX Standardization

	Estimate	Posterior S.D.	One-Tailed P-Value	95% C.I. Lower 2.5%	Upper 2.5%	Significance
**Within-Level Standardized Estimates Averaged Over Clusters**						
S1 | PHYCAT16 ON						
CHOICE15	-0.055	0.026	0.020	-0.105	-0.002	*
PHYCAT16 ON						
PHY15	0.416	0.025	0.000	0.365	0.464	*
BULLY15	0.013	0.022	0.276	-0.030	0.055	
DUTIES15	-0.026	0.022	0.122	-0.070	0.018	
PRESS15	-0.029	0.024	0.120	-0.076	0.018	
KES15	0.108	0.021	0.000	0.066	0.149	*
KES15 WITH						
PHY15	0.198	0.013	0.000	0.171	0.223	*
CHOICE15	-0.160	0.014	0.000	-0.186	-0.132	*
BULLY15	0.219	0.014	0.000	0.191	0.245	*
PRESS15	0.185	0.013	0.000	0.160	0.211	*
DUTIES15	-0.212	0.014	0.000	-0.240	-0.184	*
PHY15 WITH						
CHOICE15	-0.066	0.014	0.000	-0.094	-0.038	*
BULLY15	0.050	0.014	0.000	0.022	0.076	*
PRESS15	0.080	0.014	0.000	0.053	0.107	*
DUTIES15	-0.081	0.014	0.000	-0.108	-0.053	*
CHOICE15 WITH						
BULLY15	-0.112	0.014	0.000	-0.138	-0.085	*
PRESS15	-0.074	0.015	0.000	-0.103	-0.047	*
DUTIES15	0.179	0.014	0.000	0.151	0.206	*
BULLY15 WITH						
PRESS15	0.214	0.013	0.000	0.188	0.240	*
DUTIES15	-0.171	0.013	0.000	-0.197	-0.145	*
PRESS15 WITH						
DUTIES15	-0.199	0.013	0.000	-0.226	-0.173	*
Means						
PHY15	0.004	0.014	0.385	-0.023	0.032	
KES15	0.003	0.014	0.426	-0.025	0.030	

```
    BULLY15            0.001       0.014       0.471      -0.025       0.029
    PRESS15            0.000       0.014       0.484      -0.026       0.029
    DUTIES15          -0.001       0.014       0.488      -0.028       0.025

 Variances
    PHY15              1.000       0.000       0.000       1.000       1.000
    KES15              1.000       0.000       0.000       1.000       1.000
    CHOICE15           1.000       0.000       0.000       1.000       1.000
    BULLY15            1.000       0.000       0.000       1.000       1.000
    PRESS15            1.000       0.000       0.000       1.000       1.000
    DUTIES15           1.000       0.000       0.000       1.000       1.000

Between Level

 S1         ON
    PRAFAC             0.695       0.281       0.031      -0.046       0.989

 PHYCAT16   ON
    PRAFAC            -0.557       0.196       0.006      -0.908      -0.129      *
    CHOICE15          -0.250       0.245       0.179      -0.679       0.266

 PRAFAC   WITH
    CHOICE15           0.138       0.145       0.183      -0.167       0.401

 Means
    PRAFAC            -0.159       0.133       0.122      -0.418       0.108
    CHOICE15          13.371       1.438       0.000      10.853      16.556      *

 Intercepts
    S1                -0.598       0.489       0.047      -1.878       0.080

 Variances
    PRAFAC             1.000       0.000       0.000       1.000       1.000
    CHOICE15           1.000       0.000       0.000       1.000       1.000

 Residual Variances
    PHYCAT16           0.535       0.213       0.000       0.064       0.907      *
    S1                 0.517       0.290       0.000       0.022       0.994      *


R-SQUARE

Within-Level R-Square Averaged Across Clusters
```

Variable	Estimate	Posterior S.D.	One-Tailed P-Value	95% C.I. Lower 2.5%	Upper 2.5%
PHYCAT16	0.222	0.022	0.000	0.181	0.267

Between Level

Variable	Estimate	Posterior S.D.	One-Tailed P-Value	95% C.I. Lower 2.5%	Upper 2.5%
PHYCAT16	0.465	0.213	0.000	0.093	0.936

Variable	Estimate	Posterior S.D.	One-Tailed P-Value	95% C.I. Lower 2.5%	Upper 2.5%
S1	0.483	0.290	0.000	0.006	0.978

TECHNICAL 1 OUTPUT

PARAMETER SPECIFICATION FOR WITHIN

TAU

PHYCAT16
0

NU

PHYCAT16	PHY15	KES15	CHOICE15	BULLY15
0	0	0	0	0

NU

PRESS15	DUTIES15
0	0

LAMBDA

PHYCAT16	PHY15	KES15	CHOICE15	BULLY15

PHYCAT16	0	0	0	0	0
PHY15	0	0	0	0	0
KES15	0	0	0	0	0
CHOICE15	0	0	0	0	0
BULLY15	0	0	0	0	0
PRESS15	0	0	0	0	0
DUTIES15	0	0	0	0	0

LAMBDA

	PRESS15	DUTIES15
PHYCAT16	0	0
PHY15	0	0
KES15	0	0
CHOICE15	0	0
BULLY15	0	0
PRESS15	0	0
DUTIES15	0	0

THETA

	PHYCAT16	PHY15	KES15	CHOICE15	BULLY15
PHYCAT16	0				
PHY15	0	0			
KES15	0	0	0		
CHOICE15	0	0	0	0	
BULLY15	0	0	0	0	0
PRESS15	0	0	0	0	0
DUTIES15	0	0	0	0	0

THETA

	PRESS15	DUTIES15
PRESS15	0	
DUTIES15	0	0

ALPHA

PHYCAT16	PHY15	KES15	CHOICE15	BULLY15

0	1	2	0	3

ALPHA

PRESS15	DUTIES15
4	5

BETA

	PHYCAT16	PHY15	KES15	CHOICE15	BULLY15
PHYCAT16	0	6	7	0	8
PHY15	0	0	0	0	0
KES15	0	0	0	0	0
CHOICE15	0	0	0	0	0
BULLY15	0	0	0	0	0
PRESS15	0	0	0	0	0
DUTIES15	0	0	0	0	0

BETA

	PRESS15	DUTIES15
PHYCAT16	9	10
PHY15	0	0
KES15	0	0
CHOICE15	0	0
BULLY15	0	0
PRESS15	0	0
DUTIES15	0	0

PSI

	PHYCAT16	PHY15	KES15	CHOICE15	BULLY15
PHYCAT16	0				
PHY15	0	11			
KES15	0	12	13		
CHOICE15	0	14	15	16	
BULLY15	0	17	18	19	20
PRESS15	0	21	22	23	24
DUTIES15	0	26	27	28	29

PSI

	PRESS15	DUTIES15
PRESS15	25	
DUTIES15	30	31

PARAMETER SPECIFICATION FOR BETWEEN

TAU

PHYCAT16
43

NU

PHYCAT16	PRAFAC	CHOICE15
0	0	0

LAMBDA

	S1	PHYCAT16	PRAFAC	CHOICE15
PHYCAT16	0	0	0	0
PRAFAC	0	0	0	0
CHOICE15	0	0	0	0

THETA

	PHYCAT16	PRAFAC	CHOICE15
PHYCAT16	0		
PRAFAC	0	0	
CHOICE15	0	0	0

ALPHA

S1	PHYCAT16	PRAFAC	CHOICE15
32	0	33	34

BETA

	S1	PHYCAT16	PRAFAC	CHOICE15
S1	0	0	35	0
PHYCAT16	0	0	36	37
PRAFAC	0	0	0	0
CHOICE15	0	0	0	0

PSI

	S1	PHYCAT16	PRAFAC	CHOICE15
S1	38			
PHYCAT16	0	39		
PRAFAC	0	0	40	
CHOICE15	0	0	41	42

STARTING VALUES FOR WITHIN

TAU

PHYCAT16
0.000

NU

PHYCAT16	PHY15	KES15	CHOICE15	BULLY15
0.000	0.000	0.000	0.000	0.000

NU

PRESS15	DUTIES15
0.000	0.000

LAMBDA

	PHYCAT16	PHY15	KES15	CHOICE15	BULLY15
PHYCAT16	1.000	0.000	0.000	0.000	0.000

PHY15	0.000	1.000	0.000	0.000	0.000
KES15	0.000	0.000	1.000	0.000	0.000
CHOICE15	0.000	0.000	0.000	1.000	0.000
BULLY15	0.000	0.000	0.000	0.000	1.000
PRESS15	0.000	0.000	0.000	0.000	0.000
DUTIES15	0.000	0.000	0.000	0.000	0.000

LAMBDA

	PRESS15	DUTIES15
PHYCAT16	0.000	0.000
PHY15	0.000	0.000
KES15	0.000	0.000
CHOICE15	0.000	0.000
BULLY15	0.000	0.000
PRESS15	1.000	0.000
DUTIES15	0.000	1.000

THETA

	PHYCAT16	PHY15	KES15	CHOICE15	BULLY15
PHYCAT16	0.000				
PHY15	0.000	0.000			
KES15	0.000	0.000	0.000		
CHOICE15	0.000	0.000	0.000	0.000	
BULLY15	0.000	0.000	0.000	0.000	0.000
PRESS15	0.000	0.000	0.000	0.000	0.000
DUTIES15	0.000	0.000	0.000	0.000	0.000

THETA

	PRESS15	DUTIES15
PRESS15	0.000	
DUTIES15	0.000	0.000

ALPHA

PHYCAT16	PHY15	KES15	CHOICE15	BULLY15
0.000	0.000	0.000	0.000	0.000

ALPHA

PRESS15	DUTIES15
0.000	0.000

BETA

	PHYCAT16	PHY15	KES15	CHOICE15	BULLY15
PHYCAT16	0.000	0.000	0.000	0.000	0.000
PHY15	0.000	0.000	0.000	0.000	0.000
KES15	0.000	0.000	0.000	0.000	0.000
CHOICE15	0.000	0.000	0.000	0.000	0.000
BULLY15	0.000	0.000	0.000	0.000	0.000
PRESS15	0.000	0.000	0.000	0.000	0.000
DUTIES15	0.000	0.000	0.000	0.000	0.000

BETA

	PRESS15	DUTIES15
PHYCAT16	0.000	0.000
PHY15	0.000	0.000
KES15	0.000	0.000
CHOICE15	0.000	0.000
BULLY15	0.000	0.000
PRESS15	0.000	0.000
DUTIES15	0.000	0.000

PSI

	PHYCAT16	PHY15	KES15	CHOICE15	BULLY15
PHYCAT16	1.000				
PHY15	0.000	0.116			
KES15	0.000	0.000	0.145		
CHOICE15	0.000	0.000	0.000	0.541	
BULLY15	0.000	0.000	0.000	0.000	0.119
PRESS15	0.000	0.000	0.000	0.000	0.000
DUTIES15	0.000	0.000	0.000	0.000	0.000

PSI

```
              PRESS15       DUTIES15
              ________      ________
 PRESS15        0.501
 DUTIES15       0.000         0.430


     STARTING VALUES FOR BETWEEN


           TAU
              PHYCAT16
              ________
                1.110


           NU
              PHYCAT16      PRAFAC        CHOICE15
              ________      ________      ________
                0.000         0.000         0.000


           LAMBDA
              S1            PHYCAT16      PRAFAC        CHOICE15
              ________      ________      ________      ________
 PHYCAT16       0.000         1.000         0.000         0.000
 PRAFAC         0.000         0.000         1.000         0.000
 CHOICE15       0.000         0.000         0.000         1.000


           THETA
              PHYCAT16      PRAFAC        CHOICE15
              ________      ________      ________
 PHYCAT16       0.000
 PRAFAC         0.000         0.000
 CHOICE15       0.000         0.000         0.000


           ALPHA
              S1            PHYCAT16      PRAFAC        CHOICE15
              ________      ________      ________      ________
                0.000         0.000         0.020         3.225


           BETA
```

	S1	PHYCAT16	PRAFAC	CHOICE15
S1	0.000	0.000	0.000	0.000
PHYCAT16	0.000	0.000	0.000	0.000
PRAFAC	0.000	0.000	0.000	0.000
CHOICE15	0.000	0.000	0.000	0.000

PSI

	S1	PHYCAT16	PRAFAC	CHOICE15
S1	1.000			
PHYCAT16	0.000	1.000		
PRAFAC	0.000	0.000	0.504	
CHOICE15	0.000	0.000	0.000	0.541

PRIORS FOR ALL PARAMETERS	PRIOR MEAN	PRIOR VARIANCE	PRIOR STD. DEV.
Parameter 1~N(0.000,infinity)	0.0000	infinity	infinity
Parameter 2~N(0.000,infinity)	0.0000	infinity	infinity
Parameter 3~N(0.000,infinity)	0.0000	infinity	infinity
Parameter 4~N(0.000,infinity)	0.0000	infinity	infinity
Parameter 5~N(0.000,infinity)	0.0000	infinity	infinity
Parameter 6~N(0.000,5.000)	0.0000	5.0000	2.2361
Parameter 7~N(0.000,5.000)	0.0000	5.0000	2.2361
Parameter 8~N(0.000,5.000)	0.0000	5.0000	2.2361
Parameter 9~N(0.000,5.000)	0.0000	5.0000	2.2361
Parameter 10~N(0.000,5.000)	0.0000	5.0000	2.2361
Parameter 11~IW(1.000,7)	infinity	infinity	infinity
Parameter 12~IW(0.000,7)	infinity	infinity	infinity
Parameter 13~IW(1.000,7)	infinity	infinity	infinity
Parameter 14~IW(0.000,7)	infinity	infinity	infinity
Parameter 15~IW(0.000,7)	infinity	infinity	infinity
Parameter 16~IW(1.000,7)	infinity	infinity	infinity
Parameter 17~IW(0.000,7)	infinity	infinity	infinity
Parameter 18~IW(0.000,7)	infinity	infinity	infinity
Parameter 19~IW(0.000,7)	infinity	infinity	infinity
Parameter 20~IW(1.000,7)	infinity	infinity	infinity
Parameter 21~IW(0.000,7)	infinity	infinity	infinity
Parameter 22~IW(0.000,7)	infinity	infinity	infinity
Parameter 23~IW(0.000,7)	infinity	infinity	infinity
Parameter 24~IW(0.000,7)	infinity	infinity	infinity

```
     Parameter 25~IW(1.000,7)             infinity            infinity            infinity
     Parameter 26~IW(0.000,7)             infinity            infinity            infinity
     Parameter 27~IW(0.000,7)             infinity            infinity            infinity
     Parameter 28~IW(0.000,7)             infinity            infinity            infinity
     Parameter 29~IW(0.000,7)             infinity            infinity            infinity
     Parameter 30~IW(0.000,7)             infinity            infinity            infinity
     Parameter 31~IW(1.000,7)             infinity            infinity            infinity
     Parameter 32~N(0.000,infinity)         0.0000            infinity            infinity
     Parameter 33~N(0.000,infinity)         0.0000            infinity            infinity
     Parameter 34~N(0.000,infinity)         0.0000            infinity            infinity
     Parameter 35~N(0.000,infinity)         0.0000            infinity            infinity
     Parameter 36~N(0.000,5.000)            0.0000              5.0000              2.2361
     Parameter 37~N(0.000,5.000)            0.0000              5.0000              2.2361
     Parameter 38~IG(-1.000,0.000)        infinity            infinity            infinity
     Parameter 39~IG(-1.000,0.000)        infinity            infinity            infinity
     Parameter 40~IW(1.000,3)             infinity            infinity            infinity
     Parameter 41~IW(0.000,3)             infinity            infinity            infinity
     Parameter 42~IW(1.000,3)             infinity            infinity            infinity
     Parameter 43~N(0.000,5.000)            0.0000              5.0000              2.2361


TECHNICAL 3 OUTPUT


           ESTIMATED COVARIANCE MATRIX FOR PARAMETER ESTIMATES
                  1             2             3             4             5
              ________      ________      ________      ________      ________
        1   0.454691D-04
        2   0.104443D-04  0.607964D-04
        3   0.253943D-05  0.159997D-04  0.518004D-04
        4   0.587797D-05  0.142389D-04  0.233568D-04  0.195336D-03
        5  -0.560729D-05 -0.204433D-04 -0.202960D-04 -0.395810D-04  0.166271D-03
        6   0.137722D-04  0.460475D-06  0.234392D-04  0.960195D-05 -0.383595D-04
        7  -0.174102D-04  0.827155D-05 -0.123303D-05 -0.157704D-04  0.365434D-05
        8  -0.313247D-05 -0.141137D-04 -0.105993D-04  0.116281D-05 -0.507916D-05
        9   0.960104D-05  0.288043D-05 -0.111806D-05 -0.385139D-05  0.573227D-05
       10   0.598596D-05 -0.363500D-05 -0.106854D-07 -0.151493D-04  0.110673D-04
       11   0.649792D-06  0.148156D-06  0.245956D-06 -0.963076D-06  0.248842D-06
       12  -0.113425D-05 -0.103347D-05 -0.177841D-06 -0.186949D-05 -0.334109D-06
       13   0.805237D-06  0.110503D-05 -0.375797D-07  0.639633D-06 -0.176556D-05
       14   0.338951D-06 -0.264334D-06 -0.197824D-05  0.987403D-06  0.206000D-05
       15   0.101008D-05 -0.190279D-05 -0.154049D-05 -0.476841D-05  0.867856D-06
       16  -0.691243D-05 -0.143592D-06  0.399554D-05  0.322467D-05  0.740681D-05
       17   0.787755D-07  0.628729D-06  0.156957D-06 -0.173404D-05  0.102000D-05
```

18	-0.123416D-07	0.896028D-06	0.239822D-06	0.925562D-06	-0.398504D-06
19	0.965545D-07	-0.346435D-06	0.190927D-05	0.152427D-05	-0.255991D-06
20	0.921546D-06	0.246699D-05	0.119178D-05	0.333772D-06	-0.215414D-05
21	-0.391402D-06	0.208494D-05	0.185204D-05	0.752272D-07	-0.284686D-06
22	-0.845270D-06	-0.302273D-06	0.130358D-05	0.121996D-05	-0.876353D-06
23	-0.243234D-05	-0.177275D-05	0.297802D-07	-0.455417D-06	-0.922852D-05
24	0.106001D-05	0.100754D-05	-0.107280D-06	0.592066D-07	0.267103D-05
25	-0.107475D-05	-0.446969D-06	0.211208D-06	0.335843D-05	0.294722D-05
26	0.215822D-05	-0.125489D-05	-0.293973D-05	0.856478D-06	0.295398D-05
27	0.221211D-05	0.470331D-06	0.117977D-05	-0.281172D-05	0.129658D-05
28	-0.422918D-05	0.308911D-05	0.136868D-05	0.660841D-05	-0.132863D-06
29	0.945206D-06	-0.407389D-06	0.117772D-05	-0.502565D-05	0.234882D-05
30	0.152254D-05	0.241615D-05	-0.301703D-06	-0.245670D-05	-0.576479D-05
31	-0.464009D-05	0.523897D-06	-0.173044D-05	0.100616D-04	-0.126543D-04
32	-0.269638D-05	0.399628D-05	-0.119911D-05	-0.693038D-05	0.646013D-05
33	-0.286313D-04	-0.464533D-04	0.375387D-04	-0.432381D-04	0.266107D-04
34	-0.178936D-04	-0.253374D-04	-0.163992D-04	-0.187378D-04	0.468343D-04
35	0.294544D-05	-0.540719D-05	0.898124D-05	0.307340D-05	0.512694D-05
36	-0.367673D-05	0.918229D-05	0.165302D-04	-0.102266D-04	0.708285D-05
37	-0.783606D-05	0.398230D-04	0.771537D-04	0.847492D-04	-0.954338D-04
38	-0.152095D-05	0.348898D-06	0.110321D-05	-0.312390D-05	0.220587D-05
39	0.537810D-06	-0.278808D-05	-0.919943D-06	-0.436543D-05	-0.358996D-05
40	-0.414918D-04	-0.621235D-04	0.342249D-04	0.148251D-03	-0.538322D-04
41	-0.369010D-05	-0.362369D-05	-0.599362D-05	0.111371D-04	-0.608216D-05
42	-0.305367D-05	-0.127644D-05	-0.366688D-05	-0.230899D-05	-0.224511D-05
43	-0.198527D-04	0.130628D-03	0.254775D-03	0.283340D-03	-0.325337D-03

ESTIMATED COVARIANCE MATRIX FOR PARAMETER ESTIMATES

	6	7	8	9	10
6	0.492439D-02				
7	-0.326117D-03	0.205785D-02			
8	0.561745D-04	-0.404623D-03	0.249451D-02		
9	-0.112200D-04	-0.116708D-03	-0.285577D-03	0.718120D-03	
10	0.951383D-04	0.134584D-03	0.120326D-03	0.109229D-03	0.697183D-03
11	0.494043D-05	0.116645D-04	-0.775199D-05	0.122440D-05	0.114456D-05
12	-0.812879D-05	0.764244D-05	-0.271658D-07	-0.901588D-06	-0.325052D-05
13	-0.117686D-04	0.895915D-05	-0.542853D-05	0.198295D-05	-0.400948D-05
14	0.316467D-05	-0.693447D-05	0.171329D-04	-0.515937D-05	-0.108322D-05
15	0.343218D-04	-0.101872D-04	0.132654D-04	-0.295332D-05	0.337139D-05
16	0.394390D-05	0.479182D-04	0.199765D-04	-0.639397D-05	0.705799D-05
17	-0.288868D-05	0.746530D-06	-0.424395D-05	-0.561114D-07	-0.199096D-05
18	-0.481466D-05	0.766836D-05	-0.333524D-05	0.121071D-05	-0.281379D-05

19	0.256405D-04	-0.102780D-04	-0.284475D-05	0.624288D-05	0.988860D-06
20	-0.950807D-05	0.818356D-05	0.321507D-06	0.211493D-05	0.134431D-05
21	-0.166546D-04	-0.789874D-06	-0.276980D-05	0.557951D-05	-0.354565D-05
22	-0.148976D-04	0.113807D-04	-0.256999D-05	0.213271D-05	0.892143D-06
23	0.407405D-04	-0.219267D-04	0.372572D-06	0.121792D-04	0.281197D-06
24	-0.838944D-05	0.119172D-04	-0.123185D-04	0.500303D-05	0.732847D-05
25	-0.414661D-04	0.224046D-04	-0.131365D-04	0.183254D-05	0.143002D-04
26	0.142043D-04	-0.321734D-07	0.124634D-04	-0.346344D-05	0.599761D-05
27	0.154800D-04	-0.901334D-05	-0.818997D-05	0.249544D-05	0.232474D-05
28	-0.265652D-04	0.127163D-04	0.159990D-04	-0.723143D-05	-0.898890D-05
29	0.809967D-05	-0.200943D-04	0.151952D-04	-0.314069D-05	0.665936D-05
30	0.335051D-04	-0.140232D-04	0.218104D-04	-0.611043D-05	0.851295D-05
31	0.927097D-05	0.119516D-04	-0.147972D-04	0.401503D-05	-0.385769D-05
32	-0.104064D-03	0.211681D-03	-0.570423D-05	0.386999D-04	-0.558923D-04
33	0.370820D-04	0.208149D-03	-0.738808D-04	-0.249649D-04	-0.418369D-04
34	0.344277D-04	0.475733D-04	-0.442992D-04	-0.107558D-04	0.140733D-04
35	-0.538224D-04	-0.239443D-04	-0.706178D-04	0.268599D-04	-0.128986D-04
36	-0.872403D-04	0.843436D-04	-0.122030D-04	-0.642031D-04	0.133083D-04
37	0.540859D-03	-0.240427D-03	0.465469D-03	-0.333077D-04	-0.128760D-03
38	0.148041D-04	-0.734712D-05	-0.107798D-06	0.127148D-05	-0.296971D-05
39	0.327472D-04	0.333766D-04	-0.570054D-05	0.124287D-04	0.194979D-05
40	0.393718D-04	0.384340D-04	0.211031D-03	-0.144659D-03	-0.463176D-04
41	-0.258327D-05	-0.495559D-04	0.826381D-05	0.402470D-04	-0.454862D-05
42	0.845992D-05	-0.203276D-04	0.117833D-04	0.730639D-05	-0.256064D-06
43	0.278407D-02	-0.695031D-03	0.149218D-02	-0.116376D-03	-0.404980D-03

ESTIMATED COVARIANCE MATRIX FOR PARAMETER ESTIMATES

	11	12	13	14	15
11	0.209437D-04				
12	0.454878D-05	0.135737D-04			
13	0.534435D-06	0.640152D-05	0.342940D-04		
14	-0.339521D-05	-0.391609D-05	-0.361321D-05	0.496486D-04	
15	0.260113D-06	-0.320456D-05	-0.131631D-04	0.115753D-04	0.651023D-04
16	0.258163D-05	0.141549D-05	0.629134D-05	-0.128881D-04	-0.341771D-04
17	0.650850D-06	0.269101D-05	0.104617D-05	-0.269313D-05	-0.437740D-06
18	-0.175074D-06	0.151121D-05	0.744062D-05	-0.206441D-05	-0.538087D-05
19	-0.100140D-05	-0.131110D-05	-0.255563D-05	0.370448D-05	0.119414D-04
20	0.837109D-06	0.382700D-06	0.181092D-05	0.207660D-06	-0.591744D-06
21	0.317534D-05	0.530669D-05	0.313526D-05	-0.416754D-05	-0.200409D-05
22	0.292266D-05	0.471544D-05	0.123890D-04	-0.385557D-05	-0.926242D-05
23	-0.152551D-05	-0.311895D-05	-0.698573D-05	0.867290D-05	0.216087D-04
24	0.581395D-06	0.172907D-05	0.362361D-05	-0.149987D-05	-0.313515D-05

25	0.380952D-05	0.303383D-05	0.853824D-05	-0.816471D-05	-0.793711D-05
26	-0.422405D-05	-0.609947D-05	-0.269305D-05	0.881972D-05	0.157529D-05
27	-0.184387D-05	-0.482482D-05	-0.150501D-04	0.504338D-05	0.136247D-04
28	0.139288D-05	0.232104D-05	0.568177D-05	-0.794128D-05	-0.260227D-04
29	-0.174100D-05	-0.217546D-05	-0.351486D-05	0.271557D-05	0.401675D-05
30	-0.230074D-05	-0.217245D-05	-0.711049D-05	0.191946D-05	0.514019D-05
31	0.320914D-05	0.319811D-05	0.875906D-05	-0.196047D-05	-0.108522D-04
32	0.386128D-05	0.404755D-05	0.102349D-04	-0.693393D-05	-0.239775D-05
33	0.181843D-04	0.207017D-04	0.863613D-06	0.209327D-04	0.576996D-04
34	-0.557290D-06	0.313996D-05	0.799752D-05	-0.299148D-05	-0.336520D-05
35	-0.608051D-05	-0.161105D-05	0.948129D-05	-0.374963D-05	-0.247783D-04
36	-0.121830D-05	0.516304D-05	0.147951D-05	-0.203628D-05	-0.105960D-04
37	0.845630D-05	-0.112051D-04	-0.278463D-04	-0.290440D-05	0.767121D-05
38	0.231601D-06	0.159818D-06	0.906666D-06	0.101264D-05	0.889551D-07
39	0.260189D-05	0.803612D-06	-0.236787D-06	-0.271864D-05	0.257548D-05
40	0.146597D-04	-0.620477D-05	0.150468D-04	-0.821638D-05	-0.453758D-04
41	-0.447270D-05	-0.193324D-05	-0.170780D-05	-0.520924D-05	0.644993D-05
42	-0.440639D-06	-0.576025D-06	-0.117339D-05	-0.409472D-05	-0.238035D-05
43	0.359334D-04	-0.406278D-04	-0.991366D-04	-0.119668D-04	0.434625D-04

ESTIMATED COVARIANCE MATRIX FOR PARAMETER ESTIMATES

	16	17	18	19	20
16	0.391675D-03				
17	-0.658887D-06	0.104563D-04			
18	0.557769D-05	0.226695D-05	0.148948D-04		
19	-0.257825D-04	-0.807841D-06	-0.466087D-05	0.500599D-04	
20	0.813597D-06	0.575234D-06	0.483825D-05	-0.495151D-05	0.223020D-04
21	0.178976D-05	0.502418D-05	0.223888D-05	-0.268180D-05	0.145749D-05
22	0.126954D-04	0.186753D-05	0.792421D-05	-0.327409D-05	0.167838D-05
23	-0.391022D-04	-0.466603D-05	-0.223986D-05	0.262545D-04	-0.623366D-06
24	0.302535D-05	0.166972D-05	0.598701D-05	-0.561396D-05	0.836888D-05
25	0.792586D-05	0.219931D-05	0.525146D-05	-0.388177D-05	0.489674D-05
26	-0.430062D-05	-0.432114D-05	-0.117975D-05	0.169163D-05	0.102284D-06
27	-0.112339D-04	-0.971162D-06	-0.627764D-05	0.466077D-05	-0.238176D-05
28	0.590284D-04	0.177436D-05	0.409077D-05	-0.195184D-04	-0.129615D-05
29	-0.334085D-05	-0.191716D-05	-0.599874D-05	0.100224D-04	-0.732123D-05
30	-0.192525D-05	-0.547264D-06	-0.549520D-05	0.986225D-05	-0.447318D-05
31	0.120832D-04	0.260901D-06	0.352846D-05	-0.911460D-05	0.209991D-05
32	-0.364423D-05	0.140415D-05	0.527157D-05	-0.206337D-05	0.355490D-05
33	0.622345D-04	-0.108258D-05	-0.239407D-04	0.715251D-04	-0.918294D-05
34	0.654863D-05	0.301961D-05	0.409780D-05	0.511988D-05	-0.312642D-05
35	0.205281D-04	-0.524456D-06	0.318715D-05	-0.195192D-05	-0.299428D-05

36	-0.842091D-05	-0.101870D-05	-0.126153D-05	-0.179204D-05	0.163916D-05
37	0.148879D-04	0.152820D-04	-0.200106D-04	-0.177237D-04	0.294598D-04
38	0.291064D-06	0.401107D-06	0.621446D-06	-0.194329D-05	0.136409D-05
39	0.680818D-05	0.323042D-06	0.628173D-06	0.147538D-06	-0.885698D-06
40	-0.144670D-04	0.152911D-04	0.133558D-04	0.411379D-04	-0.108638D-04
41	-0.189525D-07	-0.153824D-05	0.218836D-05	0.665713D-05	0.612557D-05
42	0.272836D-06	0.356290D-06	-0.320559D-06	-0.166473D-05	-0.229832D-05
43	0.267140D-04	0.481361D-04	-0.660658D-04	-0.500925D-04	0.868820D-04

ESTIMATED COVARIANCE MATRIX FOR PARAMETER ESTIMATES

	21	22	23	24	25
21	0.462721D-04				
22	0.113090D-04	0.561989D-04			
23	-0.922887D-05	-0.186395D-04	0.223423D-03		
24	0.434430D-05	0.125162D-04	-0.135936D-04	0.475412D-04	
25	0.219272D-04	0.457613D-04	-0.237641D-04	0.383775D-04	0.414769D-03
26	-0.879664D-05	-0.353328D-05	0.507368D-05	-0.303919D-06	-0.522742D-05
27	-0.260117D-05	-0.141351D-04	0.985004D-05	-0.470107D-05	-0.128892D-04
28	0.574531D-05	0.756904D-05	-0.438867D-04	0.266407D-05	0.448783D-05
29	-0.312276D-05	-0.650231D-05	0.233486D-05	-0.901844D-05	-0.104145D-04
30	-0.102669D-04	-0.245240D-04	0.386372D-04	-0.203697D-04	-0.780438D-04
31	0.348212D-05	0.929518D-05	-0.851305D-05	0.505626D-05	0.199254D-04
32	0.332745D-06	0.208819D-05	0.274903D-05	0.692970D-05	0.300905D-06
33	0.650154D-05	-0.141644D-05	0.416628D-04	-0.188863D-04	-0.996481D-04
34	0.374739D-05	0.232004D-05	0.303905D-04	-0.518723D-05	0.555864D-05
35	-0.178426D-05	0.627052D-05	-0.145001D-04	-0.189811D-05	-0.619059D-05
36	-0.864735D-05	0.229087D-05	0.653019D-05	0.984537D-06	0.167957D-04
37	0.319308D-04	-0.281573D-04	-0.638480D-05	0.143689D-04	0.652961D-04
38	-0.155838D-06	-0.309542D-07	-0.186154D-05	0.139492D-05	-0.605096D-05
39	0.154441D-05	0.542010D-06	-0.270367D-05	-0.188390D-05	-0.660629D-05
40	0.357430D-05	0.424677D-04	-0.639612D-04	-0.302568D-04	0.591687D-04
41	-0.819011D-05	0.167544D-05	0.973295D-05	-0.474628D-05	0.923350D-05
42	0.390326D-06	-0.431952D-06	0.453963D-05	0.179990D-05	0.206122D-05
43	0.964670D-04	-0.100765D-03	-0.902971D-05	0.376619D-04	0.198176D-03

ESTIMATED COVARIANCE MATRIX FOR PARAMETER ESTIMATES

	26	27	28	29	30
26	0.399474D-04				
27	0.944777D-05	0.548265D-04			
28	-0.833587D-05	-0.244987D-04	0.181341D-03		

29	0.211395D-05	0.134879D-04	-0.114316D-04	0.411009D-04	
30	0.812305D-05	0.247369D-04	-0.211726D-04	0.236818D-04	0.187420D-03
31	-0.116420D-04	-0.428173D-04	0.666781D-04	-0.326547D-04	-0.760528D-04
32	0.513107D-06	-0.755796D-05	0.605135D-05	-0.121012D-04	-0.771557D-05
33	-0.205133D-04	0.229404D-04	-0.710561D-04	0.255803D-04	0.377152D-04
34	0.697562D-05	-0.959436D-06	0.135916D-05	-0.167169D-05	-0.193090D-04
35	0.880792D-05	-0.927009D-06	-0.319801D-05	0.856095D-06	-0.368499D-05
36	-0.370091D-05	0.468367D-05	0.119925D-05	0.590791D-05	0.105819D-04
37	-0.596621D-04	-0.182338D-04	0.762148D-04	0.300861D-04	-0.599043D-05
38	0.101437D-05	-0.359061D-06	0.837682D-06	-0.698834D-06	0.197492D-05
39	0.666096D-06	0.194114D-06	0.137721D-05	0.682035D-06	0.397980D-05
40	-0.990117D-05	-0.663880D-04	-0.868987D-05	0.500965D-04	0.992866D-04
41	0.158477D-05	0.191515D-05	-0.286566D-04	-0.480540D-05	0.448000D-05
42	0.216366D-05	0.141478D-05	-0.291839D-05	0.299443D-05	0.304689D-05
43	-0.197570D-03	-0.473258D-04	0.230220D-03	0.100943D-03	-0.864037D-05

ESTIMATED COVARIANCE MATRIX FOR PARAMETER ESTIMATES

	31	32	33	34	35
31	0.314756D-03				
32	0.253250D-04	0.961379D-03			
33	-0.310843D-04	0.155797D-03	0.241049D-01		
34	0.136465D-04	0.771554D-05	0.958814D-03	0.142737D-02	
35	0.737807D-05	0.964284D-04	-0.105585D-04	0.223380D-04	0.763269D-03
36	-0.100735D-04	-0.324103D-05	0.635969D-04	-0.107219D-04	0.593381D-04
37	-0.113058D-04	-0.184402D-03	0.551118D-03	-0.623869D-04	0.679902D-04
38	0.938780D-06	0.311457D-04	0.798837D-05	0.840802D-05	0.166449D-04
39	0.460025D-06	-0.588789D-05	0.892497D-05	-0.504675D-07	0.409585D-05
40	0.765306D-05	0.162498D-03	-0.448196D-03	0.393769D-03	0.240129D-03
41	0.285295D-05	0.239502D-04	-0.165583D-03	0.492645D-05	0.544394D-04
42	-0.548273D-05	0.117237D-04	0.496384D-04	0.204345D-04	-0.468610D-05
43	-0.391384D-05	-0.669043D-03	0.194419D-02	-0.230645D-03	0.223822D-03

ESTIMATED COVARIANCE MATRIX FOR PARAMETER ESTIMATES

	36	37	38	39	40
36	0.954897D-03				
37	-0.704844D-03	0.300193D-01			
38	-0.125663D-05	0.457089D-05	0.278876D-04		
39	-0.518785D-04	-0.170970D-03	-0.135607D-05	0.146271D-03	
40	0.216408D-03	0.575192D-04	-0.792808D-05	-0.161776D-04	0.674037D-01
41	0.365199D-04	0.377481D-04	0.117697D-05	-0.864665D-05	0.148217D-02

42	0.106074D-04	0.581321D-04	0.108634D-05	-0.776201D-05	0.166564D-03
43	-0.244538D-02	0.979523D-01	0.256139D-04	-0.484921D-03	0.122245D-03

ESTIMATED COVARIANCE MATRIX FOR PARAMETER ESTIMATES

	41	42	43
41	0.199298D-02		
42	0.889183D-04	0.189806D-03	
43	0.651044D-04	0.171397D-03	0.321006D+00

ESTIMATED CORRELATION MATRIX FOR PARAMETER ESTIMATES

	1	2	3	4	5
1	1.000				
2	0.199	1.000			
3	0.052	0.285	1.000		
4	0.062	0.131	0.232	1.000	
5	-0.064	-0.203	-0.219	-0.220	1.000
6	0.029	0.001	0.046	0.010	-0.042
7	-0.057	0.023	-0.004	-0.025	0.006
8	-0.009	-0.036	-0.029	0.002	-0.008
9	0.053	0.014	-0.006	-0.010	0.017
10	0.034	-0.018	0.000	-0.041	0.033
11	0.021	0.004	0.007	-0.015	0.004
12	-0.046	-0.036	-0.007	-0.036	-0.007
13	0.020	0.024	-0.001	0.008	-0.023
14	0.007	-0.005	-0.039	0.010	0.023
15	0.019	-0.030	-0.027	-0.042	0.008
16	-0.052	-0.001	0.028	0.012	0.029
17	0.004	0.025	0.007	-0.038	0.024
18	0.000	0.030	0.009	0.017	-0.008
19	0.002	-0.006	0.037	0.015	-0.003
20	0.029	0.067	0.035	0.005	-0.035
21	-0.009	0.039	0.038	0.001	-0.003
22	-0.017	-0.005	0.024	0.012	-0.009
23	-0.024	-0.015	0.000	-0.002	-0.048
24	0.023	0.019	-0.002	0.001	0.030
25	-0.008	-0.003	0.001	0.012	0.011
26	0.051	-0.025	-0.065	0.010	0.036
27	0.044	0.008	0.022	-0.027	0.014
28	-0.047	0.029	0.014	0.035	-0.001
29	0.022	-0.008	0.026	-0.056	0.028

30	0.016	0.023	-0.003	-0.013	-0.033
31	-0.039	0.004	-0.014	0.041	-0.055
32	-0.013	0.017	-0.005	-0.016	0.016
33	-0.027	-0.038	0.034	-0.020	0.013
34	-0.070	-0.086	-0.060	-0.035	0.096
35	0.016	-0.025	0.045	0.008	0.014
36	-0.018	0.038	0.074	-0.024	0.018
37	-0.007	0.029	0.062	0.035	-0.043
38	-0.043	0.008	0.029	-0.042	0.032
39	0.007	-0.030	-0.011	-0.026	-0.023
40	-0.024	-0.031	0.018	0.041	-0.016
41	-0.012	-0.010	-0.019	0.018	-0.011
42	-0.033	-0.012	-0.037	-0.012	-0.013
43	-0.005	0.030	0.062	0.036	-0.045

ESTIMATED CORRELATION MATRIX FOR PARAMETER ESTIMATES

	6	7	8	9	10
6	1.000				
7	-0.102	1.000			
8	0.016	-0.179	1.000		
9	-0.006	-0.096	-0.213	1.000	
10	0.051	0.112	0.091	0.154	1.000
11	0.015	0.056	-0.034	0.010	0.009
12	-0.031	0.046	0.000	-0.009	-0.033
13	-0.029	0.034	-0.019	0.013	-0.026
14	0.006	-0.022	0.049	-0.027	-0.006
15	0.061	-0.028	0.033	-0.014	0.016
16	0.003	0.053	0.020	-0.012	0.014
17	-0.013	0.005	-0.026	-0.001	-0.023
18	-0.018	0.044	-0.017	0.012	-0.028
19	0.052	-0.032	-0.008	0.033	0.005
20	-0.029	0.038	0.001	0.017	0.011
21	-0.035	-0.003	-0.008	0.031	-0.020
22	-0.028	0.033	-0.007	0.011	0.005
23	0.039	-0.032	0.000	0.030	0.001
24	-0.017	0.038	-0.036	0.027	0.040
25	-0.029	0.024	-0.013	0.003	0.027
26	0.032	0.000	0.039	-0.020	0.036
27	0.030	-0.027	-0.022	0.013	0.012
28	-0.028	0.021	0.024	-0.020	-0.025
29	0.018	-0.069	0.047	-0.018	0.039
30	0.035	-0.023	0.032	-0.017	0.024

31	0.007	0.015	-0.017	0.008	-0.008
32	-0.048	0.150	-0.004	0.047	-0.068
33	0.003	0.030	-0.010	-0.006	-0.010
34	0.013	0.028	-0.023	-0.011	0.014
35	-0.028	-0.019	-0.051	0.036	-0.018
36	-0.040	0.060	-0.008	-0.078	0.016
37	0.044	-0.031	0.054	-0.007	-0.028
38	0.040	-0.031	0.000	0.009	-0.021
39	0.039	0.061	-0.009	0.038	0.006
40	0.002	0.003	0.016	-0.021	-0.007
41	-0.001	-0.024	0.004	0.034	-0.004
42	0.009	-0.033	0.017	0.020	-0.001
43	0.070	-0.027	0.053	-0.008	-0.027

ESTIMATED CORRELATION MATRIX FOR PARAMETER ESTIMATES

	11	12	13	14	15
11	1.000				
12	0.270	1.000			
13	0.020	0.297	1.000		
14	-0.105	-0.151	-0.088	1.000	
15	0.007	-0.108	-0.279	0.204	1.000
16	0.029	0.019	0.054	-0.092	-0.214
17	0.044	0.226	0.055	-0.118	-0.017
18	-0.010	0.106	0.329	-0.076	-0.173
19	-0.031	-0.050	-0.062	0.074	0.209
20	0.039	0.022	0.065	0.006	-0.016
21	0.102	0.212	0.079	-0.087	-0.037
22	0.085	0.171	0.282	-0.073	-0.153
23	-0.022	-0.057	-0.080	0.082	0.179
24	0.018	0.068	0.090	-0.031	-0.056
25	0.041	0.040	0.072	-0.057	-0.048
26	-0.146	-0.262	-0.073	0.198	0.031
27	-0.054	-0.177	-0.347	0.097	0.228
28	0.023	0.047	0.072	-0.084	-0.240
29	-0.059	-0.092	-0.094	0.060	0.078
30	-0.037	-0.043	-0.089	0.020	0.047
31	0.040	0.049	0.084	-0.016	-0.076
32	0.027	0.035	0.056	-0.032	-0.010
33	0.026	0.036	0.001	0.019	0.046
34	-0.003	0.023	0.036	-0.011	-0.011
35	-0.048	-0.016	0.059	-0.019	-0.111
36	-0.009	0.045	0.008	-0.009	-0.042

37	0.011	-0.018	-0.027	-0.002	0.005
38	0.010	0.008	0.029	0.027	0.002
39	0.047	0.018	-0.003	-0.032	0.026
40	0.012	-0.006	0.010	-0.004	-0.022
41	-0.022	-0.012	-0.007	-0.017	0.018
42	-0.007	-0.011	-0.015	-0.042	-0.021
43	0.014	-0.019	-0.030	-0.003	0.010

ESTIMATED CORRELATION MATRIX FOR PARAMETER ESTIMATES

	16	17	18	19	20
16	1.000				
17	-0.010	1.000			
18	0.073	0.182	1.000		
19	-0.184	-0.035	-0.171	1.000	
20	0.009	0.038	0.265	-0.148	1.000
21	0.013	0.228	0.085	-0.056	0.045
22	0.086	0.077	0.274	-0.062	0.047
23	-0.132	-0.097	-0.039	0.248	-0.009
24	0.022	0.075	0.225	-0.115	0.257
25	0.020	0.033	0.067	-0.027	0.051
26	-0.034	-0.211	-0.048	0.038	0.003
27	-0.077	-0.041	-0.220	0.089	-0.068
28	0.221	0.041	0.079	-0.205	-0.020
29	-0.026	-0.092	-0.242	0.221	-0.242
30	-0.007	-0.012	-0.104	0.102	-0.069
31	0.034	0.005	0.052	-0.073	0.025
32	-0.006	0.014	0.044	-0.009	0.024
33	0.020	-0.002	-0.040	0.065	-0.013
34	0.009	0.025	0.028	0.019	-0.018
35	0.038	-0.006	0.030	-0.010	-0.023
36	-0.014	-0.010	-0.011	-0.008	0.011
37	0.004	0.027	-0.030	-0.014	0.036
38	0.003	0.023	0.030	-0.052	0.055
39	0.028	0.008	0.013	0.002	-0.016
40	-0.003	0.018	0.013	0.022	-0.009
41	0.000	-0.011	0.013	0.021	0.029
42	0.001	0.008	-0.006	-0.017	-0.035
43	0.002	0.026	-0.030	-0.012	0.032

ESTIMATED CORRELATION MATRIX FOR PARAMETER ESTIMATES

21	22	23	24	25

21	1.000				
22	0.222	1.000			
23	-0.091	-0.166	1.000		
24	0.093	0.242	-0.132	1.000	
25	0.158	0.300	-0.078	0.273	1.000
26	-0.205	-0.075	0.054	-0.007	-0.041
27	-0.052	-0.255	0.089	-0.092	-0.085
28	0.063	0.075	-0.218	0.029	0.016
29	-0.072	-0.135	0.024	-0.204	-0.080
30	-0.110	-0.239	0.189	-0.216	-0.280
31	0.029	0.070	-0.032	0.041	0.055
32	0.002	0.009	0.006	0.032	0.000
33	0.006	-0.001	0.018	-0.018	-0.032
34	0.015	0.008	0.054	-0.020	0.007
35	-0.009	0.030	-0.035	-0.010	-0.011
36	-0.041	0.010	0.014	0.005	0.027
37	0.027	-0.022	-0.002	0.012	0.019
38	-0.004	-0.001	-0.024	0.038	-0.056
39	0.019	0.006	-0.015	-0.023	-0.027
40	0.002	0.022	-0.016	-0.017	0.011
41	-0.027	0.005	0.015	-0.015	0.010
42	0.004	-0.004	0.022	0.019	0.007
43	0.025	-0.024	-0.001	0.010	0.017

ESTIMATED CORRELATION MATRIX FOR PARAMETER ESTIMATES

	26	27	28	29	30
26	1.000				
27	0.202	1.000			
28	-0.098	-0.246	1.000		
29	0.052	0.284	-0.132	1.000	
30	0.094	0.244	-0.115	0.270	1.000
31	-0.104	-0.326	0.279	-0.287	-0.313
32	0.003	-0.033	0.014	-0.061	-0.018
33	-0.021	0.020	-0.034	0.026	0.018
34	0.029	-0.003	0.003	-0.007	-0.037
35	0.050	-0.005	-0.009	0.005	-0.010
36	-0.019	0.020	0.003	0.030	0.025
37	-0.054	-0.014	0.033	0.027	-0.003
38	0.030	-0.009	0.012	-0.021	0.027
39	0.009	0.002	0.008	0.009	0.024
40	-0.006	-0.035	-0.002	0.030	0.028

41	0.006	0.006	-0.048	-0.017	0.007
42	0.025	0.014	-0.016	0.034	0.016
43	-0.055	-0.011	0.030	0.028	-0.001

ESTIMATED CORRELATION MATRIX FOR PARAMETER ESTIMATES

	31	32	33	34	35
31	1.000				
32	0.046	1.000			
33	-0.011	0.032	1.000		
34	0.020	0.007	0.163	1.000	
35	0.015	0.113	-0.002	0.021	1.000
36	-0.018	-0.003	0.013	-0.009	0.070
37	-0.004	-0.034	0.020	-0.010	0.014
38	0.010	0.190	0.010	0.042	0.114
39	0.002	-0.016	0.005	0.000	0.012
40	0.002	0.020	-0.011	0.040	0.033
41	0.004	0.017	-0.024	0.003	0.044
42	-0.022	0.027	0.023	0.039	-0.012
43	0.000	-0.038	0.022	-0.011	0.014

ESTIMATED CORRELATION MATRIX FOR PARAMETER ESTIMATES

	36	37	38	39	40
36	1.000				
37	-0.132	1.000			
38	-0.008	0.005	1.000		
39	-0.139	-0.082	-0.021	1.000	
40	0.027	0.001	-0.006	-0.005	1.000
41	0.026	0.005	0.005	-0.016	0.128
42	0.025	0.024	0.015	-0.047	0.047
43	-0.140	0.998	0.009	-0.071	0.001

ESTIMATED CORRELATION MATRIX FOR PARAMETER ESTIMATES

	41	42	43
41	1.000		
42	0.145	1.000	
43	0.003	0.022	1.000

TECHNICAL 8 OUTPUT

TECHNICAL 8 OUTPUT FOR BAYES ESTIMATION

CHAIN	BSEED
1	0
2	285380
3	253358

ITERATION	POTENTIAL SCALE REDUCTION	PARAMETER WITH HIGHEST PSR
100	1.007	43
200	1.007	7
300	1.017	35
400	1.008	35
500	1.003	41
600	1.007	41
700	1.003	41
800	1.001	31
900	1.002	39
1000	1.002	23

Deterioration in physical health, bullying interaction

SUMMARY OF DATA

Number of clusters 58

Size (s)	Cluster ID with Size s
7	22
16	166
23	80 71 205
25	74
27	192
31	65
32	106
35	228
37	170 72
39	50 75 181
40	182
42	165
44	114
45	173
46	107
47	169 30
48	226 81
49	36
50	116 108 40
56	162 84
57	61
59	206 93 83
60	92
61	69
63	6
64	152
65	145
66	4
68	68
69	154
72	174
78	98
84	178 2
95	156 202
96	144
108	142

131	122
172	24
175	209
224	204
269	125
364	217
738	110
860	100

COVARIANCE COVERAGE OF DATA

Minimum covariance coverage value 0.100

Number of missing data patterns 8

PROPORTION OF DATA PRESENT

Covariance Coverage

	PHYCAT16	PRAFAC	PHY15	KES15	CHOICE15
PHYCAT16	1.000				
PRAFAC	1.000	1.000			
PHY15	0.947	0.947	0.947		
KES15	0.922	0.922	0.922	0.922	
CHOICE15	0.915	0.915	0.915	0.915	0.915
BULLY15	0.915	0.915	0.915	0.915	0.915
PRESS15	0.915	0.915	0.915	0.915	0.915
DUTIES15	0.915	0.915	0.915	0.915	0.915

Covariance Coverage

	BULLY15	PRESS15	DUTIES15
BULLY15	0.915		
PRESS15	0.914	0.915	
DUTIES15	0.915	0.915	0.915

UNIVARIATE PROPORTIONS AND COUNTS FOR CATEGORICAL VARIABLES

```
    PHYCAT16
      Category 1    0.881         4884.000
      Category 2    0.119          662.000


UNIVARIATE SAMPLE STATISTICS


     UNIVARIATE HIGHER-ORDER MOMENT DESCRIPTIVE STATISTICS

         Variable/         Mean/     Skewness/   Minimum/ % with                Percentiles
        Sample Size      Variance    Kurtosis    Maximum  Min/Max      20%/60%    40%/80%    Median

     PRAFAC              -0.185       0.245      -2.338    1.72%      -1.448     -0.527     -0.318
              58.000      1.345      -0.636       2.338    1.72%       0.080      0.752
     PHY15                0.000      -0.546      -0.632   36.83%      -0.632      0.368      0.368
            5254.000      0.233      -1.702       0.368   63.17%       0.368      0.368
     KES15                0.000       1.717      -0.511   21.65%      -0.511     -0.178     -0.178
            5114.000      0.289       3.835       3.489    0.04%      -0.011      0.322
     CHOICE15             0.000      -0.402      -2.225    7.41%      -1.225     -0.225     -0.225
            5077.000      1.081      -0.336       1.775    8.49%       0.775      0.775
     BULLY15              1.170       3.272       1.000   87.25%       1.000      1.000      1.000
            5073.000      0.239      11.772       5.000    0.08%       1.000      1.000
     PRESS15              0.000       0.252      -1.462   19.10%      -0.462     -0.462     -0.462
            5074.000      1.001      -0.371       2.538    2.76%       0.538      0.538
     DUTIES15             0.000      -1.246      -3.120    2.40%      -0.120     -0.120     -0.120
            5077.000      0.861       1.673       0.880   38.74%      -0.120      0.880


THE MODEL ESTIMATION TERMINATED NORMALLY

     USE THE FBITERATIONS OPTION TO INCREASE THE NUMBER OF ITERATIONS BY A FACTOR
     OF AT LEAST TWO TO CHECK CONVERGENCE AND THAT THE PSR VALUE DOES NOT INCREASE.



MODEL FIT INFORMATION

Number of Free Parameters                              43



MODEL RESULTS
```

	Estimate	Posterior S.D.	One-Tailed P-Value	95% C.I. Lower 2.5%	Upper 2.5%	Significance
**Within Level**						
PHYCAT16 ON						
PHY15	0.979	0.070	0.000	0.841	1.119	*
CHOICE15	-0.069	0.025	0.001	-0.117	-0.024	*
DUTIES15	-0.031	0.027	0.125	-0.085	0.021	
PRESS15	-0.032	0.027	0.111	-0.086	0.019	
KES15	0.226	0.045	0.000	0.144	0.311	*
KES15 WITH						
PHY15	0.051	0.004	0.000	0.044	0.059	*
CHOICE15	-0.089	0.008	0.000	-0.105	-0.073	*
BULLY15	0.057	0.004	0.000	0.049	0.065	*
PRESS15	0.100	0.007	0.000	0.085	0.115	*
DUTIES15	-0.106	0.007	0.000	-0.120	-0.091	*
PHY15 WITH						
CHOICE15	-0.036	0.007	0.000	-0.050	-0.023	*
BULLY15	0.011	0.003	0.000	0.005	0.018	*
PRESS15	0.038	0.007	0.000	0.025	0.052	*
DUTIES15	-0.036	0.006	0.000	-0.049	-0.024	*
CHOICE15 WITH						
BULLY15	-0.057	0.007	0.000	-0.072	-0.043	*
PRESS15	-0.087	0.015	0.000	-0.118	-0.059	*
DUTIES15	0.174	0.014	0.000	0.146	0.201	*
BULLY15 WITH						
PRESS15	0.105	0.007	0.000	0.091	0.119	*
DUTIES15	-0.075	0.006	0.000	-0.088	-0.063	*
PRESS15 WITH						
DUTIES15	-0.185	0.014	0.000	-0.213	-0.159	*
Means						
PHY15	0.002	0.007	0.377	-0.011	0.016	
KES15	0.001	0.008	0.447	-0.014	0.017	
CHOICE15	-0.001	0.015	0.467	-0.030	0.029	
PRESS15	0.001	0.014	0.485	-0.025	0.029	
DUTIES15	0.000	0.013	0.484	-0.027	0.024	

Variances						
PHY15	0.233	0.005	0.000	0.225	0.242	*
KES15	0.290	0.006	0.000	0.278	0.302	*
CHOICE15	1.083	0.021	0.000	1.043	1.123	*
BULLY15	0.238	0.005	0.000	0.229	0.247	*
PRESS15	1.001	0.020	0.000	0.963	1.044	*
DUTIES15	0.861	0.018	0.000	0.829	0.897	*

Between Level

S1 ON						
PRAFAC	-0.076	0.079	0.152	-0.236	0.074	
PHYCAT16 ON						
PRAFAC	-0.088	0.031	0.003	-0.152	-0.027	*
BULLY15	0.206	0.611	0.368	-0.955	1.459	
PRAFAC WITH						
BULLY15	0.015	0.027	0.263	-0.038	0.066	
Means						
PRAFAC	-0.182	0.155	0.122	-0.488	0.116	
BULLY15	1.166	0.022	0.000	1.121	1.211	*
Intercepts						
S1	-0.016	0.086	0.425	-0.195	0.135	
Thresholds						
PHYCAT16$1	1.596	0.714	0.011	0.231	3.069	*
Variances						
PRAFAC	1.346	0.261	0.000	0.958	1.987	*
BULLY15	0.024	0.005	0.000	0.017	0.037	*
Residual Variances						
PHYCAT16	0.012	0.011	0.000	0.000	0.044	*
S1	0.061	0.062	0.000	0.008	0.250	*

STANDARDIZED MODEL RESULTS

STDYX Standardization

	Estimate	Posterior S.D.	One-Tailed P-Value	95% C.I. Lower 2.5%	Upper 2.5%	Significance
**Within-Level Standardized Estimates Averaged Over Clusters**						
S1 | PHYCAT16 ON						
BULLY15	-0.002	0.031	0.475	-0.066	0.054	
PHYCAT16 ON						
PHY15	0.415	0.025	0.000	0.363	0.463	*
CHOICE15	-0.063	0.023	0.002	-0.107	-0.021	*
DUTIES15	-0.026	0.022	0.129	-0.069	0.017	
PRESS15	-0.028	0.023	0.117	-0.075	0.017	
KES15	0.107	0.021	0.000	0.067	0.148	*
KES15 WITH						
PHY15	0.198	0.013	0.000	0.171	0.223	*
CHOICE15	-0.159	0.014	0.000	-0.185	-0.131	*
BULLY15	0.216	0.014	0.000	0.189	0.243	*
PRESS15	0.185	0.013	0.000	0.160	0.211	*
DUTIES15	-0.212	0.014	0.000	-0.240	-0.184	*
PHY15 WITH						
CHOICE15	-0.072	0.014	0.000	-0.099	-0.045	*
BULLY15	0.049	0.014	0.000	0.021	0.075	*
PRESS15	0.080	0.014	0.000	0.053	0.107	*
DUTIES15	-0.081	0.014	0.000	-0.108	-0.053	*
CHOICE15 WITH						
BULLY15	-0.113	0.014	0.000	-0.141	-0.086	*
PRESS15	-0.083	0.014	0.000	-0.112	-0.056	*
DUTIES15	0.179	0.014	0.000	0.152	0.206	*
BULLY15 WITH						
PRESS15	0.215	0.014	0.000	0.188	0.240	*
DUTIES15	-0.167	0.014	0.000	-0.194	-0.140	*
PRESS15 WITH						
DUTIES15	-0.199	0.013	0.000	-0.227	-0.173	*
Means						
PHY15	0.004	0.014	0.387	-0.023	0.032	
KES15	0.002	0.014	0.443	-0.025	0.030	

```
    CHOICE15          -0.001       0.014       0.458      -0.029       0.027
    PRESS15            0.001       0.014       0.485      -0.026       0.029
    DUTIES15          -0.001       0.014       0.477      -0.028       0.026

 Variances
    PHY15              1.000       0.000       0.000       1.000       1.000
    KES15              1.000       0.000       0.000       1.000       1.000
    CHOICE15           1.000       0.000       0.000       1.000       1.000
    BULLY15            1.000       0.000       0.000       1.000       1.000
    PRESS15            1.000       0.000       0.000       1.000       1.000
    DUTIES15           1.000       0.000       0.000       1.000       1.000

Between Level

 S1         ON
    PRAFAC            -0.337       0.299       0.152      -0.789       0.357

 PHYCAT16   ON
    PRAFAC            -0.586       0.184       0.003      -0.909      -0.202      *
    BULLY15            0.207       0.470       0.368      -0.741       0.900

 PRAFAC   WITH
    BULLY15            0.085       0.138       0.263      -0.208       0.343

 Means
    PRAFAC            -0.159       0.133       0.122      -0.416       0.107
    BULLY15            7.460       0.738       0.000       6.084       8.990      *

 Intercepts
    S1                -0.060       0.333       0.425      -0.612       0.659

 Variances
    PRAFAC             1.000       0.000       0.000       1.000       1.000
    BULLY15            1.000       0.000       0.000       1.000       1.000

 Residual Variances
    PHYCAT16           0.405       0.231       0.000       0.026       0.858      *
    S1                 0.874       0.180       0.000       0.369       1.000      *


R-SQUARE

Within-Level R-Square Averaged Across Clusters
```

```
                                Posterior  One-Tailed         95% C.I.
    Variable        Estimate       S.D.      P-Value   Lower 2.5%  Upper 2.5%

    PHYCAT16           0.230       0.023      0.000       0.186       0.278

Between Level

                                Posterior  One-Tailed         95% C.I.
    Variable        Estimate       S.D.      P-Value   Lower 2.5%  Upper 2.5%

    PHYCAT16           0.595       0.231      0.000       0.142       0.974

                                Posterior  One-Tailed         95% C.I.
    Variable        Estimate       S.D.      P-Value   Lower 2.5%  Upper 2.5%

    S1                 0.126       0.180      0.000       0.000       0.631


TECHNICAL 1 OUTPUT


     PARAMETER SPECIFICATION FOR WITHIN


           TAU
              PHYCAT16
              ________
                    0


           NU
              PHYCAT16      PHY15         KES15         CHOICE15      BULLY15
              ________      ________      ________      ________      ________
                    0             0             0             0             0


           NU
              PRESS15       DUTIES15
              ________      ________
                    0             0


           LAMBDA
              PHYCAT16      PHY15         KES15         CHOICE15      BULLY15
```

PHYCAT16	0	0	0	0	0
PHY15	0	0	0	0	0
KES15	0	0	0	0	0
CHOICE15	0	0	0	0	0
BULLY15	0	0	0	0	0
PRESS15	0	0	0	0	0
DUTIES15	0	0	0	0	0

LAMBDA

	PRESS15	DUTIES15
PHYCAT16	0	0
PHY15	0	0
KES15	0	0
CHOICE15	0	0
BULLY15	0	0
PRESS15	0	0
DUTIES15	0	0

THETA

	PHYCAT16	PHY15	KES15	CHOICE15	BULLY15
PHYCAT16	0				
PHY15	0	0			
KES15	0	0	0		
CHOICE15	0	0	0	0	
BULLY15	0	0	0	0	0
PRESS15	0	0	0	0	0
DUTIES15	0	0	0	0	0

THETA

	PRESS15	DUTIES15
PRESS15	0	
DUTIES15	0	0

ALPHA

	PHYCAT16	PHY15	KES15	CHOICE15	BULLY15

0	1	2	3	0

ALPHA

PRESS15	DUTIES15
4	5

BETA

	PHYCAT16	PHY15	KES15	CHOICE15	BULLY15
PHYCAT16	0	6	7	8	0
PHY15	0	0	0	0	0
KES15	0	0	0	0	0
CHOICE15	0	0	0	0	0
BULLY15	0	0	0	0	0
PRESS15	0	0	0	0	0
DUTIES15	0	0	0	0	0

BETA

	PRESS15	DUTIES15
PHYCAT16	9	10
PHY15	0	0
KES15	0	0
CHOICE15	0	0
BULLY15	0	0
PRESS15	0	0
DUTIES15	0	0

PSI

	PHYCAT16	PHY15	KES15	CHOICE15	BULLY15
PHYCAT16	0				
PHY15	0	11			
KES15	0	12	13		
CHOICE15	0	14	15	16	
BULLY15	0	17	18	19	20
PRESS15	0	21	22	23	24
DUTIES15	0	26	27	28	29

PSI

	PRESS15	DUTIES15
PRESS15	25	
DUTIES15	30	31

PARAMETER SPECIFICATION FOR BETWEEN

TAU

PHYCAT16
43

NU

PHYCAT16	PRAFAC	BULLY15
0	0	0

LAMBDA

	S1	PHYCAT16	PRAFAC	BULLY15
PHYCAT16	0	0	0	0
PRAFAC	0	0	0	0
BULLY15	0	0	0	0

THETA

	PHYCAT16	PRAFAC	BULLY15
PHYCAT16	0		
PRAFAC	0	0	
BULLY15	0	0	0

ALPHA

S1	PHYCAT16	PRAFAC	BULLY15
32	0	33	34

BETA

	S1	PHYCAT16	PRAFAC	BULLY15
S1	0	0	35	0
PHYCAT16	0	0	36	37
PRAFAC	0	0	0	0
BULLY15	0	0	0	0

PSI

	S1	PHYCAT16	PRAFAC	BULLY15
S1	38			
PHYCAT16	0	39		
PRAFAC	0	0	40	
BULLY15	0	0	41	42

STARTING VALUES FOR WITHIN

TAU

PHYCAT16
0.000

NU

PHYCAT16	PHY15	KES15	CHOICE15	BULLY15
0.000	0.000	0.000	0.000	0.000

NU

PRESS15	DUTIES15
0.000	0.000

LAMBDA

	PHYCAT16	PHY15	KES15	CHOICE15	BULLY15
PHYCAT16	1.000	0.000	0.000	0.000	0.000

PHY15	0.000	1.000	0.000	0.000	0.000
KES15	0.000	0.000	1.000	0.000	0.000
CHOICE15	0.000	0.000	0.000	1.000	0.000
BULLY15	0.000	0.000	0.000	0.000	1.000
PRESS15	0.000	0.000	0.000	0.000	0.000
DUTIES15	0.000	0.000	0.000	0.000	0.000

LAMBDA

	PRESS15	DUTIES15
PHYCAT16	0.000	0.000
PHY15	0.000	0.000
KES15	0.000	0.000
CHOICE15	0.000	0.000
BULLY15	0.000	0.000
PRESS15	1.000	0.000
DUTIES15	0.000	1.000

THETA

	PHYCAT16	PHY15	KES15	CHOICE15	BULLY15
PHYCAT16	0.000				
PHY15	0.000	0.000			
KES15	0.000	0.000	0.000		
CHOICE15	0.000	0.000	0.000	0.000	
BULLY15	0.000	0.000	0.000	0.000	0.000
PRESS15	0.000	0.000	0.000	0.000	0.000
DUTIES15	0.000	0.000	0.000	0.000	0.000

THETA

	PRESS15	DUTIES15
PRESS15	0.000	
DUTIES15	0.000	0.000

ALPHA

PHYCAT16	PHY15	KES15	CHOICE15	BULLY15
0.000	0.000	0.000	0.000	0.000

ALPHA

PRESS15	DUTIES15
0.000	0.000

BETA

	PHYCAT16	PHY15	KES15	CHOICE15	BULLY15
PHYCAT16	0.000	0.000	0.000	0.000	0.000
PHY15	0.000	0.000	0.000	0.000	0.000
KES15	0.000	0.000	0.000	0.000	0.000
CHOICE15	0.000	0.000	0.000	0.000	0.000
BULLY15	0.000	0.000	0.000	0.000	0.000
PRESS15	0.000	0.000	0.000	0.000	0.000
DUTIES15	0.000	0.000	0.000	0.000	0.000

BETA

	PRESS15	DUTIES15
PHYCAT16	0.000	0.000
PHY15	0.000	0.000
KES15	0.000	0.000
CHOICE15	0.000	0.000
BULLY15	0.000	0.000
PRESS15	0.000	0.000
DUTIES15	0.000	0.000

PSI

	PHYCAT16	PHY15	KES15	CHOICE15	BULLY15
PHYCAT16	1.000				
PHY15	0.000	0.116			
KES15	0.000	0.000	0.145		
CHOICE15	0.000	0.000	0.000	0.541	
BULLY15	0.000	0.000	0.000	0.000	0.119
PRESS15	0.000	0.000	0.000	0.000	0.000
DUTIES15	0.000	0.000	0.000	0.000	0.000

PSI

	PRESS15	DUTIES15
PRESS15	0.501	
DUTIES15	0.000	0.430

STARTING VALUES FOR BETWEEN

TAU

PHYCAT16
1.110

NU

PHYCAT16	PRAFAC	BULLY15
0.000	0.000	0.000

LAMBDA

	S1	PHYCAT16	PRAFAC	BULLY15
PHYCAT16	0.000	1.000	0.000	0.000
PRAFAC	0.000	0.000	1.000	0.000
BULLY15	0.000	0.000	0.000	1.000

THETA

	PHYCAT16	PRAFAC	BULLY15
PHYCAT16	0.000		
PRAFAC	0.000	0.000	
BULLY15	0.000	0.000	0.000

ALPHA

S1	PHYCAT16	PRAFAC	BULLY15
0.000	0.000	0.020	1.170

BETA

	S1	PHYCAT16	PRAFAC	BULLY15
S1	0.000	0.000	0.000	0.000
PHYCAT16	0.000	0.000	0.000	0.000
PRAFAC	0.000	0.000	0.000	0.000
BULLY15	0.000	0.000	0.000	0.000

PSI

	S1	PHYCAT16	PRAFAC	BULLY15
S1	1.000			
PHYCAT16	0.000	1.000		
PRAFAC	0.000	0.000	0.504	
BULLY15	0.000	0.000	0.000	0.119

PRIORS FOR ALL PARAMETERS	PRIOR MEAN	PRIOR VARIANCE	PRIOR STD. DEV.
Parameter 1~N(0.000,infinity)	0.0000	infinity	infinity
Parameter 2~N(0.000,infinity)	0.0000	infinity	infinity
Parameter 3~N(0.000,infinity)	0.0000	infinity	infinity
Parameter 4~N(0.000,infinity)	0.0000	infinity	infinity
Parameter 5~N(0.000,infinity)	0.0000	infinity	infinity
Parameter 6~N(0.000,5.000)	0.0000	5.0000	2.2361
Parameter 7~N(0.000,5.000)	0.0000	5.0000	2.2361
Parameter 8~N(0.000,5.000)	0.0000	5.0000	2.2361
Parameter 9~N(0.000,5.000)	0.0000	5.0000	2.2361
Parameter 10~N(0.000,5.000)	0.0000	5.0000	2.2361
Parameter 11~IW(1.000,7)	infinity	infinity	infinity
Parameter 12~IW(0.000,7)	infinity	infinity	infinity
Parameter 13~IW(1.000,7)	infinity	infinity	infinity
Parameter 14~IW(0.000,7)	infinity	infinity	infinity
Parameter 15~IW(0.000,7)	infinity	infinity	infinity
Parameter 16~IW(1.000,7)	infinity	infinity	infinity
Parameter 17~IW(0.000,7)	infinity	infinity	infinity
Parameter 18~IW(0.000,7)	infinity	infinity	infinity
Parameter 19~IW(0.000,7)	infinity	infinity	infinity
Parameter 20~IW(1.000,7)	infinity	infinity	infinity
Parameter 21~IW(0.000,7)	infinity	infinity	infinity
Parameter 22~IW(0.000,7)	infinity	infinity	infinity
Parameter 23~IW(0.000,7)	infinity	infinity	infinity
Parameter 24~IW(0.000,7)	infinity	infinity	infinity

Parameter 25~IW(1.000,7)	infinity	infinity	infinity
Parameter 26~IW(0.000,7)	infinity	infinity	infinity
Parameter 27~IW(0.000,7)	infinity	infinity	infinity
Parameter 28~IW(0.000,7)	infinity	infinity	infinity
Parameter 29~IW(0.000,7)	infinity	infinity	infinity
Parameter 30~IW(0.000,7)	infinity	infinity	infinity
Parameter 31~IW(1.000,7)	infinity	infinity	infinity
Parameter 32~N(0.000,infinity)	0.0000	infinity	infinity
Parameter 33~N(0.000,infinity)	0.0000	infinity	infinity
Parameter 34~N(0.000,infinity)	0.0000	infinity	infinity
Parameter 35~N(0.000,infinity)	0.0000	infinity	infinity
Parameter 36~N(0.000,5.000)	0.0000	5.0000	2.2361
Parameter 37~N(0.000,5.000)	0.0000	5.0000	2.2361
Parameter 38~IG(-1.000,0.000)	infinity	infinity	infinity
Parameter 39~IG(-1.000,0.000)	infinity	infinity	infinity
Parameter 40~IW(1.000,3)	infinity	infinity	infinity
Parameter 41~IW(0.000,3)	infinity	infinity	infinity
Parameter 42~IW(1.000,3)	infinity	infinity	infinity
Parameter 43~N(0.000,5.000)	0.0000	5.0000	2.2361

TECHNICAL 3 OUTPUT

ESTIMATED COVARIANCE MATRIX FOR PARAMETER ESTIMATES

	1	2	3	4	5
1	0.453643D-04				
2	0.990726D-05	0.588463D-04			
3	-0.734531D-05	-0.102417D-04	0.221473D-03		
4	0.544985D-05	0.113882D-04	-0.980580D-05	0.193914D-03	
5	-0.534856D-05	-0.169879D-04	0.270543D-04	-0.367164D-04	0.162568D-03
6	0.115226D-04	-0.224876D-05	0.580019D-04	-0.506562D-05	-0.256628D-04
7	-0.146854D-04	0.297308D-05	0.179960D-06	-0.146421D-04	0.579406D-05
8	-0.670111D-06	-0.681694D-05	0.891241D-05	0.336575D-05	-0.885874D-05
9	0.121944D-04	0.255414D-05	-0.717766D-05	0.147887D-05	0.183298D-06
10	0.456088D-05	-0.612833D-06	0.361368D-06	-0.172334D-04	0.137542D-04
11	0.609011D-06	0.799532D-09	0.543324D-06	-0.120392D-05	0.534082D-06
12	-0.110670D-05	-0.975492D-06	-0.480142D-08	-0.198658D-05	-0.472200D-06
13	0.823585D-06	0.118217D-05	-0.179802D-05	0.691808D-06	-0.216185D-05
14	0.254778D-06	-0.364412D-06	-0.443571D-05	0.171715D-05	0.131221D-05
15	0.104994D-05	-0.184702D-05	-0.896764D-06	-0.500509D-05	0.995484D-06
16	-0.698364D-05	0.242126D-05	0.775775D-05	0.479762D-05	0.718040D-05
17	0.991660D-07	0.412464D-06	-0.376441D-06	-0.204210D-05	0.124422D-05

18	0.613600D-07	0.867181D-06	0.268006D-06	0.122875D-05	-0.603800D-06
19	0.375239D-07	-0.545750D-06	0.438738D-05	-0.174101D-05	0.110162D-05
20	0.902365D-06	0.189051D-05	0.150759D-05	0.912327D-07	-0.109981D-05
21	-0.554424D-06	0.122355D-05	0.248995D-05	-0.198647D-05	0.169662D-05
22	-0.931033D-06	-0.714029D-06	0.301867D-05	0.156711D-06	0.280285D-06
23	-0.259740D-05	-0.300568D-05	0.594502D-05	-0.350519D-05	-0.863708D-05
24	0.105355D-05	0.122479D-05	-0.171773D-05	0.133896D-05	0.288888D-05
25	-0.108287D-05	-0.399325D-06	-0.314188D-05	0.385273D-05	0.297942D-05
26	0.234631D-05	-0.356800D-06	-0.503875D-05	0.335012D-05	0.513735D-06
27	0.198349D-05	-0.277618D-06	0.166610D-05	-0.431484D-05	0.281832D-05
28	-0.370444D-05	0.500516D-05	0.844852D-06	0.897310D-05	-0.210709D-05
29	0.910645D-06	-0.294930D-06	0.145904D-05	-0.565372D-05	0.204260D-05
30	0.138499D-05	0.218426D-05	-0.358972D-05	-0.197663D-05	-0.597519D-05
31	-0.465938D-05	0.888974D-06	-0.156531D-05	0.109841D-04	-0.139267D-04
32	-0.134159D-04	0.426885D-05	-0.535163D-04	0.142197D-04	0.320655D-04
33	-0.276545D-04	-0.431056D-04	0.100306D-03	-0.571354D-04	0.428322D-04
34	-0.547036D-05	0.869966D-05	-0.146791D-04	0.210831D-04	-0.101536D-04
35	0.157581D-04	-0.166391D-05	0.896435D-05	-0.208054D-04	0.162244D-04
36	-0.389525D-05	0.306702D-05	0.185769D-04	-0.228901D-04	0.211545D-04
37	0.555189D-04	0.856440D-04	0.453267D-03	-0.120880D-03	-0.215714D-03
38	-0.174080D-04	0.181496D-04	0.200075D-04	-0.597452D-05	-0.275859D-04
39	-0.693970D-06	-0.448923D-05	0.376173D-06	-0.508726D-05	-0.107661D-05
40	-0.412720D-04	-0.533783D-04	0.116054D-03	0.131717D-03	-0.315644D-04
41	-0.390236D-05	-0.412372D-05	-0.937320D-06	0.211957D-05	-0.304712D-05
42	-0.119007D-05	-0.795156D-06	-0.254130D-05	-0.195130D-05	-0.282662D-05
43	0.684934D-04	0.100403D-03	0.515707D-03	-0.129117D-03	-0.266473D-03

ESTIMATED COVARIANCE MATRIX FOR PARAMETER ESTIMATES

	6	7	8	9	10
6	0.491289D-02				
7	-0.302209D-03	0.199785D-02			
8	-0.329159D-05	0.995129D-04	0.619628D-03		
9	0.108514D-04	-0.130816D-03	0.190306D-04	0.714151D-03	
10	0.822018D-04	0.144111D-03	-0.100552D-03	0.109606D-03	0.714531D-03
11	0.510557D-05	0.855036D-05	-0.362663D-05	-0.285820D-06	0.306893D-05
12	-0.911101D-05	0.473810D-05	-0.134921D-06	-0.104971D-05	-0.257753D-05
13	-0.133294D-04	0.364561D-05	-0.403694D-05	0.297869D-06	-0.139797D-05
14	0.111627D-05	-0.187732D-05	0.105734D-04	-0.118608D-05	-0.549531D-05
15	0.335077D-04	-0.640767D-05	-0.201359D-05	-0.259289D-05	0.281133D-05
16	-0.130614D-05	0.437539D-04	0.130215D-04	-0.735040D-05	0.154860D-05
17	-0.380729D-05	0.620547D-07	-0.726409D-06	-0.370473D-06	-0.128406D-05
18	-0.304019D-05	0.596172D-05	-0.895801D-06	0.131702D-05	-0.157600D-05

19	0.257211D-04	-0.886218D-05	-0.847743D-06	0.413142D-05	0.102108D-05
20	-0.943364D-05	0.723739D-05	-0.143796D-05	0.257966D-05	0.287569D-05
21	-0.175209D-04	0.360342D-06	-0.994926D-07	0.558565D-05	-0.354365D-05
22	-0.168888D-04	0.884798D-05	0.170131D-05	0.622724D-07	0.200141D-05
23	0.553480D-04	-0.197652D-04	-0.135250D-05	0.115821D-04	0.300001D-05
24	-0.107821D-04	0.100898D-04	-0.587651D-05	0.452550D-05	0.104164D-04
25	-0.422156D-04	0.245721D-04	-0.517738D-05	-0.105298D-05	0.157916D-04
26	0.124751D-04	0.218937D-05	0.610279D-05	-0.188414D-05	0.451186D-05
27	0.146613D-04	-0.663664D-05	-0.384803D-05	0.292561D-05	0.232987D-05
28	-0.263943D-04	0.731723D-05	0.172116D-04	-0.596798D-05	-0.103766D-04
29	0.891512D-05	-0.158621D-04	0.217540D-06	-0.116305D-05	0.401779D-05
30	0.308903D-04	-0.101989D-04	0.956272D-05	-0.737362D-06	0.396257D-05
31	0.583196D-05	-0.613883D-05	-0.122755D-04	-0.274568D-05	0.392226D-05
32	-0.470228D-03	-0.909853D-04	0.190146D-04	-0.236974D-03	0.242207D-03
33	0.327320D-05	0.217022D-03	0.319244D-04	-0.387070D-04	-0.399023D-04
34	-0.167673D-04	0.410273D-04	0.570446D-05	0.218123D-05	0.259179D-04
35	-0.478331D-03	-0.148082D-03	-0.116773D-03	-0.154860D-04	-0.141208D-04
36	-0.109528D-03	0.588917D-04	-0.975940D-05	-0.772382D-04	0.104574D-04
37	0.342079D-03	0.128454D-03	0.287977D-03	0.640234D-03	-0.129897D-03
38	0.453476D-03	-0.501351D-04	-0.816130D-04	-0.962882D-05	-0.462593D-04
39	0.365265D-04	0.340739D-04	0.160010D-05	0.851616D-05	-0.382697D-06
40	-0.364487D-04	0.527904D-04	0.135157D-03	-0.190160D-03	-0.225522D-04
41	0.993938D-05	-0.368811D-04	0.258524D-04	0.198623D-04	0.108800D-05
42	-0.268936D-05	-0.103969D-04	0.566362D-05	0.165977D-05	-0.491791D-05
43	0.139053D-02	0.241307D-03	0.265846D-03	0.741042D-03	-0.132920D-03

ESTIMATED COVARIANCE MATRIX FOR PARAMETER ESTIMATES

	11	12	13	14	15
11	0.209421D-04				
12	0.454806D-05	0.135448D-04			
13	0.558288D-06	0.638712D-05	0.342496D-04		
14	-0.381564D-05	-0.394412D-05	-0.379785D-05	0.514851D-04	
15	0.218662D-06	-0.341566D-05	-0.134103D-04	0.117654D-04	0.666313D-04
16	0.286423D-05	0.160639D-05	0.654585D-05	-0.143524D-04	-0.360834D-04
17	0.631827D-06	0.264489D-05	0.111521D-05	-0.283876D-05	-0.413312D-06
18	-0.225193D-06	0.147786D-05	0.738842D-05	-0.211203D-05	-0.538425D-05
19	-0.122383D-05	-0.154800D-05	-0.278332D-05	0.420902D-05	0.114981D-04
20	0.895175D-06	0.402061D-06	0.185765D-05	0.118167D-06	-0.503665D-06
21	0.319304D-05	0.529319D-05	0.312367D-05	-0.462912D-05	-0.231329D-05
22	0.290045D-05	0.470312D-05	0.123437D-04	-0.402398D-05	-0.987872D-05
23	-0.185161D-05	-0.356117D-05	-0.724959D-05	0.909254D-05	0.211790D-04
24	0.713191D-06	0.184174D-05	0.362780D-05	-0.215150D-05	-0.331798D-05

25	0.378026D-05	0.305347D-05	0.850653D-05	-0.797377D-05	-0.875846D-05
26	-0.422677D-05	-0.608270D-05	-0.271264D-05	0.895977D-05	0.155651D-05
27	-0.185816D-05	-0.481963D-05	-0.150198D-04	0.509963D-05	0.138443D-04
28	0.158208D-05	0.240475D-05	0.605094D-05	-0.858710D-05	-0.258535D-04
29	-0.179378D-05	-0.218598D-05	-0.368546D-05	0.307645D-05	0.398029D-05
30	-0.228403D-05	-0.216823D-05	-0.705929D-05	0.190709D-05	0.565709D-05
31	0.322606D-05	0.316968D-05	0.873581D-05	-0.220620D-05	-0.109545D-04
32	-0.992956D-07	0.101014D-04	0.145246D-04	-0.152653D-04	0.142779D-04
33	0.177678D-04	0.204001D-04	0.120866D-05	0.190302D-04	0.568623D-04
34	0.523634D-06	0.620766D-06	0.524259D-05	-0.456743D-05	-0.650634D-05
35	-0.166094D-04	-0.637871D-05	0.267921D-04	0.217503D-04	-0.597733D-04
36	0.117170D-05	0.519075D-05	0.961761D-06	0.143745D-05	-0.594564D-05
37	-0.483566D-05	-0.374793D-04	-0.253431D-04	-0.750771D-04	0.155070D-03
38	0.745658D-05	-0.343943D-05	-0.132741D-04	-0.553549D-05	-0.570147D-05
39	0.296624D-05	0.775311D-06	0.113961D-05	-0.370630D-05	0.546891D-06
40	0.199759D-04	-0.524903D-05	0.158250D-04	-0.252081D-05	-0.399729D-04
41	-0.380749D-05	-0.858238D-06	-0.233931D-06	-0.341875D-05	0.101994D-05
42	-0.497488D-06	-0.106058D-06	-0.248952D-06	-0.156451D-05	-0.136868D-05
43	0.229025D-05	-0.468471D-04	-0.345649D-04	-0.960935D-04	0.200743D-03

ESTIMATED COVARIANCE MATRIX FOR PARAMETER ESTIMATES

	16	17	18	19	20
16	0.420024D-03				
17	-0.665453D-06	0.105030D-04			
18	0.594122D-05	0.229841D-05	0.149960D-04		
19	-0.271473D-04	-0.113824D-05	-0.456726D-05	0.542397D-04	
20	0.101600D-05	0.538288D-06	0.483968D-05	-0.521288D-05	0.224047D-04
21	0.202487D-05	0.503428D-05	0.231598D-05	-0.293362D-05	0.151988D-05
22	0.129921D-04	0.182144D-05	0.788298D-05	-0.327048D-05	0.169128D-05
23	-0.426064D-04	-0.455585D-05	-0.159022D-05	0.249695D-04	0.103053D-06
24	0.397789D-05	0.179593D-05	0.595048D-05	-0.712113D-05	0.888293D-05
25	0.723308D-05	0.188760D-05	0.525687D-05	-0.414012D-05	0.530475D-05
26	-0.443715D-05	-0.417132D-05	-0.109921D-05	0.123230D-05	0.217757D-06
27	-0.108236D-04	-0.928663D-06	-0.612908D-05	0.443220D-05	-0.233135D-05
28	0.620945D-04	0.177352D-05	0.399078D-05	-0.193018D-04	-0.118892D-05
29	-0.365849D-05	-0.202137D-05	-0.622319D-05	0.110056D-04	-0.746635D-05
30	-0.276369D-05	-0.436845D-06	-0.542073D-05	0.101397D-04	-0.449761D-05
31	0.129742D-04	0.306119D-06	0.322620D-05	-0.942398D-05	0.191153D-05
32	-0.601478D-05	0.903435D-05	0.403796D-05	0.685117D-05	0.146398D-05
33	0.751363D-04	-0.203619D-05	-0.232253D-04	0.545887D-04	-0.596723D-05
34	0.822595D-05	0.207458D-05	0.373012D-05	-0.299373D-05	0.119196D-05
35	0.603539D-04	-0.306664D-06	0.115799D-04	0.480609D-05	-0.101046D-04

36	-0.961766D-05	-0.944615D-06	-0.132288D-05	0.459927D-05	-0.654217D-06
37	-0.152166D-03	0.177266D-04	-0.254111D-04	0.559530D-04	0.187482D-04
38	-0.561672D-05	0.275923D-05	0.170198D-05	-0.189384D-04	0.359504D-05
39	0.242657D-05	0.324740D-06	0.428778D-06	0.256488D-06	-0.124552D-05
40	-0.279666D-04	0.154477D-04	0.879564D-05	0.401960D-04	-0.660450D-05
41	0.596529D-05	0.863098D-06	0.343511D-05	-0.348344D-06	0.541630D-05
42	0.204964D-05	0.176285D-06	-0.115442D-06	0.715026D-06	-0.840108D-06
43	-0.195759D-03	0.183921D-04	-0.285215D-04	0.718190D-04	0.108022D-04

ESTIMATED COVARIANCE MATRIX FOR PARAMETER ESTIMATES

	21	22	23	24	25
21	0.462091D-04				
22	0.112894D-04	0.561622D-04			
23	-0.104777D-04	-0.189900D-04	0.215249D-03		
24	0.440642D-05	0.122190D-04	-0.127871D-04	0.500239D-04	
25	0.217722D-04	0.457263D-04	-0.303992D-04	0.394468D-04	0.414768D-03
26	-0.878855D-05	-0.353745D-05	0.499212D-05	-0.570888D-07	-0.512596D-05
27	-0.260114D-05	-0.141207D-04	0.899200D-05	-0.415095D-05	-0.128615D-04
28	0.617460D-05	0.789555D-05	-0.409192D-04	0.373392D-05	0.555068D-05
29	-0.324408D-05	-0.673239D-05	0.120311D-05	-0.972338D-05	-0.105942D-04
30	-0.102670D-04	-0.245278D-04	0.402083D-04	-0.199391D-04	-0.779736D-04
31	0.351593D-05	0.938729D-05	-0.101098D-04	0.518014D-05	0.198374D-04
32	0.892735D-05	-0.465445D-06	0.348109D-04	0.131993D-04	0.182363D-04
33	0.579793D-05	-0.146783D-05	0.344403D-04	-0.163092D-04	-0.980696D-04
34	0.172440D-05	0.323224D-05	0.547398D-05	0.338885D-05	0.110557D-04
35	0.574987D-05	0.218833D-04	-0.488648D-05	0.926113D-05	0.365858D-04
36	-0.315332D-05	0.971942D-05	0.154311D-04	-0.184922D-05	0.138968D-04
37	-0.136955D-03	-0.177240D-03	0.175602D-03	0.486803D-05	-0.326259D-03
38	-0.196908D-04	-0.349928D-05	-0.415285D-04	0.105477D-05	-0.976100D-04
39	0.965919D-06	0.470878D-06	-0.159303D-05	-0.241283D-05	-0.298383D-05
40	0.992950D-05	0.412782D-04	-0.778507D-04	-0.573600D-05	0.434025D-04
41	-0.241072D-05	0.162712D-05	0.407171D-05	0.201175D-06	0.524917D-05
42	0.401007D-06	-0.970520D-07	0.864820D-06	-0.452574D-07	0.132490D-05
43	-0.170461D-03	-0.214754D-03	0.225490D-03	-0.522294D-05	-0.392878D-03

ESTIMATED COVARIANCE MATRIX FOR PARAMETER ESTIMATES

	26	27	28	29	30
26	0.399510D-04				
27	0.944524D-05	0.547422D-04			
28	-0.864874D-05	-0.245041D-04	0.184878D-03		

29	0.193980D-05	0.132424D-04	-0.122456D-04	0.421854D-04	
30	0.801025D-05	0.246622D-04	-0.243423D-04	0.238073D-04	0.187267D-03
31	-0.115627D-04	-0.427715D-04	0.681545D-04	-0.317785D-04	-0.760161D-04
32	-0.448698D-05	-0.325309D-05	-0.163367D-04	-0.193638D-04	-0.281764D-04
33	-0.202458D-04	0.226881D-04	-0.646958D-04	0.221656D-04	0.361906D-04
34	0.259991D-05	-0.207973D-05	0.794568D-05	-0.518494D-05	-0.730104D-05
35	0.271776D-04	-0.470847D-05	-0.666926D-05	0.421621D-05	-0.591104D-05
36	-0.591394D-05	0.242074D-05	0.143367D-05	0.569041D-05	0.441044D-05
37	-0.305620D-04	-0.123720D-04	-0.119957D-03	0.573871D-04	0.130449D-03
38	0.104491D-04	0.561448D-05	0.885514D-05	-0.131289D-04	0.282007D-04
39	-0.329760D-07	0.906519D-08	-0.550317D-06	0.985963D-06	0.144814D-05
40	-0.130229D-04	-0.684604D-04	0.713910D-05	0.457519D-04	0.102356D-03
41	0.150881D-05	0.242952D-06	-0.747642D-05	-0.703618D-05	0.220611D-05
42	-0.384119D-06	0.896232D-07	0.126955D-05	0.161455D-05	0.948831D-06
43	-0.369844D-04	-0.679960D-05	-0.156399D-03	0.679029D-04	0.156520D-03

ESTIMATED COVARIANCE MATRIX FOR PARAMETER ESTIMATES

	31	32	33	34	35
31	0.314754D-03				
32	0.192041D-04	0.738994D-02			
33	-0.302740D-04	-0.163346D-03	0.241271D-01		
34	0.851132D-05	0.334504D-04	0.441421D-03	0.502372D-03	
35	-0.141473D-04	0.161221D-02	0.297445D-03	0.244442D-04	0.623568D-02
36	-0.432132D-05	0.225948D-03	0.113134D-03	-0.331561D-04	0.168302D-03
37	0.230038D-03	-0.217954D-02	0.288580D-02	-0.145984D-03	0.172100D-02
38	0.127366D-04	-0.170279D-02	0.414074D-03	0.193440D-04	-0.694927D-03
39	0.577704D-06	0.359137D-05	0.156286D-04	0.200741D-05	-0.422682D-04
40	0.737277D-05	0.111250D-02	-0.362987D-03	0.286771D-03	0.914604D-03
41	0.466999D-06	0.125562D-04	-0.307039D-04	0.394982D-05	0.107535D-03
42	0.101445D-06	0.297327D-05	0.157016D-04	0.301904D-05	-0.632490D-05
43	0.304050D-03	-0.283881D-02	0.357751D-02	-0.161311D-03	0.162575D-02

ESTIMATED COVARIANCE MATRIX FOR PARAMETER ESTIMATES

	36	37	38	39	40
36	0.973632D-03				
37	-0.486678D-02	0.372824D+00			
38	-0.550082D-04	-0.802146D-03	0.387747D-02		
39	-0.560598D-04	-0.473008D-03	-0.347887D-04	0.128259D-03	
40	0.312657D-03	-0.248408D-02	-0.449158D-03	-0.409815D-04	0.679030D-01
41	0.341947D-05	-0.190958D-03	-0.445854D-04	0.287998D-05	0.550590D-03

42	0.409608D-05	-0.880437D-04	0.383949D-05	-0.146557D-05	0.580680D-04
43	-0.585480D-02	0.435362D+00	-0.549995D-03	-0.471040D-03	-0.309823D-02

ESTIMATED COVARIANCE MATRIX FOR PARAMETER ESTIMATES

	41	42	43
41	0.710736D-03		
42	0.566900D-05	0.253128D-04	
43	-0.244445D-03	-0.111242D-03	0.509752D+00

ESTIMATED CORRELATION MATRIX FOR PARAMETER ESTIMATES

	1	2	3	4	5
1	1.000				
2	0.192	1.000			
3	-0.073	-0.090	1.000		
4	0.058	0.107	-0.047	1.000	
5	-0.062	-0.174	0.143	-0.207	1.000
6	0.024	-0.004	0.056	-0.005	-0.029
7	-0.049	0.009	0.000	-0.024	0.010
8	-0.004	-0.036	0.024	0.010	-0.028
9	0.068	0.012	-0.018	0.004	0.001
10	0.025	-0.003	0.001	-0.046	0.040
11	0.020	0.000	0.008	-0.019	0.009
12	-0.045	-0.035	0.000	-0.039	-0.010
13	0.021	0.026	-0.021	0.008	-0.029
14	0.005	-0.007	-0.042	0.017	0.014
15	0.019	-0.029	-0.007	-0.044	0.010
16	-0.051	0.015	0.025	0.017	0.027
17	0.005	0.017	-0.008	-0.045	0.030
18	0.002	0.029	0.005	0.023	-0.012
19	0.001	-0.010	0.040	-0.017	0.012
20	0.028	0.052	0.021	0.001	-0.018
21	-0.012	0.023	0.025	-0.021	0.020
22	-0.018	-0.012	0.027	0.002	0.003
23	-0.026	-0.027	0.027	-0.017	-0.046
24	0.022	0.023	-0.016	0.014	0.032
25	-0.008	-0.003	-0.010	0.014	0.011
26	0.055	-0.007	-0.054	0.038	0.006
27	0.040	-0.005	0.015	-0.042	0.030
28	-0.040	0.048	0.004	0.047	-0.012
29	0.021	-0.006	0.015	-0.063	0.025

30	0.015	0.021	-0.018	-0.010	-0.034
31	-0.039	0.007	-0.006	0.044	-0.062
32	-0.023	0.006	-0.042	0.012	0.029
33	-0.026	-0.036	0.043	-0.026	0.022
34	-0.036	0.051	-0.044	0.068	-0.036
35	0.030	-0.003	0.008	-0.019	0.016
36	-0.019	0.013	0.040	-0.053	0.053
37	0.013	0.018	0.050	-0.014	-0.028
38	-0.042	0.038	0.022	-0.007	-0.035
39	-0.009	-0.052	0.002	-0.032	-0.007
40	-0.024	-0.027	0.030	0.036	-0.010
41	-0.022	-0.020	-0.002	0.006	-0.009
42	-0.035	-0.021	-0.034	-0.028	-0.044
43	0.014	0.018	0.049	-0.013	-0.029

ESTIMATED CORRELATION MATRIX FOR PARAMETER ESTIMATES

	6	7	8	9	10
6	1.000				
7	-0.096	1.000			
8	-0.002	0.089	1.000		
9	0.006	-0.110	0.029	1.000	
10	0.044	0.121	-0.151	0.153	1.000
11	0.016	0.042	-0.032	-0.002	0.025
12	-0.035	0.029	-0.001	-0.011	-0.026
13	-0.032	0.014	-0.028	0.002	-0.009
14	0.002	-0.006	0.059	-0.006	-0.029
15	0.059	-0.018	-0.010	-0.012	0.013
16	-0.001	0.048	0.026	-0.013	0.003
17	-0.017	0.000	-0.009	-0.004	-0.015
18	-0.011	0.034	-0.009	0.013	-0.015
19	0.050	-0.027	-0.005	0.021	0.005
20	-0.028	0.034	-0.012	0.020	0.023
21	-0.037	0.001	-0.001	0.031	-0.020
22	-0.032	0.026	0.009	0.000	0.010
23	0.054	-0.030	-0.004	0.030	0.008
24	-0.022	0.032	-0.033	0.024	0.055
25	-0.030	0.027	-0.010	-0.002	0.029
26	0.028	0.008	0.039	-0.011	0.027
27	0.028	-0.020	-0.021	0.015	0.012
28	-0.028	0.012	0.051	-0.016	-0.029
29	0.020	-0.055	0.001	-0.007	0.023
30	0.032	-0.017	0.028	-0.002	0.011

31	0.005	-0.008	-0.028	-0.006	0.008
32	-0.078	-0.024	0.009	-0.103	0.105
33	0.000	0.031	0.008	-0.009	-0.010
34	-0.011	0.041	0.010	0.004	0.043
35	-0.086	-0.042	-0.059	-0.007	-0.007
36	-0.050	0.042	-0.013	-0.093	0.013
37	0.008	0.005	0.019	0.039	-0.008
38	0.104	-0.018	-0.053	-0.006	-0.028
39	0.046	0.067	0.006	0.028	-0.001
40	-0.002	0.005	0.021	-0.027	-0.003
41	0.005	-0.031	0.039	0.028	0.002
42	-0.008	-0.046	0.045	0.012	-0.037
43	0.028	0.008	0.015	0.039	-0.007

ESTIMATED CORRELATION MATRIX FOR PARAMETER ESTIMATES

	11	12	13	14	15
11	1.000				
12	0.270	1.000			
13	0.021	0.297	1.000		
14	-0.116	-0.149	-0.090	1.000	
15	0.006	-0.114	-0.281	0.201	1.000
16	0.031	0.021	0.055	-0.098	-0.216
17	0.043	0.222	0.059	-0.122	-0.016
18	-0.013	0.104	0.326	-0.076	-0.170
19	-0.036	-0.057	-0.065	0.080	0.191
20	0.041	0.023	0.067	0.003	-0.013
21	0.103	0.212	0.079	-0.095	-0.042
22	0.085	0.171	0.281	-0.075	-0.161
23	-0.028	-0.066	-0.084	0.086	0.177
24	0.022	0.071	0.088	-0.042	-0.057
25	0.041	0.041	0.071	-0.055	-0.053
26	-0.146	-0.261	-0.073	0.198	0.030
27	-0.055	-0.177	-0.347	0.096	0.229
28	0.025	0.048	0.076	-0.088	-0.233
29	-0.060	-0.091	-0.097	0.066	0.075
30	-0.036	-0.043	-0.088	0.019	0.051
31	0.040	0.049	0.084	-0.017	-0.076
32	0.000	0.032	0.029	-0.025	0.020
33	0.025	0.036	0.001	0.017	0.045
34	0.005	0.008	0.040	-0.028	-0.036
35	-0.046	-0.022	0.058	0.038	-0.093
36	0.008	0.045	0.005	0.006	-0.023

37	-0.002	-0.017	-0.007	-0.017	0.031
38	0.026	-0.015	-0.036	-0.012	-0.011
39	0.057	0.019	0.017	-0.046	0.006
40	0.017	-0.005	0.010	-0.001	-0.019
41	-0.031	-0.009	-0.001	-0.018	0.005
42	-0.022	-0.006	-0.008	-0.043	-0.033
43	0.001	-0.018	-0.008	-0.019	0.034

ESTIMATED CORRELATION MATRIX FOR PARAMETER ESTIMATES

	16	17	18	19	20
16	1.000				
17	-0.010	1.000			
18	0.075	0.183	1.000		
19	-0.180	-0.048	-0.160	1.000	
20	0.010	0.035	0.264	-0.150	1.000
21	0.015	0.229	0.088	-0.059	0.047
22	0.085	0.075	0.272	-0.059	0.048
23	-0.142	-0.096	-0.028	0.231	0.001
24	0.027	0.078	0.217	-0.137	0.265
25	0.017	0.029	0.067	-0.028	0.055
26	-0.034	-0.204	-0.045	0.026	0.007
27	-0.071	-0.039	-0.214	0.081	-0.067
28	0.223	0.040	0.076	-0.193	-0.018
29	-0.027	-0.096	-0.247	0.230	-0.243
30	-0.010	-0.010	-0.102	0.101	-0.069
31	0.036	0.005	0.047	-0.072	0.023
32	-0.003	0.032	0.012	0.011	0.004
33	0.024	-0.004	-0.039	0.048	-0.008
34	0.018	0.029	0.043	-0.018	0.011
35	0.037	-0.001	0.038	0.008	-0.027
36	-0.015	-0.009	-0.011	0.020	-0.004
37	-0.012	0.009	-0.011	0.012	0.006
38	-0.004	0.014	0.007	-0.041	0.012
39	0.010	0.009	0.010	0.003	-0.023
40	-0.005	0.018	0.009	0.021	-0.005
41	0.011	0.010	0.033	-0.002	0.043
42	0.020	0.011	-0.006	0.019	-0.035
43	-0.013	0.008	-0.010	0.014	0.003

ESTIMATED CORRELATION MATRIX FOR PARAMETER ESTIMATES

21	22	23	24	25

21	1.000				
22	0.222	1.000			
23	-0.105	-0.173	1.000		
24	0.092	0.231	-0.123	1.000	
25	0.157	0.300	-0.102	0.274	1.000
26	-0.205	-0.075	0.054	-0.001	-0.040
27	-0.052	-0.255	0.083	-0.079	-0.085
28	0.067	0.077	-0.205	0.039	0.020
29	-0.073	-0.138	0.013	-0.212	-0.080
30	-0.110	-0.239	0.200	-0.206	-0.280
31	0.029	0.071	-0.039	0.041	0.055
32	0.015	-0.001	0.028	0.022	0.010
33	0.005	-0.001	0.015	-0.015	-0.031
34	0.011	0.019	0.017	0.021	0.024
35	0.011	0.037	-0.004	0.017	0.023
36	-0.015	0.042	0.034	-0.008	0.022
37	-0.033	-0.039	0.020	0.001	-0.026
38	-0.047	-0.007	-0.045	0.002	-0.077
39	0.013	0.006	-0.010	-0.030	-0.013
40	0.006	0.021	-0.020	-0.003	0.008
41	-0.013	0.008	0.010	0.001	0.010
42	0.012	-0.003	0.012	-0.001	0.013
43	-0.035	-0.040	0.022	-0.001	-0.027

ESTIMATED CORRELATION MATRIX FOR PARAMETER ESTIMATES

	26	27	28	29	30
26	1.000				
27	0.202	1.000			
28	-0.101	-0.244	1.000		
29	0.047	0.276	-0.139	1.000	
30	0.093	0.244	-0.131	0.268	1.000
31	-0.103	-0.326	0.283	-0.276	-0.313
32	-0.008	-0.005	-0.014	-0.035	-0.024
33	-0.021	0.020	-0.031	0.022	0.017
34	0.018	-0.013	0.026	-0.036	-0.024
35	0.054	-0.008	-0.006	0.008	-0.005
36	-0.030	0.010	0.003	0.028	0.010
37	-0.008	-0.003	-0.014	0.014	0.016
38	0.027	0.012	0.010	-0.032	0.033
39	0.000	0.000	-0.004	0.013	0.009
40	-0.008	-0.036	0.002	0.027	0.029

41	0.009	0.001	-0.021	-0.041	0.006
42	-0.012	0.002	0.019	0.049	0.014
43	-0.008	-0.001	-0.016	0.015	0.016

ESTIMATED CORRELATION MATRIX FOR PARAMETER ESTIMATES

	31	32	33	34	35
31	1.000				
32	0.013	1.000			
33	-0.011	-0.012	1.000		
34	0.021	0.017	0.127	1.000	
35	-0.010	0.237	0.024	0.014	1.000
36	-0.008	0.084	0.023	-0.047	0.068
37	0.021	-0.042	0.030	-0.011	0.036
38	0.012	-0.318	0.043	0.014	-0.141
39	0.003	0.004	0.009	0.008	-0.047
40	0.002	0.050	-0.009	0.049	0.044
41	0.001	0.005	-0.007	0.007	0.051
42	0.001	0.007	0.020	0.027	-0.016
43	0.024	-0.046	0.032	-0.010	0.029

ESTIMATED CORRELATION MATRIX FOR PARAMETER ESTIMATES

	36	37	38	39	40
36	1.000				
37	-0.255	1.000			
38	-0.028	-0.021	1.000		
39	-0.159	-0.068	-0.049	1.000	
40	0.038	-0.016	-0.028	-0.014	1.000
41	0.004	-0.012	-0.027	0.010	0.079
42	0.026	-0.029	0.012	-0.026	0.044
43	-0.263	0.999	-0.012	-0.058	-0.017

ESTIMATED CORRELATION MATRIX FOR PARAMETER ESTIMATES

	41	42	43
41	1.000		
42	0.042	1.000	
43	-0.013	-0.031	1.000

TECHNICAL 8 OUTPUT

TECHNICAL 8 OUTPUT FOR BAYES ESTIMATION

CHAIN	BSEED
1	0
2	285380
3	253358

ITERATION	POTENTIAL SCALE REDUCTION	PARAMETER WITH HIGHEST PSR
100	1.011	38
200	1.008	7
300	1.001	33
400	1.004	35
500	1.004	41
600	1.008	41
700	1.003	41
800	1.002	41
900	1.003	41
1000	1.002	23

Deterioration in physical health, time pressure interaction

SUMMARY OF DATA

Number of clusters 58

Size (s)	Cluster ID with Size s
7	22
16	166
23	80 71 205
25	74
27	192
31	65
32	106
35	228
37	170 72
39	50 75 181
40	182
42	165
44	114
45	173
46	107
47	169 30
48	226 81
49	36
50	116 108 40
56	162 84
57	61
59	206 93 83
60	92
61	69
63	6
64	152
65	145
66	4
68	68
69	154
72	174
78	98
84	178 2
95	156 202
96	144
108	142

131	122
172	24
175	209
224	204
269	125
364	217
738	110
860	100

COVARIANCE COVERAGE OF DATA

Minimum covariance coverage value 0.100

Number of missing data patterns 8

PROPORTION OF DATA PRESENT

Covariance Coverage

	PHYCAT16	PRAFAC	PHY15	KES15	CHOICE15
PHYCAT16	1.000				
PRAFAC	1.000	1.000			
PHY15	0.947	0.947	0.947		
KES15	0.922	0.922	0.922	0.922	
CHOICE15	0.915	0.915	0.915	0.915	0.915
BULLY15	0.915	0.915	0.915	0.915	0.915
PRESS15	0.915	0.915	0.915	0.915	0.915
DUTIES15	0.915	0.915	0.915	0.915	0.915

Covariance Coverage

	BULLY15	PRESS15	DUTIES15
BULLY15	0.915		
PRESS15	0.914	0.915	
DUTIES15	0.915	0.915	0.915

UNIVARIATE PROPORTIONS AND COUNTS FOR CATEGORICAL VARIABLES

PHYCAT16

Category 1	0.881	4884.000
Category 2	0.119	662.000

UNIVARIATE SAMPLE STATISTICS

UNIVARIATE HIGHER-ORDER MOMENT DESCRIPTIVE STATISTICS

Variable/ Sample Size	Mean/ Variance	Skewness/ Kurtosis	Minimum/ Maximum	% with Min/Max	Percentiles 20%/60%	40%/80%	Median
PRAFAC	-0.185	0.245	-2.338	1.72%	-1.448	-0.527	-0.318
58.000	1.345	-0.636	2.338	1.72%	0.080	0.752	
PHY15	0.000	-0.546	-0.632	36.83%	-0.632	0.368	0.368
5254.000	0.233	-1.702	0.368	63.17%	0.368	0.368	
KES15	0.000	1.717	-0.511	21.65%	-0.511	-0.178	-0.178
5114.000	0.289	3.835	3.489	0.04%	-0.011	0.322	
CHOICE15	0.000	-0.402	-2.225	7.41%	-1.225	-0.225	-0.225
5077.000	1.081	-0.336	1.775	8.49%	0.775	0.775	
BULLY15	0.000	3.272	-0.170	87.25%	-0.170	-0.170	-0.170
5073.000	0.239	11.772	3.830	0.08%	-0.170	-0.170	
PRESS15	2.462	0.252	1.000	19.10%	2.000	2.000	2.000
5074.000	1.001	-0.371	5.000	2.76%	3.000	3.000	
DUTIES15	0.000	-1.246	-3.120	2.40%	-0.120	-0.120	-0.120
5077.000	0.861	1.673	0.880	38.74%	-0.120	0.880	

THE MODEL ESTIMATION TERMINATED NORMALLY

USE THE FBITERATIONS OPTION TO INCREASE THE NUMBER OF ITERATIONS BY A FACTOR
OF AT LEAST TWO TO CHECK CONVERGENCE AND THAT THE PSR VALUE DOES NOT INCREASE.

MODEL FIT INFORMATION

Number of Free Parameters 43

MODEL RESULTS

	Estimate	Posterior S.D.	One-Tailed P-Value	95% C.I. Lower 2.5%	95% C.I. Upper 2.5%	Significance
**Within Level**						
PHYCAT16 ON						
PHY15	0.976	0.070	0.000	0.834	1.121	*
CHOICE15	-0.069	0.025	0.002	-0.118	-0.023	*
DUTIES15	-0.030	0.027	0.131	-0.083	0.020	
BULLY15	0.030	0.048	0.269	-0.060	0.123	
KES15	0.224	0.045	0.000	0.140	0.307	*
KES15 WITH						
PHY15	0.051	0.004	0.000	0.044	0.059	*
CHOICE15	-0.089	0.008	0.000	-0.105	-0.073	*
BULLY15	0.057	0.004	0.000	0.050	0.066	*
PRESS15	0.099	0.007	0.000	0.085	0.114	*
DUTIES15	-0.106	0.007	0.000	-0.120	-0.091	*
PHY15 WITH						
CHOICE15	-0.036	0.007	0.000	-0.051	-0.023	*
BULLY15	0.012	0.003	0.000	0.005	0.018	*
PRESS15	0.036	0.007	0.000	0.023	0.049	*
DUTIES15	-0.036	0.006	0.000	-0.049	-0.024	*
CHOICE15 WITH						
BULLY15	-0.058	0.007	0.000	-0.072	-0.044	*
PRESS15	-0.075	0.015	0.000	-0.106	-0.048	*
DUTIES15	0.174	0.014	0.000	0.146	0.201	*
BULLY15 WITH						
PRESS15	0.101	0.007	0.000	0.088	0.115	*
DUTIES15	-0.078	0.006	0.000	-0.090	-0.065	*
PRESS15 WITH						
DUTIES15	-0.175	0.014	0.000	-0.203	-0.150	*
Means						
PHY15	0.002	0.007	0.377	-0.011	0.016	
KES15	0.001	0.008	0.443	-0.014	0.017	
CHOICE15	-0.001	0.015	0.463	-0.030	0.029	
BULLY15	0.000	0.007	0.479	-0.012	0.014	
DUTIES15	-0.001	0.013	0.479	-0.027	0.024	

```
 Variances
    PHY15              0.233      0.005      0.000       0.225       0.242      *
    KES15              0.290      0.006      0.000       0.278       0.302      *
    CHOICE15           1.083      0.020      0.000       1.043       1.123      *
    BULLY15            0.239      0.005      0.000       0.230       0.248      *
    PRESS15            0.953      0.020      0.000       0.917       0.994      *
    DUTIES15           0.861      0.018      0.000       0.829       0.897      *

Between Level

 S1         ON
    PRAFAC            -0.003      0.029      0.457      -0.059       0.052

 PHYCAT16   ON
    PRAFAC            -0.082      0.031      0.005      -0.144      -0.020      *
    PRESS15           -0.151      0.160      0.163      -0.484       0.153

 PRAFAC   WITH
    PRESS15            0.004      0.050      0.469      -0.104       0.095

 Means
    PRAFAC            -0.182      0.155      0.122      -0.486       0.118
    PRESS15            2.386      0.041      0.000       2.302       2.465      *

 Intercepts
    S1                -0.027      0.032      0.199      -0.087       0.036

 Thresholds
    PHYCAT16$1         0.976      0.389      0.009       0.187       1.734      *

 Variances
    PRAFAC             1.342      0.261      0.000       0.958       1.980      *
    PRESS15            0.078      0.018      0.000       0.051       0.124      *

 Residual Variances
    PHYCAT16           0.014      0.012      0.000       0.001       0.045      *
    S1                 0.004      0.006      0.000       0.001       0.021      *


STANDARDIZED MODEL RESULTS


STDYX Standardization
```

	Estimate	Posterior S.D.	One-Tailed P-Value	95% C.I. Lower 2.5%	Upper 2.5%	Significance
**Within-Level Standardized Estimates Averaged Over Clusters**						
S1 | PHYCAT16 ON						
PRESS15	-0.023	0.025	0.186	-0.072	0.026	
PHYCAT16 ON						
PHY15	0.416	0.025	0.000	0.364	0.465	*
CHOICE15	-0.064	0.023	0.002	-0.108	-0.020	*
DUTIES15	-0.025	0.022	0.132	-0.068	0.018	
BULLY15	0.013	0.021	0.264	-0.027	0.055	
KES15	0.106	0.021	0.000	0.067	0.146	*
KES15 WITH						
PHY15	0.198	0.013	0.000	0.171	0.223	*
CHOICE15	-0.159	0.014	0.000	-0.185	-0.131	*
BULLY15	0.219	0.014	0.000	0.191	0.245	*
PRESS15	0.189	0.013	0.000	0.163	0.215	*
DUTIES15	-0.212	0.014	0.000	-0.239	-0.184	*
PHY15 WITH						
CHOICE15	-0.072	0.014	0.000	-0.099	-0.045	*
BULLY15	0.050	0.014	0.000	0.022	0.076	*
PRESS15	0.076	0.014	0.000	0.049	0.105	*
DUTIES15	-0.081	0.014	0.000	-0.108	-0.053	*
CHOICE15 WITH						
BULLY15	-0.114	0.014	0.000	-0.141	-0.088	*
PRESS15	-0.073	0.014	0.000	-0.103	-0.046	*
DUTIES15	0.179	0.014	0.000	0.152	0.206	*
BULLY15 WITH						
PRESS15	0.213	0.013	0.000	0.186	0.238	*
DUTIES15	-0.171	0.013	0.000	-0.197	-0.145	*
PRESS15 WITH						
DUTIES15	-0.194	0.014	0.000	-0.221	-0.167	*
Means						
PHY15	0.004	0.014	0.385	-0.023	0.032	
KES15	0.002	0.014	0.436	-0.025	0.030	

```
    CHOICE15          -0.001       0.014       0.456      -0.029       0.027
    BULLY15            0.001       0.014       0.475      -0.026       0.028
    DUTIES15          -0.001       0.014       0.474      -0.028       0.026

 Variances
    PHY15              1.000       0.000       0.000       1.000       1.000
    KES15              1.000       0.000       0.000       1.000       1.000
    CHOICE15           1.000       0.000       0.000       1.000       1.000
    BULLY15            1.000       0.000       0.000       1.000       1.000
    PRESS15            1.000       0.000       0.000       1.000       1.000
    DUTIES15           1.000       0.000       0.000       1.000       1.000

Between Level

 S1         ON
    PRAFAC            -0.050       0.455       0.457      -0.841       0.779

 PHYCAT16   ON
    PRAFAC            -0.593       0.190       0.005      -0.909      -0.172      *
    PRESS15           -0.277       0.261       0.163      -0.723       0.310

 PRAFAC   WITH
    PRESS15            0.012       0.143       0.469      -0.283       0.283

 Means
    PRAFAC            -0.159       0.133       0.122      -0.416       0.107
    PRESS15            8.525       0.929       0.000       6.823      10.564      *

 Intercepts
    S1                -0.367       0.530       0.199      -1.673       0.525

 Variances
    PRAFAC             1.000       0.000       0.000       1.000       1.000
    PRESS15            1.000       0.000       0.000       1.000       1.000

 Residual Variances
    PHYCAT16           0.510       0.213       0.000       0.066       0.900      *
    S1                 0.869       0.223       0.000       0.212       1.000      *


R-SQUARE

Within-Level R-Square Averaged Across Clusters
```

Variable	Estimate	Posterior S.D.	One-Tailed P-Value	95% C.I. Lower 2.5%	Upper 2.5%
PHYCAT16	0.221	0.022	0.000	0.180	0.266

Between Level

Variable	Estimate	Posterior S.D.	One-Tailed P-Value	95% C.I. Lower 2.5%	Upper 2.5%
PHYCAT16	0.490	0.213	0.000	0.100	0.934

Variable	Estimate	Posterior S.D.	One-Tailed P-Value	95% C.I. Lower 2.5%	Upper 2.5%
S1	0.131	0.223	0.000	0.000	0.788

TECHNICAL 1 OUTPUT

PARAMETER SPECIFICATION FOR WITHIN

TAU

PHYCAT16
0

NU

PHYCAT16	PHY15	KES15	CHOICE15	BULLY15
0	0	0	0	0

NU

PRESS15	DUTIES15
0	0

LAMBDA

PHYCAT16 PHY15 KES15 CHOICE15 BULLY15

PHYCAT16	0	0	0	0	0
PHY15	0	0	0	0	0
KES15	0	0	0	0	0
CHOICE15	0	0	0	0	0
BULLY15	0	0	0	0	0
PRESS15	0	0	0	0	0
DUTIES15	0	0	0	0	0

LAMBDA

	PRESS15	DUTIES15
PHYCAT16	0	0
PHY15	0	0
KES15	0	0
CHOICE15	0	0
BULLY15	0	0
PRESS15	0	0
DUTIES15	0	0

THETA

	PHYCAT16	PHY15	KES15	CHOICE15	BULLY15
PHYCAT16	0				
PHY15	0	0			
KES15	0	0	0		
CHOICE15	0	0	0	0	
BULLY15	0	0	0	0	0
PRESS15	0	0	0	0	0
DUTIES15	0	0	0	0	0

THETA

	PRESS15	DUTIES15
PRESS15	0	
DUTIES15	0	0

ALPHA

PHYCAT16	PHY15	KES15	CHOICE15	BULLY15

	0	1	2	3	4

ALPHA

PRESS15	DUTIES15
0	5

BETA

	PHYCAT16	PHY15	KES15	CHOICE15	BULLY15
PHYCAT16	0	6	7	8	9
PHY15	0	0	0	0	0
KES15	0	0	0	0	0
CHOICE15	0	0	0	0	0
BULLY15	0	0	0	0	0
PRESS15	0	0	0	0	0
DUTIES15	0	0	0	0	0

BETA

	PRESS15	DUTIES15
PHYCAT16	0	10
PHY15	0	0
KES15	0	0
CHOICE15	0	0
BULLY15	0	0
PRESS15	0	0
DUTIES15	0	0

PSI

	PHYCAT16	PHY15	KES15	CHOICE15	BULLY15
PHYCAT16	0				
PHY15	0	11			
KES15	0	12	13		
CHOICE15	0	14	15	16	
BULLY15	0	17	18	19	20
PRESS15	0	21	22	23	24
DUTIES15	0	26	27	28	29

PSI

	PRESS15	DUTIES15
PRESS15	25	
DUTIES15	30	31

PARAMETER SPECIFICATION FOR BETWEEN

TAU

PHYCAT16
43

NU

PHYCAT16	PRAFAC	PRESS15
0	0	0

LAMBDA

	S1	PHYCAT16	PRAFAC	PRESS15
PHYCAT16	0	0	0	0
PRAFAC	0	0	0	0
PRESS15	0	0	0	0

THETA

	PHYCAT16	PRAFAC	PRESS15
PHYCAT16	0		
PRAFAC	0	0	
PRESS15	0	0	0

ALPHA

S1	PHYCAT16	PRAFAC	PRESS15
32	0	33	34

BETA

	S1	PHYCAT16	PRAFAC	PRESS15
S1	0	0	35	0
PHYCAT16	0	0	36	37
PRAFAC	0	0	0	0
PRESS15	0	0	0	0

PSI

	S1	PHYCAT16	PRAFAC	PRESS15
S1	38			
PHYCAT16	0	39		
PRAFAC	0	0	40	
PRESS15	0	0	41	42

STARTING VALUES FOR WITHIN

TAU

PHYCAT16
0.000

NU

PHYCAT16	PHY15	KES15	CHOICE15	BULLY15
0.000	0.000	0.000	0.000	0.000

NU

PRESS15	DUTIES15
0.000	0.000

LAMBDA

	PHYCAT16	PHY15	KES15	CHOICE15	BULLY15
PHYCAT16	1.000	0.000	0.000	0.000	0.000

PHY15	0.000	1.000	0.000	0.000	0.000
KES15	0.000	0.000	1.000	0.000	0.000
CHOICE15	0.000	0.000	0.000	1.000	0.000
BULLY15	0.000	0.000	0.000	0.000	1.000
PRESS15	0.000	0.000	0.000	0.000	0.000
DUTIES15	0.000	0.000	0.000	0.000	0.000

LAMBDA

	PRESS15	DUTIES15
PHYCAT16	0.000	0.000
PHY15	0.000	0.000
KES15	0.000	0.000
CHOICE15	0.000	0.000
BULLY15	0.000	0.000
PRESS15	1.000	0.000
DUTIES15	0.000	1.000

THETA

	PHYCAT16	PHY15	KES15	CHOICE15	BULLY15
PHYCAT16	0.000				
PHY15	0.000	0.000			
KES15	0.000	0.000	0.000		
CHOICE15	0.000	0.000	0.000	0.000	
BULLY15	0.000	0.000	0.000	0.000	0.000
PRESS15	0.000	0.000	0.000	0.000	0.000
DUTIES15	0.000	0.000	0.000	0.000	0.000

THETA

	PRESS15	DUTIES15
PRESS15	0.000	
DUTIES15	0.000	0.000

ALPHA

PHYCAT16	PHY15	KES15	CHOICE15	BULLY15
0.000	0.000	0.000	0.000	0.000

ALPHA

PRESS15	DUTIES15
0.000	0.000

BETA

	PHYCAT16	PHY15	KES15	CHOICE15	BULLY15
PHYCAT16	0.000	0.000	0.000	0.000	0.000
PHY15	0.000	0.000	0.000	0.000	0.000
KES15	0.000	0.000	0.000	0.000	0.000
CHOICE15	0.000	0.000	0.000	0.000	0.000
BULLY15	0.000	0.000	0.000	0.000	0.000
PRESS15	0.000	0.000	0.000	0.000	0.000
DUTIES15	0.000	0.000	0.000	0.000	0.000

BETA

	PRESS15	DUTIES15
PHYCAT16	0.000	0.000
PHY15	0.000	0.000
KES15	0.000	0.000
CHOICE15	0.000	0.000
BULLY15	0.000	0.000
PRESS15	0.000	0.000
DUTIES15	0.000	0.000

PSI

	PHYCAT16	PHY15	KES15	CHOICE15	BULLY15
PHYCAT16	1.000				
PHY15	0.000	0.116			
KES15	0.000	0.000	0.145		
CHOICE15	0.000	0.000	0.000	0.541	
BULLY15	0.000	0.000	0.000	0.000	0.119
PRESS15	0.000	0.000	0.000	0.000	0.000
DUTIES15	0.000	0.000	0.000	0.000	0.000

PSI

	PRESS15	DUTIES15
PRESS15	0.501	
DUTIES15	0.000	0.430

STARTING VALUES FOR BETWEEN

TAU

PHYCAT16
1.110

NU

PHYCAT16	PRAFAC	PRESS15
0.000	0.000	0.000

LAMBDA

	S1	PHYCAT16	PRAFAC	PRESS15
PHYCAT16	0.000	1.000	0.000	0.000
PRAFAC	0.000	0.000	1.000	0.000
PRESS15	0.000	0.000	0.000	1.000

THETA

	PHYCAT16	PRAFAC	PRESS15
PHYCAT16	0.000		
PRAFAC	0.000	0.000	
PRESS15	0.000	0.000	0.000

ALPHA

S1	PHYCAT16	PRAFAC	PRESS15
0.000	0.000	0.020	2.462

BETA

	S1	PHYCAT16	PRAFAC	PRESS15
S1	0.000	0.000	0.000	0.000
PHYCAT16	0.000	0.000	0.000	0.000
PRAFAC	0.000	0.000	0.000	0.000
PRESS15	0.000	0.000	0.000	0.000

PSI

	S1	PHYCAT16	PRAFAC	PRESS15
S1	1.000			
PHYCAT16	0.000	1.000		
PRAFAC	0.000	0.000	0.504	
PRESS15	0.000	0.000	0.000	0.501

PRIORS FOR ALL PARAMETERS	PRIOR MEAN	PRIOR VARIANCE	PRIOR STD. DEV.
Parameter 1~N(0.000,infinity)	0.0000	infinity	infinity
Parameter 2~N(0.000,infinity)	0.0000	infinity	infinity
Parameter 3~N(0.000,infinity)	0.0000	infinity	infinity
Parameter 4~N(0.000,infinity)	0.0000	infinity	infinity
Parameter 5~N(0.000,infinity)	0.0000	infinity	infinity
Parameter 6~N(0.000,5.000)	0.0000	5.0000	2.2361
Parameter 7~N(0.000,5.000)	0.0000	5.0000	2.2361
Parameter 8~N(0.000,5.000)	0.0000	5.0000	2.2361
Parameter 9~N(0.000,5.000)	0.0000	5.0000	2.2361
Parameter 10~N(0.000,5.000)	0.0000	5.0000	2.2361
Parameter 11~IW(1.000,7)	infinity	infinity	infinity
Parameter 12~IW(0.000,7)	infinity	infinity	infinity
Parameter 13~IW(1.000,7)	infinity	infinity	infinity
Parameter 14~IW(0.000,7)	infinity	infinity	infinity
Parameter 15~IW(0.000,7)	infinity	infinity	infinity
Parameter 16~IW(1.000,7)	infinity	infinity	infinity
Parameter 17~IW(0.000,7)	infinity	infinity	infinity
Parameter 18~IW(0.000,7)	infinity	infinity	infinity
Parameter 19~IW(0.000,7)	infinity	infinity	infinity
Parameter 20~IW(1.000,7)	infinity	infinity	infinity
Parameter 21~IW(0.000,7)	infinity	infinity	infinity
Parameter 22~IW(0.000,7)	infinity	infinity	infinity
Parameter 23~IW(0.000,7)	infinity	infinity	infinity
Parameter 24~IW(0.000,7)	infinity	infinity	infinity

Parameter 25~IW(1.000,7)	infinity	infinity	infinity
Parameter 26~IW(0.000,7)	infinity	infinity	infinity
Parameter 27~IW(0.000,7)	infinity	infinity	infinity
Parameter 28~IW(0.000,7)	infinity	infinity	infinity
Parameter 29~IW(0.000,7)	infinity	infinity	infinity
Parameter 30~IW(0.000,7)	infinity	infinity	infinity
Parameter 31~IW(1.000,7)	infinity	infinity	infinity
Parameter 32~N(0.000,infinity)	0.0000	infinity	infinity
Parameter 33~N(0.000,infinity)	0.0000	infinity	infinity
Parameter 34~N(0.000,infinity)	0.0000	infinity	infinity
Parameter 35~N(0.000,infinity)	0.0000	infinity	infinity
Parameter 36~N(0.000,5.000)	0.0000	5.0000	2.2361
Parameter 37~N(0.000,5.000)	0.0000	5.0000	2.2361
Parameter 38~IG(-1.000,0.000)	infinity	infinity	infinity
Parameter 39~IG(-1.000,0.000)	infinity	infinity	infinity
Parameter 40~IW(1.000,3)	infinity	infinity	infinity
Parameter 41~IW(0.000,3)	infinity	infinity	infinity
Parameter 42~IW(1.000,3)	infinity	infinity	infinity
Parameter 43~N(0.000,5.000)	0.0000	5.0000	2.2361

TECHNICAL 3 OUTPUT

ESTIMATED COVARIANCE MATRIX FOR PARAMETER ESTIMATES

	1	2	3	4	5
1	0.455095D-04				
2	0.101841D-04	0.594211D-04			
3	-0.760538D-05	-0.103382D-04	0.220478D-03		
4	0.230080D-05	0.813749D-05	-0.807290D-05	0.456017D-04	
5	-0.553630D-05	-0.173984D-04	0.273358D-04	-0.148092D-04	0.162051D-03
6	0.140310D-04	0.602726D-06	0.676273D-04	0.154194D-05	-0.250708D-04
7	-0.151593D-04	0.487989D-05	0.680633D-06	-0.440649D-05	0.344835D-05
8	-0.501274D-06	-0.719942D-05	0.831321D-05	-0.112343D-05	-0.877262D-05
9	0.107750D-04	-0.649422D-05	-0.301063D-04	-0.233610D-05	0.552976D-05
10	0.338512D-05	-0.789700D-06	-0.741604D-06	-0.633457D-05	0.130308D-04
11	0.570804D-06	-0.797351D-07	0.652947D-06	-0.714332D-06	0.661136D-06
12	-0.108869D-05	-0.873420D-06	-0.933515D-07	-0.430554D-06	-0.678779D-06
13	0.863792D-06	0.129612D-05	-0.188597D-05	0.544664D-06	-0.234407D-05
14	0.253895D-06	-0.146592D-06	-0.457787D-05	0.117353D-05	0.108379D-05
15	0.105144D-05	-0.186688D-05	-0.728553D-06	-0.266940D-05	0.882043D-06
16	-0.680088D-05	0.213024D-05	0.844417D-05	0.276550D-05	0.659269D-05
17	0.367485D-07	0.469364D-06	-0.271461D-06	-0.100576D-05	0.113143D-05

18	0.128956D-06	0.108067D-05	0.261696D-06	0.595437D-06	-0.713279D-06
19	-0.170978D-06	-0.112083D-05	0.426082D-05	-0.131950D-05	0.184680D-05
20	0.849572D-06	0.219820D-05	0.146142D-05	-0.416728D-06	-0.126227D-05
21	-0.507358D-06	0.132683D-05	0.173162D-05	-0.799014D-06	0.139215D-05
22	-0.966008D-06	-0.685695D-06	0.306287D-05	0.476574D-06	-0.145993D-06
23	-0.296395D-05	-0.271031D-05	0.671813D-05	-0.458724D-05	-0.768668D-05
24	0.117724D-05	0.106668D-05	-0.110677D-05	0.136866D-06	0.247133D-05
25	-0.349673D-06	-0.297861D-06	-0.274141D-05	0.426498D-05	0.220457D-05
26	0.240695D-05	-0.473721D-06	-0.469341D-05	0.127453D-05	0.696083D-06
27	0.200108D-05	-0.139935D-06	0.136552D-05	-0.167299D-05	0.279261D-05
28	-0.334726D-05	0.521690D-05	0.918439D-06	0.626981D-05	-0.303754D-05
29	0.990421D-06	-0.403472D-06	0.129278D-05	-0.228070D-05	0.260313D-05
30	0.114839D-05	0.215193D-05	-0.329754D-05	-0.370203D-06	-0.644119D-05
31	-0.455721D-05	0.102153D-05	-0.136163D-05	0.636668D-05	-0.140404D-04
32	-0.472997D-05	0.545055D-05	-0.117160D-04	0.111157D-05	0.443568D-05
33	-0.269314D-04	-0.428219D-04	0.103448D-03	-0.203470D-04	0.395749D-04
34	-0.469442D-05	0.158457D-04	-0.231181D-04	0.221367D-04	-0.340679D-04
35	0.530888D-05	-0.168257D-05	0.168122D-04	0.260920D-05	0.146774D-05
36	-0.447043D-05	0.734879D-05	0.220219D-04	-0.443992D-05	0.184831D-04
37	0.106690D-05	-0.250127D-04	0.911720D-06	0.328397D-04	0.330037D-04
38	-0.203760D-05	0.396980D-06	0.192781D-05	-0.326052D-06	0.473778D-06
39	0.143061D-06	-0.369087D-05	0.114837D-05	-0.212065D-05	-0.224276D-05
40	-0.360944D-04	-0.521236D-04	0.112400D-03	0.509314D-04	-0.326711D-04
41	-0.152549D-05	-0.565084D-05	-0.409406D-05	0.164971D-04	-0.879058D-05
42	-0.360684D-05	-0.472993D-05	-0.315958D-05	-0.688258D-05	-0.866270D-05
43	0.619266D-05	-0.631095D-04	0.278163D-05	0.780143D-04	0.765098D-04

ESTIMATED COVARIANCE MATRIX FOR PARAMETER ESTIMATES

	6	7	8	9	10
6	0.487879D-02				
7	-0.292927D-03	0.198095D-02			
8	0.104512D-04	0.972718D-04	0.614314D-03		
9	0.122161D-03	-0.327166D-03	0.107537D-03	0.234472D-02	
10	0.674757D-04	0.151026D-03	-0.103262D-03	0.114454D-03	0.712163D-03
11	0.446508D-05	0.103485D-04	-0.378324D-05	-0.684250D-05	0.288187D-05
12	-0.834923D-05	0.524612D-05	-0.762861D-06	-0.309073D-05	-0.212350D-05
13	-0.110593D-04	0.462747D-05	-0.515933D-05	-0.107482D-04	-0.135370D-05
14	0.315766D-05	-0.202721D-05	0.111877D-04	0.399453D-05	-0.565738D-05
15	0.306696D-04	-0.728881D-05	-0.120854D-05	0.124590D-04	0.272718D-05
16	0.252822D-05	0.437095D-04	0.130765D-04	0.756389D-05	0.499301D-05
17	-0.348952D-05	0.925993D-07	-0.974282D-06	-0.141730D-06	-0.108992D-05
18	-0.418565D-05	0.587121D-05	-0.132404D-05	-0.176406D-05	-0.117766D-05

19	0.229313D-04	-0.106492D-04	-0.837618D-06	0.752239D-05	0.684205D-06
20	-0.973491D-05	0.637081D-05	-0.207200D-05	-0.693150D-06	0.225939D-05
21	-0.192340D-04	0.195227D-06	-0.424818D-06	0.896592D-05	-0.418443D-05
22	-0.127342D-04	0.901976D-05	0.881013D-06	0.414283D-05	0.175532D-05
23	0.419069D-04	-0.209814D-04	-0.465736D-05	0.259100D-04	0.640126D-06
24	-0.470848D-05	0.100321D-04	-0.665629D-05	0.384858D-05	0.107290D-04
25	-0.461547D-04	0.259968D-04	-0.507863D-05	0.315423D-04	0.195363D-04
26	0.146095D-04	0.748006D-06	0.661048D-05	0.309791D-05	0.397480D-05
27	0.162306D-04	-0.619355D-05	-0.362853D-05	-0.248159D-07	0.991451D-06
28	-0.266213D-04	0.826446D-05	0.168234D-04	0.481655D-05	-0.788767D-05
29	0.864325D-05	-0.161931D-04	0.132408D-05	0.448510D-05	0.364160D-05
30	0.332408D-04	-0.115614D-04	0.104801D-04	-0.759644D-05	0.217867D-06
31	0.237368D-05	-0.154963D-05	-0.128275D-04	-0.732580D-05	0.436818D-05
32	-0.142885D-03	-0.807494D-04	-0.333603D-04	-0.255388D-03	0.124881D-03
33	0.360453D-04	0.168094D-03	0.353425D-04	-0.108930D-03	-0.177935D-04
34	-0.967655D-06	0.828604D-04	0.118163D-04	-0.754571D-04	0.292157D-04
35	-0.108590D-03	-0.318377D-04	-0.249288D-04	0.347264D-06	0.263255D-05
36	-0.694836D-04	0.590980D-04	-0.131745D-04	-0.121524D-03	0.245909D-04
37	-0.366685D-03	0.710742D-04	0.499919D-04	0.922190D-05	0.784916D-04
38	0.131160D-04	-0.478586D-05	-0.144046D-05	-0.142185D-04	-0.264380D-05
39	0.286907D-04	0.309614D-04	0.540006D-05	0.149363D-04	0.840253D-07
40	-0.362933D-04	0.497556D-04	0.965644D-04	0.125777D-04	-0.379837D-04
41	0.758480D-05	-0.433662D-04	0.534485D-04	0.215773D-04	0.173064D-04
42	0.266709D-04	-0.351846D-04	0.144190D-04	-0.323773D-05	-0.678261D-05
43	0.838527D-04	0.261445D-03	0.592216D-04	0.391820D-04	0.215163D-03

ESTIMATED COVARIANCE MATRIX FOR PARAMETER ESTIMATES

	11	12	13	14	15
11	0.209618D-04				
12	0.454209D-05	0.135658D-04			
13	0.564636D-06	0.642395D-05	0.343119D-04		
14	-0.381829D-05	-0.394174D-05	-0.378614D-05	0.514711D-04	
15	0.232381D-06	-0.342127D-05	-0.134729D-04	0.117186D-04	0.665279D-04
16	0.285540D-05	0.151980D-05	0.653046D-05	-0.142662D-04	-0.360198D-04
17	0.678522D-06	0.269331D-05	0.103347D-05	-0.272544D-05	-0.395350D-06
18	-0.157018D-06	0.150621D-05	0.740224D-05	-0.212583D-05	-0.539779D-05
19	-0.114913D-05	-0.140199D-05	-0.261784D-05	0.384607D-05	0.114533D-04
20	0.822799D-06	0.376109D-06	0.178290D-05	0.260949D-06	-0.660908D-06
21	0.295967D-05	0.521721D-05	0.327585D-05	-0.428876D-05	-0.218771D-05
22	0.272104D-05	0.452193D-05	0.123393D-04	-0.385862D-05	-0.912471D-05
23	-0.169626D-05	-0.361560D-05	-0.743917D-05	0.913338D-05	0.207637D-04
24	0.598549D-06	0.168975D-05	0.370073D-05	-0.195379D-05	-0.298122D-05

25	0.374319D-05	0.281730D-05	0.862781D-05	-0.870541D-05	-0.758569D-05
26	-0.424559D-05	-0.609065D-05	-0.270691D-05	0.896711D-05	0.154326D-05
27	-0.184316D-05	-0.482993D-05	-0.150554D-04	0.508081D-05	0.138770D-04
28	0.159913D-05	0.241457D-05	0.604080D-05	-0.849397D-05	-0.258429D-04
29	-0.175697D-05	-0.216925D-05	-0.352035D-05	0.274930D-05	0.402512D-05
30	-0.224047D-05	-0.198338D-05	-0.737935D-05	0.257326D-05	0.542173D-05
31	0.314497D-05	0.316124D-05	0.872316D-05	-0.218564D-05	-0.109811D-04
32	0.192621D-05	0.479526D-05	0.973288D-05	-0.911140D-05	-0.165052D-05
33	0.181496D-04	0.208113D-04	0.172065D-05	0.184183D-04	0.561846D-04
34	-0.108903D-05	0.861104D-06	0.102199D-04	-0.773776D-05	-0.106659D-04
35	-0.755601D-05	-0.526536D-05	0.914878D-05	0.491460D-05	-0.265721D-04
36	-0.676488D-07	0.422412D-05	-0.345444D-06	-0.168790D-06	-0.897841D-05
37	0.470542D-05	0.539137D-06	-0.168963D-04	0.883863D-05	0.324804D-04
38	0.765596D-06	0.226885D-06	0.161706D-06	0.691408D-06	-0.621751D-06
39	0.294136D-05	0.785999D-06	0.670583D-06	-0.233560D-05	0.267251D-05
40	0.258790D-04	-0.104897D-05	0.197557D-04	0.419702D-07	-0.435221D-04
41	-0.537300D-05	-0.443031D-05	-0.503047D-05	-0.545278D-05	0.419072D-05
42	-0.162339D-05	-0.320128D-06	-0.135328D-05	-0.274913D-05	-0.171940D-05
43	0.190915D-04	-0.226909D-05	-0.450201D-04	0.189536D-04	0.994175D-04

ESTIMATED COVARIANCE MATRIX FOR PARAMETER ESTIMATES

	16	17	18	19	20
16	0.419405D-03				
17	-0.565310D-06	0.104499D-04			
18	0.579831D-05	0.224605D-05	0.148575D-04		
19	-0.275646D-04	-0.899787D-06	-0.459673D-05	0.515531D-04	
20	0.908861D-06	0.563375D-06	0.482646D-05	-0.509711D-05	0.222890D-04
21	0.155656D-05	0.485309D-05	0.231260D-05	-0.243696D-05	0.145145D-05
22	0.132249D-04	0.170923D-05	0.762515D-05	-0.294248D-05	0.168547D-05
23	-0.368939D-04	-0.473503D-05	-0.190872D-05	0.246555D-04	-0.239312D-06
24	0.304004D-05	0.154366D-05	0.588678D-05	-0.569025D-05	0.811124D-05
25	0.781961D-05	0.193611D-05	0.496700D-05	-0.326060D-05	0.439760D-05
26	-0.433914D-05	-0.435471D-05	-0.118365D-05	0.158402D-05	0.120411D-06
27	-0.108869D-04	-0.965050D-06	-0.626402D-05	0.458802D-05	-0.235941D-05
28	0.622795D-04	0.181139D-05	0.404201D-05	-0.192280D-04	-0.119222D-05
29	-0.331262D-05	-0.192510D-05	-0.597322D-05	0.102384D-04	-0.732597D-05
30	-0.405431D-05	-0.548173D-06	-0.538849D-05	0.995892D-05	-0.436840D-05
31	0.132460D-04	0.243898D-06	0.349986D-05	-0.924715D-05	0.209635D-05
32	-0.353292D-05	0.280535D-05	0.689715D-05	0.406430D-05	0.304605D-05
33	0.770628D-04	-0.146391D-05	-0.240372D-04	0.672187D-04	-0.872678D-05
34	0.159965D-04	0.349503D-05	0.602812D-05	0.149131D-05	-0.251359D-05
35	0.238139D-04	0.702965D-06	0.345504D-05	0.153625D-05	-0.315815D-05

36	-0.805312D-05	-0.151537D-05	-0.213828D-05	-0.878964D-06	0.216167D-05
37	-0.607594D-05	0.727671D-05	0.387569D-05	-0.232833D-04	0.406185D-04
38	0.388521D-05	0.390937D-06	0.761789D-06	-0.161055D-05	0.123630D-05
39	0.802263D-05	0.961112D-07	0.130234D-05	0.115738D-06	-0.283904D-06
40	-0.265607D-04	0.143671D-04	0.928505D-05	0.401492D-04	-0.181822D-04
41	0.355204D-05	-0.185278D-06	0.392029D-05	0.790877D-05	0.759246D-05
42	0.428121D-05	0.511290D-06	-0.111343D-05	-0.519711D-06	-0.305350D-05
43	-0.325308D-04	0.161446D-04	0.985512D-05	-0.464255D-04	0.880601D-04

ESTIMATED COVARIANCE MATRIX FOR PARAMETER ESTIMATES

	21	22	23	24	25
21	0.445590D-04				
22	0.110361D-04	0.545198D-04			
23	-0.103408D-04	-0.180627D-04	0.210846D-03		
24	0.433670D-05	0.124068D-04	-0.140209D-04	0.462031D-04	
25	0.205545D-04	0.450320D-04	-0.252451D-04	0.370278D-04	0.385468D-03
26	-0.828465D-05	-0.325164D-05	0.383504D-05	-0.630142D-07	-0.426650D-05
27	-0.249062D-05	-0.135748D-04	0.799013D-05	-0.445574D-05	-0.120187D-04
28	0.599480D-05	0.709151D-05	-0.379032D-04	0.268194D-05	0.395680D-05
29	-0.309964D-05	-0.631787D-05	0.189483D-05	-0.867354D-05	-0.955381D-05
30	-0.101154D-04	-0.249525D-04	0.389772D-04	-0.208432D-04	-0.751197D-04
31	0.369121D-05	0.872237D-05	-0.914402D-05	0.494887D-05	0.182479D-04
32	0.198108D-05	-0.170344D-05	0.231955D-05	0.468910D-05	-0.146503D-04
33	0.444514D-05	-0.197704D-05	0.162005D-04	-0.146684D-04	-0.103628D-03
34	0.989099D-05	0.130304D-04	-0.805480D-05	0.531390D-06	0.389657D-04
35	0.137829D-05	0.537931D-05	-0.100772D-04	-0.656601D-05	-0.597430D-05
36	-0.776590D-05	0.280292D-05	0.739487D-05	-0.110749D-05	0.145171D-04
37	0.452507D-05	-0.225795D-04	0.136620D-04	0.243147D-05	0.542250D-04
38	-0.292307D-08	0.732130D-06	-0.191578D-05	0.607868D-06	-0.593174D-05
39	0.498554D-06	-0.189835D-06	0.902472D-07	-0.129474D-05	-0.738848D-05
40	0.153141D-04	0.404124D-04	-0.910400D-04	-0.213372D-04	0.691876D-04
41	-0.938824D-05	0.646341D-05	-0.590283D-05	-0.313062D-05	0.282388D-04
42	-0.396569D-05	-0.146565D-05	0.175085D-05	-0.283052D-05	-0.126847D-04
43	0.146618D-05	-0.625097D-04	0.552047D-04	-0.315669D-05	0.116275D-03

ESTIMATED COVARIANCE MATRIX FOR PARAMETER ESTIMATES

	26	27	28	29	30
26	0.399634D-04				
27	0.946214D-05	0.547761D-04			
28	-0.862315D-05	-0.245136D-04	0.184927D-03		

29	0.209229D-05	0.134858D-04	-0.116967D-04	0.411529D-04	
30	0.721614D-05	0.239043D-04	-0.224555D-04	0.230001D-04	0.184264D-03
31	-0.115770D-04	-0.427960D-04	0.680902D-04	-0.326398D-04	-0.721392D-04
32	0.799853D-06	-0.359748D-05	0.453986D-05	-0.105227D-04	-0.289322D-05
33	-0.199065D-04	0.222206D-04	-0.643676D-04	0.251336D-04	0.385739D-04
34	0.507311D-05	-0.472711D-05	0.171487D-04	-0.408024D-05	-0.314103D-04
35	0.107829D-04	0.190070D-05	-0.106739D-05	-0.197804D-06	-0.258619D-05
36	-0.333331D-05	0.347099D-05	0.552863D-05	0.637499D-05	0.821294D-05
37	-0.622476D-04	-0.189081D-04	-0.281113D-04	0.268479D-04	0.271868D-04
38	0.544437D-06	-0.149166D-05	0.133767D-05	-0.170578D-05	0.261433D-05
39	0.752798D-06	0.295834D-06	0.324006D-06	0.101925D-05	0.424992D-05
40	-0.144855D-04	-0.722333D-04	0.227880D-04	0.549067D-04	0.772471D-04
41	0.398180D-05	0.286724D-05	-0.219495D-04	-0.633752D-05	-0.290834D-05
42	-0.650891D-06	-0.243681D-05	-0.177178D-05	0.320725D-05	0.124435D-04
43	-0.152350D-03	-0.327825D-04	-0.952660D-04	0.666549D-04	0.740380D-04

ESTIMATED COVARIANCE MATRIX FOR PARAMETER ESTIMATES

	31	32	33	34	35
31	0.314756D-03				
32	0.176412D-04	0.100757D-02			
33	-0.298678D-04	0.126400D-03	0.241260D-01		
34	0.153598D-04	-0.424748D-06	0.333551D-03	0.166959D-02	
35	0.580049D-05	0.671996D-04	0.371530D-04	0.775647D-05	0.865719D-03
36	-0.693897D-05	0.274238D-04	0.990872D-04	-0.395727D-04	0.392998D-04
37	0.334732D-04	-0.259892D-03	-0.893845D-03	-0.304086D-03	-0.912265D-04
38	0.419673D-05	0.526575D-05	0.279568D-04	-0.182213D-07	0.346676D-05
39	0.138189D-05	0.254995D-05	0.207592D-04	0.645541D-05	-0.131101D-04
40	0.107742D-05	0.105261D-03	-0.366362D-03	0.540125D-03	0.345330D-03
41	0.704833D-06	0.855538D-05	-0.192912D-03	0.134441D-04	0.759179D-04
42	-0.771012D-05	0.128199D-04	0.890881D-04	-0.272953D-04	-0.104006D-04
43	0.106921D-03	-0.654484D-03	-0.198029D-02	-0.779776D-03	-0.284871D-03

ESTIMATED COVARIANCE MATRIX FOR PARAMETER ESTIMATES

	36	37	38	39	40
36	0.936798D-03				
37	-0.394121D-03	0.255943D-01			
38	-0.350530D-05	0.899877D-05	0.304283D-04		
39	-0.554938D-04	-0.122440D-03	-0.397494D-05	0.132509D-03	
40	0.170170D-03	-0.355950D-05	-0.583918D-04	-0.355281D-04	0.681216D-01
41	0.167568D-04	0.976202D-04	-0.105781D-06	0.299140D-05	-0.289209D-03

42	0.110344D-04	-0.367166D-04	0.967923D-06	-0.531449D-05	0.261840D-03
43	-0.111117D-02	0.619659D-01	0.368271D-04	-0.205098D-03	-0.891720D-04

ESTIMATED COVARIANCE MATRIX FOR PARAMETER ESTIMATES

	41	42	43
41	0.245219D-02		
42	-0.552284D-04	0.325794D-03	
43	0.219818D-03	-0.110058D-03	0.151411D+00

ESTIMATED CORRELATION MATRIX FOR PARAMETER ESTIMATES

	1	2	3	4	5
1	1.000				
2	0.196	1.000			
3	-0.076	-0.090	1.000		
4	0.051	0.156	-0.081	1.000	
5	-0.064	-0.177	0.145	-0.172	1.000
6	0.030	0.001	0.065	0.003	-0.028
7	-0.050	0.014	0.001	-0.015	0.006
8	-0.003	-0.038	0.023	-0.007	-0.028
9	0.033	-0.017	-0.042	-0.007	0.009
10	0.019	-0.004	-0.002	-0.035	0.038
11	0.018	-0.002	0.010	-0.023	0.011
12	-0.044	-0.031	-0.002	-0.017	-0.014
13	0.022	0.029	-0.022	0.014	-0.031
14	0.005	-0.003	-0.043	0.024	0.012
15	0.019	-0.030	-0.006	-0.048	0.008
16	-0.049	0.013	0.028	0.020	0.025
17	0.002	0.019	-0.006	-0.046	0.027
18	0.005	0.036	0.005	0.023	-0.015
19	-0.004	-0.020	0.040	-0.027	0.020
20	0.027	0.060	0.021	-0.013	-0.021
21	-0.011	0.026	0.017	-0.018	0.016
22	-0.019	-0.012	0.028	0.010	-0.002
23	-0.030	-0.024	0.031	-0.047	-0.042
24	0.026	0.020	-0.011	0.003	0.029
25	-0.003	-0.002	-0.009	0.032	0.009
26	0.056	-0.010	-0.050	0.030	0.009
27	0.040	-0.002	0.012	-0.033	0.030
28	-0.036	0.050	0.005	0.068	-0.018
29	0.023	-0.008	0.014	-0.053	0.032

30	0.013	0.021	-0.016	-0.004	-0.037
31	-0.038	0.007	-0.005	0.053	-0.062
32	-0.022	0.022	-0.025	0.005	0.011
33	-0.026	-0.036	0.045	-0.019	0.020
34	-0.017	0.050	-0.038	0.080	-0.065
35	0.027	-0.007	0.038	0.013	0.004
36	-0.022	0.031	0.048	-0.021	0.047
37	0.001	-0.020	0.000	0.030	0.016
38	-0.055	0.009	0.024	-0.009	0.007
39	0.002	-0.042	0.007	-0.027	-0.015
40	-0.020	-0.026	0.029	0.029	-0.010
41	-0.005	-0.015	-0.006	0.049	-0.014
42	-0.030	-0.034	-0.012	-0.056	-0.038
43	0.002	-0.021	0.000	0.030	0.015

ESTIMATED CORRELATION MATRIX FOR PARAMETER ESTIMATES

	6	7	8	9	10
6	1.000				
7	-0.094	1.000			
8	0.006	0.088	1.000		
9	0.036	-0.152	0.090	1.000	
10	0.036	0.127	-0.156	0.089	1.000
11	0.014	0.051	-0.033	-0.031	0.024
12	-0.032	0.032	-0.008	-0.017	-0.022
13	-0.027	0.018	-0.036	-0.038	-0.009
14	0.006	-0.006	0.063	0.011	-0.030
15	0.054	-0.020	-0.006	0.032	0.013
16	0.002	0.048	0.026	0.008	0.009
17	-0.015	0.001	-0.012	-0.001	-0.013
18	-0.016	0.034	-0.014	-0.009	-0.011
19	0.046	-0.033	-0.005	0.022	0.004
20	-0.030	0.030	-0.018	-0.003	0.018
21	-0.041	0.001	-0.003	0.028	-0.023
22	-0.025	0.027	0.005	0.012	0.009
23	0.041	-0.032	-0.013	0.037	0.002
24	-0.010	0.033	-0.040	0.012	0.059
25	-0.034	0.030	-0.010	0.033	0.037
26	0.033	0.003	0.042	0.010	0.024
27	0.031	-0.019	-0.020	0.000	0.005
28	-0.028	0.014	0.050	0.007	-0.022
29	0.019	-0.057	0.008	0.014	0.021
30	0.035	-0.019	0.031	-0.012	0.001

31	0.002	-0.002	-0.029	-0.009	0.009
32	-0.064	-0.057	-0.042	-0.166	0.147
33	0.003	0.024	0.009	-0.014	-0.004
34	0.000	0.046	0.012	-0.038	0.027
35	-0.053	-0.024	-0.034	0.000	0.003
36	-0.033	0.043	-0.017	-0.082	0.030
37	-0.033	0.010	0.013	0.001	0.018
38	0.034	-0.019	-0.011	-0.053	-0.018
39	0.036	0.060	0.019	0.027	0.000
40	-0.002	0.004	0.015	0.001	-0.005
41	0.002	-0.020	0.044	0.009	0.013
42	0.021	-0.044	0.032	-0.004	-0.014
43	0.003	0.015	0.006	0.002	0.021

ESTIMATED CORRELATION MATRIX FOR PARAMETER ESTIMATES

	11	12	13	14	15
11	1.000				
12	0.269	1.000			
13	0.021	0.298	1.000		
14	-0.116	-0.149	-0.090	1.000	
15	0.006	-0.114	-0.282	0.200	1.000
16	0.030	0.020	0.054	-0.097	-0.216
17	0.046	0.226	0.055	-0.118	-0.015
18	-0.009	0.106	0.328	-0.077	-0.172
19	-0.035	-0.053	-0.062	0.075	0.196
20	0.038	0.022	0.064	0.008	-0.017
21	0.097	0.212	0.084	-0.090	-0.040
22	0.080	0.166	0.285	-0.073	-0.152
23	-0.026	-0.068	-0.087	0.088	0.175
24	0.019	0.067	0.093	-0.040	-0.054
25	0.042	0.039	0.075	-0.062	-0.047
26	-0.147	-0.262	-0.073	0.198	0.030
27	-0.054	-0.177	-0.347	0.096	0.230
28	0.026	0.048	0.076	-0.087	-0.233
29	-0.060	-0.092	-0.094	0.060	0.077
30	-0.036	-0.040	-0.093	0.026	0.049
31	0.039	0.048	0.084	-0.017	-0.076
32	0.013	0.041	0.052	-0.040	-0.006
33	0.026	0.036	0.002	0.017	0.044
34	-0.006	0.006	0.043	-0.026	-0.032
35	-0.056	-0.049	0.053	0.023	-0.111
36	0.000	0.037	-0.002	-0.001	-0.036

37	0.006	0.001	-0.018	0.008	0.025
38	0.030	0.011	0.005	0.017	-0.014
39	0.056	0.019	0.010	-0.028	0.028
40	0.022	-0.001	0.013	0.000	-0.020
41	-0.024	-0.024	-0.017	-0.015	0.010
42	-0.020	-0.005	-0.013	-0.021	-0.012
43	0.011	-0.002	-0.020	0.007	0.031

ESTIMATED CORRELATION MATRIX FOR PARAMETER ESTIMATES

	16	17	18	19	20
16	1.000				
17	-0.009	1.000			
18	0.073	0.180	1.000		
19	-0.187	-0.039	-0.166	1.000	
20	0.009	0.037	0.265	-0.150	1.000
21	0.011	0.225	0.090	-0.051	0.046
22	0.087	0.072	0.268	-0.056	0.048
23	-0.124	-0.101	-0.034	0.236	-0.003
24	0.022	0.070	0.225	-0.117	0.253
25	0.019	0.031	0.066	-0.023	0.047
26	-0.034	-0.213	-0.049	0.035	0.004
27	-0.072	-0.040	-0.220	0.086	-0.068
28	0.224	0.041	0.077	-0.197	-0.019
29	-0.025	-0.093	-0.242	0.222	-0.242
30	-0.015	-0.012	-0.103	0.102	-0.068
31	0.036	0.004	0.051	-0.073	0.025
32	-0.005	0.027	0.056	0.018	0.020
33	0.024	-0.003	-0.040	0.060	-0.012
34	0.019	0.026	0.038	0.005	-0.013
35	0.040	0.007	0.030	0.007	-0.023
36	-0.013	-0.015	-0.018	-0.004	0.015
37	-0.002	0.014	0.006	-0.020	0.054
38	0.034	0.022	0.036	-0.041	0.047
39	0.034	0.003	0.029	0.001	-0.005
40	-0.005	0.017	0.009	0.021	-0.015
41	0.004	-0.001	0.021	0.022	0.032
42	0.012	0.009	-0.016	-0.004	-0.036
43	-0.004	0.013	0.007	-0.017	0.048

ESTIMATED CORRELATION MATRIX FOR PARAMETER ESTIMATES

	21	22	23	24	25

21	1.000				
22	0.224	1.000			
23	-0.107	-0.168	1.000		
24	0.096	0.247	-0.142	1.000	
25	0.157	0.311	-0.089	0.277	1.000
26	-0.196	-0.070	0.042	-0.001	-0.034
27	-0.050	-0.248	0.074	-0.089	-0.083
28	0.066	0.071	-0.192	0.029	0.015
29	-0.072	-0.133	0.020	-0.199	-0.076
30	-0.112	-0.249	0.198	-0.226	-0.282
31	0.031	0.067	-0.035	0.041	0.052
32	0.009	-0.007	0.005	0.022	-0.024
33	0.004	-0.002	0.007	-0.014	-0.034
34	0.036	0.043	-0.014	0.002	0.049
35	0.007	0.025	-0.024	-0.033	-0.010
36	-0.038	0.012	0.017	-0.005	0.024
37	0.004	-0.019	0.006	0.002	0.017
38	0.000	0.018	-0.024	0.016	-0.055
39	0.006	-0.002	0.001	-0.017	-0.033
40	0.009	0.021	-0.024	-0.012	0.014
41	-0.028	0.018	-0.008	-0.009	0.029
42	-0.033	-0.011	0.007	-0.023	-0.036
43	0.001	-0.022	0.010	-0.001	0.015

ESTIMATED CORRELATION MATRIX FOR PARAMETER ESTIMATES

	26	27	28	29	30
26	1.000				
27	0.202	1.000			
28	-0.100	-0.244	1.000		
29	0.052	0.284	-0.134	1.000	
30	0.084	0.238	-0.122	0.264	1.000
31	-0.103	-0.326	0.282	-0.287	-0.300
32	0.004	-0.015	0.011	-0.052	-0.007
33	-0.020	0.019	-0.030	0.025	0.018
34	0.020	-0.016	0.031	-0.016	-0.057
35	0.058	0.009	-0.003	-0.001	-0.006
36	-0.017	0.015	0.013	0.032	0.020
37	-0.062	-0.016	-0.013	0.026	0.013
38	0.016	-0.037	0.018	-0.048	0.035
39	0.010	0.003	0.002	0.014	0.027
40	-0.009	-0.037	0.006	0.033	0.022

41	0.013	0.008	-0.033	-0.020	-0.004
42	-0.006	-0.018	-0.007	0.028	0.051
43	-0.062	-0.011	-0.018	0.027	0.014

ESTIMATED CORRELATION MATRIX FOR PARAMETER ESTIMATES

	31	32	33	34	35
31	1.000				
32	0.031	1.000			
33	-0.011	0.026	1.000		
34	0.021	0.000	0.053	1.000	
35	0.011	0.072	0.008	0.006	1.000
36	-0.013	0.028	0.021	-0.032	0.044
37	0.012	-0.051	-0.036	-0.047	-0.019
38	0.043	0.030	0.033	0.000	0.021
39	0.007	0.007	0.012	0.014	-0.039
40	0.000	0.013	-0.009	0.051	0.045
41	0.001	0.005	-0.025	0.007	0.052
42	-0.024	0.022	0.032	-0.037	-0.020
43	0.015	-0.053	-0.033	-0.049	-0.025

ESTIMATED CORRELATION MATRIX FOR PARAMETER ESTIMATES

	36	37	38	39	40
36	1.000				
37	-0.080	1.000			
38	-0.021	0.010	1.000		
39	-0.158	-0.066	-0.063	1.000	
40	0.021	0.000	-0.041	-0.012	1.000
41	0.011	0.012	0.000	0.005	-0.022
42	0.020	-0.013	0.010	-0.026	0.056
43	-0.093	0.995	0.017	-0.046	-0.001

ESTIMATED CORRELATION MATRIX FOR PARAMETER ESTIMATES

	41	42	43
41	1.000		
42	-0.062	1.000	
43	0.011	-0.016	1.000

TECHNICAL 8 OUTPUT

TECHNICAL 8 OUTPUT FOR BAYES ESTIMATION

CHAIN	BSEED
1	0
2	285380
3	253358

ITERATION	POTENTIAL SCALE REDUCTION	PARAMETER WITH HIGHEST PSR
100	1.010	43
200	1.010	7
300	1.010	35
400	1.004	35
500	1.003	39
600	1.007	41
700	1.002	41
800	1.001	31
900	1.002	41
1000	1.002	23

Deterioration in physical health, role clarity interaction

SUMMARY OF DATA

Number of clusters 58

Size (s)	Cluster ID with Size s
7	22
16	166
23	80 71 205
25	74
27	192
31	65
32	106
35	228
37	170 72
39	50 75 181
40	182
42	165
44	114
45	173
46	107
47	169 30
48	226 81
49	36
50	116 108 40
56	162 84
57	61
59	206 93 83
60	92
61	69
63	6
64	152
65	145
66	4
68	68
69	154
72	174
78	98
84	178 2
95	156 202
96	144
108	142

131	122
172	24
175	209
224	204
269	125
364	217
738	110
860	100

COVARIANCE COVERAGE OF DATA

Minimum covariance coverage value 0.100

Number of missing data patterns 8

PROPORTION OF DATA PRESENT

Covariance Coverage

	PHYCAT16	PRAFAC	PHY15	KES15	CHOICE15
PHYCAT16	1.000				
PRAFAC	1.000	1.000			
PHY15	0.947	0.947	0.947		
KES15	0.922	0.922	0.922	0.922	
CHOICE15	0.915	0.915	0.915	0.915	0.915
BULLY15	0.915	0.915	0.915	0.915	0.915
PRESS15	0.915	0.915	0.915	0.915	0.915
DUTIES15	0.915	0.915	0.915	0.915	0.915

Covariance Coverage

	BULLY15	PRESS15	DUTIES15
BULLY15	0.915		
PRESS15	0.914	0.915	
DUTIES15	0.915	0.915	0.915

UNIVARIATE PROPORTIONS AND COUNTS FOR CATEGORICAL VARIABLES

```
    PHYCAT16
      Category 1    0.881         4884.000
      Category 2    0.119          662.000


UNIVARIATE SAMPLE STATISTICS


     UNIVARIATE HIGHER-ORDER MOMENT DESCRIPTIVE STATISTICS

         Variable/         Mean/     Skewness/   Minimum/ % with                Percentiles
        Sample Size      Variance    Kurtosis    Maximum  Min/Max      20%/60%    40%/80%    Median

     PRAFAC               -0.185       0.245      -2.338    1.72%      -1.448     -0.527     -0.318
              58.000       1.345      -0.636       2.338    1.72%       0.080      0.752
     PHY15                 0.000      -0.546      -0.632   36.83%      -0.632      0.368      0.368
            5254.000       0.233      -1.702       0.368   63.17%       0.368      0.368
     KES15                 0.000       1.717      -0.511   21.65%      -0.511     -0.178     -0.178
            5114.000       0.289       3.835       3.489    0.04%      -0.011      0.322
     CHOICE15              0.000      -0.402      -2.225    7.41%      -1.225     -0.225     -0.225
            5077.000       1.081      -0.336       1.775    8.49%       0.775      0.775
     BULLY15               0.000       3.272      -0.170   87.25%      -0.170     -0.170     -0.170
            5073.000       0.239      11.772       3.830    0.08%      -0.170     -0.170
     PRESS15               0.000       0.252      -1.462   19.10%      -0.462     -0.462     -0.462
            5074.000       1.001      -0.371       2.538    2.76%       0.538      0.538
     DUTIES15              4.120      -1.246       1.000    2.40%       4.000      4.000      4.000
            5077.000       0.861       1.673       5.000   38.74%       4.000      5.000


THE MODEL ESTIMATION TERMINATED NORMALLY

     USE THE FBITERATIONS OPTION TO INCREASE THE NUMBER OF ITERATIONS BY A FACTOR
     OF AT LEAST TWO TO CHECK CONVERGENCE AND THAT THE PSR VALUE DOES NOT INCREASE.


MODEL FIT INFORMATION

Number of Free Parameters                              43


MODEL RESULTS
```

	Estimate	Posterior S.D.	One-Tailed P-Value	95% C.I. Lower 2.5%	Upper 2.5%	Significance
Within Level						
PHYCAT16 ON						
PHY15	0.978	0.070	0.000	0.837	1.119	*
CHOICE15	-0.069	0.025	0.001	-0.119	-0.024	*
PRESS15	-0.033	0.025	0.096	-0.084	0.016	
BULLY15	0.028	0.048	0.271	-0.062	0.121	
KES15	0.224	0.045	0.000	0.136	0.310	*
KES15 WITH						
PHY15	0.051	0.004	0.000	0.044	0.059	*
CHOICE15	-0.089	0.008	0.000	-0.105	-0.073	*
BULLY15	0.057	0.004	0.000	0.050	0.066	*
PRESS15	0.099	0.007	0.000	0.085	0.115	*
DUTIES15	-0.102	0.007	0.000	-0.116	-0.087	*
PHY15 WITH						
CHOICE15	-0.036	0.007	0.000	-0.050	-0.023	*
BULLY15	0.012	0.003	0.000	0.005	0.018	*
PRESS15	0.038	0.007	0.000	0.025	0.052	*
DUTIES15	-0.036	0.006	0.000	-0.048	-0.023	*
CHOICE15 WITH						
BULLY15	-0.058	0.007	0.000	-0.072	-0.044	*
PRESS15	-0.087	0.015	0.000	-0.118	-0.059	*
DUTIES15	0.173	0.014	0.000	0.145	0.200	*
BULLY15 WITH						
PRESS15	0.105	0.007	0.000	0.091	0.118	*
DUTIES15	-0.074	0.006	0.000	-0.087	-0.062	*
PRESS15 WITH						
DUTIES15	-0.179	0.014	0.000	-0.207	-0.152	*
Means						
PHY15	0.002	0.007	0.384	-0.011	0.016	
KES15	0.001	0.008	0.439	-0.014	0.017	
CHOICE15	-0.001	0.015	0.465	-0.030	0.029	
BULLY15	0.000	0.007	0.485	-0.012	0.014	
PRESS15	0.002	0.014	0.462	-0.025	0.028	

```
 Variances
    PHY15              0.233      0.005      0.000      0.225      0.242      *
    KES15              0.290      0.006      0.000      0.278      0.302      *
    CHOICE15           1.083      0.021      0.000      1.043      1.123      *
    BULLY15            0.239      0.005      0.000      0.230      0.249      *
    PRESS15            1.001      0.020      0.000      0.963      1.044      *
    DUTIES15           0.843      0.017      0.000      0.811      0.880      *

Between Level

 S1         ON
    PRAFAC            -0.007      0.032      0.412     -0.067      0.058

 PHYCAT16   ON
    PRAFAC            -0.082      0.030      0.005     -0.143     -0.022      *
    DUTIES15          -0.216      0.260      0.199     -0.716      0.296

 PRAFAC   WITH
    DUTIES15           0.029      0.038      0.209     -0.046      0.103

 Means
    PRAFAC            -0.182      0.155      0.122     -0.485      0.117
    DUTIES15           4.148      0.031      0.000      4.085      4.209      *

 Intercepts
    S1                -0.030      0.037      0.187     -0.102      0.044

 Thresholds
    PHYCAT16$1         0.458      1.074      0.321     -1.626      2.596

 Variances
    PRAFAC             1.348      0.260      0.000      0.955      1.989      *
    DUTIES15           0.041      0.009      0.000      0.027      0.064      *

 Residual Variances
    PHYCAT16           0.014      0.011      0.000      0.001      0.044      *
    S1                 0.008      0.010      0.000      0.001      0.037      *


STANDARDIZED MODEL RESULTS


STDYX Standardization
```

	Estimate	Posterior S.D.	One-Tailed P-Value	95% C.I. Lower 2.5%	Upper 2.5%	Significance
**Within-Level Standardized Estimates Averaged Over Clusters**						
S1 | PHYCAT16 ON						
DUTIES15	-0.024	0.026	0.179	-0.074	0.027	
PHYCAT16 ON						
PHY15	0.416	0.025	0.000	0.365	0.465	*
CHOICE15	-0.064	0.023	0.001	-0.109	-0.021	*
PRESS15	-0.030	0.023	0.103	-0.074	0.015	
BULLY15	0.013	0.021	0.266	-0.028	0.055	
KES15	0.106	0.021	0.000	0.065	0.147	*
KES15 WITH						
PHY15	0.198	0.013	0.000	0.171	0.223	*
CHOICE15	-0.159	0.014	0.000	-0.185	-0.131	*
BULLY15	0.219	0.014	0.000	0.191	0.245	*
PRESS15	0.185	0.013	0.000	0.160	0.212	*
DUTIES15	-0.207	0.014	0.000	-0.234	-0.179	*
PHY15 WITH						
CHOICE15	-0.072	0.014	0.000	-0.099	-0.045	*
BULLY15	0.050	0.014	0.000	0.022	0.076	*
PRESS15	0.080	0.014	0.000	0.053	0.108	*
DUTIES15	-0.080	0.014	0.000	-0.108	-0.052	*
CHOICE15 WITH						
BULLY15	-0.114	0.014	0.000	-0.141	-0.088	*
PRESS15	-0.083	0.014	0.000	-0.112	-0.056	*
DUTIES15	0.181	0.014	0.000	0.153	0.208	*
BULLY15 WITH						
PRESS15	0.214	0.013	0.000	0.188	0.240	*
DUTIES15	-0.165	0.014	0.000	-0.192	-0.139	*
PRESS15 WITH						
DUTIES15	-0.195	0.014	0.000	-0.223	-0.168	*
Means						
PHY15	0.004	0.014	0.389	-0.023	0.032	
KES15	0.003	0.014	0.429	-0.025	0.030	

```
    CHOICE15          -0.001       0.014       0.466      -0.029       0.027
    BULLY15            0.001       0.014       0.478      -0.027       0.029
    PRESS15            0.001       0.014       0.465      -0.026       0.028

 Variances
    PHY15              1.000       0.000       0.000       1.000       1.000
    KES15              1.000       0.000       0.000       1.000       1.000
    CHOICE15           1.000       0.000       0.000       1.000       1.000
    BULLY15            1.000       0.000       0.000       1.000       1.000
    PRESS15            1.000       0.000       0.000       1.000       1.000
    DUTIES15           1.000       0.000       0.000       1.000       1.000

Between Level

 S1         ON
    PRAFAC            -0.092       0.376       0.412      -0.792       0.642

 PHYCAT16   ON
    PRAFAC            -0.576       0.188       0.005      -0.910      -0.166      *
    DUTIES15          -0.266       0.289       0.199      -0.740       0.368

 PRAFAC   WITH
    DUTIES15           0.126       0.150       0.209      -0.190       0.396

 Means
    PRAFAC            -0.159       0.133       0.122      -0.419       0.108
    DUTIES15          20.484       2.252       0.000      16.291      25.229      *

 Intercepts
    S1                -0.307       0.452       0.187      -1.393       0.411

 Variances
    PRAFAC             1.000       0.000       0.000       1.000       1.000
    DUTIES15           1.000       0.000       0.000       1.000       1.000

 Residual Variances
    PHYCAT16           0.479       0.217       0.000       0.062       0.889      *
    S1                 0.924       0.185       0.000       0.307       1.000      *


R-SQUARE

Within-Level R-Square Averaged Across Clusters
```

Variable	Estimate	Posterior S.D.	One-Tailed P-Value	95% C.I. Lower 2.5%	Upper 2.5%
PHYCAT16	0.224	0.022	0.000	0.182	0.268

Between Level

Variable	Estimate	Posterior S.D.	One-Tailed P-Value	95% C.I. Lower 2.5%	Upper 2.5%
PHYCAT16	0.521	0.217	0.000	0.111	0.938

Variable	Estimate	Posterior S.D.	One-Tailed P-Value	95% C.I. Lower 2.5%	Upper 2.5%
S1	0.076	0.185	0.000	0.000	0.693

TECHNICAL 1 OUTPUT

PARAMETER SPECIFICATION FOR WITHIN

TAU

PHYCAT16
0

NU

PHYCAT16	PHY15	KES15	CHOICE15	BULLY15
0	0	0	0	0

NU

PRESS15	DUTIES15
0	0

LAMBDA

PHYCAT16 PHY15 KES15 CHOICE15 BULLY15

PHYCAT16	0	0	0	0	0
PHY15	0	0	0	0	0
KES15	0	0	0	0	0
CHOICE15	0	0	0	0	0
BULLY15	0	0	0	0	0
PRESS15	0	0	0	0	0
DUTIES15	0	0	0	0	0

LAMBDA

	PRESS15	DUTIES15
PHYCAT16	0	0
PHY15	0	0
KES15	0	0
CHOICE15	0	0
BULLY15	0	0
PRESS15	0	0
DUTIES15	0	0

THETA

	PHYCAT16	PHY15	KES15	CHOICE15	BULLY15
PHYCAT16	0				
PHY15	0	0			
KES15	0	0	0		
CHOICE15	0	0	0	0	
BULLY15	0	0	0	0	0
PRESS15	0	0	0	0	0
DUTIES15	0	0	0	0	0

THETA

	PRESS15	DUTIES15
PRESS15	0	
DUTIES15	0	0

ALPHA

PHYCAT16	PHY15	KES15	CHOICE15	BULLY15

0	1	2	3	4

ALPHA

PRESS15	DUTIES15
5	0

BETA

	PHYCAT16	PHY15	KES15	CHOICE15	BULLY15
PHYCAT16	0	6	7	8	9
PHY15	0	0	0	0	0
KES15	0	0	0	0	0
CHOICE15	0	0	0	0	0
BULLY15	0	0	0	0	0
PRESS15	0	0	0	0	0
DUTIES15	0	0	0	0	0

BETA

	PRESS15	DUTIES15
PHYCAT16	10	0
PHY15	0	0
KES15	0	0
CHOICE15	0	0
BULLY15	0	0
PRESS15	0	0
DUTIES15	0	0

PSI

	PHYCAT16	PHY15	KES15	CHOICE15	BULLY15
PHYCAT16	0				
PHY15	0	11			
KES15	0	12	13		
CHOICE15	0	14	15	16	
BULLY15	0	17	18	19	20
PRESS15	0	21	22	23	24
DUTIES15	0	26	27	28	29

PSI

	PRESS15	DUTIES15
PRESS15	25	
DUTIES15	30	31

PARAMETER SPECIFICATION FOR BETWEEN

TAU

PHYCAT16
43

NU

PHYCAT16	PRAFAC	DUTIES15
0	0	0

LAMBDA

	S1	PHYCAT16	PRAFAC	DUTIES15
PHYCAT16	0	0	0	0
PRAFAC	0	0	0	0
DUTIES15	0	0	0	0

THETA

	PHYCAT16	PRAFAC	DUTIES15
PHYCAT16	0		
PRAFAC	0	0	
DUTIES15	0	0	0

ALPHA

S1	PHYCAT16	PRAFAC	DUTIES15
32	0	33	34

BETA

	S1	PHYCAT16	PRAFAC	DUTIES15
S1	0	0	35	0
PHYCAT16	0	0	36	37
PRAFAC	0	0	0	0
DUTIES15	0	0	0	0

PSI

	S1	PHYCAT16	PRAFAC	DUTIES15
S1	38			
PHYCAT16	0	39		
PRAFAC	0	0	40	
DUTIES15	0	0	41	42

STARTING VALUES FOR WITHIN

TAU

PHYCAT16
0.000

NU

PHYCAT16	PHY15	KES15	CHOICE15	BULLY15
0.000	0.000	0.000	0.000	0.000

NU

PRESS15	DUTIES15
0.000	0.000

LAMBDA

	PHYCAT16	PHY15	KES15	CHOICE15	BULLY15
PHYCAT16	1.000	0.000	0.000	0.000	0.000

PHY15	0.000	1.000	0.000	0.000	0.000
KES15	0.000	0.000	1.000	0.000	0.000
CHOICE15	0.000	0.000	0.000	1.000	0.000
BULLY15	0.000	0.000	0.000	0.000	1.000
PRESS15	0.000	0.000	0.000	0.000	0.000
DUTIES15	0.000	0.000	0.000	0.000	0.000

LAMBDA

	PRESS15	DUTIES15
PHYCAT16	0.000	0.000
PHY15	0.000	0.000
KES15	0.000	0.000
CHOICE15	0.000	0.000
BULLY15	0.000	0.000
PRESS15	1.000	0.000
DUTIES15	0.000	1.000

THETA

	PHYCAT16	PHY15	KES15	CHOICE15	BULLY15
PHYCAT16	0.000				
PHY15	0.000	0.000			
KES15	0.000	0.000	0.000		
CHOICE15	0.000	0.000	0.000	0.000	
BULLY15	0.000	0.000	0.000	0.000	0.000
PRESS15	0.000	0.000	0.000	0.000	0.000
DUTIES15	0.000	0.000	0.000	0.000	0.000

THETA

	PRESS15	DUTIES15
PRESS15	0.000	
DUTIES15	0.000	0.000

ALPHA

PHYCAT16	PHY15	KES15	CHOICE15	BULLY15
0.000	0.000	0.000	0.000	0.000

ALPHA

PRESS15	DUTIES15
0.000	0.000

BETA

	PHYCAT16	PHY15	KES15	CHOICE15	BULLY15
PHYCAT16	0.000	0.000	0.000	0.000	0.000
PHY15	0.000	0.000	0.000	0.000	0.000
KES15	0.000	0.000	0.000	0.000	0.000
CHOICE15	0.000	0.000	0.000	0.000	0.000
BULLY15	0.000	0.000	0.000	0.000	0.000
PRESS15	0.000	0.000	0.000	0.000	0.000
DUTIES15	0.000	0.000	0.000	0.000	0.000

BETA

	PRESS15	DUTIES15
PHYCAT16	0.000	0.000
PHY15	0.000	0.000
KES15	0.000	0.000
CHOICE15	0.000	0.000
BULLY15	0.000	0.000
PRESS15	0.000	0.000
DUTIES15	0.000	0.000

PSI

	PHYCAT16	PHY15	KES15	CHOICE15	BULLY15
PHYCAT16	1.000				
PHY15	0.000	0.116			
KES15	0.000	0.000	0.145		
CHOICE15	0.000	0.000	0.000	0.541	
BULLY15	0.000	0.000	0.000	0.000	0.119
PRESS15	0.000	0.000	0.000	0.000	0.000
DUTIES15	0.000	0.000	0.000	0.000	0.000

PSI

	PRESS15	DUTIES15
PRESS15	0.501	
DUTIES15	0.000	0.430

STARTING VALUES FOR BETWEEN

TAU

PHYCAT16
1.110

NU

PHYCAT16	PRAFAC	DUTIES15
0.000	0.000	0.000

LAMBDA

	S1	PHYCAT16	PRAFAC	DUTIES15
PHYCAT16	0.000	1.000	0.000	0.000
PRAFAC	0.000	0.000	1.000	0.000
DUTIES15	0.000	0.000	0.000	1.000

THETA

	PHYCAT16	PRAFAC	DUTIES15
PHYCAT16	0.000		
PRAFAC	0.000	0.000	
DUTIES15	0.000	0.000	0.000

ALPHA

S1	PHYCAT16	PRAFAC	DUTIES15
0.000	0.000	0.020	4.120

BETA

	S1	PHYCAT16	PRAFAC	DUTIES15
S1	0.000	0.000	0.000	0.000
PHYCAT16	0.000	0.000	0.000	0.000
PRAFAC	0.000	0.000	0.000	0.000
DUTIES15	0.000	0.000	0.000	0.000

PSI

	S1	PHYCAT16	PRAFAC	DUTIES15
S1	1.000			
PHYCAT16	0.000	1.000		
PRAFAC	0.000	0.000	0.504	
DUTIES15	0.000	0.000	0.000	0.430

PRIORS FOR ALL PARAMETERS	PRIOR MEAN	PRIOR VARIANCE	PRIOR STD. DEV.
Parameter 1~N(0.000,infinity)	0.0000	infinity	infinity
Parameter 2~N(0.000,infinity)	0.0000	infinity	infinity
Parameter 3~N(0.000,infinity)	0.0000	infinity	infinity
Parameter 4~N(0.000,infinity)	0.0000	infinity	infinity
Parameter 5~N(0.000,infinity)	0.0000	infinity	infinity
Parameter 6~N(0.000,5.000)	0.0000	5.0000	2.2361
Parameter 7~N(0.000,5.000)	0.0000	5.0000	2.2361
Parameter 8~N(0.000,5.000)	0.0000	5.0000	2.2361
Parameter 9~N(0.000,5.000)	0.0000	5.0000	2.2361
Parameter 10~N(0.000,5.000)	0.0000	5.0000	2.2361
Parameter 11~IW(1.000,7)	infinity	infinity	infinity
Parameter 12~IW(0.000,7)	infinity	infinity	infinity
Parameter 13~IW(1.000,7)	infinity	infinity	infinity
Parameter 14~IW(0.000,7)	infinity	infinity	infinity
Parameter 15~IW(0.000,7)	infinity	infinity	infinity
Parameter 16~IW(1.000,7)	infinity	infinity	infinity
Parameter 17~IW(0.000,7)	infinity	infinity	infinity
Parameter 18~IW(0.000,7)	infinity	infinity	infinity
Parameter 19~IW(0.000,7)	infinity	infinity	infinity
Parameter 20~IW(1.000,7)	infinity	infinity	infinity
Parameter 21~IW(0.000,7)	infinity	infinity	infinity
Parameter 22~IW(0.000,7)	infinity	infinity	infinity
Parameter 23~IW(0.000,7)	infinity	infinity	infinity
Parameter 24~IW(0.000,7)	infinity	infinity	infinity

Parameter 25~IW(1.000,7)	infinity	infinity	infinity
Parameter 26~IW(0.000,7)	infinity	infinity	infinity
Parameter 27~IW(0.000,7)	infinity	infinity	infinity
Parameter 28~IW(0.000,7)	infinity	infinity	infinity
Parameter 29~IW(0.000,7)	infinity	infinity	infinity
Parameter 30~IW(0.000,7)	infinity	infinity	infinity
Parameter 31~IW(1.000,7)	infinity	infinity	infinity
Parameter 32~N(0.000,infinity)	0.0000	infinity	infinity
Parameter 33~N(0.000,infinity)	0.0000	infinity	infinity
Parameter 34~N(0.000,infinity)	0.0000	infinity	infinity
Parameter 35~N(0.000,infinity)	0.0000	infinity	infinity
Parameter 36~N(0.000,5.000)	0.0000	5.0000	2.2361
Parameter 37~N(0.000,5.000)	0.0000	5.0000	2.2361
Parameter 38~IG(-1.000,0.000)	infinity	infinity	infinity
Parameter 39~IG(-1.000,0.000)	infinity	infinity	infinity
Parameter 40~IW(1.000,3)	infinity	infinity	infinity
Parameter 41~IW(0.000,3)	infinity	infinity	infinity
Parameter 42~IW(1.000,3)	infinity	infinity	infinity
Parameter 43~N(0.000,5.000)	0.0000	5.0000	2.2361

TECHNICAL 3 OUTPUT

ESTIMATED COVARIANCE MATRIX FOR PARAMETER ESTIMATES

	1	2	3	4	5
1	0.455451D-04				
2	0.106367D-04	0.610745D-04			
3	-0.769776D-05	-0.114425D-04	0.221345D-03		
4	0.234537D-05	0.861675D-05	-0.732661D-05	0.453102D-04	
5	0.858757D-05	0.210713D-04	-0.166386D-04	0.192777D-04	0.188119D-03
6	0.121276D-04	-0.643977D-06	0.719344D-04	-0.847764D-06	-0.374415D-04
7	-0.189298D-04	0.867564D-05	-0.836544D-05	-0.227869D-05	-0.214395D-05
8	0.562695D-06	-0.745713D-05	0.991078D-05	-0.381821D-06	-0.939179D-05
9	0.723908D-05	-0.346076D-05	-0.372187D-04	-0.174459D-05	0.105942D-04
10	0.841012D-05	-0.165682D-05	0.616387D-05	-0.250379D-05	0.582677D-05
11	0.630048D-06	0.726189D-07	0.488007D-06	-0.586520D-06	0.381689D-06
12	-0.111985D-05	-0.955750D-06	-0.451478D-07	-0.486528D-06	-0.308140D-05
13	0.821935D-06	0.112486D-05	-0.168503D-05	0.348280D-06	-0.115501D-05
14	0.323971D-06	-0.114409D-06	-0.461383D-05	0.123633D-05	0.248943D-05
15	0.118180D-05	-0.144539D-05	-0.174025D-05	-0.230201D-05	-0.215149D-05
16	-0.754887D-05	0.215808D-06	0.106192D-04	0.109284D-05	0.429217D-05
17	0.106489D-06	0.685706D-06	-0.516722D-06	-0.812788D-06	0.688714D-06

18	-0.803237D-08	0.786717D-06	0.688748D-06	0.370690D-06	0.219257D-07
19	0.157137D-06	-0.147119D-06	0.302636D-05	-0.485149D-06	0.320167D-05
20	0.846641D-06	0.237019D-05	0.131659D-05	-0.229234D-06	-0.608067D-06
21	-0.486908D-06	0.192064D-05	0.177892D-05	-0.526133D-06	0.285275D-05
22	-0.968080D-06	-0.552613D-06	0.276809D-05	0.192326D-06	0.501745D-06
23	-0.229194D-05	-0.205644D-05	0.434683D-05	-0.209424D-05	-0.983731D-05
24	0.115845D-05	0.105403D-05	-0.125038D-05	-0.102750D-06	0.288354D-05
25	-0.112565D-05	-0.776055D-06	-0.286322D-05	0.305157D-05	0.490680D-05
26	0.216376D-05	-0.135902D-05	-0.402694D-05	0.607097D-06	-0.237986D-06
27	0.213143D-05	0.381196D-06	0.813378D-06	-0.100498D-05	0.175151D-05
28	-0.429623D-05	0.260313D-05	0.578476D-05	0.231064D-05	-0.662111D-06
29	0.971989D-06	-0.305684D-06	0.981458D-06	-0.210542D-05	0.585440D-07
30	0.120287D-05	0.252016D-05	-0.463157D-05	0.947655D-06	-0.261475D-05
31	-0.405698D-05	0.717116D-07	0.125625D-06	0.475210D-05	-0.114806D-04
32	-0.830920D-05	0.911474D-05	-0.938791D-05	0.146079D-05	0.222065D-04
33	-0.268421D-04	-0.432155D-04	0.972337D-04	-0.227614D-04	-0.102551D-04
34	-0.168835D-04	-0.279414D-04	0.317642D-04	-0.141806D-04	-0.386980D-04
35	0.347163D-05	0.864330D-06	0.157889D-04	0.394875D-05	0.814957D-05
36	-0.302241D-05	0.854823D-05	0.183523D-04	-0.211747D-05	0.730685D-05
37	0.144706D-04	0.105456D-03	0.434837D-04	0.351115D-04	0.170063D-04
38	-0.252190D-05	0.726061D-07	0.403000D-05	-0.942418D-06	-0.143763D-05
39	0.364052D-06	-0.293263D-05	-0.101272D-05	-0.181941D-05	-0.497736D-05
40	-0.431224D-04	-0.696455D-04	0.127441D-03	0.465716D-04	-0.503786D-04
41	-0.304640D-05	-0.261358D-05	-0.969189D-05	0.108048D-04	-0.378343D-05
42	-0.172735D-05	-0.228925D-05	-0.410588D-05	-0.233298D-05	-0.506177D-05
43	0.671122D-04	0.440736D-03	0.173518D-03	0.150482D-03	0.727032D-04

ESTIMATED COVARIANCE MATRIX FOR PARAMETER ESTIMATES

	6	7	8	9	10
6	0.487924D-02				
7	-0.334746D-03	0.204937D-02			
8	0.200493D-04	0.763660D-04	0.621683D-03		
9	0.104582D-03	-0.320047D-03	0.967288D-04	0.230816D-02	
10	-0.484356D-04	-0.169427D-03	0.153667D-04	-0.151859D-03	0.644832D-03
11	0.401671D-05	0.107453D-04	-0.427062D-05	-0.473840D-05	0.151811D-05
12	-0.780918D-05	0.764882D-05	-0.129825D-05	-0.103585D-05	-0.142107D-05
13	-0.139217D-04	0.808456D-05	-0.611857D-05	-0.777990D-05	0.218245D-05
14	0.308969D-05	-0.700538D-05	0.125473D-04	-0.641319D-06	0.669051D-06
15	0.318201D-04	-0.924483D-05	-0.175956D-05	0.119764D-04	0.183914D-05
16	0.115307D-04	0.456594D-04	0.129175D-04	0.339364D-05	0.207597D-05
17	-0.380456D-05	0.124886D-05	-0.957930D-06	0.102044D-05	-0.243748D-05
18	-0.451067D-05	0.696823D-05	-0.170848D-05	-0.651703D-06	-0.117401D-05

19	0.226764D-04	-0.934118D-05	-0.251805D-06	0.860303D-05	-0.774922D-06
20	-0.868219D-05	0.705025D-05	-0.233451D-05	0.643384D-06	0.291831D-05
21	-0.166096D-04	0.112265D-06	-0.929455D-08	0.840524D-05	-0.290774D-05
22	-0.153433D-04	0.118776D-04	0.110257D-05	0.525852D-05	-0.349944D-05
23	0.437699D-04	-0.185492D-04	-0.277614D-05	0.309076D-04	0.266612D-05
24	-0.666627D-05	0.104891D-04	-0.717752D-05	0.329332D-05	0.557300D-05
25	-0.459367D-04	0.249250D-04	-0.240300D-05	0.372223D-04	-0.159328D-04
26	0.108928D-04	-0.575678D-06	0.611120D-05	0.258982D-05	0.523528D-05
27	0.142600D-04	-0.894614D-05	-0.376730D-05	-0.841869D-06	0.435769D-05
28	-0.185514D-04	0.120732D-04	0.137884D-04	0.774096D-05	-0.170269D-04
29	0.967877D-05	-0.196740D-04	0.173482D-05	0.253112D-05	0.287888D-05
30	0.293496D-04	-0.152876D-04	0.116394D-04	-0.934782D-05	0.106311D-04
31	0.136784D-04	0.103015D-04	-0.167088D-04	0.535178D-05	-0.866994D-05
32	-0.662725D-04	0.291075D-03	-0.145386D-03	0.650109D-04	0.108680D-03
33	0.210365D-04	0.223778D-03	0.366656D-04	-0.753114D-04	-0.769929D-04
34	0.194955D-04	0.327948D-04	0.152610D-04	-0.673969D-04	0.527150D-05
35	-0.127391D-03	-0.180493D-04	-0.411839D-04	0.162282D-04	0.128372D-04
36	-0.756448D-04	0.685678D-04	-0.167343D-04	-0.101774D-03	0.182028D-04
37	0.209957D-03	0.443554D-03	0.691729D-04	0.360319D-03	-0.158281D-03
38	0.446931D-04	-0.251587D-04	-0.538094D-05	-0.757493D-05	-0.413519D-05
39	0.254321D-04	0.349932D-04	0.414649D-05	0.184874D-04	-0.345075D-05
40	0.153775D-03	0.121397D-03	0.103401D-03	0.167852D-03	-0.176783D-03
41	-0.193743D-05	-0.314547D-04	0.500621D-04	0.322702D-04	-0.750620D-05
42	-0.147534D-04	-0.134869D-04	0.775422D-05	-0.431247D-06	0.956962D-05
43	0.182591D-02	0.192795D-02	0.223859D-03	0.152783D-02	-0.707345D-03

ESTIMATED COVARIANCE MATRIX FOR PARAMETER ESTIMATES

	11	12	13	14	15
11	0.209334D-04				
12	0.453176D-05	0.135769D-04			
13	0.536672D-06	0.638866D-05	0.342349D-04		
14	-0.379432D-05	-0.395394D-05	-0.373927D-05	0.514743D-04	
15	0.233158D-06	-0.339458D-05	-0.133804D-04	0.116965D-04	0.665695D-04
16	0.289211D-05	0.155529D-05	0.652317D-05	-0.143288D-04	-0.360812D-04
17	0.660286D-06	0.269864D-05	0.103353D-05	-0.273513D-05	-0.386778D-06
18	-0.168820D-06	0.148224D-05	0.737568D-05	-0.210109D-05	-0.534804D-05
19	-0.113804D-05	-0.138981D-05	-0.261208D-05	0.386664D-05	0.114481D-04
20	0.852194D-06	0.377810D-06	0.178181D-05	0.231610D-06	-0.654555D-06
21	0.311980D-05	0.531239D-05	0.312991D-05	-0.460825D-05	-0.224613D-05
22	0.291128D-05	0.471372D-05	0.123933D-04	-0.400417D-05	-0.990600D-05
23	-0.184200D-05	-0.357395D-05	-0.726390D-05	0.914275D-05	0.212387D-04
24	0.568774D-06	0.171897D-05	0.360467D-05	-0.168900D-05	-0.331887D-05

25	0.381274D-05	0.294960D-05	0.842172D-05	-0.811888D-05	-0.862936D-05
26	-0.416602D-05	-0.590109D-05	-0.241304D-05	0.870796D-05	0.135369D-05
27	-0.191755D-05	-0.477085D-05	-0.143876D-04	0.510560D-05	0.136936D-04
28	0.117337D-05	0.208350D-05	0.543043D-05	-0.756463D-05	-0.248651D-04
29	-0.162711D-05	-0.207941D-05	-0.325375D-05	0.249135D-05	0.410358D-05
30	-0.186822D-05	-0.172313D-05	-0.655065D-05	0.118421D-05	0.571291D-05
31	0.286219D-05	0.298172D-05	0.811844D-05	-0.158806D-05	-0.101521D-04
32	0.431951D-05	0.409065D-05	0.991177D-05	-0.193278D-04	-0.585095D-05
33	0.181778D-04	0.204446D-04	0.988884D-06	0.185906D-04	0.568906D-04
34	0.628572D-06	0.259908D-05	0.795881D-05	-0.439272D-05	-0.929313D-05
35	-0.595347D-05	-0.561061D-05	0.126299D-04	0.235102D-05	-0.331587D-04
36	-0.712271D-06	0.443045D-05	0.934769D-07	0.616772D-06	-0.761709D-05
37	0.316878D-04	0.966816D-07	-0.380691D-04	-0.687192D-04	-0.113123D-05
38	0.493024D-06	-0.186557D-06	-0.879057D-07	0.851506D-06	0.627433D-06
39	0.288376D-05	0.104838D-05	0.132704D-06	-0.284394D-05	0.272627D-05
40	0.178010D-04	-0.486915D-05	0.158161D-04	-0.364267D-05	-0.436972D-04
41	-0.396318D-05	-0.193698D-05	-0.274415D-05	-0.834524D-05	-0.765484D-06
42	0.240241D-06	-0.670895D-07	-0.617493D-07	-0.332543D-05	-0.149528D-05
43	0.138419D-03	-0.270714D-05	-0.164496D-03	-0.287640D-03	0.163823D-04

ESTIMATED COVARIANCE MATRIX FOR PARAMETER ESTIMATES

	16	17	18	19	20
16	0.419988D-03				
17	-0.462672D-06	0.104393D-04			
18	0.574254D-05	0.224309D-05	0.148397D-04		
19	-0.276233D-04	-0.907306D-06	-0.456596D-05	0.515817D-04	
20	0.911238D-06	0.577438D-06	0.481051D-05	-0.507700D-05	0.222566D-04
21	0.205942D-05	0.501559D-05	0.226016D-05	-0.274245D-05	0.146340D-05
22	0.130347D-04	0.183230D-05	0.790046D-05	-0.336650D-05	0.170606D-05
23	-0.424199D-04	-0.469617D-05	-0.176355D-05	0.252150D-04	-0.350342D-06
24	0.349812D-05	0.168208D-05	0.593376D-05	-0.621967D-05	0.839532D-05
25	0.708486D-05	0.213510D-05	0.518297D-05	-0.414128D-05	0.500823D-05
26	-0.448086D-05	-0.422547D-05	-0.105364D-05	0.161700D-05	0.101794D-06
27	-0.102903D-04	-0.986786D-06	-0.604737D-05	0.474392D-05	-0.229256D-05
28	0.613878D-04	0.164173D-05	0.372819D-05	-0.176357D-04	-0.117944D-05
29	-0.308157D-05	-0.193778D-05	-0.576159D-05	0.100323D-04	-0.704253D-05
30	-0.147692D-05	-0.671874D-06	-0.529141D-05	0.970752D-05	-0.434604D-05
31	0.116125D-04	0.206254D-06	0.314684D-05	-0.834991D-05	0.197816D-05
32	0.373097D-05	0.160319D-05	0.580485D-05	-0.365859D-05	0.288727D-05
33	0.730476D-04	-0.136972D-05	-0.242703D-04	0.681047D-04	-0.917090D-05
34	0.159873D-04	0.288350D-05	0.449030D-05	-0.196278D-05	-0.213356D-05
35	0.240189D-04	-0.120390D-05	0.505046D-05	-0.201081D-05	-0.126867D-05

36	-0.124486D-04	-0.144241D-05	-0.206108D-05	-0.420646D-06	0.289023D-05
37	0.722952D-04	0.417850D-04	-0.171268D-05	-0.390495D-04	0.480405D-04
38	0.164553D-05	0.917552D-06	0.172435D-05	-0.375341D-05	0.134577D-05
39	0.514921D-05	0.706569D-06	0.112263D-05	0.269808D-06	-0.753922D-06
40	-0.101586D-04	0.143774D-04	0.146780D-04	0.407068D-04	-0.101936D-04
41	0.243848D-05	-0.113770D-05	0.261204D-05	0.295651D-05	0.624949D-05
42	0.308215D-05	0.818697D-07	0.118772D-06	0.248418D-06	-0.736659D-06
43	0.278668D-03	0.170883D-03	-0.585301D-05	-0.153666D-03	0.189698D-03

ESTIMATED COVARIANCE MATRIX FOR PARAMETER ESTIMATES

	21	22	23	24	25
21	0.462942D-04				
22	0.113372D-04	0.561881D-04			
23	-0.105652D-04	-0.189515D-04	0.215468D-03		
24	0.434241D-05	0.124300D-04	-0.143493D-04	0.474735D-04	
25	0.217664D-04	0.454981D-04	-0.303487D-04	0.382026D-04	0.414387D-03
26	-0.860668D-05	-0.347295D-05	0.526487D-05	-0.194358D-06	-0.544532D-05
27	-0.244155D-05	-0.137965D-04	0.945278D-05	-0.452532D-05	-0.124209D-04
28	0.624227D-05	0.786873D-05	-0.404330D-04	0.302572D-05	0.584783D-05
29	-0.321430D-05	-0.646982D-05	0.261201D-05	-0.863826D-05	-0.104596D-04
30	-0.984726D-05	-0.237880D-04	0.424732D-04	-0.187173D-04	-0.737278D-04
31	0.351931D-05	0.897762D-05	-0.119353D-04	0.474811D-05	0.190990D-04
32	0.218127D-05	0.399869D-05	0.260124D-05	0.112475D-04	0.774162D-05
33	0.529052D-05	-0.150585D-05	0.351029D-04	-0.186042D-04	-0.100375D-03
34	0.592319D-05	0.388209D-05	0.304012D-05	-0.367965D-05	0.768705D-05
35	-0.385089D-05	0.100649D-04	-0.904696D-05	-0.676900D-06	0.601153D-05
36	-0.894619D-05	0.236013D-05	0.734393D-05	0.256606D-06	0.183524D-04
37	-0.143931D-05	-0.463389D-04	-0.168998D-04	0.302233D-04	0.118769D-03
38	-0.158837D-05	-0.171514D-05	-0.342168D-05	0.183833D-05	-0.135508D-04
39	0.502219D-06	0.220164D-06	-0.223790D-05	-0.129080D-05	-0.630642D-05
40	0.687857D-05	0.454423D-04	-0.804739D-04	-0.295307D-04	0.576027D-04
41	-0.904390D-05	0.683058D-06	-0.359044D-06	-0.276117D-05	0.104176D-04
42	-0.320815D-06	0.553694D-06	0.452614D-05	0.761085D-06	-0.444105D-06
43	-0.154520D-04	-0.201105D-03	-0.491616D-04	0.116922D-03	0.474270D-03

ESTIMATED COVARIANCE MATRIX FOR PARAMETER ESTIMATES

	26	27	28	29	30
26	0.396997D-04				
27	0.956868D-05	0.541316D-04			
28	-0.967076D-05	-0.248832D-04	0.187640D-03		

29	0.232910D-05	0.134650D-04	-0.122334D-04	0.406453D-04	
30	0.877869D-05	0.249049D-04	-0.251173D-04	0.242026D-04	0.190260D-03
31	-0.115767D-04	-0.417915D-04	0.686259D-04	-0.313664D-04	-0.743325D-04
32	-0.281404D-05	-0.522897D-05	0.282421D-05	-0.127096D-04	-0.155320D-04
33	-0.181750D-04	0.232355D-04	-0.892856D-04	0.271007D-04	0.427812D-04
34	0.903484D-05	0.175723D-05	0.421991D-05	0.155414D-05	0.309263D-05
35	0.611906D-05	-0.490351D-05	-0.301686D-05	-0.397649D-05	-0.943318D-05
36	-0.241237D-05	0.601139D-05	0.409666D-05	0.648894D-05	0.652042D-05
37	-0.101688D-03	-0.107609D-04	0.438560D-04	-0.344090D-05	0.566008D-04
38	0.313424D-05	0.221756D-06	-0.516528D-06	-0.143857D-05	0.241142D-05
39	-0.167238D-06	0.567410D-07	-0.100124D-05	0.409637D-06	0.235451D-05
40	-0.156194D-04	-0.714791D-04	0.341920D-04	0.525299D-04	0.111153D-03
41	0.309481D-05	0.488739D-05	-0.255665D-04	-0.358484D-05	0.100247D-04
42	0.385867D-06	-0.211287D-07	-0.192945D-06	0.181100D-05	0.336328D-05
43	-0.424508D-03	-0.343188D-04	0.157341D-03	-0.130849D-04	0.240555D-03

ESTIMATED COVARIANCE MATRIX FOR PARAMETER ESTIMATES

	31	32	33	34	35
31	0.305692D-03				
32	0.264590D-04	0.134910D-02			
33	-0.378247D-04	0.505990D-04	0.241295D-01		
34	0.104293D-05	0.309923D-05	0.705121D-03	0.985167D-03	
35	-0.501363D-06	0.140536D-03	0.446836D-04	0.334226D-04	0.102693D-02
36	-0.105985D-04	0.451086D-04	0.102198D-03	-0.272837D-04	0.428240D-04
37	-0.968845D-05	-0.309987D-03	-0.665475D-03	0.329274D-05	0.634598D-04
38	0.218916D-05	0.368317D-04	-0.356612D-04	0.162123D-04	0.341009D-04
39	0.227935D-05	-0.976893D-05	0.134197D-04	0.199901D-05	-0.192994D-04
40	0.786588D-05	0.287146D-03	-0.424435D-03	0.377182D-03	0.247891D-03
41	-0.114983D-04	0.178004D-04	-0.752307D-04	-0.173054D-04	0.618296D-04
42	-0.403872D-05	0.850415D-05	0.466358D-05	0.907679D-05	-0.513724D-05
43	-0.288784D-05	-0.130484D-02	-0.260155D-02	-0.323510D-04	0.195767D-03

ESTIMATED COVARIANCE MATRIX FOR PARAMETER ESTIMATES

	36	37	38	39	40
36	0.923540D-03				
37	-0.100067D-02	0.673007D-01			
38	0.312222D-05	-0.650843D-04	0.103365D-03		
39	-0.443642D-04	0.139636D-03	-0.381576D-05	0.129606D-03	
40	0.175964D-03	-0.283408D-04	0.652392D-04	-0.172982D-04	0.676716D-01
41	0.559457D-04	0.568366D-04	-0.111762D-05	-0.429550D-05	0.106786D-02

42	0.630457D-05	0.525578D-05	0.229621D-05	-0.650319D-05	0.158413D-03
43	-0.427642D-02	0.278412D+00	-0.239611D-03	0.650442D-03	-0.188109D-03

ESTIMATED COVARIANCE MATRIX FOR PARAMETER ESTIMATES

	41	42	43
41	0.141641D-02		
42	0.596675D-04	0.894096D-04	
43	0.198233D-03	0.100240D-05	0.115310D+01

ESTIMATED CORRELATION MATRIX FOR PARAMETER ESTIMATES

	1	2	3	4	5
1	1.000				
2	0.202	1.000			
3	-0.077	-0.098	1.000		
4	0.052	0.164	-0.073	1.000	
5	0.093	0.197	-0.082	0.209	1.000
6	0.026	-0.001	0.069	-0.002	-0.039
7	-0.062	0.025	-0.012	-0.007	-0.003
8	0.003	-0.038	0.027	-0.002	-0.027
9	0.022	-0.009	-0.052	-0.005	0.016
10	0.049	-0.008	0.016	-0.015	0.017
11	0.020	0.002	0.007	-0.019	0.006
12	-0.045	-0.033	-0.001	-0.020	-0.061
13	0.021	0.025	-0.019	0.009	-0.014
14	0.007	-0.002	-0.043	0.026	0.025
15	0.021	-0.023	-0.014	-0.042	-0.019
16	-0.055	0.001	0.035	0.008	0.015
17	0.005	0.027	-0.011	-0.037	0.016
18	0.000	0.026	0.012	0.014	0.000
19	0.003	-0.003	0.028	-0.010	0.033
20	0.027	0.064	0.019	-0.007	-0.009
21	-0.011	0.036	0.018	-0.011	0.031
22	-0.019	-0.009	0.025	0.004	0.005
23	-0.023	-0.018	0.020	-0.021	-0.049
24	0.025	0.020	-0.012	-0.002	0.031
25	-0.008	-0.005	-0.009	0.022	0.018
26	0.051	-0.028	-0.043	0.014	-0.003
27	0.043	0.007	0.007	-0.020	0.017
28	-0.046	0.024	0.028	0.025	-0.004
29	0.023	-0.006	0.010	-0.049	0.001

30	0.013	0.023	-0.023	0.010	-0.014
31	-0.034	0.001	0.000	0.040	-0.048
32	-0.034	0.032	-0.017	0.006	0.044
33	-0.026	-0.036	0.042	-0.022	-0.005
34	-0.080	-0.114	0.068	-0.067	-0.090
35	0.016	0.003	0.033	0.018	0.019
36	-0.015	0.036	0.041	-0.010	0.018
37	0.008	0.052	0.011	0.020	0.005
38	-0.037	0.001	0.027	-0.014	-0.010
39	0.005	-0.033	-0.006	-0.024	-0.032
40	-0.025	-0.034	0.033	0.027	-0.014
41	-0.012	-0.009	-0.017	0.043	-0.007
42	-0.027	-0.031	-0.029	-0.037	-0.039
43	0.009	0.053	0.011	0.021	0.005

ESTIMATED CORRELATION MATRIX FOR PARAMETER ESTIMATES

	6	7	8	9	10
6	1.000				
7	-0.106	1.000			
8	0.012	0.068	1.000		
9	0.031	-0.147	0.081	1.000	
10	-0.027	-0.147	0.024	-0.124	1.000
11	0.013	0.052	-0.037	-0.022	0.013
12	-0.030	0.046	-0.014	-0.006	-0.015
13	-0.034	0.031	-0.042	-0.028	0.015
14	0.006	-0.022	0.070	-0.002	0.004
15	0.056	-0.025	-0.009	0.031	0.009
16	0.008	0.049	0.025	0.003	0.004
17	-0.017	0.009	-0.012	0.007	-0.030
18	-0.017	0.040	-0.018	-0.004	-0.012
19	0.045	-0.029	-0.001	0.025	-0.004
20	-0.026	0.033	-0.020	0.003	0.024
21	-0.035	0.000	0.000	0.026	-0.017
22	-0.029	0.035	0.006	0.015	-0.018
23	0.043	-0.028	-0.008	0.044	0.007
24	-0.014	0.034	-0.042	0.010	0.032
25	-0.032	0.027	-0.005	0.038	-0.031
26	0.025	-0.002	0.039	0.009	0.033
27	0.028	-0.027	-0.021	-0.002	0.023
28	-0.019	0.019	0.040	0.012	-0.049
29	0.022	-0.068	0.011	0.008	0.018
30	0.030	-0.024	0.034	-0.014	0.030

31	0.011	0.013	-0.038	0.006	-0.020
32	-0.026	0.175	-0.159	0.037	0.117
33	0.002	0.032	0.009	-0.010	-0.020
34	0.009	0.023	0.020	-0.045	0.007
35	-0.057	-0.012	-0.052	0.011	0.016
36	-0.036	0.050	-0.022	-0.070	0.024
37	0.012	0.038	0.011	0.029	-0.024
38	0.063	-0.055	-0.021	-0.016	-0.016
39	0.032	0.068	0.015	0.034	-0.012
40	0.008	0.010	0.016	0.013	-0.027
41	-0.001	-0.018	0.053	0.018	-0.008
42	-0.022	-0.032	0.033	-0.001	0.040
43	0.024	0.040	0.008	0.030	-0.026

ESTIMATED CORRELATION MATRIX FOR PARAMETER ESTIMATES

	11	12	13	14	15
11	1.000				
12	0.269	1.000			
13	0.020	0.296	1.000		
14	-0.116	-0.150	-0.089	1.000	
15	0.006	-0.113	-0.280	0.200	1.000
16	0.031	0.021	0.054	-0.097	-0.216
17	0.045	0.227	0.055	-0.118	-0.015
18	-0.010	0.104	0.327	-0.076	-0.170
19	-0.035	-0.053	-0.062	0.075	0.195
20	0.039	0.022	0.065	0.007	-0.017
21	0.100	0.212	0.079	-0.094	-0.040
22	0.085	0.171	0.283	-0.074	-0.162
23	-0.027	-0.066	-0.085	0.087	0.177
24	0.018	0.068	0.089	-0.034	-0.059
25	0.041	0.039	0.071	-0.056	-0.052
26	-0.145	-0.254	-0.065	0.193	0.026
27	-0.057	-0.176	-0.334	0.097	0.228
28	0.019	0.041	0.068	-0.077	-0.222
29	-0.056	-0.089	-0.087	0.054	0.079
30	-0.030	-0.034	-0.081	0.012	0.051
31	0.036	0.046	0.079	-0.013	-0.071
32	0.026	0.030	0.046	-0.073	-0.020
33	0.026	0.036	0.001	0.017	0.045
34	0.004	0.022	0.043	-0.020	-0.036
35	-0.041	-0.048	0.067	0.010	-0.127
36	-0.005	0.040	0.001	0.003	-0.031

37	0.027	0.000	-0.025	-0.037	-0.001
38	0.011	-0.005	-0.001	0.012	0.008
39	0.055	0.025	0.002	-0.035	0.029
40	0.015	-0.005	0.010	-0.002	-0.021
41	-0.023	-0.014	-0.012	-0.031	-0.002
42	0.006	-0.002	-0.001	-0.049	-0.019
43	0.028	-0.001	-0.026	-0.037	0.002

ESTIMATED CORRELATION MATRIX FOR PARAMETER ESTIMATES

	16	17	18	19	20
16	1.000				
17	-0.007	1.000			
18	0.073	0.180	1.000		
19	-0.188	-0.039	-0.165	1.000	
20	0.009	0.038	0.265	-0.150	1.000
21	0.015	0.228	0.086	-0.056	0.046
22	0.085	0.076	0.274	-0.063	0.048
23	-0.141	-0.099	-0.031	0.239	-0.005
24	0.025	0.076	0.224	-0.126	0.258
25	0.017	0.032	0.066	-0.028	0.052
26	-0.035	-0.208	-0.043	0.036	0.003
27	-0.068	-0.042	-0.213	0.090	-0.066
28	0.219	0.037	0.071	-0.179	-0.018
29	-0.024	-0.094	-0.235	0.219	-0.234
30	-0.005	-0.015	-0.100	0.098	-0.067
31	0.032	0.004	0.047	-0.066	0.024
32	0.005	0.014	0.041	-0.014	0.017
33	0.023	-0.003	-0.041	0.061	-0.013
34	0.025	0.028	0.037	-0.009	-0.014
35	0.037	-0.012	0.041	-0.009	-0.008
36	-0.020	-0.015	-0.018	-0.002	0.020
37	0.014	0.050	-0.002	-0.021	0.039
38	0.008	0.028	0.044	-0.051	0.028
39	0.022	0.019	0.026	0.003	-0.014
40	-0.002	0.017	0.015	0.022	-0.008
41	0.003	-0.009	0.018	0.011	0.035
42	0.016	0.003	0.003	0.004	-0.017
43	0.013	0.049	-0.001	-0.020	0.037

ESTIMATED CORRELATION MATRIX FOR PARAMETER ESTIMATES

21	22	23	24	25

21	1.000				
22	0.222	1.000			
23	-0.106	-0.172	1.000		
24	0.093	0.241	-0.142	1.000	
25	0.157	0.298	-0.102	0.272	1.000
26	-0.201	-0.074	0.057	-0.004	-0.042
27	-0.049	-0.250	0.088	-0.089	-0.083
28	0.067	0.077	-0.201	0.032	0.021
29	-0.074	-0.135	0.028	-0.197	-0.081
30	-0.105	-0.230	0.210	-0.197	-0.263
31	0.030	0.069	-0.047	0.039	0.054
32	0.009	0.015	0.005	0.044	0.010
33	0.005	-0.001	0.015	-0.017	-0.032
34	0.028	0.017	0.007	-0.017	0.012
35	-0.018	0.042	-0.019	-0.003	0.009
36	-0.043	0.010	0.016	0.001	0.030
37	-0.001	-0.024	-0.004	0.017	0.022
38	-0.023	-0.023	-0.023	0.026	-0.065
39	0.006	0.003	-0.013	-0.016	-0.027
40	0.004	0.023	-0.021	-0.016	0.011
41	-0.035	0.002	-0.001	-0.011	0.014
42	-0.005	0.008	0.033	0.012	-0.002
43	-0.002	-0.025	-0.003	0.016	0.022

ESTIMATED CORRELATION MATRIX FOR PARAMETER ESTIMATES

	26	27	28	29	30
26	1.000				
27	0.206	1.000			
28	-0.112	-0.247	1.000		
29	0.058	0.287	-0.140	1.000	
30	0.101	0.245	-0.133	0.275	1.000
31	-0.105	-0.325	0.287	-0.281	-0.308
32	-0.012	-0.019	0.006	-0.054	-0.031
33	-0.019	0.020	-0.042	0.027	0.020
34	0.046	0.008	0.010	0.008	0.007
35	0.030	-0.021	-0.007	-0.019	-0.021
36	-0.013	0.027	0.010	0.033	0.016
37	-0.062	-0.006	0.012	-0.002	0.016
38	0.049	0.003	-0.004	-0.022	0.017
39	-0.002	0.001	-0.006	0.006	0.015
40	-0.010	-0.037	0.010	0.032	0.031

41	0.013	0.018	-0.050	-0.015	0.019
42	0.006	0.000	-0.001	0.030	0.026
43	-0.063	-0.004	0.011	-0.002	0.016

ESTIMATED CORRELATION MATRIX FOR PARAMETER ESTIMATES

	31	32	33	34	35
31	1.000				
32	0.041	1.000			
33	-0.014	0.009	1.000		
34	0.002	0.003	0.145	1.000	
35	-0.001	0.119	0.009	0.033	1.000
36	-0.020	0.040	0.022	-0.029	0.044
37	-0.002	-0.033	-0.017	0.000	0.008
38	0.012	0.099	-0.023	0.051	0.105
39	0.011	-0.023	0.008	0.006	-0.053
40	0.002	0.030	-0.011	0.046	0.030
41	-0.017	0.013	-0.013	-0.015	0.051
42	-0.024	0.024	0.003	0.031	-0.017
43	0.000	-0.033	-0.016	-0.001	0.006

ESTIMATED CORRELATION MATRIX FOR PARAMETER ESTIMATES

	36	37	38	39	40
36	1.000				
37	-0.127	1.000			
38	0.010	-0.025	1.000		
39	-0.128	0.047	-0.033	1.000	
40	0.022	0.000	0.025	-0.006	1.000
41	0.049	0.006	-0.003	-0.010	0.109
42	0.022	0.002	0.024	-0.060	0.064
43	-0.131	0.999	-0.022	0.053	-0.001

ESTIMATED CORRELATION MATRIX FOR PARAMETER ESTIMATES

	41	42	43
41	1.000		
42	0.168	1.000	
43	0.005	0.000	1.000

TECHNICAL 8 OUTPUT

TECHNICAL 8 OUTPUT FOR BAYES ESTIMATION

CHAIN	BSEED
1	0
2	285380
3	253358

ITERATION	POTENTIAL SCALE REDUCTION	PARAMETER WITH HIGHEST PSR
100	1.022	38
200	1.007	7
300	1.006	35
400	1.002	23
500	1.003	41
600	1.006	41
700	1.002	41
800	1.004	38
900	1.008	38
1000	1.003	38

Improvement in physical health, job control interaction

SUMMARY OF DATA

Number of clusters 57

Size (s)	Cluster ID with Size s
2	4 166
3	65 74 80 30
4	84 22
5	205
6	228
7	93
8	106 144 165 71 170 173 192 72 50
9	40 108
11	107 83 181 61 75 68
12	69 174 206 226 81
13	162
16	92 154
19	114
20	145 2
22	116
23	36 156 169 142
24	152
26	98
28	122 202
30	178
32	6
46	24
59	204
62	125
64	209
108	217
216	110
223	100

COVARIANCE COVERAGE OF DATA

Minimum covariance coverage value 0.100

Number of missing data patterns 4

PROPORTION OF DATA PRESENT

Covariance Coverage

	PHYCAT16	PRAFAC	PHY15	KES15	CHOICE15
PHYCAT16	1.000				
PRAFAC	1.000	1.000			
PHY15	1.000	1.000	1.000		
KES15	0.971	0.971	0.971	0.971	
CHOICE15	0.963	0.963	0.963	0.963	0.963
BULLY15	0.966	0.966	0.966	0.966	0.963
PRESS15	0.966	0.966	0.966	0.966	0.963
DUTIES15	0.966	0.966	0.966	0.966	0.963

Covariance Coverage

	BULLY15	PRESS15	DUTIES15
BULLY15	0.966		
PRESS15	0.966	0.966	
DUTIES15	0.966	0.966	0.966

UNIVARIATE PROPORTIONS AND COUNTS FOR CATEGORICAL VARIABLES

PHYCAT16

Category 1	0.364	517.000
Category 2	0.636	905.000

UNIVARIATE SAMPLE STATISTICS

UNIVARIATE HIGHER-ORDER MOMENT DESCRIPTIVE STATISTICS

Variable/ Sample Size	Mean/ Variance	Skewness/ Kurtosis	Minimum/ Maximum	% with Min/Max	Percentiles 20%/60%	40%/80%	Median
PRAFAC	-0.173	0.219	-2.338	1.75%	-1.489	-0.520	-0.258
57.000	1.361	-0.662	2.338	1.75%	0.080	0.848	

```
     PHY15                 0.000       2.446      -0.155   85.58%      -0.155     -0.155     -0.155
            1422.000       0.154       5.391       1.845    1.13%      -0.155     -0.155
     KES15                 0.000       1.141      -0.953    8.62%      -0.620     -0.287     -0.120
            1381.000       0.613       1.071       3.047    0.36%       0.047      0.547
     CHOICE15              2.878      -0.138       1.000   14.60%       2.000      3.000      3.000
            1370.000       1.250      -0.758       5.000    5.77%       3.000      4.000
     BULLY15               0.000       2.479      -0.312   79.02%      -0.312     -0.312     -0.312
            1373.000       0.474       6.315       3.688    0.44%      -0.312      0.688
     PRESS15               0.000       0.183      -1.756   14.06%      -0.756     -0.756      0.244
            1373.000       1.207      -0.514       2.244    7.21%       0.244      1.244
     DUTIES15              0.000      -0.896      -2.873    3.06%      -0.873      0.127      0.127
            1373.000       0.975       0.587       1.127   27.75%       0.127      1.127


THE MODEL ESTIMATION TERMINATED NORMALLY

     USE THE FBITERATIONS OPTION TO INCREASE THE NUMBER OF ITERATIONS BY A FACTOR
     OF AT LEAST TWO TO CHECK CONVERGENCE AND THAT THE PSR VALUE DOES NOT INCREASE.



MODEL FIT INFORMATION

Number of Free Parameters                              43



MODEL RESULTS

                                Posterior  One-Tailed         95% C.I.
                    Estimate       S.D.      P-Value   Lower 2.5%  Upper 2.5%  Significance

Within Level

 PHYCAT16   ON
    PHY15              0.828       0.116      0.000       0.611       1.055      *
    BULLY15            0.049       0.056      0.201      -0.060       0.155
    DUTIES15           0.034       0.038      0.189      -0.041       0.109
    PRESS15           -0.003       0.035      0.466      -0.069       0.066
    KES15              0.166       0.052      0.000       0.060       0.262      *

 KES15    WITH
    PHY15              0.070       0.009      0.000       0.054       0.089      *
    CHOICE15          -0.131       0.024      0.000      -0.179      -0.086      *
```

```
    BULLY15            0.127      0.015      0.000      0.098      0.158      *
    PRESS15            0.206      0.024      0.000      0.161      0.255      *
    DUTIES15          -0.207      0.021      0.000     -0.247     -0.164      *

 PHY15    WITH
    CHOICE15          -0.030      0.012      0.006     -0.055     -0.008      *
    BULLY15            0.013      0.007      0.037     -0.001      0.026
    PRESS15            0.015      0.012      0.107     -0.008      0.037
    DUTIES15          -0.009      0.011      0.196     -0.029      0.012

 CHOICE15 WITH
    BULLY15           -0.122      0.021      0.000     -0.162     -0.081      *
    PRESS15           -0.170      0.032      0.000     -0.235     -0.107      *
    DUTIES15           0.163      0.030      0.000      0.106      0.225      *

 BULLY15  WITH
    PRESS15            0.178      0.020      0.000      0.136      0.218      *
    DUTIES15          -0.141      0.019      0.000     -0.180     -0.105      *

 PRESS15  WITH
    DUTIES15          -0.244      0.030      0.000     -0.306     -0.183      *

 Means
    PHY15              0.000      0.010      0.499     -0.019      0.022
    KES15              0.000      0.021      0.497     -0.042      0.042
    BULLY15            0.001      0.018      0.481     -0.035      0.037
    PRESS15            0.002      0.030      0.484     -0.059      0.058
    DUTIES15          -0.001      0.028      0.494     -0.054      0.052

 Variances
    PHY15              0.154      0.006      0.000      0.144      0.166      *
    KES15              0.615      0.024      0.000      0.569      0.661      *
    CHOICE15           1.202      0.046      0.000      1.117      1.294      *
    BULLY15            0.475      0.018      0.000      0.439      0.511      *
    PRESS15            1.208      0.046      0.000      1.119      1.301      *
    DUTIES15           0.977      0.037      0.000      0.908      1.052      *

Between Level

 S1         ON
    PRAFAC             0.031      0.038      0.223     -0.048      0.100

 PHYCAT16   ON
    PRAFAC            -0.049      0.041      0.115     -0.129      0.035
```

```
   CHOICE15          -0.105       0.185      0.271      -0.494       0.263

 PRAFAC   WITH
   CHOICE15           0.005       0.072      0.477      -0.138       0.144

 Means
   PRAFAC            -0.172       0.154      0.137      -0.467       0.132
   CHOICE15           2.954       0.057      0.000       2.841       3.066      *

 Intercepts
   S1                -0.038       0.040      0.155      -0.120       0.037

 Thresholds
   PHYCAT16$1        -0.700       0.541      0.092      -1.830       0.385

 Variances
   PRAFAC             1.369       0.264      0.000       0.992       2.003      *
   CHOICE15           0.084       0.024      0.000       0.051       0.142      *

 Residual Variances
   PHYCAT16           0.007       0.011      0.000       0.000       0.039      *
   S1                 0.005       0.009      0.000       0.000       0.031      *


STANDARDIZED MODEL RESULTS


STDYX Standardization

                               Posterior  One-Tailed         95% C.I.
                    Estimate       S.D.      P-Value   Lower 2.5%  Upper 2.5%  Significance

Within-Level Standardized Estimates Averaged Over Clusters

 S1 | PHYCAT16 ON
   CHOICE15          -0.043       0.038      0.127      -0.117       0.030

 PHYCAT16   ON
   PHY15              0.302       0.039      0.000       0.222       0.377      *
   BULLY15            0.032       0.036      0.201      -0.038       0.098
   DUTIES15           0.031       0.035      0.188      -0.039       0.098
   PRESS15           -0.002       0.035      0.473      -0.071       0.068
   KES15              0.120       0.038      0.000       0.045       0.194      *
```

```
 KES15    WITH
    PHY15              0.230      0.026      0.000       0.178       0.280      *
    CHOICE15          -0.153      0.026      0.000      -0.203      -0.100      *
    BULLY15            0.236      0.026      0.000       0.184       0.285      *
    PRESS15            0.240      0.025      0.000       0.191       0.290      *
    DUTIES15          -0.267      0.025      0.000      -0.313      -0.216      *

 PHY15    WITH
    CHOICE15          -0.070      0.027      0.005      -0.125      -0.018      *
    BULLY15            0.046      0.027      0.040      -0.005       0.098
    PRESS15            0.036      0.028      0.105      -0.019       0.088
    DUTIES15          -0.024      0.028      0.199      -0.077       0.032

 CHOICE15 WITH
    BULLY15           -0.161      0.027      0.000      -0.212      -0.107      *
    PRESS15           -0.140      0.027      0.000      -0.191      -0.086      *
    DUTIES15           0.150      0.027      0.000       0.097       0.201      *

 BULLY15  WITH
    PRESS15            0.234      0.025      0.000       0.183       0.282      *
    DUTIES15          -0.206      0.026      0.000      -0.256      -0.154      *

 PRESS15  WITH
    DUTIES15          -0.224      0.026      0.000      -0.274      -0.173      *

 Means
    PHY15              0.000      0.027      0.499      -0.050       0.055
    KES15              0.001      0.026      0.492      -0.052       0.052
    BULLY15            0.001      0.026      0.482      -0.051       0.053
    PRESS15            0.001      0.027      0.490      -0.053       0.054
    DUTIES15          -0.001      0.028      0.494      -0.055       0.054

 Variances
    PHY15              1.000      0.000      0.000       1.000       1.000
    KES15              1.000      0.000      0.000       1.000       1.000
    CHOICE15           1.000      0.000      0.000       1.000       1.000
    BULLY15            1.000      0.000      0.000       1.000       1.000
    PRESS15            1.000      0.000      0.000       1.000       1.000
    DUTIES15           1.000      0.000      0.000       1.000       1.000

Between Level

 S1         ON
    PRAFAC             0.444      0.449      0.223      -0.650       0.970
```

```
 PHYCAT16   ON
    PRAFAC            -0.485       0.343      0.115      -0.951       0.361
    CHOICE15          -0.263       0.387      0.271      -0.906       0.583

 PRAFAC   WITH
    CHOICE15           0.014       0.198      0.477      -0.374       0.379

 Means
    PRAFAC            -0.145       0.131      0.137      -0.407       0.113
    CHOICE15          10.237       1.353      0.000       7.767      13.095      *

 Intercepts
    S1                -0.425       0.594      0.155      -1.946       0.395

 Variances
    PRAFAC             1.000       0.000      0.000       1.000       1.000
    CHOICE15           1.000       0.000      0.000       1.000       1.000

 Residual Variances
    PHYCAT16           0.490       0.274      0.000       0.022       0.960      *
    S1                 0.756       0.301      0.000       0.060       0.999      *


R-SQUARE

Within-Level R-Square Averaged Across Clusters

                                Posterior  One-Tailed         95% C.I.
    Variable        Estimate       S.D.      P-Value   Lower 2.5%  Upper 2.5%

    PHYCAT16           0.143       0.027      0.000       0.096       0.201

Between Level

                                Posterior  One-Tailed         95% C.I.
    Variable        Estimate       S.D.      P-Value   Lower 2.5%  Upper 2.5%

    PHYCAT16           0.510       0.274      0.000       0.040       0.978

                                Posterior  One-Tailed         95% C.I.
    Variable        Estimate       S.D.      P-Value   Lower 2.5%  Upper 2.5%

    S1                 0.244       0.301      0.000       0.001       0.940
```

TECHNICAL 1 OUTPUT

PARAMETER SPECIFICATION FOR WITHIN

TAU

	PHYCAT16
	0

NU

PHYCAT16	PHY15	KES15	CHOICE15	BULLY15
0	0	0	0	0

NU

PRESS15	DUTIES15
0	0

LAMBDA

	PHYCAT16	PHY15	KES15	CHOICE15	BULLY15
PHYCAT16	0	0	0	0	0
PHY15	0	0	0	0	0
KES15	0	0	0	0	0
CHOICE15	0	0	0	0	0
BULLY15	0	0	0	0	0
PRESS15	0	0	0	0	0
DUTIES15	0	0	0	0	0

LAMBDA

	PRESS15	DUTIES15
PHYCAT16	0	0
PHY15	0	0
KES15	0	0

CHOICE15	0	0
BULLY15	0	0
PRESS15	0	0
DUTIES15	0	0

THETA

	PHYCAT16	PHY15	KES15	CHOICE15	BULLY15
PHYCAT16	0				
PHY15	0	0			
KES15	0	0	0		
CHOICE15	0	0	0	0	
BULLY15	0	0	0	0	0
PRESS15	0	0	0	0	0
DUTIES15	0	0	0	0	0

THETA

	PRESS15	DUTIES15
PRESS15	0	
DUTIES15	0	0

ALPHA

PHYCAT16	PHY15	KES15	CHOICE15	BULLY15
0	1	2	0	3

ALPHA

PRESS15	DUTIES15
4	5

BETA

	PHYCAT16	PHY15	KES15	CHOICE15	BULLY15
PHYCAT16	0	6	7	0	8
PHY15	0	0	0	0	0
KES15	0	0	0	0	0
CHOICE15	0	0	0	0	0

BULLY15	0	0	0	0	0
PRESS15	0	0	0	0	0
DUTIES15	0	0	0	0	0

BETA

	PRESS15	DUTIES15
PHYCAT16	9	10
PHY15	0	0
KES15	0	0
CHOICE15	0	0
BULLY15	0	0
PRESS15	0	0
DUTIES15	0	0

PSI

	PHYCAT16	PHY15	KES15	CHOICE15	BULLY15
PHYCAT16	0				
PHY15	0	11			
KES15	0	12	13		
CHOICE15	0	14	15	16	
BULLY15	0	17	18	19	20
PRESS15	0	21	22	23	24
DUTIES15	0	26	27	28	29

PSI

	PRESS15	DUTIES15
PRESS15	25	
DUTIES15	30	31

PARAMETER SPECIFICATION FOR BETWEEN

TAU

PHYCAT16
43

NU

PHYCAT16	PRAFAC	CHOICE15
0	0	0

LAMBDA

	S1	PHYCAT16	PRAFAC	CHOICE15
PHYCAT16	0	0	0	0
PRAFAC	0	0	0	0
CHOICE15	0	0	0	0

THETA

	PHYCAT16	PRAFAC	CHOICE15
PHYCAT16	0		
PRAFAC	0	0	
CHOICE15	0	0	0

ALPHA

S1	PHYCAT16	PRAFAC	CHOICE15
32	0	33	34

BETA

	S1	PHYCAT16	PRAFAC	CHOICE15
S1	0	0	35	0
PHYCAT16	0	0	36	37
PRAFAC	0	0	0	0
CHOICE15	0	0	0	0

PSI

	S1	PHYCAT16	PRAFAC	CHOICE15
S1	38			
PHYCAT16	0	39		
PRAFAC	0	0	40	

CHOICE15	0	0	41	42

STARTING VALUES FOR WITHIN

TAU

PHYCAT16
0.000

NU

PHYCAT16	PHY15	KES15	CHOICE15	BULLY15
0.000	0.000	0.000	0.000	0.000

NU

PRESS15	DUTIES15
0.000	0.000

LAMBDA

	PHYCAT16	PHY15	KES15	CHOICE15	BULLY15
PHYCAT16	1.000	0.000	0.000	0.000	0.000
PHY15	0.000	1.000	0.000	0.000	0.000
KES15	0.000	0.000	1.000	0.000	0.000
CHOICE15	0.000	0.000	0.000	1.000	0.000
BULLY15	0.000	0.000	0.000	0.000	1.000
PRESS15	0.000	0.000	0.000	0.000	0.000
DUTIES15	0.000	0.000	0.000	0.000	0.000

LAMBDA

	PRESS15	DUTIES15
PHYCAT16	0.000	0.000
PHY15	0.000	0.000
KES15	0.000	0.000
CHOICE15	0.000	0.000
BULLY15	0.000	0.000

PRESS15	1.000	0.000
DUTIES15	0.000	1.000

THETA

	PHYCAT16	PHY15	KES15	CHOICE15	BULLY15
PHYCAT16	0.000				
PHY15	0.000	0.000			
KES15	0.000	0.000	0.000		
CHOICE15	0.000	0.000	0.000	0.000	
BULLY15	0.000	0.000	0.000	0.000	0.000
PRESS15	0.000	0.000	0.000	0.000	0.000
DUTIES15	0.000	0.000	0.000	0.000	0.000

THETA

	PRESS15	DUTIES15
PRESS15	0.000	
DUTIES15	0.000	0.000

ALPHA

PHYCAT16	PHY15	KES15	CHOICE15	BULLY15
0.000	0.000	0.000	0.000	0.000

ALPHA

PRESS15	DUTIES15
0.000	0.000

BETA

	PHYCAT16	PHY15	KES15	CHOICE15	BULLY15
PHYCAT16	0.000	0.000	0.000	0.000	0.000
PHY15	0.000	0.000	0.000	0.000	0.000
KES15	0.000	0.000	0.000	0.000	0.000
CHOICE15	0.000	0.000	0.000	0.000	0.000
BULLY15	0.000	0.000	0.000	0.000	0.000
PRESS15	0.000	0.000	0.000	0.000	0.000

DUTIES15	0.000	0.000	0.000	0.000	0.000

BETA

	PRESS15	DUTIES15
PHYCAT16	0.000	0.000
PHY15	0.000	0.000
KES15	0.000	0.000
CHOICE15	0.000	0.000
BULLY15	0.000	0.000
PRESS15	0.000	0.000
DUTIES15	0.000	0.000

PSI

	PHYCAT16	PHY15	KES15	CHOICE15	BULLY15
PHYCAT16	1.000				
PHY15	0.000	0.077			
KES15	0.000	0.000	0.306		
CHOICE15	0.000	0.000	0.000	0.625	
BULLY15	0.000	0.000	0.000	0.000	0.237
PRESS15	0.000	0.000	0.000	0.000	0.000
DUTIES15	0.000	0.000	0.000	0.000	0.000

PSI

	PRESS15	DUTIES15
PRESS15	0.604	
DUTIES15	0.000	0.488

STARTING VALUES FOR BETWEEN

TAU

PHYCAT16
-0.311

NU

PHYCAT16	PRAFAC	CHOICE15
0.000	0.000	0.000

LAMBDA

	S1	PHYCAT16	PRAFAC	CHOICE15
PHYCAT16	0.000	1.000	0.000	0.000
PRAFAC	0.000	0.000	1.000	0.000
CHOICE15	0.000	0.000	0.000	1.000

THETA

	PHYCAT16	PRAFAC	CHOICE15
PHYCAT16	0.000		
PRAFAC	0.000	0.000	
CHOICE15	0.000	0.000	0.000

ALPHA

S1	PHYCAT16	PRAFAC	CHOICE15
0.000	0.000	-0.078	2.878

BETA

	S1	PHYCAT16	PRAFAC	CHOICE15
S1	0.000	0.000	0.000	0.000
PHYCAT16	0.000	0.000	0.000	0.000
PRAFAC	0.000	0.000	0.000	0.000
CHOICE15	0.000	0.000	0.000	0.000

PSI

	S1	PHYCAT16	PRAFAC	CHOICE15
S1	1.000			
PHYCAT16	0.000	1.000		
PRAFAC	0.000	0.000	0.480	
CHOICE15	0.000	0.000	0.000	0.625

PRIORS FOR ALL PARAMETERS	PRIOR MEAN	PRIOR VARIANCE	PRIOR STD. DEV.
Parameter 1~N(0.000,infinity)	0.0000	infinity	infinity
Parameter 2~N(0.000,infinity)	0.0000	infinity	infinity
Parameter 3~N(0.000,infinity)	0.0000	infinity	infinity
Parameter 4~N(0.000,infinity)	0.0000	infinity	infinity
Parameter 5~N(0.000,infinity)	0.0000	infinity	infinity
Parameter 6~N(0.000,5.000)	0.0000	5.0000	2.2361
Parameter 7~N(0.000,5.000)	0.0000	5.0000	2.2361
Parameter 8~N(0.000,5.000)	0.0000	5.0000	2.2361
Parameter 9~N(0.000,5.000)	0.0000	5.0000	2.2361
Parameter 10~N(0.000,5.000)	0.0000	5.0000	2.2361
Parameter 11~IW(1.000,7)	infinity	infinity	infinity
Parameter 12~IW(0.000,7)	infinity	infinity	infinity
Parameter 13~IW(1.000,7)	infinity	infinity	infinity
Parameter 14~IW(0.000,7)	infinity	infinity	infinity
Parameter 15~IW(0.000,7)	infinity	infinity	infinity
Parameter 16~IW(1.000,7)	infinity	infinity	infinity
Parameter 17~IW(0.000,7)	infinity	infinity	infinity
Parameter 18~IW(0.000,7)	infinity	infinity	infinity
Parameter 19~IW(0.000,7)	infinity	infinity	infinity
Parameter 20~IW(1.000,7)	infinity	infinity	infinity
Parameter 21~IW(0.000,7)	infinity	infinity	infinity
Parameter 22~IW(0.000,7)	infinity	infinity	infinity
Parameter 23~IW(0.000,7)	infinity	infinity	infinity
Parameter 24~IW(0.000,7)	infinity	infinity	infinity
Parameter 25~IW(1.000,7)	infinity	infinity	infinity
Parameter 26~IW(0.000,7)	infinity	infinity	infinity
Parameter 27~IW(0.000,7)	infinity	infinity	infinity
Parameter 28~IW(0.000,7)	infinity	infinity	infinity
Parameter 29~IW(0.000,7)	infinity	infinity	infinity
Parameter 30~IW(0.000,7)	infinity	infinity	infinity
Parameter 31~IW(1.000,7)	infinity	infinity	infinity
Parameter 32~N(0.000,infinity)	0.0000	infinity	infinity
Parameter 33~N(0.000,infinity)	0.0000	infinity	infinity
Parameter 34~N(0.000,infinity)	0.0000	infinity	infinity
Parameter 35~N(0.000,infinity)	0.0000	infinity	infinity
Parameter 36~N(0.000,5.000)	0.0000	5.0000	2.2361
Parameter 37~N(0.000,5.000)	0.0000	5.0000	2.2361
Parameter 38~IG(-1.000,0.000)	infinity	infinity	infinity
Parameter 39~IG(-1.000,0.000)	infinity	infinity	infinity
Parameter 40~IW(1.000,3)	infinity	infinity	infinity

Parameter 41~IW(0.000,3)	infinity	infinity	infinity
Parameter 42~IW(1.000,3)	infinity	infinity	infinity
Parameter 43~N(0.000,5.000)	0.0000	5.0000	2.2361

TECHNICAL 3 OUTPUT

ESTIMATED COVARIANCE MATRIX FOR PARAMETER ESTIMATES

	1	2	3	4	5
1	0.107209D-03				
2	0.481784D-04	0.448455D-03			
3	0.100709D-04	0.981932D-04	0.328538D-03		
4	0.172122D-04	0.155880D-03	0.108475D-03	0.905672D-03	
5	-0.148606D-04	-0.173316D-03	-0.105667D-03	-0.163202D-03	0.769685D-03
6	-0.246250D-04	-0.583123D-04	-0.114938D-03	0.105960D-03	0.726549D-04
7	0.728297D-05	-0.201781D-04	0.525127D-05	0.125512D-04	-0.224439D-04
8	-0.118389D-04	-0.417046D-04	-0.363307D-04	-0.619638D-05	0.378865D-04
9	0.507386D-05	0.234915D-05	-0.132520D-04	-0.184399D-04	-0.584155D-05
10	0.173838D-04	-0.794631D-05	0.108694D-04	-0.196346D-04	-0.166923D-04
11	0.372751D-06	0.976516D-05	0.611027D-05	0.595784D-05	-0.165781D-05
12	-0.101292D-05	0.532336D-05	0.895190D-05	0.933964D-06	-0.349676D-05
13	0.739552D-06	-0.833337D-05	-0.168184D-04	-0.287376D-04	-0.123170D-04
14	0.860310D-06	-0.416622D-05	-0.977077D-06	-0.290966D-05	0.896393D-05
15	0.660334D-05	-0.322610D-05	-0.172177D-04	0.814313D-05	0.273574D-04
16	0.581673D-05	0.135322D-04	0.221030D-05	0.127441D-04	-0.129383D-04
17	0.107035D-05	0.401434D-05	0.435960D-05	-0.629355D-05	0.382600D-05
18	0.114049D-06	0.578067D-05	-0.568954D-06	0.225929D-04	0.254621D-05
19	0.104028D-04	-0.102818D-04	-0.504296D-06	-0.286143D-04	0.159677D-05
20	-0.538078D-05	0.164756D-04	0.244006D-05	0.189097D-04	-0.161336D-04
21	-0.456168D-05	-0.226269D-05	-0.109277D-05	-0.579775D-05	0.306374D-05
22	0.339405D-05	0.145404D-04	-0.103285D-04	-0.165244D-04	-0.986910D-07
23	0.414382D-05	-0.465331D-05	-0.114508D-04	-0.204905D-04	-0.209794D-04
24	0.364521D-05	0.160607D-04	0.314606D-05	0.931963D-05	-0.508145D-05
25	-0.444913D-05	0.291366D-04	-0.392097D-04	-0.228117D-04	0.319205D-04
26	0.663019D-05	-0.536309D-05	0.474709D-06	-0.762296D-06	0.810016D-05
27	0.406818D-05	-0.115209D-05	0.176404D-04	0.118437D-04	0.218727D-04
28	-0.186635D-04	0.961782D-05	-0.393902D-05	0.288366D-05	-0.106497D-04
29	0.126584D-04	0.311319D-05	0.992500D-05	-0.920145D-06	-0.651836D-05
30	0.638729D-05	-0.398164D-05	0.173235D-04	0.911180D-05	-0.278545D-04
31	-0.431655D-06	-0.109010D-04	-0.329541D-04	-0.242968D-04	-0.205207D-04
32	0.808655D-05	0.231288D-04	0.128490D-04	0.103454D-04	0.138164D-04
33	0.279981D-04	-0.268963D-05	-0.115518D-03	-0.308752D-04	0.473049D-04

34	-0.585715D-05	-0.888959D-04	-0.734716D-04	-0.149071D-03	0.665426D-04
35	0.659661D-05	0.495095D-05	-0.331727D-05	0.440874D-04	0.261012D-04
36	-0.642809D-05	-0.994968D-05	0.284212D-04	-0.888977D-05	-0.465474D-05
37	0.708155D-04	-0.217110D-04	-0.793798D-04	0.314050D-04	-0.725639D-04
38	-0.362430D-05	-0.433377D-06	-0.236366D-05	0.433086D-05	-0.204964D-06
39	-0.224132D-05	0.904801D-05	0.342020D-05	-0.129205D-04	-0.331201D-06
40	0.390782D-04	-0.952598D-04	-0.724099D-04	0.584510D-04	0.179657D-03
41	0.485118D-04	0.119776D-03	0.483211D-04	0.308092D-04	-0.278665D-04
42	0.801199D-05	0.727524D-05	-0.160365D-04	-0.184206D-04	0.550903D-05
43	0.204791D-03	-0.418712D-04	-0.203797D-03	0.606511D-04	-0.216364D-03

ESTIMATED COVARIANCE MATRIX FOR PARAMETER ESTIMATES

	6	7	8	9	10
6	0.135342D-01				
7	-0.704649D-03	0.270696D-02			
8	-0.124687D-03	-0.389147D-03	0.312258D-02		
9	0.122364D-04	-0.379476D-03	-0.377430D-03	0.120768D-02	
10	0.858695D-04	0.339852D-03	0.117252D-03	0.120706D-03	0.147073D-02
11	-0.187896D-04	-0.576997D-05	0.752288D-05	-0.183430D-05	-0.779293D-05
12	-0.498067D-04	-0.116808D-04	-0.184524D-04	-0.254768D-05	-0.127202D-05
13	-0.598514D-04	0.103936D-04	0.595598D-04	-0.215577D-04	-0.133927D-04
14	-0.267752D-04	-0.276994D-04	0.219272D-04	0.760248D-05	-0.128340D-04
15	0.855316D-04	0.116758D-04	-0.574658D-05	-0.571059D-05	0.112487D-04
16	0.585617D-04	0.664768D-04	-0.251818D-04	-0.365449D-05	0.332222D-04
17	-0.445957D-04	0.410569D-05	-0.903446D-05	-0.128240D-05	0.113278D-04
18	-0.123724D-03	0.312385D-04	0.217413D-05	-0.142151D-04	0.773894D-05
19	0.196666D-04	-0.324184D-04	0.245529D-04	-0.927215D-05	-0.221860D-04
20	-0.864462D-04	0.731684D-04	0.346541D-05	0.117557D-04	0.588600D-04
21	-0.270499D-04	0.265277D-05	0.200784D-04	0.446218D-05	0.243513D-04
22	-0.436100D-04	0.232457D-04	0.102088D-03	-0.265279D-04	0.514929D-04
23	-0.409955D-04	-0.114070D-03	0.123073D-03	0.107894D-04	-0.644603D-04
24	-0.439841D-04	0.871494D-05	-0.119408D-04	0.228674D-04	0.365618D-04
25	-0.447951D-04	0.793023D-04	-0.172290D-04	0.154795D-04	0.639486D-04
26	0.211335D-04	-0.137967D-04	-0.135071D-04	0.997360D-05	-0.871227D-06
27	0.105594D-03	0.648261D-06	-0.300453D-04	0.488191D-05	-0.881014D-05
28	0.402307D-05	-0.256032D-04	-0.876174D-04	0.106875D-04	-0.117549D-04
29	0.800861D-04	-0.769531D-04	0.143935D-04	0.405559D-05	-0.320529D-04
30	-0.902671D-04	-0.530973D-04	-0.114378D-04	-0.140690D-04	-0.625620D-04
31	0.288913D-04	0.561956D-04	0.132828D-04	0.136897D-04	0.286461D-04
32	-0.616641D-04	0.638660D-04	0.182154D-03	0.144379D-03	-0.126009D-03
33	0.345739D-03	-0.783070D-04	0.437655D-03	-0.241625D-03	0.118504D-03
34	-0.584619D-04	0.334716D-04	0.687595D-05	-0.388868D-04	0.715618D-05

35	-0.848904D-04	-0.242280D-04	0.948221D-04	-0.811382D-04	0.998746D-05
36	-0.448646D-04	0.699027D-04	-0.584726D-04	-0.373977D-04	0.101540D-04
37	0.484306D-03	-0.230516D-03	0.102310D-02	0.137136D-03	0.166511D-03
38	0.241544D-04	0.867641D-05	0.196090D-04	-0.810390D-06	-0.577973D-05
39	-0.146371D-04	0.187317D-04	-0.158219D-04	-0.154211D-04	0.266423D-04
40	0.511721D-03	0.547923D-03	-0.442356D-03	-0.323545D-03	0.186823D-03
41	-0.473960D-04	-0.545361D-04	0.759448D-05	0.826677D-04	0.929659D-04
42	-0.135565D-04	-0.209193D-04	0.489197D-04	-0.242172D-04	0.132169D-04
43	0.783401D-03	-0.785169D-03	0.295394D-02	0.402058D-03	0.449322D-03

ESTIMATED COVARIANCE MATRIX FOR PARAMETER ESTIMATES

	11	12	13	14	15
11	0.329616D-04				
12	0.152826D-04	0.780588D-04			
13	0.637452D-05	0.635527D-04	0.568279D-03		
14	-0.660644D-05	-0.157985D-04	-0.887928D-05	0.144750D-03	
15	-0.227648D-06	-0.228924D-04	-0.123309D-03	0.712067D-04	0.559775D-03
16	0.452714D-05	-0.643339D-06	0.256918D-04	-0.727118D-04	-0.263876D-03
17	0.404945D-05	0.151155D-04	0.268967D-05	-0.107912D-04	-0.461091D-05
18	0.356488D-05	0.161723D-04	0.121215D-03	-0.338084D-05	-0.677635D-04
19	0.201382D-05	-0.279928D-05	-0.250983D-04	0.135362D-04	0.122469D-03
20	0.774776D-06	0.166442D-05	0.122789D-04	-0.115650D-05	-0.208328D-04
21	0.267398D-05	0.224717D-04	0.136678D-04	-0.192285D-04	0.468223D-05
22	0.164340D-05	0.162571D-04	0.197980D-03	-0.131593D-04	-0.101369D-03
23	-0.611231D-05	-0.422293D-05	-0.187978D-04	0.321609D-04	0.165691D-03
24	-0.215561D-05	0.908141D-05	0.553501D-04	-0.925087D-05	-0.355091D-04
25	-0.296480D-05	0.551000D-05	0.440047D-04	-0.204783D-04	-0.316574D-04
26	-0.140465D-05	-0.238435D-04	-0.130105D-04	0.172339D-04	0.186036D-04
27	-0.213653D-05	-0.129999D-04	-0.189094D-03	0.893551D-05	0.997388D-04
28	0.749454D-06	-0.403178D-05	0.524495D-04	0.156474D-05	-0.200957D-03
29	-0.297864D-05	-0.252910D-05	-0.583498D-04	0.587752D-05	0.332130D-04
30	0.235211D-05	0.974259D-05	-0.518937D-04	0.239138D-04	0.434889D-04
31	0.503307D-05	0.124472D-04	0.920004D-04	-0.236334D-04	-0.605594D-04
32	0.149712D-05	-0.213913D-04	-0.253682D-04	0.162191D-05	-0.383187D-04
33	-0.240382D-04	-0.118433D-03	0.691690D-04	0.385455D-04	-0.133325D-03
34	0.679729D-05	-0.174857D-05	0.612979D-04	0.663151D-05	0.186265D-05
35	-0.414640D-05	0.345301D-05	0.574066D-06	0.329060D-05	-0.426450D-04
36	-0.172783D-05	-0.146121D-04	0.195101D-04	-0.443425D-05	-0.205791D-04
37	0.544264D-04	-0.667039D-06	0.102887D-03	0.198265D-03	0.928342D-04
38	-0.460991D-06	-0.109786D-05	0.449885D-05	-0.342118D-05	0.685672D-06
39	0.255552D-05	0.133943D-05	0.410378D-05	0.947717D-07	-0.561637D-05
40	-0.394842D-04	-0.599512D-04	-0.106344D-03	0.487974D-04	0.241197D-04

41	0.801409D-05	0.196873D-04	0.806241D-04	-0.373995D-04	-0.871523D-04
42	-0.156160D-08	0.428061D-06	-0.230712D-05	-0.242809D-05	0.265577D-04
43	0.154343D-03	-0.384774D-05	0.345551D-03	0.588842D-03	0.243035D-03

ESTIMATED COVARIANCE MATRIX FOR PARAMETER ESTIMATES

	16	17	18	19	20
16	0.207501D-02				
17	0.959521D-05	0.512387D-04			
18	0.188244D-04	0.268700D-04	0.230909D-03		
19	-0.177906D-03	-0.919043D-05	-0.562372D-04	0.432906D-03	
20	0.601987D-04	0.102266D-04	0.871336D-04	-0.886211D-04	0.336638D-03
21	-0.976541D-05	0.191112D-04	0.107179D-04	0.843326D-05	0.520606D-05
22	0.375819D-04	0.105666D-04	0.103605D-03	-0.244231D-04	0.257940D-04
23	-0.351845D-03	-0.563780D-05	-0.463441D-04	0.155280D-03	-0.343716D-04
24	0.280212D-05	0.718976D-05	0.838348D-04	-0.717622D-04	0.124419D-03
25	0.215556D-04	-0.243022D-05	0.548600D-04	-0.237630D-04	0.274958D-04
26	-0.167133D-04	-0.160737D-04	-0.612071D-05	-0.289267D-05	-0.104758D-05
27	-0.567728D-04	-0.101574D-04	-0.843614D-04	0.203969D-04	-0.264304D-04
28	0.299762D-03	0.198246D-05	0.294229D-04	-0.140967D-03	0.204184D-04
29	-0.118766D-04	-0.423795D-05	-0.894786D-04	0.731970D-04	-0.105051D-03
30	-0.643937D-04	-0.172319D-05	-0.534321D-04	0.618849D-05	-0.321289D-04
31	0.450658D-04	0.568253D-05	0.519024D-04	-0.436442D-04	0.435761D-04
32	0.707425D-04	-0.486863D-05	-0.320178D-05	0.948804D-05	0.143546D-05
33	0.678633D-04	-0.433743D-04	0.348307D-04	0.363197D-04	0.508689D-04
34	-0.121296D-03	0.730473D-05	0.263285D-04	0.428453D-04	0.194198D-04
35	0.704197D-04	-0.115217D-04	0.216170D-05	-0.394646D-04	0.243598D-04
36	0.416461D-04	-0.668629D-05	-0.143523D-04	-0.168486D-05	-0.360796D-04
37	0.899183D-04	0.570670D-05	-0.446195D-04	-0.196902D-03	0.342144D-04
38	-0.384410D-05	0.121933D-05	0.316411D-05	0.316851D-05	0.225628D-05
39	0.161630D-04	-0.475184D-06	-0.244311D-05	-0.361353D-05	-0.109721D-05
40	0.127852D-03	0.452331D-04	-0.112644D-05	0.944197D-04	-0.987181D-04
41	0.445187D-04	0.249306D-04	0.381387D-05	-0.384476D-04	0.245698D-04
42	-0.336896D-04	0.152569D-05	0.982356D-05	0.400400D-04	0.113461D-04
43	0.231833D-03	0.123337D-04	-0.968354D-04	-0.544850D-03	0.108168D-03

ESTIMATED COVARIANCE MATRIX FOR PARAMETER ESTIMATES

	21	22	23	24	25
21	0.139584D-03				
22	0.595384D-04	0.580028D-03			
23	-0.100478D-04	-0.134345D-03	0.100297D-02		

24	0.170085D-04	0.142176D-03	-0.111801D-03	0.416335D-03	
25	0.118378D-04	0.364318D-03	-0.238471D-03	0.290331D-03	0.207107D-02
26	-0.252559D-04	-0.150564D-04	0.855020D-05	-0.635510D-05	0.208298D-05
27	-0.133603D-04	-0.144001D-03	0.225069D-04	-0.500813D-04	-0.686723D-04
28	0.221560D-05	0.556676D-04	-0.205075D-03	0.464969D-04	0.182695D-04
29	-0.890830D-06	-0.543889D-04	0.269322D-04	-0.985064D-04	-0.610457D-04
30	-0.801334D-05	-0.232854D-03	0.138946D-03	-0.158838D-03	-0.454776D-03
31	0.930975D-05	0.767474D-04	-0.642401D-04	0.686801D-04	0.903586D-04
32	-0.189429D-04	-0.630550D-04	0.164946D-04	-0.253739D-04	-0.323920D-04
33	-0.216415D-04	0.119520D-03	-0.495208D-05	0.966816D-05	0.152492D-03
34	-0.216490D-05	-0.211505D-04	0.101291D-03	0.444861D-04	-0.852918D-04
35	-0.173754D-04	-0.548964D-05	0.703294D-05	0.435918D-05	0.308626D-04
36	-0.828670D-05	-0.468789D-06	-0.684852D-04	0.100322D-04	0.933890D-04
37	-0.129097D-03	0.178033D-03	-0.870762D-04	0.469947D-04	0.177359D-03
38	-0.369287D-06	-0.125175D-05	0.467739D-06	0.145667D-05	-0.261644D-05
39	0.687552D-06	-0.578178D-05	-0.461101D-05	-0.435890D-05	0.717841D-06
40	0.197713D-03	-0.130273D-03	-0.524937D-04	0.250934D-03	-0.297475D-03
41	-0.426503D-04	-0.645746D-05	-0.923057D-04	0.648275D-04	-0.670576D-04
42	0.188367D-04	0.199919D-04	0.347382D-04	0.199894D-04	0.519997D-04
43	-0.354724D-03	0.541342D-03	-0.239165D-03	0.171242D-03	0.448842D-03

ESTIMATED COVARIANCE MATRIX FOR PARAMETER ESTIMATES

	26	27	28	29	30
26	0.112211D-03				
27	0.497636D-04	0.453404D-03			
28	-0.891092D-05	-0.117145D-03	0.918918D-03		
29	0.536282D-05	0.109127D-03	-0.106594D-03	0.357021D-03	
30	-0.402850D-05	0.148654D-03	-0.155390D-03	0.138877D-03	0.909321D-03
31	-0.100907D-04	-0.275079D-03	0.218306D-03	-0.176430D-03	-0.302828D-03
32	0.289299D-04	0.114973D-04	-0.301689D-04	-0.155222D-04	0.173390D-04
33	0.848230D-04	0.405333D-05	0.732906D-04	0.817615D-04	0.929427D-04
34	-0.136064D-04	-0.124869D-04	-0.306277D-04	0.848951D-05	-0.939198D-05
35	0.175853D-04	0.250286D-04	-0.107671D-04	0.573768D-05	0.189781D-04
36	0.156510D-04	0.213026D-04	-0.343543D-04	0.274690D-04	-0.236385D-04
37	0.916756D-04	0.377463D-04	0.126284D-03	-0.116849D-04	0.555471D-04
38	-0.128836D-05	-0.189679D-05	0.401087D-05	-0.147202D-05	-0.178350D-05
39	-0.796066D-06	-0.409240D-05	0.875235D-05	0.274754D-05	0.107979D-04
40	0.970223D-05	0.585216D-04	-0.573204D-06	-0.253459D-04	-0.125857D-03
41	-0.333054D-04	0.195243D-04	0.521919D-04	0.345777D-04	-0.253758D-04
42	-0.354955D-05	0.696283D-06	-0.320873D-04	0.620021D-05	0.192452D-04
43	0.256529D-03	0.531092D-04	0.348542D-03	-0.479983D-04	0.170381D-03

ESTIMATED COVARIANCE MATRIX FOR PARAMETER ESTIMATES

	31	32	33	34	35
31	0.135451D-02				
32	0.388540D-04	0.159364D-02			
33	0.875266D-04	-0.271231D-04	0.236645D-01		
34	0.203129D-04	-0.359562D-04	-0.405694D-03	0.319540D-02	
35	-0.308046D-04	0.221803D-03	0.245890D-03	0.107531D-03	0.146111D-02
36	0.535240D-05	0.898447D-04	0.491114D-04	0.763104D-04	-0.160385D-04
37	0.433503D-03	-0.151358D-03	0.267184D-03	-0.287793D-03	-0.345256D-04
38	0.109452D-04	0.211997D-04	-0.133199D-04	0.112605D-04	-0.310184D-04
39	-0.438998D-05	-0.153958D-05	0.108672D-03	-0.108557D-04	-0.927174D-05
40	0.284030D-03	0.212869D-03	0.776519D-03	-0.674091D-03	-0.552820D-03
41	-0.341701D-05	-0.205988D-04	-0.309212D-03	0.321924D-03	0.315830D-04
42	-0.389249D-05	0.171266D-04	0.843666D-04	0.294449D-04	0.309940D-04
43	0.130339D-02	-0.404387D-03	0.463243D-03	-0.797025D-03	-0.659496D-04

ESTIMATED COVARIANCE MATRIX FOR PARAMETER ESTIMATES

	36	37	38	39	40
36	0.171700D-02				
37	-0.568098D-03	0.342390D-01			
38	0.490442D-05	0.153499D-04	0.754130D-04		
39	-0.114517D-04	-0.928219D-04	-0.121238D-05	0.113563D-03	
40	0.259024D-03	0.536000D-03	0.410287D-04	0.107893D-03	0.695601D-01
41	0.143487D-03	-0.471960D-03	-0.863710D-05	-0.634780D-05	-0.331357D-03
42	-0.605362D-05	0.531240D-04	0.386524D-05	0.412693D-05	0.178626D-03
43	-0.181170D-02	0.996540D-01	0.291825D-04	-0.293971D-03	0.146560D-02

ESTIMATED COVARIANCE MATRIX FOR PARAMETER ESTIMATES

	41	42	43
41	0.511833D-02		
42	0.810232D-04	0.555632D-03	
43	-0.132066D-02	0.152804D-03	0.291953D+00

ESTIMATED CORRELATION MATRIX FOR PARAMETER ESTIMATES

	1	2	3	4	5
1	1.000				

2	0.220	1.000			
3	0.054	0.256	1.000		
4	0.055	0.245	0.199	1.000	
5	-0.052	-0.295	-0.210	-0.195	1.000
6	-0.020	-0.024	-0.055	0.030	0.023
7	0.014	-0.018	0.006	0.008	-0.016
8	-0.020	-0.035	-0.036	-0.004	0.024
9	0.014	0.003	-0.021	-0.018	-0.006
10	0.044	-0.010	0.016	-0.017	-0.016
11	0.006	0.080	0.059	0.034	-0.010
12	-0.011	0.028	0.056	0.004	-0.014
13	0.003	-0.017	-0.039	-0.040	-0.019
14	0.007	-0.016	-0.004	-0.008	0.027
15	0.027	-0.006	-0.040	0.011	0.042
16	0.012	0.014	0.003	0.009	-0.010
17	0.014	0.026	0.034	-0.029	0.019
18	0.001	0.018	-0.002	0.049	0.006
19	0.048	-0.023	-0.001	-0.046	0.003
20	-0.028	0.042	0.007	0.034	-0.032
21	-0.037	-0.009	-0.005	-0.016	0.009
22	0.014	0.029	-0.024	-0.023	0.000
23	0.013	-0.007	-0.020	-0.021	-0.024
24	0.017	0.037	0.009	0.015	-0.009
25	-0.009	0.030	-0.048	-0.017	0.025
26	0.060	-0.024	0.002	-0.002	0.028
27	0.018	-0.003	0.046	0.018	0.037
28	-0.059	0.015	-0.007	0.003	-0.013
29	0.065	0.008	0.029	-0.002	-0.012
30	0.020	-0.006	0.032	0.010	-0.033
31	-0.001	-0.014	-0.049	-0.022	-0.020
32	0.020	0.027	0.018	0.009	0.012
33	0.018	-0.001	-0.041	-0.007	0.011
34	-0.010	-0.074	-0.072	-0.088	0.042
35	0.017	0.006	-0.005	0.038	0.025
36	-0.015	-0.011	0.038	-0.007	-0.004
37	0.037	-0.006	-0.024	0.006	-0.014
38	-0.040	-0.002	-0.015	0.017	-0.001
39	-0.020	0.040	0.018	-0.040	-0.001
40	0.014	-0.017	-0.015	0.007	0.025
41	0.065	0.079	0.037	0.014	-0.014
42	0.033	0.015	-0.038	-0.026	0.008
43	0.037	-0.004	-0.021	0.004	-0.014

ESTIMATED CORRELATION MATRIX FOR PARAMETER ESTIMATES

	6	7	8	9	10
6	1.000				
7	-0.116	1.000			
8	-0.019	-0.134	1.000		
9	0.003	-0.210	-0.194	1.000	
10	0.019	0.170	0.055	0.091	1.000
11	-0.028	-0.019	0.023	-0.009	-0.035
12	-0.048	-0.025	-0.037	-0.008	-0.004
13	-0.022	0.008	0.045	-0.026	-0.015
14	-0.019	-0.044	0.033	0.018	-0.028
15	0.031	0.009	-0.004	-0.007	0.012
16	0.011	0.028	-0.010	-0.002	0.019
17	-0.054	0.011	-0.023	-0.005	0.041
18	-0.070	0.040	0.003	-0.027	0.013
19	0.008	-0.030	0.021	-0.013	-0.028
20	-0.040	0.077	0.003	0.018	0.084
21	-0.020	0.004	0.030	0.011	0.054
22	-0.016	0.019	0.076	-0.032	0.056
23	-0.011	-0.069	0.070	0.010	-0.053
24	-0.019	0.008	-0.010	0.032	0.047
25	-0.008	0.033	-0.007	0.010	0.037
26	0.017	-0.025	-0.023	0.027	-0.002
27	0.043	0.001	-0.025	0.007	-0.011
28	0.001	-0.016	-0.052	0.010	-0.010
29	0.036	-0.078	0.014	0.006	-0.044
30	-0.026	-0.034	-0.007	-0.013	-0.054
31	0.007	0.029	0.006	0.011	0.020
32	-0.013	0.031	0.082	0.104	-0.082
33	0.019	-0.010	0.051	-0.045	0.020
34	-0.009	0.011	0.002	-0.020	0.003
35	-0.019	-0.012	0.044	-0.061	0.007
36	-0.009	0.032	-0.025	-0.026	0.006
37	0.022	-0.024	0.099	0.021	0.023
38	0.024	0.019	0.040	-0.003	-0.017
39	-0.012	0.034	-0.027	-0.042	0.065
40	0.017	0.040	-0.030	-0.035	0.018
41	-0.006	-0.015	0.002	0.033	0.034
42	-0.005	-0.017	0.037	-0.030	0.015
43	0.012	-0.028	0.098	0.021	0.022

ESTIMATED CORRELATION MATRIX FOR PARAMETER ESTIMATES

	11	12	13	14	15
11	1.000				
12	0.301	1.000			
13	0.047	0.302	1.000		
14	-0.096	-0.149	-0.031	1.000	
15	-0.002	-0.110	-0.219	0.250	1.000
16	0.017	-0.002	0.024	-0.133	-0.245
17	0.099	0.239	0.016	-0.125	-0.027
18	0.041	0.120	0.335	-0.018	-0.188
19	0.017	-0.015	-0.051	0.054	0.249
20	0.007	0.010	0.028	-0.005	-0.048
21	0.039	0.215	0.049	-0.135	0.017
22	0.012	0.076	0.345	-0.045	-0.178
23	-0.034	-0.015	-0.025	0.084	0.221
24	-0.018	0.050	0.114	-0.038	-0.074
25	-0.011	0.014	0.041	-0.037	-0.029
26	-0.023	-0.255	-0.052	0.135	0.074
27	-0.017	-0.069	-0.373	0.035	0.198
28	0.004	-0.015	0.073	0.004	-0.280
29	-0.027	-0.015	-0.130	0.026	0.074
30	0.014	0.037	-0.072	0.066	0.061
31	0.024	0.038	0.105	-0.053	-0.070
32	0.007	-0.061	-0.027	0.003	-0.041
33	-0.027	-0.087	0.019	0.021	-0.037
34	0.021	-0.004	0.045	0.010	0.001
35	-0.019	0.010	0.001	0.007	-0.047
36	-0.007	-0.040	0.020	-0.009	-0.021
37	0.051	0.000	0.023	0.089	0.021
38	-0.009	-0.014	0.022	-0.033	0.003
39	0.042	0.014	0.016	0.001	-0.022
40	-0.026	-0.026	-0.017	0.015	0.004
41	0.020	0.031	0.047	-0.043	-0.051
42	0.000	0.002	-0.004	-0.009	0.048
43	0.050	-0.001	0.027	0.091	0.019

ESTIMATED CORRELATION MATRIX FOR PARAMETER ESTIMATES

	16	17	18	19	20
16	1.000				
17	0.029	1.000			
18	0.027	0.247	1.000		
19	-0.188	-0.062	-0.178	1.000	

20	0.072	0.078	0.313	-0.232	1.000
21	-0.018	0.226	0.060	0.034	0.024
22	0.034	0.061	0.283	-0.049	0.058
23	-0.244	-0.025	-0.096	0.236	-0.059
24	0.003	0.049	0.270	-0.169	0.332
25	0.010	-0.007	0.079	-0.025	0.033
26	-0.035	-0.212	-0.038	-0.013	-0.005
27	-0.059	-0.067	-0.261	0.046	-0.068
28	0.217	0.009	0.064	-0.224	0.037
29	-0.014	-0.031	-0.312	0.186	-0.303
30	-0.047	-0.008	-0.117	0.010	-0.058
31	0.027	0.022	0.093	-0.057	0.065
32	0.039	-0.017	-0.005	0.011	0.002
33	0.010	-0.039	0.015	0.011	0.018
34	-0.047	0.018	0.031	0.036	0.019
35	0.040	-0.042	0.004	-0.050	0.035
36	0.022	-0.023	-0.023	-0.002	-0.047
37	0.011	0.004	-0.016	-0.051	0.010
38	-0.010	0.020	0.024	0.018	0.014
39	0.033	-0.006	-0.015	-0.016	-0.006
40	0.011	0.024	0.000	0.017	-0.020
41	0.014	0.049	0.004	-0.026	0.019
42	-0.031	0.009	0.027	0.082	0.026
43	0.009	0.003	-0.012	-0.048	0.011

ESTIMATED CORRELATION MATRIX FOR PARAMETER ESTIMATES

	21	22	23	24	25
21	1.000				
22	0.209	1.000			
23	-0.027	-0.176	1.000		
24	0.071	0.289	-0.173	1.000	
25	0.022	0.332	-0.165	0.313	1.000
26	-0.202	-0.059	0.025	-0.029	0.004
27	-0.053	-0.281	0.033	-0.115	-0.071
28	0.006	0.076	-0.214	0.075	0.013
29	-0.004	-0.120	0.045	-0.256	-0.071
30	-0.022	-0.321	0.145	-0.258	-0.331
31	0.021	0.087	-0.055	0.091	0.054
32	-0.040	-0.066	0.013	-0.031	-0.018
33	-0.012	0.032	-0.001	0.003	0.022
34	-0.003	-0.016	0.057	0.039	-0.033
35	-0.038	-0.006	0.006	0.006	0.018

36	-0.017	0.000	-0.052	0.012	0.050
37	-0.059	0.040	-0.015	0.012	0.021
38	-0.004	-0.006	0.002	0.008	-0.007
39	0.005	-0.023	-0.014	-0.020	0.001
40	0.063	-0.021	-0.006	0.047	-0.025
41	-0.050	-0.004	-0.041	0.044	-0.021
42	0.068	0.035	0.047	0.042	0.048
43	-0.056	0.042	-0.014	0.016	0.018

ESTIMATED CORRELATION MATRIX FOR PARAMETER ESTIMATES

	26	27	28	29	30
26	1.000				
27	0.221	1.000			
28	-0.028	-0.181	1.000		
29	0.027	0.271	-0.186	1.000	
30	-0.013	0.232	-0.170	0.244	1.000
31	-0.026	-0.351	0.196	-0.254	-0.273
32	0.068	0.014	-0.025	-0.021	0.014
33	0.052	0.001	0.016	0.028	0.020
34	-0.023	-0.010	-0.018	0.008	-0.006
35	0.043	0.031	-0.009	0.008	0.016
36	0.036	0.024	-0.027	0.035	-0.019
37	0.047	0.010	0.023	-0.003	0.010
38	-0.014	-0.010	0.015	-0.009	-0.007
39	-0.007	-0.018	0.027	0.014	0.034
40	0.003	0.010	0.000	-0.005	-0.016
41	-0.044	0.013	0.024	0.026	-0.012
42	-0.014	0.001	-0.045	0.014	0.027
43	0.045	0.005	0.021	-0.005	0.010

ESTIMATED CORRELATION MATRIX FOR PARAMETER ESTIMATES

	31	32	33	34	35
31	1.000				
32	0.026	1.000			
33	0.015	-0.004	1.000		
34	0.010	-0.016	-0.047	1.000	
35	-0.022	0.145	0.042	0.050	1.000
36	0.004	0.054	0.008	0.033	-0.010
37	0.064	-0.020	0.009	-0.028	-0.005
38	0.034	0.061	-0.010	0.023	-0.093

39	-0.011	-0.004	0.066	-0.018	-0.023
40	0.029	0.020	0.019	-0.045	-0.055
41	-0.001	-0.007	-0.028	0.080	0.012
42	-0.004	0.018	0.023	0.022	0.034
43	0.066	-0.019	0.006	-0.026	-0.003

ESTIMATED CORRELATION MATRIX FOR PARAMETER ESTIMATES

	36	37	38	39	40
36	1.000				
37	-0.074	1.000			
38	0.014	0.010	1.000		
39	-0.026	-0.047	-0.013	1.000	
40	0.024	0.011	0.018	0.038	1.000
41	0.048	-0.036	-0.014	-0.008	-0.018
42	-0.006	0.012	0.019	0.016	0.029
43	-0.081	0.997	0.006	-0.051	0.010

ESTIMATED CORRELATION MATRIX FOR PARAMETER ESTIMATES

	41	42	43
41	1.000		
42	0.048	1.000	
43	-0.034	0.012	1.000

TECHNICAL 8 OUTPUT

TECHNICAL 8 OUTPUT FOR BAYES ESTIMATION

CHAIN	BSEED
1	0
2	285380
3	253358

ITERATION	POTENTIAL SCALE REDUCTION	PARAMETER WITH HIGHEST PSR
100	1.014	38
200	1.006	6
300	1.010	43
400	1.015	35

500	1.004	35
600	1.003	18
700	1.002	5
800	1.003	39
900	1.003	37
1000	1.002	14

Improvement in physical health, bullying interaction

SUMMARY OF DATA

Number of clusters 57

Size (s)	Cluster ID with Size s
2	4 166
3	65 74 80 30
4	84 22
5	205
6	228
7	93
8	106 144 165 71 170 173 192 72 50
9	40 108
11	107 83 181 61 75 68
12	69 174 206 226 81
13	162
16	92 154
19	114
20	145 2
22	116
23	36 156 169 142
24	152
26	98
28	122 202
30	178
32	6
46	24
59	204
62	125
64	209
108	217
216	110
223	100

COVARIANCE COVERAGE OF DATA

Minimum covariance coverage value 0.100

Number of missing data patterns 4

PROPORTION OF DATA PRESENT

Covariance Coverage

	PHYCAT16	PRAFAC	PHY15	KES15	CHOICE15
PHYCAT16	1.000				
PRAFAC	1.000	1.000			
PHY15	1.000	1.000	1.000		
KES15	0.971	0.971	0.971	0.971	
CHOICE15	0.963	0.963	0.963	0.963	0.963
BULLY15	0.966	0.966	0.966	0.966	0.963
PRESS15	0.966	0.966	0.966	0.966	0.963
DUTIES15	0.966	0.966	0.966	0.966	0.963

Covariance Coverage

	BULLY15	PRESS15	DUTIES15
BULLY15	0.966		
PRESS15	0.966	0.966	
DUTIES15	0.966	0.966	0.966

UNIVARIATE PROPORTIONS AND COUNTS FOR CATEGORICAL VARIABLES

PHYCAT16		
Category 1	0.364	517.000
Category 2	0.636	905.000

UNIVARIATE SAMPLE STATISTICS

UNIVARIATE HIGHER-ORDER MOMENT DESCRIPTIVE STATISTICS

Variable/ Sample Size	Mean/ Variance	Skewness/ Kurtosis	Minimum/ Maximum	% with Min/Max	Percentiles 20%/60%	40%/80%	Median
PRAFAC	-0.173	0.219	-2.338	1.75%	-1.489	-0.520	-0.258
57.000	1.361	-0.662	2.338	1.75%	0.080	0.848	

```
     PHY15                  0.000       2.446      -0.155   85.58%      -0.155     -0.155     -0.155
           1422.000         0.154       5.391       1.845    1.13%      -0.155     -0.155
     KES15                  0.000       1.141      -0.953    8.62%      -0.620     -0.287     -0.120
           1381.000         0.613       1.071       3.047    0.36%       0.047      0.547
     CHOICE15               0.000      -0.138      -1.878   14.60%      -0.878      0.122      0.122
           1370.000         1.250      -0.758       2.122    5.77%       0.122      1.122
     BULLY15                1.312       2.479       1.000   79.02%       1.000      1.000      1.000
           1373.000         0.474       6.315       5.000    0.44%       1.000      2.000
     PRESS15                0.000       0.183      -1.756   14.06%      -0.756     -0.756      0.244
           1373.000         1.207      -0.514       2.244    7.21%       0.244      1.244
     DUTIES15               0.000      -0.896      -2.873    3.06%      -0.873      0.127      0.127
           1373.000         0.975       0.587       1.127   27.75%       0.127      1.127
```

THE MODEL ESTIMATION TERMINATED NORMALLY

USE THE FBITERATIONS OPTION TO INCREASE THE NUMBER OF ITERATIONS BY A FACTOR OF AT LEAST TWO TO CHECK CONVERGENCE AND THAT THE PSR VALUE DOES NOT INCREASE.

MODEL FIT INFORMATION

Number of Free Parameters   43

MODEL RESULTS

	Estimate	Posterior S.D.	One-Tailed P-Value	95% C.I. Lower 2.5%	95% C.I. Upper 2.5%	Significance
**Within Level**						
PHYCAT16 ON						
PHY15	0.833	0.117	0.000	0.612	1.063	*
CHOICE15	-0.048	0.034	0.077	-0.117	0.018	
DUTIES15	0.033	0.039	0.195	-0.040	0.111	
PRESS15	-0.002	0.034	0.469	-0.069	0.064	
KES15	0.165	0.052	0.000	0.060	0.262	*
KES15 WITH						
PHY15	0.071	0.009	0.000	0.054	0.089	*
CHOICE15	-0.136	0.024	0.000	-0.183	-0.090	*

```
   BULLY15            0.127      0.015      0.000      0.098      0.158      *
   PRESS15            0.206      0.024      0.000      0.161      0.255      *
   DUTIES15          -0.207      0.021      0.000     -0.247     -0.164      *

 PHY15    WITH
   CHOICE15          -0.032      0.012      0.002     -0.057     -0.010      *
   BULLY15            0.013      0.007      0.040     -0.002      0.026
   PRESS15            0.015      0.012      0.107     -0.008      0.037
   DUTIES15          -0.009      0.011      0.196     -0.029      0.012

 CHOICE15 WITH
   BULLY15           -0.121      0.021      0.000     -0.164     -0.082      *
   PRESS15           -0.183      0.032      0.000     -0.248     -0.122      *
   DUTIES15           0.162      0.030      0.000      0.105      0.223      *

 BULLY15  WITH
   PRESS15            0.174      0.020      0.000      0.132      0.213      *
   DUTIES15          -0.137      0.019      0.000     -0.174     -0.102      *

 PRESS15  WITH
   DUTIES15          -0.244      0.030      0.000     -0.305     -0.183      *

 Means
   PHY15              0.000      0.010      0.499     -0.019      0.021
   KES15              0.000      0.021      0.499     -0.040      0.043
   CHOICE15           0.001      0.029      0.484     -0.056      0.056
   PRESS15            0.000      0.031      0.490     -0.062      0.056
   DUTIES15           0.001      0.028      0.494     -0.054      0.053

 Variances
   PHY15              0.154      0.006      0.000      0.144      0.166      *
   KES15              0.615      0.024      0.000      0.569      0.660      *
   CHOICE15           1.252      0.048      0.000      1.167      1.351      *
   BULLY15            0.455      0.018      0.000      0.421      0.492      *
   PRESS15            1.208      0.046      0.000      1.119      1.301      *
   DUTIES15           0.977      0.037      0.000      0.908      1.052      *

Between Level

 S1         ON
   PRAFAC            -0.072      0.081      0.184     -0.249      0.078

 PHYCAT16   ON
   PRAFAC            -0.054      0.042      0.094     -0.135      0.032
```

BULLY15	0.206	0.291	0.241	-0.378	0.747	
PRAFAC WITH						
BULLY15	-0.003	0.054	0.477	-0.109	0.105	
Means						
PRAFAC	-0.172	0.154	0.138	-0.468	0.136	
BULLY15	1.302	0.044	0.000	1.214	1.391	*
Intercepts						
S1	0.046	0.081	0.264	-0.112	0.196	
Thresholds						
PHYCAT16$1	-0.127	0.382	0.356	-0.899	0.573	
Variances						
PRAFAC	1.367	0.263	0.000	0.993	1.993	*
BULLY15	0.066	0.019	0.000	0.039	0.112	*
Residual Variances						
PHYCAT16	0.007	0.010	0.000	0.000	0.037	*
S1	0.036	0.073	0.000	0.002	0.260	*

STANDARDIZED MODEL RESULTS

STDYX Standardization

	Estimate	Posterior S.D.	One-Tailed P-Value	95% C.I. Lower 2.5%	Upper 2.5%	Significance
Within-Level Standardized Estimates Averaged Over Clusters						
S1 | PHYCAT16 ON						
BULLY15	0.036	0.045	0.208	-0.053	0.125	
PHYCAT16 ON						
PHY15	0.301	0.039	0.000	0.222	0.375	*
CHOICE15	-0.049	0.034	0.068	-0.119	0.015	
DUTIES15	0.031	0.035	0.191	-0.037	0.100	
PRESS15	-0.002	0.035	0.483	-0.069	0.068	
KES15	0.118	0.037	0.000	0.043	0.192	*

```
 KES15    WITH
    PHY15              0.230      0.026      0.000      0.179      0.280      *
    CHOICE15          -0.154      0.026      0.000     -0.204     -0.102      *
    BULLY15            0.240      0.026      0.000      0.189      0.290      *
    PRESS15            0.240      0.025      0.000      0.191      0.290      *
    DUTIES15          -0.267      0.025      0.000     -0.313     -0.216      *

 PHY15    WITH
    CHOICE15          -0.074      0.027      0.003     -0.128     -0.023      *
    BULLY15            0.047      0.027      0.044     -0.006      0.098
    PRESS15            0.036      0.028      0.104     -0.019      0.088
    DUTIES15          -0.024      0.028      0.200     -0.077      0.032

 CHOICE15 WITH
    BULLY15           -0.160      0.027      0.000     -0.211     -0.107      *
    PRESS15           -0.149      0.026      0.000     -0.197     -0.095      *
    DUTIES15           0.146      0.026      0.000      0.093      0.196      *

 BULLY15  WITH
    PRESS15            0.234      0.026      0.000      0.182      0.284      *
    DUTIES15          -0.205      0.027      0.000     -0.254     -0.152      *

 PRESS15  WITH
    DUTIES15          -0.224      0.026      0.000     -0.274     -0.173      *

 Means
    PHY15              0.000      0.027      0.498     -0.051      0.054
    KES15              0.000      0.026      0.497     -0.052      0.051
    CHOICE15           0.000      0.026      0.493     -0.050      0.052
    PRESS15            0.000      0.028      0.492     -0.054      0.052
    DUTIES15           0.000      0.028      0.495     -0.056      0.054

 Variances
    PHY15              1.000      0.000      0.000      1.000      1.000
    KES15              1.000      0.000      0.000      1.000      1.000
    CHOICE15           1.000      0.000      0.000      1.000      1.000
    BULLY15            1.000      0.000      0.000      1.000      1.000
    PRESS15            1.000      0.000      0.000      1.000      1.000
    DUTIES15           1.000      0.000      0.000      1.000      1.000

Between Level

 S1         ON
    PRAFAC            -0.389      0.371      0.184     -0.924      0.421
```

PHYCAT16 ON						
PRAFAC	-0.476	0.312	0.094	-0.911	0.314	
BULLY15	0.427	0.453	0.241	-0.712	0.941	
PRAFAC WITH						
BULLY15	-0.009	0.169	0.477	-0.325	0.315	
Means						
PRAFAC	-0.145	0.131	0.138	-0.409	0.112	
BULLY15	5.076	0.700	0.000	3.835	6.644	*
Intercepts						
S1	0.202	0.452	0.264	-0.555	1.222	
Variances						
PRAFAC	1.000	0.000	0.000	1.000	1.000	
BULLY15	1.000	0.000	0.000	1.000	1.000	
Residual Variances						
PHYCAT16	0.393	0.268	0.000	0.016	0.957	*
S1	0.826	0.255	0.000	0.146	1.000	*

R-SQUARE

Within-Level R-Square Averaged Across Clusters

Variable	Estimate	Posterior S.D.	One-Tailed P-Value	95% C.I. Lower 2.5%	Upper 2.5%
PHYCAT16	0.159	0.032	0.000	0.103	0.231

Between Level

Variable	Estimate	Posterior S.D.	One-Tailed P-Value	95% C.I. Lower 2.5%	Upper 2.5%
PHYCAT16	0.607	0.268	0.000	0.043	0.984

Variable	Estimate	Posterior S.D.	One-Tailed P-Value	95% C.I. Lower 2.5%	Upper 2.5%
S1	0.174	0.255	0.000	0.000	0.854

TECHNICAL 1 OUTPUT

PARAMETER SPECIFICATION FOR WITHIN

TAU

PHYCAT16
0

NU

PHYCAT16	PHY15	KES15	CHOICE15	BULLY15
0	0	0	0	0

NU

PRESS15	DUTIES15
0	0

LAMBDA

	PHYCAT16	PHY15	KES15	CHOICE15	BULLY15
PHYCAT16	0	0	0	0	0
PHY15	0	0	0	0	0
KES15	0	0	0	0	0
CHOICE15	0	0	0	0	0
BULLY15	0	0	0	0	0
PRESS15	0	0	0	0	0
DUTIES15	0	0	0	0	0

LAMBDA

	PRESS15	DUTIES15
PHYCAT16	0	0
PHY15	0	0
KES15	0	0

CHOICE15	0	0
BULLY15	0	0
PRESS15	0	0
DUTIES15	0	0

THETA

	PHYCAT16	PHY15	KES15	CHOICE15	BULLY15
PHYCAT16	0				
PHY15	0	0			
KES15	0	0	0		
CHOICE15	0	0	0	0	
BULLY15	0	0	0	0	0
PRESS15	0	0	0	0	0
DUTIES15	0	0	0	0	0

THETA

	PRESS15	DUTIES15
PRESS15	0	
DUTIES15	0	0

ALPHA

PHYCAT16	PHY15	KES15	CHOICE15	BULLY15
0	1	2	3	0

ALPHA

PRESS15	DUTIES15
4	5

BETA

	PHYCAT16	PHY15	KES15	CHOICE15	BULLY15
PHYCAT16	0	6	7	8	0
PHY15	0	0	0	0	0
KES15	0	0	0	0	0
CHOICE15	0	0	0	0	0

BULLY15	0	0	0	0	0
PRESS15	0	0	0	0	0
DUTIES15	0	0	0	0	0

BETA

	PRESS15	DUTIES15
PHYCAT16	9	10
PHY15	0	0
KES15	0	0
CHOICE15	0	0
BULLY15	0	0
PRESS15	0	0
DUTIES15	0	0

PSI

	PHYCAT16	PHY15	KES15	CHOICE15	BULLY15
PHYCAT16	0				
PHY15	0	11			
KES15	0	12	13		
CHOICE15	0	14	15	16	
BULLY15	0	17	18	19	20
PRESS15	0	21	22	23	24
DUTIES15	0	26	27	28	29

PSI

	PRESS15	DUTIES15
PRESS15	25	
DUTIES15	30	31

PARAMETER SPECIFICATION FOR BETWEEN

TAU

PHYCAT16
43

NU

PHYCAT16	PRAFAC	BULLY15
0	0	0

LAMBDA

	S1	PHYCAT16	PRAFAC	BULLY15
PHYCAT16	0	0	0	0
PRAFAC	0	0	0	0
BULLY15	0	0	0	0

THETA

	PHYCAT16	PRAFAC	BULLY15
PHYCAT16	0		
PRAFAC	0	0	
BULLY15	0	0	0

ALPHA

S1	PHYCAT16	PRAFAC	BULLY15
32	0	33	34

BETA

	S1	PHYCAT16	PRAFAC	BULLY15
S1	0	0	35	0
PHYCAT16	0	0	36	37
PRAFAC	0	0	0	0
BULLY15	0	0	0	0

PSI

	S1	PHYCAT16	PRAFAC	BULLY15
S1	38			
PHYCAT16	0	39		
PRAFAC	0	0	40	

BULLY15	0	0	41	42

STARTING VALUES FOR WITHIN

TAU

PHYCAT16
0.000

NU

PHYCAT16	PHY15	KES15	CHOICE15	BULLY15
0.000	0.000	0.000	0.000	0.000

NU

PRESS15	DUTIES15
0.000	0.000

LAMBDA

	PHYCAT16	PHY15	KES15	CHOICE15	BULLY15
PHYCAT16	1.000	0.000	0.000	0.000	0.000
PHY15	0.000	1.000	0.000	0.000	0.000
KES15	0.000	0.000	1.000	0.000	0.000
CHOICE15	0.000	0.000	0.000	1.000	0.000
BULLY15	0.000	0.000	0.000	0.000	1.000
PRESS15	0.000	0.000	0.000	0.000	0.000
DUTIES15	0.000	0.000	0.000	0.000	0.000

LAMBDA

	PRESS15	DUTIES15
PHYCAT16	0.000	0.000
PHY15	0.000	0.000
KES15	0.000	0.000
CHOICE15	0.000	0.000
BULLY15	0.000	0.000

PRESS15	1.000	0.000
DUTIES15	0.000	1.000

THETA

	PHYCAT16	PHY15	KES15	CHOICE15	BULLY15
PHYCAT16	0.000				
PHY15	0.000	0.000			
KES15	0.000	0.000	0.000		
CHOICE15	0.000	0.000	0.000	0.000	
BULLY15	0.000	0.000	0.000	0.000	0.000
PRESS15	0.000	0.000	0.000	0.000	0.000
DUTIES15	0.000	0.000	0.000	0.000	0.000

THETA

	PRESS15	DUTIES15
PRESS15	0.000	
DUTIES15	0.000	0.000

ALPHA

PHYCAT16	PHY15	KES15	CHOICE15	BULLY15
0.000	0.000	0.000	0.000	0.000

ALPHA

PRESS15	DUTIES15
0.000	0.000

BETA

	PHYCAT16	PHY15	KES15	CHOICE15	BULLY15
PHYCAT16	0.000	0.000	0.000	0.000	0.000
PHY15	0.000	0.000	0.000	0.000	0.000
KES15	0.000	0.000	0.000	0.000	0.000
CHOICE15	0.000	0.000	0.000	0.000	0.000
BULLY15	0.000	0.000	0.000	0.000	0.000
PRESS15	0.000	0.000	0.000	0.000	0.000

DUTIES15	0.000	0.000	0.000	0.000	0.000

BETA

	PRESS15	DUTIES15
PHYCAT16	0.000	0.000
PHY15	0.000	0.000
KES15	0.000	0.000
CHOICE15	0.000	0.000
BULLY15	0.000	0.000
PRESS15	0.000	0.000
DUTIES15	0.000	0.000

PSI

	PHYCAT16	PHY15	KES15	CHOICE15	BULLY15
PHYCAT16	1.000				
PHY15	0.000	0.077			
KES15	0.000	0.000	0.306		
CHOICE15	0.000	0.000	0.000	0.625	
BULLY15	0.000	0.000	0.000	0.000	0.237
PRESS15	0.000	0.000	0.000	0.000	0.000
DUTIES15	0.000	0.000	0.000	0.000	0.000

PSI

	PRESS15	DUTIES15
PRESS15	0.604	
DUTIES15	0.000	0.488

STARTING VALUES FOR BETWEEN

TAU

PHYCAT16
-0.311

NU

PHYCAT16	PRAFAC	BULLY15
0.000	0.000	0.000

LAMBDA

	S1	PHYCAT16	PRAFAC	BULLY15
PHYCAT16	0.000	1.000	0.000	0.000
PRAFAC	0.000	0.000	1.000	0.000
BULLY15	0.000	0.000	0.000	1.000

THETA

	PHYCAT16	PRAFAC	BULLY15
PHYCAT16	0.000		
PRAFAC	0.000	0.000	
BULLY15	0.000	0.000	0.000

ALPHA

S1	PHYCAT16	PRAFAC	BULLY15
0.000	0.000	-0.078	1.312

BETA

	S1	PHYCAT16	PRAFAC	BULLY15
S1	0.000	0.000	0.000	0.000
PHYCAT16	0.000	0.000	0.000	0.000
PRAFAC	0.000	0.000	0.000	0.000
BULLY15	0.000	0.000	0.000	0.000

PSI

	S1	PHYCAT16	PRAFAC	BULLY15
S1	1.000			
PHYCAT16	0.000	1.000		
PRAFAC	0.000	0.000	0.480	
BULLY15	0.000	0.000	0.000	0.237

PRIORS FOR ALL PARAMETERS	PRIOR MEAN	PRIOR VARIANCE	PRIOR STD. DEV.
Parameter 1~N(0.000,infinity)	0.0000	infinity	infinity
Parameter 2~N(0.000,infinity)	0.0000	infinity	infinity
Parameter 3~N(0.000,infinity)	0.0000	infinity	infinity
Parameter 4~N(0.000,infinity)	0.0000	infinity	infinity
Parameter 5~N(0.000,infinity)	0.0000	infinity	infinity
Parameter 6~N(0.000,5.000)	0.0000	5.0000	2.2361
Parameter 7~N(0.000,5.000)	0.0000	5.0000	2.2361
Parameter 8~N(0.000,5.000)	0.0000	5.0000	2.2361
Parameter 9~N(0.000,5.000)	0.0000	5.0000	2.2361
Parameter 10~N(0.000,5.000)	0.0000	5.0000	2.2361
Parameter 11~IW(1.000,7)	infinity	infinity	infinity
Parameter 12~IW(0.000,7)	infinity	infinity	infinity
Parameter 13~IW(1.000,7)	infinity	infinity	infinity
Parameter 14~IW(0.000,7)	infinity	infinity	infinity
Parameter 15~IW(0.000,7)	infinity	infinity	infinity
Parameter 16~IW(1.000,7)	infinity	infinity	infinity
Parameter 17~IW(0.000,7)	infinity	infinity	infinity
Parameter 18~IW(0.000,7)	infinity	infinity	infinity
Parameter 19~IW(0.000,7)	infinity	infinity	infinity
Parameter 20~IW(1.000,7)	infinity	infinity	infinity
Parameter 21~IW(0.000,7)	infinity	infinity	infinity
Parameter 22~IW(0.000,7)	infinity	infinity	infinity
Parameter 23~IW(0.000,7)	infinity	infinity	infinity
Parameter 24~IW(0.000,7)	infinity	infinity	infinity
Parameter 25~IW(1.000,7)	infinity	infinity	infinity
Parameter 26~IW(0.000,7)	infinity	infinity	infinity
Parameter 27~IW(0.000,7)	infinity	infinity	infinity
Parameter 28~IW(0.000,7)	infinity	infinity	infinity
Parameter 29~IW(0.000,7)	infinity	infinity	infinity
Parameter 30~IW(0.000,7)	infinity	infinity	infinity
Parameter 31~IW(1.000,7)	infinity	infinity	infinity
Parameter 32~N(0.000,infinity)	0.0000	infinity	infinity
Parameter 33~N(0.000,infinity)	0.0000	infinity	infinity
Parameter 34~N(0.000,infinity)	0.0000	infinity	infinity
Parameter 35~N(0.000,infinity)	0.0000	infinity	infinity
Parameter 36~N(0.000,5.000)	0.0000	5.0000	2.2361
Parameter 37~N(0.000,5.000)	0.0000	5.0000	2.2361
Parameter 38~IG(-1.000,0.000)	infinity	infinity	infinity
Parameter 39~IG(-1.000,0.000)	infinity	infinity	infinity
Parameter 40~IW(1.000,3)	infinity	infinity	infinity

Parameter 41~IW(0.000,3)	infinity	infinity	infinity
Parameter 42~IW(1.000,3)	infinity	infinity	infinity
Parameter 43~N(0.000,5.000)	0.0000	5.0000	2.2361

TECHNICAL 3 OUTPUT

ESTIMATED COVARIANCE MATRIX FOR PARAMETER ESTIMATES

	1	2	3	4	5
1	0.107548D-03				
2	0.491959D-04	0.437295D-03			
3	-0.194035D-04	-0.852640D-04	0.857230D-03		
4	0.188036D-04	0.152715D-03	-0.159373D-03	0.937210D-03	
5	-0.154742D-04	-0.163508D-03	0.122111D-03	-0.172014D-03	0.764130D-03
6	-0.264618D-04	-0.687516D-04	-0.109944D-03	0.124369D-03	0.502650D-04
7	0.462491D-05	-0.391047D-04	0.542483D-05	-0.143136D-05	-0.115088D-04
8	-0.195029D-05	-0.312742D-04	-0.118476D-04	0.146558D-04	-0.263391D-05
9	0.272415D-05	-0.213298D-05	-0.299765D-04	-0.335073D-05	-0.220156D-04
10	0.181054D-04	0.281770D-05	0.251800D-04	-0.293131D-04	-0.925452D-05
11	0.561202D-06	0.107932D-04	0.367876D-05	0.517403D-05	-0.139406D-05
12	-0.738817D-06	0.696238D-05	0.121683D-04	-0.395093D-06	-0.255315D-05
13	0.185072D-05	-0.183408D-05	-0.255334D-04	-0.128123D-04	-0.239451D-04
14	0.928022D-06	-0.397904D-05	0.575130D-06	0.110056D-06	0.621424D-05
15	0.675262D-05	-0.426756D-05	-0.218368D-04	0.147931D-04	0.206165D-04
16	0.881675D-05	0.101493D-04	-0.155930D-04	0.949441D-05	-0.588454D-05
17	0.691918D-06	0.362886D-05	0.277532D-05	-0.798417D-05	0.509288D-05
18	-0.254743D-06	0.472596D-05	-0.932609D-05	0.256934D-04	-0.101506D-05
19	0.108853D-04	-0.213033D-05	-0.529651D-06	-0.101979D-04	-0.660835D-05
20	-0.729335D-05	0.903429D-05	0.210971D-05	0.110255D-04	-0.127290D-04
21	-0.439539D-05	-0.986589D-06	-0.862532D-06	-0.408918D-05	0.115502D-05
22	0.300201D-05	0.118492D-04	-0.214092D-04	-0.134815D-04	-0.350216D-05
23	0.516340D-05	0.350877D-05	-0.195582D-04	-0.476725D-05	-0.361558D-04
24	0.445796D-05	0.146119D-04	-0.753298D-05	0.545995D-05	-0.268718D-05
25	-0.554141D-05	0.224479D-04	-0.668818D-04	-0.128935D-04	0.252629D-04
26	0.668512D-05	-0.500488D-05	0.191230D-05	-0.143428D-05	0.874758D-05
27	0.487549D-05	0.231022D-05	0.271208D-04	0.939204D-05	0.241983D-04
28	-0.179880D-04	0.134233D-04	-0.147935D-04	0.147287D-04	-0.135460D-04
29	0.134122D-04	0.115827D-04	0.101116D-04	-0.108509D-05	-0.104288D-04
30	0.759836D-05	0.314050D-05	0.281173D-04	0.102704D-04	-0.285959D-04
31	-0.902040D-06	-0.138280D-04	-0.585251D-04	-0.142051D-04	-0.273390D-04
32	0.175344D-04	0.325432D-04	0.633720D-04	0.692813D-05	0.213803D-04
33	0.216272D-04	-0.290312D-04	-0.106140D-03	-0.210226D-04	0.495825D-04

34	0.195919D-04	0.931411D-04	-0.682404D-04	0.865751D-04	-0.155973D-03
35	0.335660D-05	-0.142683D-04	0.265276D-04	0.106989D-03	0.457542D-04
36	-0.828614D-05	0.683045D-06	0.730568D-04	-0.371032D-04	0.467150D-05
37	0.105353D-03	-0.449964D-05	-0.907743D-04	-0.279243D-04	-0.134671D-03
38	-0.165041D-04	0.482295D-04	-0.471831D-04	0.403693D-04	-0.450977D-04
39	-0.511870D-05	0.376174D-06	0.901402D-06	-0.200245D-04	0.610706D-05
40	0.173634D-04	-0.172313D-03	-0.747028D-04	0.530197D-05	0.198272D-03
41	0.224830D-04	0.820721D-04	0.332040D-04	0.335485D-04	-0.307866D-04
42	0.998210D-05	0.785313D-05	-0.173516D-04	-0.731257D-05	-0.610272D-06
43	0.148709D-03	0.334343D-04	-0.119613D-03	-0.292843D-04	-0.182036D-03

ESTIMATED COVARIANCE MATRIX FOR PARAMETER ESTIMATES

	6	7	8	9	10
6	0.136651D-01				
7	-0.709714D-03	0.273423D-02			
8	0.336592D-04	0.130035D-03	0.113630D-02		
9	0.263060D-04	-0.373380D-03	0.511764D-04	0.116644D-02	
10	0.696984D-04	0.347499D-03	-0.132258D-03	0.128811D-03	0.151775D-02
11	-0.226821D-04	-0.713467D-05	0.815635D-05	-0.118935D-05	-0.966799D-05
12	-0.536048D-04	-0.697874D-05	-0.564233D-05	-0.237093D-05	0.410027D-06
13	-0.730559D-04	0.191602D-04	0.560507D-04	-0.846961D-05	-0.254331D-04
14	-0.292523D-04	-0.254978D-04	-0.742982D-05	0.881332D-05	-0.180737D-04
15	0.947382D-04	0.223700D-04	-0.678414D-05	0.379844D-05	0.145894D-05
16	0.406496D-04	0.343958D-04	-0.522912D-04	-0.197420D-04	0.568021D-04
17	-0.461943D-04	0.257836D-05	-0.753278D-05	-0.347204D-05	0.110820D-04
18	-0.122857D-03	0.267231D-04	0.250556D-04	-0.113506D-04	0.244425D-05
19	0.496307D-04	-0.312058D-04	0.169172D-04	-0.154831D-04	-0.238833D-04
20	-0.107628D-03	0.691514D-04	0.139792D-04	0.147709D-04	0.547579D-04
21	-0.304652D-04	0.863675D-05	0.154137D-04	0.123298D-04	0.143027D-04
22	-0.378806D-04	0.359611D-04	0.624086D-04	-0.357353D-05	0.282194D-04
23	-0.167395D-04	-0.989650D-04	0.275514D-04	0.237351D-04	-0.841989D-04
24	-0.690232D-04	0.108637D-04	-0.642304D-05	0.297585D-04	0.312213D-04
25	-0.464884D-04	0.677674D-04	-0.160635D-04	0.222652D-05	0.729861D-04
26	0.243128D-04	-0.158149D-04	-0.159932D-04	0.398174D-05	0.817821D-05
27	0.120720D-03	0.669145D-05	-0.185288D-04	0.528176D-05	-0.347425D-05
28	0.205305D-04	-0.211042D-04	-0.273619D-04	0.712650D-05	-0.404951D-05
29	0.936269D-04	-0.704843D-04	-0.674298D-06	0.781515D-05	-0.349290D-04
30	-0.904767D-04	-0.533227D-04	0.214629D-04	-0.101784D-04	-0.618816D-04
31	0.225261D-04	0.400150D-04	0.180967D-04	-0.301877D-05	0.273542D-04
32	-0.109181D-03	-0.521949D-03	0.949442D-04	-0.284740D-03	0.173628D-03
33	0.487033D-03	-0.712057D-04	0.122925D-03	-0.197142D-03	0.100368D-03
34	-0.132319D-03	0.572131D-04	0.104296D-04	-0.125294D-04	0.438015D-04

35	-0.134757D-03	0.278469D-04	0.800061D-04	-0.645375D-04	0.189774D-03
36	-0.354648D-04	-0.938122D-05	-0.500196D-04	-0.603796D-04	0.335578D-05
37	-0.567121D-03	0.822429D-04	0.476188D-03	-0.272371D-03	0.247801D-03
38	0.527361D-03	-0.325101D-04	-0.352940D-04	-0.592768D-04	-0.196685D-03
39	-0.359237D-05	0.121198D-04	-0.499575D-05	-0.103831D-04	0.202020D-04
40	0.544912D-03	0.508916D-03	-0.156562D-03	-0.492311D-03	0.198957D-03
41	0.112274D-04	-0.127672D-03	-0.729699D-04	0.752290D-04	0.382043D-04
42	-0.439194D-04	-0.117594D-04	0.362265D-04	-0.172930D-04	-0.193381D-04
43	-0.147164D-02	-0.272083D-04	0.686152D-03	-0.398301D-03	0.292953D-03

ESTIMATED COVARIANCE MATRIX FOR PARAMETER ESTIMATES

	11	12	13	14	15
11	0.329411D-04				
12	0.152678D-04	0.781073D-04			
13	0.643916D-05	0.637625D-04	0.568276D-03		
14	-0.706396D-05	-0.162379D-04	-0.825103D-05	0.146785D-03	
15	-0.566657D-06	-0.234091D-04	-0.126878D-03	0.703455D-04	0.568875D-03
16	0.485757D-05	-0.560922D-06	0.237707D-04	-0.804281D-04	-0.269144D-03
17	0.387867D-05	0.152477D-04	0.296431D-05	-0.111391D-04	-0.595256D-05
18	0.389880D-05	0.159262D-04	0.121521D-03	-0.326027D-05	-0.698706D-04
19	0.150989D-05	-0.479698D-05	-0.271461D-04	0.172996D-04	0.124276D-03
20	0.149261D-05	0.199145D-05	0.149377D-04	-0.313461D-06	-0.222242D-04
21	0.270068D-05	0.224565D-04	0.138735D-04	-0.206239D-04	0.480368D-05
22	0.170432D-05	0.163385D-04	0.196979D-03	-0.135022D-04	-0.104656D-03
23	-0.560131D-05	-0.350256D-05	-0.190470D-04	0.362223D-04	0.173109D-03
24	-0.214196D-05	0.837414D-05	0.533066D-04	-0.980744D-05	-0.363662D-04
25	-0.279549D-05	0.572712D-05	0.433135D-04	-0.209872D-04	-0.329668D-04
26	-0.141241D-05	-0.239108D-04	-0.132185D-04	0.173101D-04	0.194182D-04
27	-0.218669D-05	-0.131208D-04	-0.189016D-03	0.910220D-05	0.100043D-03
28	0.120954D-05	-0.464981D-05	0.557195D-04	0.886462D-06	-0.201613D-03
29	-0.313466D-05	-0.229445D-05	-0.591703D-04	0.456090D-05	0.332011D-04
30	0.219207D-05	0.970336D-05	-0.513173D-04	0.227659D-04	0.404729D-04
31	0.519551D-05	0.125889D-04	0.925045D-04	-0.234775D-04	-0.563566D-04
32	-0.341478D-05	-0.420482D-04	-0.102042D-04	0.953026D-05	-0.811584D-04
33	-0.239886D-04	-0.118237D-03	0.715498D-04	0.508340D-04	-0.122432D-03
34	0.288933D-05	0.333697D-05	0.355473D-04	-0.309284D-05	-0.378325D-04
35	-0.116995D-04	0.779810D-05	-0.416409D-05	-0.100820D-04	-0.817519D-04
36	-0.407451D-06	-0.191956D-04	-0.324399D-05	0.290981D-05	-0.184543D-05
37	0.610674D-04	0.255425D-05	0.142657D-03	0.668666D-04	0.241064D-04
38	-0.331312D-05	-0.187042D-04	0.260792D-04	-0.158954D-04	0.191815D-04
39	0.106929D-05	0.115773D-05	0.177416D-05	-0.460310D-05	-0.397841D-05
40	-0.353777D-04	-0.544191D-04	-0.727389D-04	0.473473D-04	0.361030D-04

41	0.650112D-05	0.199064D-04	0.713178D-04	-0.237361D-04	-0.747861D-04
42	-0.111916D-05	-0.434201D-05	-0.579534D-05	-0.180318D-05	0.118198D-04
43	0.764839D-04	0.220394D-05	0.237021D-03	0.966191D-04	-0.329015D-04

ESTIMATED COVARIANCE MATRIX FOR PARAMETER ESTIMATES

	16	17	18	19	20
16	0.225659D-02				
17	0.120415D-04	0.505294D-04			
18	0.226909D-04	0.275272D-04	0.228383D-03		
19	-0.192695D-03	-0.126287D-04	-0.648133D-04	0.443823D-03	
20	0.591094D-04	0.103395D-04	0.881330D-04	-0.100350D-03	0.327306D-03
21	-0.956628D-05	0.180168D-04	0.943630D-05	0.751420D-05	0.348426D-05
22	0.418671D-04	0.100875D-04	0.995340D-04	-0.236335D-04	0.252030D-04
23	-0.387776D-03	-0.465955D-05	-0.498555D-04	0.142847D-03	-0.306034D-04
24	0.131228D-04	0.736829D-05	0.834911D-04	-0.862031D-04	0.124003D-03
25	0.165883D-04	-0.216380D-05	0.551332D-04	-0.175755D-04	0.288564D-04
26	-0.195185D-04	-0.156035D-04	-0.590205D-05	-0.475692D-06	-0.976464D-07
27	-0.541868D-04	-0.916378D-05	-0.820913D-04	0.157298D-04	-0.259647D-04
28	0.296535D-03	0.292748D-05	0.306691D-04	-0.140272D-03	0.207341D-04
29	-0.141076D-04	-0.403943D-05	-0.902779D-04	0.796000D-04	-0.103882D-03
30	-0.702389D-04	0.389693D-07	-0.498450D-04	0.578005D-05	-0.295826D-04
31	0.313731D-04	0.564815D-05	0.489048D-04	-0.370822D-04	0.414745D-04
32	0.111376D-03	-0.173318D-04	-0.197303D-04	0.542479D-04	-0.120455D-04
33	0.458423D-04	-0.471820D-04	0.210234D-04	0.479040D-04	0.419613D-04
34	-0.657362D-04	0.981132D-05	0.330466D-04	-0.407073D-04	0.417753D-04
35	0.210035D-03	-0.135507D-04	-0.254920D-05	-0.835653D-04	0.302275D-04
36	0.389339D-04	-0.568778D-05	-0.203882D-04	0.157561D-04	-0.412081D-04
37	0.349538D-03	0.272070D-04	0.952497D-04	-0.163010D-03	-0.395423D-04
38	-0.370323D-04	-0.753803D-05	0.733955D-05	0.699277D-04	-0.440143D-05
39	0.184810D-04	-0.387183D-06	-0.141617D-05	-0.573682D-05	0.314182D-05
40	0.139149D-03	0.418350D-04	-0.262788D-05	0.904797D-04	-0.504428D-04
41	0.863169D-04	0.155849D-04	-0.127715D-05	-0.388300D-04	0.436118D-04
42	-0.338214D-04	-0.779013D-06	0.216219D-05	0.274088D-04	-0.185063D-04
43	0.488241D-03	0.320532D-04	0.150060D-03	-0.193528D-03	-0.374771D-04

ESTIMATED COVARIANCE MATRIX FOR PARAMETER ESTIMATES

	21	22	23	24	25
21	0.139645D-03				
22	0.597305D-04	0.579175D-03			
23	-0.117538D-04	-0.141118D-03	0.100464D-02		

24	0.162464D-04	0.141725D-03	-0.103766D-03	0.411999D-03	
25	0.121717D-04	0.363755D-03	-0.254583D-03	0.281236D-03	0.206964D-02
26	-0.253327D-04	-0.150085D-04	0.126408D-04	-0.754414D-05	0.195699D-05
27	-0.134154D-04	-0.143549D-03	0.275938D-04	-0.516031D-04	-0.680450D-04
28	0.181607D-06	0.558778D-04	-0.208657D-03	0.452551D-04	0.214111D-04
29	0.232161D-06	-0.516470D-04	0.318481D-04	-0.986040D-04	-0.621090D-04
30	-0.797502D-05	-0.232315D-03	0.138652D-03	-0.152747D-03	-0.452753D-03
31	0.932099D-05	0.769292D-04	-0.638609D-04	0.660426D-04	0.898115D-04
32	-0.500714D-04	-0.833922D-04	0.295233D-04	-0.719704D-04	-0.286159D-04
33	-0.211314D-04	0.121124D-03	0.164922D-05	0.135603D-04	0.160244D-03
34	-0.460364D-05	-0.136600D-04	0.412829D-04	0.664234D-04	-0.106255D-04
35	-0.469343D-04	-0.104754D-04	0.531110D-04	-0.784126D-05	0.443949D-04
36	-0.138519D-04	-0.169913D-04	-0.305890D-04	0.268472D-05	0.642535D-04
37	0.936258D-06	0.192371D-03	-0.481264D-03	0.344162D-04	0.369085D-03
38	-0.475869D-05	-0.150482D-04	-0.778097D-05	-0.159377D-04	0.546826D-04
39	0.334858D-05	-0.319034D-05	-0.102592D-04	-0.468591D-05	0.260137D-05
40	0.198481D-03	-0.955250D-04	-0.960295D-04	0.228178D-03	-0.325221D-03
41	-0.391478D-04	-0.127631D-04	-0.413190D-04	0.686514D-04	-0.266357D-04
42	0.134575D-04	0.129365D-04	0.345115D-05	0.771462D-05	0.159557D-04
43	0.138052D-04	0.272577D-03	-0.626831D-03	0.623996D-04	0.415276D-03

ESTIMATED COVARIANCE MATRIX FOR PARAMETER ESTIMATES

	26	27	28	29	30
26	0.112322D-03				
27	0.498985D-04	0.453296D-03			
28	-0.915363D-05	-0.116288D-03	0.928548D-03		
29	0.376847D-05	0.104988D-03	-0.103659D-03	0.349220D-03	
30	-0.420480D-05	0.148281D-03	-0.164459D-03	0.133430D-03	0.907669D-03
31	-0.101038D-04	-0.275239D-03	0.205343D-03	-0.164589D-03	-0.302191D-03
32	0.543243D-04	0.880961D-05	-0.756533D-04	-0.461055D-04	0.364946D-04
33	0.851384D-04	0.335380D-05	0.877555D-04	0.761959D-04	0.910124D-04
34	-0.145391D-04	-0.194703D-04	0.425559D-04	-0.114260D-04	-0.881705D-05
35	0.324497D-04	0.596321D-04	0.117531D-04	0.203981D-04	0.409729D-04
36	0.241896D-04	0.540657D-04	-0.396232D-04	0.122158D-04	-0.292975D-04
37	0.901946D-04	0.590537D-04	-0.877612D-04	0.332895D-03	0.301124D-03
38	-0.171708D-05	0.219581D-04	0.563354D-04	-0.344264D-04	-0.530233D-04
39	-0.297348D-05	-0.465135D-05	0.907066D-05	-0.265953D-06	0.983253D-05
40	0.223322D-04	0.146492D-04	-0.186015D-04	0.107454D-04	-0.145653D-03
41	-0.186863D-04	0.148997D-04	0.719264D-04	-0.100172D-05	-0.154186D-04
42	-0.199956D-05	-0.857111D-05	-0.248588D-04	0.206509D-04	0.176729D-04
43	0.104044D-03	0.192765D-04	-0.120180D-03	0.441755D-03	0.411125D-03

ESTIMATED COVARIANCE MATRIX FOR PARAMETER ESTIMATES

	31	32	33	34	35
31	0.135329D-02				
32	0.484458D-04	0.653931D-02			
33	0.884637D-04	0.229042D-03	0.236794D-01		
34	0.624442D-04	-0.188259D-03	-0.433970D-03	0.191707D-02	
35	-0.374543D-04	0.717788D-03	0.416260D-03	0.208521D-03	0.651213D-02
36	-0.281727D-04	0.151951D-03	0.764348D-04	-0.216146D-04	0.390616D-03
37	0.593730D-04	0.934304D-04	0.564940D-03	-0.142283D-03	-0.856317D-04
38	0.986463D-04	0.103325D-02	0.340855D-03	-0.977651D-04	-0.542085D-03
39	-0.868882D-05	0.279545D-04	0.103729D-03	-0.937648D-05	-0.391317D-04
40	0.257904D-03	0.400564D-03	0.673735D-03	-0.768421D-03	-0.506373D-03
41	-0.107400D-05	0.643463D-04	-0.310730D-03	0.202966D-03	-0.691375D-04
42	0.692587D-05	0.566660D-05	0.609202D-04	-0.160526D-04	0.188282D-04
43	0.999373D-04	0.316232D-04	0.440778D-03	-0.573867D-04	0.276770D-03

ESTIMATED COVARIANCE MATRIX FOR PARAMETER ESTIMATES

	36	37	38	39	40
36	0.172715D-02				
37	-0.109689D-02	0.845602D-01			
38	-0.818238D-04	0.142344D-02	0.525583D-02		
39	-0.165694D-04	-0.189101D-03	0.303048D-04	0.107342D-03	
40	0.247618D-03	0.261838D-02	0.258062D-03	0.273508D-04	0.690670D-01
41	-0.607874D-05	-0.794496D-03	0.602307D-04	0.132591D-04	-0.187694D-03
42	-0.671244D-06	-0.647816D-04	0.473195D-04	0.632303D-05	0.582101D-04
43	-0.153167D-02	0.110362D+00	0.149938D-02	-0.284431D-03	0.326365D-02

ESTIMATED COVARIANCE MATRIX FOR PARAMETER ESTIMATES

	41	42	43
41	0.293407D-02		
42	-0.473778D-04	0.345830D-03	
43	-0.992657D-03	-0.652643D-04	0.145981D+00

ESTIMATED CORRELATION MATRIX FOR PARAMETER ESTIMATES

	1	2	3	4	5
1	1.000				

2	0.227	1.000			
3	-0.064	-0.139	1.000		
4	0.059	0.239	-0.178	1.000	
5	-0.054	-0.283	0.151	-0.203	1.000
6	-0.022	-0.028	-0.032	0.035	0.016
7	0.009	-0.036	0.004	-0.001	-0.008
8	-0.006	-0.044	-0.012	0.014	-0.003
9	0.008	-0.003	-0.030	-0.003	-0.023
10	0.045	0.003	0.022	-0.025	-0.009
11	0.009	0.090	0.022	0.029	-0.009
12	-0.008	0.038	0.047	-0.001	-0.010
13	0.007	-0.004	-0.037	-0.018	-0.036
14	0.007	-0.016	0.002	0.000	0.019
15	0.027	-0.009	-0.031	0.020	0.031
16	0.018	0.010	-0.011	0.007	-0.004
17	0.009	0.024	0.013	-0.037	0.026
18	-0.002	0.015	-0.021	0.056	-0.002
19	0.050	-0.005	-0.001	-0.016	-0.011
20	-0.039	0.024	0.004	0.020	-0.025
21	-0.036	-0.004	-0.002	-0.011	0.004
22	0.012	0.024	-0.030	-0.018	-0.005
23	0.016	0.005	-0.021	-0.005	-0.041
24	0.021	0.034	-0.013	0.009	-0.005
25	-0.012	0.024	-0.050	-0.009	0.020
26	0.061	-0.023	0.006	-0.004	0.030
27	0.022	0.005	0.044	0.014	0.041
28	-0.057	0.021	-0.017	0.016	-0.016
29	0.069	0.030	0.018	-0.002	-0.020
30	0.024	0.005	0.032	0.011	-0.034
31	-0.002	-0.018	-0.054	-0.013	-0.027
32	0.021	0.019	0.027	0.003	0.010
33	0.014	-0.009	-0.024	-0.004	0.012
34	0.043	0.102	-0.053	0.065	-0.129
35	0.004	-0.008	0.011	0.043	0.021
36	-0.019	0.001	0.060	-0.029	0.004
37	0.035	-0.001	-0.011	-0.003	-0.017
38	-0.022	0.032	-0.022	0.018	-0.023
39	-0.048	0.002	0.003	-0.063	0.021
40	0.006	-0.031	-0.010	0.001	0.027
41	0.040	0.072	0.021	0.020	-0.021
42	0.052	0.020	-0.032	-0.013	-0.001
43	0.038	0.004	-0.011	-0.003	-0.017

ESTIMATED CORRELATION MATRIX FOR PARAMETER ESTIMATES

	6	7	8	9	10
6	1.000				
7	-0.116	1.000			
8	0.009	0.074	1.000		
9	0.007	-0.209	0.044	1.000	
10	0.015	0.171	-0.101	0.097	1.000
11	-0.034	-0.024	0.042	-0.006	-0.043
12	-0.052	-0.015	-0.019	-0.008	0.001
13	-0.026	0.015	0.070	-0.010	-0.027
14	-0.021	-0.040	-0.018	0.021	-0.038
15	0.034	0.018	-0.008	0.005	0.002
16	0.007	0.014	-0.033	-0.012	0.031
17	-0.056	0.007	-0.031	-0.014	0.040
18	-0.070	0.034	0.049	-0.022	0.004
19	0.020	-0.028	0.024	-0.022	-0.029
20	-0.051	0.073	0.023	0.024	0.078
21	-0.022	0.014	0.039	0.031	0.031
22	-0.013	0.029	0.077	-0.004	0.030
23	-0.005	-0.060	0.026	0.022	-0.068
24	-0.029	0.010	-0.009	0.043	0.039
25	-0.009	0.028	-0.010	0.001	0.041
26	0.020	-0.029	-0.045	0.011	0.020
27	0.049	0.006	-0.026	0.007	-0.004
28	0.006	-0.013	-0.027	0.007	-0.003
29	0.043	-0.072	-0.001	0.012	-0.048
30	-0.026	-0.034	0.021	-0.010	-0.053
31	0.005	0.021	0.015	-0.002	0.019
32	-0.012	-0.123	0.035	-0.103	0.055
33	0.027	-0.009	0.024	-0.038	0.017
34	-0.026	0.025	0.007	-0.008	0.026
35	-0.014	0.007	0.029	-0.023	0.060
36	-0.007	-0.004	-0.036	-0.043	0.002
37	-0.017	0.005	0.049	-0.027	0.022
38	0.062	-0.009	-0.014	-0.024	-0.070
39	-0.003	0.022	-0.014	-0.029	0.050
40	0.018	0.037	-0.018	-0.055	0.019
41	0.002	-0.045	-0.040	0.041	0.018
42	-0.020	-0.012	0.058	-0.027	-0.027
43	-0.033	-0.001	0.053	-0.031	0.020

ESTIMATED CORRELATION MATRIX FOR PARAMETER ESTIMATES

	11	12	13	14	15
11	1.000				
12	0.301	1.000			
13	0.047	0.303	1.000		
14	-0.102	-0.152	-0.029	1.000	
15	-0.004	-0.111	-0.223	0.243	1.000
16	0.018	-0.001	0.021	-0.140	-0.238
17	0.095	0.243	0.017	-0.129	-0.035
18	0.045	0.119	0.337	-0.018	-0.194
19	0.012	-0.026	-0.054	0.068	0.247
20	0.014	0.012	0.035	-0.001	-0.052
21	0.040	0.215	0.049	-0.144	0.017
22	0.012	0.077	0.343	-0.046	-0.182
23	-0.031	-0.013	-0.025	0.094	0.229
24	-0.018	0.047	0.110	-0.040	-0.075
25	-0.011	0.014	0.040	-0.038	-0.030
26	-0.023	-0.255	-0.052	0.135	0.077
27	-0.018	-0.070	-0.372	0.035	0.197
28	0.007	-0.017	0.077	0.002	-0.277
29	-0.029	-0.014	-0.133	0.020	0.074
30	0.013	0.036	-0.071	0.062	0.056
31	0.025	0.039	0.105	-0.053	-0.064
32	-0.007	-0.059	-0.005	0.010	-0.042
33	-0.027	-0.087	0.020	0.027	-0.033
34	0.011	0.009	0.034	-0.006	-0.036
35	-0.025	0.011	-0.002	-0.010	-0.042
36	-0.002	-0.052	-0.003	0.006	-0.002
37	0.037	0.001	0.021	0.019	0.003
38	-0.008	-0.029	0.015	-0.018	0.011
39	0.018	0.013	0.007	-0.037	-0.016
40	-0.023	-0.023	-0.012	0.015	0.006
41	0.021	0.042	0.055	-0.036	-0.058
42	-0.010	-0.026	-0.013	-0.008	0.027
43	0.035	0.001	0.026	0.021	-0.004

ESTIMATED CORRELATION MATRIX FOR PARAMETER ESTIMATES

	16	17	18	19	20
16	1.000				
17	0.036	1.000			
18	0.032	0.256	1.000		
19	-0.193	-0.084	-0.204	1.000	

20	0.069	0.080	0.322	-0.263	1.000
21	-0.017	0.214	0.053	0.030	0.016
22	0.037	0.059	0.274	-0.047	0.058
23	-0.258	-0.021	-0.104	0.214	-0.053
24	0.014	0.051	0.272	-0.202	0.338
25	0.008	-0.007	0.080	-0.018	0.035
26	-0.039	-0.207	-0.037	-0.002	-0.001
27	-0.054	-0.061	-0.255	0.035	-0.067
28	0.205	0.014	0.067	-0.219	0.038
29	-0.016	-0.030	-0.320	0.202	-0.307
30	-0.049	0.000	-0.109	0.009	-0.054
31	0.018	0.022	0.088	-0.048	0.062
32	0.029	-0.030	-0.016	0.032	-0.008
33	0.006	-0.043	0.009	0.015	0.015
34	-0.032	0.032	0.050	-0.044	0.053
35	0.055	-0.024	-0.002	-0.049	0.021
36	0.020	-0.019	-0.032	0.018	-0.055
37	0.025	0.013	0.022	-0.027	-0.008
38	-0.011	-0.015	0.007	0.046	-0.003
39	0.038	-0.005	-0.009	-0.026	0.017
40	0.011	0.022	-0.001	0.016	-0.011
41	0.034	0.040	-0.002	-0.034	0.045
42	-0.038	-0.006	0.008	0.070	-0.055
43	0.027	0.012	0.026	-0.024	-0.005

ESTIMATED CORRELATION MATRIX FOR PARAMETER ESTIMATES

	21	22	23	24	25
21	1.000				
22	0.210	1.000			
23	-0.031	-0.185	1.000		
24	0.068	0.290	-0.161	1.000	
25	0.023	0.332	-0.177	0.305	1.000
26	-0.202	-0.059	0.038	-0.035	0.004
27	-0.053	-0.280	0.041	-0.119	-0.070
28	0.001	0.076	-0.216	0.073	0.015
29	0.001	-0.115	0.054	-0.260	-0.073
30	-0.022	-0.320	0.145	-0.250	-0.330
31	0.021	0.087	-0.055	0.088	0.054
32	-0.052	-0.043	0.012	-0.044	-0.008
33	-0.012	0.033	0.000	0.004	0.023
34	-0.009	-0.013	0.030	0.075	-0.005
35	-0.049	-0.005	0.021	-0.005	0.012

36	-0.028	-0.017	-0.023	0.003	0.034
37	0.000	0.027	-0.052	0.006	0.028
38	-0.006	-0.009	-0.003	-0.011	0.017
39	0.027	-0.013	-0.031	-0.022	0.006
40	0.064	-0.015	-0.012	0.043	-0.027
41	-0.061	-0.010	-0.024	0.062	-0.011
42	0.061	0.029	0.006	0.020	0.019
43	0.003	0.030	-0.052	0.008	0.024

ESTIMATED CORRELATION MATRIX FOR PARAMETER ESTIMATES

	26	27	28	29	30
26	1.000				
27	0.221	1.000			
28	-0.028	-0.179	1.000		
29	0.019	0.264	-0.182	1.000	
30	-0.013	0.231	-0.179	0.237	1.000
31	-0.026	-0.351	0.183	-0.239	-0.273
32	0.063	0.005	-0.031	-0.031	0.015
33	0.052	0.001	0.019	0.026	0.020
34	-0.031	-0.021	0.032	-0.014	-0.007
35	0.038	0.035	0.005	0.014	0.017
36	0.055	0.061	-0.031	0.016	-0.023
37	0.029	0.010	-0.010	0.061	0.034
38	-0.002	0.014	0.026	-0.025	-0.024
39	-0.027	-0.021	0.029	-0.001	0.032
40	0.008	0.003	-0.002	0.002	-0.018
41	-0.033	0.013	0.044	-0.001	-0.009
42	-0.010	-0.022	-0.044	0.059	0.032
43	0.026	0.002	-0.010	0.062	0.036

ESTIMATED CORRELATION MATRIX FOR PARAMETER ESTIMATES

	31	32	33	34	35
31	1.000				
32	0.016	1.000			
33	0.016	0.018	1.000		
34	0.039	-0.053	-0.064	1.000	
35	-0.013	0.110	0.034	0.059	1.000
36	-0.018	0.045	0.012	-0.012	0.116
37	0.006	0.004	0.013	-0.011	-0.004
38	0.037	0.176	0.031	-0.031	-0.093

39	-0.023	0.033	0.065	-0.021	-0.047
40	0.027	0.019	0.017	-0.067	-0.024
41	-0.001	0.015	-0.037	0.086	-0.016
42	0.010	0.004	0.021	-0.020	0.013
43	0.007	0.001	0.007	-0.003	0.009

ESTIMATED CORRELATION MATRIX FOR PARAMETER ESTIMATES

	36	37	38	39	40
36	1.000				
37	-0.091	1.000			
38	-0.027	0.068	1.000		
39	-0.038	-0.063	0.040	1.000	
40	0.023	0.034	0.014	0.010	1.000
41	-0.003	-0.050	0.015	0.024	-0.013
42	-0.001	-0.012	0.035	0.033	0.012
43	-0.096	0.993	0.054	-0.072	0.033

ESTIMATED CORRELATION MATRIX FOR PARAMETER ESTIMATES

	41	42	43
41	1.000		
42	-0.047	1.000	
43	-0.048	-0.009	1.000

TECHNICAL 8 OUTPUT

TECHNICAL 8 OUTPUT FOR BAYES ESTIMATION

CHAIN	BSEED
1	0
2	285380
3	253358

ITERATION	POTENTIAL SCALE REDUCTION	PARAMETER WITH HIGHEST PSR
100	1.021	43
200	1.014	38
300	1.019	43
400	1.010	43

500	1.005	43
600	1.007	43
700	1.007	37
800	1.007	43
900	1.006	43
1000	1.004	39

Improvement in physical health, time pressure interaction

SUMMARY OF DATA

Number of clusters 57

Size (s)	Cluster ID with Size s
2	4 166
3	65 74 80 30
4	84 22
5	205
6	228
7	93
8	106 144 165 71 170 173 192 72 50
9	40 108
11	107 83 181 61 75 68
12	69 174 206 226 81
13	162
16	92 154
19	114
20	145 2
22	116
23	36 156 169 142
24	152
26	98
28	122 202
30	178
32	6
46	24
59	204
62	125
64	209
108	217
216	110
223	100

COVARIANCE COVERAGE OF DATA

Minimum covariance coverage value 0.100

Number of missing data patterns 4

PROPORTION OF DATA PRESENT

Covariance Coverage

	PHYCAT16	PRAFAC	PHY15	KES15	CHOICE15
PHYCAT16	1.000				
PRAFAC	1.000	1.000			
PHY15	1.000	1.000	1.000		
KES15	0.971	0.971	0.971	0.971	
CHOICE15	0.963	0.963	0.963	0.963	0.963
BULLY15	0.966	0.966	0.966	0.966	0.963
PRESS15	0.966	0.966	0.966	0.966	0.963
DUTIES15	0.966	0.966	0.966	0.966	0.963

Covariance Coverage

	BULLY15	PRESS15	DUTIES15
BULLY15	0.966		
PRESS15	0.966	0.966	
DUTIES15	0.966	0.966	0.966

UNIVARIATE PROPORTIONS AND COUNTS FOR CATEGORICAL VARIABLES

PHYCAT16		
Category 1	0.364	517.000
Category 2	0.636	905.000

UNIVARIATE SAMPLE STATISTICS

UNIVARIATE HIGHER-ORDER MOMENT DESCRIPTIVE STATISTICS

Variable/ Sample Size	Mean/ Variance	Skewness/ Kurtosis	Minimum/ Maximum	% with Min/Max	Percentiles 20%/60%	40%/80%	Median
PRAFAC	-0.173	0.219	-2.338	1.75%	-1.489	-0.520	-0.258
57.000	1.361	-0.662	2.338	1.75%	0.080	0.848	

```
     PHY15                  0.000       2.446      -0.155   85.58%      -0.155     -0.155     -0.155
           1422.000       0.154       5.391       1.845    1.13%      -0.155     -0.155
     KES15                  0.000       1.141      -0.953    8.62%      -0.620     -0.287     -0.120
           1381.000       0.613       1.071       3.047    0.36%       0.047      0.547
     CHOICE15               0.000      -0.138      -1.878   14.60%      -0.878      0.122      0.122
           1370.000       1.250      -0.758       2.122    5.77%       0.122      1.122
     BULLY15                0.000       2.479      -0.312   79.02%      -0.312     -0.312     -0.312
           1373.000       0.474       6.315       3.688    0.44%      -0.312      0.688
     PRESS15                2.756       0.183       1.000   14.06%       2.000      2.000      3.000
           1373.000       1.207      -0.514       5.000    7.21%       3.000      4.000
     DUTIES15               0.000      -0.896      -2.873    3.06%      -0.873      0.127      0.127
           1373.000       0.975       0.587       1.127   27.75%       0.127      1.127
```

```
THE MODEL ESTIMATION TERMINATED NORMALLY

     USE THE FBITERATIONS OPTION TO INCREASE THE NUMBER OF ITERATIONS BY A FACTOR
     OF AT LEAST TWO TO CHECK CONVERGENCE AND THAT THE PSR VALUE DOES NOT INCREASE.



MODEL FIT INFORMATION

Number of Free Parameters                              43



MODEL RESULTS

                                Posterior  One-Tailed         95% C.I.
                    Estimate       S.D.      P-Value   Lower 2.5%  Upper 2.5%  Significance

Within Level

 PHYCAT16   ON
    PHY15              0.832       0.116      0.000       0.613       1.060      *
    CHOICE15          -0.048       0.034      0.075      -0.117       0.018
    DUTIES15           0.037       0.039      0.168      -0.037       0.114
    BULLY15            0.050       0.058      0.191      -0.063       0.165
    KES15              0.167       0.052      0.000       0.066       0.264      *

 KES15    WITH
    PHY15              0.070       0.009      0.000       0.054       0.089      *
    CHOICE15          -0.135       0.024      0.000      -0.183      -0.090      *
```

BULLY15	0.127	0.015	0.000	0.098	0.158	*
PRESS15	0.209	0.024	0.000	0.164	0.257	*
DUTIES15	-0.207	0.021	0.000	-0.247	-0.164	*
PHY15 WITH						
CHOICE15	-0.032	0.012	0.001	-0.057	-0.010	*
BULLY15	0.013	0.007	0.039	-0.001	0.026	
PRESS15	0.016	0.012	0.100	-0.008	0.038	
DUTIES15	-0.009	0.011	0.198	-0.029	0.012	
CHOICE15 WITH						
BULLY15	-0.133	0.021	0.000	-0.173	-0.094	*
PRESS15	-0.174	0.032	0.000	-0.236	-0.111	*
DUTIES15	0.162	0.030	0.000	0.105	0.222	*
BULLY15 WITH						
PRESS15	0.176	0.020	0.000	0.135	0.216	*
DUTIES15	-0.141	0.019	0.000	-0.179	-0.105	*
PRESS15 WITH						
DUTIES15	-0.247	0.030	0.000	-0.309	-0.187	*
Means						
PHY15	0.000	0.010	0.497	-0.019	0.021	
KES15	0.000	0.021	0.498	-0.040	0.044	
CHOICE15	0.001	0.029	0.491	-0.056	0.056	
BULLY15	0.000	0.019	0.494	-0.039	0.036	
DUTIES15	0.001	0.028	0.491	-0.054	0.053	
Variances						
PHY15	0.154	0.006	0.000	0.144	0.166	*
KES15	0.615	0.024	0.000	0.569	0.661	*
CHOICE15	1.253	0.047	0.000	1.166	1.351	*
BULLY15	0.475	0.018	0.000	0.439	0.511	*
PRESS15	1.178	0.045	0.000	1.093	1.267	*
DUTIES15	0.977	0.037	0.000	0.908	1.052	*
Between Level						
S1 ON						
PRAFAC	0.001	0.038	0.490	-0.075	0.073	
PHYCAT16 ON						
PRAFAC	-0.051	0.042	0.117	-0.130	0.036	

```
    PRESS15            0.027       0.220       0.449      -0.453       0.465

 PRAFAC   WITH
    PRESS15            0.048       0.072       0.253      -0.084       0.197

 Means
    PRAFAC            -0.172       0.154       0.139      -0.470       0.139
    PRESS15            2.710       0.057       0.000       2.594       2.821      *

 Intercepts
    S1                -0.013       0.043       0.379      -0.103       0.069

 Thresholds
    PHYCAT16$1        -0.313       0.608       0.273      -1.655       0.874

 Variances
    PRAFAC             1.369       0.262       0.000       1.001       1.997      *
    PRESS15            0.086       0.025       0.000       0.051       0.147      *

 Residual Variances
    PHYCAT16           0.007       0.011       0.000       0.000       0.041      *
    S1                 0.008       0.011       0.000       0.001       0.043      *


STANDARDIZED MODEL RESULTS


STDYX Standardization

                                Posterior  One-Tailed         95% C.I.
                    Estimate       S.D.      P-Value   Lower 2.5%  Upper 2.5%  Significance

Within-Level Standardized Estimates Averaged Over Clusters

 S1 | PHYCAT16 ON
    PRESS15           -0.015       0.041       0.359      -0.097       0.065

 PHYCAT16   ON
    PHY15              0.301       0.039       0.000       0.223       0.377      *
    CHOICE15          -0.049       0.034       0.067      -0.120       0.016
    DUTIES15           0.034       0.035       0.167      -0.034       0.104
    BULLY15            0.033       0.036       0.185      -0.039       0.104
    KES15              0.121       0.038       0.000       0.046       0.196      *
```

```
 KES15    WITH
    PHY15              0.230       0.026      0.000       0.179       0.280      *
    CHOICE15          -0.154       0.026      0.000      -0.204      -0.102      *
    BULLY15            0.236       0.026      0.000       0.184       0.285      *
    PRESS15            0.246       0.025      0.000       0.197       0.296      *
    DUTIES15          -0.267       0.025      0.000      -0.313      -0.216      *

 PHY15    WITH
    CHOICE15          -0.074       0.027      0.002      -0.128      -0.023      *
    BULLY15            0.046       0.027      0.040      -0.005       0.097
    PRESS15            0.037       0.028      0.095      -0.018       0.090
    DUTIES15          -0.024       0.028      0.201      -0.077       0.031

 CHOICE15 WITH
    BULLY15           -0.172       0.026      0.000      -0.222      -0.120      *
    PRESS15           -0.143       0.027      0.000      -0.192      -0.088      *
    DUTIES15           0.146       0.026      0.000       0.093       0.196      *

 BULLY15  WITH
    PRESS15            0.235       0.026      0.000       0.183       0.283      *
    DUTIES15          -0.206       0.026      0.000      -0.256      -0.154      *

 PRESS15  WITH
    DUTIES15          -0.230       0.026      0.000      -0.280      -0.179      *

 Means
    PHY15              0.000       0.027      0.498      -0.051       0.054
    KES15              0.000       0.026      0.499      -0.051       0.051
    CHOICE15           0.000       0.026      0.496      -0.051       0.051
    BULLY15            0.000       0.028      0.492      -0.056       0.053
    DUTIES15           0.000       0.028      0.498      -0.056       0.054

 Variances
    PHY15              1.000       0.000      0.000       1.000       1.000
    KES15              1.000       0.000      0.000       1.000       1.000
    CHOICE15           1.000       0.000      0.000       1.000       1.000
    BULLY15            1.000       0.000      0.000       1.000       1.000
    PRESS15            1.000       0.000      0.000       1.000       1.000
    DUTIES15           1.000       0.000      0.000       1.000       1.000

Between Level

 S1         ON
    PRAFAC             0.014       0.443      0.490      -0.788       0.831
```

```
 PHYCAT16   ON
    PRAFAC            -0.490       0.349      0.117      -0.947       0.403
    PRESS15            0.072       0.458      0.449      -0.867       0.818

 PRAFAC   WITH
    PRESS15            0.142       0.192      0.253      -0.233       0.489

 Means
    PRAFAC            -0.145       0.131      0.139      -0.407       0.114
    PRESS15            9.205       1.304      0.000       7.007      12.022      *

 Intercepts
    S1                -0.132       0.513      0.379      -1.163       0.864

 Variances
    PRAFAC             1.000       0.000      0.000       1.000       1.000
    PRESS15            1.000       0.000      0.000       1.000       1.000

 Residual Variances
    PHYCAT16           0.489       0.278      0.000       0.023       0.961      *
    S1                 0.890       0.218      0.000       0.257       1.000      *


R-SQUARE

Within-Level R-Square Averaged Across Clusters

                                Posterior  One-Tailed         95% C.I.
    Variable        Estimate       S.D.      P-Value   Lower 2.5%  Upper 2.5%

    PHYCAT16           0.147       0.027      0.000       0.099       0.205

Between Level

                                Posterior  One-Tailed         95% C.I.
    Variable        Estimate       S.D.      P-Value   Lower 2.5%  Upper 2.5%

    PHYCAT16           0.511       0.278      0.000       0.039       0.977

                                Posterior  One-Tailed         95% C.I.
    Variable        Estimate       S.D.      P-Value   Lower 2.5%  Upper 2.5%

    S1                 0.110       0.218      0.000       0.000       0.743
```

TECHNICAL 1 OUTPUT

PARAMETER SPECIFICATION FOR WITHIN

TAU

PHYCAT16
0

NU

PHYCAT16	PHY15	KES15	CHOICE15	BULLY15
0	0	0	0	0

NU

PRESS15	DUTIES15
0	0

LAMBDA

	PHYCAT16	PHY15	KES15	CHOICE15	BULLY15
PHYCAT16	0	0	0	0	0
PHY15	0	0	0	0	0
KES15	0	0	0	0	0
CHOICE15	0	0	0	0	0
BULLY15	0	0	0	0	0
PRESS15	0	0	0	0	0
DUTIES15	0	0	0	0	0

LAMBDA

	PRESS15	DUTIES15
PHYCAT16	0	0
PHY15	0	0
KES15	0	0

CHOICE15	0	0
BULLY15	0	0
PRESS15	0	0
DUTIES15	0	0

THETA

	PHYCAT16	PHY15	KES15	CHOICE15	BULLY15
PHYCAT16	0				
PHY15	0	0			
KES15	0	0	0		
CHOICE15	0	0	0	0	
BULLY15	0	0	0	0	0
PRESS15	0	0	0	0	0
DUTIES15	0	0	0	0	0

THETA

	PRESS15	DUTIES15
PRESS15	0	
DUTIES15	0	0

ALPHA

PHYCAT16	PHY15	KES15	CHOICE15	BULLY15
0	1	2	3	4

ALPHA

PRESS15	DUTIES15
0	5

BETA

	PHYCAT16	PHY15	KES15	CHOICE15	BULLY15
PHYCAT16	0	6	7	8	9
PHY15	0	0	0	0	0
KES15	0	0	0	0	0
CHOICE15	0	0	0	0	0

BULLY15	0	0	0	0	0
PRESS15	0	0	0	0	0
DUTIES15	0	0	0	0	0

BETA

	PRESS15	DUTIES15
PHYCAT16	0	10
PHY15	0	0
KES15	0	0
CHOICE15	0	0
BULLY15	0	0
PRESS15	0	0
DUTIES15	0	0

PSI

	PHYCAT16	PHY15	KES15	CHOICE15	BULLY15
PHYCAT16	0				
PHY15	0	11			
KES15	0	12	13		
CHOICE15	0	14	15	16	
BULLY15	0	17	18	19	20
PRESS15	0	21	22	23	24
DUTIES15	0	26	27	28	29

PSI

	PRESS15	DUTIES15
PRESS15	25	
DUTIES15	30	31

PARAMETER SPECIFICATION FOR BETWEEN

TAU

PHYCAT16
43

NU

PHYCAT16	PRAFAC	PRESS15
0	0	0

LAMBDA

	S1	PHYCAT16	PRAFAC	PRESS15
PHYCAT16	0	0	0	0
PRAFAC	0	0	0	0
PRESS15	0	0	0	0

THETA

	PHYCAT16	PRAFAC	PRESS15
PHYCAT16	0		
PRAFAC	0	0	
PRESS15	0	0	0

ALPHA

S1	PHYCAT16	PRAFAC	PRESS15
32	0	33	34

BETA

	S1	PHYCAT16	PRAFAC	PRESS15
S1	0	0	35	0
PHYCAT16	0	0	36	37
PRAFAC	0	0	0	0
PRESS15	0	0	0	0

PSI

	S1	PHYCAT16	PRAFAC	PRESS15
S1	38			
PHYCAT16	0	39		
PRAFAC	0	0	40	

PRESS15	0	0	41	42

STARTING VALUES FOR WITHIN

TAU

PHYCAT16
0.000

NU

PHYCAT16	PHY15	KES15	CHOICE15	BULLY15
0.000	0.000	0.000	0.000	0.000

NU

PRESS15	DUTIES15
0.000	0.000

LAMBDA

	PHYCAT16	PHY15	KES15	CHOICE15	BULLY15
PHYCAT16	1.000	0.000	0.000	0.000	0.000
PHY15	0.000	1.000	0.000	0.000	0.000
KES15	0.000	0.000	1.000	0.000	0.000
CHOICE15	0.000	0.000	0.000	1.000	0.000
BULLY15	0.000	0.000	0.000	0.000	1.000
PRESS15	0.000	0.000	0.000	0.000	0.000
DUTIES15	0.000	0.000	0.000	0.000	0.000

LAMBDA

	PRESS15	DUTIES15
PHYCAT16	0.000	0.000
PHY15	0.000	0.000
KES15	0.000	0.000
CHOICE15	0.000	0.000
BULLY15	0.000	0.000

PRESS15	1.000	0.000
DUTIES15	0.000	1.000

THETA

	PHYCAT16	PHY15	KES15	CHOICE15	BULLY15
PHYCAT16	0.000				
PHY15	0.000	0.000			
KES15	0.000	0.000	0.000		
CHOICE15	0.000	0.000	0.000	0.000	
BULLY15	0.000	0.000	0.000	0.000	0.000
PRESS15	0.000	0.000	0.000	0.000	0.000
DUTIES15	0.000	0.000	0.000	0.000	0.000

THETA

	PRESS15	DUTIES15
PRESS15	0.000	
DUTIES15	0.000	0.000

ALPHA

PHYCAT16	PHY15	KES15	CHOICE15	BULLY15
0.000	0.000	0.000	0.000	0.000

ALPHA

PRESS15	DUTIES15
0.000	0.000

BETA

	PHYCAT16	PHY15	KES15	CHOICE15	BULLY15
PHYCAT16	0.000	0.000	0.000	0.000	0.000
PHY15	0.000	0.000	0.000	0.000	0.000
KES15	0.000	0.000	0.000	0.000	0.000
CHOICE15	0.000	0.000	0.000	0.000	0.000
BULLY15	0.000	0.000	0.000	0.000	0.000
PRESS15	0.000	0.000	0.000	0.000	0.000

DUTIES15	0.000	0.000	0.000	0.000	0.000

BETA

	PRESS15	DUTIES15
PHYCAT16	0.000	0.000
PHY15	0.000	0.000
KES15	0.000	0.000
CHOICE15	0.000	0.000
BULLY15	0.000	0.000
PRESS15	0.000	0.000
DUTIES15	0.000	0.000

PSI

	PHYCAT16	PHY15	KES15	CHOICE15	BULLY15
PHYCAT16	1.000				
PHY15	0.000	0.077			
KES15	0.000	0.000	0.306		
CHOICE15	0.000	0.000	0.000	0.625	
BULLY15	0.000	0.000	0.000	0.000	0.237
PRESS15	0.000	0.000	0.000	0.000	0.000
DUTIES15	0.000	0.000	0.000	0.000	0.000

PSI

	PRESS15	DUTIES15
PRESS15	0.604	
DUTIES15	0.000	0.488

STARTING VALUES FOR BETWEEN

TAU

PHYCAT16
-0.311

NU

PHYCAT16	PRAFAC	PRESS15
0.000	0.000	0.000

LAMBDA

	S1	PHYCAT16	PRAFAC	PRESS15
PHYCAT16	0.000	1.000	0.000	0.000
PRAFAC	0.000	0.000	1.000	0.000
PRESS15	0.000	0.000	0.000	1.000

THETA

	PHYCAT16	PRAFAC	PRESS15
PHYCAT16	0.000		
PRAFAC	0.000	0.000	
PRESS15	0.000	0.000	0.000

ALPHA

S1	PHYCAT16	PRAFAC	PRESS15
0.000	0.000	-0.078	2.756

BETA

	S1	PHYCAT16	PRAFAC	PRESS15
S1	0.000	0.000	0.000	0.000
PHYCAT16	0.000	0.000	0.000	0.000
PRAFAC	0.000	0.000	0.000	0.000
PRESS15	0.000	0.000	0.000	0.000

PSI

	S1	PHYCAT16	PRAFAC	PRESS15
S1	1.000			
PHYCAT16	0.000	1.000		
PRAFAC	0.000	0.000	0.480	
PRESS15	0.000	0.000	0.000	0.604

PRIORS FOR ALL PARAMETERS	PRIOR MEAN	PRIOR VARIANCE	PRIOR STD. DEV.
Parameter 1~N(0.000,infinity)	0.0000	infinity	infinity
Parameter 2~N(0.000,infinity)	0.0000	infinity	infinity
Parameter 3~N(0.000,infinity)	0.0000	infinity	infinity
Parameter 4~N(0.000,infinity)	0.0000	infinity	infinity
Parameter 5~N(0.000,infinity)	0.0000	infinity	infinity
Parameter 6~N(0.000,5.000)	0.0000	5.0000	2.2361
Parameter 7~N(0.000,5.000)	0.0000	5.0000	2.2361
Parameter 8~N(0.000,5.000)	0.0000	5.0000	2.2361
Parameter 9~N(0.000,5.000)	0.0000	5.0000	2.2361
Parameter 10~N(0.000,5.000)	0.0000	5.0000	2.2361
Parameter 11~IW(1.000,7)	infinity	infinity	infinity
Parameter 12~IW(0.000,7)	infinity	infinity	infinity
Parameter 13~IW(1.000,7)	infinity	infinity	infinity
Parameter 14~IW(0.000,7)	infinity	infinity	infinity
Parameter 15~IW(0.000,7)	infinity	infinity	infinity
Parameter 16~IW(1.000,7)	infinity	infinity	infinity
Parameter 17~IW(0.000,7)	infinity	infinity	infinity
Parameter 18~IW(0.000,7)	infinity	infinity	infinity
Parameter 19~IW(0.000,7)	infinity	infinity	infinity
Parameter 20~IW(1.000,7)	infinity	infinity	infinity
Parameter 21~IW(0.000,7)	infinity	infinity	infinity
Parameter 22~IW(0.000,7)	infinity	infinity	infinity
Parameter 23~IW(0.000,7)	infinity	infinity	infinity
Parameter 24~IW(0.000,7)	infinity	infinity	infinity
Parameter 25~IW(1.000,7)	infinity	infinity	infinity
Parameter 26~IW(0.000,7)	infinity	infinity	infinity
Parameter 27~IW(0.000,7)	infinity	infinity	infinity
Parameter 28~IW(0.000,7)	infinity	infinity	infinity
Parameter 29~IW(0.000,7)	infinity	infinity	infinity
Parameter 30~IW(0.000,7)	infinity	infinity	infinity
Parameter 31~IW(1.000,7)	infinity	infinity	infinity
Parameter 32~N(0.000,infinity)	0.0000	infinity	infinity
Parameter 33~N(0.000,infinity)	0.0000	infinity	infinity
Parameter 34~N(0.000,infinity)	0.0000	infinity	infinity
Parameter 35~N(0.000,infinity)	0.0000	infinity	infinity
Parameter 36~N(0.000,5.000)	0.0000	5.0000	2.2361
Parameter 37~N(0.000,5.000)	0.0000	5.0000	2.2361
Parameter 38~IG(-1.000,0.000)	infinity	infinity	infinity
Parameter 39~IG(-1.000,0.000)	infinity	infinity	infinity
Parameter 40~IW(1.000,3)	infinity	infinity	infinity

Parameter 41~IW(0.000,3)	infinity	infinity	infinity
Parameter 42~IW(1.000,3)	infinity	infinity	infinity
Parameter 43~N(0.000,5.000)	0.0000	5.0000	2.2361

TECHNICAL 3 OUTPUT

ESTIMATED COVARIANCE MATRIX FOR PARAMETER ESTIMATES

	1	2	3	4	5
1	0.107739D-03				
2	0.490195D-04	0.436316D-03			
3	-0.196822D-04	-0.838017D-04	0.854319D-03		
4	0.143563D-04	0.913174D-04	-0.111636D-03	0.373093D-03	
5	-0.153121D-04	-0.160076D-03	0.116052D-03	-0.976627D-04	0.757903D-03
6	-0.273219D-04	-0.816061D-04	-0.109219D-03	0.406770D-04	0.801508D-04
7	0.502284D-05	-0.369807D-04	-0.756879D-05	0.122842D-04	-0.255341D-04
8	-0.436610D-05	-0.275289D-04	-0.127656D-04	0.171561D-04	-0.696856D-05
9	-0.893157D-05	0.260370D-05	-0.296360D-04	0.880363D-06	-0.537609D-04
10	0.201053D-04	0.289633D-05	0.352124D-04	-0.126147D-04	-0.134350D-04
11	0.566735D-06	0.111774D-04	0.342798D-05	0.440125D-05	-0.178732D-05
12	-0.686264D-06	0.718792D-05	0.122290D-04	-0.529836D-06	-0.285816D-05
13	0.206023D-05	-0.514013D-06	-0.253448D-04	-0.540856D-05	-0.258730D-04
14	0.701714D-06	-0.478671D-05	0.144676D-05	0.157049D-05	0.673205D-05
15	0.663414D-05	-0.533631D-05	-0.216548D-04	0.800569D-05	0.218093D-04
16	0.872294D-05	0.153552D-04	-0.196667D-04	0.134401D-04	-0.849548D-05
17	0.111477D-05	0.454870D-05	0.289231D-05	-0.271711D-05	0.353495D-05
18	0.689309D-06	0.677532D-05	-0.846661D-05	0.185087D-04	-0.181918D-05
19	0.113449D-04	-0.452980D-05	-0.192072D-05	-0.162711D-04	-0.674186D-05
20	-0.641482D-05	0.103956D-04	0.133680D-05	0.532421D-05	-0.100846D-04
21	-0.451072D-05	0.654977D-06	-0.166370D-05	-0.197881D-05	0.831745D-06
22	0.263539D-05	0.899361D-05	-0.237355D-04	-0.897281D-05	0.678580D-06
23	0.425998D-05	0.175383D-05	-0.124782D-04	0.286182D-06	-0.323647D-04
24	0.289227D-05	0.135660D-04	-0.525446D-05	0.185690D-05	-0.262568D-05
25	-0.504205D-05	0.203165D-04	-0.634540D-04	-0.130298D-04	0.265886D-04
26	0.667578D-05	-0.519217D-05	0.165101D-05	0.753293D-06	0.872680D-05
27	0.464497D-05	0.167656D-05	0.267442D-04	0.403463D-05	0.245771D-04
28	-0.184982D-04	0.131365D-04	-0.126491D-04	0.109929D-04	-0.140910D-04
29	0.133678D-04	0.718657D-05	0.153603D-04	-0.263670D-05	-0.803712D-05
30	0.602166D-05	0.225926D-05	0.285648D-04	0.326811D-05	-0.270295D-04
31	-0.133623D-05	-0.139439D-04	-0.577339D-04	-0.357321D-05	-0.265403D-04
32	0.211442D-04	0.352900D-04	0.114764D-04	0.106238D-05	0.531809D-05
33	0.217981D-04	-0.357979D-04	-0.944912D-04	-0.157279D-04	0.509849D-04

34	0.250173D-04	0.155382D-03	-0.107016D-03	0.901152D-04	-0.231635D-03
35	0.255683D-05	-0.443120D-05	0.175740D-05	0.356974D-04	0.126166D-04
36	-0.386811D-05	0.714231D-05	0.601016D-04	-0.274621D-05	-0.139713D-04
37	-0.257625D-04	-0.946564D-04	0.125452D-03	0.468289D-04	-0.225190D-03
38	-0.415996D-05	0.836272D-05	-0.704814D-05	0.669725D-05	-0.180085D-04
39	-0.410994D-05	0.268205D-05	0.346552D-05	-0.142170D-04	0.847411D-05
40	0.403594D-04	-0.774576D-04	-0.117329D-03	0.728867D-05	0.260597D-03
41	0.414288D-04	0.124167D-03	0.332678D-04	0.394318D-04	-0.263896D-04
42	0.106177D-04	0.786777D-05	-0.743312D-05	0.266237D-05	-0.139871D-04
43	-0.619711D-04	-0.229538D-03	0.364494D-03	0.109801D-03	-0.626708D-03

ESTIMATED COVARIANCE MATRIX FOR PARAMETER ESTIMATES

	6	7	8	9	10
6	0.135382D-01				
7	-0.739207D-03	0.270083D-02			
8	0.616001D-04	0.127138D-03	0.113023D-02		
9	-0.106438D-03	-0.534321D-03	0.131675D-03	0.335004D-02	
10	0.735544D-04	0.335838D-03	-0.144867D-03	0.158264D-03	0.149803D-02
11	-0.213743D-04	-0.713191D-05	0.740664D-05	-0.936949D-05	-0.913489D-05
12	-0.503269D-04	-0.697058D-05	-0.683139D-05	-0.869914D-05	0.283889D-06
13	-0.575465D-04	0.198379D-04	0.539777D-04	-0.920100D-05	-0.239433D-04
14	-0.317493D-04	-0.288501D-04	-0.505463D-05	0.341651D-04	-0.163835D-04
15	0.872727D-04	0.175174D-04	-0.194131D-05	0.349944D-04	0.557401D-05
16	0.624641D-04	0.327703D-04	-0.622283D-04	-0.571520D-04	0.522995D-04
17	-0.450812D-04	0.447316D-05	-0.676921D-05	-0.696913D-05	0.133120D-04
18	-0.123277D-03	0.297360D-04	0.228281D-04	-0.467519D-04	0.437210D-05
19	0.218056D-04	-0.319216D-04	0.151109D-04	0.102440D-05	-0.224636D-04
20	-0.987907D-04	0.682343D-04	0.109039D-04	-0.678866D-05	0.568270D-04
21	-0.281658D-04	0.561731D-05	0.192887D-04	0.179231D-04	0.157376D-04
22	-0.417643D-04	0.443719D-04	0.653243D-04	0.272650D-04	0.256688D-04
23	0.670687D-05	-0.108450D-03	0.380583D-04	0.861744D-04	-0.658291D-04
24	-0.518114D-04	0.143075D-04	-0.789937D-05	-0.105823D-06	0.296885D-04
25	-0.785133D-04	0.838892D-04	-0.139415D-04	-0.625561D-04	0.647555D-04
26	0.194963D-04	-0.212050D-04	-0.156459D-04	0.153465D-04	0.520336D-05
27	0.106821D-03	0.517024D-05	-0.161506D-04	0.114429D-06	-0.107517D-04
28	0.163963D-04	-0.141963D-04	-0.314257D-04	-0.589159D-04	-0.725158D-05
29	0.827731D-04	-0.633703D-04	-0.917664D-06	0.440089D-04	-0.313212D-04
30	-0.740992D-04	-0.642590D-04	0.258702D-04	-0.249081D-04	-0.666601D-04
31	0.501840D-04	0.390969D-04	0.187968D-04	-0.461882D-04	0.341312D-04
32	-0.366700D-04	-0.372886D-03	0.411808D-04	-0.288842D-03	0.161884D-03
33	0.373183D-03	-0.776835D-04	0.115946D-03	-0.121426D-03	0.927009D-04
34	-0.177055D-03	0.853575D-04	0.131491D-04	-0.131251D-03	-0.959143D-07

35	-0.512002D-04	-0.151517D-04	0.249100D-04	-0.950680D-04	0.218252D-05
36	-0.347971D-04	-0.396175D-05	-0.368650D-04	-0.731954D-04	0.400810D-05
37	-0.329497D-03	-0.500357D-05	0.437260D-03	-0.116606D-03	-0.107548D-03
38	0.375037D-04	0.585283D-05	0.485950D-05	0.234867D-04	-0.978127D-05
39	-0.281902D-04	0.228526D-04	0.201938D-05	-0.424825D-04	0.240487D-04
40	0.318159D-03	0.353754D-03	-0.177182D-03	-0.783275D-03	0.276333D-03
41	-0.534799D-04	-0.129816D-03	-0.684290D-04	0.572517D-04	0.795834D-04
42	0.186126D-04	0.132077D-04	0.369791D-04	-0.389562D-04	0.203601D-04
43	-0.161771D-02	-0.122521D-03	0.124388D-02	-0.399114D-03	-0.332061D-03

ESTIMATED COVARIANCE MATRIX FOR PARAMETER ESTIMATES

	11	12	13	14	15
11	0.329673D-04				
12	0.152671D-04	0.780710D-04			
13	0.636321D-05	0.636640D-04	0.568260D-03		
14	-0.710189D-05	-0.162781D-04	-0.801375D-05	0.146581D-03	
15	-0.520597D-06	-0.233494D-04	-0.126289D-03	0.702533D-04	0.568659D-03
16	0.458485D-05	-0.466950D-06	0.234246D-04	-0.801564D-04	-0.269105D-03
17	0.403045D-05	0.151987D-04	0.271945D-05	-0.125177D-04	-0.626977D-05
18	0.337527D-05	0.161585D-04	0.120679D-03	-0.373442D-05	-0.738960D-04
19	0.304519D-05	-0.265605D-05	-0.262581D-04	0.156048D-04	0.124140D-03
20	0.547102D-06	0.154923D-05	0.121567D-04	-0.172650D-05	-0.227195D-04
21	0.292736D-05	0.228638D-04	0.137566D-04	-0.190149D-04	0.510179D-05
22	0.225145D-05	0.159816D-04	0.200092D-03	-0.128218D-04	-0.101503D-03
23	-0.701580D-05	-0.645685D-05	-0.248476D-04	0.381531D-04	0.177621D-03
24	-0.214587D-05	0.907780D-05	0.556321D-04	-0.109298D-04	-0.352167D-04
25	-0.235051D-05	0.507847D-05	0.477990D-04	-0.171678D-04	-0.356652D-04
26	-0.142232D-05	-0.239037D-04	-0.132191D-04	0.172750D-04	0.196136D-04
27	-0.208846D-05	-0.130020D-04	-0.188599D-03	0.918320D-05	0.998730D-04
28	0.123588D-05	-0.469800D-05	0.555175D-04	0.103589D-05	-0.201593D-03
29	-0.297679D-05	-0.266220D-05	-0.585031D-04	0.522040D-05	0.353537D-04
30	0.196033D-05	0.107460D-04	-0.529890D-04	0.216868D-04	0.397504D-04
31	0.508218D-05	0.125801D-04	0.920611D-04	-0.236292D-04	-0.567699D-04
32	-0.788469D-07	-0.209136D-04	-0.196935D-04	0.195249D-05	-0.447768D-04
33	-0.241245D-04	-0.118212D-03	0.720765D-04	0.506352D-04	-0.124052D-03
34	0.644928D-05	-0.262989D-05	0.584844D-04	-0.580819D-05	-0.748549D-04
35	-0.569220D-05	0.168647D-06	-0.683287D-05	-0.519510D-05	-0.297673D-04
36	-0.310106D-06	-0.180616D-04	0.519846D-05	-0.150419D-05	-0.130412D-04
37	0.873836D-05	0.535717D-05	0.186380D-03	0.106279D-03	0.206434D-04
38	0.507727D-06	-0.246770D-05	0.794944D-05	-0.165494D-05	-0.204123D-05
39	0.209514D-05	0.197629D-05	0.258253D-05	-0.195540D-05	-0.411777D-05
40	-0.230073D-04	-0.380768D-04	-0.357594D-04	0.960110D-05	-0.187585D-04

41	0.110185D-04	0.288891D-04	0.924945D-04	-0.343514D-04	-0.899972D-04
42	0.127433D-06	-0.448657D-05	-0.527705D-05	-0.591950D-05	0.805813D-05
43	0.210443D-04	0.146582D-04	0.552942D-03	0.306549D-03	0.218639D-04

ESTIMATED COVARIANCE MATRIX FOR PARAMETER ESTIMATES

	16	17	18	19	20
16	0.224752D-02				
17	0.132649D-04	0.512730D-04			
18	0.234533D-04	0.269097D-04	0.230175D-03		
19	-0.203944D-03	-0.105235D-04	-0.597497D-04	0.435245D-03	
20	0.626692D-04	0.995654D-05	0.867592D-04	-0.988878D-04	0.335409D-03
21	-0.115566D-04	0.192417D-04	0.111226D-04	0.543254D-05	0.526798D-05
22	0.382659D-04	0.111219D-04	0.103482D-03	-0.261618D-04	0.257847D-04
23	-0.388420D-03	-0.708572D-05	-0.567387D-04	0.154097D-03	-0.404137D-04
24	0.162099D-04	0.777026D-05	0.869308D-04	-0.771979D-04	0.125854D-03
25	0.178417D-04	-0.303921D-05	0.567562D-04	-0.234057D-04	0.307119D-04
26	-0.190904D-04	-0.162408D-04	-0.607381D-05	-0.151563D-05	-0.101492D-05
27	-0.539224D-04	-0.101304D-04	-0.839054D-04	0.174459D-04	-0.261697D-04
28	0.296333D-03	0.209012D-05	0.310142D-04	-0.136929D-03	0.198565D-04
29	-0.175667D-04	-0.393212D-05	-0.894257D-04	0.752624D-04	-0.104104D-03
30	-0.659212D-04	-0.208609D-05	-0.532235D-04	0.567356D-05	-0.326581D-04
31	0.316467D-04	0.551827D-05	0.513475D-04	-0.379312D-04	0.431266D-04
32	0.791537D-04	-0.728222D-06	-0.598443D-05	0.110640D-04	-0.896877D-05
33	0.487849D-04	-0.440539D-04	0.348887D-04	0.219194D-04	0.495900D-04
34	-0.510375D-04	0.628897D-05	0.261531D-04	-0.781706D-05	0.230800D-04
35	0.113652D-03	-0.113860D-04	-0.401341D-05	-0.323561D-04	0.154010D-04
36	0.322180D-04	-0.905950D-05	-0.103312D-04	-0.298097D-05	-0.206759D-04
37	-0.852974D-04	-0.198193D-05	0.619707D-05	-0.232069D-04	-0.239161D-03
38	-0.158932D-05	0.163635D-05	0.597704D-05	0.786852D-07	0.679238D-05
39	0.175532D-04	0.138556D-06	-0.320694D-05	-0.317140D-05	0.193955D-05
40	0.152447D-03	0.586441D-04	0.649504D-05	0.926909D-04	-0.322157D-04
41	0.824581D-04	0.126573D-04	0.101812D-04	-0.416191D-04	0.453542D-04
42	-0.457206D-04	-0.930670D-06	0.503262D-05	0.106621D-04	0.114074D-04
43	-0.263040D-03	-0.872272D-05	0.532619D-04	-0.321690D-04	-0.645000D-03

ESTIMATED COVARIANCE MATRIX FOR PARAMETER ESTIMATES

	21	22	23	24	25
21	0.138557D-03				
22	0.598051D-04	0.573718D-03			
23	-0.153872D-04	-0.143685D-03	0.102634D-02		

24	0.192608D-04	0.137959D-03	-0.133795D-03	0.415639D-03	
25	0.138091D-04	0.364897D-03	-0.232802D-03	0.285274D-03	0.200731D-02
26	-0.259356D-04	-0.155062D-04	0.143456D-04	-0.758231D-05	0.432511D-06
27	-0.131846D-04	-0.145608D-03	0.284689D-04	-0.478828D-04	-0.737895D-04
28	0.171594D-05	0.555429D-04	-0.220963D-03	0.469403D-04	0.250531D-04
29	-0.108088D-05	-0.554543D-04	0.395815D-04	-0.103886D-03	-0.581636D-04
30	-0.194818D-05	-0.227076D-03	0.142855D-03	-0.155684D-03	-0.453535D-03
31	0.775743D-05	0.763818D-04	-0.620491D-04	0.666935D-04	0.921156D-04
32	-0.231608D-04	-0.546846D-04	0.107200D-04	-0.258386D-04	0.700147D-05
33	-0.292432D-04	0.112525D-03	0.327310D-04	0.223860D-04	0.158779D-03
34	0.146540D-05	0.153891D-04	-0.735940D-04	0.835905D-04	0.531303D-04
35	-0.252991D-04	-0.226089D-04	0.161208D-04	-0.710217D-05	0.208559D-04
36	-0.180046D-04	-0.171040D-04	-0.374850D-04	0.100088D-04	0.839072D-04
37	0.630275D-04	0.176646D-03	0.837105D-05	-0.413544D-05	-0.222611D-03
38	-0.155662D-05	-0.278846D-05	-0.599688D-05	0.303966D-06	-0.678793D-05
39	0.198909D-05	-0.433855D-05	-0.561170D-05	-0.410973D-05	-0.711114D-08
40	0.184724D-03	-0.104598D-03	-0.143693D-03	0.331211D-03	-0.298716D-03
41	-0.374854D-04	-0.163928D-04	-0.898902D-04	0.713706D-04	0.222868D-04
42	0.171579D-04	0.137088D-04	0.204798D-04	0.918317D-05	0.360779D-04
43	0.196898D-03	0.500046D-03	0.253179D-04	0.137569D-04	-0.696134D-03

ESTIMATED COVARIANCE MATRIX FOR PARAMETER ESTIMATES

	26	27	28	29	30
26	0.112237D-03				
27	0.498261D-04	0.453202D-03			
28	-0.922931D-05	-0.116175D-03	0.927983D-03		
29	0.525345D-05	0.109225D-03	-0.110505D-03	0.356613D-03	
30	-0.484956D-05	0.146758D-03	-0.158201D-03	0.135648D-03	0.907359D-03
31	-0.101827D-04	-0.275065D-03	0.204655D-03	-0.174773D-03	-0.304803D-03
32	0.208732D-04	-0.729044D-05	-0.374445D-04	-0.174922D-04	0.245793D-04
33	0.847533D-04	0.173469D-05	0.861191D-04	0.872367D-04	0.721189D-04
34	-0.122581D-04	-0.190823D-04	0.426862D-04	0.990330D-05	-0.706591D-04
35	0.189676D-04	0.325565D-04	-0.782467D-05	0.118502D-04	0.673864D-05
36	0.211460D-04	0.396044D-04	-0.296735D-04	0.204926D-05	-0.303074D-04
37	0.851460D-04	0.108870D-03	-0.169930D-03	0.153008D-03	0.398787D-03
38	-0.214002D-05	-0.251105D-05	0.108564D-04	0.101072D-05	-0.749893D-06
39	-0.178004D-05	-0.818314D-06	0.770067D-05	0.290280D-05	0.891892D-05
40	-0.393277D-05	0.366147D-04	-0.496412D-04	-0.277648D-04	-0.112588D-03
41	-0.323845D-04	0.171032D-04	0.833420D-04	0.168323D-04	-0.558750D-04
42	0.288140D-05	0.771688D-05	-0.248370D-04	0.904603D-05	0.570877D-05
43	0.225285D-03	0.251618D-03	-0.482474D-03	0.417942D-03	0.112227D-02

ESTIMATED COVARIANCE MATRIX FOR PARAMETER ESTIMATES

	31	32	33	34	35
31	0.135244D-02				
32	0.692962D-04	0.189080D-02			
33	0.860388D-04	0.841360D-04	0.236711D-01		
34	0.398431D-04	-0.413385D-04	0.343137D-03	0.323103D-02	
35	0.145093D-05	0.267243D-03	0.141195D-03	0.114292D-03	0.147985D-02
36	-0.172891D-04	0.128948D-03	0.122749D-03	0.895402D-04	0.114334D-03
37	0.887383D-04	-0.639556D-03	0.330981D-03	-0.349064D-03	-0.666265D-05
38	0.702612D-05	-0.483947D-04	0.298716D-04	0.126692D-04	-0.208557D-04
39	-0.910834D-05	0.937075D-05	0.123150D-03	-0.179317D-05	-0.938310D-05
40	0.275531D-03	0.314353D-03	0.658902D-03	-0.888768D-03	-0.441238D-03
41	0.166312D-04	0.434199D-04	-0.291561D-03	0.380820D-03	0.229640D-04
42	0.162513D-04	0.288269D-04	0.117644D-03	-0.995922D-04	0.202814D-04
43	0.241414D-03	-0.175389D-02	0.544998D-03	-0.865619D-03	0.607337D-04

ESTIMATED COVARIANCE MATRIX FOR PARAMETER ESTIMATES

	36	37	38	39	40
36	0.173780D-02				
37	-0.205994D-02	0.482206D-01			
38	-0.134272D-04	0.368179D-06	0.130109D-03		
39	-0.135994D-04	0.545428D-04	0.218853D-05	0.116940D-03	
40	0.207785D-03	-0.489076D-03	-0.296994D-05	0.494244D-04	0.686166D-01
41	0.882577D-04	-0.145096D-02	0.186172D-04	0.589773D-05	0.219876D-02
42	0.854038D-05	-0.371362D-04	0.264623D-05	0.102153D-04	0.289438D-03
43	-0.581069D-02	0.133092D+00	-0.189668D-04	0.121907D-03	-0.139144D-02

ESTIMATED COVARIANCE MATRIX FOR PARAMETER ESTIMATES

	41	42	43
41	0.515744D-02		
42	0.376378D-03	0.621260D-03	
43	-0.396716D-02	-0.808906D-04	0.369124D+00

ESTIMATED CORRELATION MATRIX FOR PARAMETER ESTIMATES

	1	2	3	4	5
1	1.000				

2	0.226	1.000			
3	-0.065	-0.137	1.000		
4	0.072	0.226	-0.198	1.000	
5	-0.054	-0.278	0.144	-0.184	1.000
6	-0.023	-0.034	-0.032	0.018	0.025
7	0.009	-0.034	-0.005	0.012	-0.018
8	-0.013	-0.039	-0.013	0.026	-0.008
9	-0.015	0.002	-0.018	0.001	-0.034
10	0.050	0.004	0.031	-0.017	-0.013
11	0.010	0.093	0.020	0.040	-0.011
12	-0.007	0.039	0.047	-0.003	-0.012
13	0.008	-0.001	-0.036	-0.012	-0.039
14	0.006	-0.019	0.004	0.007	0.020
15	0.027	-0.011	-0.031	0.017	0.033
16	0.018	0.016	-0.014	0.015	-0.007
17	0.015	0.030	0.014	-0.020	0.018
18	0.004	0.021	-0.019	0.063	-0.004
19	0.052	-0.010	-0.003	-0.040	-0.012
20	-0.034	0.027	0.002	0.015	-0.020
21	-0.037	0.003	-0.005	-0.009	0.003
22	0.011	0.018	-0.034	-0.019	0.001
23	0.013	0.003	-0.013	0.000	-0.037
24	0.014	0.032	-0.009	0.005	-0.005
25	-0.011	0.022	-0.048	-0.015	0.022
26	0.061	-0.023	0.005	0.004	0.030
27	0.021	0.004	0.043	0.010	0.042
28	-0.059	0.021	-0.014	0.019	-0.017
29	0.068	0.018	0.028	-0.007	-0.015
30	0.019	0.004	0.032	0.006	-0.033
31	-0.004	-0.018	-0.054	-0.005	-0.026
32	0.047	0.039	0.009	0.001	0.004
33	0.014	-0.011	-0.021	-0.005	0.012
34	0.042	0.131	-0.064	0.082	-0.148
35	0.006	-0.006	0.002	0.048	0.012
36	-0.009	0.008	0.049	-0.003	-0.012
37	-0.011	-0.021	0.020	0.011	-0.037
38	-0.035	0.035	-0.021	0.030	-0.057
39	-0.037	0.012	0.011	-0.068	0.028
40	0.015	-0.014	-0.015	0.001	0.036
41	0.056	0.083	0.016	0.028	-0.013
42	0.041	0.015	-0.010	0.006	-0.020
43	-0.010	-0.018	0.021	0.009	-0.037

ESTIMATED CORRELATION MATRIX FOR PARAMETER ESTIMATES

	6	7	8	9	10
6	1.000				
7	-0.122	1.000			
8	0.016	0.073	1.000		
9	-0.016	-0.178	0.068	1.000	
10	0.016	0.167	-0.111	0.071	1.000
11	-0.032	-0.024	0.038	-0.028	-0.041
12	-0.049	-0.015	-0.023	-0.017	0.001
13	-0.021	0.016	0.067	-0.007	-0.026
14	-0.023	-0.046	-0.012	0.049	-0.035
15	0.031	0.014	-0.002	0.025	0.006
16	0.011	0.013	-0.039	-0.021	0.029
17	-0.054	0.012	-0.028	-0.017	0.048
18	-0.070	0.038	0.045	-0.053	0.007
19	0.009	-0.029	0.022	0.001	-0.028
20	-0.046	0.072	0.018	-0.006	0.080
21	-0.021	0.009	0.049	0.026	0.035
22	-0.015	0.036	0.081	0.020	0.028
23	0.002	-0.065	0.035	0.046	-0.053
24	-0.022	0.014	-0.012	0.000	0.038
25	-0.015	0.036	-0.009	-0.024	0.037
26	0.016	-0.039	-0.044	0.025	0.013
27	0.043	0.005	-0.023	0.000	-0.013
28	0.005	-0.009	-0.031	-0.033	-0.006
29	0.038	-0.065	-0.001	0.040	-0.043
30	-0.021	-0.041	0.026	-0.014	-0.057
31	0.012	0.020	0.015	-0.022	0.024
32	-0.007	-0.165	0.028	-0.115	0.096
33	0.021	-0.010	0.022	-0.014	0.016
34	-0.027	0.029	0.007	-0.040	0.000
35	-0.011	-0.008	0.019	-0.043	0.001
36	-0.007	-0.002	-0.026	-0.030	0.002
37	-0.013	0.000	0.059	-0.009	-0.013
38	0.028	0.010	0.013	0.036	-0.022
39	-0.022	0.041	0.006	-0.068	0.057
40	0.010	0.026	-0.020	-0.052	0.027
41	-0.006	-0.035	-0.028	0.014	0.029
42	0.006	0.010	0.044	-0.027	0.021
43	-0.023	-0.004	0.061	-0.011	-0.014

ESTIMATED CORRELATION MATRIX FOR PARAMETER ESTIMATES

	11	12	13	14	15
11	1.000				
12	0.301	1.000			
13	0.046	0.302	1.000		
14	-0.102	-0.152	-0.028	1.000	
15	-0.004	-0.111	-0.222	0.243	1.000
16	0.017	-0.001	0.021	-0.140	-0.238
17	0.098	0.240	0.016	-0.144	-0.037
18	0.039	0.121	0.334	-0.020	-0.204
19	0.025	-0.014	-0.053	0.062	0.250
20	0.005	0.010	0.028	-0.008	-0.052
21	0.043	0.220	0.049	-0.133	0.018
22	0.016	0.076	0.350	-0.044	-0.178
23	-0.038	-0.023	-0.033	0.098	0.233
24	-0.018	0.050	0.114	-0.044	-0.072
25	-0.009	0.013	0.045	-0.032	-0.033
26	-0.023	-0.255	-0.052	0.135	0.078
27	-0.017	-0.069	-0.372	0.036	0.197
28	0.007	-0.017	0.076	0.003	-0.278
29	-0.027	-0.016	-0.130	0.023	0.079
30	0.011	0.040	-0.074	0.059	0.055
31	0.024	0.039	0.105	-0.053	-0.065
32	0.000	-0.054	-0.019	0.004	-0.043
33	-0.027	-0.087	0.020	0.027	-0.034
34	0.020	-0.005	0.043	-0.008	-0.055
35	-0.026	0.000	-0.007	-0.011	-0.032
36	-0.001	-0.049	0.005	-0.003	-0.013
37	0.007	0.003	0.036	0.040	0.004
38	0.008	-0.024	0.029	-0.012	-0.008
39	0.034	0.021	0.010	-0.015	-0.016
40	-0.015	-0.016	-0.006	0.003	-0.003
41	0.027	0.046	0.054	-0.040	-0.053
42	0.001	-0.020	-0.009	-0.020	0.014
43	0.006	0.003	0.038	0.042	0.002

ESTIMATED CORRELATION MATRIX FOR PARAMETER ESTIMATES

	16	17	18	19	20
16	1.000				
17	0.039	1.000			
18	0.033	0.248	1.000		
19	-0.206	-0.070	-0.189	1.000	

20	0.072	0.076	0.312	-0.259	1.000
21	-0.021	0.228	0.062	0.022	0.024
22	0.034	0.065	0.285	-0.052	0.059
23	-0.256	-0.031	-0.117	0.231	-0.069
24	0.017	0.053	0.281	-0.182	0.337
25	0.008	-0.009	0.083	-0.025	0.037
26	-0.038	-0.214	-0.038	-0.007	-0.005
27	-0.053	-0.066	-0.260	0.039	-0.067
28	0.205	0.010	0.067	-0.215	0.036
29	-0.020	-0.029	-0.312	0.191	-0.301
30	-0.046	-0.010	-0.116	0.009	-0.059
31	0.018	0.021	0.092	-0.049	0.064
32	0.038	-0.002	-0.009	0.012	-0.011
33	0.007	-0.040	0.015	0.007	0.018
34	-0.019	0.015	0.030	-0.007	0.022
35	0.062	-0.041	-0.007	-0.040	0.022
36	0.016	-0.030	-0.016	-0.003	-0.027
37	-0.008	-0.001	0.002	-0.005	-0.059
38	-0.003	0.020	0.035	0.000	0.033
39	0.034	0.002	-0.020	-0.014	0.010
40	0.012	0.031	0.002	0.017	-0.007
41	0.024	0.025	0.009	-0.028	0.034
42	-0.039	-0.005	0.013	0.021	0.025
43	-0.009	-0.002	0.006	-0.003	-0.058

ESTIMATED CORRELATION MATRIX FOR PARAMETER ESTIMATES

	21	22	23	24	25
21	1.000				
22	0.212	1.000			
23	-0.041	-0.187	1.000		
24	0.080	0.283	-0.205	1.000	
25	0.026	0.340	-0.162	0.312	1.000
26	-0.208	-0.061	0.042	-0.035	0.001
27	-0.053	-0.286	0.042	-0.110	-0.077
28	0.005	0.076	-0.226	0.076	0.018
29	-0.005	-0.123	0.065	-0.270	-0.069
30	-0.005	-0.315	0.148	-0.254	-0.336
31	0.018	0.087	-0.053	0.089	0.056
32	-0.045	-0.053	0.008	-0.029	0.004
33	-0.016	0.031	0.007	0.007	0.023
34	0.002	0.011	-0.040	0.072	0.021
35	-0.056	-0.025	0.013	-0.009	0.012

36	-0.037	-0.017	-0.028	0.012	0.045
37	0.024	0.034	0.001	-0.001	-0.023
38	-0.012	-0.010	-0.016	0.001	-0.013
39	0.016	-0.017	-0.016	-0.019	0.000
40	0.060	-0.017	-0.017	0.062	-0.025
41	-0.044	-0.010	-0.039	0.049	0.007
42	0.058	0.023	0.026	0.018	0.032
43	0.028	0.034	0.001	0.001	-0.026

ESTIMATED CORRELATION MATRIX FOR PARAMETER ESTIMATES

	26	27	28	29	30
26	1.000				
27	0.221	1.000			
28	-0.029	-0.179	1.000		
29	0.026	0.272	-0.192	1.000	
30	-0.015	0.229	-0.172	0.238	1.000
31	-0.026	-0.351	0.183	-0.252	-0.275
32	0.045	-0.008	-0.028	-0.021	0.019
33	0.052	0.001	0.018	0.030	0.016
34	-0.020	-0.016	0.025	0.009	-0.041
35	0.047	0.040	-0.007	0.016	0.006
36	0.048	0.045	-0.023	0.003	-0.024
37	0.037	0.023	-0.025	0.037	0.060
38	-0.018	-0.010	0.031	0.005	-0.002
39	-0.016	-0.004	0.023	0.014	0.027
40	-0.001	0.007	-0.006	-0.006	-0.014
41	-0.043	0.011	0.038	0.012	-0.026
42	0.011	0.015	-0.033	0.019	0.008
43	0.035	0.019	-0.026	0.036	0.061

ESTIMATED CORRELATION MATRIX FOR PARAMETER ESTIMATES

	31	32	33	34	35
31	1.000				
32	0.043	1.000			
33	0.015	0.013	1.000		
34	0.019	-0.017	0.039	1.000	
35	0.001	0.160	0.024	0.052	1.000
36	-0.011	0.071	0.019	0.038	0.071
37	0.011	-0.067	0.010	-0.028	-0.001
38	0.017	-0.098	0.017	0.020	-0.048

39	-0.023	0.020	0.074	-0.003	-0.023
40	0.029	0.028	0.016	-0.060	-0.044
41	0.006	0.014	-0.026	0.093	0.008
42	0.018	0.027	0.031	-0.070	0.021
43	0.011	-0.066	0.006	-0.025	0.003

ESTIMATED CORRELATION MATRIX FOR PARAMETER ESTIMATES

	36	37	38	39	40
36	1.000				
37	-0.225	1.000			
38	-0.028	0.000	1.000		
39	-0.030	0.023	0.018	1.000	
40	0.019	-0.009	-0.001	0.017	1.000
41	0.029	-0.092	0.023	0.008	0.117
42	0.008	-0.007	0.009	0.038	0.044
43	-0.229	0.998	-0.003	0.019	-0.009

ESTIMATED CORRELATION MATRIX FOR PARAMETER ESTIMATES

	41	42	43
41	1.000		
42	0.210	1.000	
43	-0.091	-0.005	1.000

TECHNICAL 8 OUTPUT

TECHNICAL 8 OUTPUT FOR BAYES ESTIMATION

CHAIN	BSEED
1	0
2	285380
3	253358

ITERATION	POTENTIAL SCALE REDUCTION	PARAMETER WITH HIGHEST PSR
100	1.009	43
200	1.013	43
300	1.037	43
400	1.020	43

500	1.016	43
600	1.017	43
700	1.018	37
800	1.005	37
900	1.003	37
1000	1.003	39

```
Improvement in physical health, role clarity interaction

SUMMARY OF DATA

     Number of clusters                         57

       Size (s)    Cluster ID with Size s

          2        4 166
          3        65 74 80 30
          4        84 22
          5        205
          6        228
          7        93
          8        106 144 165 71 170 173 192 72 50
          9        40 108
         11        107 83 181 61 75 68
         12        69 174 206 226 81
         13        162
         16        92 154
         19        114
         20        145 2
         22        116
         23        36 156 169 142
         24        152
         26        98
         28        122 202
         30        178
         32        6
         46        24
         59        204
         62        125
         64        209
        108        217
        216        110
        223        100


COVARIANCE COVERAGE OF DATA

Minimum covariance coverage value   0.100

     Number of missing data patterns             4
```

PROPORTION OF DATA PRESENT

Covariance Coverage

	PHYCAT16	PRAFAC	PHY15	KES15	CHOICE15
PHYCAT16	1.000				
PRAFAC	1.000	1.000			
PHY15	1.000	1.000	1.000		
KES15	0.971	0.971	0.971	0.971	
CHOICE15	0.963	0.963	0.963	0.963	0.963
BULLY15	0.966	0.966	0.966	0.966	0.963
PRESS15	0.966	0.966	0.966	0.966	0.963
DUTIES15	0.966	0.966	0.966	0.966	0.963

Covariance Coverage

	BULLY15	PRESS15	DUTIES15
BULLY15	0.966		
PRESS15	0.966	0.966	
DUTIES15	0.966	0.966	0.966

UNIVARIATE PROPORTIONS AND COUNTS FOR CATEGORICAL VARIABLES

PHYCAT16

Category 1	0.364	517.000
Category 2	0.636	905.000

UNIVARIATE SAMPLE STATISTICS

UNIVARIATE HIGHER-ORDER MOMENT DESCRIPTIVE STATISTICS

Variable/ Sample Size	Mean/ Variance	Skewness/ Kurtosis	Minimum/ Maximum	% with Min/Max	Percentiles 20%/60%	40%/80%	Median
PRAFAC	-0.173	0.219	-2.338	1.75%	-1.489	-0.520	-0.258
57.000	1.361	-0.662	2.338	1.75%	0.080	0.848	

```
     PHY15                 0.000       2.446      -0.155   85.58%      -0.155     -0.155     -0.155
            1422.000       0.154       5.391       1.845    1.13%      -0.155     -0.155
     KES15                 0.000       1.141      -0.953    8.62%      -0.620     -0.287     -0.120
            1381.000       0.613       1.071       3.047    0.36%       0.047      0.547
     CHOICE15              0.000      -0.138      -1.878   14.60%      -0.878      0.122      0.122
            1370.000       1.250      -0.758       2.122    5.77%       0.122      1.122
     BULLY15               0.000       2.479      -0.312   79.02%      -0.312     -0.312     -0.312
            1373.000       0.474       6.315       3.688    0.44%      -0.312      0.688
     PRESS15               0.000       0.183      -1.756   14.06%      -0.756     -0.756      0.244
            1373.000       1.207      -0.514       2.244    7.21%       0.244      1.244
     DUTIES15              3.873      -0.896       1.000    3.06%       3.000      4.000      4.000
            1373.000       0.975       0.587       5.000   27.75%       4.000      5.000


THE MODEL ESTIMATION TERMINATED NORMALLY

     USE THE FBITERATIONS OPTION TO INCREASE THE NUMBER OF ITERATIONS BY A FACTOR
     OF AT LEAST TWO TO CHECK CONVERGENCE AND THAT THE PSR VALUE DOES NOT INCREASE.



MODEL FIT INFORMATION

Number of Free Parameters                              43



MODEL RESULTS

                                Posterior  One-Tailed         95% C.I.
                    Estimate       S.D.      P-Value   Lower 2.5%  Upper 2.5%  Significance

Within Level

 PHYCAT16   ON
    PHY15              0.831       0.116      0.000       0.615       1.062      *
    CHOICE15          -0.050       0.033      0.067      -0.116       0.016
    PRESS15            0.001       0.036      0.490      -0.070       0.069
    BULLY15            0.048       0.059      0.205      -0.069       0.162
    KES15              0.166       0.052      0.001       0.059       0.264      *

 KES15    WITH
    PHY15              0.071       0.009      0.000       0.054       0.089      *
    CHOICE15          -0.135       0.024      0.000      -0.183      -0.089      *
```

```
   BULLY15            0.127      0.015      0.000      0.098      0.158      *
   PRESS15            0.206      0.024      0.000      0.160      0.254      *
   DUTIES15          -0.203      0.021      0.000     -0.244     -0.160      *

 PHY15    WITH
   CHOICE15          -0.032      0.012      0.001     -0.057     -0.010      *
   BULLY15            0.013      0.007      0.037     -0.001      0.026
   PRESS15            0.015      0.012      0.108     -0.009      0.037
   DUTIES15          -0.010      0.011      0.171     -0.030      0.010

 CHOICE15 WITH
   BULLY15           -0.133      0.021      0.000     -0.173     -0.094      *
   PRESS15           -0.183      0.032      0.000     -0.248     -0.122      *
   DUTIES15           0.168      0.031      0.000      0.109      0.229      *

 BULLY15  WITH
   PRESS15            0.178      0.020      0.000      0.136      0.217      *
   DUTIES15          -0.139      0.019      0.000     -0.177     -0.103      *

 PRESS15  WITH
   DUTIES15          -0.248      0.030      0.000     -0.308     -0.187      *

 Means
   PHY15              0.000      0.010      0.500     -0.019      0.022
   KES15              0.000      0.021      0.498     -0.042      0.042
   CHOICE15           0.001      0.029      0.487     -0.055      0.056
   BULLY15            0.001      0.019      0.486     -0.038      0.036
   PRESS15            0.000      0.031      0.499     -0.059      0.060

 Variances
   PHY15              0.154      0.006      0.000      0.144      0.166      *
   KES15              0.615      0.024      0.000      0.569      0.661      *
   CHOICE15           1.252      0.047      0.000      1.166      1.351      *
   BULLY15            0.475      0.018      0.000      0.439      0.511      *
   PRESS15            1.208      0.046      0.000      1.120      1.302      *
   DUTIES15           0.955      0.037      0.000      0.887      1.029      *

Between Level

 S1         ON
   PRAFAC            -0.018      0.044      0.336     -0.108      0.064

 PHYCAT16   ON
   PRAFAC            -0.047      0.043      0.129     -0.131      0.039
```

```
    DUTIES15          -0.225       0.227      0.153      -0.679       0.203

 PRAFAC   WITH
    DUTIES15           0.054       0.064      0.190      -0.068       0.189

 Means
    PRAFAC            -0.172       0.154      0.137      -0.471       0.135
    DUTIES15           3.906       0.050      0.000       3.805       4.006      *

 Intercepts
    S1                 0.040       0.048      0.203      -0.062       0.128

 Thresholds
    PHYCAT16$1        -1.264       0.885      0.067      -3.036       0.359

 Variances
    PRAFAC             1.376       0.263      0.000       1.001       2.001      *
    DUTIES15           0.071       0.021      0.000       0.043       0.123      *

 Residual Variances
    PHYCAT16           0.006       0.010      0.000       0.000       0.034      *
    S1                 0.008       0.015      0.000       0.001       0.055      *


STANDARDIZED MODEL RESULTS


STDYX Standardization

                                Posterior  One-Tailed         95% C.I.
                    Estimate       S.D.      P-Value   Lower 2.5%  Upper 2.5%  Significance

Within-Level Standardized Estimates Averaged Over Clusters

 S1 | PHYCAT16 ON
    DUTIES15           0.038       0.040      0.166      -0.042       0.114

 PHYCAT16   ON
    PHY15              0.301       0.039      0.000       0.224       0.377      *
    CHOICE15          -0.051       0.034      0.060      -0.120       0.014
    PRESS15            0.001       0.036      0.487      -0.071       0.068
    BULLY15            0.031       0.036      0.200      -0.041       0.100
    KES15              0.120       0.038      0.000       0.044       0.193      *
```

```
 KES15    WITH
    PHY15              0.230      0.026      0.000      0.178      0.280      *
    CHOICE15          -0.154      0.026      0.000     -0.204     -0.102      *
    BULLY15            0.236      0.026      0.000      0.185      0.285      *
    PRESS15            0.239      0.025      0.000      0.191      0.291      *
    DUTIES15          -0.265      0.025      0.000     -0.312     -0.212      *

 PHY15    WITH
    CHOICE15          -0.074      0.027      0.002     -0.128     -0.023      *
    BULLY15            0.046      0.027      0.039     -0.005      0.097
    PRESS15            0.036      0.028      0.105     -0.019      0.087
    DUTIES15          -0.027      0.028      0.174     -0.081      0.029

 CHOICE15 WITH
    BULLY15           -0.172      0.026      0.000     -0.222     -0.120      *
    PRESS15           -0.149      0.026      0.000     -0.197     -0.094      *
    DUTIES15           0.153      0.027      0.000      0.099      0.204      *

 BULLY15  WITH
    PRESS15            0.234      0.025      0.000      0.184      0.282      *
    DUTIES15          -0.206      0.026      0.000     -0.257     -0.154      *

 PRESS15  WITH
    DUTIES15          -0.230      0.026      0.000     -0.280     -0.178      *

 Means
    PHY15              0.000      0.027      0.500     -0.051      0.055
    KES15              0.000      0.026      0.493     -0.052      0.052
    CHOICE15           0.000      0.026      0.497     -0.051      0.051
    BULLY15            0.000      0.028      0.496     -0.053      0.055
    PRESS15            0.000      0.028      0.496     -0.053      0.054

 Variances
    PHY15              1.000      0.000      0.000      1.000      1.000
    KES15              1.000      0.000      0.000      1.000      1.000
    CHOICE15           1.000      0.000      0.000      1.000      1.000
    BULLY15            1.000      0.000      0.000      1.000      1.000
    PRESS15            1.000      0.000      0.000      1.000      1.000
    DUTIES15           1.000      0.000      0.000      1.000      1.000

Between Level

 S1         ON
    PRAFAC            -0.211      0.462      0.336     -0.924      0.803
```

```
 PHYCAT16   ON
    PRAFAC            -0.415       0.340      0.129      -0.929       0.402
    DUTIES15          -0.471       0.375      0.153      -0.956       0.447

 PRAFAC   WITH
    DUTIES15           0.177       0.185      0.190      -0.209       0.501

 Means
    PRAFAC            -0.144       0.131      0.137      -0.407       0.114
    DUTIES15          14.639       1.974      0.000      11.139      18.906      *

 Intercepts
    S1                 0.369       0.593      0.203      -0.550       1.828

 Variances
    PRAFAC             1.000       0.000      0.000       1.000       1.000
    DUTIES15           1.000       0.000      0.000       1.000       1.000

 Residual Variances
    PHYCAT16           0.363       0.272      0.000       0.015       0.937      *
    S1                 0.850       0.252      0.000       0.126       1.000      *


R-SQUARE

Within-Level R-Square Averaged Across Clusters

                                Posterior  One-Tailed         95% C.I.
    Variable        Estimate       S.D.      P-Value   Lower 2.5%  Upper 2.5%

    PHYCAT16           0.148       0.028      0.000       0.098       0.205

Between Level

                                Posterior  One-Tailed         95% C.I.
    Variable        Estimate       S.D.      P-Value   Lower 2.5%  Upper 2.5%

    PHYCAT16           0.637       0.272      0.000       0.063       0.985

                                Posterior  One-Tailed         95% C.I.
    Variable        Estimate       S.D.      P-Value   Lower 2.5%  Upper 2.5%

    S1                 0.150       0.252      0.000       0.000       0.874
```

TECHNICAL 1 OUTPUT

PARAMETER SPECIFICATION FOR WITHIN

TAU

PHYCAT16
0

NU

PHYCAT16	PHY15	KES15	CHOICE15	BULLY15
0	0	0	0	0

NU

PRESS15	DUTIES15
0	0

LAMBDA

	PHYCAT16	PHY15	KES15	CHOICE15	BULLY15
PHYCAT16	0	0	0	0	0
PHY15	0	0	0	0	0
KES15	0	0	0	0	0
CHOICE15	0	0	0	0	0
BULLY15	0	0	0	0	0
PRESS15	0	0	0	0	0
DUTIES15	0	0	0	0	0

LAMBDA

	PRESS15	DUTIES15
PHYCAT16	0	0
PHY15	0	0
KES15	0	0

CHOICE15	0	0
BULLY15	0	0
PRESS15	0	0
DUTIES15	0	0

THETA

	PHYCAT16	PHY15	KES15	CHOICE15	BULLY15
PHYCAT16	0				
PHY15	0	0			
KES15	0	0	0		
CHOICE15	0	0	0	0	
BULLY15	0	0	0	0	0
PRESS15	0	0	0	0	0
DUTIES15	0	0	0	0	0

THETA

	PRESS15	DUTIES15
PRESS15	0	
DUTIES15	0	0

ALPHA

PHYCAT16	PHY15	KES15	CHOICE15	BULLY15
0	1	2	3	4

ALPHA

PRESS15	DUTIES15
5	0

BETA

	PHYCAT16	PHY15	KES15	CHOICE15	BULLY15
PHYCAT16	0	6	7	8	9
PHY15	0	0	0	0	0
KES15	0	0	0	0	0
CHOICE15	0	0	0	0	0

BULLY15	0	0	0	0	0
PRESS15	0	0	0	0	0
DUTIES15	0	0	0	0	0

BETA

	PRESS15	DUTIES15
PHYCAT16	10	0
PHY15	0	0
KES15	0	0
CHOICE15	0	0
BULLY15	0	0
PRESS15	0	0
DUTIES15	0	0

PSI

	PHYCAT16	PHY15	KES15	CHOICE15	BULLY15
PHYCAT16	0				
PHY15	0	11			
KES15	0	12	13		
CHOICE15	0	14	15	16	
BULLY15	0	17	18	19	20
PRESS15	0	21	22	23	24
DUTIES15	0	26	27	28	29

PSI

	PRESS15	DUTIES15
PRESS15	25	
DUTIES15	30	31

PARAMETER SPECIFICATION FOR BETWEEN

TAU

PHYCAT16
43

NU

PHYCAT16	PRAFAC	DUTIES15
0	0	0

LAMBDA

	S1	PHYCAT16	PRAFAC	DUTIES15
PHYCAT16	0	0	0	0
PRAFAC	0	0	0	0
DUTIES15	0	0	0	0

THETA

	PHYCAT16	PRAFAC	DUTIES15
PHYCAT16	0		
PRAFAC	0	0	
DUTIES15	0	0	0

ALPHA

S1	PHYCAT16	PRAFAC	DUTIES15
32	0	33	34

BETA

	S1	PHYCAT16	PRAFAC	DUTIES15
S1	0	0	35	0
PHYCAT16	0	0	36	37
PRAFAC	0	0	0	0
DUTIES15	0	0	0	0

PSI

	S1	PHYCAT16	PRAFAC	DUTIES15
S1	38			
PHYCAT16	0	39		
PRAFAC	0	0	40	

DUTIES15	0	0	41	42

STARTING VALUES FOR WITHIN

TAU

PHYCAT16
0.000

NU

PHYCAT16	PHY15	KES15	CHOICE15	BULLY15
0.000	0.000	0.000	0.000	0.000

NU

PRESS15	DUTIES15
0.000	0.000

LAMBDA

	PHYCAT16	PHY15	KES15	CHOICE15	BULLY15
PHYCAT16	1.000	0.000	0.000	0.000	0.000
PHY15	0.000	1.000	0.000	0.000	0.000
KES15	0.000	0.000	1.000	0.000	0.000
CHOICE15	0.000	0.000	0.000	1.000	0.000
BULLY15	0.000	0.000	0.000	0.000	1.000
PRESS15	0.000	0.000	0.000	0.000	0.000
DUTIES15	0.000	0.000	0.000	0.000	0.000

LAMBDA

	PRESS15	DUTIES15
PHYCAT16	0.000	0.000
PHY15	0.000	0.000
KES15	0.000	0.000
CHOICE15	0.000	0.000
BULLY15	0.000	0.000

PRESS15	1.000	0.000
DUTIES15	0.000	1.000

THETA

	PHYCAT16	PHY15	KES15	CHOICE15	BULLY15
PHYCAT16	0.000				
PHY15	0.000	0.000			
KES15	0.000	0.000	0.000		
CHOICE15	0.000	0.000	0.000	0.000	
BULLY15	0.000	0.000	0.000	0.000	0.000
PRESS15	0.000	0.000	0.000	0.000	0.000
DUTIES15	0.000	0.000	0.000	0.000	0.000

THETA

	PRESS15	DUTIES15
PRESS15	0.000	
DUTIES15	0.000	0.000

ALPHA

PHYCAT16	PHY15	KES15	CHOICE15	BULLY15
0.000	0.000	0.000	0.000	0.000

ALPHA

PRESS15	DUTIES15
0.000	0.000

BETA

	PHYCAT16	PHY15	KES15	CHOICE15	BULLY15
PHYCAT16	0.000	0.000	0.000	0.000	0.000
PHY15	0.000	0.000	0.000	0.000	0.000
KES15	0.000	0.000	0.000	0.000	0.000
CHOICE15	0.000	0.000	0.000	0.000	0.000
BULLY15	0.000	0.000	0.000	0.000	0.000
PRESS15	0.000	0.000	0.000	0.000	0.000

DUTIES15	0.000	0.000	0.000	0.000	0.000

BETA

	PRESS15	DUTIES15
PHYCAT16	0.000	0.000
PHY15	0.000	0.000
KES15	0.000	0.000
CHOICE15	0.000	0.000
BULLY15	0.000	0.000
PRESS15	0.000	0.000
DUTIES15	0.000	0.000

PSI

	PHYCAT16	PHY15	KES15	CHOICE15	BULLY15
PHYCAT16	1.000				
PHY15	0.000	0.077			
KES15	0.000	0.000	0.306		
CHOICE15	0.000	0.000	0.000	0.625	
BULLY15	0.000	0.000	0.000	0.000	0.237
PRESS15	0.000	0.000	0.000	0.000	0.000
DUTIES15	0.000	0.000	0.000	0.000	0.000

PSI

	PRESS15	DUTIES15
PRESS15	0.604	
DUTIES15	0.000	0.488

STARTING VALUES FOR BETWEEN

TAU

PHYCAT16
-0.311

NU

	PHYCAT16	PRAFAC	DUTIES15
	0.000	0.000	0.000

LAMBDA

	S1	PHYCAT16	PRAFAC	DUTIES15
PHYCAT16	0.000	1.000	0.000	0.000
PRAFAC	0.000	0.000	1.000	0.000
DUTIES15	0.000	0.000	0.000	1.000

THETA

	PHYCAT16	PRAFAC	DUTIES15
PHYCAT16	0.000		
PRAFAC	0.000	0.000	
DUTIES15	0.000	0.000	0.000

ALPHA

S1	PHYCAT16	PRAFAC	DUTIES15
0.000	0.000	-0.078	3.873

BETA

	S1	PHYCAT16	PRAFAC	DUTIES15
S1	0.000	0.000	0.000	0.000
PHYCAT16	0.000	0.000	0.000	0.000
PRAFAC	0.000	0.000	0.000	0.000
DUTIES15	0.000	0.000	0.000	0.000

PSI

	S1	PHYCAT16	PRAFAC	DUTIES15
S1	1.000			
PHYCAT16	0.000	1.000		
PRAFAC	0.000	0.000	0.480	
DUTIES15	0.000	0.000	0.000	0.488

PRIORS FOR ALL PARAMETERS	PRIOR MEAN	PRIOR VARIANCE	PRIOR STD. DEV.
Parameter 1~N(0.000,infinity)	0.0000	infinity	infinity
Parameter 2~N(0.000,infinity)	0.0000	infinity	infinity
Parameter 3~N(0.000,infinity)	0.0000	infinity	infinity
Parameter 4~N(0.000,infinity)	0.0000	infinity	infinity
Parameter 5~N(0.000,infinity)	0.0000	infinity	infinity
Parameter 6~N(0.000,5.000)	0.0000	5.0000	2.2361
Parameter 7~N(0.000,5.000)	0.0000	5.0000	2.2361
Parameter 8~N(0.000,5.000)	0.0000	5.0000	2.2361
Parameter 9~N(0.000,5.000)	0.0000	5.0000	2.2361
Parameter 10~N(0.000,5.000)	0.0000	5.0000	2.2361
Parameter 11~IW(1.000,7)	infinity	infinity	infinity
Parameter 12~IW(0.000,7)	infinity	infinity	infinity
Parameter 13~IW(1.000,7)	infinity	infinity	infinity
Parameter 14~IW(0.000,7)	infinity	infinity	infinity
Parameter 15~IW(0.000,7)	infinity	infinity	infinity
Parameter 16~IW(1.000,7)	infinity	infinity	infinity
Parameter 17~IW(0.000,7)	infinity	infinity	infinity
Parameter 18~IW(0.000,7)	infinity	infinity	infinity
Parameter 19~IW(0.000,7)	infinity	infinity	infinity
Parameter 20~IW(1.000,7)	infinity	infinity	infinity
Parameter 21~IW(0.000,7)	infinity	infinity	infinity
Parameter 22~IW(0.000,7)	infinity	infinity	infinity
Parameter 23~IW(0.000,7)	infinity	infinity	infinity
Parameter 24~IW(0.000,7)	infinity	infinity	infinity
Parameter 25~IW(1.000,7)	infinity	infinity	infinity
Parameter 26~IW(0.000,7)	infinity	infinity	infinity
Parameter 27~IW(0.000,7)	infinity	infinity	infinity
Parameter 28~IW(0.000,7)	infinity	infinity	infinity
Parameter 29~IW(0.000,7)	infinity	infinity	infinity
Parameter 30~IW(0.000,7)	infinity	infinity	infinity
Parameter 31~IW(1.000,7)	infinity	infinity	infinity
Parameter 32~N(0.000,infinity)	0.0000	infinity	infinity
Parameter 33~N(0.000,infinity)	0.0000	infinity	infinity
Parameter 34~N(0.000,infinity)	0.0000	infinity	infinity
Parameter 35~N(0.000,infinity)	0.0000	infinity	infinity
Parameter 36~N(0.000,5.000)	0.0000	5.0000	2.2361
Parameter 37~N(0.000,5.000)	0.0000	5.0000	2.2361
Parameter 38~IG(-1.000,0.000)	infinity	infinity	infinity
Parameter 39~IG(-1.000,0.000)	infinity	infinity	infinity
Parameter 40~IW(1.000,3)	infinity	infinity	infinity

Parameter 41~IW(0.000,3)	infinity	infinity	infinity
Parameter 42~IW(1.000,3)	infinity	infinity	infinity
Parameter 43~N(0.000,5.000)	0.0000	5.0000	2.2361

TECHNICAL 3 OUTPUT

ESTIMATED COVARIANCE MATRIX FOR PARAMETER ESTIMATES

	1	2	3	4	5
1	0.107548D-03				
2	0.472747D-04	0.448125D-03			
3	-0.169815D-04	-0.900467D-04	0.856125D-03		
4	0.124884D-04	0.884145D-04	-0.106037D-03	0.362782D-03	
5	-0.937611D-06	0.132001D-03	-0.122967D-03	0.134683D-03	0.933058D-03
6	-0.262954D-04	-0.659930D-04	-0.117331D-03	0.524247D-04	0.113546D-03
7	0.825416D-05	-0.181406D-04	-0.334072D-06	0.108455D-04	-0.376461D-04
8	-0.518415D-05	-0.301126D-04	-0.146723D-04	0.177988D-04	-0.166252D-04
9	-0.576583D-05	-0.144338D-06	-0.258992D-04	-0.215899D-05	-0.551500D-04
10	0.963618D-05	-0.167766D-04	0.169268D-04	-0.227236D-04	-0.128573D-04
11	0.378788D-06	0.874439D-05	0.528657D-05	0.261078D-05	0.333334D-05
12	-0.963848D-06	0.383610D-05	0.146058D-04	-0.257921D-05	-0.291415D-05
13	0.117587D-05	-0.932783D-05	-0.190016D-04	-0.120239D-04	-0.315741D-04
14	0.932993D-06	-0.639384D-05	0.335430D-05	0.190773D-06	0.420156D-05
15	0.673296D-05	-0.119148D-05	-0.227002D-04	0.109785D-04	0.271215D-04
16	0.845036D-05	0.106522D-04	-0.169544D-04	0.945354D-05	0.584194D-05
17	0.107969D-05	0.371078D-05	0.337462D-05	-0.340224D-05	0.164040D-05
18	0.663579D-06	0.455946D-05	-0.703066D-05	0.173843D-04	0.111503D-04
19	0.104564D-04	-0.843091D-05	0.253869D-05	-0.200146D-04	-0.207010D-04
20	-0.596477D-05	0.152721D-04	-0.263125D-05	0.923321D-05	-0.246124D-06
21	-0.454084D-05	-0.242698D-05	0.388865D-06	-0.339990D-05	-0.174099D-06
22	0.255069D-05	0.160242D-04	-0.247627D-04	-0.574570D-05	0.553567D-05
23	0.425975D-05	-0.312921D-05	-0.121768D-04	-0.873285D-05	-0.457401D-04
24	0.363238D-05	0.141132D-04	-0.104788D-04	0.340035D-05	0.129161D-04
25	-0.586041D-05	0.296008D-04	-0.745299D-04	-0.793801D-05	0.489831D-04
26	0.652261D-05	-0.437620D-05	0.215929D-05	0.211591D-06	0.477838D-05
27	0.331366D-05	-0.868027D-06	0.263276D-04	0.202429D-05	0.289332D-04
28	-0.189644D-04	0.653118D-05	-0.378693D-05	0.100137D-04	-0.295046D-05
29	0.129480D-04	0.582566D-05	0.185003D-04	-0.513757D-05	-0.344548D-05
30	0.747531D-05	0.135162D-05	0.275753D-04	0.443787D-05	-0.246392D-04
31	-0.176786D-05	-0.126942D-04	-0.563603D-04	-0.269030D-05	-0.374664D-04
32	0.262742D-04	0.338125D-04	0.197137D-04	-0.401251D-05	0.725344D-06
33	0.261041D-04	0.846891D-05	-0.139851D-03	0.215102D-04	0.992634D-05

34	0.105491D-04	-0.133955D-03	0.116215D-03	-0.122759D-03	-0.201548D-03
35	0.241992D-05	-0.452385D-05	0.194979D-04	0.408944D-04	0.529338D-04
36	-0.145768D-05	0.251244D-05	0.493576D-04	-0.570173D-05	-0.121241D-04
37	-0.197551D-05	-0.130959D-03	-0.368498D-04	0.163026D-03	-0.176608D-03
38	-0.743268D-05	0.194129D-05	-0.519535D-05	0.293862D-05	0.198931D-05
39	-0.115308D-05	0.544974D-05	-0.224579D-05	-0.763415D-05	0.191760D-05
40	0.464240D-04	-0.376817D-04	-0.178251D-03	0.126520D-04	0.201483D-03
41	0.443014D-04	0.110046D-03	0.271035D-05	0.170936D-04	0.599959D-04
42	0.640688D-05	0.690430D-05	-0.144733D-04	0.798112D-05	0.141451D-04
43	-0.448573D-05	-0.451448D-03	-0.158538D-03	0.646567D-03	-0.650337D-03

ESTIMATED COVARIANCE MATRIX FOR PARAMETER ESTIMATES

	6	7	8	9	10
6	0.135377D-01				
7	-0.726775D-03	0.268953D-02			
8	0.644418D-04	0.107944D-03	0.110968D-02		
9	-0.187756D-03	-0.466762D-03	0.123597D-03	0.342131D-02	
10	0.676401D-04	-0.376153D-03	0.570816D-04	-0.415761D-03	0.126503D-02
11	-0.223385D-04	-0.364947D-05	0.689159D-05	-0.702690D-05	0.606848D-05
12	-0.448875D-04	-0.118148D-04	-0.560266D-05	-0.128314D-04	0.872734D-05
13	-0.475102D-04	0.128714D-04	0.495344D-04	-0.109391D-04	-0.227090D-05
14	-0.271459D-04	-0.193675D-04	-0.661765D-05	0.387107D-04	-0.162252D-04
15	0.826005D-04	0.590350D-05	0.133066D-05	0.301664D-04	-0.179110D-04
16	0.478893D-04	0.603948D-04	-0.595131D-04	-0.508626D-04	0.323896D-04
17	-0.428756D-04	0.239346D-05	-0.667899D-05	-0.111283D-04	0.100026D-04
18	-0.114246D-03	0.271821D-04	0.230375D-04	-0.503109D-04	0.719927D-05
19	0.218212D-04	-0.281837D-04	0.967600D-05	0.303125D-05	-0.411550D-05
20	-0.866551D-04	0.679692D-04	0.157660D-04	-0.116824D-04	0.198846D-04
21	-0.263960D-04	0.310251D-05	0.187008D-04	0.110685D-04	0.758768D-05
22	-0.319878D-04	0.193939D-04	0.665174D-04	0.102490D-04	0.735772D-05
23	-0.268422D-04	-0.116597D-03	0.297299D-04	0.918727D-04	-0.334149D-04
24	-0.300296D-04	0.156817D-06	-0.249209D-05	-0.180482D-04	0.280701D-04
25	-0.185610D-04	0.809822D-04	-0.141131D-04	-0.565311D-04	0.876329D-04
26	0.193952D-04	-0.153575D-04	-0.173884D-04	0.181524D-04	-0.153672D-04
27	0.104970D-03	0.126219D-06	-0.117747D-04	-0.356016D-05	-0.186875D-04
28	0.499999D-04	-0.161189D-04	-0.299394D-04	-0.751440D-04	0.438358D-04
29	0.727157D-04	-0.730910D-04	0.118300D-05	0.379995D-04	-0.165049D-04
30	-0.136649D-03	-0.473598D-04	0.210761D-04	-0.487231D-05	-0.533474D-04
31	0.436704D-04	0.594530D-04	0.152392D-04	-0.345308D-04	0.318325D-04
32	-0.683916D-04	0.381035D-03	-0.156871D-03	0.332687D-03	0.218749D-03
33	0.381156D-03	-0.608374D-04	0.843932D-04	-0.505535D-04	0.690078D-04
34	-0.750627D-04	0.608652D-04	0.353777D-04	-0.113812D-03	0.420672D-04

35	-0.604535D-04	-0.246584D-04	0.379664D-04	0.708965D-04	-0.129982D-04
36	-0.678601D-04	0.667793D-04	-0.323647D-04	-0.666001D-04	0.144201D-05
37	-0.610993D-03	0.173558D-03	0.300217D-03	0.373833D-03	-0.243865D-04
38	0.526574D-04	-0.675397D-05	-0.146984D-05	0.130732D-04	0.243252D-05
39	-0.207398D-04	0.167047D-04	0.955383D-05	-0.424074D-04	0.217285D-04
40	0.371731D-03	0.497143D-03	-0.197341D-03	-0.762032D-03	0.332898D-03
41	-0.322710D-04	-0.847155D-04	-0.226829D-04	0.273009D-04	0.127896D-03
42	0.694007D-05	0.325311D-04	0.163477D-04	-0.151575D-04	0.213687D-04
43	-0.307318D-02	0.531290D-03	0.118102D-02	0.146241D-02	-0.917929D-04

ESTIMATED COVARIANCE MATRIX FOR PARAMETER ESTIMATES

	11	12	13	14	15
11	0.329447D-04				
12	0.152556D-04	0.780238D-04			
13	0.622066D-05	0.633401D-04	0.567444D-03		
14	-0.705567D-05	-0.161779D-04	-0.781118D-05	0.146597D-03	
15	-0.533409D-06	-0.232017D-04	-0.127151D-03	0.702367D-04	0.569307D-03
16	0.469170D-05	-0.784833D-06	0.251868D-04	-0.798225D-04	-0.269620D-03
17	0.402990D-05	0.151318D-04	0.262819D-05	-0.124767D-04	-0.623804D-05
18	0.333140D-05	0.160475D-04	0.120868D-03	-0.369157D-05	-0.746070D-04
19	0.300419D-05	-0.260005D-05	-0.267900D-04	0.156202D-04	0.124120D-03
20	0.531045D-06	0.138034D-05	0.124311D-04	-0.169649D-05	-0.233089D-04
21	0.271891D-05	0.224474D-04	0.130149D-04	-0.208303D-04	0.484339D-05
22	0.168135D-05	0.165039D-04	0.197766D-03	-0.139360D-04	-0.106536D-03
23	-0.545758D-05	-0.295976D-05	-0.197449D-04	0.361768D-04	0.173308D-03
24	-0.226861D-05	0.872867D-05	0.557055D-04	-0.105476D-04	-0.370080D-04
25	-0.280406D-05	0.506994D-05	0.434425D-04	-0.217336D-04	-0.341706D-04
26	-0.163655D-05	-0.233244D-04	-0.127947D-04	0.188264D-04	0.195698D-04
27	-0.181397D-05	-0.140614D-04	-0.183162D-03	0.100736D-04	0.103404D-03
28	0.986529D-06	-0.535929D-05	0.530210D-04	0.173243D-05	-0.199250D-03
29	-0.350005D-05	-0.204001D-05	-0.564758D-04	0.573866D-05	0.368248D-04
30	0.145817D-05	0.908537D-05	-0.506281D-04	0.227708D-04	0.379124D-04
31	0.492977D-05	0.143670D-04	0.892655D-04	-0.223995D-04	-0.581059D-04
32	0.166770D-06	-0.215780D-04	-0.181645D-04	0.707078D-05	-0.465998D-04
33	-0.239068D-04	-0.118232D-03	0.700252D-04	0.503302D-04	-0.125564D-03
34	0.585660D-05	-0.463153D-05	0.425392D-04	-0.163535D-06	-0.314081D-04
35	-0.200362D-05	0.688810D-05	0.507785D-05	0.221930D-05	-0.435872D-04
36	-0.346204D-05	-0.139411D-04	0.207975D-04	-0.395084D-05	-0.207002D-04
37	0.136020D-04	-0.274058D-04	0.165025D-03	0.154702D-03	-0.283281D-04
38	-0.563256D-06	-0.220823D-05	0.210143D-05	-0.443693D-06	0.145755D-05
39	0.204644D-05	0.159486D-05	0.696989D-05	0.638496D-06	-0.868096D-05
40	-0.253461D-04	-0.485908D-04	-0.615267D-04	0.563358D-05	-0.341251D-04

41	0.917467D-05	0.216093D-04	0.706365D-04	-0.459149D-04	-0.714242D-04
42	0.387367D-06	-0.513080D-05	-0.147250D-05	-0.568828D-05	0.260542D-06
43	0.476720D-04	-0.111615D-03	0.667966D-03	0.612039D-03	-0.126371D-03

ESTIMATED COVARIANCE MATRIX FOR PARAMETER ESTIMATES

	16	17	18	19	20
16	0.225196D-02				
17	0.130156D-04	0.511920D-04			
18	0.256221D-04	0.269067D-04	0.230557D-03		
19	-0.204289D-03	-0.104410D-04	-0.598517D-04	0.434898D-03	
20	0.634898D-04	0.987784D-05	0.870869D-04	-0.992995D-04	0.336500D-03
21	-0.989148D-05	0.190217D-04	0.103195D-04	0.714095D-05	0.489667D-05
22	0.421158D-04	0.103962D-04	0.102736D-03	-0.242933D-04	0.254201D-04
23	-0.386398D-03	-0.576206D-05	-0.530077D-04	0.153702D-03	-0.406678D-04
24	0.915114D-05	0.703555D-05	0.842596D-04	-0.787304D-04	0.124800D-03
25	0.174477D-04	-0.229463D-05	0.544475D-04	-0.221651D-04	0.268254D-04
26	-0.198995D-04	-0.157412D-04	-0.585601D-05	-0.376166D-05	-0.237109D-06
27	-0.559153D-04	-0.923089D-05	-0.817747D-04	0.166773D-04	-0.258108D-04
28	0.295558D-03	0.114125D-05	0.311650D-04	-0.134527D-03	0.214130D-04
29	-0.118679D-04	-0.401066D-05	-0.860423D-04	0.774474D-04	-0.101629D-03
30	-0.611275D-04	-0.288412D-05	-0.502268D-04	0.491746D-05	-0.300448D-04
31	0.260178D-04	0.434271D-05	0.471578D-04	-0.373505D-04	0.454995D-04
32	0.891067D-04	-0.624510D-05	-0.135404D-04	0.827544D-05	0.568424D-05
33	0.517762D-04	-0.444621D-04	0.346278D-04	0.214085D-04	0.487609D-04
34	-0.752280D-04	0.480500D-05	0.178608D-04	0.482590D-05	0.255378D-04
35	0.116447D-03	-0.666516D-05	0.484049D-05	-0.506063D-04	0.257348D-04
36	0.470120D-04	-0.110704D-04	-0.147331D-04	0.135642D-05	-0.296200D-04
37	0.204658D-03	-0.580663D-05	0.460273D-04	-0.310155D-03	0.309584D-04
38	0.317094D-05	0.198391D-05	0.583800D-05	0.587702D-05	0.442969D-05
39	0.155001D-04	-0.359583D-06	-0.325410D-05	-0.546976D-05	0.148525D-06
40	0.158214D-03	0.606730D-04	0.141297D-04	0.725293D-04	-0.260362D-04
41	0.469948D-04	0.204307D-04	0.150589D-04	-0.168181D-04	0.352564D-04
42	-0.348805D-04	-0.380724D-06	0.728874D-05	0.133583D-04	0.372219D-05
43	0.746214D-03	-0.262600D-04	0.209729D-03	-0.117503D-02	0.133014D-03

ESTIMATED COVARIANCE MATRIX FOR PARAMETER ESTIMATES

	21	22	23	24	25
21	0.140042D-03				
22	0.601498D-04	0.582103D-03			
23	-0.113308D-04	-0.142515D-03	0.100448D-02		

24	0.166821D-04	0.142174D-03	-0.123098D-03	0.416772D-03	
25	0.116210D-04	0.364165D-03	-0.256404D-03	0.289792D-03	0.207029D-02
26	-0.260138D-04	-0.156289D-04	0.119166D-04	-0.561712D-05	0.338991D-05
27	-0.145396D-04	-0.149524D-03	0.306349D-04	-0.525433D-04	-0.723448D-04
28	-0.613148D-06	0.561209D-04	-0.217628D-03	0.434316D-04	0.297263D-04
29	-0.276254D-06	-0.560284D-04	0.370131D-04	-0.100619D-03	-0.603677D-04
30	-0.924388D-05	-0.229822D-03	0.149225D-03	-0.159755D-03	-0.466145D-03
31	0.142983D-04	0.833516D-04	-0.674626D-04	0.744016D-04	0.104090D-03
32	-0.985713D-05	-0.527972D-04	0.879093D-05	-0.222815D-04	-0.647135D-05
33	-0.233991D-04	0.123295D-03	-0.771689D-06	0.122285D-04	0.152544D-03
34	-0.209043D-05	-0.170206D-04	0.244277D-04	0.398488D-04	-0.497747D-04
35	-0.172462D-04	-0.117946D-04	0.202636D-05	0.815063D-05	0.176402D-04
36	-0.308673D-05	0.458420D-05	-0.746191D-04	0.108369D-04	0.868879D-04
37	-0.501976D-04	0.217731D-03	-0.408880D-03	0.799351D-04	0.136314D-03
38	0.532233D-08	-0.949455D-05	-0.798270D-05	0.300762D-05	-0.717783D-05
39	0.258771D-05	-0.269131D-05	-0.244473D-05	-0.176938D-05	0.204129D-05
40	0.139936D-03	-0.143413D-03	-0.723116D-04	0.314518D-03	-0.392162D-03
41	-0.270653D-04	0.547122D-06	-0.509489D-04	0.647701D-04	-0.370836D-04
42	0.104482D-04	0.200096D-04	0.190089D-05	0.144680D-05	0.365364D-04
43	-0.176962D-03	0.853800D-03	-0.155552D-02	0.340850D-03	0.425924D-03

ESTIMATED COVARIANCE MATRIX FOR PARAMETER ESTIMATES

	26	27	28	29	30
26	0.111788D-03				
27	0.498567D-04	0.451505D-03			
28	-0.781580D-05	-0.120161D-03	0.940726D-03		
29	0.475333D-05	0.111023D-03	-0.118337D-03	0.356857D-03	
30	-0.384476D-05	0.149811D-03	-0.171581D-03	0.140383D-03	0.916933D-03
31	-0.124303D-04	-0.274900D-03	0.226603D-03	-0.176062D-03	-0.316311D-03
32	0.282910D-04	0.664878D-05	-0.391246D-04	-0.171618D-04	0.254707D-04
33	0.744298D-04	-0.903835D-05	0.137232D-03	0.893309D-04	0.947300D-04
34	0.361767D-06	0.856095D-05	-0.541800D-04	0.194688D-04	0.230424D-04
35	0.146105D-04	0.298509D-04	-0.174719D-04	0.196300D-04	0.916450D-05
36	0.173904D-04	0.254371D-04	-0.225581D-04	0.214739D-04	-0.115348D-04
37	0.539513D-04	0.889751D-04	0.271335D-03	0.104738D-04	-0.478326D-04
38	-0.113659D-05	0.285990D-05	0.206229D-04	0.631904D-06	-0.863980D-05
39	-0.854757D-06	-0.881014D-06	0.960622D-05	0.415087D-05	0.128240D-04
40	0.118999D-04	0.948430D-04	-0.643935D-04	-0.452466D-04	-0.129758D-03
41	-0.309726D-04	-0.268325D-05	0.628021D-04	0.938301D-05	-0.343116D-04
42	-0.105760D-04	-0.514505D-05	-0.956635D-05	0.111262D-04	-0.857454D-06
43	0.201950D-03	0.292275D-03	0.102274D-02	0.216207D-04	-0.149207D-03

ESTIMATED COVARIANCE MATRIX FOR PARAMETER ESTIMATES

	31	32	33	34	35
31	0.133889D-02				
32	0.318256D-04	0.228560D-02			
33	0.763500D-04	0.113725D-03	0.236682D-01		
34	0.131760D-05	-0.608287D-04	0.571059D-03	0.253228D-02	
35	-0.483397D-04	0.338953D-03	0.143808D-03	0.949156D-04	0.194781D-02
36	-0.152470D-04	0.195631D-04	-0.705503D-04	0.270039D-04	0.664261D-04
37	0.656001D-03	-0.374841D-03	0.603480D-03	-0.234135D-03	-0.443987D-03
38	0.447282D-05	-0.684968D-04	0.183342D-04	-0.246494D-05	-0.680473D-04
39	-0.137007D-05	0.138989D-04	0.117369D-03	0.590398D-05	-0.165445D-04
40	0.315797D-03	0.293090D-03	0.439115D-03	-0.278705D-03	-0.752446D-03
41	0.137780D-04	-0.637447D-06	-0.209083D-03	0.249927D-03	0.275873D-04
42	0.124709D-04	0.106714D-04	0.102879D-03	0.276957D-04	0.246195D-04
43	0.258870D-02	-0.141592D-02	0.202586D-02	-0.104674D-02	-0.166672D-02

ESTIMATED COVARIANCE MATRIX FOR PARAMETER ESTIMATES

	36	37	38	39	40
36	0.182063D-02				
37	-0.638992D-03	0.516574D-01			
38	-0.150217D-04	-0.753777D-05	0.234493D-03		
39	-0.676580D-05	0.516767D-04	0.484859D-05	0.973309D-04	
40	0.157750D-03	0.890045D-03	0.103756D-03	0.475815D-04	0.693554D-01
41	0.323747D-03	-0.203572D-03	-0.250278D-05	-0.175840D-06	0.228991D-02
42	0.369182D-04	0.257778D-03	0.103265D-04	0.177891D-05	0.163996D-03
43	-0.260668D-02	0.200742D+00	-0.648085D-04	0.170791D-03	0.348209D-02

ESTIMATED COVARIANCE MATRIX FOR PARAMETER ESTIMATES

	41	42	43
41	0.412420D-02		
42	0.453370D-03	0.425681D-03	
43	-0.698838D-03	0.998859D-03	0.781941D+00

ESTIMATED CORRELATION MATRIX FOR PARAMETER ESTIMATES

	1	2	3	4	5
1	1.000				

2	0.215	1.000			
3	-0.056	-0.145	1.000		
4	0.063	0.219	-0.190	1.000	
5	-0.003	0.204	-0.138	0.231	1.000
6	-0.022	-0.027	-0.034	0.024	0.032
7	0.015	-0.017	0.000	0.011	-0.024
8	-0.015	-0.043	-0.015	0.028	-0.016
9	-0.010	0.000	-0.015	-0.002	-0.031
10	0.026	-0.022	0.016	-0.034	-0.012
11	0.006	0.072	0.031	0.024	0.019
12	-0.011	0.021	0.057	-0.015	-0.011
13	0.005	-0.018	-0.027	-0.027	-0.043
14	0.007	-0.025	0.009	0.001	0.011
15	0.027	-0.002	-0.033	0.024	0.037
16	0.017	0.011	-0.012	0.010	0.004
17	0.015	0.024	0.016	-0.025	0.008
18	0.004	0.014	-0.016	0.060	0.024
19	0.048	-0.019	0.004	-0.050	-0.032
20	-0.031	0.039	-0.005	0.026	0.000
21	-0.037	-0.010	0.001	-0.015	0.000
22	0.010	0.031	-0.035	-0.013	0.008
23	0.013	-0.005	-0.013	-0.014	-0.047
24	0.017	0.033	-0.018	0.009	0.021
25	-0.012	0.031	-0.056	-0.009	0.035
26	0.059	-0.020	0.007	0.001	0.015
27	0.015	-0.002	0.042	0.005	0.045
28	-0.060	0.010	-0.004	0.017	-0.003
29	0.066	0.015	0.033	-0.014	-0.006
30	0.024	0.002	0.031	0.008	-0.027
31	-0.005	-0.016	-0.053	-0.004	-0.034
32	0.053	0.033	0.014	-0.004	0.000
33	0.016	0.003	-0.031	0.007	0.002
34	0.020	-0.126	0.079	-0.128	-0.131
35	0.005	-0.005	0.015	0.049	0.039
36	-0.003	0.003	0.040	-0.007	-0.009
37	-0.001	-0.027	-0.006	0.038	-0.025
38	-0.047	0.006	-0.012	0.010	0.004
39	-0.011	0.026	-0.008	-0.041	0.006
40	0.017	-0.007	-0.023	0.003	0.025
41	0.067	0.081	0.001	0.014	0.031
42	0.030	0.016	-0.024	0.020	0.022
43	0.000	-0.024	-0.006	0.038	-0.024

ESTIMATED CORRELATION MATRIX FOR PARAMETER ESTIMATES

	6	7	8	9	10
6	1.000				
7	-0.120	1.000			
8	0.017	0.062	1.000		
9	-0.028	-0.154	0.063	1.000	
10	0.016	-0.204	0.048	-0.200	1.000
11	-0.033	-0.012	0.036	-0.021	0.030
12	-0.044	-0.026	-0.019	-0.025	0.028
13	-0.017	0.010	0.062	-0.008	-0.003
14	-0.019	-0.031	-0.016	0.055	-0.038
15	0.030	0.005	0.002	0.022	-0.021
16	0.009	0.025	-0.038	-0.018	0.019
17	-0.052	0.006	-0.028	-0.027	0.039
18	-0.065	0.035	0.046	-0.057	0.013
19	0.009	-0.026	0.014	0.002	-0.006
20	-0.041	0.071	0.026	-0.011	0.030
21	-0.019	0.005	0.047	0.016	0.018
22	-0.011	0.015	0.083	0.007	0.009
23	-0.007	-0.071	0.028	0.050	-0.030
24	-0.013	0.000	-0.004	-0.015	0.039
25	-0.004	0.034	-0.009	-0.021	0.054
26	0.016	-0.028	-0.049	0.029	-0.041
27	0.042	0.000	-0.017	-0.003	-0.025
28	0.014	-0.010	-0.029	-0.042	0.040
29	0.033	-0.075	0.002	0.034	-0.025
30	-0.039	-0.030	0.021	-0.003	-0.050
31	0.010	0.031	0.013	-0.016	0.024
32	-0.012	0.154	-0.099	0.119	0.129
33	0.021	-0.008	0.016	-0.006	0.013
34	-0.013	0.023	0.021	-0.039	0.024
35	-0.012	-0.011	0.026	0.027	-0.008
36	-0.014	0.030	-0.023	-0.027	0.001
37	-0.023	0.015	0.040	0.028	-0.003
38	0.030	-0.009	-0.003	0.015	0.004
39	-0.018	0.033	0.029	-0.073	0.062
40	0.012	0.036	-0.022	-0.049	0.036
41	-0.004	-0.025	-0.011	0.007	0.056
42	0.003	0.030	0.024	-0.013	0.029
43	-0.030	0.012	0.040	0.028	-0.003

ESTIMATED CORRELATION MATRIX FOR PARAMETER ESTIMATES

	11	12	13	14	15
11	1.000				
12	0.301	1.000			
13	0.045	0.301	1.000		
14	-0.102	-0.151	-0.027	1.000	
15	-0.004	-0.110	-0.224	0.243	1.000
16	0.017	-0.002	0.022	-0.139	-0.238
17	0.098	0.239	0.015	-0.144	-0.037
18	0.038	0.120	0.334	-0.020	-0.206
19	0.025	-0.014	-0.054	0.062	0.249
20	0.005	0.009	0.028	-0.008	-0.053
21	0.040	0.215	0.046	-0.145	0.017
22	0.012	0.077	0.344	-0.048	-0.185
23	-0.030	-0.011	-0.026	0.094	0.229
24	-0.019	0.048	0.115	-0.043	-0.076
25	-0.011	0.013	0.040	-0.039	-0.031
26	-0.027	-0.250	-0.051	0.147	0.078
27	-0.015	-0.075	-0.362	0.039	0.204
28	0.006	-0.020	0.073	0.005	-0.272
29	-0.032	-0.012	-0.126	0.025	0.082
30	0.008	0.034	-0.070	0.062	0.052
31	0.023	0.044	0.102	-0.051	-0.067
32	0.001	-0.051	-0.016	0.012	-0.041
33	-0.027	-0.087	0.019	0.027	-0.034
34	0.020	-0.010	0.035	0.000	-0.026
35	-0.008	0.018	0.005	0.004	-0.041
36	-0.014	-0.037	0.020	-0.008	-0.020
37	0.010	-0.014	0.030	0.056	-0.005
38	-0.006	-0.016	0.006	-0.002	0.004
39	0.036	0.018	0.030	0.005	-0.037
40	-0.017	-0.021	-0.010	0.002	-0.005
41	0.025	0.038	0.046	-0.059	-0.047
42	0.003	-0.028	-0.003	-0.023	0.001
43	0.009	-0.014	0.032	0.057	-0.006

ESTIMATED CORRELATION MATRIX FOR PARAMETER ESTIMATES

	16	17	18	19	20
16	1.000				
17	0.038	1.000			
18	0.036	0.248	1.000		
19	-0.206	-0.070	-0.189	1.000	

20	0.073	0.075	0.313	-0.260	1.000
21	-0.018	0.225	0.057	0.029	0.023
22	0.037	0.060	0.280	-0.048	0.057
23	-0.257	-0.025	-0.110	0.233	-0.070
24	0.009	0.048	0.272	-0.185	0.333
25	0.008	-0.007	0.079	-0.023	0.032
26	-0.040	-0.208	-0.036	-0.017	-0.001
27	-0.055	-0.061	-0.253	0.038	-0.066
28	0.203	0.005	0.067	-0.210	0.038
29	-0.013	-0.030	-0.300	0.197	-0.293
30	-0.043	-0.013	-0.109	0.008	-0.054
31	0.015	0.017	0.085	-0.049	0.068
32	0.039	-0.018	-0.019	0.008	0.006
33	0.007	-0.040	0.015	0.007	0.017
34	-0.032	0.013	0.023	0.005	0.028
35	0.056	-0.021	0.007	-0.055	0.032
36	0.023	-0.036	-0.023	0.002	-0.038
37	0.019	-0.004	0.013	-0.065	0.007
38	0.004	0.018	0.025	0.018	0.016
39	0.033	-0.005	-0.022	-0.027	0.001
40	0.013	0.032	0.004	0.013	-0.005
41	0.015	0.044	0.015	-0.013	0.030
42	-0.036	-0.003	0.023	0.031	0.010
43	0.018	-0.004	0.016	-0.064	0.008

ESTIMATED CORRELATION MATRIX FOR PARAMETER ESTIMATES

	21	22	23	24	25
21	1.000				
22	0.211	1.000			
23	-0.030	-0.186	1.000		
24	0.069	0.289	-0.190	1.000	
25	0.022	0.332	-0.178	0.312	1.000
26	-0.208	-0.061	0.036	-0.026	0.007
27	-0.058	-0.292	0.045	-0.121	-0.075
28	-0.002	0.076	-0.224	0.069	0.021
29	-0.001	-0.123	0.062	-0.261	-0.070
30	-0.026	-0.315	0.155	-0.258	-0.338
31	0.033	0.094	-0.058	0.100	0.063
32	-0.017	-0.046	0.006	-0.023	-0.003
33	-0.013	0.033	0.000	0.004	0.022
34	-0.004	-0.014	0.015	0.039	-0.022
35	-0.033	-0.011	0.001	0.009	0.009

36	-0.006	0.004	-0.055	0.012	0.045
37	-0.019	0.040	-0.057	0.017	0.013
38	0.000	-0.026	-0.016	0.010	-0.010
39	0.022	-0.011	-0.008	-0.009	0.005
40	0.045	-0.023	-0.009	0.059	-0.033
41	-0.036	0.000	-0.025	0.049	-0.013
42	0.043	0.040	0.003	0.003	0.039
43	-0.017	0.040	-0.056	0.019	0.011

ESTIMATED CORRELATION MATRIX FOR PARAMETER ESTIMATES

	26	27	28	29	30
26	1.000				
27	0.222	1.000			
28	-0.024	-0.184	1.000		
29	0.024	0.277	-0.204	1.000	
30	-0.012	0.233	-0.185	0.245	1.000
31	-0.032	-0.354	0.202	-0.255	-0.285
32	0.056	0.007	-0.027	-0.019	0.018
33	0.046	-0.003	0.029	0.031	0.020
34	0.001	0.008	-0.035	0.020	0.015
35	0.031	0.032	-0.013	0.024	0.007
36	0.039	0.028	-0.017	0.027	-0.009
37	0.022	0.018	0.039	0.002	-0.007
38	-0.007	0.009	0.044	0.002	-0.019
39	-0.008	-0.004	0.032	0.022	0.043
40	0.004	0.017	-0.008	-0.009	-0.016
41	-0.046	-0.002	0.032	0.008	-0.018
42	-0.048	-0.012	-0.015	0.029	-0.001
43	0.022	0.016	0.038	0.001	-0.006

ESTIMATED CORRELATION MATRIX FOR PARAMETER ESTIMATES

	31	32	33	34	35
31	1.000				
32	0.018	1.000			
33	0.014	0.015	1.000		
34	0.001	-0.025	0.074	1.000	
35	-0.030	0.161	0.021	0.043	1.000
36	-0.010	0.010	-0.011	0.013	0.035
37	0.079	-0.034	0.017	-0.020	-0.044
38	0.008	-0.094	0.008	-0.003	-0.101

39	-0.004	0.029	0.077	0.012	-0.038
40	0.033	0.023	0.011	-0.021	-0.065
41	0.006	0.000	-0.021	0.077	0.010
42	0.017	0.011	0.032	0.027	0.027
43	0.080	-0.033	0.015	-0.024	-0.043

ESTIMATED CORRELATION MATRIX FOR PARAMETER ESTIMATES

	36	37	38	39	40
36	1.000				
37	-0.066	1.000			
38	-0.023	-0.002	1.000		
39	-0.016	0.023	0.032	1.000	
40	0.014	0.015	0.026	0.018	1.000
41	0.118	-0.014	-0.003	0.000	0.135
42	0.042	0.055	0.033	0.009	0.030
43	-0.069	0.999	-0.005	0.020	0.015

ESTIMATED CORRELATION MATRIX FOR PARAMETER ESTIMATES

	41	42	43
41	1.000		
42	0.342	1.000	
43	-0.012	0.055	1.000

TECHNICAL 8 OUTPUT

TECHNICAL 8 OUTPUT FOR BAYES ESTIMATION

CHAIN	BSEED
1	0
2	285380
3	253358

ITERATION	POTENTIAL SCALE REDUCTION	PARAMETER WITH HIGHEST PSR
100	1.023	38
200	1.011	39
300	1.014	38
400	1.014	35

500	1.008	35
600	1.004	39
700	1.006	37
800	1.003	37
900	1.001	33
1000	1.003	14

Worsening of psychological distress, job control interaction

SUMMARY OF DATA

Number of clusters 58

Size (s)	Cluster ID with Size s
7	22
17	166
20	80
26	71 74 205
29	65
31	192
33	228
37	182 106
39	72
41	170 181
42	50 30
43	75
45	173
47	226 165
48	108
50	107 114
51	81
52	40
55	84 92
57	162
59	206
61	61 83 116
62	36 93 169
63	69
64	4
69	68
73	174 152
74	6 154
75	145
89	98
90	178 2
95	144
100	156
104	202
106	142
134	122

186	24
192	209
246	204
278	125
405	217
780	110
873	100

COVARIANCE COVERAGE OF DATA

Minimum covariance coverage value 0.100

Number of missing data patterns 9

PROPORTION OF DATA PRESENT

Covariance Coverage

	KESCAT16	PRAFAC	PHY15	KES15	CHOICE15
KESCAT16	1.000				
PRAFAC	1.000	1.000			
PHY15	0.950	0.950	0.950		
KES15	0.919	0.919	0.919	0.919	
CHOICE15	0.913	0.913	0.913	0.913	0.913
BULLY15	0.913	0.913	0.913	0.913	0.912
PRESS15	0.913	0.913	0.913	0.913	0.913
DUTIES15	0.913	0.913	0.913	0.913	0.913

Covariance Coverage

	BULLY15	PRESS15	DUTIES15
BULLY15	0.913		
PRESS15	0.912	0.913	
DUTIES15	0.913	0.913	0.913

UNIVARIATE PROPORTIONS AND COUNTS FOR CATEGORICAL VARIABLES

KESCAT16

Category 1	0.877	5135.000
Category 2	0.123	720.000

UNIVARIATE SAMPLE STATISTICS

UNIVARIATE HIGHER-ORDER MOMENT DESCRIPTIVE STATISTICS

Variable/ Sample Size	Mean/ Variance	Skewness/ Kurtosis	Minimum/ Maximum	% with Min/Max	Percentiles 20%/60%	40%/80%	Median
PRAFAC	-0.185	0.245	-2.338	1.72%	-1.448	-0.527	-0.318
58.000	1.345	-0.636	2.338	1.72%	0.080	0.752	
PHY15	0.000	0.526	-0.865	32.48%	-0.865	0.135	0.135
5563.000	0.536	0.021	3.135	0.07%	0.135	0.135	
KES15	0.000	0.475	-0.374	22.78%	-0.374	-0.207	-0.041
5382.000	0.095	-0.842	0.626	6.60%	-0.041	0.293	
CHOICE15	3.221	-0.400	1.000	7.67%	2.000	3.000	3.000
5345.000	1.097	-0.356	5.000	8.62%	4.000	4.000	
BULLY15	0.000	3.522	-0.153	88.29%	-0.153	-0.153	-0.153
5344.000	0.216	14.164	3.847	0.09%	-0.153	-0.153	
PRESS15	0.000	0.267	-1.445	19.70%	-0.445	-0.445	-0.445
5345.000	1.001	-0.357	2.555	2.75%	0.555	0.555	
DUTIES15	0.000	-1.296	-3.149	2.34%	-0.149	-0.149	-0.149
5347.000	0.828	1.939	0.851	39.74%	-0.149	0.851	

THE MODEL ESTIMATION TERMINATED NORMALLY

USE THE FBITERATIONS OPTION TO INCREASE THE NUMBER OF ITERATIONS BY A FACTOR
OF AT LEAST TWO TO CHECK CONVERGENCE AND THAT THE PSR VALUE DOES NOT INCREASE.

MODEL FIT INFORMATION

Number of Free Parameters 43

MODEL RESULTS

	Estimate	Posterior S.D.	One-Tailed P-Value	95% C.I. Lower 2.5%	Upper 2.5%	Significance
**Within Level**						
KESCAT16 ON						
PHY15	0.138	0.033	0.000	0.071	0.202	*
BULLY15	0.111	0.048	0.009	0.017	0.205	*
DUTIES15	-0.081	0.028	0.001	-0.136	-0.030	*
PRESS15	-0.033	0.026	0.094	-0.085	0.017	
KES15	1.622	0.087	0.000	1.453	1.798	*
KES15 WITH						
PHY15	0.054	0.003	0.000	0.048	0.061	*
CHOICE15	-0.038	0.004	0.000	-0.047	-0.029	*
BULLY15	0.022	0.002	0.000	0.018	0.026	*
PRESS15	0.053	0.004	0.000	0.045	0.062	*
DUTIES15	-0.051	0.004	0.000	-0.058	-0.043	*
PHY15 WITH						
CHOICE15	-0.087	0.010	0.000	-0.106	-0.065	*
BULLY15	0.025	0.005	0.000	0.015	0.034	*
PRESS15	0.063	0.010	0.000	0.042	0.083	*
DUTIES15	-0.056	0.009	0.000	-0.074	-0.038	*
CHOICE15 WITH						
BULLY15	-0.049	0.007	0.000	-0.062	-0.037	*
PRESS15	-0.067	0.015	0.000	-0.095	-0.036	*
DUTIES15	0.154	0.013	0.000	0.130	0.182	*
BULLY15 WITH						
PRESS15	0.091	0.006	0.000	0.078	0.103	*
DUTIES15	-0.058	0.006	0.000	-0.070	-0.047	*
PRESS15 WITH						
DUTIES15	-0.156	0.013	0.000	-0.181	-0.131	*
Means						
PHY15	0.002	0.010	0.435	-0.018	0.021	
KES15	0.003	0.004	0.217	-0.005	0.011	
BULLY15	0.001	0.006	0.425	-0.011	0.014	
PRESS15	0.001	0.013	0.457	-0.025	0.027	
DUTIES15	-0.003	0.013	0.409	-0.029	0.022	

```
 Variances
    PHY15              0.537       0.010      0.000       0.516       0.557      *
    KES15              0.096       0.002      0.000       0.092       0.100      *
    CHOICE15           1.056       0.021      0.000       1.020       1.099      *
    BULLY15            0.216       0.004      0.000       0.208       0.225      *
    PRESS15            1.002       0.020      0.000       0.966       1.042      *
    DUTIES15           0.829       0.016      0.000       0.799       0.860      *

Between Level

 S1         ON
    PRAFAC             0.007       0.031      0.403      -0.051       0.072

 KESCAT16   ON
    PRAFAC             0.017       0.029      0.282      -0.037       0.072
    CHOICE15          -0.141       0.166      0.195      -0.464       0.178

 PRAFAC   WITH
    CHOICE15           0.027       0.046      0.264      -0.064       0.119

 Means
    PRAFAC            -0.182       0.152      0.120      -0.485       0.109
    CHOICE15           3.305       0.037      0.000       3.234       3.379      *

 Intercepts
    S1                -0.057       0.034      0.059      -0.123       0.011

 Thresholds
    KESCAT16$1         0.898       0.540      0.045      -0.159       1.961

 Variances
    PRAFAC             1.359       0.269      0.000       0.962       2.009      *
    CHOICE15           0.057       0.013      0.000       0.037       0.088      *

 Residual Variances
    KESCAT16           0.010       0.008      0.000       0.001       0.033      *
    S1                 0.009       0.010      0.000       0.001       0.039      *


STANDARDIZED MODEL RESULTS


STDYX Standardization
```

```
                              Posterior  One-Tailed         95% C.I.
                    Estimate       S.D.      P-Value   Lower 2.5%  Upper 2.5%  Significance

Within-Level Standardized Estimates Averaged Over Clusters

 S1 | KESCAT16 ON
    CHOICE15          -0.051       0.026      0.028      -0.101       0.001

 KESCAT16   ON
    PHY15              0.087       0.021      0.000       0.046       0.129      *
    BULLY15            0.044       0.019      0.009       0.006       0.080      *
    DUTIES15          -0.065       0.022      0.002      -0.107      -0.023      *
    PRESS15           -0.029       0.022      0.090      -0.073       0.015
    KES15              0.434       0.019      0.000       0.395       0.471      *

 KES15    WITH
    PHY15              0.240       0.013      0.000       0.214       0.264      *
    CHOICE15          -0.119       0.014      0.000      -0.146      -0.092      *
    BULLY15            0.154       0.013      0.000       0.128       0.180      *
    PRESS15            0.171       0.013      0.000       0.145       0.198      *
    DUTIES15          -0.180       0.013      0.000      -0.205      -0.154      *

 PHY15    WITH
    CHOICE15          -0.115       0.014      0.000      -0.142      -0.088      *
    BULLY15            0.072       0.014      0.000       0.045       0.098      *
    PRESS15            0.086       0.014      0.000       0.059       0.112      *
    DUTIES15          -0.084       0.014      0.000      -0.111      -0.058      *

 CHOICE15 WITH
    BULLY15           -0.103       0.014      0.000      -0.129      -0.076      *
    PRESS15           -0.065       0.014      0.000      -0.092      -0.036      *
    DUTIES15           0.165       0.014      0.000       0.139       0.193      *

 BULLY15  WITH
    PRESS15            0.195       0.013      0.000       0.168       0.220      *
    DUTIES15          -0.138       0.013      0.000      -0.164      -0.112      *

 PRESS15  WITH
    DUTIES15          -0.171       0.013      0.000      -0.197      -0.144      *

 Means
    PHY15              0.002       0.014      0.427      -0.024       0.028
    KES15              0.010       0.013      0.230      -0.017       0.037
    BULLY15            0.003       0.014      0.429      -0.024       0.029
```

```
    PRESS15            0.002       0.013      0.449      -0.024       0.028
    DUTIES15          -0.003       0.014      0.412      -0.032       0.025

 Variances
    PHY15              1.000       0.000      0.000       1.000       1.000
    KES15              1.000       0.000      0.000       1.000       1.000
    CHOICE15           1.000       0.000      0.000       1.000       1.000
    BULLY15            1.000       0.000      0.000       1.000       1.000
    PRESS15            1.000       0.000      0.000       1.000       1.000
    DUTIES15           1.000       0.000      0.000       1.000       1.000

Between Level

 S1         ON
    PRAFAC             0.083       0.359      0.403      -0.657       0.767

 KESCAT16   ON
    PRAFAC             0.165       0.276      0.282      -0.394       0.635
    CHOICE15          -0.295       0.319      0.195      -0.880       0.376

 PRAFAC   WITH
    CHOICE15           0.096       0.154      0.264      -0.226       0.383

 Means
    PRAFAC            -0.157       0.129      0.120      -0.416       0.094
    CHOICE15          13.889       1.529      0.000      11.190      17.251      *

 Intercepts
    S1                -0.545       0.497      0.059      -1.891       0.086

 Variances
    PRAFAC             1.000       0.000      0.000       1.000       1.000
    CHOICE15           1.000       0.000      0.000       1.000       1.000

 Residual Variances
    KESCAT16           0.785       0.217      0.000       0.172       0.991      *
    S1                 0.938       0.176      0.000       0.318       1.000      *


R-SQUARE

Within-Level R-Square Averaged Across Clusters

                                Posterior  One-Tailed         95% C.I.
```

```
    Variable        Estimate       S.D.      P-Value   Lower 2.5%  Upper 2.5%

    KESCAT16           0.255       0.018      0.000       0.220       0.291

Between Level

                               Posterior  One-Tailed         95% C.I.
    Variable        Estimate      S.D.      P-Value   Lower 2.5%  Upper 2.5%

    KESCAT16           0.215       0.217      0.000       0.009       0.828

                               Posterior  One-Tailed         95% C.I.
    Variable        Estimate      S.D.      P-Value   Lower 2.5%  Upper 2.5%

    S1                 0.062       0.176      0.000       0.000       0.682


TECHNICAL 1 OUTPUT


     PARAMETER SPECIFICATION FOR WITHIN


           TAU
              KESCAT16
              ________
                    0


           NU
              KESCAT16      PHY15         KES15         CHOICE15      BULLY15
              ________      ________      ________      ________      ________
                    0             0             0             0             0


           NU
              PRESS15       DUTIES15
              ________      ________
                    0             0


           LAMBDA
              KESCAT16      PHY15         KES15         CHOICE15      BULLY15
              ________      ________      ________      ________      ________
```

KESCAT16	0	0	0	0	0
PHY15	0	0	0	0	0
KES15	0	0	0	0	0
CHOICE15	0	0	0	0	0
BULLY15	0	0	0	0	0
PRESS15	0	0	0	0	0
DUTIES15	0	0	0	0	0

LAMBDA

	PRESS15	DUTIES15
KESCAT16	0	0
PHY15	0	0
KES15	0	0
CHOICE15	0	0
BULLY15	0	0
PRESS15	0	0
DUTIES15	0	0

THETA

	KESCAT16	PHY15	KES15	CHOICE15	BULLY15
KESCAT16	0				
PHY15	0	0			
KES15	0	0	0		
CHOICE15	0	0	0	0	
BULLY15	0	0	0	0	0
PRESS15	0	0	0	0	0
DUTIES15	0	0	0	0	0

THETA

	PRESS15	DUTIES15
PRESS15	0	
DUTIES15	0	0

ALPHA

KESCAT16	PHY15	KES15	CHOICE15	BULLY15
0	1	2	0	3

ALPHA

PRESS15	DUTIES15
4	5

BETA

	KESCAT16	PHY15	KES15	CHOICE15	BULLY15
KESCAT16	0	6	7	0	8
PHY15	0	0	0	0	0
KES15	0	0	0	0	0
CHOICE15	0	0	0	0	0
BULLY15	0	0	0	0	0
PRESS15	0	0	0	0	0
DUTIES15	0	0	0	0	0

BETA

	PRESS15	DUTIES15
KESCAT16	9	10
PHY15	0	0
KES15	0	0
CHOICE15	0	0
BULLY15	0	0
PRESS15	0	0
DUTIES15	0	0

PSI

	KESCAT16	PHY15	KES15	CHOICE15	BULLY15
KESCAT16	0				
PHY15	0	11			
KES15	0	12	13		
CHOICE15	0	14	15	16	
BULLY15	0	17	18	19	20
PRESS15	0	21	22	23	24
DUTIES15	0	26	27	28	29

PSI

	PRESS15	DUTIES15
PRESS15	25	
DUTIES15	30	31

PARAMETER SPECIFICATION FOR BETWEEN

TAU

KESCAT16
43

NU

KESCAT16	PRAFAC	CHOICE15
0	0	0

LAMBDA

	S1	KESCAT16	PRAFAC	CHOICE15
KESCAT16	0	0	0	0
PRAFAC	0	0	0	0
CHOICE15	0	0	0	0

THETA

	KESCAT16	PRAFAC	CHOICE15
KESCAT16	0		
PRAFAC	0	0	
CHOICE15	0	0	0

ALPHA

S1	KESCAT16	PRAFAC	CHOICE15
32	0	33	34

BETA

	S1	KESCAT16	PRAFAC	CHOICE15
S1	0	0	35	0
KESCAT16	0	0	36	37
PRAFAC	0	0	0	0
CHOICE15	0	0	0	0

PSI

	S1	KESCAT16	PRAFAC	CHOICE15
S1	38			
KESCAT16	0	39		
PRAFAC	0	0	40	
CHOICE15	0	0	41	42

STARTING VALUES FOR WITHIN

TAU

KESCAT16
0.000

NU

KESCAT16	PHY15	KES15	CHOICE15	BULLY15
0.000	0.000	0.000	0.000	0.000

NU

PRESS15	DUTIES15
0.000	0.000

LAMBDA

	KESCAT16	PHY15	KES15	CHOICE15	BULLY15
KESCAT16	1.000	0.000	0.000	0.000	0.000
PHY15	0.000	1.000	0.000	0.000	0.000

KES15	0.000	0.000	1.000	0.000	0.000
CHOICE15	0.000	0.000	0.000	1.000	0.000
BULLY15	0.000	0.000	0.000	0.000	1.000
PRESS15	0.000	0.000	0.000	0.000	0.000
DUTIES15	0.000	0.000	0.000	0.000	0.000

LAMBDA

	PRESS15	DUTIES15
KESCAT16	0.000	0.000
PHY15	0.000	0.000
KES15	0.000	0.000
CHOICE15	0.000	0.000
BULLY15	0.000	0.000
PRESS15	1.000	0.000
DUTIES15	0.000	1.000

THETA

	KESCAT16	PHY15	KES15	CHOICE15	BULLY15
KESCAT16	0.000				
PHY15	0.000	0.000			
KES15	0.000	0.000	0.000		
CHOICE15	0.000	0.000	0.000	0.000	
BULLY15	0.000	0.000	0.000	0.000	0.000
PRESS15	0.000	0.000	0.000	0.000	0.000
DUTIES15	0.000	0.000	0.000	0.000	0.000

THETA

	PRESS15	DUTIES15
PRESS15	0.000	
DUTIES15	0.000	0.000

ALPHA

KESCAT16	PHY15	KES15	CHOICE15	BULLY15
0.000	0.000	0.000	0.000	0.000

ALPHA

PRESS15	DUTIES15
0.000	0.000

BETA

	KESCAT16	PHY15	KES15	CHOICE15	BULLY15
KESCAT16	0.000	0.000	0.000	0.000	0.000
PHY15	0.000	0.000	0.000	0.000	0.000
KES15	0.000	0.000	0.000	0.000	0.000
CHOICE15	0.000	0.000	0.000	0.000	0.000
BULLY15	0.000	0.000	0.000	0.000	0.000
PRESS15	0.000	0.000	0.000	0.000	0.000
DUTIES15	0.000	0.000	0.000	0.000	0.000

BETA

	PRESS15	DUTIES15
KESCAT16	0.000	0.000
PHY15	0.000	0.000
KES15	0.000	0.000
CHOICE15	0.000	0.000
BULLY15	0.000	0.000
PRESS15	0.000	0.000
DUTIES15	0.000	0.000

PSI

	KESCAT16	PHY15	KES15	CHOICE15	BULLY15
KESCAT16	1.000				
PHY15	0.000	0.268			
KES15	0.000	0.000	0.048		
CHOICE15	0.000	0.000	0.000	0.549	
BULLY15	0.000	0.000	0.000	0.000	0.108
PRESS15	0.000	0.000	0.000	0.000	0.000
DUTIES15	0.000	0.000	0.000	0.000	0.000

PSI

PRESS15	DUTIES15

PRESS15	0.501	
DUTIES15	0.000	0.414

STARTING VALUES FOR BETWEEN

TAU

KESCAT16
1.091

NU

KESCAT16	PRAFAC	CHOICE15
0.000	0.000	0.000

LAMBDA

	S1	KESCAT16	PRAFAC	CHOICE15
KESCAT16	0.000	1.000	0.000	0.000
PRAFAC	0.000	0.000	1.000	0.000
CHOICE15	0.000	0.000	0.000	1.000

THETA

	KESCAT16	PRAFAC	CHOICE15
KESCAT16	0.000		
PRAFAC	0.000	0.000	
CHOICE15	0.000	0.000	0.000

ALPHA

S1	KESCAT16	PRAFAC	CHOICE15
0.000	0.000	0.006	3.221

BETA

S1	KESCAT16	PRAFAC	CHOICE15

```
              ________      ________      ________      ________
S1              0.000         0.000         0.000         0.000
KESCAT16        0.000         0.000         0.000         0.000
PRAFAC          0.000         0.000         0.000         0.000
CHOICE15        0.000         0.000         0.000         0.000


          PSI
              S1            KESCAT16      PRAFAC        CHOICE15
              ________      ________      ________      ________
S1              1.000
KESCAT16        0.000         1.000
PRAFAC          0.000         0.000         0.506
CHOICE15        0.000         0.000         0.000         0.549


    PRIORS FOR ALL PARAMETERS            PRIOR MEAN      PRIOR VARIANCE     PRIOR STD. DEV.

    Parameter 1~N(0.000,infinity)           0.0000            infinity            infinity
    Parameter 2~N(0.000,infinity)           0.0000            infinity            infinity
    Parameter 3~N(0.000,infinity)           0.0000            infinity            infinity
    Parameter 4~N(0.000,infinity)           0.0000            infinity            infinity
    Parameter 5~N(0.000,infinity)           0.0000            infinity            infinity
    Parameter 6~N(0.000,5.000)              0.0000              5.0000              2.2361
    Parameter 7~N(0.000,5.000)              0.0000              5.0000              2.2361
    Parameter 8~N(0.000,5.000)              0.0000              5.0000              2.2361
    Parameter 9~N(0.000,5.000)              0.0000              5.0000              2.2361
    Parameter 10~N(0.000,5.000)             0.0000              5.0000              2.2361
    Parameter 11~IW(1.000,7)              infinity            infinity            infinity
    Parameter 12~IW(0.000,7)              infinity            infinity            infinity
    Parameter 13~IW(1.000,7)              infinity            infinity            infinity
    Parameter 14~IW(0.000,7)              infinity            infinity            infinity
    Parameter 15~IW(0.000,7)              infinity            infinity            infinity
    Parameter 16~IW(1.000,7)              infinity            infinity            infinity
    Parameter 17~IW(0.000,7)              infinity            infinity            infinity
    Parameter 18~IW(0.000,7)              infinity            infinity            infinity
    Parameter 19~IW(0.000,7)              infinity            infinity            infinity
    Parameter 20~IW(1.000,7)              infinity            infinity            infinity
    Parameter 21~IW(0.000,7)              infinity            infinity            infinity
    Parameter 22~IW(0.000,7)              infinity            infinity            infinity
    Parameter 23~IW(0.000,7)              infinity            infinity            infinity
    Parameter 24~IW(0.000,7)              infinity            infinity            infinity
    Parameter 25~IW(1.000,7)              infinity            infinity            infinity
```

Parameter 26~IW(0.000,7)	infinity	infinity	infinity
Parameter 27~IW(0.000,7)	infinity	infinity	infinity
Parameter 28~IW(0.000,7)	infinity	infinity	infinity
Parameter 29~IW(0.000,7)	infinity	infinity	infinity
Parameter 30~IW(0.000,7)	infinity	infinity	infinity
Parameter 31~IW(1.000,7)	infinity	infinity	infinity
Parameter 32~N(0.000,infinity)	0.0000	infinity	infinity
Parameter 33~N(0.000,infinity)	0.0000	infinity	infinity
Parameter 34~N(0.000,infinity)	0.0000	infinity	infinity
Parameter 35~N(0.000,infinity)	0.0000	infinity	infinity
Parameter 36~N(0.000,5.000)	0.0000	5.0000	2.2361
Parameter 37~N(0.000,5.000)	0.0000	5.0000	2.2361
Parameter 38~IG(-1.000,0.000)	infinity	infinity	infinity
Parameter 39~IG(-1.000,0.000)	infinity	infinity	infinity
Parameter 40~IW(1.000,3)	infinity	infinity	infinity
Parameter 41~IW(0.000,3)	infinity	infinity	infinity
Parameter 42~IW(1.000,3)	infinity	infinity	infinity
Parameter 43~N(0.000,5.000)	0.0000	5.0000	2.2361

TECHNICAL 3 OUTPUT

ESTIMATED COVARIANCE MATRIX FOR PARAMETER ESTIMATES

	1	2	3	4	5
1	0.100186D-03				
2	0.103876D-04	0.165429D-04			
3	0.397912D-05	0.463320D-05	0.421709D-04		
4	0.113685D-04	0.110553D-04	0.138743D-04	0.179277D-03	
5	-0.129378D-04	-0.112494D-04	-0.118782D-04	-0.344097D-04	0.165321D-03
6	-0.984302D-05	-0.534504D-05	-0.380228D-05	-0.242839D-04	0.692096D-05
7	0.814203D-05	0.606431D-05	0.110449D-04	0.214701D-04	0.257677D-05
8	-0.268280D-04	-0.530042D-05	0.564404D-05	-0.400288D-04	0.226703D-04
9	0.476939D-06	-0.137312D-06	-0.101072D-05	0.147020D-04	-0.774041D-05
10	-0.134310D-04	-0.455885D-05	-0.384665D-05	-0.638037D-05	0.239319D-04
11	0.818940D-06	-0.357095D-08	0.168327D-05	0.508655D-05	-0.458209D-05
12	0.678046D-06	0.139355D-08	0.992089D-06	0.318646D-07	0.236586D-06
13	-0.427505D-06	0.133937D-06	0.753231D-06	-0.537980D-06	0.649073D-07
14	0.159077D-05	0.126168D-05	0.629544D-06	0.383493D-05	0.175021D-05
15	0.112620D-05	0.640203D-06	-0.109890D-05	0.301132D-05	-0.113009D-05
16	0.305889D-05	-0.293870D-05	0.283662D-05	0.624777D-05	0.555277D-05
17	0.124161D-05	0.579915D-07	0.963019D-06	0.253625D-05	0.395304D-06
18	0.231895D-06	0.225329D-06	0.923602D-06	0.335860D-06	-0.689942D-06

19	0.643637D-06	0.167217D-05	-0.108774D-05	-0.264041D-05	-0.745223D-06
20	-0.114302D-05	0.373266D-06	0.521323D-06	0.835421D-06	-0.620144D-07
21	-0.385274D-06	0.182726D-06	-0.454817D-06	0.146945D-05	0.712198D-05
22	0.474359D-06	0.192070D-06	-0.495107D-07	0.148719D-05	0.145971D-05
23	0.565353D-05	0.423877D-05	0.201995D-05	0.111998D-06	-0.111273D-04
24	-0.927988D-06	-0.665929D-07	-0.105658D-05	-0.130238D-05	0.133783D-05
25	-0.117818D-04	0.228310D-05	-0.460589D-05	-0.688837D-05	0.302253D-06
26	0.235863D-05	-0.268654D-06	-0.378619D-05	-0.843733D-06	0.172844D-05
27	-0.110098D-05	-0.870857D-06	-0.189135D-05	-0.145565D-05	0.302887D-05
28	-0.655295D-05	-0.860042D-06	-0.199261D-05	0.708612D-05	0.122862D-05
29	-0.446396D-06	-0.369022D-06	-0.118987D-05	-0.105714D-05	0.955914D-06
30	0.257234D-05	0.137260D-05	-0.200217D-05	-0.121568D-05	0.151128D-05
31	0.160739D-05	0.131855D-05	0.291767D-05	0.619689D-05	0.214347D-05
32	-0.311361D-05	-0.199322D-05	0.559426D-05	-0.215363D-05	0.375829D-05
33	-0.239991D-04	0.133360D-04	0.343337D-05	0.692522D-04	-0.207555D-04
34	-0.158505D-04	-0.894447D-05	-0.116436D-04	-0.125165D-04	0.105799D-04
35	0.636686D-06	-0.239335D-05	0.693639D-05	0.965812D-05	0.121129D-04
36	0.978189D-07	-0.760812D-06	0.374706D-05	0.141169D-04	-0.521134D-05
37	-0.538216D-04	0.951175D-05	-0.126484D-04	0.161561D-04	0.269171D-04
38	0.128930D-05	-0.338461D-06	-0.147209D-05	-0.943648D-06	-0.461786D-05
39	-0.910822D-06	-0.170214D-06	0.186012D-05	0.217580D-05	-0.816968D-06
40	-0.140528D-04	-0.148500D-04	-0.558260D-05	0.190948D-04	-0.907919D-04
41	0.126796D-04	-0.352825D-05	0.562049D-05	-0.611960D-05	0.112440D-06
42	0.167141D-05	0.312305D-05	0.448670D-05	0.896304D-06	-0.346176D-05
43	-0.174548D-03	0.344160D-04	-0.363682D-04	0.408537D-04	0.721599D-04

ESTIMATED COVARIANCE MATRIX FOR PARAMETER ESTIMATES

	6	7	8	9	10
6	0.111988D-02				
7	-0.623069D-03	0.763825D-02			
8	-0.146989D-04	-0.379192D-03	0.231203D-02		
9	-0.730545D-04	-0.270061D-03	-0.210329D-03	0.687986D-03	
10	-0.355140D-04	0.200561D-03	0.137205D-03	0.967505D-04	0.772250D-03
11	0.989755D-06	0.240829D-04	0.162619D-04	0.297958D-05	-0.894463D-05
12	-0.832310D-06	0.998949D-05	0.362302D-05	0.175759D-05	-0.498018D-06
13	-0.100268D-06	0.824122D-06	0.309394D-05	0.161753D-05	-0.100226D-06
14	-0.389104D-05	0.149374D-04	0.178875D-04	-0.821253D-05	0.148709D-04
15	-0.284361D-06	0.776966D-05	-0.674202D-06	-0.169894D-05	0.325555D-05
16	-0.274143D-04	-0.659086D-05	-0.745609D-05	0.188540D-04	-0.212925D-04
17	-0.653262D-05	0.126390D-04	-0.586044D-06	0.459620D-05	-0.321232D-05
18	0.487680D-06	-0.666109D-06	0.541566D-05	0.105254D-06	0.126566D-05
19	0.640610D-05	-0.146776D-04	0.169725D-05	-0.117460D-05	0.306499D-05

20	-0.357757D-05	0.116503D-04	0.668505D-05	0.981060D-06	-0.293180D-05
21	-0.103274D-04	0.472857D-04	0.117143D-04	0.103668D-04	0.887876D-05
22	0.504793D-08	-0.180851D-05	0.152298D-06	0.715006D-05	-0.212059D-05
23	0.103018D-04	0.172207D-04	-0.138914D-04	-0.702926D-06	0.254632D-04
24	-0.665472D-05	0.167040D-04	0.486294D-05	-0.972441D-06	-0.143974D-05
25	-0.658418D-05	0.111275D-04	0.238517D-04	-0.599710D-05	0.202618D-05
26	0.517222D-05	-0.944932D-05	-0.340873D-05	0.380708D-05	0.304163D-05
27	0.696690D-07	0.205256D-04	-0.306573D-05	0.740806D-06	0.724766D-05
28	-0.100713D-05	0.449747D-04	-0.819756D-05	-0.657440D-05	0.631607D-05
29	-0.279204D-05	-0.117406D-04	-0.275952D-05	0.554933D-05	0.791694D-05
30	-0.270029D-05	0.204158D-04	0.116470D-04	-0.112049D-04	0.123596D-04
31	-0.107125D-04	0.504044D-04	-0.411443D-05	-0.110040D-06	-0.720915D-05
32	0.405004D-04	0.890796D-04	0.812638D-04	-0.212770D-04	-0.123518D-03
33	-0.170210D-03	-0.321219D-03	0.185824D-04	-0.237773D-04	-0.142780D-03
34	0.372748D-04	-0.391633D-04	0.109557D-03	-0.264468D-04	-0.318522D-05
35	0.792546D-05	0.104746D-03	0.946617D-05	-0.202246D-04	-0.756712D-05
36	0.601608D-04	0.686230D-04	-0.107336D-04	0.307394D-04	-0.131092D-04
37	0.217473D-04	-0.679920D-03	0.111585D-03	-0.630503D-05	-0.149776D-03
38	0.552445D-05	0.692540D-04	-0.789565D-05	-0.697162D-05	0.123826D-05
39	-0.589095D-05	0.334612D-04	0.209367D-04	0.131748D-04	0.120212D-05
40	-0.650865D-04	-0.133428D-03	-0.339289D-03	-0.456677D-04	0.271266D-03
41	0.994120D-05	-0.494857D-04	0.274382D-04	-0.239266D-05	0.489091D-04
42	0.457967D-05	0.462603D-04	0.209252D-04	-0.168592D-04	0.103971D-04
43	0.142210D-03	-0.102813D-02	0.302593D-03	-0.591848D-04	-0.530914D-03

ESTIMATED COVARIANCE MATRIX FOR PARAMETER ESTIMATES

	11	12	13	14	15
11	0.105213D-03				
12	0.105802D-04	0.104192D-04			
13	0.166926D-05	0.187222D-05	0.328610D-05		
14	-0.185311D-04	-0.623815D-05	-0.157857D-05	0.110121D-03	
15	-0.159753D-05	-0.251967D-05	-0.147058D-05	0.115008D-04	0.198731D-04
16	0.627904D-05	0.234856D-05	0.135428D-06	-0.310113D-04	-0.120207D-04
17	0.370187D-05	0.212460D-05	0.332957D-06	-0.563503D-05	0.160516D-06
18	0.573667D-06	0.661142D-06	0.969637D-06	-0.897186D-06	-0.998898D-06
19	-0.268646D-05	-0.188167D-05	-0.865576D-06	0.495204D-05	0.533702D-05
20	0.271814D-05	0.360635D-06	0.260962D-06	-0.245035D-06	0.151231D-06
21	0.799045D-05	0.544436D-05	0.664931D-06	-0.955712D-05	-0.494471D-06
22	0.409461D-06	0.806138D-06	0.184521D-05	-0.616483D-06	-0.154821D-05
23	0.458481D-06	-0.137273D-05	-0.700613D-06	0.164987D-04	0.115728D-04
24	0.815487D-07	0.156157D-06	0.246512D-06	-0.701466D-07	-0.161173D-06
25	0.637818D-05	0.159957D-05	0.127967D-05	-0.517452D-05	-0.822295D-06

26	-0.111475D-04	-0.573228D-05	-0.135309D-05	0.197581D-04	0.689967D-06
27	-0.235130D-05	-0.172769D-05	-0.149191D-05	0.306574D-05	0.244373D-05
28	0.202639D-05	0.101226D-05	0.917084D-06	-0.119373D-04	-0.112152D-04
29	-0.106692D-05	-0.908474D-06	-0.550275D-06	-0.143644D-05	0.973089D-07
30	-0.399497D-05	-0.153326D-05	-0.322284D-06	0.370083D-05	0.168463D-05
31	0.484905D-05	0.696144D-07	0.209481D-06	-0.662221D-05	-0.142539D-05
32	0.893533D-05	0.768201D-06	0.105184D-05	-0.138417D-04	0.233242D-05
33	0.591084D-05	-0.244123D-05	-0.623987D-05	-0.664811D-04	0.199070D-04
34	-0.211384D-05	0.779748D-06	-0.672661D-06	0.178420D-04	0.268256D-05
35	0.272749D-05	-0.204554D-06	0.599836D-06	-0.190519D-04	-0.143755D-05
36	0.441458D-05	-0.230938D-05	-0.372562D-06	-0.800861D-05	-0.385104D-05
37	-0.122989D-04	-0.779616D-05	-0.423138D-05	0.400835D-04	0.237669D-04
38	-0.129351D-05	-0.876849D-06	-0.107025D-05	-0.234639D-05	0.134722D-05
39	0.236213D-06	0.410349D-06	-0.566393D-06	-0.680797D-06	-0.447011D-06
40	0.496946D-04	-0.491213D-05	0.103130D-05	0.403437D-04	0.544983D-05
41	-0.667388D-05	-0.371439D-05	0.277954D-05	0.442115D-04	0.141399D-04
42	0.399348D-05	-0.451033D-06	0.606179D-06	0.500294D-05	0.580367D-07
43	-0.399454D-04	-0.248960D-04	-0.146591D-04	0.141531D-03	0.773409D-04

ESTIMATED COVARIANCE MATRIX FOR PARAMETER ESTIMATES

	16	17	18	19	20
16	0.431070D-03				
17	0.175189D-05	0.218444D-04			
18	-0.110913D-06	0.258922D-05	0.397037D-05		
19	-0.138690D-04	-0.425216D-05	-0.169727D-05	0.437118D-04	
20	0.119833D-05	0.253801D-05	0.182421D-05	-0.335060D-05	0.184426D-04
21	-0.902972D-05	0.886276D-05	0.119283D-05	-0.263480D-05	-0.304205D-06
22	-0.125578D-05	0.447808D-06	0.187867D-05	-0.170900D-05	-0.810236D-07
23	-0.282389D-04	0.286024D-05	-0.359426D-06	0.192960D-04	0.155973D-05
24	0.123185D-06	0.273023D-05	0.234561D-05	-0.150909D-05	0.848320D-05
25	-0.579809D-05	0.234433D-05	0.134068D-05	-0.171782D-05	0.131438D-05
26	-0.681008D-05	-0.470705D-05	-0.791446D-06	0.312919D-05	-0.176528D-05
27	-0.389324D-05	-0.627816D-06	-0.118396D-05	0.985887D-06	-0.368697D-06
28	0.612612D-04	0.208735D-05	0.779583D-06	-0.114649D-04	0.124600D-05
29	-0.625183D-05	-0.172161D-05	-0.163466D-05	0.573199D-05	-0.504831D-05
30	-0.598946D-05	0.115843D-07	-0.313572D-06	0.238207D-05	-0.945811D-06
31	0.149768D-04	0.438199D-05	0.302766D-06	-0.339813D-05	0.189207D-06
32	0.228892D-04	0.872549D-06	0.920643D-06	-0.411994D-05	-0.884827D-05
33	0.103750D-03	0.337006D-04	-0.108159D-04	0.238176D-04	0.450966D-05
34	-0.220181D-05	-0.349824D-06	0.169533D-05	0.244546D-05	0.331618D-06
35	-0.395283D-05	-0.130931D-05	-0.182380D-05	-0.316159D-05	-0.644216D-05
36	0.107587D-04	0.371421D-05	0.420590D-06	-0.484117D-05	-0.172532D-05

37	0.445184D-05	0.899045D-05	0.966473D-06	0.800598D-04	-0.182466D-05
38	-0.461867D-06	0.524507D-06	-0.124697D-05	0.145174D-05	-0.254129D-05
39	0.535106D-05	0.251218D-05	-0.449014D-06	0.775255D-06	0.785683D-06
40	-0.286365D-03	-0.689094D-05	0.291092D-04	-0.631013D-04	-0.219129D-04
41	0.133134D-04	-0.394296D-05	0.392183D-05	-0.603480D-05	0.124606D-05
42	-0.176176D-05	-0.406512D-06	0.333383D-06	0.298848D-05	0.291614D-05
43	0.268383D-04	0.256263D-04	0.203181D-05	0.259372D-03	-0.485221D-05

ESTIMATED COVARIANCE MATRIX FOR PARAMETER ESTIMATES

	21	22	23	24	25
21	0.106399D-03				
22	0.102882D-04	0.173965D-04			
23	-0.125404D-04	-0.109408D-04	0.216592D-03		
24	0.283851D-05	0.436236D-05	-0.855746D-05	0.408485D-04	
25	0.206771D-04	0.189793D-04	-0.186166D-04	0.317497D-04	0.390315D-03
26	-0.177754D-04	-0.214138D-05	0.309560D-05	-0.651190D-06	-0.404302D-05
27	-0.227411D-05	-0.285788D-05	0.324521D-05	-0.516397D-06	-0.163368D-05
28	-0.215704D-05	0.240859D-05	-0.343337D-04	0.469514D-05	-0.243882D-05
29	-0.321677D-05	-0.104898D-05	0.416125D-06	-0.610414D-05	-0.499112D-05
30	-0.116213D-04	-0.118640D-04	0.303828D-04	-0.125453D-04	-0.583117D-04
31	0.382985D-05	0.318791D-05	-0.149552D-04	0.575261D-05	0.151769D-04
32	0.235366D-05	-0.344140D-06	0.411542D-05	-0.454149D-05	0.207052D-04
33	-0.650849D-05	-0.149609D-04	-0.344463D-05	-0.184071D-05	0.171607D-03
34	-0.542787D-05	0.496524D-07	0.535341D-05	-0.326622D-05	0.310051D-04
35	0.103103D-04	-0.316030D-05	0.970852D-05	-0.403798D-05	-0.816737D-05
36	0.492141D-05	0.271180D-05	-0.226411D-04	0.391694D-05	0.115768D-04
37	0.393189D-04	-0.145653D-04	0.132754D-03	-0.895271D-05	-0.816435D-04
38	0.295215D-05	0.231268D-06	-0.435573D-05	0.141536D-06	0.381545D-05
39	0.300895D-05	0.568790D-06	0.216899D-05	0.616756D-06	-0.627764D-06
40	0.135276D-03	0.352664D-04	-0.111933D-03	-0.228320D-04	0.115299D-04
41	-0.260430D-05	0.937807D-05	0.358647D-04	0.826012D-05	0.425160D-04
42	-0.170079D-05	0.258139D-05	0.498110D-05	-0.231103D-06	0.134187D-04
43	0.130983D-03	-0.493414D-04	0.434318D-03	-0.340187D-04	-0.272913D-03

ESTIMATED COVARIANCE MATRIX FOR PARAMETER ESTIMATES

	26	27	28	29	30
26	0.857491D-04				
27	0.954299D-05	0.147992D-04			
28	-0.156299D-04	-0.643961D-05	0.179680D-03		
29	0.259115D-05	0.344329D-05	-0.795417D-05	0.338780D-04	

30	0.917913D-05	0.973236D-05	-0.122714D-04	0.145216D-04	0.162465D-03
31	-0.226806D-04	-0.158835D-04	0.475900D-04	-0.172204D-04	-0.535690D-04
32	-0.694697D-05	0.248760D-05	-0.156496D-04	0.291056D-05	0.892567D-05
33	-0.536717D-04	-0.368588D-06	0.602272D-05	-0.305586D-04	-0.164053D-04
34	-0.237865D-06	-0.388457D-05	0.191075D-05	-0.542718D-05	-0.971737D-05
35	0.118749D-05	0.224060D-05	-0.488289D-05	0.612375D-05	-0.114246D-04
36	0.127262D-05	0.339492D-05	0.294318D-05	-0.441939D-05	0.115074D-04
37	0.943116D-04	0.189816D-04	-0.859762D-05	-0.100973D-04	-0.246470D-04
38	-0.262236D-05	0.201244D-05	-0.888991D-06	0.190654D-05	-0.337097D-05
39	0.336599D-06	0.305958D-06	0.644489D-06	-0.161452D-05	-0.291212D-05
40	0.169648D-04	0.252460D-05	-0.748221D-04	-0.145812D-04	0.239045D-04
41	0.358351D-05	-0.105230D-04	-0.171707D-04	-0.293696D-05	0.830325D-05
42	-0.677709D-06	-0.132489D-05	-0.416966D-07	-0.327750D-05	-0.266035D-05
43	0.293723D-03	0.651645D-04	-0.307230D-04	-0.342109D-04	-0.961692D-04

ESTIMATED COVARIANCE MATRIX FOR PARAMETER ESTIMATES

	31	32	33	34	35
31	0.250672D-03				
32	-0.194635D-04	0.118834D-02			
33	0.197220D-04	-0.996930D-04	0.231036D-01		
34	0.959373D-05	-0.283096D-05	0.581900D-03	0.136073D-02	
35	-0.458651D-05	0.158335D-03	-0.163238D-03	-0.265173D-04	0.987251D-03
36	-0.260284D-05	-0.131612D-04	-0.658791D-04	-0.253765D-04	0.666351D-04
37	-0.141610D-03	-0.214237D-04	0.251003D-03	0.724967D-04	-0.189680D-03
38	0.462876D-05	0.274629D-04	0.476819D-05	-0.908893D-05	0.352635D-04
39	-0.223113D-05	-0.141452D-04	0.891282D-05	0.799964D-05	0.131412D-04
40	0.584595D-04	-0.305885D-03	-0.845416D-03	0.526617D-04	-0.139180D-03
41	-0.137953D-04	0.756560D-04	0.284735D-03	0.397858D-04	-0.515103D-04
42	-0.613241D-06	0.771560D-05	0.947197D-05	0.176600D-04	-0.396133D-05
43	-0.437290D-03	-0.101396D-03	0.742057D-03	0.161684D-03	-0.539863D-03

ESTIMATED COVARIANCE MATRIX FOR PARAMETER ESTIMATES

	36	37	38	39	40
36	0.817195D-03				
37	-0.498597D-03	0.273834D-01			
38	0.125845D-04	-0.271243D-04	0.108328D-03		
39	0.240051D-04	0.136880D-03	0.536660D-05	0.700838D-04	
40	-0.304592D-04	-0.631209D-03	0.353747D-04	0.675772D-05	0.723807D-01
41	0.917844D-05	0.793009D-04	-0.501359D-05	-0.309079D-05	0.154333D-02
42	-0.167490D-04	0.170739D-03	-0.188138D-05	-0.305423D-05	0.121246D-03

43	-0.166524D-02	0.891149D-01	-0.461134D-04	0.467508D-03	-0.212983D-02

ESTIMATED COVARIANCE MATRIX FOR PARAMETER ESTIMATES

	41	42	43
41	0.210750D-02		
42	0.921724D-04	0.169030D-03	
43	0.281034D-03	0.544146D-03	0.291227D+00

ESTIMATED CORRELATION MATRIX FOR PARAMETER ESTIMATES

	1	2	3	4	5
1	1.000				
2	0.255	1.000			
3	0.061	0.175	1.000		
4	0.085	0.203	0.160	1.000	
5	-0.101	-0.215	-0.142	-0.200	1.000
6	-0.029	-0.039	-0.017	-0.054	0.016
7	0.009	0.017	0.019	0.018	0.002
8	-0.056	-0.027	0.018	-0.062	0.037
9	0.002	-0.001	-0.006	0.042	-0.023
10	-0.048	-0.040	-0.021	-0.017	0.067
11	0.008	0.000	0.025	0.037	-0.035
12	0.021	0.000	0.047	0.001	0.006
13	-0.024	0.018	0.064	-0.022	0.003
14	0.015	0.030	0.009	0.027	0.013
15	0.025	0.035	-0.038	0.050	-0.020
16	0.015	-0.035	0.021	0.022	0.021
17	0.027	0.003	0.032	0.041	0.007
18	0.012	0.028	0.071	0.013	-0.027
19	0.010	0.062	-0.025	-0.030	-0.009
20	-0.027	0.021	0.019	0.015	-0.001
21	-0.004	0.004	-0.007	0.011	0.054
22	0.011	0.011	-0.002	0.027	0.027
23	0.038	0.071	0.021	0.001	-0.059
24	-0.015	-0.003	-0.025	-0.015	0.016
25	-0.060	0.028	-0.036	-0.026	0.001
26	0.025	-0.007	-0.063	-0.007	0.015
27	-0.029	-0.056	-0.076	-0.028	0.061
28	-0.049	-0.016	-0.023	0.039	0.007
29	-0.008	-0.016	-0.031	-0.014	0.013
30	0.020	0.026	-0.024	-0.007	0.009

31	0.010	0.020	0.028	0.029	0.011
32	-0.009	-0.014	0.025	-0.005	0.008
33	-0.016	0.022	0.003	0.034	-0.011
34	-0.043	-0.060	-0.049	-0.025	0.022
35	0.002	-0.019	0.034	0.023	0.030
36	0.000	-0.007	0.020	0.037	-0.014
37	-0.032	0.014	-0.012	0.007	0.013
38	0.012	-0.008	-0.022	-0.007	-0.035
39	-0.011	-0.005	0.034	0.019	-0.008
40	-0.005	-0.014	-0.003	0.005	-0.026
41	0.028	-0.019	0.019	-0.010	0.000
42	0.013	0.059	0.053	0.005	-0.021
43	-0.032	0.016	-0.010	0.006	0.010

ESTIMATED CORRELATION MATRIX FOR PARAMETER ESTIMATES

	6	7	8	9	10
6	1.000				
7	-0.213	1.000			
8	-0.009	-0.090	1.000		
9	-0.083	-0.118	-0.167	1.000	
10	-0.038	0.083	0.103	0.133	1.000
11	0.003	0.027	0.033	0.011	-0.031
12	-0.008	0.035	0.023	0.021	-0.006
13	-0.002	0.005	0.035	0.034	-0.002
14	-0.011	0.016	0.035	-0.030	0.051
15	-0.002	0.020	-0.003	-0.015	0.026
16	-0.039	-0.004	-0.007	0.035	-0.037
17	-0.042	0.031	-0.003	0.037	-0.025
18	0.007	-0.004	0.057	0.002	0.023
19	0.029	-0.025	0.005	-0.007	0.017
20	-0.025	0.031	0.032	0.009	-0.025
21	-0.030	0.052	0.024	0.038	0.031
22	0.000	-0.005	0.001	0.065	-0.018
23	0.021	0.013	-0.020	-0.002	0.062
24	-0.031	0.030	0.016	-0.006	-0.008
25	-0.010	0.006	0.025	-0.012	0.004
26	0.017	-0.012	-0.008	0.016	0.012
27	0.001	0.061	-0.017	0.007	0.068
28	-0.002	0.038	-0.013	-0.019	0.017
29	-0.014	-0.023	-0.010	0.036	0.049
30	-0.006	0.018	0.019	-0.034	0.035
31	-0.020	0.036	-0.005	0.000	-0.016

32	0.035	0.030	0.049	-0.024	-0.129
33	-0.033	-0.024	0.003	-0.006	-0.034
34	0.030	-0.012	0.062	-0.027	-0.003
35	0.008	0.038	0.006	-0.025	-0.009
36	0.063	0.027	-0.008	0.041	-0.017
37	0.004	-0.047	0.014	-0.001	-0.033
38	0.016	0.076	-0.016	-0.026	0.004
39	-0.021	0.046	0.052	0.060	0.005
40	-0.007	-0.006	-0.026	-0.006	0.036
41	0.006	-0.012	0.012	-0.002	0.038
42	0.011	0.041	0.033	-0.049	0.029
43	0.008	-0.022	0.012	-0.004	-0.035

ESTIMATED CORRELATION MATRIX FOR PARAMETER ESTIMATES

	11	12	13	14	15
11	1.000				
12	0.320	1.000			
13	0.090	0.320	1.000		
14	-0.172	-0.184	-0.083	1.000	
15	-0.035	-0.175	-0.182	0.246	1.000
16	0.029	0.035	0.004	-0.142	-0.130
17	0.077	0.141	0.039	-0.115	0.008
18	0.028	0.103	0.268	-0.043	-0.112
19	-0.040	-0.088	-0.072	0.071	0.181
20	0.062	0.026	0.034	-0.005	0.008
21	0.076	0.164	0.036	-0.088	-0.011
22	0.010	0.060	0.244	-0.014	-0.083
23	0.003	-0.029	-0.026	0.107	0.176
24	0.001	0.008	0.021	-0.001	-0.006
25	0.031	0.025	0.036	-0.025	-0.009
26	-0.117	-0.192	-0.081	0.203	0.017
27	-0.060	-0.139	-0.214	0.076	0.142
28	0.015	0.023	0.038	-0.085	-0.188
29	-0.018	-0.048	-0.052	-0.024	0.004
30	-0.031	-0.037	-0.014	0.028	0.030
31	0.030	0.001	0.007	-0.040	-0.020
32	0.025	0.007	0.017	-0.038	0.015
33	0.004	-0.005	-0.023	-0.042	0.029
34	-0.006	0.007	-0.010	0.046	0.016
35	0.008	-0.002	0.011	-0.058	-0.010
36	0.015	-0.025	-0.007	-0.027	-0.030
37	-0.007	-0.015	-0.014	0.023	0.032

38	-0.012	-0.026	-0.057	-0.021	0.029
39	0.003	0.015	-0.037	-0.008	-0.012
40	0.018	-0.006	0.002	0.014	0.005
41	-0.014	-0.025	0.033	0.092	0.069
42	0.030	-0.011	0.026	0.037	0.001
43	-0.007	-0.014	-0.015	0.025	0.032

ESTIMATED CORRELATION MATRIX FOR PARAMETER ESTIMATES

	16	17	18	19	20
16	1.000				
17	0.018	1.000			
18	-0.003	0.278	1.000		
19	-0.101	-0.138	-0.129	1.000	
20	0.013	0.126	0.213	-0.118	1.000
21	-0.042	0.184	0.058	-0.039	-0.007
22	-0.015	0.023	0.226	-0.062	-0.005
23	-0.092	0.042	-0.012	0.198	0.025
24	0.001	0.091	0.184	-0.036	0.309
25	-0.014	0.025	0.034	-0.013	0.015
26	-0.035	-0.109	-0.043	0.051	-0.044
27	-0.049	-0.035	-0.154	0.039	-0.022
28	0.220	0.033	0.029	-0.129	0.022
29	-0.052	-0.063	-0.141	0.149	-0.202
30	-0.023	0.000	-0.012	0.028	-0.017
31	0.046	0.059	0.010	-0.032	0.003
32	0.032	0.005	0.013	-0.018	-0.060
33	0.033	0.047	-0.036	0.024	0.007
34	-0.003	-0.002	0.023	0.010	0.002
35	-0.006	-0.009	-0.029	-0.015	-0.048
36	0.018	0.028	0.007	-0.026	-0.014
37	0.001	0.012	0.003	0.073	-0.003
38	-0.002	0.011	-0.060	0.021	-0.057
39	0.031	0.064	-0.027	0.014	0.022
40	-0.051	-0.005	0.054	-0.035	-0.019
41	0.014	-0.018	0.043	-0.020	0.006
42	-0.007	-0.007	0.013	0.035	0.052
43	0.002	0.010	0.002	0.073	-0.002

ESTIMATED CORRELATION MATRIX FOR PARAMETER ESTIMATES

	21	22	23	24	25

21	1.000				
22	0.239	1.000			
23	-0.083	-0.178	1.000		
24	0.043	0.164	-0.091	1.000	
25	0.101	0.230	-0.064	0.251	1.000
26	-0.186	-0.055	0.023	-0.011	-0.022
27	-0.057	-0.178	0.057	-0.021	-0.021
28	-0.016	0.043	-0.174	0.055	-0.009
29	-0.054	-0.043	0.005	-0.164	-0.043
30	-0.088	-0.223	0.162	-0.154	-0.232
31	0.023	0.048	-0.064	0.057	0.049
32	0.007	-0.002	0.008	-0.021	0.030
33	-0.004	-0.024	-0.002	-0.002	0.057
34	-0.014	0.000	0.010	-0.014	0.043
35	0.032	-0.024	0.021	-0.020	-0.013
36	0.017	0.023	-0.054	0.021	0.020
37	0.023	-0.021	0.055	-0.008	-0.025
38	0.027	0.005	-0.028	0.002	0.019
39	0.035	0.016	0.018	0.012	-0.004
40	0.049	0.031	-0.028	-0.013	0.002
41	-0.005	0.049	0.053	0.028	0.047
42	-0.013	0.048	0.026	-0.003	0.052
43	0.024	-0.022	0.055	-0.010	-0.026

ESTIMATED CORRELATION MATRIX FOR PARAMETER ESTIMATES

	26	27	28	29	30
26	1.000				
27	0.268	1.000			
28	-0.126	-0.125	1.000		
29	0.048	0.154	-0.102	1.000	
30	0.078	0.198	-0.072	0.196	1.000
31	-0.155	-0.261	0.224	-0.187	-0.265
32	-0.022	0.019	-0.034	0.015	0.020
33	-0.038	-0.001	0.003	-0.035	-0.008
34	-0.001	-0.027	0.004	-0.025	-0.021
35	0.004	0.019	-0.012	0.033	-0.029
36	0.005	0.031	0.008	-0.027	0.032
37	0.062	0.030	-0.004	-0.010	-0.012
38	-0.027	0.050	-0.006	0.031	-0.025
39	0.004	0.010	0.006	-0.033	-0.027
40	0.007	0.002	-0.021	-0.009	0.007
41	0.008	-0.060	-0.028	-0.011	0.014

42	-0.006	-0.026	0.000	-0.043	-0.016
43	0.059	0.031	-0.004	-0.011	-0.014

ESTIMATED CORRELATION MATRIX FOR PARAMETER ESTIMATES

	31	32	33	34	35
31	1.000				
32	-0.036	1.000			
33	0.008	-0.019	1.000		
34	0.016	-0.002	0.104	1.000	
35	-0.009	0.146	-0.034	-0.023	1.000
36	-0.006	-0.013	-0.015	-0.024	0.074
37	-0.054	-0.004	0.010	0.012	-0.036
38	0.028	0.077	0.003	-0.024	0.108
39	-0.017	-0.049	0.007	0.026	0.050
40	0.014	-0.033	-0.021	0.005	-0.016
41	-0.019	0.048	0.041	0.023	-0.036
42	-0.003	0.017	0.005	0.037	-0.010
43	-0.051	-0.005	0.009	0.008	-0.032

ESTIMATED CORRELATION MATRIX FOR PARAMETER ESTIMATES

	36	37	38	39	40
36	1.000				
37	-0.105	1.000			
38	0.042	-0.016	1.000		
39	0.100	0.099	0.062	1.000	
40	-0.004	-0.014	0.013	0.003	1.000
41	0.007	0.010	-0.010	-0.008	0.125
42	-0.045	0.079	-0.014	-0.028	0.035
43	-0.108	0.998	-0.008	0.103	-0.015

ESTIMATED CORRELATION MATRIX FOR PARAMETER ESTIMATES

	41	42	43
41	1.000		
42	0.154	1.000	
43	0.011	0.078	1.000

TECHNICAL 8 OUTPUT

TECHNICAL 8 OUTPUT FOR BAYES ESTIMATION

CHAIN	BSEED
1	0
2	285380
3	253358

ITERATION	POTENTIAL SCALE REDUCTION	PARAMETER WITH HIGHEST PSR
100	1.007	7
200	1.008	34
300	1.012	9
400	1.003	4
500	1.003	36
600	1.004	38
700	1.001	37
800	1.000	39
900	1.001	26
1000	1.002	9

Worsening of psychological distress, bullying interaction

SUMMARY OF DATA

Number of clusters 58

Size (s)	Cluster ID with Size s
7	22
17	166
20	80
26	71 74 205
29	65
31	192
33	228
37	182 106
39	72
41	170 181
42	50 30
43	75
45	173
47	226 165
48	108
50	107 114
51	81
52	40
55	84 92
57	162
59	206
61	61 83 116
62	36 93 169
63	69
64	4
69	68
73	174 152
74	6 154
75	145
89	98
90	178 2
95	144
100	156
104	202
106	142
134	122

186	24
192	209
246	204
278	125
405	217
780	110
873	100

COVARIANCE COVERAGE OF DATA

Minimum covariance coverage value   0.100

Number of missing data patterns   9

PROPORTION OF DATA PRESENT

Covariance Coverage

	KESCAT16	PRAFAC	PHY15	KES15	CHOICE15
KESCAT16	1.000				
PRAFAC	1.000	1.000			
PHY15	0.950	0.950	0.950		
KES15	0.919	0.919	0.919	0.919	
CHOICE15	0.913	0.913	0.913	0.913	0.913
BULLY15	0.913	0.913	0.913	0.913	0.912
PRESS15	0.913	0.913	0.913	0.913	0.913
DUTIES15	0.913	0.913	0.913	0.913	0.913

Covariance Coverage

	BULLY15	PRESS15	DUTIES15
BULLY15	0.913		
PRESS15	0.912	0.913	
DUTIES15	0.913	0.913	0.913

UNIVARIATE PROPORTIONS AND COUNTS FOR CATEGORICAL VARIABLES

KESCAT16

Category 1	0.877	5135.000
Category 2	0.123	720.000

UNIVARIATE SAMPLE STATISTICS

UNIVARIATE HIGHER-ORDER MOMENT DESCRIPTIVE STATISTICS

Variable/ Sample Size	Mean/ Variance	Skewness/ Kurtosis	Minimum/ Maximum	% with Min/Max	Percentiles 20%/60%	40%/80%	Median
PRAFAC	-0.185	0.245	-2.338	1.72%	-1.448	-0.527	-0.318
58.000	1.345	-0.636	2.338	1.72%	0.080	0.752	
PHY15	0.000	0.526	-0.865	32.48%	-0.865	0.135	0.135
5563.000	0.536	0.021	3.135	0.07%	0.135	0.135	
KES15	0.000	0.475	-0.374	22.78%	-0.374	-0.207	-0.041
5382.000	0.095	-0.842	0.626	6.60%	-0.041	0.293	
CHOICE15	0.000	-0.400	-2.221	7.67%	-1.221	-0.221	-0.221
5345.000	1.097	-0.356	1.779	8.62%	0.779	0.779	
BULLY15	1.153	3.522	1.000	88.29%	1.000	1.000	1.000
5344.000	0.216	14.164	5.000	0.09%	1.000	1.000	
PRESS15	0.000	0.267	-1.445	19.70%	-0.445	-0.445	-0.445
5345.000	1.001	-0.357	2.555	2.75%	0.555	0.555	
DUTIES15	0.000	-1.296	-3.149	2.34%	-0.149	-0.149	-0.149
5347.000	0.828	1.939	0.851	39.74%	-0.149	0.851	

THE MODEL ESTIMATION TERMINATED NORMALLY

USE THE FBITERATIONS OPTION TO INCREASE THE NUMBER OF ITERATIONS BY A FACTOR
OF AT LEAST TWO TO CHECK CONVERGENCE AND THAT THE PSR VALUE DOES NOT INCREASE.

MODEL FIT INFORMATION

Number of Free Parameters 43

MODEL RESULTS

	Estimate	Posterior S.D.	One-Tailed P-Value	95% C.I. Lower 2.5%	95% C.I. Upper 2.5%	Significance
**Within Level**						
KESCAT16 ON						
PHY15	0.137	0.034	0.000	0.069	0.203	*
CHOICE15	-0.059	0.026	0.009	-0.110	-0.009	*
DUTIES15	-0.083	0.028	0.001	-0.138	-0.031	*
PRESS15	-0.032	0.026	0.106	-0.084	0.019	
KES15	1.618	0.087	0.000	1.449	1.793	*
KES15 WITH						
PHY15	0.054	0.003	0.000	0.048	0.061	*
CHOICE15	-0.037	0.005	0.000	-0.046	-0.028	*
BULLY15	0.022	0.002	0.000	0.018	0.026	*
PRESS15	0.053	0.004	0.000	0.045	0.062	*
DUTIES15	-0.051	0.004	0.000	-0.058	-0.043	*
PHY15 WITH						
CHOICE15	-0.092	0.011	0.000	-0.112	-0.070	*
BULLY15	0.024	0.005	0.000	0.015	0.034	*
PRESS15	0.063	0.010	0.000	0.042	0.083	*
DUTIES15	-0.056	0.009	0.000	-0.074	-0.038	*
CHOICE15 WITH						
BULLY15	-0.051	0.007	0.000	-0.064	-0.038	*
PRESS15	-0.077	0.015	0.000	-0.105	-0.047	*
DUTIES15	0.158	0.014	0.000	0.133	0.186	*
BULLY15 WITH						
PRESS15	0.090	0.007	0.000	0.077	0.103	*
DUTIES15	-0.057	0.006	0.000	-0.069	-0.046	*
PRESS15 WITH						
DUTIES15	-0.156	0.013	0.000	-0.181	-0.131	*
Means						
PHY15	0.001	0.010	0.441	-0.019	0.021	
KES15	0.003	0.004	0.221	-0.005	0.011	
CHOICE15	-0.003	0.015	0.419	-0.032	0.025	
PRESS15	0.002	0.013	0.454	-0.026	0.027	
DUTIES15	-0.003	0.013	0.426	-0.028	0.022	

```
 Variances
    PHY15              0.537      0.010      0.000      0.516      0.557      *
    KES15              0.096      0.002      0.000      0.092      0.100      *
    CHOICE15           1.097      0.022      0.000      1.060      1.141      *
    BULLY15            0.215      0.004      0.000      0.207      0.224      *
    PRESS15            1.002      0.020      0.000      0.966      1.042      *
    DUTIES15           0.829      0.016      0.000      0.799      0.860      *

Between Level

 S1         ON
    PRAFAC            -0.031      0.063      0.308     -0.152      0.094

 KESCAT16   ON
    PRAFAC             0.023      0.029      0.201     -0.030      0.079
    BULLY15           -0.900      0.555      0.047     -2.065      0.152

 PRAFAC   WITH
    BULLY15            0.010      0.028      0.348     -0.046      0.067

 Means
    PRAFAC            -0.182      0.152      0.119     -0.485      0.112
    BULLY15            1.154      0.022      0.000      1.113      1.199      *

 Intercepts
    S1                 0.137      0.070      0.017      0.009      0.286      *

 Thresholds
    KESCAT16$1         0.334      0.638      0.313     -1.017      1.528

 Variances
    PRAFAC             1.361      0.269      0.000      0.964      2.001      *
    BULLY15            0.024      0.005      0.000      0.017      0.038      *

 Residual Variances
    KESCAT16           0.007      0.007      0.000      0.001      0.026      *
    S1                 0.035      0.056      0.000      0.001      0.192      *


STANDARDIZED MODEL RESULTS


STDYX Standardization
```

	Estimate	Posterior S.D.	One-Tailed P-Value	95% C.I. Lower 2.5%	Upper 2.5%	Significance
**Within-Level Standardized Estimates Averaged Over Clusters**						
S1 | KESCAT16 ON						
BULLY15	0.056	0.024	0.008	0.012	0.104	*
KESCAT16 ON						
PHY15	0.086	0.021	0.000	0.044	0.128	*
CHOICE15	-0.054	0.023	0.008	-0.099	-0.009	*
DUTIES15	-0.066	0.021	0.002	-0.108	-0.024	*
PRESS15	-0.029	0.022	0.102	-0.073	0.015	
KES15	0.432	0.019	0.000	0.393	0.469	*
KES15 WITH						
PHY15	0.240	0.013	0.000	0.214	0.264	*
CHOICE15	-0.113	0.014	0.000	-0.139	-0.085	*
BULLY15	0.154	0.013	0.000	0.128	0.180	*
PRESS15	0.171	0.013	0.000	0.145	0.197	*
DUTIES15	-0.180	0.013	0.000	-0.205	-0.154	*
PHY15 WITH						
CHOICE15	-0.120	0.014	0.000	-0.146	-0.093	*
BULLY15	0.072	0.014	0.000	0.044	0.098	*
PRESS15	0.086	0.014	0.000	0.059	0.112	*
DUTIES15	-0.084	0.014	0.000	-0.111	-0.057	*
CHOICE15 WITH						
BULLY15	-0.104	0.014	0.000	-0.131	-0.078	*
PRESS15	-0.074	0.014	0.000	-0.100	-0.046	*
DUTIES15	0.166	0.014	0.000	0.140	0.193	*
BULLY15 WITH						
PRESS15	0.193	0.014	0.000	0.166	0.220	*
DUTIES15	-0.136	0.014	0.000	-0.162	-0.109	*
PRESS15 WITH						
DUTIES15	-0.171	0.013	0.000	-0.197	-0.144	*
Means						
PHY15	0.002	0.014	0.435	-0.024	0.029	
KES15	0.010	0.013	0.231	-0.016	0.037	
CHOICE15	-0.003	0.014	0.414	-0.030	0.024	

```
   PRESS15            0.002       0.013      0.449      -0.025       0.027
   DUTIES15          -0.003       0.014      0.424      -0.032       0.024

 Variances
   PHY15              1.000       0.000      0.000       1.000       1.000
   KES15              1.000       0.000      0.000       1.000       1.000
   CHOICE15           1.000       0.000      0.000       1.000       1.000
   BULLY15            1.000       0.000      0.000       1.000       1.000
   PRESS15            1.000       0.000      0.000       1.000       1.000
   DUTIES15           1.000       0.000      0.000       1.000       1.000

Between Level

 S1         ON
   PRAFAC            -0.191       0.380      0.308      -0.897       0.679

 KESCAT16   ON
   PRAFAC             0.152       0.201      0.201      -0.251       0.528
   BULLY15           -0.843       0.315      0.047      -1.000       0.222

 PRAFAC   WITH
   BULLY15            0.053       0.143      0.348      -0.239       0.327

 Means
   PRAFAC            -0.158       0.129      0.119      -0.416       0.094
   BULLY15            7.396       0.745      0.000       6.005       8.937      *

 Intercepts
   S1                 0.708       0.592      0.017       0.033       2.329      *

 Variances
   PRAFAC             1.000       0.000      0.000       1.000       1.000
   BULLY15            1.000       0.000      0.000       1.000       1.000

 Residual Variances
   KESCAT16           0.241       0.272      0.000       0.012       0.934      *
   S1                 0.914       0.213      0.000       0.191       1.000      *


R-SQUARE

Within-Level R-Square Averaged Across Clusters

                                Posterior  One-Tailed         95% C.I.
```

```
    Variable        Estimate       S.D.      P-Value   Lower 2.5%  Upper 2.5%

    KESCAT16           0.255       0.018      0.000       0.221       0.292

Between Level

                               Posterior  One-Tailed         95% C.I.
    Variable        Estimate       S.D.      P-Value   Lower 2.5%  Upper 2.5%

    KESCAT16           0.759       0.272      0.000       0.066       0.988

                               Posterior  One-Tailed         95% C.I.
    Variable        Estimate       S.D.      P-Value   Lower 2.5%  Upper 2.5%

    S1                 0.086       0.213      0.000       0.000       0.809


TECHNICAL 1 OUTPUT


     PARAMETER SPECIFICATION FOR WITHIN


           TAU
              KESCAT16
              ________
                    0


           NU
              KESCAT16      PHY15         KES15         CHOICE15      BULLY15
              ________      ________      ________      ________      ________
                    0             0             0             0             0


           NU
              PRESS15       DUTIES15
              ________      ________
                    0             0


           LAMBDA
              KESCAT16      PHY15         KES15         CHOICE15      BULLY15
              ________      ________      ________      ________      ________
```

KESCAT16	0	0	0	0	0
PHY15	0	0	0	0	0
KES15	0	0	0	0	0
CHOICE15	0	0	0	0	0
BULLY15	0	0	0	0	0
PRESS15	0	0	0	0	0
DUTIES15	0	0	0	0	0

LAMBDA

	PRESS15	DUTIES15
KESCAT16	0	0
PHY15	0	0
KES15	0	0
CHOICE15	0	0
BULLY15	0	0
PRESS15	0	0
DUTIES15	0	0

THETA

	KESCAT16	PHY15	KES15	CHOICE15	BULLY15
KESCAT16	0				
PHY15	0	0			
KES15	0	0	0		
CHOICE15	0	0	0	0	
BULLY15	0	0	0	0	0
PRESS15	0	0	0	0	0
DUTIES15	0	0	0	0	0

THETA

	PRESS15	DUTIES15
PRESS15	0	
DUTIES15	0	0

ALPHA

KESCAT16	PHY15	KES15	CHOICE15	BULLY15
0	1	2	3	0

ALPHA

PRESS15	DUTIES15
4	5

BETA

	KESCAT16	PHY15	KES15	CHOICE15	BULLY15
KESCAT16	0	6	7	8	0
PHY15	0	0	0	0	0
KES15	0	0	0	0	0
CHOICE15	0	0	0	0	0
BULLY15	0	0	0	0	0
PRESS15	0	0	0	0	0
DUTIES15	0	0	0	0	0

BETA

	PRESS15	DUTIES15
KESCAT16	9	10
PHY15	0	0
KES15	0	0
CHOICE15	0	0
BULLY15	0	0
PRESS15	0	0
DUTIES15	0	0

PSI

	KESCAT16	PHY15	KES15	CHOICE15	BULLY15
KESCAT16	0				
PHY15	0	11			
KES15	0	12	13		
CHOICE15	0	14	15	16	
BULLY15	0	17	18	19	20
PRESS15	0	21	22	23	24
DUTIES15	0	26	27	28	29

PSI

	PRESS15	DUTIES15
PRESS15	25	
DUTIES15	30	31

PARAMETER SPECIFICATION FOR BETWEEN

TAU

KESCAT16
43

NU

KESCAT16	PRAFAC	BULLY15
0	0	0

LAMBDA

	S1	KESCAT16	PRAFAC	BULLY15
KESCAT16	0	0	0	0
PRAFAC	0	0	0	0
BULLY15	0	0	0	0

THETA

	KESCAT16	PRAFAC	BULLY15
KESCAT16	0		
PRAFAC	0	0	
BULLY15	0	0	0

ALPHA

S1	KESCAT16	PRAFAC	BULLY15
32	0	33	34

BETA

	S1	KESCAT16	PRAFAC	BULLY15
S1	0	0	35	0
KESCAT16	0	0	36	37
PRAFAC	0	0	0	0
BULLY15	0	0	0	0

PSI

	S1	KESCAT16	PRAFAC	BULLY15
S1	38			
KESCAT16	0	39		
PRAFAC	0	0	40	
BULLY15	0	0	41	42

STARTING VALUES FOR WITHIN

TAU

KESCAT16
0.000

NU

KESCAT16	PHY15	KES15	CHOICE15	BULLY15
0.000	0.000	0.000	0.000	0.000

NU

PRESS15	DUTIES15
0.000	0.000

LAMBDA

	KESCAT16	PHY15	KES15	CHOICE15	BULLY15
KESCAT16	1.000	0.000	0.000	0.000	0.000
PHY15	0.000	1.000	0.000	0.000	0.000

KES15	0.000	0.000	1.000	0.000	0.000
CHOICE15	0.000	0.000	0.000	1.000	0.000
BULLY15	0.000	0.000	0.000	0.000	1.000
PRESS15	0.000	0.000	0.000	0.000	0.000
DUTIES15	0.000	0.000	0.000	0.000	0.000

LAMBDA

	PRESS15	DUTIES15
KESCAT16	0.000	0.000
PHY15	0.000	0.000
KES15	0.000	0.000
CHOICE15	0.000	0.000
BULLY15	0.000	0.000
PRESS15	1.000	0.000
DUTIES15	0.000	1.000

THETA

	KESCAT16	PHY15	KES15	CHOICE15	BULLY15
KESCAT16	0.000				
PHY15	0.000	0.000			
KES15	0.000	0.000	0.000		
CHOICE15	0.000	0.000	0.000	0.000	
BULLY15	0.000	0.000	0.000	0.000	0.000
PRESS15	0.000	0.000	0.000	0.000	0.000
DUTIES15	0.000	0.000	0.000	0.000	0.000

THETA

	PRESS15	DUTIES15
PRESS15	0.000	
DUTIES15	0.000	0.000

ALPHA

KESCAT16	PHY15	KES15	CHOICE15	BULLY15
0.000	0.000	0.000	0.000	0.000

ALPHA

PRESS15	DUTIES15
0.000	0.000

BETA

	KESCAT16	PHY15	KES15	CHOICE15	BULLY15
KESCAT16	0.000	0.000	0.000	0.000	0.000
PHY15	0.000	0.000	0.000	0.000	0.000
KES15	0.000	0.000	0.000	0.000	0.000
CHOICE15	0.000	0.000	0.000	0.000	0.000
BULLY15	0.000	0.000	0.000	0.000	0.000
PRESS15	0.000	0.000	0.000	0.000	0.000
DUTIES15	0.000	0.000	0.000	0.000	0.000

BETA

	PRESS15	DUTIES15
KESCAT16	0.000	0.000
PHY15	0.000	0.000
KES15	0.000	0.000
CHOICE15	0.000	0.000
BULLY15	0.000	0.000
PRESS15	0.000	0.000
DUTIES15	0.000	0.000

PSI

	KESCAT16	PHY15	KES15	CHOICE15	BULLY15
KESCAT16	1.000				
PHY15	0.000	0.268			
KES15	0.000	0.000	0.048		
CHOICE15	0.000	0.000	0.000	0.549	
BULLY15	0.000	0.000	0.000	0.000	0.108
PRESS15	0.000	0.000	0.000	0.000	0.000
DUTIES15	0.000	0.000	0.000	0.000	0.000

PSI

PRESS15	DUTIES15

```
              ________      ________
 PRESS15        0.501
 DUTIES15       0.000         0.414


     STARTING VALUES FOR BETWEEN


           TAU
              KESCAT16
              ________
                1.091


           NU
              KESCAT16      PRAFAC        BULLY15
              ________      ________      ________
                0.000         0.000         0.000


           LAMBDA
              S1            KESCAT16      PRAFAC        BULLY15
              ________      ________      ________      ________
 KESCAT16       0.000         1.000         0.000         0.000
 PRAFAC         0.000         0.000         1.000         0.000
 BULLY15        0.000         0.000         0.000         1.000


           THETA
              KESCAT16      PRAFAC        BULLY15
              ________      ________      ________
 KESCAT16       0.000
 PRAFAC         0.000         0.000
 BULLY15        0.000         0.000         0.000


           ALPHA
              S1            KESCAT16      PRAFAC        BULLY15
              ________      ________      ________      ________
                0.000         0.000         0.006         1.153


           BETA
              S1            KESCAT16      PRAFAC        BULLY15
```

S1	0.000	0.000	0.000	0.000
KESCAT16	0.000	0.000	0.000	0.000
PRAFAC	0.000	0.000	0.000	0.000
BULLY15	0.000	0.000	0.000	0.000

PSI

	S1	KESCAT16	PRAFAC	BULLY15
S1	1.000			
KESCAT16	0.000	1.000		
PRAFAC	0.000	0.000	0.506	
BULLY15	0.000	0.000	0.000	0.108

PRIORS FOR ALL PARAMETERS	PRIOR MEAN	PRIOR VARIANCE	PRIOR STD. DEV.
Parameter 1~N(0.000,infinity)	0.0000	infinity	infinity
Parameter 2~N(0.000,infinity)	0.0000	infinity	infinity
Parameter 3~N(0.000,infinity)	0.0000	infinity	infinity
Parameter 4~N(0.000,infinity)	0.0000	infinity	infinity
Parameter 5~N(0.000,infinity)	0.0000	infinity	infinity
Parameter 6~N(0.000,5.000)	0.0000	5.0000	2.2361
Parameter 7~N(0.000,5.000)	0.0000	5.0000	2.2361
Parameter 8~N(0.000,5.000)	0.0000	5.0000	2.2361
Parameter 9~N(0.000,5.000)	0.0000	5.0000	2.2361
Parameter 10~N(0.000,5.000)	0.0000	5.0000	2.2361
Parameter 11~IW(1.000,7)	infinity	infinity	infinity
Parameter 12~IW(0.000,7)	infinity	infinity	infinity
Parameter 13~IW(1.000,7)	infinity	infinity	infinity
Parameter 14~IW(0.000,7)	infinity	infinity	infinity
Parameter 15~IW(0.000,7)	infinity	infinity	infinity
Parameter 16~IW(1.000,7)	infinity	infinity	infinity
Parameter 17~IW(0.000,7)	infinity	infinity	infinity
Parameter 18~IW(0.000,7)	infinity	infinity	infinity
Parameter 19~IW(0.000,7)	infinity	infinity	infinity
Parameter 20~IW(1.000,7)	infinity	infinity	infinity
Parameter 21~IW(0.000,7)	infinity	infinity	infinity
Parameter 22~IW(0.000,7)	infinity	infinity	infinity
Parameter 23~IW(0.000,7)	infinity	infinity	infinity
Parameter 24~IW(0.000,7)	infinity	infinity	infinity
Parameter 25~IW(1.000,7)	infinity	infinity	infinity

Parameter 26~IW(0.000,7)	infinity	infinity	infinity
Parameter 27~IW(0.000,7)	infinity	infinity	infinity
Parameter 28~IW(0.000,7)	infinity	infinity	infinity
Parameter 29~IW(0.000,7)	infinity	infinity	infinity
Parameter 30~IW(0.000,7)	infinity	infinity	infinity
Parameter 31~IW(1.000,7)	infinity	infinity	infinity
Parameter 32~N(0.000,infinity)	0.0000	infinity	infinity
Parameter 33~N(0.000,infinity)	0.0000	infinity	infinity
Parameter 34~N(0.000,infinity)	0.0000	infinity	infinity
Parameter 35~N(0.000,infinity)	0.0000	infinity	infinity
Parameter 36~N(0.000,5.000)	0.0000	5.0000	2.2361
Parameter 37~N(0.000,5.000)	0.0000	5.0000	2.2361
Parameter 38~IG(-1.000,0.000)	infinity	infinity	infinity
Parameter 39~IG(-1.000,0.000)	infinity	infinity	infinity
Parameter 40~IW(1.000,3)	infinity	infinity	infinity
Parameter 41~IW(0.000,3)	infinity	infinity	infinity
Parameter 42~IW(1.000,3)	infinity	infinity	infinity
Parameter 43~N(0.000,5.000)	0.0000	5.0000	2.2361

TECHNICAL 3 OUTPUT

ESTIMATED COVARIANCE MATRIX FOR PARAMETER ESTIMATES

	1	2	3	4	5
1	0.994124D-04				
2	0.101209D-04	0.165201D-04			
3	-0.176587D-04	-0.501022D-05	0.213048D-03		
4	0.101299D-04	0.103228D-04	-0.266666D-04	0.180576D-03	
5	-0.126943D-04	-0.110654D-04	0.303954D-04	-0.354107D-04	0.165797D-03
6	-0.792586D-05	-0.408550D-05	-0.371366D-05	-0.202537D-04	0.196811D-05
7	0.626605D-05	0.459606D-05	0.340974D-04	0.404758D-05	0.214869D-04
8	-0.613200D-05	-0.366903D-05	0.170263D-04	-0.132157D-04	0.519267D-05
9	-0.469133D-05	-0.294362D-05	-0.185399D-05	0.528046D-05	0.105170D-06
10	-0.129254D-04	-0.387157D-05	-0.157306D-05	-0.174775D-05	0.235956D-04
11	0.506719D-06	-0.218616D-06	0.284571D-05	0.411673D-05	-0.353963D-05
12	0.657804D-06	0.299980D-08	0.158311D-05	-0.405630D-06	0.584909D-06
13	-0.355381D-06	0.156710D-06	0.127590D-05	-0.742315D-06	0.248617D-06
14	0.224737D-05	0.186845D-05	0.172610D-05	0.547130D-05	-0.121744D-07
15	0.978542D-06	0.678436D-06	-0.236333D-05	0.361939D-05	-0.191862D-05
16	0.332545D-05	-0.349976D-05	0.856925D-05	0.461986D-05	0.966325D-05
17	0.147694D-05	0.131771D-06	0.947488D-06	0.214166D-05	0.557714D-06
18	0.409831D-06	0.311053D-06	0.149315D-05	0.194411D-06	-0.598474D-06

19	0.651177D-06	0.182734D-05	-0.334252D-05	-0.270424D-05	-0.194860D-05
20	-0.111535D-05	0.435454D-06	0.523493D-06	0.119386D-05	-0.127489D-06
21	-0.946838D-07	0.407789D-06	0.162925D-06	0.201189D-05	0.648974D-05
22	0.614941D-06	0.298449D-06	-0.164525D-05	0.183387D-05	0.102110D-05
23	0.457208D-05	0.422932D-05	0.397384D-05	0.525956D-06	-0.100441D-04
24	-0.185164D-06	0.323023D-07	-0.357293D-05	-0.119423D-05	0.323964D-06
25	-0.113981D-04	0.261507D-05	-0.131031D-04	-0.301405D-05	-0.331117D-05
26	0.211481D-05	-0.392689D-06	-0.624664D-05	0.382346D-06	0.779753D-06
27	-0.118545D-05	-0.904193D-06	-0.286631D-05	-0.810454D-06	0.249858D-05
28	-0.600125D-05	-0.631423D-06	-0.320854D-06	0.861869D-05	-0.315781D-06
29	-0.950177D-06	-0.640760D-06	-0.223756D-05	-0.146392D-05	0.173858D-05
30	0.200422D-05	0.105011D-05	-0.324643D-05	-0.116305D-05	0.144864D-05
31	0.266702D-05	0.198458D-05	0.304937D-05	0.704222D-05	0.990521D-06
32	0.238058D-05	0.262140D-05	0.640613D-04	-0.173323D-04	0.192457D-04
33	-0.218205D-04	0.157429D-04	-0.144239D-04	0.752309D-04	-0.288809D-04
34	0.285503D-05	0.372342D-05	-0.135160D-04	0.174810D-04	-0.244474D-04
35	0.801292D-06	-0.337674D-05	0.963711D-05	-0.101438D-04	0.144983D-04
36	0.154184D-05	-0.157149D-05	0.792077D-05	0.541539D-05	-0.255985D-05
37	-0.166703D-03	0.716298D-05	-0.222921D-03	0.827012D-04	-0.129828D-03
38	0.157933D-05	-0.370029D-05	0.321815D-04	-0.160077D-04	-0.250001D-05
39	-0.190182D-06	-0.361631D-06	0.287813D-05	0.498146D-06	-0.358841D-06
40	-0.337901D-04	-0.225247D-04	-0.263578D-04	0.926683D-05	-0.741580D-04
41	0.948418D-05	-0.150928D-05	0.652889D-05	-0.564025D-05	-0.644870D-06
42	0.903873D-06	0.122674D-05	0.326309D-05	-0.210843D-05	-0.114280D-05
43	-0.194537D-03	0.570723D-05	-0.244143D-03	0.655311D-04	-0.148768D-03

ESTIMATED COVARIANCE MATRIX FOR PARAMETER ESTIMATES

	6	7	8	9	10
6	0.112255D-02				
7	-0.632267D-03	0.759780D-02			
8	0.102953D-03	-0.140750D-05	0.673456D-03		
9	-0.553963D-04	-0.251260D-03	0.146227D-04	0.688629D-03	
10	-0.399667D-04	0.208753D-03	-0.811444D-04	0.938542D-04	0.761426D-03
11	0.247127D-05	0.243152D-04	0.114579D-04	0.609548D-05	-0.114153D-04
12	-0.655072D-06	0.105194D-04	0.982470D-06	0.201920D-05	-0.708242D-06
13	0.222438D-06	0.764004D-06	0.288406D-05	0.166258D-05	-0.267535D-06
14	-0.555337D-05	0.211466D-04	0.579725D-05	-0.659177D-05	0.124816D-04
15	-0.166991D-05	0.819059D-05	0.663618D-06	-0.196811D-05	0.340153D-05
16	-0.326907D-04	-0.250657D-04	-0.686719D-05	0.125429D-04	-0.170798D-04
17	-0.665359D-05	0.976007D-05	-0.551093D-05	0.295661D-05	-0.979713D-06
18	0.678893D-06	-0.186305D-05	0.122495D-05	0.311638D-06	0.108965D-05
19	0.489233D-05	-0.166952D-04	0.621652D-06	0.416989D-06	0.246260D-05

20	-0.268433D-05	0.144403D-04	-0.209600D-05	0.259685D-05	-0.390716D-05
21	-0.909147D-05	0.442882D-04	0.664104D-05	0.114881D-04	0.725039D-05
22	-0.143126D-07	-0.288664D-05	0.419804D-05	0.759732D-05	-0.201868D-05
23	0.754490D-05	0.226392D-04	0.654326D-05	0.417500D-07	0.235107D-04
24	-0.420154D-05	0.157022D-04	0.539635D-06	-0.200213D-05	-0.105690D-05
25	-0.841079D-05	-0.642367D-05	-0.788027D-05	-0.108650D-04	0.849200D-05
26	0.448218D-05	-0.967287D-05	-0.140449D-04	0.158299D-05	0.549919D-05
27	-0.104465D-05	0.174356D-04	-0.326402D-05	-0.133551D-06	0.896700D-05
28	-0.688649D-06	0.434035D-04	-0.822279D-05	-0.715340D-05	0.808540D-05
29	-0.331056D-05	-0.130882D-04	0.139850D-05	0.535218D-05	0.629638D-05
30	-0.285711D-05	0.134031D-04	0.770389D-05	-0.126199D-04	0.130635D-04
31	-0.101090D-04	0.520524D-04	-0.438139D-05	0.231413D-05	-0.849944D-05
32	-0.170377D-03	-0.556277D-04	0.821819D-04	-0.254814D-03	0.578595D-04
33	-0.171481D-03	-0.204120D-03	-0.114305D-03	-0.326400D-04	-0.133263D-03
34	0.187745D-04	0.236896D-04	0.336553D-04	-0.700046D-05	-0.915088D-05
35	0.132304D-04	-0.116068D-03	-0.405120D-04	-0.604500D-04	-0.161692D-04
36	0.622030D-04	0.522298D-04	-0.104487D-04	0.172405D-04	-0.175291D-05
37	-0.267964D-03	-0.256049D-02	0.482602D-03	0.123799D-04	-0.182687D-03
38	-0.297451D-04	0.247209D-03	-0.625494D-04	-0.574163D-05	-0.601171D-04
39	-0.738549D-06	0.261866D-04	0.788384D-05	0.802873D-05	-0.330640D-05
40	-0.289052D-04	-0.199118D-04	-0.127786D-03	-0.470755D-04	0.242587D-03
41	-0.938159D-05	-0.608034D-04	0.352375D-05	-0.676881D-05	0.418604D-04
42	0.365309D-05	0.151124D-04	0.650568D-05	-0.454096D-05	0.857490D-06
43	-0.260641D-03	-0.181502D-02	0.470918D-03	-0.886243D-05	-0.237590D-03

ESTIMATED COVARIANCE MATRIX FOR PARAMETER ESTIMATES

	11	12	13	14	15
11	0.105267D-03				
12	0.105694D-04	0.104175D-04			
13	0.166391D-05	0.186823D-05	0.328044D-05		
14	-0.199906D-04	-0.617928D-05	-0.159859D-05	0.112542D-03	
15	-0.172698D-05	-0.254003D-05	-0.138250D-05	0.118646D-04	0.203392D-04
16	0.671568D-05	0.217583D-05	-0.120037D-06	-0.323230D-04	-0.117945D-04
17	0.387017D-05	0.211286D-05	0.335985D-06	-0.581101D-05	0.653022D-07
18	0.630414D-06	0.643380D-06	0.955224D-06	-0.962036D-06	-0.101849D-05
19	-0.285570D-05	-0.191877D-05	-0.945103D-06	0.464321D-05	0.550498D-05
20	0.268543D-05	0.345683D-06	0.316797D-06	-0.215558D-06	0.209369D-06
21	0.798479D-05	0.542108D-05	0.676642D-06	-0.109921D-04	-0.582084D-06
22	0.461643D-06	0.822248D-06	0.185230D-05	-0.807816D-06	-0.177163D-05
23	0.513023D-06	-0.129599D-05	-0.734366D-06	0.161991D-04	0.116295D-04
24	-0.793430D-07	0.103836D-06	0.289947D-06	0.357559D-06	-0.231539D-06
25	0.633882D-05	0.151743D-05	0.125595D-05	-0.589434D-05	-0.105639D-05

26	-0.111948D-04	-0.572571D-05	-0.136438D-05	0.200517D-04	0.615051D-06
27	-0.242046D-05	-0.173125D-05	-0.149007D-05	0.305520D-05	0.252451D-05
28	0.201062D-05	0.722617D-06	0.711159D-06	-0.115902D-04	-0.110707D-04
29	-0.843697D-06	-0.828543D-06	-0.508216D-06	-0.155179D-05	0.538567D-07
30	-0.394597D-05	-0.153983D-05	-0.346639D-06	0.339932D-05	0.161147D-05
31	0.483046D-05	0.784581D-07	0.220536D-06	-0.626450D-05	-0.125922D-05
32	0.964222D-05	0.798902D-05	0.134992D-05	-0.266494D-04	-0.129814D-05
33	0.644986D-05	-0.241502D-05	-0.585825D-05	-0.735595D-04	0.170231D-04
34	-0.187883D-05	0.616289D-06	-0.254771D-06	0.104168D-04	0.176915D-05
35	0.377705D-05	0.249331D-05	0.240640D-05	-0.384044D-04	-0.121911D-05
36	0.683754D-05	-0.213317D-05	0.561121D-07	-0.168902D-05	-0.523567D-05
37	-0.151782D-04	-0.536715D-04	0.439066D-05	-0.986240D-05	0.825244D-04
38	-0.691606D-05	-0.569180D-05	-0.606727D-05	-0.966543D-05	0.565467D-05
39	0.118426D-05	0.435346D-06	-0.310803D-06	-0.131642D-05	-0.533499D-06
40	0.655243D-04	-0.337852D-05	0.190326D-05	0.165934D-04	0.330148D-06
41	-0.583710D-05	-0.235204D-05	0.997523D-06	0.190252D-04	0.610826D-05
42	0.691618D-06	-0.337678D-06	0.291923D-07	0.213258D-05	0.201819D-06
43	-0.158938D-04	-0.610089D-04	0.337052D-05	-0.868722D-05	0.938550D-04

ESTIMATED COVARIANCE MATRIX FOR PARAMETER ESTIMATES

	16	17	18	19	20
16	0.463961D-03				
17	0.173039D-05	0.222517D-04			
18	-0.229714D-06	0.268029D-05	0.404499D-05		
19	-0.145442D-04	-0.454173D-05	-0.141659D-05	0.464421D-04	
20	0.934266D-06	0.250871D-05	0.184670D-05	-0.380065D-05	0.184527D-04
21	-0.961308D-05	0.883809D-05	0.114792D-05	-0.275866D-05	-0.155717D-06
22	-0.782046D-06	0.527108D-06	0.188813D-05	-0.204033D-05	-0.914587D-08
23	-0.342200D-04	0.247094D-05	-0.420729D-06	0.187141D-04	0.153185D-05
24	-0.224435D-06	0.300611D-05	0.247617D-05	-0.247610D-05	0.880187D-05
25	-0.644211D-05	0.263149D-05	0.136003D-05	-0.215610D-05	0.155662D-05
26	-0.768673D-05	-0.456885D-05	-0.721971D-06	0.373845D-05	-0.187820D-05
27	-0.407881D-05	-0.523059D-06	-0.116880D-05	0.104794D-05	-0.414998D-06
28	0.640354D-04	0.220113D-05	0.575214D-06	-0.110968D-04	0.129952D-05
29	-0.639888D-05	-0.186460D-05	-0.160755D-05	0.622092D-05	-0.512473D-05
30	-0.583062D-05	0.262154D-06	-0.222603D-06	0.219046D-05	-0.950313D-06
31	0.161992D-04	0.411460D-05	0.177888D-06	-0.324594D-05	-0.449201D-07
32	0.761524D-04	0.107356D-04	0.621276D-05	-0.138638D-04	-0.879210D-05
33	0.119750D-03	0.346227D-04	-0.102202D-04	0.253913D-04	0.534668D-06
34	0.740017D-06	0.661672D-06	0.156843D-05	-0.112169D-05	0.147539D-05
35	-0.180757D-04	-0.124274D-05	-0.171443D-05	-0.533551D-05	-0.134339D-04
36	0.824500D-05	0.314670D-05	0.376030D-06	-0.290740D-05	-0.260748D-05

37	-0.334694D-03	-0.572208D-04	0.963420D-06	0.239978D-03	0.139507D-04
38	-0.252906D-04	-0.139998D-05	-0.916810D-05	-0.546396D-05	-0.115655D-04
39	0.570831D-05	0.143149D-05	-0.333281D-06	0.860727D-06	0.265278D-06
40	-0.303182D-03	0.374318D-05	0.296603D-04	-0.741760D-04	-0.162849D-04
41	0.190402D-04	-0.696676D-06	0.274727D-05	-0.582475D-05	0.419829D-06
42	0.121196D-05	-0.764311D-07	0.138181D-06	0.987756D-06	0.657063D-06
43	-0.375725D-03	-0.684399D-04	-0.676673D-06	0.275683D-03	0.168780D-04

ESTIMATED COVARIANCE MATRIX FOR PARAMETER ESTIMATES

	21	22	23	24	25
21	0.106390D-03				
22	0.103150D-04	0.173758D-04			
23	-0.134115D-04	-0.108501D-04	0.213364D-03		
24	0.274598D-05	0.436920D-05	-0.895142D-05	0.430713D-04	
25	0.206480D-04	0.189800D-04	-0.231807D-04	0.324946D-04	0.390406D-03
26	-0.177995D-04	-0.215858D-05	0.301275D-05	-0.577226D-06	-0.400008D-05
27	-0.231378D-05	-0.285846D-05	0.345997D-05	-0.539387D-06	-0.162674D-05
28	-0.181215D-05	0.254710D-05	-0.340627D-04	0.477547D-05	-0.259327D-05
29	-0.345358D-05	-0.106720D-05	0.792862D-06	-0.650535D-05	-0.466413D-05
30	-0.116519D-04	-0.118814D-04	0.304425D-04	-0.117105D-04	-0.582336D-04
31	0.390903D-05	0.318229D-05	-0.135883D-04	0.445685D-05	0.151668D-04
32	0.179096D-04	-0.587622D-05	-0.111178D-04	0.291560D-05	0.642213D-04
33	-0.652148D-05	-0.146816D-04	0.102139D-05	-0.124868D-04	0.171875D-03
34	-0.400338D-05	0.166204D-06	-0.853292D-05	0.249803D-05	0.226794D-04
35	0.650884D-05	-0.499385D-05	0.223768D-04	-0.677453D-05	0.465148D-05
36	0.274549D-05	0.315286D-05	-0.163691D-04	0.153739D-05	0.123767D-04
37	0.735937D-04	0.140448D-04	0.668395D-04	0.113451D-03	0.246009D-03
38	-0.526277D-07	0.294081D-05	-0.191420D-04	0.640354D-06	-0.574671D-05
39	0.275398D-05	0.799534D-06	0.326177D-06	0.770757D-06	0.501250D-06
40	0.147981D-03	0.362100D-04	-0.115692D-03	-0.272057D-04	0.123449D-04
41	-0.188917D-05	0.576500D-05	0.159129D-04	0.732160D-05	0.236616D-04
42	-0.262247D-06	0.749704D-06	-0.773506D-08	0.149440D-07	0.564000D-05
43	0.839243D-04	0.135328D-04	0.761573D-04	0.124551D-03	0.279490D-03

ESTIMATED COVARIANCE MATRIX FOR PARAMETER ESTIMATES

	26	27	28	29	30
26	0.857843D-04				
27	0.957830D-05	0.148180D-04			
28	-0.163732D-04	-0.627135D-05	0.184058D-03		
29	0.257657D-05	0.339923D-05	-0.801356D-05	0.342249D-04	

30	0.914719D-05	0.975172D-05	-0.130441D-04	0.136576D-04	0.162552D-03
31	-0.227344D-04	-0.159146D-04	0.492919D-04	-0.168517D-04	-0.536204D-04
32	-0.358855D-05	0.111084D-04	-0.160120D-04	-0.486821D-06	0.199894D-04
33	-0.545037D-04	-0.407130D-06	0.127811D-04	-0.295809D-04	-0.159778D-04
34	0.580242D-06	-0.157440D-05	0.365781D-05	-0.346656D-05	-0.426533D-05
35	0.782609D-05	-0.162838D-05	-0.214446D-04	0.116484D-04	0.126064D-05
36	0.167122D-05	0.139634D-05	0.891532D-05	-0.838493D-05	0.103668D-04
37	0.213699D-03	-0.454433D-05	-0.297892D-03	0.727076D-04	-0.895644D-04
38	-0.175881D-04	0.382910D-05	0.238981D-05	0.660001D-05	-0.131393D-04
39	0.146735D-06	-0.305284D-06	-0.130249D-06	-0.118042D-05	-0.233168D-05
40	0.540591D-05	0.269395D-05	-0.704745D-04	-0.147952D-04	0.156982D-04
41	0.173008D-06	-0.559419D-05	0.112080D-05	-0.336851D-05	0.337186D-05
42	0.897967D-06	-0.596970D-06	0.394892D-06	-0.605893D-06	-0.851903D-06
43	0.231989D-03	-0.287316D-05	-0.352536D-03	0.844377D-04	-0.117399D-03

ESTIMATED COVARIANCE MATRIX FOR PARAMETER ESTIMATES

	31	32	33	34	35
31	0.250713D-03				
32	-0.348385D-04	0.491138D-02			
33	0.205326D-04	-0.105366D-03	0.230809D-01		
34	0.195714D-05	-0.379067D-05	0.230767D-03	0.499157D-03	
35	-0.166064D-04	0.444048D-03	-0.396947D-03	-0.552731D-04	0.401716D-02
36	-0.109125D-05	-0.123602D-04	-0.180304D-04	-0.335412D-04	-0.116835D-03
37	-0.140365D-03	0.196724D-03	-0.263530D-02	0.811057D-04	0.773129D-03
38	0.182578D-04	0.291505D-03	-0.156106D-03	-0.237588D-04	-0.266604D-03
39	-0.163134D-05	-0.755867D-05	0.952646D-05	0.208107D-05	0.824023D-05
40	0.646109D-04	-0.581414D-03	-0.818057D-03	0.503696D-04	-0.271649D-03
41	-0.405354D-05	0.755372D-04	0.984758D-04	0.303108D-05	-0.757704D-04
42	0.812302D-06	0.407409D-05	0.255722D-05	0.803047D-05	-0.589518D-05
43	-0.144077D-03	0.343222D-03	-0.309734D-02	0.330187D-04	0.936463D-03

ESTIMATED COVARIANCE MATRIX FOR PARAMETER ESTIMATES

	36	37	38	39	40
36	0.821599D-03				
37	-0.273029D-02	0.307677D+00			
38	0.423210D-04	-0.824060D-03	0.308532D-02		
39	0.177384D-04	0.655965D-03	0.194256D-04	0.483383D-04	
40	0.140635D-03	-0.252914D-02	0.515294D-03	-0.167068D-04	0.723834D-01
41	0.540112D-04	0.565203D-03	0.215799D-05	-0.346669D-05	0.623099D-03
42	-0.394455D-05	0.203223D-03	-0.171254D-05	0.146036D-06	0.302830D-04

43	-0.314640D-02	0.353506D+00	-0.828839D-03	0.788207D-03	-0.309049D-02

ESTIMATED COVARIANCE MATRIX FOR PARAMETER ESTIMATES

	41	42	43
41	0.779732D-03		
42	0.105495D-04	0.265948D-04	
43	0.637024D-03	0.230323D-03	0.407271D+00

ESTIMATED CORRELATION MATRIX FOR PARAMETER ESTIMATES

	1	2	3	4	5
1	1.000				
2	0.250	1.000			
3	-0.121	-0.084	1.000		
4	0.076	0.189	-0.136	1.000	
5	-0.099	-0.211	0.162	-0.205	1.000
6	-0.024	-0.030	-0.008	-0.045	0.005
7	0.007	0.013	0.027	0.003	0.019
8	-0.024	-0.035	0.045	-0.038	0.016
9	-0.018	-0.028	-0.005	0.015	0.000
10	-0.047	-0.035	-0.004	-0.005	0.066
11	0.005	-0.005	0.019	0.030	-0.027
12	0.020	0.000	0.034	-0.009	0.014
13	-0.020	0.021	0.048	-0.030	0.011
14	0.021	0.043	0.011	0.038	0.000
15	0.022	0.037	-0.036	0.060	-0.033
16	0.015	-0.040	0.027	0.016	0.035
17	0.031	0.007	0.014	0.034	0.009
18	0.020	0.038	0.051	0.007	-0.023
19	0.010	0.066	-0.034	-0.030	-0.022
20	-0.026	0.025	0.008	0.021	-0.002
21	-0.001	0.010	0.001	0.015	0.049
22	0.015	0.018	-0.027	0.033	0.019
23	0.031	0.071	0.019	0.003	-0.053
24	-0.003	0.001	-0.037	-0.014	0.004
25	-0.058	0.033	-0.045	-0.011	-0.013
26	0.023	-0.010	-0.046	0.003	0.007
27	-0.031	-0.058	-0.051	-0.016	0.050
28	-0.044	-0.011	-0.002	0.047	-0.002
29	-0.016	-0.027	-0.026	-0.019	0.023
30	0.016	0.020	-0.017	-0.007	0.009

31	0.017	0.031	0.013	0.033	0.005
32	0.003	0.009	0.063	-0.018	0.021
33	-0.014	0.025	-0.007	0.037	-0.015
34	0.013	0.041	-0.041	0.058	-0.085
35	0.001	-0.013	0.010	-0.012	0.018
36	0.005	-0.013	0.019	0.014	-0.007
37	-0.030	0.003	-0.028	0.011	-0.018
38	0.003	-0.016	0.040	-0.021	-0.003
39	-0.003	-0.013	0.028	0.005	-0.004
40	-0.013	-0.021	-0.007	0.003	-0.021
41	0.034	-0.013	0.016	-0.015	-0.002
42	0.018	0.059	0.043	-0.030	-0.017
43	-0.031	0.002	-0.026	0.008	-0.018

ESTIMATED CORRELATION MATRIX FOR PARAMETER ESTIMATES

	6	7	8	9	10
6	1.000				
7	-0.216	1.000			
8	0.118	-0.001	1.000		
9	-0.063	-0.110	0.021	1.000	
10	-0.043	0.087	-0.113	0.130	1.000
11	0.007	0.027	0.043	0.023	-0.040
12	-0.006	0.037	0.012	0.024	-0.008
13	0.004	0.005	0.061	0.035	-0.005
14	-0.016	0.023	0.021	-0.024	0.043
15	-0.011	0.021	0.006	-0.017	0.027
16	-0.045	-0.013	-0.012	0.022	-0.029
17	-0.042	0.024	-0.045	0.024	-0.008
18	0.010	-0.011	0.023	0.006	0.020
19	0.021	-0.028	0.004	0.002	0.013
20	-0.019	0.039	-0.019	0.023	-0.033
21	-0.026	0.049	0.025	0.042	0.025
22	0.000	-0.008	0.039	0.069	-0.018
23	0.015	0.018	0.017	0.000	0.058
24	-0.019	0.027	0.003	-0.012	-0.006
25	-0.013	-0.004	-0.015	-0.021	0.016
26	0.014	-0.012	-0.058	0.007	0.022
27	-0.008	0.052	-0.033	-0.001	0.084
28	-0.002	0.037	-0.023	-0.020	0.022
29	-0.017	-0.026	0.009	0.035	0.039
30	-0.007	0.012	0.023	-0.038	0.037
31	-0.019	0.038	-0.011	0.006	-0.019

32	-0.073	-0.009	0.045	-0.139	0.030
33	-0.034	-0.015	-0.029	-0.008	-0.032
34	0.025	0.012	0.058	-0.012	-0.015
35	0.006	-0.021	-0.025	-0.036	-0.009
36	0.065	0.021	-0.014	0.023	-0.002
37	-0.014	-0.053	0.034	0.001	-0.012
38	-0.016	0.051	-0.043	-0.004	-0.039
39	-0.003	0.043	0.044	0.044	-0.017
40	-0.003	-0.001	-0.018	-0.007	0.033
41	-0.010	-0.025	0.005	-0.009	0.054
42	0.021	0.034	0.049	-0.034	0.006
43	-0.012	-0.033	0.028	-0.001	-0.013

ESTIMATED CORRELATION MATRIX FOR PARAMETER ESTIMATES

	11	12	13	14	15
11	1.000				
12	0.319	1.000			
13	0.090	0.320	1.000		
14	-0.184	-0.180	-0.083	1.000	
15	-0.037	-0.174	-0.169	0.248	1.000
16	0.030	0.031	-0.003	-0.141	-0.121
17	0.080	0.139	0.039	-0.116	0.003
18	0.031	0.099	0.262	-0.045	-0.112
19	-0.041	-0.087	-0.077	0.064	0.179
20	0.061	0.025	0.041	-0.005	0.011
21	0.075	0.163	0.036	-0.100	-0.013
22	0.011	0.061	0.245	-0.018	-0.094
23	0.003	-0.027	-0.028	0.105	0.177
24	-0.001	0.005	0.024	0.005	-0.008
25	0.031	0.024	0.035	-0.028	-0.012
26	-0.118	-0.192	-0.081	0.204	0.015
27	-0.061	-0.139	-0.214	0.075	0.145
28	0.014	0.017	0.029	-0.081	-0.181
29	-0.014	-0.044	-0.048	-0.025	0.002
30	-0.030	-0.037	-0.015	0.025	0.028
31	0.030	0.002	0.008	-0.037	-0.018
32	0.013	0.035	0.011	-0.036	-0.004
33	0.004	-0.005	-0.021	-0.046	0.025
34	-0.008	0.009	-0.006	0.044	0.018
35	0.006	0.012	0.021	-0.057	-0.004
36	0.023	-0.023	0.001	-0.006	-0.041
37	-0.003	-0.030	0.004	-0.002	0.033

38	-0.012	-0.032	-0.060	-0.016	0.023
39	0.017	0.019	-0.025	-0.018	-0.017
40	0.024	-0.004	0.004	0.006	0.000
41	-0.020	-0.026	0.020	0.064	0.049
42	0.013	-0.020	0.003	0.039	0.009
43	-0.002	-0.030	0.003	-0.001	0.033

ESTIMATED CORRELATION MATRIX FOR PARAMETER ESTIMATES

	16	17	18	19	20
16	1.000				
17	0.017	1.000			
18	-0.005	0.283	1.000		
19	-0.099	-0.141	-0.103	1.000	
20	0.010	0.124	0.214	-0.130	1.000
21	-0.043	0.182	0.055	-0.039	-0.004
22	-0.009	0.027	0.225	-0.072	-0.001
23	-0.109	0.036	-0.014	0.188	0.024
24	-0.002	0.097	0.188	-0.055	0.312
25	-0.015	0.028	0.034	-0.016	0.018
26	-0.039	-0.105	-0.039	0.059	-0.047
27	-0.049	-0.029	-0.151	0.040	-0.025
28	0.219	0.034	0.021	-0.120	0.022
29	-0.051	-0.068	-0.137	0.156	-0.204
30	-0.021	0.004	-0.009	0.025	-0.017
31	0.047	0.055	0.006	-0.030	-0.001
32	0.050	0.032	0.044	-0.029	-0.029
33	0.037	0.048	-0.033	0.025	0.001
34	0.002	0.006	0.035	-0.007	0.015
35	-0.013	-0.004	-0.013	-0.012	-0.049
36	0.013	0.023	0.007	-0.015	-0.021
37	-0.028	-0.022	0.001	0.063	0.006
38	-0.021	-0.005	-0.082	-0.014	-0.048
39	0.038	0.044	-0.024	0.018	0.009
40	-0.052	0.003	0.055	-0.040	-0.014
41	0.032	-0.005	0.049	-0.031	0.004
42	0.011	-0.003	0.013	0.028	0.030
43	-0.027	-0.023	-0.001	0.063	0.006

ESTIMATED CORRELATION MATRIX FOR PARAMETER ESTIMATES

	21	22	23	24	25

21	1.000				
22	0.240	1.000			
23	-0.089	-0.178	1.000		
24	0.041	0.160	-0.093	1.000	
25	0.101	0.230	-0.080	0.251	1.000
26	-0.186	-0.056	0.022	-0.009	-0.022
27	-0.058	-0.178	0.062	-0.021	-0.021
28	-0.013	0.045	-0.172	0.054	-0.010
29	-0.057	-0.044	0.009	-0.169	-0.040
30	-0.089	-0.224	0.163	-0.140	-0.231
31	0.024	0.048	-0.059	0.043	0.048
32	0.025	-0.020	-0.011	0.006	0.046
33	-0.004	-0.023	0.000	-0.013	0.057
34	-0.017	0.002	-0.026	0.017	0.051
35	0.010	-0.019	0.024	-0.016	0.004
36	0.009	0.026	-0.039	0.008	0.022
37	0.013	0.006	0.008	0.031	0.022
38	0.000	0.013	-0.024	0.002	-0.005
39	0.038	0.028	0.003	0.017	0.004
40	0.053	0.032	-0.029	-0.015	0.002
41	-0.007	0.050	0.039	0.040	0.043
42	-0.005	0.035	0.000	0.000	0.055
43	0.013	0.005	0.008	0.030	0.022

ESTIMATED CORRELATION MATRIX FOR PARAMETER ESTIMATES

	26	27	28	29	30
26	1.000				
27	0.269	1.000			
28	-0.130	-0.120	1.000		
29	0.048	0.151	-0.101	1.000	
30	0.077	0.199	-0.075	0.183	1.000
31	-0.155	-0.261	0.229	-0.182	-0.266
32	-0.006	0.041	-0.017	-0.001	0.022
33	-0.039	-0.001	0.006	-0.033	-0.008
34	0.003	-0.018	0.012	-0.027	-0.015
35	0.013	-0.007	-0.025	0.031	0.002
36	0.006	0.013	0.023	-0.050	0.028
37	0.042	-0.002	-0.040	0.022	-0.013
38	-0.034	0.018	0.003	0.020	-0.019
39	0.002	-0.011	-0.001	-0.029	-0.026
40	0.002	0.003	-0.019	-0.009	0.005
41	0.001	-0.052	0.003	-0.021	0.009

42	0.019	-0.030	0.006	-0.020	-0.013
43	0.039	-0.001	-0.041	0.023	-0.014

ESTIMATED CORRELATION MATRIX FOR PARAMETER ESTIMATES

	31	32	33	34	35
31	1.000				
32	-0.031	1.000			
33	0.009	-0.010	1.000		
34	0.006	-0.002	0.068	1.000	
35	-0.017	0.100	-0.041	-0.039	1.000
36	-0.002	-0.006	-0.004	-0.052	-0.064
37	-0.016	0.005	-0.031	0.007	0.022
38	0.021	0.075	-0.018	-0.019	-0.076
39	-0.015	-0.016	0.009	0.013	0.019
40	0.015	-0.031	-0.020	0.008	-0.016
41	-0.009	0.039	0.023	0.005	-0.043
42	0.010	0.011	0.003	0.070	-0.018
43	-0.014	0.008	-0.032	0.002	0.023

ESTIMATED CORRELATION MATRIX FOR PARAMETER ESTIMATES

	36	37	38	39	40
36	1.000				
37	-0.172	1.000			
38	0.027	-0.027	1.000		
39	0.089	0.170	0.050	1.000	
40	0.018	-0.017	0.034	-0.009	1.000
41	0.067	0.036	0.001	-0.018	0.083
42	-0.027	0.071	-0.006	0.004	0.022
43	-0.172	0.999	-0.023	0.178	-0.018

ESTIMATED CORRELATION MATRIX FOR PARAMETER ESTIMATES

	41	42	43
41	1.000		
42	0.073	1.000	
43	0.036	0.070	1.000

TECHNICAL 8 OUTPUT

TECHNICAL 8 OUTPUT FOR BAYES ESTIMATION

CHAIN	BSEED
1	0
2	285380
3	253358

ITERATION	POTENTIAL SCALE REDUCTION	PARAMETER WITH HIGHEST PSR
100	1.051	38
200	1.006	32
300	1.007	9
400	1.002	4
500	1.004	36
600	1.004	37
700	1.002	8
800	1.001	38
900	1.002	38
1000	1.002	9

Worsening of psychological distress, time pressure interaction

SUMMARY OF DATA

Number of clusters 58

Size (s)	Cluster ID with Size s
7	22
17	166
20	80
26	71 74 205
29	65
31	192
33	228
37	182 106
39	72
41	170 181
42	50 30
43	75
45	173
47	226 165
48	108
50	107 114
51	81
52	40
55	84 92
57	162
59	206
61	61 83 116
62	36 93 169
63	69
64	4
69	68
73	174 152
74	6 154
75	145
89	98
90	178 2
95	144
100	156
104	202
106	142
134	122

186	24
192	209
246	204
278	125
405	217
780	110
873	100

COVARIANCE COVERAGE OF DATA

Minimum covariance coverage value 0.100

Number of missing data patterns 9

PROPORTION OF DATA PRESENT

Covariance Coverage

	KESCAT16	PRAFAC	PHY15	KES15	CHOICE15
KESCAT16	1.000				
PRAFAC	1.000	1.000			
PHY15	0.950	0.950	0.950		
KES15	0.919	0.919	0.919	0.919	
CHOICE15	0.913	0.913	0.913	0.913	0.913
BULLY15	0.913	0.913	0.913	0.913	0.912
PRESS15	0.913	0.913	0.913	0.913	0.913
DUTIES15	0.913	0.913	0.913	0.913	0.913

Covariance Coverage

	BULLY15	PRESS15	DUTIES15
BULLY15	0.913		
PRESS15	0.912	0.913	
DUTIES15	0.913	0.913	0.913

UNIVARIATE PROPORTIONS AND COUNTS FOR CATEGORICAL VARIABLES

KESCAT16

Category 1	0.877	5135.000
Category 2	0.123	720.000

UNIVARIATE SAMPLE STATISTICS

UNIVARIATE HIGHER-ORDER MOMENT DESCRIPTIVE STATISTICS

Variable/ Sample Size	Mean/ Variance	Skewness/ Kurtosis	Minimum/ Maximum	% with Min/Max	Percentiles 20%/60%	40%/80%	Median
PRAFAC	-0.185	0.245	-2.338	1.72%	-1.448	-0.527	-0.318
58.000	1.345	-0.636	2.338	1.72%	0.080	0.752	
PHY15	0.000	0.526	-0.865	32.48%	-0.865	0.135	0.135
5563.000	0.536	0.021	3.135	0.07%	0.135	0.135	
KES15	0.000	0.475	-0.374	22.78%	-0.374	-0.207	-0.041
5382.000	0.095	-0.842	0.626	6.60%	-0.041	0.293	
CHOICE15	0.000	-0.400	-2.221	7.67%	-1.221	-0.221	-0.221
5345.000	1.097	-0.356	1.779	8.62%	0.779	0.779	
BULLY15	0.000	3.522	-0.153	88.29%	-0.153	-0.153	-0.153
5344.000	0.216	14.164	3.847	0.09%	-0.153	-0.153	
PRESS15	2.445	0.267	1.000	19.70%	2.000	2.000	2.000
5345.000	1.001	-0.357	5.000	2.75%	3.000	3.000	
DUTIES15	0.000	-1.296	-3.149	2.34%	-0.149	-0.149	-0.149
5347.000	0.828	1.939	0.851	39.74%	-0.149	0.851	

THE MODEL ESTIMATION TERMINATED NORMALLY

USE THE FBITERATIONS OPTION TO INCREASE THE NUMBER OF ITERATIONS BY A FACTOR
OF AT LEAST TWO TO CHECK CONVERGENCE AND THAT THE PSR VALUE DOES NOT INCREASE.

MODEL FIT INFORMATION

Number of Free Parameters 43

MODEL RESULTS

	Estimate	Posterior S.D.	One-Tailed P-Value	95% C.I. Lower 2.5%	Upper 2.5%	Significance
**Within Level**						
KESCAT16 ON						
PHY15	0.137	0.033	0.000	0.070	0.203	*
CHOICE15	-0.057	0.026	0.011	-0.108	-0.006	*
DUTIES15	-0.081	0.027	0.001	-0.134	-0.029	*
BULLY15	0.113	0.049	0.013	0.021	0.210	*
KES15	1.615	0.087	0.000	1.447	1.791	*
KES15 WITH						
PHY15	0.054	0.003	0.000	0.048	0.061	*
CHOICE15	-0.037	0.005	0.000	-0.046	-0.028	*
BULLY15	0.022	0.002	0.000	0.018	0.026	*
PRESS15	0.053	0.004	0.000	0.045	0.061	*
DUTIES15	-0.051	0.004	0.000	-0.058	-0.043	*
PHY15 WITH						
CHOICE15	-0.092	0.011	0.000	-0.112	-0.070	*
BULLY15	0.025	0.005	0.000	0.015	0.034	*
PRESS15	0.060	0.010	0.000	0.039	0.080	*
DUTIES15	-0.056	0.009	0.000	-0.074	-0.038	*
CHOICE15 WITH						
BULLY15	-0.052	0.007	0.000	-0.065	-0.039	*
PRESS15	-0.065	0.015	0.000	-0.093	-0.037	*
DUTIES15	0.158	0.014	0.000	0.133	0.186	*
BULLY15 WITH						
PRESS15	0.087	0.006	0.000	0.075	0.099	*
DUTIES15	-0.058	0.006	0.000	-0.070	-0.047	*
PRESS15 WITH						
DUTIES15	-0.149	0.013	0.000	-0.173	-0.124	*
Means						
PHY15	0.002	0.010	0.441	-0.018	0.021	
KES15	0.003	0.004	0.221	-0.005	0.012	
CHOICE15	-0.003	0.015	0.413	-0.032	0.025	
BULLY15	0.001	0.006	0.421	-0.011	0.014	
DUTIES15	-0.003	0.013	0.422	-0.029	0.022	

```
 Variances
    PHY15              0.537      0.010      0.000       0.516      0.557      *
    KES15              0.096      0.002      0.000       0.092      0.100      *
    CHOICE15           1.097      0.022      0.000       1.060      1.141      *
    BULLY15            0.216      0.004      0.000       0.208      0.225      *
    PRESS15            0.959      0.019      0.000       0.923      0.997      *
    DUTIES15           0.829      0.016      0.000       0.799      0.860      *

Between Level

 S1         ON
    PRAFAC            -0.026      0.029      0.201      -0.078      0.033

 KESCAT16   ON
    PRAFAC             0.015      0.028      0.287      -0.039      0.069
    PRESS15           -0.229      0.145      0.054      -0.531      0.058

 PRAFAC   WITH
    PRESS15           -0.004      0.050      0.468      -0.108      0.093

 Means
    PRAFAC            -0.182      0.152      0.119      -0.483      0.115
    PRESS15            2.365      0.040      0.000       2.289      2.449      *

 Intercepts
    S1                -0.028      0.031      0.167      -0.089      0.035

 Thresholds
    KESCAT16$1         0.802      0.352      0.017       0.070      1.490      *

 Variances
    PRAFAC             1.359      0.269      0.000       0.966      1.995      *
    PRESS15            0.074      0.017      0.000       0.048      0.117      *

 Residual Variances
    KESCAT16           0.008      0.007      0.000       0.001      0.027      *
    S1                 0.004      0.006      0.000       0.000      0.021      *


STANDARDIZED MODEL RESULTS


STDYX Standardization
```

	Estimate	Posterior S.D.	One-Tailed P-Value	95% C.I. Lower 2.5%	Upper 2.5%	Significance
**Within-Level Standardized Estimates Averaged Over Clusters**						
S1 | KESCAT16 ON						
PRESS15	-0.020	0.025	0.207	-0.070	0.026	
KESCAT16 ON						
PHY15	0.087	0.021	0.000	0.045	0.129	*
CHOICE15	-0.053	0.023	0.010	-0.098	-0.008	*
DUTIES15	-0.064	0.021	0.002	-0.105	-0.022	*
BULLY15	0.045	0.019	0.014	0.007	0.083	*
KES15	0.433	0.020	0.000	0.394	0.470	*
KES15 WITH						
PHY15	0.240	0.013	0.000	0.214	0.265	*
CHOICE15	-0.113	0.014	0.000	-0.139	-0.085	*
BULLY15	0.154	0.013	0.000	0.128	0.180	*
PRESS15	0.174	0.013	0.000	0.148	0.201	*
DUTIES15	-0.180	0.013	0.000	-0.205	-0.154	*
PHY15 WITH						
CHOICE15	-0.120	0.014	0.000	-0.146	-0.093	*
BULLY15	0.072	0.014	0.000	0.045	0.098	*
PRESS15	0.083	0.014	0.000	0.056	0.110	*
DUTIES15	-0.084	0.014	0.000	-0.111	-0.057	*
CHOICE15 WITH						
BULLY15	-0.106	0.013	0.000	-0.132	-0.079	*
PRESS15	-0.064	0.014	0.000	-0.092	-0.036	*
DUTIES15	0.166	0.014	0.000	0.140	0.193	*
BULLY15 WITH						
PRESS15	0.192	0.013	0.000	0.165	0.217	*
DUTIES15	-0.138	0.013	0.000	-0.164	-0.112	*
PRESS15 WITH						
DUTIES15	-0.167	0.014	0.000	-0.193	-0.140	*
Means						
PHY15	0.002	0.014	0.437	-0.024	0.029	
KES15	0.010	0.013	0.232	-0.016	0.037	
CHOICE15	-0.003	0.014	0.409	-0.030	0.024	

```
    BULLY15            0.003       0.014       0.416      -0.024       0.029
    DUTIES15          -0.003       0.014       0.419      -0.032       0.025

 Variances
    PHY15              1.000       0.000       0.000       1.000       1.000
    KES15              1.000       0.000       0.000       1.000       1.000
    CHOICE15           1.000       0.000       0.000       1.000       1.000
    BULLY15            1.000       0.000       0.000       1.000       1.000
    PRESS15            1.000       0.000       0.000       1.000       1.000
    DUTIES15           1.000       0.000       0.000       1.000       1.000

Between Level

 S1         ON
    PRAFAC            -0.417       0.416       0.201      -0.976       0.527

 KESCAT16   ON
    PRAFAC             0.149       0.266       0.287      -0.399       0.621
    PRESS15           -0.543       0.278       0.054      -0.931       0.146

 PRAFAC   WITH
    PRESS15           -0.013       0.147       0.468      -0.318       0.272

 Means
    PRAFAC            -0.158       0.129       0.119      -0.417       0.093
    PRESS15            8.693       0.986       0.000       6.879      10.811      *

 Intercepts
    S1                -0.355       0.487       0.167      -1.493       0.437

 Variances
    PRAFAC             1.000       0.000       0.000       1.000       1.000
    PRESS15            1.000       0.000       0.000       1.000       1.000

 Residual Variances
    KESCAT16           0.600       0.247       0.000       0.089       0.973      *
    S1                 0.791       0.296       0.000       0.046       0.999      *


R-SQUARE

Within-Level R-Square Averaged Across Clusters

                                Posterior  One-Tailed         95% C.I.
```

```
    Variable        Estimate       S.D.      P-Value   Lower 2.5%  Upper 2.5%

    KESCAT16           0.251       0.017      0.000       0.218       0.286

Between Level

                               Posterior  One-Tailed         95% C.I.
    Variable        Estimate      S.D.      P-Value   Lower 2.5%  Upper 2.5%

    KESCAT16           0.400       0.247      0.000       0.027       0.911

                               Posterior  One-Tailed         95% C.I.
    Variable        Estimate      S.D.      P-Value   Lower 2.5%  Upper 2.5%

    S1                 0.209       0.296      0.000       0.001       0.954


TECHNICAL 1 OUTPUT


     PARAMETER SPECIFICATION FOR WITHIN


           TAU
              KESCAT16
              ________
                    0


           NU
              KESCAT16      PHY15         KES15         CHOICE15      BULLY15
              ________      ________      ________      ________      ________
                    0             0             0             0             0


           NU
              PRESS15       DUTIES15
              ________      ________
                    0             0


           LAMBDA
              KESCAT16      PHY15         KES15         CHOICE15      BULLY15
              ________      ________      ________      ________      ________
```

KESCAT16	0	0	0	0	0
PHY15	0	0	0	0	0
KES15	0	0	0	0	0
CHOICE15	0	0	0	0	0
BULLY15	0	0	0	0	0
PRESS15	0	0	0	0	0
DUTIES15	0	0	0	0	0

LAMBDA

	PRESS15	DUTIES15
KESCAT16	0	0
PHY15	0	0
KES15	0	0
CHOICE15	0	0
BULLY15	0	0
PRESS15	0	0
DUTIES15	0	0

THETA

	KESCAT16	PHY15	KES15	CHOICE15	BULLY15
KESCAT16	0				
PHY15	0	0			
KES15	0	0	0		
CHOICE15	0	0	0	0	
BULLY15	0	0	0	0	0
PRESS15	0	0	0	0	0
DUTIES15	0	0	0	0	0

THETA

	PRESS15	DUTIES15
PRESS15	0	
DUTIES15	0	0

ALPHA

KESCAT16	PHY15	KES15	CHOICE15	BULLY15
0	1	2	3	4

ALPHA

PRESS15	DUTIES15
0	5

BETA

	KESCAT16	PHY15	KES15	CHOICE15	BULLY15
KESCAT16	0	6	7	8	9
PHY15	0	0	0	0	0
KES15	0	0	0	0	0
CHOICE15	0	0	0	0	0
BULLY15	0	0	0	0	0
PRESS15	0	0	0	0	0
DUTIES15	0	0	0	0	0

BETA

	PRESS15	DUTIES15
KESCAT16	0	10
PHY15	0	0
KES15	0	0
CHOICE15	0	0
BULLY15	0	0
PRESS15	0	0
DUTIES15	0	0

PSI

	KESCAT16	PHY15	KES15	CHOICE15	BULLY15
KESCAT16	0				
PHY15	0	11			
KES15	0	12	13		
CHOICE15	0	14	15	16	
BULLY15	0	17	18	19	20
PRESS15	0	21	22	23	24
DUTIES15	0	26	27	28	29

PSI

	PRESS15	DUTIES15
PRESS15	25	
DUTIES15	30	31

PARAMETER SPECIFICATION FOR BETWEEN

TAU

KESCAT16
43

NU

KESCAT16	PRAFAC	PRESS15
0	0	0

LAMBDA

	S1	KESCAT16	PRAFAC	PRESS15
KESCAT16	0	0	0	0
PRAFAC	0	0	0	0
PRESS15	0	0	0	0

THETA

	KESCAT16	PRAFAC	PRESS15
KESCAT16	0		
PRAFAC	0	0	
PRESS15	0	0	0

ALPHA

S1	KESCAT16	PRAFAC	PRESS15
32	0	33	34

BETA

	S1	KESCAT16	PRAFAC	PRESS15
S1	0	0	35	0
KESCAT16	0	0	36	37
PRAFAC	0	0	0	0
PRESS15	0	0	0	0

PSI

	S1	KESCAT16	PRAFAC	PRESS15
S1	38			
KESCAT16	0	39		
PRAFAC	0	0	40	
PRESS15	0	0	41	42

STARTING VALUES FOR WITHIN

TAU

KESCAT16
0.000

NU

KESCAT16	PHY15	KES15	CHOICE15	BULLY15
0.000	0.000	0.000	0.000	0.000

NU

PRESS15	DUTIES15
0.000	0.000

LAMBDA

	KESCAT16	PHY15	KES15	CHOICE15	BULLY15
KESCAT16	1.000	0.000	0.000	0.000	0.000
PHY15	0.000	1.000	0.000	0.000	0.000

KES15	0.000	0.000	1.000	0.000	0.000
CHOICE15	0.000	0.000	0.000	1.000	0.000
BULLY15	0.000	0.000	0.000	0.000	1.000
PRESS15	0.000	0.000	0.000	0.000	0.000
DUTIES15	0.000	0.000	0.000	0.000	0.000

LAMBDA

	PRESS15	DUTIES15
KESCAT16	0.000	0.000
PHY15	0.000	0.000
KES15	0.000	0.000
CHOICE15	0.000	0.000
BULLY15	0.000	0.000
PRESS15	1.000	0.000
DUTIES15	0.000	1.000

THETA

	KESCAT16	PHY15	KES15	CHOICE15	BULLY15
KESCAT16	0.000				
PHY15	0.000	0.000			
KES15	0.000	0.000	0.000		
CHOICE15	0.000	0.000	0.000	0.000	
BULLY15	0.000	0.000	0.000	0.000	0.000
PRESS15	0.000	0.000	0.000	0.000	0.000
DUTIES15	0.000	0.000	0.000	0.000	0.000

THETA

	PRESS15	DUTIES15
PRESS15	0.000	
DUTIES15	0.000	0.000

ALPHA

KESCAT16	PHY15	KES15	CHOICE15	BULLY15
0.000	0.000	0.000	0.000	0.000

ALPHA

PRESS15	DUTIES15
0.000	0.000

BETA

	KESCAT16	PHY15	KES15	CHOICE15	BULLY15
KESCAT16	0.000	0.000	0.000	0.000	0.000
PHY15	0.000	0.000	0.000	0.000	0.000
KES15	0.000	0.000	0.000	0.000	0.000
CHOICE15	0.000	0.000	0.000	0.000	0.000
BULLY15	0.000	0.000	0.000	0.000	0.000
PRESS15	0.000	0.000	0.000	0.000	0.000
DUTIES15	0.000	0.000	0.000	0.000	0.000

BETA

	PRESS15	DUTIES15
KESCAT16	0.000	0.000
PHY15	0.000	0.000
KES15	0.000	0.000
CHOICE15	0.000	0.000
BULLY15	0.000	0.000
PRESS15	0.000	0.000
DUTIES15	0.000	0.000

PSI

	KESCAT16	PHY15	KES15	CHOICE15	BULLY15
KESCAT16	1.000				
PHY15	0.000	0.268			
KES15	0.000	0.000	0.048		
CHOICE15	0.000	0.000	0.000	0.549	
BULLY15	0.000	0.000	0.000	0.000	0.108
PRESS15	0.000	0.000	0.000	0.000	0.000
DUTIES15	0.000	0.000	0.000	0.000	0.000

PSI

PRESS15	DUTIES15

PRESS15	0.501	
DUTIES15	0.000	0.414

STARTING VALUES FOR BETWEEN

TAU

KESCAT16
1.091

NU

KESCAT16	PRAFAC	PRESS15
0.000	0.000	0.000

LAMBDA

	S1	KESCAT16	PRAFAC	PRESS15
KESCAT16	0.000	1.000	0.000	0.000
PRAFAC	0.000	0.000	1.000	0.000
PRESS15	0.000	0.000	0.000	1.000

THETA

	KESCAT16	PRAFAC	PRESS15
KESCAT16	0.000		
PRAFAC	0.000	0.000	
PRESS15	0.000	0.000	0.000

ALPHA

S1	KESCAT16	PRAFAC	PRESS15
0.000	0.000	0.006	2.445

BETA

S1	KESCAT16	PRAFAC	PRESS15

S1	0.000	0.000	0.000	0.000
KESCAT16	0.000	0.000	0.000	0.000
PRAFAC	0.000	0.000	0.000	0.000
PRESS15	0.000	0.000	0.000	0.000

PSI

	S1	KESCAT16	PRAFAC	PRESS15
S1	1.000			
KESCAT16	0.000	1.000		
PRAFAC	0.000	0.000	0.506	
PRESS15	0.000	0.000	0.000	0.501

PRIORS FOR ALL PARAMETERS	PRIOR MEAN	PRIOR VARIANCE	PRIOR STD. DEV.
Parameter 1~N(0.000,infinity)	0.0000	infinity	infinity
Parameter 2~N(0.000,infinity)	0.0000	infinity	infinity
Parameter 3~N(0.000,infinity)	0.0000	infinity	infinity
Parameter 4~N(0.000,infinity)	0.0000	infinity	infinity
Parameter 5~N(0.000,infinity)	0.0000	infinity	infinity
Parameter 6~N(0.000,5.000)	0.0000	5.0000	2.2361
Parameter 7~N(0.000,5.000)	0.0000	5.0000	2.2361
Parameter 8~N(0.000,5.000)	0.0000	5.0000	2.2361
Parameter 9~N(0.000,5.000)	0.0000	5.0000	2.2361
Parameter 10~N(0.000,5.000)	0.0000	5.0000	2.2361
Parameter 11~IW(1.000,7)	infinity	infinity	infinity
Parameter 12~IW(0.000,7)	infinity	infinity	infinity
Parameter 13~IW(1.000,7)	infinity	infinity	infinity
Parameter 14~IW(0.000,7)	infinity	infinity	infinity
Parameter 15~IW(0.000,7)	infinity	infinity	infinity
Parameter 16~IW(1.000,7)	infinity	infinity	infinity
Parameter 17~IW(0.000,7)	infinity	infinity	infinity
Parameter 18~IW(0.000,7)	infinity	infinity	infinity
Parameter 19~IW(0.000,7)	infinity	infinity	infinity
Parameter 20~IW(1.000,7)	infinity	infinity	infinity
Parameter 21~IW(0.000,7)	infinity	infinity	infinity
Parameter 22~IW(0.000,7)	infinity	infinity	infinity
Parameter 23~IW(0.000,7)	infinity	infinity	infinity
Parameter 24~IW(0.000,7)	infinity	infinity	infinity
Parameter 25~IW(1.000,7)	infinity	infinity	infinity

Parameter 26~IW(0.000,7)	infinity	infinity	infinity
Parameter 27~IW(0.000,7)	infinity	infinity	infinity
Parameter 28~IW(0.000,7)	infinity	infinity	infinity
Parameter 29~IW(0.000,7)	infinity	infinity	infinity
Parameter 30~IW(0.000,7)	infinity	infinity	infinity
Parameter 31~IW(1.000,7)	infinity	infinity	infinity
Parameter 32~N(0.000,infinity)	0.0000	infinity	infinity
Parameter 33~N(0.000,infinity)	0.0000	infinity	infinity
Parameter 34~N(0.000,infinity)	0.0000	infinity	infinity
Parameter 35~N(0.000,infinity)	0.0000	infinity	infinity
Parameter 36~N(0.000,5.000)	0.0000	5.0000	2.2361
Parameter 37~N(0.000,5.000)	0.0000	5.0000	2.2361
Parameter 38~IG(-1.000,0.000)	infinity	infinity	infinity
Parameter 39~IG(-1.000,0.000)	infinity	infinity	infinity
Parameter 40~IW(1.000,3)	infinity	infinity	infinity
Parameter 41~IW(0.000,3)	infinity	infinity	infinity
Parameter 42~IW(1.000,3)	infinity	infinity	infinity
Parameter 43~N(0.000,5.000)	0.0000	5.0000	2.2361

TECHNICAL 3 OUTPUT

ESTIMATED COVARIANCE MATRIX FOR PARAMETER ESTIMATES

	1	2	3	4	5
1	0.996039D-04				
2	0.101520D-04	0.164370D-04			
3	-0.179381D-04	-0.496511D-05	0.212664D-03		
4	0.416264D-05	0.420249D-05	-0.155521D-04	0.402876D-04	
5	-0.128891D-04	-0.109292D-04	0.300199D-04	-0.134880D-04	0.165608D-03
6	-0.703469D-05	-0.368876D-05	-0.390901D-05	-0.859723D-05	0.520515D-06
7	0.416369D-05	0.187586D-05	0.322498D-04	-0.878127D-05	0.302087D-04
8	-0.654594D-05	-0.342266D-05	0.174322D-04	-0.538077D-05	0.322904D-05
9	-0.209239D-04	-0.370803D-05	-0.500895D-05	0.838047D-05	-0.247168D-05
10	-0.123946D-04	-0.418829D-05	0.257788D-06	-0.179796D-05	0.251023D-04
11	0.404948D-06	-0.255476D-06	0.288001D-05	0.238911D-05	-0.314959D-05
12	0.647046D-06	-0.578960D-08	0.163291D-05	-0.768887D-07	0.602264D-06
13	-0.342679D-06	0.178513D-06	0.126145D-05	-0.195108D-06	0.179699D-06
14	0.223303D-05	0.183552D-05	0.210562D-05	0.264863D-05	0.168531D-06
15	0.867076D-06	0.553446D-06	-0.215247D-05	0.119076D-05	-0.146082D-05
16	0.346791D-05	-0.343006D-05	0.854756D-05	0.206249D-05	0.932364D-05
17	0.135988D-05	0.144676D-06	0.920570D-06	0.135233D-05	0.423817D-06
18	0.328928D-06	0.304651D-06	0.160375D-05	0.966544D-07	-0.586992D-06

19	0.720612D-06	0.180770D-05	-0.290027D-05	-0.109782D-05	-0.176370D-05
20	-0.102606D-05	0.439924D-06	0.768078D-06	0.355269D-06	0.831191D-07
21	-0.257464D-06	0.149776D-06	0.834255D-06	-0.575630D-06	0.698337D-05
22	0.745984D-06	0.268911D-06	-0.163826D-05	0.420586D-06	0.104909D-05
23	0.468235D-05	0.417812D-05	0.237479D-05	-0.211074D-06	-0.111296D-04
24	-0.829143D-06	-0.102800D-06	-0.266979D-05	-0.304555D-06	0.869434D-06
25	-0.108926D-04	0.218963D-05	-0.115786D-04	-0.126477D-05	-0.365504D-05
26	0.203207D-05	-0.503033D-06	-0.619660D-05	-0.110225D-05	0.950108D-06
27	-0.125229D-05	-0.972993D-06	-0.279248D-05	-0.683497D-06	0.270202D-05
28	-0.578124D-05	-0.435832D-06	-0.194993D-06	0.456593D-05	-0.542336D-06
29	-0.962453D-06	-0.631959D-06	-0.237308D-05	-0.123977D-05	0.150911D-05
30	0.205418D-05	0.119857D-05	-0.385638D-05	0.498594D-06	0.139859D-05
31	0.290000D-05	0.225686D-05	0.316684D-05	0.454641D-05	0.482636D-06
32	0.802328D-05	-0.518923D-06	0.191485D-04	-0.259626D-05	0.415067D-05
33	-0.188117D-04	0.171116D-04	-0.157507D-04	0.429263D-04	-0.286153D-04
34	0.902635D-05	0.738787D-05	-0.154283D-04	0.162616D-04	-0.482384D-04
35	-0.379187D-05	-0.326155D-05	0.137671D-04	-0.413482D-05	0.506433D-05
36	0.238918D-05	-0.995301D-06	0.106317D-04	0.429787D-05	0.156032D-06
37	-0.624269D-04	0.120034D-04	-0.505675D-04	0.278101D-04	-0.397332D-04
38	0.127391D-05	-0.393968D-06	0.284618D-05	-0.522433D-06	0.685287D-06
39	0.268032D-06	0.989573D-07	0.428775D-05	-0.229043D-06	-0.170848D-06
40	-0.416804D-04	-0.186746D-04	-0.171166D-04	0.647941D-05	-0.709162D-04
41	0.130578D-04	-0.380589D-05	0.342761D-05	0.260950D-06	0.300452D-06
42	-0.912409D-06	0.352836D-05	0.140712D-04	0.511779D-06	-0.463715D-05
43	-0.151273D-03	0.296808D-04	-0.123946D-03	0.607402D-04	-0.103322D-03

ESTIMATED COVARIANCE MATRIX FOR PARAMETER ESTIMATES

	6	7	8	9	10
6	0.111942D-02				
7	-0.613351D-03	0.761306D-02			
8	0.102176D-03	0.945222D-05	0.667737D-03		
9	-0.149088D-03	-0.276349D-03	0.106550D-03	0.238787D-02	
10	-0.372875D-04	0.211414D-03	-0.832847D-04	0.115480D-03	0.755681D-03
11	0.221172D-05	0.256978D-04	0.117418D-04	0.225474D-04	-0.115458D-04
12	-0.608055D-06	0.104026D-04	0.109876D-05	0.556086D-05	-0.958210D-06
13	0.157497D-06	0.152129D-05	0.277266D-05	0.327242D-05	-0.362123D-06
14	-0.535162D-05	0.200182D-04	0.649301D-05	0.111772D-05	0.136399D-04
15	-0.160626D-05	0.639608D-05	0.104019D-05	-0.443346D-05	0.414433D-05
16	-0.300010D-04	-0.229772D-04	-0.670953D-05	0.173284D-05	-0.186493D-04
17	-0.657308D-05	0.105210D-04	-0.492833D-05	0.968594D-05	-0.188380D-05
18	0.500967D-06	-0.119332D-05	0.107239D-05	0.302673D-05	0.107058D-05
19	0.591633D-05	-0.132710D-04	0.183622D-05	-0.602006D-05	0.269429D-05

20	-0.343971D-05	0.143556D-04	-0.261556D-05	0.686062D-05	-0.449255D-05
21	-0.862331D-05	0.416819D-04	0.708688D-05	0.208031D-04	0.691775D-05
22	0.512030D-06	-0.155639D-05	0.364463D-05	0.610707D-05	-0.220673D-05
23	0.535078D-05	0.181148D-04	0.225845D-05	0.990807D-05	0.236375D-04
24	-0.635560D-05	0.172495D-04	-0.172032D-06	0.710209D-05	-0.202843D-05
25	-0.647373D-05	-0.731919D-05	-0.683455D-05	-0.809089D-05	0.107632D-04
26	0.396033D-05	-0.105635D-04	-0.132830D-04	0.864334D-05	0.596190D-05
27	-0.868383D-06	0.157037D-04	-0.315771D-05	-0.820373D-06	0.933960D-05
28	0.270021D-06	0.502836D-04	-0.801237D-05	-0.284999D-05	0.705566D-05
29	-0.280133D-05	-0.101103D-04	0.166253D-05	0.433215D-05	0.709380D-05
30	-0.403108D-05	0.532755D-05	0.642027D-05	-0.154185D-04	0.124928D-04
31	-0.893375D-05	0.538890D-04	-0.390120D-05	0.307060D-04	-0.776182D-05
32	-0.254811D-04	-0.201693D-03	-0.101348D-05	-0.305236D-03	0.672337D-04
33	-0.170435D-03	-0.272314D-03	-0.122130D-03	-0.365702D-04	-0.113417D-03
34	0.428760D-04	0.617075D-05	0.629114D-04	0.314803D-04	-0.201819D-04
35	-0.450679D-04	0.252649D-04	-0.758729D-05	-0.411594D-04	-0.553280D-05
36	0.614486D-04	0.473421D-04	-0.812987D-05	0.203081D-04	-0.103893D-04
37	-0.127100D-03	-0.610430D-03	0.228057D-03	-0.159524D-03	0.924435D-04
38	-0.424358D-05	0.110621D-04	0.600506D-06	-0.938463D-06	-0.278023D-05
39	-0.242110D-05	0.290461D-04	0.631249D-05	0.181427D-04	-0.582576D-05
40	-0.160315D-04	0.607898D-04	-0.126910D-03	-0.193656D-03	0.210957D-03
41	-0.569117D-06	-0.150378D-03	0.367881D-05	0.466614D-04	0.782292D-04
42	0.531717D-05	0.734581D-04	0.131298D-04	0.112420D-04	0.128000D-05
43	-0.246212D-03	-0.337908D-03	0.474214D-03	-0.377918D-03	0.201547D-03

ESTIMATED COVARIANCE MATRIX FOR PARAMETER ESTIMATES

	11	12	13	14	15
11	0.105287D-03				
12	0.105764D-04	0.104163D-04			
13	0.167080D-05	0.186854D-05	0.327799D-05		
14	-0.200630D-04	-0.617491D-05	-0.160208D-05	0.112508D-03	
15	-0.175549D-05	-0.254610D-05	-0.139141D-05	0.118513D-04	0.203322D-04
16	0.679092D-05	0.214515D-05	-0.117409D-06	-0.322785D-04	-0.117590D-04
17	0.374011D-05	0.213191D-05	0.326184D-06	-0.607082D-05	0.644264D-07
18	0.584534D-06	0.659408D-06	0.964878D-06	-0.962201D-06	-0.105967D-05
19	-0.295623D-05	-0.185093D-05	-0.870782D-06	0.507981D-05	0.534878D-05
20	0.276615D-05	0.360769D-06	0.256483D-06	-0.191409D-06	0.163748D-06
21	0.804657D-05	0.541720D-05	0.735481D-06	-0.922546D-05	-0.504751D-06
22	0.587746D-06	0.752091D-06	0.182627D-05	-0.669604D-06	-0.155341D-05
23	0.467565D-06	-0.151936D-05	-0.974055D-06	0.141699D-04	0.115534D-04
24	0.182526D-06	0.124232D-06	0.282010D-06	-0.199006D-06	-0.241560D-06
25	0.622776D-05	0.143386D-05	0.133917D-05	-0.543756D-05	-0.887613D-06

26	-0.111984D-04	-0.572876D-05	-0.136564D-05	0.200399D-04	0.605512D-06
27	-0.242462D-05	-0.173874D-05	-0.149093D-05	0.307121D-05	0.252673D-05
28	0.197617D-05	0.697609D-06	0.730289D-06	-0.116391D-04	-0.110267D-04
29	-0.105217D-05	-0.917037D-06	-0.551427D-06	-0.151513D-05	0.255807D-07
30	-0.391508D-05	-0.135590D-05	-0.281816D-06	0.286229D-05	0.163557D-05
31	0.486732D-05	0.677490D-07	0.241627D-06	-0.634056D-05	-0.125254D-05
32	0.608109D-05	0.342974D-05	0.101284D-05	-0.132042D-04	0.770316D-06
33	0.732086D-05	-0.251176D-05	-0.591239D-05	-0.739459D-04	0.169687D-04
34	-0.200563D-05	0.147970D-05	-0.491669D-06	0.193729D-04	0.174887D-05
35	-0.323464D-06	-0.192097D-06	0.397212D-06	-0.204070D-04	0.192277D-05
36	0.866118D-05	-0.187645D-05	-0.170750D-06	-0.642414D-05	-0.478133D-05
37	-0.221486D-04	-0.178198D-04	-0.270362D-05	0.446426D-04	0.327447D-04
38	0.876537D-06	-0.265795D-06	-0.442408D-06	-0.282844D-05	0.749430D-06
39	0.106880D-05	0.551482D-06	-0.504078D-06	-0.135073D-05	-0.745301D-06
40	0.605611D-04	-0.115328D-05	0.159518D-05	-0.123609D-05	-0.405389D-05
41	-0.133089D-04	-0.422357D-05	0.215828D-05	0.342312D-04	0.128694D-04
42	0.448934D-05	-0.168721D-05	0.153790D-06	0.393859D-05	0.124949D-05
43	-0.531954D-04	-0.418017D-04	-0.749511D-05	0.114057D-03	0.767444D-04

ESTIMATED COVARIANCE MATRIX FOR PARAMETER ESTIMATES

	16	17	18	19	20
16	0.463661D-03				
17	0.184606D-05	0.218436D-04			
18	-0.241772D-06	0.258746D-05	0.396591D-05		
19	-0.147206D-04	-0.451732D-05	-0.162970D-05	0.446572D-04	
20	0.124682D-05	0.255384D-05	0.181448D-05	-0.365983D-05	0.184375D-04
21	-0.100751D-04	0.874000D-05	0.120688D-05	-0.262666D-05	-0.483975D-06
22	-0.929816D-06	0.498344D-06	0.186325D-05	-0.166747D-05	-0.109900D-06
23	-0.308471D-04	0.294467D-05	-0.226767D-06	0.187552D-04	0.163799D-05
24	-0.863876D-07	0.259119D-05	0.234193D-05	-0.151419D-05	0.826959D-05
25	-0.651425D-05	0.233576D-05	0.139255D-05	-0.134852D-05	0.109939D-05
26	-0.764753D-05	-0.476677D-05	-0.787299D-06	0.323655D-05	-0.180474D-05
27	-0.408043D-05	-0.635562D-06	-0.118529D-05	0.106989D-05	-0.365313D-06
28	0.639434D-04	0.220806D-05	0.682862D-06	-0.113264D-04	0.151379D-05
29	-0.680095D-05	-0.171309D-05	-0.161986D-05	0.603383D-05	-0.505307D-05
30	-0.593577D-05	0.347451D-07	-0.247473D-06	0.247982D-05	-0.987473D-06
31	0.162098D-04	0.441653D-05	0.336171D-06	-0.327075D-05	0.140969D-06
32	0.243442D-04	0.254454D-05	0.184892D-05	-0.317992D-05	-0.548047D-05
33	0.117259D-03	0.341957D-04	-0.104628D-04	0.260485D-04	0.458172D-05
34	0.901397D-05	-0.180547D-06	0.280822D-05	0.262336D-05	0.845766D-06
35	0.781280D-06	0.295804D-06	-0.112139D-05	0.152647D-05	-0.601793D-05
36	0.332806D-05	0.788530D-06	0.217123D-06	-0.118763D-05	-0.227418D-05

37	-0.178107D-04	-0.227242D-05	-0.703787D-05	0.133883D-04	-0.114362D-04
38	0.893108D-06	-0.194674D-06	-0.481880D-06	-0.136549D-07	-0.697987D-06
39	0.629170D-05	0.188111D-05	-0.471849D-06	0.422292D-06	-0.691002D-07
40	-0.321008D-03	0.193385D-05	0.265687D-04	-0.584491D-04	-0.208404D-04
41	0.249060D-04	-0.408700D-05	0.369186D-05	-0.634539D-05	-0.157978D-05
42	-0.225226D-05	-0.914457D-06	0.156734D-08	0.220245D-05	0.345947D-05
43	-0.313951D-04	-0.729345D-05	-0.177829D-04	0.291476D-04	-0.255196D-04

ESTIMATED COVARIANCE MATRIX FOR PARAMETER ESTIMATES

	21	22	23	24	25
21	0.104062D-03				
22	0.102724D-04	0.169863D-04			
23	-0.147965D-04	-0.109265D-04	0.213119D-03		
24	0.302889D-05	0.444072D-05	-0.924725D-05	0.397744D-04	
25	0.205349D-04	0.188744D-04	-0.196128D-04	0.301029D-04	0.364061D-03
26	-0.170796D-04	-0.195461D-05	0.351688D-05	-0.772294D-06	-0.366447D-05
27	-0.221578D-05	-0.277935D-05	0.350066D-05	-0.531955D-06	-0.169264D-05
28	-0.176772D-05	0.220807D-05	-0.327281D-04	0.452102D-05	-0.382597D-05
29	-0.325194D-05	-0.975970D-06	0.105492D-05	-0.586979D-05	-0.449173D-05
30	-0.121427D-04	-0.117164D-04	0.312818D-04	-0.123307D-04	-0.546580D-04
31	0.351407D-05	0.297629D-05	-0.136424D-04	0.523492D-05	0.128341D-04
32	0.364705D-05	0.218689D-05	-0.485675D-05	-0.455324D-05	0.216614D-04
33	-0.623590D-05	-0.108437D-04	-0.421180D-05	-0.261010D-05	0.160399D-03
34	-0.682597D-06	0.245437D-05	-0.366258D-04	-0.176216D-05	0.404648D-04
35	0.339608D-05	0.132745D-06	0.411955D-05	-0.377239D-05	0.152780D-04
36	0.529808D-05	0.412037D-05	-0.180247D-04	0.295417D-05	0.998954D-05
37	-0.344407D-05	-0.115718D-04	0.138550D-03	0.353373D-05	-0.397283D-04
38	0.120881D-05	0.475217D-06	-0.508111D-05	-0.306098D-06	0.357709D-06
39	0.221723D-05	0.352738D-06	0.164536D-05	0.267557D-06	-0.206961D-05
40	0.153378D-03	0.334914D-04	-0.143313D-03	-0.330519D-04	-0.175207D-04
41	0.108622D-04	0.122353D-04	0.150761D-04	0.102719D-04	0.500695D-04
42	-0.608806D-05	0.215910D-05	0.551129D-05	-0.809199D-06	0.474157D-05
43	-0.777860D-05	-0.291767D-04	0.331195D-03	0.445905D-05	-0.986794D-04

ESTIMATED COVARIANCE MATRIX FOR PARAMETER ESTIMATES

	26	27	28	29	30
26	0.858011D-04				
27	0.957105D-05	0.148105D-04			
28	-0.163937D-04	-0.627141D-05	0.184148D-03		
29	0.261863D-05	0.344357D-05	-0.816712D-05	0.338756D-04	

30	0.862203D-05	0.970142D-05	-0.109403D-04	0.138878D-04	0.156307D-03
31	-0.227481D-04	-0.159112D-04	0.493206D-04	-0.171954D-04	-0.520593D-04
32	-0.293667D-05	0.445487D-05	-0.143437D-04	0.309971D-05	0.106037D-04
33	-0.551339D-04	-0.334143D-06	0.127578D-04	-0.299903D-04	-0.166023D-04
34	0.820759D-06	-0.486090D-05	0.892221D-05	-0.324178D-05	-0.155987D-04
35	0.355405D-05	-0.209240D-05	-0.122874D-04	0.525479D-05	-0.627467D-05
36	0.167714D-05	0.242967D-05	0.378681D-05	-0.531212D-05	0.670720D-05
37	0.562788D-04	0.219333D-04	0.335679D-04	0.102200D-04	0.520594D-04
38	-0.219723D-05	-0.267144D-06	0.771126D-06	0.117174D-05	-0.208781D-05
39	-0.560283D-06	0.196717D-06	0.642201D-06	-0.526166D-06	-0.153613D-05
40	0.620079D-05	0.685602D-05	-0.610442D-04	-0.106782D-04	0.908556D-05
41	0.165217D-05	-0.101483D-04	-0.562718D-05	0.300126D-06	-0.401347D-05
42	-0.120328D-06	-0.210461D-05	0.567472D-05	-0.226411D-05	-0.371699D-05
43	0.121292D-03	0.550087D-04	0.791903D-04	0.229658D-04	0.112719D-03

ESTIMATED COVARIANCE MATRIX FOR PARAMETER ESTIMATES

	31	32	33	34	35
31	0.250773D-03				
32	-0.184435D-04	0.947397D-03			
33	0.199850D-04	0.739123D-04	0.230451D-01		
34	0.125938D-04	0.176187D-04	0.667564D-04	0.163015D-02	
35	-0.933892D-05	0.355321D-04	-0.205292D-03	-0.343935D-04	0.820187D-03
36	-0.247689D-05	0.424640D-05	-0.470400D-04	-0.436683D-04	-0.681611D-04
37	-0.868665D-04	-0.211448D-03	-0.451961D-03	-0.219062D-03	0.816956D-04
38	0.489849D-06	0.508204D-05	-0.290826D-04	-0.188194D-05	0.281270D-04
39	-0.193549D-05	-0.114680D-04	0.150741D-04	0.380621D-06	0.435679D-06
40	0.666654D-04	-0.971309D-04	-0.845805D-03	0.193172D-04	0.241466D-04
41	-0.898505D-05	0.525728D-04	0.223915D-03	0.413807D-04	0.993809D-06
42	0.385830D-05	-0.835774D-05	0.529839D-04	-0.282682D-04	-0.824580D-05
43	-0.185491D-03	-0.501392D-03	-0.116741D-02	-0.604284D-03	0.238957D-03

ESTIMATED COVARIANCE MATRIX FOR PARAMETER ESTIMATES

	36	37	38	39	40
36	0.756314D-03				
37	-0.214020D-03	0.211348D-01			
38	0.373330D-05	0.258350D-04	0.314203D-04		
39	0.189496D-04	0.371465D-04	0.312047D-06	0.492813D-04	
40	0.665646D-04	0.133260D-03	-0.270604D-05	0.153785D-04	0.724560D-01
41	0.448788D-04	-0.854739D-04	0.587676D-05	-0.973346D-05	-0.201727D-03
42	-0.161610D-04	0.180185D-03	0.463658D-06	-0.276992D-05	0.702035D-04

43	-0.522848D-03	0.508572D-01	0.671971D-04	0.124869D-03	0.250661D-03

ESTIMATED COVARIANCE MATRIX FOR PARAMETER ESTIMATES

	41	42	43
41	0.246886D-02		
42	-0.478416D-04	0.296937D-03	
43	-0.197652D-03	0.418147D-03	0.123480D+00

ESTIMATED CORRELATION MATRIX FOR PARAMETER ESTIMATES

	1	2	3	4	5
1	1.000				
2	0.251	1.000			
3	-0.123	-0.084	1.000		
4	0.066	0.163	-0.168	1.000	
5	-0.100	-0.209	0.160	-0.165	1.000
6	-0.021	-0.027	-0.008	-0.040	0.001
7	0.005	0.005	0.025	-0.016	0.027
8	-0.025	-0.033	0.046	-0.033	0.010
9	-0.043	-0.019	-0.007	0.027	-0.004
10	-0.045	-0.038	0.001	-0.010	0.071
11	0.004	-0.006	0.019	0.037	-0.024
12	0.020	0.000	0.035	-0.004	0.015
13	-0.019	0.024	0.048	-0.017	0.008
14	0.021	0.043	0.014	0.039	0.001
15	0.019	0.030	-0.033	0.042	-0.025
16	0.016	-0.039	0.027	0.015	0.034
17	0.029	0.008	0.014	0.046	0.007
18	0.017	0.038	0.055	0.008	-0.023
19	0.011	0.067	-0.030	-0.026	-0.021
20	-0.024	0.025	0.012	0.013	0.002
21	-0.003	0.004	0.006	-0.009	0.053
22	0.018	0.016	-0.027	0.016	0.020
23	0.032	0.071	0.011	-0.002	-0.059
24	-0.013	-0.004	-0.029	-0.008	0.011
25	-0.057	0.028	-0.042	-0.010	-0.015
26	0.022	-0.013	-0.046	-0.019	0.008
27	-0.033	-0.062	-0.050	-0.028	0.055
28	-0.043	-0.008	-0.001	0.053	-0.003
29	-0.017	-0.027	-0.028	-0.034	0.020
30	0.016	0.024	-0.021	0.006	0.009

31	0.018	0.035	0.014	0.045	0.002
32	0.026	-0.004	0.043	-0.013	0.010
33	-0.012	0.028	-0.007	0.045	-0.015
34	0.022	0.045	-0.026	0.063	-0.093
35	-0.013	-0.028	0.033	-0.023	0.014
36	0.009	-0.009	0.027	0.025	0.000
37	-0.043	0.020	-0.024	0.030	-0.021
38	0.023	-0.017	0.035	-0.015	0.010
39	0.004	0.003	0.042	-0.005	-0.002
40	-0.016	-0.017	-0.004	0.004	-0.020
41	0.026	-0.019	0.005	0.001	0.000
42	-0.005	0.051	0.056	0.005	-0.021
43	-0.043	0.021	-0.024	0.027	-0.023

ESTIMATED CORRELATION MATRIX FOR PARAMETER ESTIMATES

	6	7	8	9	10
6	1.000				
7	-0.210	1.000			
8	0.118	0.004	1.000		
9	-0.091	-0.065	0.084	1.000	
10	-0.041	0.088	-0.117	0.086	1.000
11	0.006	0.029	0.044	0.045	-0.041
12	-0.006	0.037	0.013	0.035	-0.011
13	0.003	0.010	0.059	0.037	-0.007
14	-0.015	0.022	0.024	0.002	0.047
15	-0.011	0.016	0.009	-0.020	0.033
16	-0.042	-0.012	-0.012	0.002	-0.032
17	-0.042	0.026	-0.041	0.042	-0.015
18	0.008	-0.007	0.021	0.031	0.020
19	0.026	-0.023	0.011	-0.018	0.015
20	-0.024	0.038	-0.024	0.033	-0.038
21	-0.025	0.047	0.027	0.042	0.025
22	0.004	-0.004	0.034	0.030	-0.019
23	0.011	0.014	0.006	0.014	0.059
24	-0.030	0.031	-0.001	0.023	-0.012
25	-0.010	-0.004	-0.014	-0.009	0.021
26	0.013	-0.013	-0.055	0.019	0.023
27	-0.007	0.047	-0.032	-0.004	0.088
28	0.001	0.042	-0.023	-0.004	0.019
29	-0.014	-0.020	0.011	0.015	0.044
30	-0.010	0.005	0.020	-0.025	0.036
31	-0.017	0.039	-0.010	0.040	-0.018

32	-0.025	-0.075	-0.001	-0.203	0.079
33	-0.034	-0.021	-0.031	-0.005	-0.027
34	0.032	0.002	0.060	0.016	-0.018
35	-0.047	0.010	-0.010	-0.029	-0.007
36	0.067	0.020	-0.011	0.015	-0.014
37	-0.026	-0.048	0.061	-0.022	0.023
38	-0.023	0.023	0.004	-0.003	-0.018
39	-0.010	0.047	0.035	0.053	-0.030
40	-0.002	0.003	-0.018	-0.015	0.029
41	0.000	-0.035	0.003	0.019	0.057
42	0.009	0.049	0.029	0.013	0.003
43	-0.021	-0.011	0.052	-0.022	0.021

ESTIMATED CORRELATION MATRIX FOR PARAMETER ESTIMATES

	11	12	13	14	15
11	1.000				
12	0.319	1.000			
13	0.090	0.320	1.000		
14	-0.184	-0.180	-0.083	1.000	
15	-0.038	-0.175	-0.170	0.248	1.000
16	0.031	0.031	-0.003	-0.141	-0.121
17	0.078	0.141	0.039	-0.122	0.003
18	0.029	0.103	0.268	-0.046	-0.118
19	-0.043	-0.086	-0.072	0.072	0.178
20	0.063	0.026	0.033	-0.004	0.008
21	0.077	0.165	0.040	-0.085	-0.011
22	0.014	0.057	0.245	-0.015	-0.084
23	0.003	-0.032	-0.037	0.092	0.176
24	0.003	0.006	0.025	-0.003	-0.008
25	0.032	0.023	0.039	-0.027	-0.010
26	-0.118	-0.192	-0.081	0.204	0.014
27	-0.061	-0.140	-0.214	0.075	0.146
28	0.014	0.016	0.030	-0.081	-0.180
29	-0.018	-0.049	-0.052	-0.025	0.001
30	-0.031	-0.034	-0.012	0.022	0.029
31	0.030	0.001	0.008	-0.038	-0.018
32	0.019	0.035	0.018	-0.040	0.006
33	0.005	-0.005	-0.022	-0.046	0.025
34	-0.005	0.011	-0.007	0.045	0.010
35	-0.001	-0.002	0.008	-0.067	0.015
36	0.031	-0.021	-0.003	-0.022	-0.039
37	-0.015	-0.038	-0.010	0.029	0.050

38	0.015	-0.015	-0.044	-0.048	0.030
39	0.015	0.024	-0.040	-0.018	-0.024
40	0.022	-0.001	0.003	0.000	-0.003
41	-0.026	-0.026	0.024	0.065	0.057
42	0.025	-0.030	0.005	0.022	0.016
43	-0.015	-0.037	-0.012	0.031	0.048

ESTIMATED CORRELATION MATRIX FOR PARAMETER ESTIMATES

	16	17	18	19	20
16	1.000				
17	0.018	1.000			
18	-0.006	0.278	1.000		
19	-0.102	-0.145	-0.122	1.000	
20	0.013	0.127	0.212	-0.128	1.000
21	-0.046	0.183	0.059	-0.039	-0.011
22	-0.010	0.026	0.227	-0.061	-0.006
23	-0.098	0.043	-0.008	0.192	0.026
24	-0.001	0.088	0.186	-0.036	0.305
25	-0.016	0.026	0.037	-0.011	0.013
26	-0.038	-0.110	-0.043	0.052	-0.045
27	-0.049	-0.035	-0.155	0.042	-0.022
28	0.219	0.035	0.025	-0.125	0.026
29	-0.054	-0.063	-0.140	0.155	-0.202
30	-0.022	0.001	-0.010	0.030	-0.018
31	0.048	0.060	0.011	-0.031	0.002
32	0.037	0.018	0.030	-0.015	-0.041
33	0.036	0.048	-0.035	0.026	0.007
34	0.010	-0.001	0.035	0.010	0.005
35	0.001	0.002	-0.020	0.008	-0.049
36	0.006	0.006	0.004	-0.006	-0.019
37	-0.006	-0.003	-0.024	0.014	-0.018
38	0.007	-0.007	-0.043	0.000	-0.029
39	0.042	0.057	-0.034	0.009	-0.002
40	-0.055	0.002	0.050	-0.032	-0.018
41	0.023	-0.018	0.037	-0.019	-0.007
42	-0.006	-0.011	0.000	0.019	0.047
43	-0.004	-0.004	-0.025	0.012	-0.017

ESTIMATED CORRELATION MATRIX FOR PARAMETER ESTIMATES

	21	22	23	24	25

21	1.000				
22	0.244	1.000			
23	-0.099	-0.182	1.000		
24	0.047	0.171	-0.100	1.000	
25	0.106	0.240	-0.070	0.250	1.000
26	-0.181	-0.051	0.026	-0.013	-0.021
27	-0.056	-0.175	0.062	-0.022	-0.023
28	-0.013	0.039	-0.165	0.053	-0.015
29	-0.055	-0.041	0.012	-0.160	-0.040
30	-0.095	-0.227	0.171	-0.156	-0.229
31	0.022	0.046	-0.059	0.052	0.042
32	0.012	0.017	-0.011	-0.023	0.037
33	-0.004	-0.017	-0.002	-0.003	0.055
34	-0.002	0.015	-0.062	-0.007	0.053
35	0.012	0.001	0.010	-0.021	0.028
36	0.019	0.036	-0.045	0.017	0.019
37	-0.002	-0.019	0.065	0.004	-0.014
38	0.021	0.021	-0.062	-0.009	0.003
39	0.031	0.012	0.016	0.006	-0.015
40	0.056	0.030	-0.036	-0.019	-0.003
41	0.021	0.060	0.021	0.033	0.053
42	-0.035	0.030	0.022	-0.007	0.014
43	-0.002	-0.020	0.065	0.002	-0.015

ESTIMATED CORRELATION MATRIX FOR PARAMETER ESTIMATES

	26	27	28	29	30
26	1.000				
27	0.268	1.000			
28	-0.130	-0.120	1.000		
29	0.049	0.154	-0.103	1.000	
30	0.074	0.202	-0.064	0.191	1.000
31	-0.155	-0.261	0.230	-0.187	-0.263
32	-0.010	0.038	-0.034	0.017	0.028
33	-0.039	-0.001	0.006	-0.034	-0.009
34	0.002	-0.031	0.016	-0.014	-0.031
35	0.013	-0.019	-0.032	0.032	-0.018
36	0.007	0.023	0.010	-0.033	0.020
37	0.042	0.039	0.017	0.012	0.029
38	-0.042	-0.012	0.010	0.036	-0.030
39	-0.009	0.007	0.007	-0.013	-0.018
40	0.002	0.007	-0.017	-0.007	0.003
41	0.004	-0.053	-0.008	0.001	-0.006

42	-0.001	-0.032	0.024	-0.023	-0.017
43	0.037	0.041	0.017	0.011	0.026

ESTIMATED CORRELATION MATRIX FOR PARAMETER ESTIMATES

	31	32	33	34	35
31	1.000				
32	-0.038	1.000			
33	0.008	0.016	1.000		
34	0.020	0.014	0.011	1.000	
35	-0.021	0.040	-0.047	-0.030	1.000
36	-0.006	0.005	-0.011	-0.039	-0.087
37	-0.038	-0.047	-0.020	-0.037	0.020
38	0.006	0.029	-0.034	-0.008	0.175
39	-0.017	-0.053	0.014	0.001	0.002
40	0.016	-0.012	-0.021	0.002	0.003
41	-0.011	0.034	0.030	0.021	0.001
42	0.014	-0.016	0.020	-0.041	-0.017
43	-0.033	-0.046	-0.022	-0.043	0.024

ESTIMATED CORRELATION MATRIX FOR PARAMETER ESTIMATES

	36	37	38	39	40
36	1.000				
37	-0.054	1.000			
38	0.024	0.032	1.000		
39	0.098	0.036	0.008	1.000	
40	0.009	0.003	-0.002	0.008	1.000
41	0.033	-0.012	0.021	-0.028	-0.015
42	-0.034	0.072	0.005	-0.023	0.015
43	-0.054	0.996	0.034	0.051	0.003

ESTIMATED CORRELATION MATRIX FOR PARAMETER ESTIMATES

	41	42	43
41	1.000		
42	-0.056	1.000	
43	-0.011	0.069	1.000

TECHNICAL 8 OUTPUT

TECHNICAL 8 OUTPUT FOR BAYES ESTIMATION

CHAIN	BSEED
1	0
2	285380
3	253358

ITERATION	POTENTIAL SCALE REDUCTION	PARAMETER WITH HIGHEST PSR
100	1.010	7
200	1.020	35
300	1.013	32
400	1.004	35
500	1.002	8
600	1.004	37
700	1.002	37
800	1.001	8
900	1.001	26
1000	1.001	32

Worsening of psychological distress, role clarity interaction

SUMMARY OF DATA

Number of clusters 58

Size (s)	Cluster ID with Size s
7	22
17	166
20	80
26	71 74 205
29	65
31	192
33	228
37	182 106
39	72
41	170 181
42	50 30
43	75
45	173
47	226 165
48	108
50	107 114
51	81
52	40
55	84 92
57	162
59	206
61	61 83 116
62	36 93 169
63	69
64	4
69	68
73	174 152
74	6 154
75	145
89	98
90	178 2
95	144
100	156
104	202
106	142
134	122

186	24
192	209
246	204
278	125
405	217
780	110
873	100

COVARIANCE COVERAGE OF DATA

Minimum covariance coverage value 0.100

Number of missing data patterns 9

PROPORTION OF DATA PRESENT

Covariance Coverage

	KESCAT16	PRAFAC	PHY15	KES15	CHOICE15
KESCAT16	1.000				
PRAFAC	1.000	1.000			
PHY15	0.950	0.950	0.950		
KES15	0.919	0.919	0.919	0.919	
CHOICE15	0.913	0.913	0.913	0.913	0.913
BULLY15	0.913	0.913	0.913	0.913	0.912
PRESS15	0.913	0.913	0.913	0.913	0.913
DUTIES15	0.913	0.913	0.913	0.913	0.913

Covariance Coverage

	BULLY15	PRESS15	DUTIES15
BULLY15	0.913		
PRESS15	0.912	0.913	
DUTIES15	0.913	0.913	0.913

UNIVARIATE PROPORTIONS AND COUNTS FOR CATEGORICAL VARIABLES

KESCAT16

Category 1	0.877	5135.000
Category 2	0.123	720.000

UNIVARIATE SAMPLE STATISTICS

UNIVARIATE HIGHER-ORDER MOMENT DESCRIPTIVE STATISTICS

Variable/ Sample Size	Mean/ Variance	Skewness/ Kurtosis	Minimum/ Maximum	% with Min/Max	Percentiles 20%/60%	Percentiles 40%/80%	Median
PRAFAC	-0.185	0.245	-2.338	1.72%	-1.448	-0.527	-0.318
58.000	1.345	-0.636	2.338	1.72%	0.080	0.752	
PHY15	0.000	0.526	-0.865	32.48%	-0.865	0.135	0.135
5563.000	0.536	0.021	3.135	0.07%	0.135	0.135	
KES15	0.000	0.475	-0.374	22.78%	-0.374	-0.207	-0.041
5382.000	0.095	-0.842	0.626	6.60%	-0.041	0.293	
CHOICE15	0.000	-0.400	-2.221	7.67%	-1.221	-0.221	-0.221
5345.000	1.097	-0.356	1.779	8.62%	0.779	0.779	
BULLY15	0.000	3.522	-0.153	88.29%	-0.153	-0.153	-0.153
5344.000	0.216	14.164	3.847	0.09%	-0.153	-0.153	
PRESS15	0.000	0.267	-1.445	19.70%	-0.445	-0.445	-0.445
5345.000	1.001	-0.357	2.555	2.75%	0.555	0.555	
DUTIES15	4.149	-1.296	1.000	2.34%	4.000	4.000	4.000
5347.000	0.828	1.939	5.000	39.74%	4.000	5.000	

THE MODEL ESTIMATION TERMINATED NORMALLY

USE THE FBITERATIONS OPTION TO INCREASE THE NUMBER OF ITERATIONS BY A FACTOR
OF AT LEAST TWO TO CHECK CONVERGENCE AND THAT THE PSR VALUE DOES NOT INCREASE.

MODEL FIT INFORMATION

Number of Free Parameters 43

MODEL RESULTS

	Estimate	Posterior S.D.	One-Tailed P-Value	95% C.I. Lower 2.5%	95% C.I. Upper 2.5%	Significance
**Within Level**						
KESCAT16 ON						
PHY15	0.136	0.034	0.000	0.069	0.200	*
CHOICE15	-0.057	0.026	0.014	-0.106	-0.008	*
PRESS15	-0.028	0.026	0.136	-0.080	0.020	
BULLY15	0.111	0.049	0.011	0.019	0.210	*
KES15	1.625	0.087	0.000	1.456	1.801	*
KES15 WITH						
PHY15	0.054	0.003	0.000	0.048	0.061	*
CHOICE15	-0.037	0.005	0.000	-0.046	-0.028	*
BULLY15	0.022	0.002	0.000	0.018	0.026	*
PRESS15	0.053	0.004	0.000	0.045	0.061	*
DUTIES15	-0.049	0.004	0.000	-0.057	-0.042	*
PHY15 WITH						
CHOICE15	-0.092	0.011	0.000	-0.112	-0.070	*
BULLY15	0.025	0.005	0.000	0.015	0.034	*
PRESS15	0.063	0.010	0.000	0.042	0.083	*
DUTIES15	-0.054	0.009	0.000	-0.072	-0.036	*
CHOICE15 WITH						
BULLY15	-0.052	0.007	0.000	-0.065	-0.039	*
PRESS15	-0.077	0.015	0.000	-0.105	-0.047	*
DUTIES15	0.157	0.014	0.000	0.132	0.186	*
BULLY15 WITH						
PRESS15	0.091	0.006	0.000	0.078	0.103	*
DUTIES15	-0.057	0.006	0.000	-0.068	-0.046	*
PRESS15 WITH						
DUTIES15	-0.153	0.013	0.000	-0.179	-0.128	*
Means						
PHY15	0.001	0.010	0.437	-0.019	0.021	
KES15	0.003	0.004	0.216	-0.005	0.011	
CHOICE15	-0.003	0.015	0.418	-0.032	0.025	
BULLY15	0.001	0.006	0.429	-0.011	0.014	
PRESS15	0.002	0.014	0.434	-0.026	0.029	

```
 Variances
    PHY15              0.537      0.010      0.000      0.516      0.557      *
    KES15              0.096      0.002      0.000      0.092      0.100      *
    CHOICE15           1.097      0.022      0.000      1.060      1.141      *
    BULLY15            0.216      0.004      0.000      0.208      0.225      *
    PRESS15            1.002      0.020      0.000      0.966      1.042      *
    DUTIES15           0.816      0.016      0.000      0.786      0.848      *

Between Level

 S1         ON
    PRAFAC            -0.005      0.035      0.440     -0.071      0.065

 KESCAT16   ON
    PRAFAC             0.016      0.030      0.287     -0.040      0.076
    DUTIES15          -0.226      0.263      0.197     -0.746      0.300

 PRAFAC   WITH
    DUTIES15           0.038      0.039      0.149     -0.036      0.119

 Means
    PRAFAC            -0.182      0.152      0.121     -0.489      0.109
    DUTIES15           4.172      0.031      0.000      4.113      4.235      *

 Intercepts
    S1                -0.085      0.037      0.017     -0.158     -0.010      *

 Thresholds
    KESCAT16$1         0.419      1.093      0.353     -1.724      2.596

 Variances
    PRAFAC             1.358      0.269      0.000      0.964      2.004      *
    DUTIES15           0.039      0.009      0.000      0.025      0.059      *

 Residual Variances
    KESCAT16           0.012      0.008      0.000      0.002      0.033      *
    S1                 0.014      0.012      0.000      0.001      0.046      *


STANDARDIZED MODEL RESULTS


STDYX Standardization
```

	Estimate	Posterior S.D.	One-Tailed P-Value	95% C.I. Lower 2.5%	Upper 2.5%	Significance
**Within-Level Standardized Estimates Averaged Over Clusters**						
S1 | KESCAT16 ON						
DUTIES15	-0.064	0.025	0.004	-0.111	-0.016	*
KESCAT16 ON						
PHY15	0.086	0.021	0.000	0.044	0.127	*
CHOICE15	-0.052	0.023	0.010	-0.098	-0.008	*
PRESS15	-0.025	0.022	0.135	-0.069	0.019	
BULLY15	0.045	0.019	0.013	0.007	0.083	*
KES15	0.435	0.020	0.000	0.395	0.473	*
KES15 WITH						
PHY15	0.240	0.013	0.000	0.214	0.264	*
CHOICE15	-0.113	0.014	0.000	-0.139	-0.085	*
BULLY15	0.154	0.013	0.000	0.128	0.180	*
PRESS15	0.171	0.013	0.000	0.145	0.198	*
DUTIES15	-0.176	0.013	0.000	-0.202	-0.150	*
PHY15 WITH						
CHOICE15	-0.120	0.014	0.000	-0.146	-0.093	*
BULLY15	0.072	0.014	0.000	0.045	0.098	*
PRESS15	0.086	0.014	0.000	0.059	0.112	*
DUTIES15	-0.081	0.014	0.000	-0.108	-0.054	*
CHOICE15 WITH						
BULLY15	-0.106	0.013	0.000	-0.132	-0.079	*
PRESS15	-0.074	0.014	0.000	-0.100	-0.046	*
DUTIES15	0.167	0.014	0.000	0.140	0.194	*
BULLY15 WITH						
PRESS15	0.195	0.013	0.000	0.168	0.220	*
DUTIES15	-0.135	0.014	0.000	-0.161	-0.108	*
PRESS15 WITH						
DUTIES15	-0.169	0.014	0.000	-0.196	-0.142	*
Means						
PHY15	0.002	0.014	0.428	-0.025	0.029	
KES15	0.010	0.013	0.229	-0.016	0.037	
CHOICE15	-0.003	0.014	0.413	-0.030	0.023	

```
    BULLY15            0.003       0.014       0.417      -0.024       0.029
    PRESS15            0.002       0.014       0.434      -0.026       0.030

 Variances
    PHY15              1.000       0.000       0.000       1.000       1.000
    KES15              1.000       0.000       0.000       1.000       1.000
    CHOICE15           1.000       0.000       0.000       1.000       1.000
    BULLY15            1.000       0.000       0.000       1.000       1.000
    PRESS15            1.000       0.000       0.000       1.000       1.000
    DUTIES15           1.000       0.000       0.000       1.000       1.000

Between Level

 S1         ON
    PRAFAC            -0.044       0.339       0.440      -0.765       0.564

 KESCAT16   ON
    PRAFAC             0.143       0.265       0.287      -0.404       0.617
    DUTIES15          -0.368       0.358       0.197      -0.828       0.541

 PRAFAC   WITH
    DUTIES15           0.170       0.153       0.149      -0.158       0.443

 Means
    PRAFAC            -0.158       0.129       0.121      -0.416       0.092
    DUTIES15          21.224       2.339       0.000      17.101      26.308      *

 Intercepts
    S1                -0.690       0.477       0.017      -1.860      -0.069      *

 Variances
    PRAFAC             1.000       0.000       0.000       1.000       1.000
    DUTIES15           1.000       0.000       0.000       1.000       1.000

 Residual Variances
    KESCAT16           0.753       0.207       0.000       0.252       0.989      *
    S1                 0.945       0.164       0.000       0.387       1.000      *


R-SQUARE

Within-Level R-Square Averaged Across Clusters

                                Posterior  One-Tailed         95% C.I.
```

Variable	Estimate	S.D.	P-Value	Lower 2.5%	Upper 2.5%
KESCAT16	0.255	0.018	0.000	0.219	0.291

Between Level

		Posterior	One-Tailed	95% C.I.	
Variable	Estimate	S.D.	P-Value	Lower 2.5%	Upper 2.5%
KESCAT16	0.247	0.207	0.000	0.011	0.748

		Posterior	One-Tailed	95% C.I.	
Variable	Estimate	S.D.	P-Value	Lower 2.5%	Upper 2.5%
S1	0.055	0.164	0.000	0.000	0.613

TECHNICAL 1 OUTPUT

PARAMETER SPECIFICATION FOR WITHIN

TAU

KESCAT16
0

NU

KESCAT16	PHY15	KES15	CHOICE15	BULLY15
0	0	0	0	0

NU

PRESS15	DUTIES15
0	0

LAMBDA

KESCAT16	PHY15	KES15	CHOICE15	BULLY15

KESCAT16	0	0	0	0	0
PHY15	0	0	0	0	0
KES15	0	0	0	0	0
CHOICE15	0	0	0	0	0
BULLY15	0	0	0	0	0
PRESS15	0	0	0	0	0
DUTIES15	0	0	0	0	0

LAMBDA

	PRESS15	DUTIES15
KESCAT16	0	0
PHY15	0	0
KES15	0	0
CHOICE15	0	0
BULLY15	0	0
PRESS15	0	0
DUTIES15	0	0

THETA

	KESCAT16	PHY15	KES15	CHOICE15	BULLY15
KESCAT16	0				
PHY15	0	0			
KES15	0	0	0		
CHOICE15	0	0	0	0	
BULLY15	0	0	0	0	0
PRESS15	0	0	0	0	0
DUTIES15	0	0	0	0	0

THETA

	PRESS15	DUTIES15
PRESS15	0	
DUTIES15	0	0

ALPHA

KESCAT16	PHY15	KES15	CHOICE15	BULLY15
0	1	2	3	4

ALPHA

PRESS15	DUTIES15
5	0

BETA

	KESCAT16	PHY15	KES15	CHOICE15	BULLY15
KESCAT16	0	6	7	8	9
PHY15	0	0	0	0	0
KES15	0	0	0	0	0
CHOICE15	0	0	0	0	0
BULLY15	0	0	0	0	0
PRESS15	0	0	0	0	0
DUTIES15	0	0	0	0	0

BETA

	PRESS15	DUTIES15
KESCAT16	10	0
PHY15	0	0
KES15	0	0
CHOICE15	0	0
BULLY15	0	0
PRESS15	0	0
DUTIES15	0	0

PSI

	KESCAT16	PHY15	KES15	CHOICE15	BULLY15
KESCAT16	0				
PHY15	0	11			
KES15	0	12	13		
CHOICE15	0	14	15	16	
BULLY15	0	17	18	19	20
PRESS15	0	21	22	23	24
DUTIES15	0	26	27	28	29

PSI

	PRESS15	DUTIES15
PRESS15	25	
DUTIES15	30	31

PARAMETER SPECIFICATION FOR BETWEEN

TAU

KESCAT16
43

NU

KESCAT16	PRAFAC	DUTIES15
0	0	0

LAMBDA

	S1	KESCAT16	PRAFAC	DUTIES15
KESCAT16	0	0	0	0
PRAFAC	0	0	0	0
DUTIES15	0	0	0	0

THETA

	KESCAT16	PRAFAC	DUTIES15
KESCAT16	0		
PRAFAC	0	0	
DUTIES15	0	0	0

ALPHA

S1	KESCAT16	PRAFAC	DUTIES15
32	0	33	34

BETA

	S1	KESCAT16	PRAFAC	DUTIES15
S1	0	0	35	0
KESCAT16	0	0	36	37
PRAFAC	0	0	0	0
DUTIES15	0	0	0	0

PSI

	S1	KESCAT16	PRAFAC	DUTIES15
S1	38			
KESCAT16	0	39		
PRAFAC	0	0	40	
DUTIES15	0	0	41	42

STARTING VALUES FOR WITHIN

TAU

KESCAT16
0.000

NU

KESCAT16	PHY15	KES15	CHOICE15	BULLY15
0.000	0.000	0.000	0.000	0.000

NU

PRESS15	DUTIES15
0.000	0.000

LAMBDA

	KESCAT16	PHY15	KES15	CHOICE15	BULLY15
KESCAT16	1.000	0.000	0.000	0.000	0.000
PHY15	0.000	1.000	0.000	0.000	0.000

KES15	0.000	0.000	1.000	0.000	0.000
CHOICE15	0.000	0.000	0.000	1.000	0.000
BULLY15	0.000	0.000	0.000	0.000	1.000
PRESS15	0.000	0.000	0.000	0.000	0.000
DUTIES15	0.000	0.000	0.000	0.000	0.000

LAMBDA

	PRESS15	DUTIES15
KESCAT16	0.000	0.000
PHY15	0.000	0.000
KES15	0.000	0.000
CHOICE15	0.000	0.000
BULLY15	0.000	0.000
PRESS15	1.000	0.000
DUTIES15	0.000	1.000

THETA

	KESCAT16	PHY15	KES15	CHOICE15	BULLY15
KESCAT16	0.000				
PHY15	0.000	0.000			
KES15	0.000	0.000	0.000		
CHOICE15	0.000	0.000	0.000	0.000	
BULLY15	0.000	0.000	0.000	0.000	0.000
PRESS15	0.000	0.000	0.000	0.000	0.000
DUTIES15	0.000	0.000	0.000	0.000	0.000

THETA

	PRESS15	DUTIES15
PRESS15	0.000	
DUTIES15	0.000	0.000

ALPHA

KESCAT16	PHY15	KES15	CHOICE15	BULLY15
0.000	0.000	0.000	0.000	0.000

ALPHA

PRESS15	DUTIES15
0.000	0.000

BETA

	KESCAT16	PHY15	KES15	CHOICE15	BULLY15
KESCAT16	0.000	0.000	0.000	0.000	0.000
PHY15	0.000	0.000	0.000	0.000	0.000
KES15	0.000	0.000	0.000	0.000	0.000
CHOICE15	0.000	0.000	0.000	0.000	0.000
BULLY15	0.000	0.000	0.000	0.000	0.000
PRESS15	0.000	0.000	0.000	0.000	0.000
DUTIES15	0.000	0.000	0.000	0.000	0.000

BETA

	PRESS15	DUTIES15
KESCAT16	0.000	0.000
PHY15	0.000	0.000
KES15	0.000	0.000
CHOICE15	0.000	0.000
BULLY15	0.000	0.000
PRESS15	0.000	0.000
DUTIES15	0.000	0.000

PSI

	KESCAT16	PHY15	KES15	CHOICE15	BULLY15
KESCAT16	1.000				
PHY15	0.000	0.268			
KES15	0.000	0.000	0.048		
CHOICE15	0.000	0.000	0.000	0.549	
BULLY15	0.000	0.000	0.000	0.000	0.108
PRESS15	0.000	0.000	0.000	0.000	0.000
DUTIES15	0.000	0.000	0.000	0.000	0.000

PSI

PRESS15 DUTIES15

PRESS15	0.501	
DUTIES15	0.000	0.414

STARTING VALUES FOR BETWEEN

TAU

KESCAT16
1.091

NU

KESCAT16	PRAFAC	DUTIES15
0.000	0.000	0.000

LAMBDA

	S1	KESCAT16	PRAFAC	DUTIES15
KESCAT16	0.000	1.000	0.000	0.000
PRAFAC	0.000	0.000	1.000	0.000
DUTIES15	0.000	0.000	0.000	1.000

THETA

	KESCAT16	PRAFAC	DUTIES15
KESCAT16	0.000		
PRAFAC	0.000	0.000	
DUTIES15	0.000	0.000	0.000

ALPHA

S1	KESCAT16	PRAFAC	DUTIES15
0.000	0.000	0.006	4.149

BETA

S1 KESCAT16 PRAFAC DUTIES15

S1	0.000	0.000	0.000	0.000
KESCAT16	0.000	0.000	0.000	0.000
PRAFAC	0.000	0.000	0.000	0.000
DUTIES15	0.000	0.000	0.000	0.000

PSI

	S1	KESCAT16	PRAFAC	DUTIES15
S1	1.000			
KESCAT16	0.000	1.000		
PRAFAC	0.000	0.000	0.506	
DUTIES15	0.000	0.000	0.000	0.414

PRIORS FOR ALL PARAMETERS	PRIOR MEAN	PRIOR VARIANCE	PRIOR STD. DEV.
Parameter 1~N(0.000,infinity)	0.0000	infinity	infinity
Parameter 2~N(0.000,infinity)	0.0000	infinity	infinity
Parameter 3~N(0.000,infinity)	0.0000	infinity	infinity
Parameter 4~N(0.000,infinity)	0.0000	infinity	infinity
Parameter 5~N(0.000,infinity)	0.0000	infinity	infinity
Parameter 6~N(0.000,5.000)	0.0000	5.0000	2.2361
Parameter 7~N(0.000,5.000)	0.0000	5.0000	2.2361
Parameter 8~N(0.000,5.000)	0.0000	5.0000	2.2361
Parameter 9~N(0.000,5.000)	0.0000	5.0000	2.2361
Parameter 10~N(0.000,5.000)	0.0000	5.0000	2.2361
Parameter 11~IW(1.000,7)	infinity	infinity	infinity
Parameter 12~IW(0.000,7)	infinity	infinity	infinity
Parameter 13~IW(1.000,7)	infinity	infinity	infinity
Parameter 14~IW(0.000,7)	infinity	infinity	infinity
Parameter 15~IW(0.000,7)	infinity	infinity	infinity
Parameter 16~IW(1.000,7)	infinity	infinity	infinity
Parameter 17~IW(0.000,7)	infinity	infinity	infinity
Parameter 18~IW(0.000,7)	infinity	infinity	infinity
Parameter 19~IW(0.000,7)	infinity	infinity	infinity
Parameter 20~IW(1.000,7)	infinity	infinity	infinity
Parameter 21~IW(0.000,7)	infinity	infinity	infinity
Parameter 22~IW(0.000,7)	infinity	infinity	infinity
Parameter 23~IW(0.000,7)	infinity	infinity	infinity
Parameter 24~IW(0.000,7)	infinity	infinity	infinity
Parameter 25~IW(1.000,7)	infinity	infinity	infinity

Parameter 26~IW(0.000,7)	infinity	infinity	infinity
Parameter 27~IW(0.000,7)	infinity	infinity	infinity
Parameter 28~IW(0.000,7)	infinity	infinity	infinity
Parameter 29~IW(0.000,7)	infinity	infinity	infinity
Parameter 30~IW(0.000,7)	infinity	infinity	infinity
Parameter 31~IW(1.000,7)	infinity	infinity	infinity
Parameter 32~N(0.000,infinity)	0.0000	infinity	infinity
Parameter 33~N(0.000,infinity)	0.0000	infinity	infinity
Parameter 34~N(0.000,infinity)	0.0000	infinity	infinity
Parameter 35~N(0.000,infinity)	0.0000	infinity	infinity
Parameter 36~N(0.000,5.000)	0.0000	5.0000	2.2361
Parameter 37~N(0.000,5.000)	0.0000	5.0000	2.2361
Parameter 38~IG(-1.000,0.000)	infinity	infinity	infinity
Parameter 39~IG(-1.000,0.000)	infinity	infinity	infinity
Parameter 40~IW(1.000,3)	infinity	infinity	infinity
Parameter 41~IW(0.000,3)	infinity	infinity	infinity
Parameter 42~IW(1.000,3)	infinity	infinity	infinity
Parameter 43~N(0.000,5.000)	0.0000	5.0000	2.2361

TECHNICAL 3 OUTPUT

ESTIMATED COVARIANCE MATRIX FOR PARAMETER ESTIMATES

	1	2	3	4	5
1	0.999091D-04				
2	0.103228D-04	0.164795D-04			
3	-0.180148D-04	-0.487187D-05	0.212170D-03		
4	0.443972D-05	0.442027D-05	-0.158921D-04	0.407089D-04	
5	0.910706D-05	0.836613D-05	-0.180970D-04	0.153779D-04	0.195045D-03
6	-0.102180D-04	-0.538552D-05	-0.267942D-05	-0.103968D-04	-0.106559D-04
7	0.607020D-05	0.647729D-05	0.308882D-04	0.198824D-05	0.252436D-04
8	-0.723219D-05	-0.480957D-05	0.186218D-04	-0.781877D-05	-0.976738D-05
9	-0.231101D-04	-0.536301D-05	-0.221289D-05	0.672009D-05	-0.179338D-04
10	0.581062D-05	0.255888D-05	-0.136205D-05	-0.279064D-05	0.265597D-04
11	0.757622D-06	0.451549D-07	0.222450D-05	0.275307D-05	-0.259135D-05
12	0.705281D-06	0.275729D-07	0.145182D-05	-0.569318D-07	-0.373074D-06
13	-0.403964D-06	0.136367D-06	0.134667D-05	-0.262918D-06	-0.344897D-07
14	0.172639D-05	0.138454D-05	0.324627D-05	0.201134D-05	0.185884D-05
15	0.986673D-06	0.653938D-06	-0.224778D-05	0.131712D-05	0.711486D-08
16	0.376175D-05	-0.298978D-05	0.698381D-05	0.273594D-05	0.679807D-05
17	0.122161D-05	0.432609D-07	0.104100D-05	0.115671D-05	0.829167D-06
18	0.210950D-06	0.196819D-06	0.185580D-05	-0.584277D-07	-0.577266D-06

19	0.604960D-06	0.161930D-05	-0.223376D-05	-0.131386D-05	-0.129632D-05
20	-0.118709D-05	0.316933D-06	0.101624D-05	0.185615D-06	0.431408D-06
21	-0.117542D-06	0.418980D-06	0.280952D-06	-0.182546D-06	0.655633D-05
22	0.547792D-06	0.238137D-06	-0.149227D-05	0.408009D-06	0.353053D-05
23	0.495138D-05	0.451655D-05	0.331002D-05	0.717273D-06	-0.101533D-04
24	-0.100668D-05	-0.146815D-06	-0.261488D-05	-0.540067D-06	0.786402D-06
25	-0.120392D-04	0.206811D-05	-0.115924D-04	-0.136096D-05	-0.338660D-06
26	0.211908D-05	-0.499490D-06	-0.596544D-05	-0.120657D-05	0.343270D-05
27	-0.945152D-06	-0.822339D-06	-0.325943D-05	-0.485610D-06	0.202233D-05
28	-0.684030D-05	-0.104227D-05	-0.471577D-06	0.333144D-05	-0.324109D-05
29	-0.426598D-06	-0.382542D-06	-0.287055D-05	-0.722294D-06	0.163259D-05
30	0.370473D-05	0.136536D-05	-0.425829D-05	0.849270D-07	0.388049D-06
31	0.150374D-05	0.130325D-05	0.481416D-05	0.307120D-05	0.306542D-05
32	-0.704587D-05	-0.277418D-05	0.307780D-04	-0.248756D-05	-0.325967D-05
33	-0.252118D-04	0.109671D-04	0.666841D-06	0.350487D-04	0.567792D-05
34	-0.921584D-05	-0.107630D-04	0.296324D-04	-0.109150D-04	-0.485127D-04
35	-0.115959D-04	-0.183093D-05	0.329105D-04	0.231670D-05	0.172991D-04
36	0.258540D-05	-0.115089D-05	0.611144D-05	0.529318D-05	0.220400D-05
37	-0.528025D-04	0.130593D-04	0.215593D-04	-0.133288D-04	-0.251672D-05
38	0.186329D-05	0.852929D-07	0.330381D-05	-0.167864D-05	0.253231D-06
39	-0.115437D-06	-0.530959D-06	0.669135D-05	-0.223873D-06	0.112118D-05
40	-0.327003D-05	-0.177723D-04	-0.229629D-04	0.674030D-05	-0.958471D-04
41	0.100882D-04	-0.318949D-05	0.125650D-04	-0.510946D-05	-0.731775D-05
42	-0.339054D-06	0.116572D-05	0.731490D-05	-0.106435D-05	-0.573370D-05
43	-0.223336D-03	0.575479D-04	0.880290D-04	-0.577278D-04	-0.132282D-04

ESTIMATED COVARIANCE MATRIX FOR PARAMETER ESTIMATES

	6	7	8	9	10
6	0.112463D-02				
7	-0.627563D-03	0.765091D-02			
8	0.105204D-03	0.424669D-05	0.675100D-03		
9	-0.155077D-03	-0.315365D-03	0.105835D-03	0.235606D-02	
10	-0.144806D-04	-0.275576D-03	0.143949D-05	-0.232834D-03	0.679740D-03
11	0.232258D-05	0.210620D-04	0.129978D-04	0.237268D-04	-0.494131D-05
12	-0.110505D-05	0.865212D-05	0.126346D-05	0.528652D-05	-0.871449D-06
13	-0.143493D-06	0.124698D-05	0.296068D-05	0.311205D-05	0.601472D-06
14	-0.391779D-05	0.211985D-04	0.548351D-05	-0.164619D-05	0.144421D-04
15	-0.783279D-06	0.787145D-05	0.849347D-06	-0.510255D-05	0.188986D-05
16	-0.337700D-04	-0.879501D-05	-0.917113D-05	0.920010D-05	-0.121443D-04
17	-0.655634D-05	0.123200D-04	-0.529802D-05	0.101900D-04	0.664342D-06
18	0.184153D-06	-0.101440D-05	0.110851D-05	0.289945D-05	0.123984D-05
19	0.555160D-05	-0.146146D-04	0.205438D-05	-0.558814D-05	0.525325D-05

20	-0.378336D-05	0.115166D-04	-0.148184D-05	0.416450D-05	-0.187240D-05
21	-0.965708D-05	0.393323D-04	0.765941D-05	0.183283D-04	0.539444D-05
22	-0.331131D-06	-0.292185D-05	0.349665D-05	0.481490D-05	0.178765D-05
23	0.644633D-05	0.189592D-04	0.778693D-05	-0.460537D-07	0.845644D-05
24	-0.707164D-05	0.164934D-04	0.314874D-06	0.573274D-05	-0.433859D-05
25	-0.882664D-05	0.851623D-05	-0.134038D-04	-0.359385D-06	0.864073D-05
26	0.581953D-05	-0.659688D-05	-0.154937D-04	0.103684D-04	0.242889D-05
27	0.100438D-05	0.208586D-04	-0.419296D-05	0.199701D-05	0.457989D-05
28	-0.170524D-06	0.509289D-04	-0.988351D-05	0.549877D-05	-0.243771D-05
29	-0.287446D-05	-0.109452D-04	0.139420D-05	0.356684D-05	0.376404D-05
30	0.173353D-07	0.155009D-04	0.672535D-05	-0.110139D-04	0.984748D-05
31	-0.113637D-04	0.474632D-04	-0.827549D-06	0.233436D-04	-0.617131D-05
32	-0.543863D-04	0.315438D-03	-0.846079D-04	0.333959D-04	0.793988D-04
33	-0.188390D-03	-0.150689D-03	-0.105541D-03	0.183653D-04	-0.236840D-04
34	0.175637D-04	-0.851437D-05	0.469975D-04	0.179410D-04	-0.119521D-04
35	0.132731D-04	0.117445D-03	-0.150183D-04	-0.149026D-04	0.718672D-05
36	0.592860D-04	0.941112D-04	-0.150475D-04	0.330676D-04	0.251958D-04
37	0.324317D-03	-0.808265D-03	-0.223318D-04	-0.750457D-03	0.674346D-04
38	-0.569366D-05	0.735293D-04	-0.131609D-04	-0.137826D-04	0.225664D-04
39	-0.375729D-05	0.450288D-04	0.395820D-05	0.129942D-04	0.122196D-04
40	0.244562D-04	-0.726288D-04	-0.128178D-03	-0.245910D-03	0.131777D-03
41	0.146635D-04	-0.273998D-04	0.595079D-05	0.532653D-04	0.382221D-04
42	0.104705D-04	0.222768D-04	0.105573D-04	0.101802D-04	-0.899162D-05
43	0.142302D-02	-0.220089D-02	-0.177365D-03	-0.311201D-02	0.265659D-03

ESTIMATED COVARIANCE MATRIX FOR PARAMETER ESTIMATES

	11	12	13	14	15
11	0.105262D-03				
12	0.105724D-04	0.104066D-04			
13	0.166187D-05	0.186505D-05	0.328525D-05		
14	-0.199749D-04	-0.614599D-05	-0.157940D-05	0.112569D-03	
15	-0.172328D-05	-0.253965D-05	-0.140421D-05	0.118359D-04	0.203502D-04
16	0.661050D-05	0.217379D-05	-0.832344D-07	-0.322744D-04	-0.118003D-04
17	0.374118D-05	0.213002D-05	0.327138D-06	-0.608625D-05	0.594416D-07
18	0.581147D-06	0.651949D-06	0.963578D-06	-0.961046D-06	-0.107620D-05
19	-0.291004D-05	-0.183895D-05	-0.880449D-06	0.507932D-05	0.533938D-05
20	0.274463D-05	0.364328D-06	0.269396D-06	-0.160430D-06	0.154530D-06
21	0.792006D-05	0.543545D-05	0.682357D-06	-0.108948D-04	-0.554337D-06
22	0.392784D-06	0.804609D-06	0.185179D-05	-0.739507D-06	-0.175050D-05
23	0.501836D-06	-0.133449D-05	-0.746741D-06	0.162381D-04	0.116658D-04
24	0.925078D-07	0.170820D-06	0.255121D-06	-0.926840D-07	-0.307065D-06
25	0.617905D-05	0.164898D-05	0.129372D-05	-0.595179D-05	-0.115926D-05

26	-0.103070D-04	-0.561901D-05	-0.127590D-05	0.202048D-04	0.452801D-06
27	-0.224514D-05	-0.171949D-05	-0.144774D-05	0.300947D-05	0.245747D-05
28	0.169285D-05	0.506588D-06	0.549830D-06	-0.121702D-04	-0.105767D-04
29	-0.900665D-06	-0.966678D-06	-0.531263D-06	-0.164173D-05	0.992200D-07
30	-0.442374D-05	-0.172711D-05	-0.227800D-06	0.451501D-05	0.175119D-05
31	0.453819D-05	0.171742D-06	0.262194D-06	-0.664157D-05	-0.120440D-05
32	-0.206534D-05	0.165215D-05	0.856895D-06	-0.116875D-04	0.373712D-06
33	0.489549D-05	-0.286721D-05	-0.627999D-05	-0.736975D-04	0.170365D-04
34	-0.343144D-05	0.113937D-05	-0.442228D-06	0.114764D-04	0.156529D-05
35	0.648550D-05	-0.504187D-06	0.905247D-06	-0.128755D-04	-0.293870D-06
36	0.342208D-05	-0.215887D-05	0.160029D-06	-0.814369D-05	-0.504348D-05
37	0.609235D-05	-0.450702D-04	-0.105296D-04	0.394347D-04	0.359699D-04
38	-0.158212D-05	-0.216314D-05	-0.130385D-05	-0.120883D-05	0.299289D-05
39	0.629240D-06	0.340689D-06	-0.620426D-06	-0.977972D-06	-0.355129D-06
40	0.417265D-04	-0.873074D-05	0.255200D-05	0.402116D-04	0.683758D-05
41	-0.784428D-05	-0.431060D-05	0.236238D-05	0.298257D-04	0.913772D-05
42	0.145950D-05	-0.780983D-06	0.271440D-06	0.141933D-05	-0.218476D-06
43	0.230863D-04	-0.187918D-03	-0.446284D-04	0.174762D-03	0.149238D-03

ESTIMATED COVARIANCE MATRIX FOR PARAMETER ESTIMATES

	16	17	18	19	20
16	0.463958D-03				
17	0.176402D-05	0.218601D-04			
18	-0.235687D-06	0.259098D-05	0.396598D-05		
19	-0.146679D-04	-0.450092D-05	-0.165290D-05	0.446060D-04	
20	0.107481D-05	0.256140D-05	0.183123D-05	-0.366394D-05	0.184603D-04
21	-0.967138D-05	0.892704D-05	0.117173D-05	-0.276540D-05	-0.282013D-06
22	-0.908059D-06	0.457057D-06	0.187159D-05	-0.182929D-05	-0.480817D-07
23	-0.343809D-04	0.254891D-05	-0.459121D-06	0.192474D-04	0.147871D-05
24	-0.339303D-07	0.269544D-05	0.233850D-05	-0.207394D-05	0.846277D-05
25	-0.653360D-05	0.232112D-05	0.130428D-05	-0.167664D-05	0.119718D-05
26	-0.816368D-05	-0.456020D-05	-0.717093D-06	0.304934D-05	-0.191008D-05
27	-0.395252D-05	-0.590414D-06	-0.110630D-05	0.110093D-05	-0.385200D-06
28	0.624380D-04	0.267854D-05	0.914081D-06	-0.107259D-04	0.159421D-05
29	-0.666956D-05	-0.167109D-05	-0.157898D-05	0.611225D-05	-0.485410D-05
30	-0.509867D-05	-0.183938D-07	-0.260846D-06	0.249925D-05	-0.857922D-06
31	0.150834D-04	0.446777D-05	0.366036D-06	-0.326745D-05	0.198652D-06
32	0.217582D-04	0.182675D-05	0.229248D-05	-0.478742D-05	-0.810637D-05
33	0.118643D-03	0.340377D-04	-0.106420D-04	0.258243D-04	0.426500D-05
34	0.103050D-04	-0.157618D-06	0.151619D-05	0.324784D-05	0.793930D-06
35	-0.905179D-05	-0.680203D-06	-0.198173D-05	-0.471853D-05	-0.628573D-05
36	0.177225D-04	0.410022D-05	0.463491D-06	0.144734D-06	-0.247525D-05

37	-0.119617D-03	-0.591605D-04	-0.503151D-05	0.474929D-04	0.181685D-04
38	-0.674590D-05	-0.199342D-06	-0.121659D-05	0.314655D-05	-0.261738D-05
39	0.832324D-05	0.256718D-05	-0.365795D-06	0.401140D-06	0.574335D-06
40	-0.261484D-03	-0.119949D-04	0.307162D-04	-0.685464D-04	-0.197737D-04
41	0.224708D-04	-0.394539D-05	0.312600D-05	-0.564344D-05	0.348364D-06
42	0.558734D-07	-0.533222D-06	0.683474D-07	0.888974D-06	0.166947D-05
43	-0.489174D-03	-0.249512D-03	-0.218056D-04	0.193916D-03	0.775075D-04

ESTIMATED COVARIANCE MATRIX FOR PARAMETER ESTIMATES

	21	22	23	24	25
21	0.106265D-03				
22	0.102292D-04	0.173305D-04			
23	-0.132534D-04	-0.108089D-04	0.213218D-03		
24	0.286441D-05	0.437309D-05	-0.949158D-05	0.408041D-04	
25	0.208159D-04	0.190742D-04	-0.232974D-04	0.318052D-04	0.391142D-03
26	-0.171637D-04	-0.190570D-05	0.238982D-05	-0.680989D-06	-0.363806D-05
27	-0.217093D-05	-0.270350D-05	0.333342D-05	-0.399686D-06	-0.135852D-05
28	-0.156509D-05	0.201391D-05	-0.338084D-04	0.536728D-05	-0.196677D-05
29	-0.314198D-05	-0.100514D-05	0.798635D-06	-0.592906D-05	-0.522349D-05
30	-0.120013D-04	-0.114526D-04	0.314999D-04	-0.118948D-04	-0.558891D-04
31	0.407949D-05	0.290645D-05	-0.130680D-04	0.576549D-05	0.152158D-04
32	0.472462D-05	-0.256750D-05	-0.264470D-06	0.290307D-06	0.407900D-04
33	-0.502268D-05	-0.151115D-04	0.660727D-06	-0.206990D-05	0.169501D-03
34	-0.652810D-05	-0.738892D-06	-0.110945D-04	-0.233209D-05	0.293193D-04
35	-0.550716D-06	0.567352D-06	0.108661D-04	-0.738655D-05	0.130500D-04
36	0.373619D-05	0.356012D-05	-0.189644D-04	0.403087D-05	0.156858D-04
37	-0.191100D-04	-0.421354D-04	0.341241D-04	0.238544D-05	-0.568272D-04
38	-0.223255D-05	0.678308D-06	-0.119125D-05	-0.204840D-05	-0.424247D-05
39	0.300116D-05	0.503660D-06	0.267876D-06	-0.107898D-06	-0.296641D-05
40	0.129067D-03	0.388995D-04	-0.107766D-03	-0.156738D-04	0.434582D-04
41	-0.104808D-05	0.928880D-05	0.188699D-04	0.714834D-05	0.412027D-04
42	0.536082D-06	0.151023D-05	-0.835210D-06	-0.109613D-05	0.837808D-05
43	-0.841442D-04	-0.178707D-03	0.142868D-03	0.274772D-05	-0.245156D-03

ESTIMATED COVARIANCE MATRIX FOR PARAMETER ESTIMATES

	26	27	28	29	30
26	0.857637D-04				
27	0.960428D-05	0.145906D-04			
28	-0.165064D-04	-0.601790D-05	0.186896D-03		
29	0.260929D-05	0.340741D-05	-0.844153D-05	0.336838D-04	

30	0.101769D-04	0.936063D-05	-0.155827D-04	0.147482D-04	0.167721D-03
31	-0.223677D-04	-0.152383D-04	0.506611D-04	-0.170783D-04	-0.567282D-04
32	0.355941D-05	0.850972D-05	-0.131022D-04	0.922460D-05	0.107832D-04
33	-0.517465D-04	0.106096D-05	0.922938D-05	-0.308954D-04	-0.202298D-04
34	0.291744D-05	-0.319320D-05	-0.691842D-05	-0.199507D-05	0.657018D-05
35	0.349233D-05	0.111240D-05	-0.642704D-05	0.834811D-05	-0.684647D-05
36	0.123483D-05	0.338271D-05	0.203298D-05	-0.352326D-05	0.107117D-04
37	0.131385D-03	0.204820D-04	0.724096D-04	0.164880D-04	0.355149D-04
38	-0.217977D-05	0.165500D-05	0.338742D-05	0.432083D-05	-0.481052D-05
39	-0.253336D-06	0.258595D-06	0.231860D-06	-0.940393D-06	-0.352813D-05
40	0.215715D-04	-0.876504D-06	-0.720549D-04	-0.137816D-04	0.229954D-04
41	0.133462D-04	-0.735622D-05	-0.890150D-05	-0.371248D-05	0.100783D-04
42	0.214407D-05	-0.466961D-06	-0.228603D-05	-0.767945D-06	0.125608D-05
43	0.535711D-03	0.895522D-04	0.300307D-03	0.665042D-04	0.132652D-03

ESTIMATED COVARIANCE MATRIX FOR PARAMETER ESTIMATES

	31	32	33	34	35
31	0.245700D-03				
32	-0.365496D-04	0.140167D-02			
33	0.192630D-04	-0.880926D-04	0.231178D-01		
34	-0.245294D-05	0.143410D-05	0.761218D-03	0.969335D-03	
35	0.353441D-05	0.229109D-03	-0.268002D-03	-0.185022D-04	0.122125D-02
36	0.244329D-05	-0.293696D-04	-0.103955D-03	-0.244900D-04	0.525352D-04
37	-0.241052D-03	0.274857D-03	0.124776D-03	0.154604D-03	-0.652772D-04
38	0.484055D-05	0.113112D-04	-0.232636D-04	-0.190822D-04	0.755830D-04
39	-0.430855D-06	-0.177323D-04	0.340548D-04	0.865555D-05	0.148203D-04
40	0.409171D-04	-0.876841D-04	-0.800197D-03	0.617527D-04	-0.157194D-03
41	-0.120574D-04	0.925526D-04	0.161401D-03	0.302022D-04	-0.132317D-04
42	-0.209224D-05	0.116882D-04	0.289180D-05	0.149324D-04	-0.171227D-05
43	-0.980880D-03	0.112043D-02	0.507746D-03	0.575852D-03	-0.156720D-03

ESTIMATED COVARIANCE MATRIX FOR PARAMETER ESTIMATES

	36	37	38	39	40
36	0.879431D-03				
37	-0.154021D-02	0.690078D-01			
38	0.636941D-05	-0.560166D-05	0.141747D-03		
39	0.321091D-04	-0.421831D-03	0.878861D-05	0.668060D-04	
40	0.108739D-04	0.377760D-03	0.546594D-04	0.307412D-04	0.722992D-01
41	0.578588D-05	0.378544D-03	0.889306D-05	-0.559470D-06	0.220199D-02
42	-0.837647D-06	0.125645D-03	-0.138502D-05	-0.435674D-05	0.148283D-03

43	-0.643552D-02	0.286760D+00	0.222647D-04	-0.171721D-02	0.168775D-02

ESTIMATED COVARIANCE MATRIX FOR PARAMETER ESTIMATES

	41	42	43
41	0.148883D-02		
42	0.913649D-04	0.786129D-04	
43	0.160524D-02	0.516040D-03	0.119286D+01

ESTIMATED CORRELATION MATRIX FOR PARAMETER ESTIMATES

	1	2	3	4	5
1	1.000				
2	0.254	1.000			
3	-0.124	-0.082	1.000		
4	0.070	0.171	-0.171	1.000	
5	0.065	0.148	-0.089	0.173	1.000
6	-0.030	-0.040	-0.005	-0.049	-0.023
7	0.007	0.018	0.024	0.004	0.021
8	-0.028	-0.046	0.049	-0.047	-0.027
9	-0.048	-0.027	-0.003	0.022	-0.026
10	0.022	0.024	-0.004	-0.017	0.073
11	0.007	0.001	0.015	0.042	-0.018
12	0.022	0.002	0.031	-0.003	-0.008
13	-0.022	0.019	0.051	-0.023	-0.001
14	0.016	0.032	0.021	0.030	0.013
15	0.022	0.036	-0.034	0.046	0.000
16	0.017	-0.034	0.022	0.020	0.023
17	0.026	0.002	0.015	0.039	0.013
18	0.011	0.024	0.064	-0.005	-0.021
19	0.009	0.060	-0.023	-0.031	-0.014
20	-0.028	0.018	0.016	0.007	0.007
21	-0.001	0.010	0.002	-0.003	0.046
22	0.013	0.014	-0.025	0.015	0.061
23	0.034	0.076	0.016	0.008	-0.050
24	-0.016	-0.006	-0.028	-0.013	0.009
25	-0.061	0.026	-0.040	-0.011	-0.001
26	0.023	-0.013	-0.044	-0.020	0.027
27	-0.025	-0.053	-0.059	-0.020	0.038
28	-0.050	-0.019	-0.002	0.038	-0.017
29	-0.007	-0.016	-0.034	-0.020	0.020
30	0.029	0.026	-0.023	0.001	0.002

31	0.010	0.020	0.021	0.031	0.014
32	-0.019	-0.018	0.056	-0.010	-0.006
33	-0.017	0.018	0.000	0.036	0.003
34	-0.030	-0.085	0.065	-0.055	-0.112
35	-0.033	-0.013	0.065	0.010	0.035
36	0.009	-0.010	0.014	0.028	0.005
37	-0.020	0.012	0.006	-0.008	-0.001
38	0.016	0.002	0.019	-0.022	0.002
39	-0.001	-0.016	0.056	-0.004	0.010
40	-0.001	-0.016	-0.006	0.004	-0.026
41	0.026	-0.020	0.022	-0.021	-0.014
42	-0.004	0.032	0.057	-0.019	-0.046
43	-0.020	0.013	0.006	-0.008	-0.001

ESTIMATED CORRELATION MATRIX FOR PARAMETER ESTIMATES

	6	7	8	9	10
6	1.000				
7	-0.214	1.000			
8	0.121	0.002	1.000		
9	-0.095	-0.074	0.084	1.000	
10	-0.017	-0.121	0.002	-0.184	1.000
11	0.007	0.023	0.049	0.048	-0.018
12	-0.010	0.031	0.015	0.034	-0.010
13	-0.002	0.008	0.063	0.035	0.013
14	-0.011	0.023	0.020	-0.003	0.052
15	-0.005	0.020	0.007	-0.023	0.016
16	-0.047	-0.005	-0.016	0.009	-0.022
17	-0.042	0.030	-0.044	0.045	0.005
18	0.003	-0.006	0.021	0.030	0.024
19	0.025	-0.025	0.012	-0.017	0.030
20	-0.026	0.031	-0.013	0.020	-0.017
21	-0.028	0.044	0.029	0.037	0.020
22	-0.002	-0.008	0.032	0.024	0.016
23	0.013	0.015	0.021	0.000	0.022
24	-0.033	0.030	0.002	0.018	-0.026
25	-0.013	0.005	-0.026	0.000	0.017
26	0.019	-0.008	-0.064	0.023	0.010
27	0.008	0.062	-0.042	0.011	0.046
28	0.000	0.043	-0.028	0.008	-0.007
29	-0.015	-0.022	0.009	0.013	0.025
30	0.000	0.014	0.020	-0.018	0.029
31	-0.022	0.035	-0.002	0.031	-0.015

32	-0.043	0.096	-0.087	0.018	0.081
33	-0.037	-0.011	-0.027	0.002	-0.006
34	0.017	-0.003	0.058	0.012	-0.015
35	0.011	0.038	-0.017	-0.009	0.008
36	0.060	0.036	-0.020	0.023	0.033
37	0.037	-0.035	-0.003	-0.059	0.010
38	-0.014	0.071	-0.043	-0.024	0.073
39	-0.014	0.063	0.019	0.033	0.057
40	0.003	-0.003	-0.018	-0.019	0.019
41	0.011	-0.008	0.006	0.028	0.038
42	0.035	0.029	0.046	0.024	-0.039
43	0.039	-0.023	-0.006	-0.059	0.009

ESTIMATED CORRELATION MATRIX FOR PARAMETER ESTIMATES

	11	12	13	14	15
11	1.000				
12	0.319	1.000			
13	0.089	0.319	1.000		
14	-0.184	-0.180	-0.082	1.000	
15	-0.037	-0.175	-0.172	0.247	1.000
16	0.030	0.031	-0.002	-0.141	-0.121
17	0.078	0.141	0.039	-0.123	0.003
18	0.028	0.101	0.267	-0.045	-0.120
19	-0.042	-0.085	-0.073	0.072	0.177
20	0.062	0.026	0.035	-0.004	0.008
21	0.075	0.163	0.037	-0.100	-0.012
22	0.009	0.060	0.245	-0.017	-0.093
23	0.003	-0.028	-0.028	0.105	0.177
24	0.001	0.008	0.022	-0.001	-0.011
25	0.030	0.026	0.036	-0.028	-0.013
26	-0.108	-0.188	-0.076	0.206	0.011
27	-0.057	-0.140	-0.209	0.074	0.143
28	0.012	0.011	0.022	-0.084	-0.172
29	-0.015	-0.052	-0.051	-0.027	0.004
30	-0.033	-0.041	-0.010	0.033	0.030
31	0.028	0.003	0.009	-0.040	-0.017
32	-0.005	0.014	0.013	-0.029	0.002
33	0.003	-0.006	-0.023	-0.046	0.025
34	-0.011	0.011	-0.008	0.035	0.011
35	0.018	-0.004	0.014	-0.035	-0.002
36	0.011	-0.023	0.003	-0.026	-0.038
37	0.002	-0.053	-0.022	0.014	0.030

38	-0.013	-0.056	-0.060	-0.010	0.056
39	0.008	0.013	-0.042	-0.011	-0.010
40	0.015	-0.010	0.005	0.014	0.006
41	-0.020	-0.035	0.034	0.073	0.052
42	0.016	-0.027	0.017	0.015	-0.005
43	0.002	-0.053	-0.023	0.015	0.030

ESTIMATED CORRELATION MATRIX FOR PARAMETER ESTIMATES

	16	17	18	19	20
16	1.000				
17	0.018	1.000			
18	-0.005	0.278	1.000		
19	-0.102	-0.144	-0.124	1.000	
20	0.012	0.128	0.214	-0.128	1.000
21	-0.044	0.185	0.057	-0.040	-0.006
22	-0.010	0.023	0.226	-0.066	-0.003
23	-0.109	0.037	-0.016	0.197	0.024
24	0.000	0.090	0.184	-0.049	0.308
25	-0.015	0.025	0.033	-0.013	0.014
26	-0.041	-0.105	-0.039	0.049	-0.048
27	-0.048	-0.033	-0.145	0.043	-0.023
28	0.212	0.042	0.034	-0.117	0.027
29	-0.053	-0.062	-0.137	0.158	-0.195
30	-0.018	0.000	-0.010	0.029	-0.015
31	0.045	0.061	0.012	-0.031	0.003
32	0.027	0.010	0.031	-0.019	-0.050
33	0.036	0.048	-0.035	0.025	0.007
34	0.015	-0.001	0.024	0.016	0.006
35	-0.012	-0.004	-0.028	-0.020	-0.042
36	0.028	0.030	0.008	0.001	-0.019
37	-0.021	-0.048	-0.010	0.027	0.016
38	-0.026	-0.004	-0.051	0.040	-0.051
39	0.047	0.067	-0.022	0.007	0.016
40	-0.045	-0.010	0.057	-0.038	-0.017
41	0.027	-0.022	0.041	-0.022	0.002
42	0.000	-0.013	0.004	0.015	0.044
43	-0.021	-0.049	-0.010	0.027	0.017

ESTIMATED CORRELATION MATRIX FOR PARAMETER ESTIMATES

21	22	23	24	25

21	1.000				
22	0.238	1.000			
23	-0.088	-0.178	1.000		
24	0.043	0.164	-0.102	1.000	
25	0.102	0.232	-0.081	0.252	1.000
26	-0.180	-0.049	0.018	-0.012	-0.020
27	-0.055	-0.170	0.060	-0.016	-0.018
28	-0.011	0.035	-0.169	0.061	-0.007
29	-0.053	-0.042	0.009	-0.160	-0.046
30	-0.090	-0.212	0.167	-0.144	-0.218
31	0.025	0.045	-0.057	0.058	0.049
32	0.012	-0.016	0.000	0.001	0.055
33	-0.003	-0.024	0.000	-0.002	0.056
34	-0.020	-0.006	-0.024	-0.012	0.048
35	-0.002	0.004	0.021	-0.033	0.019
36	0.012	0.029	-0.044	0.021	0.027
37	-0.007	-0.039	0.009	0.001	-0.011
38	-0.018	0.014	-0.007	-0.027	-0.018
39	0.036	0.015	0.002	-0.002	-0.018
40	0.047	0.035	-0.027	-0.009	0.008
41	-0.003	0.058	0.033	0.029	0.054
42	0.006	0.041	-0.006	-0.019	0.048
43	-0.007	-0.039	0.009	0.000	-0.011

ESTIMATED CORRELATION MATRIX FOR PARAMETER ESTIMATES

	26	27	28	29	30
26	1.000				
27	0.272	1.000			
28	-0.130	-0.115	1.000		
29	0.049	0.154	-0.106	1.000	
30	0.085	0.189	-0.088	0.196	1.000
31	-0.154	-0.255	0.236	-0.188	-0.279
32	0.010	0.060	-0.026	0.042	0.022
33	-0.037	0.002	0.004	-0.035	-0.010
34	0.010	-0.027	-0.016	-0.011	0.016
35	0.011	0.008	-0.013	0.041	-0.015
36	0.004	0.030	0.005	-0.020	0.028
37	0.054	0.020	0.020	0.011	0.010
38	-0.020	0.036	0.021	0.063	-0.031
39	-0.003	0.008	0.002	-0.020	-0.033
40	0.009	-0.001	-0.020	-0.009	0.007
41	0.037	-0.050	-0.017	-0.017	0.020

42	0.026	-0.014	-0.019	-0.015	0.011
43	0.053	0.021	0.020	0.010	0.009

ESTIMATED CORRELATION MATRIX FOR PARAMETER ESTIMATES

	31	32	33	34	35
31	1.000				
32	-0.062	1.000			
33	0.008	-0.015	1.000		
34	-0.005	0.001	0.161	1.000	
35	0.006	0.175	-0.050	-0.017	1.000
36	0.005	-0.026	-0.023	-0.027	0.051
37	-0.059	0.028	0.003	0.019	-0.007
38	0.026	0.025	-0.013	-0.051	0.182
39	-0.003	-0.058	0.027	0.034	0.052
40	0.010	-0.009	-0.020	0.007	-0.017
41	-0.020	0.064	0.028	0.025	-0.010
42	-0.015	0.035	0.002	0.054	-0.006
43	-0.057	0.027	0.003	0.017	-0.004

ESTIMATED CORRELATION MATRIX FOR PARAMETER ESTIMATES

	36	37	38	39	40
36	1.000				
37	-0.198	1.000			
38	0.018	-0.002	1.000		
39	0.132	-0.196	0.090	1.000	
40	0.001	0.005	0.017	0.014	1.000
41	0.005	0.037	0.019	-0.002	0.212
42	-0.003	0.054	-0.013	-0.060	0.062
43	-0.199	0.999	0.002	-0.192	0.006

ESTIMATED CORRELATION MATRIX FOR PARAMETER ESTIMATES

	41	42	43
41	1.000		
42	0.267	1.000	
43	0.038	0.053	1.000

TECHNICAL 8 OUTPUT

TECHNICAL 8 OUTPUT FOR BAYES ESTIMATION

CHAIN	BSEED
1	0
2	285380
3	253358

ITERATION	POTENTIAL SCALE REDUCTION	PARAMETER WITH HIGHEST PSR
100	1.005	32
200	1.008	38
300	1.006	39
400	1.002	38
500	1.003	36
600	1.004	38
700	1.001	8
800	1.001	10
900	1.004	10
1000	1.003	10

Reduced psychological distress, job control interaction

SUMMARY OF DATA

Number of clusters 58

Size (s)	Cluster ID with Size s
1	166
2	74 205
3	106 182 165
4	93 170 22 192 4
5	65 71 50 84
6	80 72
7	40 107 61 75
8	169 30 173 228
9	144 81 83 181
10	69 145 36 68
11	108 116 174 154
12	206 162
13	226 114
14	2
15	152 98
18	156
19	202
21	92 6
24	178
25	142 122
32	24
37	204
47	209
53	125
67	217
174	110
210	100

COVARIANCE COVERAGE OF DATA

Minimum covariance coverage value 0.100

Number of missing data patterns 5

PROPORTION OF DATA PRESENT

Covariance Coverage

	KESCAT16	PRAFAC	PHY15	KES15	CHOICE15
KESCAT16	1.000				
PRAFAC	1.000	1.000			
PHY15	1.000	1.000	1.000		
KES15	1.000	1.000	1.000	1.000	
CHOICE15	0.990	0.990	0.990	0.990	0.990
BULLY15	0.990	0.990	0.990	0.990	0.989
PRESS15	0.990	0.990	0.990	0.990	0.989
DUTIES15	0.991	0.991	0.991	0.991	0.990
PRAFACSQ	1.000	1.000	1.000	1.000	0.990

Covariance Coverage

	BULLY15	PRESS15	DUTIES15	PRAFACSQ
BULLY15	0.990			
PRESS15	0.989	0.990		
DUTIES15	0.990	0.990	0.991	
PRAFACSQ	0.990	0.990	0.991	1.000

UNIVARIATE PROPORTIONS AND COUNTS FOR CATEGORICAL VARIABLES

KESCAT16

Category 1	0.347	386.000
Category 2	0.653	727.000

UNIVARIATE SAMPLE STATISTICS

UNIVARIATE HIGHER-ORDER MOMENT DESCRIPTIVE STATISTICS

Variable/ Sample Size	Mean/ Variance	Skewness/ Kurtosis	Minimum/ Maximum	% with Min/Max	Percentiles 20%/60%	40%/80%	Median
PRAFAC	-0.185	0.245	-2.338	1.72%	-1.448	-0.527	-0.318

```
          58.000       1.345      -0.636       2.338    1.72%       0.080      0.752
     PHY15             0.000       0.390      -1.412   11.50%      -0.412     -0.412     -0.412
        1113.000       0.702       0.082       2.588    1.08%       0.588      0.588
     KES15             0.000       1.366      -0.556   20.75%      -0.556     -0.222     -0.222
        1113.000       0.313       1.871       2.278    0.63%      -0.056      0.444
     CHOICE15          2.814      -0.116       1.000   15.06%       2.000      3.000      3.000
        1102.000       1.193      -0.747       5.000    4.45%       3.000      4.000
     BULLY15           0.000       1.887      -0.426   71.96%      -0.426     -0.426     -0.426
        1102.000       0.602       3.132       3.574    0.45%      -0.426      0.574
     PRESS15           0.000       0.096      -1.915    9.89%      -0.915      0.085      0.085
        1102.000       1.160      -0.512       2.085    8.35%       0.085      1.085
     DUTIES15          0.000      -0.677      -2.672    3.54%      -0.672      0.328      0.328
        1103.000       1.031       0.049       1.328   20.22%       0.328      1.328
     PRAFACSQ          0.940       2.150       0.002    0.72%       0.220      0.280      0.469
        1113.000       1.309       4.555       5.467    0.36%       0.932      1.171
```

THE MODEL ESTIMATION TERMINATED NORMALLY

USE THE FBITERATIONS OPTION TO INCREASE THE NUMBER OF ITERATIONS BY A FACTOR
OF AT LEAST TWO TO CHECK CONVERGENCE AND THAT THE PSR VALUE DOES NOT INCREASE.

MODEL FIT INFORMATION

Number of Free Parameters   46

MODEL RESULTS

	Estimate	Posterior S.D.	One-Tailed P-Value	95% C.I. Lower 2.5%	95% C.I. Upper 2.5%	Significance
Within Level						
KESCAT16 ON						
PHY15	0.161	0.053	0.001	0.052	0.260	*
BULLY15	0.110	0.061	0.031	-0.008	0.237	
DUTIES15	-0.080	0.043	0.034	-0.162	0.009	
PRESS15	-0.046	0.042	0.130	-0.129	0.035	
KES15	0.800	0.093	0.000	0.614	0.983	*

KES15 WITH						
PHY15	0.107	0.014	0.000	0.081	0.136	*
CHOICE15	-0.089	0.018	0.000	-0.125	-0.053	*
BULLY15	0.073	0.013	0.000	0.048	0.099	*
PRESS15	0.062	0.019	0.001	0.026	0.100	*
DUTIES15	-0.073	0.017	0.000	-0.107	-0.041	*
PHY15 WITH						
CHOICE15	-0.088	0.027	0.001	-0.143	-0.036	*
BULLY15	0.040	0.019	0.017	0.003	0.078	*
PRESS15	0.120	0.027	0.000	0.069	0.174	*
DUTIES15	-0.058	0.027	0.015	-0.114	-0.005	*
CHOICE15 WITH						
BULLY15	-0.131	0.026	0.000	-0.181	-0.083	*
PRESS15	-0.180	0.036	0.000	-0.250	-0.112	*
DUTIES15	0.150	0.034	0.000	0.084	0.217	*
BULLY15 WITH						
PRESS15	0.200	0.026	0.000	0.149	0.251	*
DUTIES15	-0.175	0.024	0.000	-0.226	-0.129	*
PRESS15 WITH						
DUTIES15	-0.285	0.034	0.000	-0.354	-0.220	*
Means						
PHY15	0.001	0.025	0.485	-0.048	0.049	
KES15	0.001	0.017	0.484	-0.032	0.033	
BULLY15	0.002	0.024	0.481	-0.046	0.046	
PRESS15	0.001	0.033	0.486	-0.060	0.067	
DUTIES15	0.000	0.031	0.497	-0.062	0.061	
Variances						
PHY15	0.704	0.030	0.000	0.650	0.764	*
KES15	0.314	0.013	0.000	0.290	0.340	*
CHOICE15	1.141	0.050	0.000	1.048	1.246	*
BULLY15	0.601	0.026	0.000	0.555	0.657	*
PRESS15	1.159	0.050	0.000	1.068	1.266	*
DUTIES15	1.032	0.043	0.000	0.953	1.119	*
PRAFACSQ	0.000	0.000	0.000	0.000	0.000	*

Between Level

S1 ON

```
    PRAFAC             0.028       0.049       0.273      -0.070       0.130

 KESCAT16   ON
    PRAFAC            -0.095       0.056       0.048      -0.200       0.015
    CHOICE15          -0.330       0.269       0.098      -0.874       0.198

 PRAFAC   WITH
    CHOICE15           0.082       0.076       0.109      -0.055       0.254

 Means
    PRAFAC            -0.192       0.153       0.098      -0.488       0.110
    CHOICE15           2.888       0.062       0.000       2.771       3.017      *
    PRAFACSQ           1.381       0.212       0.000       0.970       1.790      *

 Intercepts
    S1                 0.033       0.051       0.257      -0.063       0.135

 Thresholds
    KESCAT16$1        -1.395       0.764       0.033      -2.927       0.130

 Variances
    PRAFAC             1.346       0.263       0.000       0.957       1.999      *
    CHOICE15           0.097       0.030       0.000       0.056       0.174      *
    PRAFACSQ           2.540       0.520       0.000       1.769       3.814      *

 Residual Variances
    KESCAT16           0.016       0.029       0.000       0.001       0.103      *
    S1                 0.013       0.018       0.000       0.001       0.070      *


STANDARDIZED MODEL RESULTS


STDYX Standardization

                                Posterior  One-Tailed         95% C.I.
                    Estimate       S.D.      P-Value   Lower 2.5%  Upper 2.5%  Significance

Within-Level Standardized Estimates Averaged Over Clusters

 S1 | KESCAT16 ON
    CHOICE15           0.027       0.044       0.269      -0.057       0.115

 KESCAT16   ON
```

PHY15	0.118	0.038	0.000	0.042	0.193	*
BULLY15	0.074	0.041	0.031	-0.005	0.157	
DUTIES15	-0.071	0.039	0.032	-0.148	0.004	
PRESS15	-0.043	0.039	0.130	-0.120	0.032	
KES15	0.393	0.039	0.000	0.312	0.465	*
KES15 WITH						
PHY15	0.228	0.028	0.000	0.173	0.283	*
CHOICE15	-0.148	0.029	0.000	-0.205	-0.089	*
BULLY15	0.168	0.029	0.000	0.110	0.222	*
PRESS15	0.104	0.031	0.001	0.046	0.163	*
DUTIES15	-0.129	0.029	0.000	-0.187	-0.072	*
PHY15 WITH						
CHOICE15	-0.099	0.030	0.001	-0.157	-0.040	*
BULLY15	0.061	0.030	0.022	0.002	0.117	*
PRESS15	0.133	0.030	0.000	0.074	0.190	*
DUTIES15	-0.068	0.031	0.013	-0.130	-0.008	*
CHOICE15 WITH						
BULLY15	-0.158	0.029	0.000	-0.217	-0.100	*
PRESS15	-0.155	0.030	0.000	-0.215	-0.097	*
DUTIES15	0.138	0.030	0.000	0.077	0.196	*
BULLY15 WITH						
PRESS15	0.239	0.029	0.000	0.181	0.294	*
DUTIES15	-0.222	0.029	0.000	-0.278	-0.166	*
PRESS15 WITH						
DUTIES15	-0.261	0.028	0.000	-0.314	-0.205	*
Means						
PHY15	0.000	0.030	0.492	-0.058	0.063	
KES15	0.001	0.030	0.495	-0.056	0.061	
BULLY15	0.002	0.031	0.481	-0.059	0.061	
PRESS15	0.001	0.030	0.492	-0.059	0.062	
DUTIES15	0.000	0.030	0.495	-0.060	0.059	
Variances						
PHY15	1.000	0.000	0.000	1.000	1.000	
KES15	1.000	0.000	0.000	1.000	1.000	
CHOICE15	1.000	0.000	0.000	1.000	1.000	
BULLY15	1.000	0.000	0.000	1.000	1.000	
PRESS15	1.000	0.000	0.000	1.000	1.000	

```
    DUTIES15           1.000       0.000       0.000       1.000       1.000
    PRAFACSQ           1.000       0.000       0.000       1.000       1.000

Between Level

 S1         ON
    PRAFAC             0.277       0.407       0.273      -0.658       0.889

 KESCAT16   ON
    PRAFAC            -0.484       0.260       0.048      -0.902       0.079
    CHOICE15          -0.475       0.315       0.098      -0.930       0.259

 PRAFAC   WITH
    CHOICE15           0.236       0.182       0.109      -0.145       0.562

 Means
    PRAFAC            -0.165       0.130       0.098      -0.413       0.093
    CHOICE15           9.226       1.337       0.000       6.926      12.206      *
    PRAFACSQ           0.869       0.153       0.000       0.577       1.157      *

 Intercepts
    S1                 0.245       0.552       0.257      -0.638       1.578

 Variances
    PRAFAC             1.000       0.000       0.000       1.000       1.000
    CHOICE15           1.000       0.000       0.000       1.000       1.000
    PRAFACSQ           1.000       0.000       0.000       1.000       1.000

 Residual Variances
    KESCAT16           0.311       0.246       0.000       0.020       0.896      *
    S1                 0.874       0.228       0.000       0.197       1.000      *


R-SQUARE

Within-Level R-Square Averaged Across Clusters

                                Posterior  One-Tailed         95% C.I.
    Variable        Estimate       S.D.      P-Value   Lower 2.5%  Upper 2.5%

    KESCAT16           0.233       0.036       0.000       0.167       0.306

Between Level
```

Variable	Estimate	Posterior S.D.	One-Tailed P-Value	95% C.I. Lower 2.5%	Upper 2.5%
KESCAT16	0.689	0.246	0.000	0.104	0.980

Variable	Estimate	Posterior S.D.	One-Tailed P-Value	95% C.I. Lower 2.5%	Upper 2.5%
S1	0.126	0.228	0.000	0.000	0.803

TECHNICAL 1 OUTPUT

PARAMETER SPECIFICATION FOR WITHIN

TAU

KESCAT16
0

NU

KESCAT16	PHY15	KES15	CHOICE15	BULLY15
0	0	0	0	0

NU

PRESS15	DUTIES15	PRAFACSQ
0	0	0

LAMBDA

	KESCAT16	PHY15	KES15	CHOICE15	BULLY15
KESCAT16	0	0	0	0	0
PHY15	0	0	0	0	0
KES15	0	0	0	0	0
CHOICE15	0	0	0	0	0
BULLY15	0	0	0	0	0
PRESS15	0	0	0	0	0

DUTIES15	0	0	0	0	0
PRAFACSQ	0	0	0	0	0

LAMBDA

	PRESS15	DUTIES15
KESCAT16	0	0
PHY15	0	0
KES15	0	0
CHOICE15	0	0
BULLY15	0	0
PRESS15	0	0
DUTIES15	0	0
PRAFACSQ	0	0

THETA

	KESCAT16	PHY15	KES15	CHOICE15	BULLY15
KESCAT16	0				
PHY15	0	0			
KES15	0	0	0		
CHOICE15	0	0	0	0	
BULLY15	0	0	0	0	0
PRESS15	0	0	0	0	0
DUTIES15	0	0	0	0	0
PRAFACSQ	0	0	0	0	0

THETA

	PRESS15	DUTIES15	PRAFACSQ
PRESS15	0		
DUTIES15	0	0	
PRAFACSQ	0	0	1

ALPHA

KESCAT16	PHY15	KES15	CHOICE15	BULLY15
0	2	3	0	4

ALPHA

PRESS15	DUTIES15
5	6

BETA

	KESCAT16	PHY15	KES15	CHOICE15	BULLY15
KESCAT16	0	7	8	0	9
PHY15	0	0	0	0	0
KES15	0	0	0	0	0
CHOICE15	0	0	0	0	0
BULLY15	0	0	0	0	0
PRESS15	0	0	0	0	0
DUTIES15	0	0	0	0	0

BETA

	PRESS15	DUTIES15
KESCAT16	10	11
PHY15	0	0
KES15	0	0
CHOICE15	0	0
BULLY15	0	0
PRESS15	0	0
DUTIES15	0	0

PSI

	KESCAT16	PHY15	KES15	CHOICE15	BULLY15
KESCAT16	0				
PHY15	0	12			
KES15	0	13	14		
CHOICE15	0	15	16	17	
BULLY15	0	18	19	20	21
PRESS15	0	22	23	24	25
DUTIES15	0	27	28	29	30

PSI

PRESS15	DUTIES15

PRESS15	26	
DUTIES15	31	32

PARAMETER SPECIFICATION FOR BETWEEN

TAU

KESCAT16
46

NU

KESCAT16	PRAFAC	CHOICE15	PRAFACSQ
0	0	0	33

LAMBDA

	S1	KESCAT16	PRAFAC	CHOICE15
KESCAT16	0	0	0	0
PRAFAC	0	0	0	0
CHOICE15	0	0	0	0
PRAFACSQ	0	0	0	0

THETA

	KESCAT16	PRAFAC	CHOICE15	PRAFACSQ
KESCAT16	0			
PRAFAC	0	0		
CHOICE15	0	0	0	
PRAFACSQ	0	0	0	34

ALPHA

S1	KESCAT16	PRAFAC	CHOICE15
35	0	36	37

BETA

	S1	KESCAT16	PRAFAC	CHOICE15
S1	0	0	38	0
KESCAT16	0	0	39	40
PRAFAC	0	0	0	0
CHOICE15	0	0	0	0

PSI

	S1	KESCAT16	PRAFAC	CHOICE15
S1	41			
KESCAT16	0	42		
PRAFAC	0	0	43	
CHOICE15	0	0	44	45

STARTING VALUES FOR WITHIN

TAU

KESCAT16
0.000

NU

KESCAT16	PHY15	KES15	CHOICE15	BULLY15
0.000	0.000	0.000	0.000	0.000

NU

PRESS15	DUTIES15	PRAFACSQ
0.000	0.000	0.000

LAMBDA

	KESCAT16	PHY15	KES15	CHOICE15	BULLY15
KESCAT16	1.000	0.000	0.000	0.000	0.000
PHY15	0.000	1.000	0.000	0.000	0.000

KES15	0.000	0.000	1.000	0.000	0.000
CHOICE15	0.000	0.000	0.000	1.000	0.000
BULLY15	0.000	0.000	0.000	0.000	1.000
PRESS15	0.000	0.000	0.000	0.000	0.000
DUTIES15	0.000	0.000	0.000	0.000	0.000
PRAFACSQ	0.000	0.000	0.000	0.000	0.000

LAMBDA

	PRESS15	DUTIES15
KESCAT16	0.000	0.000
PHY15	0.000	0.000
KES15	0.000	0.000
CHOICE15	0.000	0.000
BULLY15	0.000	0.000
PRESS15	1.000	0.000
DUTIES15	0.000	1.000
PRAFACSQ	0.000	0.000

THETA

	KESCAT16	PHY15	KES15	CHOICE15	BULLY15
KESCAT16	0.000				
PHY15	0.000	0.000			
KES15	0.000	0.000	0.000		
CHOICE15	0.000	0.000	0.000	0.000	
BULLY15	0.000	0.000	0.000	0.000	0.000
PRESS15	0.000	0.000	0.000	0.000	0.000
DUTIES15	0.000	0.000	0.000	0.000	0.000
PRAFACSQ	0.000	0.000	0.000	0.000	0.000

THETA

	PRESS15	DUTIES15	PRAFACSQ
PRESS15	0.000		
DUTIES15	0.000	0.000	
PRAFACSQ	0.000	0.000	0.654

ALPHA

	KESCAT16	PHY15	KES15	CHOICE15	BULLY15

	0.000	0.000	0.000	0.000	0.000

ALPHA

	PRESS15	DUTIES15
	0.000	0.000

BETA

	KESCAT16	PHY15	KES15	CHOICE15	BULLY15
KESCAT16	0.000	0.000	0.000	0.000	0.000
PHY15	0.000	0.000	0.000	0.000	0.000
KES15	0.000	0.000	0.000	0.000	0.000
CHOICE15	0.000	0.000	0.000	0.000	0.000
BULLY15	0.000	0.000	0.000	0.000	0.000
PRESS15	0.000	0.000	0.000	0.000	0.000
DUTIES15	0.000	0.000	0.000	0.000	0.000

BETA

	PRESS15	DUTIES15
KESCAT16	0.000	0.000
PHY15	0.000	0.000
KES15	0.000	0.000
CHOICE15	0.000	0.000
BULLY15	0.000	0.000
PRESS15	0.000	0.000
DUTIES15	0.000	0.000

PSI

	KESCAT16	PHY15	KES15	CHOICE15	BULLY15
KESCAT16	1.000				
PHY15	0.000	0.351			
KES15	0.000	0.000	0.157		
CHOICE15	0.000	0.000	0.000	0.597	
BULLY15	0.000	0.000	0.000	0.000	0.301
PRESS15	0.000	0.000	0.000	0.000	0.000
DUTIES15	0.000	0.000	0.000	0.000	0.000

PSI

	PRESS15	DUTIES15
PRESS15	0.580	
DUTIES15	0.000	0.516

STARTING VALUES FOR BETWEEN

TAU

KESCAT16
-0.352

NU

KESCAT16	PRAFAC	CHOICE15	PRAFACSQ
0.000	0.000	0.000	0.940

LAMBDA

	S1	KESCAT16	PRAFAC	CHOICE15
KESCAT16	0.000	1.000	0.000	0.000
PRAFAC	0.000	0.000	1.000	0.000
CHOICE15	0.000	0.000	0.000	1.000
PRAFACSQ	0.000	0.000	0.000	0.000

THETA

	KESCAT16	PRAFAC	CHOICE15	PRAFACSQ
KESCAT16	0.000			
PRAFAC	0.000	0.000		
CHOICE15	0.000	0.000	0.000	
PRAFACSQ	0.000	0.000	0.000	0.654

ALPHA

S1	KESCAT16	PRAFAC	CHOICE15

	0.000	0.000	-0.033	2.814

BETA

	S1	KESCAT16	PRAFAC	CHOICE15
S1	0.000	0.000	0.000	0.000
KESCAT16	0.000	0.000	0.000	0.000
PRAFAC	0.000	0.000	0.000	0.000
CHOICE15	0.000	0.000	0.000	0.000

PSI

	S1	KESCAT16	PRAFAC	CHOICE15
S1	1.000			
KESCAT16	0.000	1.000		
PRAFAC	0.000	0.000	0.469	
CHOICE15	0.000	0.000	0.000	0.597

PRIORS FOR ALL PARAMETERS	PRIOR MEAN	PRIOR VARIANCE	PRIOR STD. DEV.
Parameter 1~IG(-1.000,0.000)	infinity	infinity	infinity
Parameter 2~N(0.000,infinity)	0.0000	infinity	infinity
Parameter 3~N(0.000,infinity)	0.0000	infinity	infinity
Parameter 4~N(0.000,infinity)	0.0000	infinity	infinity
Parameter 5~N(0.000,infinity)	0.0000	infinity	infinity
Parameter 6~N(0.000,infinity)	0.0000	infinity	infinity
Parameter 7~N(0.000,5.000)	0.0000	5.0000	2.2361
Parameter 8~N(0.000,5.000)	0.0000	5.0000	2.2361
Parameter 9~N(0.000,5.000)	0.0000	5.0000	2.2361
Parameter 10~N(0.000,5.000)	0.0000	5.0000	2.2361
Parameter 11~N(0.000,5.000)	0.0000	5.0000	2.2361
Parameter 12~IW(1.000,7)	infinity	infinity	infinity
Parameter 13~IW(0.000,7)	infinity	infinity	infinity
Parameter 14~IW(1.000,7)	infinity	infinity	infinity
Parameter 15~IW(0.000,7)	infinity	infinity	infinity
Parameter 16~IW(0.000,7)	infinity	infinity	infinity
Parameter 17~IW(1.000,7)	infinity	infinity	infinity
Parameter 18~IW(0.000,7)	infinity	infinity	infinity
Parameter 19~IW(0.000,7)	infinity	infinity	infinity

```
     Parameter 20~IW(0.000,7)            infinity          infinity          infinity
     Parameter 21~IW(1.000,7)            infinity          infinity          infinity
     Parameter 22~IW(0.000,7)            infinity          infinity          infinity
     Parameter 23~IW(0.000,7)            infinity          infinity          infinity
     Parameter 24~IW(0.000,7)            infinity          infinity          infinity
     Parameter 25~IW(0.000,7)            infinity          infinity          infinity
     Parameter 26~IW(1.000,7)            infinity          infinity          infinity
     Parameter 27~IW(0.000,7)            infinity          infinity          infinity
     Parameter 28~IW(0.000,7)            infinity          infinity          infinity
     Parameter 29~IW(0.000,7)            infinity          infinity          infinity
     Parameter 30~IW(0.000,7)            infinity          infinity          infinity
     Parameter 31~IW(0.000,7)            infinity          infinity          infinity
     Parameter 32~IW(1.000,7)            infinity          infinity          infinity
     Parameter 33~N(0.000,infinity)        0.0000          infinity          infinity
     Parameter 34~IG(-1.000,0.000)       infinity          infinity          infinity
     Parameter 35~N(0.000,infinity)        0.0000          infinity          infinity
     Parameter 36~N(0.000,infinity)        0.0000          infinity          infinity
     Parameter 37~N(0.000,infinity)        0.0000          infinity          infinity
     Parameter 38~N(0.000,infinity)        0.0000          infinity          infinity
     Parameter 39~N(0.000,5.000)           0.0000            5.0000            2.2361
     Parameter 40~N(0.000,5.000)           0.0000            5.0000            2.2361
     Parameter 41~IG(-1.000,0.000)       infinity          infinity          infinity
     Parameter 42~IG(-1.000,0.000)       infinity          infinity          infinity
     Parameter 43~IW(1.000,3)            infinity          infinity          infinity
     Parameter 44~IW(0.000,3)            infinity          infinity          infinity
     Parameter 45~IW(1.000,3)            infinity          infinity          infinity
     Parameter 46~N(0.000,5.000)           0.0000            5.0000            2.2361


TECHNICAL 3 OUTPUT


           ESTIMATED COVARIANCE MATRIX FOR PARAMETER ESTIMATES
                  1             2             3             4             5
              ________      ________      ________      ________      ________
      1   0.957230D-10
      2  -0.664039D-08  0.629228D-03
      3   0.216579D-08  0.884824D-04  0.288158D-03
      4   0.268091D-08  0.304406D-04  0.464397D-04  0.553685D-03
      5  -0.873899D-08  0.106043D-03  0.554349D-04  0.173153D-03  0.107626D-02
      6   0.859295D-08 -0.631950D-04 -0.555116D-04 -0.148736D-03 -0.264012D-03
      7   0.659960D-08  0.987508D-05 -0.315784D-04 -0.263911D-04 -0.516110D-04
      8  -0.225367D-07  0.353103D-04  0.467997D-04 -0.348888D-04 -0.507809D-04
      9   0.382378D-08  0.217186D-04  0.147483D-04 -0.418669D-04 -0.289277D-04
```

10	-0.165376D-07	-0.432623D-04	0.159394D-04	-0.108233D-04	0.156815D-04
11	0.722984D-08	0.150407D-04	0.304700D-04	-0.153495D-05	0.545237D-04
12	-0.110998D-07	-0.291711D-05	0.527558D-05	0.566987D-04	0.434813D-04
13	-0.390960D-08	-0.375848D-05	0.698753D-05	0.544207D-05	0.367376D-05
14	0.351222D-09	0.564632D-05	-0.949084D-05	0.841332D-05	-0.693969D-05
15	-0.154089D-08	0.129351D-04	-0.238419D-04	-0.261034D-04	-0.152895D-04
16	-0.471352D-08	0.220481D-04	0.118158D-04	0.100847D-04	0.585724D-05
17	-0.721229D-08	-0.361154D-04	-0.387579D-04	-0.773781D-05	-0.378020D-04
18	-0.396381D-08	0.318483D-04	0.774566D-05	0.126678D-04	0.173877D-05
19	0.163541D-08	0.452549D-05	-0.997841D-05	-0.313943D-05	-0.832630D-05
20	0.124034D-08	-0.770210D-05	0.192945D-04	0.116270D-04	0.177790D-04
21	-0.538454D-08	0.698914D-05	0.839061D-05	0.598975D-05	0.106411D-04
22	-0.105015D-08	0.653039D-05	0.418219D-05	0.137086D-04	0.202542D-05
23	-0.228891D-09	-0.622413D-05	0.109590D-04	0.120152D-04	-0.977892D-05
24	-0.358596D-08	0.540271D-04	0.293784D-04	-0.136387D-04	-0.362993D-04
25	-0.357271D-08	0.183002D-04	-0.802836D-05	0.199816D-04	0.234143D-04
26	-0.139941D-07	0.218698D-04	-0.330844D-04	0.647755D-05	0.167317D-04
27	0.956986D-08	0.145112D-04	-0.117135D-04	-0.252073D-04	-0.384813D-04
28	0.518375D-09	0.390253D-05	-0.263267D-05	0.158768D-05	0.191530D-04
29	-0.892394D-08	-0.183689D-04	-0.182325D-05	0.440206D-04	0.220734D-04
30	0.122756D-08	-0.556039D-05	-0.945735D-05	-0.112605D-04	0.253322D-04
31	0.479605D-09	-0.107433D-04	0.176254D-04	0.546848D-05	0.352628D-05
32	-0.166202D-07	-0.108479D-04	-0.550899D-05	0.182403D-04	-0.376488D-04
33	-0.851971D-07	0.288355D-04	-0.313782D-04	-0.905323D-05	0.785167D-04
34	-0.210344D-06	0.550648D-04	-0.700037D-05	0.428667D-04	0.352089D-03
35	-0.245944D-07	-0.505615D-04	0.794157D-05	0.348442D-05	0.429611D-05
36	-0.482900D-07	0.453308D-04	0.334686D-04	-0.152476D-03	-0.140507D-03
37	-0.272394D-08	-0.165860D-04	-0.392947D-04	-0.148059D-03	-0.132559D-03
38	-0.109833D-07	0.133315D-05	-0.255180D-04	-0.144854D-04	-0.163166D-04
39	0.159462D-07	0.174864D-04	0.201073D-05	0.369440D-04	-0.321390D-04
40	0.371327D-08	0.487440D-04	0.569121D-04	-0.518513D-04	-0.446872D-03
41	0.941224D-09	-0.993372D-06	0.932767D-05	-0.127134D-04	-0.192305D-04
42	-0.902892D-08	-0.180286D-05	0.881685D-06	-0.227899D-04	0.375172D-05
43	-0.318534D-07	0.292605D-04	-0.179618D-03	-0.134983D-03	0.421036D-03
44	-0.126902D-07	-0.543234D-04	-0.406255D-04	0.788020D-05	-0.492678D-04
45	-0.684764D-08	0.139255D-04	0.730140D-05	-0.250800D-05	-0.280589D-04
46	0.218793D-07	0.143410D-03	0.151942D-03	-0.108002D-03	-0.115784D-02

ESTIMATED COVARIANCE MATRIX FOR PARAMETER ESTIMATES

	6	7	8	9	10
6	0.955064D-03				
7	0.405905D-04	0.275479D-02			

8	-0.439131D-04	-0.363649D-03	0.873635D-02		
9	0.132038D-04	0.154250D-03	0.885664D-05	0.376425D-02	
10	0.322234D-04	-0.297295D-03	-0.289373D-03	-0.401455D-03	0.178594D-02
11	-0.332474D-04	-0.334410D-04	0.158616D-03	0.340152D-03	0.321242D-03
12	-0.258797D-04	0.333018D-04	-0.163353D-03	0.653735D-05	-0.857466D-05
13	0.638233D-06	-0.203628D-04	-0.317145D-04	-0.370922D-04	0.139521D-04
14	0.158355D-04	0.247858D-04	0.299656D-04	0.164193D-04	0.712699D-05
15	-0.192248D-04	0.234142D-05	0.133292D-04	-0.414858D-04	0.321429D-04
16	-0.272571D-04	0.479490D-04	0.398896D-06	-0.162027D-05	-0.227654D-04
17	0.161630D-04	-0.125062D-04	0.284563D-03	0.503845D-04	0.578429D-04
18	0.228978D-04	-0.270929D-04	0.268032D-04	-0.277775D-05	-0.171694D-04
19	0.104333D-04	-0.420271D-04	-0.129351D-04	0.224136D-04	0.847752D-05
20	0.272783D-05	0.919851D-04	-0.845971D-04	0.424997D-04	-0.215633D-04
21	0.210401D-04	-0.808373D-05	-0.225336D-04	-0.126939D-04	0.472623D-05
22	-0.588912D-05	0.394658D-06	-0.103114D-03	-0.761028D-05	-0.470299D-04
23	-0.130468D-04	-0.379493D-04	-0.177893D-04	0.408308D-04	0.546094D-05
24	0.835859D-05	0.516115D-04	0.161103D-03	0.720585D-05	-0.615283D-04
25	-0.799661D-06	-0.252542D-04	-0.335590D-04	0.435956D-05	0.925254D-05
26	0.935847D-05	-0.998678D-04	-0.178783D-03	-0.348450D-04	-0.839035D-04
27	0.311264D-05	0.617248D-05	0.124686D-04	-0.137709D-04	0.351752D-04
28	0.263159D-05	0.348339D-05	-0.100958D-04	-0.377517D-04	0.115387D-04
29	-0.131728D-04	-0.217298D-04	-0.461449D-04	-0.256159D-05	0.519544D-04
30	0.157999D-04	0.274244D-04	-0.754695D-05	0.341741D-04	0.543950D-04
31	0.286131D-04	0.110875D-04	0.773851D-04	0.402016D-04	0.812682D-04
32	-0.729522D-04	0.169729D-04	-0.145860D-04	-0.155718D-04	-0.550018D-04
33	-0.186574D-03	0.279297D-03	0.402305D-03	-0.136216D-04	0.246773D-04
34	-0.148941D-03	-0.769407D-03	0.923388D-03	0.256001D-03	0.279617D-03
35	-0.313029D-04	0.153199D-03	0.315573D-03	0.275655D-03	0.178767D-03
36	0.103274D-03	0.267538D-03	0.181084D-03	0.117821D-03	0.323245D-03
37	0.114851D-03	0.866581D-04	-0.177605D-03	0.140021D-03	-0.147930D-04
38	-0.162973D-04	0.487622D-04	-0.119120D-03	-0.359271D-04	0.712204D-04
39	-0.285358D-05	0.230196D-04	0.435064D-04	-0.128013D-03	-0.214925D-03
40	0.465477D-04	-0.245511D-03	0.462980D-03	0.102198D-02	0.622064D-03
41	0.620363D-05	0.684293D-04	0.132672D-03	0.118089D-05	-0.167433D-04
42	0.979955D-05	0.958034D-04	0.283134D-03	-0.403368D-04	0.719478D-04
43	0.146503D-03	0.345850D-03	-0.137368D-02	-0.759844D-03	-0.154899D-03
44	0.524919D-04	0.146388D-03	-0.586626D-04	-0.277852D-04	-0.948205D-06
45	0.253969D-06	0.642290D-04	-0.435068D-04	0.273057D-04	0.107741D-04
46	0.107154D-03	-0.808939D-03	0.423965D-03	0.265743D-02	0.181802D-02

ESTIMATED COVARIANCE MATRIX FOR PARAMETER ESTIMATES

11	12	13	14	15

11	0.186092D-02				
12	0.269582D-05	0.894891D-03			
13	-0.322080D-04	0.121150D-03	0.200756D-03		
14	0.707724D-05	0.215400D-04	0.607770D-04	0.174564D-03	
15	-0.218380D-04	-0.935001D-04	-0.479233D-04	-0.120248D-04	0.740938D-03
16	0.250089D-04	-0.219881D-04	-0.309143D-04	-0.535273D-04	0.132453D-03
17	0.221277D-04	-0.441322D-04	-0.177391D-04	0.104688D-04	-0.200954D-03
18	0.547313D-05	0.422593D-04	0.533218D-04	0.133223D-04	-0.782100D-04
19	-0.144563D-05	0.199150D-04	0.184623D-04	0.410720D-04	-0.144561D-04
20	0.112354D-05	0.725023D-05	0.326066D-06	-0.203824D-04	0.239059D-04
21	-0.263537D-04	0.613971D-05	-0.724646D-05	-0.887036D-05	-0.108669D-04
22	-0.805538D-05	0.123938D-03	0.542573D-04	0.230110D-04	-0.129075D-03
23	0.229192D-05	0.434399D-04	0.373793D-04	0.431344D-04	-0.214111D-04
24	0.164200D-04	-0.188416D-04	-0.102173D-05	-0.649927D-05	0.161134D-03
25	0.122303D-04	0.240655D-04	0.595827D-05	0.110878D-04	-0.782506D-05
26	-0.371387D-04	0.846653D-04	0.764486D-05	0.226899D-04	-0.599018D-04
27	0.199667D-04	-0.113639D-03	-0.569437D-04	-0.226913D-04	0.112144D-03
28	0.255419D-04	-0.110256D-04	-0.227307D-04	-0.433181D-04	0.143356D-04
29	-0.636039D-04	0.208214D-04	-0.769378D-05	-0.873075D-05	-0.931971D-04
30	0.141225D-04	-0.132485D-05	-0.105717D-04	-0.149462D-04	0.833642D-05
31	0.318538D-04	0.175401D-04	0.817789D-05	-0.181564D-04	0.138701D-04
32	-0.106585D-03	0.198479D-04	0.531307D-06	0.363333D-05	-0.178968D-04
33	-0.370368D-03	0.105280D-03	-0.181922D-04	0.245803D-04	0.111038D-03
34	0.629621D-04	-0.240197D-04	-0.844492D-04	0.768057D-04	0.472060D-03
35	-0.151149D-03	-0.381868D-05	-0.563640D-05	0.868617D-05	0.367844D-04
36	-0.344682D-03	-0.409026D-04	-0.655839D-04	-0.477419D-04	0.614697D-04
37	0.373147D-04	-0.521690D-04	-0.107210D-04	0.169928D-04	0.149027D-03
38	-0.244329D-04	0.198491D-04	-0.285852D-05	0.320009D-04	-0.504694D-05
39	-0.424462D-04	-0.706243D-05	0.312439D-04	0.130613D-04	-0.125312D-03
40	0.553672D-03	0.879134D-04	-0.943416D-04	0.103864D-03	-0.128524D-03
41	-0.476285D-04	-0.280665D-04	-0.110756D-04	0.254408D-05	0.197044D-04
42	0.733719D-04	-0.854269D-05	-0.261619D-04	0.323983D-05	0.300076D-04
43	0.363826D-04	0.550379D-03	0.584485D-04	-0.297826D-04	-0.330897D-03
44	-0.209675D-04	0.235879D-04	0.695345D-04	-0.343090D-05	-0.526608D-04
45	-0.269459D-04	0.238612D-04	0.191169D-04	-0.176967D-05	0.210153D-04
46	0.170436D-02	0.331881D-03	-0.260318D-03	0.305658D-03	-0.359828D-03

ESTIMATED COVARIANCE MATRIX FOR PARAMETER ESTIMATES

	16	17	18	19	20
16	0.334950D-03				
17	-0.228593D-03	0.252320D-02			
18	-0.646462D-05	0.804519D-05	0.376772D-03		

19	-0.418968D-04	0.188922D-04	0.657365D-04	0.172243D-03	
20	0.665902D-04	-0.289590D-03	-0.487249D-04	-0.578132D-04	0.650572D-03
21	-0.690917D-05	0.132560D-04	0.607484D-04	0.715329D-04	-0.145311D-03
22	-0.221195D-04	0.200653D-04	0.122040D-03	0.281525D-04	-0.148566D-05
23	-0.406171D-04	0.279272D-04	0.354457D-04	0.634553D-04	-0.145889D-04
24	0.804685D-04	-0.396915D-03	-0.451970D-04	-0.330225D-04	0.226093D-03
25	-0.168137D-04	0.309693D-04	0.111109D-03	0.497539D-04	-0.131322D-03
26	-0.138115D-04	0.406952D-04	0.114575D-03	0.478887D-04	-0.361931D-04
27	0.527478D-04	-0.217205D-04	-0.119321D-03	-0.364979D-04	0.228277D-04
28	0.504688D-04	-0.559464D-04	-0.113362D-04	-0.507834D-04	0.139289D-04
29	-0.868179D-04	0.212110D-03	0.112207D-04	0.185194D-04	-0.178121D-03
30	0.164042D-04	-0.261965D-05	-0.615111D-04	-0.564071D-04	0.110521D-03
31	0.341681D-04	0.169958D-04	-0.647080D-04	-0.289333D-04	0.676667D-04
32	-0.233778D-04	0.420369D-04	0.345635D-04	0.239980D-04	-0.462815D-04
33	0.145835D-03	-0.330900D-03	-0.305941D-04	-0.264240D-04	-0.136985D-04
34	0.558000D-03	0.657472D-03	0.168146D-03	0.452760D-04	0.334590D-03
35	-0.152989D-04	-0.151357D-04	0.691208D-05	-0.129489D-04	0.146987D-04
36	0.713414D-04	-0.125544D-05	0.433348D-04	0.639554D-04	-0.136954D-04
37	0.355249D-04	-0.661569D-04	0.296496D-04	0.361207D-04	-0.479972D-04
38	0.180465D-04	0.100023D-03	0.131213D-04	-0.107820D-04	-0.374467D-04
39	-0.116773D-04	0.137022D-03	0.227822D-04	0.364106D-04	-0.285756D-04
40	-0.196645D-03	-0.189485D-03	0.170993D-03	0.735791D-05	-0.158952D-03
41	0.194507D-04	0.856783D-05	0.604557D-05	-0.273804D-05	0.885848D-05
42	0.225497D-04	-0.419044D-05	0.122168D-04	-0.189283D-04	-0.330107D-04
43	0.227170D-04	0.402528D-03	0.645352D-04	0.123266D-04	-0.123700D-03
44	0.293348D-04	-0.337895D-04	0.375149D-04	0.784878D-05	0.579231D-05
45	-0.418305D-05	-0.476157D-04	-0.990509D-05	0.100662D-04	0.128873D-04
46	-0.563057D-03	-0.634270D-03	0.461498D-03	0.381934D-04	-0.442660D-03

ESTIMATED COVARIANCE MATRIX FOR PARAMETER ESTIMATES

	21	22	23	24	25
21	0.669561D-03				
22	0.368747D-04	0.740072D-03			
23	0.863324D-05	0.120335D-03	0.350610D-03		
24	-0.526491D-04	-0.128147D-03	-0.115858D-03	0.130056D-02	
25	0.245097D-03	0.884866D-04	0.948820D-04	-0.158958D-03	0.690267D-03
26	0.917425D-04	0.282566D-03	0.142889D-03	-0.388922D-03	0.422829D-03
27	-0.396950D-04	-0.218266D-03	-0.346210D-04	0.723032D-04	-0.650132D-04
28	-0.225303D-04	-0.331614D-04	-0.783762D-04	0.244104D-04	-0.324998D-04
29	0.494517D-04	0.574363D-04	0.249030D-04	-0.330549D-03	0.596335D-04
30	-0.184457D-03	-0.376754D-04	-0.400788D-04	0.595545D-04	-0.199012D-03
31	-0.654725D-04	-0.106487D-03	-0.113101D-03	0.184105D-03	-0.249419D-03

32	0.329576D-04	0.362660D-04	0.575388D-04	-0.488007D-04	0.297848D-04
33	-0.189913D-03	-0.137863D-03	-0.454061D-04	0.374174D-03	-0.167563D-03
34	0.420047D-03	-0.199192D-03	-0.439356D-03	0.496358D-03	0.437901D-04
35	0.210325D-04	-0.130104D-04	-0.443698D-04	0.424705D-04	0.172297D-04
36	0.252348D-03	-0.413722D-04	-0.771859D-04	0.392143D-03	0.293633D-03
37	0.176087D-04	-0.814397D-05	-0.410257D-04	0.161621D-03	0.847897D-05
38	0.277230D-05	0.148259D-04	0.218793D-04	-0.200737D-04	0.467596D-05
39	-0.957996D-05	0.180894D-04	0.532939D-05	-0.298962D-04	-0.137429D-04
40	0.107501D-03	0.250946D-03	-0.731867D-04	0.189811D-03	0.555330D-04
41	0.103681D-04	-0.222172D-04	-0.127021D-04	0.183291D-04	-0.312151D-05
42	0.106560D-04	0.101128D-05	0.942245D-06	-0.236079D-04	0.184331D-04
43	0.249422D-03	0.290421D-03	0.162331D-04	-0.189551D-03	-0.100927D-03
44	-0.161904D-04	0.830587D-04	0.234470D-04	-0.112329D-03	-0.798870D-05
45	0.147017D-04	0.189461D-05	0.155902D-05	0.728031D-05	-0.565268D-04
46	0.290933D-03	0.754583D-03	-0.178775D-03	0.460916D-03	0.163465D-03

ESTIMATED COVARIANCE MATRIX FOR PARAMETER ESTIMATES

	26	27	28	29	30
26	0.247177D-02				
27	-0.125471D-03	0.729007D-03			
28	-0.410402D-04	0.127854D-03	0.282436D-03		
29	0.100043D-03	-0.132379D-03	-0.985677D-04	0.112464D-02	
30	-0.147570D-03	0.106218D-03	0.935383D-04	-0.157134D-03	0.594801D-03
31	-0.626073D-03	0.166643D-03	0.946770D-04	-0.211731D-03	0.301362D-03
32	0.151938D-03	-0.148725D-03	-0.134952D-03	0.224274D-03	-0.341932D-03
33	-0.428173D-03	-0.683280D-04	0.869983D-04	0.167820D-03	0.138267D-03
34	-0.729216D-03	0.162032D-03	0.237213D-04	-0.128459D-03	0.201051D-03
35	-0.335296D-04	-0.158138D-04	-0.178091D-04	-0.559333D-04	-0.865806D-05
36	-0.183398D-03	0.113590D-03	-0.286269D-05	0.760895D-04	0.275783D-04
37	-0.781005D-04	0.132245D-04	-0.482413D-04	-0.192459D-04	-0.155817D-04
38	0.213437D-04	0.264703D-04	-0.258499D-04	-0.788291D-05	-0.467466D-05
39	-0.322680D-06	-0.662582D-04	0.640489D-06	-0.457322D-04	0.157156D-04
40	-0.233715D-03	-0.132556D-03	-0.124860D-03	0.495204D-03	0.176828D-04
41	-0.183173D-04	0.330770D-04	0.504674D-05	-0.321505D-05	0.611489D-05
42	-0.673145D-04	-0.134196D-04	0.129609D-05	0.348471D-04	0.642281D-05
43	-0.109835D-03	-0.192090D-03	-0.629674D-04	-0.252842D-03	-0.519384D-04
44	0.197800D-03	0.407013D-05	0.123786D-04	0.346082D-04	-0.171145D-04
45	-0.190514D-04	-0.542421D-05	0.409420D-05	0.308046D-04	0.201740D-04
46	-0.676611D-03	-0.362919D-03	-0.366230D-03	0.148113D-02	0.442504D-05

ESTIMATED COVARIANCE MATRIX FOR PARAMETER ESTIMATES

	31	32	33	34	35
31	0.117682D-02				
32	-0.533645D-03	0.188830D-02			
33	0.155636D-03	0.827053D-04	0.448678D-01		
34	0.348264D-03	0.392861D-03	0.340469D-02	0.269790D+00	
35	-0.521914D-05	-0.569046D-04	-0.167237D-03	0.586188D-03	0.262704D-02
36	0.232891D-03	-0.937830D-04	0.741202D-03	0.317991D-03	0.296315D-03
37	-0.349865D-04	0.661572D-04	0.381174D-04	-0.527958D-03	0.179574D-03
38	-0.133579D-04	0.332876D-04	-0.409117D-04	0.234355D-03	0.112465D-03
39	0.567611D-04	0.566280D-05	0.290306D-03	-0.555699D-03	0.153012D-03
40	0.515531D-04	-0.979940D-04	-0.104629D-02	-0.481495D-02	-0.114861D-02
41	0.236419D-04	-0.129594D-04	0.133566D-03	-0.268468D-03	0.205646D-04
42	-0.128962D-04	0.212556D-04	0.151671D-03	-0.311920D-03	-0.331819D-04
43	-0.973799D-04	0.330058D-03	-0.150816D-02	-0.258560D-02	-0.243932D-03
44	0.252585D-05	0.105211D-03	-0.166623D-03	-0.612560D-03	-0.118219D-03
45	0.367956D-05	0.446574D-04	-0.880989D-04	0.139054D-03	0.539782D-04
46	0.155467D-03	-0.193761D-03	-0.306763D-02	-0.134536D-01	-0.327398D-02

ESTIMATED COVARIANCE MATRIX FOR PARAMETER ESTIMATES

	36	37	38	39	40
36	0.234002D-01				
37	0.159050D-02	0.387307D-02			
38	-0.229365D-03	0.600923D-04	0.243347D-02		
39	-0.442812D-04	0.547536D-04	-0.806527D-04	0.318799D-02	
40	-0.466958D-03	0.662226D-03	-0.108503D-02	-0.445418D-02	0.720937D-01
41	0.231125D-04	0.169421D-04	0.695982D-04	-0.714889D-04	0.286272D-04
42	-0.762682D-04	0.848191D-04	0.197695D-04	-0.210418D-03	0.912735D-03
43	-0.273758D-03	-0.124174D-03	0.325285D-03	-0.116603D-03	-0.104203D-02
44	-0.151112D-03	-0.196406D-03	0.272566D-03	0.635754D-03	-0.159271D-02
45	-0.629355D-04	0.228952D-03	0.330617D-04	0.819796D-04	0.106381D-03
46	-0.160942D-02	0.149626D-02	-0.312689D-02	-0.129124D-01	0.204419D+00

ESTIMATED COVARIANCE MATRIX FOR PARAMETER ESTIMATES

	41	42	43	44	45
41	0.336097D-03				
42	0.441370D-04	0.818420D-03			
43	0.298436D-04	0.580674D-04	0.693152D-01		
44	-0.224047D-04	-0.365963D-04	0.509202D-02	0.583473D-02	
45	0.709436D-05	-0.273283D-04	0.449573D-03	0.844219D-03	0.914010D-03

46   0.121081D-04 0.246325D-02 -0.312642D-02 -0.469970D-02 0.234949D-03

ESTIMATED COVARIANCE MATRIX FOR PARAMETER ESTIMATES

	46
46	0.582729D+00

ESTIMATED CORRELATION MATRIX FOR PARAMETER ESTIMATES

	1	2	3	4	5
1	1.000				
2	-0.027	1.000			
3	0.013	0.208	1.000		
4	0.012	0.052	0.116	1.000	
5	-0.027	0.129	0.100	0.224	1.000
6	0.028	-0.082	-0.106	-0.205	-0.260
7	0.013	0.008	-0.035	-0.021	-0.030
8	-0.025	0.015	0.029	-0.016	-0.017
9	0.006	0.014	0.014	-0.029	-0.014
10	-0.040	-0.041	0.022	-0.011	0.011
11	0.017	0.014	0.042	-0.002	0.039
12	-0.038	-0.004	0.010	0.081	0.044
13	-0.028	-0.011	0.029	0.016	0.008
14	0.003	0.017	-0.042	0.027	-0.016
15	-0.006	0.019	-0.052	-0.041	-0.017
16	-0.026	0.048	0.038	0.023	0.010
17	-0.015	-0.029	-0.045	-0.007	-0.023
18	-0.021	0.065	0.024	0.028	0.003
19	0.013	0.014	-0.045	-0.010	-0.019
20	0.005	-0.012	0.045	0.019	0.021
21	-0.021	0.011	0.019	0.010	0.013
22	-0.004	0.010	0.009	0.021	0.002
23	-0.001	-0.013	0.034	0.027	-0.016
24	-0.010	0.060	0.048	-0.016	-0.031
25	-0.014	0.028	-0.018	0.032	0.027
26	-0.029	0.018	-0.039	0.006	0.010
27	0.036	0.021	-0.026	-0.040	-0.043
28	0.003	0.009	-0.009	0.004	0.035
29	-0.027	-0.022	-0.003	0.056	0.020
30	0.005	-0.009	-0.023	-0.020	0.032
31	0.001	-0.012	0.030	0.007	0.003
32	-0.039	-0.010	-0.007	0.018	-0.026

33	-0.041	0.005	-0.009	-0.002	0.011
34	-0.041	0.004	-0.001	0.004	0.021
35	-0.049	-0.039	0.009	0.003	0.003
36	-0.032	0.012	0.013	-0.042	-0.028
37	-0.004	-0.011	-0.037	-0.101	-0.065
38	-0.023	0.001	-0.030	-0.012	-0.010
39	0.029	0.012	0.002	0.028	-0.017
40	0.001	0.007	0.012	-0.008	-0.051
41	0.005	-0.002	0.030	-0.029	-0.032
42	-0.032	-0.003	0.002	-0.034	0.004
43	-0.012	0.004	-0.040	-0.022	0.049
44	-0.017	-0.028	-0.031	0.004	-0.020
45	-0.023	0.018	0.014	-0.004	-0.028
46	0.003	0.007	0.012	-0.006	-0.046

ESTIMATED CORRELATION MATRIX FOR PARAMETER ESTIMATES

	6	7	8	9	10
6	1.000				
7	0.025	1.000			
8	-0.015	-0.074	1.000		
9	0.007	0.048	0.002	1.000	
10	0.025	-0.134	-0.073	-0.155	1.000
11	-0.025	-0.015	0.039	0.129	0.176
12	-0.028	0.021	-0.058	0.004	-0.007
13	0.001	-0.027	-0.024	-0.043	0.023
14	0.039	0.036	0.024	0.020	0.013
15	-0.023	0.002	0.005	-0.025	0.028
16	-0.048	0.050	0.000	-0.001	-0.029
17	0.010	-0.005	0.061	0.016	0.027
18	0.038	-0.027	0.015	-0.002	-0.021
19	0.026	-0.061	-0.011	0.028	0.015
20	0.003	0.069	-0.035	0.027	-0.020
21	0.026	-0.006	-0.009	-0.008	0.004
22	-0.007	0.000	-0.041	-0.005	-0.041
23	-0.023	-0.039	-0.010	0.036	0.007
24	0.007	0.027	0.048	0.003	-0.040
25	-0.001	-0.018	-0.014	0.003	0.008
26	0.006	-0.038	-0.038	-0.011	-0.040
27	0.004	0.004	0.005	-0.008	0.031
28	0.005	0.004	-0.006	-0.037	0.016
29	-0.013	-0.012	-0.015	-0.001	0.037
30	0.021	0.021	-0.003	0.023	0.053

31	0.027	0.006	0.024	0.019	0.056
32	-0.054	0.007	-0.004	-0.006	-0.030
33	-0.029	0.025	0.020	-0.001	0.003
34	-0.009	-0.028	0.019	0.008	0.013
35	-0.020	0.057	0.066	0.088	0.083
36	0.022	0.033	0.013	0.013	0.050
37	0.060	0.027	-0.031	0.037	-0.006
38	-0.011	0.019	-0.026	-0.012	0.034
39	-0.002	0.008	0.008	-0.037	-0.090
40	0.006	-0.017	0.018	0.062	0.055
41	0.011	0.071	0.077	0.001	-0.022
42	0.011	0.064	0.106	-0.023	0.060
43	0.018	0.025	-0.056	-0.047	-0.014
44	0.022	0.037	-0.008	-0.006	0.000
45	0.000	0.040	-0.015	0.015	0.008
46	0.005	-0.020	0.006	0.057	0.056

ESTIMATED CORRELATION MATRIX FOR PARAMETER ESTIMATES

	11	12	13	14	15
11	1.000				
12	0.002	1.000			
13	-0.053	0.286	1.000		
14	0.012	0.054	0.325	1.000	
15	-0.019	-0.115	-0.124	-0.033	1.000
16	0.032	-0.040	-0.119	-0.221	0.266
17	0.010	-0.029	-0.025	0.016	-0.147
18	0.007	0.073	0.194	0.052	-0.148
19	-0.003	0.051	0.099	0.237	-0.040
20	0.001	0.010	0.001	-0.060	0.034
21	-0.024	0.008	-0.020	-0.026	-0.015
22	-0.007	0.152	0.141	0.064	-0.174
23	0.003	0.078	0.141	0.174	-0.042
24	0.011	-0.017	-0.002	-0.014	0.164
25	0.011	0.031	0.016	0.032	-0.011
26	-0.017	0.057	0.011	0.035	-0.044
27	0.017	-0.141	-0.149	-0.064	0.153
28	0.035	-0.022	-0.095	-0.195	0.031
29	-0.044	0.021	-0.016	-0.020	-0.102
30	0.013	-0.002	-0.031	-0.046	0.013
31	0.022	0.017	0.017	-0.040	0.015
32	-0.057	0.015	0.001	0.006	-0.015
33	-0.041	0.017	-0.006	0.009	0.019

34	0.003	-0.002	-0.011	0.011	0.033
35	-0.068	-0.002	-0.008	0.013	0.026
36	-0.052	-0.009	-0.030	-0.024	0.015
37	0.014	-0.028	-0.012	0.021	0.088
38	-0.011	0.013	-0.004	0.049	-0.004
39	-0.017	-0.004	0.039	0.018	-0.082
40	0.048	0.011	-0.025	0.029	-0.018
41	-0.060	-0.051	-0.043	0.011	0.039
42	0.059	-0.010	-0.065	0.009	0.039
43	0.003	0.070	0.016	-0.009	-0.046
44	-0.006	0.010	0.064	-0.003	-0.025
45	-0.021	0.026	0.045	-0.004	0.026
46	0.052	0.015	-0.024	0.030	-0.017

ESTIMATED CORRELATION MATRIX FOR PARAMETER ESTIMATES

	16	17	18	19	20
16	1.000				
17	-0.249	1.000			
18	-0.018	0.008	1.000		
19	-0.174	0.029	0.258	1.000	
20	0.143	-0.226	-0.098	-0.173	1.000
21	-0.015	0.010	0.121	0.211	-0.220
22	-0.044	0.015	0.231	0.079	-0.002
23	-0.119	0.030	0.098	0.258	-0.031
24	0.122	-0.219	-0.065	-0.070	0.246
25	-0.035	0.023	0.218	0.144	-0.196
26	-0.015	0.016	0.119	0.073	-0.029
27	0.107	-0.016	-0.228	-0.103	0.033
28	0.164	-0.066	-0.035	-0.230	0.032
29	-0.141	0.126	0.017	0.042	-0.208
30	0.037	-0.002	-0.130	-0.176	0.178
31	0.054	0.010	-0.097	-0.064	0.077
32	-0.029	0.019	0.041	0.042	-0.042
33	0.038	-0.031	-0.007	-0.010	-0.003
34	0.059	0.025	0.017	0.007	0.025
35	-0.016	-0.006	0.007	-0.019	0.011
36	0.025	0.000	0.015	0.032	-0.004
37	0.031	-0.021	0.025	0.044	-0.030
38	0.020	0.040	0.014	-0.017	-0.030
39	-0.011	0.048	0.021	0.049	-0.020
40	-0.040	-0.014	0.033	0.002	-0.023
41	0.058	0.009	0.017	-0.011	0.019

42	0.043	-0.003	0.022	-0.050	-0.045
43	0.005	0.030	0.013	0.004	-0.018
44	0.021	-0.009	0.025	0.008	0.003
45	-0.008	-0.031	-0.017	0.025	0.017
46	-0.040	-0.017	0.031	0.004	-0.023

ESTIMATED CORRELATION MATRIX FOR PARAMETER ESTIMATES

	21	22	23	24	25
21	1.000				
22	0.052	1.000			
23	0.018	0.236	1.000		
24	-0.056	-0.131	-0.172	1.000	
25	0.361	0.124	0.193	-0.168	1.000
26	0.071	0.209	0.153	-0.217	0.324
27	-0.057	-0.297	-0.068	0.074	-0.092
28	-0.052	-0.073	-0.249	0.040	-0.074
29	0.057	0.063	0.040	-0.273	0.068
30	-0.292	-0.057	-0.088	0.068	-0.311
31	-0.074	-0.114	-0.176	0.149	-0.277
32	0.029	0.031	0.071	-0.031	0.026
33	-0.035	-0.024	-0.011	0.049	-0.030
34	0.031	-0.014	-0.045	0.026	0.003
35	0.016	-0.009	-0.046	0.023	0.013
36	0.064	-0.010	-0.027	0.071	0.073
37	0.011	-0.005	-0.035	0.072	0.005
38	0.002	0.011	0.024	-0.011	0.004
39	-0.007	0.012	0.005	-0.015	-0.009
40	0.015	0.034	-0.015	0.020	0.008
41	0.022	-0.045	-0.037	0.028	-0.006
42	0.014	0.001	0.002	-0.023	0.025
43	0.037	0.041	0.003	-0.020	-0.015
44	-0.008	0.040	0.016	-0.041	-0.004
45	0.019	0.002	0.003	0.007	-0.071
46	0.015	0.036	-0.013	0.017	0.008

ESTIMATED CORRELATION MATRIX FOR PARAMETER ESTIMATES

	26	27	28	29	30
26	1.000				
27	-0.093	1.000			
28	-0.049	0.282	1.000		

29	0.060	-0.146	-0.175	1.000	
30	-0.122	0.161	0.228	-0.192	1.000
31	-0.367	0.180	0.164	-0.184	0.360
32	0.070	-0.127	-0.185	0.154	-0.323
33	-0.041	-0.012	0.024	0.024	0.027
34	-0.028	0.012	0.003	-0.007	0.016
35	-0.013	-0.011	-0.021	-0.033	-0.007
36	-0.024	0.028	-0.001	0.015	0.007
37	-0.025	0.008	-0.046	-0.009	-0.010
38	0.009	0.020	-0.031	-0.005	-0.004
39	0.000	-0.043	0.001	-0.024	0.011
40	-0.018	-0.018	-0.028	0.055	0.003
41	-0.020	0.067	0.016	-0.005	0.014
42	-0.047	-0.017	0.003	0.036	0.009
43	-0.008	-0.027	-0.014	-0.029	-0.008
44	0.052	0.002	0.010	0.014	-0.009
45	-0.013	-0.007	0.008	0.030	0.027
46	-0.018	-0.018	-0.029	0.058	0.000

ESTIMATED CORRELATION MATRIX FOR PARAMETER ESTIMATES

	31	32	33	34	35
31	1.000				
32	-0.358	1.000			
33	0.021	0.009	1.000		
34	0.020	0.017	0.031	1.000	
35	-0.003	-0.026	-0.015	0.022	1.000
36	0.044	-0.014	0.023	0.004	0.038
37	-0.016	0.024	0.003	-0.016	0.056
38	-0.008	0.016	-0.004	0.009	0.044
39	0.029	0.002	0.024	-0.019	0.053
40	0.006	-0.008	-0.018	-0.035	-0.083
41	0.038	-0.016	0.034	-0.028	0.022
42	-0.013	0.017	0.025	-0.021	-0.023
43	-0.011	0.029	-0.027	-0.019	-0.018
44	0.001	0.032	-0.010	-0.015	-0.030
45	0.004	0.034	-0.014	0.009	0.035
46	0.006	-0.006	-0.019	-0.034	-0.084

ESTIMATED CORRELATION MATRIX FOR PARAMETER ESTIMATES

	36	37	38	39	40

36	1.000				
37	0.167	1.000			
38	-0.030	0.020	1.000		
39	-0.005	0.016	-0.029	1.000	
40	-0.011	0.040	-0.082	-0.294	1.000
41	0.008	0.015	0.077	-0.069	0.006
42	-0.017	0.048	0.014	-0.130	0.119
43	-0.007	-0.008	0.025	-0.008	-0.015
44	-0.013	-0.041	0.072	0.147	-0.078
45	-0.014	0.122	0.022	0.048	0.013
46	-0.014	0.031	-0.083	-0.300	0.997

ESTIMATED CORRELATION MATRIX FOR PARAMETER ESTIMATES

	41	42	43	44	45
41	1.000				
42	0.084	1.000			
43	0.006	0.008	1.000		
44	-0.016	-0.017	0.253	1.000	
45	0.013	-0.032	0.056	0.366	1.000
46	0.001	0.113	-0.016	-0.081	0.010

ESTIMATED CORRELATION MATRIX FOR PARAMETER ESTIMATES

	46
46	1.000

TECHNICAL 8 OUTPUT

TECHNICAL 8 OUTPUT FOR BAYES ESTIMATION

CHAIN	BSEED
1	0
2	285380
3	253358

ITERATION	POTENTIAL SCALE REDUCTION	PARAMETER WITH HIGHEST PSR
100	1.013	10
200	1.014	28

300	1.010	46
400	1.005	38
500	1.003	34
600	1.003	42
700	1.002	38
800	1.002	42
900	1.002	31
1000	1.003	16

```
Reduced psychological distress, bullying interaction

SUMMARY OF DATA

     Number of clusters                         58

       Size (s)    Cluster ID with Size s

          1        166
          2        74 205
          3        106 182 165
          4        93 170 22 192 4
          5        65 71 50 84
          6        80 72
          7        40 107 61 75
          8        169 30 173 228
          9        144 81 83 181
         10        69 145 36 68
         11        108 116 174 154
         12        206 162
         13        226 114
         14        2
         15        152 98
         18        156
         19        202
         21        92 6
         24        178
         25        142 122
         32        24
         37        204
         47        209
         53        125
         67        217
        174        110
        210        100


COVARIANCE COVERAGE OF DATA

Minimum covariance coverage value   0.100

     Number of missing data patterns             5
```

PROPORTION OF DATA PRESENT

Covariance Coverage

	KESCAT16	PRAFAC	PHY15	KES15	CHOICE15
KESCAT16	1.000				
PRAFAC	1.000	1.000			
PHY15	1.000	1.000	1.000		
KES15	1.000	1.000	1.000	1.000	
CHOICE15	0.990	0.990	0.990	0.990	0.990
BULLY15	0.990	0.990	0.990	0.990	0.989
PRESS15	0.990	0.990	0.990	0.990	0.989
DUTIES15	0.991	0.991	0.991	0.991	0.990

Covariance Coverage

	BULLY15	PRESS15	DUTIES15
BULLY15	0.990		
PRESS15	0.989	0.990	
DUTIES15	0.990	0.990	0.991

UNIVARIATE PROPORTIONS AND COUNTS FOR CATEGORICAL VARIABLES

KESCAT16		
Category 1	0.347	386.000
Category 2	0.653	727.000

UNIVARIATE SAMPLE STATISTICS

UNIVARIATE HIGHER-ORDER MOMENT DESCRIPTIVE STATISTICS

Variable/ Sample Size	Mean/ Variance	Skewness/ Kurtosis	Minimum/ Maximum	% with Min/Max	Percentiles 20%/60%	40%/80%	Median
PRAFAC	-0.185	0.245	-2.338	1.72%	-1.448	-0.527	-0.318
58.000	1.345	-0.636	2.338	1.72%	0.080	0.752	
PHY15	0.000	0.390	-1.412	11.50%	-0.412	-0.412	-0.412

```
        1113.000       0.702       0.082       2.588    1.08%       0.588      0.588
     KES15             0.000       1.366      -0.556   20.75%      -0.556     -0.222     -0.222
        1113.000       0.313       1.871       2.278    0.63%      -0.056      0.444
     CHOICE15          0.000      -0.116      -1.814   15.06%      -0.814      0.186      0.186
        1102.000       1.193      -0.747       2.186    4.45%       0.186      1.186
     BULLY15           1.426       1.887       1.000   71.96%       1.000      1.000      1.000
        1102.000       0.602       3.132       5.000    0.45%       1.000      2.000
     PRESS15           0.000       0.096      -1.915    9.89%      -0.915      0.085      0.085
        1102.000       1.160      -0.512       2.085    8.35%       0.085      1.085
     DUTIES15          0.000      -0.677      -2.672    3.54%      -0.672      0.328      0.328
        1103.000       1.031       0.049       1.328   20.22%       0.328      1.328


THE MODEL ESTIMATION TERMINATED NORMALLY

     USE THE FBITERATIONS OPTION TO INCREASE THE NUMBER OF ITERATIONS BY A FACTOR
     OF AT LEAST TWO TO CHECK CONVERGENCE AND THAT THE PSR VALUE DOES NOT INCREASE.



MODEL FIT INFORMATION

Number of Free Parameters                              43



MODEL RESULTS

                                Posterior  One-Tailed         95% C.I.
                    Estimate       S.D.      P-Value   Lower 2.5%  Upper 2.5%  Significance

Within Level

 KESCAT16   ON
    PHY15              0.159       0.054      0.003       0.051       0.261      *
    CHOICE15           0.027       0.041      0.248      -0.050       0.111
    DUTIES15          -0.078       0.045      0.046      -0.162       0.011
    PRESS15           -0.042       0.042      0.153      -0.128       0.039
    KES15              0.803       0.095      0.000       0.625       0.994      *

 KES15    WITH
    PHY15              0.107       0.014      0.000       0.079       0.134      *
    CHOICE15          -0.095       0.018      0.000      -0.133      -0.061      *
    BULLY15            0.073       0.013      0.000       0.050       0.100      *
```

```
    PRESS15            0.062      0.019      0.001       0.028      0.099      *
    DUTIES15          -0.073      0.018      0.000      -0.107     -0.037      *

 PHY15    WITH
    CHOICE15          -0.098      0.028      0.001      -0.152     -0.043      *
    BULLY15            0.041      0.020      0.022       0.002      0.079      *
    PRESS15            0.119      0.028      0.000       0.068      0.176      *
    DUTIES15          -0.058      0.027      0.015      -0.112     -0.004      *

 CHOICE15 WITH
    BULLY15           -0.134      0.026      0.000      -0.187     -0.081      *
    PRESS15           -0.196      0.037      0.000      -0.271     -0.124      *
    DUTIES15           0.150      0.034      0.000       0.083      0.219      *

 BULLY15  WITH
    PRESS15            0.197      0.026      0.000       0.147      0.251      *
    DUTIES15          -0.169      0.025      0.000      -0.220     -0.124      *

 PRESS15  WITH
    DUTIES15          -0.288      0.034      0.000      -0.355     -0.222      *

 Means
    PHY15             -0.001      0.025      0.481      -0.051      0.049
    KES15              0.000      0.017      0.500      -0.031      0.032
    CHOICE15          -0.001      0.033      0.489      -0.067      0.062
    PRESS15            0.000      0.033      0.493      -0.063      0.065
    DUTIES15          -0.001      0.031      0.489      -0.058      0.062

 Variances
    PHY15              0.704      0.030      0.000       0.648      0.765      *
    KES15              0.314      0.013      0.000       0.289      0.342      *
    CHOICE15           1.194      0.051      0.000       1.097      1.297      *
    BULLY15            0.593      0.026      0.000       0.546      0.645      *
    PRESS15            1.162      0.049      0.000       1.069      1.263      *
    DUTIES15           1.033      0.044      0.000       0.953      1.126      *

Between Level

 S1         ON
    PRAFAC            -0.067      0.089      0.219      -0.230      0.120

 KESCAT16   ON
    PRAFAC            -0.131      0.054      0.007      -0.238     -0.033      *
    BULLY15            0.447      0.485      0.176      -0.540      1.404
```

```
 PRAFAC   WITH
    BULLY15            0.016       0.053      0.357      -0.080       0.124

 Means
    PRAFAC            -0.194       0.151      0.102      -0.495       0.094
    BULLY15            1.417       0.045      0.000       1.330       1.505      *

 Intercepts
    S1                 0.161       0.089      0.022       0.004       0.343      *

 Thresholds
    KESCAT16$1         0.166       0.688      0.408      -1.225       1.505

 Variances
    PRAFAC             1.343       0.261      0.000       0.957       2.000      *
    BULLY15            0.054       0.015      0.000       0.034       0.092      *

 Residual Variances
    KESCAT16           0.018       0.030      0.000       0.000       0.109      *
    S1                 0.044       0.078      0.000       0.001       0.281      *


STANDARDIZED MODEL RESULTS


STDYX Standardization

                                Posterior  One-Tailed         95% C.I.
                    Estimate       S.D.      P-Value   Lower 2.5%  Upper 2.5%  Significance

Within-Level Standardized Estimates Averaged Over Clusters

 S1 | KESCAT16 ON
    BULLY15            0.107       0.052      0.015       0.007       0.209      *

 KESCAT16   ON
    PHY15              0.114       0.038      0.003       0.036       0.188      *
    CHOICE15           0.027       0.038      0.240      -0.049       0.105
    DUTIES15          -0.068       0.039      0.040      -0.143       0.007
    PRESS15           -0.040       0.038      0.146      -0.116       0.035
    KES15              0.390       0.039      0.000       0.311       0.466      *

 KES15    WITH
```

```
    PHY15              0.227       0.028      0.000       0.173       0.281      *
    CHOICE15          -0.157       0.029      0.000      -0.212      -0.100      *
    BULLY15            0.171       0.029      0.000       0.116       0.228      *
    PRESS15            0.102       0.030      0.001       0.044       0.161      *
    DUTIES15          -0.128       0.030      0.000      -0.185      -0.069      *

 PHY15    WITH
    CHOICE15          -0.106       0.030      0.001      -0.162      -0.048      *
    BULLY15            0.063       0.030      0.022       0.003       0.121      *
    PRESS15            0.132       0.030      0.000       0.074       0.191      *
    DUTIES15          -0.068       0.030      0.013      -0.127      -0.007      *

 CHOICE15 WITH
    BULLY15           -0.160       0.030      0.000      -0.217      -0.099      *
    PRESS15           -0.166       0.030      0.000      -0.225      -0.106      *
    DUTIES15           0.135       0.030      0.000       0.075       0.194      *

 BULLY15  WITH
    PRESS15            0.238       0.029      0.000       0.180       0.294      *
    DUTIES15          -0.216       0.029      0.000      -0.274      -0.159      *

 PRESS15  WITH
    DUTIES15          -0.263       0.028      0.000      -0.315      -0.207      *

 Means
    PHY15              0.000       0.030      0.494      -0.059       0.058
    KES15              0.000       0.030      0.497      -0.058       0.057
    CHOICE15          -0.001       0.030      0.493      -0.061       0.057
    PRESS15            0.000       0.030      0.498      -0.058       0.059
    DUTIES15           0.000       0.030      0.494      -0.056       0.060

 Variances
    PHY15              1.000       0.000      0.000       1.000       1.000
    KES15              1.000       0.000      0.000       1.000       1.000
    CHOICE15           1.000       0.000      0.000       1.000       1.000
    BULLY15            1.000       0.000      0.000       1.000       1.000
    PRESS15            1.000       0.000      0.000       1.000       1.000
    DUTIES15           1.000       0.000      0.000       1.000       1.000

Between Level

 S1         ON
    PRAFAC            -0.343       0.396      0.219      -0.968       0.519
```

```
 KESCAT16   ON
    PRAFAC            -0.615       0.214      0.007      -0.970      -0.158      *
    BULLY15            0.428       0.385      0.176      -0.520       0.921

 PRAFAC   WITH
    BULLY15            0.063       0.184      0.357      -0.295       0.424

 Means
    PRAFAC            -0.166       0.128      0.102      -0.419       0.080
    BULLY15            6.113       0.785      0.000       4.619       7.812      *

 Intercepts
    S1                 0.637       0.595      0.022       0.021       1.990      *

 Variances
    PRAFAC             1.000       0.000      0.000       1.000       1.000
    BULLY15            1.000       0.000      0.000       1.000       1.000

 Residual Variances
    KESCAT16           0.312       0.245      0.000       0.009       0.866      *
    S1                 0.849       0.269      0.000       0.064       1.000      *


R-SQUARE

Within-Level R-Square Averaged Across Clusters

                                Posterior  One-Tailed         95% C.I.
    Variable        Estimate       S.D.      P-Value   Lower 2.5%  Upper 2.5%

    KESCAT16           0.251       0.040      0.000       0.176       0.338

Between Level

                                Posterior  One-Tailed         95% C.I.
    Variable        Estimate       S.D.      P-Value   Lower 2.5%  Upper 2.5%

    KESCAT16           0.688       0.245      0.000       0.134       0.991

                                Posterior  One-Tailed         95% C.I.
    Variable        Estimate       S.D.      P-Value   Lower 2.5%  Upper 2.5%

    S1                 0.151       0.269      0.000       0.000       0.936
```

TECHNICAL 1 OUTPUT

PARAMETER SPECIFICATION FOR WITHIN

TAU

KESCAT16
0

NU

KESCAT16	PHY15	KES15	CHOICE15	BULLY15
0	0	0	0	0

NU

PRESS15	DUTIES15
0	0

LAMBDA

	KESCAT16	PHY15	KES15	CHOICE15	BULLY15
KESCAT16	0	0	0	0	0
PHY15	0	0	0	0	0
KES15	0	0	0	0	0
CHOICE15	0	0	0	0	0
BULLY15	0	0	0	0	0
PRESS15	0	0	0	0	0
DUTIES15	0	0	0	0	0

LAMBDA

	PRESS15	DUTIES15
KESCAT16	0	0
PHY15	0	0
KES15	0	0
CHOICE15	0	0

BULLY15	0	0
PRESS15	0	0
DUTIES15	0	0

THETA

	KESCAT16	PHY15	KES15	CHOICE15	BULLY15
KESCAT16	0				
PHY15	0	0			
KES15	0	0	0		
CHOICE15	0	0	0	0	
BULLY15	0	0	0	0	0
PRESS15	0	0	0	0	0
DUTIES15	0	0	0	0	0

THETA

	PRESS15	DUTIES15
PRESS15	0	
DUTIES15	0	0

ALPHA

KESCAT16	PHY15	KES15	CHOICE15	BULLY15
0	1	2	3	0

ALPHA

PRESS15	DUTIES15
4	5

BETA

	KESCAT16	PHY15	KES15	CHOICE15	BULLY15
KESCAT16	0	6	7	8	0
PHY15	0	0	0	0	0
KES15	0	0	0	0	0
CHOICE15	0	0	0	0	0
BULLY15	0	0	0	0	0

PRESS15	0	0	0	0	0
DUTIES15	0	0	0	0	0

BETA

	PRESS15	DUTIES15
KESCAT16	9	10
PHY15	0	0
KES15	0	0
CHOICE15	0	0
BULLY15	0	0
PRESS15	0	0
DUTIES15	0	0

PSI

	KESCAT16	PHY15	KES15	CHOICE15	BULLY15
KESCAT16	0				
PHY15	0	11			
KES15	0	12	13		
CHOICE15	0	14	15	16	
BULLY15	0	17	18	19	20
PRESS15	0	21	22	23	24
DUTIES15	0	26	27	28	29

PSI

	PRESS15	DUTIES15
PRESS15	25	
DUTIES15	30	31

PARAMETER SPECIFICATION FOR BETWEEN

TAU

KESCAT16
43

NU

	KESCAT16	PRAFAC	BULLY15
	0	0	0

LAMBDA

	S1	KESCAT16	PRAFAC	BULLY15
KESCAT16	0	0	0	0
PRAFAC	0	0	0	0
BULLY15	0	0	0	0

THETA

	KESCAT16	PRAFAC	BULLY15
KESCAT16	0		
PRAFAC	0	0	
BULLY15	0	0	0

ALPHA

	S1	KESCAT16	PRAFAC	BULLY15
	32	0	33	34

BETA

	S1	KESCAT16	PRAFAC	BULLY15
S1	0	0	35	0
KESCAT16	0	0	36	37
PRAFAC	0	0	0	0
BULLY15	0	0	0	0

PSI

	S1	KESCAT16	PRAFAC	BULLY15
S1	38			
KESCAT16	0	39		
PRAFAC	0	0	40	
BULLY15	0	0	41	42

STARTING VALUES FOR WITHIN

TAU

KESCAT16
0.000

NU

KESCAT16	PHY15	KES15	CHOICE15	BULLY15
0.000	0.000	0.000	0.000	0.000

NU

PRESS15	DUTIES15
0.000	0.000

LAMBDA

	KESCAT16	PHY15	KES15	CHOICE15	BULLY15
KESCAT16	1.000	0.000	0.000	0.000	0.000
PHY15	0.000	1.000	0.000	0.000	0.000
KES15	0.000	0.000	1.000	0.000	0.000
CHOICE15	0.000	0.000	0.000	1.000	0.000
BULLY15	0.000	0.000	0.000	0.000	1.000
PRESS15	0.000	0.000	0.000	0.000	0.000
DUTIES15	0.000	0.000	0.000	0.000	0.000

LAMBDA

	PRESS15	DUTIES15
KESCAT16	0.000	0.000
PHY15	0.000	0.000
KES15	0.000	0.000
CHOICE15	0.000	0.000
BULLY15	0.000	0.000
PRESS15	1.000	0.000

DUTIES15	0.000	1.000

THETA

	KESCAT16	PHY15	KES15	CHOICE15	BULLY15
KESCAT16	0.000				
PHY15	0.000	0.000			
KES15	0.000	0.000	0.000		
CHOICE15	0.000	0.000	0.000	0.000	
BULLY15	0.000	0.000	0.000	0.000	0.000
PRESS15	0.000	0.000	0.000	0.000	0.000
DUTIES15	0.000	0.000	0.000	0.000	0.000

THETA

	PRESS15	DUTIES15
PRESS15	0.000	
DUTIES15	0.000	0.000

ALPHA

KESCAT16	PHY15	KES15	CHOICE15	BULLY15
0.000	0.000	0.000	0.000	0.000

ALPHA

PRESS15	DUTIES15
0.000	0.000

BETA

	KESCAT16	PHY15	KES15	CHOICE15	BULLY15
KESCAT16	0.000	0.000	0.000	0.000	0.000
PHY15	0.000	0.000	0.000	0.000	0.000
KES15	0.000	0.000	0.000	0.000	0.000
CHOICE15	0.000	0.000	0.000	0.000	0.000
BULLY15	0.000	0.000	0.000	0.000	0.000
PRESS15	0.000	0.000	0.000	0.000	0.000
DUTIES15	0.000	0.000	0.000	0.000	0.000

```
           BETA
              PRESS15       DUTIES15
              ________      ________
 KESCAT16       0.000         0.000
 PHY15          0.000         0.000
 KES15          0.000         0.000
 CHOICE15       0.000         0.000
 BULLY15        0.000         0.000
 PRESS15        0.000         0.000
 DUTIES15       0.000         0.000


           PSI
              KESCAT16      PHY15         KES15         CHOICE15      BULLY15
              ________      ________      ________      ________      ________
 KESCAT16       1.000
 PHY15          0.000         0.351
 KES15          0.000         0.000         0.157
 CHOICE15       0.000         0.000         0.000         0.597
 BULLY15        0.000         0.000         0.000         0.000         0.301
 PRESS15        0.000         0.000         0.000         0.000         0.000
 DUTIES15       0.000         0.000         0.000         0.000         0.000


           PSI
              PRESS15       DUTIES15
              ________      ________
 PRESS15        0.580
 DUTIES15       0.000         0.516


     STARTING VALUES FOR BETWEEN


           TAU
              KESCAT16
              ________
               -0.352


           NU
              KESCAT16      PRAFAC        BULLY15
```

	0.000	0.000	0.000

LAMBDA

	S1	KESCAT16	PRAFAC	BULLY15
KESCAT16	0.000	1.000	0.000	0.000
PRAFAC	0.000	0.000	1.000	0.000
BULLY15	0.000	0.000	0.000	1.000

THETA

	KESCAT16	PRAFAC	BULLY15
KESCAT16	0.000		
PRAFAC	0.000	0.000	
BULLY15	0.000	0.000	0.000

ALPHA

S1	KESCAT16	PRAFAC	BULLY15
0.000	0.000	-0.033	1.426

BETA

	S1	KESCAT16	PRAFAC	BULLY15
S1	0.000	0.000	0.000	0.000
KESCAT16	0.000	0.000	0.000	0.000
PRAFAC	0.000	0.000	0.000	0.000
BULLY15	0.000	0.000	0.000	0.000

PSI

	S1	KESCAT16	PRAFAC	BULLY15
S1	1.000			
KESCAT16	0.000	1.000		
PRAFAC	0.000	0.000	0.469	
BULLY15	0.000	0.000	0.000	0.301

PRIORS FOR ALL PARAMETERS	PRIOR MEAN	PRIOR VARIANCE	PRIOR STD. DEV.
Parameter 1~N(0.000,infinity)	0.0000	infinity	infinity
Parameter 2~N(0.000,infinity)	0.0000	infinity	infinity
Parameter 3~N(0.000,infinity)	0.0000	infinity	infinity
Parameter 4~N(0.000,infinity)	0.0000	infinity	infinity
Parameter 5~N(0.000,infinity)	0.0000	infinity	infinity
Parameter 6~N(0.000,5.000)	0.0000	5.0000	2.2361
Parameter 7~N(0.000,5.000)	0.0000	5.0000	2.2361
Parameter 8~N(0.000,5.000)	0.0000	5.0000	2.2361
Parameter 9~N(0.000,5.000)	0.0000	5.0000	2.2361
Parameter 10~N(0.000,5.000)	0.0000	5.0000	2.2361
Parameter 11~IW(1.000,7)	infinity	infinity	infinity
Parameter 12~IW(0.000,7)	infinity	infinity	infinity
Parameter 13~IW(1.000,7)	infinity	infinity	infinity
Parameter 14~IW(0.000,7)	infinity	infinity	infinity
Parameter 15~IW(0.000,7)	infinity	infinity	infinity
Parameter 16~IW(1.000,7)	infinity	infinity	infinity
Parameter 17~IW(0.000,7)	infinity	infinity	infinity
Parameter 18~IW(0.000,7)	infinity	infinity	infinity
Parameter 19~IW(0.000,7)	infinity	infinity	infinity
Parameter 20~IW(1.000,7)	infinity	infinity	infinity
Parameter 21~IW(0.000,7)	infinity	infinity	infinity
Parameter 22~IW(0.000,7)	infinity	infinity	infinity
Parameter 23~IW(0.000,7)	infinity	infinity	infinity
Parameter 24~IW(0.000,7)	infinity	infinity	infinity
Parameter 25~IW(1.000,7)	infinity	infinity	infinity
Parameter 26~IW(0.000,7)	infinity	infinity	infinity
Parameter 27~IW(0.000,7)	infinity	infinity	infinity
Parameter 28~IW(0.000,7)	infinity	infinity	infinity
Parameter 29~IW(0.000,7)	infinity	infinity	infinity
Parameter 30~IW(0.000,7)	infinity	infinity	infinity
Parameter 31~IW(1.000,7)	infinity	infinity	infinity
Parameter 32~N(0.000,infinity)	0.0000	infinity	infinity
Parameter 33~N(0.000,infinity)	0.0000	infinity	infinity
Parameter 34~N(0.000,infinity)	0.0000	infinity	infinity
Parameter 35~N(0.000,infinity)	0.0000	infinity	infinity
Parameter 36~N(0.000,5.000)	0.0000	5.0000	2.2361
Parameter 37~N(0.000,5.000)	0.0000	5.0000	2.2361
Parameter 38~IG(-1.000,0.000)	infinity	infinity	infinity
Parameter 39~IG(-1.000,0.000)	infinity	infinity	infinity
Parameter 40~IW(1.000,3)	infinity	infinity	infinity
Parameter 41~IW(0.000,3)	infinity	infinity	infinity

Parameter 42~IW(1.000,3)	infinity	infinity	infinity
Parameter 43~N(0.000,5.000)	0.0000	5.0000	2.2361

TECHNICAL 3 OUTPUT

ESTIMATED COVARIANCE MATRIX FOR PARAMETER ESTIMATES

	1	2	3	4	5
1	0.634743D-03				
2	0.936330D-04	0.273415D-03			
3	-0.747239D-04	-0.769098D-04	0.107308D-02		
4	0.106893D-03	0.533816D-04	-0.146073D-03	0.108671D-02	
5	-0.729167D-04	-0.463593D-04	0.101218D-03	-0.267248D-03	0.949355D-03
6	-0.612155D-04	0.615690D-05	0.975289D-04	0.394019D-05	-0.512776D-04
7	-0.647118D-05	-0.123082D-04	-0.120587D-03	0.431751D-04	-0.401535D-04
8	-0.816590D-05	0.120762D-04	0.100418D-04	0.258923D-05	0.185051D-04
9	-0.346088D-04	-0.411295D-05	-0.689579D-05	-0.692906D-04	0.991357D-05
10	-0.387721D-04	0.282717D-05	-0.237137D-04	-0.113387D-04	-0.375994D-05
11	0.177481D-04	0.738664D-05	-0.851117D-05	0.418191D-04	-0.651993D-04
12	-0.873261D-05	-0.243511D-05	0.414332D-05	0.250268D-05	-0.170534D-04
13	0.591066D-05	0.801235D-05	-0.150571D-04	0.709245D-05	-0.107290D-04
14	-0.532692D-05	0.840251D-05	-0.217832D-04	-0.662401D-05	-0.991358D-05
15	-0.799189D-05	-0.125230D-05	0.105978D-04	-0.585602D-05	-0.161180D-04
16	0.124786D-04	0.444000D-05	0.525701D-04	0.309396D-04	-0.281939D-04
17	-0.820143D-05	-0.227526D-05	0.147051D-06	-0.521280D-05	0.224489D-06
18	0.477210D-05	0.827543D-05	0.894042D-05	0.457805D-05	-0.133235D-04
19	0.142817D-04	0.355969D-05	0.127656D-04	0.217601D-04	-0.121489D-04
20	-0.187776D-04	-0.813173D-06	-0.153529D-04	-0.341121D-04	0.366646D-04
21	-0.178861D-04	0.865749D-05	0.159596D-04	0.940043D-05	-0.256042D-05
22	-0.436159D-05	0.351455D-05	0.493059D-04	-0.212145D-04	0.283906D-05
23	-0.450541D-05	0.781077D-05	-0.473748D-04	0.192584D-04	-0.150034D-04
24	-0.144844D-04	-0.833321D-06	0.232674D-04	-0.373596D-04	-0.648047D-05
25	-0.387580D-05	-0.312591D-04	0.775630D-04	-0.495659D-04	-0.147166D-04
26	0.189677D-04	-0.333205D-05	0.124937D-04	0.174560D-05	0.643271D-05
27	0.718697D-05	-0.885799D-05	-0.659953D-05	0.309780D-04	-0.149396D-04
28	-0.216975D-04	-0.134180D-05	0.167310D-04	-0.146717D-04	0.380145D-04
29	0.137706D-04	-0.387411D-05	0.123147D-04	0.585242D-04	-0.663579D-05
30	-0.287030D-04	-0.464505D-05	0.161749D-04	0.511821D-04	0.210969D-04
31	0.397933D-04	0.279938D-04	-0.889165D-04	0.431514D-04	-0.329886D-04
32	-0.536481D-04	-0.635372D-05	0.363213D-04	-0.454806D-04	0.423732D-04
33	0.141284D-04	-0.457643D-05	0.174487D-03	-0.226805D-03	0.128645D-03
34	0.907234D-04	0.613443D-04	-0.129160D-03	0.205507D-03	-0.125471D-03

35	-0.159467D-04	-0.225076D-04	0.819503D-04	0.650897D-04	-0.720447D-04
36	-0.492408D-04	0.605166D-05	0.698683D-04	0.259062D-04	0.466614D-04
37	0.574399D-04	0.561794D-04	0.198331D-03	-0.335287D-03	0.380736D-04
38	-0.182385D-04	-0.387073D-04	-0.727435D-05	0.431871D-04	-0.366381D-04
39	0.391022D-04	-0.191278D-04	-0.192589D-05	0.144962D-04	-0.175541D-04
40	0.124823D-03	0.219463D-03	0.365321D-04	0.444685D-04	-0.513171D-03
41	-0.157652D-04	0.996748D-05	0.456434D-04	0.247765D-04	-0.747981D-05
42	-0.413826D-06	-0.133398D-04	0.264207D-05	-0.205120D-05	-0.199300D-05
43	0.119040D-03	0.748392D-04	0.274914D-03	-0.422528D-03	0.540560D-04

ESTIMATED COVARIANCE MATRIX FOR PARAMETER ESTIMATES

	6	7	8	9	10
6	0.292441D-02				
7	-0.401990D-03	0.902182D-02			
8	0.258846D-05	0.302936D-03	0.168354D-02		
9	-0.232290D-03	-0.320625D-03	0.179118D-03	0.177960D-02	
10	0.117338D-03	0.200662D-03	-0.194315D-03	0.412082D-03	0.200432D-02
11	-0.386406D-04	-0.118141D-03	-0.458713D-04	-0.517880D-04	-0.572385D-04
12	0.124676D-05	0.496499D-04	-0.116222D-04	-0.335116D-04	-0.272677D-04
13	-0.191460D-04	0.375302D-04	-0.179155D-04	-0.471277D-05	-0.257336D-04
14	-0.855183D-04	0.214678D-04	0.840119D-05	0.130627D-04	-0.136042D-05
15	-0.238544D-04	0.439815D-04	0.486403D-04	0.343601D-05	0.400849D-04
16	0.130265D-03	-0.651946D-04	-0.118219D-03	-0.133077D-04	0.237263D-04
17	-0.899271D-05	0.129801D-04	-0.197204D-05	-0.245377D-04	0.421091D-04
18	-0.263188D-04	-0.180410D-04	0.364105D-05	0.673861D-05	0.218226D-04
19	0.180244D-04	-0.353422D-04	0.254271D-04	0.308587D-05	-0.414180D-05
20	-0.227613D-04	0.417555D-04	0.150074D-04	-0.751612D-05	-0.229427D-04
21	0.295724D-04	-0.222555D-04	-0.536557D-04	-0.803715D-05	0.342564D-04
22	-0.752563D-05	-0.282996D-04	-0.245369D-04	-0.150851D-04	-0.264998D-04
23	-0.556135D-04	-0.115927D-03	0.139451D-04	0.565581D-04	-0.424460D-04
24	0.787179D-05	-0.553448D-04	-0.462862D-04	-0.199550D-04	-0.219272D-04
25	0.444555D-04	-0.456644D-04	-0.167712D-04	-0.498752D-04	-0.587483D-04
26	0.248937D-05	-0.332179D-05	0.287848D-04	0.267467D-04	-0.281251D-04
27	0.224009D-04	0.382784D-04	0.270869D-04	-0.164743D-05	-0.261867D-05
28	0.770538D-04	-0.102651D-03	-0.209546D-04	-0.619449D-04	0.139313D-04
29	0.352659D-04	0.103623D-03	-0.860296D-05	-0.147657D-04	0.501968D-04
30	-0.317208D-04	0.202509D-03	0.380255D-04	0.530676D-04	0.690185D-05
31	-0.993858D-05	-0.354879D-03	-0.516345D-04	-0.510115D-04	0.496273D-04
32	-0.910085D-04	-0.108561D-03	0.168408D-03	-0.422392D-03	0.342498D-03
33	0.445456D-03	-0.340996D-03	-0.459116D-04	-0.489880D-04	0.508593D-04
34	-0.614062D-04	0.309408D-04	0.671969D-04	0.119341D-03	0.579909D-04
35	0.163625D-03	0.178419D-03	-0.300243D-03	0.104941D-03	0.144549D-03

36	0.266130D-03	0.301755D-03	-0.118977D-03	-0.220956D-03	0.439143D-04
37	-0.276854D-03	0.108373D-02	-0.849762D-03	0.173020D-03	0.101649D-02
38	-0.529579D-04	0.633436D-03	-0.748899D-04	-0.210053D-04	0.433977D-04
39	0.320738D-04	0.349325D-03	0.246677D-04	-0.281762D-04	0.820757D-05
40	0.688315D-04	-0.336733D-03	0.389684D-03	0.134481D-03	0.141114D-03
41	-0.185596D-04	0.548494D-04	-0.785966D-05	-0.305706D-04	-0.477643D-04
42	-0.460000D-04	-0.118623D-04	0.207888D-04	0.116817D-04	0.233118D-04
43	-0.569908D-03	0.240064D-03	-0.114145D-02	0.279185D-03	0.136384D-02

ESTIMATED COVARIANCE MATRIX FOR PARAMETER ESTIMATES

	11	12	13	14	15
11	0.887362D-03				
12	0.125821D-03	0.198322D-03			
13	0.869297D-05	0.578684D-04	0.174244D-03		
14	-0.139785D-03	-0.751726D-04	-0.119293D-04	0.780742D-03	
15	-0.100883D-04	-0.360394D-04	-0.459393D-04	0.118144D-03	0.333117D-03
16	-0.208015D-05	-0.161657D-04	0.138670D-04	-0.203011D-03	-0.214764D-03
17	0.505014D-04	0.461118D-04	0.239768D-04	-0.961848D-04	-0.238588D-04
18	0.588247D-05	0.110494D-04	0.339133D-04	-0.889997D-05	-0.416144D-04
19	0.469447D-05	-0.134701D-04	-0.130025D-04	0.331144D-04	0.913625D-04
20	-0.407839D-04	-0.847992D-05	0.928429D-05	0.421888D-05	-0.130507D-04
21	0.145416D-03	0.515239D-04	0.183504D-04	-0.161967D-03	-0.349440D-04
22	0.943199D-05	0.368730D-04	0.291254D-04	-0.344600D-04	-0.566464D-04
23	0.823026D-05	-0.257604D-04	-0.173658D-04	0.170524D-03	0.104373D-03
24	-0.357183D-04	0.131002D-04	0.560488D-05	-0.176034D-04	-0.266373D-04
25	-0.142633D-04	0.217400D-04	0.289901D-05	-0.619675D-04	-0.480518D-04
26	-0.716242D-04	-0.682911D-04	-0.733840D-05	0.131127D-03	0.334449D-04
27	-0.687061D-06	0.650903D-06	-0.233787D-04	0.270500D-04	0.393237D-04
28	0.656834D-05	0.129618D-05	0.244024D-04	-0.799335D-04	-0.122007D-03
29	0.405161D-04	0.872290D-05	0.867315D-05	0.214845D-04	0.255130D-04
30	0.216962D-04	-0.965368D-05	-0.213412D-05	0.803915D-04	0.356227D-04
31	-0.257000D-04	-0.344978D-04	-0.140999D-04	-0.443931D-04	-0.311343D-04
32	-0.177032D-04	-0.132247D-04	-0.673257D-04	0.362448D-05	0.502283D-04
33	-0.155048D-03	-0.576077D-05	-0.258947D-04	0.148799D-03	-0.326647D-05
34	0.127975D-04	0.283432D-06	0.117258D-04	0.229769D-04	-0.632182D-05
35	-0.178418D-05	-0.110517D-05	0.646135D-04	0.182108D-04	-0.175207D-04
36	-0.368617D-04	0.125174D-04	0.142061D-04	-0.135237D-04	0.103554D-04
37	-0.441051D-03	-0.402600D-04	0.131976D-03	-0.438620D-04	-0.144154D-03
38	0.146620D-04	-0.215343D-04	0.341561D-04	0.812914D-04	0.801145D-05
39	-0.110751D-04	-0.110944D-04	-0.704467D-05	-0.221016D-04	-0.608087D-05
40	0.111347D-03	0.300759D-05	-0.777383D-04	-0.562769D-04	0.236292D-03
41	-0.291805D-04	-0.224524D-05	0.493544D-04	0.246249D-04	-0.386263D-05

42	0.986634D-06	-0.151819D-04	-0.883493D-05	0.994206D-06	0.114833D-04
43	-0.511280D-03	-0.257971D-04	0.195451D-03	-0.289587D-04	-0.208354D-03

ESTIMATED COVARIANCE MATRIX FOR PARAMETER ESTIMATES

	16	17	18	19	20
16	0.256992D-02				
17	0.529924D-04	0.390286D-03			
18	0.149872D-04	0.616989D-04	0.170475D-03		
19	-0.224090D-03	-0.755608D-04	-0.698668D-04	0.692767D-03	
20	0.620920D-05	0.418929D-04	0.943950D-04	-0.151319D-03	0.657845D-03
21	0.320564D-04	0.131389D-03	0.390262D-04	-0.196017D-04	0.353422D-04
22	0.214613D-04	0.498813D-04	0.675742D-04	-0.518567D-04	0.446248D-04
23	-0.464905D-03	-0.427554D-04	-0.431611D-04	0.252857D-03	-0.445491D-04
24	0.415548D-04	0.792641D-04	0.422071D-04	-0.155084D-03	0.207803D-03
25	0.797816D-04	0.857643D-04	0.315923D-04	-0.612977D-04	0.187735D-04
26	-0.285987D-04	-0.943991D-04	-0.233250D-04	0.181292D-04	-0.366753D-04
27	-0.413660D-04	-0.256992D-04	-0.539339D-04	0.307186D-04	-0.382650D-04
28	0.301361D-03	0.189946D-04	0.438897D-04	-0.172192D-03	0.379869D-04
29	0.949373D-05	-0.258364D-04	-0.560531D-04	0.130272D-03	-0.196890D-03
30	-0.775329D-04	-0.336107D-04	-0.152525D-04	0.563235D-04	-0.983725D-04
31	0.134714D-03	0.156496D-04	0.154001D-04	-0.153206D-04	0.644781D-04
32	0.117482D-03	0.651656D-05	-0.938506D-05	0.392882D-04	-0.376555D-04
33	-0.251904D-03	-0.712815D-04	0.275000D-05	0.370747D-04	0.445836D-04
34	-0.166704D-04	-0.179233D-04	0.410757D-04	-0.764454D-04	-0.169569D-04
35	-0.149879D-04	0.204982D-04	0.434135D-04	0.752112D-05	0.975069D-04
36	0.131383D-04	-0.220091D-04	-0.150828D-04	0.585422D-04	-0.499708D-04
37	-0.620850D-03	0.636367D-03	0.275786D-03	0.595679D-03	0.238678D-03
38	-0.537025D-04	0.257785D-04	0.265666D-04	0.606660D-04	-0.517810D-04
39	-0.544863D-04	-0.836664D-05	-0.414731D-05	-0.149998D-04	0.296118D-05
40	-0.726042D-03	-0.386037D-04	0.109911D-04	0.146424D-03	0.807453D-04
41	0.144645D-03	0.422670D-04	0.571451D-04	-0.116532D-03	0.272206D-05
42	-0.467209D-05	0.492864D-05	-0.837271D-06	0.201804D-05	-0.211178D-04
43	-0.103207D-02	0.915555D-03	0.436753D-03	0.776556D-03	0.315521D-03

ESTIMATED COVARIANCE MATRIX FOR PARAMETER ESTIMATES

	21	22	23	24	25
21	0.761424D-03				
22	0.136176D-03	0.344910D-03			
23	-0.151305D-03	-0.137015D-03	0.134024D-02		
24	0.641303D-04	0.108620D-03	-0.164410D-03	0.677001D-03	

25	0.272000D-03	0.155268D-03	-0.433635D-03	0.323371D-03	0.240870D-02
26	-0.187949D-03	-0.619558D-04	0.698438D-04	-0.368403D-04	-0.870768D-04
27	-0.274796D-04	-0.915045D-04	0.378124D-04	-0.367248D-04	-0.163229D-04
28	0.616806D-04	0.627075D-04	-0.386331D-03	0.680384D-04	0.715211D-04
29	-0.213716D-04	-0.424801D-04	0.944411D-04	-0.184182D-03	-0.817957D-04
30	-0.121423D-03	-0.104622D-03	0.241718D-03	-0.224856D-03	-0.629246D-03
31	-0.415086D-05	0.351954D-04	-0.431124D-04	0.316829D-04	0.151409D-03
32	0.155499D-04	0.202923D-04	0.666328D-04	-0.613387D-04	0.292361D-03
33	0.104243D-03	-0.740710D-04	-0.652367D-04	-0.281234D-04	-0.147917D-05
34	0.127760D-04	0.312515D-04	0.147088D-04	0.631694D-04	-0.917494D-04
35	0.365812D-04	0.566407D-04	-0.771587D-05	0.113373D-03	0.978991D-04
36	0.418419D-04	0.435148D-04	-0.108557D-04	-0.194182D-04	0.292161D-04
37	-0.266959D-03	-0.332045D-03	0.225661D-03	-0.239163D-03	-0.105192D-02
38	0.103586D-04	0.675359D-05	-0.350409D-04	-0.952212D-04	0.185811D-03
39	-0.297992D-04	-0.143255D-04	0.401908D-04	0.409687D-05	0.213631D-05
40	0.365953D-03	-0.486739D-04	0.166505D-03	0.516368D-04	0.468756D-03
41	-0.461514D-05	-0.157521D-05	-0.350831D-04	-0.456589D-04	-0.120026D-04
42	0.625592D-05	-0.656326D-05	-0.201863D-04	-0.134417D-04	0.598419D-05
43	-0.358366D-03	-0.438491D-03	0.355879D-03	-0.295187D-03	-0.153429D-02

ESTIMATED COVARIANCE MATRIX FOR PARAMETER ESTIMATES

	26	27	28	29	30
26	0.704258D-03				
27	0.967295D-04	0.314211D-03			
28	-0.104564D-03	-0.104257D-03	0.114127D-02		
29	0.453027D-04	0.998312D-04	-0.143800D-03	0.605343D-03	
30	0.133846D-03	0.103066D-03	-0.244102D-03	0.242756D-03	0.118704D-02
31	-0.402685D-04	-0.163607D-03	0.288082D-03	-0.298948D-03	-0.492123D-03
32	-0.145673D-05	-0.354802D-04	0.125278D-04	0.376257D-04	-0.886894D-04
33	-0.998554D-04	0.853338D-05	0.130999D-03	-0.263306D-04	-0.779899D-04
34	-0.492377D-06	-0.471341D-05	-0.183000D-04	0.146983D-04	0.542355D-04
35	-0.177652D-04	-0.721103D-04	-0.120984D-03	-0.762850D-04	0.452957D-04
36	-0.898453D-05	0.106538D-05	-0.279211D-04	0.379943D-04	-0.174135D-04
37	0.959134D-04	0.126098D-03	0.274373D-03	-0.576325D-04	0.643264D-03
38	0.367514D-04	-0.154755D-04	-0.154768D-03	0.685475D-04	0.236423D-04
39	0.205979D-04	0.117571D-05	-0.399743D-05	0.935032D-05	0.369134D-04
40	0.224201D-03	-0.694951D-04	-0.371537D-03	0.122283D-04	-0.400977D-04
41	-0.915504D-05	-0.108446D-04	-0.463591D-04	0.257522D-04	-0.820327D-05
42	-0.152490D-04	0.542310D-05	-0.496134D-05	0.711906D-05	0.648664D-05
43	0.142995D-03	0.214881D-03	0.400095D-03	-0.815003D-04	0.815892D-03

ESTIMATED COVARIANCE MATRIX FOR PARAMETER ESTIMATES

	31	32	33	34	35
31	0.194621D-02				
32	0.212413D-03	0.784998D-02			
33	0.305853D-03	-0.973351D-04	0.228530D-01		
34	0.270122D-04	-0.289068D-04	0.215706D-03	0.202176D-02	
35	-0.123097D-03	0.355028D-03	0.376617D-03	-0.358443D-05	0.789639D-02
36	-0.660398D-04	-0.118364D-03	0.406388D-03	-0.168606D-04	0.793065D-03
37	-0.743530D-03	-0.264671D-02	-0.179772D-02	-0.446006D-03	-0.231713D-02
38	0.927308D-04	0.149174D-02	-0.217081D-03	-0.719053D-04	0.647421D-03
39	0.556773D-04	-0.109880D-05	0.446352D-04	0.398189D-04	0.661988D-04
40	0.265365D-04	-0.367591D-03	-0.146008D-02	-0.371305D-03	-0.609093D-04
41	-0.611325D-04	-0.804383D-04	-0.219743D-03	-0.992358D-04	0.473099D-03
42	-0.175603D-04	0.134860D-04	0.252632D-04	0.151308D-04	-0.615960D-04
43	-0.102168D-02	-0.451327D-02	-0.199941D-02	-0.444065D-03	-0.328826D-02

ESTIMATED COVARIANCE MATRIX FOR PARAMETER ESTIMATES

	36	37	38	39	40
36	0.290704D-02				
37	-0.476365D-02	0.234964D+00			
38	-0.209275D-04	-0.455796D-03	0.610174D-02		
39	-0.147144D-03	-0.140164D-02	0.205867D-03	0.924426D-03	
40	0.429182D-03	-0.183921D-02	0.557478D-03	0.597388D-04	0.682777D-01
41	-0.171558D-03	-0.158173D-02	-0.374768D-04	0.681471D-05	0.853075D-03
42	-0.241782D-04	-0.290036D-03	0.472678D-04	-0.960217D-06	0.121650D-03
43	-0.704472D-02	0.332162D+00	-0.123623D-02	-0.191856D-02	-0.285441D-02

ESTIMATED COVARIANCE MATRIX FOR PARAMETER ESTIMATES

	41	42	43
41	0.276127D-02		
42	0.426744D-04	0.219271D-03	
43	-0.214572D-02	-0.418543D-03	0.472934D+00

ESTIMATED CORRELATION MATRIX FOR PARAMETER ESTIMATES

	1	2	3	4	5
1	1.000				
2	0.225	1.000			

3	-0.091	-0.142	1.000		
4	0.129	0.098	-0.135	1.000	
5	-0.094	-0.091	0.100	-0.263	1.000
6	-0.045	0.007	0.055	0.002	-0.031
7	-0.003	-0.008	-0.039	0.014	-0.014
8	-0.008	0.018	0.007	0.002	0.015
9	-0.033	-0.006	-0.005	-0.050	0.008
10	-0.034	0.004	-0.016	-0.008	-0.003
11	0.024	0.015	-0.009	0.043	-0.071
12	-0.025	-0.010	0.009	0.005	-0.039
13	0.018	0.037	-0.035	0.016	-0.026
14	-0.008	0.018	-0.024	-0.007	-0.012
15	-0.017	-0.004	0.018	-0.010	-0.029
16	0.010	0.005	0.032	0.019	-0.018
17	-0.016	-0.007	0.000	-0.008	0.000
18	0.015	0.038	0.021	0.011	-0.033
19	0.022	0.008	0.015	0.025	-0.015
20	-0.029	-0.002	-0.018	-0.040	0.046
21	-0.026	0.019	0.018	0.010	-0.003
22	-0.009	0.011	0.081	-0.035	0.005
23	-0.005	0.013	-0.040	0.016	-0.013
24	-0.022	-0.002	0.027	-0.044	-0.008
25	-0.003	-0.039	0.048	-0.031	-0.010
26	0.028	-0.008	0.014	0.002	0.008
27	0.016	-0.030	-0.011	0.053	-0.027
28	-0.025	-0.002	0.015	-0.013	0.037
29	0.022	-0.010	0.015	0.072	-0.009
30	-0.033	-0.008	0.014	0.045	0.020
31	0.036	0.038	-0.062	0.030	-0.024
32	-0.024	-0.004	0.013	-0.016	0.016
33	0.004	-0.002	0.035	-0.046	0.028
34	0.080	0.083	-0.088	0.139	-0.091
35	-0.007	-0.015	0.028	0.022	-0.026
36	-0.036	0.007	0.040	0.015	0.028
37	0.005	0.007	0.012	-0.021	0.003
38	-0.009	-0.030	-0.003	0.017	-0.015
39	0.051	-0.038	-0.002	0.014	-0.019
40	0.019	0.051	0.004	0.005	-0.064
41	-0.012	0.011	0.027	0.014	-0.005
42	-0.001	-0.054	0.005	-0.004	-0.004
43	0.007	0.007	0.012	-0.019	0.003

ESTIMATED CORRELATION MATRIX FOR PARAMETER ESTIMATES

	6	7	8	9	10
6	1.000				
7	-0.078	1.000			
8	0.001	0.078	1.000		
9	-0.102	-0.080	0.103	1.000	
10	0.048	0.047	-0.106	0.218	1.000
11	-0.024	-0.042	-0.038	-0.041	-0.043
12	0.002	0.037	-0.020	-0.056	-0.043
13	-0.027	0.030	-0.033	-0.008	-0.044
14	-0.057	0.008	0.007	0.011	-0.001
15	-0.024	0.025	0.065	0.004	0.049
16	0.048	-0.014	-0.057	-0.006	0.010
17	-0.008	0.007	-0.002	-0.029	0.048
18	-0.037	-0.015	0.007	0.012	0.037
19	0.013	-0.014	0.024	0.003	-0.004
20	-0.016	0.017	0.014	-0.007	-0.020
21	0.020	-0.008	-0.047	-0.007	0.028
22	-0.007	-0.016	-0.032	-0.019	-0.032
23	-0.028	-0.033	0.009	0.037	-0.026
24	0.006	-0.022	-0.043	-0.018	-0.019
25	0.017	-0.010	-0.008	-0.024	-0.027
26	0.002	-0.001	0.026	0.024	-0.024
27	0.023	0.023	0.037	-0.002	-0.003
28	0.042	-0.032	-0.015	-0.043	0.009
29	0.027	0.044	-0.009	-0.014	0.046
30	-0.017	0.062	0.027	0.037	0.004
31	-0.004	-0.085	-0.029	-0.027	0.025
32	-0.019	-0.013	0.046	-0.113	0.086
33	0.054	-0.024	-0.007	-0.008	0.008
34	-0.025	0.007	0.036	0.063	0.029
35	0.034	0.021	-0.082	0.028	0.036
36	0.091	0.059	-0.054	-0.097	0.018
37	-0.011	0.024	-0.043	0.008	0.047
38	-0.013	0.085	-0.023	-0.006	0.012
39	0.020	0.121	0.020	-0.022	0.006
40	0.005	-0.014	0.036	0.012	0.012
41	-0.007	0.011	-0.004	-0.014	-0.020
42	-0.057	-0.008	0.034	0.019	0.035
43	-0.015	0.004	-0.040	0.010	0.044

ESTIMATED CORRELATION MATRIX FOR PARAMETER ESTIMATES

11	12	13	14	15

11	1.000				
12	0.300	1.000			
13	0.022	0.311	1.000		
14	-0.168	-0.191	-0.032	1.000	
15	-0.019	-0.140	-0.191	0.232	1.000
16	-0.001	-0.023	0.021	-0.143	-0.232
17	0.086	0.166	0.092	-0.174	-0.066
18	0.015	0.060	0.197	-0.024	-0.175
19	0.006	-0.036	-0.037	0.045	0.190
20	-0.053	-0.023	0.027	0.006	-0.028
21	0.177	0.133	0.050	-0.210	-0.069
22	0.017	0.141	0.119	-0.066	-0.167
23	0.008	-0.050	-0.036	0.167	0.156
24	-0.046	0.036	0.016	-0.024	-0.056
25	-0.010	0.031	0.004	-0.045	-0.054
26	-0.091	-0.183	-0.021	0.177	0.069
27	-0.001	0.003	-0.100	0.055	0.122
28	0.007	0.003	0.055	-0.085	-0.198
29	0.055	0.025	0.027	0.031	0.057
30	0.021	-0.020	-0.005	0.084	0.057
31	-0.020	-0.056	-0.024	-0.036	-0.039
32	-0.007	-0.011	-0.058	0.001	0.031
33	-0.034	-0.003	-0.013	0.035	-0.001
34	0.010	0.000	0.020	0.018	-0.008
35	-0.001	-0.001	0.055	0.007	-0.011
36	-0.023	0.016	0.020	-0.009	0.011
37	-0.031	-0.006	0.021	-0.003	-0.016
38	0.006	-0.020	0.033	0.037	0.006
39	-0.012	-0.026	-0.018	-0.026	-0.011
40	0.014	0.001	-0.023	-0.008	0.050
41	-0.019	-0.003	0.071	0.017	-0.004
42	0.002	-0.073	-0.045	0.002	0.042
43	-0.025	-0.003	0.022	-0.002	-0.017

ESTIMATED CORRELATION MATRIX FOR PARAMETER ESTIMATES

	16	17	18	19	20
16	1.000				
17	0.053	1.000			
18	0.023	0.239	1.000		
19	-0.168	-0.145	-0.203	1.000	
20	0.005	0.083	0.282	-0.224	1.000

21	0.023	0.241	0.108	-0.027	0.050
22	0.023	0.136	0.279	-0.106	0.094
23	-0.251	-0.059	-0.090	0.262	-0.047
24	0.032	0.154	0.124	-0.226	0.311
25	0.032	0.088	0.049	-0.047	0.015
26	-0.021	-0.180	-0.067	0.026	-0.054
27	-0.046	-0.073	-0.233	0.066	-0.084
28	0.176	0.028	0.100	-0.194	0.044
29	0.008	-0.053	-0.174	0.201	-0.312
30	-0.044	-0.049	-0.034	0.062	-0.111
31	0.060	0.018	0.027	-0.013	0.057
32	0.026	0.004	-0.008	0.017	-0.017
33	-0.033	-0.024	0.001	0.009	0.011
34	-0.007	-0.020	0.070	-0.065	-0.015
35	-0.003	0.012	0.037	0.003	0.043
36	0.005	-0.021	-0.021	0.041	-0.036
37	-0.025	0.066	0.044	0.047	0.019
38	-0.014	0.017	0.026	0.030	-0.026
39	-0.035	-0.014	-0.010	-0.019	0.004
40	-0.055	-0.007	0.003	0.021	0.012
41	0.054	0.041	0.083	-0.084	0.002
42	-0.006	0.017	-0.004	0.005	-0.056
43	-0.030	0.067	0.049	0.043	0.018

ESTIMATED CORRELATION MATRIX FOR PARAMETER ESTIMATES

	21	22	23	24	25
21	1.000				
22	0.266	1.000			
23	-0.150	-0.202	1.000		
24	0.089	0.225	-0.173	1.000	
25	0.201	0.170	-0.241	0.253	1.000
26	-0.257	-0.126	0.072	-0.053	-0.067
27	-0.056	-0.278	0.058	-0.080	-0.019
28	0.066	0.100	-0.312	0.077	0.043
29	-0.031	-0.093	0.105	-0.288	-0.068
30	-0.128	-0.164	0.192	-0.251	-0.372
31	-0.003	0.043	-0.027	0.028	0.070
32	0.006	0.012	0.021	-0.027	0.067
33	0.025	-0.026	-0.012	-0.007	0.000
34	0.010	0.037	0.009	0.054	-0.042
35	0.015	0.034	-0.002	0.049	0.022
36	0.028	0.043	-0.005	-0.014	0.011

37	-0.020	-0.037	0.013	-0.019	-0.044
38	0.005	0.005	-0.012	-0.047	0.048
39	-0.036	-0.025	0.036	0.005	0.001
40	0.051	-0.010	0.017	0.008	0.037
41	-0.003	-0.002	-0.018	-0.033	-0.005
42	0.015	-0.024	-0.037	-0.035	0.008
43	-0.019	-0.034	0.014	-0.016	-0.045

ESTIMATED CORRELATION MATRIX FOR PARAMETER ESTIMATES

	26	27	28	29	30
26	1.000				
27	0.206	1.000			
28	-0.117	-0.174	1.000		
29	0.069	0.229	-0.173	1.000	
30	0.146	0.169	-0.210	0.286	1.000
31	-0.034	-0.209	0.193	-0.275	-0.324
32	-0.001	-0.023	0.004	0.017	-0.029
33	-0.025	0.003	0.026	-0.007	-0.015
34	0.000	-0.006	-0.012	0.013	0.035
35	-0.008	-0.046	-0.040	-0.035	0.015
36	-0.006	0.001	-0.015	0.029	-0.009
37	0.007	0.015	0.017	-0.005	0.039
38	0.018	-0.011	-0.059	0.036	0.009
39	0.026	0.002	-0.004	0.012	0.035
40	0.032	-0.015	-0.042	0.002	-0.004
41	-0.007	-0.012	-0.026	0.020	-0.005
42	-0.039	0.021	-0.010	0.020	0.013
43	0.008	0.018	0.017	-0.005	0.034

ESTIMATED CORRELATION MATRIX FOR PARAMETER ESTIMATES

	31	32	33	34	35
31	1.000				
32	0.054	1.000			
33	0.046	-0.007	1.000		
34	0.014	-0.007	0.032	1.000	
35	-0.031	0.045	0.028	-0.001	1.000
36	-0.028	-0.025	0.050	-0.007	0.166
37	-0.035	-0.062	-0.025	-0.020	-0.054
38	0.027	0.216	-0.018	-0.020	0.093
39	0.042	0.000	0.010	0.029	0.025

40	0.002	-0.016	-0.037	-0.032	-0.003
41	-0.026	-0.017	-0.028	-0.042	0.101
42	-0.027	0.010	0.011	0.023	-0.047
43	-0.034	-0.074	-0.019	-0.014	-0.054

ESTIMATED CORRELATION MATRIX FOR PARAMETER ESTIMATES

	36	37	38	39	40
36	1.000				
37	-0.182	1.000			
38	-0.005	-0.012	1.000		
39	-0.090	-0.095	0.087	1.000	
40	0.030	-0.015	0.027	0.008	1.000
41	-0.061	-0.062	-0.009	0.004	0.062
42	-0.030	-0.040	0.041	-0.002	0.031
43	-0.190	0.996	-0.023	-0.092	-0.016

ESTIMATED CORRELATION MATRIX FOR PARAMETER ESTIMATES

	41	42	43
41	1.000		
42	0.055	1.000	
43	-0.059	-0.041	1.000

TECHNICAL 8 OUTPUT

TECHNICAL 8 OUTPUT FOR BAYES ESTIMATION

CHAIN	BSEED
1	0
2	285380
3	253358

ITERATION	POTENTIAL SCALE REDUCTION	PARAMETER WITH HIGHEST PSR
100	1.035	38
200	1.017	35
300	1.014	38
400	1.012	34
500	1.007	32

600	1.010	39
700	1.005	39
800	1.006	39
900	1.004	39
1000	1.003	7

Reduced psychological distress, time pressure interaction

SUMMARY OF DATA

Number of clusters 58

Size (s)	Cluster ID with Size s
1	166
2	74 205
3	106 182 165
4	93 170 22 192 4
5	65 71 50 84
6	80 72
7	40 107 61 75
8	169 30 173 228
9	144 81 83 181
10	69 145 36 68
11	108 116 174 154
12	206 162
13	226 114
14	2
15	152 98
18	156
19	202
21	92 6
24	178
25	142 122
32	24
37	204
47	209
53	125
67	217
174	110
210	100

COVARIANCE COVERAGE OF DATA

Minimum covariance coverage value 0.100

Number of missing data patterns 5

PROPORTION OF DATA PRESENT

Covariance Coverage

	KESCAT16	PRAFAC	PHY15	KES15	CHOICE15
KESCAT16	1.000				
PRAFAC	1.000	1.000			
PHY15	1.000	1.000	1.000		
KES15	1.000	1.000	1.000	1.000	
CHOICE15	0.990	0.990	0.990	0.990	0.990
BULLY15	0.990	0.990	0.990	0.990	0.989
PRESS15	0.990	0.990	0.990	0.990	0.989
DUTIES15	0.991	0.991	0.991	0.991	0.990

Covariance Coverage

	BULLY15	PRESS15	DUTIES15
BULLY15	0.990		
PRESS15	0.989	0.990	
DUTIES15	0.990	0.990	0.991

UNIVARIATE PROPORTIONS AND COUNTS FOR CATEGORICAL VARIABLES

KESCAT16

Category 1	0.347	386.000
Category 2	0.653	727.000

UNIVARIATE SAMPLE STATISTICS

UNIVARIATE HIGHER-ORDER MOMENT DESCRIPTIVE STATISTICS

Variable/ Sample Size	Mean/ Variance	Skewness/ Kurtosis	Minimum/ Maximum	% with Min/Max	Percentiles 20%/60%	40%/80%	Median
PRAFAC	-0.185	0.245	-2.338	1.72%	-1.448	-0.527	-0.318
58.000	1.345	-0.636	2.338	1.72%	0.080	0.752	
PHY15	0.000	0.390	-1.412	11.50%	-0.412	-0.412	-0.412

1113.000	0.702	0.082	2.588	1.08%	0.588	0.588	
KES15	0.000	1.366	-0.556	20.75%	-0.556	-0.222	-0.222
1113.000	0.313	1.871	2.278	0.63%	-0.056	0.444	
CHOICE15	0.000	-0.116	-1.814	15.06%	-0.814	0.186	0.186
1102.000	1.193	-0.747	2.186	4.45%	0.186	1.186	
BULLY15	0.000	1.887	-0.426	71.96%	-0.426	-0.426	-0.426
1102.000	0.602	3.132	3.574	0.45%	-0.426	0.574	
PRESS15	2.915	0.096	1.000	9.89%	2.000	3.000	3.000
1102.000	1.160	-0.512	5.000	8.35%	3.000	4.000	
DUTIES15	0.000	-0.677	-2.672	3.54%	-0.672	0.328	0.328
1103.000	1.031	0.049	1.328	20.22%	0.328	1.328	

THE MODEL ESTIMATION TERMINATED NORMALLY

USE THE FBITERATIONS OPTION TO INCREASE THE NUMBER OF ITERATIONS BY A FACTOR
OF AT LEAST TWO TO CHECK CONVERGENCE AND THAT THE PSR VALUE DOES NOT INCREASE.

MODEL FIT INFORMATION

Number of Free Parameters 43

MODEL RESULTS

	Estimate	Posterior S.D.	One-Tailed P-Value	95% C.I. Lower 2.5%	Upper 2.5%	Significance
**Within Level**						
KESCAT16 ON						
PHY15	0.165	0.054	0.003	0.058	0.272	*
CHOICE15	0.025	0.041	0.266	-0.051	0.111	
DUTIES15	-0.076	0.045	0.050	-0.164	0.012	
BULLY15	0.114	0.061	0.031	-0.005	0.235	
KES15	0.804	0.096	0.000	0.626	0.996	*
KES15 WITH						
PHY15	0.107	0.014	0.000	0.079	0.135	*
CHOICE15	-0.095	0.018	0.000	-0.133	-0.061	*
BULLY15	0.072	0.013	0.000	0.049	0.099	*

```
   PRESS15            0.070       0.019      0.000       0.036       0.108      *
   DUTIES15          -0.073       0.018      0.000      -0.107      -0.037      *

 PHY15    WITH
   CHOICE15          -0.098       0.028      0.001      -0.152      -0.043      *
   BULLY15            0.041       0.020      0.018       0.002       0.077      *
   PRESS15            0.114       0.028      0.000       0.064       0.172      *
   DUTIES15          -0.058       0.027      0.015      -0.112      -0.005      *

 CHOICE15 WITH
   BULLY15           -0.137       0.026      0.000      -0.189      -0.086      *
   PRESS15           -0.184       0.037      0.000      -0.261      -0.112      *
   DUTIES15           0.151       0.034      0.000       0.083       0.219      *

 BULLY15  WITH
   PRESS15            0.196       0.025      0.000       0.144       0.247      *
   DUTIES15          -0.176       0.024      0.000      -0.225      -0.130      *

 PRESS15  WITH
   DUTIES15          -0.283       0.035      0.000      -0.352      -0.217      *

 Means
   PHY15             -0.001       0.025      0.483      -0.051       0.048
   KES15              0.000       0.017      0.498      -0.031       0.032
   CHOICE15           0.000       0.033      0.493      -0.066       0.061
   BULLY15            0.000       0.024      0.500      -0.048       0.046
   DUTIES15           0.000       0.031      0.497      -0.059       0.063

 Variances
   PHY15              0.704       0.030      0.000       0.648       0.765      *
   KES15              0.314       0.013      0.000       0.289       0.341      *
   CHOICE15           1.194       0.051      0.000       1.097       1.296      *
   BULLY15            0.603       0.026      0.000       0.555       0.656      *
   PRESS15            1.118       0.048      0.000       1.030       1.217      *
   DUTIES15           1.033       0.044      0.000       0.953       1.126      *

Between Level

 S1         ON
   PRAFAC             0.000       0.049      0.495      -0.090       0.096

 KESCAT16   ON
   PRAFAC            -0.112       0.058      0.023      -0.234      -0.002      *
   PRESS15           -0.157       0.316      0.315      -0.770       0.432
```

```
 PRAFAC   WITH
    PRESS15            0.084       0.072      0.100      -0.047       0.242

 Means
    PRAFAC            -0.194       0.151      0.102      -0.495       0.093
    PRESS15            2.873       0.062      0.000       2.744       2.991      *

 Intercepts
    S1                -0.044       0.056      0.213      -0.163       0.062

 Thresholds
    KESCAT16$1        -0.930       0.924      0.170      -2.727       0.807

 Variances
    PRAFAC             1.334       0.265      0.000       0.958       1.998      *
    PRESS15            0.096       0.029      0.000       0.054       0.169      *

 Residual Variances
    KESCAT16           0.021       0.035      0.000       0.001       0.130      *
    S1                 0.019       0.030      0.000       0.001       0.114      *


STANDARDIZED MODEL RESULTS


STDYX Standardization

                                Posterior  One-Tailed         95% C.I.
                    Estimate       S.D.      P-Value   Lower 2.5%  Upper 2.5%  Significance

Within-Level Standardized Estimates Averaged Over Clusters

 S1 | KESCAT16 ON
    PRESS15           -0.045       0.046      0.160      -0.137       0.047

 KESCAT16   ON
    PHY15              0.120       0.039      0.002       0.042       0.195      *
    CHOICE15           0.025       0.039      0.257      -0.050       0.104
    DUTIES15          -0.068       0.039      0.045      -0.143       0.008
    BULLY15            0.075       0.041      0.030      -0.003       0.157
    KES15              0.393       0.039      0.000       0.314       0.471      *

 KES15    WITH
```

```
    PHY15              0.227       0.028       0.000       0.172       0.282      *
    CHOICE15          -0.157       0.029       0.000      -0.213      -0.101      *
    BULLY15            0.166       0.029       0.000       0.113       0.222      *
    PRESS15            0.118       0.031       0.000       0.059       0.177      *
    DUTIES15          -0.128       0.030       0.000      -0.185      -0.069      *

 PHY15    WITH
    CHOICE15          -0.106       0.030       0.001      -0.162      -0.048      *
    BULLY15            0.062       0.030       0.019       0.003       0.117      *
    PRESS15            0.129       0.030       0.000       0.070       0.189      *
    DUTIES15          -0.068       0.030       0.014      -0.127      -0.007      *

 CHOICE15 WITH
    BULLY15           -0.164       0.029       0.000      -0.219      -0.103      *
    PRESS15           -0.159       0.031       0.000      -0.219      -0.096      *
    DUTIES15           0.135       0.030       0.000       0.076       0.194      *

 BULLY15  WITH
    PRESS15            0.239       0.029       0.000       0.179       0.294      *
    DUTIES15          -0.223       0.028       0.000      -0.277      -0.167      *

 PRESS15  WITH
    DUTIES15          -0.263       0.028       0.000      -0.316      -0.207      *

 Means
    PHY15              0.000       0.030       0.498      -0.059       0.058
    KES15              0.000       0.030       0.497      -0.057       0.058
    CHOICE15           0.000       0.030       0.497      -0.059       0.057
    BULLY15            0.001       0.030       0.493      -0.060       0.059
    DUTIES15           0.001       0.030       0.491      -0.056       0.060

 Variances
    PHY15              1.000       0.000       0.000       1.000       1.000
    KES15              1.000       0.000       0.000       1.000       1.000
    CHOICE15           1.000       0.000       0.000       1.000       1.000
    BULLY15            1.000       0.000       0.000       1.000       1.000
    PRESS15            1.000       0.000       0.000       1.000       1.000
    DUTIES15           1.000       0.000       0.000       1.000       1.000

Between Level

 S1         ON
    PRAFAC             0.005       0.399       0.495      -0.760       0.830
```

KESCAT16 ON						
PRAFAC	-0.569	0.258	0.023	-0.988	-0.011	*
PRESS15	-0.216	0.385	0.315	-0.845	0.552	
PRAFAC WITH						
PRESS15	0.239	0.177	0.100	-0.127	0.573	
Means						
PRAFAC	-0.166	0.128	0.102	-0.419	0.080	
PRESS15	9.296	1.393	0.000	6.942	12.328	*
Intercepts						
S1	-0.290	0.462	0.213	-1.440	0.533	
Variances						
PRAFAC	1.000	0.000	0.000	1.000	1.000	
PRESS15	1.000	0.000	0.000	1.000	1.000	
Residual Variances						
KESCAT16	0.412	0.252	0.000	0.021	0.895	*
S1	0.925	0.202	0.000	0.233	1.000	*

R-SQUARE

Within-Level R-Square Averaged Across Clusters

Variable	Estimate	Posterior S.D.	One-Tailed P-Value	95% C.I. Lower 2.5%	Upper 2.5%
KESCAT16	0.240	0.037	0.000	0.171	0.316

Between Level

Variable	Estimate	Posterior S.D.	One-Tailed P-Value	95% C.I. Lower 2.5%	Upper 2.5%
KESCAT16	0.588	0.252	0.000	0.105	0.979

Variable	Estimate	Posterior S.D.	One-Tailed P-Value	95% C.I. Lower 2.5%	Upper 2.5%
S1	0.075	0.202	0.000	0.000	0.767

TECHNICAL 1 OUTPUT

PARAMETER SPECIFICATION FOR WITHIN

TAU

KESCAT16
0

NU

KESCAT16	PHY15	KES15	CHOICE15	BULLY15
0	0	0	0	0

NU

PRESS15	DUTIES15
0	0

LAMBDA

	KESCAT16	PHY15	KES15	CHOICE15	BULLY15
KESCAT16	0	0	0	0	0
PHY15	0	0	0	0	0
KES15	0	0	0	0	0
CHOICE15	0	0	0	0	0
BULLY15	0	0	0	0	0
PRESS15	0	0	0	0	0
DUTIES15	0	0	0	0	0

LAMBDA

	PRESS15	DUTIES15
KESCAT16	0	0
PHY15	0	0
KES15	0	0
CHOICE15	0	0

BULLY15	0	0
PRESS15	0	0
DUTIES15	0	0

THETA

	KESCAT16	PHY15	KES15	CHOICE15	BULLY15
KESCAT16	0				
PHY15	0	0			
KES15	0	0	0		
CHOICE15	0	0	0	0	
BULLY15	0	0	0	0	0
PRESS15	0	0	0	0	0
DUTIES15	0	0	0	0	0

THETA

	PRESS15	DUTIES15
PRESS15	0	
DUTIES15	0	0

ALPHA

KESCAT16	PHY15	KES15	CHOICE15	BULLY15
0	1	2	3	4

ALPHA

PRESS15	DUTIES15
0	5

BETA

	KESCAT16	PHY15	KES15	CHOICE15	BULLY15
KESCAT16	0	6	7	8	9
PHY15	0	0	0	0	0
KES15	0	0	0	0	0
CHOICE15	0	0	0	0	0
BULLY15	0	0	0	0	0

```
PRESS15            0             0             0             0             0
DUTIES15           0             0             0             0             0


           BETA
              PRESS15       DUTIES15
              ________      ________
KESCAT16           0            10
PHY15              0             0
KES15              0             0
CHOICE15           0             0
BULLY15            0             0
PRESS15            0             0
DUTIES15           0             0


           PSI
              KESCAT16      PHY15         KES15         CHOICE15      BULLY15
              ________      ________      ________      ________      ________
KESCAT16           0
PHY15              0            11
KES15              0            12            13
CHOICE15           0            14            15            16
BULLY15            0            17            18            19            20
PRESS15            0            21            22            23            24
DUTIES15           0            26            27            28            29


           PSI
              PRESS15       DUTIES15
              ________      ________
PRESS15           25
DUTIES15          30            31


     PARAMETER SPECIFICATION FOR BETWEEN


           TAU
              KESCAT16
              ________
                  43
```

NU

KESCAT16	PRAFAC	PRESS15
0	0	0

LAMBDA

	S1	KESCAT16	PRAFAC	PRESS15
KESCAT16	0	0	0	0
PRAFAC	0	0	0	0
PRESS15	0	0	0	0

THETA

	KESCAT16	PRAFAC	PRESS15
KESCAT16	0		
PRAFAC	0	0	
PRESS15	0	0	0

ALPHA

S1	KESCAT16	PRAFAC	PRESS15
32	0	33	34

BETA

	S1	KESCAT16	PRAFAC	PRESS15
S1	0	0	35	0
KESCAT16	0	0	36	37
PRAFAC	0	0	0	0
PRESS15	0	0	0	0

PSI

	S1	KESCAT16	PRAFAC	PRESS15
S1	38			
KESCAT16	0	39		
PRAFAC	0	0	40	
PRESS15	0	0	41	42

STARTING VALUES FOR WITHIN

TAU

KESCAT16
0.000

NU

KESCAT16	PHY15	KES15	CHOICE15	BULLY15
0.000	0.000	0.000	0.000	0.000

NU

PRESS15	DUTIES15
0.000	0.000

LAMBDA

	KESCAT16	PHY15	KES15	CHOICE15	BULLY15
KESCAT16	1.000	0.000	0.000	0.000	0.000
PHY15	0.000	1.000	0.000	0.000	0.000
KES15	0.000	0.000	1.000	0.000	0.000
CHOICE15	0.000	0.000	0.000	1.000	0.000
BULLY15	0.000	0.000	0.000	0.000	1.000
PRESS15	0.000	0.000	0.000	0.000	0.000
DUTIES15	0.000	0.000	0.000	0.000	0.000

LAMBDA

	PRESS15	DUTIES15
KESCAT16	0.000	0.000
PHY15	0.000	0.000
KES15	0.000	0.000
CHOICE15	0.000	0.000
BULLY15	0.000	0.000
PRESS15	1.000	0.000

DUTIES15	0.000	1.000

THETA

	KESCAT16	PHY15	KES15	CHOICE15	BULLY15
KESCAT16	0.000				
PHY15	0.000	0.000			
KES15	0.000	0.000	0.000		
CHOICE15	0.000	0.000	0.000	0.000	
BULLY15	0.000	0.000	0.000	0.000	0.000
PRESS15	0.000	0.000	0.000	0.000	0.000
DUTIES15	0.000	0.000	0.000	0.000	0.000

THETA

	PRESS15	DUTIES15
PRESS15	0.000	
DUTIES15	0.000	0.000

ALPHA

KESCAT16	PHY15	KES15	CHOICE15	BULLY15
0.000	0.000	0.000	0.000	0.000

ALPHA

PRESS15	DUTIES15
0.000	0.000

BETA

	KESCAT16	PHY15	KES15	CHOICE15	BULLY15
KESCAT16	0.000	0.000	0.000	0.000	0.000
PHY15	0.000	0.000	0.000	0.000	0.000
KES15	0.000	0.000	0.000	0.000	0.000
CHOICE15	0.000	0.000	0.000	0.000	0.000
BULLY15	0.000	0.000	0.000	0.000	0.000
PRESS15	0.000	0.000	0.000	0.000	0.000
DUTIES15	0.000	0.000	0.000	0.000	0.000

BETA

	PRESS15	DUTIES15
KESCAT16	0.000	0.000
PHY15	0.000	0.000
KES15	0.000	0.000
CHOICE15	0.000	0.000
BULLY15	0.000	0.000
PRESS15	0.000	0.000
DUTIES15	0.000	0.000

PSI

	KESCAT16	PHY15	KES15	CHOICE15	BULLY15
KESCAT16	1.000				
PHY15	0.000	0.351			
KES15	0.000	0.000	0.157		
CHOICE15	0.000	0.000	0.000	0.597	
BULLY15	0.000	0.000	0.000	0.000	0.301
PRESS15	0.000	0.000	0.000	0.000	0.000
DUTIES15	0.000	0.000	0.000	0.000	0.000

PSI

	PRESS15	DUTIES15
PRESS15	0.580	
DUTIES15	0.000	0.516

STARTING VALUES FOR BETWEEN

TAU

KESCAT16
-0.352

NU

KESCAT16 PRAFAC PRESS15

0.000	0.000	0.000	

LAMBDA

	S1	KESCAT16	PRAFAC	PRESS15
KESCAT16	0.000	1.000	0.000	0.000
PRAFAC	0.000	0.000	1.000	0.000
PRESS15	0.000	0.000	0.000	1.000

THETA

	KESCAT16	PRAFAC	PRESS15
KESCAT16	0.000		
PRAFAC	0.000	0.000	
PRESS15	0.000	0.000	0.000

ALPHA

S1	KESCAT16	PRAFAC	PRESS15
0.000	0.000	-0.033	2.915

BETA

	S1	KESCAT16	PRAFAC	PRESS15
S1	0.000	0.000	0.000	0.000
KESCAT16	0.000	0.000	0.000	0.000
PRAFAC	0.000	0.000	0.000	0.000
PRESS15	0.000	0.000	0.000	0.000

PSI

	S1	KESCAT16	PRAFAC	PRESS15
S1	1.000			
KESCAT16	0.000	1.000		
PRAFAC	0.000	0.000	0.469	
PRESS15	0.000	0.000	0.000	0.580

PRIORS FOR ALL PARAMETERS	PRIOR MEAN	PRIOR VARIANCE	PRIOR STD. DEV.
Parameter 1~N(0.000,infinity)	0.0000	infinity	infinity
Parameter 2~N(0.000,infinity)	0.0000	infinity	infinity
Parameter 3~N(0.000,infinity)	0.0000	infinity	infinity
Parameter 4~N(0.000,infinity)	0.0000	infinity	infinity
Parameter 5~N(0.000,infinity)	0.0000	infinity	infinity
Parameter 6~N(0.000,5.000)	0.0000	5.0000	2.2361
Parameter 7~N(0.000,5.000)	0.0000	5.0000	2.2361
Parameter 8~N(0.000,5.000)	0.0000	5.0000	2.2361
Parameter 9~N(0.000,5.000)	0.0000	5.0000	2.2361
Parameter 10~N(0.000,5.000)	0.0000	5.0000	2.2361
Parameter 11~IW(1.000,7)	infinity	infinity	infinity
Parameter 12~IW(0.000,7)	infinity	infinity	infinity
Parameter 13~IW(1.000,7)	infinity	infinity	infinity
Parameter 14~IW(0.000,7)	infinity	infinity	infinity
Parameter 15~IW(0.000,7)	infinity	infinity	infinity
Parameter 16~IW(1.000,7)	infinity	infinity	infinity
Parameter 17~IW(0.000,7)	infinity	infinity	infinity
Parameter 18~IW(0.000,7)	infinity	infinity	infinity
Parameter 19~IW(0.000,7)	infinity	infinity	infinity
Parameter 20~IW(1.000,7)	infinity	infinity	infinity
Parameter 21~IW(0.000,7)	infinity	infinity	infinity
Parameter 22~IW(0.000,7)	infinity	infinity	infinity
Parameter 23~IW(0.000,7)	infinity	infinity	infinity
Parameter 24~IW(0.000,7)	infinity	infinity	infinity
Parameter 25~IW(1.000,7)	infinity	infinity	infinity
Parameter 26~IW(0.000,7)	infinity	infinity	infinity
Parameter 27~IW(0.000,7)	infinity	infinity	infinity
Parameter 28~IW(0.000,7)	infinity	infinity	infinity
Parameter 29~IW(0.000,7)	infinity	infinity	infinity
Parameter 30~IW(0.000,7)	infinity	infinity	infinity
Parameter 31~IW(1.000,7)	infinity	infinity	infinity
Parameter 32~N(0.000,infinity)	0.0000	infinity	infinity
Parameter 33~N(0.000,infinity)	0.0000	infinity	infinity
Parameter 34~N(0.000,infinity)	0.0000	infinity	infinity
Parameter 35~N(0.000,infinity)	0.0000	infinity	infinity
Parameter 36~N(0.000,5.000)	0.0000	5.0000	2.2361
Parameter 37~N(0.000,5.000)	0.0000	5.0000	2.2361
Parameter 38~IG(-1.000,0.000)	infinity	infinity	infinity
Parameter 39~IG(-1.000,0.000)	infinity	infinity	infinity
Parameter 40~IW(1.000,3)	infinity	infinity	infinity
Parameter 41~IW(0.000,3)	infinity	infinity	infinity

Parameter 42~IW(1.000,3)	infinity	infinity	infinity
Parameter 43~N(0.000,5.000)	0.0000	5.0000	2.2361

TECHNICAL 3 OUTPUT

ESTIMATED COVARIANCE MATRIX FOR PARAMETER ESTIMATES

	1	2	3	4	5
1	0.641572D-03				
2	0.941574D-04	0.275171D-03			
3	-0.758596D-04	-0.767209D-04	0.106791D-02		
4	0.357094D-04	0.651602D-04	-0.987995D-04	0.565754D-03	
5	-0.766828D-04	-0.488775D-04	0.103775D-03	-0.166960D-03	0.946576D-03
6	-0.643333D-04	0.135763D-05	0.947579D-04	0.117652D-04	-0.384498D-04
7	0.646692D-05	-0.778291D-05	-0.124910D-03	0.211935D-04	-0.310287D-04
8	-0.137355D-05	0.135930D-04	0.451441D-05	0.576934D-05	0.169513D-04
9	-0.475314D-04	0.462853D-04	-0.475539D-04	-0.398219D-04	-0.301441D-05
10	-0.382490D-04	-0.176089D-05	-0.167417D-04	-0.966743D-05	-0.241373D-07
11	0.163543D-04	0.598452D-05	-0.545989D-05	0.291466D-04	-0.624680D-04
12	-0.844192D-05	-0.286101D-05	0.419443D-05	0.252728D-05	-0.186964D-04
13	0.596321D-05	0.772697D-05	-0.130252D-04	0.412041D-05	-0.100458D-04
14	-0.492119D-05	0.880713D-05	-0.208508D-04	-0.445659D-05	-0.103704D-04
15	-0.762489D-05	-0.831957D-06	0.988986D-05	-0.617905D-06	-0.186449D-04
16	0.993567D-05	0.388630D-05	0.513494D-04	0.190015D-04	-0.242714D-04
17	-0.874031D-05	-0.385801D-05	-0.351757D-05	-0.925805D-05	-0.380025D-05
18	0.655972D-05	0.644186D-05	0.923378D-05	0.960187D-06	-0.107771D-04
19	0.123442D-04	0.647308D-05	0.998572D-05	0.237391D-04	-0.829287D-05
20	-0.216310D-04	0.205621D-05	-0.142309D-04	-0.292856D-04	0.318025D-04
21	-0.105409D-04	0.145695D-04	0.144293D-04	0.120333D-04	0.364800D-05
22	-0.267572D-05	0.459842D-05	0.492591D-04	-0.143339D-04	-0.902510D-06
23	-0.546411D-05	0.250184D-05	-0.392181D-04	-0.660481D-06	-0.167500D-04
24	-0.193934D-04	0.426036D-06	0.298789D-04	-0.324263D-04	-0.623607D-05
25	0.765874D-05	-0.212189D-04	0.614819D-04	-0.201216D-04	-0.768959D-05
26	0.194748D-04	-0.267734D-05	0.122443D-04	-0.943980D-06	0.577175D-05
27	0.762303D-05	-0.831505D-05	-0.793926D-05	0.240825D-04	-0.153136D-04
28	-0.226253D-04	-0.241395D-05	0.187111D-04	-0.119797D-04	0.430571D-04
29	0.203783D-04	-0.331959D-05	0.117996D-04	0.391591D-04	-0.894330D-05
30	-0.352694D-04	-0.131547D-04	0.211190D-04	0.265668D-04	0.234387D-04
31	0.377082D-04	0.272141D-04	-0.844485D-04	0.304117D-04	-0.280511D-04
32	0.986177D-06	-0.203137D-04	0.590426D-04	-0.392939D-04	0.111398D-04
33	0.107514D-04	0.288677D-05	0.176694D-03	-0.162577D-03	0.109262D-03
34	0.180978D-03	0.805283D-04	-0.153774D-03	0.186285D-03	-0.193759D-03

35	0.247992D-05	-0.126868D-04	0.870638D-05	0.192764D-05	-0.462921D-04
36	-0.454111D-04	0.532832D-06	0.501920D-04	0.298493D-04	0.544626D-04
37	0.474179D-04	0.468614D-04	0.599887D-04	-0.333591D-03	0.418367D-04
38	-0.149780D-04	-0.119236D-04	0.137162D-04	0.123251D-04	0.112064D-04
39	0.373609D-04	-0.128799D-04	0.111117D-04	0.606520D-05	0.593052D-05
40	0.127784D-03	0.190683D-03	0.374335D-04	0.616176D-04	-0.466317D-03
41	0.993850D-06	-0.284712D-06	0.687909D-05	0.325827D-04	-0.257679D-04
42	-0.101344D-04	-0.276835D-04	0.203605D-04	-0.729256D-05	0.338241D-05
43	0.114227D-03	0.116158D-03	0.194599D-03	-0.989537D-03	0.160515D-03

ESTIMATED COVARIANCE MATRIX FOR PARAMETER ESTIMATES

	6	7	8	9	10
6	0.293184D-02				
7	-0.372438D-03	0.920943D-02			
8	-0.154925D-05	0.321507D-03	0.167760D-02		
9	0.178734D-03	-0.441830D-03	0.265010D-03	0.374257D-02	
10	0.108715D-03	0.212469D-03	-0.200936D-03	0.362110D-03	0.200576D-02
11	-0.239134D-04	-0.103890D-03	-0.464536D-04	-0.101870D-03	-0.599216D-04
12	0.224273D-05	0.398586D-04	-0.118296D-04	-0.496243D-04	-0.280263D-04
13	-0.144715D-04	0.380691D-04	-0.208935D-04	-0.457059D-04	-0.332786D-04
14	-0.797419D-04	0.312957D-04	0.536245D-05	0.987295D-05	0.892277D-05
15	-0.257373D-04	0.334579D-04	0.429944D-04	-0.294706D-06	0.394857D-04
16	0.110345D-03	-0.929896D-04	-0.116506D-03	0.102847D-03	0.245150D-04
17	-0.171580D-04	0.191115D-04	0.251561D-05	-0.266098D-04	0.429113D-04
18	-0.167157D-04	-0.255822D-04	0.255011D-06	-0.860451D-05	0.167399D-04
19	0.782600D-05	-0.221239D-04	0.127322D-04	0.198706D-04	-0.104776D-04
20	-0.311592D-04	0.253100D-04	0.179025D-04	0.566978D-05	-0.156712D-04
21	0.178283D-04	0.107718D-04	-0.530061D-04	-0.663345D-04	0.143479D-04
22	-0.144019D-04	-0.217194D-04	-0.138327D-04	-0.572482D-05	-0.241713D-04
23	-0.672459D-04	-0.101695D-03	0.321881D-04	-0.341419D-06	-0.464446D-04
24	0.136468D-04	-0.709956D-04	-0.359684D-04	-0.116759D-04	-0.262742D-04
25	0.196868D-04	-0.100978D-03	-0.171528D-04	0.591445D-04	-0.338231D-04
26	-0.271981D-05	0.987527D-05	0.264813D-04	0.314781D-04	-0.268682D-04
27	0.158781D-04	0.421111D-04	0.257895D-04	-0.909601D-05	-0.420992D-05
28	0.829619D-04	-0.102318D-03	-0.184482D-04	-0.540914D-05	0.205970D-04
29	0.394358D-04	0.127415D-03	-0.137349D-05	0.772212D-05	0.368192D-04
30	-0.269293D-04	0.224491D-03	0.307749D-04	0.930992D-05	-0.590482D-05
31	-0.249260D-04	-0.355352D-03	-0.553100D-04	-0.205618D-04	0.588429D-04
32	-0.316702D-03	-0.420092D-03	0.990856D-04	-0.486113D-03	0.359262D-03
33	0.417530D-03	-0.415048D-03	-0.720447D-04	-0.251900D-03	0.874147D-04
34	-0.820899D-04	-0.630033D-04	0.870164D-04	0.213389D-03	0.929547D-04
35	-0.915780D-05	-0.711713D-04	-0.121354D-03	0.958890D-04	-0.148176D-04

36	0.320884D-03	0.184442D-03	-0.164139D-03	-0.173185D-04	0.717299D-04
37	-0.180425D-02	0.157392D-02	0.189528D-03	-0.576064D-03	0.108791D-03
38	0.479325D-04	0.344398D-03	0.591270D-05	0.131098D-05	0.212939D-04
39	0.746428D-04	0.473403D-03	0.360653D-04	-0.106018D-03	0.218838D-04
40	0.599208D-04	0.202961D-03	0.361650D-03	0.416832D-03	0.116542D-03
41	-0.116382D-03	-0.381140D-04	-0.153855D-05	-0.673110D-04	-0.520770D-04
42	-0.181245D-04	0.138860D-04	0.409055D-04	-0.330476D-04	0.532399D-04
43	-0.540393D-02	0.342335D-02	0.603098D-03	-0.189067D-02	0.216674D-03

ESTIMATED COVARIANCE MATRIX FOR PARAMETER ESTIMATES

	11	12	13	14	15
11	0.887525D-03				
12	0.126315D-03	0.198051D-03			
13	0.842420D-05	0.578666D-04	0.174205D-03		
14	-0.140225D-03	-0.755801D-04	-0.122975D-04	0.780693D-03	
15	-0.975053D-05	-0.362713D-04	-0.460245D-04	0.118354D-03	0.333335D-03
16	-0.771691D-06	-0.165143D-04	0.132642D-04	-0.203727D-03	-0.215419D-03
17	0.489456D-04	0.459013D-04	0.239485D-04	-0.998604D-04	-0.221209D-04
18	0.491739D-05	0.123025D-04	0.342818D-04	-0.111244D-04	-0.426404D-04
19	0.300256D-05	-0.154593D-04	-0.123516D-04	0.427560D-04	0.884413D-04
20	-0.382846D-04	-0.819919D-05	0.997671D-05	0.924782D-05	-0.130562D-04
21	0.141380D-03	0.552407D-04	0.213870D-04	-0.152553D-03	-0.355704D-04
22	0.803228D-05	0.349859D-04	0.337318D-04	-0.315408D-04	-0.546534D-04
23	0.855818D-05	-0.234757D-04	-0.184807D-04	0.153809D-03	0.108065D-03
24	-0.380489D-04	0.129358D-04	0.742781D-05	-0.187087D-04	-0.265127D-04
25	-0.183592D-04	0.212951D-04	0.161481D-05	-0.660361D-04	-0.407533D-04
26	-0.715288D-04	-0.682985D-04	-0.777802D-05	0.130640D-03	0.335927D-04
27	-0.806245D-06	0.612113D-06	-0.234994D-04	0.277061D-04	0.393078D-04
28	0.710732D-05	0.109429D-05	0.243205D-04	-0.792643D-04	-0.122628D-03
29	0.400524D-04	0.946371D-05	0.671494D-05	0.135379D-04	0.311538D-04
30	0.250578D-04	-0.842622D-05	0.565433D-07	0.873029D-04	0.276019D-04
31	-0.255498D-04	-0.349066D-04	-0.144370D-04	-0.450988D-04	-0.308948D-04
32	-0.307518D-04	-0.312116D-05	-0.402313D-04	0.276386D-05	0.338123D-04
33	-0.149962D-03	-0.371638D-05	-0.263751D-04	0.145499D-03	-0.592574D-05
34	0.218913D-04	0.542271D-05	0.182683D-04	0.316357D-04	0.126207D-04
35	-0.184552D-05	0.378191D-05	0.385798D-04	0.124464D-04	-0.220400D-04
36	-0.386048D-04	0.115406D-05	0.297199D-04	-0.245478D-04	-0.292618D-06
37	0.302488D-05	-0.358225D-04	0.118136D-03	0.201051D-03	-0.236517D-03
38	0.173749D-04	-0.216127D-04	0.679267D-05	0.442461D-04	-0.342093D-05
39	-0.393026D-04	-0.173867D-04	-0.162192D-04	-0.107005D-04	-0.460147D-05
40	-0.216565D-04	0.359639D-05	-0.189944D-04	0.716257D-04	0.266647D-03
41	-0.688670D-04	-0.101105D-04	0.533734D-04	0.645062D-04	0.284850D-04

42	-0.136340D-04	-0.154358D-04	-0.690826D-05	-0.994170D-06	0.249656D-04
43	0.120226D-03	-0.682932D-04	0.354797D-03	0.569204D-03	-0.718261D-03

ESTIMATED COVARIANCE MATRIX FOR PARAMETER ESTIMATES

	16	17	18	19	20
16	0.257082D-02				
17	0.505337D-04	0.381941D-03			
18	0.136158D-04	0.577458D-04	0.166347D-03		
19	-0.223079D-03	-0.691168D-04	-0.623631D-04	0.666200D-03	
20	0.972218D-05	0.383340D-04	0.918082D-04	-0.142195D-03	0.658335D-03
21	0.294912D-04	0.131325D-03	0.411117D-04	-0.263392D-04	0.392497D-04
22	0.183222D-04	0.488605D-04	0.693634D-04	-0.512359D-04	0.457799D-04
23	-0.440711D-03	-0.392174D-04	-0.487351D-04	0.256584D-03	-0.416361D-04
24	0.551671D-04	0.737845D-04	0.471463D-04	-0.126351D-03	0.191271D-03
25	0.661000D-04	0.828301D-04	0.292131D-04	-0.507228D-04	0.142176D-04
26	-0.277394D-04	-0.995292D-04	-0.229060D-04	0.156483D-04	-0.360283D-04
27	-0.419579D-04	-0.249558D-04	-0.557696D-04	0.304522D-04	-0.369156D-04
28	0.300520D-03	0.239874D-04	0.444862D-04	-0.188927D-03	0.447175D-04
29	-0.317056D-05	-0.238110D-04	-0.566543D-04	0.128746D-03	-0.196339D-03
30	-0.652069D-04	-0.373093D-04	-0.155792D-04	0.576465D-04	-0.950863D-04
31	0.136227D-03	0.252309D-04	0.206219D-04	-0.256184D-04	0.684995D-04
32	0.191319D-04	0.862070D-05	-0.907809D-05	-0.299635D-05	-0.198602D-04
33	-0.250120D-03	-0.835809D-04	-0.567577D-05	0.231114D-04	0.548434D-04
34	-0.576203D-04	-0.577462D-04	0.283549D-04	-0.215622D-04	-0.685504D-04
35	-0.155173D-04	-0.156788D-04	0.231189D-04	0.242060D-04	0.286953D-04
36	0.132927D-03	-0.108266D-04	0.994702D-05	0.142477D-04	-0.689885D-04
37	-0.356710D-03	0.172135D-04	0.190414D-04	0.443007D-04	0.324283D-03
38	-0.237105D-04	0.566069D-05	0.469455D-05	0.254696D-04	-0.121580D-04
39	-0.111656D-03	-0.975110D-06	-0.271054D-05	0.877263D-05	0.271832D-04
40	-0.589730D-03	-0.163093D-04	0.497755D-04	0.413137D-04	0.159412D-03
41	0.210889D-03	0.177788D-04	0.574261D-04	-0.634214D-04	-0.955872D-06
42	0.155381D-04	0.182395D-04	0.212141D-05	0.600827D-05	-0.302318D-04
43	-0.111627D-02	0.661835D-04	0.878806D-04	0.106826D-03	0.939082D-03

ESTIMATED COVARIANCE MATRIX FOR PARAMETER ESTIMATES

	21	22	23	24	25
21	0.759301D-03				
22	0.142069D-03	0.346361D-03			
23	-0.149373D-03	-0.140677D-03	0.135262D-02		
24	0.609890D-04	0.105350D-03	-0.184507D-03	0.648268D-03	

25	0.255675D-03	0.169339D-03	-0.457610D-03	0.331290D-03	0.234075D-02
26	-0.183126D-03	-0.616617D-04	0.672533D-04	-0.397121D-04	-0.710777D-04
27	-0.314852D-04	-0.926213D-04	0.390699D-04	-0.368603D-04	-0.192248D-04
28	0.543947D-04	0.613490D-04	-0.358624D-03	0.723984D-04	0.383670D-04
29	-0.265920D-04	-0.397581D-04	0.101200D-03	-0.169803D-03	-0.763616D-04
30	-0.113072D-03	-0.106982D-03	0.243663D-03	-0.248704D-03	-0.645228D-03
31	-0.117662D-04	0.292121D-04	-0.258248D-04	0.388195D-04	0.139215D-03
32	0.179222D-06	0.312595D-04	0.525874D-04	0.903261D-05	0.136747D-03
33	0.901513D-04	-0.778031D-04	-0.456790D-04	-0.393329D-04	-0.139705D-04
34	0.570675D-04	0.683818D-04	-0.105125D-03	0.627584D-04	0.533273D-04
35	0.356582D-04	0.261765D-04	-0.230818D-04	0.686534D-04	0.660561D-04
36	-0.618641D-05	0.298657D-04	-0.583637D-04	-0.765142D-05	0.477245D-04
37	0.279075D-03	-0.358430D-04	0.628503D-03	-0.125110D-03	-0.629166D-03
38	0.330726D-05	0.437249D-05	-0.268218D-04	-0.302449D-04	0.418506D-04
39	-0.262622D-04	-0.159756D-04	0.665673D-04	-0.259136D-05	-0.114099D-04
40	0.290339D-03	-0.481631D-04	0.147076D-03	0.141495D-04	0.260653D-03
41	0.102125D-03	0.298599D-04	-0.136554D-03	-0.983071D-06	0.848529D-04
42	-0.158599D-04	-0.780242D-05	0.871271D-05	-0.131597D-04	-0.588079D-04
43	0.848539D-03	-0.904328D-04	0.189263D-02	-0.320667D-03	-0.184158D-02

ESTIMATED COVARIANCE MATRIX FOR PARAMETER ESTIMATES

	26	27	28	29	30
26	0.703840D-03				
27	0.967100D-04	0.314456D-03			
28	-0.104327D-03	-0.104667D-03	0.114265D-02		
29	0.468243D-04	0.970762D-04	-0.155100D-03	0.580253D-03	
30	0.124333D-03	0.113577D-03	-0.222881D-03	0.246956D-03	0.119809D-02
31	-0.403955D-04	-0.163541D-03	0.289111D-03	-0.305639D-03	-0.485607D-03
32	-0.241713D-04	-0.166082D-04	0.130367D-04	-0.469822D-05	-0.853891D-04
33	-0.103120D-03	0.813401D-05	0.132212D-03	-0.426688D-04	-0.604500D-04
34	0.192720D-04	0.639338D-05	-0.257719D-04	0.538588D-04	-0.462232D-04
35	-0.422149D-04	-0.441371D-04	-0.733732D-04	-0.206606D-04	-0.313071D-04
36	-0.156995D-04	0.884162D-05	-0.622188D-04	0.458767D-04	-0.643347D-04
37	0.107444D-03	0.435919D-04	0.336430D-03	-0.145588D-03	0.501712D-03
38	0.193750D-04	-0.197891D-05	-0.580363D-04	0.366087D-04	0.313537D-04
39	0.375538D-05	-0.630101D-05	-0.246629D-04	0.115824D-05	0.201035D-04
40	0.249356D-03	-0.657878D-04	-0.385057D-03	0.383516D-04	0.312362D-04
41	-0.348206D-04	-0.308806D-04	-0.529229D-04	0.445600D-04	0.850826D-05
42	-0.350630D-04	-0.507833D-06	-0.140194D-04	0.793654D-05	0.396111D-04
43	0.301246D-03	0.146082D-03	0.993469D-03	-0.431582D-03	0.137232D-02

ESTIMATED COVARIANCE MATRIX FOR PARAMETER ESTIMATES

	31	32	33	34	35
31	0.194632D-02				
32	0.963954D-04	0.316195D-02			
33	0.307149D-03	0.146490D-03	0.227567D-01		
34	0.474566D-04	0.329502D-04	0.137579D-02	0.377986D-02	
35	-0.474640D-04	0.172646D-03	0.208625D-03	-0.829919D-05	0.243268D-02
36	-0.977906D-04	0.105353D-03	0.399277D-03	0.141266D-03	0.286334D-03
37	-0.302494D-03	-0.189290D-02	-0.165995D-02	-0.726642D-03	-0.221744D-03
38	-0.268127D-04	-0.329788D-03	-0.362088D-04	-0.225633D-04	-0.421167D-04
39	0.293571D-04	-0.129385D-04	-0.354332D-04	0.394663D-04	0.174398D-04
40	0.478891D-05	-0.417720D-03	-0.166987D-02	-0.722693D-03	-0.247263D-03
41	-0.100592D-03	0.402817D-04	-0.622940D-03	-0.879528D-04	0.133172D-03
42	-0.397903D-04	0.487445D-04	0.999042D-04	-0.219764D-03	-0.256264D-05
43	-0.855449D-03	-0.569790D-02	-0.438801D-02	-0.219062D-02	-0.747278D-03

ESTIMATED COVARIANCE MATRIX FOR PARAMETER ESTIMATES

	36	37	38	39	40
36	0.340098D-02				
37	-0.756928D-02	0.999126D-01			
38	-0.113094D-03	0.764487D-03	0.892741D-03		
39	-0.179449D-03	-0.791910D-03	0.593574D-04	0.121167D-02	
40	0.151420D-03	-0.317024D-02	0.225605D-03	0.238510D-03	0.702785D-01
41	0.207759D-03	-0.117429D-02	-0.824916D-04	-0.336659D-05	0.445645D-02
42	0.721148D-04	-0.818279D-03	0.489373D-06	0.107057D-04	0.466722D-03
43	-0.222757D-01	0.291515D+00	0.207789D-02	-0.219618D-02	-0.992975D-02

ESTIMATED COVARIANCE MATRIX FOR PARAMETER ESTIMATES

	41	42	43
41	0.515653D-02		
42	0.557125D-03	0.866577D-03	
43	-0.354080D-02	-0.242291D-02	0.853834D+00

ESTIMATED CORRELATION MATRIX FOR PARAMETER ESTIMATES

	1	2	3	4	5
1	1.000				
2	0.224	1.000			

3	-0.092	-0.142	1.000		
4	0.059	0.165	-0.127	1.000	
5	-0.098	-0.096	0.103	-0.228	1.000
6	-0.047	0.002	0.054	0.009	-0.023
7	0.003	-0.005	-0.040	0.009	-0.011
8	-0.001	0.020	0.003	0.006	0.013
9	-0.031	0.046	-0.024	-0.027	-0.002
10	-0.034	-0.002	-0.011	-0.009	0.000
11	0.022	0.012	-0.006	0.041	-0.068
12	-0.024	-0.012	0.009	0.008	-0.043
13	0.018	0.035	-0.030	0.013	-0.025
14	-0.007	0.019	-0.023	-0.007	-0.012
15	-0.016	-0.003	0.017	-0.001	-0.033
16	0.008	0.005	0.031	0.016	-0.016
17	-0.018	-0.012	-0.006	-0.020	-0.006
18	0.020	0.030	0.022	0.003	-0.027
19	0.019	0.015	0.012	0.039	-0.010
20	-0.033	0.005	-0.017	-0.048	0.040
21	-0.015	0.032	0.016	0.018	0.004
22	-0.006	0.015	0.081	-0.032	-0.002
23	-0.006	0.004	-0.033	-0.001	-0.015
24	-0.030	0.001	0.036	-0.054	-0.008
25	0.006	-0.026	0.039	-0.017	-0.005
26	0.029	-0.006	0.014	-0.001	0.007
27	0.017	-0.028	-0.014	0.057	-0.028
28	-0.026	-0.004	0.017	-0.015	0.041
29	0.033	-0.008	0.015	0.068	-0.012
30	-0.040	-0.023	0.019	0.032	0.022
31	0.034	0.037	-0.059	0.029	-0.021
32	0.001	-0.022	0.032	-0.029	0.006
33	0.003	0.001	0.036	-0.045	0.024
34	0.116	0.079	-0.077	0.127	-0.102
35	0.002	-0.016	0.005	0.002	-0.031
36	-0.031	0.001	0.026	0.022	0.030
37	0.006	0.009	0.006	-0.044	0.004
38	-0.020	-0.024	0.014	0.017	0.012
39	0.042	-0.022	0.010	0.007	0.006
40	0.019	0.043	0.004	0.010	-0.057
41	0.001	0.000	0.003	0.019	-0.012
42	-0.014	-0.057	0.021	-0.010	0.004
43	0.005	0.008	0.006	-0.045	0.006

ESTIMATED CORRELATION MATRIX FOR PARAMETER ESTIMATES

	6	7	8	9	10
6	1.000				
7	-0.072	1.000			
8	-0.001	0.082	1.000		
9	0.054	-0.075	0.106	1.000	
10	0.045	0.049	-0.110	0.132	1.000
11	-0.015	-0.036	-0.038	-0.056	-0.045
12	0.003	0.030	-0.021	-0.058	-0.044
13	-0.020	0.030	-0.039	-0.057	-0.056
14	-0.053	0.012	0.005	0.006	0.007
15	-0.026	0.019	0.057	0.000	0.048
16	0.040	-0.019	-0.056	0.033	0.011
17	-0.016	0.010	0.003	-0.022	0.049
18	-0.024	-0.021	0.000	-0.011	0.029
19	0.006	-0.009	0.012	0.013	-0.009
20	-0.022	0.010	0.017	0.004	-0.014
21	0.012	0.004	-0.047	-0.039	0.012
22	-0.014	-0.012	-0.018	-0.005	-0.029
23	-0.034	-0.029	0.021	0.000	-0.028
24	0.010	-0.029	-0.034	-0.007	-0.023
25	0.008	-0.022	-0.009	0.020	-0.016
26	-0.002	0.004	0.024	0.019	-0.023
27	0.017	0.025	0.036	-0.008	-0.005
28	0.045	-0.032	-0.013	-0.003	0.014
29	0.030	0.055	-0.001	0.005	0.034
30	-0.014	0.068	0.022	0.004	-0.004
31	-0.010	-0.084	-0.031	-0.008	0.030
32	-0.104	-0.078	0.043	-0.141	0.143
33	0.051	-0.029	-0.012	-0.027	0.013
34	-0.025	-0.011	0.035	0.057	0.034
35	-0.003	-0.015	-0.060	0.032	-0.007
36	0.102	0.033	-0.069	-0.005	0.027
37	-0.105	0.052	0.015	-0.030	0.008
38	0.030	0.120	0.005	0.001	0.016
39	0.040	0.142	0.025	-0.050	0.014
40	0.004	0.008	0.033	0.026	0.010
41	-0.030	-0.006	-0.001	-0.015	-0.016
42	-0.011	0.005	0.034	-0.018	0.040
43	-0.108	0.039	0.016	-0.033	0.005

ESTIMATED CORRELATION MATRIX FOR PARAMETER ESTIMATES

11	12	13	14	15

11	1.000				
12	0.301	1.000			
13	0.021	0.312	1.000		
14	-0.168	-0.192	-0.033	1.000	
15	-0.018	-0.141	-0.191	0.232	1.000
16	-0.001	-0.023	0.020	-0.144	-0.233
17	0.084	0.167	0.093	-0.183	-0.062
18	0.013	0.068	0.201	-0.031	-0.181
19	0.004	-0.043	-0.036	0.059	0.188
20	-0.050	-0.023	0.029	0.013	-0.028
21	0.172	0.142	0.059	-0.198	-0.071
22	0.014	0.134	0.137	-0.061	-0.161
23	0.008	-0.045	-0.038	0.150	0.161
24	-0.050	0.036	0.022	-0.026	-0.057
25	-0.013	0.031	0.003	-0.049	-0.046
26	-0.091	-0.183	-0.022	0.176	0.069
27	-0.002	0.002	-0.100	0.056	0.121
28	0.007	0.002	0.055	-0.084	-0.199
29	0.056	0.028	0.021	0.020	0.071
30	0.024	-0.017	0.000	0.090	0.044
31	-0.019	-0.056	-0.025	-0.037	-0.038
32	-0.018	-0.004	-0.054	0.002	0.033
33	-0.033	-0.002	-0.013	0.035	-0.002
34	0.012	0.006	0.023	0.018	0.011
35	-0.001	0.005	0.059	0.009	-0.024
36	-0.022	0.001	0.039	-0.015	0.000
37	0.000	-0.008	0.028	0.023	-0.041
38	0.020	-0.051	0.017	0.053	-0.006
39	-0.038	-0.035	-0.035	-0.011	-0.007
40	-0.003	0.001	-0.005	0.010	0.055
41	-0.032	-0.010	0.056	0.032	0.022
42	-0.016	-0.037	-0.018	-0.001	0.046
43	0.004	-0.005	0.029	0.022	-0.043

ESTIMATED CORRELATION MATRIX FOR PARAMETER ESTIMATES

	16	17	18	19	20
16	1.000				
17	0.051	1.000			
18	0.021	0.229	1.000		
19	-0.170	-0.137	-0.187	1.000	
20	0.007	0.076	0.277	-0.215	1.000

21	0.021	0.244	0.116	-0.037	0.056
22	0.019	0.134	0.289	-0.107	0.096
23	-0.236	-0.055	-0.103	0.270	-0.044
24	0.043	0.148	0.144	-0.192	0.293
25	0.027	0.088	0.047	-0.041	0.011
26	-0.021	-0.192	-0.067	0.023	-0.053
27	-0.047	-0.072	-0.244	0.067	-0.081
28	0.175	0.036	0.102	-0.217	0.052
29	-0.003	-0.051	-0.182	0.207	-0.318
30	-0.037	-0.055	-0.035	0.065	-0.107
31	0.061	0.029	0.036	-0.022	0.061
32	0.007	0.008	-0.013	-0.002	-0.014
33	-0.033	-0.028	-0.003	0.006	0.014
34	-0.018	-0.048	0.036	-0.014	-0.043
35	-0.006	-0.016	0.036	0.019	0.023
36	0.045	-0.009	0.013	0.009	-0.046
37	-0.022	0.003	0.005	0.005	0.040
38	-0.016	0.010	0.012	0.033	-0.016
39	-0.063	-0.001	-0.006	0.010	0.030
40	-0.044	-0.003	0.015	0.006	0.023
41	0.058	0.013	0.062	-0.034	-0.001
42	0.010	0.032	0.006	0.008	-0.040
43	-0.024	0.004	0.007	0.004	0.040

ESTIMATED CORRELATION MATRIX FOR PARAMETER ESTIMATES

	21	22	23	24	25
21	1.000				
22	0.277	1.000			
23	-0.147	-0.206	1.000		
24	0.087	0.222	-0.197	1.000	
25	0.192	0.188	-0.257	0.269	1.000
26	-0.250	-0.125	0.069	-0.059	-0.055
27	-0.064	-0.281	0.060	-0.082	-0.022
28	0.058	0.098	-0.288	0.084	0.023
29	-0.040	-0.089	0.114	-0.277	-0.066
30	-0.119	-0.166	0.191	-0.282	-0.385
31	-0.010	0.036	-0.016	0.035	0.065
32	0.000	0.030	0.025	0.006	0.050
33	0.022	-0.028	-0.008	-0.010	-0.002
34	0.034	0.060	-0.046	0.040	0.018
35	0.026	0.029	-0.013	0.055	0.028
36	-0.004	0.028	-0.027	-0.005	0.017

37	0.032	-0.006	0.054	-0.016	-0.041
38	0.004	0.008	-0.024	-0.040	0.029
39	-0.027	-0.025	0.052	-0.003	-0.007
40	0.040	-0.010	0.015	0.002	0.020
41	0.052	0.022	-0.052	-0.001	0.024
42	-0.020	-0.014	0.008	-0.018	-0.041
43	0.033	-0.005	0.056	-0.014	-0.041

ESTIMATED CORRELATION MATRIX FOR PARAMETER ESTIMATES

	26	27	28	29	30
26	1.000				
27	0.206	1.000			
28	-0.116	-0.175	1.000		
29	0.073	0.227	-0.190	1.000	
30	0.135	0.185	-0.190	0.296	1.000
31	-0.035	-0.209	0.194	-0.288	-0.318
32	-0.016	-0.017	0.007	-0.003	-0.044
33	-0.026	0.003	0.026	-0.012	-0.012
34	0.012	0.006	-0.012	0.036	-0.022
35	-0.032	-0.050	-0.044	-0.017	-0.018
36	-0.010	0.009	-0.032	0.033	-0.032
37	0.013	0.008	0.031	-0.019	0.046
38	0.024	-0.004	-0.057	0.051	0.030
39	0.004	-0.010	-0.021	0.001	0.017
40	0.035	-0.014	-0.043	0.006	0.003
41	-0.018	-0.024	-0.022	0.026	0.003
42	-0.045	-0.001	-0.014	0.011	0.039
43	0.012	0.009	0.032	-0.019	0.043

ESTIMATED CORRELATION MATRIX FOR PARAMETER ESTIMATES

	31	32	33	34	35
31	1.000				
32	0.039	1.000			
33	0.046	0.017	1.000		
34	0.017	0.010	0.148	1.000	
35	-0.022	0.062	0.028	-0.003	1.000
36	-0.038	0.032	0.045	0.039	0.100
37	-0.022	-0.106	-0.035	-0.037	-0.014
38	-0.020	-0.196	-0.008	-0.012	-0.029
39	0.019	-0.007	-0.007	0.018	0.010

40	0.000	-0.028	-0.042	-0.044	-0.019
41	-0.032	0.010	-0.058	-0.020	0.038
42	-0.031	0.029	0.022	-0.121	-0.002
43	-0.021	-0.110	-0.031	-0.039	-0.016

ESTIMATED CORRELATION MATRIX FOR PARAMETER ESTIMATES

	36	37	38	39	40
36	1.000				
37	-0.411	1.000			
38	-0.065	0.081	1.000		
39	-0.088	-0.072	0.057	1.000	
40	0.010	-0.038	0.028	0.026	1.000
41	0.050	-0.052	-0.038	-0.001	0.234
42	0.042	-0.088	0.001	0.010	0.060
43	-0.413	0.998	0.075	-0.068	-0.041

ESTIMATED CORRELATION MATRIX FOR PARAMETER ESTIMATES

	41	42	43
41	1.000		
42	0.264	1.000	
43	-0.053	-0.089	1.000

TECHNICAL 8 OUTPUT

TECHNICAL 8 OUTPUT FOR BAYES ESTIMATION

CHAIN	BSEED
1	0
2	285380
3	253358

ITERATION	POTENTIAL SCALE REDUCTION	PARAMETER WITH HIGHEST PSR
100	1.053	38
200	1.021	35
300	1.022	38
400	1.006	34
500	1.005	28

600	1.007	39
700	1.002	39
800	1.003	39
900	1.007	39
1000	1.003	39

Reduced psychological distress, role clarity interaction

SUMMARY OF DATA

Number of clusters 58

Size (s)	Cluster ID with Size s
1	166
2	74 205
3	106 182 165
4	93 170 22 192 4
5	65 71 50 84
6	80 72
7	40 107 61 75
8	169 30 173 228
9	144 81 83 181
10	69 145 36 68
11	108 116 174 154
12	206 162
13	226 114
14	2
15	152 98
18	156
19	202
21	92 6
24	178
25	142 122
32	24
37	204
47	209
53	125
67	217
174	110
210	100

COVARIANCE COVERAGE OF DATA

Minimum covariance coverage value 0.100

Number of missing data patterns 5

PROPORTION OF DATA PRESENT

Covariance Coverage

	KESCAT16	PRAFAC	PHY15	KES15	CHOICE15
KESCAT16	1.000				
PRAFAC	1.000	1.000			
PHY15	1.000	1.000	1.000		
KES15	1.000	1.000	1.000	1.000	
CHOICE15	0.990	0.990	0.990	0.990	0.990
BULLY15	0.990	0.990	0.990	0.990	0.989
PRESS15	0.990	0.990	0.990	0.990	0.989
DUTIES15	0.991	0.991	0.991	0.991	0.990

Covariance Coverage

	BULLY15	PRESS15	DUTIES15
BULLY15	0.990		
PRESS15	0.989	0.990	
DUTIES15	0.990	0.990	0.991

UNIVARIATE PROPORTIONS AND COUNTS FOR CATEGORICAL VARIABLES

KESCAT16

Category 1	0.347	386.000
Category 2	0.653	727.000

UNIVARIATE SAMPLE STATISTICS

UNIVARIATE HIGHER-ORDER MOMENT DESCRIPTIVE STATISTICS

Variable/ Sample Size	Mean/ Variance	Skewness/ Kurtosis	Minimum/ Maximum	% with Min/Max	Percentiles 20%/60%	40%/80%	Median
PRAFAC	-0.185	0.245	-2.338	1.72%	-1.448	-0.527	-0.318
58.000	1.345	-0.636	2.338	1.72%	0.080	0.752	
PHY15	0.000	0.390	-1.412	11.50%	-0.412	-0.412	-0.412

```
        1113.000       0.702       0.082       2.588    1.08%       0.588      0.588
     KES15             0.000       1.366      -0.556   20.75%      -0.556     -0.222     -0.222
        1113.000       0.313       1.871       2.278    0.63%      -0.056      0.444
     CHOICE15          0.000      -0.116      -1.814   15.06%      -0.814      0.186      0.186
        1102.000       1.193      -0.747       2.186    4.45%       0.186      1.186
     BULLY15           0.000       1.887      -0.426   71.96%      -0.426     -0.426     -0.426
        1102.000       0.602       3.132       3.574    0.45%      -0.426      0.574
     PRESS15           0.000       0.096      -1.915    9.89%      -0.915      0.085      0.085
        1102.000       1.160      -0.512       2.085    8.35%       0.085      1.085
     DUTIES15          3.672      -0.677       1.000    3.54%       3.000      4.000      4.000
        1103.000       1.031       0.049       5.000   20.22%       4.000      5.000


THE MODEL ESTIMATION TERMINATED NORMALLY

     USE THE FBITERATIONS OPTION TO INCREASE THE NUMBER OF ITERATIONS BY A FACTOR
     OF AT LEAST TWO TO CHECK CONVERGENCE AND THAT THE PSR VALUE DOES NOT INCREASE.



MODEL FIT INFORMATION

Number of Free Parameters                              43



MODEL RESULTS

                                Posterior  One-Tailed         95% C.I.
                    Estimate       S.D.      P-Value   Lower 2.5%  Upper 2.5%  Significance

Within Level

 KESCAT16   ON
    PHY15              0.163       0.054      0.002       0.057       0.270      *
    CHOICE15           0.027       0.041      0.252      -0.049       0.112
    PRESS15           -0.042       0.041      0.162      -0.119       0.038
    BULLY15            0.111       0.061      0.032      -0.003       0.232
    KES15              0.807       0.095      0.000       0.627       0.993      *

 KES15    WITH
    PHY15              0.107       0.014      0.000       0.079       0.135      *
    CHOICE15          -0.095       0.018      0.000      -0.133      -0.061      *
    BULLY15            0.072       0.013      0.000       0.049       0.098      *
```

PRESS15	0.062	0.019	0.001	0.028	0.099	*
DUTIES15	-0.071	0.018	0.000	-0.105	-0.036	*
PHY15 WITH						
CHOICE15	-0.098	0.028	0.001	-0.152	-0.043	*
BULLY15	0.041	0.020	0.017	0.002	0.077	*
PRESS15	0.118	0.028	0.000	0.068	0.176	*
DUTIES15	-0.055	0.026	0.023	-0.108	0.000	*
CHOICE15 WITH						
BULLY15	-0.137	0.026	0.000	-0.189	-0.086	*
PRESS15	-0.196	0.037	0.000	-0.271	-0.124	*
DUTIES15	0.155	0.035	0.000	0.087	0.223	*
BULLY15 WITH						
PRESS15	0.201	0.026	0.000	0.151	0.252	*
DUTIES15	-0.169	0.024	0.000	-0.218	-0.122	*
PRESS15 WITH						
DUTIES15	-0.282	0.035	0.000	-0.351	-0.215	*
Means						
PHY15	-0.001	0.025	0.477	-0.049	0.049	
KES15	0.000	0.017	0.499	-0.032	0.032	
CHOICE15	-0.001	0.033	0.491	-0.067	0.062	
BULLY15	0.000	0.024	0.498	-0.046	0.048	
PRESS15	0.000	0.033	0.496	-0.064	0.065	
Variances						
PHY15	0.704	0.030	0.000	0.648	0.765	*
KES15	0.314	0.013	0.000	0.289	0.341	*
CHOICE15	1.194	0.051	0.000	1.098	1.296	*
BULLY15	0.604	0.026	0.000	0.555	0.656	*
PRESS15	1.163	0.049	0.000	1.068	1.260	*
DUTIES15	0.998	0.043	0.000	0.919	1.092	*
Between Level						
S1 ON						
PRAFAC	-0.039	0.053	0.250	-0.137	0.070	
KESCAT16 ON						
PRAFAC	-0.120	0.053	0.012	-0.230	-0.021	*
DUTIES15	-0.320	0.305	0.163	-0.878	0.329	

```
 PRAFAC   WITH
    DUTIES15           0.014       0.067      0.409      -0.112       0.156

 Means
    PRAFAC            -0.194       0.151      0.102      -0.495       0.097
    DUTIES15           3.708       0.056      0.000       3.595       3.820      *

 Intercepts
    S1                -0.075       0.055      0.087      -0.183       0.026

 Thresholds
    KESCAT16$1        -1.624       1.129      0.081      -3.698       0.740

 Variances
    PRAFAC             1.339       0.263      0.000       0.955       1.991      *
    DUTIES15           0.081       0.026      0.000       0.046       0.150      *

 Residual Variances
    KESCAT16           0.020       0.033      0.000       0.001       0.126      *
    S1                 0.011       0.021      0.000       0.001       0.071      *


STANDARDIZED MODEL RESULTS


STDYX Standardization

                                Posterior  One-Tailed         95% C.I.
                    Estimate       S.D.      P-Value   Lower 2.5%  Upper 2.5%  Significance

Within-Level Standardized Estimates Averaged Over Clusters

 S1 | KESCAT16 ON
    DUTIES15          -0.062       0.044      0.085      -0.147       0.026

 KESCAT16   ON
    PHY15              0.119       0.039      0.002       0.042       0.194      *
    CHOICE15           0.027       0.039      0.242      -0.048       0.106
    PRESS15           -0.039       0.039      0.160      -0.114       0.037
    BULLY15            0.074       0.041      0.035      -0.004       0.156
    KES15              0.396       0.039      0.000       0.317       0.473      *

 KES15    WITH
```

```
    PHY15              0.227      0.028      0.000      0.172      0.282      *
    CHOICE15          -0.157      0.029      0.000     -0.212     -0.101      *
    BULLY15            0.166      0.029      0.000      0.113      0.223      *
    PRESS15            0.102      0.030      0.001      0.044      0.161      *
    DUTIES15          -0.127      0.030      0.000     -0.184     -0.066      *

 PHY15    WITH
    CHOICE15          -0.106      0.030      0.001     -0.162     -0.048      *
    BULLY15            0.062      0.030      0.019      0.003      0.117      *
    PRESS15            0.132      0.030      0.000      0.074      0.192      *
    DUTIES15          -0.066      0.030      0.018     -0.124     -0.003      *

 CHOICE15 WITH
    BULLY15           -0.164      0.029      0.000     -0.219     -0.103      *
    PRESS15           -0.166      0.030      0.000     -0.225     -0.105      *
    DUTIES15           0.142      0.031      0.000      0.081      0.202      *

 BULLY15  WITH
    PRESS15            0.240      0.029      0.000      0.183      0.296      *
    DUTIES15          -0.217      0.029      0.000     -0.273     -0.159      *

 PRESS15  WITH
    DUTIES15          -0.262      0.028      0.000     -0.316     -0.205      *

 Means
    PHY15              0.000      0.030      0.498     -0.058      0.058
    KES15              0.000      0.030      0.497     -0.060      0.057
    CHOICE15          -0.001      0.030      0.493     -0.059      0.057
    BULLY15            0.001      0.030      0.493     -0.059      0.059
    PRESS15            0.001      0.030      0.484     -0.058      0.059

 Variances
    PHY15              1.000      0.000      0.000      1.000      1.000
    KES15              1.000      0.000      0.000      1.000      1.000
    CHOICE15           1.000      0.000      0.000      1.000      1.000
    BULLY15            1.000      0.000      0.000      1.000      1.000
    PRESS15            1.000      0.000      0.000      1.000      1.000
    DUTIES15           1.000      0.000      0.000      1.000      1.000

Between Level

 S1         ON
    PRAFAC            -0.370      0.437      0.250     -0.949      0.682
```

KESCAT16 ON						
PRAFAC	-0.587	0.220	0.012	-0.948	-0.106	*
DUTIES15	-0.383	0.323	0.163	-0.826	0.410	
PRAFAC WITH						
DUTIES15	0.045	0.188	0.409	-0.319	0.413	
Means						
PRAFAC	-0.166	0.128	0.102	-0.419	0.082	
DUTIES15	13.005	1.960	0.000	9.542	17.467	*
Intercepts						
S1	-0.570	0.568	0.087	-1.941	0.224	
Variances						
PRAFAC	1.000	0.000	0.000	1.000	1.000	
DUTIES15	1.000	0.000	0.000	1.000	1.000	
Residual Variances						
KESCAT16	0.372	0.252	0.000	0.027	0.907	*
S1	0.805	0.271	0.000	0.093	0.999	*

R-SQUARE

Within-Level R-Square Averaged Across Clusters

Variable	Estimate	Posterior S.D.	One-Tailed P-Value	95% C.I. Lower 2.5%	Upper 2.5%
KESCAT16	0.234	0.036	0.000	0.168	0.307

Between Level

Variable	Estimate	Posterior S.D.	One-Tailed P-Value	95% C.I. Lower 2.5%	Upper 2.5%
KESCAT16	0.628	0.252	0.000	0.093	0.973

Variable	Estimate	Posterior S.D.	One-Tailed P-Value	95% C.I. Lower 2.5%	Upper 2.5%
S1	0.195	0.271	0.000	0.001	0.907

TECHNICAL 1 OUTPUT

PARAMETER SPECIFICATION FOR WITHIN

TAU

KESCAT16
0

NU

KESCAT16	PHY15	KES15	CHOICE15	BULLY15
0	0	0	0	0

NU

PRESS15	DUTIES15
0	0

LAMBDA

	KESCAT16	PHY15	KES15	CHOICE15	BULLY15
KESCAT16	0	0	0	0	0
PHY15	0	0	0	0	0
KES15	0	0	0	0	0
CHOICE15	0	0	0	0	0
BULLY15	0	0	0	0	0
PRESS15	0	0	0	0	0
DUTIES15	0	0	0	0	0

LAMBDA

	PRESS15	DUTIES15
KESCAT16	0	0
PHY15	0	0
KES15	0	0
CHOICE15	0	0

BULLY15	0	0
PRESS15	0	0
DUTIES15	0	0

THETA

	KESCAT16	PHY15	KES15	CHOICE15	BULLY15
KESCAT16	0				
PHY15	0	0			
KES15	0	0	0		
CHOICE15	0	0	0	0	
BULLY15	0	0	0	0	0
PRESS15	0	0	0	0	0
DUTIES15	0	0	0	0	0

THETA

	PRESS15	DUTIES15
PRESS15	0	
DUTIES15	0	0

ALPHA

KESCAT16	PHY15	KES15	CHOICE15	BULLY15
0	1	2	3	4

ALPHA

PRESS15	DUTIES15
5	0

BETA

	KESCAT16	PHY15	KES15	CHOICE15	BULLY15
KESCAT16	0	6	7	8	9
PHY15	0	0	0	0	0
KES15	0	0	0	0	0
CHOICE15	0	0	0	0	0
BULLY15	0	0	0	0	0

PRESS15	0	0	0	0	0
DUTIES15	0	0	0	0	0

BETA

	PRESS15	DUTIES15
KESCAT16	10	0
PHY15	0	0
KES15	0	0
CHOICE15	0	0
BULLY15	0	0
PRESS15	0	0
DUTIES15	0	0

PSI

	KESCAT16	PHY15	KES15	CHOICE15	BULLY15
KESCAT16	0				
PHY15	0	11			
KES15	0	12	13		
CHOICE15	0	14	15	16	
BULLY15	0	17	18	19	20
PRESS15	0	21	22	23	24
DUTIES15	0	26	27	28	29

PSI

	PRESS15	DUTIES15
PRESS15	25	
DUTIES15	30	31

PARAMETER SPECIFICATION FOR BETWEEN

TAU

KESCAT16
43

NU

	KESCAT16	PRAFAC	DUTIES15
	0	0	0

LAMBDA

	S1	KESCAT16	PRAFAC	DUTIES15
KESCAT16	0	0	0	0
PRAFAC	0	0	0	0
DUTIES15	0	0	0	0

THETA

	KESCAT16	PRAFAC	DUTIES15
KESCAT16	0		
PRAFAC	0	0	
DUTIES15	0	0	0

ALPHA

	S1	KESCAT16	PRAFAC	DUTIES15
	32	0	33	34

BETA

	S1	KESCAT16	PRAFAC	DUTIES15
S1	0	0	35	0
KESCAT16	0	0	36	37
PRAFAC	0	0	0	0
DUTIES15	0	0	0	0

PSI

	S1	KESCAT16	PRAFAC	DUTIES15
S1	38			
KESCAT16	0	39		
PRAFAC	0	0	40	
DUTIES15	0	0	41	42

STARTING VALUES FOR WITHIN

TAU

KESCAT16
0.000

NU

KESCAT16	PHY15	KES15	CHOICE15	BULLY15
0.000	0.000	0.000	0.000	0.000

NU

PRESS15	DUTIES15
0.000	0.000

LAMBDA

	KESCAT16	PHY15	KES15	CHOICE15	BULLY15
KESCAT16	1.000	0.000	0.000	0.000	0.000
PHY15	0.000	1.000	0.000	0.000	0.000
KES15	0.000	0.000	1.000	0.000	0.000
CHOICE15	0.000	0.000	0.000	1.000	0.000
BULLY15	0.000	0.000	0.000	0.000	1.000
PRESS15	0.000	0.000	0.000	0.000	0.000
DUTIES15	0.000	0.000	0.000	0.000	0.000

LAMBDA

	PRESS15	DUTIES15
KESCAT16	0.000	0.000
PHY15	0.000	0.000
KES15	0.000	0.000
CHOICE15	0.000	0.000
BULLY15	0.000	0.000
PRESS15	1.000	0.000

DUTIES15	0.000	1.000

THETA

	KESCAT16	PHY15	KES15	CHOICE15	BULLY15
KESCAT16	0.000				
PHY15	0.000	0.000			
KES15	0.000	0.000	0.000		
CHOICE15	0.000	0.000	0.000	0.000	
BULLY15	0.000	0.000	0.000	0.000	0.000
PRESS15	0.000	0.000	0.000	0.000	0.000
DUTIES15	0.000	0.000	0.000	0.000	0.000

THETA

	PRESS15	DUTIES15
PRESS15	0.000	
DUTIES15	0.000	0.000

ALPHA

KESCAT16	PHY15	KES15	CHOICE15	BULLY15
0.000	0.000	0.000	0.000	0.000

ALPHA

PRESS15	DUTIES15
0.000	0.000

BETA

	KESCAT16	PHY15	KES15	CHOICE15	BULLY15
KESCAT16	0.000	0.000	0.000	0.000	0.000
PHY15	0.000	0.000	0.000	0.000	0.000
KES15	0.000	0.000	0.000	0.000	0.000
CHOICE15	0.000	0.000	0.000	0.000	0.000
BULLY15	0.000	0.000	0.000	0.000	0.000
PRESS15	0.000	0.000	0.000	0.000	0.000
DUTIES15	0.000	0.000	0.000	0.000	0.000

BETA

	PRESS15	DUTIES15
KESCAT16	0.000	0.000
PHY15	0.000	0.000
KES15	0.000	0.000
CHOICE15	0.000	0.000
BULLY15	0.000	0.000
PRESS15	0.000	0.000
DUTIES15	0.000	0.000

PSI

	KESCAT16	PHY15	KES15	CHOICE15	BULLY15
KESCAT16	1.000				
PHY15	0.000	0.351			
KES15	0.000	0.000	0.157		
CHOICE15	0.000	0.000	0.000	0.597	
BULLY15	0.000	0.000	0.000	0.000	0.301
PRESS15	0.000	0.000	0.000	0.000	0.000
DUTIES15	0.000	0.000	0.000	0.000	0.000

PSI

	PRESS15	DUTIES15
PRESS15	0.580	
DUTIES15	0.000	0.516

STARTING VALUES FOR BETWEEN

TAU

KESCAT16
-0.352

NU

KESCAT16	PRAFAC	DUTIES15

0.000	0.000	0.000	

LAMBDA

	S1	KESCAT16	PRAFAC	DUTIES15
KESCAT16	0.000	1.000	0.000	0.000
PRAFAC	0.000	0.000	1.000	0.000
DUTIES15	0.000	0.000	0.000	1.000

THETA

	KESCAT16	PRAFAC	DUTIES15
KESCAT16	0.000		
PRAFAC	0.000	0.000	
DUTIES15	0.000	0.000	0.000

ALPHA

S1	KESCAT16	PRAFAC	DUTIES15
0.000	0.000	-0.033	3.672

BETA

	S1	KESCAT16	PRAFAC	DUTIES15
S1	0.000	0.000	0.000	0.000
KESCAT16	0.000	0.000	0.000	0.000
PRAFAC	0.000	0.000	0.000	0.000
DUTIES15	0.000	0.000	0.000	0.000

PSI

	S1	KESCAT16	PRAFAC	DUTIES15
S1	1.000			
KESCAT16	0.000	1.000		
PRAFAC	0.000	0.000	0.469	
DUTIES15	0.000	0.000	0.000	0.516

PRIORS FOR ALL PARAMETERS	PRIOR MEAN	PRIOR VARIANCE	PRIOR STD. DEV.
Parameter 1~N(0.000,infinity)	0.0000	infinity	infinity
Parameter 2~N(0.000,infinity)	0.0000	infinity	infinity
Parameter 3~N(0.000,infinity)	0.0000	infinity	infinity
Parameter 4~N(0.000,infinity)	0.0000	infinity	infinity
Parameter 5~N(0.000,infinity)	0.0000	infinity	infinity
Parameter 6~N(0.000,5.000)	0.0000	5.0000	2.2361
Parameter 7~N(0.000,5.000)	0.0000	5.0000	2.2361
Parameter 8~N(0.000,5.000)	0.0000	5.0000	2.2361
Parameter 9~N(0.000,5.000)	0.0000	5.0000	2.2361
Parameter 10~N(0.000,5.000)	0.0000	5.0000	2.2361
Parameter 11~IW(1.000,7)	infinity	infinity	infinity
Parameter 12~IW(0.000,7)	infinity	infinity	infinity
Parameter 13~IW(1.000,7)	infinity	infinity	infinity
Parameter 14~IW(0.000,7)	infinity	infinity	infinity
Parameter 15~IW(0.000,7)	infinity	infinity	infinity
Parameter 16~IW(1.000,7)	infinity	infinity	infinity
Parameter 17~IW(0.000,7)	infinity	infinity	infinity
Parameter 18~IW(0.000,7)	infinity	infinity	infinity
Parameter 19~IW(0.000,7)	infinity	infinity	infinity
Parameter 20~IW(1.000,7)	infinity	infinity	infinity
Parameter 21~IW(0.000,7)	infinity	infinity	infinity
Parameter 22~IW(0.000,7)	infinity	infinity	infinity
Parameter 23~IW(0.000,7)	infinity	infinity	infinity
Parameter 24~IW(0.000,7)	infinity	infinity	infinity
Parameter 25~IW(1.000,7)	infinity	infinity	infinity
Parameter 26~IW(0.000,7)	infinity	infinity	infinity
Parameter 27~IW(0.000,7)	infinity	infinity	infinity
Parameter 28~IW(0.000,7)	infinity	infinity	infinity
Parameter 29~IW(0.000,7)	infinity	infinity	infinity
Parameter 30~IW(0.000,7)	infinity	infinity	infinity
Parameter 31~IW(1.000,7)	infinity	infinity	infinity
Parameter 32~N(0.000,infinity)	0.0000	infinity	infinity
Parameter 33~N(0.000,infinity)	0.0000	infinity	infinity
Parameter 34~N(0.000,infinity)	0.0000	infinity	infinity
Parameter 35~N(0.000,infinity)	0.0000	infinity	infinity
Parameter 36~N(0.000,5.000)	0.0000	5.0000	2.2361
Parameter 37~N(0.000,5.000)	0.0000	5.0000	2.2361
Parameter 38~IG(-1.000,0.000)	infinity	infinity	infinity
Parameter 39~IG(-1.000,0.000)	infinity	infinity	infinity
Parameter 40~IW(1.000,3)	infinity	infinity	infinity
Parameter 41~IW(0.000,3)	infinity	infinity	infinity

Parameter 42~IW(1.000,3)	infinity	infinity	infinity
Parameter 43~N(0.000,5.000)	0.0000	5.0000	2.2361

TECHNICAL 3 OUTPUT

ESTIMATED COVARIANCE MATRIX FOR PARAMETER ESTIMATES

	1	2	3	4	5
1	0.625715D-03				
2	0.855927D-04	0.273162D-03			
3	-0.555678D-04	-0.734755D-04	0.106415D-02		
4	0.166311D-04	0.637115D-04	-0.970784D-04	0.568556D-03	
5	0.699770D-04	0.678643D-04	-0.187886D-03	0.173600D-03	0.107173D-02
6	-0.535478D-04	0.727769D-05	0.972222D-04	0.119350D-04	-0.670019D-04
7	-0.575707D-05	-0.168263D-04	-0.997407D-04	0.112468D-04	0.170678D-04
8	-0.124361D-04	0.320908D-05	0.151435D-04	-0.872822D-05	0.613563D-05
9	-0.430197D-04	0.444949D-04	-0.571316D-06	-0.707673D-04	-0.117448D-04
10	-0.200696D-04	-0.144372D-04	-0.114191D-05	-0.250984D-04	-0.162161D-04
11	0.165759D-04	0.788556D-05	-0.729993D-05	0.315374D-04	-0.508408D-04
12	-0.105602D-04	-0.456308D-05	0.897734D-05	-0.241700D-05	-0.262268D-04
13	0.476118D-05	0.663574D-05	-0.121875D-04	0.172131D-05	-0.571248D-05
14	-0.516406D-05	0.737610D-05	-0.194863D-04	-0.714586D-05	-0.370846D-05
15	-0.922351D-05	-0.274971D-05	0.133239D-04	-0.347982D-05	-0.211435D-04
16	0.159747D-04	0.868063D-05	0.427038D-04	0.273406D-04	-0.990462D-05
17	-0.941422D-05	-0.333092D-05	-0.203908D-05	-0.957971D-05	-0.116965D-04
18	0.396843D-05	0.410211D-05	0.149703D-04	-0.534793D-05	-0.142040D-04
19	0.150440D-04	0.852731D-05	0.315364D-05	0.303032D-04	0.925548D-05
20	-0.172092D-04	0.566140D-05	-0.230186D-04	-0.178654D-04	0.185508D-04
21	-0.160322D-04	0.110005D-04	0.131148D-04	0.143065D-04	-0.533256D-05
22	-0.524615D-05	0.261306D-06	0.548301D-04	-0.221915D-04	-0.146157D-04
23	-0.935973D-05	0.133322D-05	-0.336034D-04	-0.235138D-05	-0.669469D-05
24	-0.174761D-04	0.437112D-05	0.181290D-04	-0.217376D-04	-0.289307D-04
25	0.303567D-05	-0.179413D-04	0.509554D-04	-0.110047D-04	-0.457419D-04
26	0.249690D-04	0.297103D-05	0.144989D-04	0.114729D-05	0.177563D-04
27	0.596334D-05	-0.654305D-05	-0.673978D-05	0.232253D-04	-0.120458D-04
28	-0.190291D-04	-0.152762D-05	0.848076D-05	-0.155088D-04	0.368707D-04
29	0.130704D-04	-0.102795D-04	0.300879D-04	0.239691D-04	0.144658D-04
30	-0.208250D-04	-0.895840D-05	0.284460D-04	0.234017D-04	0.416332D-04
31	0.319731D-04	0.216606D-04	-0.839403D-04	0.297646D-04	-0.224416D-05
32	-0.860843D-05	-0.130256D-04	0.484559D-04	-0.505687D-04	0.208836D-04
33	0.220679D-04	0.120791D-04	0.139724D-03	-0.135229D-03	0.292246D-04
34	0.268869D-04	-0.528447D-04	0.150363D-03	-0.142797D-03	-0.176046D-03

35	0.232917D-04	-0.345506D-05	0.385639D-05	0.233147D-04	-0.489352D-04
36	-0.351969D-04	-0.256441D-05	0.592160D-04	0.239281D-04	0.738630D-04
37	0.206622D-03	0.259783D-04	-0.127287D-03	-0.228578D-03	-0.140699D-03
38	-0.884393D-05	-0.744008D-05	-0.470817D-05	0.165565D-04	0.211724D-04
39	0.442865D-04	-0.821697D-05	0.127426D-04	-0.594904D-05	0.667318D-06
40	0.132034D-03	0.191208D-03	0.100847D-04	0.452653D-04	-0.422233D-03
41	0.488894D-05	0.121714D-04	-0.184717D-05	0.519774D-04	-0.376175D-04
42	-0.160940D-04	-0.225270D-04	-0.301204D-05	-0.203193D-04	0.149301D-04
43	0.727892D-03	0.103376D-03	-0.505071D-03	-0.804934D-03	-0.476866D-03

ESTIMATED COVARIANCE MATRIX FOR PARAMETER ESTIMATES

	6	7	8	9	10
6	0.290521D-02				
7	-0.381609D-03	0.903310D-02			
8	0.409603D-04	0.360708D-03	0.169480D-02		
9	0.202100D-03	-0.486755D-03	0.244168D-03	0.369890D-02	
10	-0.311533D-03	-0.175345D-03	0.158540D-03	-0.369990D-03	0.170745D-02
11	-0.344715D-04	-0.110569D-03	-0.435867D-04	-0.108678D-03	-0.403725D-04
12	0.557436D-05	0.405421D-04	-0.124606D-04	-0.515938D-04	-0.881825D-05
13	-0.187085D-04	0.260610D-04	-0.144893D-04	-0.569786D-04	0.299027D-05
14	-0.765815D-04	0.323073D-04	0.568461D-05	0.157644D-04	0.126681D-04
15	-0.269235D-04	0.478280D-04	0.441651D-04	0.752439D-05	0.205112D-04
16	0.115604D-03	-0.747612D-04	-0.121665D-03	0.103689D-03	-0.858404D-04
17	-0.931205D-05	0.171219D-04	-0.319890D-05	-0.117090D-04	0.387589D-04
18	-0.177404D-04	-0.279839D-04	0.326509D-05	-0.144421D-04	0.273822D-04
19	0.141428D-04	-0.233558D-04	0.306612D-05	0.421553D-04	-0.198481D-04
20	-0.279553D-04	0.227810D-04	0.281537D-04	-0.213273D-04	-0.234543D-04
21	0.357333D-04	0.219803D-04	-0.561691D-04	-0.629501D-04	0.322896D-04
22	-0.132694D-04	-0.233422D-04	-0.182018D-04	-0.261427D-04	0.704988D-05
23	-0.533200D-04	-0.139119D-03	0.105373D-04	0.349815D-04	-0.338750D-04
24	0.769821D-05	-0.422683D-04	-0.290414D-04	-0.948465D-05	0.642686D-05
25	0.333373D-04	-0.338323D-04	-0.237279D-04	0.742877D-04	0.140474D-05
26	-0.227445D-04	-0.515572D-05	0.303165D-04	0.188156D-04	-0.422802D-04
27	0.157071D-04	0.298491D-04	0.268842D-04	0.581630D-05	-0.129786D-04
28	0.889755D-04	-0.782352D-04	-0.220508D-04	-0.404318D-05	-0.146192D-04
29	0.397299D-04	0.116045D-03	-0.829586D-05	0.120957D-04	-0.885526D-05
30	-0.337886D-04	0.140572D-03	0.464080D-04	-0.900137D-05	0.577013D-05
31	-0.769171D-05	-0.282261D-03	-0.694069D-04	-0.292981D-05	0.984442D-04
32	-0.669162D-04	0.408731D-04	-0.255732D-03	0.354844D-03	0.405117D-03
33	0.430861D-03	-0.248000D-03	-0.731287D-04	-0.223270D-03	0.187193D-03
34	-0.439099D-04	-0.462106D-04	0.715089D-04	0.150959D-03	0.921571D-05
35	-0.103594D-03	0.975511D-04	-0.187993D-03	0.128020D-03	0.592928D-05

36	0.195157D-03	0.293820D-03	-0.698897D-04	-0.383885D-04	-0.179245D-03
37	-0.322866D-03	-0.457326D-03	-0.882849D-04	-0.575469D-03	-0.127651D-03
38	0.685844D-05	0.200176D-03	0.689946D-05	-0.524440D-04	0.227346D-04
39	0.701277D-04	0.414817D-03	0.651756D-04	-0.116342D-03	-0.292331D-04
40	-0.120380D-04	-0.720497D-04	0.536065D-03	0.517200D-03	0.188659D-03
41	-0.127552D-03	-0.998719D-04	-0.108792D-04	-0.805988D-04	-0.395792D-04
42	-0.167643D-04	0.440371D-04	0.348919D-04	-0.182449D-04	0.397830D-04
43	-0.134838D-02	-0.297807D-02	-0.318526D-03	-0.239856D-02	-0.504046D-03

ESTIMATED COVARIANCE MATRIX FOR PARAMETER ESTIMATES

	11	12	13	14	15
11	0.887237D-03				
12	0.125820D-03	0.198289D-03			
13	0.892938D-05	0.578656D-04	0.174013D-03		
14	-0.139815D-03	-0.753453D-04	-0.122061D-04	0.781346D-03	
15	-0.101475D-04	-0.361207D-04	-0.458337D-04	0.118487D-03	0.332947D-03
16	-0.177381D-05	-0.165260D-04	0.142164D-04	-0.203745D-03	-0.215317D-03
17	0.489968D-04	0.456016D-04	0.234146D-04	-0.998906D-04	-0.221236D-04
18	0.510343D-05	0.121321D-04	0.340235D-04	-0.113181D-04	-0.426139D-04
19	0.305844D-05	-0.154511D-04	-0.118945D-04	0.423015D-04	0.885089D-04
20	-0.380669D-04	-0.845378D-05	0.987403D-05	0.903671D-05	-0.136039D-04
21	0.145045D-03	0.518017D-04	0.189275D-04	-0.162047D-03	-0.357326D-04
22	0.964018D-05	0.365825D-04	0.286477D-04	-0.350816D-04	-0.566368D-04
23	0.960592D-05	-0.244520D-04	-0.171607D-04	0.170457D-03	0.104318D-03
24	-0.311849D-04	0.116685D-04	0.646436D-05	-0.162988D-04	-0.271795D-04
25	-0.166485D-04	0.207275D-04	0.303010D-05	-0.615682D-04	-0.493233D-04
26	-0.687254D-04	-0.683067D-04	-0.642233D-05	0.131806D-03	0.334240D-04
27	-0.233821D-05	0.238912D-06	-0.220618D-04	0.280201D-04	0.406979D-04
28	0.396776D-05	0.191524D-05	0.236707D-04	-0.874314D-04	-0.126384D-03
29	0.343487D-04	0.100432D-04	0.649185D-05	0.136231D-04	0.328088D-04
30	0.249145D-04	-0.858829D-05	-0.564347D-05	0.775162D-04	0.390365D-04
31	-0.234355D-04	-0.307539D-04	-0.109881D-04	-0.391717D-04	-0.314221D-04
32	-0.310505D-04	-0.178837D-04	-0.411247D-04	-0.920411D-05	0.523045D-04
33	-0.152669D-03	-0.411158D-05	-0.264291D-04	0.147321D-03	-0.365222D-05
34	0.216895D-04	0.770960D-05	0.153421D-04	0.150639D-04	0.109438D-04
35	0.433905D-04	-0.921034D-06	0.226364D-04	-0.757835D-05	0.228232D-06
36	-0.223857D-04	-0.104763D-04	0.315193D-04	-0.175345D-04	-0.228251D-04
37	-0.528199D-04	0.716328D-04	0.951974D-04	-0.796969D-04	-0.320911D-03
38	0.161581D-05	-0.112268D-04	0.473421D-05	0.331507D-04	-0.611152D-05
39	-0.376018D-04	-0.161883D-04	-0.137041D-04	-0.630397D-05	-0.129456D-04
40	0.300664D-04	-0.307499D-04	-0.906590D-04	-0.402964D-04	0.246092D-03
41	-0.547198D-04	-0.858691D-05	0.477094D-04	0.417718D-04	0.346649D-04

42	-0.167610D-04	-0.167864D-04	-0.783279D-05	-0.118991D-04	0.349417D-04
43	-0.832233D-04	0.304527D-03	0.371363D-03	-0.319820D-03	-0.123068D-02

ESTIMATED COVARIANCE MATRIX FOR PARAMETER ESTIMATES

	16	17	18	19	20
16	0.256869D-02				
17	0.510706D-04	0.381349D-03			
18	0.143612D-04	0.575326D-04	0.166619D-03		
19	-0.223301D-03	-0.687374D-04	-0.622942D-04	0.665957D-03	
20	0.101232D-04	0.373381D-04	0.921601D-04	-0.141470D-03	0.658843D-03
21	0.333341D-04	0.134223D-03	0.397684D-04	-0.235198D-04	0.370035D-04
22	0.223745D-04	0.503767D-04	0.687767D-04	-0.523696D-04	0.461430D-04
23	-0.466298D-03	-0.448444D-04	-0.451057D-04	0.254833D-03	-0.372674D-04
24	0.491597D-04	0.766243D-04	0.435656D-04	-0.131341D-03	0.197085D-03
25	0.745168D-04	0.942819D-04	0.319657D-04	-0.552369D-04	0.160303D-04
26	-0.314722D-04	-0.943729D-04	-0.209657D-04	0.134312D-04	-0.370414D-04
27	-0.426225D-04	-0.249825D-04	-0.523254D-04	0.300853D-04	-0.342816D-04
28	0.314424D-03	0.260322D-04	0.410131D-04	-0.175903D-03	0.405149D-04
29	-0.105348D-05	-0.233720D-04	-0.564640D-04	0.128550D-03	-0.193191D-03
30	-0.848450D-04	-0.457197D-04	-0.220834D-04	0.563223D-04	-0.104367D-03
31	0.152879D-03	0.260982D-04	0.230059D-04	-0.176214D-04	0.675163D-04
32	0.581529D-04	0.202222D-04	-0.573788D-05	0.381878D-04	-0.477290D-04
33	-0.246613D-03	-0.837552D-04	-0.849774D-05	0.266073D-04	0.526161D-04
34	-0.633173D-04	-0.435298D-04	0.219811D-04	-0.193087D-04	-0.464711D-04
35	-0.480383D-04	0.100669D-05	0.227951D-04	0.192493D-04	0.191077D-04
36	0.123347D-03	-0.140977D-05	0.178829D-04	0.184167D-04	-0.428059D-04
37	-0.228609D-03	0.774433D-04	0.152752D-03	0.247585D-03	0.150911D-03
38	-0.158913D-04	0.100951D-04	0.931942D-05	0.101646D-04	0.145994D-04
39	-0.860146D-04	-0.142296D-05	-0.630882D-05	0.101404D-04	0.170384D-04
40	-0.732677D-03	0.205350D-04	0.366764D-04	0.290728D-04	0.192421D-03
41	0.170363D-03	0.136194D-04	0.327493D-04	-0.390911D-04	0.888857D-05
42	-0.379245D-04	0.902225D-05	-0.113017D-04	0.662395D-05	-0.422702D-04
43	-0.853953D-03	0.317580D-03	0.602213D-03	0.893309D-03	0.522527D-03

ESTIMATED COVARIANCE MATRIX FOR PARAMETER ESTIMATES

	21	22	23	24	25
21	0.760640D-03				
22	0.135640D-03	0.343724D-03			
23	-0.149382D-03	-0.137601D-03	0.134092D-02		
24	0.615009D-04	0.105372D-03	-0.166742D-03	0.654623D-03	

25	0.271281D-03	0.154837D-03	-0.431066D-03	0.330397D-03	0.240688D-02
26	-0.181282D-03	-0.589571D-04	0.689193D-04	-0.373620D-04	-0.864890D-04
27	-0.249026D-04	-0.869616D-04	0.410505D-04	-0.305607D-04	-0.145660D-04
28	0.648271D-04	0.602272D-04	-0.379721D-03	0.656980D-04	0.500138D-04
29	-0.268381D-04	-0.417413D-04	0.939130D-04	-0.167912D-03	-0.707835D-04
30	-0.118563D-03	-0.101696D-03	0.235484D-03	-0.227334D-03	-0.616342D-03
31	-0.102684D-04	0.296465D-04	-0.431704D-04	0.195764D-04	0.116423D-03
32	-0.173115D-04	-0.719883D-05	0.732452D-04	-0.262813D-04	0.105899D-03
33	0.956778D-04	-0.788505D-04	-0.655425D-04	-0.282716D-04	-0.867820D-05
34	0.178362D-04	0.420021D-04	0.238609D-04	0.171799D-04	-0.108443D-03
35	0.133542D-04	0.793993D-05	-0.105754D-04	0.588796D-04	0.526052D-04
36	0.328024D-04	0.326887D-04	0.566326D-05	-0.246960D-04	0.413851D-04
37	0.436695D-04	-0.124803D-03	0.285501D-03	-0.105110D-03	-0.711893D-03
38	-0.736931D-05	-0.334992D-05	-0.132748D-04	-0.328341D-05	0.214181D-04
39	-0.243150D-04	-0.214278D-04	0.494654D-04	-0.240719D-04	-0.308525D-04
40	0.350027D-03	-0.399810D-04	0.147931D-03	0.364120D-04	0.465832D-03
41	0.259493D-04	-0.205529D-04	0.164427D-04	0.119502D-04	-0.174502D-05
42	0.202900D-04	-0.191885D-04	-0.274056D-04	-0.366488D-05	0.339841D-04
43	0.169201D-03	-0.434465D-03	0.111729D-02	-0.363651D-03	-0.262384D-02

ESTIMATED COVARIANCE MATRIX FOR PARAMETER ESTIMATES

	26	27	28	29	30
26	0.697710D-03				
27	0.961823D-04	0.308423D-03			
28	-0.119640D-03	-0.112460D-03	0.119233D-02		
29	0.474176D-04	0.947141D-04	-0.160806D-03	0.575122D-03	
30	0.147658D-03	0.999501D-04	-0.262575D-03	0.245229D-03	0.119463D-02
31	-0.544618D-04	-0.158075D-03	0.322706D-03	-0.288774D-03	-0.484594D-03
32	0.102321D-04	-0.413398D-05	0.605166D-05	0.250105D-04	-0.422782D-04
33	-0.107719D-03	0.906020D-05	0.154880D-03	-0.545029D-04	-0.956591D-04
34	0.506493D-04	0.293347D-04	-0.109498D-03	0.676566D-04	0.113976D-03
35	-0.925273D-05	-0.195473D-04	-0.846756D-04	-0.583058D-05	0.608476D-05
36	-0.100632D-04	0.348907D-05	-0.774410D-04	0.904967D-05	-0.719435D-04
37	0.977189D-04	0.139879D-03	0.353872D-03	0.265130D-04	0.333152D-03
38	-0.721999D-06	-0.192829D-05	-0.252648D-04	0.208659D-04	0.556597D-05
39	-0.844535D-06	-0.956528D-05	-0.509811D-05	0.750567D-05	0.109673D-04
40	0.200733D-03	-0.101663D-03	-0.351647D-03	-0.465852D-04	-0.558453D-04
41	0.526332D-04	0.242834D-04	-0.126860D-03	0.306003D-04	-0.332820D-04
42	-0.519049D-05	0.169986D-04	-0.663558D-04	0.154399D-04	0.403508D-04
43	0.360962D-03	0.539178D-03	0.132758D-02	0.903554D-04	0.112310D-02

ESTIMATED COVARIANCE MATRIX FOR PARAMETER ESTIMATES

	31	32	33	34	35
31	0.188982D-02				
32	0.864313D-04	0.302403D-02			
33	0.325669D-03	0.109843D-03	0.228261D-01		
34	0.728404D-05	0.956996D-04	0.193299D-03	0.316669D-02	
35	-0.764490D-04	0.337840D-03	0.721456D-04	-0.570377D-04	0.276315D-02
36	-0.114058D-03	-0.711202D-04	0.316439D-03	0.121852D-04	-0.137865D-03
37	-0.321959D-03	-0.861657D-03	-0.181459D-02	0.763603D-04	-0.521938D-03
38	-0.725156D-05	-0.195285D-03	0.547478D-05	-0.235414D-04	-0.288042D-04
39	0.168132D-04	-0.460568D-04	-0.860272D-04	0.435454D-04	-0.108939D-04
40	0.727393D-04	-0.284878D-03	-0.147173D-02	-0.619745D-03	-0.405160D-03
41	-0.961700D-04	0.321551D-05	-0.606411D-03	-0.100263D-03	0.184699D-03
42	-0.785047D-04	0.980145D-05	0.448146D-04	-0.439736D-04	0.972972D-05
43	-0.114393D-02	-0.325456D-02	-0.632538D-02	0.356553D-04	-0.200702D-02

ESTIMATED COVARIANCE MATRIX FOR PARAMETER ESTIMATES

	36	37	38	39	40
36	0.276375D-02				
37	0.173915D-02	0.928733D-01			
38	-0.345997D-04	0.191222D-03	0.423050D-03		
39	-0.152465D-03	0.330016D-03	0.288334D-04	0.110577D-02	
40	0.150935D-04	-0.941238D-03	0.197187D-03	0.148578D-03	0.689807D-01
41	0.217035D-03	-0.930155D-03	-0.305950D-04	-0.208236D-04	0.100508D-02
42	-0.337655D-04	-0.236979D-03	0.145696D-04	0.913023D-05	0.208692D-03
43	0.626651D-02	0.343421D+00	0.623309D-03	0.122429D-02	-0.418531D-02

ESTIMATED COVARIANCE MATRIX FOR PARAMETER ESTIMATES

	41	42	43
41	0.442545D-02		
42	0.163482D-03	0.687408D-03	
43	-0.346933D-02	-0.950835D-03	0.127314D+01

ESTIMATED CORRELATION MATRIX FOR PARAMETER ESTIMATES

	1	2	3	4	5
1	1.000				
2	0.207	1.000			

3	-0.068	-0.136	1.000		
4	0.028	0.162	-0.125	1.000	
5	0.085	0.125	-0.176	0.222	1.000
6	-0.040	0.008	0.055	0.009	-0.038
7	-0.002	-0.011	-0.032	0.005	0.005
8	-0.012	0.005	0.011	-0.009	0.005
9	-0.028	0.044	0.000	-0.049	-0.006
10	-0.019	-0.021	-0.001	-0.025	-0.012
11	0.022	0.016	-0.008	0.044	-0.052
12	-0.030	-0.020	0.020	-0.007	-0.057
13	0.014	0.030	-0.028	0.005	-0.013
14	-0.007	0.016	-0.021	-0.011	-0.004
15	-0.020	-0.009	0.022	-0.008	-0.035
16	0.013	0.010	0.026	0.023	-0.006
17	-0.019	-0.010	-0.003	-0.021	-0.018
18	0.012	0.019	0.036	-0.017	-0.034
19	0.023	0.020	0.004	0.049	0.011
20	-0.027	0.013	-0.027	-0.029	0.022
21	-0.023	0.024	0.015	0.022	-0.006
22	-0.011	0.001	0.091	-0.050	-0.024
23	-0.010	0.002	-0.028	-0.003	-0.006
24	-0.027	0.010	0.022	-0.036	-0.035
25	0.002	-0.022	0.032	-0.009	-0.028
26	0.038	0.007	0.017	0.002	0.021
27	0.014	-0.023	-0.012	0.055	-0.021
28	-0.022	-0.003	0.008	-0.019	0.033
29	0.022	-0.026	0.038	0.042	0.018
30	-0.024	-0.016	0.025	0.028	0.037
31	0.029	0.030	-0.059	0.029	-0.002
32	-0.006	-0.014	0.027	-0.039	0.012
33	0.006	0.005	0.028	-0.038	0.006
34	0.019	-0.057	0.082	-0.106	-0.096
35	0.018	-0.004	0.002	0.019	-0.028
36	-0.027	-0.003	0.035	0.019	0.043
37	0.027	0.005	-0.013	-0.031	-0.014
38	-0.017	-0.022	-0.007	0.034	0.031
39	0.053	-0.015	0.012	-0.008	0.001
40	0.020	0.044	0.001	0.007	-0.049
41	0.003	0.011	-0.001	0.033	-0.017
42	-0.025	-0.052	-0.004	-0.033	0.017
43	0.026	0.006	-0.014	-0.030	-0.013

ESTIMATED CORRELATION MATRIX FOR PARAMETER ESTIMATES

	6	7	8	9	10
6	1.000				
7	-0.074	1.000			
8	0.018	0.092	1.000		
9	0.062	-0.084	0.098	1.000	
10	-0.140	-0.045	0.093	-0.147	1.000
11	-0.021	-0.039	-0.036	-0.060	-0.033
12	0.007	0.030	-0.021	-0.060	-0.015
13	-0.026	0.021	-0.027	-0.071	0.005
14	-0.051	0.012	0.005	0.009	0.011
15	-0.027	0.028	0.059	0.007	0.027
16	0.042	-0.016	-0.058	0.034	-0.041
17	-0.009	0.009	-0.004	-0.010	0.048
18	-0.025	-0.023	0.006	-0.018	0.051
19	0.010	-0.010	0.003	0.027	-0.019
20	-0.020	0.009	0.027	-0.014	-0.022
21	0.024	0.008	-0.049	-0.038	0.028
22	-0.013	-0.013	-0.024	-0.023	0.009
23	-0.027	-0.040	0.007	0.016	-0.022
24	0.006	-0.017	-0.028	-0.006	0.006
25	0.013	-0.007	-0.012	0.025	0.001
26	-0.016	-0.002	0.028	0.012	-0.039
27	0.017	0.018	0.037	0.005	-0.018
28	0.048	-0.024	-0.016	-0.002	-0.010
29	0.031	0.051	-0.008	0.008	-0.009
30	-0.018	0.043	0.033	-0.004	0.004
31	-0.003	-0.068	-0.039	-0.001	0.055
32	-0.023	0.008	-0.113	0.106	0.178
33	0.053	-0.017	-0.012	-0.024	0.030
34	-0.014	-0.009	0.031	0.044	0.004
35	-0.037	0.020	-0.087	0.040	0.003
36	0.069	0.059	-0.032	-0.012	-0.083
37	-0.020	-0.016	-0.007	-0.031	-0.010
38	0.006	0.102	0.008	-0.042	0.027
39	0.039	0.131	0.048	-0.058	-0.021
40	-0.001	-0.003	0.050	0.032	0.017
41	-0.036	-0.016	-0.004	-0.020	-0.014
42	-0.012	0.018	0.032	-0.011	0.037
43	-0.022	-0.028	-0.007	-0.035	-0.011

ESTIMATED CORRELATION MATRIX FOR PARAMETER ESTIMATES

11	12	13	14	15

11	1.000				
12	0.300	1.000			
13	0.023	0.312	1.000		
14	-0.168	-0.191	-0.033	1.000	
15	-0.019	-0.141	-0.190	0.232	1.000
16	-0.001	-0.023	0.021	-0.144	-0.233
17	0.084	0.166	0.091	-0.183	-0.062
18	0.013	0.067	0.200	-0.031	-0.181
19	0.004	-0.043	-0.035	0.059	0.188
20	-0.050	-0.023	0.029	0.013	-0.029
21	0.177	0.133	0.052	-0.210	-0.071
22	0.017	0.140	0.117	-0.068	-0.167
23	0.009	-0.047	-0.036	0.167	0.156
24	-0.041	0.032	0.019	-0.023	-0.058
25	-0.011	0.030	0.005	-0.045	-0.055
26	-0.087	-0.184	-0.018	0.179	0.069
27	-0.004	0.001	-0.095	0.057	0.127
28	0.004	0.004	0.052	-0.091	-0.201
29	0.048	0.030	0.021	0.020	0.075
30	0.024	-0.018	-0.012	0.080	0.062
31	-0.018	-0.050	-0.019	-0.032	-0.040
32	-0.019	-0.023	-0.057	-0.006	0.052
33	-0.034	-0.002	-0.013	0.035	-0.001
34	0.013	0.010	0.021	0.010	0.011
35	0.028	-0.001	0.033	-0.005	0.000
36	-0.014	-0.014	0.045	-0.012	-0.024
37	-0.006	0.017	0.024	-0.009	-0.058
38	0.003	-0.039	0.017	0.058	-0.016
39	-0.038	-0.035	-0.031	-0.007	-0.021
40	0.004	-0.008	-0.026	-0.005	0.051
41	-0.028	-0.009	0.054	0.022	0.029
42	-0.021	-0.045	-0.023	-0.016	0.073
43	-0.002	0.019	0.025	-0.010	-0.060

ESTIMATED CORRELATION MATRIX FOR PARAMETER ESTIMATES

	16	17	18	19	20
16	1.000				
17	0.052	1.000			
18	0.022	0.228	1.000		
19	-0.171	-0.136	-0.187	1.000	
20	0.008	0.074	0.278	-0.214	1.000

21	0.024	0.249	0.112	-0.033	0.052
22	0.024	0.139	0.287	-0.109	0.097
23	-0.251	-0.063	-0.095	0.270	-0.040
24	0.038	0.153	0.132	-0.199	0.300
25	0.030	0.098	0.050	-0.044	0.013
26	-0.024	-0.183	-0.061	0.020	-0.055
27	-0.048	-0.073	-0.231	0.066	-0.076
28	0.180	0.039	0.092	-0.197	0.046
29	-0.001	-0.050	-0.182	0.208	-0.314
30	-0.048	-0.068	-0.049	0.063	-0.118
31	0.069	0.031	0.041	-0.016	0.061
32	0.021	0.019	-0.008	0.027	-0.034
33	-0.032	-0.028	-0.004	0.007	0.014
34	-0.022	-0.040	0.030	-0.013	-0.032
35	-0.018	0.001	0.034	0.014	0.014
36	0.046	-0.001	0.026	0.014	-0.032
37	-0.015	0.013	0.039	0.031	0.019
38	-0.015	0.025	0.035	0.019	0.028
39	-0.051	-0.002	-0.015	0.012	0.020
40	-0.055	0.004	0.011	0.004	0.029
41	0.051	0.010	0.038	-0.023	0.005
42	-0.029	0.018	-0.033	0.010	-0.063
43	-0.015	0.014	0.041	0.031	0.018

ESTIMATED CORRELATION MATRIX FOR PARAMETER ESTIMATES

	21	22	23	24	25
21	1.000				
22	0.265	1.000			
23	-0.148	-0.203	1.000		
24	0.087	0.222	-0.178	1.000	
25	0.200	0.170	-0.240	0.263	1.000
26	-0.249	-0.120	0.071	-0.055	-0.067
27	-0.051	-0.267	0.064	-0.068	-0.017
28	0.068	0.094	-0.300	0.074	0.030
29	-0.041	-0.094	0.107	-0.274	-0.060
30	-0.124	-0.159	0.186	-0.257	-0.363
31	-0.009	0.037	-0.027	0.018	0.055
32	-0.011	-0.007	0.036	-0.019	0.039
33	0.023	-0.028	-0.012	-0.007	-0.001
34	0.011	0.040	0.012	0.012	-0.039
35	0.009	0.008	-0.005	0.044	0.020
36	0.023	0.034	0.003	-0.018	0.016

37	0.005	-0.022	0.026	-0.013	-0.048
38	-0.013	-0.009	-0.018	-0.006	0.021
39	-0.027	-0.035	0.041	-0.028	-0.019
40	0.048	-0.008	0.015	0.005	0.036
41	0.014	-0.017	0.007	0.007	-0.001
42	0.028	-0.039	-0.029	-0.005	0.026
43	0.005	-0.021	0.027	-0.013	-0.047

ESTIMATED CORRELATION MATRIX FOR PARAMETER ESTIMATES

	26	27	28	29	30
26	1.000				
27	0.207	1.000			
28	-0.131	-0.185	1.000		
29	0.075	0.225	-0.194	1.000	
30	0.162	0.165	-0.220	0.296	1.000
31	-0.047	-0.207	0.215	-0.277	-0.323
32	0.007	-0.004	0.003	0.019	-0.022
33	-0.027	0.003	0.030	-0.015	-0.018
34	0.034	0.030	-0.056	0.050	0.059
35	-0.007	-0.021	-0.047	-0.005	0.003
36	-0.007	0.004	-0.043	0.007	-0.040
37	0.012	0.026	0.034	0.004	0.032
38	-0.001	-0.005	-0.036	0.042	0.008
39	-0.001	-0.016	-0.004	0.009	0.010
40	0.029	-0.022	-0.039	-0.007	-0.006
41	0.030	0.021	-0.055	0.019	-0.014
42	-0.007	0.037	-0.073	0.025	0.045
43	0.012	0.027	0.034	0.003	0.029

ESTIMATED CORRELATION MATRIX FOR PARAMETER ESTIMATES

	31	32	33	34	35
31	1.000				
32	0.036	1.000			
33	0.050	0.013	1.000		
34	0.003	0.031	0.023	1.000	
35	-0.033	0.117	0.009	-0.019	1.000
36	-0.050	-0.025	0.040	0.004	-0.050
37	-0.024	-0.051	-0.039	0.004	-0.033
38	-0.008	-0.173	0.002	-0.020	-0.027
39	0.012	-0.025	-0.017	0.023	-0.006

40	0.006	-0.020	-0.037	-0.042	-0.029
41	-0.033	0.001	-0.060	-0.027	0.053
42	-0.069	0.007	0.011	-0.030	0.007
43	-0.023	-0.052	-0.037	0.001	-0.034

ESTIMATED CORRELATION MATRIX FOR PARAMETER ESTIMATES

	36	37	38	39	40
36	1.000				
37	0.109	1.000			
38	-0.032	0.031	1.000		
39	-0.087	0.033	0.042	1.000	
40	0.001	-0.012	0.037	0.017	1.000
41	0.062	-0.046	-0.022	-0.009	0.058
42	-0.024	-0.030	0.027	0.010	0.030
43	0.106	0.999	0.027	0.033	-0.014

ESTIMATED CORRELATION MATRIX FOR PARAMETER ESTIMATES

	41	42	43
41	1.000		
42	0.094	1.000	
43	-0.046	-0.032	1.000

TECHNICAL 8 OUTPUT

TECHNICAL 8 OUTPUT FOR BAYES ESTIMATION

CHAIN	BSEED
1	0
2	285380
3	253358

ITERATION	POTENTIAL SCALE REDUCTION	PARAMETER WITH HIGHEST PSR
100	1.042	19
200	1.027	35
300	1.017	35
400	1.005	34
500	1.007	32

600	1.005	39
700	1.004	35
800	1.004	39
900	1.006	39
1000	1.002	25

These are models with the Kessler score and physical health score in 2016 treated as continuous not dichotomous variables, with 2015 values of Kessler and physical health interacting with health and wellbeing practices (the respective cross-level interactions are represented by the regression of health and wellbeing practices on the random slopes (s1 for 2015 Kessler and s2 for 2015 physical health).

Physical health model

SUMMARY OF DATA

Number of clusters 58

Size (s)	Cluster ID with Size s
11	22
18	166
26	80
28	74 205
31	71
34	65
35	192
40	106 182
41	228
45	72 170
47	50
50	165 30 75 181
53	173
57	107
59	108 40
60	81 226 84
63	114
66	93
68	61 4
69	162
70	83 169
71	206
72	116 36
73	69
76	92
79	68
84	174
85	145 154
88	152
95	6
104	144 98 2

114	178
118	156
123	202
131	142
159	122
218	24
239	209
283	204
331	125
472	217
954	110
1083	100

COVARIANCE COVERAGE OF DATA

Minimum covariance coverage value 0.100

Number of missing data patterns 9

PROPORTION OF DATA PRESENT

Covariance Coverage

	PHY15	KES15	PHY16	CHOICE15	BULLY15
PHY15	0.958				
KES15	0.932	0.932			
PHY16	0.958	0.932	1.000		
CHOICE15	0.925	0.925	0.925	0.925	
BULLY15	0.925	0.925	0.925	0.925	0.925
PRESS15	0.925	0.925	0.925	0.925	0.925
DUTIES15	0.926	0.926	0.926	0.925	0.925
PRAFAC	0.958	0.932	1.000	0.925	0.925

Covariance Coverage

	PRESS15	DUTIES15	PRAFAC
PRESS15	0.925		
DUTIES15	0.925	0.926	
PRAFAC	0.925	0.926	1.000

UNIVARIATE SAMPLE STATISTICS

UNIVARIATE HIGHER-ORDER MOMENT DESCRIPTIVE STATISTICS

Variable/ Sample Size	Mean/ Variance	Skewness/ Kurtosis	Minimum/ Maximum	% with Min/Max	Percentiles 20%/60%	40%/80%	Median
PHY15	1.956	0.559	1.000	28.98%	1.000	2.000	2.000
6676.000	0.605	0.164	5.000	0.24%	2.000	3.000	
KES15	0.605	1.665	0.000	18.88%	0.167	0.333	0.500
6495.000	0.391	3.340	4.000	0.11%	0.500	1.000	
PHY16	1.968	0.532	1.000	28.85%	1.000	2.000	2.000
6968.000	0.615	0.123	5.000	0.32%	2.000	3.000	
CHOICE15	0.000	-0.355	-2.151	8.93%	-1.151	-0.151	-0.151
6447.000	1.137	-0.460	1.849	7.91%	0.849	0.849	
BULLY15	0.000	3.100	-0.200	85.49%	-0.200	-0.200	-0.200
6446.000	0.292	10.556	3.800	0.16%	-0.200	-0.200	
PRESS15	0.000	0.260	-1.525	18.02%	-0.525	-0.525	0.475
6447.000	1.060	-0.377	2.475	3.71%	0.475	0.475	
DUTIES15	0.000	-1.160	-3.067	2.54%	-1.067	-0.067	-0.067
6450.000	0.895	1.352	0.933	36.40%	-0.067	0.933	
PRAFAC	-0.185	0.245	-2.338	1.72%	-1.448	-0.527	-0.318
58.000	1.345	-0.636	2.338	1.72%	0.080	0.752	

THE MODEL ESTIMATION TERMINATED NORMALLY

USE THE FBITERATIONS OPTION TO INCREASE THE NUMBER OF ITERATIONS BY A FACTOR
OF AT LEAST TWO TO CHECK CONVERGENCE AND THAT THE PSR VALUE DOES NOT INCREASE.

MODEL FIT INFORMATION

Number of Free Parameters 44

Information Criteria

Deviance (DIC) 103390.444
Estimated Number of Parameters (pD) 92.742

MODEL RESULTS

	Estimate	Posterior S.D.	One-Tailed P-Value	95% C.I. Lower 2.5%	95% C.I. Upper 2.5%	Significance
Within Level						
PHY16 ON						
KES15	0.115	0.013	0.000	0.089	0.141	*
CHOICE15	-0.017	0.007	0.011	-0.032	-0.003	*
BULLY15	0.030	0.015	0.020	0.001	0.059	*
DUTIES15	-0.007	0.008	0.186	-0.024	0.009	
PRESS15	-0.009	0.008	0.119	-0.024	0.006	
KES15 WITH						
PHY15	0.166	0.006	0.000	0.154	0.179	*
CHOICE15	-0.126	0.009	0.000	-0.143	-0.109	*
BULLY15	0.083	0.005	0.000	0.074	0.092	*
PRESS15	0.147	0.008	0.000	0.131	0.164	*
DUTIES15	-0.144	0.008	0.000	-0.159	-0.129	*
PHY15 WITH						
CHOICE15	-0.122	0.011	0.000	-0.143	-0.101	*
BULLY15	0.048	0.005	0.000	0.038	0.058	*
PRESS15	0.110	0.010	0.000	0.090	0.130	*
DUTIES15	-0.092	0.009	0.000	-0.110	-0.074	*
CHOICE15 WITH						
BULLY15	-0.082	0.007	0.000	-0.096	-0.068	*
PRESS15	-0.125	0.014	0.000	-0.152	-0.099	*
DUTIES15	0.185	0.013	0.000	0.159	0.209	*
BULLY15 WITH						
PRESS15	0.127	0.007	0.000	0.114	0.140	*
DUTIES15	-0.097	0.007	0.000	-0.111	-0.085	*
PRESS15 WITH						
DUTIES15	-0.210	0.013	0.000	-0.236	-0.186	*
Means						
CHOICE15	0.000	0.013	0.494	-0.025	0.026	

```
   BULLY15            0.000      0.007      0.497     -0.014      0.013
   PRESS15            0.000      0.013      0.497     -0.025      0.026
   DUTIES15           0.000      0.012      0.497     -0.023      0.023

 Variances
   PHY15              0.601      0.010      0.000      0.582      0.621      *
   KES15              0.388      0.007      0.000      0.376      0.402      *
   CHOICE15           1.141      0.021      0.000      1.099      1.181      *
   BULLY15            0.293      0.005      0.000      0.283      0.303      *
   PRESS15            1.061      0.019      0.000      1.026      1.099      *
   DUTIES15           0.897      0.016      0.000      0.868      0.929      *

 Residual Variances
   PHY16              0.361      0.006      0.000      0.349      0.373      *

Between Level

 S2         ON
   PRAFAC            -0.008      0.011      0.238     -0.029      0.015

 PHY16      ON
   PRAFAC            -0.023      0.010      0.007     -0.043     -0.004      *
   PHY15              1.082      0.322      0.001      0.599      1.857      *
   KES15             -0.203      0.358      0.245     -1.001      0.372

 PHY15    WITH
   KES15              0.005      0.002      0.003      0.001      0.009      *

 Means
   PHY15              1.949      0.018      0.000      1.914      1.987      *
   KES15              0.602      0.014      0.000      0.576      0.631      *

 Intercepts
   PHY16             -0.025      0.484      0.479     -1.142      0.728
   S2                 0.601      0.013      0.000      0.576      0.626      *

 Variances
   PHY15              0.009      0.004      0.000      0.004      0.019      *
   KES15              0.005      0.002      0.000      0.002      0.009      *

 Residual Variances
   PHY16              0.001      0.001      0.000      0.000      0.004      *
   S2                 0.001      0.001      0.000      0.001      0.004      *
```

STANDARDIZED MODEL RESULTS

STDYX Standardization

	Estimate	Posterior S.D.	One-Tailed P-Value	95% C.I. Lower 2.5%	95% C.I. Upper 2.5%	Significance
**Within-Level Standardized Estimates Averaged Over Clusters**						
S2 | PHY16 ON						
PHY15	0.595	0.009	0.000	0.578	0.612	*
PHY16 ON						
KES15	0.092	0.011	0.000	0.070	0.112	*
CHOICE15	-0.023	0.010	0.010	-0.043	-0.003	*
BULLY15	0.021	0.010	0.017	0.001	0.041	*
DUTIES15	-0.009	0.010	0.190	-0.029	0.011	
PRESS15	-0.012	0.010	0.119	-0.032	0.008	
KES15 WITH						
PHY15	0.343	0.011	0.000	0.321	0.365	*
CHOICE15	-0.189	0.012	0.000	-0.213	-0.165	*
BULLY15	0.246	0.012	0.000	0.223	0.270	*
PRESS15	0.230	0.012	0.000	0.206	0.253	*
DUTIES15	-0.244	0.012	0.000	-0.267	-0.222	*
PHY15 WITH						
CHOICE15	-0.147	0.013	0.000	-0.172	-0.123	*
BULLY15	0.114	0.012	0.000	0.090	0.139	*
PRESS15	0.138	0.012	0.000	0.113	0.162	*
DUTIES15	-0.125	0.012	0.000	-0.149	-0.100	*
CHOICE15 WITH						
BULLY15	-0.143	0.012	0.000	-0.166	-0.118	*
PRESS15	-0.114	0.012	0.000	-0.138	-0.090	*
DUTIES15	0.183	0.012	0.000	0.159	0.206	*
BULLY15 WITH						
PRESS15	0.228	0.012	0.000	0.205	0.252	*
DUTIES15	-0.189	0.012	0.000	-0.213	-0.165	*
PRESS15 WITH						

```
    DUTIES15          -0.215       0.012      0.000      -0.238      -0.193      *

 Means
    CHOICE15           0.000       0.013      0.493      -0.025       0.025
    BULLY15            0.000       0.012      0.498      -0.025       0.024
    PRESS15            0.000       0.012      0.497      -0.024       0.025
    DUTIES15           0.000       0.012      0.498      -0.024       0.023

 Variances
    PHY15              1.000       0.000      0.000       1.000       1.000
    KES15              1.000       0.000      0.000       1.000       1.000
    CHOICE15           1.000       0.000      0.000       1.000       1.000
    BULLY15            1.000       0.000      0.000       1.000       1.000
    PRESS15            1.000       0.000      0.000       1.000       1.000
    DUTIES15           1.000       0.000      0.000       1.000       1.000

 Residual Variances
    PHY16              0.589       0.010      0.000       0.570       0.609      *

Between Level

 S2         ON
    PRAFAC            -0.141       0.208      0.238      -0.543       0.252

 PHY16      ON
    PRAFAC            -0.159       0.082      0.007      -0.350      -0.027      *
    PHY15              0.997       0.221      0.001       0.612       1.479      *
    KES15             -0.134       0.254      0.245      -0.675       0.272

 PHY15    WITH
    KES15              0.690       0.176      0.003       0.232       0.922      *

 Means
    PHY15             20.245       4.515      0.000      14.186      31.047      *
    KES15              8.561       1.550      0.000       6.193      12.268      *

 Intercepts
    PHY16             -0.242       5.096      0.479     -10.307       9.428
    S2                15.588       3.661      0.000       8.841      23.081      *

 Variances
    PHY15              1.000       0.000      0.000       1.000       1.000
    KES15              1.000       0.000      0.000       1.000       1.000
```

```
 Residual Variances
    PHY16              0.101       0.107      0.000       0.006       0.402      *
    S2                 0.969       0.086      0.000       0.705       1.000      *


R-SQUARE

Within-Level R-Square Averaged Across Clusters

                                Posterior  One-Tailed         95% C.I.
    Variable        Estimate       S.D.      P-Value   Lower 2.5%  Upper 2.5%

    PHY16              0.411       0.010      0.000       0.391       0.430

Between Level

                                Posterior  One-Tailed         95% C.I.
    Variable        Estimate       S.D.      P-Value   Lower 2.5%  Upper 2.5%

    PHY16              0.899       0.107      0.000       0.598       0.994

                                Posterior  One-Tailed         95% C.I.
    Variable        Estimate       S.D.      P-Value   Lower 2.5%  Upper 2.5%

    S2                 0.031       0.086      0.000       0.000       0.295


TECHNICAL 1 OUTPUT


     PARAMETER SPECIFICATION FOR WITHIN


           NU
              PHY15         KES15         PHY16         CHOICE15      BULLY15
              ________      ________      ________      ________      ________
                    0             0             0             0             0


           NU
              PRESS15       DUTIES15
              ________      ________
                    0             0
```

LAMBDA

	PHY15	KES15	PHY16	CHOICE15	BULLY15
PHY15	0	0	0	0	0
KES15	0	0	0	0	0
PHY16	0	0	0	0	0
CHOICE15	0	0	0	0	0
BULLY15	0	0	0	0	0
PRESS15	0	0	0	0	0
DUTIES15	0	0	0	0	0

LAMBDA

	PRESS15	DUTIES15
PHY15	0	0
KES15	0	0
PHY16	0	0
CHOICE15	0	0
BULLY15	0	0
PRESS15	0	0
DUTIES15	0	0

THETA

	PHY15	KES15	PHY16	CHOICE15	BULLY15
PHY15	0				
KES15	0	0			
PHY16	0	0	0		
CHOICE15	0	0	0	0	
BULLY15	0	0	0	0	0
PRESS15	0	0	0	0	0
DUTIES15	0	0	0	0	0

THETA

	PRESS15	DUTIES15
PRESS15	0	
DUTIES15	0	0

ALPHA

PHY15	KES15	PHY16	CHOICE15	BULLY15
0	0	0	1	2

ALPHA

PRESS15	DUTIES15
3	4

BETA

	PHY15	KES15	PHY16	CHOICE15	BULLY15
PHY15	0	0	0	0	0
KES15	0	0	0	0	0
PHY16	0	5	0	6	7
CHOICE15	0	0	0	0	0
BULLY15	0	0	0	0	0
PRESS15	0	0	0	0	0
DUTIES15	0	0	0	0	0

BETA

	PRESS15	DUTIES15
PHY15	0	0
KES15	0	0
PHY16	8	9
CHOICE15	0	0
BULLY15	0	0
PRESS15	0	0
DUTIES15	0	0

PSI

	PHY15	KES15	PHY16	CHOICE15	BULLY15
PHY15	10				
KES15	11	12			
PHY16	0	0	13		
CHOICE15	14	15	0	16	
BULLY15	17	18	0	19	20

PRESS15	21	22	0	23	24
DUTIES15	26	27	0	28	29

PSI

	PRESS15	DUTIES15
PRESS15	25	
DUTIES15	30	31

PARAMETER SPECIFICATION FOR BETWEEN

NU

PHY15	KES15	PHY16	PRAFAC
0	0	0	0

LAMBDA

	S2	PHY15	KES15	PHY16	PRAFAC
PHY15	0	0	0	0	0
KES15	0	0	0	0	0
PHY16	0	0	0	0	0
PRAFAC	0	0	0	0	0

THETA

	PHY15	KES15	PHY16	PRAFAC
PHY15	0			
KES15	0	0		
PHY16	0	0	0	
PRAFAC	0	0	0	0

ALPHA

S2	PHY15	KES15	PHY16	PRAFAC
32	33	34	35	0

BETA

	S2	PHY15	KES15	PHY16	PRAFAC
S2	0	0	0	0	36
PHY15	0	0	0	0	0
KES15	0	0	0	0	0
PHY16	0	37	38	0	39
PRAFAC	0	0	0	0	0

PSI

	S2	PHY15	KES15	PHY16	PRAFAC
S2	40				
PHY15	0	41			
KES15	0	42	43		
PHY16	0	0	0	44	
PRAFAC	0	0	0	0	0

STARTING VALUES FOR WITHIN

NU

PHY15	KES15	PHY16	CHOICE15	BULLY15
0.000	0.000	0.000	0.000	0.000

NU

PRESS15	DUTIES15
0.000	0.000

LAMBDA

	PHY15	KES15	PHY16	CHOICE15	BULLY15
PHY15	1.000	0.000	0.000	0.000	0.000
KES15	0.000	1.000	0.000	0.000	0.000
PHY16	0.000	0.000	1.000	0.000	0.000
CHOICE15	0.000	0.000	0.000	1.000	0.000
BULLY15	0.000	0.000	0.000	0.000	1.000
PRESS15	0.000	0.000	0.000	0.000	0.000

DUTIES15	0.000	0.000	0.000	0.000	0.000

LAMBDA

	PRESS15	DUTIES15
PHY15	0.000	0.000
KES15	0.000	0.000
PHY16	0.000	0.000
CHOICE15	0.000	0.000
BULLY15	0.000	0.000
PRESS15	1.000	0.000
DUTIES15	0.000	1.000

THETA

	PHY15	KES15	PHY16	CHOICE15	BULLY15
PHY15	0.000				
KES15	0.000	0.000			
PHY16	0.000	0.000	0.000		
CHOICE15	0.000	0.000	0.000	0.000	
BULLY15	0.000	0.000	0.000	0.000	0.000
PRESS15	0.000	0.000	0.000	0.000	0.000
DUTIES15	0.000	0.000	0.000	0.000	0.000

THETA

	PRESS15	DUTIES15
PRESS15	0.000	
DUTIES15	0.000	0.000

ALPHA

PHY15	KES15	PHY16	CHOICE15	BULLY15
0.000	0.000	0.000	0.000	0.000

ALPHA

PRESS15	DUTIES15
0.000	0.000

BETA

	PHY15	KES15	PHY16	CHOICE15	BULLY15
PHY15	0.000	0.000	0.000	0.000	0.000
KES15	0.000	0.000	0.000	0.000	0.000
PHY16	0.000	0.000	0.000	0.000	0.000
CHOICE15	0.000	0.000	0.000	0.000	0.000
BULLY15	0.000	0.000	0.000	0.000	0.000
PRESS15	0.000	0.000	0.000	0.000	0.000
DUTIES15	0.000	0.000	0.000	0.000	0.000

BETA

	PRESS15	DUTIES15
PHY15	0.000	0.000
KES15	0.000	0.000
PHY16	0.000	0.000
CHOICE15	0.000	0.000
BULLY15	0.000	0.000
PRESS15	0.000	0.000
DUTIES15	0.000	0.000

PSI

	PHY15	KES15	PHY16	CHOICE15	BULLY15
PHY15	0.303				
KES15	0.000	0.195			
PHY16	0.000	0.000	0.307		
CHOICE15	0.000	0.000	0.000	0.569	
BULLY15	0.000	0.000	0.000	0.000	0.146
PRESS15	0.000	0.000	0.000	0.000	0.000
DUTIES15	0.000	0.000	0.000	0.000	0.000

PSI

	PRESS15	DUTIES15
PRESS15	0.530	
DUTIES15	0.000	0.448

STARTING VALUES FOR BETWEEN

NU

PHY15	KES15	PHY16	PRAFAC
0.000	0.000	0.000	0.000

LAMBDA

	S2	PHY15	KES15	PHY16	PRAFAC
PHY15	0.000	1.000	0.000	0.000	0.000
KES15	0.000	0.000	1.000	0.000	0.000
PHY16	0.000	0.000	0.000	1.000	0.000
PRAFAC	0.000	0.000	0.000	0.000	1.000

THETA

	PHY15	KES15	PHY16	PRAFAC
PHY15	0.000			
KES15	0.000	0.000		
PHY16	0.000	0.000	0.000	
PRAFAC	0.000	0.000	0.000	0.000

ALPHA

S2	PHY15	KES15	PHY16	PRAFAC
0.000	1.956	0.605	1.968	0.000

BETA

	S2	PHY15	KES15	PHY16	PRAFAC
S2	0.000	0.000	0.000	0.000	0.000
PHY15	0.000	0.000	0.000	0.000	0.000
KES15	0.000	0.000	0.000	0.000	0.000
PHY16	0.000	0.000	0.000	0.000	0.000
PRAFAC	0.000	0.000	0.000	0.000	0.000

PSI

	S2	PHY15	KES15	PHY16	PRAFAC
S2	1.000				
PHY15	0.000	0.303			
KES15	0.000	0.000	0.195		
PHY16	0.000	0.000	0.000	0.307	
PRAFAC	0.000	0.000	0.000	0.000	0.500

PRIORS FOR ALL PARAMETERS	PRIOR MEAN	PRIOR VARIANCE	PRIOR STD. DEV.
Parameter 1~N(0.000,infinity)	0.0000	infinity	infinity
Parameter 2~N(0.000,infinity)	0.0000	infinity	infinity
Parameter 3~N(0.000,infinity)	0.0000	infinity	infinity
Parameter 4~N(0.000,infinity)	0.0000	infinity	infinity
Parameter 5~N(0.000,infinity)	0.0000	infinity	infinity
Parameter 6~N(0.000,infinity)	0.0000	infinity	infinity
Parameter 7~N(0.000,infinity)	0.0000	infinity	infinity
Parameter 8~N(0.000,infinity)	0.0000	infinity	infinity
Parameter 9~N(0.000,infinity)	0.0000	infinity	infinity
Parameter 10~IW(0.000,-7)	infinity	infinity	infinity
Parameter 11~IW(0.000,-7)	infinity	infinity	infinity
Parameter 12~IW(0.000,-7)	infinity	infinity	infinity
Parameter 13~IG(-1.000,0.000)	infinity	infinity	infinity
Parameter 14~IW(0.000,-7)	infinity	infinity	infinity
Parameter 15~IW(0.000,-7)	infinity	infinity	infinity
Parameter 16~IW(0.000,-7)	infinity	infinity	infinity
Parameter 17~IW(0.000,-7)	infinity	infinity	infinity
Parameter 18~IW(0.000,-7)	infinity	infinity	infinity
Parameter 19~IW(0.000,-7)	infinity	infinity	infinity
Parameter 20~IW(0.000,-7)	infinity	infinity	infinity
Parameter 21~IW(0.000,-7)	infinity	infinity	infinity
Parameter 22~IW(0.000,-7)	infinity	infinity	infinity
Parameter 23~IW(0.000,-7)	infinity	infinity	infinity
Parameter 24~IW(0.000,-7)	infinity	infinity	infinity
Parameter 25~IW(0.000,-7)	infinity	infinity	infinity
Parameter 26~IW(0.000,-7)	infinity	infinity	infinity
Parameter 27~IW(0.000,-7)	infinity	infinity	infinity
Parameter 28~IW(0.000,-7)	infinity	infinity	infinity
Parameter 29~IW(0.000,-7)	infinity	infinity	infinity
Parameter 30~IW(0.000,-7)	infinity	infinity	infinity
Parameter 31~IW(0.000,-7)	infinity	infinity	infinity

Parameter 32~N(0.000,infinity)	0.0000	infinity	infinity
Parameter 33~N(0.000,infinity)	0.0000	infinity	infinity
Parameter 34~N(0.000,infinity)	0.0000	infinity	infinity
Parameter 35~N(0.000,infinity)	0.0000	infinity	infinity
Parameter 36~N(0.000,infinity)	0.0000	infinity	infinity
Parameter 37~N(0.000,infinity)	0.0000	infinity	infinity
Parameter 38~N(0.000,infinity)	0.0000	infinity	infinity
Parameter 39~N(0.000,infinity)	0.0000	infinity	infinity
Parameter 40~IG(-1.000,0.000)	infinity	infinity	infinity
Parameter 41~IW(0.000,-3)	infinity	infinity	infinity
Parameter 42~IW(0.000,-3)	infinity	infinity	infinity
Parameter 43~IW(0.000,-3)	infinity	infinity	infinity
Parameter 44~IG(-1.000,0.000)	infinity	infinity	infinity

TECHNICAL 3 OUTPUT

ESTIMATED COVARIANCE MATRIX FOR PARAMETER ESTIMATES

	1	2	3	4	5
1	0.179948D-03				
2	-0.142668D-04	0.446923D-04			
3	-0.208749D-04	0.213967D-04	0.161804D-03		
4	0.258804D-04	-0.143372D-04	-0.274989D-04	0.141147D-03	
5	-0.272188D-06	-0.291122D-05	-0.410999D-05	-0.499066D-05	0.173034D-03
6	-0.478302D-06	0.407847D-06	-0.101203D-05	-0.867886D-06	0.125800D-04
7	-0.114258D-05	0.740239D-06	0.369931D-05	0.971002D-05	-0.392431D-04
8	0.304194D-05	-0.378734D-06	-0.310989D-07	-0.351795D-05	-0.902070D-05
9	-0.464183D-05	0.144736D-05	0.460879D-05	0.773738D-06	0.151138D-04
10	0.231588D-05	-0.551610D-07	-0.371125D-05	-0.175561D-06	-0.582853D-05
11	-0.595499D-06	0.139723D-05	-0.115057D-05	-0.216049D-05	-0.146399D-05
12	-0.260087D-07	0.745157D-06	0.738301D-06	-0.274720D-05	-0.290298D-05
13	0.153480D-05	-0.686279D-07	0.106425D-05	-0.711869D-07	-0.277029D-05
14	-0.574046D-06	-0.838301D-06	0.253643D-05	0.130193D-05	0.604227D-05
15	0.255723D-05	0.913183D-06	0.118424D-05	-0.367866D-05	0.238493D-05
16	-0.782701D-05	0.254824D-05	-0.153909D-05	-0.270494D-05	-0.296324D-05
17	-0.860852D-06	-0.165507D-05	-0.737113D-06	-0.310654D-06	-0.183896D-06
18	-0.716537D-06	-0.858182D-06	-0.170416D-05	0.137524D-05	-0.934465D-06
19	0.479659D-06	0.483954D-06	0.560390D-06	-0.167068D-05	-0.708531D-06
20	-0.410192D-06	0.353840D-06	-0.171477D-06	0.708373D-06	-0.195211D-05
21	0.278865D-05	-0.403932D-06	-0.283606D-05	0.151008D-05	-0.228567D-05
22	-0.133872D-05	0.463424D-06	-0.775815D-06	-0.209046D-05	-0.265759D-05
23	-0.702164D-05	-0.301484D-05	0.153496D-06	0.204292D-05	0.782686D-05

24	0.881930D-06	-0.134363D-06	-0.403808D-05	0.334583D-05	-0.190719D-05
25	-0.369597D-05	0.343402D-06	0.144663D-04	0.221859D-05	-0.893939D-05
26	0.637745D-06	-0.571905D-06	-0.240333D-05	0.180935D-05	0.224629D-06
27	0.523552D-05	0.117253D-05	-0.273002D-05	-0.540118D-05	0.670499D-06
28	-0.508346D-05	-0.152108D-05	-0.151125D-05	0.424955D-05	0.129903D-08
29	0.469900D-05	0.667841D-06	0.203113D-05	0.128744D-05	-0.224955D-05
30	0.151057D-05	0.270589D-06	0.775722D-06	0.190954D-05	-0.502882D-05
31	-0.777931D-05	-0.209941D-05	0.324093D-05	0.252987D-05	0.494240D-05
32	-0.153510D-05	0.301732D-05	0.584434D-05	-0.555134D-05	-0.361227D-04
33	-0.265206D-04	0.589867D-05	0.150302D-04	-0.185167D-04	-0.582315D-06
34	-0.256815D-04	0.978995D-05	0.149955D-04	-0.233556D-04	-0.310586D-05
35	0.459159D-05	-0.476981D-04	-0.339729D-03	0.264403D-03	0.154561D-04
36	0.602750D-05	0.270455D-05	0.516172D-05	-0.171131D-05	-0.500998D-05
37	0.477026D-07	0.383969D-04	0.238341D-03	-0.188312D-03	0.144993D-04
38	0.209423D-05	-0.397006D-04	-0.217664D-03	0.180809D-03	-0.728004D-04
39	-0.226722D-05	0.125906D-05	0.935987D-06	-0.212418D-05	-0.496184D-05
40	0.439306D-06	-0.575790D-09	-0.358329D-06	-0.512394D-07	0.341789D-06
41	-0.848817D-06	0.768795D-06	-0.133752D-05	0.451725D-06	0.128166D-06
42	-0.237539D-07	0.350601D-07	-0.338864D-06	0.455285D-06	-0.685817D-06
43	0.229546D-06	-0.364344D-06	-0.482024D-06	0.646796D-06	-0.523206D-06
44	0.188246D-06	0.635059D-07	0.254951D-06	0.185923D-06	0.444974D-06

ESTIMATED COVARIANCE MATRIX FOR PARAMETER ESTIMATES

	6	7	8	9	10
6	0.560116D-04				
7	0.251863D-05	0.225706D-03			
8	0.337322D-05	-0.183786D-04	0.576877D-04		
9	-0.665548D-05	0.115866D-04	0.936305D-05	0.697379D-04	
10	0.278891D-05	-0.472709D-05	0.356196D-05	-0.420049D-05	0.105846D-03
11	-0.559095D-06	0.124528D-05	-0.156202D-05	-0.243279D-05	0.309597D-04
12	-0.104787D-05	-0.369831D-06	-0.131647D-05	-0.385479D-06	0.885398D-05
13	-0.587759D-06	-0.104429D-05	-0.872243D-06	-0.207364D-05	0.845328D-06
14	0.147782D-05	0.312417D-06	-0.143247D-05	0.227303D-05	-0.277863D-04
15	0.870115D-06	-0.633974D-05	0.994051D-06	0.437221D-06	-0.645001D-05
16	-0.558029D-06	0.841798D-05	0.694352D-05	0.128862D-06	0.708137D-05
17	0.401855D-07	-0.180907D-05	0.663939D-07	0.102180D-06	0.988015D-05
18	-0.828716D-06	0.435294D-06	0.155985D-06	0.788379D-06	0.341255D-05
19	0.152748D-05	0.139918D-05	-0.276252D-06	0.459705D-06	-0.770730D-06
20	-0.662538D-07	-0.328348D-06	0.319817D-06	0.248623D-05	0.175312D-05
21	-0.262522D-05	0.106146D-05	0.827985D-06	0.256174D-05	0.215047D-04
22	-0.312263D-06	-0.423726D-06	-0.434457D-07	0.132743D-05	0.107926D-04
23	-0.239899D-05	-0.838110D-05	-0.338637D-05	0.406195D-05	-0.108907D-04

24	-0.978179D-07	-0.269065D-05	-0.611895D-07	0.316094D-05	0.347048D-05
25	-0.603080D-05	0.213631D-05	0.978563D-05	0.500333D-05	-0.202638D-06
26	0.295203D-05	0.648310D-05	0.290987D-06	0.158742D-05	-0.113767D-04
27	0.194900D-05	0.239317D-05	-0.136958D-05	-0.260985D-05	-0.353438D-05
28	-0.463934D-05	0.246210D-05	-0.278782D-06	0.306687D-05	0.130974D-05
29	-0.145724D-05	0.173720D-05	-0.301127D-05	-0.404615D-05	0.120746D-06
30	-0.163725D-05	-0.728888D-05	-0.429295D-05	-0.523242D-05	-0.619609D-05
31	-0.103645D-05	-0.544364D-05	0.184561D-05	0.233292D-05	0.259846D-05
32	0.114797D-04	0.154057D-05	-0.697692D-05	-0.986835D-06	0.254993D-05
33	-0.188689D-05	0.386549D-05	0.302582D-05	0.585843D-06	0.417415D-05
34	0.121108D-05	-0.366827D-05	-0.115822D-05	-0.364513D-05	-0.898316D-06
35	0.902363D-04	-0.246119D-03	-0.147947D-03	-0.105960D-03	-0.453590D-03
36	0.890396D-06	0.179941D-05	-0.193954D-05	0.175550D-06	0.106318D-05
37	-0.145550D-04	0.123283D-03	0.738666D-04	0.145325D-04	0.282690D-03
38	-0.100831D-03	0.433372D-05	0.459848D-05	0.127340D-03	-0.165380D-03
39	0.148742D-05	0.180942D-05	0.126230D-05	0.128244D-06	-0.800763D-06
40	0.336620D-06	0.277834D-06	-0.156378D-06	-0.264237D-06	-0.262039D-06
41	0.618409D-06	-0.984157D-06	-0.158256D-05	-0.804864D-06	-0.266623D-05
42	-0.413735D-06	0.455311D-06	-0.323036D-06	0.410739D-07	-0.207757D-06
43	-0.652154D-06	0.958298D-06	-0.190788D-06	0.128603D-06	-0.334782D-06
44	0.144051D-06	-0.362773D-06	0.253255D-06	0.183697D-06	-0.419895D-06

ESTIMATED COVARIANCE MATRIX FOR PARAMETER ESTIMATES

	11	12	13	14	15
11	0.401928D-04				
12	0.189890D-04	0.436892D-04			
13	-0.141536D-06	0.262160D-06	0.386507D-04		
14	-0.181473D-04	-0.657189D-05	0.524983D-06	0.121134D-03	
15	-0.941754D-05	-0.139496D-04	0.223890D-06	0.345524D-04	0.736784D-04
16	0.557847D-05	0.656904D-05	0.565138D-06	-0.530328D-04	-0.431228D-04
17	0.851997D-05	0.407720D-05	0.473051D-06	-0.113101D-04	-0.115962D-05
18	0.513916D-05	0.100112D-04	-0.753407D-08	-0.285428D-05	-0.555108D-05
19	0.725886D-07	-0.187630D-05	0.307543D-05	0.881323D-05	0.146820D-04
20	0.928538D-06	0.240719D-05	0.675436D-06	-0.199606D-05	-0.172075D-05
21	0.158079D-04	0.643176D-05	-0.888145D-06	-0.201084D-04	-0.499801D-05
22	0.124634D-04	0.158623D-04	0.610339D-06	-0.822122D-05	-0.113247D-04
23	-0.721489D-05	-0.703117D-05	0.243037D-06	0.284373D-04	0.276453D-04
24	0.316993D-05	0.342594D-05	-0.300699D-06	-0.431949D-05	-0.872408D-06
25	0.155701D-05	0.312901D-05	-0.156364D-06	-0.718637D-05	-0.144249D-05
26	-0.129703D-04	-0.600838D-05	0.797825D-06	0.194108D-04	0.650965D-05
27	-0.920238D-05	-0.158116D-04	0.527520D-07	0.654313D-05	0.144691D-04
28	0.160198D-05	0.993212D-06	0.500391D-06	-0.211375D-04	-0.278678D-04

29	-0.232806D-05	-0.375853D-05	0.183581D-05	0.237671D-05	0.354601D-05
30	-0.487124D-05	-0.701777D-05	-0.168887D-05	0.741210D-05	0.487718D-05
31	0.378786D-05	0.775961D-05	-0.125725D-05	-0.449853D-05	-0.119073D-04
32	-0.312094D-05	-0.176789D-05	0.105345D-05	-0.891840D-05	-0.197560D-05
33	0.270978D-05	0.144399D-05	-0.286436D-05	-0.960216D-05	-0.328581D-05
34	0.293011D-05	0.437416D-05	0.148725D-05	-0.364591D-05	-0.325568D-05
35	-0.224919D-04	0.211063D-04	0.105209D-03	0.256009D-03	0.369805D-04
36	0.368417D-06	-0.283261D-06	0.957893D-08	0.888339D-06	-0.398512D-09
37	0.143131D-04	-0.211621D-04	-0.816688D-04	-0.176411D-03	-0.159563D-04
38	-0.905388D-05	0.268111D-04	0.890423D-04	0.146521D-03	-0.929630D-05
39	-0.701357D-07	-0.222086D-06	-0.171333D-05	-0.116991D-06	-0.235145D-05
40	-0.131599D-06	-0.688602D-07	0.551963D-08	-0.226583D-06	-0.158926D-07
41	-0.512334D-06	0.269037D-06	-0.572079D-07	0.248057D-05	0.115634D-05
42	-0.202119D-06	0.206741D-06	-0.304879D-06	-0.589533D-06	-0.195031D-06
43	-0.315816D-06	0.233722D-06	-0.412860D-06	-0.793472D-06	-0.596632D-06
44	0.565181D-07	-0.583408D-07	0.688668D-07	-0.437638D-06	-0.509348D-07

ESTIMATED COVARIANCE MATRIX FOR PARAMETER ESTIMATES

	16	17	18	19	20
16	0.426745D-03				
17	0.361715D-05	0.273805D-04			
18	0.799644D-05	0.843785D-05	0.203576D-04		
19	-0.302496D-04	-0.442787D-05	-0.774730D-05	0.529135D-04	
20	0.619408D-05	0.423198D-05	0.861690D-05	-0.900138D-05	0.267814D-04
21	0.345852D-05	0.121974D-04	0.360431D-05	-0.224093D-05	0.473794D-06
22	0.253020D-05	0.637413D-05	0.101785D-04	-0.533405D-05	0.368349D-05
23	-0.514166D-04	-0.488153D-05	-0.382711D-05	0.251863D-04	-0.482193D-05
24	0.419229D-05	0.659974D-05	0.926218D-05	-0.887909D-05	0.117162D-04
25	0.167077D-05	0.394150D-05	0.367008D-05	-0.670774D-05	0.246690D-05
26	-0.106776D-04	-0.961346D-05	-0.254281D-05	0.875025D-06	0.128115D-05
27	-0.107974D-04	-0.402678D-05	-0.793491D-05	0.359882D-05	-0.210167D-05
28	0.620323D-04	0.318537D-05	0.654362D-05	-0.182263D-04	0.500583D-05
29	-0.118454D-04	-0.479692D-05	-0.901243D-05	0.107338D-04	-0.941134D-05
30	-0.142664D-04	-0.517193D-05	-0.746432D-05	0.915949D-05	-0.394011D-05
31	0.132615D-04	0.535373D-05	0.576212D-05	-0.819885D-05	0.180466D-05
32	0.519133D-06	0.644157D-06	0.646442D-06	0.192919D-05	-0.111452D-05
33	-0.517888D-05	0.114787D-05	0.359312D-06	-0.247937D-05	-0.202155D-05
34	-0.169960D-04	-0.140827D-05	0.841121D-06	0.942883D-07	-0.158133D-05
35	-0.343298D-03	0.692436D-04	0.515629D-04	0.755873D-04	0.777951D-04
36	-0.461511D-05	0.789707D-06	0.646284D-06	0.510364D-07	0.979257D-06
37	0.279827D-03	-0.390968D-04	-0.278891D-04	-0.313444D-04	-0.675446D-04
38	-0.345666D-03	0.114843D-04	0.569557D-05	-0.249864D-04	0.934668D-04

39	0.343132D-05	-0.742031D-06	0.436445D-07	-0.246924D-05	0.589637D-06
40	-0.165169D-06	0.258291D-07	0.241716D-07	0.233822D-06	-0.238935D-06
41	-0.313958D-05	-0.298514D-06	0.247295D-06	0.575691D-06	0.343552D-06
42	-0.871185D-06	0.386590D-07	-0.729384D-08	0.240757D-06	-0.289698D-06
43	-0.734353D-06	-0.121731D-06	-0.538411D-07	0.308279D-06	-0.571712D-06
44	-0.735424D-06	0.798580D-07	-0.205272D-06	0.219235D-06	-0.943339D-07

ESTIMATED COVARIANCE MATRIX FOR PARAMETER ESTIMATES

	21	22	23	24	25
21	0.103423D-03				
22	0.300274D-04	0.700060D-04			
23	-0.263068D-04	-0.252719D-04	0.183839D-03		
24	0.114928D-04	0.166632D-04	-0.168912D-04	0.497912D-04	
25	0.401965D-04	0.583052D-04	-0.423235D-04	0.399074D-04	0.369216D-03
26	-0.217368D-04	-0.790082D-05	0.116281D-04	-0.177509D-05	-0.714610D-05
27	-0.772951D-05	-0.149658D-04	0.884077D-05	-0.537503D-05	-0.117865D-04
28	0.402055D-05	0.840172D-05	-0.379529D-04	0.250820D-05	0.135747D-04
29	-0.151237D-05	-0.555538D-05	0.700219D-05	-0.120719D-04	-0.861075D-05
30	-0.141365D-04	-0.310341D-04	0.408177D-04	-0.208080D-04	-0.741724D-04
31	0.600443D-05	0.875304D-05	-0.125498D-04	0.496001D-05	0.125410D-04
32	0.101551D-05	-0.425403D-05	0.313068D-05	0.170991D-05	0.478462D-05
33	0.107742D-04	0.827093D-05	0.242797D-05	0.731840D-06	0.676412D-05
34	0.654614D-05	0.719285D-05	0.188805D-05	-0.101098D-05	0.188458D-05
35	-0.204959D-03	0.147718D-04	0.194094D-03	0.683830D-04	-0.283116D-03
36	-0.653852D-06	0.163586D-05	0.402264D-05	-0.602480D-06	-0.295005D-06
37	0.118757D-03	-0.240434D-04	-0.147443D-03	-0.467336D-04	0.161860D-03
38	-0.481452D-04	0.473568D-04	0.165758D-03	0.360382D-04	-0.910325D-04
39	-0.250958D-05	-0.480139D-05	-0.266853D-06	-0.167083D-05	0.136696D-05
40	0.107187D-06	0.274873D-06	0.343282D-06	-0.209819D-06	0.210834D-06
41	-0.207681D-05	-0.185482D-05	0.239067D-06	-0.530657D-06	-0.311147D-05
42	-0.830711D-07	-0.750632D-06	-0.779870D-06	-0.515385D-07	-0.513677D-06
43	0.253644D-06	0.233974D-06	-0.327303D-06	-0.100958D-06	-0.698435D-06
44	-0.192747D-06	-0.368118D-07	0.167677D-06	0.198720D-06	0.449605D-06

ESTIMATED COVARIANCE MATRIX FOR PARAMETER ESTIMATES

	26	27	28	29	30
26	0.855329D-04				
27	0.245245D-04	0.578796D-04			
28	-0.169414D-04	-0.214508D-04	0.162969D-03		
29	0.499497D-05	0.141624D-04	-0.143653D-04	0.434824D-04	

30	0.198389D-04	0.238621D-04	-0.264705D-04	0.210439D-04	0.158001D-03
31	-0.237916D-04	-0.394104D-04	0.540240D-04	-0.266539D-04	-0.615595D-04
32	0.366945D-05	0.427960D-05	0.186501D-05	0.282409D-05	0.849874D-05
33	-0.257652D-05	-0.179136D-05	-0.371655D-05	0.217531D-05	0.127173D-05
34	-0.608846D-06	-0.371487D-05	-0.746029D-06	0.316978D-05	-0.377808D-05
35	0.361027D-03	0.123948D-03	0.137869D-03	0.208606D-04	0.246571D-03
36	0.254743D-05	0.139782D-05	-0.551171D-05	-0.219598D-05	0.572862D-06
37	-0.235841D-03	-0.709683D-04	-0.574227D-04	-0.141479D-04	-0.159675D-03
38	0.170983D-03	0.261429D-04	-0.439860D-04	0.110031D-04	0.116885D-03
39	0.307603D-05	0.957811D-06	0.295862D-05	-0.137100D-05	0.143885D-06
40	-0.253666D-06	-0.263687D-06	-0.940138D-07	-0.175000D-06	-0.496826D-07
41	0.172685D-05	-0.905304D-06	0.136099D-05	-0.124101D-06	0.605479D-06
42	0.198018D-06	-0.403353D-06	0.787093D-06	-0.654810D-07	0.185028D-06
43	0.340731D-06	-0.289763D-06	0.353349D-06	0.100837D-07	0.213878D-06
44	0.244677D-06	0.234651D-06	0.213967D-06	0.207507D-06	0.161778D-06

ESTIMATED COVARIANCE MATRIX FOR PARAMETER ESTIMATES

	31	32	33	34	35
31	0.248100D-03				
32	-0.143501D-04	0.167504D-03			
33	-0.824312D-06	0.836061D-05	0.340454D-03		
34	0.536623D-06	0.683627D-05	0.140834D-03	0.187504D-03	
35	-0.210168D-03	0.381755D-03	-0.635708D-03	0.208866D-03	0.234393D+00
36	-0.538712D-05	0.169286D-04	-0.343015D-06	-0.127945D-05	0.162729D-03
37	0.169538D-03	-0.278304D-03	0.363811D-03	-0.164045D-03	-0.149211D+00
38	-0.194104D-03	0.266570D-03	-0.206671D-03	0.192149D-03	0.954611D-01
39	0.330334D-05	0.229338D-05	0.108943D-04	0.278228D-05	-0.939826D-03
40	0.398592D-06	0.783038D-06	-0.228074D-06	-0.329516D-06	0.118214D-04
41	-0.101790D-05	-0.102722D-05	-0.597110D-05	0.731086D-06	0.543489D-03
42	0.183252D-06	-0.300750D-06	-0.886621D-06	-0.962596D-06	-0.316995D-04
43	-0.109379D-06	0.122874D-07	0.642339D-06	-0.176659D-06	-0.560855D-04
44	-0.250934D-06	-0.286398D-06	-0.270580D-06	0.411234D-06	0.111781D-03

ESTIMATED COVARIANCE MATRIX FOR PARAMETER ESTIMATES

	36	37	38	39	40
36	0.129385D-03				
37	-0.107231D-03	0.103511D+00			
38	0.874135D-04	-0.883162D-01	0.128264D+00		
39	0.113858D-05	0.433968D-03	0.161838D-03	0.100428D-03	
40	0.801313D-06	-0.133089D-04	0.240608D-04	0.292198D-06	0.988840D-06

41	-0.641652D-06	-0.303347D-03	0.864093D-04	0.188533D-05	0.779504D-07
42	-0.606824D-06	0.459982D-04	-0.951558D-04	0.100967D-05	0.143893D-07
43	-0.472487D-06	0.215689D-04	0.231703D-04	0.962845D-07	0.138628D-07
44	-0.236938D-06	-0.895272D-04	0.104287D-03	-0.117178D-05	0.311811D-07

ESTIMATED COVARIANCE MATRIX FOR PARAMETER ESTIMATES

	41	42	43	44
41	0.146374D-04			
42	0.495152D-05	0.431242D-05		
43	0.194360D-05	0.259665D-05	0.308671D-05	
44	0.102691D-06	-0.444714D-07	0.172522D-10	0.111981D-05

ESTIMATED CORRELATION MATRIX FOR PARAMETER ESTIMATES

	1	2	3	4	5
1	1.000				
2	-0.159	1.000			
3	-0.122	0.252	1.000		
4	0.162	-0.181	-0.182	1.000	
5	-0.002	-0.033	-0.025	-0.032	1.000
6	-0.005	0.008	-0.011	-0.010	0.128
7	-0.006	0.007	0.019	0.054	-0.199
8	0.030	-0.007	0.000	-0.039	-0.090
9	-0.041	0.026	0.043	0.008	0.138
10	0.017	-0.001	-0.028	-0.001	-0.043
11	-0.007	0.033	-0.014	-0.029	-0.018
12	0.000	0.017	0.009	-0.035	-0.033
13	0.018	-0.002	0.013	-0.001	-0.034
14	-0.004	-0.011	0.018	0.010	0.042
15	0.022	0.016	0.011	-0.036	0.021
16	-0.028	0.018	-0.006	-0.011	-0.011
17	-0.012	-0.047	-0.011	-0.005	-0.003
18	-0.012	-0.028	-0.030	0.026	-0.016
19	0.005	0.010	0.006	-0.019	-0.007
20	-0.006	0.010	-0.003	0.012	-0.029
21	0.020	-0.006	-0.022	0.012	-0.017
22	-0.012	0.008	-0.007	-0.021	-0.024
23	-0.039	-0.033	0.001	0.013	0.044
24	0.009	-0.003	-0.045	0.040	-0.021
25	-0.014	0.003	0.059	0.010	-0.035
26	0.005	-0.009	-0.020	0.016	0.002

27	0.051	0.023	-0.028	-0.060	0.007
28	-0.030	-0.018	-0.009	0.028	0.000
29	0.053	0.015	0.024	0.016	-0.026
30	0.009	0.003	0.005	0.013	-0.030
31	-0.037	-0.020	0.016	0.014	0.024
32	-0.009	0.035	0.036	-0.036	-0.212
33	-0.107	0.048	0.064	-0.084	-0.002
34	-0.140	0.107	0.086	-0.144	-0.017
35	0.001	-0.015	-0.055	0.046	0.002
36	0.040	0.036	0.036	-0.013	-0.033
37	0.000	0.018	0.058	-0.049	0.003
38	0.000	-0.017	-0.048	0.042	-0.015
39	-0.017	0.019	0.007	-0.018	-0.038
40	0.033	0.000	-0.028	-0.004	0.026
41	-0.017	0.030	-0.027	0.010	0.003
42	-0.001	0.003	-0.013	0.018	-0.025
43	0.010	-0.031	-0.022	0.031	-0.023
44	0.013	0.009	0.019	0.015	0.032

ESTIMATED CORRELATION MATRIX FOR PARAMETER ESTIMATES

	6	7	8	9	10
6	1.000				
7	0.022	1.000			
8	0.059	-0.161	1.000		
9	-0.106	0.092	0.148	1.000	
10	0.036	-0.031	0.046	-0.049	1.000
11	-0.012	0.013	-0.032	-0.046	0.475
12	-0.021	-0.004	-0.026	-0.007	0.130
13	-0.013	-0.011	-0.018	-0.040	0.013
14	0.018	0.002	-0.017	0.025	-0.245
15	0.014	-0.049	0.015	0.006	-0.073
16	-0.004	0.027	0.044	0.001	0.033
17	0.001	-0.023	0.002	0.002	0.184
18	-0.025	0.006	0.005	0.021	0.074
19	0.028	0.013	-0.005	0.008	-0.010
20	-0.002	-0.004	0.008	0.058	0.033
21	-0.034	0.007	0.011	0.030	0.206
22	-0.005	-0.003	-0.001	0.019	0.125
23	-0.024	-0.041	-0.033	0.036	-0.078
24	-0.002	-0.025	-0.001	0.054	0.048
25	-0.042	0.007	0.067	0.031	-0.001
26	0.043	0.047	0.004	0.021	-0.120

27	0.034	0.021	-0.024	-0.041	-0.045
28	-0.049	0.013	-0.003	0.029	0.010
29	-0.030	0.018	-0.060	-0.073	0.002
30	-0.017	-0.039	-0.045	-0.050	-0.048
31	-0.009	-0.023	0.015	0.018	0.016
32	0.119	0.008	-0.071	-0.009	0.019
33	-0.014	0.014	0.022	0.004	0.022
34	0.012	-0.018	-0.011	-0.032	-0.006
35	0.025	-0.034	-0.040	-0.026	-0.091
36	0.010	0.011	-0.022	0.002	0.009
37	-0.006	0.026	0.030	0.005	0.085
38	-0.038	0.001	0.002	0.043	-0.045
39	0.020	0.012	0.017	0.002	-0.008
40	0.045	0.019	-0.021	-0.032	-0.026
41	0.022	-0.017	-0.054	-0.025	-0.068
42	-0.027	0.015	-0.020	0.002	-0.010
43	-0.050	0.036	-0.014	0.009	-0.019
44	0.018	-0.023	0.032	0.021	-0.039

ESTIMATED CORRELATION MATRIX FOR PARAMETER ESTIMATES

	11	12	13	14	15
11	1.000				
12	0.453	1.000			
13	-0.004	0.006	1.000		
14	-0.260	-0.090	0.008	1.000	
15	-0.173	-0.246	0.004	0.366	1.000
16	0.043	0.048	0.004	-0.233	-0.243
17	0.257	0.118	0.015	-0.196	-0.026
18	0.180	0.336	0.000	-0.057	-0.143
19	0.002	-0.039	0.068	0.110	0.235
20	0.028	0.070	0.021	-0.035	-0.039
21	0.245	0.096	-0.014	-0.180	-0.057
22	0.235	0.287	0.012	-0.089	-0.158
23	-0.084	-0.078	0.003	0.191	0.238
24	0.071	0.073	-0.007	-0.056	-0.014
25	0.013	0.025	-0.001	-0.034	-0.009
26	-0.221	-0.098	0.014	0.191	0.082
27	-0.191	-0.314	0.001	0.078	0.222
28	0.020	0.012	0.006	-0.150	-0.254
29	-0.056	-0.086	0.045	0.033	0.063
30	-0.061	-0.084	-0.022	0.054	0.045
31	0.038	0.075	-0.013	-0.026	-0.088

32	-0.038	-0.021	0.013	-0.063	-0.018
33	0.023	0.012	-0.025	-0.047	-0.021
34	0.034	0.048	0.017	-0.024	-0.028
35	-0.007	0.007	0.035	0.048	0.009
36	0.005	-0.004	0.000	0.007	0.000
37	0.007	-0.010	-0.041	-0.050	-0.006
38	-0.004	0.011	0.040	0.037	-0.003
39	-0.001	-0.003	-0.028	-0.001	-0.027
40	-0.021	-0.010	0.001	-0.021	-0.002
41	-0.021	0.011	-0.002	0.059	0.035
42	-0.015	0.015	-0.024	-0.026	-0.011
43	-0.028	0.020	-0.038	-0.041	-0.040
44	0.008	-0.008	0.010	-0.038	-0.006

ESTIMATED CORRELATION MATRIX FOR PARAMETER ESTIMATES

	16	17	18	19	20
16	1.000				
17	0.033	1.000			
18	0.086	0.357	1.000		
19	-0.201	-0.116	-0.236	1.000	
20	0.058	0.156	0.369	-0.239	1.000
21	0.016	0.229	0.079	-0.030	0.009
22	0.015	0.146	0.270	-0.088	0.085
23	-0.184	-0.069	-0.063	0.255	-0.069
24	0.029	0.179	0.291	-0.173	0.321
25	0.004	0.039	0.042	-0.048	0.025
26	-0.056	-0.199	-0.061	0.013	0.027
27	-0.069	-0.101	-0.231	0.065	-0.053
28	0.235	0.048	0.114	-0.196	0.076
29	-0.087	-0.139	-0.303	0.224	-0.276
30	-0.055	-0.079	-0.132	0.100	-0.061
31	0.041	0.065	0.081	-0.072	0.022
32	0.002	0.010	0.011	0.020	-0.017
33	-0.014	0.012	0.004	-0.018	-0.021
34	-0.060	-0.020	0.014	0.001	-0.022
35	-0.034	0.027	0.024	0.021	0.031
36	-0.020	0.013	0.013	0.001	0.017
37	0.042	-0.023	-0.019	-0.013	-0.041
38	-0.047	0.006	0.004	-0.010	0.050
39	0.017	-0.014	0.001	-0.034	0.011
40	-0.008	0.005	0.005	0.032	-0.046
41	-0.040	-0.015	0.014	0.021	0.017

42	-0.020	0.004	-0.001	0.016	-0.027
43	-0.020	-0.013	-0.007	0.024	-0.063
44	-0.034	0.014	-0.043	0.028	-0.017

ESTIMATED CORRELATION MATRIX FOR PARAMETER ESTIMATES

	21	22	23	24	25
21	1.000				
22	0.353	1.000			
23	-0.191	-0.223	1.000		
24	0.160	0.282	-0.177	1.000	
25	0.206	0.363	-0.162	0.294	1.000
26	-0.231	-0.102	0.093	-0.027	-0.040
27	-0.100	-0.235	0.086	-0.100	-0.081
28	0.031	0.079	-0.219	0.028	0.055
29	-0.023	-0.101	0.078	-0.259	-0.068
30	-0.111	-0.295	0.239	-0.235	-0.307
31	0.037	0.066	-0.059	0.045	0.041
32	0.008	-0.039	0.018	0.019	0.019
33	0.057	0.054	0.010	0.006	0.019
34	0.047	0.063	0.010	-0.010	0.007
35	-0.042	0.004	0.030	0.020	-0.030
36	-0.006	0.017	0.026	-0.008	-0.001
37	0.036	-0.009	-0.034	-0.021	0.026
38	-0.013	0.016	0.034	0.014	-0.013
39	-0.025	-0.057	-0.002	-0.024	0.007
40	0.011	0.033	0.025	-0.030	0.011
41	-0.053	-0.058	0.005	-0.020	-0.042
42	-0.004	-0.043	-0.028	-0.004	-0.013
43	0.014	0.016	-0.014	-0.008	-0.021
44	-0.018	-0.004	0.012	0.027	0.022

ESTIMATED CORRELATION MATRIX FOR PARAMETER ESTIMATES

	26	27	28	29	30
26	1.000				
27	0.349	1.000			
28	-0.143	-0.221	1.000		
29	0.082	0.282	-0.171	1.000	
30	0.171	0.250	-0.165	0.254	1.000
31	-0.163	-0.329	0.269	-0.257	-0.311
32	0.031	0.043	0.011	0.033	0.052

33	-0.015	-0.013	-0.016	0.018	0.005
34	-0.005	-0.036	-0.004	0.035	-0.022
35	0.081	0.034	0.022	0.007	0.041
36	0.024	0.016	-0.038	-0.029	0.004
37	-0.079	-0.029	-0.014	-0.007	-0.039
38	0.052	0.010	-0.010	0.005	0.026
39	0.033	0.013	0.023	-0.021	0.001
40	-0.028	-0.035	-0.007	-0.027	-0.004
41	0.049	-0.031	0.028	-0.005	0.013
42	0.010	-0.026	0.030	-0.005	0.007
43	0.021	-0.022	0.016	0.001	0.010
44	0.025	0.029	0.016	0.030	0.012

ESTIMATED CORRELATION MATRIX FOR PARAMETER ESTIMATES

	31	32	33	34	35
31	1.000				
32	-0.070	1.000			
33	-0.003	0.035	1.000		
34	0.002	0.039	0.557	1.000	
35	-0.028	0.061	-0.071	0.032	1.000
36	-0.030	0.115	-0.002	-0.008	0.030
37	0.033	-0.067	0.061	-0.037	-0.958
38	-0.034	0.058	-0.031	0.039	0.551
39	0.021	0.018	0.059	0.020	-0.194
40	0.025	0.061	-0.012	-0.024	0.025
41	-0.017	-0.021	-0.085	0.014	0.293
42	0.006	-0.011	-0.023	-0.034	-0.032
43	-0.004	0.001	0.020	-0.007	-0.066
44	-0.015	-0.021	-0.014	0.028	0.218

ESTIMATED CORRELATION MATRIX FOR PARAMETER ESTIMATES

	36	37	38	39	40
36	1.000				
37	-0.029	1.000			
38	0.021	-0.766	1.000		
39	0.010	0.135	0.045	1.000	
40	0.071	-0.042	0.068	0.029	1.000
41	-0.015	-0.246	0.063	0.049	0.020
42	-0.026	0.069	-0.128	0.049	0.007
43	-0.024	0.038	0.037	0.005	0.008

44	-0.020	-0.263	0.275	-0.110	0.030

ESTIMATED CORRELATION MATRIX FOR PARAMETER ESTIMATES

	41	42	43	44
41	1.000			
42	0.623	1.000		
43	0.289	0.712	1.000	
44	0.025	-0.020	0.000	1.000

TECHNICAL 8 OUTPUT

TECHNICAL 8 OUTPUT FOR BAYES ESTIMATION

CHAIN	BSEED
1	0
2	285380
3	253358

ITERATION	POTENTIAL SCALE REDUCTION	PARAMETER WITH HIGHEST PSR
100	1.009	33
200	1.019	39
300	1.018	37
400	1.013	38
500	1.003	5
600	1.003	1
700	1.002	4
800	1.003	4
900	1.003	4
1000	1.002	4

Kessler score model

SUMMARY OF DATA

Number of clusters 58

Size (s)	Cluster ID with Size s
11	22
18	166
26	80
28	74 205
31	71
34	65
35	192
40	106 182
41	228
45	72 170
47	50
50	165 30 75 181
53	173
57	107
59	108 40
60	81 226 84
63	114
66	93
68	61 4
69	162
70	83 169
71	206
72	116 36
73	69
76	92
79	68
84	174
85	145 154
88	152
95	6
104	144 98 2
114	178
118	156
123	202
131	142

159	122
218	24
239	209
283	204
331	125
472	217
954	110
1083	100

COVARIANCE COVERAGE OF DATA

Minimum covariance coverage value 0.100

Number of missing data patterns 9

PROPORTION OF DATA PRESENT

Covariance Coverage

	PHY15	KES15	KES16	CHOICE15	BULLY15
PHY15	0.958				
KES15	0.932	0.932			
KES16	0.958	0.932	1.000		
CHOICE15	0.925	0.925	0.925	0.925	
BULLY15	0.925	0.925	0.925	0.925	0.925
PRESS15	0.925	0.925	0.925	0.925	0.925
DUTIES15	0.926	0.926	0.926	0.925	0.925
PRAFAC	0.958	0.932	1.000	0.925	0.925

Covariance Coverage

	PRESS15	DUTIES15	PRAFAC
PRESS15	0.925		
DUTIES15	0.925	0.926	
PRAFAC	0.925	0.926	1.000

UNIVARIATE SAMPLE STATISTICS

UNIVARIATE HIGHER-ORDER MOMENT DESCRIPTIVE STATISTICS

Variable/ Sample Size	Mean/ Variance	Skewness/ Kurtosis	Minimum/ Maximum	% with Min/Max	Percentiles 20%/60%	40%/80%	Median
PHY15	1.956	0.559	1.000	28.98%	1.000	2.000	2.000
6676.000	0.605	0.164	5.000	0.24%	2.000	3.000	
KES15	0.605	1.665	0.000	18.88%	0.167	0.333	0.500
6495.000	0.391	3.340	4.000	0.11%	0.500	1.000	
KES16	0.673	1.459	0.000	15.84%	0.167	0.333	0.500
6968.000	0.430	2.260	4.000	0.07%	0.667	1.167	
CHOICE15	0.000	-0.355	-2.151	8.93%	-1.151	-0.151	-0.151
6447.000	1.137	-0.460	1.849	7.91%	0.849	0.849	
BULLY15	0.000	3.100	-0.200	85.49%	-0.200	-0.200	-0.200
6446.000	0.292	10.556	3.800	0.16%	-0.200	-0.200	
PRESS15	0.000	0.260	-1.525	18.02%	-0.525	-0.525	0.475
6447.000	1.060	-0.377	2.475	3.71%	0.475	0.475	
DUTIES15	0.000	-1.160	-3.067	2.54%	-1.067	-0.067	-0.067
6450.000	0.895	1.352	0.933	36.40%	-0.067	0.933	
PRAFAC	-0.185	0.245	-2.338	1.72%	-1.448	-0.527	-0.318
58.000	1.345	-0.636	2.338	1.72%	0.080	0.752	

THE MODEL ESTIMATION TERMINATED NORMALLY

USE THE FBITERATIONS OPTION TO INCREASE THE NUMBER OF ITERATIONS BY A FACTOR
OF AT LEAST TWO TO CHECK CONVERGENCE AND THAT THE PSR VALUE DOES NOT INCREASE.

MODEL FIT INFORMATION

Number of Free Parameters 44

Information Criteria

Deviance (DIC) 100102.952
Estimated Number of Parameters (pD) 153.699

MODEL RESULTS

	Estimate	Posterior S.D.	One-Tailed P-Value	95% C.I. Lower 2.5%	Upper 2.5%	Significance
**Within Level**						
KES16 ON						
PHY15	0.057	0.008	0.000	0.041	0.072	*
CHOICE15	-0.013	0.006	0.009	-0.025	-0.002	*
BULLY15	0.049	0.012	0.000	0.025	0.072	*
DUTIES15	-0.029	0.007	0.000	-0.042	-0.016	*
PRESS15	-0.010	0.006	0.067	-0.021	0.003	
KES15 WITH						
PHY15	0.166	0.006	0.000	0.154	0.180	*
CHOICE15	-0.126	0.009	0.000	-0.142	-0.108	*
BULLY15	0.083	0.005	0.000	0.074	0.092	*
PRESS15	0.148	0.008	0.000	0.131	0.164	*
DUTIES15	-0.144	0.008	0.000	-0.159	-0.130	*
PHY15 WITH						
CHOICE15	-0.123	0.011	0.000	-0.144	-0.101	*
BULLY15	0.048	0.005	0.000	0.039	0.058	*
PRESS15	0.110	0.010	0.000	0.091	0.130	*
DUTIES15	-0.092	0.009	0.000	-0.110	-0.074	*
CHOICE15 WITH						
BULLY15	-0.082	0.007	0.000	-0.097	-0.068	*
PRESS15	-0.125	0.014	0.000	-0.152	-0.099	*
DUTIES15	0.185	0.013	0.000	0.159	0.209	*
BULLY15 WITH						
PRESS15	0.127	0.007	0.000	0.114	0.141	*
DUTIES15	-0.097	0.007	0.000	-0.111	-0.085	*
PRESS15 WITH						
DUTIES15	-0.211	0.013	0.000	-0.236	-0.187	*
Means						
CHOICE15	-0.001	0.013	0.479	-0.026	0.024	
BULLY15	0.001	0.007	0.459	-0.013	0.014	
PRESS15	0.001	0.013	0.469	-0.024	0.027	
DUTIES15	-0.001	0.012	0.463	-0.024	0.021	

```
 Variances
    PHY15              0.601      0.010      0.000      0.582      0.621      *
    KES15              0.389      0.007      0.000      0.376      0.403      *
    CHOICE15           1.141      0.021      0.000      1.100      1.181      *
    BULLY15            0.293      0.005      0.000      0.283      0.303      *
    PRESS15            1.062      0.019      0.000      1.026      1.099      *
    DUTIES15           0.898      0.016      0.000      0.868      0.930      *

 Residual Variances
    KES16              0.223      0.004      0.000      0.215      0.231      *

Between Level

 S1         ON
    PRAFAC            -0.041      0.016      0.007     -0.073     -0.011      *

 KES16      ON
    PRAFAC            -0.015      0.008      0.028     -0.030      0.000
    PHY15              0.026      0.390      0.455     -0.594      0.620
    KES15              0.797      0.360      0.012      0.205      1.397      *

 PHY15    WITH
    KES15              0.005      0.002      0.001      0.001      0.010      *

 Means
    PHY15              1.951      0.018      0.000      1.915      1.987      *
    KES15              0.602      0.014      0.000      0.576      0.631      *

 Intercepts
    KES16              0.130      0.578      0.362     -0.740      1.051
    S1                 0.654      0.019      0.000      0.617      0.690      *

 Variances
    PHY15              0.009      0.004      0.000      0.003      0.018      *
    KES15              0.005      0.002      0.000      0.003      0.009      *

 Residual Variances
    KES16              0.001      0.001      0.000      0.000      0.003      *
    S1                 0.008      0.004      0.000      0.003      0.018      *


STANDARDIZED MODEL RESULTS
```

STDYX Standardization

	Estimate	Posterior S.D.	One-Tailed P-Value	95% C.I. Lower 2.5%	95% C.I. Upper 2.5%	Significance
Within-Level Standardized Estimates Averaged Over Clusters						
S1 | KES16 ON						
KES15	0.630	0.010	0.000	0.609	0.648	*
KES16 ON						
PHY15	0.068	0.010	0.000	0.049	0.086	*
CHOICE15	-0.022	0.010	0.010	-0.041	-0.003	*
BULLY15	0.040	0.010	0.000	0.022	0.060	*
DUTIES15	-0.042	0.010	0.000	-0.062	-0.023	*
PRESS15	-0.015	0.010	0.060	-0.033	0.005	
KES15 WITH						
PHY15	0.343	0.011	0.000	0.322	0.365	*
CHOICE15	-0.188	0.012	0.000	-0.212	-0.165	*
BULLY15	0.246	0.012	0.000	0.222	0.270	*
PRESS15	0.230	0.012	0.000	0.207	0.253	*
DUTIES15	-0.244	0.012	0.000	-0.267	-0.222	*
PHY15 WITH						
CHOICE15	-0.148	0.013	0.000	-0.172	-0.123	*
BULLY15	0.115	0.012	0.000	0.091	0.139	*
PRESS15	0.138	0.012	0.000	0.114	0.162	*
DUTIES15	-0.124	0.012	0.000	-0.149	-0.100	*
CHOICE15 WITH						
BULLY15	-0.143	0.012	0.000	-0.166	-0.118	*
PRESS15	-0.113	0.012	0.000	-0.138	-0.091	*
DUTIES15	0.183	0.012	0.000	0.160	0.206	*
BULLY15 WITH						
PRESS15	0.228	0.012	0.000	0.205	0.252	*
DUTIES15	-0.189	0.012	0.000	-0.213	-0.165	*
PRESS15 WITH						
DUTIES15	-0.216	0.012	0.000	-0.238	-0.192	*
Means						
CHOICE15	-0.001	0.013	0.461	-0.026	0.024	

```
    BULLY15            0.001       0.012      0.456      -0.023       0.025
    PRESS15            0.001       0.012      0.463      -0.023       0.026
    DUTIES15          -0.002       0.012      0.448      -0.025       0.022

 Variances
    PHY15              1.000       0.000      0.000       1.000       1.000
    KES15              1.000       0.000      0.000       1.000       1.000
    CHOICE15           1.000       0.000      0.000       1.000       1.000
    BULLY15            1.000       0.000      0.000       1.000       1.000
    PRESS15            1.000       0.000      0.000       1.000       1.000
    DUTIES15           1.000       0.000      0.000       1.000       1.000

 Residual Variances
    KES16              0.533       0.011      0.000       0.513       0.555      *

Between Level

 S1         ON
    PRAFAC            -0.302       0.113      0.007      -0.514      -0.078      *

 KES16      ON
    PRAFAC            -0.151       0.094      0.028      -0.361       0.001
    PHY15              0.035       0.413      0.455      -0.703       0.705
    KES15              0.849       0.362      0.012       0.214       1.433      *

 PHY15    WITH
    KES15              0.729       0.172      0.001       0.279       0.960      *

 Means
    PHY15             21.101       5.329      0.000      14.489      34.903      *
    KES15              8.337       1.455      0.000       6.119      11.738      *

 Intercepts
    KES16              1.884       8.414      0.362     -10.287      16.950
    S1                 6.714       1.469      0.000       4.651      10.301      *

 Variances
    PHY15              1.000       0.000      0.000       1.000       1.000
    KES15              1.000       0.000      0.000       1.000       1.000

 Residual Variances
    KES16              0.155       0.133      0.000       0.009       0.510      *
    S1                 0.909       0.069      0.000       0.736       0.993      *
```

R-SQUARE

Within-Level R-Square Averaged Across Clusters

Variable	Estimate	Posterior S.D.	One-Tailed P-Value	95% C.I. Lower 2.5%	Upper 2.5%
KES16	0.467	0.011	0.000	0.445	0.487

Between Level

Variable	Estimate	Posterior S.D.	One-Tailed P-Value	95% C.I. Lower 2.5%	Upper 2.5%
KES16	0.845	0.133	0.000	0.490	0.991

Variable	Estimate	Posterior S.D.	One-Tailed P-Value	95% C.I. Lower 2.5%	Upper 2.5%
S1	0.091	0.069	0.000	0.007	0.264

TECHNICAL 1 OUTPUT

PARAMETER SPECIFICATION FOR WITHIN

NU

PHY15	KES15	KES16	CHOICE15	BULLY15
0	0	0	0	0

NU

PRESS15	DUTIES15
0	0

LAMBDA

PHY15	KES15	KES16	CHOICE15	BULLY15

PHY15	0	0	0	0	0
KES15	0	0	0	0	0
KES16	0	0	0	0	0
CHOICE15	0	0	0	0	0
BULLY15	0	0	0	0	0
PRESS15	0	0	0	0	0
DUTIES15	0	0	0	0	0

LAMBDA

	PRESS15	DUTIES15
PHY15	0	0
KES15	0	0
KES16	0	0
CHOICE15	0	0
BULLY15	0	0
PRESS15	0	0
DUTIES15	0	0

THETA

	PHY15	KES15	KES16	CHOICE15	BULLY15
PHY15	0				
KES15	0	0			
KES16	0	0	0		
CHOICE15	0	0	0	0	
BULLY15	0	0	0	0	0
PRESS15	0	0	0	0	0
DUTIES15	0	0	0	0	0

THETA

	PRESS15	DUTIES15
PRESS15	0	
DUTIES15	0	0

ALPHA

PHY15	KES15	KES16	CHOICE15	BULLY15
0	0	0	1	2

ALPHA

PRESS15	DUTIES15
3	4

BETA

	PHY15	KES15	KES16	CHOICE15	BULLY15
PHY15	0	0	0	0	0
KES15	0	0	0	0	0
KES16	5	0	0	6	7
CHOICE15	0	0	0	0	0
BULLY15	0	0	0	0	0
PRESS15	0	0	0	0	0
DUTIES15	0	0	0	0	0

BETA

	PRESS15	DUTIES15
PHY15	0	0
KES15	0	0
KES16	8	9
CHOICE15	0	0
BULLY15	0	0
PRESS15	0	0
DUTIES15	0	0

PSI

	PHY15	KES15	KES16	CHOICE15	BULLY15
PHY15	10				
KES15	11	12			
KES16	0	0	13		
CHOICE15	14	15	0	16	
BULLY15	17	18	0	19	20
PRESS15	21	22	0	23	24
DUTIES15	26	27	0	28	29

PSI

	PRESS15	DUTIES15
PRESS15	25	
DUTIES15	30	31

PARAMETER SPECIFICATION FOR BETWEEN

NU

PHY15	KES15	KES16	PRAFAC
0	0	0	0

LAMBDA

	S1	PHY15	KES15	KES16	PRAFAC
PHY15	0	0	0	0	0
KES15	0	0	0	0	0
KES16	0	0	0	0	0
PRAFAC	0	0	0	0	0

THETA

	PHY15	KES15	KES16	PRAFAC
PHY15	0			
KES15	0	0		
KES16	0	0	0	
PRAFAC	0	0	0	0

ALPHA

S1	PHY15	KES15	KES16	PRAFAC
32	33	34	35	0

BETA

	S1	PHY15	KES15	KES16	PRAFAC
S1	0	0	0	0	36

PHY15	0	0	0	0	0
KES15	0	0	0	0	0
KES16	0	37	38	0	39
PRAFAC	0	0	0	0	0

PSI

	S1	PHY15	KES15	KES16	PRAFAC
S1	40				
PHY15	0	41			
KES15	0	42	43		
KES16	0	0	0	44	
PRAFAC	0	0	0	0	0

STARTING VALUES FOR WITHIN

NU

PHY15	KES15	KES16	CHOICE15	BULLY15
0.000	0.000	0.000	0.000	0.000

NU

PRESS15	DUTIES15
0.000	0.000

LAMBDA

	PHY15	KES15	KES16	CHOICE15	BULLY15
PHY15	1.000	0.000	0.000	0.000	0.000
KES15	0.000	1.000	0.000	0.000	0.000
KES16	0.000	0.000	1.000	0.000	0.000
CHOICE15	0.000	0.000	0.000	1.000	0.000
BULLY15	0.000	0.000	0.000	0.000	1.000
PRESS15	0.000	0.000	0.000	0.000	0.000
DUTIES15	0.000	0.000	0.000	0.000	0.000

LAMBDA

	PRESS15	DUTIES15
PHY15	0.000	0.000
KES15	0.000	0.000
KES16	0.000	0.000
CHOICE15	0.000	0.000
BULLY15	0.000	0.000
PRESS15	1.000	0.000
DUTIES15	0.000	1.000

THETA

	PHY15	KES15	KES16	CHOICE15	BULLY15
PHY15	0.000				
KES15	0.000	0.000			
KES16	0.000	0.000	0.000		
CHOICE15	0.000	0.000	0.000	0.000	
BULLY15	0.000	0.000	0.000	0.000	0.000
PRESS15	0.000	0.000	0.000	0.000	0.000
DUTIES15	0.000	0.000	0.000	0.000	0.000

THETA

	PRESS15	DUTIES15
PRESS15	0.000	
DUTIES15	0.000	0.000

ALPHA

PHY15	KES15	KES16	CHOICE15	BULLY15
0.000	0.000	0.000	0.000	0.000

ALPHA

PRESS15	DUTIES15
0.000	0.000

BETA

PHY15	KES15	KES16	CHOICE15	BULLY15

PHY15	0.000	0.000	0.000	0.000	0.000
KES15	0.000	0.000	0.000	0.000	0.000
KES16	0.000	0.000	0.000	0.000	0.000
CHOICE15	0.000	0.000	0.000	0.000	0.000
BULLY15	0.000	0.000	0.000	0.000	0.000
PRESS15	0.000	0.000	0.000	0.000	0.000
DUTIES15	0.000	0.000	0.000	0.000	0.000

BETA

	PRESS15	DUTIES15
PHY15	0.000	0.000
KES15	0.000	0.000
KES16	0.000	0.000
CHOICE15	0.000	0.000
BULLY15	0.000	0.000
PRESS15	0.000	0.000
DUTIES15	0.000	0.000

PSI

	PHY15	KES15	KES16	CHOICE15	BULLY15
PHY15	0.303				
KES15	0.000	0.195			
KES16	0.000	0.000	0.215		
CHOICE15	0.000	0.000	0.000	0.569	
BULLY15	0.000	0.000	0.000	0.000	0.146
PRESS15	0.000	0.000	0.000	0.000	0.000
DUTIES15	0.000	0.000	0.000	0.000	0.000

PSI

	PRESS15	DUTIES15
PRESS15	0.530	
DUTIES15	0.000	0.448

STARTING VALUES FOR BETWEEN

NU

PHY15	KES15	KES16	PRAFAC
0.000	0.000	0.000	0.000

LAMBDA

	S1	PHY15	KES15	KES16	PRAFAC
PHY15	0.000	1.000	0.000	0.000	0.000
KES15	0.000	0.000	1.000	0.000	0.000
KES16	0.000	0.000	0.000	1.000	0.000
PRAFAC	0.000	0.000	0.000	0.000	1.000

THETA

	PHY15	KES15	KES16	PRAFAC
PHY15	0.000			
KES15	0.000	0.000		
KES16	0.000	0.000	0.000	
PRAFAC	0.000	0.000	0.000	0.000

ALPHA

S1	PHY15	KES15	KES16	PRAFAC
0.000	1.956	0.605	0.673	0.000

BETA

	S1	PHY15	KES15	KES16	PRAFAC
S1	0.000	0.000	0.000	0.000	0.000
PHY15	0.000	0.000	0.000	0.000	0.000
KES15	0.000	0.000	0.000	0.000	0.000
KES16	0.000	0.000	0.000	0.000	0.000
PRAFAC	0.000	0.000	0.000	0.000	0.000

PSI

	S1	PHY15	KES15	KES16	PRAFAC
S1	1.000				

PHY15	0.000	0.303			
KES15	0.000	0.000	0.195		
KES16	0.000	0.000	0.000	0.215	
PRAFAC	0.000	0.000	0.000	0.000	0.500

PRIORS FOR ALL PARAMETERS	PRIOR MEAN	PRIOR VARIANCE	PRIOR STD. DEV.
Parameter 1~N(0.000,infinity)	0.0000	infinity	infinity
Parameter 2~N(0.000,infinity)	0.0000	infinity	infinity
Parameter 3~N(0.000,infinity)	0.0000	infinity	infinity
Parameter 4~N(0.000,infinity)	0.0000	infinity	infinity
Parameter 5~N(0.000,infinity)	0.0000	infinity	infinity
Parameter 6~N(0.000,infinity)	0.0000	infinity	infinity
Parameter 7~N(0.000,infinity)	0.0000	infinity	infinity
Parameter 8~N(0.000,infinity)	0.0000	infinity	infinity
Parameter 9~N(0.000,infinity)	0.0000	infinity	infinity
Parameter 10~IW(0.000,-7)	infinity	infinity	infinity
Parameter 11~IW(0.000,-7)	infinity	infinity	infinity
Parameter 12~IW(0.000,-7)	infinity	infinity	infinity
Parameter 13~IG(-1.000,0.000)	infinity	infinity	infinity
Parameter 14~IW(0.000,-7)	infinity	infinity	infinity
Parameter 15~IW(0.000,-7)	infinity	infinity	infinity
Parameter 16~IW(0.000,-7)	infinity	infinity	infinity
Parameter 17~IW(0.000,-7)	infinity	infinity	infinity
Parameter 18~IW(0.000,-7)	infinity	infinity	infinity
Parameter 19~IW(0.000,-7)	infinity	infinity	infinity
Parameter 20~IW(0.000,-7)	infinity	infinity	infinity
Parameter 21~IW(0.000,-7)	infinity	infinity	infinity
Parameter 22~IW(0.000,-7)	infinity	infinity	infinity
Parameter 23~IW(0.000,-7)	infinity	infinity	infinity
Parameter 24~IW(0.000,-7)	infinity	infinity	infinity
Parameter 25~IW(0.000,-7)	infinity	infinity	infinity
Parameter 26~IW(0.000,-7)	infinity	infinity	infinity
Parameter 27~IW(0.000,-7)	infinity	infinity	infinity
Parameter 28~IW(0.000,-7)	infinity	infinity	infinity
Parameter 29~IW(0.000,-7)	infinity	infinity	infinity
Parameter 30~IW(0.000,-7)	infinity	infinity	infinity
Parameter 31~IW(0.000,-7)	infinity	infinity	infinity
Parameter 32~N(0.000,infinity)	0.0000	infinity	infinity
Parameter 33~N(0.000,infinity)	0.0000	infinity	infinity
Parameter 34~N(0.000,infinity)	0.0000	infinity	infinity
Parameter 35~N(0.000,infinity)	0.0000	infinity	infinity

Parameter 36~N(0.000,infinity)	0.0000	infinity	infinity
Parameter 37~N(0.000,infinity)	0.0000	infinity	infinity
Parameter 38~N(0.000,infinity)	0.0000	infinity	infinity
Parameter 39~N(0.000,infinity)	0.0000	infinity	infinity
Parameter 40~IG(-1.000,0.000)	infinity	infinity	infinity
Parameter 41~IW(0.000,-3)	infinity	infinity	infinity
Parameter 42~IW(0.000,-3)	infinity	infinity	infinity
Parameter 43~IW(0.000,-3)	infinity	infinity	infinity
Parameter 44~IG(-1.000,0.000)	infinity	infinity	infinity

TECHNICAL 3 OUTPUT

ESTIMATED COVARIANCE MATRIX FOR PARAMETER ESTIMATES

	1	2	3	4	5
1	0.179306D-03				
2	-0.142852D-04	0.445934D-04			
3	-0.208264D-04	0.214302D-04	0.162020D-03		
4	0.255912D-04	-0.141064D-04	-0.273633D-04	0.140640D-03	
5	-0.648108D-06	-0.996805D-06	-0.275744D-05	-0.245803D-05	0.631897D-04
6	-0.402903D-06	0.591053D-07	-0.854305D-06	-0.954809D-06	0.489375D-05
7	-0.218363D-05	0.550146D-06	0.171903D-05	0.742676D-05	-0.351742D-05
8	0.178006D-05	-0.911362D-07	0.126615D-05	-0.372145D-05	-0.180888D-05
9	-0.285365D-05	0.221352D-05	0.413360D-05	-0.648808D-06	0.845983D-06
10	0.192456D-05	0.526022D-06	-0.332938D-05	-0.428879D-06	-0.374957D-05
11	-0.554081D-06	0.183380D-05	-0.124029D-05	-0.209991D-05	-0.180640D-05
12	0.326567D-06	0.860679D-06	0.349493D-06	-0.248361D-05	-0.205696D-05
13	0.100395D-05	-0.163243D-06	0.550721D-06	-0.135636D-06	-0.771034D-06
14	-0.898402D-06	-0.158481D-05	0.207117D-05	0.917288D-06	0.204837D-05
15	0.293951D-05	0.523742D-06	0.395940D-06	-0.373337D-05	0.369015D-06
16	-0.836147D-05	0.312074D-05	-0.608591D-06	-0.297118D-05	-0.682215D-06
17	-0.832274D-06	-0.129028D-05	-0.780193D-06	-0.566340D-06	-0.289014D-06
18	-0.238920D-06	-0.858595D-06	-0.235543D-05	0.203299D-05	-0.633035D-06
19	0.403186D-06	0.382150D-06	0.565203D-06	-0.182955D-05	-0.151979D-06
20	-0.192179D-06	0.299649D-06	-0.548003D-06	0.104273D-05	-0.123602D-05
21	0.278621D-05	-0.384582D-06	-0.201048D-05	0.138004D-05	-0.190071D-05
22	-0.959863D-06	0.555304D-06	-0.237466D-06	-0.227877D-05	-0.283071D-05
23	-0.703193D-05	-0.331896D-05	-0.736475D-06	0.231867D-05	0.511385D-05
24	0.108463D-05	-0.885373D-07	-0.406206D-05	0.337842D-05	-0.280078D-05
25	-0.366004D-05	0.728764D-06	0.154657D-04	0.134300D-05	-0.602846D-05
26	0.685483D-06	-0.789686D-06	-0.247754D-05	0.124508D-05	0.110180D-05
27	0.524199D-05	0.137727D-05	-0.282916D-05	-0.575348D-05	0.930383D-06

28	-0.539892D-05	-0.145366D-05	-0.109535D-05	0.458048D-05	0.122732D-05
29	0.436791D-05	0.834777D-06	0.245617D-05	0.768813D-06	-0.162498D-05
30	0.129731D-05	0.290354D-06	0.387683D-06	0.190121D-05	-0.190723D-05
31	-0.797209D-05	-0.244376D-05	0.330952D-05	0.303909D-05	0.244613D-05
32	-0.550964D-05	0.325020D-05	0.154795D-04	-0.127500D-04	-0.257280D-04
33	-0.235482D-04	0.688780D-05	0.127501D-04	-0.167646D-04	0.205579D-05
34	-0.268604D-04	0.947890D-05	0.166056D-04	-0.233939D-04	-0.651784D-06
35	0.182393D-03	-0.145249D-03	-0.154500D-03	0.487914D-05	0.254475D-03
36	0.191197D-05	0.393501D-05	0.612140D-05	-0.178309D-05	-0.598187D-05
37	-0.102660D-03	0.104238D-03	0.113174D-03	0.785575D-05	-0.165272D-03
38	0.387595D-04	-0.964083D-04	-0.120244D-03	-0.208835D-04	0.116195D-03
39	-0.153702D-05	-0.299230D-07	0.117217D-05	-0.109737D-05	-0.113469D-05
40	0.220397D-05	-0.166391D-06	0.181008D-06	-0.975340D-06	0.894166D-06
41	-0.486143D-06	0.693200D-06	-0.109792D-05	0.218986D-07	0.414955D-06
42	-0.648938D-07	0.316183D-07	-0.312153D-06	0.523049D-06	-0.283975D-06
43	0.177839D-06	-0.380702D-06	-0.545741D-06	0.844831D-06	-0.662376D-07
44	0.262884D-07	0.151252D-06	0.369828D-06	-0.270486D-06	0.420231D-06

ESTIMATED COVARIANCE MATRIX FOR PARAMETER ESTIMATES

	6	7	8	9	10
6	0.347558D-04				
7	0.416175D-05	0.145073D-03			
8	0.122209D-05	-0.104005D-04	0.363995D-04		
9	-0.511069D-05	0.725117D-05	0.570549D-05	0.443731D-04	
10	0.229904D-05	-0.420431D-05	0.242342D-05	-0.259207D-05	0.106447D-03
11	-0.682398D-07	0.186603D-05	-0.138456D-05	-0.138185D-05	0.314982D-04
12	-0.139732D-05	-0.597198D-06	-0.111809D-05	0.381888D-06	0.920922D-05
13	0.952988D-08	-0.459487D-06	-0.511309D-06	-0.796329D-06	0.610034D-06
14	0.109742D-06	0.904835D-06	-0.817103D-06	0.143794D-05	-0.285834D-04
15	0.817012D-06	-0.509646D-05	0.490994D-06	-0.265973D-06	-0.584255D-05
16	-0.890966D-06	0.351774D-05	0.431476D-05	0.187151D-06	0.551507D-05
17	0.718150D-07	-0.129040D-05	-0.419280D-06	-0.402682D-06	0.987421D-05
18	-0.549156D-06	0.435979D-06	-0.606895D-07	0.273912D-06	0.323953D-05
19	0.146466D-05	0.541611D-06	-0.207438D-06	0.480032D-06	-0.820908D-07
20	-0.149253D-06	0.480189D-07	0.424733D-06	0.184753D-05	0.153645D-05
21	-0.220900D-05	0.858207D-06	-0.727298D-06	0.321180D-05	0.215796D-04
22	-0.329647D-06	-0.279500D-06	0.416029D-06	0.186061D-05	0.108216D-04
23	-0.120922D-05	-0.733051D-05	-0.262223D-05	0.190010D-05	-0.110790D-04
24	0.130167D-06	-0.334909D-05	-0.354013D-06	0.340157D-05	0.335807D-05
25	-0.387308D-05	0.147527D-05	0.760180D-05	0.539201D-05	-0.779399D-06
26	0.189055D-05	0.437865D-05	0.123147D-05	0.921564D-06	-0.114471D-04
27	0.142460D-05	0.177771D-05	-0.752298D-06	-0.155875D-05	-0.337851D-05

28	-0.333000D-05	0.344722D-05	0.324339D-06	0.148765D-05	0.115442D-05
29	-0.149612D-05	0.100309D-05	-0.252014D-05	-0.269727D-05	0.557301D-06
30	-0.994823D-06	-0.603671D-05	-0.325040D-05	-0.438378D-05	-0.600702D-05
31	-0.670003D-06	-0.275243D-05	0.199672D-05	0.789050D-06	0.245165D-05
32	0.146708D-04	-0.121470D-04	-0.152797D-04	0.632445D-05	0.509580D-05
33	0.886615D-06	0.571995D-05	0.794614D-06	0.351234D-05	0.504077D-05
34	0.412602D-06	-0.165771D-05	0.399923D-07	-0.816986D-06	-0.777068D-06
35	0.596920D-04	-0.517340D-03	0.239315D-04	-0.161320D-03	-0.219187D-03
36	-0.206713D-05	0.916069D-06	-0.416411D-05	0.221287D-05	0.804816D-06
37	-0.928274D-05	0.331974D-03	-0.388364D-04	0.850694D-04	0.133089D-03
38	-0.679421D-04	-0.220132D-03	0.864048D-04	-0.811555D-05	-0.684543D-04
39	0.808883D-06	0.260480D-06	0.124529D-05	0.583802D-06	0.121381D-05
40	-0.179724D-06	-0.281478D-05	-0.124983D-06	-0.920900D-06	-0.708424D-07
41	0.338351D-06	-0.694158D-06	-0.852383D-06	-0.734523D-06	-0.242972D-05
42	-0.120567D-06	0.983537D-07	-0.404619D-06	0.119534D-06	-0.252411D-06
43	-0.332587D-06	0.457945D-06	-0.217727D-06	0.226716D-07	-0.560448D-06
44	0.121758D-06	0.180452D-06	0.476415D-07	0.215065D-06	-0.167864D-06

ESTIMATED COVARIANCE MATRIX FOR PARAMETER ESTIMATES

	11	12	13	14	15
11	0.401388D-04				
12	0.188867D-04	0.439629D-04			
13	-0.153635D-06	-0.307092D-06	0.150343D-04		
14	-0.189012D-04	-0.711003D-05	0.334744D-06	0.120773D-03	
15	-0.951367D-05	-0.141468D-04	-0.294216D-06	0.348820D-04	0.734804D-04
16	0.559667D-05	0.632350D-05	0.117956D-05	-0.519525D-04	-0.418043D-04
17	0.854820D-05	0.394666D-05	0.164867D-06	-0.114066D-04	-0.149019D-05
18	0.519488D-05	0.101875D-04	-0.261012D-07	-0.307199D-05	-0.589101D-05
19	0.150821D-07	-0.232911D-05	0.184618D-05	0.835948D-05	0.148404D-04
20	0.868158D-06	0.260493D-05	0.384973D-06	-0.228560D-05	-0.188734D-05
21	0.162850D-04	0.620516D-05	-0.441338D-06	-0.207363D-04	-0.513879D-05
22	0.121465D-04	0.159338D-04	-0.372929D-06	-0.827445D-05	-0.110832D-04
23	-0.755307D-05	-0.716253D-05	0.145917D-06	0.294045D-04	0.270594D-04
24	0.302929D-05	0.391952D-05	-0.691061D-06	-0.437308D-05	-0.119974D-05
25	0.174841D-05	0.376355D-05	-0.352933D-06	-0.700077D-05	-0.153176D-05
26	-0.132812D-04	-0.561636D-05	0.788669D-06	0.198548D-04	0.679960D-05
27	-0.877945D-05	-0.157026D-04	0.465857D-06	0.657176D-05	0.139806D-04
28	0.176564D-05	0.117110D-05	0.203906D-06	-0.211893D-04	-0.274302D-04
29	-0.234937D-05	-0.414112D-05	0.123316D-05	0.206768D-05	0.345062D-05
30	-0.438822D-05	-0.653567D-05	-0.947672D-06	0.769275D-05	0.397735D-05
31	0.371306D-05	0.699282D-05	-0.733153D-06	-0.438446D-05	-0.108821D-04
32	-0.319233D-05	-0.504377D-05	-0.507531D-06	-0.483952D-05	0.374922D-05

33	0.244318D-05	0.964824D-06	-0.292060D-05	-0.946111D-05	-0.528968D-06
34	0.226949D-05	0.401794D-05	0.937380D-06	-0.364883D-05	-0.290093D-05
35	0.723282D-04	-0.918973D-06	-0.325042D-04	-0.170339D-03	0.645847D-04
36	0.123944D-05	-0.172448D-05	0.787011D-06	0.513706D-06	0.694732D-06
37	-0.376880D-04	-0.334390D-05	0.206081D-04	0.112635D-03	-0.480660D-04
38	0.286776D-05	0.785913D-05	-0.135976D-04	-0.851923D-04	0.490164D-04
39	0.932700D-06	0.335286D-07	-0.126246D-05	-0.500510D-06	-0.207396D-05
40	-0.455511D-06	0.326928D-09	-0.944779D-07	-0.157377D-05	0.797957D-06
41	-0.544573D-06	0.186282D-06	0.265738D-06	0.206298D-05	0.183810D-05
42	-0.195948D-06	0.310173D-06	-0.106698D-06	-0.462034D-06	0.602716D-07
43	-0.225852D-06	0.320571D-06	-0.236574D-06	-0.695421D-06	-0.605899D-06
44	-0.294147D-07	-0.660219D-07	-0.218058D-07	-0.199088D-06	0.107972D-06

ESTIMATED COVARIANCE MATRIX FOR PARAMETER ESTIMATES

	16	17	18	19	20
16	0.424693D-03				
17	0.353812D-05	0.273074D-04			
18	0.799590D-05	0.841083D-05	0.203840D-04		
19	-0.297083D-04	-0.450622D-05	-0.792574D-05	0.529738D-04	
20	0.611474D-05	0.409628D-05	0.874326D-05	-0.908574D-05	0.268921D-04
21	0.314505D-05	0.120464D-04	0.320799D-05	-0.197815D-05	0.376898D-06
22	0.178734D-05	0.603072D-05	0.102358D-04	-0.527051D-05	0.378963D-05
23	-0.503567D-04	-0.492412D-05	-0.407466D-05	0.250571D-04	-0.494750D-05
24	0.406612D-05	0.640502D-05	0.912556D-05	-0.883507D-05	0.116332D-04
25	0.821815D-06	0.364837D-05	0.391791D-05	-0.652525D-05	0.250349D-05
26	-0.107191D-04	-0.100881D-04	-0.274438D-05	0.111805D-05	0.115304D-05
27	-0.100132D-04	-0.425558D-05	-0.803259D-05	0.362646D-05	-0.216738D-05
28	0.611634D-04	0.342851D-05	0.676659D-05	-0.182711D-04	0.511549D-05
29	-0.115286D-04	-0.473744D-05	-0.911888D-05	0.107341D-04	-0.949062D-05
30	-0.135414D-04	-0.485980D-05	-0.734051D-05	0.890040D-05	-0.387954D-05
31	0.124052D-04	0.565005D-05	0.555473D-05	-0.800198D-05	0.172570D-05
32	0.104461D-04	0.114143D-05	0.148721D-05	0.180509D-05	-0.170282D-05
33	-0.661647D-05	0.360905D-06	-0.891289D-06	-0.822393D-06	-0.177732D-05
34	-0.155562D-04	-0.996719D-06	0.473268D-06	-0.212494D-06	-0.219547D-05
35	-0.127334D-03	0.432696D-04	-0.772058D-05	0.414456D-04	0.344125D-04
36	-0.863283D-05	-0.525496D-06	0.211651D-06	-0.582390D-06	0.161265D-06
37	0.948577D-04	-0.312310D-04	0.769584D-06	-0.201468D-04	-0.381066D-04
38	-0.997180D-04	0.273283D-04	0.995418D-05	-0.238745D-05	0.668379D-04
39	0.525267D-05	-0.240805D-06	0.449467D-06	-0.279807D-05	0.836683D-06
40	0.736352D-08	0.552761D-07	-0.548919D-06	0.136964D-05	-0.675746D-06
41	-0.284160D-05	-0.312521D-07	0.517277D-06	0.331706D-06	0.281405D-06
42	-0.136700D-05	0.371294D-07	-0.587203D-07	0.461376D-06	-0.272344D-06

43	-0.879986D-06	-0.126312D-06	-0.629729D-07	0.393095D-06	-0.548747D-06
44	-0.561258D-06	0.122024D-06	-0.946930D-07	0.114125D-06	-0.409593D-07

ESTIMATED COVARIANCE MATRIX FOR PARAMETER ESTIMATES

	21	22	23	24	25
21	0.103836D-03				
22	0.300664D-04	0.705849D-04			
23	-0.257957D-04	-0.254390D-04	0.183387D-03		
24	0.114229D-04	0.173054D-04	-0.170900D-04	0.498803D-04	
25	0.404883D-04	0.599037D-04	-0.424453D-04	0.404955D-04	0.370798D-03
26	-0.224548D-04	-0.729421D-05	0.118778D-04	-0.194171D-05	-0.704537D-05
27	-0.699874D-05	-0.142712D-04	0.785984D-05	-0.547578D-05	-0.112438D-04
28	0.412454D-05	0.819937D-05	-0.374047D-04	0.270279D-05	0.132556D-04
29	-0.992188D-06	-0.535426D-05	0.670763D-05	-0.119461D-04	-0.834401D-05
30	-0.138377D-04	-0.306422D-04	0.401943D-04	-0.209264D-04	-0.742518D-04
31	0.563779D-05	0.789566D-05	-0.113846D-04	0.480047D-05	0.115410D-04
32	0.402443D-05	-0.832093D-05	0.499219D-05	0.434356D-05	0.498783D-05
33	0.988786D-05	0.686466D-05	0.709611D-05	0.189203D-06	-0.291902D-06
34	0.644765D-05	0.707419D-05	0.109651D-05	-0.125692D-05	0.451898D-05
35	-0.805537D-04	-0.584427D-04	-0.786647D-04	0.216673D-05	-0.176980D-03
36	-0.556530D-05	-0.313236D-05	0.844755D-05	-0.410824D-05	-0.770324D-05
37	0.413338D-04	0.403664D-04	0.534958D-04	-0.676701D-06	0.939734D-04
38	-0.293378D-07	-0.366695D-04	-0.301964D-04	-0.433152D-05	-0.360064D-04
39	-0.131022D-05	-0.221834D-05	-0.182834D-05	-0.756927D-06	0.525427D-06
40	0.108758D-05	0.967477D-06	0.188729D-05	-0.725713D-06	0.233884D-05
41	-0.244949D-05	-0.217333D-05	0.490781D-06	-0.559071D-06	-0.401176D-05
42	-0.858285D-07	-0.764803D-06	-0.253503D-06	-0.921714D-07	-0.761145D-06
43	0.169990D-06	0.237320D-06	-0.227417D-06	-0.124728D-06	-0.720861D-06
44	-0.346395D-07	-0.256721D-07	-0.209770D-06	0.158909D-06	0.523373D-06

ESTIMATED COVARIANCE MATRIX FOR PARAMETER ESTIMATES

	26	27	28	29	30
26	0.861865D-04				
27	0.248503D-04	0.580166D-04			
28	-0.178087D-04	-0.213694D-04	0.162921D-03		
29	0.521831D-05	0.146554D-04	-0.144342D-04	0.436830D-04	
30	0.191937D-04	0.233653D-04	-0.260385D-04	0.209626D-04	0.157467D-03
31	-0.237022D-04	-0.393066D-04	0.537728D-04	-0.269232D-04	-0.611108D-04
32	0.621787D-06	0.780722D-05	-0.308916D-05	0.501624D-05	0.785557D-05
33	-0.441320D-07	-0.287223D-06	-0.641625D-05	0.374283D-05	0.458855D-05

34	-0.247908D-06	-0.304621D-05	-0.725185D-06	0.387225D-05	-0.325823D-05
35	0.207492D-03	0.208860D-03	0.101601D-04	0.156983D-04	0.271100D-03
36	0.217205D-05	0.416033D-05	-0.390102D-05	-0.729668D-07	0.864208D-05
37	-0.133154D-03	-0.139436D-03	0.147555D-04	-0.173267D-04	-0.171726D-03
38	0.909602D-04	0.107381D-03	-0.687038D-04	0.325074D-04	0.115575D-03
39	0.140817D-05	0.626257D-06	0.377089D-05	-0.186633D-05	-0.304162D-06
40	-0.114642D-05	-0.284971D-06	0.520199D-06	-0.160613D-06	0.494474D-08
41	0.178946D-05	-0.164651D-06	0.583448D-06	0.196462D-06	0.108867D-05
42	0.639896D-06	0.692432D-07	0.445823D-06	0.119870D-06	0.404550D-06
43	0.320450D-06	-0.149099D-06	0.484177D-06	-0.435039D-07	0.414813D-06
44	-0.439074D-07	0.890531D-07	0.346888D-06	0.128846D-06	0.177642D-06

ESTIMATED COVARIANCE MATRIX FOR PARAMETER ESTIMATES

	31	32	33	34	35
31	0.248124D-03				
32	-0.212557D-04	0.362981D-03			
33	-0.197702D-05	0.147719D-04	0.329350D-03		
34	-0.542657D-06	0.922829D-05	0.143995D-03	0.192217D-03	
35	-0.599772D-03	0.377143D-03	0.205929D-04	-0.116127D-03	0.334065D+00
36	-0.145625D-04	0.378688D-04	-0.173350D-05	0.372421D-05	0.251953D-03
37	0.414421D-03	-0.220796D-03	-0.264237D-04	0.784612D-04	-0.221879D+00
38	-0.342680D-03	0.842875D-04	0.435748D-04	-0.786876D-04	0.164737D+00
39	0.392506D-05	-0.135938D-05	0.634393D-05	0.739537D-06	-0.802797D-03
40	0.146402D-05	-0.140885D-05	-0.490695D-06	-0.777437D-06	0.857854D-04
41	-0.150488D-05	-0.146123D-05	-0.677190D-05	-0.203460D-06	-0.353276D-04
42	-0.417956D-06	0.207752D-06	-0.102293D-05	-0.818546D-06	0.180152D-04
43	-0.186949D-06	0.237209D-06	0.468082D-06	-0.434891D-06	-0.123879D-04
44	-0.582797D-07	-0.632898D-06	0.117522D-06	0.339233D-06	0.391737D-07

ESTIMATED COVARIANCE MATRIX FOR PARAMETER ESTIMATES

	36	37	38	39	40
36	0.248137D-03				
37	-0.167650D-03	0.152000D+00			
38	0.131653D-03	-0.124353D+00	0.129613D+00		
39	0.622541D-06	0.399655D-03	0.450628D-04	0.633346D-04	
40	-0.684443D-05	-0.661761D-04	0.722723D-04	-0.513206D-07	0.148034D-04
41	-0.160383D-05	0.325533D-04	-0.435000D-04	-0.258878D-06	0.695187D-07
42	-0.235336D-05	-0.871739D-05	-0.595791D-06	0.477838D-06	0.382853D-06
43	-0.221988D-05	0.137678D-04	-0.236007D-04	0.167491D-06	0.123167D-06
44	-0.258747D-06	0.929748D-06	-0.450026D-05	-0.434456D-06	-0.304374D-07

ESTIMATED COVARIANCE MATRIX FOR PARAMETER ESTIMATES

	41	42	43	44
41	0.148864D-04			
42	0.515298D-05	0.437463D-05		
43	0.212935D-05	0.278602D-05	0.326925D-05	
44	0.416584D-07	-0.203995D-07	-0.238417D-07	0.490885D-06

ESTIMATED CORRELATION MATRIX FOR PARAMETER ESTIMATES

	1	2	3	4	5
1	1.000				
2	-0.160	1.000			
3	-0.122	0.252	1.000		
4	0.161	-0.178	-0.181	1.000	
5	-0.006	-0.019	-0.027	-0.026	1.000
6	-0.005	0.002	-0.011	-0.014	0.104
7	-0.014	0.007	0.011	0.052	-0.037
8	0.022	-0.002	0.016	-0.052	-0.038
9	-0.032	0.050	0.049	-0.008	0.016
10	0.014	0.008	-0.025	-0.004	-0.046
11	-0.007	0.043	-0.015	-0.028	-0.036
12	0.004	0.019	0.004	-0.032	-0.039
13	0.019	-0.006	0.011	-0.003	-0.025
14	-0.006	-0.022	0.015	0.007	0.023
15	0.026	0.009	0.004	-0.037	0.005
16	-0.030	0.023	-0.002	-0.012	-0.004
17	-0.012	-0.037	-0.012	-0.009	-0.007
18	-0.004	-0.028	-0.041	0.038	-0.018
19	0.004	0.008	0.006	-0.021	-0.003
20	-0.003	0.009	-0.008	0.017	-0.030
21	0.020	-0.006	-0.016	0.011	-0.023
22	-0.009	0.010	-0.002	-0.023	-0.042
23	-0.039	-0.037	-0.004	0.014	0.048
24	0.011	-0.002	-0.045	0.040	-0.050
25	-0.014	0.006	0.063	0.006	-0.039
26	0.006	-0.013	-0.021	0.011	0.015
27	0.051	0.027	-0.029	-0.064	0.015
28	-0.032	-0.017	-0.007	0.030	0.012
29	0.049	0.019	0.029	0.010	-0.031
30	0.008	0.003	0.002	0.013	-0.019

31	-0.038	-0.023	0.017	0.016	0.020
32	-0.022	0.026	0.064	-0.056	-0.170
33	-0.097	0.057	0.055	-0.078	0.014
34	-0.145	0.102	0.094	-0.142	-0.006
35	0.024	-0.038	-0.021	0.001	0.055
36	0.009	0.037	0.031	-0.010	-0.048
37	-0.020	0.040	0.023	0.002	-0.053
38	0.008	-0.040	-0.026	-0.005	0.041
39	-0.014	-0.001	0.012	-0.012	-0.018
40	0.043	-0.006	0.004	-0.021	0.029
41	-0.009	0.027	-0.022	0.000	0.014
42	-0.002	0.002	-0.012	0.021	-0.017
43	0.007	-0.032	-0.024	0.039	-0.005
44	0.003	0.032	0.041	-0.033	0.075

ESTIMATED CORRELATION MATRIX FOR PARAMETER ESTIMATES

	6	7	8	9	10
6	1.000				
7	0.059	1.000			
8	0.034	-0.143	1.000		
9	-0.130	0.090	0.142	1.000	
10	0.038	-0.034	0.039	-0.038	1.000
11	-0.002	0.024	-0.036	-0.033	0.482
12	-0.036	-0.007	-0.028	0.009	0.135
13	0.000	-0.010	-0.022	-0.031	0.015
14	0.002	0.007	-0.012	0.020	-0.252
15	0.016	-0.049	0.009	-0.005	-0.066
16	-0.007	0.014	0.035	0.001	0.026
17	0.002	-0.021	-0.013	-0.012	0.183
18	-0.021	0.008	-0.002	0.009	0.070
19	0.034	0.006	-0.005	0.010	-0.001
20	-0.005	0.001	0.014	0.053	0.029
21	-0.037	0.007	-0.012	0.047	0.205
22	-0.007	-0.003	0.008	0.033	0.125
23	-0.015	-0.045	-0.032	0.021	-0.079
24	0.003	-0.039	-0.008	0.072	0.046
25	-0.034	0.006	0.065	0.042	-0.004
26	0.035	0.039	0.022	0.015	-0.120
27	0.032	0.019	-0.016	-0.031	-0.043
28	-0.044	0.022	0.004	0.017	0.009
29	-0.038	0.013	-0.063	-0.061	0.008
30	-0.013	-0.040	-0.043	-0.052	-0.046

31	-0.007	-0.015	0.021	0.008	0.015
32	0.131	-0.053	-0.133	0.050	0.026
33	0.008	0.026	0.007	0.029	0.027
34	0.005	-0.010	0.000	-0.009	-0.005
35	0.018	-0.074	0.007	-0.042	-0.037
36	-0.022	0.005	-0.044	0.021	0.005
37	-0.004	0.071	-0.017	0.033	0.033
38	-0.032	-0.051	0.040	-0.003	-0.018
39	0.017	0.003	0.026	0.011	0.015
40	-0.008	-0.061	-0.005	-0.036	-0.002
41	0.015	-0.015	-0.037	-0.029	-0.061
42	-0.010	0.004	-0.032	0.009	-0.012
43	-0.031	0.021	-0.020	0.002	-0.030
44	0.029	0.021	0.011	0.046	-0.023

ESTIMATED CORRELATION MATRIX FOR PARAMETER ESTIMATES

	11	12	13	14	15
11	1.000				
12	0.450	1.000			
13	-0.006	-0.012	1.000		
14	-0.271	-0.098	0.008	1.000	
15	-0.175	-0.249	-0.009	0.370	1.000
16	0.043	0.046	0.015	-0.229	-0.237
17	0.258	0.114	0.008	-0.199	-0.033
18	0.182	0.340	-0.001	-0.062	-0.152
19	0.000	-0.048	0.065	0.105	0.238
20	0.026	0.076	0.019	-0.040	-0.042
21	0.252	0.092	-0.011	-0.185	-0.059
22	0.228	0.286	-0.011	-0.090	-0.154
23	-0.088	-0.080	0.003	0.198	0.233
24	0.068	0.084	-0.025	-0.056	-0.020
25	0.014	0.029	-0.005	-0.033	-0.009
26	-0.226	-0.091	0.022	0.195	0.085
27	-0.182	-0.311	0.016	0.079	0.214
28	0.022	0.014	0.004	-0.151	-0.251
29	-0.056	-0.094	0.048	0.028	0.061
30	-0.055	-0.079	-0.019	0.056	0.037
31	0.037	0.067	-0.012	-0.025	-0.081
32	-0.026	-0.040	-0.007	-0.023	0.023
33	0.021	0.008	-0.042	-0.047	-0.003
34	0.026	0.044	0.017	-0.024	-0.024
35	0.020	0.000	-0.015	-0.027	0.013

36	0.012	-0.017	0.013	0.003	0.005
37	-0.015	-0.001	0.014	0.026	-0.014
38	0.001	0.003	-0.010	-0.022	0.016
39	0.018	0.001	-0.041	-0.006	-0.030
40	-0.019	0.000	-0.006	-0.037	0.024
41	-0.022	0.007	0.018	0.049	0.056
42	-0.015	0.022	-0.013	-0.020	0.003
43	-0.020	0.027	-0.034	-0.035	-0.039
44	-0.007	-0.014	-0.008	-0.026	0.018

ESTIMATED CORRELATION MATRIX FOR PARAMETER ESTIMATES

	16	17	18	19	20
16	1.000				
17	0.033	1.000			
18	0.086	0.356	1.000		
19	-0.198	-0.118	-0.241	1.000	
20	0.057	0.151	0.373	-0.241	1.000
21	0.015	0.226	0.070	-0.027	0.007
22	0.010	0.137	0.270	-0.086	0.087
23	-0.180	-0.070	-0.067	0.254	-0.070
24	0.028	0.174	0.286	-0.172	0.318
25	0.002	0.036	0.045	-0.047	0.025
26	-0.056	-0.208	-0.065	0.017	0.024
27	-0.064	-0.107	-0.234	0.065	-0.055
28	0.233	0.051	0.117	-0.197	0.077
29	-0.085	-0.137	-0.306	0.223	-0.277
30	-0.052	-0.074	-0.130	0.097	-0.060
31	0.038	0.069	0.078	-0.070	0.021
32	0.027	0.011	0.017	0.013	-0.017
33	-0.018	0.004	-0.011	-0.006	-0.019
34	-0.054	-0.014	0.008	-0.002	-0.031
35	-0.011	0.014	-0.003	0.010	0.011
36	-0.027	-0.006	0.003	-0.005	0.002
37	0.012	-0.015	0.000	-0.007	-0.019
38	-0.013	0.015	0.006	-0.001	0.036
39	0.032	-0.006	0.013	-0.048	0.020
40	0.000	0.003	-0.032	0.049	-0.034
41	-0.036	-0.002	0.030	0.012	0.014
42	-0.032	0.003	-0.006	0.030	-0.025
43	-0.024	-0.013	-0.008	0.030	-0.059
44	-0.039	0.033	-0.030	0.022	-0.011

ESTIMATED CORRELATION MATRIX FOR PARAMETER ESTIMATES

	21	22	23	24	25
21	1.000				
22	0.351	1.000			
23	-0.187	-0.224	1.000		
24	0.159	0.292	-0.179	1.000	
25	0.206	0.370	-0.163	0.298	1.000
26	-0.237	-0.094	0.094	-0.030	-0.039
27	-0.090	-0.223	0.076	-0.102	-0.077
28	0.032	0.076	-0.216	0.030	0.054
29	-0.015	-0.096	0.075	-0.256	-0.066
30	-0.108	-0.291	0.237	-0.236	-0.307
31	0.035	0.060	-0.053	0.043	0.038
32	0.021	-0.052	0.019	0.032	0.014
33	0.053	0.045	0.029	0.001	-0.001
34	0.046	0.061	0.006	-0.013	0.017
35	-0.014	-0.012	-0.010	0.001	-0.016
36	-0.035	-0.024	0.040	-0.037	-0.025
37	0.010	0.012	0.010	0.000	0.013
38	0.000	-0.012	-0.006	-0.002	-0.005
39	-0.016	-0.033	-0.017	-0.013	0.003
40	0.028	0.030	0.036	-0.027	0.032
41	-0.062	-0.067	0.009	-0.021	-0.054
42	-0.004	-0.044	-0.009	-0.006	-0.019
43	0.009	0.016	-0.009	-0.010	-0.021
44	-0.005	-0.004	-0.022	0.032	0.039

ESTIMATED CORRELATION MATRIX FOR PARAMETER ESTIMATES

	26	27	28	29	30
26	1.000				
27	0.351	1.000			
28	-0.150	-0.220	1.000		
29	0.085	0.291	-0.171	1.000	
30	0.165	0.244	-0.163	0.253	1.000
31	-0.162	-0.328	0.267	-0.259	-0.309
32	0.004	0.054	-0.013	0.040	0.033
33	0.000	-0.002	-0.028	0.031	0.020
34	-0.002	-0.029	-0.004	0.042	-0.019
35	0.039	0.047	0.001	0.004	0.037
36	0.015	0.035	-0.019	-0.001	0.044

37	-0.037	-0.047	0.003	-0.007	-0.035
38	0.027	0.039	-0.015	0.014	0.026
39	0.019	0.010	0.037	-0.035	-0.003
40	-0.032	-0.010	0.011	-0.006	0.000
41	0.050	-0.006	0.012	0.008	0.022
42	0.033	0.004	0.017	0.009	0.015
43	0.019	-0.011	0.021	-0.004	0.018
44	-0.007	0.017	0.039	0.028	0.020

ESTIMATED CORRELATION MATRIX FOR PARAMETER ESTIMATES

	31	32	33	34	35
31	1.000				
32	-0.071	1.000			
33	-0.007	0.043	1.000		
34	-0.002	0.035	0.572	1.000	
35	-0.066	0.034	0.002	-0.014	1.000
36	-0.059	0.126	-0.006	0.017	0.028
37	0.067	-0.030	-0.004	0.015	-0.985
38	-0.060	0.012	0.007	-0.016	0.792
39	0.031	-0.009	0.044	0.007	-0.175
40	0.024	-0.019	-0.007	-0.015	0.039
41	-0.025	-0.020	-0.097	-0.004	-0.016
42	-0.013	0.005	-0.027	-0.028	0.015
43	-0.007	0.007	0.014	-0.017	-0.012
44	-0.005	-0.047	0.009	0.035	0.000

ESTIMATED CORRELATION MATRIX FOR PARAMETER ESTIMATES

	36	37	38	39	40
36	1.000				
37	-0.027	1.000			
38	0.023	-0.886	1.000		
39	0.005	0.129	0.016	1.000	
40	-0.113	-0.044	0.052	-0.002	1.000
41	-0.026	0.022	-0.031	-0.008	0.005
42	-0.071	-0.011	-0.001	0.029	0.048
43	-0.078	0.020	-0.036	0.012	0.018
44	-0.023	0.003	-0.018	-0.078	-0.011

ESTIMATED CORRELATION MATRIX FOR PARAMETER ESTIMATES

	41	42	43	44
41	1.000			
42	0.639	1.000		
43	0.305	0.737	1.000	
44	0.015	-0.014	-0.019	1.000

TECHNICAL 8 OUTPUT

TECHNICAL 8 OUTPUT FOR BAYES ESTIMATION

CHAIN	BSEED
1	0
2	285380
3	253358

ITERATION	POTENTIAL SCALE REDUCTION	PARAMETER WITH HIGHEST PSR
100	1.009	33
200	1.006	39
300	1.004	35
400	1.002	15
500	1.001	1
600	1.002	1
700	1.002	4
800	1.003	4
900	1.003	4
1000	1.002	4

These are the models using categories from the latent profile analysis instead of the dimension derived from principal component analysis

This is deterioration in physical health

SUMMARY OF DATA

Number of clusters 58

Size (s)	Cluster ID with Size s
7	22
16	166
23	80 71 205
25	74
27	192
31	65
32	106
35	228
37	170 72
39	50 75 181
40	182
42	165
44	114
45	173
46	107
47	169 30
48	226 81
49	36
50	116 108 40
56	162 84
57	61
59	206 93 83
60	92
61	69
63	6
64	152
65	145
66	4
68	68
69	154
72	174
78	98
84	178 2

95	156 202
96	144
108	142
131	122
172	24
175	209
224	204
269	125
364	217
738	110
860	100

COVARIANCE COVERAGE OF DATA

Minimum covariance coverage value 0.100

Number of missing data patterns 8

PROPORTION OF DATA PRESENT

Covariance Coverage

	PHYCAT16	CLU2	CLU3	PHY15	KES15
PHYCAT16	1.000				
CLU2	1.000	1.000			
CLU3	1.000	1.000	1.000		
PHY15	0.947	0.947	0.947	0.947	
KES15	0.922	0.922	0.922	0.922	0.922
CHOICE15	0.915	0.915	0.915	0.915	0.915
BULLY15	0.915	0.915	0.915	0.915	0.915
PRESS15	0.915	0.915	0.915	0.915	0.915
DUTIES15	0.915	0.915	0.915	0.915	0.915

Covariance Coverage

	CHOICE15	BULLY15	PRESS15	DUTIES15
CHOICE15	0.915			
BULLY15	0.915	0.915		
PRESS15	0.915	0.914	0.915	

DUTIES15	0.915	0.915	0.915	0.915

UNIVARIATE PROPORTIONS AND COUNTS FOR CATEGORICAL VARIABLES

PHYCAT16		
Category 1	0.881	4884.000
Category 2	0.119	662.000

UNIVARIATE SAMPLE STATISTICS

UNIVARIATE HIGHER-ORDER MOMENT DESCRIPTIVE STATISTICS

Variable/ Sample Size	Mean/ Variance	Skewness/ Kurtosis	Minimum/ Maximum	% with Min/Max	Percentiles 20%/60%	40%/80%	Median
CLU2	0.310	0.820	0.000	68.97%	0.000	0.000	0.000
58.000	0.214	-1.328	1.000	31.03%	0.000	1.000	
CLU3	0.448	0.208	0.000	55.17%	0.000	0.000	0.000
58.000	0.247	-1.957	1.000	44.83%	1.000	1.000	
PHY15	0.000	-0.546	-0.632	36.83%	-0.632	0.368	0.368
5254.000	0.233	-1.702	0.368	63.17%	0.368	0.368	
KES15	0.000	1.717	-0.511	21.65%	-0.511	-0.178	-0.178
5114.000	0.289	3.835	3.489	0.04%	-0.011	0.322	
CHOICE15	0.000	-0.402	-2.225	7.41%	-1.225	-0.225	-0.225
5077.000	1.081	-0.336	1.775	8.49%	0.775	0.775	
BULLY15	0.000	3.272	-0.170	87.25%	-0.170	-0.170	-0.170
5073.000	0.239	11.772	3.830	0.08%	-0.170	-0.170	
PRESS15	0.000	0.252	-1.462	19.10%	-0.462	-0.462	-0.462
5074.000	1.001	-0.371	2.538	2.76%	0.538	0.538	
DUTIES15	0.000	-1.246	-3.120	2.40%	-0.120	-0.120	-0.120
5077.000	0.861	1.673	0.880	38.74%	-0.120	0.880	

THE MODEL ESTIMATION TERMINATED NORMALLY

USE THE FBITERATIONS OPTION TO INCREASE THE NUMBER OF ITERATIONS BY A FACTOR
OF AT LEAST TWO TO CHECK CONVERGENCE AND THAT THE PSR VALUE DOES NOT INCREASE.

MODEL FIT INFORMATION

Number of Free Parameters 42

Bayesian Posterior Predictive Checking using Chi-Square

95% Confidence Interval for the Difference Between
the Observed and the Replicated Chi-Square Values

-26.525 22.393

Posterior Predictive P-Value 0.529

MODEL RESULTS

	Estimate	Posterior S.D.	One-Tailed P-Value	95% C.I. Lower 2.5%	Upper 2.5%	Significance
Within Level						
PHYCAT16 ON						
PHY15	0.971	0.068	0.000	0.839	1.106	*
CHOICE15	-0.067	0.025	0.003	-0.116	-0.018	*
BULLY15	0.028	0.049	0.281	-0.070	0.119	
DUTIES15	-0.029	0.027	0.133	-0.084	0.023	
PRESS15	-0.032	0.027	0.107	-0.082	0.022	
KES15	0.224	0.044	0.000	0.139	0.311	*
KES15 WITH						
PHY15	0.051	0.004	0.000	0.044	0.059	*
CHOICE15	-0.089	0.008	0.000	-0.105	-0.074	*
BULLY15	0.057	0.004	0.000	0.050	0.065	*
PRESS15	0.100	0.008	0.000	0.084	0.115	*
DUTIES15	-0.106	0.007	0.000	-0.121	-0.093	*
PHY15 WITH						
CHOICE15	-0.036	0.007	0.000	-0.050	-0.023	*
BULLY15	0.012	0.003	0.001	0.005	0.018	*
PRESS15	0.038	0.007	0.000	0.026	0.052	*
DUTIES15	-0.036	0.006	0.000	-0.048	-0.024	*
CHOICE15 WITH						

BULLY15	-0.058	0.007	0.000	-0.072	-0.044	*
PRESS15	-0.086	0.015	0.000	-0.118	-0.060	*
DUTIES15	0.173	0.013	0.000	0.148	0.201	*
BULLY15 WITH						
PRESS15	0.104	0.007	0.000	0.092	0.118	*
DUTIES15	-0.077	0.006	0.000	-0.090	-0.065	*
PRESS15 WITH						
DUTIES15	-0.185	0.013	0.000	-0.212	-0.160	*
Means						
PHY15	0.002	0.007	0.370	-0.011	0.015	
KES15	0.001	0.008	0.446	-0.014	0.015	
CHOICE15	-0.002	0.015	0.449	-0.029	0.028	
BULLY15	0.001	0.007	0.475	-0.013	0.015	
PRESS15	0.001	0.014	0.481	-0.026	0.029	
DUTIES15	-0.001	0.013	0.471	-0.026	0.025	
Variances						
PHY15	0.233	0.005	0.000	0.225	0.243	*
KES15	0.289	0.006	0.000	0.279	0.301	*
CHOICE15	1.081	0.021	0.000	1.041	1.122	*
BULLY15	0.239	0.005	0.000	0.230	0.248	*
PRESS15	1.000	0.020	0.000	0.963	1.040	*
DUTIES15	0.861	0.017	0.000	0.829	0.895	*

Between Level

PHYCAT16 ON						
CLU2	-0.206	0.095	0.013	-0.398	-0.025	*
CLU3	-0.021	0.088	0.411	-0.186	0.155	
CLU2 WITH						
CLU3	-0.137	0.038	0.000	-0.228	-0.076	*
Means						
CLU2	0.309	0.063	0.000	0.180	0.439	*
CLU3	0.447	0.068	0.000	0.314	0.585	*
Thresholds						
PHYCAT16$1	1.259	0.076	0.000	1.112	1.406	*
Variances						

CLU2	0.230	0.044	0.000	0.166	0.330	*
CLU3	0.262	0.050	0.000	0.185	0.376	*

Residual Variances						
PHYCAT16	0.015	0.012	0.000	0.001	0.048	*

STANDARDIZED MODEL RESULTS

STDYX Standardization

	Estimate	Posterior S.D.	One-Tailed P-Value	95% C.I. Lower 2.5%	Upper 2.5%	Significance
**Within Level**						
PHYCAT16 ON						
PHY15	0.416	0.024	0.000	0.367	0.462	*
CHOICE15	-0.062	0.023	0.003	-0.106	-0.017	*
BULLY15	0.012	0.021	0.281	-0.030	0.051	
DUTIES15	-0.024	0.022	0.133	-0.069	0.019	
PRESS15	-0.028	0.024	0.107	-0.073	0.019	
KES15	0.107	0.021	0.000	0.067	0.149	*
KES15 WITH						
PHY15	0.198	0.013	0.000	0.170	0.223	*
CHOICE15	-0.159	0.014	0.000	-0.187	-0.132	*
BULLY15	0.218	0.013	0.000	0.193	0.244	*
PRESS15	0.186	0.014	0.000	0.157	0.212	*
DUTIES15	-0.213	0.013	0.000	-0.240	-0.187	*
PHY15 WITH						
CHOICE15	-0.072	0.014	0.000	-0.099	-0.045	*
BULLY15	0.049	0.014	0.001	0.021	0.076	*
PRESS15	0.079	0.014	0.000	0.053	0.107	*
DUTIES15	-0.081	0.014	0.000	-0.107	-0.054	*
CHOICE15 WITH						
BULLY15	-0.114	0.014	0.000	-0.141	-0.088	*
PRESS15	-0.083	0.014	0.000	-0.113	-0.058	*
DUTIES15	0.179	0.013	0.000	0.154	0.206	*
BULLY15 WITH						
PRESS15	0.214	0.013	0.000	0.189	0.239	*

```
    DUTIES15          -0.171       0.013      0.000      -0.197      -0.145      *

 PRESS15  WITH
    DUTIES15          -0.200       0.013      0.000      -0.227      -0.174      *

 Means
    PHY15              0.004       0.014      0.370      -0.024       0.031
    KES15              0.002       0.014      0.446      -0.025       0.028
    CHOICE15          -0.002       0.014      0.449      -0.028       0.026
    BULLY15            0.001       0.014      0.475      -0.026       0.030
    PRESS15            0.001       0.014      0.481      -0.026       0.029
    DUTIES15          -0.001       0.014      0.471      -0.028       0.026

 Variances
    PHY15              1.000       0.000      0.000       1.000       1.000
    KES15              1.000       0.000      0.000       1.000       1.000
    CHOICE15           1.000       0.000      0.000       1.000       1.000
    BULLY15            1.000       0.000      0.000       1.000       1.000
    PRESS15            1.000       0.000      0.000       1.000       1.000
    DUTIES15           1.000       0.000      0.000       1.000       1.000

Between Level

 PHYCAT16   ON
    CLU2              -0.630       0.243      0.013      -1.047      -0.079      *
    CLU3              -0.072       0.280      0.411      -0.578       0.528

 CLU2     WITH
    CLU3              -0.562       0.089      0.000      -0.711      -0.365      *

 Means
    CLU2               0.646       0.143      0.000       0.365       0.928      *
    CLU3               0.878       0.154      0.000       0.575       1.192      *

 Thresholds
    PHYCAT16$1         7.840       2.941      0.000       4.642      15.956      *

 Variances
    CLU2               1.000       0.000      0.000       1.000       1.000
    CLU3               1.000       0.000      0.000       1.000       1.000

 Residual Variances
    PHYCAT16           0.587       0.199      0.000       0.169       0.927      *
```

```
R-SQUARE

Within Level

                                 Posterior  One-Tailed         95% C.I.
    Variable        Estimate       S.D.      P-Value   Lower 2.5%  Upper 2.5%

    PHYCAT16           0.215       0.021      0.000       0.177       0.258

Between Level

                                 Posterior  One-Tailed         95% C.I.
    Variable        Estimate       S.D.      P-Value   Lower 2.5%  Upper 2.5%

    PHYCAT16           0.413       0.199      0.000       0.073       0.831


TECHNICAL 1 OUTPUT


     PARAMETER SPECIFICATION FOR WITHIN


           TAU
              PHYCAT16
              ________
                    0


           NU
              PHYCAT16      PHY15         KES15         CHOICE15      BULLY15
              ________      ________      ________      ________      ________
                    0             0             0             0             0


           NU
              PRESS15       DUTIES15
              ________      ________
                    0             0


           LAMBDA
              PHYCAT16      PHY15         KES15         CHOICE15      BULLY15
```

PHYCAT16	0	0	0	0	0
PHY15	0	0	0	0	0
KES15	0	0	0	0	0
CHOICE15	0	0	0	0	0
BULLY15	0	0	0	0	0
PRESS15	0	0	0	0	0
DUTIES15	0	0	0	0	0

LAMBDA

	PRESS15	DUTIES15
PHYCAT16	0	0
PHY15	0	0
KES15	0	0
CHOICE15	0	0
BULLY15	0	0
PRESS15	0	0
DUTIES15	0	0

THETA

	PHYCAT16	PHY15	KES15	CHOICE15	BULLY15
PHYCAT16	0				
PHY15	0	0			
KES15	0	0	0		
CHOICE15	0	0	0	0	
BULLY15	0	0	0	0	0
PRESS15	0	0	0	0	0
DUTIES15	0	0	0	0	0

THETA

	PRESS15	DUTIES15
PRESS15	0	
DUTIES15	0	0

ALPHA

PHYCAT16	PHY15	KES15	CHOICE15	BULLY15

0	1	2	3	4

ALPHA

PRESS15	DUTIES15
5	6

BETA

	PHYCAT16	PHY15	KES15	CHOICE15	BULLY15
PHYCAT16	0	7	8	9	10
PHY15	0	0	0	0	0
KES15	0	0	0	0	0
CHOICE15	0	0	0	0	0
BULLY15	0	0	0	0	0
PRESS15	0	0	0	0	0
DUTIES15	0	0	0	0	0

BETA

	PRESS15	DUTIES15
PHYCAT16	11	12
PHY15	0	0
KES15	0	0
CHOICE15	0	0
BULLY15	0	0
PRESS15	0	0
DUTIES15	0	0

PSI

	PHYCAT16	PHY15	KES15	CHOICE15	BULLY15
PHYCAT16	0				
PHY15	0	13			
KES15	0	14	15		
CHOICE15	0	16	17	18	
BULLY15	0	19	20	21	22
PRESS15	0	23	24	25	26
DUTIES15	0	28	29	30	31

PSI

	PRESS15	DUTIES15
PRESS15	27	
DUTIES15	32	33

PARAMETER SPECIFICATION FOR BETWEEN

TAU

PHYCAT16
42

NU

PHYCAT16	CLU2	CLU3
0	0	0

LAMBDA

	PHYCAT16	CLU2	CLU3
PHYCAT16	0	0	0
CLU2	0	0	0
CLU3	0	0	0

THETA

	PHYCAT16	CLU2	CLU3
PHYCAT16	0		
CLU2	0	0	
CLU3	0	0	0

ALPHA

PHYCAT16	CLU2	CLU3
0	34	35

BETA

	PHYCAT16	CLU2	CLU3
PHYCAT16	0	36	37
CLU2	0	0	0
CLU3	0	0	0

PSI

	PHYCAT16	CLU2	CLU3
PHYCAT16	38		
CLU2	0	39	
CLU3	0	40	41

STARTING VALUES FOR WITHIN

TAU

PHYCAT16
0.000

NU

PHYCAT16	PHY15	KES15	CHOICE15	BULLY15
0.000	0.000	0.000	0.000	0.000

NU

PRESS15	DUTIES15
0.000	0.000

LAMBDA

	PHYCAT16	PHY15	KES15	CHOICE15	BULLY15
PHYCAT16	1.000	0.000	0.000	0.000	0.000
PHY15	0.000	1.000	0.000	0.000	0.000
KES15	0.000	0.000	1.000	0.000	0.000

CHOICE15	0.000	0.000	0.000	1.000	0.000
BULLY15	0.000	0.000	0.000	0.000	1.000
PRESS15	0.000	0.000	0.000	0.000	0.000
DUTIES15	0.000	0.000	0.000	0.000	0.000

LAMBDA

	PRESS15	DUTIES15
PHYCAT16	0.000	0.000
PHY15	0.000	0.000
KES15	0.000	0.000
CHOICE15	0.000	0.000
BULLY15	0.000	0.000
PRESS15	1.000	0.000
DUTIES15	0.000	1.000

THETA

	PHYCAT16	PHY15	KES15	CHOICE15	BULLY15
PHYCAT16	0.000				
PHY15	0.000	0.000			
KES15	0.000	0.000	0.000		
CHOICE15	0.000	0.000	0.000	0.000	
BULLY15	0.000	0.000	0.000	0.000	0.000
PRESS15	0.000	0.000	0.000	0.000	0.000
DUTIES15	0.000	0.000	0.000	0.000	0.000

THETA

	PRESS15	DUTIES15
PRESS15	0.000	
DUTIES15	0.000	0.000

ALPHA

PHYCAT16	PHY15	KES15	CHOICE15	BULLY15
0.000	0.000	0.000	0.000	0.000

ALPHA

	PRESS15	DUTIES15
	0.000	0.000

BETA

	PHYCAT16	PHY15	KES15	CHOICE15	BULLY15
PHYCAT16	0.000	0.000	0.000	0.000	0.000
PHY15	0.000	0.000	0.000	0.000	0.000
KES15	0.000	0.000	0.000	0.000	0.000
CHOICE15	0.000	0.000	0.000	0.000	0.000
BULLY15	0.000	0.000	0.000	0.000	0.000
PRESS15	0.000	0.000	0.000	0.000	0.000
DUTIES15	0.000	0.000	0.000	0.000	0.000

BETA

	PRESS15	DUTIES15
PHYCAT16	0.000	0.000
PHY15	0.000	0.000
KES15	0.000	0.000
CHOICE15	0.000	0.000
BULLY15	0.000	0.000
PRESS15	0.000	0.000
DUTIES15	0.000	0.000

PSI

	PHYCAT16	PHY15	KES15	CHOICE15	BULLY15
PHYCAT16	1.000				
PHY15	0.000	0.116			
KES15	0.000	0.000	0.145		
CHOICE15	0.000	0.000	0.000	0.541	
BULLY15	0.000	0.000	0.000	0.000	0.119
PRESS15	0.000	0.000	0.000	0.000	0.000
DUTIES15	0.000	0.000	0.000	0.000	0.000

PSI

PRESS15	DUTIES15

```
 PRESS15        0.501
 DUTIES15       0.000         0.430


     STARTING VALUES FOR BETWEEN


           TAU
              PHYCAT16
              ________
                1.110


           NU
              PHYCAT16      CLU2          CLU3
              ________      ________      ________
                0.000         0.000         0.000


           LAMBDA
              PHYCAT16      CLU2          CLU3
              ________      ________      ________
 PHYCAT16       1.000         0.000         0.000
 CLU2           0.000         1.000         0.000
 CLU3           0.000         0.000         1.000


           THETA
              PHYCAT16      CLU2          CLU3
              ________      ________      ________
 PHYCAT16       0.000
 CLU2           0.000         0.000
 CLU3           0.000         0.000         0.000


           ALPHA
              PHYCAT16      CLU2          CLU3
              ________      ________      ________
                0.000         0.406         0.471


           BETA
              PHYCAT16      CLU2          CLU3
              ________      ________      ________
```

PHYCAT16	0.000	0.000	0.000
CLU2	0.000	0.000	0.000
CLU3	0.000	0.000	0.000

PSI

	PHYCAT16	CLU2	CLU3
PHYCAT16	1.000		
CLU2	0.000	0.121	
CLU3	0.000	0.000	0.125

PRIORS FOR ALL PARAMETERS	PRIOR MEAN	PRIOR VARIANCE	PRIOR STD. DEV.
Parameter 1~N(0.000,infinity)	0.0000	infinity	infinity
Parameter 2~N(0.000,infinity)	0.0000	infinity	infinity
Parameter 3~N(0.000,infinity)	0.0000	infinity	infinity
Parameter 4~N(0.000,infinity)	0.0000	infinity	infinity
Parameter 5~N(0.000,infinity)	0.0000	infinity	infinity
Parameter 6~N(0.000,infinity)	0.0000	infinity	infinity
Parameter 7~N(0.000,5.000)	0.0000	5.0000	2.2361
Parameter 8~N(0.000,5.000)	0.0000	5.0000	2.2361
Parameter 9~N(0.000,5.000)	0.0000	5.0000	2.2361
Parameter 10~N(0.000,5.000)	0.0000	5.0000	2.2361
Parameter 11~N(0.000,5.000)	0.0000	5.0000	2.2361
Parameter 12~N(0.000,5.000)	0.0000	5.0000	2.2361
Parameter 13~IW(1.000,7)	infinity	infinity	infinity
Parameter 14~IW(0.000,7)	infinity	infinity	infinity
Parameter 15~IW(1.000,7)	infinity	infinity	infinity
Parameter 16~IW(0.000,7)	infinity	infinity	infinity
Parameter 17~IW(0.000,7)	infinity	infinity	infinity
Parameter 18~IW(1.000,7)	infinity	infinity	infinity
Parameter 19~IW(0.000,7)	infinity	infinity	infinity
Parameter 20~IW(0.000,7)	infinity	infinity	infinity
Parameter 21~IW(0.000,7)	infinity	infinity	infinity
Parameter 22~IW(1.000,7)	infinity	infinity	infinity
Parameter 23~IW(0.000,7)	infinity	infinity	infinity
Parameter 24~IW(0.000,7)	infinity	infinity	infinity
Parameter 25~IW(0.000,7)	infinity	infinity	infinity
Parameter 26~IW(0.000,7)	infinity	infinity	infinity
Parameter 27~IW(1.000,7)	infinity	infinity	infinity
Parameter 28~IW(0.000,7)	infinity	infinity	infinity

Parameter 29~IW(0.000,7)	infinity	infinity	infinity
Parameter 30~IW(0.000,7)	infinity	infinity	infinity
Parameter 31~IW(0.000,7)	infinity	infinity	infinity
Parameter 32~IW(0.000,7)	infinity	infinity	infinity
Parameter 33~IW(1.000,7)	infinity	infinity	infinity
Parameter 34~N(0.000,infinity)	0.0000	infinity	infinity
Parameter 35~N(0.000,infinity)	0.0000	infinity	infinity
Parameter 36~N(0.000,5.000)	0.0000	5.0000	2.2361
Parameter 37~N(0.000,5.000)	0.0000	5.0000	2.2361
Parameter 38~IG(-1.000,0.000)	infinity	infinity	infinity
Parameter 39~IW(1.000,3)	infinity	infinity	infinity
Parameter 40~IW(0.000,3)	infinity	infinity	infinity
Parameter 41~IW(1.000,3)	infinity	infinity	infinity
Parameter 42~N(0.000,5.000)	0.0000	5.0000	2.2361

TECHNICAL 3 OUTPUT

ESTIMATED COVARIANCE MATRIX FOR PARAMETER ESTIMATES

	1	2	3	4	5
1	0.448757D-04				
2	0.119472D-04	0.581402D-04			
3	-0.839971D-05	-0.194671D-04	0.221296D-03		
4	0.414070D-05	0.135792D-04	-0.120735D-04	0.493765D-04	
5	0.584452D-05	0.187815D-04	-0.187500D-04	0.223986D-04	0.195630D-03
6	-0.946674D-05	-0.167614D-04	0.353855D-04	-0.142923D-04	-0.310581D-04
7	-0.118671D-04	-0.355603D-05	-0.189452D-04	-0.100782D-04	-0.408880D-04
8	-0.375134D-05	-0.622916D-05	0.221375D-04	0.235138D-05	-0.877889D-05
9	-0.959530D-05	-0.254040D-05	0.176568D-04	-0.119344D-05	0.202089D-05
10	0.102786D-04	-0.572257D-05	-0.109233D-04	0.102000D-04	-0.231774D-04
11	0.907085D-05	0.586503D-05	0.102546D-04	-0.247166D-05	0.476186D-05
12	0.435816D-05	0.402073D-05	-0.156487D-04	0.508802D-05	0.187976D-05
13	-0.408432D-06	0.661011D-06	0.207347D-05	-0.109538D-05	-0.879992D-06
14	-0.575373D-06	0.105647D-05	0.127843D-05	-0.680810D-06	-0.470715D-06
15	-0.190219D-05	0.128419D-05	0.167714D-06	0.193245D-06	0.840695D-06
16	-0.363094D-06	-0.135399D-07	0.112514D-05	0.491739D-06	0.176442D-05
17	0.211867D-05	-0.980027D-07	-0.210117D-05	0.275883D-06	0.212959D-05
18	0.519158D-06	0.455528D-05	0.315691D-05	0.252053D-05	-0.253887D-05
19	0.377450D-06	-0.216309D-06	-0.113368D-05	-0.264684D-06	0.137067D-06
20	-0.316140D-06	-0.144363D-06	-0.581434D-06	0.609327D-07	0.111075D-06
21	0.194970D-06	0.957168D-06	0.378496D-07	-0.164117D-07	-0.345621D-06
22	-0.820405D-07	-0.690389D-06	0.130108D-05	-0.549650D-06	0.195094D-05

23	-0.105695D-05	-0.345476D-06	-0.379689D-05	-0.161210D-05	-0.960127D-08
24	-0.322183D-06	0.213954D-05	-0.137062D-05	0.127330D-05	-0.687784D-06
25	0.169264D-05	-0.337500D-06	-0.458598D-05	-0.115375D-05	-0.479702D-05
26	-0.117155D-05	-0.181743D-05	0.137332D-05	0.454739D-06	0.361313D-06
27	-0.366938D-05	-0.546629D-05	-0.406505D-05	0.625914D-06	-0.414648D-05
28	-0.108520D-05	0.310582D-06	0.794066D-06	0.477404D-06	-0.435901D-06
29	0.198922D-05	0.501736D-06	0.205976D-05	-0.511461D-06	0.852036D-06
30	-0.129947D-05	0.636171D-06	-0.278587D-05	0.580901D-06	-0.405170D-05
31	0.152877D-05	0.181483D-05	0.796202D-06	-0.342100D-06	0.171144D-05
32	0.678236D-06	0.169586D-06	0.403751D-05	-0.374840D-05	-0.315889D-05
33	-0.737514D-06	-0.280340D-06	0.120023D-05	0.717735D-06	0.380422D-05
34	-0.638042D-05	-0.923949D-05	-0.362582D-04	-0.303406D-05	-0.435924D-05
35	0.335519D-05	0.612237D-05	0.476876D-04	0.223127D-05	0.126270D-04
36	-0.300743D-05	0.353858D-05	0.265273D-04	-0.674983D-07	0.147544D-04
37	-0.963287D-05	0.308843D-05	0.331848D-04	-0.902855D-05	-0.633070D-05
38	-0.420672D-06	-0.340746D-05	0.926320D-06	0.853088D-07	-0.120173D-05
39	-0.256769D-05	0.890618D-05	0.252041D-04	0.107937D-04	-0.224540D-04
40	-0.122227D-05	-0.135429D-04	-0.366269D-04	-0.930982D-05	0.266412D-04
41	0.550015D-05	0.255855D-04	0.406212D-04	0.950907D-05	-0.117603D-04
42	-0.171926D-04	0.570089D-05	0.135217D-04	-0.153236D-04	-0.257683D-04

ESTIMATED COVARIANCE MATRIX FOR PARAMETER ESTIMATES

	6	7	8	9	10
6	0.172143D-03				
7	0.240056D-05	0.458619D-02			
8	0.117349D-04	-0.338452D-03	0.191196D-02		
9	-0.477845D-05	-0.346399D-05	0.166616D-03	0.605097D-03	
10	0.667639D-05	0.655793D-04	-0.341281D-03	0.541034D-04	0.236948D-02
11	0.101617D-04	-0.234891D-04	-0.111060D-03	-0.135203D-04	-0.222910D-03
12	0.111028D-04	0.411422D-04	0.158924D-03	-0.628309D-04	0.148185D-03
13	0.203381D-06	0.141488D-05	0.213984D-05	-0.789248D-06	-0.680157D-05
14	-0.148547D-05	0.269970D-05	-0.601389D-06	-0.222738D-05	-0.846942D-05
15	0.104729D-05	-0.290596D-05	0.618672D-05	-0.143905D-05	-0.146711D-05
16	0.142511D-05	-0.866479D-06	0.105748D-04	-0.143381D-05	0.527528D-06
17	0.293553D-05	-0.160357D-05	-0.152656D-04	-0.340398D-05	-0.103203D-04
18	0.410768D-05	0.195194D-04	0.562724D-04	0.244709D-04	-0.351877D-05
19	0.272746D-05	0.111036D-04	0.256660D-07	-0.223735D-06	-0.570255D-05
20	0.311854D-06	-0.235901D-05	-0.416751D-05	0.112919D-05	-0.356130D-05
21	-0.107672D-05	-0.782448D-05	-0.690975D-05	-0.114773D-05	0.148491D-04
22	0.904914D-06	-0.840747D-05	-0.136543D-05	0.160563D-06	-0.644307D-05
23	0.280578D-05	-0.106734D-05	0.955122D-05	-0.279150D-05	-0.104169D-04
24	0.257522D-05	-0.115087D-05	-0.199299D-05	-0.168314D-05	-0.857760D-05

25	0.445514D-05	0.226356D-04	-0.194211D-04	-0.244020D-04	0.215277D-04
26	0.318036D-05	-0.447711D-05	0.150612D-05	-0.187679D-05	-0.175545D-04
27	0.350000D-05	0.213606D-04	-0.187861D-04	0.340014D-05	0.329422D-05
28	-0.389511D-06	-0.592881D-05	0.171812D-05	0.105444D-04	0.170803D-04
29	-0.778833D-06	0.171840D-04	-0.497530D-05	-0.329088D-05	0.839914D-06
30	0.138523D-05	0.293418D-04	0.122801D-04	-0.237258D-05	-0.883117D-06
31	-0.379498D-05	-0.494497D-05	0.271054D-05	0.760494D-06	0.310611D-05
32	-0.346393D-06	-0.122106D-05	-0.853368D-05	-0.141386D-05	0.158323D-04
33	0.842293D-05	0.295482D-04	0.368445D-06	0.892118D-05	0.897106D-05
34	-0.880889D-05	-0.134920D-03	0.487310D-04	-0.297013D-05	0.146387D-03
35	-0.700965D-05	0.429827D-04	-0.159944D-03	-0.519188D-06	-0.111452D-03
36	0.443296D-05	-0.155426D-03	-0.760972D-04	-0.852852D-05	-0.349661D-04
37	-0.272381D-04	-0.276182D-03	-0.175127D-04	0.353821D-04	0.480363D-05
38	-0.423030D-05	0.194895D-04	0.121973D-04	-0.100166D-04	-0.161663D-04
39	-0.163365D-06	-0.129777D-03	-0.544884D-05	-0.871235D-05	0.768810D-05
40	0.141538D-04	0.119192D-03	-0.690637D-04	-0.110983D-04	0.415417D-04
41	-0.128368D-04	-0.997065D-04	0.694637D-04	0.383872D-04	-0.152878D-04
42	-0.260250D-04	0.814466D-03	0.112777D-03	-0.202061D-04	-0.520952D-04

ESTIMATED COVARIANCE MATRIX FOR PARAMETER ESTIMATES

	11	12	13	14	15
11	0.704224D-03				
12	0.705727D-04	0.734793D-03			
13	0.445559D-05	0.971049D-06	0.214981D-04		
14	0.186653D-05	0.136341D-05	0.485135D-05	0.138783D-04	
15	-0.303201D-05	0.539176D-05	0.852146D-06	0.596767D-05	0.329717D-04
16	-0.421550D-05	0.772359D-05	-0.438409D-05	-0.441630D-05	-0.104982D-05
17	0.248874D-05	0.261010D-05	-0.145954D-05	-0.322309D-05	-0.827429D-05
18	0.545683D-06	-0.129693D-04	0.141892D-05	0.355578D-05	0.277816D-05
19	0.636355D-05	0.396114D-05	0.891544D-06	0.307591D-05	0.118468D-05
20	0.442335D-05	0.741344D-06	0.582611D-06	0.130366D-05	0.640352D-05
21	-0.408458D-05	0.538800D-05	0.772940D-07	-0.117742D-05	-0.154030D-05
22	-0.392852D-07	-0.328839D-05	-0.152747D-07	0.871177D-06	0.144604D-05
23	0.757521D-05	0.428000D-05	0.444248D-05	0.518556D-05	0.181706D-05
24	0.112033D-04	-0.392786D-05	0.177155D-05	0.357331D-05	0.120585D-04
25	-0.485139D-05	0.228710D-04	-0.885318D-06	-0.257006D-05	-0.182481D-05
26	0.541446D-05	-0.997067D-05	-0.791054D-06	0.471459D-06	0.213094D-05
27	-0.514828D-05	-0.842938D-05	-0.260431D-06	0.101935D-05	0.141642D-05
28	-0.734682D-05	-0.154609D-05	-0.512053D-05	-0.563404D-05	-0.259342D-05
29	-0.209742D-05	-0.726885D-06	-0.138491D-05	-0.339828D-05	-0.106532D-04
30	-0.815410D-06	0.138426D-04	-0.355617D-06	0.166339D-05	0.241435D-05
31	-0.157878D-05	0.202997D-05	-0.572872D-06	-0.615539D-06	-0.110096D-06

32	-0.104618D-04	0.759832D-05	-0.108834D-05	-0.192495D-05	-0.137091D-05
33	-0.671134D-06	0.377898D-05	-0.186732D-07	0.410563D-06	0.157480D-05
34	-0.632551D-04	0.419732D-04	-0.869024D-05	0.252141D-07	0.935587D-05
35	-0.991612D-06	0.225283D-04	0.887585D-05	0.752318D-05	-0.157102D-04
36	-0.106340D-03	-0.637462D-04	0.102937D-04	0.375561D-05	-0.258569D-04
37	-0.758667D-04	0.505575D-04	0.167285D-04	0.582503D-05	-0.137708D-04
38	0.137333D-04	0.291934D-04	0.162297D-05	0.159358D-05	0.308353D-05
39	-0.214823D-04	0.132085D-04	0.385857D-05	-0.120481D-05	-0.127046D-05
40	-0.153993D-05	0.130429D-04	-0.814240D-05	-0.245221D-05	0.729748D-05
41	-0.440952D-05	-0.775354D-05	0.105119D-04	-0.573796D-06	-0.895449D-05
42	-0.104752D-03	0.225999D-04	0.969233D-05	0.962333D-05	-0.527209D-05

ESTIMATED COVARIANCE MATRIX FOR PARAMETER ESTIMATES

	16	17	18	19	20
16	0.505687D-04				
17	0.100045D-04	0.615420D-04			
18	-0.149661D-04	-0.320715D-04	0.447054D-03		
19	-0.296265D-05	-0.111141D-05	0.161407D-05	0.111932D-04	
20	-0.132057D-05	-0.249413D-05	-0.108682D-05	0.277664D-05	0.141281D-04
21	0.220242D-05	0.116363D-04	-0.167733D-04	-0.198765D-05	-0.522933D-05
22	-0.797741D-06	-0.172507D-05	0.162304D-05	0.125918D-05	0.574457D-05
23	-0.229587D-05	-0.107870D-05	-0.155241D-05	0.436576D-05	0.207609D-05
24	-0.149775D-05	-0.747246D-05	0.449850D-05	0.150374D-05	0.788637D-05
25	0.123235D-04	0.250382D-04	-0.446319D-04	-0.242699D-06	-0.246007D-05
26	-0.574132D-06	-0.162645D-05	0.250214D-05	0.202481D-05	0.627807D-05
27	-0.154637D-05	-0.272628D-05	-0.122485D-04	0.161053D-05	0.444263D-05
28	0.782146D-05	0.160519D-05	-0.941444D-06	-0.407089D-05	-0.104191D-05
29	0.228949D-05	0.106606D-04	-0.695541D-05	-0.149179D-05	-0.450084D-05
30	-0.447733D-05	-0.206402D-04	0.725644D-04	0.194819D-06	-0.130080D-05
31	0.816404D-06	0.176001D-05	-0.747087D-05	-0.106603D-05	-0.499890D-05
32	-0.107982D-05	0.299067D-05	0.183509D-05	-0.157736D-05	-0.124833D-05
33	-0.634285D-06	-0.167391D-05	-0.320306D-06	0.290038D-05	0.271566D-05
34	-0.142108D-04	0.132226D-04	-0.236254D-04	-0.258262D-05	0.521696D-06
35	0.900196D-05	0.131903D-05	-0.203357D-04	0.401633D-06	-0.295668D-05
36	0.102519D-04	0.387841D-04	-0.545639D-04	-0.449732D-05	0.729731D-05
37	0.980412D-07	0.118527D-04	-0.741471D-04	-0.111789D-04	0.116466D-05
38	0.139823D-05	-0.285598D-05	0.431943D-05	0.102345D-05	0.244045D-05
39	0.375431D-05	-0.250894D-05	-0.475007D-05	-0.453062D-05	0.640963D-05
40	0.203538D-05	-0.672471D-05	-0.434681D-05	0.792705D-06	-0.272192D-05
41	-0.101972D-04	0.631161D-05	0.261875D-04	0.160345D-05	0.401436D-06
42	0.874319D-05	0.136939D-04	-0.342520D-04	-0.336059D-05	0.410990D-05

ESTIMATED COVARIANCE MATRIX FOR PARAMETER ESTIMATES

	21	22	23	24	25
21	0.513634D-04				
22	-0.542377D-05	0.215536D-04			
23	-0.400515D-06	0.131709D-05	0.437043D-04		
24	-0.394452D-05	0.284627D-05	0.108568D-04	0.616663D-04	
25	0.179140D-04	-0.120826D-05	-0.675482D-05	-0.273987D-04	0.217469D-03
26	-0.760592D-05	0.788776D-05	0.189775D-05	0.116583D-04	-0.151789D-04
27	-0.872484D-05	0.400062D-05	0.144344D-04	0.425992D-04	-0.512405D-04
28	0.165424D-05	-0.106133D-05	-0.765666D-05	-0.277516D-05	0.281166D-05
29	0.327445D-05	-0.225851D-05	-0.362422D-05	-0.121311D-04	0.631827D-05
30	-0.146361D-04	0.133815D-05	-0.116684D-08	0.325975D-05	-0.433263D-04
31	0.929445D-05	-0.688771D-05	-0.548248D-06	-0.363141D-05	0.705965D-05
32	0.638498D-05	-0.430649D-05	-0.108957D-04	-0.243240D-04	0.435515D-04
33	-0.323136D-05	0.311929D-05	0.285587D-05	-0.523602D-06	-0.103701D-04
34	0.904734D-05	-0.765966D-05	-0.268938D-04	-0.121872D-04	0.512813D-05
35	-0.267731D-05	0.270659D-06	0.195694D-04	0.657776D-05	0.326027D-04
36	-0.170486D-04	0.204719D-04	0.169685D-04	-0.624825D-05	-0.303794D-04
37	0.432142D-05	0.933162D-05	-0.148363D-04	-0.159189D-04	-0.318386D-04
38	0.445818D-06	0.225352D-05	-0.139256D-05	0.677367D-06	0.101157D-05
39	0.142448D-06	-0.343936D-05	0.901273D-05	0.277201D-04	0.817832D-07
40	-0.905781D-05	0.368201D-05	-0.428600D-07	-0.118405D-04	-0.143337D-05
41	0.201479D-04	-0.695975D-05	-0.610820D-05	0.595229D-05	0.287885D-04
42	-0.769680D-05	0.515259D-05	0.625859D-05	0.705783D-05	-0.397401D-04

ESTIMATED COVARIANCE MATRIX FOR PARAMETER ESTIMATES

	26	27	28	29	30
26	0.454249D-04				
27	0.373949D-04	0.389058D-03			
28	0.247172D-07	-0.178896D-06	0.388876D-04		
29	-0.137728D-05	-0.101917D-04	0.844751D-05	0.494089D-04	
30	0.299785D-06	0.105489D-04	-0.576238D-05	-0.173671D-04	0.180873D-03
31	-0.789021D-05	-0.783265D-05	0.369556D-06	0.100103D-04	-0.125975D-04
32	-0.153489D-04	-0.672017D-04	0.664787D-05	0.182894D-04	-0.280035D-04
33	-0.136685D-05	0.309394D-05	-0.101841D-04	-0.372453D-04	0.620931D-04
34	-0.382666D-05	-0.342502D-04	0.190061D-04	-0.624183D-06	0.100173D-04
35	-0.116126D-04	0.230184D-04	-0.313610D-05	0.334359D-05	-0.257024D-04
36	-0.607194D-05	0.189153D-04	0.351583D-05	0.964406D-06	-0.831945D-04
37	-0.163995D-05	-0.114175D-04	0.891226D-06	0.959859D-05	-0.563056D-04
38	0.887741D-06	0.809305D-05	-0.257435D-05	-0.137809D-06	0.570429D-05

39	0.481843D-05	0.147230D-06	-0.534289D-07	-0.473176D-05	0.476115D-05
40	-0.148692D-05	-0.187840D-04	0.536712D-05	0.296944D-05	-0.204541D-04
41	-0.310806D-05	0.499999D-04	-0.829038D-05	-0.870881D-06	0.200990D-04
42	-0.128015D-04	-0.974896D-06	-0.893760D-05	-0.486954D-05	-0.258914D-04

ESTIMATED COVARIANCE MATRIX FOR PARAMETER ESTIMATES

	31	32	33	34	35
31	0.403404D-04				
32	0.190746D-04	0.174396D-03			
33	-0.287522D-04	-0.649412D-04	0.289356D-03		
34	0.105570D-04	0.155427D-04	0.329575D-04	0.395787D-02	
35	0.162046D-05	-0.207842D-04	-0.836107D-05	-0.226115D-02	0.467671D-02
36	-0.102182D-05	-0.301297D-04	-0.217690D-04	0.582224D-04	0.327464D-04
37	0.883931D-05	-0.110716D-04	-0.432684D-04	0.743705D-04	-0.674570D-04
38	0.118711D-05	0.467311D-05	0.798377D-06	0.899107D-05	-0.246538D-04
39	-0.776570D-05	-0.243485D-04	-0.105567D-04	-0.573708D-05	0.289884D-04
40	0.111508D-04	0.401797D-04	-0.139224D-04	-0.438822D-04	0.737231D-04
41	-0.882952D-05	-0.542655D-04	0.582685D-04	0.118491D-03	-0.579224D-04
42	0.130283D-04	-0.729813D-05	-0.267488D-04	-0.350880D-04	-0.365118D-04

ESTIMATED COVARIANCE MATRIX FOR PARAMETER ESTIMATES

	36	37	38	39	40
36	0.904724D-02				
37	0.543376D-02	0.769950D-02			
38	-0.195736D-03	-0.237303D-04	0.145748D-03		
39	0.276678D-03	0.123559D-03	0.678635D-06	0.192085D-02	
40	-0.185277D-03	-0.470774D-04	-0.483784D-05	-0.114534D-02	0.145512D-02
41	0.225171D-03	0.708226D-04	0.104220D-04	0.697443D-03	-0.134992D-02
42	0.545813D-02	0.546318D-02	0.316162D-04	0.165591D-03	-0.883802D-04

ESTIMATED COVARIANCE MATRIX FOR PARAMETER ESTIMATES

	41	42
41	0.245688D-02	
42	0.645189D-04	0.574528D-02

ESTIMATED CORRELATION MATRIX FOR PARAMETER ESTIMATES

1	2	3	4	5

1	1.000				
2	0.234	1.000			
3	-0.084	-0.172	1.000		
4	0.088	0.253	-0.116	1.000	
5	0.062	0.176	-0.090	0.228	1.000
6	-0.108	-0.168	0.181	-0.155	-0.169
7	-0.026	-0.007	-0.019	-0.021	-0.043
8	-0.013	-0.019	0.034	0.008	-0.014
9	-0.058	-0.014	0.048	-0.007	0.006
10	0.032	-0.015	-0.015	0.030	-0.034
11	0.051	0.029	0.026	-0.013	0.013
12	0.024	0.019	-0.039	0.027	0.005
13	-0.013	0.019	0.030	-0.034	-0.014
14	-0.023	0.037	0.023	-0.026	-0.009
15	-0.049	0.029	0.002	0.005	0.010
16	-0.008	0.000	0.011	0.010	0.018
17	0.040	-0.002	-0.018	0.005	0.019
18	0.004	0.028	0.010	0.017	-0.009
19	0.017	-0.008	-0.023	-0.011	0.003
20	-0.013	-0.005	-0.010	0.002	0.002
21	0.004	0.018	0.000	0.000	-0.003
22	-0.003	-0.020	0.019	-0.017	0.030
23	-0.024	-0.007	-0.039	-0.035	0.000
24	-0.006	0.036	-0.012	0.023	-0.006
25	0.017	-0.003	-0.021	-0.011	-0.023
26	-0.026	-0.035	0.014	0.010	0.004
27	-0.028	-0.036	-0.014	0.005	-0.015
28	-0.026	0.007	0.009	0.011	-0.005
29	0.042	0.009	0.020	-0.010	0.009
30	-0.014	0.006	-0.014	0.006	-0.022
31	0.036	0.037	0.008	-0.008	0.019
32	0.008	0.002	0.021	-0.040	-0.017
33	-0.006	-0.002	0.005	0.006	0.016
34	-0.015	-0.019	-0.039	-0.007	-0.005
35	0.007	0.012	0.047	0.005	0.013
36	-0.005	0.005	0.019	0.000	0.011
37	-0.016	0.005	0.025	-0.015	-0.005
38	-0.005	-0.037	0.005	0.001	-0.007
39	-0.009	0.027	0.039	0.035	-0.037
40	-0.005	-0.047	-0.065	-0.035	0.050
41	0.017	0.068	0.055	0.027	-0.017
42	-0.034	0.010	0.012	-0.029	-0.024

ESTIMATED CORRELATION MATRIX FOR PARAMETER ESTIMATES

	6	7	8	9	10
6	1.000				
7	0.003	1.000			
8	0.020	-0.114	1.000		
9	-0.015	-0.002	0.155	1.000	
10	0.010	0.020	-0.160	0.045	1.000
11	0.029	-0.013	-0.096	-0.021	-0.173
12	0.031	0.022	0.134	-0.094	0.112
13	0.003	0.005	0.011	-0.007	-0.030
14	-0.030	0.011	-0.004	-0.024	-0.047
15	0.014	-0.007	0.025	-0.010	-0.005
16	0.015	-0.002	0.034	-0.008	0.002
17	0.029	-0.003	-0.045	-0.018	-0.027
18	0.015	0.014	0.061	0.047	-0.003
19	0.062	0.049	0.000	-0.003	-0.035
20	0.006	-0.009	-0.025	0.012	-0.019
21	-0.011	-0.016	-0.022	-0.007	0.043
22	0.015	-0.027	-0.007	0.001	-0.029
23	0.032	-0.002	0.033	-0.017	-0.032
24	0.025	-0.002	-0.006	-0.009	-0.022
25	0.023	0.023	-0.030	-0.067	0.030
26	0.036	-0.010	0.005	-0.011	-0.054
27	0.014	0.016	-0.022	0.007	0.003
28	-0.005	-0.014	0.006	0.069	0.056
29	-0.008	0.036	-0.016	-0.019	0.002
30	0.008	0.032	0.021	-0.007	-0.001
31	-0.046	-0.011	0.010	0.005	0.010
32	-0.002	-0.001	-0.015	-0.004	0.025
33	0.038	0.026	0.000	0.021	0.011
34	-0.011	-0.032	0.018	-0.002	0.048
35	-0.008	0.009	-0.053	0.000	-0.033
36	0.004	-0.024	-0.018	-0.004	-0.008
37	-0.024	-0.046	-0.005	0.016	0.001
38	-0.027	0.024	0.023	-0.034	-0.028
39	0.000	-0.044	-0.003	-0.008	0.004
40	0.028	0.046	-0.041	-0.012	0.022
41	-0.020	-0.030	0.032	0.031	-0.006
42	-0.026	0.159	0.034	-0.011	-0.014

ESTIMATED CORRELATION MATRIX FOR PARAMETER ESTIMATES

	11	12	13	14	15
11	1.000				
12	0.098	1.000			
13	0.036	0.008	1.000		
14	0.019	0.014	0.281	1.000	
15	-0.020	0.035	0.032	0.279	1.000
16	-0.022	0.040	-0.133	-0.167	-0.026
17	0.012	0.012	-0.040	-0.110	-0.184
18	0.001	-0.023	0.014	0.045	0.023
19	0.072	0.044	0.057	0.247	0.062
20	0.044	0.007	0.033	0.093	0.297
21	-0.021	0.028	0.002	-0.044	-0.037
22	0.000	-0.026	-0.001	0.050	0.054
23	0.043	0.024	0.145	0.211	0.048
24	0.054	-0.018	0.049	0.122	0.267
25	-0.012	0.057	-0.013	-0.047	-0.022
26	0.030	-0.055	-0.025	0.019	0.055
27	-0.010	-0.016	-0.003	0.014	0.013
28	-0.044	-0.009	-0.177	-0.243	-0.072
29	-0.011	-0.004	-0.042	-0.130	-0.264
30	-0.002	0.038	-0.006	0.033	0.031
31	-0.009	0.012	-0.019	-0.026	-0.003
32	-0.030	0.021	-0.018	-0.039	-0.018
33	-0.001	0.008	0.000	0.006	0.016
34	-0.038	0.025	-0.030	0.000	0.026
35	-0.001	0.012	0.028	0.030	-0.040
36	-0.042	-0.025	0.023	0.011	-0.047
37	-0.033	0.021	0.041	0.018	-0.027
38	0.043	0.089	0.029	0.035	0.044
39	-0.018	0.011	0.019	-0.007	-0.005
40	-0.002	0.013	-0.046	-0.017	0.033
41	-0.003	-0.006	0.046	-0.003	-0.031
42	-0.052	0.011	0.028	0.034	-0.012

ESTIMATED CORRELATION MATRIX FOR PARAMETER ESTIMATES

	16	17	18	19	20
16	1.000				
17	0.179	1.000			
18	-0.100	-0.193	1.000		
19	-0.125	-0.042	0.023	1.000	
20	-0.049	-0.085	-0.014	0.221	1.000

21	0.043	0.207	-0.111	-0.083	-0.194
22	-0.024	-0.047	0.017	0.081	0.329
23	-0.049	-0.021	-0.011	0.197	0.084
24	-0.027	-0.121	0.027	0.057	0.267
25	0.118	0.216	-0.143	-0.005	-0.044
26	-0.012	-0.031	0.018	0.090	0.248
27	-0.011	-0.018	-0.029	0.024	0.060
28	0.176	0.033	-0.007	-0.195	-0.044
29	0.046	0.193	-0.047	-0.063	-0.170
30	-0.047	-0.196	0.255	0.004	-0.026
31	0.018	0.035	-0.056	-0.050	-0.209
32	-0.011	0.029	0.007	-0.036	-0.025
33	-0.005	-0.013	-0.001	0.051	0.042
34	-0.032	0.027	-0.018	-0.012	0.002
35	0.019	0.002	-0.014	0.002	-0.012
36	0.015	0.052	-0.027	-0.014	0.020
37	0.000	0.017	-0.040	-0.038	0.004
38	0.016	-0.030	0.017	0.025	0.054
39	0.012	-0.007	-0.005	-0.031	0.039
40	0.008	-0.022	-0.005	0.006	-0.019
41	-0.029	0.016	0.025	0.010	0.002
42	0.016	0.023	-0.021	-0.013	0.014

ESTIMATED CORRELATION MATRIX FOR PARAMETER ESTIMATES

	21	22	23	24	25
21	1.000				
22	-0.163	1.000			
23	-0.008	0.043	1.000		
24	-0.070	0.078	0.209	1.000	
25	0.169	-0.018	-0.069	-0.237	1.000
26	-0.157	0.252	0.043	0.220	-0.153
27	-0.062	0.044	0.111	0.275	-0.176
28	0.037	-0.037	-0.186	-0.057	0.031
29	0.065	-0.069	-0.078	-0.220	0.061
30	-0.152	0.021	0.000	0.031	-0.218
31	0.204	-0.234	-0.013	-0.073	0.075
32	0.067	-0.070	-0.125	-0.235	0.224
33	-0.027	0.039	0.025	-0.004	-0.041
34	0.020	-0.026	-0.065	-0.025	0.006
35	-0.005	0.001	0.043	0.012	0.032
36	-0.025	0.046	0.027	-0.008	-0.022
37	0.007	0.023	-0.026	-0.023	-0.025

38	0.005	0.040	-0.017	0.007	0.006
39	0.000	-0.017	0.031	0.081	0.000
40	-0.033	0.021	0.000	-0.040	-0.003
41	0.057	-0.030	-0.019	0.015	0.039
42	-0.014	0.015	0.012	0.012	-0.036

ESTIMATED CORRELATION MATRIX FOR PARAMETER ESTIMATES

	26	27	28	29	30
26	1.000				
27	0.281	1.000			
28	0.001	-0.001	1.000		
29	-0.029	-0.074	0.193	1.000	
30	0.003	0.040	-0.069	-0.184	1.000
31	-0.184	-0.063	0.009	0.224	-0.147
32	-0.172	-0.258	0.081	0.197	-0.158
33	-0.012	0.009	-0.096	-0.311	0.271
34	-0.009	-0.028	0.048	-0.001	0.012
35	-0.025	0.017	-0.007	0.007	-0.028
36	-0.009	0.010	0.006	0.001	-0.065
37	-0.003	-0.007	0.002	0.016	-0.048
38	0.011	0.034	-0.034	-0.002	0.035
39	0.016	0.000	0.000	-0.015	0.008
40	-0.006	-0.025	0.023	0.011	-0.040
41	-0.009	0.051	-0.027	-0.002	0.030
42	-0.025	-0.001	-0.019	-0.009	-0.025

ESTIMATED CORRELATION MATRIX FOR PARAMETER ESTIMATES

	31	32	33	34	35
31	1.000				
32	0.227	1.000			
33	-0.266	-0.289	1.000		
34	0.026	0.019	0.031	1.000	
35	0.004	-0.023	-0.007	-0.526	1.000
36	-0.002	-0.024	-0.013	0.010	0.005
37	0.016	-0.010	-0.029	0.013	-0.011
38	0.015	0.029	0.004	0.012	-0.030
39	-0.028	-0.042	-0.014	-0.002	0.010
40	0.046	0.080	-0.021	-0.018	0.028
41	-0.028	-0.083	0.069	0.038	-0.017
42	0.027	-0.007	-0.021	-0.007	-0.007

ESTIMATED CORRELATION MATRIX FOR PARAMETER ESTIMATES

	36	37	38	39	40
36	1.000				
37	0.651	1.000			
38	-0.170	-0.022	1.000		
39	0.066	0.032	0.001	1.000	
40	-0.051	-0.014	-0.011	-0.685	1.000
41	0.048	0.016	0.017	0.321	-0.714
42	0.757	0.821	0.035	0.050	-0.031

ESTIMATED CORRELATION MATRIX FOR PARAMETER ESTIMATES

	41	42
41	1.000	
42	0.017	1.000

TECHNICAL 8 OUTPUT

TECHNICAL 8 OUTPUT FOR BAYES ESTIMATION

CHAIN	BSEED
1	0
2	285380
3	253358

ITERATION	POTENTIAL SCALE REDUCTION	PARAMETER WITH HIGHEST PSR
100	1.014	34
200	1.004	32
300	1.001	42
400	1.003	3
500	1.005	37
600	1.002	3
700	1.001	27
800	1.002	8
900	1.001	8
1000	1.002	30

This is improvement in physical health

SUMMARY OF DATA

Number of clusters 57

Size (s)	Cluster ID with Size s
2	4 166
3	65 74 80 30
4	84 22
5	205
6	228
7	93
8	106 144 165 71 170 173 192 72 50
9	40 108
11	107 83 181 61 75 68
12	69 174 206 226 81
13	162
16	92 154
19	114
20	145 2
22	116
23	36 156 169 142
24	152
26	98
28	122 202
30	178
32	6
46	24
59	204
62	125
64	209
108	217
216	110
223	100

COVARIANCE COVERAGE OF DATA

Minimum covariance coverage value 0.100

Number of missing data patterns 4

PROPORTION OF DATA PRESENT

Covariance Coverage

	PHYCAT16	CLU2	CLU3	PHY15	KES15
PHYCAT16	1.000				
CLU2	1.000	1.000			
CLU3	1.000	1.000	1.000		
PHY15	1.000	1.000	1.000	1.000	
KES15	0.971	0.971	0.971	0.971	0.971
CHOICE15	0.963	0.963	0.963	0.963	0.963
BULLY15	0.966	0.966	0.966	0.966	0.966
PRESS15	0.966	0.966	0.966	0.966	0.966
DUTIES15	0.966	0.966	0.966	0.966	0.966

Covariance Coverage

	CHOICE15	BULLY15	PRESS15	DUTIES15
CHOICE15	0.963			
BULLY15	0.963	0.966		
PRESS15	0.963	0.966	0.966	
DUTIES15	0.963	0.966	0.966	0.966

UNIVARIATE PROPORTIONS AND COUNTS FOR CATEGORICAL VARIABLES

PHYCAT16

Category 1	0.364	517.000
Category 2	0.636	905.000

UNIVARIATE SAMPLE STATISTICS

UNIVARIATE HIGHER-ORDER MOMENT DESCRIPTIVE STATISTICS

Variable/ Sample Size	Mean/ Variance	Skewness/ Kurtosis	Minimum/ Maximum	% with Min/Max	Percentiles 20%/60%	40%/80%	Median

```
     CLU2                        0.316       0.793       0.000   68.42%       0.000      0.000      0.000
              57.000             0.216      -1.372       1.000   31.58%       0.000      1.000
     CLU3                        0.439       0.247       0.000   56.14%       0.000      0.000      0.000
              57.000             0.246      -1.939       1.000   43.86%       1.000      1.000
     PHY15                       0.000       2.446      -0.155   85.58%      -0.155     -0.155     -0.155
            1422.000             0.154       5.391       1.845    1.13%      -0.155     -0.155
     KES15                       0.000       1.141      -0.953    8.62%      -0.620     -0.287     -0.120
            1381.000             0.613       1.071       3.047    0.36%       0.047      0.547
     CHOICE15                    0.000      -0.138      -1.878   14.60%      -0.878      0.122      0.122
            1370.000             1.250      -0.758       2.122    5.77%       0.122      1.122
     BULLY15                     0.000       2.479      -0.312   79.02%      -0.312     -0.312     -0.312
            1373.000             0.474       6.315       3.688    0.44%      -0.312      0.688
     PRESS15                     0.000       0.183      -1.756   14.06%      -0.756     -0.756      0.244
            1373.000             1.207      -0.514       2.244    7.21%       0.244      1.244
     DUTIES15                    0.000      -0.896      -2.873    3.06%      -0.873      0.127      0.127
            1373.000             0.975       0.587       1.127   27.75%       0.127      1.127


THE MODEL ESTIMATION TERMINATED NORMALLY

     USE THE FBITERATIONS OPTION TO INCREASE THE NUMBER OF ITERATIONS BY A FACTOR
     OF AT LEAST TWO TO CHECK CONVERGENCE AND THAT THE PSR VALUE DOES NOT INCREASE.



MODEL FIT INFORMATION

Number of Free Parameters                              42

Bayesian Posterior Predictive Checking using Chi-Square

          95% Confidence Interval for the Difference Between
          the Observed and the Replicated Chi-Square Values

                              -22.496            26.467

          Posterior Predictive P-Value              0.458



MODEL RESULTS

                                Posterior  One-Tailed         95% C.I.
                    Estimate       S.D.      P-Value   Lower 2.5%  Upper 2.5%  Significance
```

Within Level

PHYCAT16 ON						
PHY15	0.813	0.115	0.000	0.597	1.055	*
CHOICE15	-0.047	0.032	0.075	-0.111	0.016	
BULLY15	0.049	0.056	0.204	-0.059	0.162	
DUTIES15	0.033	0.038	0.178	-0.036	0.111	
PRESS15	-0.003	0.035	0.463	-0.073	0.066	
KES15	0.167	0.051	0.002	0.062	0.265	*
KES15 WITH						
PHY15	0.070	0.008	0.000	0.055	0.087	*
CHOICE15	-0.134	0.024	0.000	-0.182	-0.088	*
BULLY15	0.128	0.015	0.000	0.098	0.158	*
PRESS15	0.206	0.024	0.000	0.158	0.253	*
DUTIES15	-0.206	0.021	0.000	-0.250	-0.166	*
PHY15 WITH						
CHOICE15	-0.032	0.012	0.003	-0.056	-0.009	*
BULLY15	0.012	0.007	0.046	-0.002	0.026	
PRESS15	0.016	0.012	0.107	-0.008	0.037	
DUTIES15	-0.009	0.010	0.181	-0.029	0.011	
CHOICE15 WITH						
BULLY15	-0.133	0.021	0.000	-0.174	-0.092	*
PRESS15	-0.181	0.033	0.000	-0.253	-0.119	*
DUTIES15	0.161	0.029	0.000	0.107	0.219	*
BULLY15 WITH						
PRESS15	0.177	0.020	0.000	0.139	0.218	*
DUTIES15	-0.140	0.019	0.000	-0.178	-0.103	*
PRESS15 WITH						
DUTIES15	-0.242	0.030	0.000	-0.306	-0.182	*
Means						
PHY15	0.000	0.010	0.484	-0.020	0.020	
KES15	0.000	0.021	0.488	-0.040	0.041	
CHOICE15	-0.001	0.030	0.487	-0.060	0.059	
BULLY15	0.000	0.019	0.499	-0.035	0.036	
PRESS15	0.002	0.031	0.473	-0.061	0.063	
DUTIES15	-0.002	0.027	0.465	-0.053	0.053	

Variances						
PHY15	0.154	0.006	0.000	0.144	0.167	*
KES15	0.613	0.023	0.000	0.571	0.660	*
CHOICE15	1.251	0.048	0.000	1.162	1.356	*
BULLY15	0.475	0.018	0.000	0.441	0.513	*
PRESS15	1.209	0.045	0.000	1.124	1.299	*
DUTIES15	0.974	0.038	0.000	0.908	1.053	*
**Between Level**						
PHYCAT16 ON						
CLU2	-0.128	0.117	0.135	-0.360	0.093	
CLU3	-0.032	0.112	0.393	-0.238	0.194	
CLU2 WITH						
CLU3	-0.137	0.038	0.000	-0.225	-0.078	*
Means						
CLU2	0.317	0.064	0.000	0.188	0.442	*
CLU3	0.435	0.069	0.000	0.297	0.567	*
Thresholds						
PHYCAT16$1	-0.451	0.096	0.000	-0.640	-0.259	*
Variances						
CLU2	0.232	0.045	0.000	0.164	0.338	*
CLU3	0.264	0.050	0.000	0.186	0.376	*
Residual Variances						
PHYCAT16	0.008	0.011	0.000	0.000	0.039	*

STANDARDIZED MODEL RESULTS

STDYX Standardization

	Estimate	Posterior S.D.	One-Tailed P-Value	95% C.I. Lower 2.5%	Upper 2.5%	Significance
**Within Level**						
PHYCAT16 ON						
PHY15	0.297	0.039	0.000	0.223	0.376	*
CHOICE15	-0.049	0.034	0.075	-0.116	0.017	

```
   BULLY15            0.031      0.036      0.204     -0.038      0.102
   DUTIES15           0.030      0.035      0.178     -0.034      0.103
   PRESS15           -0.003      0.035      0.463     -0.074      0.067
   KES15              0.122      0.037      0.002      0.044      0.192      *

KES15    WITH
   PHY15              0.230      0.025      0.000      0.181      0.280      *
   CHOICE15          -0.153      0.027      0.000     -0.205     -0.102      *
   BULLY15            0.237      0.026      0.000      0.185      0.286      *
   PRESS15            0.240      0.026      0.000      0.187      0.288      *
   DUTIES15          -0.266      0.024      0.000     -0.315     -0.217      *

PHY15    WITH
   CHOICE15          -0.073      0.027      0.003     -0.125     -0.021      *
   BULLY15            0.045      0.027      0.046     -0.007      0.096
   PRESS15            0.036      0.027      0.107     -0.017      0.087
   DUTIES15          -0.023      0.027      0.181     -0.076      0.029

CHOICE15 WITH
   BULLY15           -0.172      0.027      0.000     -0.224     -0.119      *
   PRESS15           -0.148      0.026      0.000     -0.204     -0.097      *
   DUTIES15           0.146      0.025      0.000      0.097      0.196      *

BULLY15  WITH
   PRESS15            0.234      0.025      0.000      0.187      0.282      *
   DUTIES15          -0.205      0.026      0.000     -0.257     -0.152      *

PRESS15  WITH
   DUTIES15          -0.223      0.026      0.000     -0.276     -0.172      *

Means
   PHY15              0.001      0.026      0.484     -0.052      0.052
   KES15              0.001      0.027      0.488     -0.051      0.053
   CHOICE15          -0.001      0.027      0.487     -0.054      0.053
   BULLY15            0.000      0.027      0.499     -0.051      0.053
   PRESS15            0.002      0.028      0.473     -0.055      0.057
   DUTIES15          -0.002      0.027      0.465     -0.054      0.053

Variances
   PHY15              1.000      0.000      0.000      1.000      1.000
   KES15              1.000      0.000      0.000      1.000      1.000
   CHOICE15           1.000      0.000      0.000      1.000      1.000
   BULLY15            1.000      0.000      0.000      1.000      1.000
   PRESS15            1.000      0.000      0.000      1.000      1.000
```

```
    DUTIES15           1.000       0.000       0.000       1.000       1.000

Between Level

 PHYCAT16   ON
    CLU2              -0.536       0.421       0.135      -1.113       0.447
    CLU3              -0.141       0.460       0.393      -0.923       0.815

 CLU2     WITH
    CLU3              -0.560       0.090       0.000      -0.707      -0.365      *

 Means
    CLU2               0.658       0.144       0.000       0.375       0.945      *
    CLU3               0.852       0.153       0.000       0.548       1.147      *

 Thresholds
    PHYCAT16$1        -3.822       1.701       0.000      -8.351      -1.627      *

 Variances
    CLU2               1.000       0.000       0.000       1.000       1.000
    CLU3               1.000       0.000       0.000       1.000       1.000

 Residual Variances
    PHYCAT16           0.597       0.265       0.000       0.061       0.981      *


R-SQUARE

Within Level

                                Posterior  One-Tailed         95% C.I.
    Variable        Estimate       S.D.      P-Value   Lower 2.5%  Upper 2.5%

    PHYCAT16           0.134       0.026       0.000       0.084       0.189

Between Level

                                Posterior  One-Tailed         95% C.I.
    Variable        Estimate       S.D.      P-Value   Lower 2.5%  Upper 2.5%

    PHYCAT16           0.403       0.265       0.000       0.019       0.939


TECHNICAL 1 OUTPUT
```

PARAMETER SPECIFICATION FOR WITHIN

TAU

PHYCAT16
0

NU

PHYCAT16	PHY15	KES15	CHOICE15	BULLY15
0	0	0	0	0

NU

PRESS15	DUTIES15
0	0

LAMBDA

	PHYCAT16	PHY15	KES15	CHOICE15	BULLY15
PHYCAT16	0	0	0	0	0
PHY15	0	0	0	0	0
KES15	0	0	0	0	0
CHOICE15	0	0	0	0	0
BULLY15	0	0	0	0	0
PRESS15	0	0	0	0	0
DUTIES15	0	0	0	0	0

LAMBDA

	PRESS15	DUTIES15
PHYCAT16	0	0
PHY15	0	0
KES15	0	0
CHOICE15	0	0
BULLY15	0	0
PRESS15	0	0

DUTIES15	0	0

THETA

	PHYCAT16	PHY15	KES15	CHOICE15	BULLY15
PHYCAT16	0				
PHY15	0	0			
KES15	0	0	0		
CHOICE15	0	0	0	0	
BULLY15	0	0	0	0	0
PRESS15	0	0	0	0	0
DUTIES15	0	0	0	0	0

THETA

	PRESS15	DUTIES15
PRESS15	0	
DUTIES15	0	0

ALPHA

PHYCAT16	PHY15	KES15	CHOICE15	BULLY15
0	1	2	3	4

ALPHA

PRESS15	DUTIES15
5	6

BETA

	PHYCAT16	PHY15	KES15	CHOICE15	BULLY15
PHYCAT16	0	7	8	9	10
PHY15	0	0	0	0	0
KES15	0	0	0	0	0
CHOICE15	0	0	0	0	0
BULLY15	0	0	0	0	0
PRESS15	0	0	0	0	0
DUTIES15	0	0	0	0	0

BETA

	PRESS15	DUTIES15
PHYCAT16	11	12
PHY15	0	0
KES15	0	0
CHOICE15	0	0
BULLY15	0	0
PRESS15	0	0
DUTIES15	0	0

PSI

	PHYCAT16	PHY15	KES15	CHOICE15	BULLY15
PHYCAT16	0				
PHY15	0	13			
KES15	0	14	15		
CHOICE15	0	16	17	18	
BULLY15	0	19	20	21	22
PRESS15	0	23	24	25	26
DUTIES15	0	28	29	30	31

PSI

	PRESS15	DUTIES15
PRESS15	27	
DUTIES15	32	33

PARAMETER SPECIFICATION FOR BETWEEN

TAU

PHYCAT16
42

NU

PHYCAT16	CLU2	CLU3

	0	0	0

LAMBDA

	PHYCAT16	CLU2	CLU3
PHYCAT16	0	0	0
CLU2	0	0	0
CLU3	0	0	0

THETA

	PHYCAT16	CLU2	CLU3
PHYCAT16	0		
CLU2	0	0	
CLU3	0	0	0

ALPHA

PHYCAT16	CLU2	CLU3
0	34	35

BETA

	PHYCAT16	CLU2	CLU3
PHYCAT16	0	36	37
CLU2	0	0	0
CLU3	0	0	0

PSI

	PHYCAT16	CLU2	CLU3
PHYCAT16	38		
CLU2	0	39	
CLU3	0	40	41

STARTING VALUES FOR WITHIN

TAU

PHYCAT16
0.000

NU

PHYCAT16	PHY15	KES15	CHOICE15	BULLY15
0.000	0.000	0.000	0.000	0.000

NU

PRESS15	DUTIES15
0.000	0.000

LAMBDA

	PHYCAT16	PHY15	KES15	CHOICE15	BULLY15
PHYCAT16	1.000	0.000	0.000	0.000	0.000
PHY15	0.000	1.000	0.000	0.000	0.000
KES15	0.000	0.000	1.000	0.000	0.000
CHOICE15	0.000	0.000	0.000	1.000	0.000
BULLY15	0.000	0.000	0.000	0.000	1.000
PRESS15	0.000	0.000	0.000	0.000	0.000
DUTIES15	0.000	0.000	0.000	0.000	0.000

LAMBDA

	PRESS15	DUTIES15
PHYCAT16	0.000	0.000
PHY15	0.000	0.000
KES15	0.000	0.000
CHOICE15	0.000	0.000
BULLY15	0.000	0.000
PRESS15	1.000	0.000
DUTIES15	0.000	1.000

THETA

	PHYCAT16	PHY15	KES15	CHOICE15	BULLY15
PHYCAT16	0.000				
PHY15	0.000	0.000			
KES15	0.000	0.000	0.000		
CHOICE15	0.000	0.000	0.000	0.000	
BULLY15	0.000	0.000	0.000	0.000	0.000
PRESS15	0.000	0.000	0.000	0.000	0.000
DUTIES15	0.000	0.000	0.000	0.000	0.000

THETA

	PRESS15	DUTIES15
PRESS15	0.000	
DUTIES15	0.000	0.000

ALPHA

PHYCAT16	PHY15	KES15	CHOICE15	BULLY15
0.000	0.000	0.000	0.000	0.000

ALPHA

PRESS15	DUTIES15
0.000	0.000

BETA

	PHYCAT16	PHY15	KES15	CHOICE15	BULLY15
PHYCAT16	0.000	0.000	0.000	0.000	0.000
PHY15	0.000	0.000	0.000	0.000	0.000
KES15	0.000	0.000	0.000	0.000	0.000
CHOICE15	0.000	0.000	0.000	0.000	0.000
BULLY15	0.000	0.000	0.000	0.000	0.000
PRESS15	0.000	0.000	0.000	0.000	0.000
DUTIES15	0.000	0.000	0.000	0.000	0.000

BETA

PRESS15	DUTIES15

PHYCAT16	0.000	0.000
PHY15	0.000	0.000
KES15	0.000	0.000
CHOICE15	0.000	0.000
BULLY15	0.000	0.000
PRESS15	0.000	0.000
DUTIES15	0.000	0.000

PSI

	PHYCAT16	PHY15	KES15	CHOICE15	BULLY15
PHYCAT16	1.000				
PHY15	0.000	0.077			
KES15	0.000	0.000	0.306		
CHOICE15	0.000	0.000	0.000	0.625	
BULLY15	0.000	0.000	0.000	0.000	0.237
PRESS15	0.000	0.000	0.000	0.000	0.000
DUTIES15	0.000	0.000	0.000	0.000	0.000

PSI

	PRESS15	DUTIES15
PRESS15	0.604	
DUTIES15	0.000	0.488

STARTING VALUES FOR BETWEEN

TAU

PHYCAT16
-0.311

NU

PHYCAT16	CLU2	CLU3
0.000	0.000	0.000

LAMBDA

	PHYCAT16	CLU2	CLU3
PHYCAT16	1.000	0.000	0.000
CLU2	0.000	1.000	0.000
CLU3	0.000	0.000	1.000

THETA

	PHYCAT16	CLU2	CLU3
PHYCAT16	0.000		
CLU2	0.000	0.000	
CLU3	0.000	0.000	0.000

ALPHA

PHYCAT16	CLU2	CLU3
0.000	0.375	0.482

BETA

	PHYCAT16	CLU2	CLU3
PHYCAT16	0.000	0.000	0.000
CLU2	0.000	0.000	0.000
CLU3	0.000	0.000	0.000

PSI

	PHYCAT16	CLU2	CLU3
PHYCAT16	1.000		
CLU2	0.000	0.117	
CLU3	0.000	0.000	0.125

PRIORS FOR ALL PARAMETERS	PRIOR MEAN	PRIOR VARIANCE	PRIOR STD. DEV.
Parameter 1~N(0.000,infinity)	0.0000	infinity	infinity
Parameter 2~N(0.000,infinity)	0.0000	infinity	infinity
Parameter 3~N(0.000,infinity)	0.0000	infinity	infinity

Parameter 4~N(0.000,infinity)	0.0000	infinity	infinity
Parameter 5~N(0.000,infinity)	0.0000	infinity	infinity
Parameter 6~N(0.000,infinity)	0.0000	infinity	infinity
Parameter 7~N(0.000,5.000)	0.0000	5.0000	2.2361
Parameter 8~N(0.000,5.000)	0.0000	5.0000	2.2361
Parameter 9~N(0.000,5.000)	0.0000	5.0000	2.2361
Parameter 10~N(0.000,5.000)	0.0000	5.0000	2.2361
Parameter 11~N(0.000,5.000)	0.0000	5.0000	2.2361
Parameter 12~N(0.000,5.000)	0.0000	5.0000	2.2361
Parameter 13~IW(1.000,7)	infinity	infinity	infinity
Parameter 14~IW(0.000,7)	infinity	infinity	infinity
Parameter 15~IW(1.000,7)	infinity	infinity	infinity
Parameter 16~IW(0.000,7)	infinity	infinity	infinity
Parameter 17~IW(0.000,7)	infinity	infinity	infinity
Parameter 18~IW(1.000,7)	infinity	infinity	infinity
Parameter 19~IW(0.000,7)	infinity	infinity	infinity
Parameter 20~IW(0.000,7)	infinity	infinity	infinity
Parameter 21~IW(0.000,7)	infinity	infinity	infinity
Parameter 22~IW(1.000,7)	infinity	infinity	infinity
Parameter 23~IW(0.000,7)	infinity	infinity	infinity
Parameter 24~IW(0.000,7)	infinity	infinity	infinity
Parameter 25~IW(0.000,7)	infinity	infinity	infinity
Parameter 26~IW(0.000,7)	infinity	infinity	infinity
Parameter 27~IW(1.000,7)	infinity	infinity	infinity
Parameter 28~IW(0.000,7)	infinity	infinity	infinity
Parameter 29~IW(0.000,7)	infinity	infinity	infinity
Parameter 30~IW(0.000,7)	infinity	infinity	infinity
Parameter 31~IW(0.000,7)	infinity	infinity	infinity
Parameter 32~IW(0.000,7)	infinity	infinity	infinity
Parameter 33~IW(1.000,7)	infinity	infinity	infinity
Parameter 34~N(0.000,infinity)	0.0000	infinity	infinity
Parameter 35~N(0.000,infinity)	0.0000	infinity	infinity
Parameter 36~N(0.000,5.000)	0.0000	5.0000	2.2361
Parameter 37~N(0.000,5.000)	0.0000	5.0000	2.2361
Parameter 38~IG(-1.000,0.000)	infinity	infinity	infinity
Parameter 39~IW(1.000,3)	infinity	infinity	infinity
Parameter 40~IW(0.000,3)	infinity	infinity	infinity
Parameter 41~IW(1.000,3)	infinity	infinity	infinity
Parameter 42~N(0.000,5.000)	0.0000	5.0000	2.2361

TECHNICAL 3 OUTPUT

ESTIMATED COVARIANCE MATRIX FOR PARAMETER ESTIMATES

	1	2	3	4	5
1	0.105262D-03				
2	0.522099D-04	0.442628D-03			
3	-0.262419D-04	-0.110192D-03	0.929481D-03		
4	0.322525D-05	0.104716D-03	-0.755626D-04	0.348455D-03	
5	0.635873D-06	0.156523D-03	-0.148962D-03	0.121344D-03	0.967276D-03
6	-0.227550D-04	-0.163706D-03	0.101547D-03	-0.990022D-04	-0.194197D-03
7	-0.473830D-04	-0.106192D-04	0.860037D-04	-0.114620D-03	0.440447D-04
8	0.883148D-05	-0.141897D-04	-0.397684D-04	-0.265663D-04	0.305252D-04
9	0.338668D-06	-0.868866D-05	-0.125421D-04	-0.374014D-05	0.116240D-04
10	0.137523D-04	-0.296978D-04	0.171386D-04	-0.880256D-05	-0.530262D-04
11	-0.826442D-05	-0.242502D-04	0.157286D-04	-0.465916D-05	-0.697701D-05
12	0.472652D-05	-0.147078D-04	0.460416D-04	-0.579648D-05	-0.106324D-04
13	-0.667530D-06	-0.223376D-05	0.696678D-06	-0.102916D-05	-0.391225D-05
14	-0.119208D-06	0.675570D-05	-0.115155D-04	0.536184D-05	-0.837803D-05
15	0.385787D-06	0.752307D-05	-0.386848D-05	-0.169163D-04	-0.620009D-04
16	-0.630384D-06	-0.520426D-05	0.147215D-05	-0.364536D-05	-0.864527D-05
17	-0.166895D-05	0.960727D-05	0.335476D-04	-0.675295D-05	0.119197D-04
18	0.125509D-04	0.304573D-05	0.132964D-04	0.370760D-04	0.489762D-04
19	-0.133879D-05	0.741788D-05	-0.119494D-05	0.889856D-05	0.977515D-05
20	-0.413096D-05	0.927670D-06	-0.215175D-04	0.531646D-06	-0.216484D-04
21	0.222737D-05	0.230024D-07	0.825189D-05	-0.403222D-05	-0.206894D-04
22	-0.117320D-05	-0.235379D-05	0.110312D-05	-0.140579D-05	-0.650264D-05
23	0.368566D-07	0.766144D-05	0.187407D-05	0.371081D-05	0.900057D-05
24	-0.200801D-06	-0.125305D-05	0.153906D-04	-0.647541D-05	-0.439211D-04
25	0.402973D-05	0.221260D-04	0.255007D-04	0.103903D-04	0.319160D-04
26	0.226490D-05	0.196939D-05	-0.218037D-04	0.140120D-04	-0.336102D-04
27	0.239432D-05	-0.139019D-04	0.262102D-04	0.105460D-04	-0.671932D-04
28	-0.828849D-06	0.303366D-05	-0.551657D-05	-0.186707D-05	0.164074D-05
29	-0.251799D-05	-0.624424D-05	-0.111665D-04	0.133501D-04	0.275866D-04
30	-0.407606D-05	-0.144474D-05	-0.107754D-04	0.644528D-05	0.102420D-04
31	0.385096D-05	0.485731D-05	0.638391D-05	0.268388D-05	0.150427D-04
32	0.352522D-05	-0.218272D-05	-0.170428D-04	-0.129129D-04	0.251570D-04
33	0.864598D-05	-0.117286D-04	0.911237D-05	-0.171027D-04	-0.238279D-04
34	-0.147325D-04	-0.782214D-05	0.162146D-04	0.264196D-04	0.327775D-04
35	0.116100D-04	-0.800991D-06	-0.130347D-04	-0.354020D-04	-0.833499D-05
36	-0.382444D-04	-0.306458D-04	0.113670D-04	-0.534542D-05	0.156327D-04
37	-0.271925D-05	0.301085D-05	-0.101750D-03	-0.952608D-04	0.329996D-04
38	-0.348012D-05	-0.359984D-05	0.583665D-05	0.303689D-05	0.769826D-05
39	-0.811066D-05	-0.133140D-04	0.575212D-04	0.182196D-04	-0.494844D-04
40	0.290374D-05	-0.659219D-05	-0.266997D-04	-0.197596D-04	0.687205D-05
41	-0.227541D-04	-0.162512D-05	-0.126322D-06	-0.222750D-05	0.581103D-05

42	-0.103715D-04	-0.777695D-05	-0.225545D-04	-0.434172D-04	-0.233159D-04

ESTIMATED COVARIANCE MATRIX FOR PARAMETER ESTIMATES

	6	7	8	9	10
6	0.732610D-03				
7	-0.416575D-04	0.131352D-01			
8	0.333638D-04	-0.601810D-03	0.264118D-02		
9	0.584974D-05	0.113485D-03	0.104799D-03	0.104991D-02	
10	0.275440D-04	-0.395860D-04	-0.298240D-03	0.194338D-03	0.311253D-02
11	-0.105327D-04	0.325892D-04	-0.221780D-03	0.936031D-04	-0.292622D-03
12	0.411934D-04	0.127188D-04	0.388605D-03	-0.116288D-03	0.313098D-03
13	0.130688D-05	0.288756D-04	-0.370168D-05	0.450656D-05	-0.497342D-05
14	0.854572D-05	-0.970948D-05	0.909446D-05	-0.332586D-05	-0.263917D-04
15	0.227027D-04	0.862508D-04	0.508199D-04	-0.370093D-04	-0.317446D-04
16	-0.397170D-05	-0.408811D-04	0.671065D-05	0.758014D-06	0.177444D-04
17	0.165907D-04	0.703443D-04	-0.465567D-04	0.833559D-05	0.623819D-04
18	0.860930D-05	-0.107706D-03	0.326083D-04	0.545779D-04	-0.487552D-04
19	-0.104889D-05	0.185832D-04	0.415247D-05	-0.110765D-05	0.639190D-05
20	0.108409D-04	-0.192009D-04	-0.269587D-05	0.754843D-06	0.342125D-04
21	0.135240D-04	0.178030D-04	-0.584506D-04	0.296171D-04	-0.278575D-04
22	0.643929D-05	0.184501D-04	0.292051D-04	-0.129381D-04	-0.314103D-04
23	0.237922D-05	0.198891D-04	0.226259D-04	0.577878D-05	0.976011D-05
24	0.177698D-04	0.286658D-04	0.153561D-04	-0.205340D-04	-0.717849D-05
25	-0.557422D-05	0.108849D-03	0.113543D-04	0.652516D-04	-0.626230D-04
26	-0.423460D-05	-0.617858D-04	0.328575D-04	-0.163965D-04	-0.233205D-04
27	0.275099D-04	-0.533628D-04	0.371963D-04	-0.323979D-04	-0.309568D-04
28	-0.150370D-04	0.250008D-04	-0.159558D-04	-0.542051D-05	-0.132027D-05
29	-0.962862D-05	0.697472D-04	-0.281458D-04	0.164996D-04	-0.298103D-04
30	0.158223D-04	-0.111784D-03	0.104109D-03	0.262871D-05	0.443154D-04
31	-0.144690D-04	0.772394D-04	-0.202786D-04	0.617595D-05	-0.216926D-04
32	-0.112092D-04	0.179038D-03	-0.250388D-04	0.312937D-04	-0.303517D-04
33	0.112155D-04	0.276182D-04	0.517607D-04	-0.101719D-04	0.181919D-04
34	0.326140D-04	-0.115772D-03	-0.224997D-04	-0.436560D-04	0.137634D-05
35	0.125378D-04	-0.140707D-04	-0.380870D-04	-0.244202D-04	0.127416D-03
36	-0.373112D-04	-0.706321D-03	0.232243D-03	0.137215D-03	0.520451D-04
37	0.301894D-04	-0.107102D-03	0.828094D-04	0.132892D-03	0.281374D-04
38	0.357938D-05	0.289684D-04	-0.790695D-06	-0.143817D-04	-0.873297D-06
39	0.279077D-04	0.152743D-03	-0.690305D-06	-0.337519D-05	-0.185418D-04
40	-0.115845D-04	-0.275550D-04	-0.997092D-05	0.153833D-04	-0.520365D-04
41	0.234466D-04	0.107975D-03	-0.327709D-04	-0.160956D-04	0.110195D-03
42	-0.643628D-06	-0.120470D-02	0.937982D-04	0.210510D-03	-0.801495D-04

ESTIMATED COVARIANCE MATRIX FOR PARAMETER ESTIMATES

	11	12	13	14	15
11	0.120007D-02				
12	0.199191D-03	0.144320D-02			
13	0.967296D-05	0.479133D-05	0.334098D-04		
14	0.238085D-05	-0.487018D-05	0.127730D-04	0.699864D-04	
15	0.129664D-04	0.113038D-04	0.233631D-05	0.637586D-04	0.513344D-03
16	-0.693712D-05	0.148725D-04	-0.738005D-05	-0.171912D-04	-0.242837D-04
17	-0.188319D-04	0.221222D-04	0.162191D-05	-0.161146D-04	-0.104965D-03
18	0.299912D-04	0.175785D-04	-0.133682D-05	-0.131544D-05	0.185195D-04
19	-0.599504D-05	-0.744187D-05	0.291034D-05	0.157631D-04	0.221027D-04
20	0.251703D-04	-0.284388D-05	0.508043D-06	0.752150D-05	0.103457D-03
21	0.189246D-04	-0.465523D-04	0.337874D-05	-0.258457D-05	-0.257098D-04
22	0.145121D-04	0.212089D-04	-0.455866D-08	0.741619D-06	0.267868D-04
23	-0.202310D-04	-0.670458D-05	0.267143D-05	0.214806D-04	0.283768D-04
24	0.664984D-05	-0.220640D-04	0.136601D-05	0.141315D-04	0.175213D-03
25	-0.105950D-04	0.150718D-04	0.266874D-05	0.180930D-05	-0.392724D-04
26	0.883777D-05	-0.508897D-05	0.403797D-05	-0.403811D-06	0.341716D-04
27	0.392927D-04	0.280575D-04	0.110914D-04	-0.179285D-05	0.258423D-05
28	0.149130D-04	0.913933D-05	0.121453D-05	-0.251590D-04	-0.206300D-04
29	-0.180296D-04	0.536992D-05	0.330982D-05	-0.138419D-04	-0.167877D-03
30	-0.536362D-04	0.139934D-04	-0.278197D-05	0.614801D-05	0.424993D-04
31	-0.561393D-05	-0.157006D-04	-0.487125D-06	0.783586D-05	-0.219792D-04
32	0.230333D-04	-0.132701D-04	-0.629055D-05	-0.412592D-05	-0.508391D-04
33	-0.247600D-04	0.252237D-04	0.254428D-05	-0.179706D-05	0.172148D-04
34	-0.339577D-04	-0.302631D-05	-0.896517D-05	-0.130133D-04	-0.117426D-04
35	-0.174033D-04	0.919791D-04	0.331713D-05	-0.109309D-04	-0.147262D-04
36	-0.135080D-03	0.590875D-05	0.737915D-06	0.393619D-06	-0.130353D-03
37	-0.264264D-03	0.884354D-04	0.470838D-05	-0.167648D-04	-0.137657D-03
38	0.153589D-04	0.156208D-04	-0.109148D-05	-0.112305D-05	-0.762715D-05
39	0.354721D-06	-0.113861D-04	0.476982D-05	-0.886923D-05	0.455608D-04
40	0.422895D-05	0.461113D-04	-0.101338D-05	0.658260D-05	-0.304989D-04
41	0.259212D-04	-0.670791D-04	-0.426031D-06	-0.496838D-05	0.103079D-04
42	-0.157349D-03	0.181059D-04	-0.250326D-05	-0.564762D-06	-0.942640D-04

ESTIMATED COVARIANCE MATRIX FOR PARAMETER ESTIMATES

	16	17	18	19	20
16	0.141546D-03				
17	0.782531D-04	0.576807D-03			
18	-0.662612D-04	-0.227266D-03	0.233904D-02		

19	-0.161769D-04	-0.115753D-04	0.905695D-05	0.542308D-04	
20	-0.163592D-04	-0.733395D-04	0.142766D-04	0.276678D-04	0.226623D-03
21	0.155231D-04	0.125424D-03	-0.193905D-03	-0.123377D-04	-0.619677D-04
22	-0.328587D-05	-0.296149D-04	-0.374699D-05	0.782425D-05	0.902621D-04
23	-0.166842D-04	-0.843030D-05	0.540976D-05	0.199138D-04	0.844172D-05
24	-0.580184D-06	-0.104475D-03	0.292537D-05	0.556211D-05	0.989620D-04
25	0.261122D-04	0.206565D-03	-0.346644D-03	0.627955D-05	-0.646066D-04
26	0.534902D-05	-0.312907D-04	-0.144789D-04	0.107005D-05	0.931959D-04
27	-0.161632D-04	-0.519570D-04	0.397874D-04	0.193734D-05	0.669769D-04
28	0.145867D-04	0.139803D-04	0.138956D-04	-0.172224D-04	-0.123211D-04
29	0.154126D-04	0.922912D-04	0.477028D-05	-0.111185D-04	-0.841011D-04
30	-0.135083D-04	-0.170872D-03	0.262874D-03	0.866959D-05	0.392426D-04
31	0.101218D-04	0.398435D-04	-0.882048D-05	-0.432072D-05	-0.925204D-04
32	0.250584D-05	0.653101D-04	-0.563045D-04	0.789109D-05	-0.407126D-04
33	-0.130643D-04	-0.872268D-04	0.751266D-04	0.234806D-05	0.311233D-04
34	-0.237344D-04	0.178461D-04	0.828291D-04	0.102473D-04	-0.113776D-04
35	0.466308D-04	-0.162373D-04	-0.111989D-03	0.568362D-05	0.143684D-04
36	-0.465245D-05	-0.612658D-04	0.254975D-03	-0.192414D-04	-0.215994D-04
37	0.505146D-05	-0.480326D-04	0.215668D-03	-0.199700D-04	0.122847D-04
38	0.618158D-06	0.260227D-05	0.129748D-04	-0.464292D-06	-0.967182D-06
39	-0.361021D-05	0.125318D-04	-0.883618D-04	-0.158869D-04	-0.260618D-05
40	0.109284D-05	-0.104016D-04	0.788431D-04	0.167096D-04	0.567483D-05
41	-0.105937D-05	0.416842D-04	-0.139881D-03	-0.253515D-05	-0.999231D-05
42	0.365565D-05	-0.556599D-04	0.176514D-03	-0.278726D-04	-0.403227D-05

ESTIMATED COVARIANCE MATRIX FOR PARAMETER ESTIMATES

	21	22	23	24	25
21	0.456523D-03				
22	-0.105292D-03	0.326154D-03			
23	0.109324D-05	0.167885D-05	0.132919D-03		
24	-0.289039D-04	0.333685D-04	0.555626D-04	0.586208D-03	
25	0.175293D-03	-0.354710D-04	-0.292478D-04	-0.124285D-03	0.108530D-02
26	-0.728277D-04	0.124673D-03	0.558426D-05	0.126734D-03	-0.126802D-03
27	-0.585220D-04	0.272959D-04	0.241503D-04	0.351976D-03	-0.280893D-03
28	-0.266629D-05	-0.862837D-05	-0.255695D-04	-0.115745D-04	-0.216264D-05
29	0.288361D-04	-0.319028D-04	-0.112125D-04	-0.142992D-03	0.502475D-04
30	-0.132951D-03	0.425921D-04	0.826911D-05	0.553672D-04	-0.245986D-03
31	0.914036D-04	-0.100629D-03	-0.130577D-05	-0.558501D-04	0.754557D-04
32	0.201080D-04	-0.388019D-04	-0.127838D-04	-0.195939D-03	0.196348D-03
33	-0.428002D-04	0.485785D-04	-0.509376D-06	0.463466D-04	-0.521639D-04
34	0.259470D-04	-0.391854D-05	-0.536588D-06	-0.139991D-04	-0.326988D-04
35	-0.169700D-04	0.280870D-04	-0.175560D-06	0.397501D-05	0.585080D-04

36	-0.641270D-04	0.198649D-04	-0.259264D-04	-0.157051D-03	-0.204652D-04
37	-0.149579D-04	-0.277332D-04	-0.202721D-04	-0.837269D-04	-0.273885D-04
38	0.228070D-05	-0.176800D-05	0.216808D-05	-0.101603D-04	0.106443D-04
39	0.315767D-04	0.170188D-04	0.229160D-04	0.264110D-04	0.558869D-04
40	-0.307242D-05	-0.373294D-05	-0.196256D-04	-0.130527D-04	-0.246651D-04
41	0.129541D-04	0.128897D-05	0.130237D-04	-0.469624D-05	0.288154D-04
42	-0.566860D-04	0.552613D-05	-0.248855D-04	-0.585751D-04	-0.338371D-05

ESTIMATED COVARIANCE MATRIX FOR PARAMETER ESTIMATES

	26	27	28	29	30
26	0.418034D-03				
27	0.288601D-03	0.203282D-02			
28	-0.112653D-04	0.400952D-05	0.110115D-03		
29	-0.381555D-04	-0.786376D-04	0.554706D-04	0.448356D-03	
30	0.411276D-04	0.131701D-04	-0.328641D-04	-0.117504D-03	0.848391D-03
31	-0.952302D-04	-0.566817D-04	0.583598D-05	0.107277D-03	-0.122335D-03
32	-0.137263D-03	-0.367233D-03	0.113036D-04	0.179865D-03	-0.192958D-03
33	0.464803D-04	0.835150D-04	-0.199160D-04	-0.305554D-03	0.253325D-03
34	0.194053D-04	0.683297D-04	0.152880D-04	0.576830D-04	-0.820378D-04
35	-0.825007D-06	-0.794450D-04	-0.257463D-04	-0.355240D-04	0.598554D-04
36	0.128914D-04	-0.214455D-03	0.212137D-04	0.115086D-03	0.264782D-04
37	0.201118D-05	-0.254526D-03	0.130683D-05	0.972030D-04	0.627520D-04
38	-0.143618D-04	-0.140415D-04	-0.225480D-05	0.460860D-05	-0.117291D-04
39	0.311069D-04	-0.635273D-04	-0.222031D-04	-0.893470D-06	-0.538331D-04
40	-0.205049D-04	0.546583D-04	0.590527D-05	-0.111369D-04	0.454946D-04
41	0.478959D-05	0.840974D-05	-0.442249D-05	0.708294D-05	-0.487707D-04
42	0.574060D-05	-0.145426D-03	0.155732D-04	0.461919D-04	0.607025D-04

ESTIMATED COVARIANCE MATRIX FOR PARAMETER ESTIMATES

	31	32	33	34	35
31	0.365390D-03				
32	0.162409D-03	0.917620D-03			
33	-0.191993D-03	-0.354666D-03	0.145035D-02		
34	0.242642D-05	0.480017D-04	-0.148708D-03	0.407087D-02	
35	-0.157937D-04	-0.640317D-04	0.214188D-04	-0.234014D-02	0.479336D-02
36	-0.387683D-04	-0.146826D-06	-0.921447D-04	0.149312D-03	-0.189911D-03
37	-0.535372D-04	-0.153994D-03	-0.102357D-03	0.183770D-03	-0.127142D-03
38	0.652295D-05	0.123827D-04	-0.416541D-05	-0.155990D-04	0.338645D-04
39	-0.233512D-04	-0.160226D-05	0.327527D-04	0.757971D-05	0.348131D-04
40	0.915615D-05	0.269809D-04	-0.155887D-04	-0.402497D-04	0.334928D-04

41	-0.170862D-04	0.128027D-04	0.584113D-04	-0.138550D-05	0.634848D-05
42	-0.588780D-04	-0.825874D-04	-0.442122D-04	0.128807D-03	-0.897964D-04

ESTIMATED COVARIANCE MATRIX FOR PARAMETER ESTIMATES

	36	37	38	39	40
36	0.136548D-01				
37	0.885535D-02	0.125403D-01			
38	-0.489292D-04	0.456647D-04	0.113248D-03		
39	0.215782D-03	0.232124D-05	-0.270945D-05	0.198931D-02	
40	-0.158874D-03	-0.258958D-04	-0.511132D-05	-0.115270D-02	0.144324D-02
41	0.252664D-03	0.141571D-03	0.663934D-05	0.635372D-03	-0.128713D-02
42	0.900914D-02	0.910591D-02	-0.122099D-04	0.181665D-04	-0.905200D-06

ESTIMATED COVARIANCE MATRIX FOR PARAMETER ESTIMATES

	41	42
41	0.250501D-02	
42	0.282926D-04	0.920400D-02

ESTIMATED CORRELATION MATRIX FOR PARAMETER ESTIMATES

	1	2	3	4	5
1	1.000				
2	0.242	1.000			
3	-0.084	-0.172	1.000		
4	0.017	0.267	-0.133	1.000	
5	0.002	0.239	-0.157	0.209	1.000
6	-0.082	-0.287	0.123	-0.196	-0.231
7	-0.040	-0.004	0.025	-0.054	0.012
8	0.017	-0.013	-0.025	-0.028	0.019
9	0.001	-0.013	-0.013	-0.006	0.012
10	0.024	-0.025	0.010	-0.008	-0.031
11	-0.023	-0.033	0.015	-0.007	-0.006
12	0.012	-0.018	0.040	-0.008	-0.009
13	-0.011	-0.018	0.004	-0.010	-0.022
14	-0.001	0.038	-0.045	0.034	-0.032
15	0.002	0.016	-0.006	-0.040	-0.088
16	-0.005	-0.021	0.004	-0.016	-0.023
17	-0.007	0.019	0.046	-0.015	0.016
18	0.025	0.003	0.009	0.041	0.033

19	-0.018	0.048	-0.005	0.065	0.043
20	-0.027	0.003	-0.047	0.002	-0.046
21	0.010	0.000	0.013	-0.010	-0.031
22	-0.006	-0.006	0.002	-0.004	-0.012
23	0.000	0.032	0.005	0.017	0.025
24	-0.001	-0.002	0.021	-0.014	-0.058
25	0.012	0.032	0.025	0.017	0.031
26	0.011	0.005	-0.035	0.037	-0.053
27	0.005	-0.015	0.019	0.013	-0.048
28	-0.008	0.014	-0.017	-0.010	0.005
29	-0.012	-0.014	-0.017	0.034	0.042
30	-0.014	-0.002	-0.012	0.012	0.011
31	0.020	0.012	0.011	0.008	0.025
32	0.011	-0.003	-0.018	-0.023	0.027
33	0.022	-0.015	0.008	-0.024	-0.020
34	-0.023	-0.006	0.008	0.022	0.017
35	0.016	-0.001	-0.006	-0.027	-0.004
36	-0.032	-0.012	0.003	-0.002	0.004
37	-0.002	0.001	-0.030	-0.046	0.009
38	-0.032	-0.016	0.018	0.015	0.023
39	-0.018	-0.014	0.042	0.022	-0.036
40	0.007	-0.008	-0.023	-0.028	0.006
41	-0.044	-0.002	0.000	-0.002	0.004
42	-0.011	-0.004	-0.008	-0.024	-0.008

ESTIMATED CORRELATION MATRIX FOR PARAMETER ESTIMATES

	6	7	8	9	10
6	1.000				
7	-0.013	1.000			
8	0.024	-0.102	1.000		
9	0.007	0.031	0.063	1.000	
10	0.018	-0.006	-0.104	0.108	1.000
11	-0.011	0.008	-0.125	0.083	-0.151
12	0.040	0.003	0.199	-0.094	0.148
13	0.008	0.044	-0.012	0.024	-0.015
14	0.038	-0.010	0.021	-0.012	-0.057
15	0.037	0.033	0.044	-0.050	-0.025
16	-0.012	-0.030	0.011	0.002	0.027
17	0.026	0.026	-0.038	0.011	0.047
18	0.007	-0.019	0.013	0.035	-0.018
19	-0.005	0.022	0.011	-0.005	0.016
20	0.027	-0.011	-0.003	0.002	0.041

21	0.023	0.007	-0.053	0.043	-0.023
22	0.013	0.009	0.031	-0.022	-0.031
23	0.008	0.015	0.038	0.015	0.015
24	0.027	0.010	0.012	-0.026	-0.005
25	-0.006	0.029	0.007	0.061	-0.034
26	-0.008	-0.026	0.031	-0.025	-0.020
27	0.023	-0.010	0.016	-0.022	-0.012
28	-0.053	0.021	-0.030	-0.016	-0.002
29	-0.017	0.029	-0.026	0.024	-0.025
30	0.020	-0.033	0.070	0.003	0.027
31	-0.028	0.035	-0.021	0.010	-0.020
32	-0.014	0.052	-0.016	0.032	-0.018
33	0.011	0.006	0.026	-0.008	0.009
34	0.019	-0.016	-0.007	-0.021	0.000
35	0.007	-0.002	-0.011	-0.011	0.033
36	-0.012	-0.053	0.039	0.036	0.008
37	0.010	-0.008	0.014	0.037	0.005
38	0.012	0.024	-0.001	-0.042	-0.001
39	0.023	0.030	0.000	-0.002	-0.007
40	-0.011	-0.006	-0.005	0.012	-0.025
41	0.017	0.019	-0.013	-0.010	0.039
42	0.000	-0.110	0.019	0.068	-0.015

ESTIMATED CORRELATION MATRIX FOR PARAMETER ESTIMATES

	11	12	13	14	15
11	1.000				
12	0.151	1.000			
13	0.048	0.022	1.000		
14	0.008	-0.015	0.264	1.000	
15	0.017	0.013	0.018	0.336	1.000
16	-0.017	0.033	-0.107	-0.173	-0.090
17	-0.023	0.024	0.012	-0.080	-0.193
18	0.018	0.010	-0.005	-0.003	0.017
19	-0.023	-0.027	0.068	0.256	0.132
20	0.048	-0.005	0.006	0.060	0.303
21	0.026	-0.057	0.027	-0.014	-0.053
22	0.023	0.031	0.000	0.005	0.065
23	-0.051	-0.015	0.040	0.223	0.109
24	0.008	-0.024	0.010	0.070	0.319
25	-0.009	0.012	0.014	0.007	-0.053
26	0.012	-0.007	0.034	-0.002	0.074
27	0.025	0.016	0.043	-0.005	0.003

28	0.041	0.023	0.020	-0.287	-0.087
29	-0.025	0.007	0.027	-0.078	-0.350
30	-0.053	0.013	-0.017	0.025	0.064
31	-0.008	-0.022	-0.004	0.049	-0.051
32	0.022	-0.012	-0.036	-0.016	-0.074
33	-0.019	0.017	0.012	-0.006	0.020
34	-0.015	-0.001	-0.024	-0.024	-0.008
35	-0.007	0.035	0.008	-0.019	-0.009
36	-0.033	0.001	0.001	0.000	-0.049
37	-0.068	0.021	0.007	-0.018	-0.054
38	0.042	0.039	-0.018	-0.013	-0.032
39	0.000	-0.007	0.019	-0.024	0.045
40	0.003	0.032	-0.005	0.021	-0.035
41	0.015	-0.035	-0.001	-0.012	0.009
42	-0.047	0.005	-0.005	-0.001	-0.043

ESTIMATED CORRELATION MATRIX FOR PARAMETER ESTIMATES

	16	17	18	19	20
16	1.000				
17	0.274	1.000			
18	-0.115	-0.196	1.000		
19	-0.185	-0.065	0.025	1.000	
20	-0.091	-0.203	0.020	0.250	1.000
21	0.061	0.244	-0.188	-0.078	-0.193
22	-0.015	-0.068	-0.004	0.059	0.332
23	-0.122	-0.030	0.010	0.235	0.049
24	-0.002	-0.180	0.002	0.031	0.272
25	0.067	0.261	-0.218	0.026	-0.130
26	0.022	-0.064	-0.015	0.007	0.303
27	-0.030	-0.048	0.018	0.006	0.099
28	0.117	0.055	0.027	-0.223	-0.078
29	0.061	0.181	0.005	-0.071	-0.264
30	-0.039	-0.244	0.187	0.040	0.089
31	0.045	0.087	-0.010	-0.031	-0.322
32	0.007	0.090	-0.038	0.035	-0.089
33	-0.029	-0.095	0.041	0.008	0.054
34	-0.031	0.012	0.027	0.022	-0.012
35	0.057	-0.010	-0.033	0.011	0.014
36	-0.003	-0.022	0.045	-0.022	-0.012
37	0.004	-0.018	0.040	-0.024	0.007
38	0.005	0.010	0.025	-0.006	-0.006
39	-0.007	0.012	-0.041	-0.048	-0.004

40	0.002	-0.011	0.043	0.060	0.010
41	-0.002	0.035	-0.058	-0.007	-0.013
42	0.003	-0.024	0.038	-0.039	-0.003

ESTIMATED CORRELATION MATRIX FOR PARAMETER ESTIMATES

	21	22	23	24	25
21	1.000				
22	-0.273	1.000			
23	0.004	0.008	1.000		
24	-0.056	0.076	0.199	1.000	
25	0.249	-0.060	-0.077	-0.156	1.000
26	-0.167	0.338	0.024	0.256	-0.188
27	-0.061	0.034	0.046	0.322	-0.189
28	-0.012	-0.046	-0.211	-0.046	-0.006
29	0.064	-0.083	-0.046	-0.279	0.072
30	-0.214	0.081	0.025	0.079	-0.256
31	0.224	-0.291	-0.006	-0.121	0.120
32	0.031	-0.071	-0.037	-0.267	0.197
33	-0.053	0.071	-0.001	0.050	-0.042
34	0.019	-0.003	-0.001	-0.009	-0.016
35	-0.011	0.022	0.000	0.002	0.026
36	-0.026	0.009	-0.019	-0.056	-0.005
37	-0.006	-0.014	-0.016	-0.031	-0.007
38	0.010	-0.009	0.018	-0.039	0.030
39	0.033	0.021	0.045	0.024	0.038
40	-0.004	-0.005	-0.045	-0.014	-0.020
41	0.012	0.001	0.023	-0.004	0.017
42	-0.028	0.003	-0.022	-0.025	-0.001

ESTIMATED CORRELATION MATRIX FOR PARAMETER ESTIMATES

	26	27	28	29	30
26	1.000				
27	0.313	1.000			
28	-0.053	0.008	1.000		
29	-0.088	-0.082	0.250	1.000	
30	0.069	0.010	-0.108	-0.191	1.000
31	-0.244	-0.066	0.029	0.265	-0.220
32	-0.222	-0.269	0.036	0.280	-0.219
33	0.060	0.049	-0.050	-0.379	0.228
34	0.015	0.024	0.023	0.043	-0.044

35	-0.001	-0.025	-0.035	-0.024	0.030
36	0.005	-0.041	0.017	0.047	0.008
37	0.001	-0.050	0.001	0.041	0.019
38	-0.066	-0.029	-0.020	0.020	-0.038
39	0.034	-0.032	-0.047	-0.001	-0.041
40	-0.026	0.032	0.015	-0.014	0.041
41	0.005	0.004	-0.008	0.007	-0.033
42	0.003	-0.034	0.015	0.023	0.022

ESTIMATED CORRELATION MATRIX FOR PARAMETER ESTIMATES

	31	32	33	34	35
31	1.000				
32	0.280	1.000			
33	-0.264	-0.307	1.000		
34	0.002	0.025	-0.061	1.000	
35	-0.012	-0.031	0.008	-0.530	1.000
36	-0.017	0.000	-0.021	0.020	-0.023
37	-0.025	-0.045	-0.024	0.026	-0.016
38	0.032	0.038	-0.010	-0.023	0.046
39	-0.027	-0.001	0.019	0.003	0.011
40	0.013	0.023	-0.011	-0.017	0.013
41	-0.018	0.008	0.031	0.000	0.002
42	-0.032	-0.028	-0.012	0.021	-0.014

ESTIMATED CORRELATION MATRIX FOR PARAMETER ESTIMATES

	36	37	38	39	40
36	1.000				
37	0.677	1.000			
38	-0.039	0.038	1.000		
39	0.041	0.000	-0.006	1.000	
40	-0.036	-0.006	-0.013	-0.680	1.000
41	0.043	0.025	0.012	0.285	-0.677
42	0.804	0.848	-0.012	0.004	0.000

ESTIMATED CORRELATION MATRIX FOR PARAMETER ESTIMATES

	41	42
41	1.000	
42	0.006	1.000

TECHNICAL 8 OUTPUT

TECHNICAL 8 OUTPUT FOR BAYES ESTIMATION

CHAIN	BSEED
1	0
2	285380
3	253358

ITERATION	POTENTIAL SCALE REDUCTION	PARAMETER WITH HIGHEST PSR
100	1.022	32
200	1.012	36
300	1.007	36
400	1.006	28
500	1.008	42
600	1.006	37
700	1.003	42
800	1.001	27
900	1.002	42
1000	1.002	38

```
This is worsening of psychological distress

SUMMARY OF ANALYSIS

Number of groups                                                 1
Number of observations                                        5855

Number of dependent variables                                    2
Number of independent variables                                  8
Number of continuous latent variables                            0

Observed dependent variables

  Continuous
   PRAFACSQ

  Binary and ordered categorical (ordinal)
   KESCAT16

Observed independent variables
   PHY15       KES15       CLU2        CLU3        CHOICE15    BULLY15
   PRESS15     DUTIES15

Variables with special functions

  Cluster variable      CID15

  Within variables
   PHY15       KES15       CHOICE15    BULLY15     PRESS15     DUTIES15

  Between variables
   CLU2        CLU3

  Centering (GRANDMEAN)
   PHY15       KES15       CHOICE15    BULLY15     PRESS15     DUTIES15


Estimator                                                    BAYES
Specifications for Bayesian Estimation
  Point estimate                                            MEDIAN
  Number of Markov chain Monte Carlo (MCMC) chains               3
  Random seed for the first chain                                0
  Starting value information                           UNPERTURBED
  Algorithm used for Markov chain Monte Carlo           GIBBS(PX1)
```

```
  Convergence criterion                                     0.100D-01
  Maximum number of iterations                                  50000
  K-th iteration used for thinning                                 20
Link                                                           PROBIT

Input data file(s)
  multicomponent for mplus cat.dat
Input data format  FREE


SUMMARY OF DATA

     Number of clusters                         58

       Size (s)    Cluster ID with Size s

          7        22
         17        166
         20        80
         26        71 74 205
         29        65
         31        192
         33        228
         37        182 106
         39        72
         41        170 181
         42        50 30
         43        75
         45        173
         47        226 165
         48        108
         50        107 114
         51        81
         52        40
         55        84 92
         57        162
         59        206
         61        61 83 116
         62        36 93 169
         63        69
         64        4
         69        68
         73        174 152
         74        6 154
```

75	145
89	98
90	178 2
95	144
100	156
104	202
106	142
134	122
186	24
192	209
246	204
278	125
405	217
780	110
873	100

COVARIANCE COVERAGE OF DATA

Minimum covariance coverage value 0.100

Number of missing data patterns 9

PROPORTION OF DATA PRESENT

Covariance Coverage

	KESCAT16	CLU2	CLU3	PHY15	KES15
KESCAT16	1.000				
CLU2	1.000	1.000			
CLU3	1.000	1.000	1.000		
PHY15	0.950	0.950	0.950	0.950	
KES15	0.919	0.919	0.919	0.919	0.919
CHOICE15	0.913	0.913	0.913	0.913	0.913
BULLY15	0.913	0.913	0.913	0.913	0.913
PRESS15	0.913	0.913	0.913	0.913	0.913
DUTIES15	0.913	0.913	0.913	0.913	0.913
PRAFACSQ	1.000	1.000	1.000	0.950	0.919

Covariance Coverage

	CHOICE15	BULLY15	PRESS15	DUTIES15	PRAFACSQ
CHOICE15	0.913				
BULLY15	0.912	0.913			
PRESS15	0.913	0.912	0.913		
DUTIES15	0.913	0.913	0.913	0.913	
PRAFACSQ	0.913	0.913	0.913	0.913	1.000

UNIVARIATE PROPORTIONS AND COUNTS FOR CATEGORICAL VARIABLES

KESCAT16		
Category 1	0.877	5135.000
Category 2	0.123	720.000

UNIVARIATE SAMPLE STATISTICS

UNIVARIATE HIGHER-ORDER MOMENT DESCRIPTIVE STATISTICS

Variable/ Sample Size	Mean/ Variance	Skewness/ Kurtosis	Minimum/ Maximum	% with Min/Max	Percentiles 20%/60%	Percentiles 40%/80%	Median
CLU2	0.310	0.820	0.000	68.97%	0.000	0.000	0.000
58.000	0.214	-1.328	1.000	31.03%	0.000	1.000	
CLU3	0.448	0.208	0.000	55.17%	0.000	0.000	0.000
58.000	0.247	-1.957	1.000	44.83%	1.000	1.000	
PHY15	0.000	0.526	-0.865	32.48%	-0.865	0.135	0.135
5563.000	0.536	0.021	3.135	0.07%	0.135	0.135	
KES15	0.000	0.475	-0.374	22.78%	-0.374	-0.207	-0.041
5382.000	0.095	-0.842	0.626	6.60%	-0.041	0.293	
CHOICE15	0.000	-0.400	-2.221	7.67%	-1.221	-0.221	-0.221
5345.000	1.097	-0.356	1.779	8.62%	0.779	0.779	
BULLY15	0.000	3.522	-0.153	88.29%	-0.153	-0.153	-0.153
5344.000	0.216	14.164	3.847	0.09%	-0.153	-0.153	
PRESS15	0.000	0.267	-1.445	19.70%	-0.445	-0.445	-0.445
5345.000	1.001	-0.357	2.555	2.75%	0.555	0.555	
DUTIES15	0.000	-1.296	-3.149	2.34%	-0.149	-0.149	-0.149
5347.000	0.828	1.939	0.851	39.74%	-0.149	0.851	
PRAFACSQ	1.011	2.017	0.002	0.56%	0.213	0.337	0.469
5855.000	1.509	3.818	5.467	1.09%	0.932	1.597	

THE MODEL ESTIMATION TERMINATED NORMALLY

USE THE FBITERATIONS OPTION TO INCREASE THE NUMBER OF ITERATIONS BY A FACTOR
OF AT LEAST TWO TO CHECK CONVERGENCE AND THAT THE PSR VALUE DOES NOT INCREASE.

MODEL FIT INFORMATION

Number of Free Parameters 45

Bayesian Posterior Predictive Checking using Chi-Square

95% Confidence Interval for the Difference Between
the Observed and the Replicated Chi-Square Values

27.954 628.825

Posterior Predictive P-Value 0.000

MODEL RESULTS

	Estimate	Posterior S.D.	One-Tailed P-Value	95% C.I. Lower 2.5%	95% C.I. Upper 2.5%	Significance
Within Level						
KESCAT16 ON						
PHY15	0.136	0.033	0.000	0.072	0.198	*
CHOICE15	-0.058	0.024	0.007	-0.106	-0.011	*
BULLY15	0.108	0.048	0.015	0.013	0.200	*
DUTIES15	-0.082	0.027	0.003	-0.133	-0.028	*
PRESS15	-0.034	0.026	0.106	-0.083	0.017	
KES15	1.616	0.084	0.000	1.457	1.780	*
KES15 WITH						
PHY15	0.054	0.003	0.000	0.048	0.061	*
CHOICE15	-0.037	0.004	0.000	-0.045	-0.028	*
BULLY15	0.022	0.002	0.000	0.018	0.026	*
PRESS15	0.053	0.004	0.000	0.045	0.061	*
DUTIES15	-0.051	0.004	0.000	-0.058	-0.043	*

```
 PHY15    WITH
    CHOICE15          -0.092       0.011      0.000      -0.114      -0.071      *
    BULLY15            0.024       0.005      0.000       0.016       0.033      *
    PRESS15            0.063       0.010      0.000       0.043       0.084      *
    DUTIES15          -0.056       0.009      0.000      -0.074      -0.039      *

 CHOICE15 WITH
    BULLY15           -0.052       0.007      0.000      -0.065      -0.038      *
    PRESS15           -0.078       0.014      0.000      -0.106      -0.049      *
    DUTIES15           0.159       0.013      0.000       0.133       0.184      *

 BULLY15  WITH
    PRESS15            0.090       0.006      0.000       0.078       0.103      *
    DUTIES15          -0.059       0.006      0.000      -0.070      -0.047      *

 PRESS15  WITH
    DUTIES15          -0.156       0.012      0.000      -0.180      -0.132      *

 Means
    PHY15              0.002       0.010      0.422      -0.017       0.021
    KES15              0.003       0.004      0.221      -0.005       0.011
    CHOICE15          -0.003       0.014      0.416      -0.032       0.025
    BULLY15            0.001       0.006      0.427      -0.011       0.013
    PRESS15            0.002       0.014      0.444      -0.025       0.030
    DUTIES15          -0.003       0.012      0.405      -0.028       0.020

 Variances
    PHY15              0.536       0.010      0.000       0.517       0.556      *
    KES15              0.096       0.002      0.000       0.092       0.100      *
    CHOICE15           1.099       0.021      0.000       1.057       1.141      *
    BULLY15            0.216       0.004      0.000       0.208       0.224      *
    PRESS15            1.002       0.019      0.000       0.966       1.039      *
    DUTIES15           0.829       0.016      0.000       0.799       0.862      *
    PRAFACSQ           0.000       0.000      0.000       0.000       0.000      *

Between Level

 KESCAT16   ON
    CLU2               0.036       0.091      0.344      -0.127       0.241
    CLU3               0.040       0.086      0.311      -0.115       0.219

 CLU2     WITH
    CLU3              -0.138       0.038      0.000      -0.224      -0.077      *
```

	Estimate	Posterior S.D.	One-Tailed P-Value	95% C.I. Lower 2.5%	Upper 2.5%	Significance
Means						
CLU2	0.310	0.063	0.000	0.194	0.436	*
CLU3	0.447	0.070	0.000	0.313	0.589	*
PRAFACSQ	1.384	0.211	0.000	0.962	1.826	*
Thresholds						
KESCAT16$1	1.389	0.076	0.000	1.254	1.549	*
Variances						
CLU2	0.231	0.044	0.000	0.163	0.337	*
CLU3	0.266	0.051	0.000	0.187	0.378	*
PRAFACSQ	2.504	0.510	0.000	1.760	3.740	*
Residual Variances						
KESCAT16	0.010	0.008	0.000	0.001	0.033	*

STANDARDIZED MODEL RESULTS

STDYX Standardization

	Estimate	Posterior S.D.	One-Tailed P-Value	95% C.I. Lower 2.5%	Upper 2.5%	Significance
Within Level						
KESCAT16 ON						
PHY15	0.086	0.021	0.000	0.045	0.126	*
CHOICE15	-0.053	0.022	0.007	-0.096	-0.010	*
BULLY15	0.044	0.019	0.015	0.005	0.080	*
DUTIES15	-0.065	0.022	0.003	-0.105	-0.022	*
PRESS15	-0.029	0.023	0.106	-0.072	0.015	
KES15	0.435	0.019	0.000	0.398	0.472	*
KES15 WITH						
PHY15	0.240	0.013	0.000	0.215	0.267	*
CHOICE15	-0.113	0.013	0.000	-0.139	-0.087	*
BULLY15	0.154	0.013	0.000	0.127	0.181	*
PRESS15	0.171	0.013	0.000	0.145	0.197	*
DUTIES15	-0.180	0.013	0.000	-0.205	-0.155	*
PHY15 WITH						
CHOICE15	-0.120	0.014	0.000	-0.148	-0.094	*

BULLY15	0.072	0.013	0.000	0.047	0.098	*
PRESS15	0.086	0.014	0.000	0.059	0.115	*
DUTIES15	-0.084	0.013	0.000	-0.111	-0.059	*
CHOICE15 WITH						
BULLY15	-0.106	0.013	0.000	-0.133	-0.080	*
PRESS15	-0.074	0.014	0.000	-0.099	-0.047	*
DUTIES15	0.167	0.013	0.000	0.140	0.192	*
BULLY15 WITH						
PRESS15	0.194	0.013	0.000	0.170	0.221	*
DUTIES15	-0.139	0.014	0.000	-0.165	-0.112	*
PRESS15 WITH						
DUTIES15	-0.171	0.013	0.000	-0.196	-0.146	*
Means						
PHY15	0.003	0.013	0.422	-0.024	0.028	
KES15	0.010	0.013	0.221	-0.015	0.035	
CHOICE15	-0.003	0.014	0.416	-0.030	0.024	
BULLY15	0.003	0.013	0.427	-0.023	0.028	
PRESS15	0.002	0.014	0.444	-0.025	0.030	
DUTIES15	-0.003	0.013	0.405	-0.030	0.022	
Variances						
PHY15	1.000	0.000	0.000	1.000	1.000	
KES15	1.000	0.000	0.000	1.000	1.000	
CHOICE15	1.000	0.000	0.000	1.000	1.000	
BULLY15	1.000	0.000	0.000	1.000	1.000	
PRESS15	1.000	0.000	0.000	1.000	1.000	
DUTIES15	1.000	0.000	0.000	1.000	1.000	
PRAFACSQ	1.000	0.000	0.000	1.000	1.000	

Between Level

KESCAT16 ON						
CLU2	0.168	0.384	0.344	-0.649	0.831	
CLU3	0.206	0.381	0.311	-0.589	0.906	
CLU2 WITH						
CLU3	-0.568	0.090	0.000	-0.712	-0.367	*
Means						
CLU2	0.649	0.142	0.000	0.376	0.931	*

CLU3	0.873	0.153	0.000	0.578	1.174	*
PRAFACSQ	0.874	0.156	0.000	0.562	1.176	*
**Thresholds**						
KESCAT16$1	12.490	5.576	0.000	7.254	28.219	*
**Variances**						
CLU2	1.000	0.000	0.000	1.000	1.000	
CLU3	1.000	0.000	0.000	1.000	1.000	
PRAFACSQ	1.000	0.000	0.000	1.000	1.000	
**Residual Variances**						
KESCAT16	0.842	0.183	0.000	0.311	0.995	*

R-SQUARE

Within Level

Variable	Estimate	Posterior S.D.	One-Tailed P-Value	95% C.I. Lower 2.5%	95% C.I. Upper 2.5%
KESCAT16	0.246	0.017	0.000	0.212	0.279

Between Level

Variable	Estimate	Posterior S.D.	One-Tailed P-Value	95% C.I. Lower 2.5%	95% C.I. Upper 2.5%
KESCAT16	0.158	0.183	0.000	0.005	0.689

TECHNICAL 1 OUTPUT

PARAMETER SPECIFICATION FOR WITHIN

TAU

KESCAT16
0

NU

KESCAT16	PHY15	KES15	CHOICE15	BULLY15
0	0	0	0	0

NU

PRESS15	DUTIES15	PRAFACSQ
0	0	0

LAMBDA

	KESCAT16	PHY15	KES15	CHOICE15	BULLY15
KESCAT16	0	0	0	0	0
PHY15	0	0	0	0	0
KES15	0	0	0	0	0
CHOICE15	0	0	0	0	0
BULLY15	0	0	0	0	0
PRESS15	0	0	0	0	0
DUTIES15	0	0	0	0	0
PRAFACSQ	0	0	0	0	0

LAMBDA

	PRESS15	DUTIES15
KESCAT16	0	0
PHY15	0	0
KES15	0	0
CHOICE15	0	0
BULLY15	0	0
PRESS15	0	0
DUTIES15	0	0
PRAFACSQ	0	0

THETA

	KESCAT16	PHY15	KES15	CHOICE15	BULLY15
KESCAT16	0				
PHY15	0	0			
KES15	0	0	0		

CHOICE15	0	0	0	0	
BULLY15	0	0	0	0	0
PRESS15	0	0	0	0	0
DUTIES15	0	0	0	0	0
PRAFACSQ	0	0	0	0	0

THETA

	PRESS15	DUTIES15	PRAFACSQ
PRESS15	0		
DUTIES15	0	0	
PRAFACSQ	0	0	1

ALPHA

KESCAT16	PHY15	KES15	CHOICE15	BULLY15
0	2	3	4	5

ALPHA

PRESS15	DUTIES15
6	7

BETA

	KESCAT16	PHY15	KES15	CHOICE15	BULLY15
KESCAT16	0	8	9	10	11
PHY15	0	0	0	0	0
KES15	0	0	0	0	0
CHOICE15	0	0	0	0	0
BULLY15	0	0	0	0	0
PRESS15	0	0	0	0	0
DUTIES15	0	0	0	0	0

BETA

	PRESS15	DUTIES15
KESCAT16	12	13
PHY15	0	0

KES15	0	0
CHOICE15	0	0
BULLY15	0	0
PRESS15	0	0
DUTIES15	0	0

PSI

	KESCAT16	PHY15	KES15	CHOICE15	BULLY15
KESCAT16	0				
PHY15	0	14			
KES15	0	15	16		
CHOICE15	0	17	18	19	
BULLY15	0	20	21	22	23
PRESS15	0	24	25	26	27
DUTIES15	0	29	30	31	32

PSI

	PRESS15	DUTIES15
PRESS15	28	
DUTIES15	33	34

PARAMETER SPECIFICATION FOR BETWEEN

TAU

KESCAT16
45

NU

KESCAT16	CLU2	CLU3	PRAFACSQ
0	0	0	35

LAMBDA

KESCAT16	CLU2	CLU3

KESCAT16	0	0	0
CLU2	0	0	0
CLU3	0	0	0
PRAFACSQ	0	0	0

THETA

	KESCAT16	CLU2	CLU3	PRAFACSQ
KESCAT16	0			
CLU2	0	0		
CLU3	0	0	0	
PRAFACSQ	0	0	0	36

ALPHA

KESCAT16	CLU2	CLU3
0	37	38

BETA

	KESCAT16	CLU2	CLU3
KESCAT16	0	39	40
CLU2	0	0	0
CLU3	0	0	0

PSI

	KESCAT16	CLU2	CLU3
KESCAT16	41		
CLU2	0	42	
CLU3	0	43	44

STARTING VALUES FOR WITHIN

TAU

KESCAT16
0.000

NU

KESCAT16	PHY15	KES15	CHOICE15	BULLY15
0.000	0.000	0.000	0.000	0.000

NU

PRESS15	DUTIES15	PRAFACSQ
0.000	0.000	0.000

LAMBDA

	KESCAT16	PHY15	KES15	CHOICE15	BULLY15
KESCAT16	1.000	0.000	0.000	0.000	0.000
PHY15	0.000	1.000	0.000	0.000	0.000
KES15	0.000	0.000	1.000	0.000	0.000
CHOICE15	0.000	0.000	0.000	1.000	0.000
BULLY15	0.000	0.000	0.000	0.000	1.000
PRESS15	0.000	0.000	0.000	0.000	0.000
DUTIES15	0.000	0.000	0.000	0.000	0.000
PRAFACSQ	0.000	0.000	0.000	0.000	0.000

LAMBDA

	PRESS15	DUTIES15
KESCAT16	0.000	0.000
PHY15	0.000	0.000
KES15	0.000	0.000
CHOICE15	0.000	0.000
BULLY15	0.000	0.000
PRESS15	1.000	0.000
DUTIES15	0.000	1.000
PRAFACSQ	0.000	0.000

THETA

	KESCAT16	PHY15	KES15	CHOICE15	BULLY15
KESCAT16	0.000				

PHY15	0.000	0.000			
KES15	0.000	0.000	0.000		
CHOICE15	0.000	0.000	0.000	0.000	
BULLY15	0.000	0.000	0.000	0.000	0.000
PRESS15	0.000	0.000	0.000	0.000	0.000
DUTIES15	0.000	0.000	0.000	0.000	0.000
PRAFACSQ	0.000	0.000	0.000	0.000	0.000

THETA

	PRESS15	DUTIES15	PRAFACSQ
PRESS15	0.000		
DUTIES15	0.000	0.000	
PRAFACSQ	0.000	0.000	0.754

ALPHA

KESCAT16	PHY15	KES15	CHOICE15	BULLY15
0.000	0.000	0.000	0.000	0.000

ALPHA

PRESS15	DUTIES15
0.000	0.000

BETA

	KESCAT16	PHY15	KES15	CHOICE15	BULLY15
KESCAT16	0.000	0.000	0.000	0.000	0.000
PHY15	0.000	0.000	0.000	0.000	0.000
KES15	0.000	0.000	0.000	0.000	0.000
CHOICE15	0.000	0.000	0.000	0.000	0.000
BULLY15	0.000	0.000	0.000	0.000	0.000
PRESS15	0.000	0.000	0.000	0.000	0.000
DUTIES15	0.000	0.000	0.000	0.000	0.000

BETA

PRESS15	DUTIES15

KESCAT16	0.000	0.000
PHY15	0.000	0.000
KES15	0.000	0.000
CHOICE15	0.000	0.000
BULLY15	0.000	0.000
PRESS15	0.000	0.000
DUTIES15	0.000	0.000

PSI

	KESCAT16	PHY15	KES15	CHOICE15	BULLY15
KESCAT16	1.000				
PHY15	0.000	0.268			
KES15	0.000	0.000	0.048		
CHOICE15	0.000	0.000	0.000	0.549	
BULLY15	0.000	0.000	0.000	0.000	0.108
PRESS15	0.000	0.000	0.000	0.000	0.000
DUTIES15	0.000	0.000	0.000	0.000	0.000

PSI

	PRESS15	DUTIES15
PRESS15	0.501	
DUTIES15	0.000	0.414

STARTING VALUES FOR BETWEEN

TAU

KESCAT16
1.091

NU

KESCAT16	CLU2	CLU3	PRAFACSQ
0.000	0.000	0.000	1.011

LAMBDA

	KESCAT16	CLU2	CLU3
KESCAT16	1.000	0.000	0.000
CLU2	0.000	1.000	0.000
CLU3	0.000	0.000	1.000
PRAFACSQ	0.000	0.000	0.000

THETA

	KESCAT16	CLU2	CLU3	PRAFACSQ
KESCAT16	0.000			
CLU2	0.000	0.000		
CLU3	0.000	0.000	0.000	
PRAFACSQ	0.000	0.000	0.000	0.754

ALPHA

KESCAT16	CLU2	CLU3
0.000	0.403	0.469

BETA

	KESCAT16	CLU2	CLU3
KESCAT16	0.000	0.000	0.000
CLU2	0.000	0.000	0.000
CLU3	0.000	0.000	0.000

PSI

	KESCAT16	CLU2	CLU3
KESCAT16	1.000		
CLU2	0.000	0.120	
CLU3	0.000	0.000	0.125

PRIORS FOR ALL PARAMETERS	PRIOR MEAN	PRIOR VARIANCE	PRIOR STD. DEV.
Parameter 1~IG(-1.000,0.000)	infinity	infinity	infinity
Parameter 2~N(0.000,infinity)	0.0000	infinity	infinity

Parameter 3~N(0.000,infinity)	0.0000	infinity	infinity
Parameter 4~N(0.000,infinity)	0.0000	infinity	infinity
Parameter 5~N(0.000,infinity)	0.0000	infinity	infinity
Parameter 6~N(0.000,infinity)	0.0000	infinity	infinity
Parameter 7~N(0.000,infinity)	0.0000	infinity	infinity
Parameter 8~N(0.000,5.000)	0.0000	5.0000	2.2361
Parameter 9~N(0.000,5.000)	0.0000	5.0000	2.2361
Parameter 10~N(0.000,5.000)	0.0000	5.0000	2.2361
Parameter 11~N(0.000,5.000)	0.0000	5.0000	2.2361
Parameter 12~N(0.000,5.000)	0.0000	5.0000	2.2361
Parameter 13~N(0.000,5.000)	0.0000	5.0000	2.2361
Parameter 14~IW(1.000,7)	infinity	infinity	infinity
Parameter 15~IW(0.000,7)	infinity	infinity	infinity
Parameter 16~IW(1.000,7)	infinity	infinity	infinity
Parameter 17~IW(0.000,7)	infinity	infinity	infinity
Parameter 18~IW(0.000,7)	infinity	infinity	infinity
Parameter 19~IW(1.000,7)	infinity	infinity	infinity
Parameter 20~IW(0.000,7)	infinity	infinity	infinity
Parameter 21~IW(0.000,7)	infinity	infinity	infinity
Parameter 22~IW(0.000,7)	infinity	infinity	infinity
Parameter 23~IW(1.000,7)	infinity	infinity	infinity
Parameter 24~IW(0.000,7)	infinity	infinity	infinity
Parameter 25~IW(0.000,7)	infinity	infinity	infinity
Parameter 26~IW(0.000,7)	infinity	infinity	infinity
Parameter 27~IW(0.000,7)	infinity	infinity	infinity
Parameter 28~IW(1.000,7)	infinity	infinity	infinity
Parameter 29~IW(0.000,7)	infinity	infinity	infinity
Parameter 30~IW(0.000,7)	infinity	infinity	infinity
Parameter 31~IW(0.000,7)	infinity	infinity	infinity
Parameter 32~IW(0.000,7)	infinity	infinity	infinity
Parameter 33~IW(0.000,7)	infinity	infinity	infinity
Parameter 34~IW(1.000,7)	infinity	infinity	infinity
Parameter 35~N(0.000,infinity)	0.0000	infinity	infinity
Parameter 36~IG(-1.000,0.000)	infinity	infinity	infinity
Parameter 37~N(0.000,infinity)	0.0000	infinity	infinity
Parameter 38~N(0.000,infinity)	0.0000	infinity	infinity
Parameter 39~N(0.000,5.000)	0.0000	5.0000	2.2361
Parameter 40~N(0.000,5.000)	0.0000	5.0000	2.2361
Parameter 41~IG(-1.000,0.000)	infinity	infinity	infinity
Parameter 42~IW(1.000,3)	infinity	infinity	infinity
Parameter 43~IW(0.000,3)	infinity	infinity	infinity
Parameter 44~IW(1.000,3)	infinity	infinity	infinity
Parameter 45~N(0.000,5.000)	0.0000	5.0000	2.2361

TECHNICAL 3 OUTPUT

ESTIMATED COVARIANCE MATRIX FOR PARAMETER ESTIMATES

	1	2	3	4	5
1	0.339746D-11				
2	0.512799D-09	0.972549D-04			
3	-0.115449D-10	0.915767D-05	0.159231D-04		
4	-0.100337D-08	-0.161322D-04	-0.623704D-05	0.208226D-03	
5	0.301115D-09	0.407970D-05	0.307087D-05	-0.102077D-04	0.380748D-04
6	0.278231D-09	0.160503D-04	0.974672D-05	-0.196331D-04	0.190215D-04
7	-0.797014D-10	-0.363718D-05	-0.664907D-05	0.304278D-04	-0.851290D-05
8	0.475601D-09	-0.228666D-05	-0.554512D-05	0.133464D-04	-0.157842D-05
9	0.218510D-08	-0.335435D-04	0.101135D-04	0.990424D-06	0.600531D-05
10	0.257119D-08	-0.499878D-05	-0.235886D-05	-0.214588D-05	0.437538D-05
11	-0.670567D-09	0.100315D-04	0.101694D-05	-0.217055D-04	0.817194D-05
12	0.344450D-09	0.979659D-05	0.413802D-05	0.350140D-05	0.454005D-05
13	0.604273D-09	0.197096D-05	-0.117729D-05	0.599974D-05	-0.386840D-05
14	0.433214D-09	-0.536749D-06	-0.699477D-06	0.335624D-05	0.360807D-05
15	0.131656D-09	0.957874D-06	-0.576910D-07	-0.415235D-06	0.457994D-06
16	0.172769D-09	-0.467035D-06	-0.154907D-06	-0.395686D-06	-0.375117D-06
17	-0.402654D-10	-0.358141D-05	-0.502931D-06	0.668073D-05	-0.117114D-05
18	-0.596943D-09	-0.129261D-05	0.552415D-06	0.307140D-05	-0.614345D-06
19	0.276549D-08	0.536339D-05	0.975824D-06	-0.571252D-05	0.303462D-06
20	0.382660D-10	0.140082D-05	0.913705D-06	-0.307763D-05	0.108939D-05
21	-0.461788D-10	-0.235511D-06	0.194274D-07	0.608864D-07	-0.227426D-07
22	-0.199322D-09	0.476455D-06	0.394674D-06	0.259569D-05	-0.404126D-06
23	-0.199896D-09	-0.204532D-06	0.345146D-06	0.208410D-05	0.116151D-05
24	0.173742D-09	0.149840D-05	-0.979209D-06	-0.727554D-05	-0.173367D-07
25	0.124797D-09	-0.810286D-07	-0.408430D-06	-0.505007D-05	0.428016D-06
26	0.311482D-09	-0.227228D-05	-0.121797D-05	0.330792D-05	-0.110148D-05
27	-0.345144D-09	0.831420D-06	0.177736D-06	0.192502D-06	0.150463D-05
28	-0.906710D-09	-0.189560D-05	0.135104D-05	-0.219052D-04	0.111251D-05
29	0.401495D-09	0.112634D-05	0.190003D-06	0.172876D-05	-0.119077D-06
30	-0.356687D-10	0.216887D-06	0.238990D-06	0.768733D-06	-0.386766D-06
31	-0.350207D-09	-0.119031D-06	0.174936D-05	0.152231D-05	-0.928771D-06
32	0.254304D-09	0.575393D-06	0.151811D-06	-0.273194D-05	0.912787D-07
33	0.445239D-09	-0.351374D-05	-0.245460D-05	0.107245D-04	-0.594494D-06
34	-0.448971D-09	-0.535862D-05	-0.204503D-06	0.886898D-05	-0.101033D-06
35	0.745468D-08	0.130746D-04	0.328070D-04	0.126259D-04	0.211345D-05
36	-0.555793D-07	-0.134216D-03	-0.146570D-04	0.946459D-04	0.118314D-03
37	-0.155794D-08	0.173175D-04	-0.167140D-05	0.157329D-04	0.274422D-05

38	0.247899D-09	0.106098D-04	0.122679D-04	-0.115221D-04	0.789908D-05
39	0.533019D-08	0.501488D-05	0.106102D-04	0.108931D-04	0.614687D-07
40	0.721026D-08	0.115212D-04	0.980154D-05	0.390397D-05	-0.748010D-05
41	-0.176633D-09	-0.187276D-05	-0.369050D-06	0.151994D-06	0.380785D-05
42	-0.402505D-09	-0.218845D-05	-0.696830D-06	0.274330D-04	-0.506434D-05
43	0.103666D-08	0.114939D-05	-0.137958D-06	-0.675777D-05	0.823227D-06
44	0.120022D-08	0.456704D-05	-0.298408D-05	-0.433204D-05	-0.324001D-05
45	0.311533D-08	-0.686658D-05	0.145974D-04	-0.199465D-04	0.327440D-06

ESTIMATED COVARIANCE MATRIX FOR PARAMETER ESTIMATES

	6	7	8	9	10
6	0.200736D-03				
7	-0.313808D-04	0.146884D-03			
8	-0.899724D-05	0.734903D-05	0.111250D-02		
9	0.391807D-05	-0.695558D-05	-0.387077D-03	0.711417D-02	
10	0.237900D-05	-0.675564D-05	0.482407D-04	-0.525017D-04	0.590179D-03
11	-0.174612D-04	-0.577155D-05	-0.831040D-04	-0.219243D-03	0.141334D-03
12	0.135157D-04	0.109168D-05	-0.265785D-04	-0.278918D-03	0.233002D-04
13	-0.414641D-05	0.815337D-05	0.323402D-04	0.142548D-03	-0.346834D-04
14	0.199214D-05	-0.160898D-05	0.109304D-04	0.133044D-04	0.122922D-04
15	0.927493D-06	-0.301572D-06	0.255584D-05	0.512363D-05	0.211055D-05
16	0.917311D-07	-0.294316D-06	0.279343D-05	-0.361536D-05	0.642446D-07
17	0.106781D-06	0.882832D-07	-0.350355D-05	0.128678D-04	0.388093D-05
18	-0.318016D-05	0.312959D-05	-0.170523D-05	0.451559D-05	0.487136D-05
19	0.967935D-05	0.314686D-05	0.216607D-04	-0.553310D-04	-0.940381D-05
20	-0.655677D-06	-0.866623D-06	-0.166321D-05	0.112235D-05	0.529721D-05
21	-0.757903D-06	-0.179487D-06	0.217723D-06	-0.553533D-05	-0.120847D-05
22	-0.187485D-05	0.114897D-05	0.206440D-06	0.465344D-04	0.438539D-05
23	-0.104041D-05	-0.308431D-06	-0.312596D-05	0.685771D-05	0.118018D-06
24	0.226171D-06	-0.241215D-05	-0.351235D-05	0.244786D-05	0.495122D-05
25	0.169856D-05	-0.200772D-05	0.304118D-05	-0.814905D-06	0.271838D-05
26	-0.203847D-05	0.311735D-06	-0.706059D-05	0.461427D-04	-0.716412D-06
27	-0.385832D-05	0.124093D-05	-0.580242D-05	-0.730908D-05	0.297743D-05
28	-0.207298D-06	-0.127144D-05	0.448754D-04	-0.417676D-04	0.260231D-05
29	0.313433D-05	0.429482D-05	-0.157375D-05	-0.199162D-04	-0.600134D-05
30	-0.961957D-06	0.198523D-05	-0.800770D-06	0.754442D-05	-0.390753D-05
31	-0.830503D-05	0.385982D-06	-0.342187D-05	-0.350845D-04	0.232602D-05
32	0.346989D-06	-0.241846D-05	-0.940135D-06	0.216366D-04	0.309587D-05
33	-0.150204D-07	0.510955D-05	0.402457D-05	0.914802D-04	-0.383972D-05
34	-0.157247D-05	0.207306D-06	0.742961D-05	-0.198282D-04	0.123180D-04
35	0.887982D-04	0.262671D-04	0.117458D-03	0.446902D-03	-0.348464D-04
36	0.454246D-05	0.945919D-04	0.267253D-03	0.694210D-03	-0.352332D-04

37	-0.167789D-04	0.326052D-04	-0.828996D-05	0.125416D-03	0.421397D-04
38	0.207884D-04	-0.197268D-04	-0.536709D-05	-0.132340D-03	-0.883099D-04
39	0.348279D-04	0.113702D-05	-0.407094D-04	0.520323D-03	-0.531479D-05
40	0.175933D-04	0.381853D-06	-0.136005D-03	0.112241D-03	0.103437D-03
41	0.101376D-05	-0.271914D-05	0.293755D-05	0.460366D-04	-0.105446D-05
42	0.220557D-04	0.120335D-04	0.268556D-04	0.886169D-04	0.222616D-04
43	-0.826247D-05	-0.966006D-05	-0.148152D-04	-0.687493D-04	-0.275541D-04
44	0.844019D-05	0.826105D-06	0.229839D-04	0.757112D-04	-0.715421D-05
45	0.176259D-04	-0.244572D-05	-0.129898D-04	0.124835D-02	0.279949D-04

ESTIMATED COVARIANCE MATRIX FOR PARAMETER ESTIMATES

	11	12	13	14	15
11	0.228222D-02				
12	-0.246421D-03	0.681675D-03			
13	0.113437D-03	0.111849D-03	0.745614D-03		
14	-0.128147D-04	0.876602D-05	-0.561249D-05	0.102073D-03	
15	-0.138917D-05	0.474821D-06	0.893160D-06	0.917872D-05	0.973891D-05
16	-0.211526D-05	0.781847D-06	0.770739D-06	0.331921D-06	0.164874D-05
17	0.100139D-04	-0.883043D-05	-0.643272D-05	-0.211798D-04	-0.587741D-05
18	0.444226D-06	-0.248405D-05	0.119490D-05	-0.229637D-05	-0.206722D-05
19	-0.139180D-04	0.128531D-05	-0.173025D-04	0.144229D-04	0.207375D-05
20	-0.129348D-05	0.457198D-05	0.440766D-05	0.443504D-05	0.236198D-05
21	0.371187D-05	-0.883758D-06	0.634128D-06	0.366972D-06	0.673261D-06
22	-0.798965D-06	-0.131731D-05	0.130312D-05	-0.719081D-06	-0.561065D-06
23	0.364344D-05	-0.424100D-05	0.105133D-05	-0.183666D-05	0.868351D-06
24	-0.106833D-05	-0.767398D-05	-0.691534D-05	0.124918D-04	0.663421D-05
25	-0.115306D-05	0.455140D-06	-0.285879D-05	0.133655D-05	0.211924D-05
26	-0.116773D-04	-0.156962D-04	0.505057D-05	0.865533D-06	-0.284585D-05
27	0.764215D-05	-0.552443D-05	0.724038D-06	-0.192530D-06	0.142458D-05
28	0.293904D-04	0.814502D-05	-0.796250D-06	0.582912D-05	0.246452D-05
29	-0.222813D-04	-0.451319D-05	0.119511D-05	-0.133045D-04	-0.523543D-05
30	-0.619358D-05	-0.281026D-05	0.715316D-05	-0.232897D-05	-0.161336D-05
31	-0.131635D-04	-0.817711D-05	-0.195224D-04	0.660025D-05	0.156682D-05
32	-0.160751D-04	0.399502D-05	0.149754D-05	-0.245911D-06	-0.101807D-05
33	-0.184646D-04	-0.528716D-05	0.442100D-05	-0.111025D-05	-0.280632D-05
34	0.138051D-04	0.516918D-05	-0.914942D-05	0.456350D-05	0.180271D-05
35	-0.308132D-04	-0.804924D-04	0.216355D-04	0.118970D-03	0.209312D-04
36	0.341520D-03	-0.636671D-04	-0.741029D-03	-0.167936D-04	0.238289D-05
37	0.146298D-03	-0.474122D-04	0.262194D-04	0.112691D-04	-0.123482D-05
38	-0.737560D-04	-0.209321D-04	-0.979841D-04	-0.266794D-05	-0.509455D-05
39	0.112713D-03	-0.612950D-04	-0.132666D-03	-0.106617D-04	-0.563023D-05
40	-0.788014D-04	0.365698D-04	-0.559317D-04	-0.801251D-05	-0.303848D-05

41	0.142229D-04	0.101546D-04	-0.771217D-05	0.264319D-06	0.218153D-06
42	-0.681594D-04	-0.250921D-04	0.219004D-04	0.103676D-05	0.179443D-05
43	0.245132D-04	-0.122926D-04	-0.999144D-05	-0.768696D-06	0.884809D-06
44	0.267293D-04	0.459843D-05	-0.149759D-04	-0.127428D-04	-0.280608D-05
45	0.548388D-04	-0.599289D-04	-0.138587D-03	0.298994D-05	-0.168209D-05

ESTIMATED COVARIANCE MATRIX FOR PARAMETER ESTIMATES

	16	17	18	19	20
16	0.347356D-05				
17	-0.515397D-06	0.120885D-03			
18	-0.120613D-05	0.128394D-04	0.196056D-04		
19	-0.451549D-06	-0.483671D-04	-0.175459D-04	0.449769D-03	
20	0.234154D-06	-0.727010D-05	-0.892452D-06	0.281381D-05	0.204676D-04
21	0.710348D-06	-0.502824D-06	-0.583775D-06	-0.562747D-06	0.228237D-05
22	-0.268570D-06	0.533084D-05	0.437099D-05	-0.195707D-04	-0.458204D-05
23	0.218110D-06	-0.150995D-05	-0.293267D-06	-0.377719D-06	0.175090D-05
24	0.326043D-07	-0.140725D-04	-0.290009D-05	0.999475D-05	0.963608D-05
25	0.174407D-05	-0.692536D-06	-0.599615D-06	0.307043D-05	0.954746D-06
26	-0.958603D-06	0.180221D-04	0.105243D-04	-0.381123D-04	-0.242236D-05
27	0.237878D-06	-0.810274D-06	-0.265911D-07	-0.216766D-05	0.232607D-05
28	0.284611D-06	0.441013D-06	-0.431972D-06	0.237680D-05	-0.293647D-06
29	-0.613092D-06	0.210257D-04	0.154524D-05	-0.453770D-05	-0.501445D-05
30	-0.173498D-05	0.156901D-05	0.236386D-05	-0.257939D-05	-0.654181D-06
31	0.759171D-06	-0.143494D-04	-0.771112D-05	0.603280D-04	-0.769969D-06
32	-0.482420D-06	0.229133D-05	0.693709D-06	-0.302295D-05	-0.236664D-05
33	-0.117206D-06	0.647819D-05	0.236816D-06	-0.631356D-05	-0.510186D-06
34	-0.403441D-06	-0.269837D-05	-0.227656D-05	0.192535D-04	0.109732D-05
35	-0.115412D-04	-0.733337D-04	0.206771D-04	-0.101217D-03	0.217022D-05
36	-0.351334D-04	0.176245D-03	0.961279D-04	-0.653149D-04	-0.955045D-04
37	0.234911D-05	-0.666752D-05	0.697762D-06	0.156485D-04	-0.480177D-05
38	-0.131307D-05	0.255025D-04	0.838745D-05	0.541081D-04	0.126389D-04
39	0.972583D-05	-0.276125D-04	-0.219198D-04	0.812478D-04	0.148550D-04
40	0.370809D-05	-0.227473D-04	-0.980020D-05	0.110844D-03	0.148186D-04
41	0.508225D-06	0.237744D-05	-0.582245D-06	0.165883D-06	-0.145812D-05
42	0.310204D-05	0.373380D-04	0.492059D-05	-0.570833D-04	-0.725930D-05
43	-0.258583D-05	-0.202272D-04	0.774045D-06	0.206027D-04	0.939619D-06
44	0.204339D-05	0.101494D-04	-0.120656D-04	0.288076D-04	0.263105D-05
45	0.717266D-05	-0.245169D-04	-0.231056D-04	0.634311D-04	0.812166D-05

ESTIMATED COVARIANCE MATRIX FOR PARAMETER ESTIMATES

21	22	23	24	25

21	0.391529D-05				
22	-0.155685D-05	0.444088D-04			
23	0.138040D-05	-0.368302D-05	0.168159D-04		
24	0.451514D-06	-0.329039D-05	0.808762D-06	0.108842D-03	
25	0.194617D-05	-0.187157D-05	0.100921D-05	0.104677D-04	0.179730D-04
26	-0.179232D-05	0.169641D-04	-0.544083D-06	-0.219950D-04	-0.490250D-05
27	0.236598D-05	-0.325457D-05	0.669583D-05	0.736494D-05	0.463621D-05
28	0.191313D-05	-0.258958D-05	0.386886D-05	0.234835D-04	0.172702D-04
29	-0.449981D-06	0.360761D-05	-0.188195D-05	-0.155954D-04	-0.221303D-05
30	-0.118592D-05	0.153373D-05	-0.102200D-05	-0.158159D-05	-0.322273D-05
31	0.473802D-06	-0.112038D-04	0.749366D-06	0.942682D-05	0.192080D-05
32	-0.191022D-05	0.710189D-05	-0.483679D-05	-0.323634D-05	-0.310668D-05
33	-0.135429D-05	0.214477D-05	-0.150399D-05	-0.917508D-05	-0.108184D-04
34	0.708653D-06	-0.780002D-05	0.183064D-05	-0.358949D-05	0.125122D-05
35	-0.137450D-05	0.382874D-04	-0.287203D-05	0.280863D-04	-0.298533D-04
36	-0.134299D-05	-0.295629D-05	0.390771D-04	-0.381252D-03	-0.486763D-04
37	0.392765D-05	0.560414D-05	-0.140559D-06	-0.958564D-06	0.143321D-04
38	0.458267D-06	-0.145321D-04	0.266019D-05	0.229057D-04	-0.112122D-04
39	0.164536D-05	0.300478D-04	-0.963117D-05	0.279549D-04	0.440924D-05
40	0.374765D-05	0.460040D-04	-0.191828D-04	0.179278D-04	-0.124514D-04
41	-0.346297D-06	0.254983D-06	0.129761D-05	0.762732D-06	-0.586866D-06
42	0.614558D-06	0.127049D-04	-0.763362D-05	-0.237900D-04	-0.519013D-05
43	-0.960206D-06	0.638245D-06	0.485535D-05	0.671101D-05	-0.271711D-06
44	0.182367D-06	-0.123367D-04	-0.537757D-06	0.641825D-05	0.130880D-05
45	0.102232D-05	0.386187D-04	-0.999474D-05	0.147523D-04	-0.756679D-06

ESTIMATED COVARIANCE MATRIX FOR PARAMETER ESTIMATES

	26	27	28	29	30
26	0.205880D-03				
27	-0.139108D-04	0.418453D-04			
28	-0.296428D-04	0.376049D-04	0.359068D-03		
29	0.112923D-04	-0.154326D-05	0.223141D-05	0.803330D-04	
30	0.310237D-05	-0.182956D-06	-0.587206D-05	0.941128D-05	0.153211D-04
31	-0.331982D-04	0.538644D-06	0.235768D-05	-0.137462D-04	-0.752918D-05
32	0.883572D-05	-0.651419D-05	-0.602246D-05	0.574497D-05	0.416484D-05
33	0.364925D-04	-0.137304D-04	-0.579525D-04	0.148710D-04	0.862568D-05
34	-0.164170D-04	0.132459D-05	0.535587D-05	-0.212578D-04	-0.151092D-04
35	-0.121025D-03	-0.148598D-04	-0.426680D-04	-0.445671D-06	-0.121403D-06
36	-0.821915D-04	-0.132151D-04	0.471585D-04	0.107034D-04	-0.454653D-04
37	-0.527741D-05	0.102695D-04	0.125190D-05	-0.137144D-04	0.272995D-05
38	-0.436263D-05	-0.441925D-05	-0.184398D-04	-0.370277D-05	-0.297296D-05

39	0.196812D-05	-0.115584D-04	-0.408847D-05	-0.924442D-05	-0.109793D-04
40	-0.187460D-05	-0.455053D-05	-0.330024D-04	-0.154129D-04	-0.131456D-04
41	-0.110466D-05	-0.796087D-06	0.160315D-05	-0.326550D-05	-0.242892D-05
42	0.178291D-04	-0.353593D-05	0.182560D-04	0.909716D-05	-0.623557D-05
43	-0.712311D-05	0.370578D-05	-0.217540D-04	0.105355D-05	0.415389D-05
44	-0.582227D-05	0.323524D-05	0.162001D-04	-0.789491D-05	-0.309083D-05
45	-0.236778D-04	-0.181600D-05	0.201869D-05	-0.171638D-04	-0.105008D-04

ESTIMATED COVARIANCE MATRIX FOR PARAMETER ESTIMATES

	31	32	33	34	35
31	0.172727D-03				
32	-0.860262D-05	0.376122D-04			
33	-0.135545D-04	0.172912D-04	0.152677D-03		
34	0.562508D-04	-0.190075D-04	-0.477274D-04	0.255314D-03	
35	0.100515D-03	-0.130155D-04	0.179053D-05	0.448652D-04	0.445133D-01
36	0.679377D-04	-0.499289D-04	-0.172186D-03	-0.144752D-04	-0.238268D-03
37	0.101627D-04	-0.295332D-05	0.166909D-05	-0.479549D-04	0.118256D-03
38	-0.394693D-05	-0.824855D-06	0.301804D-04	0.510974D-04	0.286355D-03
39	0.569039D-04	0.925185D-06	-0.670974D-05	0.120548D-04	-0.355365D-03
40	0.430747D-04	-0.436133D-06	-0.407943D-05	-0.139600D-04	0.323930D-03
41	0.157001D-05	-0.440028D-06	0.857746D-06	0.375584D-06	-0.979732D-04
42	-0.133156D-04	0.181940D-06	0.583910D-05	0.185749D-04	-0.317015D-04
43	0.169030D-04	0.221275D-05	-0.179385D-05	-0.164073D-04	0.299987D-04
44	-0.186191D-04	-0.253597D-05	-0.215597D-04	0.327829D-04	-0.802156D-04
45	0.465210D-04	-0.755823D-05	-0.188189D-04	-0.204060D-05	-0.204797D-03

ESTIMATED COVARIANCE MATRIX FOR PARAMETER ESTIMATES

	36	37	38	39	40
36	0.260148D+00				
37	-0.226121D-03	0.397620D-02			
38	-0.226049D-02	-0.243516D-02	0.487692D-02		
39	-0.540414D-03	-0.331774D-04	-0.527830D-04	0.824244D-02	
40	0.364384D-03	-0.506232D-04	-0.771096D-04	0.543136D-02	0.737596D-02
41	0.284983D-03	-0.603206D-05	-0.152790D-04	0.109347D-03	0.654977D-04
42	-0.215431D-03	0.688082D-04	0.331566D-05	0.141014D-03	-0.229541D-04
43	0.172244D-03	0.380491D-04	-0.752144D-04	-0.108278D-03	-0.337242D-05
44	-0.469831D-03	-0.908719D-04	0.173595D-03	0.104601D-03	0.207676D-04
45	0.545433D-03	-0.852423D-04	-0.661620D-04	0.567447D-02	0.544625D-02

ESTIMATED COVARIANCE MATRIX FOR PARAMETER ESTIMATES

	41	42	43	44	45
41	0.699959D-04				
42	0.652080D-05	0.191008D-02			
43	-0.208879D-05	-0.113056D-02	0.143357D-02		
44	0.154472D-04	0.644480D-03	-0.129409D-02	0.256722D-02	
45	0.112497D-03	0.903482D-04	-0.820482D-04	0.118566D-03	0.580703D-02

ESTIMATED CORRELATION MATRIX FOR PARAMETER ESTIMATES

	1	2	3	4	5
1	1.000				
2	0.028	1.000			
3	-0.002	0.233	1.000		
4	-0.038	-0.113	-0.108	1.000	
5	0.026	0.067	0.125	-0.115	1.000
6	0.011	0.115	0.172	-0.096	0.218
7	-0.004	-0.030	-0.137	0.174	-0.114
8	0.008	-0.007	-0.042	0.028	-0.008
9	0.014	-0.040	0.030	0.001	0.012
10	0.057	-0.021	-0.024	-0.006	0.029
11	-0.008	0.021	0.005	-0.031	0.028
12	0.007	0.038	0.040	0.009	0.028
13	0.012	0.007	-0.011	0.015	-0.023
14	0.023	-0.005	-0.017	0.023	0.058
15	0.023	0.031	-0.005	-0.009	0.024
16	0.050	-0.025	-0.021	-0.015	-0.033
17	-0.002	-0.033	-0.011	0.042	-0.017
18	-0.073	-0.030	0.031	0.048	-0.022
19	0.071	0.026	0.012	-0.019	0.002
20	0.005	0.031	0.051	-0.047	0.039
21	-0.013	-0.012	0.002	0.002	-0.002
22	-0.016	0.007	0.015	0.027	-0.010
23	-0.026	-0.005	0.021	0.035	0.046
24	0.009	0.015	-0.024	-0.048	0.000
25	0.016	-0.002	-0.024	-0.083	0.016
26	0.012	-0.016	-0.021	0.016	-0.012
27	-0.029	0.013	0.007	0.002	0.038
28	-0.026	-0.010	0.018	-0.080	0.010
29	0.024	0.013	0.005	0.013	-0.002
30	-0.005	0.006	0.015	0.014	-0.016
31	-0.014	-0.001	0.033	0.008	-0.011

32	0.022	0.010	0.006	-0.031	0.002
33	0.020	-0.029	-0.050	0.060	-0.008
34	-0.015	-0.034	-0.003	0.038	-0.001
35	0.019	0.006	0.039	0.004	0.002
36	-0.059	-0.027	-0.007	0.013	0.038
37	-0.013	0.028	-0.007	0.017	0.007
38	0.002	0.015	0.044	-0.011	0.018
39	0.032	0.006	0.029	0.008	0.000
40	0.046	0.014	0.029	0.003	-0.014
41	-0.011	-0.023	-0.011	0.001	0.074
42	-0.005	-0.005	-0.004	0.043	-0.019
43	0.015	0.003	-0.001	-0.012	0.004
44	0.013	0.009	-0.015	-0.006	-0.010
45	0.022	-0.009	0.048	-0.018	0.001

ESTIMATED CORRELATION MATRIX FOR PARAMETER ESTIMATES

	6	7	8	9	10
6	1.000				
7	-0.183	1.000			
8	-0.019	0.018	1.000		
9	0.003	-0.007	-0.138	1.000	
10	0.007	-0.023	0.060	-0.026	1.000
11	-0.026	-0.010	-0.052	-0.054	0.122
12	0.037	0.003	-0.031	-0.127	0.037
13	-0.011	0.025	0.036	0.062	-0.052
14	0.014	-0.013	0.032	0.016	0.050
15	0.021	-0.008	0.025	0.019	0.028
16	0.003	-0.013	0.045	-0.023	0.001
17	0.001	0.001	-0.010	0.014	0.015
18	-0.051	0.058	-0.012	0.012	0.045
19	0.032	0.012	0.031	-0.031	-0.018
20	-0.010	-0.016	-0.011	0.003	0.048
21	-0.027	-0.007	0.003	-0.033	-0.025
22	-0.020	0.014	0.001	0.083	0.027
23	-0.018	-0.006	-0.023	0.020	0.001
24	0.002	-0.019	-0.010	0.003	0.020
25	0.028	-0.039	0.022	-0.002	0.026
26	-0.010	0.002	-0.015	0.038	-0.002
27	-0.042	0.016	-0.027	-0.013	0.019
28	-0.001	-0.006	0.071	-0.026	0.006
29	0.025	0.040	-0.005	-0.026	-0.028
30	-0.017	0.042	-0.006	0.023	-0.041

31	-0.045	0.002	-0.008	-0.032	0.007
32	0.004	-0.033	-0.005	0.042	0.021
33	0.000	0.034	0.010	0.088	-0.013
34	-0.007	0.001	0.014	-0.015	0.032
35	0.030	0.010	0.017	0.025	-0.007
36	0.001	0.015	0.016	0.016	-0.003
37	-0.019	0.043	-0.004	0.024	0.028
38	0.021	-0.023	-0.002	-0.022	-0.052
39	0.027	0.001	-0.013	0.068	-0.002
40	0.014	0.000	-0.047	0.015	0.050
41	0.009	-0.027	0.011	0.065	-0.005
42	0.036	0.023	0.018	0.024	0.021
43	-0.015	-0.021	-0.012	-0.022	-0.030
44	0.012	0.001	0.014	0.018	-0.006
45	0.016	-0.003	-0.005	0.194	0.015

ESTIMATED CORRELATION MATRIX FOR PARAMETER ESTIMATES

	11	12	13	14	15
11	1.000				
12	-0.198	1.000			
13	0.087	0.157	1.000		
14	-0.027	0.033	-0.020	1.000	
15	-0.009	0.006	0.010	0.291	1.000
16	-0.024	0.016	0.015	0.018	0.283
17	0.019	-0.031	-0.021	-0.191	-0.171
18	0.002	-0.021	0.010	-0.051	-0.150
19	-0.014	0.002	-0.030	0.067	0.031
20	-0.006	0.039	0.036	0.097	0.167
21	0.039	-0.017	0.012	0.018	0.109
22	-0.003	-0.008	0.007	-0.011	-0.027
23	0.019	-0.040	0.009	-0.044	0.068
24	-0.002	-0.028	-0.024	0.119	0.204
25	-0.006	0.004	-0.025	0.031	0.160
26	-0.017	-0.042	0.013	0.006	-0.064
27	0.025	-0.033	0.004	-0.003	0.071
28	0.032	0.016	-0.002	0.030	0.042
29	-0.052	-0.019	0.005	-0.147	-0.187
30	-0.033	-0.027	0.067	-0.059	-0.132
31	-0.021	-0.024	-0.054	0.050	0.038
32	-0.055	0.025	0.009	-0.004	-0.053
33	-0.031	-0.016	0.013	-0.009	-0.073
34	0.018	0.012	-0.021	0.028	0.036

35	-0.003	-0.015	0.004	0.056	0.032
36	0.014	-0.005	-0.053	-0.003	0.001
37	0.049	-0.029	0.015	0.018	-0.006
38	-0.022	-0.011	-0.051	-0.004	-0.023
39	0.026	-0.026	-0.054	-0.012	-0.020
40	-0.019	0.016	-0.024	-0.009	-0.011
41	0.036	0.046	-0.034	0.003	0.008
42	-0.033	-0.022	0.018	0.002	0.013
43	0.014	-0.012	-0.010	-0.002	0.007
44	0.011	0.003	-0.011	-0.025	-0.018
45	0.015	-0.030	-0.067	0.004	-0.007

ESTIMATED CORRELATION MATRIX FOR PARAMETER ESTIMATES

	16	17	18	19	20
16	1.000				
17	-0.025	1.000			
18	-0.146	0.264	1.000		
19	-0.011	-0.207	-0.187	1.000	
20	0.028	-0.146	-0.045	0.029	1.000
21	0.193	-0.023	-0.067	-0.013	0.255
22	-0.022	0.073	0.148	-0.138	-0.152
23	0.029	-0.033	-0.016	-0.004	0.094
24	0.002	-0.123	-0.063	0.045	0.204
25	0.221	-0.015	-0.032	0.034	0.050
26	-0.036	0.114	0.166	-0.125	-0.037
27	0.020	-0.011	-0.001	-0.016	0.079
28	0.008	0.002	-0.005	0.006	-0.003
29	-0.037	0.213	0.039	-0.024	-0.124
30	-0.238	0.036	0.136	-0.031	-0.037
31	0.031	-0.099	-0.133	0.216	-0.013
32	-0.042	0.034	0.026	-0.023	-0.085
33	-0.005	0.048	0.004	-0.024	-0.009
34	-0.014	-0.015	-0.032	0.057	0.015
35	-0.029	-0.032	0.022	-0.023	0.002
36	-0.037	0.031	0.043	-0.006	-0.041
37	0.020	-0.010	0.002	0.012	-0.017
38	-0.010	0.033	0.027	0.037	0.040
39	0.057	-0.028	-0.055	0.042	0.036
40	0.023	-0.024	-0.026	0.061	0.038
41	0.033	0.026	-0.016	0.001	-0.039
42	0.038	0.078	0.025	-0.062	-0.037
43	-0.037	-0.049	0.005	0.026	0.005

44	0.022	0.018	-0.054	0.027	0.011
45	0.051	-0.029	-0.068	0.039	0.024

ESTIMATED CORRELATION MATRIX FOR PARAMETER ESTIMATES

	21	22	23	24	25
21	1.000				
22	-0.118	1.000			
23	0.170	-0.135	1.000		
24	0.022	-0.047	0.019	1.000	
25	0.232	-0.066	0.058	0.237	1.000
26	-0.063	0.177	-0.009	-0.147	-0.081
27	0.185	-0.075	0.252	0.109	0.169
28	0.051	-0.021	0.050	0.119	0.215
29	-0.025	0.060	-0.051	-0.167	-0.058
30	-0.153	0.059	-0.064	-0.039	-0.194
31	0.018	-0.128	0.014	0.069	0.034
32	-0.157	0.174	-0.192	-0.051	-0.119
33	-0.055	0.026	-0.030	-0.071	-0.207
34	0.022	-0.073	0.028	-0.022	0.018
35	-0.003	0.027	-0.003	0.013	-0.033
36	-0.001	-0.001	0.019	-0.072	-0.023
37	0.031	0.013	-0.001	-0.001	0.054
38	0.003	-0.031	0.009	0.031	-0.038
39	0.009	0.050	-0.026	0.030	0.011
40	0.022	0.080	-0.054	0.020	-0.034
41	-0.021	0.005	0.038	0.009	-0.017
42	0.007	0.044	-0.043	-0.052	-0.028
43	-0.013	0.003	0.031	0.017	-0.002
44	0.002	-0.037	-0.003	0.012	0.006
45	0.007	0.076	-0.032	0.019	-0.002

ESTIMATED CORRELATION MATRIX FOR PARAMETER ESTIMATES

	26	27	28	29	30
26	1.000				
27	-0.150	1.000			
28	-0.109	0.307	1.000		
29	0.088	-0.027	0.013	1.000	
30	0.055	-0.007	-0.079	0.268	1.000
31	-0.176	0.006	0.009	-0.117	-0.146
32	0.100	-0.164	-0.052	0.105	0.173

33	0.206	-0.172	-0.248	0.134	0.178
34	-0.072	0.013	0.018	-0.148	-0.242
35	-0.040	-0.011	-0.011	0.000	0.000
36	-0.011	-0.004	0.005	0.002	-0.023
37	-0.006	0.025	0.001	-0.024	0.011
38	-0.004	-0.010	-0.014	-0.006	-0.011
39	0.002	-0.020	-0.002	-0.011	-0.031
40	-0.002	-0.008	-0.020	-0.020	-0.039
41	-0.009	-0.015	0.010	-0.044	-0.074
42	0.028	-0.013	0.022	0.023	-0.036
43	-0.013	0.015	-0.030	0.003	0.028
44	-0.008	0.010	0.017	-0.017	-0.016
45	-0.022	-0.004	0.001	-0.025	-0.035

ESTIMATED CORRELATION MATRIX FOR PARAMETER ESTIMATES

	31	32	33	34	35
31	1.000				
32	-0.107	1.000			
33	-0.083	0.228	1.000		
34	0.268	-0.194	-0.242	1.000	
35	0.036	-0.010	0.001	0.013	1.000
36	0.010	-0.016	-0.027	-0.002	-0.002
37	0.012	-0.008	0.002	-0.048	0.009
38	-0.004	-0.002	0.035	0.046	0.019
39	0.048	0.002	-0.006	0.008	-0.019
40	0.038	-0.001	-0.004	-0.010	0.018
41	0.014	-0.009	0.008	0.003	-0.056
42	-0.023	0.001	0.011	0.027	-0.003
43	0.034	0.010	-0.004	-0.027	0.004
44	-0.028	-0.008	-0.034	0.040	-0.008
45	0.046	-0.016	-0.020	-0.002	-0.013

ESTIMATED CORRELATION MATRIX FOR PARAMETER ESTIMATES

	36	37	38	39	40
36	1.000				
37	-0.007	1.000			
38	-0.063	-0.553	1.000		
39	-0.012	-0.006	-0.008	1.000	
40	0.008	-0.009	-0.013	0.697	1.000
41	0.067	-0.011	-0.026	0.144	0.091

42	-0.010	0.025	0.001	0.036	-0.006
43	0.009	0.016	-0.028	-0.031	-0.001
44	-0.018	-0.028	0.049	0.023	0.005
45	0.014	-0.018	-0.012	0.820	0.832

ESTIMATED CORRELATION MATRIX FOR PARAMETER ESTIMATES

	41	42	43	44	45
41	1.000				
42	0.018	1.000			
43	-0.007	-0.683	1.000		
44	0.036	0.291	-0.675	1.000	
45	0.176	0.027	-0.028	0.031	1.000

TECHNICAL 8 OUTPUT

TECHNICAL 8 OUTPUT FOR BAYES ESTIMATION

CHAIN	BSEED
1	0
2	285380
3	253358

ITERATION	POTENTIAL SCALE REDUCTION	PARAMETER WITH HIGHEST PSR
100	1.015	6
200	1.018	9
300	1.002	43
400	1.008	9
500	1.004	9
600	1.002	9
700	1.006	13
800	1.001	17
900	1.001	17
1000	1.001	9

This is reduced psychological distress

SUMMARY OF DATA

Number of clusters 58

Size (s)	Cluster ID with Size s
1	166
2	74 205
3	106 182 165
4	93 170 22 192 4
5	65 71 50 84
6	80 72
7	40 107 61 75
8	169 30 173 228
9	144 81 83 181
10	69 145 36 68
11	108 116 174 154
12	206 162
13	226 114
14	2
15	152 98
18	156
19	202
21	92 6
24	178
25	142 122
32	24
37	204
47	209
53	125
67	217
174	110
210	100

COVARIANCE COVERAGE OF DATA

Minimum covariance coverage value 0.100

Number of missing data patterns 5

PROPORTION OF DATA PRESENT

Covariance Coverage

	KESCAT16	CLU2	CLU3	PHY15	KES15
KESCAT16	1.000				
CLU2	1.000	1.000			
CLU3	1.000	1.000	1.000		
PHY15	1.000	1.000	1.000	1.000	
KES15	1.000	1.000	1.000	1.000	1.000
CHOICE15	0.990	0.990	0.990	0.990	0.990
BULLY15	0.990	0.990	0.990	0.990	0.990
PRESS15	0.990	0.990	0.990	0.990	0.990
DUTIES15	0.991	0.991	0.991	0.991	0.991

Covariance Coverage

	CHOICE15	BULLY15	PRESS15	DUTIES15
CHOICE15	0.990			
BULLY15	0.989	0.990		
PRESS15	0.989	0.989	0.990	
DUTIES15	0.990	0.990	0.990	0.991

UNIVARIATE PROPORTIONS AND COUNTS FOR CATEGORICAL VARIABLES

KESCAT16

Category 1	0.347	386.000
Category 2	0.653	727.000

UNIVARIATE SAMPLE STATISTICS

UNIVARIATE HIGHER-ORDER MOMENT DESCRIPTIVE STATISTICS

Variable/ Sample Size	Mean/ Variance	Skewness/ Kurtosis	Minimum/ Maximum	% with Min/Max	Percentiles 20%/60%	40%/80%	Median
CLU2	0.310	0.820	0.000	68.97%	0.000	0.000	0.000

```
          58.000       0.214      -1.328       1.000   31.03%       0.000      1.000
     CLU3                0.448       0.208       0.000   55.17%       0.000      0.000      0.000
          58.000       0.247      -1.957       1.000   44.83%       1.000      1.000
     PHY15               0.000       0.390      -1.412   11.50%      -0.412     -0.412     -0.412
        1113.000       0.702       0.082       2.588    1.08%       0.588      0.588
     KES15               0.000       1.366      -0.556   20.75%      -0.556     -0.222     -0.222
        1113.000       0.313       1.871       2.278    0.63%      -0.056      0.444
     CHOICE15            0.000      -0.116      -1.814   15.06%      -0.814      0.186      0.186
        1102.000       1.193      -0.747       2.186    4.45%       0.186      1.186
     BULLY15             0.000       1.887      -0.426   71.96%      -0.426     -0.426     -0.426
        1102.000       0.602       3.132       3.574    0.45%      -0.426      0.574
     PRESS15             0.000       0.096      -1.915    9.89%      -0.915      0.085      0.085
        1102.000       1.160      -0.512       2.085    8.35%       0.085      1.085
     DUTIES15            0.000      -0.677      -2.672    3.54%      -0.672      0.328      0.328
        1103.000       1.031       0.049       1.328   20.22%       0.328      1.328


THE MODEL ESTIMATION TERMINATED NORMALLY

     USE THE FBITERATIONS OPTION TO INCREASE THE NUMBER OF ITERATIONS BY A FACTOR
     OF AT LEAST TWO TO CHECK CONVERGENCE AND THAT THE PSR VALUE DOES NOT INCREASE.



MODEL FIT INFORMATION

Number of Free Parameters                              42

Bayesian Posterior Predictive Checking using Chi-Square

          95% Confidence Interval for the Difference Between
          the Observed and the Replicated Chi-Square Values

                             -22.381           26.792

          Posterior Predictive P-Value              0.503



MODEL RESULTS

                                Posterior  One-Tailed         95% C.I.
                    Estimate       S.D.      P-Value   Lower 2.5%  Upper 2.5%  Significance
```

Within Level

KESCAT16 ON						
PHY15	0.164	0.051	0.001	0.063	0.260	*
CHOICE15	0.028	0.039	0.241	-0.050	0.107	
BULLY15	0.103	0.059	0.033	-0.005	0.222	
DUTIES15	-0.079	0.043	0.027	-0.168	0.004	
PRESS15	-0.048	0.040	0.117	-0.125	0.030	
KES15	0.802	0.095	0.000	0.615	0.991	*
KES15 WITH						
PHY15	0.107	0.014	0.000	0.080	0.136	*
CHOICE15	-0.096	0.019	0.000	-0.134	-0.061	*
BULLY15	0.073	0.013	0.000	0.045	0.100	*
PRESS15	0.063	0.018	0.001	0.029	0.101	*
DUTIES15	-0.073	0.017	0.000	-0.106	-0.041	*
PHY15 WITH						
CHOICE15	-0.096	0.028	0.000	-0.152	-0.043	*
BULLY15	0.040	0.020	0.021	0.003	0.077	*
PRESS15	0.120	0.027	0.000	0.068	0.174	*
DUTIES15	-0.059	0.026	0.010	-0.112	-0.007	*
CHOICE15 WITH						
BULLY15	-0.139	0.026	0.000	-0.191	-0.089	*
PRESS15	-0.197	0.036	0.000	-0.266	-0.123	*
DUTIES15	0.149	0.034	0.000	0.086	0.218	*
BULLY15 WITH						
PRESS15	0.201	0.026	0.000	0.152	0.253	*
DUTIES15	-0.176	0.024	0.000	-0.222	-0.130	*
PRESS15 WITH						
DUTIES15	-0.285	0.034	0.000	-0.357	-0.223	*
Means						
PHY15	0.000	0.025	0.496	-0.049	0.047	
KES15	0.001	0.017	0.491	-0.033	0.033	
CHOICE15	0.000	0.033	0.497	-0.064	0.065	
BULLY15	-0.001	0.023	0.494	-0.046	0.044	
PRESS15	0.000	0.033	0.497	-0.065	0.066	
DUTIES15	0.001	0.030	0.479	-0.058	0.060	
Variances						

PHY15	0.704	0.030	0.000	0.650	0.766	*
KES15	0.314	0.013	0.000	0.289	0.342	*
CHOICE15	1.194	0.049	0.000	1.107	1.298	*
BULLY15	0.603	0.026	0.000	0.559	0.657	*
PRESS15	1.160	0.049	0.000	1.074	1.259	*
DUTIES15	1.031	0.044	0.000	0.947	1.123	*

Between Level

KESCAT16 ON						
CLU2	-0.130	0.160	0.205	-0.448	0.196	
CLU3	0.076	0.152	0.294	-0.212	0.394	
CLU2 WITH						
CLU3	-0.138	0.038	0.000	-0.225	-0.079	*
Means						
CLU2	0.309	0.063	0.000	0.187	0.435	*
CLU3	0.453	0.071	0.000	0.312	0.587	*
Thresholds						
KESCAT16$1	-0.471	0.130	0.000	-0.721	-0.205	*
Variances						
CLU2	0.229	0.045	0.000	0.162	0.341	*
CLU3	0.264	0.050	0.000	0.187	0.380	*
Residual Variances						
KESCAT16	0.033	0.040	0.000	0.001	0.157	*

STANDARDIZED MODEL RESULTS

STDYX Standardization

	Estimate	Posterior S.D.	One-Tailed P-Value	95% C.I. Lower 2.5%	Upper 2.5%	Significance
Within Level						
KESCAT16 ON						
PHY15	0.121	0.037	0.001	0.047	0.191	*
CHOICE15	0.027	0.037	0.241	-0.048	0.101	
BULLY15	0.071	0.040	0.033	-0.003	0.153	

DUTIES15	-0.071	0.038	0.027	-0.147	0.004	
PRESS15	-0.045	0.038	0.117	-0.118	0.030	
KES15	0.396	0.040	0.000	0.311	0.473	*
KES15 WITH						
PHY15	0.227	0.028	0.000	0.173	0.282	*
CHOICE15	-0.157	0.030	0.000	-0.215	-0.101	*
BULLY15	0.168	0.030	0.000	0.106	0.224	*
PRESS15	0.104	0.030	0.001	0.047	0.165	*
DUTIES15	-0.128	0.029	0.000	-0.185	-0.072	*
PHY15 WITH						
CHOICE15	-0.104	0.030	0.000	-0.164	-0.048	*
BULLY15	0.062	0.030	0.021	0.004	0.119	*
PRESS15	0.132	0.029	0.000	0.075	0.190	*
DUTIES15	-0.069	0.030	0.010	-0.129	-0.008	*
CHOICE15 WITH						
BULLY15	-0.164	0.030	0.000	-0.220	-0.105	*
PRESS15	-0.168	0.029	0.000	-0.224	-0.105	*
DUTIES15	0.135	0.029	0.000	0.077	0.192	*
BULLY15 WITH						
PRESS15	0.241	0.028	0.000	0.186	0.295	*
DUTIES15	-0.223	0.029	0.000	-0.277	-0.168	*
PRESS15 WITH						
DUTIES15	-0.261	0.028	0.000	-0.319	-0.206	*
Means						
PHY15	0.000	0.029	0.496	-0.058	0.057	
KES15	0.001	0.030	0.491	-0.059	0.060	
CHOICE15	0.000	0.031	0.497	-0.060	0.060	
BULLY15	-0.001	0.030	0.494	-0.061	0.057	
PRESS15	0.000	0.031	0.497	-0.060	0.062	
DUTIES15	0.001	0.029	0.479	-0.057	0.060	
Variances						
PHY15	1.000	0.000	0.000	1.000	1.000	
KES15	1.000	0.000	0.000	1.000	1.000	
CHOICE15	1.000	0.000	0.000	1.000	1.000	
BULLY15	1.000	0.000	0.000	1.000	1.000	
PRESS15	1.000	0.000	0.000	1.000	1.000	
DUTIES15	1.000	0.000	0.000	1.000	1.000	

Between Level

KESCAT16 ON						
CLU2	-0.309	0.356	0.205	-0.989	0.425	
CLU3	0.188	0.359	0.294	-0.563	0.863	
CLU2 WITH						
CLU3	-0.566	0.086	0.000	-0.708	-0.383	*
Means						
CLU2	0.649	0.145	0.000	0.362	0.930	*
CLU3	0.882	0.156	0.000	0.577	1.195	*
Thresholds						
KESCAT16$1	-2.170	1.248	0.000	-5.631	-0.676	*
Variances						
CLU2	1.000	0.000	0.000	1.000	1.000	
CLU3	1.000	0.000	0.000	1.000	1.000	
Residual Variances						
KESCAT16	0.713	0.232	0.000	0.103	0.980	*

R-SQUARE

Within Level

Variable	Estimate	Posterior S.D.	One-Tailed P-Value	95% C.I. Lower 2.5%	Upper 2.5%
KESCAT16	0.221	0.034	0.000	0.157	0.287

Between Level

Variable	Estimate	Posterior S.D.	One-Tailed P-Value	95% C.I. Lower 2.5%	Upper 2.5%
KESCAT16	0.287	0.232	0.000	0.020	0.897

TECHNICAL 1 OUTPUT

PARAMETER SPECIFICATION FOR WITHIN

TAU

KESCAT16
0

NU

KESCAT16	PHY15	KES15	CHOICE15	BULLY15
0	0	0	0	0

NU

PRESS15	DUTIES15
0	0

LAMBDA

	KESCAT16	PHY15	KES15	CHOICE15	BULLY15
KESCAT16	0	0	0	0	0
PHY15	0	0	0	0	0
KES15	0	0	0	0	0
CHOICE15	0	0	0	0	0
BULLY15	0	0	0	0	0
PRESS15	0	0	0	0	0
DUTIES15	0	0	0	0	0

LAMBDA

	PRESS15	DUTIES15
KESCAT16	0	0
PHY15	0	0
KES15	0	0
CHOICE15	0	0
BULLY15	0	0
PRESS15	0	0
DUTIES15	0	0

THETA

	KESCAT16	PHY15	KES15	CHOICE15	BULLY15
KESCAT16	0				
PHY15	0	0			
KES15	0	0	0		
CHOICE15	0	0	0	0	
BULLY15	0	0	0	0	0
PRESS15	0	0	0	0	0
DUTIES15	0	0	0	0	0

THETA

	PRESS15	DUTIES15
PRESS15	0	
DUTIES15	0	0

ALPHA

KESCAT16	PHY15	KES15	CHOICE15	BULLY15
0	1	2	3	4

ALPHA

PRESS15	DUTIES15
5	6

BETA

	KESCAT16	PHY15	KES15	CHOICE15	BULLY15
KESCAT16	0	7	8	9	10
PHY15	0	0	0	0	0
KES15	0	0	0	0	0
CHOICE15	0	0	0	0	0
BULLY15	0	0	0	0	0
PRESS15	0	0	0	0	0
DUTIES15	0	0	0	0	0

BETA

	PRESS15	DUTIES15
KESCAT16	11	12
PHY15	0	0
KES15	0	0
CHOICE15	0	0
BULLY15	0	0
PRESS15	0	0
DUTIES15	0	0

PSI

	KESCAT16	PHY15	KES15	CHOICE15	BULLY15
KESCAT16	0				
PHY15	0	13			
KES15	0	14	15		
CHOICE15	0	16	17	18	
BULLY15	0	19	20	21	22
PRESS15	0	23	24	25	26
DUTIES15	0	28	29	30	31

PSI

	PRESS15	DUTIES15
PRESS15	27	
DUTIES15	32	33

PARAMETER SPECIFICATION FOR BETWEEN

TAU

KESCAT16
42

NU

KESCAT16	CLU2	CLU3

0	0	0

LAMBDA

	KESCAT16	CLU2	CLU3
KESCAT16	0	0	0
CLU2	0	0	0
CLU3	0	0	0

THETA

	KESCAT16	CLU2	CLU3
KESCAT16	0		
CLU2	0	0	
CLU3	0	0	0

ALPHA

KESCAT16	CLU2	CLU3
0	34	35

BETA

	KESCAT16	CLU2	CLU3
KESCAT16	0	36	37
CLU2	0	0	0
CLU3	0	0	0

PSI

	KESCAT16	CLU2	CLU3
KESCAT16	38		
CLU2	0	39	
CLU3	0	40	41

STARTING VALUES FOR WITHIN

TAU

KESCAT16
0.000

NU

KESCAT16	PHY15	KES15	CHOICE15	BULLY15
0.000	0.000	0.000	0.000	0.000

NU

PRESS15	DUTIES15
0.000	0.000

LAMBDA

	KESCAT16	PHY15	KES15	CHOICE15	BULLY15
KESCAT16	1.000	0.000	0.000	0.000	0.000
PHY15	0.000	1.000	0.000	0.000	0.000
KES15	0.000	0.000	1.000	0.000	0.000
CHOICE15	0.000	0.000	0.000	1.000	0.000
BULLY15	0.000	0.000	0.000	0.000	1.000
PRESS15	0.000	0.000	0.000	0.000	0.000
DUTIES15	0.000	0.000	0.000	0.000	0.000

LAMBDA

	PRESS15	DUTIES15
KESCAT16	0.000	0.000
PHY15	0.000	0.000
KES15	0.000	0.000
CHOICE15	0.000	0.000
BULLY15	0.000	0.000
PRESS15	1.000	0.000
DUTIES15	0.000	1.000

THETA

KESCAT16	PHY15	KES15	CHOICE15	BULLY15

KESCAT16	0.000				
PHY15	0.000	0.000			
KES15	0.000	0.000	0.000		
CHOICE15	0.000	0.000	0.000	0.000	
BULLY15	0.000	0.000	0.000	0.000	0.000
PRESS15	0.000	0.000	0.000	0.000	0.000
DUTIES15	0.000	0.000	0.000	0.000	0.000

THETA

	PRESS15	DUTIES15
PRESS15	0.000	
DUTIES15	0.000	0.000

ALPHA

KESCAT16	PHY15	KES15	CHOICE15	BULLY15
0.000	0.000	0.000	0.000	0.000

ALPHA

PRESS15	DUTIES15
0.000	0.000

BETA

	KESCAT16	PHY15	KES15	CHOICE15	BULLY15
KESCAT16	0.000	0.000	0.000	0.000	0.000
PHY15	0.000	0.000	0.000	0.000	0.000
KES15	0.000	0.000	0.000	0.000	0.000
CHOICE15	0.000	0.000	0.000	0.000	0.000
BULLY15	0.000	0.000	0.000	0.000	0.000
PRESS15	0.000	0.000	0.000	0.000	0.000
DUTIES15	0.000	0.000	0.000	0.000	0.000

BETA

PRESS15	DUTIES15

KESCAT16	0.000	0.000
PHY15	0.000	0.000
KES15	0.000	0.000
CHOICE15	0.000	0.000
BULLY15	0.000	0.000
PRESS15	0.000	0.000
DUTIES15	0.000	0.000

PSI

	KESCAT16	PHY15	KES15	CHOICE15	BULLY15
KESCAT16	1.000				
PHY15	0.000	0.351			
KES15	0.000	0.000	0.157		
CHOICE15	0.000	0.000	0.000	0.597	
BULLY15	0.000	0.000	0.000	0.000	0.301
PRESS15	0.000	0.000	0.000	0.000	0.000
DUTIES15	0.000	0.000	0.000	0.000	0.000

PSI

	PRESS15	DUTIES15
PRESS15	0.580	
DUTIES15	0.000	0.516

STARTING VALUES FOR BETWEEN

TAU

KESCAT16
-0.352

NU

KESCAT16	CLU2	CLU3
0.000	0.000	0.000

LAMBDA

	KESCAT16	CLU2	CLU3
KESCAT16	1.000	0.000	0.000
CLU2	0.000	1.000	0.000
CLU3	0.000	0.000	1.000

THETA

	KESCAT16	CLU2	CLU3
KESCAT16	0.000		
CLU2	0.000	0.000	
CLU3	0.000	0.000	0.000

ALPHA

KESCAT16	CLU2	CLU3
0.000	0.380	0.495

BETA

	KESCAT16	CLU2	CLU3
KESCAT16	0.000	0.000	0.000
CLU2	0.000	0.000	0.000
CLU3	0.000	0.000	0.000

PSI

	KESCAT16	CLU2	CLU3
KESCAT16	1.000		
CLU2	0.000	0.118	
CLU3	0.000	0.000	0.125

PRIORS FOR ALL PARAMETERS	PRIOR MEAN	PRIOR VARIANCE	PRIOR STD. DEV.
Parameter 1~N(0.000,infinity)	0.0000	infinity	infinity
Parameter 2~N(0.000,infinity)	0.0000	infinity	infinity
Parameter 3~N(0.000,infinity)	0.0000	infinity	infinity
Parameter 4~N(0.000,infinity)	0.0000	infinity	infinity

Parameter 5~N(0.000,infinity)	0.0000	infinity	infinity
Parameter 6~N(0.000,infinity)	0.0000	infinity	infinity
Parameter 7~N(0.000,5.000)	0.0000	5.0000	2.2361
Parameter 8~N(0.000,5.000)	0.0000	5.0000	2.2361
Parameter 9~N(0.000,5.000)	0.0000	5.0000	2.2361
Parameter 10~N(0.000,5.000)	0.0000	5.0000	2.2361
Parameter 11~N(0.000,5.000)	0.0000	5.0000	2.2361
Parameter 12~N(0.000,5.000)	0.0000	5.0000	2.2361
Parameter 13~IW(1.000,7)	infinity	infinity	infinity
Parameter 14~IW(0.000,7)	infinity	infinity	infinity
Parameter 15~IW(1.000,7)	infinity	infinity	infinity
Parameter 16~IW(0.000,7)	infinity	infinity	infinity
Parameter 17~IW(0.000,7)	infinity	infinity	infinity
Parameter 18~IW(1.000,7)	infinity	infinity	infinity
Parameter 19~IW(0.000,7)	infinity	infinity	infinity
Parameter 20~IW(0.000,7)	infinity	infinity	infinity
Parameter 21~IW(0.000,7)	infinity	infinity	infinity
Parameter 22~IW(1.000,7)	infinity	infinity	infinity
Parameter 23~IW(0.000,7)	infinity	infinity	infinity
Parameter 24~IW(0.000,7)	infinity	infinity	infinity
Parameter 25~IW(0.000,7)	infinity	infinity	infinity
Parameter 26~IW(0.000,7)	infinity	infinity	infinity
Parameter 27~IW(1.000,7)	infinity	infinity	infinity
Parameter 28~IW(0.000,7)	infinity	infinity	infinity
Parameter 29~IW(0.000,7)	infinity	infinity	infinity
Parameter 30~IW(0.000,7)	infinity	infinity	infinity
Parameter 31~IW(0.000,7)	infinity	infinity	infinity
Parameter 32~IW(0.000,7)	infinity	infinity	infinity
Parameter 33~IW(1.000,7)	infinity	infinity	infinity
Parameter 34~N(0.000,infinity)	0.0000	infinity	infinity
Parameter 35~N(0.000,infinity)	0.0000	infinity	infinity
Parameter 36~N(0.000,5.000)	0.0000	5.0000	2.2361
Parameter 37~N(0.000,5.000)	0.0000	5.0000	2.2361
Parameter 38~IG(-1.000,0.000)	infinity	infinity	infinity
Parameter 39~IW(1.000,3)	infinity	infinity	infinity
Parameter 40~IW(0.000,3)	infinity	infinity	infinity
Parameter 41~IW(1.000,3)	infinity	infinity	infinity
Parameter 42~N(0.000,5.000)	0.0000	5.0000	2.2361

TECHNICAL 3 OUTPUT

ESTIMATED COVARIANCE MATRIX FOR PARAMETER ESTIMATES

	1	2	3	4	5
1	0.606068D-03				
2	0.104890D-03	0.277347D-03			
3	-0.925407D-04	-0.649532D-04	0.111153D-02		
4	0.451962D-04	0.736537D-04	-0.136566D-03	0.544158D-03	
5	0.101066D-03	0.446049D-04	-0.215397D-03	0.177234D-03	0.108758D-02
6	-0.408520D-04	-0.473487D-04	0.976788D-04	-0.161986D-03	-0.253058D-03
7	-0.267905D-04	-0.140657D-04	-0.672284D-04	-0.110193D-04	0.242929D-04
8	0.653779D-04	-0.777897D-05	0.780222D-04	-0.124543D-04	-0.617926D-04
9	0.137540D-04	0.125661D-04	0.404128D-04	0.178970D-04	0.620300D-04
10	-0.980739D-05	0.288049D-04	0.352162D-04	0.103342D-04	0.369512D-04
11	0.352650D-04	0.102974D-04	-0.187129D-04	-0.129657D-04	0.120991D-04
12	0.974989D-06	0.156877D-04	-0.188928D-04	0.151876D-04	-0.910874D-04
13	0.611672D-04	0.212392D-04	-0.183465D-04	0.535780D-06	0.323046D-04
14	-0.108321D-05	0.118215D-04	-0.293400D-05	-0.151596D-04	-0.196764D-04
15	0.586035D-05	0.141467D-05	0.195669D-04	-0.166318D-04	-0.973637D-05
16	-0.436382D-04	-0.212681D-04	0.229361D-05	0.728133D-05	-0.174009D-04
17	0.579645D-06	0.109632D-04	-0.787867D-05	0.178073D-06	0.138677D-04
18	-0.383079D-04	-0.125196D-05	-0.341442D-04	0.775521D-05	0.209361D-04
19	0.998042D-05	0.174761D-04	0.153319D-04	-0.703121D-05	-0.129144D-04
20	0.517479D-05	0.215186D-05	0.654335D-06	0.933503D-05	0.686907D-05
21	0.830887D-05	-0.827599D-05	0.110508D-04	0.132026D-05	-0.288521D-04
22	0.169987D-04	0.430258D-05	-0.561622D-05	0.192135D-04	0.568239D-04
23	0.254761D-04	0.125191D-04	0.830616D-05	-0.153088D-04	0.219502D-04
24	0.452282D-05	0.212138D-05	0.163361D-04	0.962751D-05	0.159410D-04
25	-0.284351D-05	-0.388770D-05	-0.173818D-04	0.186676D-04	-0.510699D-05
26	-0.326295D-05	-0.116934D-04	0.199705D-05	0.236659D-04	0.304977D-04
27	0.343437D-04	-0.451642D-05	-0.246357D-04	0.183626D-04	0.476331D-04
28	-0.949421D-05	-0.209439D-04	-0.878791D-05	-0.345178D-04	-0.194420D-04
29	-0.212446D-05	0.179388D-05	-0.109482D-04	-0.408132D-05	0.198266D-04
30	-0.609548D-05	-0.126385D-04	-0.169529D-04	0.102243D-04	0.467616D-04
31	-0.882280D-05	0.161219D-05	0.306216D-04	-0.184860D-04	-0.476247D-05
32	-0.429035D-04	0.405188D-05	0.729290D-04	-0.344713D-04	-0.114694D-04
33	-0.108749D-05	0.623345D-05	-0.420032D-04	0.265209D-04	-0.690616D-05
34	-0.937185D-05	-0.144500D-04	0.921708D-05	0.754493D-04	0.191113D-04
35	0.261785D-04	0.171226D-04	-0.683531D-04	-0.648787D-04	0.350576D-05
36	-0.729541D-04	-0.364474D-04	0.135248D-04	0.178141D-03	0.118058D-03
37	-0.313811D-04	0.854813D-04	-0.266742D-04	0.294812D-03	0.290768D-03
38	0.468117D-05	0.541438D-05	0.144916D-04	0.604184D-04	-0.115320D-04
39	-0.211752D-05	0.990317D-05	0.471781D-06	0.273120D-05	0.158670D-04
40	0.153319D-04	0.423260D-05	-0.146330D-04	0.312743D-05	-0.385494D-04
41	-0.307704D-04	-0.133610D-04	-0.214811D-04	-0.133001D-04	0.853221D-04
42	-0.255723D-05	0.339023D-04	0.833233D-05	0.230010D-03	0.243154D-03

ESTIMATED COVARIANCE MATRIX FOR PARAMETER ESTIMATES

	6	7	8	9	10
6	0.881818D-03				
7	-0.578541D-05	0.258388D-02			
8	-0.156271D-04	-0.561939D-03	0.900730D-02		
9	0.563992D-05	0.813226D-04	0.459394D-03	0.150354D-02	
10	-0.128222D-05	0.653451D-05	-0.235461D-03	0.155128D-03	0.350930D-02
11	-0.156568D-04	-0.275069D-03	-0.123550D-03	0.460558D-04	-0.288397D-03
12	0.175116D-04	-0.743550D-05	0.436517D-03	-0.178985D-03	0.317185D-03
13	-0.161022D-04	0.758579D-04	0.102698D-04	0.681499D-05	-0.212120D-04
14	0.166971D-04	0.262200D-04	-0.397984D-04	0.432071D-05	0.228677D-04
15	0.692786D-05	-0.814899D-05	-0.165013D-04	0.137199D-04	0.358294D-04
16	-0.201800D-04	0.444488D-05	0.693483D-05	0.112135D-04	0.354931D-05
17	-0.197663D-04	0.230240D-05	-0.232152D-04	-0.962987D-05	0.544129D-05
18	-0.428532D-04	0.547064D-04	-0.623263D-04	-0.165672D-04	0.353136D-04
19	0.116598D-04	-0.142715D-04	-0.538397D-05	-0.564978D-06	-0.257965D-04
20	0.330779D-05	-0.975228D-05	0.668216D-04	0.629829D-05	-0.117808D-04
21	0.471631D-05	0.499411D-05	-0.144901D-03	-0.298993D-04	0.110150D-04
22	-0.325239D-04	0.168366D-04	0.620622D-04	-0.392007D-05	-0.252035D-04
23	-0.407865D-05	-0.100863D-04	0.730802D-05	-0.172208D-04	-0.307401D-04
24	-0.330584D-05	0.121077D-04	-0.264893D-04	0.188989D-04	0.153268D-04
25	-0.215178D-04	0.285042D-04	-0.139330D-03	-0.204947D-04	-0.502911D-04
26	-0.221780D-05	0.282388D-04	0.981949D-04	0.416605D-04	0.273747D-05
27	-0.133000D-04	0.117231D-03	0.488424D-04	0.543903D-04	0.820411D-04
28	0.255060D-04	-0.173069D-04	-0.813182D-04	0.310221D-04	0.523601D-04
29	-0.447372D-05	0.189739D-04	-0.497017D-04	0.388928D-05	-0.163806D-04
30	-0.523529D-05	0.406021D-05	0.117621D-03	0.288970D-05	0.813347D-04
31	0.261951D-05	-0.130218D-04	-0.114960D-03	-0.204428D-04	0.106671D-04
32	-0.580432D-05	0.228842D-04	-0.134131D-03	-0.359473D-04	0.329017D-05
33	0.387279D-04	0.520010D-04	0.118981D-03	0.172875D-04	0.119314D-04
34	-0.495298D-04	0.286637D-04	-0.176773D-03	-0.148143D-03	-0.285421D-04
35	-0.419752D-05	0.534776D-04	0.523040D-04	0.608369D-04	-0.115353D-04
36	-0.120692D-03	0.260084D-03	0.104291D-02	-0.788241D-04	-0.380417D-03
37	-0.659904D-04	0.185062D-03	0.114929D-02	0.962151D-04	-0.446792D-03
38	0.297510D-04	0.841953D-04	0.288887D-03	0.128320D-03	0.553261D-04
39	0.720392D-05	-0.371980D-04	-0.344942D-04	0.103095D-04	0.242470D-05
40	-0.427405D-05	0.299451D-04	0.888588D-04	0.883166D-05	-0.204930D-04
41	-0.447229D-04	-0.139533D-04	-0.308375D-04	-0.413135D-04	-0.271844D-04
42	-0.853137D-04	-0.583688D-05	0.115237D-04	0.103185D-03	-0.546519D-03

ESTIMATED COVARIANCE MATRIX FOR PARAMETER ESTIMATES

	11	12	13	14	15
11	0.161158D-02				
12	0.302937D-03	0.184579D-02			
13	-0.333882D-05	-0.181314D-04	0.887203D-03		
14	0.108495D-04	0.287017D-04	0.144168D-03	0.209517D-03	
15	-0.400411D-05	0.320134D-05	0.245882D-04	0.632896D-04	0.178540D-03
16	0.778415D-05	-0.380072D-04	-0.152795D-03	-0.702363D-04	-0.312474D-04
17	0.398044D-04	-0.174851D-04	-0.515465D-04	-0.385931D-04	-0.503970D-04
18	-0.479642D-04	0.956121D-04	0.342412D-04	0.115829D-04	-0.743436D-05
19	-0.208298D-04	0.927271D-05	0.671993D-04	0.296124D-04	0.604346D-05
20	-0.325044D-04	-0.661685D-05	0.115442D-04	0.103524D-04	0.370391D-04
21	0.304416D-04	0.116278D-04	-0.192582D-04	-0.531529D-05	-0.923290D-05
22	0.172695D-05	0.845336D-05	0.110005D-04	-0.118893D-04	0.892717D-05
23	0.109350D-04	0.418754D-04	0.167284D-03	0.404970D-04	0.601199D-05
24	-0.240241D-04	0.121050D-04	0.306065D-04	0.384780D-04	0.344864D-04
25	0.345648D-04	-0.329399D-04	-0.228235D-04	-0.347875D-04	-0.407283D-05
26	-0.392725D-05	0.178142D-04	0.650927D-05	0.160553D-05	0.119161D-04
27	0.437516D-04	0.809063D-04	0.386216D-04	-0.102869D-04	-0.152035D-04
28	-0.104864D-04	-0.207003D-04	-0.849150D-04	-0.497665D-04	-0.142780D-04
29	0.348659D-04	0.217049D-04	-0.125976D-04	-0.204254D-04	-0.363760D-04
30	0.417510D-06	-0.574673D-07	0.759248D-04	0.268147D-04	0.103357D-04
31	0.295505D-05	0.446685D-06	-0.110000D-04	-0.694664D-05	-0.112200D-04
32	-0.969991D-05	0.207903D-04	0.792511D-05	-0.177103D-04	-0.679923D-06
33	0.845626D-06	0.345522D-05	0.344622D-04	0.562352D-05	0.132042D-05
34	-0.428275D-05	-0.421241D-05	0.449452D-05	0.644652D-05	0.224759D-04
35	-0.182040D-04	0.876921D-04	-0.388785D-04	-0.373261D-04	-0.490661D-04
36	-0.245269D-03	0.697305D-04	0.365714D-04	-0.199297D-04	0.118815D-04
37	-0.135235D-03	0.360563D-03	0.128181D-03	0.248733D-04	0.299805D-04
38	0.129684D-03	0.902621D-04	0.874277D-04	0.487928D-04	0.130069D-04
39	0.424634D-04	0.188495D-04	-0.231895D-04	0.624119D-05	0.201012D-04
40	-0.211146D-04	-0.269459D-04	0.411600D-04	0.180852D-04	-0.441162D-05
41	-0.491559D-04	0.139317D-04	-0.238129D-04	-0.118816D-04	0.700994D-05
42	-0.202138D-03	0.242572D-03	0.122234D-03	-0.106479D-04	-0.686529D-05

ESTIMATED COVARIANCE MATRIX FOR PARAMETER ESTIMATES

	16	17	18	19	20
16	0.769023D-03				
17	0.156220D-03	0.354735D-03			
18	-0.227033D-03	-0.189496D-03	0.243851D-02		
19	-0.865755D-04	-0.203839D-04	0.315684D-04	0.382768D-03	

20	-0.222579D-04	-0.469605D-04	-0.745564D-05	0.626890D-04	0.180145D-03
21	0.291711D-04	0.785635D-04	-0.291510D-03	-0.493614D-04	-0.695887D-04
22	-0.111506D-04	-0.191057D-04	0.505279D-04	0.403942D-04	0.785291D-04
23	-0.105874D-03	-0.310519D-04	0.594953D-04	0.140422D-03	0.267940D-04
24	-0.243760D-04	-0.662525D-04	0.113869D-04	0.237718D-04	0.630437D-04
25	0.126729D-03	0.658481D-04	-0.445683D-03	-0.697037D-06	-0.137481D-04
26	-0.290299D-05	-0.194880D-04	0.621654D-04	0.585994D-04	0.497118D-04
27	-0.685374D-05	-0.268158D-04	0.178435D-03	0.227626D-04	0.209103D-04
28	0.981788D-04	0.245375D-04	-0.700843D-04	-0.123493D-03	-0.325835D-04
29	0.251583D-04	0.505806D-04	0.575691D-05	-0.167924D-04	-0.546241D-04
30	-0.612891D-04	-0.711088D-04	0.278648D-03	0.387184D-04	0.125651D-04
31	0.719329D-05	0.232516D-04	-0.154680D-05	-0.352416D-04	-0.495727D-04
32	0.706190D-05	0.326913D-05	-0.324011D-04	-0.122235D-04	-0.576785D-05
33	-0.293562D-05	-0.142432D-04	-0.379930D-04	-0.542256D-07	0.177153D-04
34	0.463216D-04	-0.560077D-04	0.124092D-03	0.413382D-04	-0.163250D-06
35	0.119098D-05	0.780854D-04	-0.611796D-04	-0.231919D-04	-0.322494D-04
36	0.179443D-03	0.183297D-03	-0.185851D-03	0.398342D-04	0.561458D-04
37	0.845966D-04	0.878890D-04	-0.116349D-03	0.221645D-04	0.134803D-03
38	-0.328610D-04	-0.158439D-04	0.107532D-03	0.175739D-05	0.403514D-05
39	-0.773914D-05	-0.295857D-04	0.129103D-04	-0.273920D-04	-0.232139D-04
40	-0.186220D-04	0.187971D-05	0.214567D-04	0.276973D-04	0.129941D-04
41	0.193630D-04	-0.296199D-05	-0.964260D-04	-0.347404D-04	-0.171330D-04
42	0.855706D-04	0.113520D-03	-0.190330D-03	0.708810D-04	0.875948D-04

ESTIMATED COVARIANCE MATRIX FOR PARAMETER ESTIMATES

	21	22	23	24	25
21	0.679763D-03				
22	-0.137480D-03	0.652675D-03			
23	-0.300008D-04	0.352269D-04	0.732779D-03		
24	-0.239364D-04	0.149473D-04	0.119152D-03	0.338056D-03	
25	0.221868D-03	-0.480911D-04	-0.103083D-03	-0.980351D-04	0.129239D-02
26	-0.139735D-03	0.196813D-03	0.696429D-04	0.830331D-04	-0.137377D-03
27	-0.645420D-04	0.875112D-04	0.222553D-03	0.134558D-03	-0.394987D-03
28	0.286525D-04	-0.260079D-04	-0.206203D-03	-0.374963D-04	0.515302D-04
29	0.958080D-05	-0.238873D-04	-0.251859D-04	-0.784263D-04	0.282819D-04
30	-0.168756D-03	0.559358D-04	0.104494D-03	0.317068D-04	-0.300929D-03
31	0.801572D-04	-0.186278D-03	-0.155612D-04	-0.182870D-04	0.401996D-04
32	0.216876D-04	-0.449318D-04	-0.836151D-04	-0.752821D-04	0.166215D-03
33	-0.190499D-04	0.252375D-04	0.296101D-04	0.414061D-04	-0.418953D-04
34	-0.929585D-04	0.366244D-04	-0.270224D-04	0.253256D-04	-0.685861D-04
35	0.799979D-04	0.139078D-04	0.257934D-04	-0.154364D-04	-0.304930D-04
36	0.551728D-04	0.617383D-04	-0.189554D-03	0.327306D-04	0.321982D-04

37	0.284151D-04	0.162745D-03	-0.517605D-04	0.314535D-05	0.170799D-04
38	0.174812D-04	0.135392D-04	0.382226D-04	0.143197D-04	0.342967D-05
39	0.244351D-04	0.233685D-04	0.107982D-04	0.207435D-04	-0.901312D-05
40	-0.292339D-05	-0.309727D-04	-0.571076D-05	-0.239880D-04	0.464931D-04
41	0.799358D-05	0.395477D-05	-0.936124D-05	0.238564D-04	-0.268407D-04
42	0.617925D-04	0.653454D-04	-0.229309D-04	0.293786D-04	0.663819D-04

ESTIMATED COVARIANCE MATRIX FOR PARAMETER ESTIMATES

	26	27	28	29	30
26	0.650042D-03				
27	0.437868D-03	0.241815D-02			
28	-0.322584D-04	-0.687200D-04	0.686017D-03		
29	-0.368514D-04	-0.517779D-04	0.112367D-03	0.287860D-03	
30	0.842621D-04	0.150283D-03	-0.120971D-03	-0.785464D-04	0.112439D-02
31	-0.185725D-03	-0.123267D-03	0.743724D-04	0.729877D-04	-0.167886D-03
32	-0.234912D-03	-0.575809D-03	0.152305D-03	0.920734D-04	-0.231369D-03
33	0.832382D-04	0.153437D-03	-0.160455D-03	-0.131425D-03	0.271585D-03
34	0.319496D-04	-0.191123D-04	0.116788D-04	-0.731433D-06	0.285102D-04
35	0.231558D-04	-0.292501D-04	-0.220769D-04	0.111008D-04	0.129634D-04
36	0.108204D-03	-0.388513D-04	0.280342D-04	-0.184745D-04	-0.134917D-03
37	0.105334D-03	-0.283921D-03	-0.146976D-03	-0.287556D-04	-0.245190D-04
38	-0.360249D-04	-0.183402D-04	-0.543147D-04	-0.111943D-04	0.786283D-04
39	0.227889D-04	0.964838D-05	-0.139721D-04	-0.176100D-04	0.198007D-04
40	-0.289683D-04	-0.586032D-04	0.869431D-05	-0.389333D-05	-0.151144D-04
41	0.531660D-04	0.792157D-04	0.237439D-04	0.245898D-04	-0.328725D-04
42	0.407651D-04	-0.150897D-03	-0.833832D-04	-0.212208D-04	-0.165147D-04

ESTIMATED COVARIANCE MATRIX FOR PARAMETER ESTIMATES

	31	32	33	34	35
31	0.597737D-03				
32	0.241932D-03	0.115790D-02			
33	-0.349923D-03	-0.537269D-03	0.195420D-02		
34	0.264938D-04	-0.862556D-04	0.239201D-04	0.393977D-02	
35	-0.289242D-04	0.290030D-04	-0.584262D-06	-0.251807D-02	0.499533D-02
36	-0.721495D-04	-0.185374D-04	-0.292864D-03	0.738000D-04	-0.136985D-03
37	-0.175247D-03	-0.108907D-03	0.559835D-04	0.376540D-04	-0.263236D-03
38	0.455754D-05	-0.240458D-04	0.443934D-04	-0.249576D-04	0.301582D-04
39	0.285810D-04	-0.392519D-04	0.316378D-05	-0.953659D-04	0.392639D-04
40	-0.371446D-04	0.360840D-04	0.423977D-04	0.120177D-03	-0.722946D-04
41	0.588565D-04	-0.169737D-04	-0.569866D-04	-0.102482D-03	0.737389D-04

42	-0.103511D-03	-0.159621D-04	-0.109423D-03	0.113979D-03	-0.257916D-03

ESTIMATED COVARIANCE MATRIX FOR PARAMETER ESTIMATES

	36	37	38	39	40
36	0.257342D-01				
37	0.163702D-01	0.230273D-01			
38	-0.475416D-03	0.534091D-03	0.162781D-02		
39	-0.674209D-04	0.232548D-04	-0.983552D-05	0.202930D-02	
40	0.131071D-03	-0.215536D-04	-0.474598D-05	-0.123290D-02	0.143881D-02
41	-0.698813D-05	0.229771D-04	-0.693246D-04	0.771142D-03	-0.130200D-02
42	0.166046D-01	0.165489D-01	0.502705D-04	0.483595D-04	0.428982D-04

ESTIMATED COVARIANCE MATRIX FOR PARAMETER ESTIMATES

	41	42
41	0.245975D-02	
42	-0.502649D-04	0.169554D-01

ESTIMATED CORRELATION MATRIX FOR PARAMETER ESTIMATES

	1	2	3	4	5
1	1.000				
2	0.256	1.000			
3	-0.113	-0.117	1.000		
4	0.079	0.190	-0.176	1.000	
5	0.124	0.081	-0.196	0.230	1.000
6	-0.056	-0.096	0.099	-0.234	-0.258
7	-0.021	-0.017	-0.040	-0.009	0.014
8	0.028	-0.005	0.025	-0.006	-0.020
9	0.014	0.019	0.031	0.020	0.049
10	-0.007	0.029	0.018	0.007	0.019
11	0.036	0.015	-0.014	-0.014	0.009
12	0.001	0.022	-0.013	0.015	-0.064
13	0.083	0.043	-0.018	0.001	0.033
14	-0.003	0.049	-0.006	-0.045	-0.041
15	0.018	0.006	0.044	-0.053	-0.022
16	-0.064	-0.046	0.002	0.011	-0.019
17	0.001	0.035	-0.013	0.000	0.022
18	-0.032	-0.002	-0.021	0.007	0.013
19	0.021	0.054	0.024	-0.015	-0.020

20	0.016	0.010	0.001	0.030	0.016
21	0.013	-0.019	0.013	0.002	-0.034
22	0.027	0.010	-0.007	0.032	0.067
23	0.038	0.028	0.009	-0.024	0.025
24	0.010	0.007	0.027	0.022	0.026
25	-0.003	-0.006	-0.015	0.022	-0.004
26	-0.005	-0.028	0.002	0.040	0.036
27	0.028	-0.006	-0.015	0.016	0.029
28	-0.015	-0.048	-0.010	-0.056	-0.023
29	-0.005	0.006	-0.019	-0.010	0.035
30	-0.007	-0.023	-0.015	0.013	0.042
31	-0.015	0.004	0.038	-0.032	-0.006
32	-0.051	0.007	0.064	-0.043	-0.010
33	-0.001	0.008	-0.028	0.026	-0.005
34	-0.006	-0.014	0.004	0.052	0.009
35	0.015	0.015	-0.029	-0.039	0.002
36	-0.018	-0.014	0.003	0.048	0.022
37	-0.008	0.034	-0.005	0.083	0.058
38	0.005	0.008	0.011	0.064	-0.009
39	-0.002	0.013	0.000	0.003	0.011
40	0.016	0.007	-0.012	0.004	-0.031
41	-0.025	-0.016	-0.013	-0.011	0.052
42	-0.001	0.016	0.002	0.076	0.057

ESTIMATED CORRELATION MATRIX FOR PARAMETER ESTIMATES

	6	7	8	9	10
6	1.000				
7	-0.004	1.000			
8	-0.006	-0.116	1.000		
9	0.005	0.041	0.125	1.000	
10	-0.001	0.002	-0.042	0.068	1.000
11	-0.013	-0.135	-0.032	0.030	-0.121
12	0.014	-0.003	0.107	-0.107	0.125
13	-0.018	0.050	0.004	0.006	-0.012
14	0.039	0.036	-0.029	0.008	0.027
15	0.017	-0.012	-0.013	0.026	0.045
16	-0.025	0.003	0.003	0.010	0.002
17	-0.035	0.002	-0.013	-0.013	0.005
18	-0.029	0.022	-0.013	-0.009	0.012
19	0.020	-0.014	-0.003	-0.001	-0.022
20	0.008	-0.014	0.052	0.012	-0.015
21	0.006	0.004	-0.059	-0.030	0.007

22	-0.043	0.013	0.026	-0.004	-0.017
23	-0.005	-0.007	0.003	-0.016	-0.019
24	-0.006	0.013	-0.015	0.027	0.014
25	-0.020	0.016	-0.041	-0.015	-0.024
26	-0.003	0.022	0.041	0.042	0.002
27	-0.009	0.047	0.010	0.029	0.028
28	0.033	-0.013	-0.033	0.031	0.034
29	-0.009	0.022	-0.031	0.006	-0.016
30	-0.005	0.002	0.037	0.002	0.041
31	0.004	-0.010	-0.050	-0.022	0.007
32	-0.006	0.013	-0.042	-0.027	0.002
33	0.030	0.023	0.028	0.010	0.005
34	-0.027	0.009	-0.030	-0.061	-0.008
35	-0.002	0.015	0.008	0.022	-0.003
36	-0.025	0.032	0.069	-0.013	-0.040
37	-0.015	0.024	0.080	0.016	-0.050
38	0.025	0.041	0.075	0.082	0.023
39	0.005	-0.016	-0.008	0.006	0.001
40	-0.004	0.016	0.025	0.006	-0.009
41	-0.030	-0.006	-0.007	-0.021	-0.009
42	-0.022	-0.001	0.001	0.020	-0.071

ESTIMATED CORRELATION MATRIX FOR PARAMETER ESTIMATES

	11	12	13	14	15
11	1.000				
12	0.176	1.000			
13	-0.003	-0.014	1.000		
14	0.019	0.046	0.334	1.000	
15	-0.007	0.006	0.062	0.327	1.000
16	0.007	-0.032	-0.185	-0.175	-0.084
17	0.053	-0.022	-0.092	-0.142	-0.200
18	-0.024	0.045	0.023	0.016	-0.011
19	-0.027	0.011	0.115	0.105	0.023
20	-0.060	-0.011	0.029	0.053	0.207
21	0.029	0.010	-0.025	-0.014	-0.027
22	0.002	0.008	0.014	-0.032	0.026
23	0.010	0.036	0.207	0.103	0.017
24	-0.033	0.015	0.056	0.145	0.140
25	0.024	-0.021	-0.021	-0.067	-0.008
26	-0.004	0.016	0.009	0.004	0.035
27	0.022	0.038	0.026	-0.014	-0.023
28	-0.010	-0.018	-0.109	-0.131	-0.041

29	0.051	0.030	-0.025	-0.083	-0.160
30	0.000	0.000	0.076	0.055	0.023
31	0.003	0.000	-0.015	-0.020	-0.034
32	-0.007	0.014	0.008	-0.036	-0.001
33	0.000	0.002	0.026	0.009	0.002
34	-0.002	-0.002	0.002	0.007	0.027
35	-0.006	0.029	-0.018	-0.036	-0.052
36	-0.038	0.010	0.008	-0.009	0.006
37	-0.022	0.055	0.028	0.011	0.015
38	0.080	0.052	0.073	0.084	0.024
39	0.023	0.010	-0.017	0.010	0.033
40	-0.014	-0.017	0.036	0.033	-0.009
41	-0.025	0.007	-0.016	-0.017	0.011
42	-0.039	0.043	0.032	-0.006	-0.004

ESTIMATED CORRELATION MATRIX FOR PARAMETER ESTIMATES

	16	17	18	19	20
16	1.000				
17	0.299	1.000			
18	-0.166	-0.204	1.000		
19	-0.160	-0.055	0.033	1.000	
20	-0.060	-0.186	-0.011	0.239	1.000
21	0.040	0.160	-0.226	-0.097	-0.199
22	-0.016	-0.040	0.040	0.081	0.229
23	-0.141	-0.061	0.045	0.265	0.074
24	-0.048	-0.191	0.013	0.066	0.255
25	0.127	0.097	-0.251	-0.001	-0.028
26	-0.004	-0.041	0.049	0.117	0.145
27	-0.005	-0.029	0.073	0.024	0.032
28	0.135	0.050	-0.054	-0.241	-0.093
29	0.053	0.158	0.007	-0.051	-0.240
30	-0.066	-0.113	0.168	0.059	0.028
31	0.011	0.050	-0.001	-0.074	-0.151
32	0.007	0.005	-0.019	-0.018	-0.013
33	-0.002	-0.017	-0.017	0.000	0.030
34	0.027	-0.047	0.040	0.034	0.000
35	0.001	0.059	-0.018	-0.017	-0.034
36	0.040	0.061	-0.023	0.013	0.026
37	0.020	0.031	-0.016	0.007	0.066
38	-0.029	-0.021	0.054	0.002	0.007
39	-0.006	-0.035	0.006	-0.031	-0.038
40	-0.018	0.003	0.011	0.037	0.026

41	0.014	-0.003	-0.039	-0.036	-0.026
42	0.024	0.046	-0.030	0.028	0.050

ESTIMATED CORRELATION MATRIX FOR PARAMETER ESTIMATES

	21	22	23	24	25
21	1.000				
22	-0.206	1.000			
23	-0.043	0.051	1.000		
24	-0.050	0.032	0.239	1.000	
25	0.237	-0.052	-0.106	-0.148	1.000
26	-0.210	0.302	0.101	0.177	-0.150
27	-0.050	0.070	0.167	0.149	-0.223
28	0.042	-0.039	-0.291	-0.078	0.055
29	0.022	-0.055	-0.055	-0.251	0.046
30	-0.193	0.065	0.115	0.051	-0.250
31	0.126	-0.298	-0.024	-0.041	0.046
32	0.024	-0.052	-0.091	-0.120	0.136
33	-0.017	0.022	0.025	0.051	-0.026
34	-0.057	0.023	-0.016	0.022	-0.030
35	0.043	0.008	0.013	-0.012	-0.012
36	0.013	0.015	-0.044	0.011	0.006
37	0.007	0.042	-0.013	0.001	0.003
38	0.017	0.013	0.035	0.019	0.002
39	0.021	0.020	0.009	0.025	-0.006
40	-0.003	-0.032	-0.006	-0.034	0.034
41	0.006	0.003	-0.007	0.026	-0.015
42	0.018	0.020	-0.007	0.012	0.014

ESTIMATED CORRELATION MATRIX FOR PARAMETER ESTIMATES

	26	27	28	29	30
26	1.000				
27	0.349	1.000			
28	-0.048	-0.053	1.000		
29	-0.085	-0.062	0.253	1.000	
30	0.099	0.091	-0.138	-0.138	1.000
31	-0.298	-0.103	0.116	0.176	-0.205
32	-0.271	-0.344	0.171	0.159	-0.203
33	0.074	0.071	-0.139	-0.175	0.183
34	0.020	-0.006	0.007	-0.001	0.014
35	0.013	-0.008	-0.012	0.009	0.005

36	0.026	-0.005	0.007	-0.007	-0.025
37	0.027	-0.038	-0.037	-0.011	-0.005
38	-0.035	-0.009	-0.051	-0.016	0.058
39	0.020	0.004	-0.012	-0.023	0.013
40	-0.030	-0.031	0.009	-0.006	-0.012
41	0.042	0.032	0.018	0.029	-0.020
42	0.012	-0.024	-0.024	-0.010	-0.004

ESTIMATED CORRELATION MATRIX FOR PARAMETER ESTIMATES

	31	32	33	34	35
31	1.000				
32	0.291	1.000			
33	-0.324	-0.357	1.000		
34	0.017	-0.040	0.009	1.000	
35	-0.017	0.012	0.000	-0.568	1.000
36	-0.018	-0.003	-0.041	0.007	-0.012
37	-0.047	-0.021	0.008	0.004	-0.025
38	0.005	-0.018	0.025	-0.010	0.011
39	0.026	-0.026	0.002	-0.034	0.012
40	-0.040	0.028	0.025	0.050	-0.027
41	0.049	-0.010	-0.026	-0.033	0.021
42	-0.033	-0.004	-0.019	0.014	-0.028

ESTIMATED CORRELATION MATRIX FOR PARAMETER ESTIMATES

	36	37	38	39	40
36	1.000				
37	0.672	1.000			
38	-0.073	0.087	1.000		
39	-0.009	0.003	-0.005	1.000	
40	0.022	-0.004	-0.003	-0.722	1.000
41	-0.001	0.003	-0.035	0.345	-0.692
42	0.795	0.838	0.010	0.008	0.009

ESTIMATED CORRELATION MATRIX FOR PARAMETER ESTIMATES

	41	42
41	1.000	
42	-0.008	1.000

TECHNICAL 8 OUTPUT

TECHNICAL 8 OUTPUT FOR BAYES ESTIMATION

CHAIN	BSEED
1	0
2	285380
3	253358

ITERATION	POTENTIAL SCALE REDUCTION	PARAMETER WITH HIGHEST PSR
100	1.017	13
200	1.015	38
300	1.005	13
400	1.004	37
500	1.009	37
600	1.005	38
700	1.002	42
800	1.004	42
900	1.001	42
1000	1.000	20

This is the interaction between latent profile categories and job control on worsening of physical health

SUMMARY OF DATA

Number of clusters 58

Size (s)	Cluster ID with Size s
7	22
16	166
23	80 71 205
25	74
27	192
31	65
32	106
35	228
37	170 72
39	50 75 181
40	182
42	165
44	114
45	173
46	107
47	169 30
48	226 81
49	36
50	116 108 40
56	162 84
57	61
59	206 93 83
60	92
61	69
63	6
64	152
65	145
66	4
68	68
69	154
72	174
78	98
84	178 2
95	156 202
96	144
108	142

131	122
172	24
175	209
224	204
269	125
364	217
738	110
860	100

COVARIANCE COVERAGE OF DATA

Minimum covariance coverage value 0.100

Number of missing data patterns 8

PROPORTION OF DATA PRESENT

Covariance Coverage

	PHYCAT16	CLU2	CLU3	PHY15	KES15
PHYCAT16	1.000				
CLU2	1.000	1.000			
CLU3	1.000	1.000	1.000		
PHY15	0.947	0.947	0.947	0.947	
KES15	0.922	0.922	0.922	0.922	0.922
CHOICE15	0.915	0.915	0.915	0.915	0.915
BULLY15	0.915	0.915	0.915	0.915	0.915
PRESS15	0.915	0.915	0.915	0.915	0.915
DUTIES15	0.915	0.915	0.915	0.915	0.915

Covariance Coverage

	CHOICE15	BULLY15	PRESS15	DUTIES15
CHOICE15	0.915			
BULLY15	0.915	0.915		
PRESS15	0.915	0.914	0.915	
DUTIES15	0.915	0.915	0.915	0.915

UNIVARIATE PROPORTIONS AND COUNTS FOR CATEGORICAL VARIABLES

PHYCAT16		
Category 1	0.881	4884.000
Category 2	0.119	662.000

UNIVARIATE SAMPLE STATISTICS

UNIVARIATE HIGHER-ORDER MOMENT DESCRIPTIVE STATISTICS

Variable/ Sample Size	Mean/ Variance	Skewness/ Kurtosis	Minimum/ Maximum	% with Min/Max	Percentiles 20%/60%	40%/80%	Median
CLU2	0.310	0.820	0.000	68.97%	0.000	0.000	0.000
58.000	0.214	-1.328	1.000	31.03%	0.000	1.000	
CLU3	0.448	0.208	0.000	55.17%	0.000	0.000	0.000
58.000	0.247	-1.957	1.000	44.83%	1.000	1.000	
PHY15	0.000	-0.546	-0.632	36.83%	-0.632	0.368	0.368
5254.000	0.233	-1.702	0.368	63.17%	0.368	0.368	
KES15	0.000	1.717	-0.511	21.65%	-0.511	-0.178	-0.178
5114.000	0.289	3.835	3.489	0.04%	-0.011	0.322	
CHOICE15	3.225	-0.402	1.000	7.41%	2.000	3.000	3.000
5077.000	1.081	-0.336	5.000	8.49%	4.000	4.000	
BULLY15	0.000	3.272	-0.170	87.25%	-0.170	-0.170	-0.170
5073.000	0.239	11.772	3.830	0.08%	-0.170	-0.170	
PRESS15	0.000	0.252	-1.462	19.10%	-0.462	-0.462	-0.462
5074.000	1.001	-0.371	2.538	2.76%	0.538	0.538	
DUTIES15	0.000	-1.246	-3.120	2.40%	-0.120	-0.120	-0.120
5077.000	0.861	1.673	0.880	38.74%	-0.120	0.880	

THE MODEL ESTIMATION TERMINATED NORMALLY

USE THE FBITERATIONS OPTION TO INCREASE THE NUMBER OF ITERATIONS BY A FACTOR
OF AT LEAST TWO TO CHECK CONVERGENCE AND THAT THE PSR VALUE DOES NOT INCREASE.

MODEL FIT INFORMATION

Number of Free Parameters   49

MODEL RESULTS

	Estimate	Posterior S.D.	One-Tailed P-Value	95% C.I. Lower 2.5%	95% C.I. Upper 2.5%	Significance
**Within Level**						
PHYCAT16 ON						
PHY15	0.967	0.069	0.000	0.838	1.107	*
BULLY15	0.027	0.048	0.294	-0.069	0.121	
DUTIES15	-0.032	0.027	0.117	-0.086	0.020	
PRESS15	-0.033	0.026	0.105	-0.084	0.019	
KES15	0.230	0.044	0.000	0.142	0.318	*
KES15 WITH						
PHY15	0.051	0.004	0.000	0.044	0.059	*
CHOICE15	-0.088	0.008	0.000	-0.103	-0.073	*
BULLY15	0.057	0.004	0.000	0.050	0.065	*
PRESS15	0.100	0.008	0.000	0.085	0.114	*
DUTIES15	-0.106	0.007	0.000	-0.119	-0.091	*
PHY15 WITH						
CHOICE15	-0.033	0.007	0.000	-0.046	-0.019	*
BULLY15	0.012	0.003	0.000	0.005	0.019	*
PRESS15	0.039	0.007	0.000	0.026	0.052	*
DUTIES15	-0.036	0.006	0.000	-0.048	-0.024	*
CHOICE15 WITH						
BULLY15	-0.056	0.007	0.000	-0.069	-0.042	*
PRESS15	-0.076	0.015	0.000	-0.105	-0.047	*
DUTIES15	0.168	0.014	0.000	0.141	0.196	*
BULLY15 WITH						
PRESS15	0.105	0.007	0.000	0.091	0.119	*
DUTIES15	-0.078	0.006	0.000	-0.090	-0.065	*
PRESS15 WITH						
DUTIES15	-0.186	0.014	0.000	-0.212	-0.161	*
Means						
PHY15	0.003	0.007	0.350	-0.010	0.015	

KES15	0.001	0.007	0.423	-0.013	0.017	
BULLY15	0.001	0.007	0.439	-0.013	0.015	
PRESS15	0.001	0.014	0.482	-0.029	0.029	
DUTIES15	-0.001	0.013	0.453	-0.027	0.024	
Variances						
PHY15	0.233	0.005	0.000	0.224	0.243	*
KES15	0.289	0.006	0.000	0.279	0.301	*
CHOICE15	1.040	0.022	0.000	1.000	1.082	*
BULLY15	0.239	0.005	0.000	0.230	0.249	*
PRESS15	1.003	0.020	0.000	0.965	1.044	*
DUTIES15	0.861	0.017	0.000	0.828	0.896	*
Between Level						
S1 ON						
CLU2	0.114	0.090	0.088	-0.058	0.290	
CLU3	0.024	0.084	0.373	-0.136	0.192	
PHYCAT16 ON						
CLU2	-0.183	0.100	0.021	-0.402	-0.008	*
CLU3	-0.009	0.093	0.461	-0.197	0.171	
CHOICE15	-0.114	0.171	0.228	-0.462	0.219	
CHOICE15 WITH						
CLU2	0.022	0.018	0.094	-0.012	0.062	
CLU3	-0.014	0.019	0.207	-0.055	0.023	
CLU2 WITH						
CLU3	-0.137	0.038	0.000	-0.228	-0.079	*
Means						
CLU2	0.309	0.064	0.000	0.182	0.440	*
CLU3	0.447	0.069	0.000	0.306	0.579	*
CHOICE15	3.309	0.038	0.000	3.235	3.387	*
Intercepts						
S1	-0.111	0.072	0.057	-0.250	0.041	
Thresholds						
PHYCAT16$1	0.902	0.565	0.055	-0.263	2.022	
Variances						
CLU2	0.230	0.044	0.000	0.163	0.334	*

CLU3	0.262	0.052	0.000	0.189	0.391	*
CHOICE15	0.060	0.013	0.000	0.040	0.092	*
**Residual Variances**						
PHYCAT16	0.016	0.013	0.000	0.002	0.052	*
S1	0.004	0.006	0.000	0.000	0.020	*

STANDARDIZED MODEL RESULTS

STDYX Standardization

	Estimate	Posterior S.D.	One-Tailed P-Value	95% C.I. Lower 2.5%	Upper 2.5%	Significance
**Within-Level Standardized Estimates Averaged Over Clusters**						
**S1 | PHYCAT16 ON**						
CHOICE15	-0.056	0.027	0.022	-0.108	-0.002	*
**PHYCAT16 ON**						
PHY15	0.414	0.024	0.000	0.365	0.461	*
BULLY15	0.011	0.021	0.302	-0.032	0.053	
DUTIES15	-0.026	0.023	0.124	-0.071	0.018	
PRESS15	-0.029	0.023	0.107	-0.074	0.017	
KES15	0.109	0.021	0.000	0.067	0.150	*
**KES15 WITH**						
PHY15	0.198	0.014	0.000	0.171	0.225	*
CHOICE15	-0.160	0.014	0.000	-0.188	-0.134	*
BULLY15	0.219	0.013	0.000	0.192	0.244	*
PRESS15	0.186	0.013	0.000	0.159	0.212	*
DUTIES15	-0.212	0.013	0.000	-0.237	-0.186	*
**PHY15 WITH**						
CHOICE15	-0.066	0.014	0.000	-0.093	-0.038	*
BULLY15	0.050	0.014	0.000	0.021	0.078	*
PRESS15	0.080	0.014	0.000	0.052	0.106	*
DUTIES15	-0.080	0.014	0.000	-0.108	-0.054	*
**CHOICE15 WITH**						
BULLY15	-0.112	0.014	0.000	-0.139	-0.084	*
PRESS15	-0.075	0.014	0.000	-0.101	-0.047	*

```
    DUTIES15           0.178      0.014      0.000       0.150       0.205      *

 BULLY15  WITH
    PRESS15            0.214      0.013      0.000       0.188       0.240      *
    DUTIES15          -0.171      0.013      0.000      -0.198      -0.144      *

 PRESS15  WITH
    DUTIES15          -0.200      0.014      0.000      -0.227      -0.173      *

 Means
    PHY15              0.005      0.014      0.367      -0.022       0.031
    KES15              0.002      0.014      0.424      -0.025       0.030
    BULLY15            0.001      0.014      0.458      -0.026       0.029
    PRESS15            0.001      0.014      0.485      -0.028       0.028
    DUTIES15          -0.002      0.014      0.450      -0.029       0.026

 Variances
    PHY15              1.000      0.000      0.000       1.000       1.000
    KES15              1.000      0.000      0.000       1.000       1.000
    CHOICE15           1.000      0.000      0.000       1.000       1.000
    BULLY15            1.000      0.000      0.000       1.000       1.000
    PRESS15            1.000      0.000      0.000       1.000       1.000
    DUTIES15           1.000      0.000      0.000       1.000       1.000

Between Level

 S1         ON
    CLU2               0.651      0.407      0.088      -0.326       1.187
    CLU3               0.143      0.455      0.373      -0.811       0.913

 PHYCAT16   ON
    CLU2              -0.534      0.246      0.021      -0.975      -0.030      *
    CLU3              -0.029      0.277      0.461      -0.555       0.547
    CHOICE15          -0.170      0.239      0.228      -0.601       0.337

 CHOICE15 WITH
    CLU2               0.196      0.141      0.094      -0.108       0.457
    CLU3              -0.119      0.143      0.207      -0.392       0.166

 CLU2     WITH
    CLU3              -0.565      0.086      0.000      -0.718      -0.377      *

 Means
    CLU2               0.649      0.143      0.000       0.364       0.924      *
```

```
    CLU3               0.878       0.156       0.000       0.558       1.166      *
    CHOICE15          13.456       1.456       0.000      10.959      16.608      *

 Intercepts
    S1                -1.268       0.833       0.057      -2.888       0.411

 Variances
    CLU2               1.000       0.000       0.000       1.000       1.000
    CLU3               1.000       0.000       0.000       1.000       1.000
    CHOICE15           1.000       0.000       0.000       1.000       1.000

 Residual Variances
    PHYCAT16           0.556       0.190       0.000       0.158       0.893      *
    S1                 0.514       0.274       0.000       0.021       0.971      *


R-SQUARE

Within-Level R-Square Averaged Across Clusters

                                Posterior  One-Tailed         95% C.I.
    Variable        Estimate       S.D.      P-Value   Lower 2.5%  Upper 2.5%

    PHYCAT16           0.220       0.021       0.000       0.180       0.264

Between Level

                                Posterior  One-Tailed         95% C.I.
    Variable        Estimate       S.D.      P-Value   Lower 2.5%  Upper 2.5%

    PHYCAT16           0.444       0.190       0.000       0.107       0.842

                                Posterior  One-Tailed         95% C.I.
    Variable        Estimate       S.D.      P-Value   Lower 2.5%  Upper 2.5%

    S1                 0.486       0.274       0.000       0.029       0.979


TECHNICAL 1 OUTPUT


     PARAMETER SPECIFICATION FOR WITHIN
```

TAU

	PHYCAT16
	0

NU

PHYCAT16	PHY15	KES15	CHOICE15	BULLY15
0	0	0	0	0

NU

PRESS15	DUTIES15
0	0

LAMBDA

	PHYCAT16	PHY15	KES15	CHOICE15	BULLY15
PHYCAT16	0	0	0	0	0
PHY15	0	0	0	0	0
KES15	0	0	0	0	0
CHOICE15	0	0	0	0	0
BULLY15	0	0	0	0	0
PRESS15	0	0	0	0	0
DUTIES15	0	0	0	0	0

LAMBDA

	PRESS15	DUTIES15
PHYCAT16	0	0
PHY15	0	0
KES15	0	0
CHOICE15	0	0
BULLY15	0	0
PRESS15	0	0
DUTIES15	0	0

THETA

PHYCAT16 PHY15 KES15 CHOICE15 BULLY15

PHYCAT16	0				
PHY15	0	0			
KES15	0	0	0		
CHOICE15	0	0	0	0	
BULLY15	0	0	0	0	0
PRESS15	0	0	0	0	0
DUTIES15	0	0	0	0	0

THETA

	PRESS15	DUTIES15
PRESS15	0	
DUTIES15	0	0

ALPHA

PHYCAT16	PHY15	KES15	CHOICE15	BULLY15
0	1	2	0	3

ALPHA

PRESS15	DUTIES15
4	5

BETA

	PHYCAT16	PHY15	KES15	CHOICE15	BULLY15
PHYCAT16	0	6	7	0	8
PHY15	0	0	0	0	0
KES15	0	0	0	0	0
CHOICE15	0	0	0	0	0
BULLY15	0	0	0	0	0
PRESS15	0	0	0	0	0
DUTIES15	0	0	0	0	0

BETA

PRESS15	DUTIES15

PHYCAT16	9	10
PHY15	0	0
KES15	0	0
CHOICE15	0	0
BULLY15	0	0
PRESS15	0	0
DUTIES15	0	0

PSI

	PHYCAT16	PHY15	KES15	CHOICE15	BULLY15
PHYCAT16	0				
PHY15	0	11			
KES15	0	12	13		
CHOICE15	0	14	15	16	
BULLY15	0	17	18	19	20
PRESS15	0	21	22	23	24
DUTIES15	0	26	27	28	29

PSI

	PRESS15	DUTIES15
PRESS15	25	
DUTIES15	30	31

PARAMETER SPECIFICATION FOR BETWEEN

TAU

PHYCAT16
49

NU

PHYCAT16	CLU2	CLU3	CHOICE15
0	0	0	0

LAMBDA

	S1	PHYCAT16	CLU2	CLU3	CHOICE15
PHYCAT16	0	0	0	0	0
CLU2	0	0	0	0	0
CLU3	0	0	0	0	0
CHOICE15	0	0	0	0	0

THETA

	PHYCAT16	CLU2	CLU3	CHOICE15
PHYCAT16	0			
CLU2	0	0		
CLU3	0	0	0	
CHOICE15	0	0	0	0

ALPHA

S1	PHYCAT16	CLU2	CLU3	CHOICE15
32	0	33	34	35

BETA

	S1	PHYCAT16	CLU2	CLU3	CHOICE15
S1	0	0	36	37	0
PHYCAT16	0	0	38	39	40
CLU2	0	0	0	0	0
CLU3	0	0	0	0	0
CHOICE15	0	0	0	0	0

PSI

	S1	PHYCAT16	CLU2	CLU3	CHOICE15
S1	41				
PHYCAT16	0	42			
CLU2	0	0	43		
CLU3	0	0	44	45	
CHOICE15	0	0	46	47	48

STARTING VALUES FOR WITHIN

TAU

PHYCAT16
0.000

NU

PHYCAT16	PHY15	KES15	CHOICE15	BULLY15
0.000	0.000	0.000	0.000	0.000

NU

PRESS15	DUTIES15
0.000	0.000

LAMBDA

	PHYCAT16	PHY15	KES15	CHOICE15	BULLY15
PHYCAT16	1.000	0.000	0.000	0.000	0.000
PHY15	0.000	1.000	0.000	0.000	0.000
KES15	0.000	0.000	1.000	0.000	0.000
CHOICE15	0.000	0.000	0.000	1.000	0.000
BULLY15	0.000	0.000	0.000	0.000	1.000
PRESS15	0.000	0.000	0.000	0.000	0.000
DUTIES15	0.000	0.000	0.000	0.000	0.000

LAMBDA

	PRESS15	DUTIES15
PHYCAT16	0.000	0.000
PHY15	0.000	0.000
KES15	0.000	0.000
CHOICE15	0.000	0.000
BULLY15	0.000	0.000
PRESS15	1.000	0.000
DUTIES15	0.000	1.000

THETA

	PHYCAT16	PHY15	KES15	CHOICE15	BULLY15
PHYCAT16	0.000				
PHY15	0.000	0.000			
KES15	0.000	0.000	0.000		
CHOICE15	0.000	0.000	0.000	0.000	
BULLY15	0.000	0.000	0.000	0.000	0.000
PRESS15	0.000	0.000	0.000	0.000	0.000
DUTIES15	0.000	0.000	0.000	0.000	0.000

THETA

	PRESS15	DUTIES15
PRESS15	0.000	
DUTIES15	0.000	0.000

ALPHA

PHYCAT16	PHY15	KES15	CHOICE15	BULLY15
0.000	0.000	0.000	0.000	0.000

ALPHA

PRESS15	DUTIES15
0.000	0.000

BETA

	PHYCAT16	PHY15	KES15	CHOICE15	BULLY15
PHYCAT16	0.000	0.000	0.000	0.000	0.000
PHY15	0.000	0.000	0.000	0.000	0.000
KES15	0.000	0.000	0.000	0.000	0.000
CHOICE15	0.000	0.000	0.000	0.000	0.000
BULLY15	0.000	0.000	0.000	0.000	0.000
PRESS15	0.000	0.000	0.000	0.000	0.000
DUTIES15	0.000	0.000	0.000	0.000	0.000

BETA

	PRESS15	DUTIES15
PHYCAT16	0.000	0.000
PHY15	0.000	0.000
KES15	0.000	0.000
CHOICE15	0.000	0.000
BULLY15	0.000	0.000
PRESS15	0.000	0.000
DUTIES15	0.000	0.000

PSI

	PHYCAT16	PHY15	KES15	CHOICE15	BULLY15
PHYCAT16	1.000				
PHY15	0.000	0.116			
KES15	0.000	0.000	0.145		
CHOICE15	0.000	0.000	0.000	0.541	
BULLY15	0.000	0.000	0.000	0.000	0.119
PRESS15	0.000	0.000	0.000	0.000	0.000
DUTIES15	0.000	0.000	0.000	0.000	0.000

PSI

	PRESS15	DUTIES15
PRESS15	0.501	
DUTIES15	0.000	0.430

STARTING VALUES FOR BETWEEN

TAU

PHYCAT16
1.110

NU

PHYCAT16	CLU2	CLU3	CHOICE15
0.000	0.000	0.000	0.000

LAMBDA

	S1	PHYCAT16	CLU2	CLU3	CHOICE15
PHYCAT16	0.000	1.000	0.000	0.000	0.000
CLU2	0.000	0.000	1.000	0.000	0.000
CLU3	0.000	0.000	0.000	1.000	0.000
CHOICE15	0.000	0.000	0.000	0.000	1.000

THETA

	PHYCAT16	CLU2	CLU3	CHOICE15
PHYCAT16	0.000			
CLU2	0.000	0.000		
CLU3	0.000	0.000	0.000	
CHOICE15	0.000	0.000	0.000	0.000

ALPHA

S1	PHYCAT16	CLU2	CLU3	CHOICE15
0.000	0.000	0.406	0.471	3.225

BETA

	S1	PHYCAT16	CLU2	CLU3	CHOICE15
S1	0.000	0.000	0.000	0.000	0.000
PHYCAT16	0.000	0.000	0.000	0.000	0.000
CLU2	0.000	0.000	0.000	0.000	0.000
CLU3	0.000	0.000	0.000	0.000	0.000
CHOICE15	0.000	0.000	0.000	0.000	0.000

PSI

	S1	PHYCAT16	CLU2	CLU3	CHOICE15
S1	1.000				
PHYCAT16	0.000	1.000			
CLU2	0.000	0.000	0.121		
CLU3	0.000	0.000	0.000	0.125	
CHOICE15	0.000	0.000	0.000	0.000	0.541

PRIORS FOR ALL PARAMETERS	PRIOR MEAN	PRIOR VARIANCE	PRIOR STD. DEV.
Parameter 1~N(0.000,infinity)	0.0000	infinity	infinity
Parameter 2~N(0.000,infinity)	0.0000	infinity	infinity
Parameter 3~N(0.000,infinity)	0.0000	infinity	infinity
Parameter 4~N(0.000,infinity)	0.0000	infinity	infinity
Parameter 5~N(0.000,infinity)	0.0000	infinity	infinity
Parameter 6~N(0.000,5.000)	0.0000	5.0000	2.2361
Parameter 7~N(0.000,5.000)	0.0000	5.0000	2.2361
Parameter 8~N(0.000,5.000)	0.0000	5.0000	2.2361
Parameter 9~N(0.000,5.000)	0.0000	5.0000	2.2361
Parameter 10~N(0.000,5.000)	0.0000	5.0000	2.2361
Parameter 11~IW(1.000,7)	infinity	infinity	infinity
Parameter 12~IW(0.000,7)	infinity	infinity	infinity
Parameter 13~IW(1.000,7)	infinity	infinity	infinity
Parameter 14~IW(0.000,7)	infinity	infinity	infinity
Parameter 15~IW(0.000,7)	infinity	infinity	infinity
Parameter 16~IW(1.000,7)	infinity	infinity	infinity
Parameter 17~IW(0.000,7)	infinity	infinity	infinity
Parameter 18~IW(0.000,7)	infinity	infinity	infinity
Parameter 19~IW(0.000,7)	infinity	infinity	infinity
Parameter 20~IW(1.000,7)	infinity	infinity	infinity
Parameter 21~IW(0.000,7)	infinity	infinity	infinity
Parameter 22~IW(0.000,7)	infinity	infinity	infinity
Parameter 23~IW(0.000,7)	infinity	infinity	infinity
Parameter 24~IW(0.000,7)	infinity	infinity	infinity
Parameter 25~IW(1.000,7)	infinity	infinity	infinity
Parameter 26~IW(0.000,7)	infinity	infinity	infinity
Parameter 27~IW(0.000,7)	infinity	infinity	infinity
Parameter 28~IW(0.000,7)	infinity	infinity	infinity
Parameter 29~IW(0.000,7)	infinity	infinity	infinity
Parameter 30~IW(0.000,7)	infinity	infinity	infinity
Parameter 31~IW(1.000,7)	infinity	infinity	infinity
Parameter 32~N(0.000,infinity)	0.0000	infinity	infinity
Parameter 33~N(0.000,infinity)	0.0000	infinity	infinity
Parameter 34~N(0.000,infinity)	0.0000	infinity	infinity
Parameter 35~N(0.000,infinity)	0.0000	infinity	infinity
Parameter 36~N(0.000,infinity)	0.0000	infinity	infinity
Parameter 37~N(0.000,infinity)	0.0000	infinity	infinity
Parameter 38~N(0.000,5.000)	0.0000	5.0000	2.2361
Parameter 39~N(0.000,5.000)	0.0000	5.0000	2.2361
Parameter 40~N(0.000,5.000)	0.0000	5.0000	2.2361

Parameter 41~IG(-1.000,0.000)	infinity	infinity	infinity
Parameter 42~IG(-1.000,0.000)	infinity	infinity	infinity
Parameter 43~IW(1.000,4)	infinity	infinity	infinity
Parameter 44~IW(0.000,4)	infinity	infinity	infinity
Parameter 45~IW(1.000,4)	infinity	infinity	infinity
Parameter 46~IW(0.000,4)	infinity	infinity	infinity
Parameter 47~IW(0.000,4)	infinity	infinity	infinity
Parameter 48~IW(1.000,4)	infinity	infinity	infinity
Parameter 49~N(0.000,5.000)	0.0000	5.0000	2.2361

TECHNICAL 3 OUTPUT

ESTIMATED COVARIANCE MATRIX FOR PARAMETER ESTIMATES

	1	2	3	4	5
1	0.434429D-04				
2	0.106561D-04	0.558506D-04			
3	0.322561D-05	0.957834D-05	0.475830D-04		
4	0.511760D-05	0.199604D-04	0.216983D-04	0.210026D-03	
5	-0.115213D-04	-0.244846D-04	-0.171767D-04	-0.435383D-04	0.168971D-03
6	0.114384D-04	0.425869D-05	-0.171162D-04	-0.396122D-04	0.282740D-04
7	-0.133981D-04	0.610001D-05	-0.393813D-05	0.105584D-04	-0.221056D-04
8	0.606868D-05	-0.138659D-04	-0.207043D-05	-0.269538D-04	0.205561D-04
9	-0.204784D-05	-0.366864D-05	0.486362D-05	0.126012D-04	0.258732D-05
10	0.981217D-07	-0.575342D-05	0.102316D-05	-0.103084D-04	0.430624D-05
11	0.363127D-07	-0.543075D-06	0.122679D-05	-0.139087D-05	0.345273D-06
12	-0.829661D-06	-0.335523D-07	-0.644487D-06	-0.937016D-06	0.645191D-07
13	0.857947D-06	0.111936D-05	-0.220497D-05	-0.284349D-05	0.965544D-06
14	0.575207D-06	-0.128881D-05	-0.361371D-06	-0.162692D-05	-0.189812D-05
15	0.959326D-06	0.137064D-05	0.349147D-05	0.587775D-05	-0.389959D-05
16	-0.170135D-05	0.135272D-05	-0.339106D-05	-0.402687D-05	0.866083D-05
17	-0.165321D-05	-0.357309D-06	-0.227988D-06	-0.225894D-06	0.817956D-06
18	-0.726742D-06	0.980497D-06	-0.919468D-06	0.779635D-06	0.746674D-06
19	-0.629099D-06	0.171877D-06	0.718109D-06	0.349363D-05	-0.815010D-06
20	-0.486297D-06	-0.149074D-06	0.812824D-06	-0.184134D-05	0.658910D-06
21	0.618465D-06	0.767540D-06	-0.632784D-06	-0.542932D-06	0.844133D-07
22	-0.100515D-05	0.283033D-06	-0.109200D-05	-0.383076D-05	-0.242897D-05
23	0.199487D-05	-0.174669D-05	-0.313399D-06	0.683436D-05	-0.163441D-05
24	0.120126D-05	0.488534D-06	0.139467D-05	0.329752D-05	-0.121695D-05
25	-0.299815D-05	0.240250D-05	-0.925155D-07	0.957231D-05	-0.280449D-05
26	0.146097D-05	-0.233143D-05	0.771676D-06	0.219424D-05	0.155633D-05
27	-0.401515D-06	-0.667190D-06	0.909694D-06	0.450256D-05	0.190645D-06

28	-0.353818D-06	0.267312D-05	-0.110713D-05	-0.506245D-05	-0.968306D-06
29	0.681250D-06	-0.192278D-06	0.546035D-06	-0.693260D-06	-0.192769D-06
30	0.162253D-05	-0.309402D-05	-0.897080D-06	-0.247052D-05	0.991175D-06
31	-0.588106D-05	0.234678D-06	0.715779D-06	-0.270099D-05	-0.142525D-05
32	0.464524D-05	0.136873D-05	0.661518D-05	0.225028D-04	0.194437D-04
33	-0.771442D-05	-0.361691D-05	-0.168221D-04	-0.594447D-05	-0.121397D-04
34	0.562445D-05	0.520910D-05	0.168861D-04	-0.311030D-07	0.139563D-04
35	-0.554414D-05	-0.204858D-04	-0.123613D-04	-0.732210D-06	0.255754D-04
36	-0.177351D-04	0.284787D-05	-0.955285D-05	-0.151219D-04	-0.126466D-04
37	-0.123603D-05	0.676946D-05	-0.860612D-05	-0.209328D-04	-0.128776D-04
38	-0.142927D-04	-0.372494D-04	0.143441D-04	-0.256435D-04	0.184075D-04
39	-0.148483D-05	-0.595880D-05	-0.550538D-05	-0.483740D-05	0.254931D-04
40	0.188965D-04	0.666163D-05	-0.136118D-04	-0.391518D-04	0.171205D-04
41	-0.117945D-05	-0.206131D-05	-0.474785D-06	0.664483D-06	0.231472D-05
42	0.120914D-05	0.208284D-05	-0.645324D-06	-0.297441D-06	0.494187D-05
43	-0.349285D-05	-0.155965D-04	0.400530D-05	0.310080D-04	0.231821D-04
44	0.426801D-05	0.109198D-04	0.560534D-06	-0.223910D-04	-0.234172D-04
45	-0.579726D-05	-0.944305D-05	-0.780101D-05	-0.810064D-05	0.396856D-04
46	-0.209210D-06	-0.484970D-05	-0.577011D-05	0.129493D-04	0.470220D-05
47	-0.680588D-06	0.654249D-05	0.644963D-05	-0.108316D-04	-0.130898D-05
48	0.374948D-05	-0.502258D-06	-0.268506D-06	-0.658595D-06	-0.729711D-05
49	0.641526D-04	0.165991D-05	-0.487739D-04	-0.163443D-03	0.874018D-04

ESTIMATED COVARIANCE MATRIX FOR PARAMETER ESTIMATES

	6	7	8	9	10
6	0.475899D-02				
7	-0.268059D-03	0.192603D-02			
8	-0.860021D-05	-0.398906D-03	0.228693D-02		
9	-0.103387D-03	-0.113919D-03	-0.209002D-03	0.690155D-03	
10	-0.730854D-05	0.113746D-03	0.113011D-03	0.918343D-04	0.737939D-03
11	-0.234381D-05	-0.509760D-05	0.236245D-05	0.855807D-06	-0.652484D-06
12	-0.583427D-05	0.442469D-05	-0.584020D-05	-0.195228D-05	0.807558D-07
13	-0.146319D-05	0.122220D-05	0.123903D-04	0.413471D-06	-0.303322D-05
14	0.346353D-06	-0.165666D-04	0.767917D-05	-0.386905D-06	-0.727595D-05
15	-0.207013D-05	-0.123836D-04	0.536652D-05	-0.844039D-06	0.676563D-05
16	-0.227417D-04	-0.492433D-05	0.186341D-04	-0.192394D-05	0.590584D-05
17	-0.345093D-05	0.199721D-05	0.509740D-05	-0.217355D-05	0.417673D-05
18	0.990074D-07	0.204079D-05	-0.322864D-05	-0.293596D-05	-0.606992D-05
19	0.100027D-05	-0.742007D-05	-0.102001D-04	0.194197D-06	-0.795987D-06
20	-0.457088D-07	-0.335124D-05	0.281871D-05	-0.516676D-05	0.132318D-05
21	0.139978D-04	0.242500D-05	-0.576259D-05	0.526154D-05	0.668602D-05
22	-0.778290D-05	0.552419D-06	0.250312D-05	-0.418415D-05	-0.650934D-05

23	0.694643D-06	-0.140703D-04	-0.438366D-05	-0.292060D-05	-0.143177D-04
24	-0.209941D-04	0.137627D-04	-0.830175D-05	0.595974D-05	0.408666D-05
25	-0.480343D-04	0.103704D-04	0.388823D-04	0.214210D-04	0.797682D-05
26	-0.249499D-04	-0.489476D-05	0.725687D-05	-0.288494D-05	-0.359583D-05
27	0.656884D-05	-0.810705D-05	0.334170D-05	0.110296D-05	0.856337D-06
28	-0.277897D-05	0.271507D-04	-0.665388D-05	0.208581D-05	0.482798D-05
29	0.534693D-05	-0.126220D-04	0.185699D-05	0.793479D-05	0.447681D-05
30	0.249319D-04	-0.130675D-04	0.479142D-05	-0.119878D-05	0.393902D-05
31	-0.167321D-04	0.549103D-05	-0.233157D-04	-0.123280D-04	-0.146275D-04
32	0.281184D-04	0.285687D-04	0.933721D-04	0.301593D-04	-0.109298D-03
33	0.418024D-05	0.677450D-04	-0.715895D-04	-0.590715D-04	-0.273030D-04
34	0.269712D-04	-0.747668D-04	0.453678D-04	-0.176861D-05	0.577046D-05
35	-0.119457D-04	-0.298790D-05	-0.725480D-04	0.218514D-04	0.286296D-06
36	-0.305874D-04	0.617891D-05	-0.941853D-04	-0.157487D-04	0.272773D-04
37	-0.419983D-04	0.118918D-03	0.294984D-04	0.329191D-04	0.627174D-04
38	-0.111205D-03	-0.224473D-04	-0.194296D-03	-0.174462D-03	-0.197170D-04
39	-0.247040D-05	-0.103460D-03	0.123046D-05	-0.850890D-04	0.757018D-04
40	-0.272134D-03	-0.295820D-04	0.742130D-04	0.198967D-03	-0.345055D-04
41	0.754672D-05	0.595485D-05	-0.307179D-05	-0.363924D-05	0.360707D-05
42	0.341843D-04	-0.119618D-04	-0.209778D-04	0.300587D-04	0.646866D-05
43	-0.136360D-03	0.421049D-04	-0.255618D-04	0.331689D-04	0.328100D-04
44	0.328643D-04	-0.714747D-04	0.768247D-04	-0.294822D-04	-0.999927D-05
45	0.316702D-04	0.562587D-04	-0.113856D-03	0.401918D-04	-0.102023D-04
46	0.503278D-05	0.159555D-04	0.325486D-04	-0.436586D-05	-0.642864D-06
47	-0.113564D-04	-0.458238D-04	-0.283344D-04	-0.890064D-05	0.580611D-05
48	0.245187D-05	0.185881D-04	0.157324D-04	-0.314276D-05	-0.100430D-04
49	0.113378D-03	-0.509405D-04	0.152121D-03	0.499894D-03	-0.105418D-03

ESTIMATED COVARIANCE MATRIX FOR PARAMETER ESTIMATES

	11	12	13	14	15
11	0.211882D-04				
12	0.587197D-05	0.143751D-04			
13	0.125085D-05	0.625247D-05	0.320625D-04		
14	-0.279493D-05	-0.466583D-05	-0.172369D-05	0.482169D-04	
15	0.458484D-06	-0.243658D-05	-0.100474D-04	0.138808D-04	0.596507D-04
16	-0.207164D-05	-0.712106D-06	0.532754D-05	-0.138009D-04	-0.446239D-04
17	0.171775D-05	0.302984D-05	0.116936D-05	-0.303776D-05	-0.127747D-05
18	0.836280D-06	0.983850D-06	0.576541D-05	-0.222545D-05	-0.463918D-05
19	-0.124231D-05	-0.342252D-06	-0.181990D-05	0.456158D-05	0.146606D-04
20	0.242330D-05	0.116398D-05	0.122854D-05	-0.179041D-05	-0.254188D-05
21	0.308816D-05	0.528591D-05	0.137114D-05	-0.453820D-05	0.139678D-06
22	0.215504D-06	0.260354D-05	0.111885D-04	-0.133320D-06	-0.576288D-05

23	-0.538261D-06	-0.296208D-05	-0.955811D-06	0.871270D-05	0.238670D-04
24	0.664573D-06	0.526826D-06	0.193699D-05	-0.688082D-06	-0.181382D-05
25	-0.915658D-06	-0.195857D-05	0.975709D-06	-0.290237D-06	0.301520D-05
26	-0.305763D-05	-0.580182D-05	-0.328383D-05	0.883734D-05	0.265576D-05
27	-0.565906D-06	-0.314109D-05	-0.101837D-04	0.472040D-06	0.115906D-04
28	0.151654D-05	0.105600D-06	0.215208D-05	-0.976191D-05	-0.241109D-04
29	-0.145748D-05	-0.742369D-06	-0.100375D-05	0.162882D-05	0.348783D-05
30	-0.258978D-06	-0.218816D-05	-0.181584D-05	0.677615D-06	0.217721D-05
31	-0.635343D-07	-0.131512D-06	0.255242D-05	0.115824D-05	-0.826341D-05
32	-0.974829D-05	0.228843D-06	-0.128230D-05	0.608545D-05	-0.825256D-05
33	0.850041D-06	-0.251313D-05	-0.845830D-05	-0.518074D-05	-0.222697D-04
34	-0.175100D-04	0.359097D-05	-0.611811D-06	-0.575934D-05	0.131361D-04
35	-0.220777D-05	-0.105894D-05	0.923260D-05	0.223102D-05	-0.607284D-05
36	0.906816D-05	-0.676398D-05	-0.707441D-05	0.675083D-05	0.291747D-04
37	0.203747D-04	0.100158D-06	0.768055D-05	-0.132115D-04	0.699413D-05
38	0.593960D-05	-0.957164D-05	0.317228D-04	-0.781204D-05	-0.198420D-04
39	0.401746D-05	-0.119366D-04	0.117306D-04	0.555477D-05	0.240832D-05
40	-0.765756D-05	0.203023D-06	0.343357D-04	-0.167092D-04	-0.227220D-04
41	0.918661D-06	0.966418D-07	0.357444D-06	-0.394365D-06	0.952654D-06
42	-0.128786D-05	-0.184195D-05	0.308823D-06	0.120124D-05	0.319162D-05
43	0.919823D-05	-0.908448D-06	-0.519156D-05	-0.155896D-05	-0.486460D-06
44	-0.461772D-05	0.150716D-05	0.274218D-05	0.996973D-05	0.677305D-05
45	0.454335D-05	0.631326D-06	-0.441163D-05	-0.149896D-04	-0.214645D-04
46	0.120509D-05	-0.112496D-05	0.246779D-05	0.319246D-05	0.516126D-05
47	0.278341D-05	0.134003D-05	-0.122017D-06	0.134430D-05	-0.240065D-05
48	0.125573D-06	-0.118575D-05	0.138053D-05	-0.703999D-06	0.229163D-05
49	-0.262503D-04	-0.105138D-04	0.130111D-03	-0.526599D-04	-0.857082D-04

ESTIMATED COVARIANCE MATRIX FOR PARAMETER ESTIMATES

	16	17	18	19	20
16	0.462428D-03				
17	0.693813D-06	0.114577D-04			
18	0.590295D-05	0.273264D-05	0.136780D-04		
19	-0.257068D-04	-0.173801D-05	-0.564120D-05	0.500288D-04	
20	-0.112715D-05	0.155809D-05	0.505351D-05	-0.557446D-05	0.233577D-04
21	-0.224300D-05	0.582126D-05	0.136941D-05	-0.743053D-06	0.946819D-06
22	0.872142D-05	0.170888D-05	0.540474D-05	-0.381646D-05	0.203427D-05
23	-0.444785D-04	-0.177227D-05	-0.301819D-05	0.263897D-04	-0.441748D-05
24	0.842178D-05	0.234110D-05	0.546676D-05	-0.515357D-05	0.107401D-04
25	0.191261D-04	-0.121033D-05	0.275337D-05	-0.239305D-05	0.208894D-05
26	0.313387D-06	-0.401063D-05	-0.122871D-05	0.961836D-06	-0.147648D-05
27	-0.867413D-05	-0.754057D-06	-0.398837D-05	0.382274D-05	-0.148438D-05

28	0.702213D-04	-0.512379D-06	0.181403D-05	-0.206245D-04	0.124166D-05
29	-0.556384D-05	-0.175868D-05	-0.580471D-05	0.786167D-05	-0.707335D-05
30	-0.140158D-04	-0.156544D-05	-0.820602D-06	0.410818D-07	-0.836975D-06
31	0.466150D-05	0.137701D-05	0.183623D-05	-0.767320D-05	0.219097D-05
32	0.383562D-04	-0.642165D-05	0.745001D-07	-0.109241D-04	0.142929D-04
33	-0.611206D-05	0.392192D-05	-0.585051D-05	-0.650479D-05	0.249301D-04
34	0.178890D-04	-0.137731D-05	-0.827746D-05	0.158834D-04	-0.193491D-04
35	-0.811967D-05	0.152557D-05	0.398672D-05	-0.541664D-06	0.707080D-06
36	-0.569838D-04	-0.368345D-05	-0.943757D-06	0.389147D-05	-0.809955D-05
37	-0.557451D-04	0.142231D-05	0.170401D-05	0.811538D-05	-0.139366D-04
38	-0.449327D-04	-0.949706D-05	-0.366661D-05	0.931849D-05	0.571215D-05
39	0.246687D-05	0.103937D-04	-0.127240D-04	0.424139D-05	-0.113341D-04
40	0.440699D-05	0.164442D-04	0.194179D-04	0.676753D-05	-0.146658D-04
41	0.275772D-05	-0.512222D-06	0.119099D-05	0.926416D-06	0.103369D-05
42	0.816317D-06	0.361855D-06	0.100737D-05	-0.302074D-05	-0.113114D-06
43	0.489339D-04	-0.311309D-06	-0.927395D-07	0.338574D-05	-0.447271D-05
44	-0.182576D-04	0.267379D-05	-0.324080D-05	0.625909D-06	0.447513D-05
45	0.191614D-05	-0.330223D-05	0.496076D-06	-0.415322D-05	-0.831969D-05
46	-0.603319D-05	-0.614050D-06	0.220713D-05	-0.519942D-05	0.154093D-05
47	-0.681762D-05	0.318039D-05	0.120302D-05	-0.754355D-06	-0.417991D-05
48	-0.175348D-04	0.736218D-06	-0.250360D-06	-0.101679D-05	0.280972D-05
49	-0.605522D-05	0.553668D-04	0.619922D-04	0.162642D-04	-0.501447D-04

ESTIMATED COVARIANCE MATRIX FOR PARAMETER ESTIMATES

	21	22	23	24	25
21	0.464732D-04				
22	0.100815D-04	0.564919D-04			
23	-0.864080D-05	-0.176853D-04	0.216997D-03		
24	0.265574D-05	0.116840D-04	-0.161068D-04	0.502662D-04	
25	0.990806D-05	0.368172D-04	-0.331884D-04	0.461987D-04	0.408254D-03
26	-0.937051D-05	-0.221042D-05	0.228645D-05	0.270823D-06	0.799438D-06
27	-0.338544D-05	-0.109145D-04	0.571006D-05	-0.137687D-05	-0.199546D-05
28	-0.101158D-05	0.223036D-05	-0.445947D-04	0.339804D-05	0.488534D-05
29	-0.243254D-05	-0.403618D-05	0.554998D-05	-0.913199D-05	-0.827655D-05
30	-0.815161D-05	-0.217219D-04	0.380663D-04	-0.195512D-04	-0.788173D-04
31	-0.277209D-06	0.659009D-05	-0.140987D-04	0.564162D-05	0.653477D-05
32	0.609982D-06	-0.381116D-06	-0.609029D-05	0.933713D-05	0.135673D-05
33	-0.860713D-05	0.739176D-05	-0.403184D-04	0.100929D-04	0.282317D-04
34	0.941189D-05	-0.114571D-04	0.502569D-04	-0.176959D-04	-0.629301D-04
35	0.444518D-05	0.526470D-05	-0.472861D-05	-0.872748D-05	-0.971541D-06
36	-0.141135D-04	0.381118D-05	0.595092D-05	-0.228682D-05	0.330865D-04
37	-0.450507D-05	-0.125790D-04	-0.553028D-06	-0.656781D-05	-0.297105D-05

38	-0.325655D-04	-0.129408D-04	0.176309D-04	-0.100715D-04	-0.452633D-04
39	-0.322863D-04	-0.203321D-04	0.352739D-04	-0.313762D-04	-0.773508D-04
40	0.439460D-04	0.318978D-04	-0.222897D-04	-0.433336D-04	0.223942D-03
41	0.164945D-06	0.139570D-05	-0.182192D-06	0.234606D-05	0.193697D-06
42	-0.437770D-05	0.366246D-05	0.844424D-07	0.295816D-05	0.811010D-05
43	-0.802187D-06	-0.113704D-04	0.815153D-05	-0.888989D-05	-0.161751D-04
44	-0.158192D-06	0.105402D-04	-0.578992D-05	0.150658D-04	0.187034D-04
45	0.103018D-05	-0.545004D-05	0.571267D-05	-0.138121D-04	-0.193257D-04
46	-0.192613D-05	-0.539551D-05	-0.401907D-06	-0.739780D-05	-0.721468D-05
47	0.284250D-05	0.769587D-05	-0.190960D-05	0.277617D-05	0.676170D-05
48	0.319093D-06	-0.400781D-05	0.125644D-04	-0.435217D-05	-0.119372D-04
49	0.119867D-03	0.106120D-03	-0.892987D-04	-0.156646D-03	0.690139D-03

ESTIMATED COVARIANCE MATRIX FOR PARAMETER ESTIMATES

	26	27	28	29	30
26	0.387649D-04				
27	0.896408D-05	0.499527D-04			
28	-0.588592D-05	-0.142552D-04	0.187462D-03		
29	0.326897D-05	0.114589D-04	-0.129594D-04	0.388984D-04	
30	0.651130D-05	0.171198D-04	-0.215396D-04	0.199562D-04	0.184971D-03
31	-0.384435D-05	-0.369986D-04	0.647938D-04	-0.282861D-04	-0.671294D-04
32	0.701589D-05	-0.117413D-04	0.397101D-05	-0.130987D-04	-0.280460D-05
33	-0.271831D-05	0.159279D-06	0.143623D-04	-0.199392D-04	-0.605118D-04
34	-0.259861D-04	0.523315D-05	-0.281098D-04	0.813482D-05	0.387472D-04
35	-0.581399D-06	0.128060D-05	-0.860247D-05	0.321323D-05	-0.987055D-05
36	-0.971868D-05	0.344698D-04	-0.313714D-04	0.234488D-04	0.524664D-05
37	-0.113453D-04	0.176215D-04	0.376118D-05	0.662083D-05	-0.466121D-05
38	0.282919D-04	-0.515789D-05	-0.215025D-05	-0.459181D-05	0.417571D-04
39	0.139636D-04	-0.866738D-05	-0.114296D-04	-0.123277D-04	0.187137D-04
40	-0.365545D-04	-0.137376D-04	0.392495D-04	-0.367805D-04	-0.112973D-03
41	0.192750D-06	-0.999156D-06	0.203292D-05	-0.171352D-05	-0.282936D-05
42	0.103518D-05	0.324248D-05	-0.812241D-05	0.518075D-05	-0.212478D-05
43	0.840798D-05	-0.608245D-06	0.118560D-05	0.245395D-05	0.379507D-04
44	-0.734652D-05	0.289074D-05	-0.444907D-05	0.437746D-05	-0.371664D-04
45	-0.210333D-05	0.420362D-06	0.757070D-05	-0.599702D-05	0.202057D-04
46	-0.177109D-05	0.213373D-05	-0.732703D-05	-0.299883D-05	0.786677D-05
47	0.913898D-06	0.208898D-06	-0.290794D-05	0.652427D-05	0.548904D-06
48	-0.218526D-05	0.178371D-05	-0.817572D-05	-0.321959D-06	0.657648D-05
49	-0.103758D-03	-0.487680D-04	0.110870D-03	-0.124059D-03	-0.336402D-03

ESTIMATED COVARIANCE MATRIX FOR PARAMETER ESTIMATES

	31	32	33	34	35
31	0.306022D-03				
32	-0.110430D-04	0.523148D-02			
33	0.348843D-04	0.179153D-03	0.412814D-02		
34	0.132972D-04	-0.737343D-04	-0.246933D-02	0.470564D-02	
35	-0.845893D-05	0.366113D-04	0.451337D-03	-0.321950D-03	0.146771D-02
36	-0.180338D-04	-0.527355D-02	-0.184667D-03	0.109604D-03	0.559763D-05
37	0.265787D-04	-0.520536D-02	-0.102523D-03	0.855156D-04	-0.517070D-04
38	-0.831610D-05	-0.973801D-03	-0.204304D-03	0.364164D-03	0.433094D-04
39	-0.109436D-04	-0.714595D-03	-0.349329D-04	0.230345D-03	0.128256D-03
40	-0.921836D-04	0.767124D-04	0.573107D-03	-0.382963D-03	0.285370D-03
41	0.142686D-05	0.424946D-04	-0.378435D-05	0.288644D-05	-0.693033D-05
42	-0.119625D-04	0.313167D-05	-0.364062D-05	-0.458174D-05	0.103312D-04
43	-0.224688D-05	0.113278D-04	0.137489D-03	-0.191997D-03	-0.306221D-04
44	-0.262853D-05	0.191187D-04	-0.397628D-04	0.138777D-03	0.412551D-04
45	0.704146D-05	-0.693163D-04	-0.361448D-05	-0.101903D-03	-0.611854D-04
46	-0.495897D-05	0.703526D-05	0.309906D-04	-0.386600D-04	-0.529097D-04
47	-0.734094D-05	0.732297D-06	-0.245052D-04	0.375220D-04	0.124612D-04
48	0.417336D-05	-0.217244D-04	0.682032D-05	-0.113308D-04	0.324332D-04
49	-0.335001D-03	-0.483441D-03	0.184650D-02	-0.108630D-02	0.100441D-02

ESTIMATED COVARIANCE MATRIX FOR PARAMETER ESTIMATES

	36	37	38	39	40
36	0.804076D-02				
37	0.533784D-02	0.702428D-02			
38	0.120937D-02	0.900687D-03	0.100287D-01		
39	0.794969D-03	0.846677D-03	0.604819D-02	0.870280D-02	
40	-0.335433D-03	-0.269067D-03	-0.158016D-02	0.211032D-02	0.292426D-01
41	0.153042D-04	-0.252715D-04	0.272425D-05	-0.635082D-05	-0.598936D-04
42	0.573549D-05	-0.407793D-05	-0.230568D-03	-0.498859D-04	-0.242354D-03
43	0.101662D-03	0.742165D-04	-0.106603D-03	0.714868D-05	-0.135488D-03
44	-0.650231D-04	-0.564214D-04	0.718575D-04	-0.940270D-05	-0.583646D-04
45	0.793108D-04	0.116319D-03	0.395617D-04	-0.303358D-05	0.181707D-03
46	0.726224D-05	-0.402730D-04	0.148418D-04	0.312256D-04	0.796588D-04
47	0.596477D-05	0.639173D-05	-0.620193D-05	-0.325468D-05	-0.206271D-03
48	0.366057D-04	0.157702D-04	0.277120D-04	0.945300D-05	-0.637868D-05
49	-0.218862D-03	-0.167960D-03	0.936223D-03	0.130448D-01	0.955810D-01

ESTIMATED COVARIANCE MATRIX FOR PARAMETER ESTIMATES

41	42	43	44	45

41	0.302622D-04				
42	-0.931719D-06	0.164902D-03			
43	0.655666D-05	0.667011D-05	0.194791D-02		
44	-0.533920D-06	-0.949428D-05	-0.116664D-02	0.143657D-02	
45	-0.116490D-04	-0.135213D-05	0.716397D-03	-0.135986D-02	0.265123D-02
46	0.305764D-05	-0.195535D-05	0.194067D-03	-0.134491D-03	0.572951D-04
47	-0.623816D-06	0.103629D-04	-0.114013D-03	0.148443D-03	-0.111760D-03
48	0.541804D-06	-0.521278D-05	0.159191D-04	-0.285934D-04	0.255439D-04
49	-0.178479D-03	-0.816220D-03	-0.510206D-03	-0.138182D-03	0.593408D-03

ESTIMATED COVARIANCE MATRIX FOR PARAMETER ESTIMATES

	46	47	48	49
46	0.338048D-03			
47	-0.202317D-03	0.377712D-03		
48	0.601377D-04	-0.384733D-04	0.180340D-03	
49	0.274631D-03	-0.671697D-03	-0.135139D-04	0.318784D+00

ESTIMATED CORRELATION MATRIX FOR PARAMETER ESTIMATES

	1	2	3	4	5
1	1.000				
2	0.216	1.000			
3	0.071	0.186	1.000		
4	0.054	0.184	0.217	1.000	
5	-0.134	-0.252	-0.192	-0.231	1.000
6	0.025	0.008	-0.036	-0.040	0.032
7	-0.046	0.019	-0.013	0.017	-0.039
8	0.019	-0.039	-0.006	-0.039	0.033
9	-0.012	-0.019	0.027	0.033	0.008
10	0.001	-0.028	0.005	-0.026	0.012
11	0.001	-0.016	0.039	-0.021	0.006
12	-0.033	-0.001	-0.025	-0.017	0.001
13	0.023	0.026	-0.056	-0.035	0.013
14	0.013	-0.025	-0.008	-0.016	-0.021
15	0.019	0.024	0.066	0.053	-0.039
16	-0.012	0.008	-0.023	-0.013	0.031
17	-0.074	-0.014	-0.010	-0.005	0.019
18	-0.030	0.035	-0.036	0.015	0.016
19	-0.013	0.003	0.015	0.034	-0.009
20	-0.015	-0.004	0.024	-0.026	0.010

21	0.014	0.015	-0.013	-0.005	0.001
22	-0.020	0.005	-0.021	-0.035	-0.025
23	0.021	-0.016	-0.003	0.032	-0.009
24	0.026	0.009	0.029	0.032	-0.013
25	-0.023	0.016	-0.001	0.033	-0.011
26	0.036	-0.050	0.018	0.024	0.019
27	-0.009	-0.013	0.019	0.044	0.002
28	-0.004	0.026	-0.012	-0.026	-0.005
29	0.017	-0.004	0.013	-0.008	-0.002
30	0.018	-0.030	-0.010	-0.013	0.006
31	-0.051	0.002	0.006	-0.011	-0.006
32	0.010	0.003	0.013	0.021	0.021
33	-0.018	-0.008	-0.038	-0.006	-0.015
34	0.012	0.010	0.036	0.000	0.016
35	-0.022	-0.072	-0.047	-0.001	0.051
36	-0.030	0.004	-0.015	-0.012	-0.011
37	-0.002	0.011	-0.015	-0.017	-0.012
38	-0.022	-0.050	0.021	-0.018	0.014
39	-0.002	-0.009	-0.009	-0.004	0.021
40	0.017	0.005	-0.012	-0.016	0.008
41	-0.033	-0.050	-0.013	0.008	0.032
42	0.014	0.022	-0.007	-0.002	0.030
43	-0.012	-0.047	0.013	0.048	0.040
44	0.017	0.039	0.002	-0.041	-0.048
45	-0.017	-0.025	-0.022	-0.011	0.059
46	-0.002	-0.035	-0.045	0.049	0.020
47	-0.005	0.045	0.048	-0.038	-0.005
48	0.042	-0.005	-0.003	-0.003	-0.042
49	0.017	0.000	-0.013	-0.020	0.012

ESTIMATED CORRELATION MATRIX FOR PARAMETER ESTIMATES

	6	7	8	9	10
6	1.000				
7	-0.089	1.000			
8	-0.003	-0.190	1.000		
9	-0.057	-0.099	-0.166	1.000	
10	-0.004	0.095	0.087	0.129	1.000
11	-0.007	-0.025	0.011	0.007	-0.005
12	-0.022	0.027	-0.032	-0.020	0.001
13	-0.004	0.005	0.046	0.003	-0.020
14	0.001	-0.054	0.023	-0.002	-0.039
15	-0.004	-0.037	0.015	-0.004	0.032

16	-0.015	-0.005	0.018	-0.003	0.010
17	-0.015	0.013	0.031	-0.024	0.045
18	0.000	0.013	-0.018	-0.030	-0.060
19	0.002	-0.024	-0.030	0.001	-0.004
20	0.000	-0.016	0.012	-0.041	0.010
21	0.030	0.008	-0.018	0.029	0.036
22	-0.015	0.002	0.007	-0.021	-0.032
23	0.001	-0.022	-0.006	-0.008	-0.036
24	-0.043	0.044	-0.024	0.032	0.021
25	-0.034	0.012	0.040	0.040	0.015
26	-0.058	-0.018	0.024	-0.018	-0.021
27	0.013	-0.026	0.010	0.006	0.004
28	-0.003	0.045	-0.010	0.006	0.013
29	0.012	-0.046	0.006	0.048	0.026
30	0.027	-0.022	0.007	-0.003	0.011
31	-0.014	0.007	-0.028	-0.027	-0.031
32	0.006	0.009	0.027	0.016	-0.056
33	0.001	0.024	-0.023	-0.035	-0.016
34	0.006	-0.025	0.014	-0.001	0.003
35	-0.005	-0.002	-0.040	0.022	0.000
36	-0.005	0.002	-0.022	-0.007	0.011
37	-0.007	0.032	0.007	0.015	0.028
38	-0.016	-0.005	-0.041	-0.066	-0.007
39	0.000	-0.025	0.000	-0.035	0.030
40	-0.023	-0.004	0.009	0.044	-0.007
41	0.020	0.025	-0.012	-0.025	0.024
42	0.039	-0.021	-0.034	0.089	0.019
43	-0.045	0.022	-0.012	0.029	0.027
44	0.013	-0.043	0.042	-0.030	-0.010
45	0.009	0.025	-0.046	0.030	-0.007
46	0.004	0.020	0.037	-0.009	-0.001
47	-0.008	-0.054	-0.030	-0.017	0.011
48	0.003	0.032	0.024	-0.009	-0.028
49	0.003	-0.002	0.006	0.034	-0.007

ESTIMATED CORRELATION MATRIX FOR PARAMETER ESTIMATES

	11	12	13	14	15
11	1.000				
12	0.336	1.000			
13	0.048	0.291	1.000		
14	-0.087	-0.177	-0.044	1.000	
15	0.013	-0.083	-0.230	0.259	1.000

16	-0.021	-0.009	0.044	-0.092	-0.269
17	0.110	0.236	0.061	-0.129	-0.049
18	0.049	0.070	0.275	-0.087	-0.162
19	-0.038	-0.013	-0.045	0.093	0.268
20	0.109	0.064	0.045	-0.053	-0.068
21	0.098	0.205	0.036	-0.096	0.003
22	0.006	0.091	0.263	-0.003	-0.099
23	-0.008	-0.053	-0.011	0.085	0.210
24	0.020	0.020	0.048	-0.014	-0.033
25	-0.010	-0.026	0.009	-0.002	0.019
26	-0.107	-0.246	-0.093	0.204	0.055
27	-0.017	-0.117	-0.254	0.010	0.212
28	0.024	0.002	0.028	-0.103	-0.228
29	-0.051	-0.031	-0.028	0.038	0.072
30	-0.004	-0.042	-0.024	0.007	0.021
31	-0.001	-0.002	0.026	0.010	-0.061
32	-0.029	0.001	-0.003	0.012	-0.015
33	0.003	-0.010	-0.023	-0.012	-0.045
34	-0.055	0.014	-0.002	-0.012	0.025
35	-0.013	-0.007	0.043	0.008	-0.021
36	0.022	-0.020	-0.014	0.011	0.042
37	0.053	0.000	0.016	-0.023	0.011
38	0.013	-0.025	0.056	-0.011	-0.026
39	0.009	-0.034	0.022	0.009	0.003
40	-0.010	0.000	0.035	-0.014	-0.017
41	0.036	0.005	0.011	-0.010	0.022
42	-0.022	-0.038	0.004	0.013	0.032
43	0.045	-0.005	-0.021	-0.005	-0.001
44	-0.026	0.010	0.013	0.038	0.023
45	0.019	0.003	-0.015	-0.042	-0.054
46	0.014	-0.016	0.024	0.025	0.036
47	0.031	0.018	-0.001	0.010	-0.016
48	0.002	-0.023	0.018	-0.008	0.022
49	-0.010	-0.005	0.041	-0.013	-0.020

ESTIMATED CORRELATION MATRIX FOR PARAMETER ESTIMATES

	16	17	18	19	20
16	1.000				
17	0.010	1.000			
18	0.074	0.218	1.000		
19	-0.169	-0.073	-0.216	1.000	
20	-0.011	0.095	0.283	-0.163	1.000

21	-0.015	0.252	0.054	-0.015	0.029
22	0.054	0.067	0.194	-0.072	0.056
23	-0.140	-0.036	-0.055	0.253	-0.062
24	0.055	0.098	0.208	-0.103	0.313
25	0.044	-0.018	0.037	-0.017	0.021
26	0.002	-0.190	-0.053	0.022	-0.049
27	-0.057	-0.032	-0.153	0.076	-0.043
28	0.239	-0.011	0.036	-0.213	0.019
29	-0.041	-0.083	-0.252	0.178	-0.235
30	-0.048	-0.034	-0.016	0.000	-0.013
31	0.012	0.023	0.028	-0.062	0.026
32	0.025	-0.026	0.000	-0.021	0.041
33	-0.004	0.018	-0.025	-0.014	0.080
34	0.012	-0.006	-0.033	0.033	-0.058
35	-0.010	0.012	0.028	-0.002	0.004
36	-0.030	-0.012	-0.003	0.006	-0.019
37	-0.031	0.005	0.005	0.014	-0.034
38	-0.021	-0.028	-0.010	0.013	0.012
39	0.001	0.033	-0.037	0.006	-0.025
40	0.001	0.028	0.031	0.006	-0.018
41	0.023	-0.028	0.059	0.024	0.039
42	0.003	0.008	0.021	-0.033	-0.002
43	0.052	-0.002	-0.001	0.011	-0.021
44	-0.022	0.021	-0.023	0.002	0.024
45	0.002	-0.019	0.003	-0.011	-0.033
46	-0.015	-0.010	0.032	-0.040	0.017
47	-0.016	0.048	0.017	-0.005	-0.045
48	-0.061	0.016	-0.005	-0.011	0.043
49	0.000	0.029	0.030	0.004	-0.018

ESTIMATED CORRELATION MATRIX FOR PARAMETER ESTIMATES

	21	22	23	24	25
21	1.000				
22	0.197	1.000			
23	-0.086	-0.160	1.000		
24	0.055	0.219	-0.154	1.000	
25	0.072	0.242	-0.112	0.322	1.000
26	-0.221	-0.047	0.025	0.006	0.006
27	-0.070	-0.205	0.055	-0.027	-0.014
28	-0.011	0.022	-0.221	0.035	0.018
29	-0.057	-0.086	0.060	-0.207	-0.066
30	-0.088	-0.212	0.190	-0.203	-0.287

31	-0.002	0.050	-0.055	0.045	0.018
32	0.001	-0.001	-0.006	0.018	0.001
33	-0.020	0.015	-0.043	0.022	0.022
34	0.020	-0.022	0.050	-0.036	-0.045
35	0.017	0.018	-0.008	-0.032	-0.001
36	-0.023	0.006	0.005	-0.004	0.018
37	-0.008	-0.020	0.000	-0.011	-0.002
38	-0.048	-0.017	0.012	-0.014	-0.022
39	-0.051	-0.029	0.026	-0.047	-0.041
40	0.038	0.025	-0.009	-0.036	0.065
41	0.004	0.034	-0.002	0.060	0.002
42	-0.050	0.038	0.000	0.032	0.031
43	-0.003	-0.034	0.013	-0.028	-0.018
44	-0.001	0.037	-0.010	0.056	0.024
45	0.003	-0.014	0.008	-0.038	-0.019
46	-0.015	-0.039	-0.001	-0.057	-0.019
47	0.021	0.053	-0.007	0.020	0.017
48	0.003	-0.040	0.064	-0.046	-0.044
49	0.031	0.025	-0.011	-0.039	0.060

ESTIMATED CORRELATION MATRIX FOR PARAMETER ESTIMATES

	26	27	28	29	30
26	1.000				
27	0.204	1.000			
28	-0.069	-0.147	1.000		
29	0.084	0.260	-0.152	1.000	
30	0.077	0.178	-0.116	0.235	1.000
31	-0.035	-0.299	0.271	-0.259	-0.282
32	0.016	-0.023	0.004	-0.029	-0.003
33	-0.007	0.000	0.016	-0.050	-0.069
34	-0.061	0.011	-0.030	0.019	0.042
35	-0.002	0.005	-0.016	0.013	-0.019
36	-0.017	0.054	-0.026	0.042	0.004
37	-0.022	0.030	0.003	0.013	-0.004
38	0.045	-0.007	-0.002	-0.007	0.031
39	0.024	-0.013	-0.009	-0.021	0.015
40	-0.034	-0.011	0.017	-0.034	-0.049
41	0.006	-0.026	0.027	-0.050	-0.038
42	0.013	0.036	-0.046	0.065	-0.012
43	0.031	-0.002	0.002	0.009	0.063
44	-0.031	0.011	-0.009	0.019	-0.072
45	-0.007	0.001	0.011	-0.019	0.029

46	-0.015	0.016	-0.029	-0.026	0.031
47	0.008	0.002	-0.011	0.054	0.002
48	-0.026	0.019	-0.044	-0.004	0.036
49	-0.030	-0.012	0.014	-0.035	-0.044

ESTIMATED CORRELATION MATRIX FOR PARAMETER ESTIMATES

	31	32	33	34	35
31	1.000				
32	-0.009	1.000			
33	0.031	0.039	1.000		
34	0.011	-0.015	-0.560	1.000	
35	-0.013	0.013	0.183	-0.123	1.000
36	-0.011	-0.813	-0.032	0.018	0.002
37	0.018	-0.859	-0.019	0.015	-0.016
38	-0.005	-0.134	-0.032	0.053	0.011
39	-0.007	-0.106	-0.006	0.036	0.036
40	-0.031	0.006	0.052	-0.033	0.044
41	0.015	0.107	-0.011	0.008	-0.033
42	-0.053	0.003	-0.004	-0.005	0.021
43	-0.003	0.004	0.048	-0.063	-0.018
44	-0.004	0.007	-0.016	0.053	0.028
45	0.008	-0.019	-0.001	-0.029	-0.031
46	-0.015	0.005	0.026	-0.031	-0.075
47	-0.022	0.001	-0.020	0.028	0.017
48	0.018	-0.022	0.008	-0.012	0.063
49	-0.034	-0.012	0.051	-0.028	0.046

ESTIMATED CORRELATION MATRIX FOR PARAMETER ESTIMATES

	36	37	38	39	40
36	1.000				
37	0.710	1.000			
38	0.135	0.107	1.000		
39	0.095	0.108	0.647	1.000	
40	-0.022	-0.019	-0.092	0.132	1.000
41	0.031	-0.055	0.005	-0.012	-0.064
42	0.005	-0.004	-0.179	-0.042	-0.110
43	0.026	0.020	-0.024	0.002	-0.018
44	-0.019	-0.018	0.019	-0.003	-0.009
45	0.017	0.027	0.008	-0.001	0.021
46	0.004	-0.026	0.008	0.018	0.025

47	0.003	0.004	-0.003	-0.002	-0.062
48	0.030	0.014	0.021	0.008	-0.003
49	-0.004	-0.004	0.017	0.248	0.990

ESTIMATED CORRELATION MATRIX FOR PARAMETER ESTIMATES

	41	42	43	44	45
41	1.000				
42	-0.013	1.000			
43	0.027	0.012	1.000		
44	-0.003	-0.020	-0.697	1.000	
45	-0.041	-0.002	0.315	-0.697	1.000
46	0.030	-0.008	0.239	-0.193	0.061
47	-0.006	0.042	-0.133	0.202	-0.112
48	0.007	-0.030	0.027	-0.056	0.037
49	-0.057	-0.113	-0.020	-0.006	0.020

ESTIMATED CORRELATION MATRIX FOR PARAMETER ESTIMATES

	46	47	48	49
46	1.000			
47	-0.566	1.000		
48	0.244	-0.147	1.000	
49	0.026	-0.061	-0.002	1.000

TECHNICAL 8 OUTPUT

TECHNICAL 8 OUTPUT FOR BAYES ESTIMATION

CHAIN	BSEED
1	0
2	285380
3	253358

ITERATION	POTENTIAL SCALE REDUCTION	PARAMETER WITH HIGHEST PSR
100	1.004	22
200	1.013	37
300	1.004	40
400	1.006	32

500	1.004	36
600	1.011	36
700	1.012	36
800	1.004	36
900	1.003	7
1000	1.002	40

This is the interaction between latent profile categories and bullying on worsening of physical health

SUMMARY OF DATA

Number of clusters 58

Size (s)	Cluster ID with Size s
7	22
16	166
23	80 71 205
25	74
27	192
31	65
32	106
35	228
37	170 72
39	50 75 181
40	182
42	165
44	114
45	173
46	107
47	169 30
48	226 81
49	36
50	116 108 40
56	162 84
57	61
59	206 93 83
60	92
61	69
63	6
64	152
65	145
66	4
68	68
69	154
72	174
78	98
84	178 2
95	156 202
96	144
108	142

131	122
172	24
175	209
224	204
269	125
364	217
738	110
860	100

COVARIANCE COVERAGE OF DATA

Minimum covariance coverage value 0.100

Number of missing data patterns 8

PROPORTION OF DATA PRESENT

Covariance Coverage

	PHYCAT16	CLU2	CLU3	PHY15	KES15
PHYCAT16	1.000				
CLU2	1.000	1.000			
CLU3	1.000	1.000	1.000		
PHY15	0.947	0.947	0.947	0.947	
KES15	0.922	0.922	0.922	0.922	0.922
CHOICE15	0.915	0.915	0.915	0.915	0.915
BULLY15	0.915	0.915	0.915	0.915	0.915
PRESS15	0.915	0.915	0.915	0.915	0.915
DUTIES15	0.915	0.915	0.915	0.915	0.915

Covariance Coverage

	CHOICE15	BULLY15	PRESS15	DUTIES15
CHOICE15	0.915			
BULLY15	0.915	0.915		
PRESS15	0.915	0.914	0.915	
DUTIES15	0.915	0.915	0.915	0.915

UNIVARIATE PROPORTIONS AND COUNTS FOR CATEGORICAL VARIABLES

PHYCAT16

Category 1	0.881	4884.000
Category 2	0.119	662.000

UNIVARIATE SAMPLE STATISTICS

UNIVARIATE HIGHER-ORDER MOMENT DESCRIPTIVE STATISTICS

Variable/ Sample Size	Mean/ Variance	Skewness/ Kurtosis	Minimum/ Maximum	% with Min/Max	Percentiles 20%/60%	40%/80%	Median
CLU2	0.310	0.820	0.000	68.97%	0.000	0.000	0.000
58.000	0.214	-1.328	1.000	31.03%	0.000	1.000	
CLU3	0.448	0.208	0.000	55.17%	0.000	0.000	0.000
58.000	0.247	-1.957	1.000	44.83%	1.000	1.000	
PHY15	0.000	-0.546	-0.632	36.83%	-0.632	0.368	0.368
5254.000	0.233	-1.702	0.368	63.17%	0.368	0.368	
KES15	0.000	1.717	-0.511	21.65%	-0.511	-0.178	-0.178
5114.000	0.289	3.835	3.489	0.04%	-0.011	0.322	
CHOICE15	0.000	-0.402	-2.225	7.41%	-1.225	-0.225	-0.225
5077.000	1.081	-0.336	1.775	8.49%	0.775	0.775	
BULLY15	1.170	3.272	1.000	87.25%	1.000	1.000	1.000
5073.000	0.239	11.772	5.000	0.08%	1.000	1.000	
PRESS15	0.000	0.252	-1.462	19.10%	-0.462	-0.462	-0.462
5074.000	1.001	-0.371	2.538	2.76%	0.538	0.538	
DUTIES15	0.000	-1.246	-3.120	2.40%	-0.120	-0.120	-0.120
5077.000	0.861	1.673	0.880	38.74%	-0.120	0.880	

THE MODEL ESTIMATION TERMINATED NORMALLY

USE THE FBITERATIONS OPTION TO INCREASE THE NUMBER OF ITERATIONS BY A FACTOR
OF AT LEAST TWO TO CHECK CONVERGENCE AND THAT THE PSR VALUE DOES NOT INCREASE.

MODEL FIT INFORMATION

Number of Free Parameters 49

MODEL RESULTS

	Estimate	Posterior S.D.	One-Tailed P-Value	95% C.I. Lower 2.5%	95% C.I. Upper 2.5%	Significance
**Within Level**						
PHYCAT16 ON						
PHY15	0.971	0.069	0.000	0.843	1.112	*
CHOICE15	-0.069	0.025	0.002	-0.119	-0.019	*
DUTIES15	-0.032	0.027	0.109	-0.085	0.023	
PRESS15	-0.034	0.026	0.102	-0.084	0.018	
KES15	0.229	0.044	0.000	0.144	0.313	*
KES15 WITH						
PHY15	0.051	0.004	0.000	0.044	0.059	*
CHOICE15	-0.089	0.008	0.000	-0.104	-0.074	*
BULLY15	0.057	0.004	0.000	0.049	0.064	*
PRESS15	0.100	0.008	0.000	0.085	0.114	*
DUTIES15	-0.106	0.007	0.000	-0.119	-0.091	*
PHY15 WITH						
CHOICE15	-0.036	0.007	0.000	-0.050	-0.022	*
BULLY15	0.011	0.003	0.000	0.004	0.018	*
PRESS15	0.039	0.007	0.000	0.026	0.052	*
DUTIES15	-0.036	0.006	0.000	-0.048	-0.024	*
CHOICE15 WITH						
BULLY15	-0.057	0.007	0.000	-0.071	-0.043	*
PRESS15	-0.088	0.015	0.000	-0.117	-0.058	*
DUTIES15	0.172	0.014	0.000	0.146	0.201	*
BULLY15 WITH						
PRESS15	0.105	0.007	0.000	0.090	0.119	*
DUTIES15	-0.075	0.006	0.000	-0.088	-0.062	*
PRESS15 WITH						
DUTIES15	-0.186	0.014	0.000	-0.212	-0.161	*
Means						
PHY15	0.003	0.007	0.355	-0.011	0.016	

KES15	0.001	0.008	0.432	-0.013	0.016	
CHOICE15	-0.001	0.015	0.479	-0.031	0.028	
PRESS15	0.000	0.014	0.498	-0.028	0.028	
DUTIES15	-0.002	0.013	0.459	-0.027	0.025	
Variances						
PHY15	0.233	0.005	0.000	0.224	0.243	*
KES15	0.289	0.006	0.000	0.279	0.301	*
CHOICE15	1.083	0.022	0.000	1.041	1.126	*
BULLY15	0.238	0.005	0.000	0.229	0.248	*
PRESS15	1.003	0.020	0.000	0.965	1.044	*
DUTIES15	0.861	0.017	0.000	0.828	0.896	*
Between Level						
S1 ON						
CLU2	-0.210	0.230	0.185	-0.658	0.245	
CLU3	-0.025	0.210	0.440	-0.424	0.416	
PHYCAT16 ON						
CLU2	-0.196	0.100	0.013	-0.408	-0.019	*
CLU3	-0.012	0.092	0.447	-0.193	0.169	
BULLY15	0.063	0.592	0.452	-1.135	1.166	
CLU2 WITH						
CLU3	-0.137	0.038	0.000	-0.227	-0.078	*
BULLY15	0.002	0.011	0.419	-0.020	0.024	
CLU3 WITH						
BULLY15	0.000	0.012	0.493	-0.023	0.023	
Means						
CLU2	0.309	0.064	0.000	0.181	0.438	*
CLU3	0.447	0.069	0.000	0.307	0.580	*
BULLY15	1.167	0.023	0.000	1.121	1.211	*
Intercepts						
S1	0.072	0.181	0.346	-0.313	0.404	
Thresholds						
PHYCAT16$1	1.350	0.678	0.029	-0.056	2.639	
Variances						
CLU2	0.230	0.044	0.000	0.163	0.338	*

```
    CLU3               0.262       0.052      0.000       0.187       0.386      *
    BULLY15            0.024       0.005      0.000       0.017       0.036      *

 Residual Variances
    PHYCAT16           0.014       0.012      0.000       0.001       0.046      *
    S1                 0.069       0.065      0.000       0.012       0.254      *


STANDARDIZED MODEL RESULTS


STDYX Standardization

                                Posterior  One-Tailed         95% C.I.
                    Estimate       S.D.      P-Value   Lower 2.5%  Upper 2.5%  Significance

Within-Level Standardized Estimates Averaged Over Clusters

 S1 | PHYCAT16 ON
    BULLY15           -0.004       0.033      0.448      -0.076       0.054

 PHYCAT16   ON
    PHY15              0.413       0.024      0.000       0.364       0.459      *
    CHOICE15          -0.063       0.023      0.003      -0.108      -0.018      *
    DUTIES15          -0.026       0.023      0.123      -0.070       0.018
    PRESS15           -0.029       0.023      0.107      -0.073       0.016
    KES15              0.108       0.021      0.000       0.066       0.148      *

 KES15    WITH
    PHY15              0.198       0.014      0.000       0.171       0.225      *
    CHOICE15          -0.159       0.013      0.000      -0.186      -0.133      *
    BULLY15            0.216       0.013      0.000       0.190       0.242      *
    PRESS15            0.186       0.013      0.000       0.159       0.212      *
    DUTIES15          -0.212       0.013      0.000      -0.237      -0.186      *

 PHY15    WITH
    CHOICE15          -0.072       0.014      0.000      -0.099      -0.044      *
    BULLY15            0.049       0.014      0.000       0.019       0.077      *
    PRESS15            0.080       0.014      0.000       0.052       0.106      *
    DUTIES15          -0.080       0.014      0.000      -0.107      -0.054      *

 CHOICE15 WITH
    BULLY15           -0.113       0.014      0.000      -0.140      -0.085      *
    PRESS15           -0.084       0.014      0.000      -0.111      -0.057      *
```

```
   DUTIES15           0.179       0.014      0.000       0.152       0.205      *

 BULLY15  WITH
   PRESS15            0.214       0.014      0.000       0.188       0.241      *
   DUTIES15          -0.167       0.014      0.000      -0.194      -0.139      *

 PRESS15  WITH
   DUTIES15          -0.200       0.014      0.000      -0.227      -0.173      *

 Means
   PHY15              0.005       0.014      0.372      -0.022       0.031
   KES15              0.002       0.014      0.428      -0.024       0.030
   CHOICE15          -0.001       0.014      0.470      -0.029       0.026
   PRESS15            0.000       0.014      0.493      -0.028       0.028
   DUTIES15          -0.002       0.014      0.461      -0.029       0.027

 Variances
   PHY15              1.000       0.000      0.000       1.000       1.000
   KES15              1.000       0.000      0.000       1.000       1.000
   CHOICE15           1.000       0.000      0.000       1.000       1.000
   BULLY15            1.000       0.000      0.000       1.000       1.000
   PRESS15            1.000       0.000      0.000       1.000       1.000
   DUTIES15           1.000       0.000      0.000       1.000       1.000

Between Level

 S1         ON
   CLU2              -0.347       0.342      0.185      -0.881       0.456
   CLU3              -0.045       0.354      0.440      -0.710       0.666

 PHYCAT16   ON
   CLU2              -0.548       0.238      0.013      -0.964      -0.051      *
   CLU3              -0.034       0.263      0.447      -0.538       0.512
   BULLY15            0.071       0.448      0.452      -0.795       0.796

 CLU2     WITH
   CLU3              -0.565       0.086      0.000      -0.718      -0.372      *
   BULLY15            0.032       0.141      0.419      -0.256       0.314

 CLU3     WITH
   BULLY15            0.004       0.137      0.493      -0.264       0.265

 Means
   CLU2               0.648       0.143      0.000       0.364       0.928      *
```

CLU3	0.877	0.156	0.000	0.555	1.172	*
BULLY15	7.524	0.746	0.000	6.137	9.016	*
**Intercepts**						
S1	0.226	0.607	0.346	-0.919	1.451	
**Variances**						
CLU2	1.000	0.000	0.000	1.000	1.000	
CLU3	1.000	0.000	0.000	1.000	1.000	
BULLY15	1.000	0.000	0.000	1.000	1.000	
**Residual Variances**						
PHYCAT16	0.454	0.211	0.000	0.060	0.862	*
S1	0.806	0.163	0.000	0.385	0.992	*

R-SQUARE

Within-Level R-Square Averaged Across Clusters

Variable	Estimate	Posterior S.D.	One-Tailed P-Value	95% C.I. Lower 2.5%	Upper 2.5%
PHYCAT16	0.230	0.022	0.000	0.189	0.278

Between Level

Variable	Estimate	Posterior S.D.	One-Tailed P-Value	95% C.I. Lower 2.5%	Upper 2.5%
PHYCAT16	0.546	0.211	0.000	0.138	0.940

Variable	Estimate	Posterior S.D.	One-Tailed P-Value	95% C.I. Lower 2.5%	Upper 2.5%
S1	0.194	0.163	0.000	0.008	0.615

TECHNICAL 1 OUTPUT

PARAMETER SPECIFICATION FOR WITHIN

TAU

PHYCAT16
0

NU

PHYCAT16	PHY15	KES15	CHOICE15	BULLY15
0	0	0	0	0

NU

PRESS15	DUTIES15
0	0

LAMBDA

	PHYCAT16	PHY15	KES15	CHOICE15	BULLY15
PHYCAT16	0	0	0	0	0
PHY15	0	0	0	0	0
KES15	0	0	0	0	0
CHOICE15	0	0	0	0	0
BULLY15	0	0	0	0	0
PRESS15	0	0	0	0	0
DUTIES15	0	0	0	0	0

LAMBDA

	PRESS15	DUTIES15
PHYCAT16	0	0
PHY15	0	0
KES15	0	0
CHOICE15	0	0
BULLY15	0	0
PRESS15	0	0
DUTIES15	0	0

THETA

PHYCAT16 PHY15 KES15 CHOICE15 BULLY15

PHYCAT16	0				
PHY15	0	0			
KES15	0	0	0		
CHOICE15	0	0	0	0	
BULLY15	0	0	0	0	0
PRESS15	0	0	0	0	0
DUTIES15	0	0	0	0	0

THETA

	PRESS15	DUTIES15
PRESS15	0	
DUTIES15	0	0

ALPHA

PHYCAT16	PHY15	KES15	CHOICE15	BULLY15
0	1	2	3	0

ALPHA

PRESS15	DUTIES15
4	5

BETA

	PHYCAT16	PHY15	KES15	CHOICE15	BULLY15
PHYCAT16	0	6	7	8	0
PHY15	0	0	0	0	0
KES15	0	0	0	0	0
CHOICE15	0	0	0	0	0
BULLY15	0	0	0	0	0
PRESS15	0	0	0	0	0
DUTIES15	0	0	0	0	0

BETA

PRESS15	DUTIES15

PHYCAT16	9	10
PHY15	0	0
KES15	0	0
CHOICE15	0	0
BULLY15	0	0
PRESS15	0	0
DUTIES15	0	0

PSI

	PHYCAT16	PHY15	KES15	CHOICE15	BULLY15
PHYCAT16	0				
PHY15	0	11			
KES15	0	12	13		
CHOICE15	0	14	15	16	
BULLY15	0	17	18	19	20
PRESS15	0	21	22	23	24
DUTIES15	0	26	27	28	29

PSI

	PRESS15	DUTIES15
PRESS15	25	
DUTIES15	30	31

PARAMETER SPECIFICATION FOR BETWEEN

TAU

PHYCAT16
49

NU

PHYCAT16	CLU2	CLU3	BULLY15
0	0	0	0

LAMBDA

	S1	PHYCAT16	CLU2	CLU3	BULLY15
PHYCAT16	0	0	0	0	0
CLU2	0	0	0	0	0
CLU3	0	0	0	0	0
BULLY15	0	0	0	0	0

THETA

	PHYCAT16	CLU2	CLU3	BULLY15
PHYCAT16	0			
CLU2	0	0		
CLU3	0	0	0	
BULLY15	0	0	0	0

ALPHA

S1	PHYCAT16	CLU2	CLU3	BULLY15
32	0	33	34	35

BETA

	S1	PHYCAT16	CLU2	CLU3	BULLY15
S1	0	0	36	37	0
PHYCAT16	0	0	38	39	40
CLU2	0	0	0	0	0
CLU3	0	0	0	0	0
BULLY15	0	0	0	0	0

PSI

	S1	PHYCAT16	CLU2	CLU3	BULLY15
S1	41				
PHYCAT16	0	42			
CLU2	0	0	43		
CLU3	0	0	44	45	
BULLY15	0	0	46	47	48

STARTING VALUES FOR WITHIN

TAU

	PHYCAT16
	0.000

NU

PHYCAT16	PHY15	KES15	CHOICE15	BULLY15
0.000	0.000	0.000	0.000	0.000

NU

PRESS15	DUTIES15
0.000	0.000

LAMBDA

	PHYCAT16	PHY15	KES15	CHOICE15	BULLY15
PHYCAT16	1.000	0.000	0.000	0.000	0.000
PHY15	0.000	1.000	0.000	0.000	0.000
KES15	0.000	0.000	1.000	0.000	0.000
CHOICE15	0.000	0.000	0.000	1.000	0.000
BULLY15	0.000	0.000	0.000	0.000	1.000
PRESS15	0.000	0.000	0.000	0.000	0.000
DUTIES15	0.000	0.000	0.000	0.000	0.000

LAMBDA

	PRESS15	DUTIES15
PHYCAT16	0.000	0.000
PHY15	0.000	0.000
KES15	0.000	0.000
CHOICE15	0.000	0.000
BULLY15	0.000	0.000
PRESS15	1.000	0.000
DUTIES15	0.000	1.000

THETA

	PHYCAT16	PHY15	KES15	CHOICE15	BULLY15
PHYCAT16	0.000				
PHY15	0.000	0.000			
KES15	0.000	0.000	0.000		
CHOICE15	0.000	0.000	0.000	0.000	
BULLY15	0.000	0.000	0.000	0.000	0.000
PRESS15	0.000	0.000	0.000	0.000	0.000
DUTIES15	0.000	0.000	0.000	0.000	0.000

THETA

	PRESS15	DUTIES15
PRESS15	0.000	
DUTIES15	0.000	0.000

ALPHA

PHYCAT16	PHY15	KES15	CHOICE15	BULLY15
0.000	0.000	0.000	0.000	0.000

ALPHA

PRESS15	DUTIES15
0.000	0.000

BETA

	PHYCAT16	PHY15	KES15	CHOICE15	BULLY15
PHYCAT16	0.000	0.000	0.000	0.000	0.000
PHY15	0.000	0.000	0.000	0.000	0.000
KES15	0.000	0.000	0.000	0.000	0.000
CHOICE15	0.000	0.000	0.000	0.000	0.000
BULLY15	0.000	0.000	0.000	0.000	0.000
PRESS15	0.000	0.000	0.000	0.000	0.000
DUTIES15	0.000	0.000	0.000	0.000	0.000

BETA

	PRESS15	DUTIES15
PHYCAT16	0.000	0.000
PHY15	0.000	0.000
KES15	0.000	0.000
CHOICE15	0.000	0.000
BULLY15	0.000	0.000
PRESS15	0.000	0.000
DUTIES15	0.000	0.000

PSI

	PHYCAT16	PHY15	KES15	CHOICE15	BULLY15
PHYCAT16	1.000				
PHY15	0.000	0.116			
KES15	0.000	0.000	0.145		
CHOICE15	0.000	0.000	0.000	0.541	
BULLY15	0.000	0.000	0.000	0.000	0.119
PRESS15	0.000	0.000	0.000	0.000	0.000
DUTIES15	0.000	0.000	0.000	0.000	0.000

PSI

	PRESS15	DUTIES15
PRESS15	0.501	
DUTIES15	0.000	0.430

STARTING VALUES FOR BETWEEN

TAU

PHYCAT16
1.110

NU

PHYCAT16	CLU2	CLU3	BULLY15
0.000	0.000	0.000	0.000

LAMBDA

	S1	PHYCAT16	CLU2	CLU3	BULLY15
PHYCAT16	0.000	1.000	0.000	0.000	0.000
CLU2	0.000	0.000	1.000	0.000	0.000
CLU3	0.000	0.000	0.000	1.000	0.000
BULLY15	0.000	0.000	0.000	0.000	1.000

THETA

	PHYCAT16	CLU2	CLU3	BULLY15
PHYCAT16	0.000			
CLU2	0.000	0.000		
CLU3	0.000	0.000	0.000	
BULLY15	0.000	0.000	0.000	0.000

ALPHA

S1	PHYCAT16	CLU2	CLU3	BULLY15
0.000	0.000	0.406	0.471	1.170

BETA

	S1	PHYCAT16	CLU2	CLU3	BULLY15
S1	0.000	0.000	0.000	0.000	0.000
PHYCAT16	0.000	0.000	0.000	0.000	0.000
CLU2	0.000	0.000	0.000	0.000	0.000
CLU3	0.000	0.000	0.000	0.000	0.000
BULLY15	0.000	0.000	0.000	0.000	0.000

PSI

	S1	PHYCAT16	CLU2	CLU3	BULLY15
S1	1.000				
PHYCAT16	0.000	1.000			
CLU2	0.000	0.000	0.121		
CLU3	0.000	0.000	0.000	0.125	
BULLY15	0.000	0.000	0.000	0.000	0.119

PRIORS FOR ALL PARAMETERS	PRIOR MEAN	PRIOR VARIANCE	PRIOR STD. DEV.
Parameter 1~N(0.000,infinity)	0.0000	infinity	infinity
Parameter 2~N(0.000,infinity)	0.0000	infinity	infinity
Parameter 3~N(0.000,infinity)	0.0000	infinity	infinity
Parameter 4~N(0.000,infinity)	0.0000	infinity	infinity
Parameter 5~N(0.000,infinity)	0.0000	infinity	infinity
Parameter 6~N(0.000,5.000)	0.0000	5.0000	2.2361
Parameter 7~N(0.000,5.000)	0.0000	5.0000	2.2361
Parameter 8~N(0.000,5.000)	0.0000	5.0000	2.2361
Parameter 9~N(0.000,5.000)	0.0000	5.0000	2.2361
Parameter 10~N(0.000,5.000)	0.0000	5.0000	2.2361
Parameter 11~IW(1.000,7)	infinity	infinity	infinity
Parameter 12~IW(0.000,7)	infinity	infinity	infinity
Parameter 13~IW(1.000,7)	infinity	infinity	infinity
Parameter 14~IW(0.000,7)	infinity	infinity	infinity
Parameter 15~IW(0.000,7)	infinity	infinity	infinity
Parameter 16~IW(1.000,7)	infinity	infinity	infinity
Parameter 17~IW(0.000,7)	infinity	infinity	infinity
Parameter 18~IW(0.000,7)	infinity	infinity	infinity
Parameter 19~IW(0.000,7)	infinity	infinity	infinity
Parameter 20~IW(1.000,7)	infinity	infinity	infinity
Parameter 21~IW(0.000,7)	infinity	infinity	infinity
Parameter 22~IW(0.000,7)	infinity	infinity	infinity
Parameter 23~IW(0.000,7)	infinity	infinity	infinity
Parameter 24~IW(0.000,7)	infinity	infinity	infinity
Parameter 25~IW(1.000,7)	infinity	infinity	infinity
Parameter 26~IW(0.000,7)	infinity	infinity	infinity
Parameter 27~IW(0.000,7)	infinity	infinity	infinity
Parameter 28~IW(0.000,7)	infinity	infinity	infinity
Parameter 29~IW(0.000,7)	infinity	infinity	infinity
Parameter 30~IW(0.000,7)	infinity	infinity	infinity
Parameter 31~IW(1.000,7)	infinity	infinity	infinity
Parameter 32~N(0.000,infinity)	0.0000	infinity	infinity
Parameter 33~N(0.000,infinity)	0.0000	infinity	infinity
Parameter 34~N(0.000,infinity)	0.0000	infinity	infinity
Parameter 35~N(0.000,infinity)	0.0000	infinity	infinity
Parameter 36~N(0.000,infinity)	0.0000	infinity	infinity
Parameter 37~N(0.000,infinity)	0.0000	infinity	infinity
Parameter 38~N(0.000,5.000)	0.0000	5.0000	2.2361
Parameter 39~N(0.000,5.000)	0.0000	5.0000	2.2361
Parameter 40~N(0.000,5.000)	0.0000	5.0000	2.2361

Parameter 41~IG(-1.000,0.000)	infinity	infinity	infinity
Parameter 42~IG(-1.000,0.000)	infinity	infinity	infinity
Parameter 43~IW(1.000,4)	infinity	infinity	infinity
Parameter 44~IW(0.000,4)	infinity	infinity	infinity
Parameter 45~IW(1.000,4)	infinity	infinity	infinity
Parameter 46~IW(0.000,4)	infinity	infinity	infinity
Parameter 47~IW(0.000,4)	infinity	infinity	infinity
Parameter 48~IW(1.000,4)	infinity	infinity	infinity
Parameter 49~N(0.000,5.000)	0.0000	5.0000	2.2361

TECHNICAL 3 OUTPUT

ESTIMATED COVARIANCE MATRIX FOR PARAMETER ESTIMATES

	1	2	3	4	5
1	0.438474D-04				
2	0.115267D-04	0.570655D-04			
3	-0.618484D-05	-0.201064D-04	0.215709D-03		
4	0.565003D-05	0.208145D-04	-0.183937D-04	0.207455D-03	
5	-0.125749D-04	-0.267715D-04	0.347462D-04	-0.435647D-04	0.170423D-03
6	0.119547D-04	0.137243D-05	-0.278384D-04	-0.427709D-04	0.219653D-04
7	-0.118172D-04	0.468951D-05	0.541065D-06	0.112175D-04	-0.270745D-04
8	0.508295D-05	0.428626D-05	-0.174671D-05	-0.542312D-05	-0.759642D-05
9	-0.160265D-05	-0.748908D-05	0.151276D-04	0.486678D-05	0.933624D-05
10	-0.173018D-05	-0.404663D-05	0.109615D-04	-0.931950D-05	0.728322D-05
11	0.178496D-06	-0.137537D-06	0.294819D-05	-0.146832D-05	0.352004D-06
12	-0.851049D-06	-0.905962D-08	-0.452286D-06	-0.766681D-06	-0.280527D-06
13	0.889600D-06	0.121640D-05	-0.482421D-05	-0.160888D-05	-0.458697D-06
14	0.617947D-06	-0.136381D-05	0.301114D-06	-0.195094D-05	-0.160165D-05
15	0.848289D-06	0.128972D-05	0.552877D-05	0.381977D-05	-0.239274D-05
16	-0.163844D-05	-0.302646D-06	-0.787026D-05	-0.364769D-05	0.994719D-05
17	-0.164934D-05	-0.294595D-06	-0.500719D-06	0.170459D-06	0.315104D-06
18	-0.716718D-06	0.971100D-06	-0.270752D-05	0.151517D-05	0.178381D-06
19	-0.573818D-06	0.106527D-05	0.241565D-05	0.312586D-05	-0.115894D-05
20	-0.546051D-06	0.122345D-06	0.132843D-05	-0.165551D-05	0.563090D-06
21	0.639822D-06	0.835742D-06	-0.314474D-05	-0.122576D-06	-0.628876D-06
22	-0.107235D-05	0.195672D-06	-0.322961D-05	-0.321270D-05	-0.296957D-05
23	0.233535D-05	-0.238177D-05	0.149379D-06	0.559344D-05	-0.141713D-05
24	0.819659D-06	-0.738892D-07	0.151157D-05	0.263703D-05	-0.336618D-07
25	-0.332760D-05	0.156527D-05	-0.456607D-05	0.955464D-05	-0.267873D-05
26	0.152320D-05	-0.201984D-05	0.258711D-05	0.225929D-05	0.163785D-05
27	-0.259859D-06	0.222568D-07	0.303265D-05	0.458038D-05	0.131481D-06

28	-0.426439D-06	0.217261D-05	-0.564683D-05	-0.341032D-05	-0.204174D-05
29	0.850761D-06	0.786846D-06	0.217610D-05	-0.642011D-06	-0.949172D-06
30	0.173160D-05	-0.294251D-05	0.967134D-06	-0.203469D-05	0.102624D-05
31	-0.607028D-05	-0.366446D-06	0.127249D-05	-0.329367D-05	-0.980295D-06
32	0.135574D-04	0.206132D-04	0.182776D-04	0.512579D-04	0.348290D-04
33	-0.878017D-05	-0.804689D-05	-0.249420D-04	-0.816290D-05	-0.114274D-04
34	0.654979D-05	0.805822D-05	0.261149D-04	-0.302098D-05	0.155103D-04
35	0.256473D-05	0.840354D-05	-0.101491D-04	0.264044D-04	-0.203093D-04
36	-0.722545D-04	-0.508533D-05	-0.184184D-04	-0.572535D-05	-0.225752D-04
37	-0.256984D-04	-0.244423D-04	-0.224506D-04	-0.948532D-04	0.266600D-04
38	-0.144967D-04	-0.378362D-04	0.397418D-04	-0.377439D-04	0.963262D-05
39	0.237154D-05	-0.363222D-05	-0.298846D-04	-0.106595D-05	0.701036D-05
40	0.341367D-04	0.212692D-04	0.155766D-03	-0.646083D-04	0.263957D-05
41	-0.455039D-05	-0.142209D-04	-0.254482D-04	0.163952D-05	0.150546D-04
42	0.150090D-05	0.377741D-05	-0.611069D-05	0.256400D-05	0.435980D-05
43	-0.389676D-05	-0.171512D-04	0.200255D-04	0.223249D-04	0.271471D-04
44	0.522171D-05	0.118441D-04	-0.133649D-04	-0.163585D-04	-0.270753D-04
45	-0.721126D-05	-0.117598D-04	0.415765D-05	-0.125013D-04	0.435441D-04
46	0.422746D-07	-0.987107D-06	-0.253026D-05	0.110534D-04	0.276114D-05
47	0.144337D-06	0.266454D-05	0.443616D-05	-0.653080D-05	-0.460903D-06
48	0.127959D-05	0.111500D-05	-0.153148D-05	0.903212D-06	-0.323874D-05
49	0.436256D-04	0.526684D-05	0.156767D-03	-0.109156D-03	0.166401D-04

ESTIMATED COVARIANCE MATRIX FOR PARAMETER ESTIMATES

	6	7	8	9	10
6	0.476778D-02				
7	-0.285107D-03	0.192495D-02			
8	-0.365178D-05	0.113344D-03	0.628773D-03		
9	-0.799162D-04	-0.117993D-03	0.445918D-04	0.681609D-03	
10	-0.173454D-04	0.102359D-03	-0.864812D-04	0.888889D-04	0.753151D-03
11	-0.174050D-05	-0.623192D-05	-0.857701D-06	0.502180D-06	0.368889D-06
12	-0.665362D-05	0.459894D-05	-0.145511D-05	-0.221077D-05	-0.111919D-06
13	-0.105320D-05	0.655643D-06	0.863876D-05	0.198600D-05	-0.536286D-05
14	-0.235493D-05	-0.147778D-04	0.287162D-05	0.100599D-05	-0.849117D-05
15	-0.555681D-05	-0.132040D-04	-0.641761D-06	-0.117369D-05	0.904015D-05
16	-0.190783D-04	-0.165810D-05	0.450465D-05	0.263523D-05	0.513651D-05
17	-0.305480D-05	0.415431D-05	0.216400D-06	-0.168914D-05	0.297095D-05
18	0.269875D-05	0.178496D-05	0.213955D-05	-0.267915D-05	-0.635736D-05
19	-0.270788D-06	-0.520178D-05	-0.708545D-05	-0.268992D-06	-0.157852D-05
20	0.151127D-06	-0.518714D-05	0.110054D-05	-0.533484D-05	0.165297D-05
21	0.116617D-04	0.435490D-05	-0.188942D-05	0.520602D-05	0.690750D-05
22	-0.761504D-05	0.160660D-05	-0.125709D-05	-0.356048D-05	-0.657664D-05

23	-0.176256D-05	-0.134659D-04	-0.106954D-05	-0.184498D-05	-0.150510D-04
24	-0.144321D-04	0.138648D-04	-0.223721D-05	0.240912D-05	0.564794D-05
25	-0.476031D-04	0.772000D-05	0.224868D-04	0.207388D-04	0.703979D-05
26	-0.236094D-04	-0.449089D-05	0.165574D-05	-0.169292D-05	-0.410878D-05
27	0.650626D-05	-0.810349D-05	0.256331D-05	0.111228D-05	0.720694D-06
28	-0.271754D-05	0.271034D-04	0.356276D-05	0.249035D-05	0.174428D-05
29	0.633665D-05	-0.122312D-04	-0.255739D-05	0.837405D-05	0.382289D-05
30	0.270623D-04	-0.101504D-04	-0.253024D-05	0.158302D-05	0.177833D-05
31	-0.133903D-04	0.913337D-05	-0.456600D-06	-0.122142D-04	-0.146067D-04
32	0.435759D-03	-0.593307D-03	0.172315D-03	-0.359471D-04	0.114893D-03
33	0.120005D-04	0.548017D-04	-0.318453D-04	-0.709138D-04	-0.625478D-05
34	0.637129D-05	-0.723639D-04	0.664406D-04	0.850136D-05	-0.772460D-05
35	-0.912412D-05	-0.174002D-04	-0.153742D-04	0.237582D-05	0.875620D-05
36	-0.100138D-02	-0.181646D-04	-0.213976D-03	-0.244469D-03	0.143211D-03
37	-0.614554D-03	0.326580D-03	-0.600688D-04	-0.315783D-04	0.108148D-03
38	-0.124678D-03	-0.175961D-03	-0.997735D-04	-0.155988D-03	-0.244814D-04
39	0.401202D-04	-0.173032D-03	0.415979D-04	-0.109273D-03	0.621019D-04
40	-0.725192D-03	0.698693D-03	-0.402233D-03	-0.241679D-03	0.651860D-03
41	0.382117D-03	-0.643454D-04	-0.297037D-04	0.830683D-05	0.948052D-04
42	0.237491D-04	-0.960239D-05	-0.695585D-05	0.219415D-04	0.479286D-05
43	-0.133503D-03	0.461485D-04	0.786138D-05	0.256450D-04	0.427824D-04
44	0.225334D-04	-0.724259D-04	0.187899D-04	-0.198679D-04	-0.190567D-04
45	0.326103D-04	0.440393D-04	-0.246074D-04	0.262720D-04	-0.737622D-06
46	-0.532366D-05	0.166349D-04	0.971651D-05	0.637269D-06	-0.222246D-05
47	0.330280D-05	-0.352849D-04	-0.780737D-05	-0.678704D-05	0.412863D-05
48	0.343632D-05	0.148837D-05	0.672768D-05	-0.105449D-05	-0.435446D-05
49	0.198109D-03	0.757096D-03	-0.535959D-03	-0.425063D-03	0.752835D-03

ESTIMATED COVARIANCE MATRIX FOR PARAMETER ESTIMATES

	11	12	13	14	15
11	0.212239D-04				
12	0.590445D-05	0.144075D-04			
13	0.127020D-05	0.626967D-05	0.320454D-04		
14	-0.307315D-05	-0.469227D-05	-0.172466D-05	0.497998D-04	
15	0.164963D-06	-0.268415D-05	-0.102591D-04	0.139881D-04	0.607528D-04
16	-0.154283D-05	-0.296901D-06	0.590686D-05	-0.156366D-04	-0.459517D-04
17	0.164441D-05	0.295810D-05	0.114036D-05	-0.320049D-05	-0.137998D-05
18	0.906554D-06	0.985845D-06	0.568163D-05	-0.225833D-05	-0.470389D-05
19	-0.154204D-05	-0.166545D-06	-0.206175D-05	0.468923D-05	0.147217D-04
20	0.247260D-05	0.107938D-05	0.120793D-05	-0.162454D-05	-0.256894D-05
21	0.310546D-05	0.532162D-05	0.138725D-05	-0.501619D-05	-0.383557D-06
22	0.233537D-06	0.263916D-05	0.112212D-04	-0.346272D-07	-0.627155D-05

23	-0.772462D-06	-0.258212D-05	-0.127731D-05	0.891415D-05	0.245072D-04
24	0.730655D-06	0.348520D-06	0.200217D-05	-0.657053D-06	-0.210336D-05
25	-0.984873D-06	-0.200895D-05	0.879830D-06	0.318709D-06	0.343119D-05
26	-0.310499D-05	-0.581914D-05	-0.329968D-05	0.898510D-05	0.285623D-05
27	-0.549533D-06	-0.313870D-05	-0.101486D-04	0.325197D-06	0.118469D-04
28	0.184418D-05	0.822178D-07	0.239768D-05	-0.945383D-05	-0.232986D-04
29	-0.155975D-05	-0.796809D-06	-0.107108D-05	0.164548D-05	0.401788D-05
30	-0.234617D-06	-0.215808D-05	-0.178351D-05	0.647490D-06	0.249382D-05
31	-0.560179D-07	-0.110004D-06	0.257117D-05	0.940649D-06	-0.837122D-05
32	-0.438863D-04	-0.622304D-05	-0.204957D-04	0.404051D-04	0.270773D-04
33	0.592668D-06	-0.246666D-05	-0.837509D-05	-0.510288D-05	-0.221788D-04
34	-0.173780D-04	0.352786D-05	-0.624864D-06	-0.573753D-05	0.113772D-04
35	-0.126732D-05	-0.110938D-06	0.465799D-05	0.701763D-06	-0.573992D-05
36	0.450029D-04	0.494635D-05	0.929039D-05	-0.364647D-04	0.829105D-05
37	0.740387D-04	0.194490D-04	0.305172D-04	-0.555049D-04	-0.314401D-04
38	0.930844D-05	-0.354478D-05	0.312076D-04	-0.181725D-04	-0.253177D-04
39	0.544382D-05	-0.609487D-05	0.150229D-04	0.195176D-05	-0.371446D-05
40	-0.441412D-04	-0.204521D-04	0.105546D-03	-0.944131D-04	0.344046D-04
41	0.508536D-05	-0.136052D-04	0.248984D-05	0.710995D-05	-0.103917D-04
42	-0.103402D-05	-0.186979D-05	-0.523459D-08	0.291013D-05	0.365854D-05
43	0.863618D-05	-0.596103D-06	-0.587044D-05	-0.586201D-06	0.104167D-05
44	-0.465335D-05	0.127637D-05	0.278201D-05	0.888942D-05	0.685584D-05
45	0.494802D-05	0.103249D-05	-0.440442D-05	-0.132420D-04	-0.203632D-04
46	0.426425D-06	-0.519898D-06	0.118222D-05	0.123112D-05	0.930780D-06
47	0.175052D-05	0.678867D-06	0.196323D-06	0.145604D-05	0.449125D-06
48	-0.258673D-07	-0.353218D-07	0.252982D-06	-0.722578D-06	0.346598D-06
49	-0.480733D-04	-0.319220D-04	0.141205D-03	-0.114680D-03	0.212003D-04

ESTIMATED COVARIANCE MATRIX FOR PARAMETER ESTIMATES

	16	17	18	19	20
16	0.492216D-03				
17	0.924552D-06	0.116216D-04			
18	0.609865D-05	0.282214D-05	0.138844D-04		
19	-0.274707D-04	-0.207274D-05	-0.595862D-05	0.534078D-04	
20	-0.678888D-06	0.158416D-05	0.514167D-05	-0.603829D-05	0.236278D-04
21	-0.795691D-06	0.580346D-05	0.154701D-05	-0.153120D-05	0.104860D-05
22	0.906921D-05	0.165997D-05	0.544091D-05	-0.338914D-05	0.204013D-05
23	-0.492571D-04	-0.209445D-05	-0.350408D-05	0.270227D-04	-0.591697D-05
24	0.105519D-04	0.235831D-05	0.562400D-05	-0.620174D-05	0.114756D-04
25	0.190317D-04	-0.164639D-05	0.247228D-05	-0.659705D-06	0.146819D-05
26	-0.289867D-06	-0.388471D-05	-0.127604D-05	0.784421D-06	-0.142420D-05
27	-0.882114D-05	-0.701541D-06	-0.389315D-05	0.361559D-05	-0.153058D-05

28	0.726238D-04	-0.894740D-06	0.130395D-05	-0.193118D-04	0.126053D-05
29	-0.610687D-05	-0.176455D-05	-0.608999D-05	0.824782D-05	-0.742528D-05
30	-0.155355D-04	-0.152849D-05	-0.107919D-05	-0.445096D-06	-0.110767D-05
31	0.414691D-05	0.132101D-05	0.161738D-05	-0.729488D-05	0.230357D-05
32	0.169084D-04	-0.202212D-04	-0.104933D-04	-0.128315D-05	0.476216D-05
33	-0.544097D-05	0.409247D-05	-0.620346D-05	-0.713873D-05	0.244096D-04
34	0.172973D-04	-0.168296D-05	-0.695761D-05	0.183426D-04	-0.193636D-04
35	0.543385D-05	0.193237D-05	0.319866D-05	-0.112202D-04	0.139020D-05
36	-0.808368D-05	0.100882D-04	-0.515085D-05	-0.324078D-04	-0.781296D-05
37	-0.461747D-04	0.193349D-05	0.297199D-05	0.123560D-04	-0.125979D-04
38	-0.504431D-05	-0.602927D-05	-0.358836D-05	-0.200964D-05	0.106371D-04
39	0.552781D-04	0.116540D-04	-0.104250D-04	-0.116219D-04	-0.490070D-05
40	-0.116630D-03	-0.242124D-04	0.960915D-04	-0.436853D-04	-0.155654D-04
41	-0.903921D-05	-0.327245D-05	0.135883D-04	0.856537D-05	0.129792D-04
42	0.220793D-05	0.291285D-06	0.234392D-06	-0.244663D-05	-0.523759D-06
43	0.534529D-04	-0.638967D-06	-0.236710D-05	0.102755D-04	-0.608539D-05
44	-0.199502D-04	0.170967D-05	-0.324137D-05	-0.976612D-06	0.622166D-05
45	0.163161D-05	-0.155489D-05	0.128238D-05	-0.587410D-05	-0.101254D-04
46	0.149181D-05	-0.355024D-06	0.152931D-05	-0.553738D-05	0.425193D-06
47	-0.883361D-05	0.170734D-05	0.675512D-06	0.163549D-05	-0.189833D-05
48	-0.627295D-05	0.320966D-06	0.266484D-06	-0.640165D-06	0.165982D-05
49	-0.117273D-03	-0.257219D-04	0.112586D-03	-0.685786D-04	-0.127222D-04

ESTIMATED COVARIANCE MATRIX FOR PARAMETER ESTIMATES

	21	22	23	24	25
21	0.465112D-04				
22	0.101075D-04	0.565764D-04			
23	-0.978518D-05	-0.183348D-04	0.217077D-03		
24	0.289198D-05	0.118838D-04	-0.170527D-04	0.519895D-04	
25	0.100079D-04	0.367785D-04	-0.373299D-04	0.455431D-04	0.408190D-03
26	-0.939358D-05	-0.220069D-05	0.183557D-05	0.354759D-06	0.781995D-06
27	-0.336725D-05	-0.109095D-04	0.534165D-05	-0.146047D-05	-0.196718D-05
28	-0.103060D-06	0.218825D-05	-0.438575D-04	0.349600D-05	0.536236D-05
29	-0.289728D-05	-0.404285D-05	0.672240D-05	-0.100559D-04	-0.775180D-05
30	-0.815532D-05	-0.217806D-04	0.404040D-04	-0.198272D-04	-0.789041D-04
31	-0.353039D-06	0.666654D-05	-0.159770D-04	0.689790D-05	0.661813D-05
32	-0.202900D-04	0.264531D-05	0.286017D-04	0.932530D-05	0.536392D-04
33	-0.858273D-05	0.759161D-05	-0.474641D-04	0.105730D-04	0.301376D-04
34	0.948826D-05	-0.113824D-04	0.544747D-04	-0.158399D-04	-0.638106D-04
35	0.226979D-05	0.311079D-05	-0.176713D-04	0.138575D-05	-0.685607D-06
36	0.481119D-05	-0.344454D-05	0.682458D-04	0.174680D-04	0.201615D-04
37	0.127604D-04	-0.396842D-04	-0.288617D-04	-0.146200D-04	-0.418443D-04

38	-0.233862D-04	-0.137538D-04	0.291413D-04	-0.456080D-07	-0.212037D-04
39	-0.245166D-04	-0.171619D-04	0.307902D-04	-0.173930D-04	-0.749104D-04
40	0.103163D-03	0.287100D-03	-0.457523D-03	0.393805D-04	0.633033D-03
41	-0.478741D-05	0.255447D-05	-0.420846D-04	-0.420344D-05	-0.413128D-04
42	-0.402901D-05	0.347947D-05	0.166017D-05	0.241079D-05	0.674422D-05
43	-0.129290D-06	-0.104084D-04	0.119751D-04	-0.614699D-05	-0.155041D-04
44	-0.533459D-06	0.818094D-05	-0.687242D-05	0.145416D-04	0.188880D-04
45	0.893249D-06	-0.265837D-05	0.667366D-05	-0.148404D-04	-0.187301D-04
46	-0.161100D-05	-0.304630D-05	-0.300482D-05	-0.391296D-05	-0.596883D-05
47	0.168810D-05	0.432523D-05	-0.232957D-06	0.154770D-05	0.529257D-05
48	0.505955D-07	-0.402917D-06	0.195301D-05	-0.598128D-06	-0.238364D-05
49	0.102658D-03	0.338017D-03	-0.545033D-03	0.415552D-04	0.697797D-03

ESTIMATED COVARIANCE MATRIX FOR PARAMETER ESTIMATES

	26	27	28	29	30
26	0.387839D-04				
27	0.891890D-05	0.498486D-04			
28	-0.632505D-05	-0.145837D-04	0.189400D-03		
29	0.324246D-05	0.116316D-04	-0.131116D-04	0.400386D-04	
30	0.652072D-05	0.170799D-04	-0.249337D-04	0.207277D-04	0.185124D-03
31	-0.387169D-05	-0.370114D-04	0.667727D-04	-0.277899D-04	-0.671792D-04
32	0.393865D-04	0.214494D-05	-0.244340D-05	-0.999996D-05	0.322172D-04
33	-0.291707D-05	0.746646D-07	0.169379D-04	-0.203613D-04	-0.617739D-04
34	-0.255390D-04	0.503593D-05	-0.320041D-04	0.836115D-05	0.395883D-04
35	-0.503685D-06	-0.843807D-06	0.442121D-05	-0.102265D-05	-0.392928D-05
36	-0.746862D-04	0.358040D-04	-0.927386D-04	0.260956D-04	-0.172810D-04
37	-0.478405D-04	0.121022D-04	-0.162183D-04	0.426853D-05	-0.301715D-04
38	0.241453D-04	0.336192D-05	-0.143097D-04	-0.361585D-05	0.475854D-04
39	0.135003D-04	-0.657143D-05	-0.252515D-04	-0.124190D-04	0.288915D-04
40	-0.224558D-03	-0.255106D-03	0.401758D-03	-0.217629D-03	-0.573003D-03
41	0.133048D-04	-0.962475D-05	0.471603D-04	-0.526743D-05	-0.111327D-04
42	0.189210D-05	0.284596D-05	-0.713724D-05	0.540395D-05	-0.152115D-06
43	0.899477D-05	-0.188532D-05	0.448942D-05	0.554727D-05	0.343186D-04
44	-0.816586D-05	0.377715D-05	-0.540463D-05	0.199829D-05	-0.366806D-04
45	-0.140917D-05	0.106261D-06	0.523793D-05	-0.387158D-05	0.207235D-04
46	-0.129595D-06	0.140874D-05	-0.350420D-06	-0.302076D-05	0.479877D-05
47	0.543271D-06	0.273119D-06	-0.469576D-05	0.494998D-05	0.208456D-05
48	-0.130516D-05	0.203988D-06	-0.198651D-05	-0.870970D-06	0.204131D-05
49	-0.245013D-03	-0.298477D-03	0.440069D-03	-0.257988D-03	-0.627423D-03

ESTIMATED COVARIANCE MATRIX FOR PARAMETER ESTIMATES

	31	32	33	34	35
31	0.305989D-03				
32	-0.545693D-04	0.328992D-01			
33	0.354678D-04	0.497175D-03	0.412921D-02		
34	0.132231D-04	-0.109489D-04	-0.246944D-02	0.470498D-02	
35	-0.715696D-05	0.302740D-04	0.600477D-04	-0.326934D-04	0.517667D-03
36	-0.123185D-03	-0.314894D-01	-0.562114D-03	0.171587D-03	0.446740D-04
37	0.706593D-04	-0.325033D-01	-0.296660D-03	0.144455D-03	-0.194729D-04
38	-0.808689D-05	-0.742123D-04	-0.162775D-03	0.354490D-03	0.398396D-04
39	-0.317675D-05	0.485060D-03	-0.699147D-04	0.299592D-03	0.700498D-04
40	-0.249818D-04	-0.140318D-02	0.691731D-03	-0.184876D-03	-0.949100D-03
41	0.169534D-04	-0.126208D-02	-0.176717D-04	0.301111D-04	-0.204254D-04
42	-0.111177D-04	0.562153D-04	-0.114055D-04	0.315563D-06	0.565273D-05
43	0.261862D-05	-0.244446D-03	0.121187D-03	-0.179901D-03	-0.208576D-04
44	-0.324943D-05	0.219531D-03	-0.304691D-04	0.125475D-03	0.271945D-04
45	0.493939D-05	-0.495641D-03	-0.602549D-05	-0.929299D-04	-0.321057D-04
46	-0.495081D-05	0.430492D-04	0.184317D-04	-0.264493D-04	-0.208464D-04
47	-0.265068D-05	-0.773400D-05	-0.160101D-04	0.267976D-04	0.598592D-05
48	0.184250D-05	-0.111887D-04	-0.292652D-06	0.445972D-07	0.672206D-06
49	-0.535946D-04	-0.133145D-02	0.754742D-03	-0.116675D-04	-0.104406D-02

ESTIMATED COVARIANCE MATRIX FOR PARAMETER ESTIMATES

	36	37	38	39	40
36	0.530625D-01				
37	0.324968D-01	0.439128D-01			
38	0.114573D-02	0.249104D-03	0.100777D-01		
39	-0.119284D-03	-0.403131D-03	0.632794D-02	0.851316D-02	
40	-0.537979D-03	-0.252834D-02	-0.113328D-01	-0.103485D-01	0.349824D+00
41	-0.234662D-02	0.612593D-03	-0.738286D-04	-0.135926D-04	-0.130490D-02
42	-0.132991D-03	-0.799439D-04	-0.259126D-03	-0.526274D-04	0.467267D-04
43	0.490030D-03	0.501688D-03	-0.779242D-04	-0.167020D-04	-0.135720D-02
44	-0.155738D-03	-0.352365D-03	0.176455D-04	-0.671963D-05	0.654550D-03
45	0.350412D-03	0.694557D-03	0.978503D-04	0.127778D-04	0.196035D-03
46	-0.985052D-05	-0.107464D-03	-0.163677D-05	0.337667D-04	-0.114629D-03
47	-0.234750D-04	0.374353D-04	0.114915D-04	-0.616739D-06	-0.175856D-03
48	0.224453D-04	0.993961D-06	-0.506357D-06	-0.712395D-05	-0.845934D-05
49	-0.112520D-02	-0.331939D-02	-0.698959D-02	-0.583955D-02	0.398266D+00

ESTIMATED COVARIANCE MATRIX FOR PARAMETER ESTIMATES

41	42	43	44	45

41	0.426948D-02				
42	0.310987D-04	0.145275D-03			
43	0.758838D-04	0.569319D-05	0.194598D-02		
44	-0.487150D-04	-0.560561D-05	-0.115665D-02	0.143465D-02	
45	-0.119325D-03	-0.179862D-05	0.716132D-03	-0.137380D-02	0.268053D-02
46	0.198420D-04	0.574264D-07	0.184518D-04	-0.117391D-04	-0.123161D-04
47	-0.715092D-06	0.619773D-05	-0.893638D-05	0.688969D-05	0.256798D-04
48	-0.513215D-05	-0.107907D-05	0.296762D-07	-0.487558D-05	0.471385D-05
49	-0.110356D-02	0.322305D-04	-0.165940D-02	0.792628D-03	0.249256D-03

ESTIMATED COVARIANCE MATRIX FOR PARAMETER ESTIMATES

	46	47	48	49
46	0.123456D-03			
47	-0.732790D-04	0.134880D-03		
48	0.637450D-06	-0.101639D-05	0.241577D-04	
49	-0.113101D-03	-0.202259D-03	-0.149859D-04	0.459618D+00

ESTIMATED CORRELATION MATRIX FOR PARAMETER ESTIMATES

	1	2	3	4	5
1	1.000				
2	0.230	1.000			
3	-0.064	-0.181	1.000		
4	0.059	0.191	-0.087	1.000	
5	-0.145	-0.271	0.181	-0.232	1.000
6	0.026	0.003	-0.027	-0.043	0.024
7	-0.041	0.014	0.001	0.018	-0.047
8	0.031	0.023	-0.005	-0.015	-0.023
9	-0.009	-0.038	0.039	0.013	0.027
10	-0.010	-0.020	0.027	-0.024	0.020
11	0.006	-0.004	0.044	-0.022	0.006
12	-0.034	0.000	-0.008	-0.014	-0.006
13	0.024	0.028	-0.058	-0.020	-0.006
14	0.013	-0.026	0.003	-0.019	-0.017
15	0.016	0.022	0.048	0.034	-0.024
16	-0.011	-0.002	-0.024	-0.011	0.034
17	-0.073	-0.011	-0.010	0.003	0.007
18	-0.029	0.034	-0.049	0.028	0.004
19	-0.012	0.019	0.023	0.030	-0.012
20	-0.017	0.003	0.019	-0.024	0.009

21	0.014	0.016	-0.031	-0.001	-0.007
22	-0.022	0.003	-0.029	-0.030	-0.030
23	0.024	-0.021	0.001	0.026	-0.007
24	0.017	-0.001	0.014	0.025	0.000
25	-0.025	0.010	-0.015	0.033	-0.010
26	0.037	-0.043	0.028	0.025	0.020
27	-0.006	0.000	0.029	0.045	0.001
28	-0.005	0.021	-0.028	-0.017	-0.011
29	0.020	0.016	0.023	-0.007	-0.011
30	0.019	-0.029	0.005	-0.010	0.006
31	-0.052	-0.003	0.005	-0.013	-0.004
32	0.011	0.015	0.007	0.020	0.015
33	-0.021	-0.017	-0.026	-0.009	-0.014
34	0.014	0.016	0.026	-0.003	0.017
35	0.017	0.049	-0.030	0.081	-0.068
36	-0.047	-0.003	-0.005	-0.002	-0.008
37	-0.019	-0.015	-0.007	-0.031	0.010
38	-0.022	-0.050	0.027	-0.026	0.007
39	0.004	-0.005	-0.022	-0.001	0.006
40	0.009	0.005	0.018	-0.008	0.000
41	-0.011	-0.029	-0.027	0.002	0.018
42	0.019	0.041	-0.035	0.015	0.028
43	-0.013	-0.051	0.031	0.035	0.047
44	0.021	0.041	-0.024	-0.030	-0.055
45	-0.021	-0.030	0.005	-0.017	0.064
46	0.001	-0.012	-0.016	0.069	0.019
47	0.002	0.030	0.026	-0.039	-0.003
48	0.039	0.030	-0.021	0.013	-0.050
49	0.010	0.001	0.016	-0.011	0.002

ESTIMATED CORRELATION MATRIX FOR PARAMETER ESTIMATES

	6	7	8	9	10
6	1.000				
7	-0.094	1.000			
8	-0.002	0.103	1.000		
9	-0.044	-0.103	0.068	1.000	
10	-0.009	0.085	-0.126	0.124	1.000
11	-0.005	-0.031	-0.007	0.004	0.003
12	-0.025	0.028	-0.015	-0.022	-0.001
13	-0.003	0.003	0.061	0.013	-0.035
14	-0.005	-0.048	0.016	0.005	-0.044
15	-0.010	-0.039	-0.003	-0.006	0.042

16	-0.012	-0.002	0.008	0.005	0.008
17	-0.013	0.028	0.003	-0.019	0.032
18	0.010	0.011	0.023	-0.028	-0.062
19	-0.001	-0.016	-0.039	-0.001	-0.008
20	0.000	-0.024	0.009	-0.042	0.012
21	0.025	0.015	-0.011	0.029	0.037
22	-0.015	0.005	-0.007	-0.018	-0.032
23	-0.002	-0.021	-0.003	-0.005	-0.037
24	-0.029	0.044	-0.012	0.013	0.029
25	-0.034	0.009	0.044	0.039	0.013
26	-0.055	-0.016	0.011	-0.010	-0.024
27	0.013	-0.026	0.014	0.006	0.004
28	-0.003	0.045	0.010	0.007	0.005
29	0.015	-0.044	-0.016	0.051	0.022
30	0.029	-0.017	-0.007	0.004	0.005
31	-0.011	0.012	-0.001	-0.027	-0.030
32	0.035	-0.075	0.038	-0.008	0.023
33	0.003	0.019	-0.020	-0.042	-0.004
34	0.001	-0.024	0.039	0.005	-0.004
35	-0.006	-0.017	-0.027	0.004	0.014
36	-0.063	-0.002	-0.037	-0.041	0.023
37	-0.042	0.036	-0.011	-0.006	0.019
38	-0.018	-0.040	-0.040	-0.060	-0.009
39	0.006	-0.043	0.018	-0.045	0.025
40	-0.018	0.027	-0.027	-0.016	0.040
41	0.085	-0.022	-0.018	0.005	0.053
42	0.029	-0.018	-0.023	0.070	0.014
43	-0.044	0.024	0.007	0.022	0.035
44	0.009	-0.044	0.020	-0.020	-0.018
45	0.009	0.019	-0.019	0.019	-0.001
46	-0.007	0.034	0.035	0.002	-0.007
47	0.004	-0.069	-0.027	-0.022	0.013
48	0.010	0.007	0.055	-0.008	-0.032
49	0.004	0.025	-0.032	-0.024	0.040

ESTIMATED CORRELATION MATRIX FOR PARAMETER ESTIMATES

	11	12	13	14	15
11	1.000				
12	0.338	1.000			
13	0.049	0.292	1.000		
14	-0.095	-0.175	-0.043	1.000	
15	0.005	-0.091	-0.233	0.254	1.000

16	-0.015	-0.004	0.047	-0.100	-0.266
17	0.105	0.229	0.059	-0.133	-0.052
18	0.053	0.070	0.269	-0.086	-0.162
19	-0.046	-0.006	-0.050	0.091	0.258
20	0.110	0.059	0.044	-0.047	-0.068
21	0.099	0.206	0.036	-0.104	-0.007
22	0.007	0.092	0.264	-0.001	-0.107
23	-0.011	-0.046	-0.015	0.086	0.213
24	0.022	0.013	0.049	-0.013	-0.037
25	-0.011	-0.026	0.008	0.002	0.022
26	-0.108	-0.246	-0.094	0.204	0.059
27	-0.017	-0.117	-0.254	0.007	0.215
28	0.029	0.002	0.031	-0.097	-0.217
29	-0.054	-0.033	-0.030	0.037	0.081
30	-0.004	-0.042	-0.023	0.007	0.024
31	-0.001	-0.002	0.026	0.008	-0.061
32	-0.053	-0.009	-0.020	0.032	0.019
33	0.002	-0.010	-0.023	-0.011	-0.044
34	-0.055	0.014	-0.002	-0.012	0.021
35	-0.012	-0.001	0.036	0.004	-0.032
36	0.042	0.006	0.007	-0.022	0.005
37	0.077	0.024	0.026	-0.038	-0.019
38	0.020	-0.009	0.055	-0.026	-0.032
39	0.013	-0.017	0.029	0.003	-0.005
40	-0.016	-0.009	0.032	-0.023	0.007
41	0.017	-0.055	0.007	0.015	-0.020
42	-0.019	-0.041	0.000	0.034	0.039
43	0.042	-0.004	-0.024	-0.002	0.003
44	-0.027	0.009	0.013	0.033	0.023
45	0.021	0.005	-0.015	-0.036	-0.050
46	0.008	-0.012	0.019	0.016	0.011
47	0.033	0.015	0.003	0.018	0.005
48	-0.001	-0.002	0.009	-0.021	0.009
49	-0.015	-0.012	0.037	-0.024	0.004

ESTIMATED CORRELATION MATRIX FOR PARAMETER ESTIMATES

	16	17	18	19	20
16	1.000				
17	0.012	1.000			
18	0.074	0.222	1.000		
19	-0.169	-0.083	-0.219	1.000	
20	-0.006	0.096	0.284	-0.170	1.000

21	-0.005	0.250	0.061	-0.031	0.032
22	0.054	0.065	0.194	-0.062	0.056
23	-0.151	-0.042	-0.064	0.251	-0.083
24	0.066	0.096	0.209	-0.118	0.327
25	0.042	-0.024	0.033	-0.004	0.015
26	-0.002	-0.183	-0.055	0.017	-0.047
27	-0.056	-0.029	-0.148	0.070	-0.045
28	0.238	-0.019	0.025	-0.192	0.019
29	-0.044	-0.082	-0.258	0.178	-0.241
30	-0.051	-0.033	-0.021	-0.004	-0.017
31	0.011	0.022	0.025	-0.057	0.027
32	0.004	-0.033	-0.016	-0.001	0.005
33	-0.004	0.019	-0.026	-0.015	0.078
34	0.011	-0.007	-0.027	0.037	-0.058
35	0.011	0.025	0.038	-0.067	0.013
36	-0.002	0.013	-0.006	-0.019	-0.007
37	-0.010	0.003	0.004	0.008	-0.012
38	-0.002	-0.018	-0.010	-0.003	0.022
39	0.027	0.037	-0.030	-0.017	-0.011
40	-0.009	-0.012	0.044	-0.010	-0.005
41	-0.006	-0.015	0.056	0.018	0.041
42	0.008	0.007	0.005	-0.028	-0.009
43	0.055	-0.004	-0.014	0.032	-0.028
44	-0.024	0.013	-0.023	-0.004	0.034
45	0.001	-0.009	0.007	-0.016	-0.040
46	0.006	-0.009	0.037	-0.068	0.008
47	-0.034	0.043	0.016	0.019	-0.034
48	-0.058	0.019	0.015	-0.018	0.069
49	-0.008	-0.011	0.045	-0.014	-0.004

ESTIMATED CORRELATION MATRIX FOR PARAMETER ESTIMATES

	21	22	23	24	25
21	1.000				
22	0.197	1.000			
23	-0.097	-0.165	1.000		
24	0.059	0.219	-0.161	1.000	
25	0.073	0.242	-0.125	0.313	1.000
26	-0.221	-0.047	0.020	0.008	0.006
27	-0.070	-0.205	0.051	-0.029	-0.014
28	-0.001	0.021	-0.216	0.035	0.019
29	-0.067	-0.085	0.072	-0.220	-0.061
30	-0.088	-0.213	0.202	-0.202	-0.287

31	-0.003	0.051	-0.062	0.055	0.019
32	-0.016	0.002	0.011	0.007	0.015
33	-0.020	0.016	-0.050	0.023	0.023
34	0.020	-0.022	0.054	-0.032	-0.046
35	0.015	0.018	-0.053	0.008	-0.001
36	0.003	-0.002	0.020	0.011	0.004
37	0.009	-0.025	-0.009	-0.010	-0.010
38	-0.034	-0.018	0.020	0.000	-0.010
39	-0.039	-0.025	0.023	-0.026	-0.040
40	0.026	0.065	-0.053	0.009	0.053
41	-0.011	0.005	-0.044	-0.009	-0.031
42	-0.049	0.038	0.009	0.028	0.028
43	0.000	-0.031	0.018	-0.019	-0.017
44	-0.002	0.029	-0.012	0.053	0.025
45	0.003	-0.007	0.009	-0.040	-0.018
46	-0.021	-0.036	-0.018	-0.049	-0.027
47	0.021	0.050	-0.001	0.018	0.023
48	0.002	-0.011	0.027	-0.017	-0.024
49	0.022	0.066	-0.055	0.009	0.051

ESTIMATED CORRELATION MATRIX FOR PARAMETER ESTIMATES

	26	27	28	29	30
26	1.000				
27	0.203	1.000			
28	-0.074	-0.150	1.000		
29	0.082	0.260	-0.151	1.000	
30	0.077	0.178	-0.133	0.241	1.000
31	-0.036	-0.300	0.277	-0.251	-0.282
32	0.035	0.002	-0.001	-0.009	0.013
33	-0.007	0.000	0.019	-0.050	-0.071
34	-0.060	0.010	-0.034	0.019	0.042
35	-0.004	-0.005	0.014	-0.007	-0.013
36	-0.052	0.022	-0.029	0.018	-0.006
37	-0.037	0.008	-0.006	0.003	-0.011
38	0.039	0.005	-0.010	-0.006	0.035
39	0.023	-0.010	-0.020	-0.021	0.023
40	-0.061	-0.061	0.049	-0.058	-0.071
41	0.033	-0.021	0.052	-0.013	-0.013
42	0.025	0.033	-0.043	0.071	-0.001
43	0.033	-0.006	0.007	0.020	0.057
44	-0.035	0.014	-0.010	0.008	-0.071
45	-0.004	0.000	0.007	-0.012	0.029

46	-0.002	0.018	-0.002	-0.043	0.032
47	0.008	0.003	-0.029	0.067	0.013
48	-0.043	0.006	-0.029	-0.028	0.031
49	-0.058	-0.062	0.047	-0.060	-0.068

ESTIMATED CORRELATION MATRIX FOR PARAMETER ESTIMATES

	31	32	33	34	35
31	1.000				
32	-0.017	1.000			
33	0.032	0.043	1.000		
34	0.011	-0.001	-0.560	1.000	
35	-0.018	0.007	0.041	-0.021	1.000
36	-0.031	-0.754	-0.038	0.011	0.009
37	0.019	-0.855	-0.022	0.010	-0.004
38	-0.005	-0.004	-0.025	0.051	0.017
39	-0.002	0.029	-0.012	0.047	0.033
40	-0.002	-0.013	0.018	-0.005	-0.071
41	0.015	-0.106	-0.004	0.007	-0.014
42	-0.053	0.026	-0.015	0.000	0.021
43	0.003	-0.031	0.043	-0.059	-0.021
44	-0.005	0.032	-0.013	0.048	0.032
45	0.005	-0.053	-0.002	-0.026	-0.027
46	-0.025	0.021	0.026	-0.035	-0.082
47	-0.013	-0.004	-0.021	0.034	0.023
48	0.021	-0.013	-0.001	0.000	0.006
49	-0.005	-0.011	0.017	0.000	-0.068

ESTIMATED CORRELATION MATRIX FOR PARAMETER ESTIMATES

	36	37	38	39	40
36	1.000				
37	0.673	1.000			
38	0.050	0.012	1.000		
39	-0.006	-0.021	0.683	1.000	
40	-0.004	-0.020	-0.191	-0.190	1.000
41	-0.156	0.045	-0.011	-0.002	-0.034
42	-0.048	-0.032	-0.214	-0.047	0.007
43	0.048	0.054	-0.018	-0.004	-0.052
44	-0.018	-0.044	0.005	-0.002	0.029
45	0.029	0.064	0.019	0.003	0.006
46	-0.004	-0.046	-0.001	0.033	-0.017

47	-0.009	0.015	0.010	-0.001	-0.026
48	0.020	0.001	-0.001	-0.016	-0.003
49	-0.007	-0.023	-0.103	-0.093	0.993

ESTIMATED CORRELATION MATRIX FOR PARAMETER ESTIMATES

	41	42	43	44	45
41	1.000				
42	0.039	1.000			
43	0.026	0.011	1.000		
44	-0.020	-0.012	-0.692	1.000	
45	-0.035	-0.003	0.314	-0.701	1.000
46	0.027	0.000	0.038	-0.028	-0.021
47	-0.001	0.044	-0.017	0.016	0.043
48	-0.016	-0.018	0.000	-0.026	0.019
49	-0.025	0.004	-0.055	0.031	0.007

ESTIMATED CORRELATION MATRIX FOR PARAMETER ESTIMATES

	46	47	48	49
46	1.000			
47	-0.568	1.000		
48	0.012	-0.018	1.000	
49	-0.015	-0.026	-0.004	1.000

TECHNICAL 8 OUTPUT

TECHNICAL 8 OUTPUT FOR BAYES ESTIMATION

CHAIN	BSEED
1	0
2	285380
3	253358

ITERATION	POTENTIAL SCALE REDUCTION	PARAMETER WITH HIGHEST PSR
100	1.008	39
200	1.008	37
300	1.008	40
400	1.001	28

500	1.003	41
600	1.005	41
700	1.003	42
800	1.003	7
900	1.004	7
1000	1.002	7

This is the interaction between latent profile categories and time pressure on worsening of physical health

SUMMARY OF DATA

```
     Number of clusters                         58

       Size (s)    Cluster ID with Size s

          7        22
         16        166
         23        80 71 205
         25        74
         27        192
         31        65
         32        106
         35        228
         37        170 72
         39        50 75 181
         40        182
         42        165
         44        114
         45        173
         46        107
         47        169 30
         48        226 81
         49        36
         50        116 108 40
         56        162 84
         57        61
         59        206 93 83
         60        92
         61        69
         63        6
         64        152
         65        145
         66        4
         68        68
         69        154
         72        174
         78        98
         84        178 2
         95        156 202
         96        144
        108        142
```

131	122
172	24
175	209
224	204
269	125
364	217
738	110
860	100

COVARIANCE COVERAGE OF DATA

Minimum covariance coverage value   0.100

Number of missing data patterns   8

PROPORTION OF DATA PRESENT

Covariance Coverage

	PHYCAT16	CLU2	CLU3	PHY15	KES15
PHYCAT16	1.000				
CLU2	1.000	1.000			
CLU3	1.000	1.000	1.000		
PHY15	0.947	0.947	0.947	0.947	
KES15	0.922	0.922	0.922	0.922	0.922
CHOICE15	0.915	0.915	0.915	0.915	0.915
BULLY15	0.915	0.915	0.915	0.915	0.915
PRESS15	0.915	0.915	0.915	0.915	0.915
DUTIES15	0.915	0.915	0.915	0.915	0.915

Covariance Coverage

	CHOICE15	BULLY15	PRESS15	DUTIES15
CHOICE15	0.915			
BULLY15	0.915	0.915		
PRESS15	0.915	0.914	0.915	
DUTIES15	0.915	0.915	0.915	0.915

UNIVARIATE PROPORTIONS AND COUNTS FOR CATEGORICAL VARIABLES

PHYCAT16

Category 1	0.881	4884.000
Category 2	0.119	662.000

UNIVARIATE SAMPLE STATISTICS

UNIVARIATE HIGHER-ORDER MOMENT DESCRIPTIVE STATISTICS

Variable/ Sample Size	Mean/ Variance	Skewness/ Kurtosis	Minimum/ Maximum	% with Min/Max	Percentiles 20%/60%	40%/80%	Median
CLU2	0.310	0.820	0.000	68.97%	0.000	0.000	0.000
58.000	0.214	-1.328	1.000	31.03%	0.000	1.000	
CLU3	0.448	0.208	0.000	55.17%	0.000	0.000	0.000
58.000	0.247	-1.957	1.000	44.83%	1.000	1.000	
PHY15	0.000	-0.546	-0.632	36.83%	-0.632	0.368	0.368
5254.000	0.233	-1.702	0.368	63.17%	0.368	0.368	
KES15	0.000	1.717	-0.511	21.65%	-0.511	-0.178	-0.178
5114.000	0.289	3.835	3.489	0.04%	-0.011	0.322	
CHOICE15	0.000	-0.402	-2.225	7.41%	-1.225	-0.225	-0.225
5077.000	1.081	-0.336	1.775	8.49%	0.775	0.775	
BULLY15	0.000	3.272	-0.170	87.25%	-0.170	-0.170	-0.170
5073.000	0.239	11.772	3.830	0.08%	-0.170	-0.170	
PRESS15	2.462	0.252	1.000	19.10%	2.000	2.000	2.000
5074.000	1.001	-0.371	5.000	2.76%	3.000	3.000	
DUTIES15	0.000	-1.246	-3.120	2.40%	-0.120	-0.120	-0.120
5077.000	0.861	1.673	0.880	38.74%	-0.120	0.880	

THE MODEL ESTIMATION TERMINATED NORMALLY

USE THE FBITERATIONS OPTION TO INCREASE THE NUMBER OF ITERATIONS BY A FACTOR
OF AT LEAST TWO TO CHECK CONVERGENCE AND THAT THE PSR VALUE DOES NOT INCREASE.

MODEL FIT INFORMATION

Number of Free Parameters 49

MODEL RESULTS

	Estimate	Posterior S.D.	One-Tailed P-Value	95% C.I. Lower 2.5%	95% C.I. Upper 2.5%	Significance
**Within Level**						
PHYCAT16 ON						
PHY15	0.968	0.069	0.000	0.838	1.105	*
CHOICE15	-0.068	0.025	0.003	-0.118	-0.019	*
DUTIES15	-0.031	0.027	0.127	-0.084	0.026	
BULLY15	0.024	0.047	0.297	-0.066	0.118	
KES15	0.226	0.044	0.000	0.138	0.312	*
KES15 WITH						
PHY15	0.051	0.004	0.000	0.044	0.059	*
CHOICE15	-0.089	0.008	0.000	-0.104	-0.074	*
BULLY15	0.057	0.004	0.000	0.050	0.065	*
PRESS15	0.099	0.007	0.000	0.084	0.113	*
DUTIES15	-0.106	0.007	0.000	-0.119	-0.091	*
PHY15 WITH						
CHOICE15	-0.036	0.007	0.000	-0.050	-0.022	*
BULLY15	0.012	0.003	0.000	0.005	0.019	*
PRESS15	0.036	0.007	0.000	0.024	0.049	*
DUTIES15	-0.036	0.006	0.000	-0.048	-0.024	*
CHOICE15 WITH						
BULLY15	-0.058	0.007	0.000	-0.072	-0.044	*
PRESS15	-0.076	0.015	0.000	-0.104	-0.047	*
DUTIES15	0.173	0.014	0.000	0.146	0.201	*
BULLY15 WITH						
PRESS15	0.101	0.007	0.000	0.088	0.115	*
DUTIES15	-0.078	0.006	0.000	-0.090	-0.065	*
PRESS15 WITH						
DUTIES15	-0.177	0.014	0.000	-0.204	-0.152	*
Means						
PHY15	0.003	0.007	0.355	-0.011	0.016	

```
   KES15               0.001      0.008      0.426     -0.013      0.017
   CHOICE15           -0.001      0.015      0.471     -0.031      0.027
   BULLY15             0.000      0.007      0.479     -0.013      0.015
   DUTIES15           -0.002      0.013      0.447     -0.026      0.025

 Variances
   PHY15               0.233      0.005      0.000      0.224      0.243      *
   KES15               0.289      0.006      0.000      0.279      0.301      *
   CHOICE15            1.083      0.022      0.000      1.041      1.127      *
   BULLY15             0.239      0.005      0.000      0.230      0.249      *
   PRESS15             0.955      0.019      0.000      0.919      0.994      *
   DUTIES15            0.861      0.017      0.000      0.828      0.896      *

Between Level

 S1         ON
   CLU2                0.017      0.094      0.422     -0.166      0.205
   CLU3                0.054      0.089      0.286     -0.121      0.222

 PHYCAT16   ON
   CLU2               -0.186      0.098      0.018     -0.393     -0.012      *
   CLU3               -0.007      0.090      0.472     -0.189      0.169
   PRESS15            -0.160      0.157      0.145     -0.455      0.155

 CLU2     WITH
   CLU3               -0.137      0.038      0.000     -0.227     -0.078      *
   PRESS15             0.002      0.020      0.454     -0.040      0.042

 CLU3     WITH
   PRESS15            -0.007      0.021      0.379     -0.051      0.035

 Means
   CLU2                0.309      0.064      0.000      0.181      0.437      *
   CLU3                0.447      0.069      0.000      0.308      0.579      *
   PRESS15             2.387      0.042      0.000      2.306      2.467      *

 Intercepts
   S1                 -0.055      0.078      0.232     -0.213      0.095

 Thresholds
   PHYCAT16$1          0.876      0.384      0.010      0.145      1.645      *

 Variances
   CLU2                0.229      0.044      0.000      0.163      0.337      *
```

CLU3	0.262	0.052	0.000	0.187	0.386	*
PRESS15	0.077	0.018	0.000	0.051	0.121	*
**Residual Variances**						
PHYCAT16	0.015	0.012	0.000	0.002	0.047	*
S1	0.004	0.006	0.000	0.000	0.021	*

STANDARDIZED MODEL RESULTS

STDYX Standardization

	Estimate	Posterior S.D.	One-Tailed P-Value	95% C.I. Lower 2.5%	Upper 2.5%	Significance
**Within-Level Standardized Estimates Averaged Over Clusters**						
**S1 | PHYCAT16 ON**						
PRESS15	-0.026	0.027	0.175	-0.078	0.027	
**PHYCAT16 ON**						
PHY15	0.414	0.024	0.000	0.365	0.460	*
CHOICE15	-0.063	0.023	0.003	-0.107	-0.019	*
DUTIES15	-0.025	0.023	0.137	-0.069	0.020	
BULLY15	0.011	0.021	0.296	-0.030	0.054	
KES15	0.107	0.021	0.000	0.065	0.148	*
**KES15 WITH**						
PHY15	0.198	0.014	0.000	0.171	0.225	*
CHOICE15	-0.159	0.013	0.000	-0.186	-0.133	*
BULLY15	0.219	0.013	0.000	0.191	0.244	*
PRESS15	0.189	0.014	0.000	0.162	0.215	*
DUTIES15	-0.212	0.013	0.000	-0.237	-0.186	*
**PHY15 WITH**						
CHOICE15	-0.072	0.014	0.000	-0.099	-0.044	*
BULLY15	0.050	0.014	0.000	0.021	0.078	*
PRESS15	0.077	0.014	0.000	0.049	0.103	*
DUTIES15	-0.080	0.014	0.000	-0.107	-0.054	*
**CHOICE15 WITH**						
BULLY15	-0.114	0.014	0.000	-0.141	-0.087	*
PRESS15	-0.074	0.014	0.000	-0.101	-0.046	*

```
    DUTIES15           0.179      0.014      0.000      0.151      0.206      *

 BULLY15  WITH
    PRESS15            0.212      0.014      0.000      0.186      0.238      *
    DUTIES15          -0.171      0.013      0.000     -0.198     -0.145      *

 PRESS15  WITH
    DUTIES15          -0.195      0.014      0.000     -0.222     -0.166      *

 Means
    PHY15              0.005      0.014      0.371     -0.022      0.031
    KES15              0.003      0.014      0.422     -0.024      0.030
    CHOICE15          -0.001      0.014      0.468     -0.029      0.026
    BULLY15            0.001      0.014      0.477     -0.027      0.029
    DUTIES15          -0.002      0.014      0.453     -0.029      0.026

 Variances
    PHY15              1.000      0.000      0.000      1.000      1.000
    KES15              1.000      0.000      0.000      1.000      1.000
    CHOICE15           1.000      0.000      0.000      1.000      1.000
    BULLY15            1.000      0.000      0.000      1.000      1.000
    PRESS15            1.000      0.000      0.000      1.000      1.000
    DUTIES15           1.000      0.000      0.000      1.000      1.000

Between Level

 S1         ON
    CLU2               0.106      0.526      0.422     -0.860      1.031
    CLU3               0.355      0.521      0.286     -0.799      1.144

 PHYCAT16   ON
    CLU2              -0.548      0.242      0.018     -0.976     -0.033      *
    CLU3              -0.020      0.270      0.472     -0.537      0.517
    PRESS15           -0.266      0.247      0.145     -0.698      0.270

 CLU2     WITH
    CLU3              -0.565      0.086      0.000     -0.716     -0.371      *
    PRESS15            0.018      0.143      0.454     -0.280      0.294

 CLU3     WITH
    PRESS15           -0.049      0.140      0.379     -0.317      0.232

 Means
    CLU2               0.647      0.143      0.000      0.363      0.929      *
```

CLU3	0.877	0.156	0.000	0.558	1.173	*
PRESS15	8.606	0.943	0.000	6.853	10.667	*
**Intercepts**						
S1	-0.703	0.950	0.232	-2.542	1.131	
**Variances**						
CLU2	1.000	0.000	0.000	1.000	1.000	
CLU3	1.000	0.000	0.000	1.000	1.000	
PRESS15	1.000	0.000	0.000	1.000	1.000	
**Residual Variances**						
PHYCAT16	0.527	0.196	0.000	0.135	0.884	*
S1	0.671	0.279	0.000	0.034	0.987	*

R-SQUARE

Within-Level R-Square Averaged Across Clusters

Variable	Estimate	Posterior S.D.	One-Tailed P-Value	95% C.I. Lower 2.5%	Upper 2.5%
PHYCAT16	0.220	0.021	0.000	0.181	0.265

Between Level

Variable	Estimate	Posterior S.D.	One-Tailed P-Value	95% C.I. Lower 2.5%	Upper 2.5%
PHYCAT16	0.473	0.196	0.000	0.116	0.865

Variable	Estimate	Posterior S.D.	One-Tailed P-Value	95% C.I. Lower 2.5%	Upper 2.5%
S1	0.329	0.279	0.000	0.013	0.966

TECHNICAL 1 OUTPUT

PARAMETER SPECIFICATION FOR WITHIN

TAU

PHYCAT16
0

NU

PHYCAT16	PHY15	KES15	CHOICE15	BULLY15
0	0	0	0	0

NU

PRESS15	DUTIES15
0	0

LAMBDA

	PHYCAT16	PHY15	KES15	CHOICE15	BULLY15
PHYCAT16	0	0	0	0	0
PHY15	0	0	0	0	0
KES15	0	0	0	0	0
CHOICE15	0	0	0	0	0
BULLY15	0	0	0	0	0
PRESS15	0	0	0	0	0
DUTIES15	0	0	0	0	0

LAMBDA

	PRESS15	DUTIES15
PHYCAT16	0	0
PHY15	0	0
KES15	0	0
CHOICE15	0	0
BULLY15	0	0
PRESS15	0	0
DUTIES15	0	0

THETA

PHYCAT16 PHY15 KES15 CHOICE15 BULLY15

PHYCAT16	0				
PHY15	0	0			
KES15	0	0	0		
CHOICE15	0	0	0	0	
BULLY15	0	0	0	0	0
PRESS15	0	0	0	0	0
DUTIES15	0	0	0	0	0

THETA

	PRESS15	DUTIES15
PRESS15	0	
DUTIES15	0	0

ALPHA

PHYCAT16	PHY15	KES15	CHOICE15	BULLY15
0	1	2	3	4

ALPHA

PRESS15	DUTIES15
0	5

BETA

	PHYCAT16	PHY15	KES15	CHOICE15	BULLY15
PHYCAT16	0	6	7	8	9
PHY15	0	0	0	0	0
KES15	0	0	0	0	0
CHOICE15	0	0	0	0	0
BULLY15	0	0	0	0	0
PRESS15	0	0	0	0	0
DUTIES15	0	0	0	0	0

BETA

PRESS15	DUTIES15

PHYCAT16	0	10
PHY15	0	0
KES15	0	0
CHOICE15	0	0
BULLY15	0	0
PRESS15	0	0
DUTIES15	0	0

PSI

	PHYCAT16	PHY15	KES15	CHOICE15	BULLY15
PHYCAT16	0				
PHY15	0	11			
KES15	0	12	13		
CHOICE15	0	14	15	16	
BULLY15	0	17	18	19	20
PRESS15	0	21	22	23	24
DUTIES15	0	26	27	28	29

PSI

	PRESS15	DUTIES15
PRESS15	25	
DUTIES15	30	31

PARAMETER SPECIFICATION FOR BETWEEN

TAU

PHYCAT16
49

NU

PHYCAT16	CLU2	CLU3	PRESS15
0	0	0	0

LAMBDA

	S1	PHYCAT16	CLU2	CLU3	PRESS15
PHYCAT16	0	0	0	0	0
CLU2	0	0	0	0	0
CLU3	0	0	0	0	0
PRESS15	0	0	0	0	0

THETA

	PHYCAT16	CLU2	CLU3	PRESS15
PHYCAT16	0			
CLU2	0	0		
CLU3	0	0	0	
PRESS15	0	0	0	0

ALPHA

S1	PHYCAT16	CLU2	CLU3	PRESS15
32	0	33	34	35

BETA

	S1	PHYCAT16	CLU2	CLU3	PRESS15
S1	0	0	36	37	0
PHYCAT16	0	0	38	39	40
CLU2	0	0	0	0	0
CLU3	0	0	0	0	0
PRESS15	0	0	0	0	0

PSI

	S1	PHYCAT16	CLU2	CLU3	PRESS15
S1	41				
PHYCAT16	0	42			
CLU2	0	0	43		
CLU3	0	0	44	45	
PRESS15	0	0	46	47	48

STARTING VALUES FOR WITHIN

TAU

PHYCAT16
0.000

NU

PHYCAT16	PHY15	KES15	CHOICE15	BULLY15
0.000	0.000	0.000	0.000	0.000

NU

PRESS15	DUTIES15
0.000	0.000

LAMBDA

	PHYCAT16	PHY15	KES15	CHOICE15	BULLY15
PHYCAT16	1.000	0.000	0.000	0.000	0.000
PHY15	0.000	1.000	0.000	0.000	0.000
KES15	0.000	0.000	1.000	0.000	0.000
CHOICE15	0.000	0.000	0.000	1.000	0.000
BULLY15	0.000	0.000	0.000	0.000	1.000
PRESS15	0.000	0.000	0.000	0.000	0.000
DUTIES15	0.000	0.000	0.000	0.000	0.000

LAMBDA

	PRESS15	DUTIES15
PHYCAT16	0.000	0.000
PHY15	0.000	0.000
KES15	0.000	0.000
CHOICE15	0.000	0.000
BULLY15	0.000	0.000
PRESS15	1.000	0.000
DUTIES15	0.000	1.000

THETA

	PHYCAT16	PHY15	KES15	CHOICE15	BULLY15
PHYCAT16	0.000				
PHY15	0.000	0.000			
KES15	0.000	0.000	0.000		
CHOICE15	0.000	0.000	0.000	0.000	
BULLY15	0.000	0.000	0.000	0.000	0.000
PRESS15	0.000	0.000	0.000	0.000	0.000
DUTIES15	0.000	0.000	0.000	0.000	0.000

THETA

	PRESS15	DUTIES15
PRESS15	0.000	
DUTIES15	0.000	0.000

ALPHA

PHYCAT16	PHY15	KES15	CHOICE15	BULLY15
0.000	0.000	0.000	0.000	0.000

ALPHA

PRESS15	DUTIES15
0.000	0.000

BETA

	PHYCAT16	PHY15	KES15	CHOICE15	BULLY15
PHYCAT16	0.000	0.000	0.000	0.000	0.000
PHY15	0.000	0.000	0.000	0.000	0.000
KES15	0.000	0.000	0.000	0.000	0.000
CHOICE15	0.000	0.000	0.000	0.000	0.000
BULLY15	0.000	0.000	0.000	0.000	0.000
PRESS15	0.000	0.000	0.000	0.000	0.000
DUTIES15	0.000	0.000	0.000	0.000	0.000

BETA

	PRESS15	DUTIES15
PHYCAT16	0.000	0.000
PHY15	0.000	0.000
KES15	0.000	0.000
CHOICE15	0.000	0.000
BULLY15	0.000	0.000
PRESS15	0.000	0.000
DUTIES15	0.000	0.000

PSI

	PHYCAT16	PHY15	KES15	CHOICE15	BULLY15
PHYCAT16	1.000				
PHY15	0.000	0.116			
KES15	0.000	0.000	0.145		
CHOICE15	0.000	0.000	0.000	0.541	
BULLY15	0.000	0.000	0.000	0.000	0.119
PRESS15	0.000	0.000	0.000	0.000	0.000
DUTIES15	0.000	0.000	0.000	0.000	0.000

PSI

	PRESS15	DUTIES15
PRESS15	0.501	
DUTIES15	0.000	0.430

STARTING VALUES FOR BETWEEN

TAU

PHYCAT16
1.110

NU

PHYCAT16	CLU2	CLU3	PRESS15
0.000	0.000	0.000	0.000

LAMBDA

	S1	PHYCAT16	CLU2	CLU3	PRESS15
PHYCAT16	0.000	1.000	0.000	0.000	0.000
CLU2	0.000	0.000	1.000	0.000	0.000
CLU3	0.000	0.000	0.000	1.000	0.000
PRESS15	0.000	0.000	0.000	0.000	1.000

THETA

	PHYCAT16	CLU2	CLU3	PRESS15
PHYCAT16	0.000			
CLU2	0.000	0.000		
CLU3	0.000	0.000	0.000	
PRESS15	0.000	0.000	0.000	0.000

ALPHA

S1	PHYCAT16	CLU2	CLU3	PRESS15
0.000	0.000	0.406	0.471	2.462

BETA

	S1	PHYCAT16	CLU2	CLU3	PRESS15
S1	0.000	0.000	0.000	0.000	0.000
PHYCAT16	0.000	0.000	0.000	0.000	0.000
CLU2	0.000	0.000	0.000	0.000	0.000
CLU3	0.000	0.000	0.000	0.000	0.000
PRESS15	0.000	0.000	0.000	0.000	0.000

PSI

	S1	PHYCAT16	CLU2	CLU3	PRESS15
S1	1.000				
PHYCAT16	0.000	1.000			
CLU2	0.000	0.000	0.121		
CLU3	0.000	0.000	0.000	0.125	
PRESS15	0.000	0.000	0.000	0.000	0.501

PRIORS FOR ALL PARAMETERS	PRIOR MEAN	PRIOR VARIANCE	PRIOR STD. DEV.
Parameter 1~N(0.000,infinity)	0.0000	infinity	infinity
Parameter 2~N(0.000,infinity)	0.0000	infinity	infinity
Parameter 3~N(0.000,infinity)	0.0000	infinity	infinity
Parameter 4~N(0.000,infinity)	0.0000	infinity	infinity
Parameter 5~N(0.000,infinity)	0.0000	infinity	infinity
Parameter 6~N(0.000,5.000)	0.0000	5.0000	2.2361
Parameter 7~N(0.000,5.000)	0.0000	5.0000	2.2361
Parameter 8~N(0.000,5.000)	0.0000	5.0000	2.2361
Parameter 9~N(0.000,5.000)	0.0000	5.0000	2.2361
Parameter 10~N(0.000,5.000)	0.0000	5.0000	2.2361
Parameter 11~IW(1.000,7)	infinity	infinity	infinity
Parameter 12~IW(0.000,7)	infinity	infinity	infinity
Parameter 13~IW(1.000,7)	infinity	infinity	infinity
Parameter 14~IW(0.000,7)	infinity	infinity	infinity
Parameter 15~IW(0.000,7)	infinity	infinity	infinity
Parameter 16~IW(1.000,7)	infinity	infinity	infinity
Parameter 17~IW(0.000,7)	infinity	infinity	infinity
Parameter 18~IW(0.000,7)	infinity	infinity	infinity
Parameter 19~IW(0.000,7)	infinity	infinity	infinity
Parameter 20~IW(1.000,7)	infinity	infinity	infinity
Parameter 21~IW(0.000,7)	infinity	infinity	infinity
Parameter 22~IW(0.000,7)	infinity	infinity	infinity
Parameter 23~IW(0.000,7)	infinity	infinity	infinity
Parameter 24~IW(0.000,7)	infinity	infinity	infinity
Parameter 25~IW(1.000,7)	infinity	infinity	infinity
Parameter 26~IW(0.000,7)	infinity	infinity	infinity
Parameter 27~IW(0.000,7)	infinity	infinity	infinity
[truncated: 390,584 more chars]
